# IDOPS, a Profile HMM-Based Tool to Detect Pesticidal Sequences and Compare Their Genetic Context

**DOI:** 10.3389/fmicb.2021.664476

**Published:** 2021-06-28

**Authors:** Stefani Díaz-Valerio, Anat Lev Hacohen, Raphael Schöppe, Heiko Liesegang

**Affiliations:** Genomic and Applied Microbiology & Göttingen Genomics Laboratory, Institute of Microbiology and Genetics, Georg-August University of Göttingen, Göttingen, Germany

**Keywords:** biopesticide, hidden markov model, insecticidal protein, toxin identification, pesticidal, genetic context, IDOPS, comparative genomics

## Abstract

Biopesticide-based crop protection is constantly challenged by insect resistance. Thus, expansion of available biopesticides is crucial for sustainable agriculture. Although *Bacillus thuringiensis* is the major agent for pesticide bioprotection, the number of bacteria species synthesizing proteins with biopesticidal potential is much higher. The Bacterial Pesticidal Protein Resource Center (BPPRC) offers a database of sequences for the control of insect pests, grouped in structural classes. Here we present IDOPS, a tool that detects novel biopesticidal sequences and analyzes them within their genetic environment. The backbone of the IDOPS detection unit is a curated collection of high-quality hidden Markov models that is in accordance with the BPPRC nomenclature. IDOPS was positively benchmarked with BtToxin_Digger and Cry_Processor. In addition, a scan of the UniProtKB database using the IDOPS models returned an abundance of new pesticidal protein candidates distributed across all of the structural groups. Gene expression depends on the genomic environment, therefore, IDOPS provides a comparative genomics module to investigate the genetic regions surrounding pesticidal genes. This feature enables the investigation of accessory elements and evolutionary traits relevant for optimal toxin expression and functional diversification. IDOPS contributes and expands our current arsenal of pesticidal proteins used for crop protection.

## 1. Introduction

Agricultural management strategies regard biopesticides as an environmentally friendly alternative to the chemical formulations used to suppress invertebrate pests (Mnif and Ghribi, 2015; Kachhawa, 2017). Plant-associated, soil, and entomopathogenic bacteria are a natural source of agents with pesticidal potential. Among those, *Bacillus thuringiensis* (Bt) strains and their derived crop protection products are safe for humans, highly specific to the targeted pests, and affordable to manufacture in bulk, making Bt the most successful biopesticide implemented worldwide (George and Crickmore, 2012; Jouzani et al., 2017). Nevertheless, nature fights back and target insects develop resistance mechanisms against Bt, which creates a constant need for novel and improved pest control agents (Vílchez, 2020).

Recently, it became clear that there is a wider variety of pesticidal proteins, synthesized by other bacteria that also interact with insects as part of their lifestyle (Castagnola and Stock, 2014; Ruiu, 2018). These have the potential to replace, supplement, and expand current options for biopest control (Waterfield et al., 2001). Bacteria like *Dickeya* spp., (Loth et al., 2015) *Yersinia* spp., and *Photorhabdus* spp. (Heermann and Fuchs, 2008) are now known sources of such proteins. The varied pesticidal proteins were re-classified in 16 structural groups by the Bacterial Pesticidal Protein Resource Center (BPPRC) (Crickmore et al., 2020a,b).

Biological databases are constantly enriched due to the proliferation of sequencing projects and advancements in sequencing technologies, thus they are a promising reservoir of sequences with uncharacterized pesticidal potential. However, screening such vast amounts of data requires sophisticated computational approaches in order to gain insight and take advantage of less explored resources. The use of profile hidden Markov models (HMMs) to analyze biological data has proven to be robust and sensitive (Eddy, 1998). A profile HMM condenses the information of a multiple alignment of homologous sequences, and therefore, has a higher discriminative power than pairwise similarity-based search tools like BLAST (Söding, 2005). Profile HMMs are broadly applied for protein family assignment, domain analysis, and detection of remote homologies in resources such as InterProScan (Jones et al., 2014; Blum et al., 2020), PFAM (Sonnhammer et al., 1998; Finn et al., 2007), and TIGRFAM (Haft et al., 2012). Examples of dedicated collections of profile HMMs are RVDB-prot (Bigot et al., 2019), a database for detection of viral proteins, and TASmania, a tool for the discovery of toxin-antitoxin systems in bacterial genomes (Akarsu et al., 2019).

Consequently, previous efforts to implement profile HMMs for detection of pesticidal proteins are not surprising. Cry_Processor (Shikov et al., 2020) is a tool for identification of 3 domain Cry sequences based on 4 profile HMMs, one for each domain and one full-length protein, making single domain delimitation possible. BtToxin_Digger (Liu et al., 2020), the successor of BtToxin_scanner, relies on a combination of HMMs, BLAST, and support vector machine (SVM) for prediction of not only 3 domain toxins but also members of the other structural groups.

The focus of the existing toxin prediction tools is to recognize and classify pesticidal protein sequences. Currently, none of them take into consideration the genetic environment of the genes coding for pesticidal proteins. Surrounding elements often include chaperones, crystallization domains, mobile elements, transporters, prophages, and virulence factors (Koni and Ellar, 1993; Shao et al., 2001; Elleuch et al., 2016; Adalat et al., 2017; Fayad et al., 2020; Lechuga et al., 2020). Moreover, the arrangement and distribution of such elements across genomes reveal crucial details about toxin functionality, host adaptation, diversification, and evolution of biopesticides (Khasdan et al., 2007; Peng et al., 2015; Ruffner et al., 2015; Fiedoruk et al., 2017; Zheng et al., 2017; Fayad et al., 2020; Wang et al., 2020). Hence, a more exhaustive approach would not only detect pesticidal sequences but also enable comparative genomics analysis of the candidate toxin in order to characterize the complete expression unit.

The goal of this study was to develop such tool. Our efforts originated IDOPS (Identification of Pesticidal Sequences), a software based on an extensive collection of high-quality profile HMMs. IDOPS aims to provide (i) a detection unit to aid in the finding of pesticidal proteins, especially novel variants within recently expanding groups, (ii) a basic classification system in accordance with the BPPRC nomenclature system, and (iii) a comparative genomics module to investigate toxin genes and their complete expression unit within their genomic environment.

## 2. Materials and Methods

### 2.1. Building Profile HMMs

In order to better represent the great diversity of pesticidal proteins, we created a collection of known and putative pesticidal sequences. Our initial collection combined data from the previous *Bacillus thuringiensis* Toxin Nomenclature website (Crickmore et al., 1998), and matches for various pesticidal sequences found at UniProtKB-2020_06 (UniProt Consortium, 2019). Since UniProt-TrEMBL includes fragmented and repeated entries, redundancy was removed by clustering sequences of 100% identity with CD-HIT v.4.8.1 (Li and Godzik, 2006); then the longest member of each cluster was preserved. The representative sequences were used to create an all vs. all matrix with BLASTp v.2.9.0 (Camacho et al., 2009). This matrix was the input for clustering with the Markov Clustering Algorithm implemented by MCL (Enright et al., 2002). Resulting groups containing at least five members were aligned by ClustalO 1.2.4 (Sievers and Higgins, 2018), and the alignment was passed to hmmbuild-HMMER v.3.3 (Eddy, 1998) to create profile hidden Markov models (HMMs). Furthermore, an individual profile HMM was created to represent the C-terminal region of Cry toxins longer than 1,000 amino acids. For such purpose, the toxin core of the Cry sequences was removed from the alignment before the hmmbuild step. The preliminary profile HMM database accurately described pesticidal proteins of *B. thuringiensis* and was useful for detection of novel toxins. Nevertheless, at the time of this study, the BPPRC released the updated classification of pesticidal proteins and their source organisms. Therefore, the initial profile model collection undergone further examination and additional models were created to represent sequences from non Bt organisms.

### 2.2. Model Refinement and Validation

Several rounds of manual refinement and optimization were necessary to ensure high sensitivity and specificity of the models. This step included evaluation of the sequences used for each profile HMM, their phylogenetic relationship, domain signature predicted by the InterPro consortium (Blum et al., 2020), removal of biases produced by over-represented sequences and performance when databases were scanned using hmmsearch-HMMER v.3.3 (Eddy, 1998).

Protein sequences from the BPPRC database were considered as true positives. For a control dataset of true negatives, a collection of bacterial pore-forming toxins and related proteins from other bacteria was used (Gonzalez et al., 2008; Supplementary Table 1). The overall performance of the models was evaluated by scanning the UniProtKB databases. Furthermore, the distribution of the sequences matched by each model was analyzed and served to determine a trusted cutoff value above which true protein class members are found.

Criterion to define a good model are:

- Identification of all the true positive members of the protein class (or subclass) described by the model within the high score range.
- Additional matches within the high score range can be consistently assigned to the protein group by evaluation of domain signatures, source organism, and quality of alignment with true members.
- Distantly related pore-forming toxins from the true negative control dataset are not found at all or found below the trusted cutoff value.
- The distribution of the proteins matched by the model when searching UniProtKB databases indicates a clear separation between true members and other protein matches.

### 2.3. Pipeline Implementation

This collection of profile HMMs can be used effectively to search for matches within query sequences, genomes, and whole databases. It was implemented in a pipeline named Identification of Pesticidal Sequences (IDOPS). The software applied in IDOPS and the corresponding versions are presented in Table 1. To ease distribution and installation, IDOPS is available as a conda package (https://anaconda.org/GAMB-GO/idops) and its source code is found at Github (https://github.com/GAMB-GO/IDOPS), under a GPLv3 license.

**Table 1:** Software dependencies and versions implemented in IDOPS.

**Software**	**Version**
Python	3.7.6
HMMer (hmmbuild, hmmsearch, hmmscan, hmmalign)	3.3
Biopython	1.76
Easyfig	2.2.5
Clustal Omega	1.2.4
BLAST	2.9.0
Prokka	1.14.6

Valid input formats for IDOPS are fasta (for single or multiple proteins) and genbank (for complete or draft genomes). Candidate pesticidal sequences are identified by hmmscan-HMMER v.3.3 (Eddy, 1998) and evaluated against the trusted cutoffs of the refined profile models. To facilitate the assessment of the found candidates, IDOPS internally generates profile alignments and reports phylogenetic trees for each match with the 10 nearest sequences of the corresponding protein groups.

Furthermore, in order to characterize the genomic context of identified pesticidal proteins and produce relevant insights regarding evolution and functionality, IDOPS offers an additional feature when genomic data is provided in genbank format. First, it retrieves the 5000 bp upstream and downstream regions of each match. Secondly, it does a Prokka annotation (Seemann, 2014) of such sequences. Finally, it creates an EasyFig (Sullivan et al., 2011) comparison that depicts BLAST identities between conserved regions.

### 2.4. Comparative Analysis of Genetic Environments

We used IDOPS to analyze eight *B. thuringiensis* plasmids carrying *cry1* genes (detailed strains and accession ID list in Supplementary Table 2). It was previously reported that *cry1* can occur alone or as part of an insecticidal pathogenicity island (Fiedoruk et al., 2017). We used this observation to demonstrate the value of IDOPS to facilitate the discovery of different genetic arrangements.

### 2.5. Benchmarking

The performance of IDOPS was compared with that of current tools for pesticidal protein detection: Cry_Processor (Shikov et al., 2020) and BtToxin_digger (Liu et al., 2020). Cry_Processor was developed to identify 3 domain Cry sequences. Therefore, the tool was examined taking in consideration only this group of proteins. It provides two search modes, Find Domains (FD) that is based upon a hmmsearch against generalized HMM models, and Domains Only (DO) which searches directly for each of the domains without a filtering step. The two modes were applied. Both tools were tested with sequences from the BPPRC as positive dataset and the true negative collection of distantly related pore-forming toxins.

## 3. Results

### 3.1. A Collection of High-Quality Profile Hidden Markov Models

We developed highly specific profile HMMs to accurately represent each of the 16 structural groups defined by the Bacterial Pesticidal Protein Resource Center. The iterative refinement and manual curation process led to the creation of subgroups within some of the protein classes; particularly for highly populated and diverse groups, like Cry and Cyt. Our final collection consists of 31 profile hidden Markov models (detailed list in Supplementary Table 3).

The Xpp category for unclassified homology groups contains some proteins that could not be modeled based on multiple sequence alignments due to insufficient available sequences at the databases, namely Xpp37, Xpp76, and Xpp77. For those proteins single sequence models were created and incorporated in IDOPS. Furthermore, an individual profile HMM was dedicated to the C-terminal region of the 3 domain pesticidal proteins.

Every profile HMM satisfies the criteria for a good model. The final subsets of sequences selected to construct the models are enough to represent the whole diversity of each group while retaining specificity. Consequently, there is no significant overlap between the matches identified by each profile HMM, as shown at the Supplementary Table 4. None of the pore-forming proteins from the true negative dataset was identified by IDOPS models.

**Figure 1:**
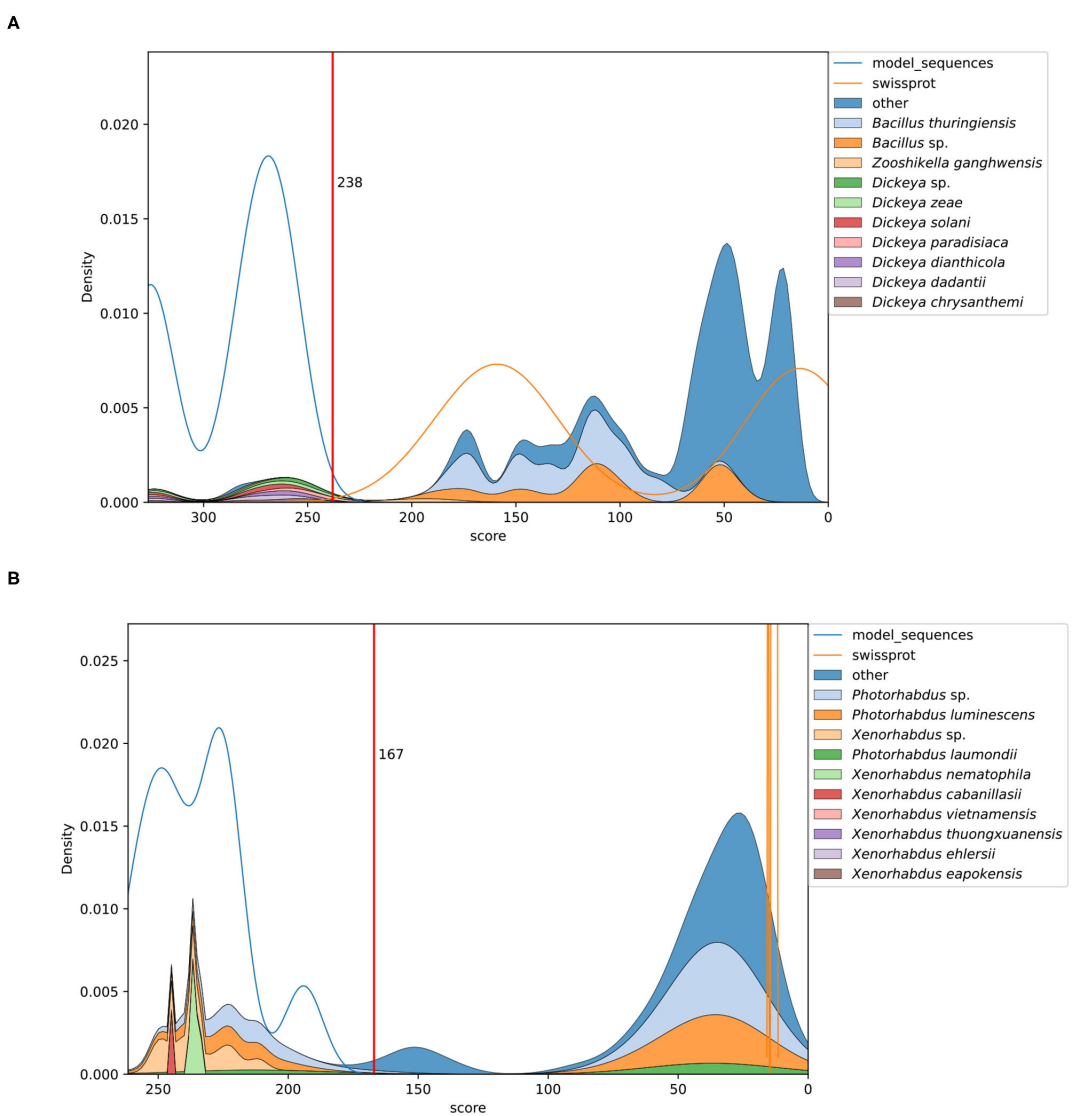
Kernel density plots depicting distribution of model matches in UniProtKb. **(A)** The profile HMM identifies Cyt 4/5/6/7 proteins from *Dickeya* spp. within the high score range. The red line marks the curated gathering cutoff. Matches scoring below 238 correspond to related Cyt proteins from other source organisms. **(B)** A model targeting Pra sequences produced by *Photorhabdus* spp. and *Xenorhabdus* spp. Some *Yersinia* spp. proteins score directly next to the gathering cutoff.

The scanning of UniProtKB databases was beneficial to elucidate the selective power of each model and to determine the gathering cutoff values. A density estimate of the hit distribution against UniProt-TrEMBL and UniProt-SwissProt depicts how accurately our models discriminate true members of each protein class from the whole database. In Figure 1, two examples are shown.

Figure 1A depicts the sequences identified by a model targeting Cyt 4/5/6/7 from *Dickeya* spp. Only proteins from *Dickeya* spp. score above the gathering cutoff and those below it correspond to related Cyt proteins produced by other organisms like *Bacillus thuringiensis*. The example in Figure 1B shows the matches of a model built to identify Pra proteins. Here, the high scoring sequences are clearly separated from most of the non-relevant hits. Interestingly, some entries, annotated as "uncharacterized proteins," score just below the gathering threshold. These are produced by members of the *Yersinia* genus. A similar distribution is observed when analyzing the matches of the toxin partner component, represented by the Prb HMM (Plots for each model are found as Supplementary Figures 1–31).

In addition, it is of notice the abundance of potential new pesticidal proteins identified by our approach. When the UniProtKb-TrEMBL database was scanned, a total of 2,460 protein sequences were found within the trusted score range of IDOPS profile HMMs, many of them annotated as uncharacterized or hypothetical proteins (Table 2).

**Table 2:** Protein sequences identified by IDOPS profile HMMs in UniProtKb-TrEMBL compared with the amount of sequences at the Bacterial Pesticidal Protein Resource Center database.

	**Number of protein Sequences above gathering cutoff**	
**Protein class**	**BPPRC database**	**UniProtKB-TrEMBL**
app	10	121
cry	720	1,123
cyt	40	87
gpp	11	6
mcf	5	82
mpf	5	14
mpp	40	130
mtx	1	3
pra	3	59
prb	3	46
spp	2	482
tpp	30	52
vip	108	120
vpa	20	40
vpb	20	79
xpp	14	16

Notably, models representing groups with few members in the BPPRC collection; like App and Spp, with 10 and 2 entries, respectively, detected plenty of potentially novel pesticidal sequences; 121 for App and 482 in the case of Spp. In other cases, no new entry is matched by our profile HMMs, this is true for Xpp76 and Xpp77 proteins, members of the Xpp group.

### 3.2. Comparative Analysis of Genetic Environments

**Figure 2:**
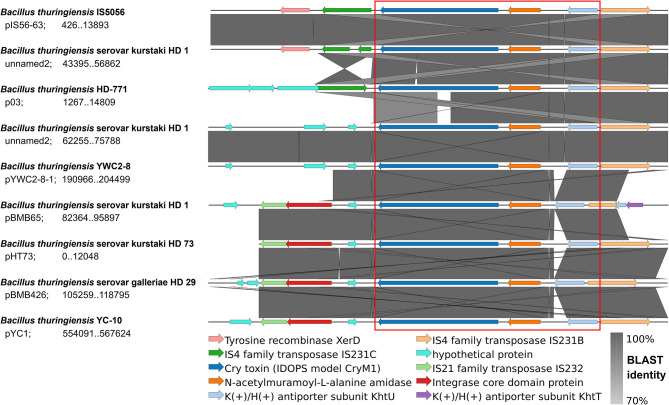
IDOPS genetic environment comparison. IDOPS retrieved, annotated, and aligned the genetic region surrounding *cry1* genes reported as a cassette within Bt plasmids by Fiedoruk et al. (2017). The visualization is generated by EasyFig as implemented internally in IDOPS. The reported *cry1* cassette is marked in red. Strain names, plasmid, and coordinates appear next to each row.

In order to demonstrate the potential of IDOPS to investigate the genetic context of pesticidal proteins, we tested our tool against known reported *cry1* cassettes in plasmids of *B. thuringiensis* (Figure 2).

Using the sequences of eight Bt plasmids (retrieved in genbank format), IDOPS automatically generated a color coded and uniformly annotated alignment of genome regions encoding the *cry1* cassettes. In the red box, the figure shows the *cry1* cassette components (Cry1 -N-acetylmuramoyl-L-alanine amidase - K(+)/H(+) antiporter) and their genetic environment. IDOPS's output already allows the identification and grouping of the *cry1* cassette variants according to their particular elements, such as transposases from different families. Moreover, the figure is in accordance with the manually generated figure created by Fiedoruk et al. (2017), showing how IDOPS identifies, aligns, and displays the genomic surrounding of a toxin of interest.

### 3.3. Benchmarking

To evaluate the specificity and the sensitivity in comparison with currently implemented toxin identification tools, BtToxin_Digger and Cry_Processor, we applied benchmark tests (Table 3). The proteins from the BPPRC database were used as true positives and distantly related pore forming toxins sequences as a true negative dataset.

**Table 3:** Benchmark of tools identifying pesticidal sequences from the Bacterial Pesticidal Protein Resource Center (BPPRC).

**Structural group**	**Sequences at the BPPRC**	**IDOPS**	**BtToxin_digger**	**Cry_processor**	
				**full_domain**	**domain_only**
Cry	720	716	706	696	715
App	10	10	10	N\A	
Cyt	40	40	40	N\A	
Gpp	11	11	11	N\A	
Mcf	5	5	5	N\A	
Mpf	5	5	5	N\A	
Mpp	40	40	39	N\A	
Mtx	1	1	1	N\A	
Pra	3	3	2	N\A	
Prb	3	3	3	N\A	
Spp	2	2	0	N\A	
Tpp	30	30	30	N\A	
Vip	108	108	107	N\A	
Vpa	20	20	20	N\A	
Vpb	20	20	0	N\A	
Xpp	14	14	14	N\A	

All three programs performed convincingly throughout the specificity test. None of the sequences from the true negative dataset were missannotated as a pesticidal protein by either Cry_Processor, BtToxin_Digger or IDOPS. The generalized profile HMMs of Cry_Processor returned some single domain matches. Nevertheless, those did not meet the criteria to be reported as pesticidal proteins.

Concerning the sensitivity Cry_Processor showed some discrepancy between the FD (Full Domain) and DO (Domain only) modes, as 696 and 715 out of 720 sequences were identified as 3 domain toxins, respectively (Table 3). In a similar way, BtToxin_Digger successfully recognized 994 of the 1,033 input sequences from the BPPRC. It failed to detect any of Spp and Vpb sequences, while identification of Cry, Vip, Mpp, Pra, and Tpp groups was incomplete (Table 3). IDOPS had the highest retrieval rate regarding this dataset. It recognized all but three Cry sequences above its gathering cutoff values. In the case of the missed toxins, Cry1Ca10, Cry3Bb3, and Cry11Aa2, a closer look revealed that these three sequences represent truncated toxins. The proteins sequences have been, nevertheless, recognized as hits that scored below trusted cut off. Exclusively IDOPS recovered all complete protein sequences from the tested true positive data set.

## 4. Discussion

Here we present IDOPS, a tool to detect bacterial pesticidal protein sequences and compare their genetic environment. The power of IDOPS comes from a collection of high-quality profile hidden Markov models; each one carefully designed to represent a structural group as defined by the Bacterial Pesticidal Protein Resource Center (BPPRC) (Crickmore et al., 2020a,b). To this date, the tool comprises the most exhaustive and complete collection of models describing pesticidal proteins.

We compared IDOPS with other tools implementing profile HMMs, Cry_Processor (Shikov et al., 2020) and BtToxin_Digger (Liu et al., 2020). Neither of those recognized all of the complete sequences from the positive dataset, making the search against genomes or full databases potentially incomplete. Moreover, Cry_Processor's database is not up to date with the current BPPRC nomenclature. On the other hand, BtToxin_Digger has a greedy approach behind its profile HMMs. While this could work well for closely related and not so diverse structural groups, it is not the best option when dealing with varying sequences, such as members of the Cry group or even proteins of the Xpp class, which lack in shared homology between them. IDOPS overcomes such limitations with its comprehensive collection of profile HMMs. Additionally, it provides a unique genetic context comparison feature.

Gathering cutoff values for IDOPS' models are optimized to recognize full-length proteins, thus shorter or incomplete sequences will score below it and won't be reported. In our tests, IDOPS recognized 717 from the 720 Cry sequences at the BPPRC database. A detailed look revealed that none of the three missed sequences are full-length 3 domain proteins. Cry3Bb3 presents only the InterPro signatures of the central (IPR001178) and the C-terminal domains (IPR005638). Moreover, Cry1Ca10 and Cry11Aa2 are both annotated as partial proteins with sequence lengths of 181aa and 78aa, respectively. Cry1Cb3 is a particular case, since it is not a full-length toxin but rather just the C-terminal portion of a long Cry protein, with the C-terminal (IPR005638) and domain V (IPR041587) regions. Since we developed a dedicated model for the C-terminal region of a 3 domain protein, and the model recognized the sequence as such, it was reported, but under this considerations. However, in case a researcher using IDOPS needs to retrieve the low-scoring proteins, we set up an option to disable the gathering cutoff, so even incomplete matches will be reported for further manual evaluation.

Bias within biological databases affects the creation and refinement of profile HMMs. Cry is one of the most studied pesticidal protein groups, as reflected by the amount of available sequences and their diversity, which are valuable to build rich and diverse profile HMMs. Nevertheless, there is a composition bias within the available Cry sequences. The BPPRC database contains 720 Cry entries and 276 of those correspond to Cry1 sequences. Such skewed composition may be carried over to further studies and databases. To ensure that the profile HMMs do not suffer from this bias, several rounds of model training and refinement were done to find the adequate sequences to represent each pesticidal class. Conversely, other groups, such as Mcf, Mtx, and Spp have significantly fewer representatives, sometimes single entries; this in turn makes model building a challenging task.

IDOPS' models take into consideration common properties of distinct subgroups within each pesticidal protein group. For instance, Cyt proteins that are synthesized by the *Dickeya* spp. cluster separately from the well resolved Cyt proteins of the *Bacillus* clade. They have a shorter N-terminal region and lack hemolytic activity when compared with Cyts from the *Bacillus* spp. (Soberón et al., 2013; Loth et al., 2015). Consequently, it becomes reasonable to have a distinct model to better represent this subgroup. In a similar way, the Pra and Prb proteins of *Vibrio* spp. were modeled separately from their counterparts found in *Xenorhabdus* spp. and *Photorhabdus* spp.

The current BPPRC database contains three entries for the Pra category, which corresponds to "*Photorhabdus* Insect-Related toxin A component" produced by *Photorhabdus luminescens* subsp. *luminescens, Xenorhabdus nematophila*, and *Vibrio parahaemolyticus* M0605 (Crickmore et al., 2020a). Nevertheless, it is intriguing to find sequences from *Yersinia* spp. scoring very close to the BPPRC proteins. *Yersinia* spp. shares insecticidal potential with *P. luminescens*, as homologous proteins with similar genetic arrangements have been found, perhaps as product of horizontal gene transfer (Heermann and Fuchs, 2008; Ahantarig et al., 2009; Castagnola and Stock, 2014; Hurst et al., 2016). Therefore, the sequences encoded by *Yersinia* spp. and matched by the Pra model might represent related variants of the Pra toxin. Supporting this idea, the match distribution of the Prb model shows *Yersinia* spp. proteins in a similar position, meaning this bacteria might potentially produce both the PirA and the PirB toxin components.

Besides source organisms, structural variations within each pesticidal protein group were contemplated for IDOPS' models. Cry2, Cry11, and Cry18, despite being part of the 3 domain category, lack some of the conserved blocks described for this group (Schnepf et al., 1998; Palma et al., 2014). Accordingly, a distinct model was created for the proteins of this subgroup. In a similar way, another subgroup of Cry proteins present variant and alternate versions of such blocks (Schnepf et al., 1998; de Maagd et al., 2001); for example, Cry5, Cry12, and Cry21, thus they were grouped and modeled apart.

IDOPS provides a profile HMM dedicated to the C-terminal extension of the Cry proteins. The rationale for its creation is the evidence of such sequences encoded in proximity to the short variants of *cry* genes. These proteins have homology to the C-terminal region of the long Cry toxins (de Maagd et al., 2003). A role in crystal formation, packing, and stabilization has been reported for the C-terminal extension (Naimov et al., 2006; Peng et al., 2015). Moreover, chimeric toxins made by artificial recombination of N-terminal and C-terminal regions of Cry proteins have shown increased crystal stability and toxicity (Naimov et al., 2006; Zghal et al., 2016). Therefore, with the C-terminal model, IDOPS contributes to identify independent instances of this C-terminal extension. These instances may be useful to investigate crystallization properties and toxic activity of pesticidal proteins.

IDOPS' profile HMMss were meticulously tested against a) the sequence collection at BPPRC, b) a true negative dataset of pore-forming toxins, and c) the whole UniProtKB database. IDOPS recognized all complete sequences of the true positive dataset, none of the false positives, and 2,460 further sequences with pesticidal potential from the UniProtKB database. The abundance of sequences identified at UniProtKB exposes the unexplored potential of the less investigated pesticidal groups (Table 1). Having such dedicated models allows to infer some aspects regarding the candidate pesticidal protein identified by IDOPS. For example, whether it is: a Cry with the conserved blocks, a member of Xpp of a specific subtype, or to which kind of Cyt it belongs. Altogether, the final models are sensitive, specific for each structural group and constitute a promising aid in the search for novel pesticidal protein sequences.

Further developments of IDOPS will include expansion of the search toward other sequence collections, such as metagenomic data. By extended scanning, novel members of the less populated groups could be detected and used to improve the current single sequences models such as Xpp37, Xpp76, and Xpp77. Moreover, as other virulence factors have been shown to support the toxic activity of pesticidal proteins, especially in *B. thuringiensis* (George and Crickmore, 2012; Malovichko et al., 2019), it may be worth to target some of these features by dedicated profile HMMs. Another course of action for IDOPS will be the implementation of the tool as a website service to facilitate its access to the scientific community without the need of local installation.

IDOPS facilitates the comparison of the genetic environment of pesticidal sequences in a systematic and reliable way. The analysis of plasmids carrying *cry1* genes automatically generated an output consistent with Fiedoruk et al. (2017) results. Comparative genomics have proven relevant to understand genetic dynamics of pesticidal proteins. For example, Lechuga et al. (2020) proposed a plasmidial origin to a chromosomally-located Cry1Ba4 only after examination and comparison of the genetic context with that of plasmid-encoded toxins. In a further extensive analysis, we used IDOPS to detect a previously unreported chromosomal *cry* cassette. By comparative genomics we discovered *cry5, cry10*, and *cry13* variants in a rather highly conserved genetic environment. Moreover, we consistently found a *Siphoviridae*-like prophage region in the vicinity of the cassette (Lev Hacohen et al. unpublished data). IDOPS was of great help to detect such arrangement in several Bt strains, opening intriguing evolutionary questions.

We achieved a sophisticated and comprehensive tool that provides not only detection and structural classification of pesticidal proteins, but also a feature that identifies, retrieves, and aligns the genomic context of the pesticidal sequences. IDOPS was designed in accordance to the BPPRC nomenclature system. The benchmark confirmed it has the highest sensitivity available among other toxin search tools. This combination of a highly sensitive toxin detection engine with a solid genome comparison module is, to our knowledge, unique. All considered, we created IDOPS as a tool that addresses comparative genomics of pesticidal proteins to gain novel insights on biopesticides.

## Data Availability Statement

The datasets presented in this study can be found in online repositories. The names of the repository/repositories and accession number(s) can be found in the article/Supplementary Material.

## Author Contributions

SD-V build the initial profile HMMs, performed research, analyzed data, and wrote the manuscript. ALH performed research, refined profile HMMs, and wrote the manuscript. RS performed research, coded the software tool, and wrote the manuscript. HL designed the study, performed research, evaluated results, and wrote the manuscript. All authors contributed to the article and approved the submitted version.

## Conflict of Interest

The authors declare that the research was conducted in the absence of any commercial or financial relationships that could be construed as a potential conflict of interest.
