# Supplementary material for: IDOPS, a Profile HMM-Based Tool to Detect Pesticidal Sequences and Compare Their Genetic Context
Source: Front Microbiol. 2021 Jun 28;12:664476. doi: 10.3389/fmicb.2021.664476 (PMC8279765; doi:10.3389/fmicb.2021.664476)
Supplement: Supplementary file 3 [file Data_Sheet_2.PDF]

Supplementary Material

The file IDOPS.hmm contains the profile HMM database. The file idops-0.2.2.py38.tar.bz2 contains the IDOPS software and the README file describes how to install the software.

Table S1. Bacterial pore-forming toxins used as true negative dataset used for model validation and benchmarking.

**Toxin**	**Organism**	**Toxin type**	**Acession**
Aerolysin	*Aeromonas hydrophila*	Small β-PFT	P09167
α-toxin	*Staphylococcus aureus*	Small β-PFT	P09616
Perfringolysin O	*Clostridium perfringens*	CDCs (β-PFT)	P0C2E9
Streptolysin O	*Streptococcus pyogenes*	CDCs (β-PFT)	P0DF97
Listeriolysin O	*Listeria monocytogenes*	CDCs (β-PFT)	P13128
Pneumolysin O	*Streptococcus pneumoniae*	CDCs (β-PFT)	Q04IN8
Anthrax toxin	*Bacillus anthracis*	AB/Small β-PFT	P13423
Anthrax toxin	*Bacillus anthracis*	AB/Small β-PFT	P15917
Diphtheria toxin	*Corynebacterium diphtheriae*	AB	P00588
Vcc	*Vibrio cholerae*	Small β-PFT	P09545
Hemolysin BL	*Bacillus cereus*	α-helical	P80172

Table S2. Genomic Data used for IDOPS Easyfig visualization.

Organism	Strain	Accession	Plasmid ID	Toxin Locus Tag
Bacillus thuringiensis serovar thuringiensis	IS5056	CP004131	pIS56-63	H175_63p05
Bacillus thuringiensis serovar kurstaki	HD 1	CP009999	unnamed2	BG08_6909<br>BG08_6342
Bacillus thuringiensis	HD-771	CP003755	p03	BTG_32568
Bacillus thuringiensis	YWC2-8	CP013056	pYWC2-8-1	AQ980_30485
Bacillus thuringiensis serovar kurstaki	HD-1	CP004873	pBMB65	BTK_33446
Bacillus thuringiensis serovar kurstaki	HD73	CP004070	pHT73	HD73_6004
Bacillus thuringiensis serovar galleriae	HD-29	CP010090	pBMB426	BT4G5_33165
Bacillus thuringiensis	YC-10	CP011350	pYC1	XI92_31970

Table S3. IDOPS' high-quality profile HMM collection and their targets.

Profile HMM	Target description
appM1	Pesticidal proteins with predominately alpha helical structures
cryM1	Proteins containing the classical 3 Domains of Cry toxins
cryM2	Cry proteins belonging to Cry2 or Cry18 subgroup
cryM3	Cry proteins that belong to Cry11 subgroup
cryM4	Cry proteins that belong to Cry31/70/63 subgroup
cryM5	Cry proteins with less conserved versions of the classical 3 Domains
cryM6	The C-terminal region of Cry pesticidal proteins
cytM1	Pesticidal proteins of the Cyt1 subgroup
cytM2	Pesticidal proteins of the Cyt2 subgroup
cytM3	Pesticidal proteins of the Cyt3 subgroup
cytM4	Pesticidal proteins related to Cyt4/5/6/7 produced by *Dickeya* spp.
gppM1	Pesticidal proteins with homology to the aeGerolysins
mcfM1	Pesticidal proteins related to the "Makes Caterpillars Floppy" toxins
mpfM1	Pesticidal proteins of the Membrane Attack Complex/Perforin superfamily
mppM1	Pesticidal proteins related to Sip1
mppM2	Beta pore-forming pesticidal proteins from the ETX/Mtx2 family
mtxM1	Pesticidal proteins related to the Mtx1 toxin
praM1	Pesticidal proteins related to the *Photorhabdus* Insect-Related toxin A component
praM2	Pesticidal proteins related to the *Photorhabdus* Insect-Related toxin A component produced by *Vibrio* spp.
prbM1	Pesticidal proteins related to the *Photorhabdus* Insect-Related toxin B component
prbM2	Pesticidal proteins related to the *Photorhabdus* Insect-Related toxin B component produced by *Vibrio* spp.
sppM1	Sphaericolysin like pesticidal proteins
tppM1	Beta pore-forming pesticidal proteins containing the Toxin_10 (Bin-like) domain
vipM1	Multi-domain proteins related to the toxin originally identified as Vip3
vpaM1	Pesticidal proteins related to the ADP-ribosyltransferase active component of binary toxins
vpbM1	Pesticidal proteins related to the binding component of binary toxins such as Vip1
vpbM2	Pesticidal proteins related to the binding component of binary toxins such as Vip4
xppM22	Pesticidal proteins related to Xpp22
xppM37	Pesticidal proteins related to Xpp37
xppM55	Pesticidal proteins related to Xpp55
xppM76	Pesticidal proteins related to Xpp76
xppM77	Pesticidal proteins related to Xpp77

Table S4. Cross-Evaluation of HMMs (rows) against Sequences (columns)

	App	Cry	Cyt	Gpp	Mcf	Mpf	Mpp	Mtx	Pra	Prb	Spp	Tpp	Vip	Vpa	Vpb	Xpp	_control
App	10/10	-	-	-	-	-	-	-	-	-	-	-	-	-	-	-	-
Cry	-	717/720	-	-	-	-	-	-	-	-	-	-	-	-	-	-	-
Cyt	-	-	40/40	-	-	-	-	-	-	-	-	-	-	-	-	-	-
Gpp	-	-	-	11/11	-	-	-	-	-	-	-	-	-	-	-	-	-
Mcf	-	-	-	-	5/5	-	-	-	-	-	-	-	-	-	-	-	-
Mpf	-	-	-	-	-	5/5	-	-	-	-	-	-	-	-	-	-	-
Mpp	-	-	-	-	-	-	40/40	-	-	-	-	-	-	-	-	-	-
Mtx	-	-	-	-	-	-	-	1/1	-	-	-	-	-	-	-	-	-
Pra	-	-	-	-	-	-	-	-	3/3	-	-	-	-	-	-	-	-
Prb	-	-	-	-	-	-	-	-	-	3/3	-	-	-	-	-	-	-
Spp	-	-	-	-	-	-	-	-	-	-	2/2	-	-	-	-	-	-
Tpp	-	-	-	-	-	-	-	-	-	-	-	30/30	-	-	-	-	-
Vip	-	-	-	-	-	-	-	-	-	-	-	-	108/108	-	-	-	-
Vpa	-	-	-	-	-	-	-	-	-	-	-	-	-	20/20	-	-	-
Vpb	-	-	-	-	-	-	-	-	-	-	-	-	-	-	20/20	-	-
Xpp	-	-	-	-	-	-	-	-	-	-	-	-	-	-	-	14/14	-

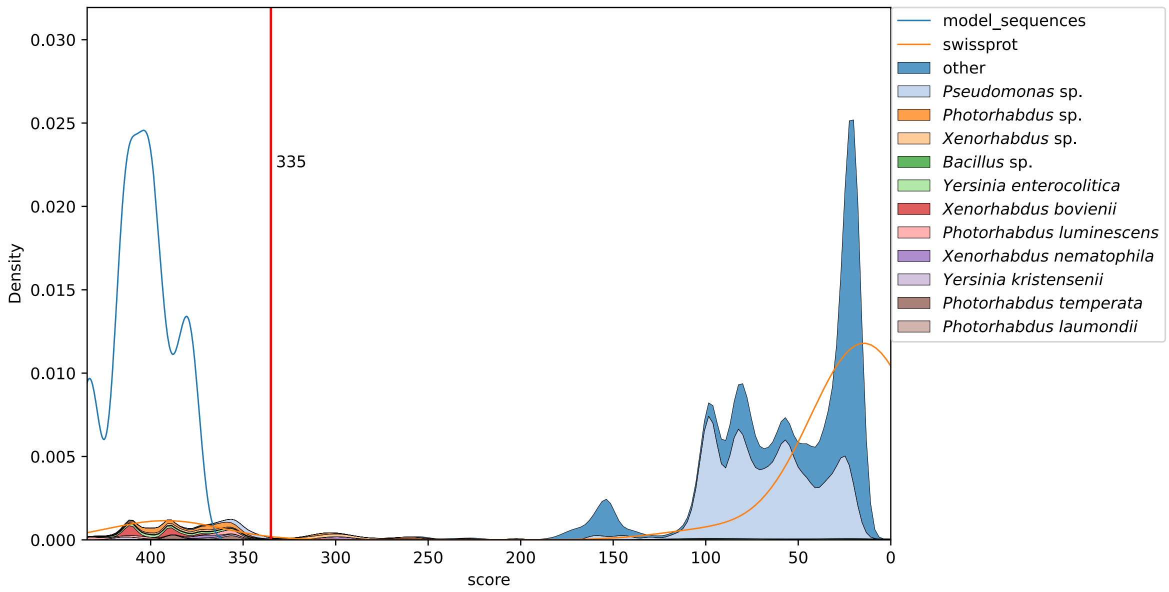

Figure S1. Kernel density plot depicting distribution of model matches in UniProtKb The profile HMM identifies sequences that belong to the App group. The red line marks the curated gathering cutoff.

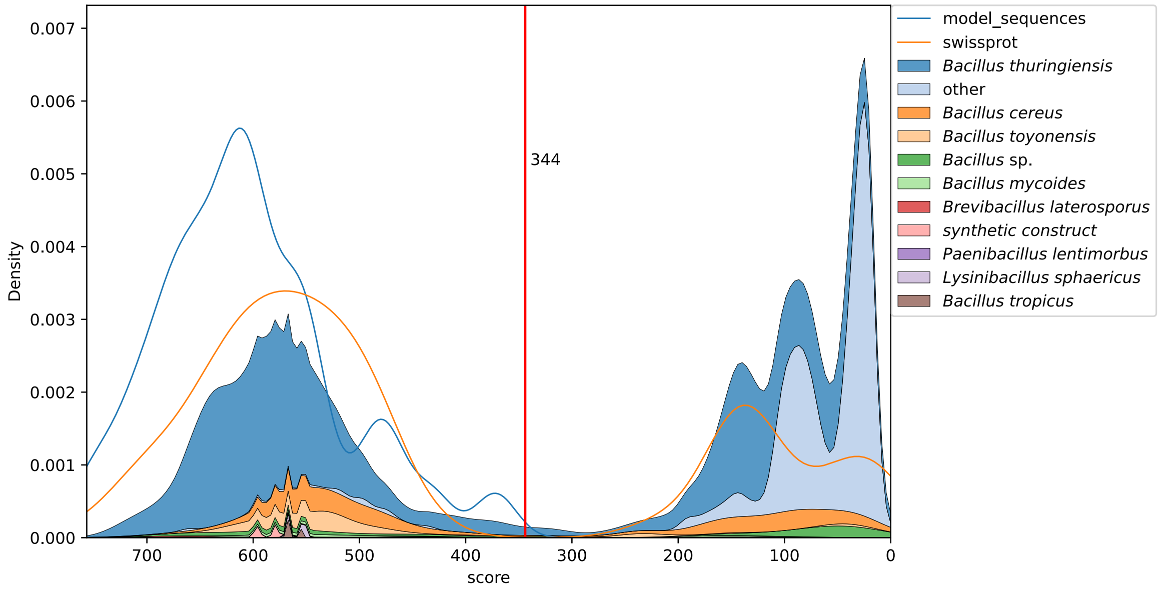

Figure S2. Kernel density plot depicting distribution of model matches in UniProtKb The profile HMM identifies sequences that belong to the Cry group, specifically those with the conserved 3 Domains. The red line marks the curated gathering cutoff.

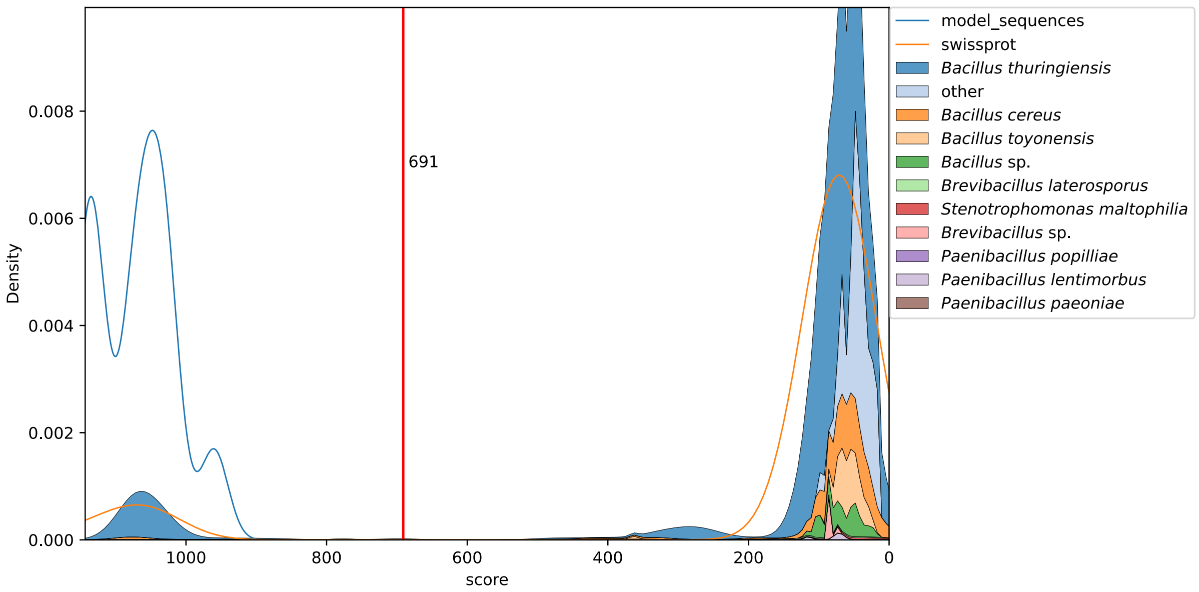

Figure S3. Kernel density plot depicting distribution of model matches in UniProtKb The profile HMM identifies sequences that belong to the Cry group, specifically those closer to the Cry2/Cry18 subgroup. The red line marks the curated gathering cutoff.

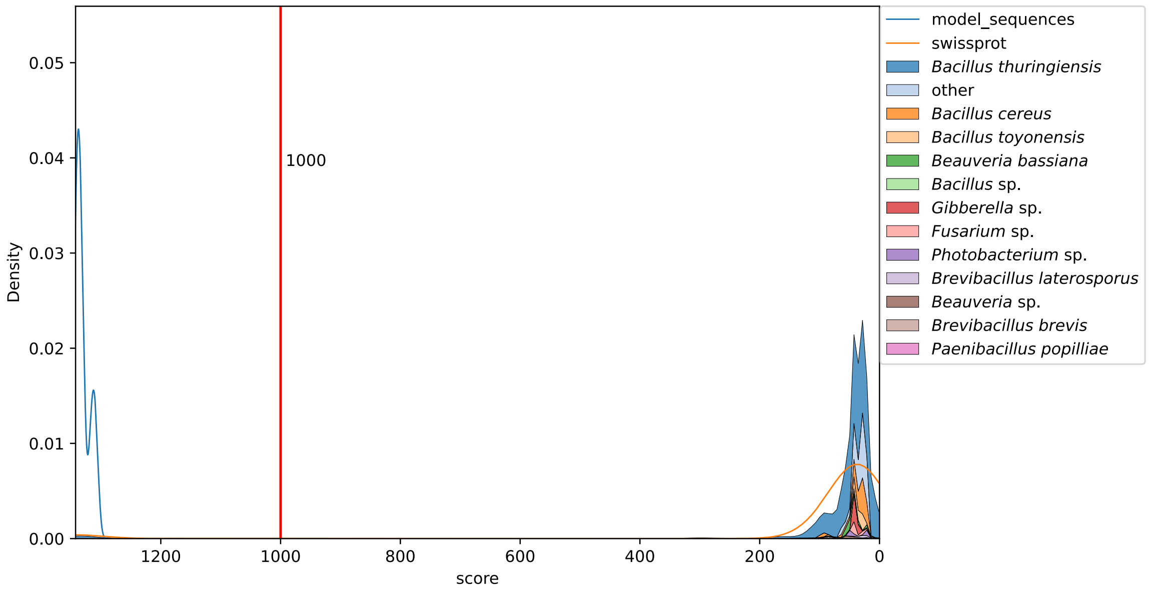

Figure S4. Kernel density plot depicting distribution of model matches in UniProtKb The profile HMM identifies sequences that belong to the Cry group, specifically those closer to the Cry11 subgroup. The red line marks the curated gathering cutoff.

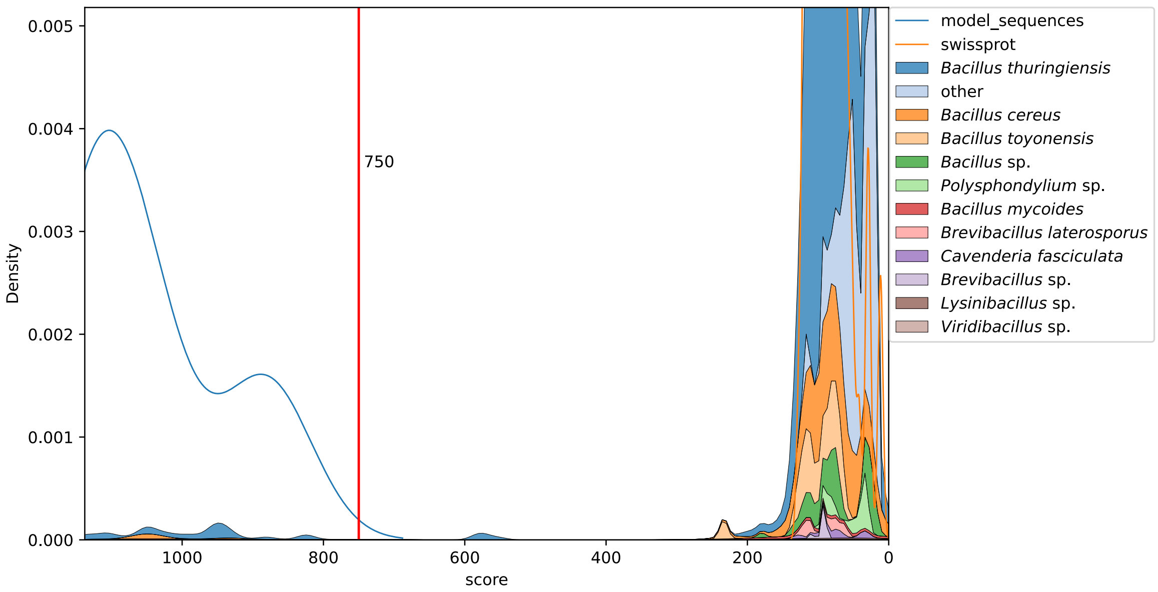

Figure S5. Kernel density plot depicting distribution of model matches in UniProtKb The profile HMM identifies sequences that belong to the Cry group, specifically those closer to the Cry31/60/63 subgroup. The red line marks the curated gathering cutoff.

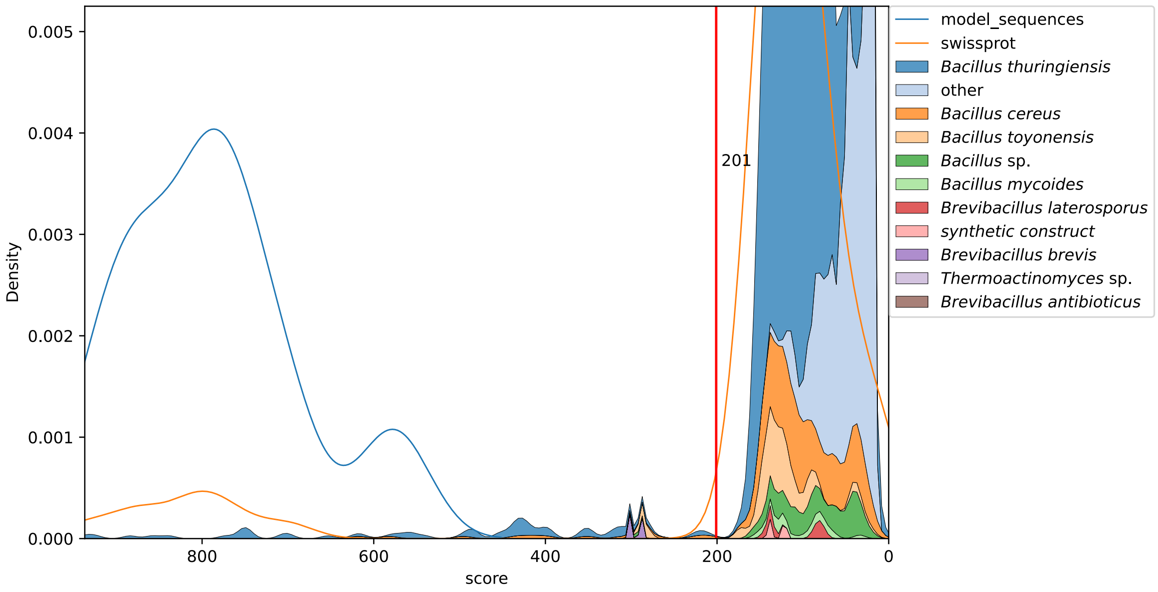

Figure S6. Kernel density plot depicting distribution of model matches in UniProtKb The profile HMM identifies sequences that belong to the Cry group, specifically those with less conserved versions of the classical 3 Domains of the Cry proteins. The red line marks the curated gathering cutoff.

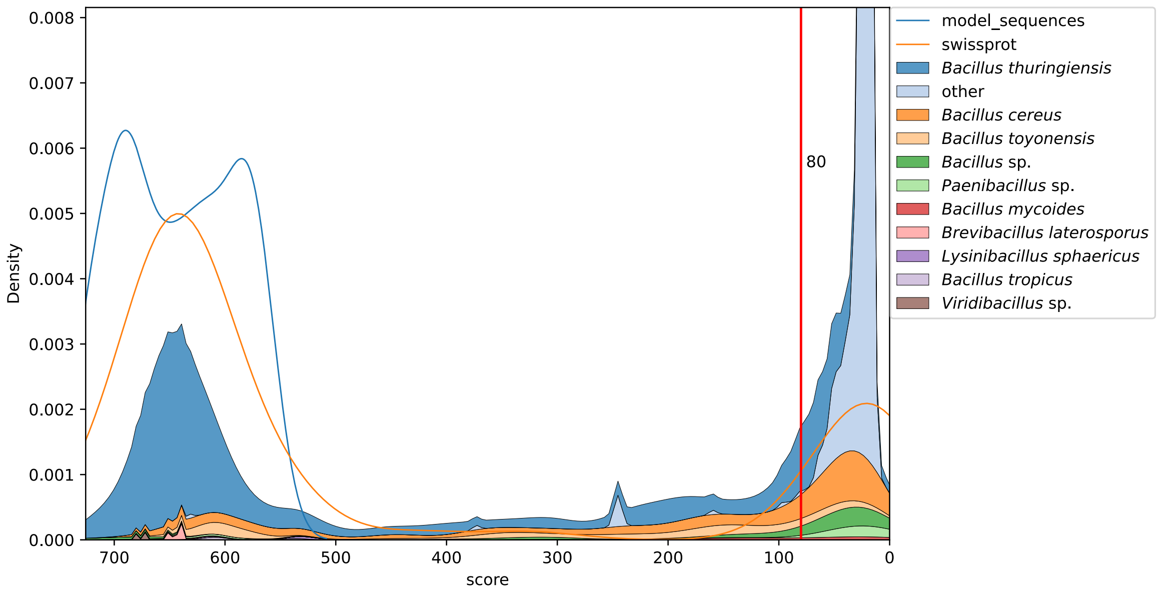

Figure S7. Kernel density plot depicting distribution of model matches in UniProtKb The profile HMM identifies sequences corresponding the C-terminal portion of the pesticidal Cry proteins. The red line marks the curated gathering cutoff.

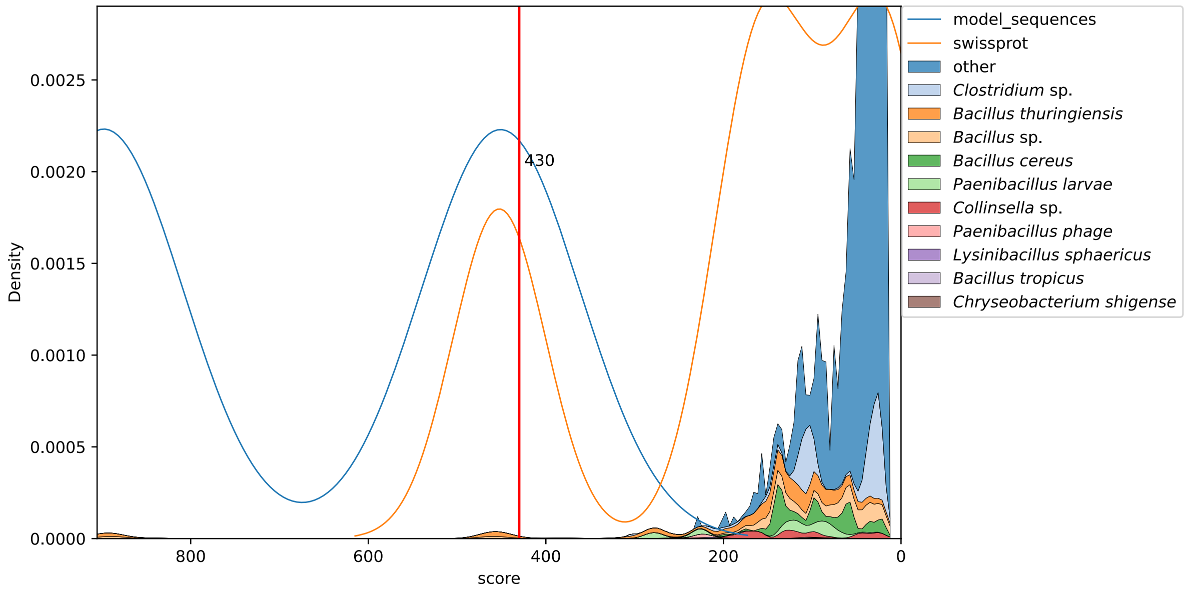

Figure S8. Kernel density plot depicting distribution of model matches in UniProtKb The profile HMM identifies sequences that belong to the Cyt group, specially those of Cyt1 subgroup. The red line marks the curated gathering cutoff.

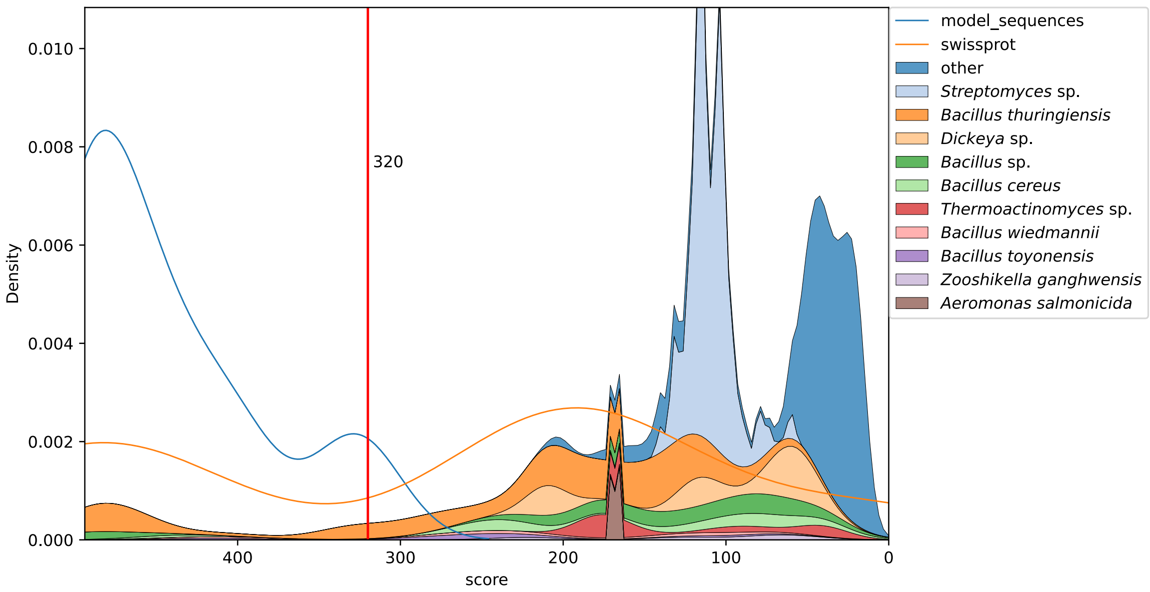

Figure S9. Kernel density plot depicting distribution of model matches in UniProtKb The profile HMM identifies sequences that belong to the Cyt group, specially those of Cyt2 subgroup. The red line marks the curated gathering cutoff.

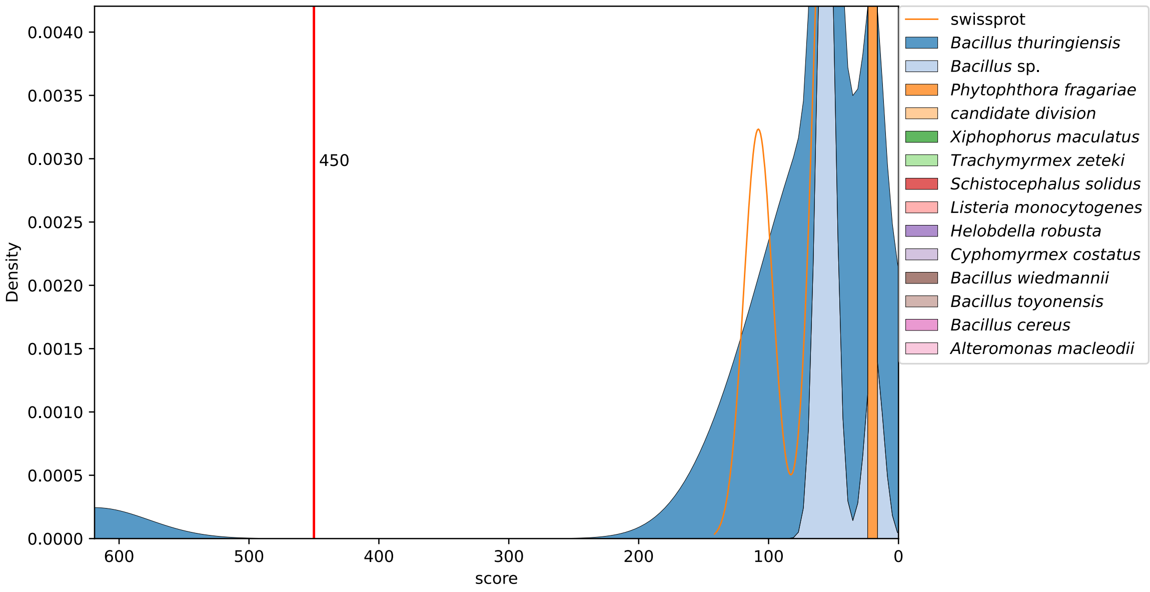

Figure S10. Kernel density plot depicting distribution of model matches in UniProtKb The profile HMM identifies sequences that belong to the Cyt group, specially those of Cyt3 subgroup. The red line marks the curated gathering cutoff.

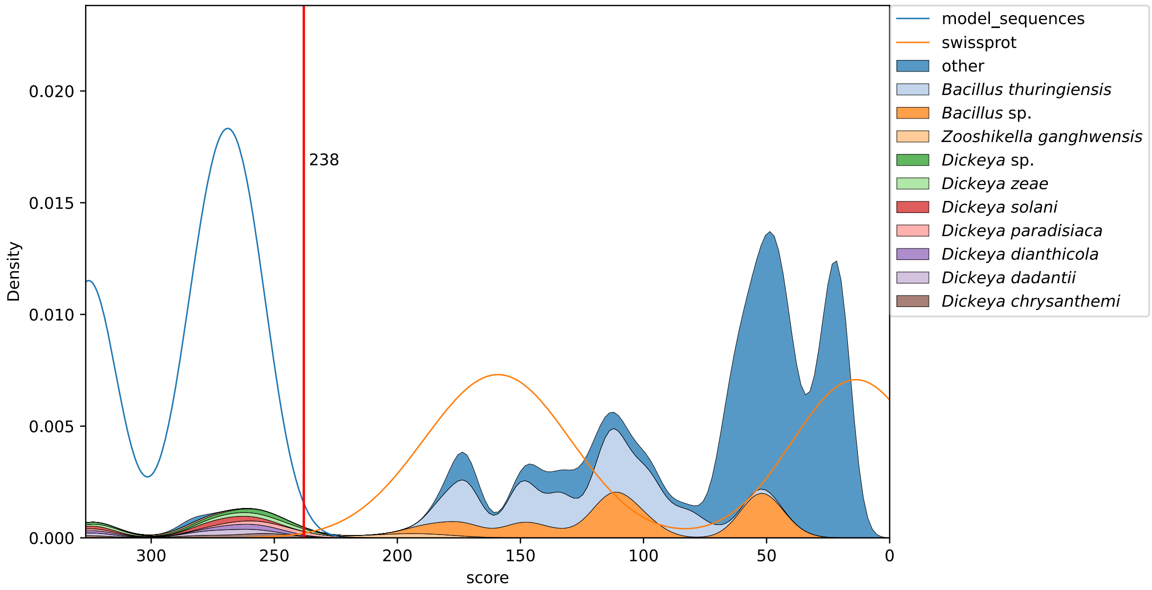

Figure S11. Kernel density plot depicting distribution of model matches in UniProtKb The profile HMM identifies sequences that belong to the Cyt group, specially those related to Cyt4/5/6/7 produced by *Dickeya* spp. The red line marks the curated gathering cutoff.

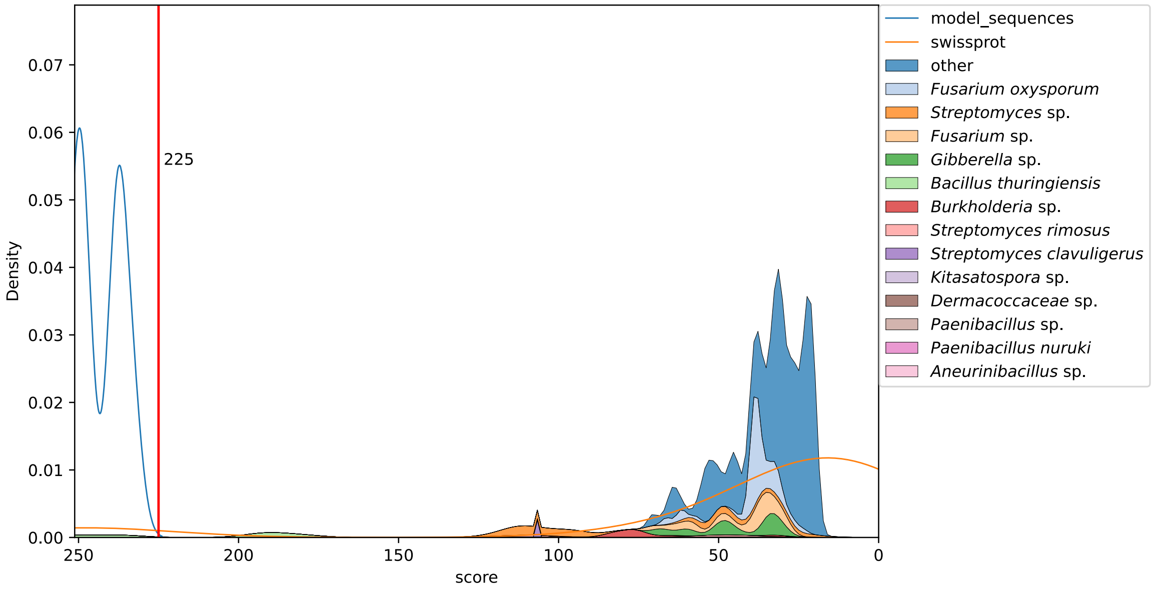

Figure S12. Kernel density plot depicting distribution of model matches in UniProtKb The profile HMM identifies sequences that belong to the Gpp group. The red line marks the curated gathering cutoff.

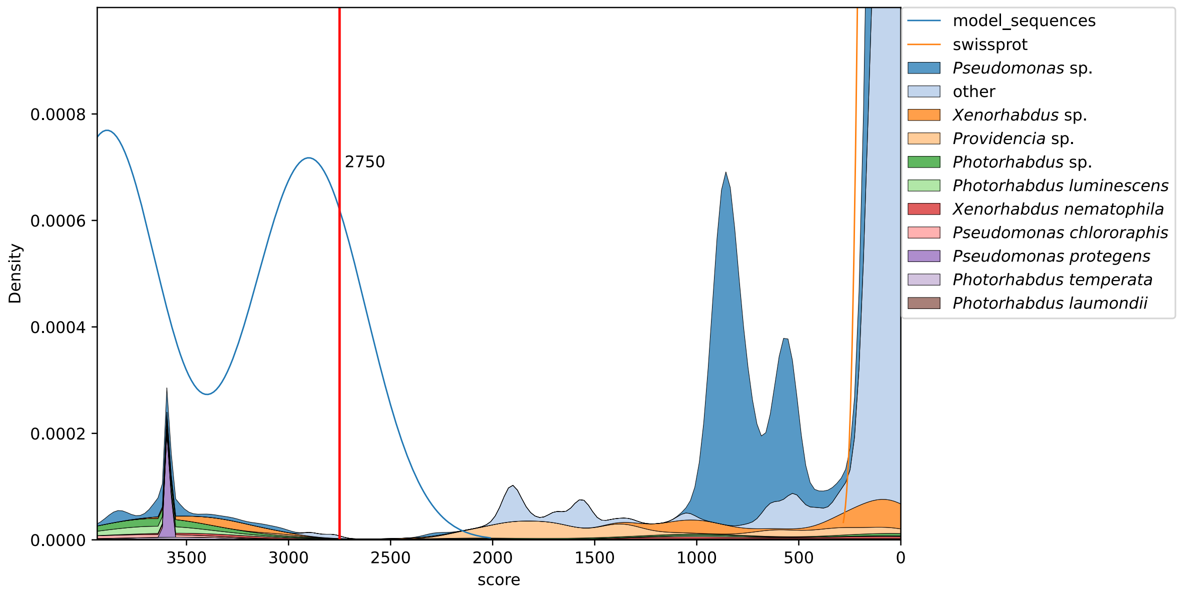

Figure S13. Kernel density plot depicting distribution of model matches in UniProtKb The profile HMM identifies sequences that belong to the Mcf group. The red line marks the curated gathering cutoff.

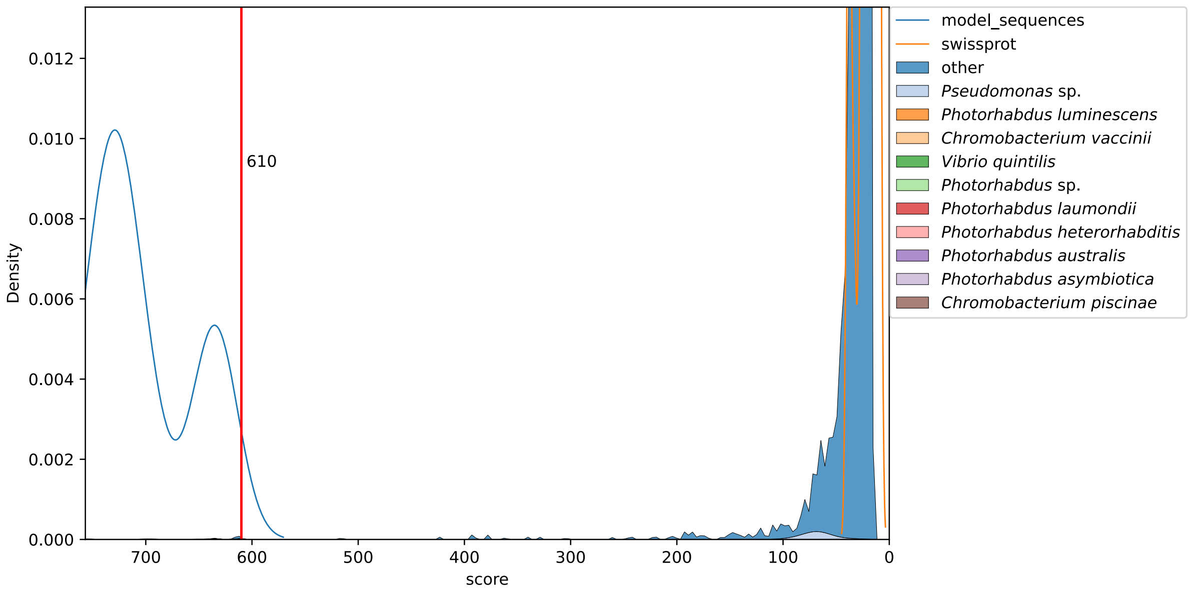

Figure S14. Kernel density plot depicting distribution of model matches in UniProtKb The profile HMM identifies sequences that belong to the Mpf group. The red line marks the curated gathering cutoff.

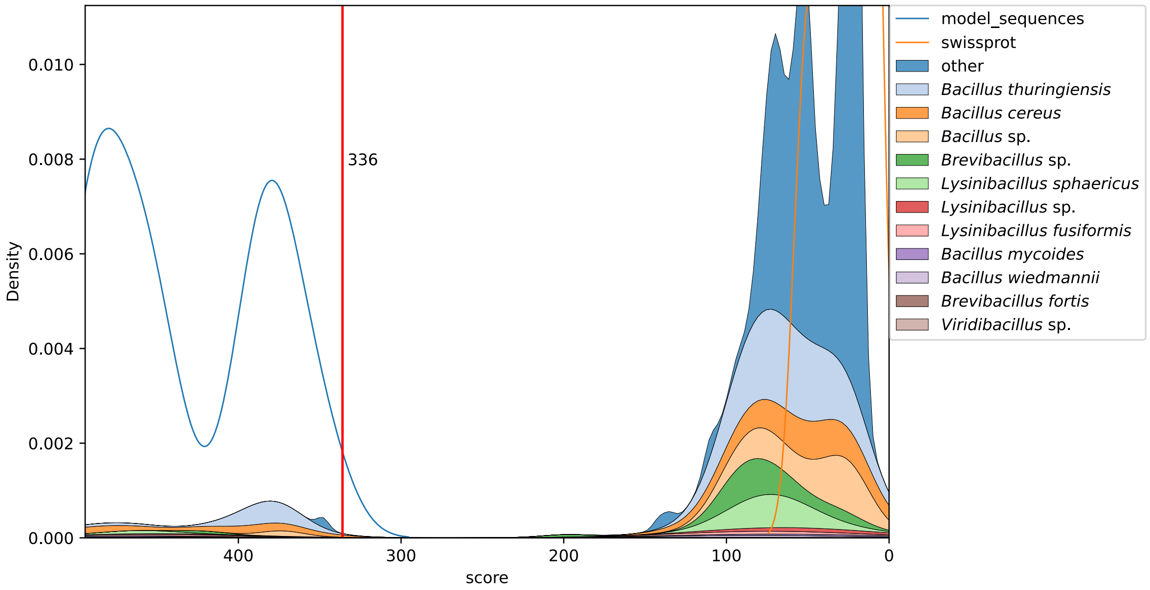

Figure S15. Kernel density plot depicting distribution of model matches in UniProtKb The profile HMM identifies sequences that belong to the Mpp group, specially those related to the toxin originally identified as Sip1. The red line marks the curated gathering cutoff.

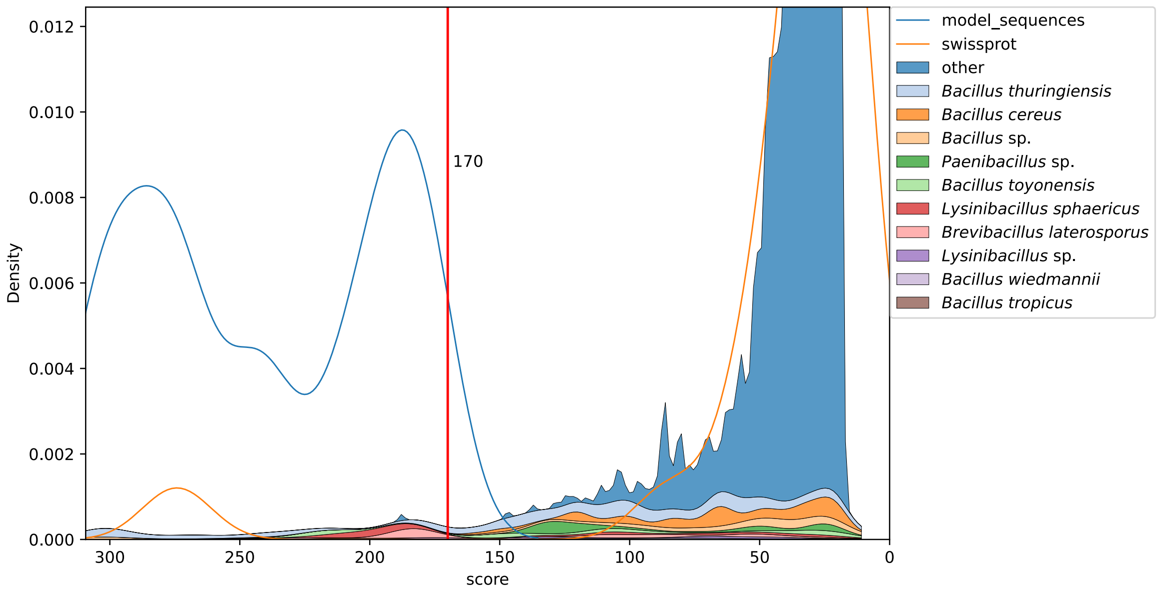

Figure S16. Kernel density plot depicting distribution of model matches in UniProtKb The profile HMM identifies sequences that belong to the Mpp group. The red line marks the curated gathering cutoff.

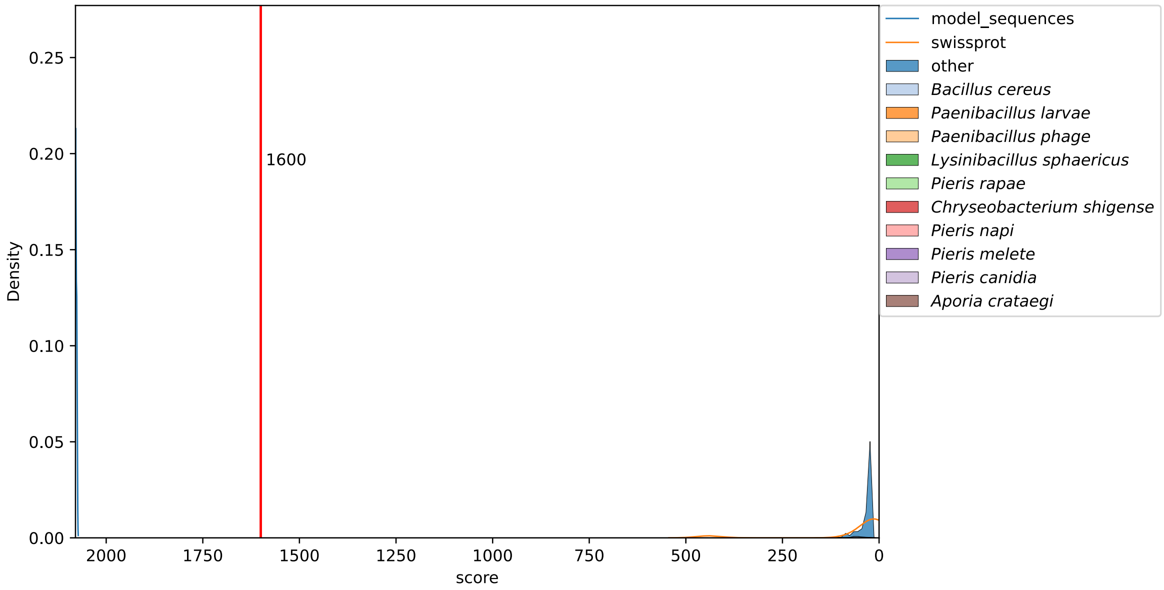

Figure S17. Kernel density plot depicting distribution of model matches in UniProtKb The profile HMM identifies sequences that belong to the Mtx group. The red line marks the curated gathering cutoff.

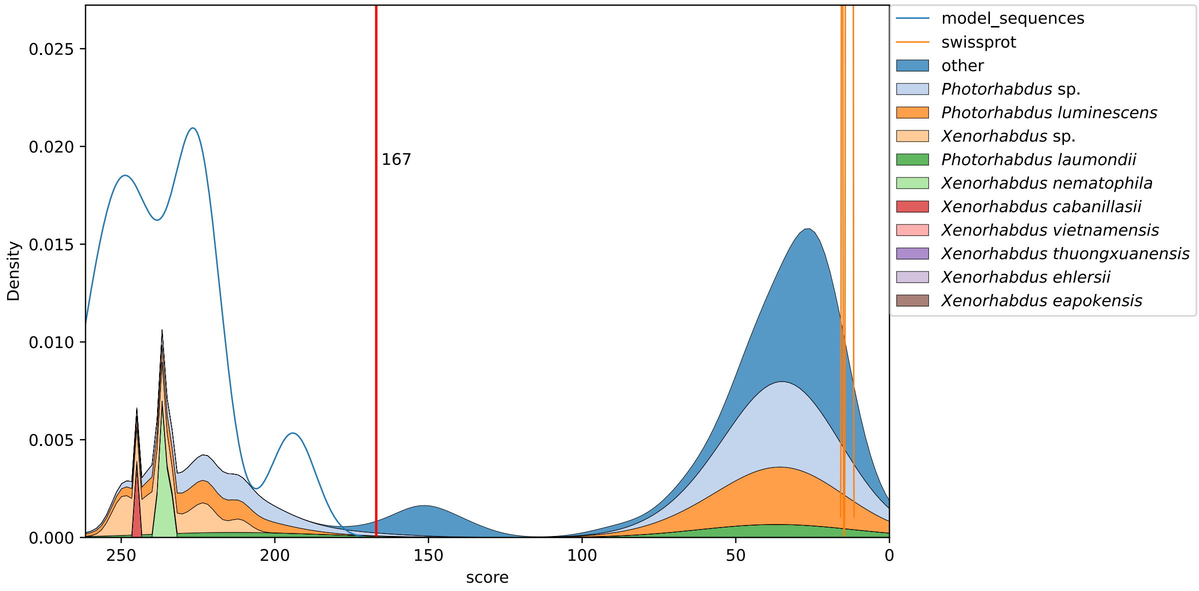

Figure S18. Kernel density plot depicting distribution of model matches in UniProtKb The profile HMM identifies sequences that belong to the Pra group. The red line marks the curated gathering cutoff.

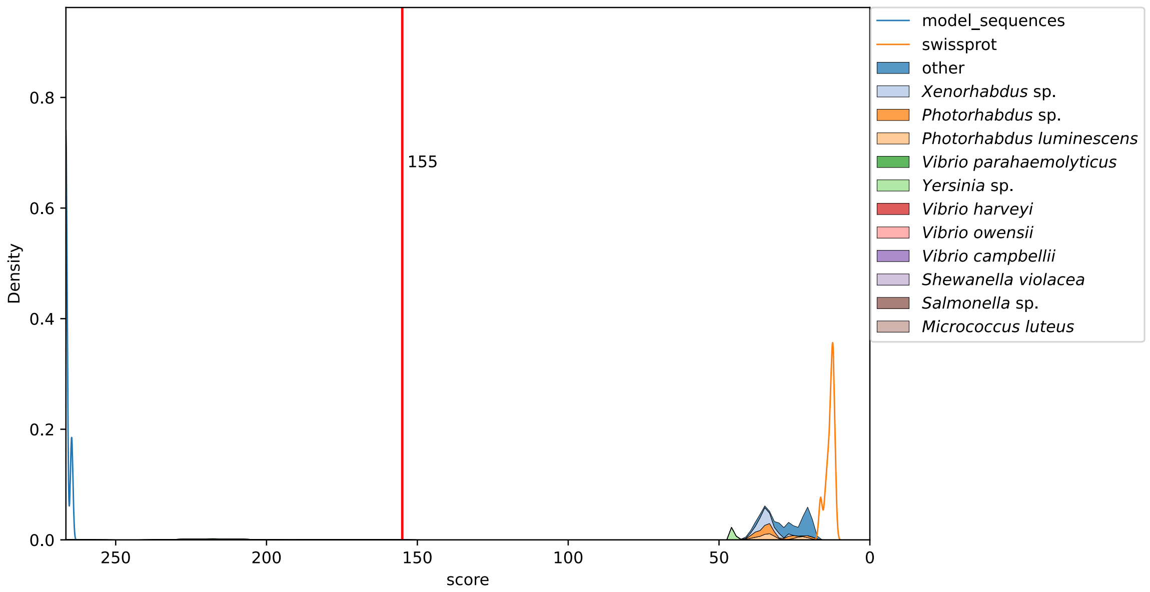

Figure S19. Kernel density plot depicting distribution of model matches in UniProtKb The profile HMM identifies sequences that belong to the Pra group, specially those produced by *Vibrio* spp. The red line marks the curated gathering cutoff.

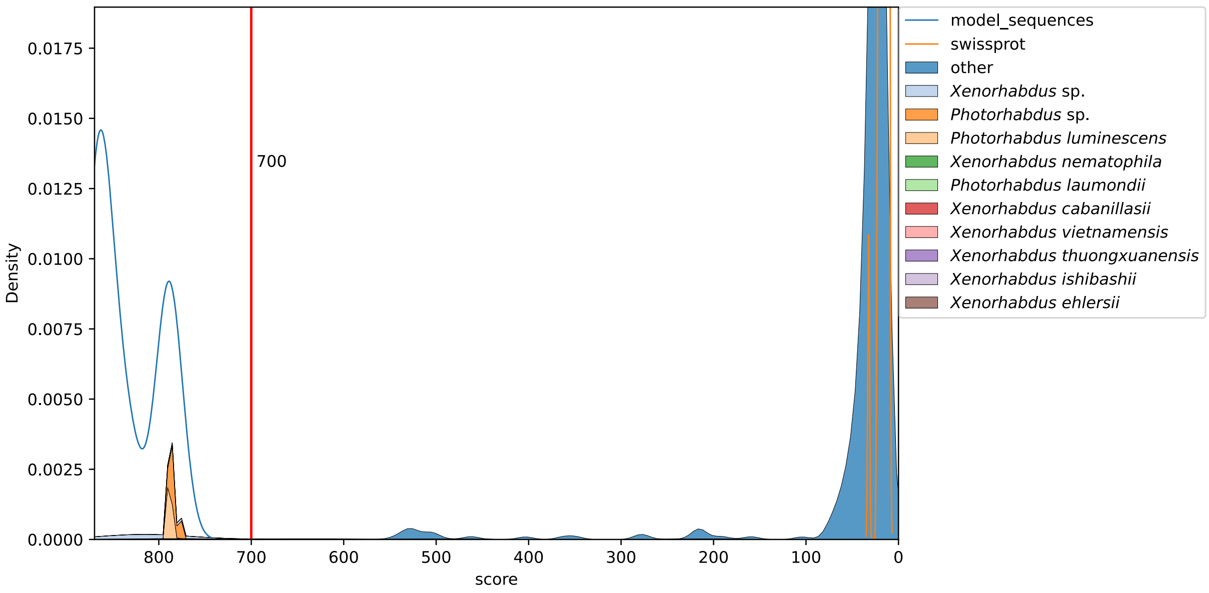

Figure S20. Kernel density plot depicting distribution of model matches in UniProtKb The profile HMM identifies sequences that belong to the Prb group. The red line marks the curated gathering cutoff.

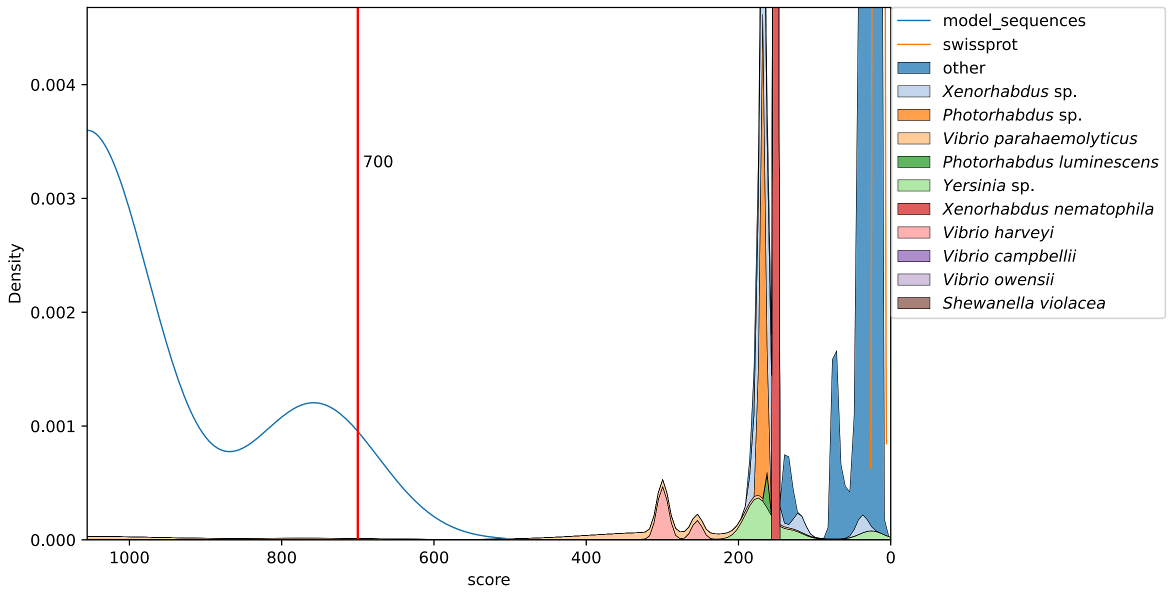

Figure S21. Kernel density plot depicting distribution of model matches in UniProtKb The profile HMM identifies sequences that belong to the Prb group, specially those produced by *Vibrio* spp. The red line marks the curated gathering cutoff.

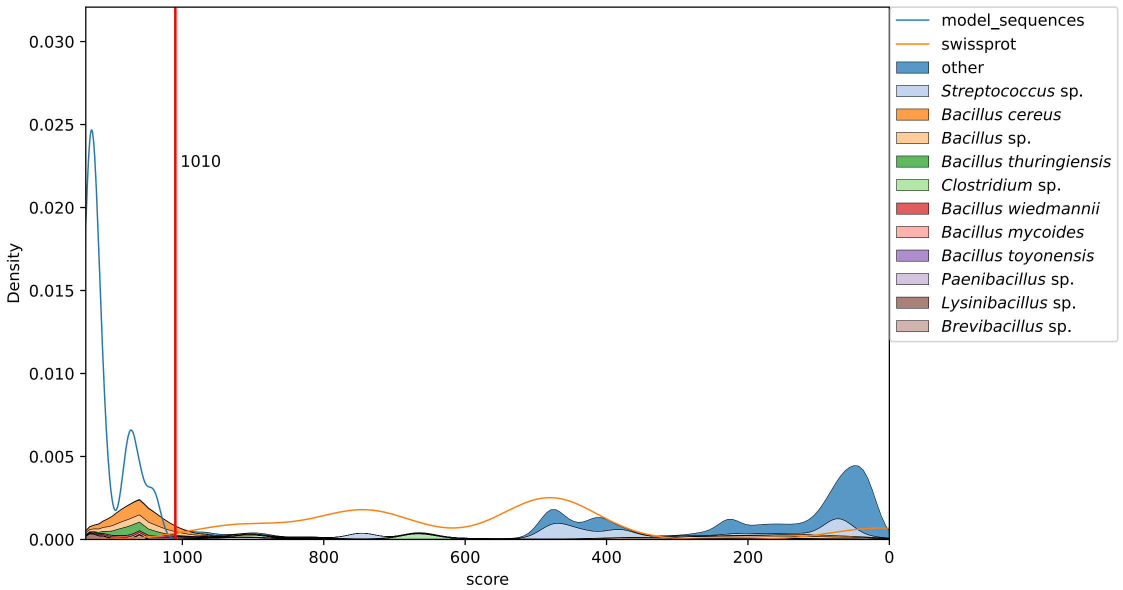

Figure S22. Kernel density plot depicting distribution of model matches in UniProtKb The profile HMM identifies sequences that belong to the Spp group. The red line marks the curated gathering cutoff.

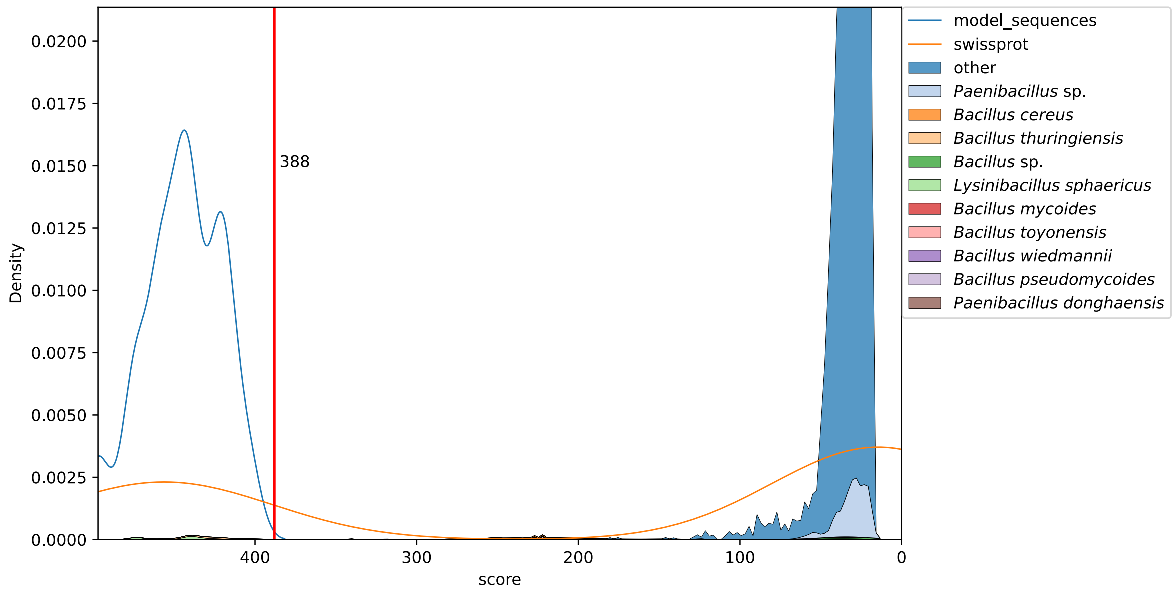

Figure S23. Kernel density plot depicting distribution of model matches in UniProtKb The profile HMM identifies sequences that belong to the Tpp group. The red line marks the curated gathering cutoff.

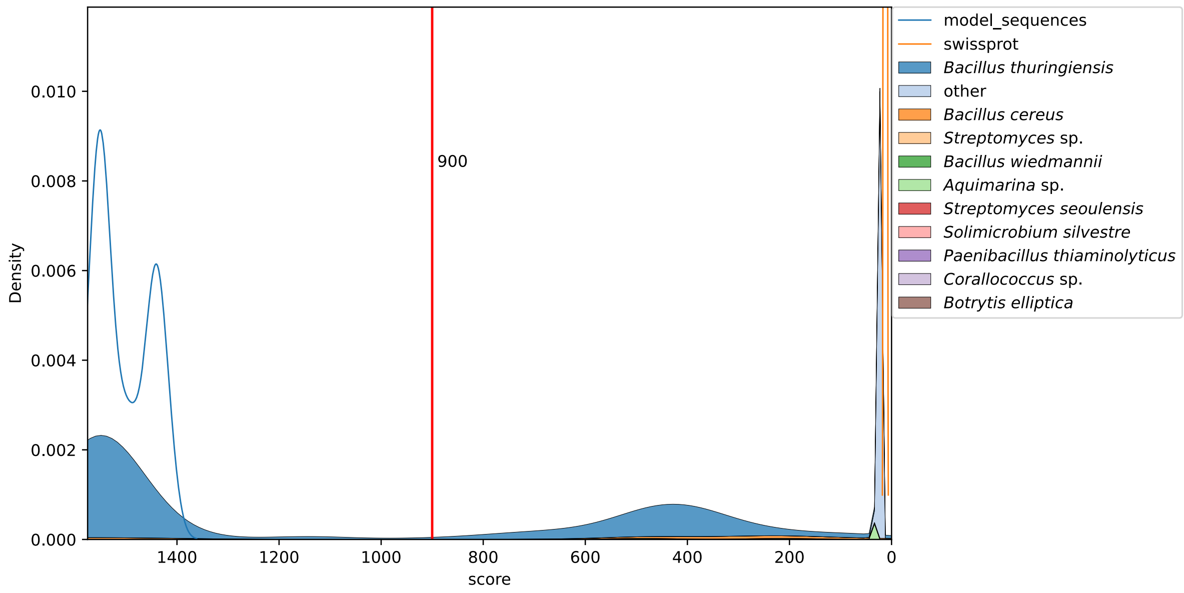

Figure S24. Kernel density plot depicting distribution of model matches in UniProtKb The profile HMM identifies sequences that belong to the Vip group. The red line marks the curated gathering cutoff.

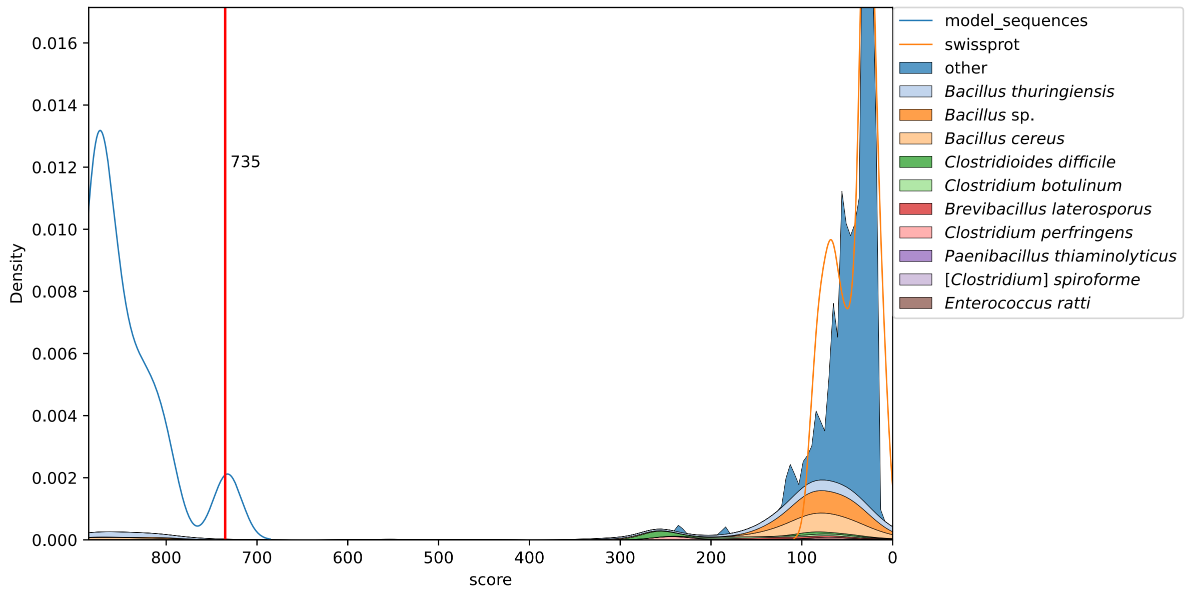

Figure S25. Kernel density plot depicting distribution of model matches in UniProtKb The profile HMM identifies sequences that belong to the Vpa group. The red line marks the curated gathering cutoff.

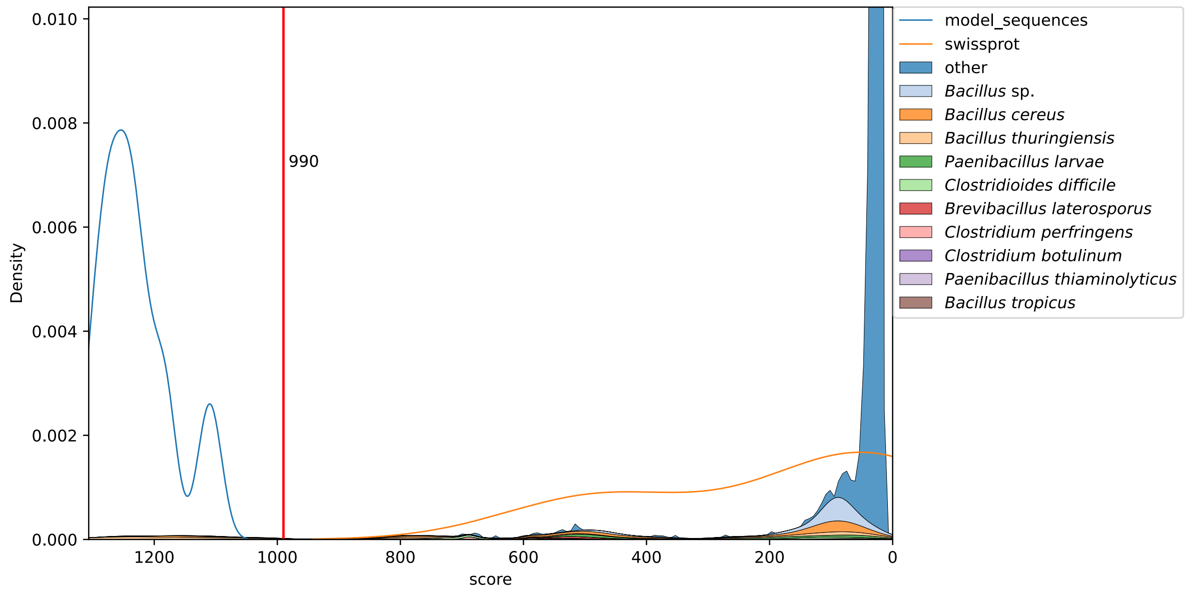

Figure S26. Kernel density plot depicting distribution of model matches in UniProtKb The profile HMM identifies sequences that belong to the Vpb group. The red line marks the curated gathering cutoff.

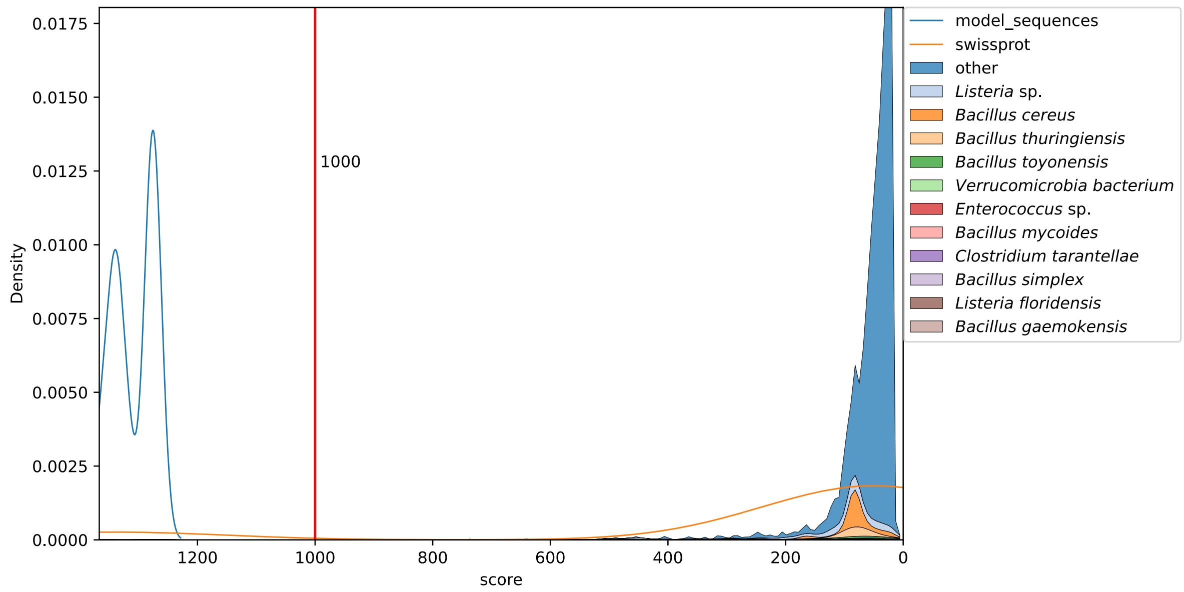

Figure S27. Kernel density plot depicting distribution of model matches in UniProtKb The profile HMM identifies sequences that belong to the Xpp group, in particular those related to Xpp22. The red line marks the curated gathering cutoff.

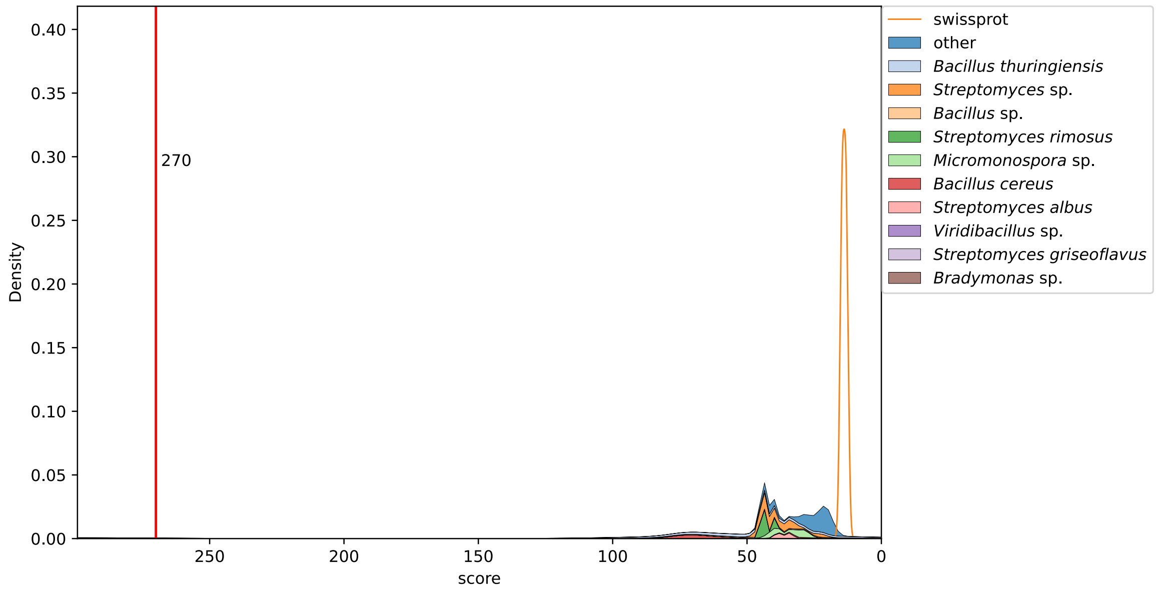

Figure S28. Kernel density plot depicting distribution of model matches in UniProtKb The profile HMM identifies sequences that belong to the Xpp group, in particular those related to Xpp37. The red line marks the curated gathering cutoff.

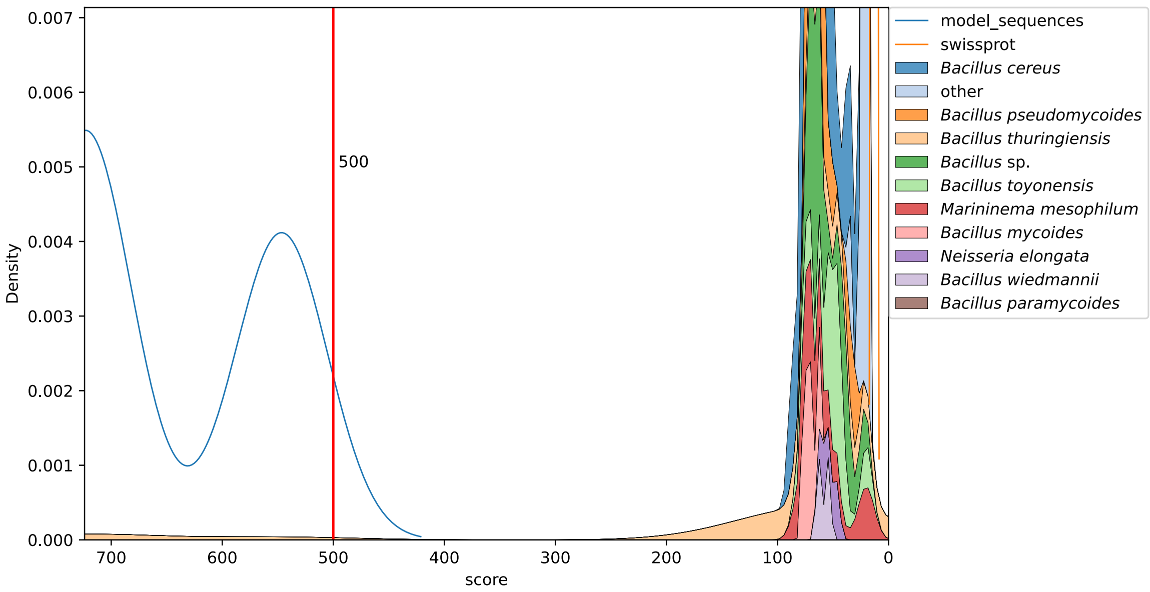

Figure S29. Kernel density plot depicting distribution of model matches in UniProtKb The profile HMM identifies sequences that belong to the Xpp group, in particular those related to Xpp55. The red line marks the curated gathering cutoff.

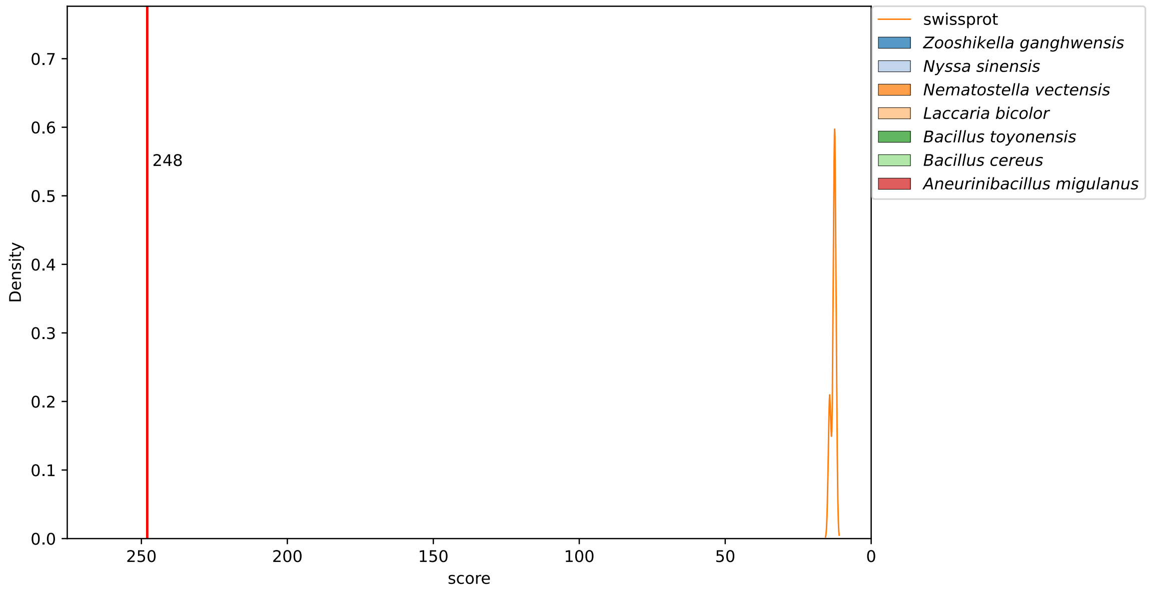

Figure S30. Kernel density plot depicting distribution of model matches in UniProtKb The profile HMM identifies sequences that belong to the Xpp group, in particular those related to Xpp76. The red line marks the curated gathering cutoff.

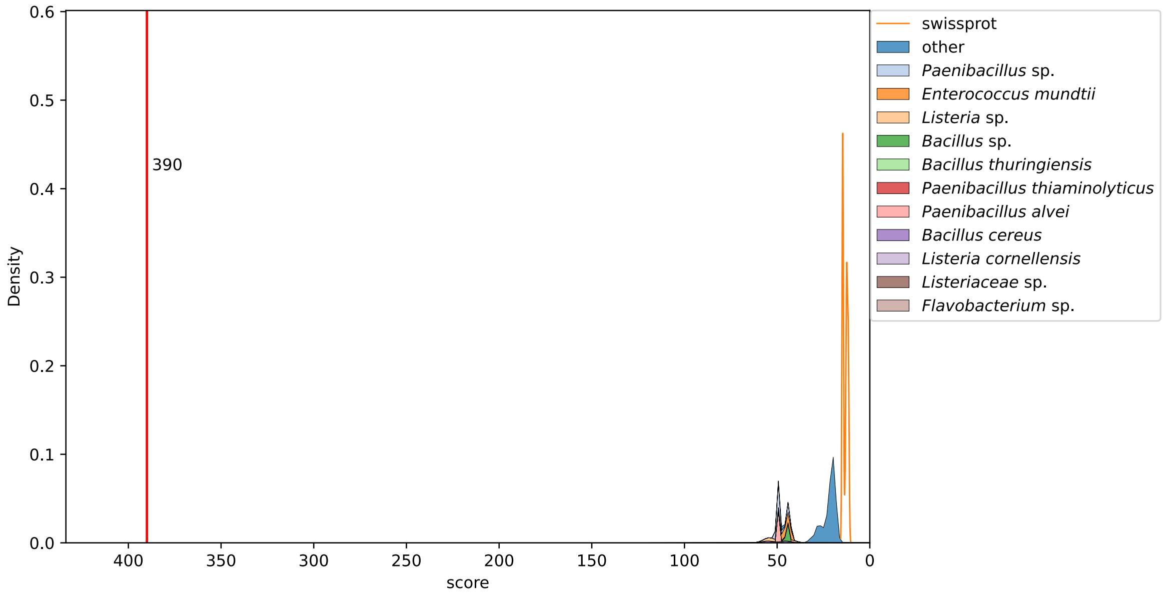

Figure S31. Kernel density plot depicting distribution of model matches in UniProtKb The profile HMM identifies sequences that belong to the Xpp group, in particular those related to Xpp77. The red line marks the curated gathering cutoff.
