# Supplementary material for: IDOPS, a Profile HMM-Based Tool to Detect Pesticidal Sequences and Compare Their Genetic Context
Source: Front Microbiol. 2021 Jun 28;12:664476. doi: 10.3389/fmicb.2021.664476 (PMC8279765; doi:10.3389/fmicb.2021.664476)
Supplement: Supplementary file 4 [file Data_Sheet_3.PDF]

Installation instructions

```
conda install
```

Make idops conda environment with local package:

```
conda create -n idops -c gamb-go idops
```

Test installation with:

```
conda activate idops
idops --help
```

Other dependencies

Not included in the conda requirements is prokka. Please make sure to have a version of prokka installed with databases configured. Please find idops arguments with idops --help

Run IDOPS

Recommended options:

```
idops -o idops_out -i -t   *.gbk
```

-o: output folder -i: analyse and plot genomic environment -t: add NearestNeighbor to output table and save trees of closest sequences from the official database

General Usage:

```
usage: idops [-h] [-v] [-o OUTPUT] [-d] [-i] [-c
value] [-k] [-t] sequence_file [sequence_file ...]
```

IDentification Of Pesticidal Sequences

positional arguments:

  sequence_file   File(s) containing input sequences. Supported formats: *.faa [Protein], *.fasta [Protein], *.gbk [DNA with CDS Features]

optional arguments:

  -h, --help   show this help message and exit

  -v, --verbosity   increase output verbosity

  -o OUTPUT, --output OUTPUT

                        Output directory, default 'IDOPS_{DATE_TIME}'

  -d, --disable_tc   No cutoff for hmmscan

  -i, --identify-conserved-env

                        Analyze genomic environment of hits (requires properly configured prokka) and plot Easyfig

  -c value, --cluster-cutoff value

                        Uses the value as distance cutoff for annotation distance single linkage clustering:1 (one cluster) >= value >= 0 (no clusters) , default 0.6

  -k, --keep-annotations

                        switches off the reannotation with Prokka. BEWARE: for proper genomic analysis it is crucial that the annotations are done by the same tool!

  -t, --tree   Create phylogenetic tree for each hit with the 10 closest sequences of the corresponding protein group. Adds the column 'NearestNeighbor' in summary table.
