# Supplementary material for: IDOPS, a Profile HMM-Based Tool to Detect Pesticidal Sequences and Compare Their Genetic Context
Source: Front Microbiol. 2021 Jun 28;12:664476. doi: 10.3389/fmicb.2021.664476 (PMC8279765; doi:10.3389/fmicb.2021.664476)
Supplement: Supplementary file 2 [file Data_Sheet_1.PDF]

```
HMMER3/f [3.3 | Nov 2019]
NAME  AppM1
DESC  App toxin (IDOPS Model 1)
LENG  409
ALPH  amino
RF    no
MM    no
CONS  yes
CS    no
MAP   yes
DATE  Wed Feb  3 11:08:29 2021
NSEQ  15
EFFN  2.142334
CKSUM 1242355269
GA 335.0 335.0
STATS LOCAL MSV       -11.4788  0.69911
STATS LOCAL VITERBI   -12.3608  0.69911
STATS LOCAL FORWARD    -5.6417  0.69911
HMM          A        C        D        E        F        G        H
I        K        L        M        N        P        Q        R        S
T        V        W        Y
            m->m     m->i     m->d     i->m     i->i     d->m     d->d
  COMPO   2.58331  4.76967  2.78352  2.51401  3.54802  3.14121  3.80609
2.67489  2.49753  2.37823  3.74528  3.00040  3.57560  2.87011  3.02423
2.67171  2.85893  2.71826  4.73463  3.60657
          2.68628  4.42259  2.77527  2.73135  3.46387  2.40546  3.72528
3.29344  2.67754  2.69285  4.24235  2.90351  2.73773  3.18103  2.89810
2.37907  2.77502  2.98552  4.58510  3.61484
          0.72082  1.62532  1.14950  1.63352  0.21721  0.00000        *
      1   2.29803  4.26770  3.82698  3.28805  3.50408  3.58463  4.08375
2.71353  3.20469  2.51966  1.85138  3.57406  4.05555  3.49020  3.48766
2.26198  2.90587  2.18073  5.00608  3.79664     11 m - - -
          2.68618  4.42225  2.77519  2.73123  3.46354  2.40513  3.72494
3.29354  2.67741  2.69355  4.24690  2.90347  2.73739  3.18146  2.89801
2.37887  2.77519  2.98518  4.58477  3.61503
          0.02037  4.29947  5.02181  0.61958  0.77255  0.97767  0.47190
      2   2.34955  4.42006  3.64154  3.08623  3.52254  3.72159  3.99418
1.88946  2.98188  2.16941  3.45737  3.48082  4.10227  2.25705  3.30933
2.98793  2.97200  2.49780  5.03965  3.81365     12 i - - -
          2.68618  4.42225  2.77519  2.73123  3.46354  2.40513  3.72494
3.29354  2.67741  2.69355  4.24690  2.90347  2.73739  3.18146  2.89801
2.37887  2.77519  2.98518  4.58477  3.61503
          0.02037  4.29947  5.02181  0.61958  0.77255  0.97767  0.47190
      3   2.35041  4.12466  4.28349  3.69595  2.46566  3.82649  4.15081
2.03172  3.55933  1.90947  3.21336  3.85774  4.18953  3.75992  3.70251
2.70239  2.92390  1.97949  4.76932  3.58155     13 l - - -
          2.68618  4.42225  2.77519  2.73123  3.46354  2.40513  3.72494
3.29354  2.67741  2.69355  4.24690  2.90347  2.73739  3.18146  2.89801
2.37887  2.77519  2.98518  4.58477  3.61503
          0.02037  4.29947  5.02181  0.61958  0.77255  0.97767  0.47190
      4   2.64022  4.89958  2.36872  2.47457  4.31751  2.59643  3.74208
3.75781  2.54935  3.32961  4.12239  2.98896  2.58740  2.88136  3.02597
2.14195  2.91436  2.85980  5.53595  4.17756     14 s - - -
```

```
         2.68618  4.42225  2.77519  2.73123  3.46354  2.40513  3.72494
3.29354  2.67741  2.69355  4.24690  2.90347  2.73739  3.18146  2.89801
2.37887  2.77519  2.98518  4.58477  3.61503
         0.02037  4.29947  5.02181  0.61958  0.77255  0.97767  0.47190
      5  1.69935  4.58567  3.22686  2.74713  4.00889  3.39390  3.87968
3.39170  2.75014  2.51525  3.91065  2.43371  3.90108  3.08893  3.16286
2.18280  2.89387  3.06629  5.35066  4.06480     15 a - - -
         2.68655  4.42262  2.77528  2.73047  3.46390  2.40549  3.72531
3.29278  2.67711  2.69370  4.24726  2.90311  2.73755  3.18144  2.89837
2.37855  2.77488  2.98555  4.58513  3.61540
         0.47685  0.98706  5.02181  1.49352  0.25435  0.97767  0.47190
      6  2.65521  4.85111  3.03149  2.27571  4.10115  2.50254  3.68569
3.51400  2.14022  2.70582  3.93299  3.00914  3.87026  2.83705  2.92495
2.37701  2.88884  2.81049  5.35986  4.02785     21 k - - -
         2.68618  4.42225  2.77519  2.73123  3.46354  2.40513  3.72494
3.29354  2.67741  2.69355  4.24690  2.90347  2.73739  3.18146  2.89801
2.37887  2.77519  2.98518  4.58477  3.61503
         0.02037  4.29947  5.02181  0.61958  0.77255  0.97767  0.47190
      7  2.41525  4.93591  2.96541  2.47227  4.31074  3.42180  3.70931
3.74638  2.09468  3.30992  4.10242  2.25795  3.86270  2.84347  2.92485
2.40119  2.16305  3.36917  5.50818  4.15564     22 k - - -
         2.68618  4.42225  2.77519  2.73123  3.46354  2.40513  3.72494
3.29354  2.67741  2.69355  4.24690  2.90347  2.73739  3.18146  2.89801
2.37887  2.77519  2.98518  4.58477  3.61503
         0.02037  4.29947  5.02181  0.61958  0.77255  0.97767  0.47190
      8  2.83531  5.32095  2.25385  1.70500  4.63471  3.39801  3.75279
4.11686  2.60394  3.62284  4.40922  2.39090  3.90072  2.88356  3.12842
2.77642  1.96995  3.70119  5.78430  4.35015     23 e - - -
         2.68618  4.42225  2.77519  2.73123  3.46354  2.40513  3.72494
3.29354  2.67741  2.69355  4.24690  2.90347  2.73739  3.18146  2.89801
2.37887  2.77519  2.98518  4.58477  3.61503
         0.02037  4.29947  5.02181  0.61958  0.77255  0.97767  0.47190
      9  3.46052  4.73912  5.20170  4.65279  3.31141  4.80436  5.18379
1.85098  4.51814  0.82809  3.08482  4.87835  4.97363  4.60552  4.61191
4.17655  3.69043  1.88320  5.43011  4.36906     24 l - - -
         2.68618  4.42225  2.77519  2.73123  3.46354  2.40513  3.72494
3.29354  2.67741  2.69355  4.24690  2.90347  2.73739  3.18146  2.89801
2.37887  2.77519  2.98518  4.58477  3.61503
         0.02037  4.29947  5.02181  0.61958  0.77255  0.97767  0.47190
     10  3.42193  5.08398  4.07742  3.99213  4.91690  3.74854  5.01414
4.59908  4.09141  4.16687  5.24835  4.24665  0.31557  4.46523  4.26886
3.60719  3.89973  4.19021  5.96121  5.04967     25 P - - -
         2.68618  4.42225  2.77519  2.73123  3.46354  2.40513  3.72494
3.29354  2.67741  2.69355  4.24690  2.90347  2.73739  3.18146  2.89801
2.37887  2.77519  2.98518  4.58477  3.61503
         0.02037  4.29947  5.02181  0.61958  0.77255  0.97767  0.47190
     11  2.47685  5.08737  3.02446  2.45877  4.41908  3.48921  3.64586
3.85867  1.82819  3.37167  4.15069  2.98170  3.87968  2.40892  2.27931
2.42835  2.61780  3.47483  5.52243  4.17343     26 k - - -
         2.68618  4.42225  2.77519  2.73123  3.46354  2.40513  3.72494
3.29354  2.67741  2.69355  4.24690  2.90347  2.73739  3.18146  2.89801
2.37887  2.77519  2.98518  4.58477  3.61503
         0.02037  4.29947  5.02181  0.61958  0.77255  0.97767  0.47190
```

```
     12   2.09866  4.36807  3.58287  3.02962  3.51765  3.63261  2.31795
 2.49221  2.95682  2.56639  3.49358  3.41661  4.03471  3.27682  3.29381
 2.90060  2.90798  2.06630  4.98744  3.74486     27 v - - -
          2.68618  4.42225  2.77519  2.73123  3.46354  2.40513  3.72494
 3.29354  2.67741  2.69355  4.24690  2.90347  2.73739  3.18146  2.89801
 2.37887  2.77519  2.98518  4.58477  3.61503
          0.02037  4.29947  5.02181  0.61958  0.77255  0.97767  0.47190
     13   2.67222  4.26672  3.92407  3.37524  3.49783  3.69302  4.14422
 2.05920  3.29417  2.49697  3.45907  3.66090  4.13184  3.57081  3.56892
 2.35957  2.05725  1.80582  5.02045  3.80988     28 v - - -
          2.68623  4.42230  2.77524  2.73128  3.46358  2.40517  3.72499
 3.29359  2.67745  2.69324  4.24694  2.90351  2.73744  3.18151  2.89762
 2.37891  2.77524  2.98523  4.58482  3.61508
          0.29198  1.39990  5.02181  0.19103  1.74933  0.97767  0.47190
     14   3.56323  4.83689  5.24343  4.71191  3.24841  4.87616  5.21466
 1.73951  4.53713  0.74725  3.03552  4.95008  5.02221  4.62487  4.61723
 4.27012  3.79065  2.28314  5.40475  4.33146     30 l - - -
          2.68618  4.42225  2.77519  2.73123  3.46354  2.40513  3.72494
 3.29354  2.67741  2.69355  4.24690  2.90347  2.73739  3.18146  2.89801
 2.37887  2.77519  2.98518  4.58477  3.61503
          0.02037  4.29947  5.02181  0.61958  0.77255  0.97767  0.47190
     15   2.27443  5.14256  2.49664  2.36762  4.45983  3.43479  2.43143
 3.92508  2.04397  3.43216  4.19846  2.91250  3.85213  2.47425  2.86718
 2.67478  2.93860  3.52054  5.58569  4.19416     31 k - - -
          2.68618  4.42225  2.77519  2.73123  3.46354  2.40513  3.72494
 3.29354  2.67741  2.69355  4.24690  2.90347  2.73739  3.18146  2.89801
 2.37887  2.77519  2.98518  4.58477  3.61503
          0.02037  4.29947  5.02181  0.61958  0.77255  0.97767  0.47190
     16   2.67644  4.20615  3.94566  3.36912  2.72111  3.74492  2.85340
 2.34112  3.26832  1.75530  3.27044  3.64668  4.11255  3.52022  3.50615
 3.02185  2.36911  2.42668  4.76690  3.53534     32 l - - -
          2.68618  4.42225  2.77519  2.73123  3.46354  2.40513  3.72494
 3.29354  2.67741  2.69355  4.24690  2.90347  2.73739  3.18146  2.89801
 2.37887  2.77519  2.98518  4.58477  3.61503
          0.02037  4.29947  5.02181  0.61958  0.77255  0.97767  0.47190
     17   3.44601  4.71062  5.21035  4.69300  3.40523  4.82003  5.24031
 1.05720  4.53619  1.29593  3.20036  4.90545  5.01302  4.68448  4.64490
 4.21576  3.68574  1.99414  5.50734  4.38332     33 i - - -
          2.68618  4.42225  2.77519  2.73123  3.46354  2.40513  3.72494
 3.29354  2.67741  2.69355  4.24690  2.90347  2.73739  3.18146  2.89801
 2.37887  2.77519  2.98518  4.58477  3.61503
          0.02037  4.29947  5.02181  0.61958  0.77255  0.97767  0.47190
     18   2.64068  4.56151  3.27739  2.73945  2.79275  3.51828  3.80568
 3.15719  2.24605  2.83758  3.70602  3.19481  3.93649  3.04149  3.08142
 2.40060  1.93565  2.89115  5.15029  3.87257     34 t - - -
          2.68631  4.42309  2.77565  2.73146  3.46438  2.40465  3.72579
 3.29405  2.67665  2.69368  4.24774  2.90257  2.73713  3.17996  2.89885
 2.37866  2.77565  2.98576  4.58561  3.61433
          0.94755  0.50134  5.02181  1.85125  0.17084  0.97767  0.47190
     19   2.41668  5.14550  2.10474  2.14278  4.48062  2.62793  3.68832
 3.94955  2.48437  3.46678  4.23801  2.89964  2.99725  2.80813  2.98568
 2.68552  2.55403  3.54071  5.63254  4.22863     51 d - - -
          2.68618  4.42225  2.77519  2.73123  3.46354  2.40513  3.72494
 3.29354  2.67741  2.69355  4.24690  2.90347  2.73739  3.18146  2.89801
 2.37887  2.77519  2.98518  4.58477  3.61503
```

```
          0.02037  4.29947  5.02181  0.61958  0.77255  0.26824  1.44701
     20   2.71836  4.53239  3.91367  3.53054  4.11528  1.88397  4.44849
3.48003  3.48996  3.19519  1.15082  3.73920  4.17015  3.80044  3.77258
2.43953  3.12214  3.15551  5.55930  4.33905     52 m - - -
          2.68618  4.42225  2.77519  2.73123  3.46354  2.40513  3.72494
3.29354  2.67741  2.69355  4.24690  2.90347  2.73739  3.18146  2.89801
2.37887  2.77519  2.98518  4.58477  3.61503
          0.01397  4.67381  5.39616  0.61958  0.77255  0.48576  0.95510
     21   1.59180  4.45879  4.07607  3.58768  4.04064  3.52583  4.43687
3.28646  3.52947  3.10616  3.99984  3.76754  4.15272  3.80091  3.81921
2.02583  2.10061  1.62779  5.49256  4.28943     53 a - - -
          2.68618  4.42225  2.77519  2.73123  3.46354  2.40513  3.72494
3.29354  2.67741  2.69355  4.24690  2.90347  2.73739  3.18146  2.89801
2.37887  2.77519  2.98518  4.58477  3.61503
          0.01397  4.67381  5.39616  0.61958  0.77255  0.48576  0.95510
     22   1.78619  5.23926  2.92226  1.51917  4.62257  3.54846  3.87533
4.07927  2.67629  3.61498  4.39641  2.50531  4.01888  3.00300  3.16665
2.84961  2.52501  3.67291  5.79093  4.39935     54 e - - -
          2.68618  4.42225  2.77519  2.73123  3.46354  2.40513  3.72494
3.29354  2.67741  2.69355  4.24690  2.90347  2.73739  3.18146  2.89801
2.37887  2.77519  2.98518  4.58477  3.61503
          0.01397  4.67381  5.39616  0.61958  0.77255  0.48576  0.95510
     23   2.79954  4.91245  3.25921  2.69259  4.14163  2.87409  3.82909
2.22408  2.25012  3.16137  3.99335  2.76700  4.02566  2.99692  1.94401
2.85669  3.02767  2.86783  5.42063  4.11505     55 r - - -
          2.68618  4.42225  2.77519  2.73123  3.46354  2.40513  3.72494
3.29354  2.67741  2.69355  4.24690  2.90347  2.73739  3.18146  2.89801
2.37887  2.77519  2.98518  4.58477  3.61503
          0.07402  4.67381  2.78046  0.61958  0.77255  0.48576  0.95510
     24   2.53984  5.09974  2.28231  2.50447  4.40219  3.53177  3.76027
2.72150  2.53716  3.39930  4.18095  2.38931  2.90736  2.88458  3.01961
2.10051  2.99462  3.47228  5.59288  4.22019     56 s - - -
          2.68618  4.42225  2.77519  2.73123  3.46354  2.40513  3.72494
3.29354  2.67741  2.69355  4.24690  2.90347  2.73739  3.18146  2.89801
2.37887  2.77519  2.98518  4.58477  3.61503
          0.01483  4.61462  5.33697  0.61958  0.77255  0.42233  1.06571
     25   2.41630  4.75340  3.35652  2.82610  4.06485  1.95498  3.94638
2.87595  2.80776  3.10923  3.95759  3.28918  4.04265  2.56825  3.22985
2.85558  1.85041  3.14547  5.40744  4.12088     57 t - - -
          2.68618  4.42225  2.77519  2.73123  3.46354  2.40513  3.72494
3.29354  2.67741  2.69355  4.24690  2.90347  2.73739  3.18146  2.89801
2.37887  2.77519  2.98518  4.58477  3.61503
          0.01397  4.67381  5.39616  0.61958  0.77255  0.48576  0.95510
     26   3.01412  4.54040  4.38799  3.90335  1.28246  1.83670  4.30122
2.42453  3.80218  2.57483  3.64431  4.05703  4.45458  4.01613  3.98633
3.35394  3.29798  2.73000  4.83337  3.39190     58 f - - -
          2.68618  4.42225  2.77519  2.73123  3.46354  2.40513  3.72494
3.29354  2.67741  2.69355  4.24690  2.90347  2.73739  3.18146  2.89801
2.37887  2.77519  2.98518  4.58477  3.61503
          0.01397  4.67381  5.39616  0.61958  0.77255  0.48576  0.95510
     27   2.84568  4.40627  4.12075  3.57855  3.56292  3.87985  4.28599
1.45931  3.47148  2.24911  3.54191  3.84704  2.01001  3.74953  3.71639
2.68312  3.10790  2.49970  5.09874  3.89077     59 i - - -
```

```
          2.68618  4.42225  2.77519  2.73123  3.46354  2.40513  3.72494
3.29354  2.67741  2.69355  4.24690  2.90347  2.73739  3.18146  2.89801
2.37887  2.77519  2.98518  4.58477  3.61503
          0.39472  4.67381  1.14950  0.61958  0.77255  0.48576  0.95510
     28   3.34409  4.64182  5.08764  4.53523  1.67227  4.60313  4.76797
1.48631  4.39095  1.47921  3.08687  4.66134  4.81709  4.44837  4.44675
3.95159  3.56825  2.29135  5.02573  3.74268     60 l - - -
          2.68618  4.42225  2.77519  2.73123  3.46354  2.40513  3.72494
3.29354  2.67741  2.69355  4.24690  2.90347  2.73739  3.18146  2.89801
2.37887  2.77519  2.98518  4.58477  3.61503
          0.02037  4.29947  5.02181  0.61958  0.77255  0.97767  0.47190
     29   2.41044  4.34898  3.64715  3.33493  4.43190  3.13994  4.38331
3.80454  3.34500  3.52931  4.37294  3.49006  3.88066  3.65623  3.65134
1.48587  1.24252  3.29291  5.78645  4.56649     61 t - - -
          2.68618  4.42225  2.77519  2.73123  3.46354  2.40513  3.72494
3.29354  2.67741  2.69355  4.24690  2.90347  2.73739  3.18146  2.89801
2.37887  2.77519  2.98518  4.58477  3.61503
          0.02037  4.29947  5.02181  0.61958  0.77255  0.97767  0.47190
     30   3.42907  5.35907  3.63054  3.19764  4.84712  3.84502  4.08140
4.31087  0.57208  3.79137  4.74978  3.59472  4.33144  3.26283  2.61892
3.45502  3.65964  4.00201  5.73822  4.62540     62 K - - -
          2.68618  4.42225  2.77519  2.73123  3.46354  2.40513  3.72494
3.29354  2.67741  2.69355  4.24690  2.90347  2.73739  3.18146  2.89801
2.37887  2.77519  2.98518  4.58477  3.61503
          0.02037  4.29947  5.02181  0.61958  0.77255  0.97767  0.47190
     31   3.00710  5.45366  2.55065  1.21102  4.76327  3.45190  3.83367
4.25311  2.61421  3.74097  4.57685  2.93168  3.99263  1.95717  3.05469
2.93378  3.26369  3.85636  5.87489  4.46606     63 e - - -
          2.68618  4.42225  2.77519  2.73123  3.46354  2.40513  3.72494
3.29354  2.67741  2.69355  4.24690  2.90347  2.73739  3.18146  2.89801
2.37887  2.77519  2.98518  4.58477  3.61503
          0.02037  4.29947  5.02181  0.61958  0.77255  0.97767  0.47190
     32   3.18902  5.84508  1.13453  1.39901  5.12418  3.37300  3.96557
4.67801  3.04540  4.14785  5.01843  2.82612  4.02498  3.13816  3.68688
3.04291  3.48858  4.22518  6.28659  4.73819     64 d - - -
          2.68618  4.42225  2.77519  2.73123  3.46354  2.40513  3.72494
3.29354  2.67741  2.69355  4.24690  2.90347  2.73739  3.18146  2.89801
2.37887  2.77519  2.98518  4.58477  3.61503
          0.02037  4.29947  5.02181  0.61958  0.77255  0.97767  0.47190
     33   3.03654  4.44134  4.57465  4.00806  3.01781  4.16180  4.32627
2.47759  3.82121  1.28548  3.15662  4.16329  4.47645  4.01028  3.94826
3.48062  3.26776  2.06909  2.14379  3.43068     65 l - - -
          2.68618  4.42225  2.77519  2.73123  3.46354  2.40513  3.72494
3.29354  2.67741  2.69355  4.24690  2.90347  2.73739  3.18146  2.89801
2.37887  2.77519  2.98518  4.58477  3.61503
          0.02037  4.29947  5.02181  0.61958  0.77255  0.97767  0.47190
     34   1.74004  4.41722  4.37424  3.92661  3.72116  3.93526  4.68536
1.20660  3.82235  2.43129  3.60417  4.11264  4.44144  4.11522  4.04657
3.33350  3.21937  2.00501  5.45325  4.24245     66 i - - -
          2.68618  4.42225  2.77519  2.73123  3.46354  2.40513  3.72494
3.29354  2.67741  2.69355  4.24690  2.90347  2.73739  3.18146  2.89801
2.37887  2.77519  2.98518  4.58477  3.61503
          0.02037  4.29947  5.02181  0.61958  0.77255  0.97767  0.47190
```

```
     35   2.64672  4.75848  3.08393  2.54973  2.93096  3.47931  3.71794
3.40392  2.25555  3.03226  3.86337  2.39509  3.88492  2.89069  2.98062
2.26421  2.40558  3.09876  5.29643  3.98013     67 k - - -
          2.68618  4.42225  2.77519  2.73123  3.46354  2.40513  3.72494
3.29354  2.67741  2.69355  4.24690  2.90347  2.73739  3.18146  2.89801
2.37887  2.77519  2.98518  4.58477  3.61503
          0.02037  4.29947  5.02181  0.61958  0.77255  0.97767  0.47190
     36   3.33514  4.58204  5.22558  4.71214  3.64571  4.80403  5.34019
1.00916  4.61054  1.79549  3.41961  4.90279  5.02899  4.80253  4.75412
4.19468  3.58387  1.42822  5.69331  4.53629     68 i - - -
          2.68618  4.42225  2.77519  2.73123  3.46354  2.40513  3.72494
3.29354  2.67741  2.69355  4.24690  2.90347  2.73739  3.18146  2.89801
2.37887  2.77519  2.98518  4.58477  3.61503
          0.02037  4.29947  5.02181  0.61958  0.77255  0.97767  0.47190
     37   2.91005  5.22399  3.02472  2.56872  4.62398  2.56458  3.72428
4.04506  1.36651  3.52628  4.33755  3.07027  3.99009  2.23449  2.58469
2.88650  3.13215  3.66693  5.63332  4.33094     69 k - - -
          2.68618  4.42225  2.77519  2.73123  3.46354  2.40513  3.72494
3.29354  2.67741  2.69355  4.24690  2.90347  2.73739  3.18146  2.89801
2.37887  2.77519  2.98518  4.58477  3.61503
          0.02037  4.29947  5.02181  0.61958  0.77255  0.97767  0.47190
     38   1.85616  4.50573  3.48172  2.98114  3.64602  3.61429  3.95890
2.94014  2.86428  1.70695  3.58606  3.38687  4.04941  2.62554  3.19683
2.91549  2.98173  2.74575  5.12875  3.88610     70 l - - -
          2.68618  4.42225  2.77519  2.73123  3.46354  2.40513  3.72494
3.29354  2.67741  2.69355  4.24690  2.90347  2.73739  3.18146  2.89801
2.37887  2.77519  2.98518  4.58477  3.61503
          0.02037  4.29947  5.02181  0.61958  0.77255  0.97767  0.47190
     39   3.92221  5.23510  4.49909  4.32636  2.44627  4.35140  3.90623
3.88116  4.17467  3.21920  4.51293  4.29020  4.82317  4.37129  4.26143
4.00746  4.21536  3.77389  4.09983  0.47322     71 Y - - -
          2.68618  4.42225  2.77519  2.73123  3.46354  2.40513  3.72494
3.29354  2.67741  2.69355  4.24690  2.90347  2.73739  3.18146  2.89801
2.37887  2.77519  2.98518  4.58477  3.61503
          0.02037  4.29947  5.02181  0.61958  0.77255  0.97767  0.47190
     40   3.28318  4.55336  5.14481  4.67059  3.81032  4.71679  5.38739
1.47412  4.56947  2.28834  3.59199  4.85334  5.01514  4.83534  4.75238
4.13075  3.55359  0.81141  5.83181  4.61392     72 v - - -
          2.68618  4.42225  2.77519  2.73123  3.46354  2.40513  3.72494
3.29354  2.67741  2.69355  4.24690  2.90347  2.73739  3.18146  2.89801
2.37887  2.77519  2.98518  4.58477  3.61503
          0.02037  4.29947  5.02181  0.61958  0.77255  0.26824  1.44701
     41   2.84627  5.26376  3.13735  2.56275  4.60524  3.61195  2.77670
4.05717  1.80428  3.54701  4.31315  3.09074  3.99783  1.86473  2.52102
2.82053  2.55609  3.65499  5.67789  3.59960     73 k - - -
          2.68618  4.42225  2.77519  2.73123  3.46354  2.40513  3.72494
3.29354  2.67741  2.69355  4.24690  2.90347  2.73739  3.18146  2.89801
2.37887  2.77519  2.98518  4.58477  3.61503
          0.01397  4.67381  5.39616  0.61958  0.77255  0.48576  0.95510
     42   2.81843  5.23511  3.08293  2.53314  4.56701  3.58404  2.88824
4.02308  1.68193  3.52579  4.29107  3.06710  3.97893  2.48197  2.89062
2.18179  2.22664  3.62160  5.67125  4.29866     74 k - - -
          2.68618  4.42225  2.77519  2.73123  3.46354  2.40513  3.72494
3.29354  2.67741  2.69355  4.24690  2.90347  2.73739  3.18146  2.89801
2.37887  2.77519  2.98518  4.58477  3.61503
```

```
          0.01397  4.67381  5.39616  0.61958  0.77255  0.48576  0.95510
     43   2.13241  4.88212  3.24519  2.41666  4.53453  1.40558  4.08332
3.96825  2.93080  3.56968  4.37330  3.29092  4.04640  3.24945  3.38089
2.51524  2.22580  3.54061  5.79108  4.46953     75 g - - -
          2.68618  4.42225  2.77519  2.73123  3.46354  2.40513  3.72494
3.29354  2.67741  2.69355  4.24690  2.90347  2.73739  3.18146  2.89801
2.37887  2.77519  2.98518  4.58477  3.61503
          0.01397  4.67381  5.39616  0.61958  0.77255  0.48576  0.95510
     44   3.32468  4.64448  5.14523  4.58030  3.56677  4.61530  5.01721
1.81484  4.43946  0.95715  3.40559  4.71834  4.89329  4.58134  4.53108
3.95809  2.60007  1.87679  5.43597  4.30732     76 l - - -
          2.68618  4.42225  2.77519  2.73123  3.46354  2.40513  3.72494
3.29354  2.67741  2.69355  4.24690  2.90347  2.73739  3.18146  2.89801
2.37887  2.77519  2.98518  4.58477  3.61503
          0.01397  4.67381  5.39616  0.61958  0.77255  0.48576  0.95510
     45   2.76557  4.97911  3.13623  2.40310  4.30818  2.38428  3.84584
3.73222  2.64942  3.32635  4.13224  2.66428  3.99111  2.99323  3.11512
1.77636  2.70060  2.46444  5.55731  4.21640     77 s - - -
          2.68618  4.42225  2.77519  2.73123  3.46354  2.40513  3.72494
3.29354  2.67741  2.69355  4.24690  2.90347  2.73739  3.18146  2.89801
2.37887  2.77519  2.98518  4.58477  3.61503
          0.01397  4.67381  5.39616  0.61958  0.77255  0.48576  0.95510
     46   3.81055  5.11253  5.09235  4.68644  2.73318  4.76314  4.28097
3.13477  4.44812  0.95672  3.72590  4.62796  5.05346  4.55078  4.50287
4.14731  4.03783  3.19139  4.46741  1.26651     78 l - - -
          2.68618  4.42225  2.77519  2.73123  3.46354  2.40513  3.72494
3.29354  2.67741  2.69355  4.24690  2.90347  2.73739  3.18146  2.89801
2.37887  2.77519  2.98518  4.58477  3.61503
          0.01397  4.67381  5.39616  0.61958  0.77255  0.48576  0.95510
     47   4.12568  5.67158  4.81316  4.79836  5.62986  4.31894  5.73753
5.47571  4.94087  4.96294  6.08536  5.00509  0.13823  5.29369  5.03136
4.33059  4.63000  5.01057  6.51436  5.77914     79 P - - -
          2.68618  4.42225  2.77519  2.73123  3.46354  2.40513  3.72494
3.29354  2.67741  2.69355  4.24690  2.90347  2.73739  3.18146  2.89801
2.37887  2.77519  2.98518  4.58477  3.61503
          0.01397  4.67381  5.39616  0.61958  0.77255  0.48576  0.95510
     48   2.75683  4.85639  2.41531  2.02126  3.01975  3.61061  3.82391
2.80828  2.65573  3.10252  3.93599  3.17036  3.99998  2.99885  3.10612
2.51477  2.45317  2.65021  5.37994  4.06746     80 e - - -
          2.68627  4.42234  2.77529  2.73132  3.46363  2.40522  3.72504
3.29195  2.67750  2.69364  4.24699  2.90356  2.73749  3.18155  2.89810
2.37896  2.77529  2.98467  4.58486  3.61512
          0.39472  1.13445  5.39616  0.11573  2.21384  0.48576  0.95510
     49   2.86804  5.28230  2.95748  2.25155  4.62918  3.57019  3.82416
4.09145  2.57141  3.60250  4.37579  1.39903  4.00900  2.94262  2.64903
2.33166  2.78485  3.68532  5.75845  4.37063     82 n - - -
          2.68618  4.42225  2.77519  2.73123  3.46354  2.40513  3.72494
3.29354  2.67741  2.69355  4.24690  2.90347  2.73739  3.18146  2.89801
2.37887  2.77519  2.98518  4.58477  3.61503
          0.01397  4.67381  5.39616  0.61958  0.77255  0.48576  0.95510
     50   2.76340  4.48181  2.85650  2.56442  3.20942  3.74371  3.99788
2.30715  3.02968  1.84998  3.58027  3.48033  4.12277  2.87220  3.38324
2.98914  2.99520  2.20773  5.06589  3.83190     83 l - - -
```

```
        2.68618  4.42225  2.77519  2.73123  3.46354  2.40513  3.72494
3.29354  2.67741  2.69355  4.24690  2.90347  2.73739  3.18146  2.89801
2.37887  2.77519  2.98518  4.58477  3.61503
        0.01397  4.67381  5.39616  0.61958  0.77255  0.48576  0.95510
     51  2.50849  5.37565  2.20698  2.11036  4.71404  3.55022  3.78965
4.20127  1.58656  3.67489  4.42520  3.01410  3.98277  2.24659  3.03900
2.80950  3.09495  3.76408  5.80408  4.38575     84 k - - -
        2.68618  4.42225  2.77519  2.73123  3.46354  2.40513  3.72494
3.29354  2.67741  2.69355  4.24690  2.90347  2.73739  3.18146  2.89801
2.37887  2.77519  2.98518  4.58477  3.61503
        0.01397  4.67381  5.39616  0.61958  0.77255  0.48576  0.95510
     52  2.24150  5.20416  2.78019  1.77772  4.51991  3.10210  3.74168
3.47836  2.49458  3.49798  4.25612  3.02126  3.94048  2.45554  2.98317
2.74349  2.25046  3.57950  5.65734  4.26372     85 e - - -
        2.68618  4.42225  2.77519  2.73123  3.46354  2.40513  3.72494
3.29354  2.67741  2.69355  4.24690  2.90347  2.73739  3.18146  2.89801
2.37887  2.77519  2.98518  4.58477  3.61503
        0.01397  4.67381  5.39616  0.61958  0.77255  0.48576  0.95510
     53  3.58048  4.86043  5.47133  4.88544  2.89737  4.88119  5.19984
2.46608  4.73369  0.94871  2.40054  5.01484  5.05773  4.73232  4.73683
4.23271  3.79828  1.47766  5.42088  4.37031     86 l - - -
        2.68618  4.42225  2.77519  2.73123  3.46354  2.40513  3.72494
3.29354  2.67741  2.69355  4.24690  2.90347  2.73739  3.18146  2.89801
2.37887  2.77519  2.98518  4.58477  3.61503
        0.01397  4.67381  5.39616  0.61958  0.77255  0.48576  0.95510
     54  2.79758  5.27924  3.02993  2.08421  4.62476  3.55782  3.73843
4.09903  2.00036  3.57753  4.32480  2.37205  3.95415  2.83944  2.18240
2.13294  2.78565  3.67121  5.70551  4.31080     87 k - - -
        2.68618  4.42225  2.77519  2.73123  3.46354  2.40513  3.72494
3.29354  2.67741  2.69355  4.24690  2.90347  2.73739  3.18146  2.89801
2.37887  2.77519  2.98518  4.58477  3.61503
        0.01397  4.67381  5.39616  0.61958  0.77255  0.48576  0.95510
     55  2.50765  5.23194  3.02075  2.30305  4.56224  3.54450  3.73669
4.03270  2.47063  3.53025  4.28221  2.72436  3.94193  1.77953  2.51515
2.30660  2.55577  3.61584  5.67581  4.28047     88 q - - -
        2.68618  4.42225  2.77519  2.73123  3.46354  2.40513  3.72494
3.29354  2.67741  2.69355  4.24690  2.90347  2.73739  3.18146  2.89801
2.37887  2.77519  2.98518  4.58477  3.61503
        0.01397  4.67381  5.39616  0.61958  0.77255  0.48576  0.95510
     56  1.99624  4.76060  3.37441  2.87366  3.79722  2.46131  2.41598
3.38601  2.86265  3.04361  3.91372  3.33308  4.09717  3.20168  3.26896
2.93529  3.07189  3.11319  5.21577  2.04904     89 a - - -
        2.68618  4.42225  2.77519  2.73123  3.46354  2.40513  3.72494
3.29354  2.67741  2.69355  4.24690  2.90347  2.73739  3.18146  2.89801
2.37887  2.77519  2.98518  4.58477  3.61503
        0.01397  4.67381  5.39616  0.61958  0.77255  0.48576  0.95510
     57  3.58230  4.79506  5.61576  5.11598  3.91355  5.12825  5.72355
1.55660  5.03025  1.62474  3.66823  5.27176  5.32819  5.19936  5.14509
4.53762  3.83146  0.86460  6.00099  4.84957     90 v - - -
        2.68618  4.42225  2.77519  2.73123  3.46354  2.40513  3.72494
3.29354  2.67741  2.69355  4.24690  2.90347  2.73739  3.18146  2.89801
2.37887  2.77519  2.98518  4.58477  3.61503
        0.01397  4.67381  5.39616  0.61958  0.77255  0.48576  0.95510
```

```
      58   3.03861  5.39332  3.00054  2.66167  4.85845  1.77703  3.95486
 4.31548  1.42450  3.80225  4.60096  2.16779  4.13294  3.08588  2.99947
 3.01163  3.29841  3.89539  5.91890  4.56614     91 k - - -
          2.68625  4.42232  2.77527  2.73130  3.46361  2.40520  3.72502
 3.29361  2.67748  2.69362  4.24697  2.90354  2.73747  3.18154  2.89808
 2.37894  2.77527  2.98526  4.58484  3.61237
          0.54664  1.36141  1.80297  0.13931  2.03992  0.48576  0.95510
      59   3.02161  5.37065  3.15013  2.65759  4.76209  3.67679  3.78516
 4.17880  2.03090  3.63465  4.44005  1.34670  4.08395  2.51555  2.23618
 2.98637  3.22839  3.79781  5.72150  4.42508     93 n - - -
          2.68618  4.42225  2.77519  2.73123  3.46354  2.40513  3.72494
 3.29354  2.67741  2.69355  4.24690  2.90347  2.73739  3.18146  2.89801
 2.37887  2.77519  2.98518  4.58477  3.61503
          0.01666  4.49913  5.22148  0.61958  0.77255  0.76805  0.62347
      60   2.77686  4.92230  2.51903  2.61381  4.16038  3.58004  3.78049
 1.81160  2.05258  3.16764  4.00209  3.12139  3.97964  2.94208  2.61286
 2.82048  3.00575  3.23317  5.42219  4.10853     94 i - - -
          2.68618  4.42225  2.77519  2.73123  3.46354  2.40513  3.72494
 3.29354  2.67741  2.69355  4.24690  2.90347  2.73739  3.18146  2.89801
 2.37887  2.77519  2.98518  4.58477  3.61503
          0.01666  4.49913  5.22148  0.61958  0.77255  0.76805  0.62347
      61   2.74318  4.21770  4.23247  3.65413  3.28183  3.84005  4.16043
 1.76923  3.51733  2.36870  3.32706  3.84578  4.22097  3.74534  3.69132
 2.21988  2.98386  2.17359  2.39507  3.59608     95 i - - -
          2.68618  4.42225  2.77519  2.73123  3.46354  2.40513  3.72494
 3.29354  2.67741  2.69355  4.24690  2.90347  2.73739  3.18146  2.89801
 2.37887  2.77519  2.98518  4.58477  3.61503
          0.01666  4.49913  5.22148  0.61958  0.77255  0.76805  0.62347
      62   2.74733  5.18136  2.00842  2.43875  4.49832  3.50164  2.53338
 3.96512  2.42824  3.47105  4.23395  2.98040  3.90592  2.59551  2.20308
 2.72017  2.97822  3.11081  5.62112  4.23302     96 d - - -
          2.68618  4.42225  2.77519  2.73123  3.46354  2.40513  3.72494
 3.29354  2.67741  2.69355  4.24690  2.90347  2.73739  3.18146  2.89801
 2.37887  2.77519  2.98518  4.58477  3.61503
          0.01666  4.49913  5.22148  0.61958  0.77255  0.76805  0.62347
      63   3.52677  4.76650  5.45381  4.91630  3.54439  4.96417  5.41708
 1.53038  4.80088  1.01274  3.30977  5.08882  5.13679  4.88165  4.87113
 4.34940  3.76196  1.56833  5.64540  4.56765     97 l - - -
          2.68618  4.42225  2.77519  2.73123  3.46354  2.40513  3.72494
 3.29354  2.67741  2.69355  4.24690  2.90347  2.73739  3.18146  2.89801
 2.37887  2.77519  2.98518  4.58477  3.61503
          0.01666  4.49913  5.22148  0.61958  0.77255  0.76805  0.62347
      64   1.52190  4.78387  2.77336  2.67866  4.21506  3.46009  3.86345
 3.62751  2.69098  3.24702  4.06242  3.15550  3.16651  3.02855  3.14188
 2.31174  2.59801  2.89600  5.49549  4.17790     98 a - - -
          2.68618  4.42225  2.77519  2.73123  3.46354  2.40513  3.72494
 3.29354  2.67741  2.69355  4.24690  2.90347  2.73739  3.18146  2.89801
 2.37887  2.77519  2.98518  4.58477  3.61503
          0.01666  4.49913  5.22148  0.61958  0.77255  0.76805  0.62347
      65   2.91013  5.21707  2.92368  2.56238  4.51087  1.80942  2.45691
 4.03092  2.55768  3.55868  4.37069  3.08714  4.02646  1.85133  2.96490
 2.89640  3.16094  3.65257  5.68232  4.30858     99 g - - -
          2.68618  4.42225  2.77519  2.73123  3.46354  2.40513  3.72494
 3.29354  2.67741  2.69355  4.24690  2.90347  2.73739  3.18146  2.89801
 2.37887  2.77519  2.98518  4.58477  3.61503
```

```
        0.01666  4.49913  5.22148  0.61958  0.77255  0.76805  0.62347
     66   2.83716  4.56028  3.68514  3.10897  3.08716  3.79087  3.99028
2.96180  2.90484  1.38991  3.53609  3.50901  4.15955  2.37751  2.69121
3.05262  3.06334  2.79922  5.05418  3.79557    100 l - - -
        2.68618  4.42225  2.77519  2.73123  3.46354  2.40513  3.72494
3.29354  2.67741  2.69355  4.24690  2.90347  2.73739  3.18146  2.89801
2.37887  2.77519  2.98518  4.58477  3.61503
        0.01666  4.49913  5.22148  0.61958  0.77255  0.34269  1.23738
     67   2.89761  5.28935  3.12977  1.95714  4.62562  3.63761  3.79039
4.06746  1.71081  2.35824  4.34282  3.11896  4.03363  2.06308  2.82319
2.87271  3.11869  3.67776  5.70014  4.34997    101 k - - -
        2.68618  4.42225  2.77519  2.73123  3.46354  2.40513  3.72494
3.29354  2.67741  2.69355  4.24690  2.90347  2.73739  3.18146  2.89801
2.37887  2.77519  2.98518  4.58477  3.61503
        0.01397  4.67381  5.39616  0.61958  0.77255  0.48576  0.95510
     68   2.79146  4.99019  3.22193  2.72953  4.47971  3.54988  3.93284
3.90835  2.09544  3.48469  4.28485  3.21502  1.77028  3.07795  3.10732
2.34575  1.99005  3.51713  5.68684  4.35815    102 p - - -
        2.68618  4.42225  2.77519  2.73123  3.46354  2.40513  3.72494
3.29354  2.67741  2.69355  4.24690  2.90347  2.73739  3.18146  2.89801
2.37887  2.77519  2.98518  4.58477  3.61503
        0.01397  4.67381  5.39616  0.61958  0.77255  0.48576  0.95510
     69   2.37551  5.23983  2.99671  1.97944  4.57101  2.99076  3.74161
4.04444  2.27952  3.54163  4.29259  3.01545  3.94133  2.44865  2.97567
1.86186  2.78388  3.62477  5.68790  4.28751    103 s - - -
        2.68618  4.42225  2.77519  2.73123  3.46354  2.40513  3.72494
3.29354  2.67741  2.69355  4.24690  2.90347  2.73739  3.18146  2.89801
2.37887  2.77519  2.98518  4.58477  3.61503
        0.01397  4.67381  5.39616  0.61958  0.77255  0.48576  0.95510
     70   3.04794  5.54988  1.53847  2.48369  4.90592  1.64738  3.96111
4.40303  2.81711  3.89105  4.67852  2.64155  4.09463  3.09390  2.55163
2.98204  3.31164  3.96716  6.03711  4.59280    104 d - - -
        2.68618  4.42225  2.77519  2.73123  3.46354  2.40513  3.72494
3.29354  2.67741  2.69355  4.24690  2.90347  2.73739  3.18146  2.89801
2.37887  2.77519  2.98518  4.58477  3.61503
        0.01397  4.67381  5.39616  0.61958  0.77255  0.48576  0.95510
     71   3.60153  4.81690  5.62939  5.18476  4.09302  5.15823  5.94144
0.63062  5.10803  2.48972  3.83330  5.34577  5.40629  5.36011  5.27232
4.60948  3.87164  1.47346  6.24160  5.03031    105 I - - -
        2.68618  4.42225  2.77519  2.73123  3.46354  2.40513  3.72494
3.29354  2.67741  2.69355  4.24690  2.90347  2.73739  3.18146  2.89801
2.37887  2.77519  2.98518  4.58477  3.61503
        0.01397  4.67381  5.39616  0.61958  0.77255  0.48576  0.95510
     72   2.49188  5.25071  3.00270  1.74606  4.58414  3.53808  3.20186
4.06067  2.26750  3.54987  4.29632  3.01023  3.93620  2.22573  2.95530
2.37941  2.52817  3.63547  5.68922  4.28681    106 e - - -
        2.68618  4.42225  2.77519  2.73123  3.46354  2.40513  3.72494
3.29354  2.67741  2.69355  4.24690  2.90347  2.73739  3.18146  2.89801
2.37887  2.77519  2.98518  4.58477  3.61503
        0.01397  4.67381  5.39616  0.61958  0.77255  0.48576  0.95510
     73   3.09128  4.47013  3.51176  4.15155  3.69216  4.33190  4.72349
1.17559  4.04521  2.23605  3.60697  4.35014  4.67083  4.27505  4.22478
3.65046  2.83086  1.45187  5.34887  4.14838    107 i - - -
```

2.68618 4.42225 2.77519 2.73123 3.46354 2.40513 3.72494
3.29354 2.67741 2.69355 4.24690 2.90347 2.73739 3.18146 2.89801
2.37887 2.77519 2.98518 4.58477 3.61503
0.01397 4.67381 5.39616 0.61958 0.77255 0.48576 0.95510
74 3.26595 4.66339 4.83667 4.28073 3.45786 4.43613 4.79898
1.70676 4.12709 1.05302 3.32129 4.48013 2.31300 4.31474 4.26588
3.76857 3.50547 2.46335 5.29986 4.16519 108 l - - -
2.68618 4.42225 2.77519 2.73123 3.46354 2.40513 3.72494
3.29354 2.67741 2.69355 4.24690 2.90347 2.73739 3.18146 2.89801
2.37887 2.77519 2.98518 4.58477 3.61503
0.01397 4.67381 5.39616 0.61958 0.77255 0.48576 0.95510
75 2.82702 5.19245 2.48412 2.26573 2.22105 3.56342 3.80313
3.92515 2.59317 3.47147 4.25683 3.05456 3.98723 1.87429 3.08111
2.39354 3.06349 3.55091 5.65829 4.27455 109 q - - -
2.68618 4.42225 2.77519 2.73123 3.46354 2.40513 3.72494
3.29354 2.67741 2.69355 4.24690 2.90347 2.73739 3.18146 2.89801
2.37887 2.77519 2.98518 4.58477 3.61503
0.01397 4.67381 5.39616 0.61958 0.77255 0.48576 0.95510
76 3.19615 5.81231 1.48298 1.31553 5.11901 3.58407 4.03001
4.64179 1.98866 4.09905 4.90205 3.03209 4.15261 3.17064 3.50345
3.08921 3.46290 4.18957 6.23243 4.74016 110 e - - -
2.68618 4.42225 2.77519 2.73123 3.46354 2.40513 3.72494
3.29354 2.67741 2.69355 4.24690 2.90347 2.73739 3.18146 2.89801
2.37887 2.77519 2.98518 4.58477 3.61503
0.01397 4.67381 5.39616 0.61958 0.77255 0.48576 0.95510
77 2.84476 5.25598 2.66125 1.97840 2.59689 3.56892 3.79699
4.01643 1.48845 3.53873 4.31479 3.05048 3.99167 2.91665 3.03348
2.81860 3.07933 3.62472 5.70058 4.30897 111 k - - -
2.68618 4.42225 2.77519 2.73123 3.46354 2.40513 3.72494
3.29354 2.67741 2.69355 4.24690 2.90347 2.73739 3.18146 2.89801
2.37887 2.77519 2.98518 4.58477 3.61503
0.30223 4.67381 1.38033 0.61958 0.77255 0.48576 0.95510
78 1.76699 4.58735 3.32721 2.88359 4.34467 3.30108 4.04826
3.76300 2.91615 3.39721 4.21017 2.38273 3.90523 3.23914 3.33270
2.09252 1.76548 3.32498 5.63767 4.35306 112 t - - -
2.68618 4.42225 2.77519 2.73123 3.46354 2.40513 3.72494
3.29354 2.67741 2.69355 4.24690 2.90347 2.73739 3.18146 2.89801
2.37887 2.77519 2.98518 4.58477 3.61503
0.01859 4.39017 5.11252 0.61958 0.77255 0.89293 0.52671
79 2.90929 4.93288 3.34258 2.79398 4.11432 3.68580 3.81071
3.49390 2.09400 1.78176 3.97572 3.25451 4.07462 1.83789 2.68990
2.97683 3.12275 3.24505 5.37779 4.11573 113 l - - -
2.68618 4.42225 2.77519 2.73123 3.46354 2.40513 3.72494
3.29354 2.67741 2.69355 4.24690 2.90347 2.73739 3.18146 2.89801
2.37887 2.77519 2.98518 4.58477 3.61503
0.01859 4.39017 5.11252 0.61958 0.77255 0.89293 0.52671
80 3.16275 4.97731 3.90937 3.25107 3.97166 3.92002 3.99749
3.34519 2.43979 1.72265 3.92768 3.60988 4.30422 3.21890 1.19988
3.29856 3.37452 3.20918 5.35916 4.12315 114 r - - -
2.68618 4.42225 2.77519 2.73123 3.46354 2.40513 3.72494
3.29354 2.67741 2.69355 4.24690 2.90347 2.73739 3.18146 2.89801
2.37887 2.77519 2.98518 4.58477 3.61503
0.01859 4.39017 5.11252 0.61958 0.77255 0.89293 0.52671

```
     81   2.98021  4.35624  4.63800  4.07882  3.27347  4.17859  4.45436
1.43897  3.92981  2.23329  3.36352  4.21743  4.51369  4.12713  4.06028
3.50067  3.21912  1.67933  4.95299  2.09102    115 i - - -
          2.68618  4.42225  2.77519  2.73123  3.46354  2.40513  3.72494
3.29354  2.67741  2.69355  4.24690  2.90347  2.73739  3.18146  2.89801
2.37887  2.77519  2.98518  4.58477  3.61503
          0.01859  4.39017  5.11252  0.61958  0.77255  0.89293  0.52671
     82   2.78120  5.24973  2.25853  2.36459  4.59300  2.73528  3.73190
4.07292  2.12377  3.57211  4.33986  2.63213  3.89841  2.85024  3.03372
1.78179  3.02882  3.64987  5.72374  4.30726    116 s - - -
          2.68618  4.42225  2.77519  2.73123  3.46354  2.40513  3.72494
3.29354  2.67741  2.69355  4.24690  2.90347  2.73739  3.18146  2.89801
2.37887  2.77519  2.98518  4.58477  3.61503
          0.01859  4.39017  5.11252  0.61958  0.77255  0.89293  0.52671
     83   3.42347  4.73576  5.13498  4.62268  3.42268  4.70501  5.16587
2.15027  4.46212  0.92735  3.22899  4.81942  4.95753  4.61879  4.57526
4.09797  3.67503  1.43446  5.48534  4.36425    117 l - - -
          2.68618  4.42225  2.77519  2.73123  3.46354  2.40513  3.72494
3.29354  2.67741  2.69355  4.24690  2.90347  2.73739  3.18146  2.89801
2.37887  2.77519  2.98518  4.58477  3.61503
          0.01859  4.39017  5.11252  0.61958  0.77255  0.89293  0.52671
     84   4.07757  5.35252  4.64594  4.49691  2.51074  4.46712  3.98814
4.02938  4.34497  3.34189  4.65130  4.41915  4.93978  4.51579  4.40910
4.14987  4.37273  3.92690  4.16495  0.40834    118 Y - - -
          2.68618  4.42225  2.77519  2.73123  3.46354  2.40513  3.72494
3.29354  2.67741  2.69355  4.24690  2.90347  2.73739  3.18146  2.89801
2.37887  2.77519  2.98518  4.58477  3.61503
          0.01859  4.39017  5.11252  0.61958  0.77255  0.89293  0.52671
     85   2.48494  2.12511  3.66384  3.24629  4.24859  3.25458  4.27047
3.66067  3.22685  3.35105  4.19272  2.36349  3.93238  3.53869  3.56573
1.31169  2.91910  3.22483  5.61708  4.37962    119 s - - -
          2.68618  4.42225  2.77519  2.73123  3.46354  2.40513  3.72494
3.29354  2.67741  2.69355  4.24690  2.90347  2.73739  3.18146  2.89801
2.37887  2.77519  2.98518  4.58477  3.61503
          0.01859  4.39017  5.11252  0.61958  0.77255  0.89293  0.52671
     86   2.81132  5.21670  2.99642  2.10399  4.56961  3.53040  3.69263
4.01545  2.32063  3.50028  4.27778  3.01134  3.93540  2.06938  1.89696
2.30198  3.03388  3.61703  5.62499  4.27331    120 r - - -
          2.68618  4.42225  2.77519  2.73123  3.46354  2.40513  3.72494
3.29354  2.67741  2.69355  4.24690  2.90347  2.73739  3.18146  2.89801
2.37887  2.77519  2.98518  4.58477  3.61503
          0.01859  4.39017  5.11252  0.61958  0.77255  0.29582  1.36227
     87   2.85409  5.09398  1.70221  2.61074  4.35304  3.61752  3.83681
2.08534  2.42529  3.35139  4.16950  3.13492  4.03131  2.98202  2.39968
2.86945  3.08639  3.41958  5.57990  4.23928    121 d - - -
          2.68618  4.42225  2.77519  2.73123  3.46354  2.40513  3.72494
3.29354  2.67741  2.69355  4.24690  2.90347  2.73739  3.18146  2.89801
2.37887  2.77519  2.98518  4.58477  3.61503
          0.01397  4.67381  5.39616  0.61958  0.77255  0.48576  0.95510
     88   2.78838  4.67435  3.45027  2.87843  3.12660  3.69794  2.60382
2.84776  2.08839  1.99521  3.76443  3.33591  4.07902  3.15350  2.48020
2.92944  3.01696  2.96647  5.21502  3.94774    122 l - - -
          2.68618  4.42225  2.77519  2.73123  3.46354  2.40513  3.72494
3.29354  2.67741  2.69355  4.24690  2.90347  2.73739  3.18146  2.89801
2.37887  2.77519  2.98518  4.58477  3.61503
```

```
          0.01397  4.67381  5.39616  0.61958  0.77255  0.48576  0.95510
     89   2.81227  5.20353  1.66841  2.50029  4.49811  3.55551  2.92550
3.17087  2.56633  3.49127  4.26766  2.47831  3.97470  2.90866  3.05510
2.79353  2.34065  3.56972  5.66963  4.28216    123 d - - -
          2.68618  4.42225  2.77519  2.73123  3.46354  2.40513  3.72494
3.29354  2.67741  2.69355  4.24690  2.90347  2.73739  3.18146  2.89801
2.37887  2.77519  2.98518  4.58477  3.61503
          0.01397  4.67381  5.39616  0.61958  0.77255  0.48576  0.95510
     90   2.76485  4.89910  3.19357  2.00572  4.12253  3.60461  2.19167
3.52675  2.63339  2.62939  3.97897  3.15545  3.99732  2.98404  3.08456
2.42327  2.76250  2.56899  5.41620  4.09629    124 e - - -
          2.68618  4.42225  2.77519  2.73123  3.46354  2.40513  3.72494
3.29354  2.67741  2.69355  4.24690  2.90347  2.73739  3.18146  2.89801
2.37887  2.77519  2.98518  4.58477  3.61503
          0.01397  4.67381  5.39616  0.61958  0.77255  0.48576  0.95510
     91   1.22382  2.95209  3.88565  3.35166  3.78235  3.62220  4.21215
2.52544  3.29229  2.86053  3.75399  2.82467  4.13943  3.57800  3.60485
2.57362  3.02564  2.85620  5.23588  4.01936    125 a - - -
          2.68618  4.42225  2.77519  2.73123  3.46354  2.40513  3.72494
3.29354  2.67741  2.69355  4.24690  2.90347  2.73739  3.18146  2.89801
2.37887  2.77519  2.98518  4.58477  3.61503
          0.01397  4.67381  5.39616  0.61958  0.77255  0.48576  0.95510
     92   2.21543  4.44697  3.70597  2.59662  3.58511  3.75856  4.02524
2.67763  3.08687  2.07157  3.55647  3.52605  4.13937  3.38212  3.42444
2.26094  2.69240  2.04075  5.04354  3.81572    126 v - - -
          2.68618  4.42225  2.77519  2.73123  3.46354  2.40513  3.72494
3.29354  2.67741  2.69355  4.24690  2.90347  2.73739  3.18146  2.89801
2.37887  2.77519  2.98518  4.58477  3.61503
          0.01397  4.67381  5.39616  0.61958  0.77255  0.48576  0.95510
     93   2.88351  5.02149  3.32798  2.76089  4.24714  3.68104  3.85868
3.71685  2.53168  3.30550  4.13657  2.77834  4.08127  3.02276  1.66049
1.98852  3.11486  3.39908  2.61279  4.16579    127 r - - -
          2.68618  4.42225  2.77519  2.73123  3.46354  2.40513  3.72494
3.29354  2.67741  2.69355  4.24690  2.90347  2.73739  3.18146  2.89801
2.37887  2.77519  2.98518  4.58477  3.61503
          0.01397  4.67381  5.39616  0.61958  0.77255  0.48576  0.95510
     94   2.19344  4.81276  3.26702  2.11266  4.04429  3.63035  3.95036
3.46499  2.81603  3.12153  3.97857  3.28175  4.07565  3.16509  3.23003
2.53140  3.07390  3.17746  1.69465  4.09487    128 w - - -
          2.68618  4.42225  2.77519  2.73123  3.46354  2.40513  3.72494
3.29354  2.67741  2.69355  4.24690  2.90347  2.73739  3.18146  2.89801
2.37887  2.77519  2.98518  4.58477  3.61503
          0.39472  4.67381  1.14950  0.61958  0.77255  0.48576  0.95510
     95   2.87472  5.29168  2.09344  2.33592  4.71942  3.36160  3.85371
4.22118  2.76844  3.73987  4.54723  1.58685  3.93210  3.00399  3.30498
1.88156  3.16849  3.78304  5.90100  4.45789    129 n - - -
          2.68618  4.42225  2.77519  2.73123  3.46354  2.40513  3.72494
3.29354  2.67741  2.69355  4.24690  2.90347  2.73739  3.18146  2.89801
2.37887  2.77519  2.98518  4.58477  3.61503
          0.02037  4.29947  5.02181  0.61958  0.77255  0.97767  0.47190
     96   2.34394  5.17666  2.08491  2.35961  4.54405  2.05433  3.74551
4.01092  2.10816  3.53526  4.32103  2.90921  3.88693  2.87632  3.06453
2.73864  3.03092  3.60057  5.70540  4.29984    130 g - - -
```

```
         2.68618  4.42225  2.77519  2.73123  3.46354  2.40513  3.72494
3.29354  2.67741  2.69355  4.24690  2.90347  2.73739  3.18146  2.89801
2.37887  2.77519  2.98518  4.58477  3.61503
         0.02037  4.29947  5.02181  0.61958  0.77255  0.97767  0.47190
     97  2.75574  4.31944  3.92158  3.40682  2.46483  3.77725  4.09089
2.54842  3.33127  2.44096  3.44288  2.63843  4.18862  3.60172  3.59207
3.08671  3.01304  1.43294  4.84348  3.51326    131 v - - -
         2.68618  4.42225  2.77519  2.73123  3.46354  2.40513  3.72494
3.29354  2.67741  2.69355  4.24690  2.90347  2.73739  3.18146  2.89801
2.37887  2.77519  2.98518  4.58477  3.61503
         0.02037  4.29947  5.02181  0.61958  0.77255  0.97767  0.47190
     98  2.92962  4.94026  3.02917  1.43908  3.98658  3.61991  3.94734
3.25967  2.76768  1.78121  3.92348  3.21708  4.08823  3.15508  3.15761
3.00418  3.18110  3.07555  5.43821  4.12113    132 e - - -
         2.68618  4.42225  2.77519  2.73123  3.46354  2.40513  3.72494
3.29354  2.67741  2.69355  4.24690  2.90347  2.73739  3.18146  2.89801
2.37887  2.77519  2.98518  4.58477  3.61503
         0.02037  4.29947  5.02181  0.61958  0.77255  0.97767  0.47190
     99  2.65111  4.74445  3.08982  2.35432  2.92776  3.49607  3.71007
3.35658  2.54696  2.98939  3.82567  2.70297  3.88954  2.55094  2.99347
2.20724  2.88319  3.06203  5.24651  2.93645    133 s - - -
         2.68618  4.42225  2.77519  2.73123  3.46354  2.40513  3.72494
3.29354  2.67741  2.69355  4.24690  2.90347  2.73739  3.18146  2.89801
2.37887  2.77519  2.98518  4.58477  3.61503
         0.02037  4.29947  5.02181  0.61958  0.77255  0.97767  0.47190
    100  2.38967  5.14913  2.46299  2.35884  4.47882  3.41797  3.68160
3.94494  1.99567  3.45978  4.23163  2.60198  2.32301  2.80040  2.94553
2.69024  2.96334  3.53908  5.62189  4.22361    134 k - - -
         2.68618  4.42225  2.77519  2.73123  3.46354  2.40513  3.72494
3.29354  2.67741  2.69355  4.24690  2.90347  2.73739  3.18146  2.89801
2.37887  2.77519  2.98518  4.58477  3.61503
         0.02037  4.29947  5.02181  0.61958  0.77255  0.97767  0.47190
    101  3.32710  4.58750  5.19461  4.66124  3.51853  4.74698  5.21710
1.48459  4.55008  1.33503  3.30081  4.84393  4.96455  4.69778  4.66544
4.12252  3.56833  1.33038  5.55001  4.42658    135 v - - -
         2.68618  4.42225  2.77519  2.73123  3.46354  2.40513  3.72494
3.29354  2.67741  2.69355  4.24690  2.90347  2.73739  3.18146  2.89801
2.37887  2.77519  2.98518  4.58477  3.61503
         0.02037  4.29947  5.02181  0.61958  0.77255  0.97767  0.47190
    102  2.83258  4.30267  4.21439  3.63575  3.29375  3.95718  4.26030
1.76868  3.52202  1.86718  2.70244  3.89595  4.29689  2.63248  3.73264
3.24686  3.06377  1.90453  4.94419  3.77126    136 i - - -
         2.68618  4.42225  2.77519  2.73123  3.46354  2.40513  3.72494
3.29354  2.67741  2.69355  4.24690  2.90347  2.73739  3.18146  2.89801
2.37887  2.77519  2.98518  4.58477  3.61503
         0.02037  4.29947  5.02181  0.61958  0.77255  0.97767  0.47190
    103  2.88339  4.62366  3.65184  3.10132  3.53854  3.82766  4.04864
2.20060  2.91786  1.90588  3.45174  3.52516  4.19303  1.71737  3.24033
3.10080  3.11415  2.63980  5.15212  3.90973    137 q - - -
         2.68618  4.42225  2.77519  2.73123  3.46354  2.40513  3.72494
3.29354  2.67741  2.69355  4.24690  2.90347  2.73739  3.18146  2.89801
2.37887  2.77519  2.98518  4.58477  3.61503
         0.02037  4.29947  5.02181  0.61958  0.77255  0.97767  0.47190
```

```
    104   3.09714  5.33607  3.21790  2.73190  4.74842  3.68786  3.75277
4.15161  1.46472  3.59133  4.44221  3.19593  4.10085  1.46507  2.39402
3.06872  3.29118  3.80206  5.64803  4.40917    138 k - - -
          2.68622  4.42372  2.77523  2.73075  3.46501  2.40470  3.72641
3.28716  2.67800  2.69343  4.24737  2.90301  2.73886  3.18197  2.89923
2.37809  2.77485  2.98602  4.58624  3.61650
          0.47685  0.98706  5.02181  2.82376  0.06122  0.97767  0.47190
    105   2.71287  4.22128  4.05706  3.48721  3.37593  3.80704  4.15275
1.97985  3.38971  2.10565  3.34626  3.75134  4.18777  3.64341  3.62467
2.44210  2.35908  1.79833  4.91829  3.71654    159 v - - -
          2.68618  4.42225  2.77519  2.73123  3.46354  2.40513  3.72494
3.29354  2.67741  2.69355  4.24690  2.90347  2.73739  3.18146  2.89801
2.37887  2.77519  2.98518  4.58477  3.61503
          0.02037  4.29947  5.02181  0.61958  0.77255  0.97767  0.47190
    106   1.74004  4.41722  4.37424  3.92661  3.72116  3.93526  4.68536
1.20660  3.82235  2.43129  3.60417  4.11264  4.44144  4.11522  4.04657
3.33350  3.21937  2.00501  5.45325  4.24245    160 i - - -
          2.68618  4.42225  2.77519  2.73123  3.46354  2.40513  3.72494
3.29354  2.67741  2.69355  4.24690  2.90347  2.73739  3.18146  2.89801
2.37887  2.77519  2.98518  4.58477  3.61503
          0.02037  4.29947  5.02181  0.61958  0.77255  0.97767  0.47190
    107   2.95678  5.45103  2.15689  2.30871  4.76983  3.40466  3.82345
4.26770  2.21495  3.76394  4.57660  1.32682  3.95555  2.96870  3.19360
2.87918  3.22195  3.84922  5.90951  4.46434    161 n - - -
          2.68618  4.42225  2.77519  2.73123  3.46354  2.40513  3.72494
3.29354  2.67741  2.69355  4.24690  2.90347  2.73739  3.18146  2.89801
2.37887  2.77519  2.98518  4.58477  3.61503
          0.02037  4.29947  5.02181  0.61958  0.77255  0.26824  1.44701
    108   3.02663  5.58413  1.90852  1.82796  4.90433  3.56147  3.92073
4.40832  2.77402  3.88177  4.65672  3.01887  4.07047  2.32883  3.30974
1.56493  3.27984  3.96645  6.02046  4.56394    162 s - - -
          2.68618  4.42225  2.77519  2.73123  3.46354  2.40513  3.72494
3.29354  2.67741  2.69355  4.24690  2.90347  2.73739  3.18146  2.89801
2.37887  2.77519  2.98518  4.58477  3.61503
          0.01397  4.67381  5.39616  0.61958  0.77255  0.48576  0.95510
    109   3.57640  4.83594  5.52409  4.95454  3.49762  4.93898  5.31721
1.63026  4.81421  1.30704  1.67171  5.08248  5.11880  4.83962  4.83117
4.30243  3.80149  1.77740  5.54385  4.48909    163 l - - -
          2.68618  4.42225  2.77519  2.73123  3.46354  2.40513  3.72494
3.29354  2.67741  2.69355  4.24690  2.90347  2.73739  3.18146  2.89801
2.37887  2.77519  2.98518  4.58477  3.61503
          0.01397  4.67381  5.39616  0.61958  0.77255  0.48576  0.95510
    110   2.02003  4.58693  3.95065  3.65596  4.90178  3.34224  4.72806
4.34844  3.71819  4.01043  4.80480  3.76427  1.23223  3.98756  4.03304
1.31302  3.13140  3.70696  6.21595  5.01699    164 p - - -
          2.68618  4.42225  2.77519  2.73123  3.46354  2.40513  3.72494
3.29354  2.67741  2.69355  4.24690  2.90347  2.73739  3.18146  2.89801
2.37887  2.77519  2.98518  4.58477  3.61503
          0.01397  4.67381  5.39616  0.61958  0.77255  0.48576  0.95510
    111   2.33512  4.64832  3.47743  2.94934  3.88550  3.66372  3.99481
1.82483  2.90670  2.93326  3.81473  3.38693  4.09515  2.17589  3.29306
2.03694  3.03485  2.95591  5.28383  4.02389    165 i - - -
          2.68618  4.42225  2.77519  2.73123  3.46354  2.40513  3.72494
3.29354  2.67741  2.69355  4.24690  2.90347  2.73739  3.18146  2.89801
2.37887  2.77519  2.98518  4.58477  3.61503
```

0.01397 4.67381 5.39616 0.61958 0.77255 0.48576 0.95510
112 2.93823 4.32550 4.71944 4.11987 3.29318 4.10608 4.42352
1.96319 3.94600 1.33697 2.71207 4.20423 4.44487 4.09345 4.01469
3.41510 2.69589 2.44880 4.92792 2.31394 166 l - - -
2.68618 4.42225 2.77519 2.73123 3.46354 2.40513 3.72494
3.29354 2.67741 2.69355 4.24690 2.90347 2.73739 3.18146 2.89801
2.37887 2.77519 2.98518 4.58477 3.61503
0.01397 4.67381 5.39616 0.61958 0.77255 0.48576 0.95510
113 2.86506 5.35532 2.51245 1.91616 4.67810 2.35716 3.80551
4.15913 2.58514 3.03393 4.41092 3.01807 3.98961 1.73955 3.08643
2.82023 3.10384 3.73577 5.79672 4.38040 167 q - - -
2.68618 4.42225 2.77519 2.73123 3.46354 2.40513 3.72494
3.29354 2.67741 2.69355 4.24690 2.90347 2.73739 3.18146 2.89801
2.37887 2.77519 2.98518 4.58477 3.61503
0.01397 4.67381 5.39616 0.61958 0.77255 0.48576 0.95510
114 2.76834 4.47124 3.69308 3.12505 2.39644 3.74836 4.02016
2.96160 3.05019 2.68338 3.58458 3.51469 3.24538 3.36545 2.02103
3.00357 2.33881 2.12478 5.06448 3.83269 168 r - - -
2.68618 4.42225 2.77519 2.73123 3.46354 2.40513 3.72494
3.29354 2.67741 2.69355 4.24690 2.90347 2.73739 3.18146 2.89801
2.37887 2.77519 2.98518 4.58477 3.61503
0.01397 4.67381 5.39616 0.61958 0.77255 0.48576 0.95510
115 2.91234 4.61632 3.80080 3.23245 3.82908 3.89521 4.16857
2.44381 1.70471 2.77785 3.74383 3.63627 4.28051 3.46337 3.39418
3.16367 2.67538 1.49748 5.30845 4.07202 169 v - - -
2.68618 4.42225 2.77519 2.73123 3.46354 2.40513 3.72494
3.29354 2.67741 2.69355 4.24690 2.90347 2.73739 3.18146 2.89801
2.37887 2.77519 2.98518 4.58477 3.61503
0.39472 4.67381 1.14950 0.61958 0.77255 0.48576 0.95510
116 2.72537 3.01048 3.35057 2.75775 3.90109 3.58629 3.77252
3.24810 1.87347 2.91935 3.78683 3.21071 3.97489 2.97905 2.19439
2.83632 2.95389 2.40799 5.21433 3.95631 170 k - - -
2.68618 4.42225 2.77519 2.73123 3.46354 2.40513 3.72494
3.29354 2.67741 2.69355 4.24690 2.90347 2.73739 3.18146 2.89801
2.37887 2.77519 2.98518 4.58477 3.61503
0.02037 4.29947 5.02181 0.61958 0.77255 0.97767 0.47190
117 1.71975 4.58859 3.28836 2.82997 4.13614 3.37146 3.94477
3.46997 2.35761 3.16522 4.01845 3.23604 3.91879 3.15028 3.12765
2.71443 1.69721 3.12461 5.45318 4.18346 171 t - - -
2.68618 4.42225 2.77519 2.73123 3.46354 2.40513 3.72494
3.29354 2.67741 2.69355 4.24690 2.90347 2.73739 3.18146 2.89801
2.37887 2.77519 2.98518 4.58477 3.61503
0.02037 4.29947 5.02181 0.61958 0.77255 0.97767 0.47190
118 2.68885 4.80553 3.12894 2.60508 4.11978 2.19778 3.74991
3.50503 1.89544 2.64818 3.96423 3.09255 3.91481 2.91576 2.88102
2.74673 2.49226 3.18723 5.37865 4.07541 172 k - - -
2.68618 4.42225 2.77519 2.73123 3.46354 2.40513 3.72494
3.29354 2.67741 2.69355 4.24690 2.90347 2.73739 3.18146 2.89801
2.37887 2.77519 2.98518 4.58477 3.61503
0.02037 4.29947 5.02181 0.61958 0.77255 0.26824 1.44701
119 2.99357 4.96967 2.51012 2.83283 4.10086 3.73470 4.02998
3.45278 2.33111 1.12358 4.02519 3.32800 4.18564 3.25012 3.25079
3.08255 3.23550 3.22197 5.49129 4.19735 173 l - - -

```
        2.68618  4.42225  2.77519  2.73123  3.46354  2.40513  3.72494
3.29354  2.67741  2.69355  4.24690  2.90347  2.73739  3.18146  2.89801
2.37887  2.77519  2.98518  4.58477  3.61503
        0.01397  4.67381  5.39616  0.61958  0.77255  0.48576  0.95510
    120  2.90541  5.30996  3.12714  2.41669  4.66807  1.80841  3.79488
4.11358  2.02669  3.59865  4.37332  3.12073  2.97527  2.90641  2.01587
2.87738  3.12828  3.71334  5.72372  4.37250    174 g - - -
        2.68618  4.42225  2.77519  2.73123  3.46354  2.40513  3.72494
3.29354  2.67741  2.69355  4.24690  2.90347  2.73739  3.18146  2.89801
2.37887  2.77519  2.98518  4.58477  3.61503
        0.01397  4.67381  5.39616  0.61958  0.77255  0.48576  0.95510
    121  2.40548  5.37980  2.16750  2.24545  4.71283  3.54226  3.79863
4.20262  2.57832  3.68108  4.43252  2.38359  3.98262  1.89445  3.08571
2.24402  3.09984  3.76571  5.81617  4.39109    175 q - - -
        2.68618  4.42225  2.77519  2.73123  3.46354  2.40513  3.72494
3.29354  2.67741  2.69355  4.24690  2.90347  2.73739  3.18146  2.89801
2.37887  2.77519  2.98518  4.58477  3.61503
        0.41791  4.67381  1.10189  0.61958  0.77255  0.48576  0.95510
    122  3.05787  4.44541  4.62619  4.07405  3.39931  4.26985  4.66663
1.51424  3.93456  1.36025  3.25536  4.29420  4.58746  4.15829  4.10695
3.60302  2.45732  2.02746  5.22369  4.06733    176 l - - -
        2.68618  4.42225  2.77519  2.73123  3.46354  2.40513  3.72494
3.29354  2.67741  2.69355  4.24690  2.90347  2.73739  3.18146  2.89801
2.37887  2.77519  2.98518  4.58477  3.61503
        0.02085  4.27675  4.99910  0.61958  0.77255  0.99671  0.46060
    123  2.32827  4.63835  3.23595  2.70167  3.86971  3.52350  3.78545
3.22201  2.17248  2.09483  3.76661  3.16751  2.56056  2.99626  2.97562
2.78148  2.91611  2.95399  5.21757  3.94276    177 l - - -
        2.68618  4.42225  2.77519  2.73123  3.46354  2.40513  3.72494
3.29354  2.67741  2.69355  4.24690  2.90347  2.73739  3.18146  2.89801
2.37887  2.77519  2.98518  4.58477  3.61503
        0.02085  4.27675  4.99910  0.61958  0.77255  0.76000  0.63048
    124  1.96277  4.78526  3.15073  2.64023  4.21711  3.43821  3.81865
3.62311  2.16868  3.23740  4.05451  3.11813  2.52989  2.97990  3.03253
2.71288  2.09241  3.26597  5.47654  4.16193    178 a - - -
        2.68618  4.42225  2.77519  2.73123  3.46354  2.40513  3.72494
3.29354  2.67741  2.69355  4.24690  2.90347  2.73739  3.18146  2.89801
2.37887  2.77519  2.98518  4.58477  3.61503
        0.01859  4.39017  5.11252  0.61958  0.77255  0.89293  0.52671
    125  2.75563  5.16120  2.39677  2.42387  4.46970  3.48167  3.69285
3.92545  2.07648  2.74851  4.21955  2.96383  3.89901  1.78122  2.87056
2.72980  2.98716  3.53481  5.60144  4.22239    179 q - - -
        2.68618  4.42225  2.77519  2.73123  3.46354  2.40513  3.72494
3.29354  2.67741  2.69355  4.24690  2.90347  2.73739  3.18146  2.89801
2.37887  2.77519  2.98518  4.58477  3.61503
        0.01859  4.39017  5.11252  0.61958  0.77255  0.89293  0.52671
    126  2.86994  5.32276  1.95042  2.05459  4.66384  2.07570  3.81123
4.14198  2.67642  3.65847  4.45016  2.91847  3.94295  2.94671  3.20322
2.81870  2.09271  3.72715  5.82802  4.39829    180 d - - -
        2.68618  4.42225  2.77519  2.73123  3.46354  2.40513  3.72494
3.29354  2.67741  2.69355  4.24690  2.90347  2.73739  3.18146  2.89801
2.37887  2.77519  2.98518  4.58477  3.61503
        0.01859  4.39017  5.11252  0.61958  0.77255  0.89293  0.52671
```

127 4.07757 5.35252 4.64594 4.49691 2.51074 4.46712 3.98814
4.02938 4.34497 3.34189 4.65130 4.41915 4.93978 4.51579 4.40910
4.14987 4.37273 3.92690 4.16495 0.40834 181 Y - - -
2.68618 4.42225 2.77519 2.73123 3.46354 2.40513 3.72494
3.29354 2.67741 2.69355 4.24690 2.90347 2.73739 3.18146 2.89801
2.37887 2.77519 2.98518 4.58477 3.61503
0.01859 4.39017 5.11252 0.61958 0.77255 0.89293 0.52671
128 4.17665 5.27443 5.12944 4.93358 0.66102 4.81750 3.69036
3.76311 4.74582 3.00013 4.32966 4.45057 5.10283 4.56570 4.66301
4.21665 4.39061 3.72012 3.78899 1.55779 182 F - - -
2.68618 4.42225 2.77519 2.73123 3.46354 2.40513 3.72494
3.29354 2.67741 2.69355 4.24690 2.90347 2.73739 3.18146 2.89801
2.37887 2.77519 2.98518 4.58477 3.61503
0.01859 4.39017 5.11252 0.61958 0.77255 0.89293 0.52671
129 2.71845 5.05579 3.00368 2.12992 4.35019 3.49612 3.67940
3.78674 2.08474 2.72587 4.11616 2.99257 3.89192 2.26348 2.83560
2.71261 2.20836 3.41909 5.51206 4.15555 183 k - - -
2.68618 4.42225 2.77519 2.73123 3.46354 2.40513 3.72494
3.29354 2.67741 2.69355 4.24690 2.90347 2.73739 3.18146 2.89801
2.37887 2.77519 2.98518 4.58477 3.61503
0.01859 4.39017 5.11252 0.61958 0.77255 0.89293 0.52671
130 2.71520 4.61177 3.43038 2.91045 3.94015 3.56366 3.94662
3.13139 2.22485 2.93852 3.84302 3.33008 4.02812 3.17788 3.09413
2.86583 1.50109 2.28996 5.31671 4.06027 184 t - - -
2.68618 4.42225 2.77519 2.73123 3.46354 2.40513 3.72494
3.29354 2.67741 2.69355 4.24690 2.90347 2.73739 3.18146 2.89801
2.37887 2.77519 2.98518 4.58477 3.61503
0.01859 4.39017 5.11252 0.61958 0.77255 0.29582 1.36227
131 3.92544 5.13903 5.83035 5.24991 3.38453 5.31486 5.61070
1.68668 5.11206 0.75640 1.99939 5.45853 5.34088 4.99108 5.06932
4.70440 4.12862 2.69563 5.61698 4.66827 185 l - - -
2.68618 4.42225 2.77519 2.73123 3.46354 2.40513 3.72494
3.29354 2.67741 2.69355 4.24690 2.90347 2.73739 3.18146 2.89801
2.37887 2.77519 2.98518 4.58477 3.61503
0.01397 4.67381 5.39616 0.61958 0.77255 0.48576 0.95510
132 2.78381 5.09327 3.07387 2.04867 4.37641 3.57054 3.78335
3.23797 2.55071 3.37508 4.16614 3.07982 3.97389 2.17600 3.01880
2.53597 1.77232 3.45076 5.57942 4.21898 186 t - - -
2.68618 4.42225 2.77519 2.73123 3.46354 2.40513 3.72494
3.29354 2.67741 2.69355 4.24690 2.90347 2.73739 3.18146 2.89801
2.37887 2.77519 2.98518 4.58477 3.61503
0.01397 4.67381 5.39616 0.61958 0.77255 0.48576 0.95510
133 3.40816 6.10887 1.23011 1.12873 5.39606 2.69822 4.18445
4.96049 3.26883 4.40766 5.26666 3.03986 4.24601 3.35255 3.92507
3.25704 3.70759 4.48832 6.55064 4.98050 187 e - - -
2.68618 4.42225 2.77519 2.73123 3.46354 2.40513 3.72494
3.29354 2.67741 2.69355 4.24690 2.90347 2.73739 3.18146 2.89801
2.37887 2.77519 2.98518 4.58477 3.61503
0.01397 4.67381 5.39616 0.61958 0.77255 0.48576 0.95510
134 2.78952 5.20522 2.54851 2.19551 4.51426 3.55592 3.75580
2.02168 2.01815 3.49212 4.25802 2.66566 3.95769 2.86817 2.68385
2.76783 3.02098 3.57651 5.65627 4.27084 188 k - - -
2.68618 4.42225 2.77519 2.73123 3.46354 2.40513 3.72494
3.29354 2.67741 2.69355 4.24690 2.90347 2.73739 3.18146 2.89801
2.37887 2.77519 2.98518 4.58477 3.61503

0.01397 4.67381 5.39616 0.61958 0.77255 0.48576 0.95510
135 2.83450 5.11786 1.62418 2.56802 4.36475 3.59120 3.83218
3.76190 2.62597 2.70597 4.18125 3.10212 4.01201 2.06937 3.09865
2.84092 3.07176 2.73805 5.60676 4.24351 189 d - - -
2.68618 4.42225 2.77519 2.73123 3.46354 2.40513 3.72494
3.29354 2.67741 2.69355 4.24690 2.90347 2.73739 3.18146 2.89801
2.37887 2.77519 2.98518 4.58477 3.61503
0.01397 4.67381 5.39616 0.61958 0.77255 0.48576 0.95510
136 2.81502 5.17723 2.51283 1.75502 4.46151 3.56405 3.79331
3.90602 2.40468 2.06716 4.23949 3.05593 3.98101 2.91841 3.05581
2.80249 2.38054 3.53310 5.64694 4.26897 190 e - - -
2.68618 4.42225 2.77519 2.73123 3.46354 2.40513 3.72494
3.29354 2.67741 2.69355 4.24690 2.90347 2.73739 3.18146 2.89801
2.37887 2.77519 2.98518 4.58477 3.61503
0.01397 4.67381 5.39616 0.61958 0.77255 0.48576 0.95510
137 1.25476 5.34109 2.81033 1.94008 4.77700 3.56272 4.01310
4.23526 2.87371 3.78394 4.59160 2.03287 4.10451 3.16151 3.37067
2.97555 3.28471 3.82296 5.96667 4.56466 191 a - - -
2.68618 4.42225 2.77519 2.73123 3.46354 2.40513 3.72494
3.29354 2.67741 2.69355 4.24690 2.90347 2.73739 3.18146 2.89801
2.37887 2.77519 2.98518 4.58477 3.61503
0.01397 4.67381 5.39616 0.61958 0.77255 0.48576 0.95510
138 2.76882 4.54974 3.52282 2.47489 3.70037 3.04588 3.96886
2.21404 2.95340 1.69109 3.65425 2.92009 4.10138 3.26721 3.32779
2.65423 3.00269 2.82662 5.12784 3.88197 192 l - - -
2.68618 4.42225 2.77519 2.73123 3.46354 2.40513 3.72494
3.29354 2.67741 2.69355 4.24690 2.90347 2.73739 3.18146 2.89801
2.37887 2.77519 2.98518 4.58477 3.61503
0.01397 4.67381 5.39616 0.61958 0.77255 0.48576 0.95510
139 2.47222 4.44194 3.83823 2.44108 3.60519 3.83031 4.13624
1.61554 3.23308 2.64282 3.57155 3.64798 4.21897 3.52134 3.54819
2.53589 3.05315 1.96315 5.09323 3.87166 193 i - - -
2.68618 4.42225 2.77519 2.73123 3.46354 2.40513 3.72494
3.29354 2.67741 2.69355 4.24690 2.90347 2.73739 3.18146 2.89801
2.37887 2.77519 2.98518 4.58477 3.61503
0.01397 4.67381 5.39616 0.61958 0.77255 0.48576 0.95510
140 2.93615 5.33863 1.58422 2.53272 4.77793 3.53937 3.95000
4.25795 2.79797 3.77538 4.55765 2.66993 4.05717 3.08285 3.31300
1.91992 1.95477 3.82215 5.93934 4.52294 194 d - - -
2.68618 4.42225 2.77519 2.73123 3.46354 2.40513 3.72494
3.29354 2.67741 2.69355 4.24690 2.90347 2.73739 3.18146 2.89801
2.37887 2.77519 2.98518 4.58477 3.61503
0.01397 4.67381 5.39616 0.61958 0.77255 0.48576 0.95510
141 2.78360 4.93987 3.16881 2.63858 4.14008 3.60585 3.82145
3.58520 2.63265 2.91026 4.02317 2.05775 2.71106 2.75638 3.08336
2.57054 3.01737 3.27300 5.43241 2.21337 195 n - - -
2.68618 4.42225 2.77519 2.73123 3.46354 2.40513 3.72494
3.29354 2.67741 2.69355 4.24690 2.90347 2.73739 3.18146 2.89801
2.37887 2.77519 2.98518 4.58477 3.61503
0.01397 4.67381 5.39616 0.61958 0.77255 0.48576 0.95510
142 2.98803 5.53079 1.83314 1.77627 4.85566 2.69423 3.89666
4.35512 2.73742 3.83290 4.60300 2.43721 4.05142 3.01849 3.26887
2.91977 2.02076 3.91566 5.97350 4.52476 196 e - - -

2.68618 4.42225 2.77519 2.73123 3.46354 2.40513 3.72494
3.29354 2.67741 2.69355 4.24690 2.90347 2.73739 3.18146 2.89801
2.37887 2.77519 2.98518 4.58477 3.61503
0.01397 4.67381 5.39616 0.61958 0.77255 0.48576 0.95510
143 2.77094 4.37947 3.85398 3.28601 3.47791 3.79719 4.06792
2.17608 3.21290 1.92191 3.48868 2.74817 4.17524 3.49243 3.50661
1.96124 3.00419 2.63504 4.96053 2.87485 197 l - - -
2.68618 4.42225 2.77519 2.73123 3.46354 2.40513 3.72494
3.29354 2.67741 2.69355 4.24690 2.90347 2.73739 3.18146 2.89801
2.37887 2.77519 2.98518 4.58477 3.61503
0.01397 4.67381 5.39616 0.61958 0.77255 0.48576 0.95510
144 2.66207 6.02947 0.79361 2.13621 5.32286 3.58893 4.16216
4.86993 3.22423 4.33253 5.18380 2.45846 4.23000 3.32793 3.86337
3.22759 3.66250 4.40968 6.48542 4.93255 198 d - - -
2.68618 4.42225 2.77519 2.73123 3.46354 2.40513 3.72494
3.29354 2.67741 2.69355 4.24690 2.90347 2.73739 3.18146 2.89801
2.37887 2.77519 2.98518 4.58477 3.61503
0.01397 4.67381 5.39616 0.61958 0.77255 0.48576 0.95510
145 3.34109 6.01068 0.90751 1.74557 5.29587 3.59536 4.12787
4.83959 2.35171 4.29025 5.12879 3.03862 4.21730 3.28588 3.73531
3.20476 3.62538 4.38020 6.42894 4.89315 199 d - - -
2.68618 4.42225 2.77519 2.73123 3.46354 2.40513 3.72494
3.29354 2.67741 2.69355 4.24690 2.90347 2.73739 3.18146 2.89801
2.37887 2.77519 2.98518 4.58477 3.61503
0.01397 4.67381 5.39616 0.61958 0.77255 0.48576 0.95510
146 2.79608 5.29189 2.27696 1.71793 4.62537 2.97717 3.13943
4.10717 2.03224 3.59182 4.33834 3.00597 3.94824 2.85328 2.99650
2.75710 3.02985 2.90098 5.72892 4.31814 200 e - - -
2.68618 4.42225 2.77519 2.73123 3.46354 2.40513 3.72494
3.29354 2.67741 2.69355 4.24690 2.90347 2.73739 3.18146 2.89801
2.37887 2.77519 2.98518 4.58477 3.61503
0.01397 4.67381 5.39616 0.61958 0.77255 0.48576 0.95510
147 2.09219 5.26438 2.57440 1.70460 4.61024 3.54051 3.79970
4.08187 2.57725 3.59062 4.35273 3.03294 3.97547 2.91330 3.07454
2.15364 2.37041 3.66503 5.74757 4.34422 201 e - - -
2.68618 4.42225 2.77519 2.73123 3.46354 2.40513 3.72494
3.29354 2.67741 2.69355 4.24690 2.90347 2.73739 3.18146 2.89801
2.37887 2.77519 2.98518 4.58477 3.61503
0.30223 4.67381 1.38033 0.61958 0.77255 0.48576 0.95510
148 2.94390 5.17233 3.35112 2.71329 4.52381 3.68157 3.72187
2.75881 1.77472 3.41336 4.23673 3.18518 4.04710 2.12337 1.71758
2.94370 3.13997 3.57112 5.54104 4.28099 202 r - - -
2.68618 4.42225 2.77519 2.73123 3.46354 2.40513 3.72494
3.29354 2.67741 2.69355 4.24690 2.90347 2.73739 3.18146 2.89801
2.37887 2.77519 2.98518 4.58477 3.61503
0.01859 4.39017 5.11252 0.61958 0.77255 0.89293 0.52671
149 2.24923 5.11359 2.78570 2.03537 4.51757 3.42495 3.78664
3.97703 2.60175 3.51632 4.30383 2.17417 3.91330 2.92025 3.09202
1.78569 3.03135 3.56959 5.69642 4.30689 203 s - - -
2.68618 4.42225 2.77519 2.73123 3.46354 2.40513 3.72494
3.29354 2.67741 2.69355 4.24690 2.90347 2.73739 3.18146 2.89801
2.37887 2.77519 2.98518 4.58477 3.61503
0.01859 4.39017 5.11252 0.61958 0.77255 0.89293 0.52671

```
     150   2.40439  5.11848  2.92532  1.90404  4.44887  3.47939  3.70184
 3.89123  1.82656  3.42147  4.20229  2.97902  3.89998  2.82232  2.87376
 2.72757  2.26398  3.50420  5.58925  4.21920    204 k - - -
          2.68618  4.42225  2.77519  2.73123  3.46354  2.40513  3.72494
 3.29354  2.67741  2.69355  4.24690  2.90347  2.73739  3.18146  2.89801
 2.37887  2.77519  2.98518  4.58477  3.61503
          0.26300  4.39017  1.51933  0.61958  0.77255  0.29582  1.36227
     151   3.40663  4.68267  5.30901  4.74065  3.41760  4.75123  5.13277
 1.78499  4.60314  1.29402  2.03229  4.87597  4.95872  4.66702  4.64710
 4.10692  3.63467  1.51590  5.41793  4.34088    205 l - - -
          2.68618  4.42225  2.77519  2.73123  3.46354  2.40513  3.72494
 3.29354  2.67741  2.69355  4.24690  2.90347  2.73739  3.18146  2.89801
 2.37887  2.77519  2.98518  4.58477  3.61503
          0.01666  4.49913  5.22148  0.61958  0.77255  0.76805  0.62347
     152   1.97113  4.62770  3.41096  2.85590  3.87813  3.59092  3.89557
 3.24368  2.73756  2.00643  3.79416  3.29657  4.01786  3.12925  2.22590
 2.31938  2.97273  2.97581  5.24640  3.98436    206 a - - -
          2.68618  4.42225  2.77519  2.73123  3.46354  2.40513  3.72494
 3.29354  2.67741  2.69355  4.24690  2.90347  2.73739  3.18146  2.89801
 2.37887  2.77519  2.98518  4.58477  3.61503
          0.01666  4.49913  5.22148  0.61958  0.77255  0.76805  0.62347
     153   2.71017  5.11825  2.67935  1.99656  4.42225  3.48935  3.68837
 3.88055  1.97933  3.40565  4.17134  2.97411  3.88732  2.80100  2.92745
 2.20675  2.94208  2.76964  5.57558  3.41258    207 k - - -
          2.68618  4.42225  2.77519  2.73123  3.46354  2.40513  3.72494
 3.29354  2.67741  2.69355  4.24690  2.90347  2.73739  3.18146  2.89801
 2.37887  2.77519  2.98518  4.58477  3.61503
          0.01666  4.49913  5.22148  0.61958  0.77255  0.76805  0.62347
     154   1.42107  5.14820  2.81528  1.33738  4.68138  3.48392  4.04172
 4.05755  2.91523  3.69051  4.53950  3.13427  4.06610  3.21080  3.38222
 2.93972  3.24681  3.66578  5.91141  4.54400    208 e - - -
          2.68618  4.42225  2.77519  2.73123  3.46354  2.40513  3.72494
 3.29354  2.67741  2.69355  4.24690  2.90347  2.73739  3.18146  2.89801
 2.37887  2.77519  2.98518  4.58477  3.61503
          0.01666  4.49913  5.22148  0.61958  0.77255  0.76805  0.62347
     155   2.99346  4.69526  3.91407  3.29662  3.70321  3.92443  4.11287
 2.89814  2.82611  1.66994  3.61302  3.66685  4.29442  3.39446  1.50560
 3.22465  3.22173  2.34293  5.24235  4.02903    209 r - - -
          2.68618  4.42225  2.77519  2.73123  3.46354  2.40513  3.72494
 3.29354  2.67741  2.69355  4.24690  2.90347  2.73739  3.18146  2.89801
 2.37887  2.77519  2.98518  4.58477  3.61503
          0.01666  4.49913  5.22148  0.61958  0.77255  0.76805  0.62347
     156   2.70688  4.89944  3.09520  2.13239  4.13979  2.77734  3.74086
 2.88313  2.30571  2.69095  3.97464  3.06638  3.92579  2.89306  2.99582
 2.02328  2.93905  3.23169  5.40520  4.07265    210 s - - -
          2.68618  4.42225  2.77519  2.73123  3.46354  2.40513  3.72494
 3.29354  2.67741  2.69355  4.24690  2.90347  2.73739  3.18146  2.89801
 2.37887  2.77519  2.98518  4.58477  3.61503
          0.01666  4.49913  5.22148  0.61958  0.77255  0.76805  0.62347
     157   3.07743  5.69541  1.83730  1.43875  4.99809  3.47875  3.92296
 4.52272  2.85065  3.98295  4.78113  1.88136  4.04355  2.48994  3.42174
 2.97283  3.34402  4.06961  6.12064  4.62478    211 e - - -
          2.68618  4.42225  2.77519  2.73123  3.46354  2.40513  3.72494
 3.29354  2.67741  2.69355  4.24690  2.90347  2.73739  3.18146  2.89801
 2.37887  2.77519  2.98518  4.58477  3.61503
```

```
          0.01666  4.49913  5.22148  0.61958  0.77255  0.76805  0.62347
    158   2.79170  4.35575  3.94340  3.37274  3.38029  3.82977  2.98333
1.53236  3.19579  1.90694  3.39945  3.68286  4.19756  2.97027  3.44499
3.11033  3.02149  2.55707  4.90898  3.67667    212 i - - -
          2.68618  4.42225  2.77519  2.73123  3.46354  2.40513  3.72494
3.29354  2.67741  2.69355  4.24690  2.90347  2.73739  3.18146  2.89801
2.37887  2.77519  2.98518  4.58477  3.61503
          0.01666  4.49913  5.22148  0.61958  0.77255  0.76805  0.62347
    159   3.53837  4.76918  5.47703  4.95179  3.60339  5.00670  5.49378
1.03467  4.84152  1.30913  3.36385  5.13141  5.17890  4.94424  4.92685
4.40178  3.77757  1.76504  5.72117  4.62930    213 i - - -
          2.68618  4.42225  2.77519  2.73123  3.46354  2.40513  3.72494
3.29354  2.67741  2.69355  4.24690  2.90347  2.73739  3.18146  2.89801
2.37887  2.77519  2.98518  4.58477  3.61503
          0.01666  4.49913  5.22148  0.61958  0.77255  0.76805  0.62347
    160   2.72804  5.22809  2.38790  2.22960  4.57190  3.48379  3.67604
4.05324  1.94629  3.52911  4.27155  2.69279  3.88437  2.47474  2.29599
2.68944  2.59684  3.61857  5.65702  4.25160    214 k - - -
          2.68618  4.42225  2.77519  2.73123  3.46354  2.40513  3.72494
3.29354  2.67741  2.69355  4.24690  2.90347  2.73739  3.18146  2.89801
2.37887  2.77519  2.98518  4.58477  3.61503
          0.01666  4.49913  5.22148  0.61958  0.77255  0.76805  0.62347
    161   2.80760  5.15296  2.46774  2.46750  4.47796  3.48681  3.80636
3.93816  2.62430  2.82589  4.28559  1.65224  3.95505  2.94274  3.11585
2.00392  3.06303  3.55464  5.67832  4.28947    215 n - - -
          2.68618  4.42225  2.77519  2.73123  3.46354  2.40513  3.72494
3.29354  2.67741  2.69355  4.24690  2.90347  2.73739  3.18146  2.89801
2.37887  2.77519  2.98518  4.58477  3.61503
          0.01666  4.49913  5.22148  0.61958  0.77255  0.34269  1.23738
    162   2.04754  4.65313  4.81573  4.24942  3.41120  4.37808  4.75419
2.58152  4.09701  1.15443  1.73538  4.43988  4.69926  4.26829  4.22960
3.70770  3.47857  2.59075  5.25275  4.13644    216 l - - -
          2.68618  4.42225  2.77519  2.73123  3.46354  2.40513  3.72494
3.29354  2.67741  2.69355  4.24690  2.90347  2.73739  3.18146  2.89801
2.37887  2.77519  2.98518  4.58477  3.61503
          0.01397  4.67381  5.39616  0.61958  0.77255  0.48576  0.95510
    163   2.20293  5.10346  3.08151  2.52970  4.39070  2.96112  2.95949
3.00059  1.72150  3.38472  4.16642  2.76268  3.95641  2.88192  2.98844
2.58465  2.99679  3.46401  5.57782  4.21158    217 k - - -
          2.68618  4.42225  2.77519  2.73123  3.46354  2.40513  3.72494
3.29354  2.67741  2.69355  4.24690  2.90347  2.73739  3.18146  2.89801
2.37887  2.77519  2.98518  4.58477  3.61503
          0.01397  4.67381  5.39616  0.61958  0.77255  0.48576  0.95510
    164   2.50087  5.28338  3.03671  2.60687  4.71629  2.94737  3.87374
4.17089  1.29877  3.67173  4.45132  2.04236  4.05337  2.99496  2.98288
2.89334  3.16539  3.75288  5.80954  4.44498    218 k - - -
          2.68618  4.42225  2.77519  2.73123  3.46354  2.40513  3.72494
3.29354  2.67741  2.69355  4.24690  2.90347  2.73739  3.18146  2.89801
2.37887  2.77519  2.98518  4.58477  3.61503
          0.01397  4.67381  5.39616  0.61958  0.77255  0.48576  0.95510
    165   4.08985  6.12731  0.23971  3.20110  5.66984  4.04604  4.92330
5.49390  4.18879  4.96129  6.01868  3.77687  4.74876  4.20483  4.76141
4.00381  4.46126  5.06278  6.63751  5.49385    219 D - - -
```

```
          2.68618  4.42225  2.77519  2.73123  3.46354  2.40513  3.72494
3.29354  2.67741  2.69355  4.24690  2.90347  2.73739  3.18146  2.89801
2.37887  2.77519  2.98518  4.58477  3.61503
          0.01397  4.67381  5.39616  0.61958  0.77255  0.48576  0.95510
    166   3.49035  4.74370  5.47588  4.92992  2.60840  4.89769  5.32771
0.96939  4.80243  1.78188  3.45973  5.04005  5.11856  4.89890  4.85525
4.26773  3.72704  1.63240  5.62133  4.48503    220 i - - -
          2.68618  4.42225  2.77519  2.73123  3.46354  2.40513  3.72494
3.29354  2.67741  2.69355  4.24690  2.90347  2.73739  3.18146  2.89801
2.37887  2.77519  2.98518  4.58477  3.61503
          0.01397  4.67381  5.39616  0.61958  0.77255  0.48576  0.95510
    167   2.31715  5.27905  2.64008  2.29891  4.61813  3.53451  3.73452
4.10092  1.87017  3.58104  4.32316  2.67676  3.93732  2.07534  2.96716
2.74046  2.55393  3.66684  5.71364  4.30411    221 k - - -
          2.68618  4.42225  2.77519  2.73123  3.46354  2.40513  3.72494
3.29354  2.67741  2.69355  4.24690  2.90347  2.73739  3.18146  2.89801
2.37887  2.77519  2.98518  4.58477  3.61503
          0.30223  4.67381  1.38033  0.61958  0.77255  0.48576  0.95510
    168   1.98892  5.07054  2.91152  2.43656  4.38814  2.76837  2.99580
3.83879  2.46768  3.38029  4.16337  2.63577  3.88755  2.12268  2.93676
2.70852  2.95868  3.45737  5.56241  4.18887    222 a - - -
          2.68618  4.42225  2.77519  2.73123  3.46354  2.40513  3.72494
3.29354  2.67741  2.69355  4.24690  2.90347  2.73739  3.18146  2.89801
2.37887  2.77519  2.98518  4.58477  3.61503
          0.01859  4.39017  5.11252  0.61958  0.77255  0.89293  0.52671
    169   2.87747  4.47995  3.96078  3.36875  3.53120  3.88930  4.14394
2.61306  3.05144  1.77077  3.46258  3.70760  4.25868  3.50109  2.06512
3.18352  3.11246  1.69778  5.10087  3.89172    223 v - - -
          2.68618  4.42225  2.77519  2.73123  3.46354  2.40513  3.72494
3.29354  2.67741  2.69355  4.24690  2.90347  2.73739  3.18146  2.89801
2.37887  2.77519  2.98518  4.58477  3.61503
          0.01859  4.39017  5.11252  0.61958  0.77255  0.89293  0.52671
    170   2.72290  2.41064  3.74263  3.14863  3.52345  3.71012  3.95349
1.93808  2.40871  2.57591  3.49087  3.50239  4.09059  3.31166  2.60141
2.98411  2.95447  2.60513  4.97199  3.75790    224 i - - -
          2.68618  4.42225  2.77519  2.73123  3.46354  2.40513  3.72494
3.29354  2.67741  2.69355  4.24690  2.90347  2.73739  3.18146  2.89801
2.37887  2.77519  2.98518  4.58477  3.61503
          0.01859  4.39017  5.11252  0.61958  0.77255  0.89293  0.52671
    171   2.77297  5.17418  3.03661  2.48272  4.52682  2.72756  3.67515
3.97286  1.79865  3.46308  4.23554  3.00718  3.91609  2.36670  2.31723
2.19988  2.99586  3.57398  5.59308  4.24042    225 k - - -
          2.68618  4.42225  2.77519  2.73123  3.46354  2.40513  3.72494
3.29354  2.67741  2.69355  4.24690  2.90347  2.73739  3.18146  2.89801
2.37887  2.77519  2.98518  4.58477  3.61503
          0.01859  4.39017  5.11252  0.61958  0.77255  0.29582  1.36227
    172   2.78158  4.85828  3.26739  2.70288  4.07099  3.63539  3.83534
2.39853  2.24121  2.34253  3.93944  2.53419  4.02186  3.01421  2.41743
2.30978  3.01071  3.17504  5.37671  4.07514    226 k - - -
          2.68618  4.42225  2.77519  2.73123  3.46354  2.40513  3.72494
3.29354  2.67741  2.69355  4.24690  2.90347  2.73739  3.18146  2.89801
2.37887  2.77519  2.98518  4.58477  3.61503
          0.01397  4.67381  5.39616  0.61958  0.77255  0.48576  0.95510
```

```
    173   2.96637  4.58342  3.94765  3.39575  2.01013  3.95148  3.13865
  3.01897  3.27544  1.57722  3.62093  3.72659  4.31856  2.11488  3.56265
  3.22213  3.19531  2.84571  4.78184  3.34455    227 l - - -
          2.68618  4.42225  2.77519  2.73123  3.46354  2.40513  3.72494
  3.29354  2.67741  2.69355  4.24690  2.90347  2.73739  3.18146  2.89801
  2.37887  2.77519  2.98518  4.58477  3.61503
          0.01397  4.67381  5.39616  0.61958  0.77255  0.48576  0.95510
    174   2.93583  4.96718  3.44833  2.85692  4.17830  3.75519  3.19359
  1.83696  2.51535  3.19023  4.04869  3.32347  4.13291  1.76270  2.30898
  3.00793  3.15072  3.29660  5.43203  4.15959    228 q - - -
          2.68618  4.42225  2.77519  2.73123  3.46354  2.40513  3.72494
  3.29354  2.67741  2.69355  4.24690  2.90347  2.73739  3.18146  2.89801
  2.37887  2.77519  2.98518  4.58477  3.61503
          0.01397  4.67381  5.39616  0.61958  0.77255  0.48576  0.95510
    175   2.54097  5.26960  2.65140  1.89683  4.60899  2.99432  3.73000
  4.09083  1.79574  3.57183  4.31370  3.00341  3.93406  2.82984  2.67868
  2.73535  2.49181  3.65758  5.70483  4.29690    229 k - - -
          2.68618  4.42225  2.77519  2.73123  3.46354  2.40513  3.72494
  3.29354  2.67741  2.69355  4.24690  2.90347  2.73739  3.18146  2.89801
  2.37887  2.77519  2.98518  4.58477  3.61503
          0.01397  4.67381  5.39616  0.61958  0.77255  0.48576  0.95510
    176   2.50410  5.31576  2.95643  1.53128  4.65557  3.56252  3.78446
  4.12801  1.76974  3.61709  4.37528  2.71776  3.98449  2.89319  2.99055
  2.80794  2.69742  3.70673  5.75539  4.35633    230 e - - -
          2.68618  4.42225  2.77519  2.73123  3.46354  2.40513  3.72494
  3.29354  2.67741  2.69355  4.24690  2.90347  2.73739  3.18146  2.89801
  2.37887  2.77519  2.98518  4.58477  3.61503
          0.01397  4.67381  5.39616  0.61958  0.77255  0.48576  0.95510
    177   1.56779  4.74164  3.45620  2.70690  4.42795  3.50236  4.27002
  3.75540  3.16800  3.47171  4.32597  3.47621  4.11768  3.49358  3.55538
  2.89590  1.17490  3.37623  5.76775  4.50100    231 t - - -
          2.68618  4.42225  2.77519  2.73123  3.46354  2.40513  3.72494
  3.29354  2.67741  2.69355  4.24690  2.90347  2.73739  3.18146  2.89801
  2.37887  2.77519  2.98518  4.58477  3.61503
          0.01397  4.67381  5.39616  0.61958  0.77255  0.48576  0.95510
    178   2.78192  5.28824  2.52984  1.76528  4.63074  3.53618  3.73416
  4.11486  2.18120  3.12893  4.33140  2.18931  3.93873  2.61356  2.61657
  2.74242  3.01387  3.67787  5.71955  4.30959    232 e - - -
          2.68618  4.42225  2.77519  2.73123  3.46354  2.40513  3.72494
  3.29354  2.67741  2.69355  4.24690  2.90347  2.73739  3.18146  2.89801
  2.37887  2.77519  2.98518  4.58477  3.61503
          0.01397  4.67381  5.39616  0.61958  0.77255  0.48576  0.95510
    179   2.79066  5.13065  3.11029  2.30566  4.42231  3.58309  3.76228
  3.27399  2.29754  2.34534  4.18858  2.72492  3.97312  1.81463  2.66524
  2.78643  3.01856  3.49278  5.58964  4.23058    233 q - - -
          2.68618  4.42225  2.77519  2.73123  3.46354  2.40513  3.72494
  3.29354  2.67741  2.69355  4.24690  2.90347  2.73739  3.18146  2.89801
  2.37887  2.77519  2.98518  4.58477  3.61503
          0.01397  4.67381  5.39616  0.61958  0.77255  0.48576  0.95510
    180   3.07520  4.44385  4.87206  4.27668  3.29336  4.25967  4.55819
  2.54407  4.10189  1.35634  2.31595  4.36055  4.57516  4.22777  4.15944
  3.57522  3.30352  1.71930  5.00777  2.26812    234 l - - -
          2.68618  4.42225  2.77519  2.73123  3.46354  2.40513  3.72494
  3.29354  2.67741  2.69355  4.24690  2.90347  2.73739  3.18146  2.89801
  2.37887  2.77519  2.98518  4.58477  3.61503
```

```
         0.01397  4.67381  5.39616  0.61958  0.77255  0.48576  0.95510
    181  2.43810  5.27372  2.30960  2.17427  4.61245  3.53884  3.73546
4.09253  1.70334  3.57465  4.31871  3.00857  3.94025  2.83631  2.64295
2.74409  2.45167  3.66146  5.70825  4.30239    235 k - - -
         2.68618  4.42225  2.77519  2.73123  3.46354  2.40513  3.72494
3.29354  2.67741  2.69355  4.24690  2.90347  2.73739  3.18146  2.89801
2.37887  2.77519  2.98518  4.58477  3.61503
         0.01397  4.67381  5.39616  0.61958  0.77255  0.48576  0.95510
    182  2.39708  5.26776  2.63713  2.17152  4.60616  3.00691  3.73090
4.08850  1.88807  3.57088  4.31281  2.72327  3.93279  2.83104  2.96815
2.24241  2.51791  3.65536  5.70542  4.29611    236 k - - -
         2.68618  4.42225  2.77519  2.73123  3.46354  2.40513  3.72494
3.29354  2.67741  2.69355  4.24690  2.90347  2.73739  3.18146  2.89801
2.37887  2.77519  2.98518  4.58477  3.61503
         0.01397  4.67381  5.39616  0.61958  0.77255  0.48576  0.95510
    183  1.87783  5.23624  3.08941  2.33245  4.57409  3.58635  3.76143
4.02888  1.92220  3.53064  4.29619  3.07161  3.98208  2.87094  2.25043
2.18024  3.04984  3.62666  5.67498  4.30417    237 a - - -
         2.68618  4.42225  2.77519  2.73123  3.46354  2.40513  3.72494
3.29354  2.67741  2.69355  4.24690  2.90347  2.73739  3.18146  2.89801
2.37887  2.77519  2.98518  4.58477  3.61503
         0.01397  4.67381  5.39616  0.61958  0.77255  0.48576  0.95510
    184  1.77728  4.38502  4.49983  3.95091  3.62650  4.01255  4.51914
1.57580  3.83721  2.58629  3.58495  4.11519  4.44303  4.06577  4.01851
2.64170  3.18352  1.60195  5.20337  4.00792    238 i - - -
         2.68618  4.42225  2.77519  2.73123  3.46354  2.40513  3.72494
3.29354  2.67741  2.69355  4.24690  2.90347  2.73739  3.18146  2.89801
2.37887  2.77519  2.98518  4.58477  3.61503
         0.01397  4.67381  5.39616  0.61958  0.77255  0.48576  0.95510
    185  2.03961  5.22894  2.51697  2.48364  4.57128  3.53775  3.76662
4.04186  2.21015  3.54821  4.30513  2.66118  3.95381  2.87583  3.01634
1.80165  2.72682  3.62550  5.70219  4.30391    239 s - - -
         2.68618  4.42225  2.77519  2.73123  3.46354  2.40513  3.72494
3.29354  2.67741  2.69355  4.24690  2.90347  2.73739  3.18146  2.89801
2.37887  2.77519  2.98518  4.58477  3.61503
         0.01397  4.67381  5.39616  0.61958  0.77255  0.48576  0.95510
    186  3.51596  6.15869  0.67413  2.49633  5.47584  3.61822  4.29701
5.11266  3.46577  4.57019  5.47940  1.91870  4.31258  3.48823  4.15435
3.36020  3.84714  4.62779  6.66978  5.08435    240 D - - -
         2.68618  4.42225  2.77519  2.73123  3.46354  2.40513  3.72494
3.29354  2.67741  2.69355  4.24690  2.90347  2.73739  3.18146  2.89801
2.37887  2.77519  2.98518  4.58477  3.61503
         0.01397  4.67381  5.39616  0.61958  0.77255  0.48576  0.95510
    187  3.02458  4.37713  4.93166  4.33411  2.01078  4.22420  4.52575
1.88611  4.14413  1.68571  3.28651  4.36031  4.55009  4.25740  4.16842
3.54373  3.25496  1.65462  4.95803  2.98949    241 v - - -
         2.68618  4.42225  2.77519  2.73123  3.46354  2.40513  3.72494
3.29354  2.67741  2.69355  4.24690  2.90347  2.73739  3.18146  2.89801
2.37887  2.77519  2.98518  4.58477  3.61503
         0.01397  4.67381  5.39616  0.61958  0.77255  0.48576  0.95510
    188  2.85691  5.20762  3.17993  2.60298  4.52969  3.63067  2.56443
3.96579  1.80382  3.48368  4.26675  3.12312  4.01624  2.89387  2.10852
2.13814  3.07844  2.83161  5.63745  4.29443    242 k - - -
```

```
          2.68618  4.42225  2.77519  2.73123  3.46354  2.40513  3.72494
3.29354  2.67741  2.69355  4.24690  2.90347  2.73739  3.18146  2.89801
2.37887  2.77519  2.98518  4.58477  3.61503
          0.19134  4.67381  1.80297  0.61958  0.77255  0.48576  0.95510
    189   2.77853  4.62488  3.45296  2.90305  3.77196  3.68239  3.91130
2.13164  2.76459  2.49665  3.71695  1.73083  4.07392  3.16718  2.77109
2.93802  3.01215  2.83841  5.18286  3.91657    243 n - - -
          2.68618  4.42225  2.77519  2.73123  3.46354  2.40513  3.72494
3.29354  2.67741  2.69355  4.24690  2.90347  2.73739  3.18146  2.89801
2.37887  2.77519  2.98518  4.58477  3.61503
          0.01666  4.49913  5.22148  0.61958  0.77255  0.76805  0.62347
    190   2.71719  4.51345  3.47298  2.53711  3.67327  3.66063  3.90586
2.98159  2.55770  2.72498  2.97770  3.34939  4.04525  3.18988  3.22833
2.90306  2.20016  1.76913  5.09624  3.84803    244 v - - -
          2.68618  4.42225  2.77519  2.73123  3.46354  2.40513  3.72494
3.29354  2.67741  2.69355  4.24690  2.90347  2.73739  3.18146  2.89801
2.37887  2.77519  2.98518  4.58477  3.61503
          0.01666  4.49913  5.22148  0.61958  0.77255  0.76805  0.62347
    191   3.47296  4.71705  5.40085  4.87116  3.62021  4.91883  5.40253
1.73130  4.75941  1.20283  3.39342  5.03925  5.11884  4.88311  4.85310
4.30379  3.71365  1.17041  5.68412  4.57341    245 v - - -
          2.68618  4.42225  2.77519  2.73123  3.46354  2.40513  3.72494
3.29354  2.67741  2.69355  4.24690  2.90347  2.73739  3.18146  2.89801
2.37887  2.77519  2.98518  4.58477  3.61503
          0.01666  4.49913  5.22148  0.61958  0.77255  0.76805  0.62347
    192   2.80922  4.39207  4.25934  3.79817  3.74016  3.79410  4.51150
1.01144  3.68420  2.65502  3.68619  3.96998  4.32490  3.97286  3.90100
2.53527  2.56041  2.29631  5.32569  4.11914    246 i - - -
          2.68627  4.42234  2.77529  2.73132  3.46363  2.40522  3.72504
3.29253  2.67750  2.69312  4.24699  2.90356  2.73749  3.18155  2.89810
2.37877  2.77529  2.98527  4.58486  3.61512
          1.14721  0.95977  1.20564  0.11573  2.21384  0.34269  1.23738
    193   2.86384  5.25920  2.79853  1.35634  4.60144  3.49454  3.76522
4.04171  2.05816  3.55968  4.35652  2.98567  3.95811  2.89369  2.91161
2.82807  2.42642  3.65115  5.71372  4.33865    248 e - - -
          2.68618  4.42225  2.77519  2.73123  3.46354  2.40513  3.72494
3.29354  2.67741  2.69355  4.24690  2.90347  2.73739  3.18146  2.89801
2.37887  2.77519  2.98518  4.58477  3.61503
          0.01859  4.39017  5.11252  0.61958  0.77255  0.89293  0.52671
    194   2.75385  5.03153  3.06509  2.54414  4.38613  2.33984  2.76470
3.82715  2.40596  3.37009  4.16768  3.05348  3.92988  2.86212  1.93133
2.28660  2.99588  3.45607  5.53933  4.20257    249 r - - -
          2.68618  4.42225  2.77519  2.73123  3.46354  2.40513  3.72494
3.29354  2.67741  2.69355  4.24690  2.90347  2.73739  3.18146  2.89801
2.37887  2.77519  2.98518  4.58477  3.61503
          0.01859  4.39017  5.11252  0.61958  0.77255  0.89293  0.52671
    195   3.00027  4.56813  4.29424  3.83087  3.49885  3.97350  4.55810
2.55327  3.65153  1.01135  3.39747  4.06599  4.44057  3.97185  3.86346
3.36185  1.99316  2.49272  5.28556  4.10001    250 l - - -
          2.68618  4.42225  2.77519  2.73123  3.46354  2.40513  3.72494
3.29354  2.67741  2.69355  4.24690  2.90347  2.73739  3.18146  2.89801
2.37887  2.77519  2.98518  4.58477  3.61503
          0.01859  4.39017  5.11252  0.61958  0.77255  0.89293  0.52671
```

```
    196   3.09855  5.73789  1.38166  1.67492  5.03269  2.60105  3.92163
4.56775  2.91101  4.02895  4.84902  2.16163  4.01795  3.07273  3.51328
2.97829  3.37752  4.11210  6.17408  4.65299    251 d - - -
          2.68618  4.42225  2.77519  2.73123  3.46354  2.40513  3.72494
3.29354  2.67741  2.69355  4.24690  2.90347  2.73739  3.18146  2.89801
2.37887  2.77519  2.98518  4.58477  3.61503
          0.01859  4.39017  5.11252  0.61958  0.77255  0.89293  0.52671
    197   2.70379  5.19235  2.51790  2.18226  4.52467  3.06611  3.65745
4.00418  1.92461  3.49151  4.24023  2.91468  3.85593  2.34822  2.89913
2.47973  2.45526  3.57689  5.63057  4.22238    252 k - - -
          2.68618  4.42225  2.77519  2.73123  3.46354  2.40513  3.72494
3.29354  2.67741  2.69355  4.24690  2.90347  2.73739  3.18146  2.89801
2.37887  2.77519  2.98518  4.58477  3.61503
          0.01859  4.39017  5.11252  0.61958  0.77255  0.89293  0.52671
    198   2.68123  4.20318  3.99473  3.41645  2.18452  3.75327  4.04440
2.62064  3.30402  2.11380  3.30508  3.67836  4.12792  3.55794  2.97690
2.50902  2.91591  1.88150  4.79531  3.57872    253 v - - -
          2.68618  4.42225  2.77519  2.73123  3.46354  2.40513  3.72494
3.29354  2.67741  2.69355  4.24690  2.90347  2.73739  3.18146  2.89801
2.37887  2.77519  2.98518  4.58477  3.61503
          0.01859  4.39017  5.11252  0.61958  0.77255  0.89293  0.52671
    199   3.79659  5.12199  5.02308  4.69437  3.42476  4.63707  5.19504
2.68084  4.46208  0.42707  3.33574  4.92420  5.00170  4.69872  4.54113
4.31835  4.07643  2.84682  5.45820  4.29191    254 L - - -
          2.68618  4.42225  2.77519  2.73123  3.46354  2.40513  3.72494
3.29354  2.67741  2.69355  4.24690  2.90347  2.73739  3.18146  2.89801
2.37887  2.77519  2.98518  4.58477  3.61503
          0.01859  4.39017  5.11252  0.61958  0.77255  0.89293  0.52671
    200   2.44395  5.20076  3.08041  2.53775  4.54967  3.56531  2.75008
3.98508  1.61201  3.47482  4.26319  3.05180  3.95984  2.05913  2.64718
2.81514  3.05466  3.60185  5.59755  4.26784    255 k - - -
          2.68618  4.42225  2.77519  2.73123  3.46354  2.40513  3.72494
3.29354  2.67741  2.69355  4.24690  2.90347  2.73739  3.18146  2.89801
2.37887  2.77519  2.98518  4.58477  3.61503
          0.01859  4.39017  5.11252  0.61958  0.77255  0.29582  1.36227
    201   2.11310  5.31056  2.85260  1.99389  4.70145  1.46394  3.94361
4.16159  2.76780  3.69799  4.49460  3.09924  4.06960  2.59695  3.25558
2.92700  3.21859  3.75753  5.87783  4.48354    256 g - - -
          2.68618  4.42225  2.77519  2.73123  3.46354  2.40513  3.72494
3.29354  2.67741  2.69355  4.24690  2.90347  2.73739  3.18146  2.89801
2.37887  2.77519  2.98518  4.58477  3.61503
          0.01397  4.67381  5.39616  0.61958  0.77255  0.48576  0.95510
    202   2.57634  5.68674  2.12648  1.46629  5.03288  1.53421  4.03106
4.54197  2.96271  4.02731  4.83020  3.04183  4.13677  3.17441  3.52848
3.05627  3.41803  4.09784  6.18022  4.70237    257 e - - -
          2.68618  4.42225  2.77519  2.73123  3.46354  2.40513  3.72494
3.29354  2.67741  2.69355  4.24690  2.90347  2.73739  3.18146  2.89801
2.37887  2.77519  2.98518  4.58477  3.61503
          0.01397  4.67381  5.39616  0.61958  0.77255  0.48576  0.95510
    203   2.85385  5.33267  2.56077  2.25134  4.66417  3.56063  3.78890
4.13949  2.53363  3.62787  4.38639  3.03106  3.98663  1.40712  2.68339
2.81297  2.52479  3.71792  5.76717  4.36325    258 q - - -
          2.68618  4.42225  2.77519  2.73123  3.46354  2.40513  3.72494
3.29354  2.67741  2.69355  4.24690  2.90347  2.73739  3.18146  2.89801
2.37887  2.77519  2.98518  4.58477  3.61503
```

```
          0.01397  4.67381  5.39616  0.61958  0.77255  0.48576  0.95510
    204   2.83942  5.18514  2.48133  2.06357  4.46247  3.57262  3.81395
3.90566  2.24401  1.68682  4.25099  3.06656  3.99816  2.94428  3.08042
2.49983  3.07545  3.53925  5.65715  4.28241    259 l - - -
          2.68618  4.42225  2.77519  2.73123  3.46354  2.40513  3.72494
3.29354  2.67741  2.69355  4.24690  2.90347  2.73739  3.18146  2.89801
2.37887  2.77519  2.98518  4.58477  3.61503
          0.09806  4.67381  2.47616  0.61958  0.77255  0.48576  0.95510
    205   2.99176  5.23070  3.32065  2.78176  4.66977  3.69325  3.86238
4.08961  2.37465  3.58955  4.40488  3.25678  4.11850  2.51907  1.59307
1.49851  3.22550  3.71558  5.70549  4.42247    260 s - - -
          2.68618  4.42225  2.77519  2.73123  3.46354  2.40513  3.72494
3.29354  2.67741  2.69355  4.24690  2.90347  2.73739  3.18146  2.89801
2.37887  2.77519  2.98518  4.58477  3.61503
          0.01518  4.59094  5.31329  0.61958  0.77255  0.63577  0.75402
    206   2.81449  5.32725  2.21795  2.42546  4.66502  3.51442  3.75138
4.15168  1.87607  3.62797  4.37758  2.44066  3.94406  2.17224  3.00455
2.21246  3.05087  3.71559  5.75926  4.34161    261 k - - -
          2.68618  4.42225  2.77519  2.73123  3.46354  2.40513  3.72494
3.29354  2.67741  2.69355  4.24690  2.90347  2.73739  3.18146  2.89801
2.37887  2.77519  2.98518  4.58477  3.61503
          0.10846  4.59094  2.37897  0.61958  0.77255  0.63577  0.75402
    207   2.36751  5.19462  2.91306  2.25998  4.56694  1.88550  3.79669
4.01335  1.73433  3.53861  4.32816  3.03965  3.97473  2.92453  2.96023
2.82021  3.08640  3.61910  5.70179  4.33417    262 k - - -
          2.68618  4.42225  2.77519  2.73123  3.46354  2.40513  3.72494
3.29354  2.67741  2.69355  4.24690  2.90347  2.73739  3.18146  2.89801
2.37887  2.77519  2.98518  4.58477  3.61503
          0.01666  4.49913  5.22148  0.61958  0.77255  0.76805  0.62347
    208   2.81117  5.34071  2.44670  1.76585  4.67525  2.95090  3.73923
4.16742  2.07481  3.64010  4.39204  2.61359  3.92346  2.12315  3.02229
2.75718  3.05049  3.72721  5.77074  4.34312    263 e - - -
          2.68618  4.42225  2.77519  2.73123  3.46354  2.40513  3.72494
3.29354  2.67741  2.69355  4.24690  2.90347  2.73739  3.18146  2.89801
2.37887  2.77519  2.98518  4.58477  3.61503
          0.01666  4.49913  5.22148  0.61958  0.77255  0.76805  0.62347
    209   2.72369  4.54242  3.46086  2.88998  3.70038  3.65498  3.87805
2.34061  2.47033  1.80745  3.63977  2.94118  3.30742  3.14999  2.68763
2.89525  2.95282  2.82531  5.10366  3.85532    264 l - - -
          2.68618  4.42225  2.77519  2.73123  3.46354  2.40513  3.72494
3.29354  2.67741  2.69355  4.24690  2.90347  2.73739  3.18146  2.89801
2.37887  2.77519  2.98518  4.58477  3.61503
          0.01666  4.49913  5.22148  0.61958  0.77255  0.76805  0.62347
    210   2.03775  4.64459  3.35360  2.81933  3.94652  3.55020  3.91068
3.26192  2.78167  2.98006  3.84845  3.26914  3.99866  2.07558  3.18816
2.81703  2.15469  2.26675  5.31319  4.03794    265 a - - -
          2.68618  4.42225  2.77519  2.73123  3.46354  2.40513  3.72494
3.29354  2.67741  2.69355  4.24690  2.90347  2.73739  3.18146  2.89801
2.37887  2.77519  2.98518  4.58477  3.61503
          0.01666  4.49913  5.22148  0.61958  0.77255  0.76805  0.62347
    211   2.19965  5.12174  2.68798  1.96282  2.93598  3.48916  3.69928
3.87868  2.46448  3.40811  4.17742  2.63539  3.89367  2.55989  2.95195
2.41834  2.95362  3.48836  5.58273  4.19662    266 e - - -
```

2.68618 4.42225 2.77519 2.73123 3.46354 2.40513 3.72494
3.29354 2.67741 2.69355 4.24690 2.90347 2.73739 3.18146 2.89801
2.37887 2.77519 2.98518 4.58477 3.61503
0.01666 4.49913 5.22148 0.61958 0.77255 0.76805 0.62347
212 2.37427 5.27455 1.35756 2.53064 4.94981 1.55159 4.10481
4.43783 3.09886 3.98620 4.81001 3.06779 4.06589 3.27629 3.64933
2.95477 3.32436 3.94918 6.15843 4.72691 267 d - - -
2.68618 4.42225 2.77519 2.73123 3.46354 2.40513 3.72494
3.29354 2.67741 2.69355 4.24690 2.90347 2.73739 3.18146 2.89801
2.37887 2.77519 2.98518 4.58477 3.61503
0.01666 4.49913 5.22148 0.61958 0.77255 0.76805 0.62347
213 3.65533 4.88906 5.50907 4.98617 3.46612 5.06845 5.48048
1.08525 4.83636 1.06696 3.23704 5.18847 5.20538 4.90407 4.89672
4.47594 3.88778 2.17943 5.63360 4.56130 268 l - - -
2.68618 4.42225 2.77519 2.73123 3.46354 2.40513 3.72494
3.29354 2.67741 2.69355 4.24690 2.90347 2.73739 3.18146 2.89801
2.37887 2.77519 2.98518 4.58477 3.61503
0.01666 4.49913 5.22148 0.61958 0.77255 0.76805 0.62347
214 2.22958 5.07033 2.98869 2.05194 4.37805 2.88026 3.71209
3.82484 2.47649 3.37010 4.14788 2.99830 3.90009 2.01045 2.95458
2.70788 2.65503 3.08039 5.55683 4.18321 269 q - - -
2.68618 4.42225 2.77519 2.73123 3.46354 2.40513 3.72494
3.29354 2.67741 2.69355 4.24690 2.90347 2.73739 3.18146 2.89801
2.37887 2.77519 2.98518 4.58477 3.61503
0.01666 4.49913 5.22148 0.61958 0.77255 0.76805 0.62347
215 2.70288 4.82295 3.16129 2.62117 4.09257 3.53238 3.78897
2.85128 2.59973 3.12030 3.94912 3.12123 2.33413 2.08519 3.04167
2.43399 2.50254 3.18054 5.38495 4.07002 270 q - - -
2.68618 4.42225 2.77519 2.73123 3.46354 2.40513 3.72494
3.29354 2.67741 2.69355 4.24690 2.90347 2.73739 3.18146 2.89801
2.37887 2.77519 2.98518 4.58477 3.61503
0.01666 4.49913 5.22148 0.61958 0.77255 0.76805 0.62347
216 3.05811 5.43859 3.09114 2.25582 4.84146 3.67690 3.79845
4.26023 1.44048 3.69794 4.50375 3.14984 4.09658 1.60785 2.61199
3.01083 3.26485 3.87002 5.77178 4.47041 271 k - - -
2.68618 4.42225 2.77519 2.73123 3.46354 2.40513 3.72494
3.29354 2.67741 2.69355 4.24690 2.90347 2.73739 3.18146 2.89801
2.37887 2.77519 2.98518 4.58477 3.61503
0.01666 4.49913 5.22148 0.61958 0.77255 0.76805 0.62347
217 2.40506 4.76529 3.22455 1.63470 4.08180 3.62453 4.02315
3.06954 2.90316 3.03666 3.96555 3.30556 4.09747 3.23598 3.32091
2.94943 3.09473 1.65482 5.48619 4.19359 272 e - - -
2.68618 4.42225 2.77519 2.73123 3.46354 2.40513 3.72494
3.29354 2.67741 2.69355 4.24690 2.90347 2.73739 3.18146 2.89801
2.37887 2.77519 2.98518 4.58477 3.61503
0.01666 4.49913 5.22148 0.61958 0.77255 0.76805 0.62347
218 2.44087 5.14291 3.01515 2.26441 4.46022 3.51167 3.68859
3.91514 2.08041 2.46157 4.19438 2.48744 3.90348 2.37979 2.52038
2.71378 2.96178 3.51927 5.58507 4.20968 273 k - - -
2.68618 4.42225 2.77519 2.73123 3.46354 2.40513 3.72494
3.29354 2.67741 2.69355 4.24690 2.90347 2.73739 3.18146 2.89801
2.37887 2.77519 2.98518 4.58477 3.61503
0.01666 4.49913 5.22148 0.61958 0.77255 0.76805 0.62347

219 2.86024 5.37381 2.41141 1.66902 4.70204 3.47636 3.78683
4.19244 2.60384 3.67892 4.44527 2.03147 3.95117 2.90525 3.12266
2.18732 2.64165 3.76063 5.82340 4.38970 274 e - - -
2.68618 4.42225 2.77519 2.73123 3.46354 2.40513 3.72494
3.29354 2.67741 2.69355 4.24690 2.90347 2.73739 3.18146 2.89801
2.37887 2.77519 2.98518 4.58477 3.61503
0.01666 4.49913 5.22148 0.61958 0.77255 0.76805 0.62347
220 3.05969 5.28506 3.47529 2.81524 4.66731 3.77429 3.77771
2.92031 1.16205 3.52061 4.34903 3.27364 4.13375 2.44838 2.07594
3.05280 3.24608 3.70226 5.62127 4.38229 275 k - - -
2.68618 4.42225 2.77519 2.73123 3.46354 2.40513 3.72494
3.29354 2.67741 2.69355 4.24690 2.90347 2.73739 3.18146 2.89801
2.37887 2.77519 2.98518 4.58477 3.61503
0.01666 4.49913 5.22148 0.61958 0.77255 0.76805 0.62347
221 2.19677 5.06463 3.10140 2.54123 4.35179 3.55518 3.72365
3.78476 1.70656 3.33548 4.13093 2.65146 3.94562 2.85231 2.54332
2.77215 2.99442 3.42923 5.51936 2.77940 276 k - - -
2.68618 4.42225 2.77519 2.73123 3.46354 2.40513 3.72494
3.29354 2.67741 2.69355 4.24690 2.90347 2.73739 3.18146 2.89801
2.37887 2.77519 2.98518 4.58477 3.61503
0.01666 4.49913 5.22148 0.61958 0.77255 0.76805 0.62347
222 2.99790 5.54999 1.97515 1.76597 4.87460 3.50042 3.85954
4.37267 1.40238 3.83930 4.62517 2.95635 4.01766 2.98807 3.18484
2.91701 3.24963 3.93525 5.96857 4.52226 277 k - - -
2.68618 4.42225 2.77519 2.73123 3.46354 2.40513 3.72494
3.29354 2.67741 2.69355 4.24690 2.90347 2.73739 3.18146 2.89801
2.37887 2.77519 2.98518 4.58477 3.61503
0.01666 4.49913 5.22148 0.61958 0.77255 0.76805 0.62347
223 3.75487 4.98857 5.55814 5.02341 3.36303 5.13037 5.48029
1.60184 4.86119 0.69299 3.12802 5.24660 5.22413 4.87357 4.89440
4.53882 3.97717 2.38861 5.56980 4.53751 278 L - - -
2.68618 4.42225 2.77519 2.73123 3.46354 2.40513 3.72494
3.29354 2.67741 2.69355 4.24690 2.90347 2.73739 3.18146 2.89801
2.37887 2.77519 2.98518 4.58477 3.61503
0.01666 4.49913 5.22148 0.61958 0.77255 0.76805 0.62347
224 2.72133 4.63478 2.82130 2.13425 3.80560 3.62460 3.85967
2.75042 2.76161 2.82785 2.07875 3.24967 4.01196 3.09110 3.17784
2.85505 2.95395 2.44815 5.20170 3.92786 279 m - - -
2.68611 4.42243 2.77454 2.73142 3.46372 2.40531 3.72513
3.29372 2.67640 2.69373 4.24708 2.90365 2.73737 3.18165 2.89819
2.37905 2.77538 2.98495 4.58495 3.61521
1.14721 0.95977 1.20564 0.68296 0.70344 0.53134 0.88629
225 2.97963 4.99498 3.48086 3.01155 4.63039 2.24919 3.98167
4.08164 2.44358 3.60801 4.47645 3.40172 4.11436 3.15635 0.99611
3.04793 3.28040 3.69646 5.65703 4.47426 282 r - - -
2.68618 4.42225 2.77519 2.73123 3.46354 2.40513 3.72494
3.29354 2.67741 2.69355 4.24690 2.90347 2.73739 3.18146 2.89801
2.37887 2.77519 2.98518 4.58477 3.61503
0.02081 4.27856 5.00090 0.61958 0.77255 0.99523 0.46146
226 2.34598 5.39284 1.49486 2.29198 4.78720 3.35379 3.86191
4.28336 2.81068 3.80216 4.62012 1.77869 3.94013 3.01583 3.36928
2.85988 3.22065 3.84771 5.96709 4.50245 283 d - - -
2.68618 4.42225 2.77519 2.73123 3.46354 2.40513 3.72494
3.29354 2.67741 2.69355 4.24690 2.90347 2.73739 3.18146 2.89801
2.37887 2.77519 2.98518 4.58477 3.61503

```
          0.02081  4.27856  5.00090  0.61958  0.77255  0.76169  0.62900
    227   3.04665  5.45113  2.09595  2.48624  4.81401  3.53251  3.87592
4.29016  1.04717  3.77945  4.60434  3.03236  4.05157  3.01844  2.99067
2.98635  3.29797  3.88892  5.87843  4.50684    284 k - - -
          2.68618  4.42225  2.77519  2.73123  3.46354  2.40513  3.72494
3.29354  2.67741  2.69355  4.24690  2.90347  2.73739  3.18146  2.89801
2.37887  2.77519  2.98518  4.58477  3.61503
          0.01859  4.39017  5.11252  0.61958  0.77255  0.89293  0.52671
    228   3.41100  4.65137  5.33433  4.81229  3.61478  4.87684  5.38436
1.08602  4.70746  1.49749  3.38452  4.98997  5.08042  4.85441  4.81923
4.26600  3.65446  1.52103  5.68236  4.55722    285 i - - -
          2.68618  4.42225  2.77519  2.73123  3.46354  2.40513  3.72494
3.29354  2.67741  2.69355  4.24690  2.90347  2.73739  3.18146  2.89801
2.37887  2.77519  2.98518  4.58477  3.61503
          0.01859  4.39017  5.11252  0.61958  0.77255  0.89293  0.52671
    229   3.04552  4.63326  3.71888  2.42622  3.78080  3.98668  4.39876
1.02461  3.36045  2.55450  3.69503  3.78524  4.42011  3.73310  3.65950
3.38494  3.30602  2.24966  5.42107  4.17438    286 i - - -
          2.68618  4.42225  2.77519  2.73123  3.46354  2.40513  3.72494
3.29354  2.67741  2.69355  4.24690  2.90347  2.73739  3.18146  2.89801
2.37887  2.77519  2.98518  4.58477  3.61503
          0.01859  4.39017  5.11252  0.61958  0.77255  0.89293  0.52671
    230   3.08990  5.42071  2.58888  1.05149  4.92554  1.78233  4.06064
4.43130  3.00167  3.96230  4.81353  3.05210  4.08645  3.23571  3.50967
3.04274  3.40732  3.99584  6.07529  4.68417    287 e - - -
          2.68618  4.42225  2.77519  2.73123  3.46354  2.40513  3.72494
3.29354  2.67741  2.69355  4.24690  2.90347  2.73739  3.18146  2.89801
2.37887  2.77519  2.98518  4.58477  3.61503
          0.01859  4.39017  5.11252  0.61958  0.77255  0.89293  0.52671
    231   1.68124  4.33351  3.89385  3.45064  4.03769  3.32920  4.35149
3.19603  3.40601  3.08061  3.98393  3.61576  3.99299  3.68801  3.70132
1.61809  2.92937  1.72107  5.48537  4.27797    288 s - - -
          2.68618  4.42225  2.77519  2.73123  3.46354  2.40513  3.72494
3.29354  2.67741  2.69355  4.24690  2.90347  2.73739  3.18146  2.89801
2.37887  2.77519  2.98518  4.58477  3.61503
          0.01859  4.39017  5.11252  0.61958  0.77255  0.89293  0.52671
    232   2.72823  4.69337  3.25818  2.28300  3.84431  3.59973  3.81505
2.56106  2.64771  2.11160  3.73735  3.19960  3.98788  2.06706  3.04733
2.83737  2.95804  2.93861  5.22878  3.95043    289 q - - -
          2.68618  4.42225  2.77519  2.73123  3.46354  2.40513  3.72494
3.29354  2.67741  2.69355  4.24690  2.90347  2.73739  3.18146  2.89801
2.37887  2.77519  2.98518  4.58477  3.61503
          0.01859  4.39017  5.11252  0.61958  0.77255  0.89293  0.52671
    233   2.23631  5.47029  1.29864  2.34412  4.87412  3.40742  3.92761
4.37486  2.88656  3.88856  4.70742  1.94592  4.00052  3.08236  3.45182
2.92435  3.29164  3.93287  6.05159  4.58057    290 d - - -
          2.68618  4.42225  2.77519  2.73123  3.46354  2.40513  3.72494
3.29354  2.67741  2.69355  4.24690  2.90347  2.73739  3.18146  2.89801
2.37887  2.77519  2.98518  4.58477  3.61503
          0.01859  4.39017  5.11252  0.61958  0.77255  0.89293  0.52671
    234   3.37046  4.60683  5.32780  4.85748  3.90427  4.88251  5.58012
1.18022  4.77334  2.35639  3.66410  5.02943  5.14681  5.02993  4.94666
4.30531  3.63612  0.92028  5.96697  4.75207    291 v - - -
```

```
          2.68606  4.42249  2.77543  2.73147  3.46378  2.40536  3.72518
3.29378  2.67696  2.69308  4.24713  2.90370  2.73763  3.17997  2.89781
2.37910  2.77543  2.98494  4.58501  3.61527
          0.26300  1.49060  5.11252  1.44205  0.26977  0.89293  0.52671
    235   2.81736  4.71977  3.20632  2.26604  3.88289  3.63715  3.89532
1.56955  2.72093  2.86435  3.79763  3.25490  4.05786  2.45092  3.08550
2.94441  3.05258  2.89157  5.27881  4.00686    297 i - - -
          2.68618  4.42225  2.77519  2.73123  3.46354  2.40513  3.72494
3.29354  2.67741  2.69355  4.24690  2.90347  2.73739  3.18146  2.89801
2.37887  2.77519  2.98518  4.58477  3.61503
          0.01859  4.39017  5.11252  0.61958  0.77255  0.29582  1.36227
    236   2.81405  5.23411  2.73655  1.97611  4.55431  3.57308  3.76191
4.01065  2.28470  3.52077  4.28696  3.05372  3.97372  2.87301  1.76991
2.78965  3.04332  2.43990  5.67435  4.29469    298 r - - -
          2.68618  4.42225  2.77519  2.73123  3.46354  2.40513  3.72494
3.29354  2.67741  2.69355  4.24690  2.90347  2.73739  3.18146  2.89801
2.37887  2.77519  2.98518  4.58477  3.61503
          0.01397  4.67381  5.39616  0.61958  0.77255  0.48576  0.95510
    237   2.84400  5.35342  2.12686  2.08841  4.70017  3.55882  3.77079
4.18302  2.00109  3.65194  4.39991  3.02366  3.97686  1.85604  2.59535
2.79828  3.07638  3.74627  5.77448  4.36682    299 q - - -
          2.68618  4.42225  2.77519  2.73123  3.46354  2.40513  3.72494
3.29354  2.67741  2.69355  4.24690  2.90347  2.73739  3.18146  2.89801
2.37887  2.77519  2.98518  4.58477  3.61503
          0.01397  4.67381  5.39616  0.61958  0.77255  0.48576  0.95510
    238   2.78409  5.18557  2.53948  2.49402  4.47877  3.55019  2.35513
3.94431  2.52720  3.47286  4.24268  2.14081  3.95609  2.87780  3.01403
2.49453  2.55286  3.55359  5.64264  2.86587    300 n - - -
          2.68618  4.42225  2.77519  2.73123  3.46354  2.40513  3.72494
3.29354  2.67741  2.69355  4.24690  2.90347  2.73739  3.18146  2.89801
2.37887  2.77519  2.98518  4.58477  3.61503
          0.01397  4.67381  5.39616  0.61958  0.77255  0.48576  0.95510
    239   2.41221  5.31860  2.96027  2.60170  4.65666  3.62006  3.16602
4.11848  2.35018  3.64504  4.44774  1.17423  4.08811  3.04146  3.05076
2.95444  3.22317  3.73502  5.80056  4.43268    301 n - - -
          2.68618  4.42225  2.77519  2.73123  3.46354  2.40513  3.72494
3.29354  2.67741  2.69355  4.24690  2.90347  2.73739  3.18146  2.89801
2.37887  2.77519  2.98518  4.58477  3.61503
          0.01397  4.67381  5.39616  0.61958  0.77255  0.48576  0.95510
    240   4.02915  5.22934  5.89716  5.31523  3.36273  5.42496  5.69042
2.14809  5.17573  0.55328  2.18389  5.56007  5.40083  5.02293  5.12391
4.82463  4.22515  2.80225  5.63844  4.71461    302 L - - -
          2.68618  4.42225  2.77519  2.73123  3.46354  2.40513  3.72494
3.29354  2.67741  2.69355  4.24690  2.90347  2.73739  3.18146  2.89801
2.37887  2.77519  2.98518  4.58477  3.61503
          0.01397  4.67381  5.39616  0.61958  0.77255  0.48576  0.95510
    241   2.07405  4.95983  2.63833  2.59676  4.20389  3.58353  3.79230
2.99038  2.59188  3.22333  4.03498  2.62341  3.97611  2.94160  2.71501
2.21695  2.98818  2.48321  5.46629  4.13013    303 a - - -
          2.68618  4.42225  2.77519  2.73123  3.46354  2.40513  3.72494
3.29354  2.67741  2.69355  4.24690  2.90347  2.73739  3.18146  2.89801
2.37887  2.77519  2.98518  4.58477  3.61503
          0.30223  4.67381  1.38033  0.61958  0.77255  0.48576  0.95510
```

```
    242   3.06145  5.60136  1.15749  2.31817  4.93861  2.39971  3.92814
4.46045  2.89210  3.94815  4.77236  2.89824  4.01800  2.27233  3.46281
2.96568  3.34605  4.02001  6.09851  4.61196    304 d - - -
          2.68618  4.42225  2.77519  2.73123  3.46354  2.40513  3.72494
3.29354  2.67741  2.69355  4.24690  2.90347  2.73739  3.18146  2.89801
2.37887  2.77519  2.98518  4.58477  3.61503
          0.01859  4.39017  5.11252  0.61958  0.77255  0.89293  0.52671
    243   1.68697  4.42427  4.19714  3.64528  3.39581  3.94729  4.34646
2.51022  3.52372  1.78095  1.95716  3.91863  4.33703  3.78795  3.75788
3.26173  3.14932  2.41697  5.08558  3.91024    305 a - - -
          2.68618  4.42225  2.77519  2.73123  3.46354  2.40513  3.72494
3.29354  2.67741  2.69355  4.24690  2.90347  2.73739  3.18146  2.89801
2.37887  2.77519  2.98518  4.58477  3.61503
          0.01859  4.39017  5.11252  0.61958  0.77255  0.89293  0.52671
    244   4.04317  5.21966  4.94036  4.65818  0.84888  4.71997  2.26489
3.83337  4.49333  3.13975  4.38691  4.33043  5.01257  4.41396  4.50079
4.08177  4.25225  3.72455  3.77481  1.56060    306 f - - -
          2.68618  4.42225  2.77519  2.73123  3.46354  2.40513  3.72494
3.29354  2.67741  2.69355  4.24690  2.90347  2.73739  3.18146  2.89801
2.37887  2.77519  2.98518  4.58477  3.61503
          0.01859  4.39017  5.11252  0.61958  0.77255  0.89293  0.52671
    245   2.88612  5.20809  2.89785  2.54070  4.65485  2.06374  3.81131
4.10284  1.45286  3.60694  4.41134  2.44126  3.99242  2.94563  2.84467
2.86844  3.14415  3.69829  5.73626  4.39350    307 k - - -
          2.68618  4.42225  2.77519  2.73123  3.46354  2.40513  3.72494
3.29354  2.67741  2.69355  4.24690  2.90347  2.73739  3.18146  2.89801
2.37887  2.77519  2.98518  4.58477  3.61503
          0.01859  4.39017  5.11252  0.61958  0.77255  0.89293  0.52671
    246   3.30660  5.97518  0.81385  1.77110  5.25268  3.43135  4.06330
4.82171  3.18977  4.29038  5.18570  2.88729  4.10493  3.24527  3.85637
3.14783  3.61705  4.36514  6.42243  4.85872    308 d - - -
          2.68618  4.42225  2.77519  2.73123  3.46354  2.40513  3.72494
3.29354  2.67741  2.69355  4.24690  2.90347  2.73739  3.18146  2.89801
2.37887  2.77519  2.98518  4.58477  3.61503
          0.01859  4.39017  5.11252  0.61958  0.77255  0.89293  0.52671
    247   2.69395  4.34033  3.68753  3.12051  2.78387  3.69636  3.92173
2.81945  2.46840  1.94812  3.44406  3.48188  4.07272  3.33954  3.35524
2.42411  2.92551  2.60787  4.82995  2.48983    309 l - - -
          2.68618  4.42225  2.77519  2.73123  3.46354  2.40513  3.72494
3.29354  2.67741  2.69355  4.24690  2.90347  2.73739  3.18146  2.89801
2.37887  2.77519  2.98518  4.58477  3.61503
          0.01859  4.39017  5.11252  0.61958  0.77255  0.89293  0.52671
    248   2.77063  4.41098  4.05751  3.63771  3.72529  3.69718  4.43607
1.20955  3.54234  2.64550  3.71033  3.84895  4.25349  3.85759  3.78768
1.83982  3.11681  2.36610  5.32032  4.07414    310 i - - -
          2.68618  4.42225  2.77519  2.73123  3.46354  2.40513  3.72494
3.29354  2.67741  2.69355  4.24690  2.90347  2.73739  3.18146  2.89801
2.37887  2.77519  2.98518  4.58477  3.61503
          0.01859  4.39017  5.11252  0.61958  0.77255  0.29582  1.36227
    249   2.79864  5.04300  3.10806  2.32795  4.34056  3.58122  3.82580
3.76357  2.34944  3.34788  4.15261  3.12307  1.58771  2.96783  3.05492
2.55710  2.75263  2.96421  5.56869  4.22461    311 p - - -
          2.68618  4.42225  2.77519  2.73123  3.46354  2.40513  3.72494
3.29354  2.67741  2.69355  4.24690  2.90347  2.73739  3.18146  2.89801
2.37887  2.77519  2.98518  4.58477  3.61503
```

```
          0.01397  4.67381  5.39616  0.61958  0.77255  0.48576  0.95510
    250   2.37539  4.99684  3.14611  2.59432  4.29347  3.56621  3.80038
3.27942  2.58265  3.30481  4.10507  2.25048  3.97315  2.94000  2.56010
2.07901  2.12938  3.37151  5.52825  4.18358    312 s - - -
          2.68618  4.42225  2.77519  2.73123  3.46354  2.40513  3.72494
3.29354  2.67741  2.69355  4.24690  2.90347  2.73739  3.18146  2.89801
2.37887  2.77519  2.98518  4.58477  3.61503
          0.01397  4.67381  5.39616  0.61958  0.77255  0.48576  0.95510
    251   2.81591  4.45128  3.82677  3.28248  3.53634  2.24200  2.45495
1.64454  3.17930  2.38994  3.56557  3.63226  4.20448  3.49786  3.47435
3.09005  3.05620  2.67971  5.02394  3.77442    313 i - - -
          2.68618  4.42225  2.77519  2.73123  3.46354  2.40513  3.72494
3.29354  2.67741  2.69355  4.24690  2.90347  2.73739  3.18146  2.89801
2.37887  2.77519  2.98518  4.58477  3.61503
          0.01397  4.67381  5.39616  0.61958  0.77255  0.48576  0.95510
    252   2.50034  5.31617  2.94823  1.77605  4.65775  3.55409  3.77729
4.13501  1.68597  3.62020  4.37405  2.64556  3.97523  2.88417  2.99780
2.23375  3.06951  3.70832  5.75634  4.35201    314 k - - -
          2.68618  4.42225  2.77519  2.73123  3.46354  2.40513  3.72494
3.29354  2.67741  2.69355  4.24690  2.90347  2.73739  3.18146  2.89801
2.37887  2.77519  2.98518  4.58477  3.61503
          0.01397  4.67381  5.39616  0.61958  0.77255  0.48576  0.95510
    253   3.23540  5.87812  1.56285  1.30616  5.17723  3.57755  4.06440
4.71261  3.03560  4.16888  4.98007  2.03297  4.16789  3.21092  3.63071
2.52741  3.51066  4.25317  6.30871  4.79178    315 e - - -
          2.68618  4.42225  2.77519  2.73123  3.46354  2.40513  3.72494
3.29354  2.67741  2.69355  4.24690  2.90347  2.73739  3.18146  2.89801
2.37887  2.77519  2.98518  4.58477  3.61503
          0.01397  4.67381  5.39616  0.61958  0.77255  0.48576  0.95510
    254   3.51928  4.85812  5.21105  4.66330  3.48410  4.75451  5.15800
2.09820  4.49028  0.65347  3.28501  4.85291  5.00586  4.62547  4.58765
4.11736  2.81919  2.46929  5.50376  4.42553    316 L - - -
          2.68618  4.42225  2.77519  2.73123  3.46354  2.40513  3.72494
3.29354  2.67741  2.69355  4.24690  2.90347  2.73739  3.18146  2.89801
2.37887  2.77519  2.98518  4.58477  3.61503
          0.01397  4.67381  5.39616  0.61958  0.77255  0.48576  0.95510
    255   2.78948  5.21107  1.89090  2.48863  4.52024  3.55002  3.75923
3.98232  2.51505  2.42153  4.26583  2.76391  3.95625  2.61463  2.63717
2.17921  3.02260  3.58344  5.66551  4.27550    317 d - - -
          2.68618  4.42225  2.77519  2.73123  3.46354  2.40513  3.72494
3.29354  2.67741  2.69355  4.24690  2.90347  2.73739  3.18146  2.89801
2.37887  2.77519  2.98518  4.58477  3.61503
          0.01397  4.67381  5.39616  0.61958  0.77255  0.48576  0.95510
    256   2.87375  5.39526  2.35242  1.62187  4.73283  3.55197  3.80056
4.22150  1.69960  3.69401  4.44588  3.01483  3.99087  2.53631  3.05765
2.82223  2.79160  3.78341  5.82293  4.40182    318 e - - -
          2.68636  4.42243  2.77411  2.73142  3.46372  2.40531  3.72513
3.29372  2.67759  2.69236  4.24708  2.90340  2.73758  3.18165  2.89819
2.37905  2.77538  2.98537  4.58495  3.61521
          0.39472  1.13445  5.39616  0.68296  0.70344  0.48576  0.95510
    257   2.86200  4.62978  3.65913  3.08786  3.01268  3.80475  4.02801
3.04619  1.59987  1.73141  3.66235  3.50662  4.18307  3.32147  3.26702
3.05893  2.64798  2.85245  5.17391  3.92390    321 k - - -
```

2.68618 4.42225 2.77519 2.73123 3.46354 2.40513 3.72494
3.29354 2.67741 2.69355 4.24690 2.90347 2.73739 3.18146 2.89801
2.37887 2.77519 2.98518 4.58477 3.61503
0.01397 4.67381 5.39616 0.61958 0.77255 0.48576 0.95510
258 1.69932 4.48221 4.78343 4.24094 3.76544 4.28572 4.80646
1.41126 4.12864 2.59652 3.67209 4.39552 4.67781 4.35786 4.29893
3.63042 2.71821 1.51998 5.43199 4.23223 322 i - - -
2.68618 4.42225 2.77519 2.73123 3.46354 2.40513 3.72494
3.29354 2.67741 2.69355 4.24690 2.90347 2.73739 3.18146 2.89801
2.37887 2.77519 2.98518 4.58477 3.61503
0.01397 4.67381 5.39616 0.61958 0.77255 0.48576 0.95510
259 2.80795 5.30802 2.30635 2.45970 4.64665 3.54173 3.75614
4.12931 1.58215 3.60957 4.35602 2.57830 3.95535 2.54047 2.99218
2.52357 2.51592 3.69637 5.74269 4.33203 323 k - - -
2.68618 4.42225 2.77519 2.73123 3.46354 2.40513 3.72494
3.29354 2.67741 2.69355 4.24690 2.90347 2.73739 3.18146 2.89801
2.37887 2.77519 2.98518 4.58477 3.61503
0.01397 4.67381 5.39616 0.61958 0.77255 0.48576 0.95510
260 2.93840 5.46909 2.31704 1.72853 4.79571 3.55459 3.85508
4.28946 2.66195 3.76693 4.52968 2.46421 1.83569 2.97105 2.77566
2.87798 3.18296 3.85281 5.90397 4.46833 324 e - - -
2.68618 4.42225 2.77519 2.73123 3.46354 2.40513 3.72494
3.29354 2.67741 2.69355 4.24690 2.90347 2.73739 3.18146 2.89801
2.37887 2.77519 2.98518 4.58477 3.61503
0.01397 4.67381 5.39616 0.61958 0.77255 0.48576 0.95510
261 3.03109 5.30328 3.24890 2.03846 4.61798 3.73793 3.86673
4.01002 1.23952 2.24785 4.36593 3.23846 4.13924 2.99791 2.75852
3.02288 3.24430 3.67160 5.70304 4.40117 325 k - - -
2.68618 4.42225 2.77519 2.73123 3.46354 2.40513 3.72494
3.29354 2.67741 2.69355 4.24690 2.90347 2.73739 3.18146 2.89801
2.37887 2.77519 2.98518 4.58477 3.61503
0.01397 4.67381 5.39616 0.61958 0.77255 0.48576 0.95510
262 2.90950 5.13452 3.30415 2.71151 4.42431 3.69458 3.81855
2.96197 1.29524 2.78650 4.20200 2.57108 4.07371 2.95518 2.47461
2.92502 3.12520 3.49389 5.57325 4.26974 326 k - - -
2.68618 4.42225 2.77519 2.73123 3.46354 2.40513 3.72494
3.29354 2.67741 2.69355 4.24690 2.90347 2.73739 3.18146 2.89801
2.37887 2.77519 2.98518 4.58477 3.61503
0.01397 4.67381 5.39616 0.61958 0.77255 0.48576 0.95510
263 2.85157 5.37055 2.52447 1.47049 4.70549 3.54372 3.78843
4.19432 2.20746 3.67047 4.42001 2.72261 3.97759 2.56474 3.05809
2.80259 2.33670 3.75692 5.80274 4.38080 327 e - - -
2.68618 4.42225 2.77519 2.73123 3.46354 2.40513 3.72494
3.29354 2.67741 2.69355 4.24690 2.90347 2.73739 3.18146 2.89801
2.37887 2.77519 2.98518 4.58477 3.61503
0.01397 4.67381 5.39616 0.61958 0.77255 0.48576 0.95510
264 2.48787 4.94446 3.19924 1.88991 4.18309 3.61503 3.82089
3.58264 2.60118 1.99156 4.02483 3.16072 4.00919 2.98006 2.40209
2.83371 2.54276 3.27222 5.45505 4.13528 328 e - - -
2.68618 4.42225 2.77519 2.73123 3.46354 2.40513 3.72494
3.29354 2.67741 2.69355 4.24690 2.90347 2.73739 3.18146 2.89801
2.37887 2.77519 2.98518 4.58477 3.61503
0.01397 4.67381 5.39616 0.61958 0.77255 0.48576 0.95510

```
    265   3.79112  5.00376  5.71255  5.20232  3.62101  5.25441  5.70699
0.86019  5.06209  1.22181  3.38249  5.39739  5.37621  5.11899  5.11358
4.67878  4.02642  2.22018  5.81752  4.74574    329 i - - -
          2.68618  4.42225  2.77519  2.73123  3.46354  2.40513  3.72494
3.29354  2.67741  2.69355  4.24690  2.90347  2.73739  3.18146  2.89801
2.37887  2.77519  2.98518  4.58477  3.61503
          0.01397  4.67381  5.39616  0.61958  0.77255  0.48576  0.95510
    266   2.84449  5.28460  2.69114  1.68678  4.60210  3.56719  3.78854
2.81800  1.59370  3.57187  4.33998  3.04363  3.98745  2.90192  3.00593
2.81257  3.07805  3.65599  5.72744  4.33455    330 k - - -
          2.68618  4.42225  2.77519  2.73123  3.46354  2.40513  3.72494
3.29354  2.67741  2.69355  4.24690  2.90347  2.73739  3.18146  2.89801
2.37887  2.77519  2.98518  4.58477  3.61503
          0.16399  4.67381  1.95253  0.61958  0.77255  0.48576  0.95510
    267   2.34656  5.18944  3.26180  2.73889  4.59930  3.66075  3.83373
4.00897  2.36406  3.52300  4.34708  3.21865  4.08581  1.27208  2.19051
2.96507  3.19098  3.64789  5.66146  4.37546    331 q - - -
          2.68536  4.42255  2.77549  2.73153  3.46384  2.40543  3.72524
3.29115  2.67625  2.69385  4.24720  2.90376  2.73769  3.18176  2.89830
2.37917  2.77511  2.98548  4.58507  3.61533
          0.51854  0.91790  5.24838  1.04606  0.43282  0.35769  1.20162
    268   2.03119  4.77424  3.51261  3.00609  3.93066  3.74313  4.05198
3.25458  2.88321  1.74973  3.84495  3.44717  4.17393  1.69190  3.23898
3.03161  3.14319  3.04508  5.37098  4.11105    335 q - - -
          2.68618  4.42225  2.77519  2.73123  3.46354  2.40513  3.72494
3.29354  2.67741  2.69355  4.24690  2.90347  2.73739  3.18146  2.89801
2.37887  2.77519  2.98518  4.58477  3.61503
          0.01397  4.67381  5.39616  0.61958  0.77255  0.48576  0.95510
    269   2.87637  5.39447  2.49147  1.87696  4.72896  2.28299  3.81024
4.21855  1.87505  3.69685  4.45080  3.01142  3.99231  2.91995  3.09703
2.14757  3.11657  3.78201  5.83187  4.40657    336 k - - -
          2.68618  4.42225  2.77519  2.73123  3.46354  2.40513  3.72494
3.29354  2.67741  2.69355  4.24690  2.90347  2.73739  3.18146  2.89801
2.37887  2.77519  2.98518  4.58477  3.61503
          0.01397  4.67381  5.39616  0.61958  0.77255  0.48576  0.95510
    270   2.77588  4.84126  3.26895  2.70624  4.04840  3.63487  3.83939
3.00875  1.93771  2.33538  3.92155  2.65294  4.02138  2.34621  3.08107
2.85037  3.00562  2.40295  5.36467  4.06370    337 k - - -
          2.68618  4.42225  2.77519  2.73123  3.46354  2.40513  3.72494
3.29354  2.67741  2.69355  4.24690  2.90347  2.73739  3.18146  2.89801
2.37887  2.77519  2.98518  4.58477  3.61503
          0.01397  4.67381  5.39616  0.61958  0.77255  0.48576  0.95510
    271   3.51362  6.24360  1.13982  1.00882  5.51250  3.61564  4.25315
5.10053  3.39623  4.54261  5.43701  3.06206  4.29469  3.43340  4.08484
3.34224  3.82492  4.62473  6.67222  5.08130    338 e - - -
          2.68618  4.42225  2.77519  2.73123  3.46354  2.40513  3.72494
3.29354  2.67741  2.69355  4.24690  2.90347  2.73739  3.18146  2.89801
2.37887  2.77519  2.98518  4.58477  3.61503
          0.01397  4.67381  5.39616  0.61958  0.77255  0.48576  0.95510
    272   3.29804  4.63689  5.02831  4.47133  3.18941  4.48763  4.60730
1.35408  4.29042  1.55203  3.38142  4.53907  4.77933  4.41027  4.35313
3.81763  3.52674  2.48762  4.95037  1.77443    339 i - - -
          2.68618  4.42225  2.77519  2.73123  3.46354  2.40513  3.72494
3.29354  2.67741  2.69355  4.24690  2.90347  2.73739  3.18146  2.89801
2.37887  2.77519  2.98518  4.58477  3.61503
```

0.01397 4.67381 5.39616 0.61958 0.77255 0.48576 0.95510
273 2.18189 5.41967 1.73070 1.83796 4.75625 3.55414 3.88422
4.23491 2.71264 3.74012 4.51555 3.03453 4.03786 3.00813 3.23099
2.89288 2.06493 3.81323 5.89749 4.47059 340 d - - -
2.68618 4.42225 2.77519 2.73123 3.46354 2.40513 3.72494
3.29354 2.67741 2.69355 4.24690 2.90347 2.73739 3.18146 2.89801
2.37887 2.77519 2.98518 4.58477 3.61503
0.01397 4.67381 5.39616 0.61958 0.77255 0.48576 0.95510
274 3.35971 5.50163 3.82078 3.10594 4.82446 4.00860 3.91700
4.28516 0.78448 3.71834 4.57903 3.51421 4.35579 3.05408 2.10575
3.34967 3.51897 3.96481 5.72232 3.09603 341 k - - -
2.68618 4.42225 2.77519 2.73123 3.46354 2.40513 3.72494
3.29354 2.67741 2.69355 4.24690 2.90347 2.73739 3.18146 2.89801
2.37887 2.77519 2.98518 4.58477 3.61503
0.01397 4.67381 5.39616 0.61958 0.77255 0.48576 0.95510
275 3.25295 5.03611 3.87248 3.33313 2.21487 4.08035 3.90505
3.59569 1.14377 3.13183 4.13234 3.69297 4.44919 3.51722 3.39675
3.35407 3.47485 3.38276 4.57802 1.99132 342 k - - -
2.68618 4.42225 2.77519 2.73123 3.46354 2.40513 3.72494
3.29354 2.67741 2.69355 4.24690 2.90347 2.73739 3.18146 2.89801
2.37887 2.77519 2.98518 4.58477 3.61503
0.01397 4.67381 5.39616 0.61958 0.77255 0.48576 0.95510
276 3.58493 4.78617 5.64473 5.16413 4.03993 5.18083 5.85208
1.37821 5.09538 2.04211 3.78188 5.32644 5.38554 5.31019 5.24004
4.60457 3.83966 0.78192 6.14623 4.95920 343 v - - -
2.68618 4.42225 2.77519 2.73123 3.46354 2.40513 3.72494
3.29354 2.67741 2.69355 4.24690 2.90347 2.73739 3.18146 2.89801
2.37887 2.77519 2.98518 4.58477 3.61503
0.01397 4.67381 5.39616 0.61958 0.77255 0.48576 0.95510
277 2.88244 4.99809 3.15749 2.28796 4.23521 2.01906 3.89021
3.62961 2.25296 1.74908 4.09340 3.20843 4.07549 3.06362 3.06255
2.93101 3.11709 3.32977 5.51101 4.20281 344 l - - -
2.68618 4.42225 2.77519 2.73123 3.46354 2.40513 3.72494
3.29354 2.67741 2.69355 4.24690 2.90347 2.73739 3.18146 2.89801
2.37887 2.77519 2.98518 4.58477 3.61503
0.01397 4.67381 5.39616 0.61958 0.77255 0.48576 0.95510
278 2.76167 4.40850 3.76424 2.89792 3.53174 2.17648 4.03822
2.88942 3.13577 1.91634 2.46611 3.56502 4.14946 3.42524 3.45211
2.70862 2.99419 2.67522 4.99856 2.91460 345 l - - -
2.68618 4.42225 2.77519 2.73123 3.46354 2.40513 3.72494
3.29354 2.67741 2.69355 4.24690 2.90347 2.73739 3.18146 2.89801
2.37887 2.77519 2.98518 4.58477 3.61503
0.01397 4.67381 5.39616 0.61958 0.77255 0.48576 0.95510
279 2.34254 5.23458 3.00731 2.50842 4.57931 3.55547 3.77884
4.04380 2.27504 3.55161 4.31456 2.37852 3.97209 2.22088 2.98934
1.69935 3.04793 3.63355 5.70478 4.31646 346 s - - -
2.68618 4.42225 2.77519 2.73123 3.46354 2.40513 3.72494
3.29354 2.67741 2.69355 4.24690 2.90347 2.73739 3.18146 2.89801
2.37887 2.77519 2.98518 4.58477 3.61503
0.01397 4.67381 5.39616 0.61958 0.77255 0.48576 0.95510
280 3.52124 4.81005 5.34057 4.81085 0.99022 4.79329 4.96253
1.80765 4.66455 2.05003 3.40864 4.88276 5.03976 4.73057 4.71090
4.15503 3.75742 2.04050 5.21544 3.86291 347 f - - -

```
          2.68618  4.42225  2.77519  2.73123  3.46354  2.40513  3.72494
3.29354  2.67741  2.69355  4.24690  2.90347  2.73739  3.18146  2.89801
2.37887  2.77519  2.98518  4.58477  3.61503
          0.01397  4.67381  5.39616  0.61958  0.77255  0.48576  0.95510
    281   2.14429  4.57116  3.81609  3.34072  4.23406  3.48624  4.34221
3.55981  3.30272  2.46741  4.16322  3.61712  4.11040  3.61571  3.65143
1.02130  2.54295  3.20418  5.62912  4.39681    348 s - - -
          2.68618  4.42225  2.77519  2.73123  3.46354  2.40513  3.72494
3.29354  2.67741  2.69355  4.24690  2.90347  2.73739  3.18146  2.89801
2.37887  2.77519  2.98518  4.58477  3.61503
          0.01397  4.67381  5.39616  0.61958  0.77255  0.48576  0.95510
    282   2.44019  4.90574  3.19045  2.25489  4.13574  2.14717  3.81471
3.54037  2.32229  2.29861  3.98735  3.15061  3.99371  2.97812  3.08002
2.81252  2.70574  2.94400  5.42442  4.10289    349 g - - -
          2.68618  4.42225  2.77519  2.73123  3.46354  2.40513  3.72494
3.29354  2.67741  2.69355  4.24690  2.90347  2.73739  3.18146  2.89801
2.37887  2.77519  2.98518  4.58477  3.61503
          0.01397  4.67381  5.39616  0.61958  0.77255  0.48576  0.95510
    283   2.08455  4.50620  4.07533  3.61654  4.10912  1.03041  4.50034
2.64571  3.56350  3.11743  4.05273  3.80491  4.19858  3.84867  3.85649
2.95881  3.11650  2.48705  5.58490  4.37581    350 g - - -
          2.68618  4.42225  2.77519  2.73123  3.46354  2.40513  3.72494
3.29354  2.67741  2.69355  4.24690  2.90347  2.73739  3.18146  2.89801
2.37887  2.77519  2.98518  4.58477  3.61503
          0.01397  4.67381  5.39616  0.61958  0.77255  0.48576  0.95510
    284   2.60032  4.41273  4.41821  3.84555  3.46168  4.06652  4.42048
2.25547  3.72742  1.28207  3.40261  4.06481  2.22241  3.95056  3.91587
3.36946  3.18993  2.17629  5.08380  3.90677    351 l - - -
          2.68629  4.42236  2.77531  2.73134  3.46365  2.40400  3.72505
3.29365  2.67752  2.69366  4.24701  2.90358  2.73751  3.18157  2.89812
2.37898  2.77531  2.98529  4.58488  3.61514
          0.72082  1.62532  1.14950  0.67761  0.70893  0.48576  0.95510
    285   2.99215  4.43482  4.41933  3.86633  3.31717  4.12892  4.48044
1.65804  3.72653  1.28224  3.20400  4.11276  2.98827  3.96296  3.91691
3.44868  3.23379  2.24413  5.09156  3.92760    354 l - - -
          2.68618  4.42225  2.77519  2.73123  3.46354  2.40513  3.72494
3.29354  2.67741  2.69355  4.24690  2.90347  2.73739  3.18146  2.89801
2.37887  2.77519  2.98518  4.58477  3.61503
          0.02037  4.29947  5.02181  0.61958  0.77255  0.97767  0.47190
    286   3.45974  4.72399  5.22294  4.70112  3.37922  4.82903  5.23686
1.12720  4.54452  1.20057  3.17099  4.91468  5.01316  4.67780  4.64589
4.22280  3.69701  2.03072  5.48930  4.37743    355 i - - -
          2.68618  4.42225  2.77519  2.73123  3.46354  2.40513  3.72494
3.29354  2.67741  2.69355  4.24690  2.90347  2.73739  3.18146  2.89801
2.37887  2.77519  2.98518  4.58477  3.61503
          0.02037  4.29947  5.02181  0.61958  0.77255  0.97767  0.47190
    287   2.10190  4.36834  3.61860  3.22344  2.68608  1.32300  4.18021
3.27587  3.23772  3.00659  3.92160  3.47860  3.95767  3.52470  3.56089
2.72703  2.92186  2.95490  5.29980  4.00496    356 g - - -
          2.68618  4.42225  2.77519  2.73123  3.46354  2.40513  3.72494
3.29354  2.67741  2.69355  4.24690  2.90347  2.73739  3.18146  2.89801
2.37887  2.77519  2.98518  4.58477  3.61503
          0.02037  4.29947  5.02181  0.61958  0.77255  0.97767  0.47190
```

```
    288   3.07478  4.43290  4.73715  4.18049  1.75278  4.20282  4.20021
2.58443  4.01215  1.56737  2.52461  4.22234  4.50284  4.10034  4.06769
3.52117  3.29851  2.57405  2.47335  3.08004    357 l - - -
          2.68618  4.42225  2.77519  2.73123  3.46354  2.40513  3.72494
3.29354  2.67741  2.69355  4.24690  2.90347  2.73739  3.18146  2.89801
2.37887  2.77519  2.98518  4.58477  3.61503
          0.02037  4.29947  5.02181  0.61958  0.77255  0.97767  0.47190
    289   1.52146  4.31215  3.81078  3.35105  3.86175  3.38786  4.25138
2.92362  3.30041  2.86196  3.80395  3.57341  2.77493  3.59442  3.60101
2.77358  2.91526  1.66508  5.34013  4.12668    358 a - - -
          2.68618  4.42225  2.77519  2.73123  3.46354  2.40513  3.72494
3.29354  2.67741  2.69355  4.24690  2.90347  2.73739  3.18146  2.89801
2.37887  2.77519  2.98518  4.58477  3.61503
          0.02037  4.29947  5.02181  0.61958  0.77255  0.26824  1.44701
    290   3.08653  4.63218  4.20775  3.63545  3.84407  4.17004  4.46525
1.53536  1.64112  2.65719  3.73270  3.98543  4.53045  3.80765  3.64342
3.46956  3.32570  1.67144  5.44146  4.22206    359 i - - -
          2.68618  4.42225  2.77519  2.73123  3.46354  2.40513  3.72494
3.29354  2.67741  2.69355  4.24690  2.90347  2.73739  3.18146  2.89801
2.37887  2.77519  2.98518  4.58477  3.61503
          0.01397  4.67381  5.39616  0.61958  0.77255  0.48576  0.95510
    291   2.85004  4.61489  3.82295  3.39443  3.56173  3.69272  4.19726
3.35531  3.33889  3.05113  3.97193  3.68541  4.23673  3.65769  3.64026
2.51098  1.90130  3.08523  5.08288  1.21093    360 y - - -
          2.68618  4.42225  2.77519  2.73123  3.46354  2.40513  3.72494
3.29354  2.67741  2.69355  4.24690  2.90347  2.73739  3.18146  2.89801
2.37887  2.77519  2.98518  4.58477  3.61503
          0.01397  4.67381  5.39616  0.61958  0.77255  0.48576  0.95510
    292   2.46334  4.66367  3.42824  2.11968  3.87252  2.07534  3.97059
2.05622  2.89246  2.91105  3.79468  3.35802  4.09314  3.21757  3.29743
2.93523  3.02986  2.60120  5.27469  4.00801    361 i - - -
          2.68618  4.42225  2.77519  2.73123  3.46354  2.40513  3.72494
3.29354  2.67741  2.69355  4.24690  2.90347  2.73739  3.18146  2.89801
2.37887  2.77519  2.98518  4.58477  3.61503
          0.01397  4.67381  5.39616  0.61958  0.77255  0.48576  0.95510
    293   2.85631  5.25222  2.19393  1.86733  4.54968  2.24445  3.82525
2.47134  2.62661  3.54298  4.32499  3.04668  3.99956  2.95066  3.12440
2.48338  3.09732  3.61869  5.72625  4.33280    362 e - - -
          2.68618  4.42225  2.77519  2.73123  3.46354  2.40513  3.72494
3.29354  2.67741  2.69355  4.24690  2.90347  2.73739  3.18146  2.89801
2.37887  2.77519  2.98518  4.58477  3.61503
          0.01397  4.67381  5.39616  0.61958  0.77255  0.48576  0.95510
    294   3.90610  5.11332  5.79722  5.26496  3.49365  5.33518  5.70683
1.36179  5.11648  0.72922  3.24505  5.48008  5.39625  5.08338  5.12189
4.75685  4.12716  2.45387  5.73295  4.72229    363 l - - -
          2.68618  4.42225  2.77519  2.73123  3.46354  2.40513  3.72494
3.29354  2.67741  2.69355  4.24690  2.90347  2.73739  3.18146  2.89801
2.37887  2.77519  2.98518  4.58477  3.61503
          0.39472  4.67381  1.14950  0.61958  0.77255  0.48576  0.95510
    295   2.98270  4.81608  3.23690  1.74039  1.49296  3.71167  3.92829
3.24161  3.04442  2.82209  3.87606  3.37917  4.17622  3.35347  3.41828
3.10381  3.23969  3.05691  4.87228  3.35694    364 f - - -
          2.68618  4.42225  2.77519  2.73123  3.46354  2.40513  3.72494
3.29354  2.67741  2.69355  4.24690  2.90347  2.73739  3.18146  2.89801
2.37887  2.77519  2.98518  4.58477  3.61503
```

0.02037 4.29947 5.02181 0.61958 0.77255 0.26824 1.44701
296 1.92041 4.74002 3.39536 2.88087 4.15520 1.94288 4.00884
3.54695 2.87820 2.55027 4.04439 2.61829 4.04312 3.21096 3.30167
2.83774 2.18794 3.21952 5.49043 4.20450 365 a - - -
2.68618 4.42225 2.77519 2.73123 3.46354 2.40513 3.72494
3.29354 2.67741 2.69355 4.24690 2.90347 2.73739 3.18146 2.89801
2.37887 2.77519 2.98518 4.58477 3.61503
0.01397 4.67381 5.39616 0.61958 0.77255 0.48576 0.95510
297 2.30237 5.34276 1.59795 2.18410 4.67970 3.54858 3.84361
4.15499 2.64789 3.66269 4.43226 3.03373 3.12292 2.96299 3.15573
2.84600 2.17749 3.73765 5.82123 4.40684 366 d - - -
2.68618 4.42225 2.77519 2.73123 3.46354 2.40513 3.72494
3.29354 2.67741 2.69355 4.24690 2.90347 2.73739 3.18146 2.89801
2.37887 2.77519 2.98518 4.58477 3.61503
0.01397 4.67381 5.39616 0.61958 0.77255 0.48576 0.95510
298 1.06191 5.03691 3.49211 3.07217 4.71763 3.65327 4.16473
4.06741 1.52573 3.68823 4.54297 3.47887 4.21100 3.33546 3.05323
3.05372 3.30617 3.67517 5.87212 4.61978 367 a - - -
2.68618 4.42225 2.77519 2.73123 3.46354 2.40513 3.72494
3.29354 2.67741 2.69355 4.24690 2.90347 2.73739 3.18146 2.89801
2.37887 2.77519 2.98518 4.58477 3.61503
0.01397 4.67381 5.39616 0.61958 0.77255 0.48576 0.95510
299 1.68633 4.70493 3.70146 3.14688 4.08310 3.68467 4.12031
3.28983 2.89272 3.08358 3.98950 3.52762 4.17068 3.36183 1.60823
3.00032 3.13024 2.03514 5.45676 4.21651 368 r - - -
2.68618 4.42225 2.77519 2.73123 3.46354 2.40513 3.72494
3.29354 2.67741 2.69355 4.24690 2.90347 2.73739 3.18146 2.89801
2.37887 2.77519 2.98518 4.58477 3.61503
0.01397 4.67381 5.39616 0.61958 0.77255 0.48576 0.95510
300 2.43451 5.16999 3.05711 1.77934 4.47619 3.55660 3.74526
3.32418 1.83115 3.45539 4.22322 3.04295 3.94998 2.46017 2.95719
2.75524 3.00162 3.00909 5.62508 4.24572 369 e - - -
2.68618 4.42225 2.77519 2.73123 3.46354 2.40513 3.72494
3.29354 2.67741 2.69355 4.24690 2.90347 2.73739 3.18146 2.89801
2.37887 2.77519 2.98518 4.58477 3.61503
0.01397 4.67381 5.39616 0.61958 0.77255 0.48576 0.95510
301 2.81517 5.27711 3.02158 2.30108 4.61482 3.56574 2.93307
4.08368 1.45102 3.57185 4.32688 2.68749 3.96790 2.85986 2.92545
2.38385 2.66070 3.66542 5.70774 4.31762 370 k - - -
2.68618 4.42225 2.77519 2.73123 3.46354 2.40513 3.72494
3.29354 2.67741 2.69355 4.24690 2.90347 2.73739 3.18146 2.89801
2.37887 2.77519 2.98518 4.58477 3.61503
0.01397 4.67381 5.39616 0.61958 0.77255 0.48576 0.95510
302 1.90199 4.55958 4.99292 4.42869 3.58986 4.47596 4.88573
1.64516 4.29327 1.28267 3.46016 4.56880 4.78836 4.46464 4.40646
3.81150 3.45700 1.93621 5.37377 4.21858 371 l - - -
2.68618 4.42225 2.77519 2.73123 3.46354 2.40513 3.72494
3.29354 2.67741 2.69355 4.24690 2.90347 2.73739 3.18146 2.89801
2.37887 2.77519 2.98518 4.58477 3.61503
0.01397 4.67381 5.39616 0.61958 0.77255 0.48576 0.95510
303 2.83411 5.31361 1.91223 2.49614 4.65758 3.56967 2.91292
4.13112 2.15962 3.60899 4.36186 3.03967 3.97682 2.86654 1.90591
2.48644 3.06378 3.70569 5.73608 4.34221 372 r - - -

```
          2.68618  4.42225  2.77519  2.73123  3.46354  2.40513  3.72494
3.29354  2.67741  2.69355  4.24690  2.90347  2.73739  3.18146  2.89801
2.37887  2.77519  2.98518  4.58477  3.61503
          0.01397  4.67381  5.39616  0.61958  0.77255  0.48576  0.95510
    304   2.99809  5.48077  2.94170  1.97865  4.84131  3.63071  3.85104
4.30784  1.45090  3.76432  4.53838  1.91733  4.07488  2.61351  2.93158
2.94287  3.22726  3.88447  5.87104  4.48911    373 k - - -
          2.68618  4.42225  2.77519  2.73123  3.46354  2.40513  3.72494
3.29354  2.67741  2.69355  4.24690  2.90347  2.73739  3.18146  2.89801
2.37887  2.77519  2.98518  4.58477  3.61503
          0.01397  4.67381  5.39616  0.61958  0.77255  0.48576  0.95510
    305   3.10825  5.48125  3.36413  2.13641  4.91006  3.79208  3.83710
4.31759  1.95375  3.73872  4.53387  3.26472  4.16961  1.55941  1.73450
3.06855  3.30319  3.92383  5.79542  4.51545    374 q - - -
          2.68618  4.42225  2.77519  2.73123  3.46354  2.40513  3.72494
3.29354  2.67741  2.69355  4.24690  2.90347  2.73739  3.18146  2.89801
2.37887  2.77519  2.98518  4.58477  3.61503
          0.01397  4.67381  5.39616  0.61958  0.77255  0.48576  0.95510
    306   2.91852  4.62040  3.82624  3.22035  3.78790  3.87766  4.07293
1.47077  1.95766  2.78207  3.71974  3.60381  4.24771  3.37279  2.66247
3.15104  3.14242  2.28652  5.21747  3.99887    375 i - - -
          2.68618  4.42225  2.77519  2.73123  3.46354  2.40513  3.72494
3.29354  2.67741  2.69355  4.24690  2.90347  2.73739  3.18146  2.89801
2.37887  2.77519  2.98518  4.58477  3.61503
          0.01397  4.67381  5.39616  0.61958  0.77255  0.48576  0.95510
    307   2.70908  5.90363  1.39079  1.99014  5.20174  3.58082  4.08807
4.73664  3.07892  4.19811  5.01888  1.41343  4.18304  3.23952  3.68169
3.14408  3.54443  4.28006  6.34313  4.82109    376 d - - -
          2.68618  4.42225  2.77519  2.73123  3.46354  2.40513  3.72494
3.29354  2.67741  2.69355  4.24690  2.90347  2.73739  3.18146  2.89801
2.37887  2.77519  2.98518  4.58477  3.61503
          0.01397  4.67381  5.39616  0.61958  0.77255  0.48576  0.95510
    308   1.87576  5.17500  3.01245  2.04918  4.50450  3.56068  3.80880
3.95165  2.57348  3.49312  4.27403  3.07250  3.98740  2.09651  3.04637
2.80584  2.11512  3.56602  5.67528  4.29952    377 a - - -
          2.68618  4.42225  2.77519  2.73123  3.46354  2.40513  3.72494
3.29354  2.67741  2.69355  4.24690  2.90347  2.73739  3.18146  2.89801
2.37887  2.77519  2.98518  4.58477  3.61503
          0.01397  4.67381  5.39616  0.61958  0.77255  0.48576  0.95510
    309   2.84223  4.59887  5.01847  4.46027  3.56966  4.49581  4.91721
1.50430  4.31009  1.03567  3.42766  4.60004  4.80888  4.48174  4.41784
3.83813  2.98203  2.19752  5.38831  4.24626    378 l - - -
          2.68618  4.42225  2.77519  2.73123  3.46354  2.40513  3.72494
3.29354  2.67741  2.69355  4.24690  2.90347  2.73739  3.18146  2.89801
2.37887  2.77519  2.98518  4.58477  3.61503
          0.01397  4.67381  5.39616  0.61958  0.77255  0.48576  0.95510
    310   2.77047  5.04343  3.11962  2.56717  4.30978  3.57896  3.77718
2.39610  2.05236  3.31402  4.11150  2.64703  3.97206  2.48484  3.00444
2.19773  2.44262  3.39165  5.52967  4.18031    379 k - - -
          2.68618  4.42225  2.77519  2.73123  3.46354  2.40513  3.72494
3.29354  2.67741  2.69355  4.24690  2.90347  2.73739  3.18146  2.89801
2.37887  2.77519  2.98518  4.58477  3.61503
          0.01397  4.67381  5.39616  0.61958  0.77255  0.48576  0.95510
```

311 2.18627 5.23750 2.74132 2.28582 4.56840 3.09557 3.74155
4.04197 1.75177 3.53998 4.29092 3.01438 3.94020 2.84635 2.97985
2.48252 2.31949 3.62240 5.68711 4.28619 380 k - - -
2.68618 4.42225 2.77519 2.73123 3.46354 2.40513 3.72494
3.29354 2.67741 2.69355 4.24690 2.90347 2.73739 3.18146 2.89801
2.37887 2.77519 2.98518 4.58477 3.61503
0.01397 4.67381 5.39616 0.61958 0.77255 0.48576 0.95510
312 2.93791 5.46681 2.18480 1.35473 4.80171 3.56579 3.84402
4.29243 2.61803 3.76204 4.52313 3.02623 4.02714 2.43383 2.24140
2.87832 3.17857 3.85476 5.88984 4.46357 381 e - - -
2.68618 4.42225 2.77519 2.73123 3.46354 2.40513 3.72494
3.29354 2.67741 2.69355 4.24690 2.90347 2.73739 3.18146 2.89801
2.37887 2.77519 2.98518 4.58477 3.61503
0.01397 4.67381 5.39616 0.61958 0.77255 0.48576 0.95510
313 2.77068 3.06570 4.45612 3.87846 3.38040 3.17408 4.29099
2.63705 3.72721 1.51250 3.39042 3.98503 4.28046 3.92089 3.84864
2.69900 3.02860 1.61733 4.90651 3.72203 382 l - - -
2.68618 4.42225 2.77519 2.73123 3.46354 2.40513 3.72494
3.29354 2.67741 2.69355 4.24690 2.90347 2.73739 3.18146 2.89801
2.37887 2.77519 2.98518 4.58477 3.61503
0.01397 4.67381 5.39616 0.61958 0.77255 0.48576 0.95510
314 2.50237 5.16713 1.67355 2.49662 4.47441 3.54788 3.74942
3.42708 2.32506 3.45852 4.22556 3.03665 3.94670 2.86291 2.69848
2.54838 2.45909 3.53838 5.63054 4.24699 383 d - - -
2.68618 4.42225 2.77519 2.73123 3.46354 2.40513 3.72494
3.29354 2.67741 2.69355 4.24690 2.90347 2.73739 3.18146 2.89801
2.37887 2.77519 2.98518 4.58477 3.61503
0.01397 4.67381 5.39616 0.61958 0.77255 0.48576 0.95510
315 2.49258 5.30514 2.28894 1.82664 4.64998 2.49665 3.79385
4.12997 2.56952 3.62469 4.38044 3.02036 3.97371 2.90395 3.07083
2.37557 2.28820 3.70293 5.77107 4.35887 384 e - - -
2.68618 4.42225 2.77519 2.73123 3.46354 2.40513 3.72494
3.29354 2.67741 2.69355 4.24690 2.90347 2.73739 3.18146 2.89801
2.37887 2.77519 2.98518 4.58477 3.61503
0.01397 4.67381 5.39616 0.61958 0.77255 0.48576 0.95510
316 1.92691 4.77040 4.69647 4.28278 3.66710 4.17292 4.93398
2.73585 4.12605 0.73668 3.53956 4.42436 4.68253 4.38089 4.28652
3.60043 3.55634 2.71245 5.53710 4.38571 385 l - - -
2.68618 4.42225 2.77519 2.73123 3.46354 2.40513 3.72494
3.29354 2.67741 2.69355 4.24690 2.90347 2.73739 3.18146 2.89801
2.37887 2.77519 2.98518 4.58477 3.61503
0.01397 4.67381 5.39616 0.61958 0.77255 0.48576 0.95510
317 2.96217 5.45504 2.91965 2.06993 4.80747 3.60785 3.83771
4.28042 1.98175 3.74287 4.51091 1.79366 4.05104 1.86812 2.96845
2.90829 3.19404 3.85349 5.85712 4.46364 386 n - - -
2.68618 4.42225 2.77519 2.73123 3.46354 2.40513 3.72494
3.29354 2.67741 2.69355 4.24690 2.90347 2.73739 3.18146 2.89801
2.37887 2.77519 2.98518 4.58477 3.61503
0.01397 4.67381 5.39616 0.61958 0.77255 0.48576 0.95510
318 2.76161 5.19992 2.23955 2.11050 4.51551 3.54060 2.59853
2.82402 2.48334 3.49234 4.24916 3.02003 3.93645 2.84235 2.57718
2.48404 2.65846 3.57475 5.65021 4.25726 387 e - - -
2.68618 4.42225 2.77519 2.73123 3.46354 2.40513 3.72494
3.29354 2.67741 2.69355 4.24690 2.90347 2.73739 3.18146 2.89801
2.37887 2.77519 2.98518 4.58477 3.61503

```
         0.01397  4.67381  5.39616  0.61958  0.77255  0.48576  0.95510
    319   2.81791  5.32291  2.47712  2.14868  4.65850  3.54044  3.76473
4.14296  2.29325  3.62347  4.37084  3.00742  3.96002  1.67891  3.01641
2.27138  2.67194  3.70939  5.75781  4.34321    388 q - - -
         2.68618  4.42225  2.77519  2.73123  3.46354  2.40513  3.72494
3.29354  2.67741  2.69355  4.24690  2.90347  2.73739  3.18146  2.89801
2.37887  2.77519  2.98518  4.58477  3.61503
         0.01397  4.67381  5.39616  0.61958  0.77255  0.48576  0.95510
    320   3.69776  4.92844  5.63712  5.09889  3.60598  5.13867  5.58110
1.98312  4.97895  0.81739  3.36331  5.27547  5.28403  5.02343  5.02824
4.53162  3.93013  1.44913  5.75048  4.69732    389 l - - -
         2.68618  4.42225  2.77519  2.73123  3.46354  2.40513  3.72494
3.29354  2.67741  2.69355  4.24690  2.90347  2.73739  3.18146  2.89801
2.37887  2.77519  2.98518  4.58477  3.61503
         0.01397  4.67381  5.39616  0.61958  0.77255  0.48576  0.95510
    321   2.87387  5.40093  2.08854  1.94459  4.73737  3.54786  3.80270
4.22849  1.71331  3.70070  4.45173  3.01007  3.98964  2.54753  3.07534
2.23956  3.11231  3.78847  5.83028  4.40512    390 k - - -
         2.68618  4.42225  2.77519  2.73123  3.46354  2.40513  3.72494
3.29354  2.67741  2.69355  4.24690  2.90347  2.73739  3.18146  2.89801
2.37887  2.77519  2.98518  4.58477  3.61503
         0.01397  4.67381  5.39616  0.61958  0.77255  0.48576  0.95510
    322   2.79256  5.28991  2.70062  2.24430  4.63082  2.07818  3.74130
4.11173  2.22307  3.59040  4.33469  3.01135  3.94695  2.47520  2.20593
2.48614  3.02427  3.67894  5.72105  4.31483    391 g - - -
         2.68618  4.42225  2.77519  2.73123  3.46354  2.40513  3.72494
3.29354  2.67741  2.69355  4.24690  2.90347  2.73739  3.18146  2.89801
2.37887  2.77519  2.98518  4.58477  3.61503
         0.01397  4.67381  5.39616  0.61958  0.77255  0.48576  0.95510
    323   2.76892  5.20611  2.24994  2.48002  4.52460  3.54260  3.74122
3.98999  1.89046  3.50091  4.25864  3.02331  3.94151  2.56565  2.97470
2.18324  2.65962  2.67144  5.65842  4.26571    392 k - - -
         2.68618  4.42225  2.77519  2.73123  3.46354  2.40513  3.72494
3.29354  2.67741  2.69355  4.24690  2.90347  2.73739  3.18146  2.89801
2.37887  2.77519  2.98518  4.58477  3.61503
         0.01397  4.67381  5.39616  0.61958  0.77255  0.48576  0.95510
    324   2.78682  5.22510  2.59697  1.63377  4.54152  3.09167  3.75601
4.00757  2.51299  3.51954  4.27981  3.02461  3.95191  2.86609  2.44498
2.55041  3.02065  2.45670  5.67870  4.28348    393 e - - -
         2.68618  4.42225  2.77519  2.73123  3.46354  2.40513  3.72494
3.29354  2.67741  2.69355  4.24690  2.90347  2.73739  3.18146  2.89801
2.37887  2.77519  2.98518  4.58477  3.61503
         0.01397  4.67381  5.39616  0.61958  0.77255  0.48576  0.95510
    325   2.02709  5.26766  3.14158  2.57938  4.62009  3.61924  3.77323
4.06799  1.64310  3.55920  4.32911  3.10477  4.01132  2.07497  2.60407
2.49912  3.08646  3.66723  5.69065  4.33488    394 k - - -
         2.68618  4.42225  2.77519  2.73123  3.46354  2.40513  3.72494
3.29354  2.67741  2.69355  4.24690  2.90347  2.73739  3.18146  2.89801
2.37887  2.77519  2.98518  4.58477  3.61503
         0.01397  4.67381  5.39616  0.61958  0.77255  0.48576  0.95510
    326   3.20312  4.66115  4.58601  3.97661  3.48195  4.30967  4.55997
1.83782  3.60692  1.11127  3.36419  4.23341  4.62156  4.00045  2.14381
3.62385  3.42796  2.58786  5.23739  4.08613    395 l - - -
```

```
          2.68618  4.42225  2.77519  2.73123  3.46354  2.40513  3.72494
3.29354  2.67741  2.69355  4.24690  2.90347  2.73739  3.18146  2.89801
2.37887  2.77519  2.98518  4.58477  3.61503
          0.01397  4.67381  5.39616  0.61958  0.77255  0.48576  0.95510
    327   3.09173  4.55479  4.44574  3.87340  3.52111  4.22886  4.53606
1.51591  3.74153  1.72934  3.41674  4.15188  4.55835  1.94027  3.96165
3.52492  3.32405  2.07099  5.22589  4.05486    396 i - - -
          2.68618  4.42225  2.77519  2.73123  3.46354  2.40513  3.72494
3.29354  2.67741  2.69355  4.24690  2.90347  2.73739  3.18146  2.89801
2.37887  2.77519  2.98518  4.58477  3.61503
          0.01397  4.67381  5.39616  0.61958  0.77255  0.48576  0.95510
    328   2.10752  5.09751  3.07196  2.34249  4.45657  2.39930  3.80407
3.90504  2.36847  3.45290  4.23120  3.08234  3.96773  2.92796  3.05244
1.91564  2.46141  3.51609  5.64065  4.27092    397 s - - -
          2.68618  4.42225  2.77519  2.73123  3.46354  2.40513  3.72494
3.29354  2.67741  2.69355  4.24690  2.90347  2.73739  3.18146  2.89801
2.37887  2.77519  2.98518  4.58477  3.61503
          0.01397  4.67381  5.39616  0.61958  0.77255  0.48576  0.95510
    329   1.82237  4.54891  3.79952  3.31939  4.15731  1.54907  4.30850
3.53275  3.29331  3.23301  2.20678  3.60264  4.10190  3.59450  3.64259
2.21856  3.05493  3.18414  5.55653  4.32698    398 g - - -
          2.68618  4.42225  2.77519  2.73123  3.46354  2.40513  3.72494
3.29354  2.67741  2.69355  4.24690  2.90347  2.73739  3.18146  2.89801
2.37887  2.77519  2.98518  4.58477  3.61503
          0.01397  4.67381  5.39616  0.61958  0.77255  0.48576  0.95510
    330   3.58456  4.83539  5.54446  4.98444  3.55086  4.97565  5.37882
1.19210  4.85211  1.48796  1.89376  5.11831  5.15470  4.89254  4.88073
4.34543  3.81288  1.90116  5.60656  4.54378    399 i - - -
          2.68618  4.42225  2.77519  2.73123  3.46354  2.40513  3.72494
3.29354  2.67741  2.69355  4.24690  2.90347  2.73739  3.18146  2.89801
2.37887  2.77519  2.98518  4.58477  3.61503
          0.19134  4.67381  1.80297  0.61958  0.77255  0.48576  0.95510
    331   2.27756  5.22784  2.85993  1.78415  4.55463  3.48549  3.72777
4.02594  2.49418  3.52984  4.29273  2.20613  3.91506  2.35380  2.98435
2.73823  2.60733  3.61232  5.68298  4.27996    400 e - - -
          2.68618  4.42225  2.77519  2.73123  3.46354  2.40513  3.72494
3.29354  2.67741  2.69355  4.24690  2.90347  2.73739  3.18146  2.89801
2.37887  2.77519  2.98518  4.58477  3.61503
          0.01666  4.49913  5.22148  0.61958  0.77255  0.34269  1.23738
    332   1.90426  5.36898  1.90534  2.52022  4.80721  3.53879  3.94595
4.29309  2.79376  3.80081  4.57921  1.77014  4.05656  3.07634  3.31290
2.22097  3.22094  3.85018  5.95791  4.53430    401 n - - -
          2.68647  4.42254  2.77526  2.73152  3.46383  2.40542  3.72524
3.29345  2.67770  2.69214  4.24719  2.90319  2.73769  3.18146  2.89830
2.37813  2.77549  2.98524  4.58506  3.61532
          0.30223  1.36141  5.39616  1.28839  0.32257  0.48576  0.95510
    333   2.78799  4.30447  4.29496  3.73894  1.91291  3.84090  4.28555
2.70227  3.62353  2.53032  3.47731  3.91421  4.27558  3.84731  3.80696
2.14063  2.60375  1.60260  4.97851  3.76668    406 v - - -
          2.68618  4.42225  2.77519  2.73123  3.46354  2.40513  3.72494
3.29354  2.67741  2.69355  4.24690  2.90347  2.73739  3.18146  2.89801
2.37887  2.77519  2.98518  4.58477  3.61503
          0.01397  4.67381  5.39616  0.61958  0.77255  0.48576  0.95510
```

```
    334   2.44426  4.43099  3.72558  3.16428  3.56136  3.23270  2.74432
1.63745  3.10715  2.64553  3.54468  3.54076  4.14093  3.40152  3.43393
2.41538  2.99625  2.70443  5.02212  3.20691    407 i - - -
          2.68618  4.42225  2.77519  2.73123  3.46354  2.40513  3.72494
3.29354  2.67741  2.69355  4.24690  2.90347  2.73739  3.18146  2.89801
2.37887  2.77519  2.98518  4.58477  3.61503
          0.01397  4.67381  5.39616  0.61958  0.77255  0.48576  0.95510
    335   2.93342  5.47097  1.67101  2.45318  4.80306  3.01704  3.84682
4.29806  2.01740  3.76916  4.52860  2.46820  4.02226  2.03732  3.14984
2.87239  3.17644  3.85736  5.90022  4.46576    408 d - - -
          2.68618  4.42225  2.77519  2.73123  3.46354  2.40513  3.72494
3.29354  2.67741  2.69355  4.24690  2.90347  2.73739  3.18146  2.89801
2.37887  2.77519  2.98518  4.58477  3.61503
          0.01397  4.67381  5.39616  0.61958  0.77255  0.48576  0.95510
    336   3.24683  4.67211  4.92691  4.49241  3.96919  4.39371  5.17403
0.78309  4.34723  2.59027  3.81774  4.62363  4.86134  4.64302  4.52396
3.81300  2.05689  2.05259  5.80199  4.59473    409 i - - -
          2.68618  4.42225  2.77519  2.73123  3.46354  2.40513  3.72494
3.29354  2.67741  2.69355  4.24690  2.90347  2.73739  3.18146  2.89801
2.37887  2.77519  2.98518  4.58477  3.61503
          0.01397  4.67381  5.39616  0.61958  0.77255  0.48576  0.95510
    337   3.33603  5.89294  0.79424  2.49393  5.20131  2.27773  3.09167
4.79170  3.22298  4.27163  5.12432  3.07989  4.23702  3.35078  3.83230
3.22214  3.63944  4.33930  6.39250  4.87046    410 d - - -
          2.68642  4.42249  2.77467  2.73147  3.46378  2.40536  3.72518
3.29249  2.67764  2.69308  4.24545  2.90283  2.73763  3.18170  2.89824
2.37910  2.77543  2.98542  4.58501  3.61439
          0.19134  1.77424  5.39616  1.44205  0.26977  0.48576  0.95510
    338   2.77179  5.14544  3.06526  2.04192  4.44219  3.56038  3.75178
2.59132  1.91554  3.42757  4.20152  3.05161  3.95426  2.50111  2.96878
2.39271  2.49339  3.50985  5.60671  4.23314    416 k - - -
          2.68618  4.42225  2.77519  2.73123  3.46354  2.40513  3.72494
3.29354  2.67741  2.69355  4.24690  2.90347  2.73739  3.18146  2.89801
2.37887  2.77519  2.98518  4.58477  3.61503
          0.01397  4.67381  5.39616  0.61958  0.77255  0.48576  0.95510
    339   2.85727  3.76607  3.12239  1.60999  4.59524  3.61380  3.77400
4.04316  2.43041  3.54255  4.31495  3.09829  4.00751  2.12721  1.98199
2.83439  3.08149  3.64755  5.68202  4.32330    417 e - - -
          2.68618  4.42225  2.77519  2.73123  3.46354  2.40513  3.72494
3.29354  2.67741  2.69355  4.24690  2.90347  2.73739  3.18146  2.89801
2.37887  2.77519  2.98518  4.58477  3.61503
          0.01397  4.67381  5.39616  0.61958  0.77255  0.48576  0.95510
    340   2.86235  4.97899  3.32844  2.74290  4.22389  2.40560  3.83955
3.61761  1.99398  2.82594  2.32037  3.23345  4.06609  3.00152  2.06699
2.91325  3.08372  3.31601  5.46283  4.17094    418 k - - -
          2.68618  4.42225  2.77519  2.73123  3.46354  2.40513  3.72494
3.29354  2.67741  2.69355  4.24690  2.90347  2.73739  3.18146  2.89801
2.37887  2.77519  2.98518  4.58477  3.61503
          0.01397  4.67381  5.39616  0.61958  0.77255  0.48576  0.95510
    341   2.47001  4.94861  3.16099  2.27699  4.18590  3.58842  3.79323
3.60092  2.59348  2.43247  4.02109  3.12205  3.97868  2.51621  2.81309
2.33272  2.18622  2.86980  5.45335  4.12054    419 t - - -
          2.68618  4.42225  2.77519  2.73123  3.46354  2.40513  3.72494
3.29354  2.67741  2.69355  4.24690  2.90347  2.73739  3.18146  2.89801
2.37887  2.77519  2.98518  4.58477  3.61503
```

0.01397 4.67381 5.39616 0.61958 0.77255 0.48576 0.95510
342 2.42929 5.15151 2.18299 2.20688 4.45090 3.00721 3.74350
2.96781 2.50124 3.43749 4.20578 3.03517 3.94185 2.46032 2.98546
2.74437 2.39690 3.18753 5.61363 4.23172 420 d - - -
2.68618 4.42225 2.77519 2.73123 3.46354 2.40513 3.72494
3.29354 2.67741 2.69355 4.24690 2.90347 2.73739 3.18146 2.89801
2.37887 2.77519 2.98518 4.58477 3.61503
0.01397 4.67381 5.39616 0.61958 0.77255 0.48576 0.95510
343 1.29477 4.52838 4.59750 4.07137 3.69722 4.12655 4.70507
2.42643 3.95472 1.69835 3.60098 4.25105 4.56942 4.19655 4.15096
3.48074 3.32883 1.72158 5.38764 4.20327 421 a - - -
2.68618 4.42225 2.77519 2.73123 3.46354 2.40513 3.72494
3.29354 2.67741 2.69355 4.24690 2.90347 2.73739 3.18146 2.89801
2.37887 2.77519 2.98518 4.58477 3.61503
0.01397 4.67381 5.39616 0.61958 0.77255 0.48576 0.95510
344 3.02833 4.54820 4.21154 1.92382 3.61482 4.13420 4.44059
2.09597 3.57758 1.53581 3.51770 3.98856 4.48116 3.85111 3.85621
3.41744 3.26309 1.80867 5.25922 4.06366 422 l - - -
2.68618 4.42225 2.77519 2.73123 3.46354 2.40513 3.72494
3.29354 2.67741 2.69355 4.24690 2.90347 2.73739 3.18146 2.89801
2.37887 2.77519 2.98518 4.58477 3.61503
0.01397 4.67381 5.39616 0.61958 0.77255 0.48576 0.95510
345 1.87759 4.44502 3.76891 3.20592 3.60371 3.76669 4.06891
2.89392 2.56901 2.13736 3.57508 3.57417 4.16268 3.43594 3.44953
2.65347 3.01788 1.76171 5.06794 3.84479 423 v - - -
2.68618 4.42225 2.77519 2.73123 3.46354 2.40513 3.72494
3.29354 2.67741 2.69355 4.24690 2.90347 2.73739 3.18146 2.89801
2.37887 2.77519 2.98518 4.58477 3.61503
0.01397 4.67381 5.39616 0.61958 0.77255 0.48576 0.95510
346 1.85117 5.02781 3.10013 1.89631 4.29893 3.58965 3.82930
3.69493 2.62044 3.30692 4.12183 3.12536 4.00449 2.33497 3.07963
2.82750 3.04175 2.33128 5.54708 4.20476 424 a - - -
2.68618 4.42225 2.77519 2.73123 3.46354 2.40513 3.72494
3.29354 2.67741 2.69355 4.24690 2.90347 2.73739 3.18146 2.89801
2.37887 2.77519 2.98518 4.58477 3.61503
0.01397 4.67381 5.39616 0.61958 0.77255 0.48576 0.95510
347 1.66056 4.45437 4.67576 4.09884 2.14915 4.18016 4.51721
2.56372 3.95803 1.41636 3.31880 4.24561 4.52752 4.12610 4.07959
3.49566 3.27722 2.29390 5.04643 3.85559 425 l - - -
2.68618 4.42225 2.77519 2.73123 3.46354 2.40513 3.72494
3.29354 2.67741 2.69355 4.24690 2.90347 2.73739 3.18146 2.89801
2.37887 2.77519 2.98518 4.58477 3.61503
0.01397 4.67381 5.39616 0.61958 0.77255 0.48576 0.95510
348 2.43374 5.21568 3.02378 2.02563 4.53903 3.54200 3.73335
4.00748 2.47317 3.51088 4.26448 2.00373 3.93748 2.45107 2.52415
2.73879 2.71692 3.04749 5.66175 4.26736 426 n - - -
2.68618 4.42225 2.77519 2.73123 3.46354 2.40513 3.72494
3.29354 2.67741 2.69355 4.24690 2.90347 2.73739 3.18146 2.89801
2.37887 2.77519 2.98518 4.58477 3.61503
0.01397 4.67381 5.39616 0.61958 0.77255 0.48576 0.95510
349 3.10359 5.27225 3.49145 2.88919 4.50705 3.83039 2.16543
3.02326 1.10392 3.50167 4.35096 3.35251 4.20762 3.03078 2.64122
3.11625 3.30375 3.65736 5.61180 4.32275 427 k - - -

```
         2.68618  4.42225  2.77519  2.73123  3.46354  2.40513  3.72494
3.29354  2.67741  2.69355  4.24690  2.90347  2.73739  3.18146  2.89801
2.37887  2.77519  2.98518  4.58477  3.61503
         0.01397  4.67381  5.39616  0.61958  0.77255  0.48576  0.95510
    350  4.01188  5.22005  5.82681  5.29445  3.42273  5.39363  5.71086
1.97975  5.12136  0.48429  3.17745  5.53501  5.42172  5.06023  5.11149
4.82645  4.22596  2.65561  5.68984  4.69396    428 L - - -
         2.68618  4.42225  2.77519  2.73123  3.46354  2.40513  3.72494
3.29354  2.67741  2.69355  4.24690  2.90347  2.73739  3.18146  2.89801
2.37887  2.77519  2.98518  4.58477  3.61503
         0.01397  4.67381  5.39616  0.61958  0.77255  0.48576  0.95510
    351  2.40771  5.41277  1.97933  1.82986  4.74493  3.54395  3.81442
4.23809  2.60388  3.71349  4.46642  2.59617  3.99395  2.43883  3.11605
2.14523  3.12400  3.79832  5.84728  4.41653    429 e - - -
         2.68618  4.42225  2.77519  2.73123  3.46354  2.40513  3.72494
3.29354  2.67741  2.69355  4.24690  2.90347  2.73739  3.18146  2.89801
2.37887  2.77519  2.98518  4.58477  3.61503
         0.01397  4.67381  5.39616  0.61958  0.77255  0.48576  0.95510
    352  2.76157  4.91458  3.19071  2.63254  2.71526  2.46834  3.08004
3.55034  2.61363  3.16668  3.98953  3.14512  3.99080  2.17063  2.62932
2.80903  2.63849  3.24034  5.41791  3.38653    430 q - - -
         2.68618  4.42225  2.77519  2.73123  3.46354  2.40513  3.72494
3.29354  2.67741  2.69355  4.24690  2.90347  2.73739  3.18146  2.89801
2.37887  2.77519  2.98518  4.58477  3.61503
         0.01397  4.67381  5.39616  0.61958  0.77255  0.48576  0.95510
    353  1.70704  4.43179  4.79641  4.21495  3.47570  4.27028  4.62876
1.73663  4.07024  2.00843  2.05356  4.34585  4.60026  4.24335  4.17891
3.58718  3.29418  2.02765  5.15718  3.99197    431 a - - -
         2.68618  4.42225  2.77519  2.73123  3.46354  2.40513  3.72494
3.29354  2.67741  2.69355  4.24690  2.90347  2.73739  3.18146  2.89801
2.37887  2.77519  2.98518  4.58477  3.61503
         0.01397  4.67381  5.39616  0.61958  0.77255  0.48576  0.95510
    354  4.92490  5.95244  5.45073  5.38466  3.90329  4.76916  5.19445
5.09935  5.17267  4.32566  5.67374  5.42615  5.35432  5.50479  5.13586
5.12690  5.27754  4.99751  0.14963  3.87663    432 W - - -
         2.68618  4.42225  2.77519  2.73123  3.46354  2.40513  3.72494
3.29354  2.67741  2.69355  4.24690  2.90347  2.73739  3.18146  2.89801
2.37887  2.77519  2.98518  4.58477  3.61503
         0.01397  4.67381  5.39616  0.61958  0.77255  0.48576  0.95510
    355  2.39695  5.14109  3.05503  2.51369  4.43890  3.55577  2.62683
3.89019  2.51173  2.96379  4.20300  2.37359  3.95377  2.35797  2.98796
1.97212  3.00263  3.50876  5.60927  4.23366    433 s - - -
         2.68618  4.42225  2.77519  2.73123  3.46354  2.40513  3.72494
3.29354  2.67741  2.69355  4.24690  2.90347  2.73739  3.18146  2.89801
2.37887  2.77519  2.98518  4.58477  3.61503
         0.01397  4.67381  5.39616  0.61958  0.77255  0.48576  0.95510
    356  2.77673  4.29652  4.10199  3.52431  3.41831  3.85421  4.16269
1.92635  3.42526  2.01030  3.41674  3.23178  4.23100  3.67088  3.65315
2.45885  2.07545  2.15614  4.92305  3.72349    434 i - - -
         2.68618  4.42225  2.77519  2.73123  3.46354  2.40513  3.72494
3.29354  2.67741  2.69355  4.24690  2.90347  2.73739  3.18146  2.89801
2.37887  2.77519  2.98518  4.58477  3.61503
         0.01397  4.67381  5.39616  0.61958  0.77255  0.48576  0.95510
```

357 3.37529 4.68039 5.28449 4.69982 1.54450 4.64800 4.97434
1.53159 4.53580 1.92312 2.15803 4.78377 4.89369 4.59480 4.55521
3.98807 3.60132 2.11159 5.29403 4.16946 435 i - - -
2.68618 4.42225 2.77519 2.73123 3.46354 2.40513 3.72494
3.29354 2.67741 2.69355 4.24690 2.90347 2.73739 3.18146 2.89801
2.37887 2.77519 2.98518 4.58477 3.61503
0.01397 4.67381 5.39616 0.61958 0.77255 0.48576 0.95510
358 2.09963 4.31994 4.02657 3.45837 3.46830 2.87803 4.14960
2.12215 3.37058 1.69842 3.46456 3.73358 4.19869 3.62572 3.62009
2.52457 2.73516 2.58147 4.96118 3.75860 436 l - - -
2.68618 4.42225 2.77519 2.73123 3.46354 2.40513 3.72494
3.29354 2.67741 2.69355 4.24690 2.90347 2.73739 3.18146 2.89801
2.37887 2.77519 2.98518 4.58477 3.61503
0.01397 4.67381 5.39616 0.61958 0.77255 0.48576 0.95510
359 2.39464 5.08414 3.08659 2.53463 4.36436 3.56286 3.02965
3.80362 2.25420 2.76329 4.14794 2.07631 3.95681 2.88736 2.99958
2.47699 2.33117 3.44057 5.56272 4.19951 437 n - - -
2.68618 4.42225 2.77519 2.73123 3.46354 2.40513 3.72494
3.29354 2.67741 2.69355 4.24690 2.90347 2.73739 3.18146 2.89801
2.37887 2.77519 2.98518 4.58477 3.61503
0.01397 4.67381 5.39616 0.61958 0.77255 0.48576 0.95510
360 3.01879 5.51209 2.87136 1.92405 4.86063 3.61887 3.87633
4.33365 2.25922 3.79634 4.57451 2.46578 4.08262 1.33256 3.01516
2.95847 3.25374 3.90983 5.91159 4.51527 438 q - - -
2.68618 4.42225 2.77519 2.73123 3.46354 2.40513 3.72494
3.29354 2.67741 2.69355 4.24690 2.90347 2.73739 3.18146 2.89801
2.37887 2.77519 2.98518 4.58477 3.61503
0.01397 4.67381 5.39616 0.61958 0.77255 0.48576 0.95510
361 3.76529 4.97947 5.68733 5.18597 3.66380 5.23592 5.71330
0.77073 5.04549 1.38717 3.42996 5.37813 5.37529 5.13310 5.11246
4.66377 4.00500 2.16179 5.84896 4.75461 439 i - - -
2.68618 4.42225 2.77519 2.73123 3.46354 2.40513 3.72494
3.29354 2.67741 2.69355 4.24690 2.90347 2.73739 3.18146 2.89801
2.37887 2.77519 2.98518 4.58477 3.61503
0.01397 4.67381 5.39616 0.61958 0.77255 0.48576 0.95510
362 2.31639 5.24360 3.00359 2.01237 4.57645 2.85903 3.73100
4.05249 2.47129 3.54386 4.29072 2.52405 3.93436 2.30427 2.63217
2.73507 2.33637 3.62834 5.68514 4.28287 440 e - - -
2.68618 4.42225 2.77519 2.73123 3.46354 2.40513 3.72494
3.29354 2.67741 2.69355 4.24690 2.90347 2.73739 3.18146 2.89801
2.37887 2.77519 2.98518 4.58477 3.61503
0.01397 4.67381 5.39616 0.61958 0.77255 0.48576 0.95510
363 1.98650 5.17948 2.98605 2.11450 4.56203 3.53939 3.83661
4.02081 2.62127 3.55404 4.32857 1.79178 3.98900 2.95942 3.10816
2.46058 2.49773 3.61458 5.72914 4.34410 441 n - - -
2.68618 4.42225 2.77519 2.73123 3.46354 2.40513 3.72494
3.29354 2.67741 2.69355 4.24690 2.90347 2.73739 3.18146 2.89801
2.37887 2.77519 2.98518 4.58477 3.61503
0.01397 4.67381 5.39616 0.61958 0.77255 0.48576 0.95510
364 2.76130 4.49536 3.95111 3.46611 3.83839 3.65089 4.31985
3.12074 3.38289 1.37475 3.80962 3.72930 4.19926 3.69130 3.67125
1.68769 2.21136 2.88057 5.32890 4.11175 442 l - - -
2.68618 4.42225 2.77519 2.73123 3.46354 2.40513 3.72494
3.29354 2.67741 2.69355 4.24690 2.90347 2.73739 3.18146 2.89801
2.37887 2.77519 2.98518 4.58477 3.61503

```
          0.01397  4.67381  5.39616  0.61958  0.77255  0.48576  0.95510
    365   2.49094  5.18096  3.09034  2.37580  4.49188  3.57515  3.75045
2.78299  2.15216  3.46380  4.23424  3.06199  3.96523  2.17980  2.04495
2.48794  3.01899  3.55378  5.62755  4.25739    443 r - - -
          2.68618  4.42225  2.77519  2.73123  3.46354  2.40513  3.72494
3.29354  2.67741  2.69355  4.24690  2.90347  2.73739  3.18146  2.89801
2.37887  2.77519  2.98518  4.58477  3.61503
          0.01397  4.67381  5.39616  0.61958  0.77255  0.48576  0.95510
    366   2.80678  5.27172  2.04001  2.02250  4.59215  2.28004  3.76658
3.28531  2.53109  3.56732  4.32412  3.01646  3.95913  2.54135  3.02683
2.77285  2.55524  3.64984  5.71908  4.31422    444 e - - -
          2.68618  4.42225  2.77519  2.73123  3.46354  2.40513  3.72494
3.29354  2.67741  2.69355  4.24690  2.90347  2.73739  3.18146  2.89801
2.37887  2.77519  2.98518  4.58477  3.61503
          0.30223  4.67381  1.38033  0.61958  0.77255  0.48576  0.95510
    367   2.65472  4.80630  2.52520  2.64969  4.45059  2.51915  3.95336
3.88933  2.82383  3.48705  4.29370  3.12007  3.91978  3.12062  3.28836
2.27593  1.42838  3.45860  5.70457  4.36858    445 t - - -
          2.68618  4.42225  2.77519  2.73123  3.46354  2.40513  3.72494
3.29354  2.67741  2.69355  4.24690  2.90347  2.73739  3.18146  2.89801
2.37887  2.77519  2.98518  4.58477  3.61503
          0.01859  4.39017  5.11252  0.61958  0.77255  0.29582  1.36227
    368   2.46865  5.24505  3.09408  2.57406  4.61562  3.59649  3.79887
4.06733  1.78380  3.56977  4.34161  3.10121  4.01001  1.94281  2.89974
1.87343  3.08965  3.66288  5.71185  4.34833    446 k - - -
          2.68618  4.42225  2.77519  2.73123  3.46354  2.40513  3.72494
3.29354  2.67741  2.69355  4.24690  2.90347  2.73739  3.18146  2.89801
2.37887  2.77519  2.98518  4.58477  3.61503
          0.01397  4.67381  5.39616  0.61958  0.77255  0.48576  0.95510
    369   3.31025  4.64050  5.15377  4.56540  1.74459  4.54090  4.84496
1.86301  4.39868  1.15356  3.22696  4.65842  4.80524  4.47446  4.43298
3.87192  2.89594  2.50215  5.19929  4.05807    447 l - - -
          2.68618  4.42225  2.77519  2.73123  3.46354  2.40513  3.72494
3.29354  2.67741  2.69355  4.24690  2.90347  2.73739  3.18146  2.89801
2.37887  2.77519  2.98518  4.58477  3.61503
          0.01397  4.67381  5.39616  0.61958  0.77255  0.48576  0.95510
    370   2.04825  5.29027  2.41046  2.23095  4.63151  3.53425  3.73618
4.11638  2.18182  3.59298  4.33377  2.24026  3.93873  2.44047  2.69000
2.74279  3.01522  3.67921  5.72289  4.31123    448 a - - -
          2.68618  4.42225  2.77519  2.73123  3.46354  2.40513  3.72494
3.29354  2.67741  2.69355  4.24690  2.90347  2.73739  3.18146  2.89801
2.37887  2.77519  2.98518  4.58477  3.61503
          0.01397  4.67381  5.39616  0.61958  0.77255  0.48576  0.95510
    371   2.85767  5.26544  3.08429  1.39236  4.59353  3.60576  3.77764
4.04470  2.45175  3.54476  3.36249  3.08695  4.00492  2.08077  2.52764
2.83255  3.08320  3.64965  5.69044  4.32483    449 e - - -
          2.68618  4.42225  2.77519  2.73123  3.46354  2.40513  3.72494
3.29354  2.67741  2.69355  4.24690  2.90347  2.73739  3.18146  2.89801
2.37887  2.77519  2.98518  4.58477  3.61503
          0.01397  4.67381  5.39616  0.61958  0.77255  0.48576  0.95510
    372   2.59575  4.46565  3.82403  3.25664  3.61158  3.83494  4.10131
1.48686  2.69285  2.64488  3.57478  3.62425  4.21256  2.52069  3.43859
3.10064  3.05785  2.09943  5.08840  3.86832    450 i - - -
```

```
          2.68618  4.42225  2.77519  2.73123  3.46354  2.40513  3.72494
3.29354  2.67741  2.69355  4.24690  2.90347  2.73739  3.18146  2.89801
2.37887  2.77519  2.98518  4.58477  3.61503
          0.01397  4.67381  5.39616  0.61958  0.77255  0.48576  0.95510
    373   2.96094  5.51533  2.08559  1.95956  4.84190  2.57119  3.87058
4.34351  2.69451  3.81365  4.57565  1.84316  4.03439  2.43616  3.22134
2.48775  3.20804  3.89882  5.94710  4.49974    451 n - - -
          2.68618  4.42225  2.77519  2.73123  3.46354  2.40513  3.72494
3.29354  2.67741  2.69355  4.24690  2.90347  2.73739  3.18146  2.89801
2.37887  2.77519  2.98518  4.58477  3.61503
          0.01397  4.67381  5.39616  0.61958  0.77255  0.48576  0.95510
    374   3.01750  5.44466  1.72223  2.09242  4.71601  3.57911  3.93751
4.18131  2.80762  1.71165  4.52478  2.33436  4.08360  3.07921  3.32998
2.96816  3.26944  3.79748  5.90807  4.48803    452 l - - -
          2.68618  4.42225  2.77519  2.73123  3.46354  2.40513  3.72494
3.29354  2.67741  2.69355  4.24690  2.90347  2.73739  3.18146  2.89801
2.37887  2.77519  2.98518  4.58477  3.61503
          0.01397  4.67381  5.39616  0.61958  0.77255  0.48576  0.95510
    375   1.57796  4.90598  3.22872  2.28295  4.45080  3.50811  3.99384
3.87569  2.81656  3.47742  4.28114  3.24079  4.02459  3.15118  3.26977
1.79917  2.21456  3.47707  5.70198  4.37412    453 a - - -
          2.68618  4.42225  2.77519  2.73123  3.46354  2.40513  3.72494
3.29354  2.67741  2.69355  4.24690  2.90347  2.73739  3.18146  2.89801
2.37887  2.77519  2.98518  4.58477  3.61503
          0.01397  4.67381  5.39616  0.61958  0.77255  0.48576  0.95510
    376   2.78762  5.23694  2.54566  2.48083  4.55838  3.54854  3.74841
4.02645  1.74224  2.33124  4.28687  2.55399  3.95111  2.53799  2.97102
2.47507  3.01942  3.61551  5.68112  4.28659    454 k - - -
          2.68618  4.42225  2.77519  2.73123  3.46354  2.40513  3.72494
3.29354  2.67741  2.69355  4.24690  2.90347  2.73739  3.18146  2.89801
2.37887  2.77519  2.98518  4.58477  3.61503
          0.01397  4.67381  5.39616  0.61958  0.77255  0.48576  0.95510
    377   2.76569  5.12909  2.27775  2.51767  4.42091  3.55987  3.75062
3.08219  2.04869  2.66679  4.18527  3.05315  2.69709  2.86899  2.39797
2.75788  2.99614  3.49036  5.59332  4.22176    455 k - - -
          2.68618  4.42225  2.77519  2.73123  3.46354  2.40513  3.72494
3.29354  2.67741  2.69355  4.24690  2.90347  2.73739  3.18146  2.89801
2.37887  2.77519  2.98518  4.58477  3.61503
          0.01397  4.67381  5.39616  0.61958  0.77255  0.48576  0.95510
    378   2.40103  4.45900  2.91798  3.56390  3.54941  3.99568  4.31863
1.85509  3.50430  1.32908  3.49403  3.87805  4.36777  3.76281  3.77013
3.28600  3.16276  2.42545  5.12973  3.92899    456 l - - -
          2.68618  4.42225  2.77519  2.73123  3.46354  2.40513  3.72494
3.29354  2.67741  2.69355  4.24690  2.90347  2.73739  3.18146  2.89801
2.37887  2.77519  2.98518  4.58477  3.61503
          0.01397  4.67381  5.39616  0.61958  0.77255  0.48576  0.95510
    379   2.78664  5.05024  2.24280  2.56288  4.30666  3.57619  2.71613
2.58960  2.59416  3.32031  4.12316  2.70598  3.98266  2.93969  3.06698
2.80106  1.96073  3.39346  5.54347  4.19001    457 t - - -
          2.68618  4.42225  2.77519  2.73123  3.46354  2.40513  3.72494
3.29354  2.67741  2.69355  4.24690  2.90347  2.73739  3.18146  2.89801
2.37887  2.77519  2.98518  4.58477  3.61503
          0.01397  4.67381  5.39616  0.61958  0.77255  0.48576  0.95510
```

```
    380   2.40113  5.31486  2.62993  2.15389  4.64921  3.54534  3.78156
4.12841  1.83023  3.61874  4.37318  3.01839  3.97179  2.88980  3.03723
1.71064  3.06839  3.70269  5.76093  4.35067    458 s - - -
          2.68618  4.42225  2.77519  2.73123  3.46354  2.40513  3.72494
3.29354  2.67741  2.69355  4.24690  2.90347  2.73739  3.18146  2.89801
2.37887  2.77519  2.98518  4.58477  3.61503
          0.01397  4.67381  5.39616  0.61958  0.77255  0.48576  0.95510
    381   3.08349  4.59152  4.31568  3.74766  1.81914  4.15647  4.35441
2.25889  3.63088  1.86836  3.37379  4.03455  4.48849  1.71764  3.86266
3.44163  3.31102  2.69320  4.99730  3.70840    459 q - - -
          2.68618  4.42225  2.77519  2.73123  3.46354  2.40513  3.72494
3.29354  2.67741  2.69355  4.24690  2.90347  2.73739  3.18146  2.89801
2.37887  2.77519  2.98518  4.58477  3.61503
          0.01397  4.67381  5.39616  0.61958  0.77255  0.48576  0.95510
    382   2.82564  4.29863  4.27700  3.69086  3.35111  3.93249  4.23151
2.40551  3.56966  1.76412  2.90153  3.90719  4.29307  2.15621  3.75258
3.21954  3.05685  1.91017  4.90325  2.83006    460 l - - -
          2.68618  4.42225  2.77519  2.73123  3.46354  2.40513  3.72494
3.29354  2.67741  2.69355  4.24690  2.90347  2.73739  3.18146  2.89801
2.37887  2.77519  2.98518  4.58477  3.61503
          0.01397  4.67381  5.39616  0.61958  0.77255  0.48576  0.95510
    383   2.12094  4.89688  2.57818  2.65165  4.14852  3.19138  3.83427
2.16034  2.65820  3.17851  4.00326  2.77072  3.99718  3.00002  3.11455
2.46799  2.49118  3.24261  5.44114  4.11974    461 a - - -
          2.68618  4.42225  2.77519  2.73123  3.46354  2.40513  3.72494
3.29354  2.67741  2.69355  4.24690  2.90347  2.73739  3.18146  2.89801
2.37887  2.77519  2.98518  4.58477  3.61503
          0.01397  4.67381  5.39616  0.61958  0.77255  0.48576  0.95510
    384   2.55436  4.85990  3.22293  1.90287  2.74812  3.61604  3.82776
3.47126  2.23367  3.10504  3.93993  3.17666  4.00633  3.00543  3.10262
2.83029  2.71084  3.17639  5.37534  2.47537    462 e - - -
          2.68618  4.42225  2.77519  2.73123  3.46354  2.40513  3.72494
3.29354  2.67741  2.69355  4.24690  2.90347  2.73739  3.18146  2.89801
2.37887  2.77519  2.98518  4.58477  3.61503
          0.01397  4.67381  5.39616  0.61958  0.77255  0.48576  0.95510
    385   4.05488  5.26417  5.71678  5.23798  1.52697  5.25687  5.04417
2.74283  5.07766  0.68853  3.24104  5.28492  5.34313  4.94293  5.02338
4.67116  4.26102  3.01195  5.08923  3.66578    463 L - - -
          2.68618  4.42225  2.77519  2.73123  3.46354  2.40513  3.72494
3.29354  2.67741  2.69355  4.24690  2.90347  2.73739  3.18146  2.89801
2.37887  2.77519  2.98518  4.58477  3.61503
          0.01397  4.67381  5.39616  0.61958  0.77255  0.48576  0.95510
    386   2.50673  5.38342  2.05603  1.74583  4.71886  2.78559  3.79300
4.21033  2.19314  3.68437  4.43321  3.00460  3.97951  2.34113  3.07333
2.80610  3.09539  3.77014  5.81563  4.38983    464 e - - -
          2.68618  4.42225  2.77519  2.73123  3.46354  2.40513  3.72494
3.29354  2.67741  2.69355  4.24690  2.90347  2.73739  3.18146  2.89801
2.37887  2.77519  2.98518  4.58477  3.61503
          0.01397  4.67381  5.39616  0.61958  0.77255  0.48576  0.95510
    387   2.83820  5.31744  1.92051  2.46518  4.64214  3.54554  3.78872
4.12141  2.56098  2.98691  4.37451  2.40729  3.97589  2.04463  3.05959
2.15947  3.07595  3.69996  5.76357  4.35194    465 d - - -
          2.68618  4.42225  2.77519  2.73123  3.46354  2.40513  3.72494
3.29354  2.67741  2.69355  4.24690  2.90347  2.73739  3.18146  2.89801
2.37887  2.77519  2.98518  4.58477  3.61503
```

```
          0.01397  4.67381  5.39616  0.61958  0.77255  0.48576  0.95510
    388   2.02471  4.55727  4.87157  4.33456  3.65954  4.34670  4.86237
2.29166  4.20101  1.30704  3.53948  4.47778  4.72597  4.40786  4.34486
3.69988  3.42624  1.37345  5.41853  4.24906    466 l - - -
          2.68618  4.42225  2.77519  2.73123  3.46354  2.40513  3.72494
3.29354  2.67741  2.69355  4.24690  2.90347  2.73739  3.18146  2.89801
2.37887  2.77519  2.98518  4.58477  3.61503
          0.01397  4.67381  5.39616  0.61958  0.77255  0.48576  0.95510
    389   1.85249  4.50426  3.70020  2.78642  3.72849  3.73372  4.13604
1.47836  3.18161  2.77688  3.69076  3.57819  4.17906  3.48308  3.51468
2.47252  3.06248  2.72316  5.19136  3.96262    467 i - - -
          2.68618  4.42225  2.77519  2.73123  3.46354  2.40513  3.72494
3.29354  2.67741  2.69355  4.24690  2.90347  2.73739  3.18146  2.89801
2.37887  2.77519  2.98518  4.58477  3.61503
          0.01397  4.67381  5.39616  0.61958  0.77255  0.48576  0.95510
    390   2.77193  5.19480  2.31506  2.48736  4.51484  2.85019  3.75098
3.97792  2.03387  2.88596  4.25624  3.02956  3.94626  2.86151  2.99146
1.90465  2.67723  3.57473  5.65774  4.26728    468 s - - -
          2.68618  4.42225  2.77519  2.73123  3.46354  2.40513  3.72494
3.29354  2.67741  2.69355  4.24690  2.90347  2.73739  3.18146  2.89801
2.37887  2.77519  2.98518  4.58477  3.61503
          0.01397  4.67381  5.39616  0.61958  0.77255  0.48576  0.95510
    391   2.30487  5.05654  3.16975  2.79523  4.63517  3.56030  4.04780
4.07188  2.79892  3.64099  4.45898  3.27228  1.88893  1.36696  3.20446
2.91947  3.19103  3.65925  5.83477  4.50583    469 q - - -
          2.68618  4.42225  2.77519  2.73123  3.46354  2.40513  3.72494
3.29354  2.67741  2.69355  4.24690  2.90347  2.73739  3.18146  2.89801
2.37887  2.77519  2.98518  4.58477  3.61503
          0.01397  4.67381  5.39616  0.61958  0.77255  0.48576  0.95510
    392   4.48706  5.51214  5.49863  5.34982  1.68240  5.10688  3.86699
4.11119  5.13201  2.76036  4.65708  4.70256  5.36998  4.84799  4.96971
4.49366  4.68637  4.03895  1.05175  1.37812    470 w - - -
          2.68618  4.42225  2.77519  2.73123  3.46354  2.40513  3.72494
3.29354  2.67741  2.69355  4.24690  2.90347  2.73739  3.18146  2.89801
2.37887  2.77519  2.98518  4.58477  3.61503
          0.01397  4.67381  5.39616  0.61958  0.77255  0.48576  0.95510
    393   2.75928  5.10767  3.07927  2.52122  4.39312  3.55921  3.03198
3.39065  1.85810  2.78864  4.16502  2.70188  3.95048  2.58591  2.64020
2.52967  2.76572  2.91420  5.57662  4.20789    471 k - - -
          2.68618  4.42225  2.77519  2.73123  3.46354  2.40513  3.72494
3.29354  2.67741  2.69355  4.24690  2.90347  2.73739  3.18146  2.89801
2.37887  2.77519  2.98518  4.58477  3.61503
          0.04999  4.67381  3.23336  0.61958  0.77255  0.48576  0.95510
    394   2.74955  5.19177  2.43966  1.93914  4.50868  3.08654  3.72285
3.97565  2.47252  3.48522  3.36443  3.00588  3.92365  2.35361  2.96102
2.21212  2.98140  2.87941  5.64204  4.24760    472 e - - -
          2.68618  4.42225  2.77519  2.73123  3.46354  2.40513  3.72494
3.29354  2.67741  2.69355  4.24690  2.90347  2.73739  3.18146  2.89801
2.37887  2.77519  2.98518  4.58477  3.61503
          0.05343  4.63830  3.16166  0.61958  0.77255  0.55436  0.85435
    395   3.44657  4.69955  5.41962  4.87548  3.64596  4.86566  5.32385
1.59600  4.75377  1.61281  2.57177  5.00184  5.08684  4.86622  4.82158
4.23672  3.68447  1.08309  5.63419  4.51787    473 v - - -
```

```
          2.68618  4.42225  2.77519  2.73123  3.46354  2.40513  3.72494
3.29354  2.67741  2.69355  4.24690  2.90347  2.73739  3.18146  2.89801
2.37887  2.77519  2.98518  4.58477  3.61503
          0.09009  4.59992  2.57569  0.61958  0.77255  0.62113  0.77075
    396   2.06887  5.06339  3.03384  2.29227  4.34810  2.99333  3.71784
3.78697  2.04276  2.52108  4.12530  3.02322  3.91898  2.39513  2.92709
2.73178  2.96388  3.42187  5.53301  4.17139    474 k - - -
          2.68618  4.42225  2.77519  2.73123  3.46354  2.40513  3.72494
3.29354  2.67741  2.69355  4.24690  2.90347  2.73739  3.18146  2.89801
2.37887  2.77519  2.98518  4.58477  3.61503
          0.08633  4.52603  2.63268  0.61958  0.77255  0.73228  0.65549
    397   2.75458  5.20709  2.50644  2.07371  4.51839  2.42904  3.70986
3.98880  2.48581  2.52154  4.26250  2.55143  3.89735  2.37189  2.98228
2.72036  2.99124  3.58060  5.65677  4.25292    475 e - - -
          2.68618  4.42225  2.77519  2.73123  3.46354  2.40513  3.72494
3.29354  2.67741  2.69355  4.24690  2.90347  2.73739  3.18146  2.89801
2.37887  2.77519  2.98518  4.58477  3.61503
          0.01738  4.45708  5.17943  0.61958  0.77255  0.81975  0.58079
    398   2.78225  5.16008  2.87374  1.82442  4.50488  3.48352  3.75490
3.95781  2.27791  3.48742  4.26980  2.99222  3.92951  2.87965  2.98333
1.68959  2.57276  3.56338  5.65850  4.27727    476 s - - -
          2.68618  4.42225  2.77519  2.73123  3.46354  2.40513  3.72494
3.29354  2.67741  2.69355  4.24690  2.90347  2.73739  3.18146  2.89801
2.37887  2.77519  2.98518  4.58477  3.61503
          0.17799  4.45708  1.88750  0.61958  0.77255  0.81975  0.58079
    399   0.54015  4.46712  3.95872  3.79615  4.53970  3.27587  4.75805
3.73478  3.82541  3.59419  4.59413  3.81780  4.05711  4.12857  4.02939
2.82190  3.13004  3.30985  5.91999  4.77853    477 A - - -
          2.68618  4.42225  2.77519  2.73123  3.46354  2.40513  3.72494
3.29354  2.67741  2.69355  4.24690  2.90347  2.73739  3.18146  2.89801
2.37887  2.77519  2.98518  4.58477  3.61503
          0.02037  4.29947  5.02181  0.61958  0.77255  0.97767  0.47190
    400   2.47108  5.08711  2.99413  2.44372  4.42031  2.85591  3.64781
3.86416  2.12332  3.37786  4.15459  2.97005  3.87268  2.31051  2.08544
2.43378  2.93987  3.47647  5.53065  4.17458    478 r - - -
          2.68618  4.42225  2.77519  2.73123  3.46354  2.40513  3.72494
3.29354  2.67741  2.69355  4.24690  2.90347  2.73739  3.18146  2.89801
2.37887  2.77519  2.98518  4.58477  3.61503
          0.02037  4.29947  5.02181  0.61958  0.77255  0.97767  0.47190
    401   2.84039  5.35938  2.08991  1.99980  4.67495  3.39941  3.74361
4.16686  2.58200  3.65497  4.43435  2.86269  3.89876  1.79620  3.10358
2.21257  3.09129  3.73964  5.80266  4.36195    479 q - - -
          2.68618  4.42225  2.77519  2.73123  3.46354  2.40513  3.72494
3.29354  2.67741  2.69355  4.24690  2.90347  2.73739  3.18146  2.89801
2.37887  2.77519  2.98518  4.58477  3.61503
          0.02037  4.29947  5.02181  0.61958  0.77255  0.97767  0.47190
    402   3.59948  4.88337  5.13605  4.64542  1.63628  4.74781  4.62216
2.50317  4.48942  0.89525  3.07395  4.73924  4.93221  4.49490  4.52765
4.12944  3.81917  2.68087  4.77876  3.33183    480 l - - -
          2.68618  4.42225  2.77519  2.73123  3.46354  2.40513  3.72494
3.29354  2.67741  2.69355  4.24690  2.90347  2.73739  3.18146  2.89801
2.37887  2.77519  2.98518  4.58477  3.61503
          0.02037  4.29947  5.02181  0.61958  0.77255  0.97767  0.47190
```

```
    403   2.83392  4.40135  4.39112  3.97807  3.85980  3.82852  4.75666
2.18910  3.86193  2.58738  3.73721  4.10812  4.40107  4.17274  4.08057
3.25244  2.26476  0.93070  5.57809  4.36183    481 v - - -
          2.68618  4.42225  2.77519  2.73123  3.46354  2.40513  3.72494
3.29354  2.67741  2.69355  4.24690  2.90347  2.73739  3.18146  2.89801
2.37887  2.77519  2.98518  4.58477  3.61503
          0.02037  4.29947  5.02181  0.61958  0.77255  0.97767  0.47190
    404   2.69118  4.86828  2.61087  2.51131  4.11303  3.48682  3.70978
3.50107  1.94046  2.14782  3.95150  3.02654  3.89484  2.86647  2.92428
2.72741  2.51855  3.18836  5.37754  4.05031    482 k - - -
          2.68618  4.42225  2.77519  2.73123  3.46354  2.40513  3.72494
3.29354  2.67741  2.69355  4.24690  2.90347  2.73739  3.18146  2.89801
2.37887  2.77519  2.98518  4.58477  3.61503
          0.02037  4.29947  5.02181  0.61958  0.77255  0.97767  0.47190
    405   2.67837  4.41992  3.54786  3.01069  3.63858  3.63186  3.97814
2.07836  2.96143  2.64008  3.57724  2.15421  4.05105  3.28355  3.31947
2.90744  2.32665  2.23268  5.10743  3.86397    483 i - - -
          2.68516  4.42269  2.77446  2.73054  3.46163  2.40557  3.72538
3.29355  2.67741  2.69314  4.24734  2.90358  2.73784  3.18190  2.89845
2.37931  2.77564  2.98562  4.58521  3.61547
          0.47685  0.98706  5.02181  1.66365  0.21004  0.97767  0.47190
    406   1.73442  5.06004  2.70200  1.33348  4.54445  3.39142  3.91447
3.90829  2.77830  3.54836  4.40190  3.01825  3.96104  3.08232  3.24242
2.84001  3.14036  3.53220  5.77808  4.40954    490 e - - -
          2.68618  4.42225  2.77519  2.73123  3.46354  2.40513  3.72494
3.29354  2.67741  2.69355  4.24690  2.90347  2.73739  3.18146  2.89801
2.37887  2.77519  2.98518  4.58477  3.61503
          0.02037  4.29947  5.02181  0.61958  0.77255  0.97767  0.47190
    407   3.92221  5.23510  4.49909  4.32636  2.44627  4.35140  3.90623
3.88116  4.17467  3.21920  4.51293  4.29020  4.82317  4.37129  4.26143
4.00746  4.21536  3.77389  4.09983  0.47322    491 Y - - -
          2.68618  4.42225  2.77519  2.73123  3.46354  2.40513  3.72494
3.29354  2.67741  2.69355  4.24690  2.90347  2.73739  3.18146  2.89801
2.37887  2.77519  2.98518  4.58477  3.61503
          0.02037  4.29947  5.02181  0.61958  0.77255  0.97767  0.47190
    408   2.72716  4.91515  3.11324  2.62568  4.40159  3.45585  3.78062
3.80736  1.45103  3.37594  4.19038  3.10138  3.92902  2.92548  2.78737
2.20650  2.32891  3.42826  5.55539  4.25053    492 k - - -
          2.68618  4.42225  2.77519  2.73123  3.46354  2.40513  3.72494
3.29354  2.67741  2.69355  4.24690  2.90347  2.73739  3.18146  2.89801
2.37887  2.77519  2.98518  4.58477  3.61503
          0.02037  4.29947  5.02181  0.61958  0.77255  0.97767  0.47190
    409   2.75675  5.07411  3.02049  2.49659  4.41862  3.49924  3.68034
3.84702  1.62575  3.37275  4.16727  3.01174  2.81103  2.47491  2.71250
2.74997  2.40855  3.47483  5.53211  4.19766    493 k - - -
          2.68848  4.41865  2.77636  2.73244  3.46433  2.40725  3.72626
3.29329  2.67936  2.69407  4.24095  2.89594  2.73823  3.18205  2.89919
2.37738  2.77484  2.98670  4.58013  3.60805
          0.67639  0.71019        *  3.03010  0.04952  0.00000        *
//
HMMER3/f [3.3 | Nov 2019]
NAME  CryM1
DESC  Cry toxin (IDOPS Model 1)
LENG  677
ALPH  amino
```

```
RF    no
MM    no
CONS  yes
CS    no
MAP   yes
DATE  Wed Feb  3 11:08:43 2021
NSEQ  34
EFFN  3.897217
CKSUM 3627063154
GA 344.0 344.0
STATS LOCAL MSV       -12.3121  0.69675
STATS LOCAL VITERBI   -13.2993  0.69675
STATS LOCAL FORWARD    -5.8328  0.69675
HMM          A        C        D        E        F        G        H
I        K        L        M        N        P        Q        R        S
T        V        W        Y
            m->m     m->i     m->d     i->m     i->i     d->m     d->d
  COMPO   2.61989  4.65092  2.95789  2.67075  3.28686  3.02706  3.68860
2.83518  2.68756  2.52490  3.75247  2.79948  3.36363  2.98655  2.95619
2.57327  2.70442  2.81671  4.38788  3.16919
          2.68661  4.42268  2.77544  2.73133  3.46397  2.40482  3.72489
3.29371  2.67718  2.69380  4.24284  2.90243  2.73782  3.18080  2.89809
2.37908  2.77562  2.98561  4.58520  3.61465
          0.32072  1.96465  2.00864  1.87133  0.16714  0.00000        *
      1   2.96561  4.73785  3.94684  3.64805  4.11384  3.75137  4.55068
3.52582  3.54075  3.23478  0.69853  3.22229  4.37725  3.93670  3.78416
2.75839  3.34918  3.25170  5.59742  4.32180     14 m - - -
          2.68618  4.42225  2.77519  2.73123  3.46354  2.40513  3.72494
3.29354  2.67741  2.69355  4.24690  2.90347  2.73739  3.18146  2.89801
2.37887  2.77519  2.98518  4.58477  3.61503
          0.04071  4.85532  3.43884  0.61958  0.77255  0.56545  0.83957
      2   2.90133  5.04443  3.27650  2.75758  4.26170  3.71253  3.93279
3.69388  2.52991  3.30800  3.11366  1.19689  4.11756  3.10006  3.15408
2.55628  3.13619  3.38356  5.54602  3.55027     15 n - - -
          2.68618  4.42225  2.77519  2.73123  3.46354  2.40513  3.72494
3.29354  2.67741  2.69355  4.24690  2.90347  2.73739  3.18146  2.89801
2.37887  2.77519  2.98518  4.58477  3.61503
          0.01121  4.89254  5.61489  0.61958  0.77255  0.72865  0.65886
      3   2.83712  5.32075  2.84189  2.39339  4.65468  3.61233  3.80214
4.13275  2.54140  3.03599  4.36479  2.84595  2.60117  2.27384  2.63359
1.80291  2.81135  3.70549  5.76001  4.35592     16 s - - -
          2.68618  4.42225  2.77519  2.73123  3.46354  2.40513  3.72494
3.29354  2.67741  2.69355  4.24690  2.90347  2.73739  3.18146  2.89801
2.37887  2.77519  2.98518  4.58477  3.61503
          0.01121  4.89254  5.61489  0.61958  0.77255  0.72865  0.65886
      4   2.85038  4.87028  3.01980  2.81764  3.52445  3.72421  3.93614
3.09542  2.80263  3.10529  3.43291  1.85681  4.11152  2.66620  3.23875
2.94058  3.08186  3.17698  5.37607  1.94151     17 n - - -
          2.68618  4.42225  2.77519  2.73123  3.46354  2.40513  3.72494
3.29354  2.67741  2.69355  4.24690  2.90347  2.73739  3.18146  2.89801
2.37887  2.77519  2.98518  4.58477  3.61503
          0.04337  4.89254  3.35416  0.61958  0.77255  0.72865  0.65886
      5   2.83942  4.04391  2.48077  2.04874  4.66683  3.60200  3.80043
4.14765  2.54457  3.63280  4.37617  2.16614  4.00258  1.79175  3.03772
2.61258  3.07170  3.71728  5.76956  3.71030     18 q - - -
```

```
         2.68618  4.42225  2.77519  2.73123  3.46354  2.40513  3.72494
3.29354  2.67741  2.69355  4.24690  2.90347  2.73739  3.18146  2.89801
2.37887  2.77519  2.98518  4.58477  3.61503
         0.32363  4.86075  1.31402  0.61958  0.77255  0.78896  0.60572
      6  3.21249  5.48323  2.89138  2.70862  4.90845  3.65472  4.05597
4.42738  2.10378  3.92695  4.78881  0.87691  4.21502  3.22109  3.03432
3.17787  3.49138  4.02994  5.98561  4.64196     19 n - - -
         2.68618  4.42225  2.77519  2.73123  3.46354  2.40513  3.72494
3.29354  2.67741  2.69355  4.24690  2.90347  2.73739  3.18146  2.89801
2.37887  2.77519  2.98518  4.58477  3.61503
         0.01578  4.55289  5.27524  0.61958  0.77255  0.45997  0.99778
      7  2.83368  5.30299  3.09883  2.54100  3.80516  3.22837  3.37949
4.10844  1.49655  3.27101  4.34804  2.62949  4.00327  2.42308  2.60420
2.47433  3.06370  3.68713  5.74069  4.34377     20 k - - -
         2.68618  4.42225  2.77519  2.73123  3.46354  2.40513  3.72494
3.29354  2.67741  2.69355  4.24690  2.90347  2.73739  3.18146  2.89801
2.37887  2.77519  2.98518  4.58477  3.61503
         0.01157  4.86075  5.58309  0.61958  0.77255  0.78896  0.60572
      8  3.29310  5.88008  2.07774  2.56782  5.19080  3.69479  4.13925
4.70692  2.69944  4.17520  4.97993  0.89720  4.26078  2.90900  3.60588
2.85626  3.56058  4.26277  6.31621  4.83276     21 n - - -
         2.68618  4.42225  2.77519  2.73123  3.46354  2.40513  3.72494
3.29354  2.67741  2.69355  4.24690  2.90347  2.73739  3.18146  2.89801
2.37887  2.77519  2.98518  4.58477  3.61503
         0.04677  4.86075  3.27162  0.61958  0.77255  0.68251  0.70390
      9  3.27676  5.89121  2.43436  0.87651  5.19757  2.78091  4.12429
4.72094  2.69862  4.18144  4.97902  2.30782  4.24536  3.26310  3.61746
3.17333  3.54330  4.26783  6.31910  4.82510     22 e - - -
         2.68618  4.42225  2.77519  2.73123  3.46354  2.40513  3.72494
3.29354  2.67741  2.69355  4.24690  2.90347  2.73739  3.18146  2.89801
2.37887  2.77519  2.98518  4.58477  3.61503
         0.01164  4.85471  5.57706  0.61958  0.77255  0.67862  0.70788
     10  2.86034  4.61620  3.22934  3.11286  3.71795  3.82136  3.53989
2.84897  2.58740  2.61651  2.95589  3.53163  4.20145  3.37610  3.42113
3.06342  3.09150  2.89348  5.16026  1.38220     23 y - - -
         2.68618  4.42225  2.77519  2.73123  3.46354  2.40513  3.72494
3.29354  2.67741  2.69355  4.24690  2.90347  2.73739  3.18146  2.89801
2.37887  2.77519  2.98518  4.58477  3.61503
         0.04470  4.88670  3.31955  0.61958  0.77255  0.61238  0.78102
     11  3.20869  5.79543  2.19025  0.89524  5.11179  3.68964  4.07723
4.62391  2.44452  4.08668  4.86901  3.14115  4.21962  2.83207  3.48885
2.77257  3.46557  4.17536  6.21994  4.74989     24 e - - -
         2.68618  4.42225  2.77519  2.73123  3.46354  2.40513  3.72494
3.29354  2.67741  2.69355  4.24690  2.90347  2.73739  3.18146  2.89801
2.37887  2.77519  2.98518  4.58477  3.61503
         0.01127  4.88750  5.60985  0.61958  0.77255  0.73859  0.64968
     12  2.92562  4.38559  3.68570  3.82579  3.50498  4.04952  4.36367
1.10142  3.28366  2.21468  3.09237  4.03396  4.41347  3.92882  3.88535
3.34016  2.67513  2.30081  5.03943  3.84711     25 i - - -
         2.68618  4.42225  2.77519  2.73123  3.46354  2.40513  3.72494
3.29354  2.67741  2.69355  4.24690  2.90347  2.73739  3.18146  2.89801
2.37887  2.77519  2.98518  4.58477  3.61503
         0.01127  4.88750  5.60985  0.61958  0.77255  0.73859  0.64968
```

```
     13   3.18539  4.54866  5.00285  4.41081  3.49776  4.39014  4.73457
1.74178  4.24276  1.02869  2.98666  4.49992  4.71050  4.38044  4.30783
2.59285  3.41742  2.26527  5.20698  4.05939     26 l - - -
          2.68618  4.42225  2.77519  2.73123  3.46354  2.40513  3.72494
3.29354  2.67741  2.69355  4.24690  2.90347  2.73739  3.18146  2.89801
2.37887  2.77519  2.98518  4.58477  3.61503
          0.15352  4.88750  2.00413  0.61958  0.77255  0.73859  0.64968
     14   3.14625  5.74224  1.40109  2.26570  5.05413  3.05355  4.01464
4.57090  2.91098  4.03517  4.82053  1.69767  4.14755  2.48300  3.46915
2.69762  3.40680  4.12037  6.17232  4.69224     27 d - - -
          2.68618  4.42225  2.77519  2.73123  3.46354  2.40513  3.72494
3.29354  2.67741  2.69355  4.24690  2.90347  2.73739  3.18146  2.89801
2.37887  2.77519  2.98518  4.58477  3.61503
          0.01298  4.74696  5.46930  0.61958  0.77255  0.96698  0.47841
     15   1.79789  5.12460  2.87587  2.55228  4.43095  3.57575  3.78810
3.87724  2.55358  3.42600  4.20354  2.63840  3.43546  2.90914  2.78710
2.27575  2.29525  3.11450  5.61646  4.24587     28 a - - -
          2.68618  4.42225  2.77519  2.73123  3.46354  2.40513  3.72494
3.29354  2.67741  2.69355  4.24690  2.90347  2.73739  3.18146  2.89801
2.37887  2.77519  2.98518  4.58477  3.61503
          0.01298  4.74696  5.46930  0.61958  0.77255  0.96698  0.47841
     16   2.41604  5.04685  3.14899  2.60067  4.33212  2.69581  3.31563
3.29737  2.42867  3.34136  4.13843  3.12429  2.84511  2.95175  3.06595
1.64791  3.02206  3.41208  5.56043  4.21031     29 s - - -
          2.68618  4.42225  2.77519  2.73123  3.46354  2.40513  3.72494
3.29354  2.67741  2.69355  4.24690  2.90347  2.73739  3.18146  2.89801
2.37887  2.77519  2.98518  4.58477  3.61503
          0.12576  4.74696  2.21190  0.61958  0.77255  0.96698  0.47841
     17   2.75333  5.19445  3.02921  2.31313  4.51600  3.53464  3.71911
3.98136  2.20832  3.10439  4.24252  3.01318  3.02942  2.31133  2.37480
2.02943  2.98332  3.57266  5.63878  3.57580     30 s - - -
          2.68618  4.42225  2.77519  2.73123  3.46354  2.40513  3.72494
3.29354  2.67741  2.69355  4.24690  2.90347  2.73739  3.18146  2.89801
2.37887  2.77519  2.98518  4.58477  3.61503
          0.05022  4.63571  3.23698  0.61958  0.77255  0.58732  0.81152
     18   2.86263  5.33434  2.61244  2.38007  4.65832  3.59473  3.81613
4.13315  2.05041  2.66303  4.38531  1.62361  4.01149  2.92418  3.06403
2.46143  3.09713  3.71453  5.77663  4.37170     31 n - - -
          2.68611  4.42150  2.77522  2.73127  3.46375  2.40534  3.72473
3.29344  2.67762  2.69342  4.24711  2.90347  2.73680  3.18168  2.89822
2.37908  2.77474  2.98521  4.58498  3.61524
          0.40218  1.81186  1.78504  1.27570  0.32744  0.78949  0.60528
     19   2.74823  3.81513  3.35446  2.79281  3.86043  3.64920  3.30310
2.74977  2.42761  2.91471  3.27472  1.90908  4.03425  3.10154  3.18850
2.69180  2.72998  2.98119  5.23500  3.02615     42 n - - -
          2.68618  4.42225  2.77519  2.73123  3.46354  2.40513  3.72494
3.29354  2.67741  2.69355  4.24690  2.90347  2.73739  3.18146  2.89801
2.37887  2.77519  2.98518  4.58477  3.61503
          0.04892  4.65340  3.26462  0.61958  0.77255  1.08106  0.41436
     20   2.73486  3.78443  2.91465  2.47612  3.41977  2.91108  3.82342
3.37784  2.67389  2.57523  3.86778  3.18339  3.99244  3.01363  3.11523
1.87841  2.40288  3.09337  5.31661  4.01636     43 s - - -
          2.68618  4.42225  2.77519  2.73123  3.46354  2.40513  3.72494
3.29354  2.67741  2.69355  4.24690  2.90347  2.73739  3.18146  2.89801
2.37887  2.77519  2.98518  4.58477  3.61503
```

0.01476 4.61924 5.34159 0.61958 0.77255 0.65475 0.73308
21 2.79788 3.14417 3.22092 2.51866 4.19111 3.22997 3.36506
3.60214 2.65425 3.21674 4.03663 1.78820 4.02674 3.00250 2.88836
2.50470 2.81549 2.74625 5.47255 3.49012 44 n - - -
2.68618 4.42225 2.77519 2.73123 3.46354 2.40513 3.72494
3.29354 2.67741 2.69355 4.24690 2.90347 2.73739 3.18146 2.89801
2.37887 2.77519 2.98518 4.58477 3.61503
0.01241 4.79134 5.51368 0.61958 0.77255 0.36759 1.17897
22 2.87959 4.55520 3.83449 3.26516 3.68537 3.38404 4.13964
2.63685 3.21155 2.16730 1.80767 2.72251 3.64951 3.13683 3.54479
2.52606 2.67528 2.66503 5.14337 3.91738 45 m - - -
2.68618 4.42225 2.77519 2.73123 3.46354 2.40513 3.72494
3.29354 2.67741 2.69355 4.24690 2.90347 2.73739 3.18146 2.89801
2.37887 2.77519 2.98518 4.58477 3.61503
0.01041 4.96582 5.68817 0.61958 0.77255 0.56492 0.84027
23 2.60188 4.91981 3.38587 2.82691 4.12861 3.73819 3.96218
3.52310 2.80820 2.26419 3.48450 3.06239 2.59296 3.15151 2.76218
1.61094 2.88562 3.23706 5.45920 4.15920 46 s - - -
2.68618 4.42225 2.77519 2.73123 3.46354 2.40513 3.72494
3.29354 2.67741 2.69355 4.24690 2.90347 2.73739 3.18146 2.89801
2.37887 2.77519 2.98518 4.58477 3.61503
0.20363 4.96582 1.73012 0.61958 0.77255 0.56492 0.84027
24 2.63922 5.26873 2.01398 2.50679 4.59400 3.57509 3.78034
4.06581 2.53183 3.56797 4.32117 1.92691 3.97844 2.88638 2.77306
2.31388 2.63194 3.65014 5.71873 3.62313 47 n - - -
2.68618 4.42225 2.77519 2.73123 3.46354 2.40513 3.72494
3.29354 2.67741 2.69355 4.24690 2.90347 2.73739 3.18146 2.89801
2.37887 2.77519 2.98518 4.58477 3.61503
0.01262 4.77481 5.49716 0.61958 0.77255 0.82156 0.57936
25 2.80003 4.01773 2.70359 2.52458 4.54616 2.93696 3.77703
4.01055 2.34937 3.52427 3.69337 2.11432 3.52170 2.88451 2.59739
2.10082 3.03124 3.60613 5.68440 3.27224 48 s - - -
2.68618 4.42225 2.77519 2.73123 3.46354 2.40513 3.72494
3.29354 2.67741 2.69355 4.24690 2.90347 2.73739 3.18146 2.89801
2.37887 2.77519 2.98518 4.58477 3.61503
0.04738 4.80505 3.26799 0.61958 0.77255 0.76141 0.62925
26 2.80369 5.09098 3.15345 2.59663 4.35497 3.61598 3.42073
3.78730 2.39977 3.08659 4.15421 2.78308 3.46838 2.26705 3.06168
2.38424 2.79621 3.13178 5.57497 2.08116 49 y - - -
2.68618 4.42225 2.77519 2.73123 3.46354 2.40513 3.72494
3.29354 2.67741 2.69355 4.24690 2.90347 2.73739 3.18146 2.89801
2.37887 2.77519 2.98518 4.58477 3.61503
0.09247 4.80510 2.52400 0.61958 0.77255 0.37394 1.16481
27 2.84577 5.13552 3.18435 2.47486 4.41646 3.18238 3.86409
3.85040 2.64324 2.72274 4.21415 1.97452 2.13134 2.99597 3.11705
2.35897 2.85449 3.17779 5.63405 4.27704 50 n - - -
2.68618 4.42225 2.77519 2.73123 3.46354 2.40513 3.72494
3.29354 2.67741 2.69355 4.24690 2.90347 2.73739 3.18146 2.89801
2.37887 2.77519 2.98518 4.58477 3.61503
0.01112 4.90004 5.62239 0.61958 0.77255 0.71359 0.67311
28 2.86939 5.32595 2.83261 2.57760 4.65908 3.24762 3.82059
3.54661 1.96483 3.61981 4.37234 2.90073 4.03511 2.74709 1.46473
2.84018 3.09797 3.71078 5.75975 3.65611 51 r - - -

2.68618 4.42225 2.77519 2.73123 3.46354 2.40513 3.72494
3.29354 2.67741 2.69355 4.24690 2.90347 2.73739 3.18146 2.89801
2.37887 2.77519 2.98518 4.58477 3.61503
0.03889 4.90004 3.48355 0.61958 0.77255 0.71359 0.67311
29 3.20057 4.93750 3.89300 3.37268 3.31561 4.07116 3.33572
3.48576 2.71302 2.76787 4.05533 3.74073 4.45353 3.56695 3.41361
2.92068 3.42572 3.26248 4.86185 0.91040 52 y - - -
2.68618 4.42225 2.77519 2.73123 3.46354 2.40513 3.72494
3.29354 2.67741 2.69355 4.24690 2.90347 2.73739 3.18146 2.89801
2.37887 2.77519 2.98518 4.58477 3.61503
0.01144 4.87258 5.59493 0.61958 0.77255 0.76715 0.62424
30 2.91304 3.45927 4.26630 3.71214 3.70014 3.91248 4.40689
2.93658 3.59598 2.27681 3.16776 3.95009 1.05761 3.86718 3.83680
3.23470 2.85227 2.74165 5.22844 4.03103 53 p - - -
2.68618 4.42225 2.77519 2.73123 3.46354 2.40513 3.72494
3.29354 2.67741 2.69355 4.24690 2.90347 2.73739 3.18146 2.89801
2.37887 2.77519 2.98518 4.58477 3.61503
0.01108 4.90377 5.62612 0.61958 0.77255 0.51194 0.91462
31 2.74376 4.37678 4.92948 4.31795 2.25500 4.17644 4.50383
2.23581 4.11377 1.26223 2.52323 4.32629 4.52684 4.23913 4.13161
3.48857 2.95747 2.58489 4.98417 2.72527 54 l - - -
2.68618 4.42225 2.77519 2.73123 3.46354 2.40513 3.72494
3.29354 2.67741 2.69355 4.24690 2.90347 2.73739 3.18146 2.89801
2.37887 2.77519 2.98518 4.58477 3.61503
0.01009 4.99692 5.71927 0.61958 0.77255 0.62213 0.76960
32 1.44134 5.34095 2.91968 2.34143 4.66436 3.66103 3.85378
4.13506 2.29097 3.26448 4.38841 3.13740 3.58748 2.40548 3.08611
2.85635 2.52362 3.72010 5.78690 4.38946 55 a - - -
2.68618 4.42225 2.77519 2.73123 3.46354 2.40513 3.72494
3.29354 2.67741 2.69355 4.24690 2.90347 2.73739 3.18146 2.89801
2.37887 2.77519 2.98518 4.58477 3.61503
0.03599 4.99692 3.55465 0.61958 0.77255 0.62213 0.76960
33 2.87339 5.37095 2.71595 2.56628 4.71104 3.64428 3.83363
4.19289 1.99260 3.67341 4.41354 1.64622 4.03960 2.54116 2.85800
2.52876 2.67809 3.75905 5.80574 3.54528 56 n - - -
2.68618 4.42225 2.77519 2.73123 3.46354 2.40513 3.72494
3.29354 2.67741 2.69355 4.24690 2.90347 2.73739 3.18146 2.89801
2.37887 2.77519 2.98518 4.58477 3.61503
0.01036 4.97128 5.69363 0.61958 0.77255 0.68235 0.70406
34 2.41175 5.33838 1.91238 2.33023 4.66641 3.64556 3.83648
4.14084 2.40497 3.63639 3.69789 2.28873 4.04005 2.93877 3.06941
2.66736 2.47785 3.71988 5.78129 3.05545 57 d - - -
2.68618 4.42225 2.77519 2.73123 3.46354 2.40513 3.72494
3.29354 2.67741 2.69355 4.24690 2.90347 2.73739 3.18146 2.89801
2.37887 2.77519 2.98518 4.58477 3.61503
0.01036 4.97128 5.69363 0.61958 0.77255 0.68235 0.70406
35 2.69597 4.93802 3.43200 2.87255 4.14245 3.77324 3.98384
3.53545 2.79702 2.56064 4.02913 3.36041 1.35406 2.87823 2.67010
2.79555 3.14255 3.25464 5.46845 3.56267 58 p - - -
2.68618 4.42225 2.77519 2.73123 3.46354 2.40513 3.72494
3.29354 2.67741 2.69355 4.24690 2.90347 2.73739 3.18146 2.89801
2.37887 2.77519 2.98518 4.58477 3.61503
0.01036 4.97128 5.69363 0.61958 0.77255 0.68235 0.70406

```
     36   2.86490  4.14271  2.90157  2.58490  4.62583  3.00319  3.83957
3.53921  2.10664  2.78746  4.35600  1.90788  3.38106  2.32003  3.07342
2.68164  2.91196  3.68393  5.75739  4.36357     59 n - - -
          2.68618  4.42225  2.77519  2.73123  3.46354  2.40513  3.72494
3.29354  2.67741  2.69355  4.24690  2.90347  2.73739  3.18146  2.89801
2.37887  2.77519  2.98518  4.58477  3.61503
          0.01036  4.97128  5.69363  0.61958  0.77255  0.68235  0.70406
     37   2.04231  4.79471  3.21796  2.93682  3.96649  3.78178  4.00966
2.80696  2.69572  2.71344  3.08072  3.40814  3.63858  2.45528  3.33005
2.20592  3.10662  2.19587  5.34766  4.07425     60 a - - -
          2.68618  4.42225  2.77519  2.73123  3.46354  2.40513  3.72494
3.29354  2.67741  2.69355  4.24690  2.90347  2.73739  3.18146  2.89801
2.37887  2.77519  2.98518  4.58477  3.61503
          0.04331  4.97128  3.33960  0.61958  0.77255  0.68235  0.70406
     38   2.39284  5.00098  3.29280  2.17054  4.22393  3.29434  3.90841
2.96726  2.72509  2.99389  3.12322  3.24669  2.73160  3.07163  2.83365
2.08563  2.70615  3.32419  5.51463  3.59272     61 s - - -
          2.68626  4.42233  2.77528  2.73132  3.46362  2.40521  3.72503
3.29362  2.67729  2.69363  4.24539  2.90329  2.73730  3.18127  2.89809
2.37883  2.77528  2.98527  4.58485  3.61511
          0.11151  2.28243  5.66102  0.77750  0.61536  0.52756  0.89170
     39   2.70379  4.32060  4.84025  4.22923  2.79571  4.10160  4.43160
2.39945  3.36096  1.30924  2.04163  4.24378  4.46357  4.16856  4.05707
3.41064  2.88848  2.39595  3.86956  3.76444     65 l - - -
          2.68618  4.42225  2.77519  2.73123  3.46354  2.40513  3.72494
3.29354  2.67741  2.69355  4.24690  2.90347  2.73739  3.18146  2.89801
2.37887  2.77519  2.98518  4.58477  3.61503
          0.03722  5.02644  3.50730  0.61958  0.77255  0.51634  0.90807
     40   2.89728  5.40145  2.82193  2.33872  4.74568  3.66764  3.85427
3.19819  2.02923  3.70489  4.44307  2.54411  4.06215  1.63603  2.50832
2.70076  3.12806  3.79180  5.83307  4.42531     66 q - - -
          2.68618  4.42225  2.77519  2.73123  3.46354  2.40513  3.72494
3.29354  2.67741  2.69355  4.24690  2.90347  2.73739  3.18146  2.89801
2.37887  2.77519  2.98518  4.58477  3.61503
          0.00982  5.02422  5.74656  0.61958  0.77255  0.63134  0.75903
     41   2.63803  5.38913  2.68632  2.46752  4.72616  3.28811  3.40512
3.62700  2.46793  3.69098  4.43240  1.45354  4.06194  2.75569  3.09059
2.38855  2.92349  3.77554  5.82629  4.41889     67 n - - -
          2.68618  4.42225  2.77519  2.73123  3.46354  2.40513  3.72494
3.29354  2.67741  2.69355  4.24690  2.90347  2.73739  3.18146  2.89801
2.37887  2.77519  2.98518  4.58477  3.61503
          0.00982  5.02422  5.74656  0.61958  0.77255  0.51260  0.91364
     42   2.51063  3.97050  3.41106  2.59777  4.15958  3.36833  3.98983
3.20420  2.63104  2.76387  2.64893  3.05467  3.30889  3.17803  2.78913
2.77281  1.76670  3.26963  5.48997  4.18829     68 t - - -
          2.68618  4.42225  2.77519  2.73123  3.46354  2.40513  3.72494
3.29354  2.67741  2.69355  4.24690  2.90347  2.73739  3.18146  2.89801
2.37887  2.77519  2.98518  4.58477  3.61503
          0.00956  5.05071  5.77306  0.61958  0.77255  0.56193  0.84423
     43   2.93918  5.45200  2.87124  2.27390  4.79318  3.68513  3.11357
4.27998  2.46659  3.75537  4.49560  1.35721  3.61641  2.56857  3.13510
2.52991  2.94862  3.84092  5.88486  4.47024     69 n - - -
          2.68618  4.42225  2.77519  2.73123  3.46354  2.40513  3.72494
3.29354  2.67741  2.69355  4.24690  2.90347  2.73739  3.18146  2.89801
2.37887  2.77519  2.98518  4.58477  3.61503
```

```
          0.00956  5.05071  5.77306  0.61958  0.77255  0.56193  0.84423
     44   2.96037  4.55352  4.05648  3.12675  3.64139  3.43469  4.27303
2.57439  3.44445  2.76584  3.67866  3.83914  4.37086  3.72279  3.72068
2.90013  3.19997  2.61130  5.13091  1.04942     70 y - - -
          2.68618  4.42225  2.77519  2.73123  3.46354  2.40513  3.72494
3.29354  2.67741  2.69355  4.24690  2.90347  2.73739  3.18146  2.89801
2.37887  2.77519  2.98518  4.58477  3.61503
          0.03669  5.05071  3.51924  0.61958  0.77255  0.56193  0.84423
     45   3.01227  5.45893  3.22958  2.49819  4.80962  3.75483  3.92167
4.26919  1.04828  3.74877  4.51018  2.96764  4.15315  2.46078  3.00895
2.52787  3.23726  3.23276  5.87319  4.50078     71 k - - -
          2.68618  4.42225  2.77519  2.73123  3.46354  2.40513  3.72494
3.29354  2.67741  2.69355  4.24690  2.90347  2.73739  3.18146  2.89801
2.37887  2.77519  2.98518  4.58477  3.61503
          0.04122  5.02384  3.38711  0.61958  0.77255  0.63227  0.75797
     46   3.31695  5.91644  0.96432  1.97258  5.23085  3.75653  4.18209
4.75035  3.09368  4.21467  5.00462  2.57579  2.81722  3.31711  3.65912
2.85704  3.14873  4.29805  6.35333  4.86727     72 d - - -
          2.68618  4.42225  2.77519  2.73123  3.46354  2.40513  3.72494
3.29354  2.67741  2.69355  4.24690  2.90347  2.73739  3.18146  2.89801
2.37887  2.77519  2.98518  4.58477  3.61503
          0.03459  4.99275  3.60397  0.61958  0.77255  0.46501  0.98921
     47   3.31492  3.69492  4.94600  4.40686  2.34842  4.39579  4.29654
3.14311  4.22991  2.82006  3.80656  4.39140  4.74741  3.42853  4.29173
3.70818  3.54455  2.69687  0.92561  2.60057     73 w - - -
          2.68618  4.42225  2.77519  2.73123  3.46354  2.40513  3.72494
3.29354  2.67741  2.69355  4.24690  2.90347  2.73739  3.18146  2.89801
2.37887  2.77519  2.98518  4.58477  3.61503
          0.00978  5.02805  5.75040  0.61958  0.77255  0.50140  0.93060
     48   2.92983  4.42713  4.32023  3.29970  2.81328  4.01991  4.31719
2.50847  3.10174  1.25705  2.68271  3.06225  4.39038  3.37918  3.83039
3.30128  2.91913  2.52354  5.03609  3.84093     74 l - - -
          2.68618  4.42225  2.77519  2.73123  3.46354  2.40513  3.72494
3.29354  2.67741  2.69355  4.24690  2.90347  2.73739  3.18146  2.89801
2.37887  2.77519  2.98518  4.58477  3.61503
          0.00952  5.05481  5.77716  0.61958  0.77255  0.44726  1.01992
     49   2.56312  5.41864  2.16573  2.48207  4.76160  3.68401  3.87311
4.24636  2.35439  3.72302  4.46008  1.74397  3.19762  2.79120  2.91424
2.42398  2.94076  3.39633  5.85245  4.44228     75 n - - -
          2.68618  4.42225  2.77519  2.73123  3.46354  2.40513  3.72494
3.29354  2.67741  2.69355  4.24690  2.90347  2.73739  3.18146  2.89801
2.37887  2.77519  2.98518  4.58477  3.61503
          0.00931  5.07704  5.79939  0.61958  0.77255  0.48576  0.95510
     50   2.67777  4.45400  4.27497  2.73141  3.56209  3.44906  4.31329
2.24679  3.59263  2.47533  1.55535  3.43190  4.39134  3.83258  3.81402
3.29780  2.72514  2.32759  5.06138  3.12903     76 m - - -
          2.68618  4.42225  2.77519  2.73123  3.46354  2.40513  3.72494
3.29354  2.67741  2.69355  4.24690  2.90347  2.73739  3.18146  2.89801
2.37887  2.77519  2.98518  4.58477  3.61503
          0.00931  5.07704  5.79939  0.61958  0.77255  0.48576  0.95510
     51   2.93246  1.58793  3.25573  3.29555  3.75502  3.92355  4.18559
3.11646  3.24509  2.83438  3.73026  2.96010  4.30400  3.54053  3.58494
2.42491  2.75959  2.66787  4.02120  2.51105     77 c - - -
```

```
         2.68618  4.42225  2.77519  2.73123  3.46354  2.40513  3.72494
3.29354  2.67741  2.69355  4.24690  2.90347  2.73739  3.18146  2.89801
2.37887  2.77519  2.98518  4.58477  3.61503
         0.06040  5.07704  2.94934  0.61958  0.77255  0.48576  0.95510
     52  2.44310  5.38710  2.75819  1.86891  4.72788  3.36498  3.34462
3.29967  2.45881  3.31156  4.42889  2.71554  4.05535  2.20012  2.84785
2.60677  2.72185  3.36723  5.82235  4.41399     78 e - - -
         2.68618  4.42225  2.77519  2.73123  3.46354  2.40513  3.72494
3.29354  2.67741  2.69355  4.24690  2.90347  2.73739  3.18146  2.89801
2.37887  2.77519  2.98518  4.58477  3.61503
         0.00980  5.02644  5.74878  0.61958  0.77255  0.62577  0.76539
     53  2.90021  5.39588  2.16310  2.32492  3.90778  2.33407  3.86402
3.69921  2.02201  3.69849  3.67894  2.91312  3.57689  2.77882  2.73728
2.68582  2.90161  3.78314  5.83234  3.70205     79 k - - -
         2.68618  4.42225  2.77519  2.73123  3.46354  2.40513  3.72494
3.29354  2.67741  2.69355  4.24690  2.90347  2.73739  3.18146  2.89801
2.37887  2.77519  2.98518  4.58477  3.61503
         0.03237  5.05822  3.66944  0.61958  0.77255  0.62577  0.76539
     54  2.89609  5.11625  2.84093  2.02982  3.30035  3.72325  3.92593
2.63290  2.72121  3.37703  4.18530  1.84963  4.11336  3.07176  2.95300
2.72531  2.72374  3.03376  4.14234  4.27533     80 n - - -
         2.68618  4.42225  2.77519  2.73123  3.46354  2.40513  3.72494
3.29354  2.67741  2.69355  4.24690  2.90347  2.73739  3.18146  2.89801
2.37887  2.77519  2.98518  4.58477  3.61503
         0.00971  5.03556  5.75790  0.61958  0.77255  0.68235  0.70406
     55  2.68402  5.38693  2.93921  2.45740  4.72587  3.29457  3.85450
3.47982  2.08995  3.68960  4.42922  2.11307  3.59653  2.36461  3.08341
2.27805  2.52218  3.77428  5.82328  3.40299     81 k - - -
         2.68618  4.42225  2.77519  2.73123  3.46354  2.40513  3.72494
3.29354  2.67741  2.69355  4.24690  2.90347  2.73739  3.18146  2.89801
2.37887  2.77519  2.98518  4.58477  3.61503
         0.04073  5.03556  3.39882  0.61958  0.77255  0.68235  0.70406
     56  2.87876  5.31403  2.71840  2.60210  4.62878  3.32480  3.85516
3.41402  2.45057  3.60629  4.36280  2.70031  2.11922  2.45534  2.86997
2.53523  2.59526  2.77144  5.76530  3.75542     82 p - - -
         2.68618  4.42225  2.77519  2.73123  3.46354  2.40513  3.72494
3.29354  2.67741  2.69355  4.24690  2.90347  2.73739  3.18146  2.89801
2.37887  2.77519  2.98518  4.58477  3.61503
         0.10512  5.00484  2.37431  0.61958  0.77255  0.53939  0.87492
     57  2.86313  5.30205  2.51871  1.87848  4.61811  2.80773  3.83866
4.08419  2.58593  2.78956  3.68062  2.71584  4.04096  2.75585  3.07346
2.22729  3.09435  3.29270  5.75247  3.05781     83 e - - -
         2.68618  4.42225  2.77519  2.73123  3.46354  2.40513  3.72494
3.29354  2.67741  2.69355  4.24690  2.90347  2.73739  3.18146  2.89801
2.37887  2.77519  2.98518  4.58477  3.61503
         0.05197  4.96582  3.13116  0.61958  0.77255  0.72279  0.66436
     58  2.27261  3.44650  3.10077  2.49863  3.54031  2.61896  3.97434
2.79875  2.67400  2.88698  3.93361  3.35349  3.55216  3.18520  3.27985
2.15956  2.84820  2.59044  5.38910  4.10276     84 s - - -
         2.68618  4.42225  2.77519  2.73123  3.46354  2.40513  3.72494
3.29354  2.67741  2.69355  4.24690  2.90347  2.73739  3.18146  2.89801
2.37887  2.77519  2.98518  4.58477  3.61503
         0.04737  4.95155  3.23933  0.61958  0.77255  0.72491  0.66236
```

```
     59   2.87086  4.57374  3.77771  2.92360  3.03863  3.40110  4.11404
2.60354  3.16099  1.63166  3.67788  2.90398  4.23667  3.10491  3.13797
2.48855  2.63158  2.48111  5.15798  3.92696     85 l - - -
          2.68618  4.42225  2.77519  2.73123  3.46354  2.40513  3.72494
3.29354  2.67741  2.69355  4.24690  2.90347  2.73739  3.18146  2.89801
2.37887  2.77519  2.98518  4.58477  3.61503
          0.09724  4.94870  2.45839  0.61958  0.77255  0.86442  0.54696
     60   2.65490  4.83411  2.84198  2.80881  3.47672  3.04690  3.92693
2.85334  2.79229  2.30871  3.47545  2.76204  3.16458  2.69874  3.22674
2.40869  2.79933  2.64402  5.37265  4.08086     86 l - - -
          2.68618  4.42225  2.77519  2.73123  3.46354  2.40513  3.72494
3.29354  2.67741  2.69355  4.24690  2.90347  2.73739  3.18146  2.89801
2.37887  2.77519  2.98518  4.58477  3.61503
          0.17821  4.86300  1.86109  0.61958  0.77255  1.00475  0.45592
     61   2.40582  5.23064  2.42994  2.33259  4.55781  3.16964  3.73270
3.53212  2.47551  3.15687  4.27641  2.34582  3.08284  2.64748  2.96539
2.28714  2.76080  3.61170  5.67440  4.27425     87 s - - -
          2.68618  4.42225  2.77519  2.73123  3.46354  2.40513  3.72494
3.29354  2.67741  2.69355  4.24690  2.90347  2.73739  3.18146  2.89801
2.37887  2.77519  2.98518  4.58477  3.61503
          0.13778  4.69842  2.12362  0.61958  0.77255  0.45156  1.01235
     62   2.65346  5.22759  2.52392  2.29678  4.52557  3.29606  3.82333
2.22137  2.58061  3.16551  4.28130  2.87121  2.62247  2.72836  3.06434
2.39662  2.86581  3.59273  5.68970  4.30877     88 i - - -
          2.68656  4.42278  2.77554  2.72987  3.46189  2.40566  3.72491
3.29348  2.67794  2.69334  4.24743  2.90302  2.73739  3.18140  2.89830
2.37900  2.77496  2.98549  4.58417  3.61556
          1.08762  0.72732  1.71602  1.06992  0.42013  0.81168  0.58719
     63   2.41841  4.48611  3.23475  2.60780  3.12408  3.77159  4.02790
2.74684  3.07583  2.19956  3.59028  3.52139  3.57298  3.02027  3.42164
3.01851  2.27041  2.39614  3.34209  3.34033     95 l - - -
          2.68618  4.42225  2.77519  2.73123  3.46354  2.40513  3.72494
3.29354  2.67741  2.69355  4.24690  2.90347  2.73739  3.18146  2.89801
2.37887  2.77519  2.98518  4.58477  3.61503
          0.08752  4.74569  2.58875  0.61958  0.77255  0.85361  0.55491
     64   2.46340  5.21305  2.49867  2.38277  3.45428  3.57608  3.76732
3.98610  2.51704  2.86080  4.26301  3.05824  3.45034  2.48625  2.44991
2.05318  2.81310  3.58509  5.66693  4.27776     96 s - - -
          2.68618  4.42225  2.77519  2.73123  3.46354  2.40513  3.72494
3.29354  2.67741  2.69355  4.24690  2.90347  2.73739  3.18146  2.89801
2.37887  2.77519  2.98518  4.58477  3.61503
          0.01264  4.77333  5.49567  0.61958  0.77255  0.90661  0.51733
     65   2.46105  5.14727  3.13949  2.44432  4.42605  3.27811  3.81267
2.94395  2.24359  3.42378  4.20605  2.35642  4.00856  2.74693  2.82903
2.35465  2.61891  2.93382  5.62180  3.21796     97 k - - -
          2.68618  4.42225  2.77519  2.73123  3.46354  2.40513  3.72494
3.29354  2.67741  2.69355  4.24690  2.90347  2.73739  3.18146  2.89801
2.37887  2.77519  2.98518  4.58477  3.61503
          0.06747  4.83735  2.85912  0.61958  0.77255  0.72814  0.65934
     66   2.50063  5.21846  2.12607  2.57237  4.51746  2.36239  3.81672
3.51637  2.57388  3.18843  4.27343  2.39270  4.01528  2.74339  3.05738
2.51029  2.86394  2.94437  5.68199  4.30142     98 d - - -
          2.68618  4.42225  2.77519  2.73123  3.46354  2.40513  3.72494
3.29354  2.67741  2.69355  4.24690  2.90347  2.73739  3.18146  2.89801
2.37887  2.77519  2.98518  4.58477  3.61503
```

```
          0.01138  4.87742  5.59976  0.61958  0.77255  0.77772  0.61518
     67   2.46503  4.42467  4.08186  2.87069  3.53811  3.12519  4.20850
2.66551  3.42067  1.94971  3.08025  3.80485  4.29818  3.67846  3.67959
2.55736  2.18857  2.00659  5.02716  3.37680     99 l - - -
          2.68618  4.42225  2.77519  2.73123  3.46354  2.40513  3.72494
3.29354  2.67741  2.69355  4.24690  2.90347  2.73739  3.18146  2.89801
2.37887  2.77519  2.98518  4.58477  3.61503
          0.04832  4.93218  3.21996  0.61958  0.77255  0.89404  0.52594
     68   2.45887  5.25423  2.87593  2.41204  4.56317  3.01555  3.81421
3.50148  2.10968  3.21563  4.30459  3.10600  3.15632  2.74195  2.49264
2.52776  3.06568  2.31142  5.70915  4.32119    100 k - - -
          2.68618  4.42225  2.77519  2.73123  3.46354  2.40513  3.72494
3.29354  2.67741  2.69355  4.24690  2.90347  2.73739  3.18146  2.89801
2.37887  2.77519  2.98518  4.58477  3.61503
          0.01118  4.89504  5.61739  0.61958  0.77255  0.95594  0.48524
     69   2.12900  5.11160  2.59325  2.36103  3.62784  3.04060  3.85164
2.80877  2.63328  3.06988  4.17619  2.90533  4.04305  2.98625  3.10575
2.04728  2.65663  3.45571  5.59936  4.24666    101 s - - -
          2.68618  4.42225  2.77519  2.73123  3.46354  2.40513  3.72494
3.29354  2.67741  2.69355  4.24690  2.90347  2.73739  3.18146  2.89801
2.37887  2.77519  2.98518  4.58477  3.61503
          0.01118  4.89504  5.61739  0.61958  0.77255  0.58618  0.81294
     70   1.41750  4.51911  4.00253  3.43213  3.65755  2.62068  4.22469
2.77401  3.36187  2.40262  3.64354  3.76936  4.31161  3.63950  3.26908
3.19088  2.47563  2.14109  5.13401  3.91992    102 a - - -
          2.68618  4.42225  2.77519  2.73123  3.46354  2.40513  3.72494
3.29354  2.67741  2.69355  4.24690  2.90347  2.73739  3.18146  2.89801
2.37887  2.77519  2.98518  4.58477  3.61503
          0.01008  4.99797  5.72032  0.61958  0.77255  0.76720  0.62420
     71   2.53008  4.30261  4.85936  4.24720  2.39863  3.42766  4.42841
2.14351  4.04002  1.77657  3.06893  4.24726  4.45713  4.17664  4.05957
3.40578  3.15765  1.50905  4.92876  3.31263    103 v - - -
          2.68618  4.42225  2.77519  2.73123  3.46354  2.40513  3.72494
3.29354  2.67741  2.69355  4.24690  2.90347  2.73739  3.18146  2.89801
2.37887  2.77519  2.98518  4.58477  3.61503
          0.35269  4.99797  1.23630  0.61958  0.77255  0.54772  0.86338
     72   2.57844  4.92774  3.20627  2.48993  3.53164  2.93827  3.82536
3.17502  2.63968  2.66825  4.00287  2.92221  4.00689  2.47325  3.09725
1.89146  2.56472  3.25242  5.44031  4.11627    104 s - - -
          2.68618  4.42225  2.77519  2.73123  3.46354  2.40513  3.72494
3.29354  2.67741  2.69355  4.24690  2.90347  2.73739  3.18146  2.89801
2.37887  2.77519  2.98518  4.58477  3.61503
          0.01317  4.73202  5.45437  0.61958  0.77255  0.41143  1.08680
     73   1.83845  4.51196  3.99637  3.42323  2.95560  3.15570  4.20969
2.54669  2.98471  2.71664  3.27242  3.76410  4.31052  3.62944  3.64972
2.68853  1.72881  2.58259  5.10988  3.89602    105 t - - -
          2.68618  4.42225  2.77519  2.73123  3.46354  2.40513  3.72494
3.29354  2.67741  2.69355  4.24690  2.90347  2.73739  3.18146  2.89801
2.37887  2.77519  2.98518  4.58477  3.61503
          0.00993  5.01251  5.73486  0.61958  0.77255  0.73559  0.65243
     74   2.11135  4.48336  4.12473  3.55260  3.62224  1.83235  4.27264
2.26460  3.47150  2.70579  2.91974  3.84931  4.33952  3.73183  3.73580
2.50153  2.47497  2.38674  5.10955  3.90365    106 g - - -
```

2.68618 4.42225 2.77519 2.73123 3.46354 2.40513 3.72494
3.29354 2.67741 2.69355 4.24690 2.90347 2.73739 3.18146 2.89801
2.37887 2.77519 2.98518 4.58477 3.61503
0.00993 5.01251 5.73486 0.61958 0.77255 0.35627 1.20493
75 2.48775 4.36073 4.79893 4.19265 3.07435 4.12118 4.44851
1.54048 4.00596 2.16128 3.47930 3.49183 4.48598 4.16195 4.06063
3.08621 2.40243 1.73478 4.98289 3.37653 107 i - - -
2.68618 4.42225 2.77519 2.73123 3.46354 2.40513 3.72494
3.29354 2.67741 2.69355 4.24690 2.90347 2.73739 3.18146 2.89801
2.37887 2.77519 2.98518 4.58477 3.61503
0.00903 5.10728 5.82962 0.61958 0.77255 0.48576 0.95510
76 2.59260 5.35879 2.73164 2.49973 4.67367 2.04819 3.89987
3.02097 2.50193 3.65120 4.40773 2.73519 4.10194 3.00694 2.90504
1.98624 2.76970 3.09400 5.81020 4.41826 108 s - - -
2.68618 4.42225 2.77519 2.73123 3.46354 2.40513 3.72494
3.29354 2.67741 2.69355 4.24690 2.90347 2.73739 3.18146 2.89801
2.37887 2.77519 2.98518 4.58477 3.61503
0.03261 5.10728 3.64836 0.61958 0.77255 0.48576 0.95510
77 2.51764 4.41834 4.77562 4.18136 3.54088 4.17035 4.51729
1.36853 4.01585 2.27321 3.54801 4.26810 3.76371 4.19126 4.10434
2.86237 2.70145 1.58780 5.08057 3.89732 109 i - - -
2.68618 4.42225 2.77519 2.73123 3.46354 2.40513 3.72494
3.29354 2.67741 2.69355 4.24690 2.90347 2.73739 3.18146 2.89801
2.37887 2.77519 2.98518 4.58477 3.61503
0.03258 5.08391 3.65492 0.61958 0.77255 0.55575 0.85247
78 2.71461 4.38905 3.71810 3.25429 2.78819 4.05698 4.36733
1.72144 3.77266 2.15861 3.50539 4.07612 4.42502 3.97654 3.92084
2.81007 2.37077 1.77306 5.00539 3.81740 110 i - - -
2.68618 4.42225 2.77519 2.73123 3.46354 2.40513 3.72494
3.29354 2.67741 2.69355 4.24690 2.90347 2.73739 3.18146 2.89801
2.37887 2.77519 2.98518 4.58477 3.61503
0.00946 5.06079 5.78314 0.61958 0.77255 0.50766 0.92106
79 2.58249 5.28610 2.75023 2.66591 4.57765 1.93761 3.10083
3.54126 2.49801 2.78252 4.34126 2.94335 4.10330 2.81231 3.14677
2.42168 2.45000 3.64765 4.23498 4.37610 111 g - - -
2.68595 4.42250 2.77545 2.73148 3.46379 2.40512 3.72520
3.29260 2.67750 2.69265 4.24715 2.90351 2.73765 3.18172 2.89826
2.37889 2.77492 2.98544 4.58502 3.61528
0.14404 2.25879 3.51755 1.55397 0.23751 0.44562 1.02284
80 2.39843 5.38529 3.17419 2.61833 4.71571 3.04635 3.87902
3.28421 2.33755 3.68432 4.43013 2.56033 3.52465 2.26946 2.58562
2.33438 2.10994 3.76810 5.82707 4.42550 118 t - - -
2.68618 4.42225 2.77519 2.73123 3.46354 2.40513 3.72494
3.29354 2.67741 2.69355 4.24690 2.90347 2.73739 3.18146 2.89801
2.37887 2.77519 2.98518 4.58477 3.61503
0.03556 5.08086 3.55012 0.61958 0.77255 0.43954 1.03376
81 2.95167 4.35401 3.82333 4.14146 2.45853 4.10218 4.42673
1.61054 3.34589 1.67428 2.84040 4.20682 3.73863 4.12546 4.03082
3.40591 2.71663 2.29322 4.97404 3.79398 119 i - - -
2.68618 4.42225 2.77519 2.73123 3.46354 2.40513 3.72494
3.29354 2.67741 2.69355 4.24690 2.90347 2.73739 3.18146 2.89801
2.37887 2.77519 2.98518 4.58477 3.61503
0.00927 5.08192 5.80427 0.61958 0.77255 0.56141 0.84491

```
     82   2.72406  4.36469  4.68401  4.08361  2.43077  4.09275  3.67893
2.02637  3.91498  1.19600  3.47942  4.17612  4.45871  3.51290  4.00387
3.00922  2.92352  2.61214  4.98167  3.79925    120 l - - -
          2.68618  4.42225  2.77519  2.73123  3.46354  2.40513  3.72494
3.29354  2.67741  2.69355  4.24690  2.90347  2.73739  3.18146  2.89801
2.37887  2.77519  2.98518  4.58477  3.61503
          0.03360  5.08192  3.61802  0.61958  0.77255  0.44121  1.03075
     83   2.71299  5.24029  3.24653  2.68967  4.51692  1.95603  3.91737
3.95679  2.37527  2.95926  3.69494  2.69983  3.58471  3.04247  2.92351
2.12058  2.41861  3.24660  5.71748  4.35278    121 g - - -
          2.68618  4.42225  2.77519  2.73123  3.46354  2.40513  3.72494
3.29354  2.67741  2.69355  4.24690  2.90347  2.73739  3.18146  2.89801
2.37887  2.77519  2.98518  4.58477  3.61503
          0.06945  5.08378  2.79859  0.61958  0.77255  0.55613  0.85196
     84   2.06218  4.51800  3.45795  3.42414  2.40826  3.45809  4.21246
2.99169  3.35608  2.01432  3.62709  3.25340  4.31488  3.63120  3.65212
2.38309  2.64536  2.21263  5.11434  3.39940    122 l - - -
          2.68618  4.42225  2.77519  2.73123  3.46354  2.40513  3.72494
3.29354  2.67741  2.69355  4.24690  2.90347  2.73739  3.18146  2.89801
2.37887  2.77519  2.98518  4.58477  3.61503
          0.19649  5.02415  1.76133  0.61958  0.77255  0.45575  1.00505
     85   2.65145  4.28212  4.69393  3.50717  2.75438  4.04034  4.36715
2.05976  3.90899  1.36017  3.03045  3.39199  4.40346  4.06903  3.97268
3.34548  3.11684  1.98512  4.90734  3.72834    123 l - - -
          2.68618  4.42225  2.77519  2.73123  3.46354  2.40513  3.72494
3.29354  2.67741  2.69355  4.24690  2.90347  2.73739  3.18146  2.89801
2.37887  2.77519  2.98518  4.58477  3.61503
          0.09961  4.91078  2.43678  0.61958  0.77255  0.93045  0.50150
     86   2.29973  4.74983  3.41820  2.62501  3.44613  1.83638  3.93967
2.93724  2.83524  2.66593  3.84040  3.33228  3.50524  3.16634  2.95571
2.52181  3.04405  3.04591  5.30007  3.44370    124 g - - -
          2.68618  4.42225  2.77519  2.73123  3.46354  2.40513  3.72494
3.29354  2.67741  2.69355  4.24690  2.90347  2.73739  3.18146  2.89801
2.37887  2.77519  2.98518  4.58477  3.61503
          0.04637  4.82319  3.28957  0.61958  0.77255  1.06029  0.42519
     87   2.41172  4.59004  3.58174  2.76844  3.74247  2.73284  4.00201
2.74265  2.98277  2.59906  3.69398  3.07070  4.13613  3.29574  3.36174
2.34684  2.75166  1.77663  5.16744  3.92041    125 v - - -
          2.68618  4.42225  2.77519  2.73123  3.46354  2.40513  3.72494
3.29354  2.67741  2.69355  4.24690  2.90347  2.73739  3.18146  2.89801
2.37887  2.77519  2.98518  4.58477  3.61503
          0.04792  4.78925  3.25778  0.61958  0.77255  0.77950  0.61366
     88   2.81087  4.67127  4.00712  3.56517  4.33267  3.02863  4.52851
3.62302  3.50925  2.92474  4.27577  3.80296  0.82875  3.82958  3.82663
3.01141  2.76569  2.83085  5.74987  4.52875    126 p - - -
          2.68618  4.42225  2.77519  2.73123  3.46354  2.40513  3.72494
3.29354  2.67741  2.69355  4.24690  2.90347  2.73739  3.18146  2.89801
2.37887  2.77519  2.98518  4.58477  3.61503
          0.11204  4.85571  2.32066  0.61958  0.77255  0.25693  1.48466
     89   2.68905  4.60132  3.83649  3.26814  2.13432  2.43926  4.16074
2.65116  3.21796  2.81190  3.70746  2.89488  2.45019  3.51371  3.55896
2.51232  2.85387  2.66132  5.18838  3.44159    127 f - - -
          2.68616  4.42210  2.77601  2.73225  3.46296  2.40450  3.72596
3.29194  2.67802  2.69331  4.24693  2.90330  2.73747  3.18181  2.89903
2.37912  2.77418  2.98328  4.58579  3.61605
```

0.64771 0.74742 5.75336 1.58958 0.22817 0.37498 1.16252
90 1.87253 4.49955 4.20000 3.62190 3.61320 3.15611 4.30488
1.96141 3.10734 2.15052 3.61302 3.91066 3.68423 3.78727 3.78327
2.51167 2.69273 2.15464 5.10508 3.90190 146 a - - -
2.68618 4.42225 2.77519 2.73123 3.46354 2.40513 3.72494
3.29354 2.67741 2.69355 4.24690 2.90347 2.73739 3.18146 2.89801
2.37887 2.77519 2.98518 4.58477 3.61503
0.00903 5.10728 5.82962 0.61958 0.77255 0.48576 0.95510
91 2.18816 5.04092 3.42407 2.87563 4.30010 1.52966 4.03710
3.70401 2.86581 2.80619 3.58449 3.07249 4.19060 2.93208 3.31341
2.02882 3.18397 3.39612 5.60509 4.29309 147 g - - -
2.68618 4.42225 2.77519 2.73123 3.46354 2.40513 3.72494
3.29354 2.67741 2.69355 4.24690 2.90347 2.73739 3.18146 2.89801
2.37887 2.77519 2.98518 4.58477 3.61503
0.05644 5.10728 3.01943 0.61958 0.77255 0.48576 0.95510
92 1.92842 5.09711 3.06320 2.45356 3.76739 3.02381 3.94631
3.06091 2.52280 2.67438 4.16873 3.27544 4.13125 2.33718 3.21247
2.94509 2.35375 2.86085 5.60205 4.26948 148 a - - -
2.68618 4.42225 2.77519 2.73123 3.46354 2.40513 3.72494
3.29354 2.67741 2.69355 4.24690 2.90347 2.73739 3.18146 2.89801
2.37887 2.77519 2.98518 4.58477 3.61503
0.04717 5.06030 3.22563 0.61958 0.77255 0.50835 0.92001
93 2.25279 4.40116 4.56012 3.97035 3.50873 2.21346 4.41085
1.80825 3.82853 1.84049 3.51749 4.12414 4.45679 3.37965 3.96661
3.38451 3.19470 1.93576 5.03416 3.84748 149 i - - -
2.68618 4.42225 2.77519 2.73123 3.46354 2.40513 3.72494
3.29354 2.67741 2.69355 4.24690 2.90347 2.73739 3.18146 2.89801
2.37887 2.77519 2.98518 4.58477 3.61503
0.03380 5.04791 3.61892 0.61958 0.77255 0.48788 0.95173
94 2.17731 4.59602 2.97548 3.01518 3.30613 2.87688 4.18702
1.96351 3.26314 2.20694 3.70184 3.69857 4.30330 3.55453 3.17906
3.17676 2.45561 2.36148 5.18455 3.95926 150 i - - -
2.68618 4.42225 2.77519 2.73123 3.46354 2.40513 3.72494
3.29354 2.67741 2.69355 4.24690 2.90347 2.73739 3.18146 2.89801
2.37887 2.77519 2.98518 4.58477 3.61503
0.00946 5.06124 5.78359 0.61958 0.77255 0.61787 0.77455
95 2.42837 5.16913 2.82400 2.71312 3.66018 2.34821 3.92438
2.50872 2.54891 3.12696 4.23448 2.65124 4.11511 2.52199 3.17988
2.34262 2.92374 3.07871 5.65915 4.30938 151 s - - -
2.68618 4.42225 2.77519 2.73123 3.46354 2.40513 3.72494
3.29354 2.67741 2.69355 4.24690 2.90347 2.73739 3.18146 2.89801
2.37887 2.77519 2.98518 4.58477 3.61503
0.00946 5.06124 5.78359 0.61958 0.77255 0.50844 0.91988
96 2.94857 4.38940 4.55446 3.28691 2.44881 3.53620 4.38614
1.56526 3.81702 1.81737 3.50526 4.10959 4.44087 3.37824 3.94969
2.43395 3.18071 2.34003 5.00492 3.81870 152 i - - -
2.68618 4.42225 2.77519 2.73123 3.46354 2.40513 3.72494
3.29354 2.67741 2.69355 4.24690 2.90347 2.73739 3.18146 2.89801
2.37887 2.77519 2.98518 4.58477 3.61503
0.00924 5.08470 5.80704 0.61958 0.77255 0.44562 1.02284
97 2.27524 4.36239 4.93948 4.32668 2.07193 4.15669 4.47872
1.80649 4.11424 1.84683 3.46594 4.31374 4.51807 4.24411 4.12496
3.46819 3.22005 2.31050 3.28669 2.53716 153 i - - -

```
         2.68618  4.42225  2.77519  2.73123  3.46354  2.40513  3.72494
3.29354  2.67741  2.69355  4.24690  2.90347  2.73739  3.18146  2.89801
2.37887  2.77519  2.98518  4.58477  3.61503
         0.00903  5.10728  5.82962  0.61958  0.77255  0.48576  0.95510
     98  2.31793  5.38443  2.72554  2.63824  4.70876  2.03492  3.89830
4.17991  2.46758  2.72295  4.43225  2.94442  4.10082  2.51782  3.13223
2.05871  2.76085  3.76423  5.83137  4.43353    154 g - - -
         2.68618  4.42225  2.77519  2.73123  3.46354  2.40513  3.72494
3.29354  2.67741  2.69355  4.24690  2.90347  2.73739  3.18146  2.89801
2.37887  2.77519  2.98518  4.58477  3.61503
         0.00903  5.10728  5.82962  0.61958  0.77255  0.48576  0.95510
     99  2.31087  5.07210  3.06391  2.60029  2.73604  2.90447  3.98321
3.30639  2.36911  3.04508  4.14757  3.32208  4.16403  3.14712  2.99371
2.38566  2.17797  2.60431  5.58625  4.26457    155 t - - -
         2.68618  4.42225  2.77519  2.73123  3.46354  2.40513  3.72494
3.29354  2.67741  2.69355  4.24690  2.90347  2.73739  3.18146  2.89801
2.37887  2.77519  2.98518  4.58477  3.61503
         0.00903  5.10728  5.82962  0.61958  0.77255  0.48576  0.95510
    100  2.60953  4.43685  4.95315  4.35025  3.20120  4.24303  4.58685
1.65572  4.15933  1.16674  3.53124  4.38027  4.60008  4.30673  4.20040
3.03816  3.01095  2.22596  5.09546  3.91941    156 l - - -
         2.68618  4.42225  2.77519  2.73123  3.46354  2.40513  3.72494
3.29354  2.67741  2.69355  4.24690  2.90347  2.73739  3.18146  2.89801
2.37887  2.77519  2.98518  4.58477  3.61503
         0.09053  5.10728  2.51954  0.61958  0.77255  0.48576  0.95510
    101  2.29224  4.34395  4.83568  4.22718  2.58659  4.12134  4.45178
2.04042  3.30369  1.41517  3.03295  4.25467  4.48374  4.17909  4.07145
3.42961  2.68009  2.16980  4.96922  3.79224    157 l - - -
         2.68618  4.42225  2.77519  2.73123  3.46354  2.40513  3.72494
3.29354  2.67741  2.69355  4.24690  2.90347  2.73739  3.18146  2.89801
2.37887  2.77519  2.98518  4.58477  3.61503
         0.00948  5.05890  5.78124  0.61958  0.77255  0.70366  0.68274
    102  2.92731  5.41943  2.33766  2.35103  3.69038  2.36991  3.88808
4.23526  2.63460  3.72088  4.46489  2.42121  2.00855  2.98937  2.89062
2.51024  3.16005  3.80506  5.85825  4.44976    158 p - - -
         2.68618  4.42225  2.77519  2.73123  3.46354  2.40513  3.72494
3.29354  2.67741  2.69355  4.24690  2.90347  2.73739  3.18146  2.89801
2.37887  2.77519  2.98518  4.58477  3.61503
         0.00948  5.05890  5.78124  0.61958  0.77255  0.70366  0.68274
    103  2.74679  3.51806  4.81483  4.20533  2.19670  4.09699  4.42604
1.84494  3.50756  1.88245  3.44353  4.23074  4.46148  3.26701  4.04756
3.40452  2.35036  2.14235  4.94595  3.30940    159 i - - -
         2.68618  4.42225  2.77519  2.73123  3.46354  2.40513  3.72494
3.29354  2.67741  2.69355  4.24690  2.90347  2.73739  3.18146  2.89801
2.37887  2.77519  2.98518  4.58477  3.61503
         0.00948  5.05890  5.78124  0.61958  0.77255  0.37025  1.17300
    104  3.00300  4.39367  4.85409  4.24659  3.10694  4.15966  4.47669
1.81422  3.33211  1.10156  3.49396  4.28276  4.52190  4.20305  3.46475
3.46677  3.23478  2.63667  3.33177  3.38313    160 l - - -
         2.68618  4.42225  2.77519  2.73123  3.46354  2.40513  3.72494
3.29354  2.67741  2.69355  4.24690  2.90347  2.73739  3.18146  2.89801
2.37887  2.77519  2.98518  4.58477  3.61503
         0.00877  5.13716  5.85950  0.61958  0.77255  0.48576  0.95510
```

    105   2.78855 4.63294 4.21854 3.66027 2.94690 4.04199 4.34507
3.14646 3.52896 2.86826 3.78479 3.94811 3.25720 3.82962 3.28811
2.97641 3.28914 2.93372 0.98312 3.86326   161 w - - -
          2.68618 4.42225 2.77519 2.73123 3.46354 2.40513 3.72494
3.29354 2.67741 2.69355 4.24690 2.90347 2.73739 3.18146 2.89801
2.37887 2.77519 2.98518 4.58477 3.61503
          0.11645 5.13716 2.26283 0.61958 0.77255 0.48576 0.95510
    106   2.49544 5.27241 3.20132 2.48847 4.57293 3.33257 3.93090
4.01638 2.39051 3.19951 4.35068 2.93652 1.31667 3.05572 3.16720
2.93154 2.91058 3.64404 5.75948 4.39144   162 p - - -
          2.68618 4.42225 2.77519 2.73123 3.46354 2.40513 3.72494
3.29354 2.67741 2.69355 4.24690 2.90347 2.73739 3.18146 2.89801
2.37887 2.77519 2.98518 4.58477 3.61503
          0.03767 5.03046 3.49208 0.61958 0.77255 0.76892 0.62271
    107   2.28534 5.37328 2.40797 2.03007 4.71229 3.28181 3.84072
3.66697 2.32407 3.67598 3.64121 2.69928 4.04632 2.93903 2.86181
2.31527 2.33074 3.76067 5.80962 4.40232   163 e - - -
          2.68618 4.42225 2.77519 2.73123 3.46354 2.40513 3.72494
3.29354 2.67741 2.69355 4.24690 2.90347 2.73739 3.18146 2.89801
2.37887 2.77519 2.98518 4.58477 3.61503
          0.01003 5.00282 5.72516 0.61958 0.77255 0.70575 0.68071
    108   2.88993 5.37140 2.43217 2.45557 3.50428 2.43796 3.85643
3.29633 2.48363 3.67145 4.41537 2.55933 2.55874 2.34624 3.08722
2.53468 2.88949 3.75545 5.81145 4.40781   164 q - - -
          2.68618 4.42225 2.77519 2.73123 3.46354 2.40513 3.72494
3.29354 2.67741 2.69355 4.24690 2.90347 2.73739 3.18146 2.89801
2.37887 2.77519 2.98518 4.58477 3.61503
          0.00976 5.03046 5.75280 0.61958 0.77255 0.76892 0.62271
    109   2.18264 5.40077 2.40038 2.11326 4.74249 2.52702 3.85689
4.22678 2.34345 3.70470 4.44280 2.39979 4.06167 2.95475 3.08831
2.38730 2.68265 3.78970 5.83529 3.76827   165 e - - -
          2.68618 4.42225 2.77519 2.73123 3.46354 2.40513 3.72494
3.29354 2.67741 2.69355 4.24690 2.90347 2.73739 3.18146 2.89801
2.37887 2.77519 2.98518 4.58477 3.61503
          0.29195 5.03046 1.39975 0.61958 0.77255 0.34233 1.23826
    110   2.45309 5.34819 2.32811 2.12788 4.69142 2.61549 3.79954
4.17686 2.53756 3.65279 4.38993 2.35150 3.48694 2.71083 2.78132
2.44612 2.59270 3.73796 5.78182 4.37062   166 e - - -
          2.68625 4.42266 2.77452 2.73148 3.46395 2.40517 3.72494
3.29395 2.67758 2.69396 4.24731 2.90322 2.73637 3.18135 2.89842
2.37873 2.77524 2.98539 4.58518 3.61484
          0.82205 0.78060 2.27944 0.87839 0.53691 0.56005 0.84672
    111   2.85399 5.36311 2.48350 2.40018 4.70699 3.62411 3.81400
4.19252 1.97845 3.66782 4.40440 2.37506 3.08916 2.06824 3.04334
2.46037 2.68298 3.41907 5.79622 4.38528   174 k - - -
          2.68618 4.42225 2.77519 2.73123 3.46354 2.40513 3.72494
3.29354 2.67741 2.69355 4.24690 2.90347 2.73739 3.18146 2.89801
2.37887 2.77519 2.98518 4.58477 3.61503
          0.01076 4.93313 5.65548 0.61958 0.77255 0.27569 1.42317
    112   2.61678 5.39870 2.49841 2.23837 3.40558 3.32467 3.90604
3.63301 2.39653 3.08897 4.44452 2.75703 3.15552 3.00896 2.74700
2.25050 2.44567 3.38457 5.84322 3.85505   175 e - - -
          2.68618 4.42225 2.77519 2.73123 3.46354 2.40513 3.72494
3.29354 2.67741 2.69355 4.24690 2.90347 2.73739 3.18146 2.89801
2.37887 2.77519 2.98518 4.58477 3.61503

```
          0.00877  5.13716  5.85950  0.61958  0.77255  0.48576  0.95510
    113   2.96424  4.58796  4.02014  3.44781  3.01165  3.98726  4.25720
2.01620  3.07263  2.58885  3.69614  3.35915  3.38799  2.50225  3.68836
2.68226  2.15763  1.94407  5.18235  3.96561    176 v - - -
          2.68618  4.42225  2.77519  2.73123  3.46354  2.40513  3.72494
3.29354  2.67741  2.69355  4.24690  2.90347  2.73739  3.18146  2.89801
2.37887  2.77519  2.98518  4.58477  3.61503
          0.05936  5.13716  2.96125  0.61958  0.77255  0.48576  0.95510
    114   2.97739  4.47915  3.69748  3.76538  2.80216  3.45822  4.32929
2.68650  3.65913  2.43376  3.59438  4.00142  4.43058  3.89136  3.86665
2.78486  3.21063  2.73642  1.15789  3.77985    177 w - - -
          2.68618  4.42225  2.77519  2.73123  3.46354  2.40513  3.72494
3.29354  2.67741  2.69355  4.24690  2.90347  2.73739  3.18146  2.89801
2.37887  2.77519  2.98518  4.58477  3.61503
          0.00922  5.08702  5.80936  0.61958  0.77255  0.40381  1.10194
    115   2.76718  5.44478  2.61865  2.10995  4.78930  2.87029  3.89623
3.67537  2.09558  3.74983  4.48563  2.31865  4.10248  2.46242  3.12408
2.16169  2.74745  3.42345  5.87777  4.46706    178 k - - -
          2.68618  4.42225  2.77519  2.73123  3.46354  2.40513  3.72494
3.29354  2.67741  2.69355  4.24690  2.90347  2.73739  3.18146  2.89801
2.37887  2.77519  2.98518  4.58477  3.61503
          0.00877  5.13716  5.85950  0.61958  0.77255  0.48576  0.95510
    116   2.25257  5.41401  2.80643  1.71987  4.74553  3.37783  3.90322
3.73035  2.50007  2.50958  4.45829  2.95036  4.10778  2.33433  3.13401
2.40596  3.16713  3.41232  5.85487  4.45216    179 e - - -
          2.68618  4.42225  2.77519  2.73123  3.46354  2.40513  3.72494
3.29354  2.67741  2.69355  4.24690  2.90347  2.73739  3.18146  2.89801
2.37887  2.77519  2.98518  4.58477  3.61503
          0.00877  5.13716  5.85950  0.61958  0.77255  0.48576  0.95510
    117   2.98163  3.55733  4.88029  3.44012  1.74090  4.14436  4.47470
2.47414  4.06888  1.59138  2.42706  4.28518  4.50827  4.21216  4.10013
2.86011  2.90631  2.42862  4.98746  3.81024    180 l - - -
          2.68618  4.42225  2.77519  2.73123  3.46354  2.40513  3.72494
3.29354  2.67741  2.69355  4.24690  2.90347  2.73739  3.18146  2.89801
2.37887  2.77519  2.98518  4.58477  3.61503
          0.00877  5.13716  5.85950  0.61958  0.77255  0.48576  0.95510
    118   2.74590  4.67421  5.22543  4.63642  3.69912  4.55840  4.92552
1.25345  4.46024  1.93685  1.56746  4.69384  4.88377  4.59880  4.50927
3.88385  2.83629  2.48856  5.38987  4.23258    181 i - - -
          2.68618  4.42225  2.77519  2.73123  3.46354  2.40513  3.72494
3.29354  2.67741  2.69355  4.24690  2.90347  2.73739  3.18146  2.89801
2.37887  2.77519  2.98518  4.58477  3.61503
          0.00877  5.13716  5.85950  0.61958  0.77255  0.48576  0.95510
    119   2.45338  4.18453  2.77774  2.18764  4.76273  3.02275  3.89980
3.33361  1.97358  3.72767  4.46875  2.60263  4.10516  2.99883  2.23183
2.90116  2.43377  3.44262  5.86356  4.45766    182 k - - -
          2.68618  4.42225  2.77519  2.73123  3.46354  2.40513  3.72494
3.29354  2.67741  2.69355  4.24690  2.90347  2.73739  3.18146  2.89801
2.37887  2.77519  2.98518  4.58477  3.61503
          0.00877  5.13716  5.85950  0.61958  0.77255  0.48576  0.95510
    120   2.23787  5.41408  2.41638  2.27859  3.82031  3.71399  2.34414
3.66224  2.64244  3.71373  3.77085  2.94342  4.10715  2.21992  2.69820
2.74135  2.93487  3.39779  5.85473  4.45186    183 q - - -
```

```
          2.68618  4.42225  2.77519  2.73123  3.46354  2.40513  3.72494
 3.29354  2.67741  2.69355  4.24690  2.90347  2.73739  3.18146  2.89801
 2.37887  2.77519  2.98518  4.58477  3.61503
          0.00877  5.13716  5.85950  0.61958  0.77255  0.48576  0.95510
    121   2.38724  4.47087  4.39098  3.39751  3.58163  2.47900  4.37766
 2.29411  3.69680  2.32756  3.58898  4.03526  4.44402  3.92568  3.89494
 3.04250  2.27021  1.47512  5.08781  3.89334    184 v - - -
          2.68618  4.42225  2.77519  2.73123  3.46354  2.40513  3.72494
 3.29354  2.67741  2.69355  4.24690  2.90347  2.73739  3.18146  2.89801
 2.37887  2.77519  2.98518  4.58477  3.61503
          0.00877  5.13716  5.85950  0.61958  0.77255  0.48576  0.95510
    122   2.65152  5.47437  3.21765  1.05861  4.81495  3.75505  3.93982
 4.28755  2.37978  3.07386  4.52034  2.96712  4.15387  2.59206  2.88496
 2.96391  3.22804  3.86348  5.90204  4.50797    185 e - - -
          2.68618  4.42225  2.77519  2.73123  3.46354  2.40513  3.72494
 3.29354  2.67741  2.69355  4.24690  2.90347  2.73739  3.18146  2.89801
 2.37887  2.77519  2.98518  4.58477  3.61503
          0.00877  5.13716  5.85950  0.61958  0.77255  0.48576  0.95510
    123   2.61103  5.43282  2.76605  1.80035  4.77258  3.00681  3.89816
 4.25483  2.63492  3.35770  3.74409  2.48017  4.10390  2.36657  2.70231
 2.53583  2.73658  3.17323  5.86870  3.83197    186 e - - -
          2.68618  4.42225  2.77519  2.73123  3.46354  2.40513  3.72494
 3.29354  2.67741  2.69355  4.24690  2.90347  2.73739  3.18146  2.89801
 2.37887  2.77519  2.98518  4.58477  3.61503
          0.00877  5.13716  5.85950  0.61958  0.77255  0.48576  0.95510
    124   4.00452  5.27025  5.91094  5.31822  3.66348  5.30797  5.61419
 1.81189  5.15419  0.56558  2.59828  5.45707  3.81164  5.10572  5.12800
 4.66762  4.21585  3.01560  5.76527  4.76232    187 L - - -
          2.68618  4.42225  2.77519  2.73123  3.46354  2.40513  3.72494
 3.29354  2.67741  2.69355  4.24690  2.90347  2.73739  3.18146  2.89801
 2.37887  2.77519  2.98518  4.58477  3.61503
          0.00877  5.13716  5.85950  0.61958  0.77255  0.48576  0.95510
    125   3.16545  4.51904  5.13364  4.52887  2.87414  4.37096  4.71857
 1.12522  4.32686  2.08226  2.86672  4.53094  4.71400  4.45300  4.34116
 3.68987  2.98938  1.73144  5.18612  3.48875    188 i - - -
          2.68618  4.42225  2.77519  2.73123  3.46354  2.40513  3.72494
 3.29354  2.67741  2.69355  4.24690  2.90347  2.73739  3.18146  2.89801
 2.37887  2.77519  2.98518  4.58477  3.61503
          0.03069  5.13716  3.71528  0.61958  0.77255  0.48576  0.95510
    126   2.54178  5.44058  1.78156  2.62088  4.78449  3.26703  3.89149
 4.26994  2.25543  3.74524  4.48189  2.11891  4.09712  2.44529  2.71469
 2.57118  3.16303  3.83066  5.87361  3.80691    189 d - - -
          2.68618  4.42225  2.77519  2.73123  3.46354  2.40513  3.72494
 3.29354  2.67741  2.69355  4.24690  2.90347  2.73739  3.18146  2.89801
 2.37887  2.77519  2.98518  4.58477  3.61503
          0.00896  5.11543  5.83777  0.61958  0.77255  0.44591  1.02232
    127   2.93717  5.42773  3.19340  2.06962  3.82175  3.71354  3.90087
 4.24495  1.83936  3.72934  4.47063  3.18421  3.59787  1.80994  2.90256
 2.44942  2.52299  3.44804  5.86480  4.45941    190 q - - -
          2.68618  4.42225  2.77519  2.73123  3.46354  2.40513  3.72494
 3.29354  2.67741  2.69355  4.24690  2.90347  2.73739  3.18146  2.89801
 2.37887  2.77519  2.98518  4.58477  3.61503
          0.00877  5.13716  5.85950  0.61958  0.77255  0.48576  0.95510
```

```
    128   2.41365  5.44167  2.90583  1.92391  4.01773  3.34513  3.89634
4.26998  1.75331  3.24862  4.48276  2.90650  3.59283  2.79465  2.47715
2.89786  2.57532  3.83146  5.87528  4.46532    191 k - - -
          2.68612  4.42243  2.77537  2.73141  3.46371  2.40530  3.72512
3.29328  2.67758  2.69372  4.24595  2.90335  2.73757  3.18048  2.89818
2.37887  2.77511  2.98504  4.58494  3.61461
          0.04360  3.22356  5.85950  1.87317  0.16681  0.48576  0.95510
    129   3.00786  4.42090  4.73022  4.13032  3.51477  4.15328  4.47414
1.21671  3.28647  1.68294  3.09332  3.64236  4.51673  3.59452  4.05058
3.45487  3.24002  2.19005  5.04696  3.86567    203 i - - -
          2.68618  4.42225  2.77519  2.73123  3.46354  2.40513  3.72494
3.29354  2.67741  2.69355  4.24690  2.90347  2.73739  3.18146  2.89801
2.37887  2.77519  2.98518  4.58477  3.61503
          0.00877  5.13716  5.85950  0.61958  0.77255  0.48576  0.95510
    130   2.28800  5.42038  2.62367  1.76724  4.75474  3.34354  3.90137
3.66964  2.37673  3.34874  4.46390  3.18466  3.19779  3.00124  3.13141
1.99148  2.76367  3.80526  5.85954  3.84624    204 e - - -
          2.68618  4.42225  2.77519  2.73123  3.46354  2.40513  3.72494
3.29354  2.67741  2.69355  4.24690  2.90347  2.73739  3.18146  2.89801
2.37887  2.77519  2.98518  4.58477  3.61503
          0.00877  5.13716  5.85950  0.61958  0.77255  0.48576  0.95510
    131   2.71708  5.44748  2.61179  1.69497  4.79293  3.35186  3.46402
4.27924  2.36643  3.36811  4.48809  2.76066  4.10244  2.16898  2.90252
2.73014  2.35722  3.83833  5.87986  4.46856    205 e - - -
          2.68618  4.42225  2.77519  2.73123  3.46354  2.40513  3.72494
3.29354  2.67741  2.69355  4.24690  2.90347  2.73739  3.18146  2.89801
2.37887  2.77519  2.98518  4.58477  3.61503
          0.00877  5.13716  5.85950  0.61958  0.77255  0.48576  0.95510
    132   2.45587  5.15096  2.65345  2.49393  3.38596  3.77162  3.97498
3.01207  2.77324  2.88528  4.22094  3.30034  4.16153  2.26980  3.01220
2.43902  2.74847  3.48397  5.65200  2.23330    206 y - - -
          2.68618  4.42225  2.77519  2.73123  3.46354  2.40513  3.72494
3.29354  2.67741  2.69355  4.24690  2.90347  2.73739  3.18146  2.89801
2.37887  2.77519  2.98518  4.58477  3.61503
          0.00877  5.13716  5.85950  0.61958  0.77255  0.48576  0.95510
    133   1.85094  4.41683  4.57524  3.98158  3.51628  4.09846  3.67196
2.63585  3.09514  2.00048  3.16920  4.13260  4.46612  4.03579  3.97306
3.39213  2.68087  1.53027  5.03201  3.06301    207 v - - -
          2.68619  4.42226  2.77521  2.73124  3.46355  2.40514  3.72495
3.29355  2.67742  2.69356  4.24691  2.90348  2.73741  3.18147  2.89802
2.37888  2.77521  2.98496  4.58478  3.61504
          0.03376  3.49520  5.85950  0.39967  1.11030  0.48576  0.95510
    134   2.96615  4.64490  3.91538  3.34470  3.33140  3.96293  4.22080
2.15369  2.17515  2.51558  3.34383  3.72953  4.34050  3.10380  1.72585
3.21362  3.19761  2.53933  5.23129  3.14162    209 r - - -
          2.68618  4.42225  2.77519  2.73123  3.46354  2.40513  3.72494
3.29354  2.67741  2.69355  4.24690  2.90347  2.73739  3.18146  2.89801
2.37887  2.77519  2.98518  4.58477  3.61503
          0.00877  5.13716  5.85950  0.61958  0.77255  0.48576  0.95510
    135   2.93729  5.44209  2.37578  2.05887  4.78441  3.06965  3.39240
3.01132  2.49293  3.74619  4.48358  2.12231  4.10461  2.99623  2.92634
2.14073  2.60992  3.83127  5.87631  4.46666    210 e - - -
          2.68618  4.42225  2.77519  2.73123  3.46354  2.40513  3.72494
3.29354  2.67741  2.69355  4.24690  2.90347  2.73739  3.18146  2.89801
2.37887  2.77519  2.98518  4.58477  3.61503
```

0.00877 5.13716 5.85950 0.61958 0.77255 0.48576 0.95510
136 2.58641 5.42229 2.76490 2.24754 4.75758 3.03170 3.90063
2.83877 1.97733 3.06769 4.46553 2.33704 4.10580 2.81907 2.68897
2.90197 2.62948 3.28482 5.86086 4.45590 211 k - - -
2.68618 4.42225 2.77519 2.73123 3.46354 2.40513 3.72494
3.29354 2.67741 2.69355 4.24690 2.90347 2.73739 3.18146 2.89801
2.37887 2.77519 2.98518 4.58477 3.61503
0.00877 5.13716 5.85950 0.61958 0.77255 0.48576 0.95510
137 0.74211 4.74598 4.80817 4.28216 3.97050 4.26827 4.89526
2.34213 4.15747 2.18653 3.89746 4.43386 4.74635 4.39930 4.34717
3.62702 3.01891 2.63737 5.60286 4.41938 212 a - - -
2.68618 4.42225 2.77519 2.73123 3.46354 2.40513 3.72494
3.29354 2.67741 2.69355 4.24690 2.90347 2.73739 3.18146 2.89801
2.37887 2.77519 2.98518 4.58477 3.61503
0.00877 5.13716 5.85950 0.61958 0.77255 0.48576 0.95510
138 2.74864 4.57804 4.04236 2.87448 2.94455 3.03755 4.26441
2.52903 3.09760 1.80725 3.24657 2.59771 4.36815 3.67894 3.70144
2.76062 2.41046 2.49971 5.17361 3.46386 213 l - - -
2.68618 4.42225 2.77519 2.73123 3.46354 2.40513 3.72494
3.29354 2.67741 2.69355 4.24690 2.90347 2.73739 3.18146 2.89801
2.37887 2.77519 2.98518 4.58477 3.61503
0.00877 5.13716 5.85950 0.61958 0.77255 0.48576 0.95510
139 1.93623 5.41249 2.81109 2.64313 4.74339 2.85193 3.90526
4.21923 2.36003 2.94175 4.45772 2.76123 4.10932 2.59033 3.13664
2.04778 2.34460 3.79567 5.85458 4.45250 214 a - - -
2.68618 4.42225 2.77519 2.73123 3.46354 2.40513 3.72494
3.29354 2.67741 2.69355 4.24690 2.90347 2.73739 3.18146 2.89801
2.37887 2.77519 2.98518 4.58477 3.61503
0.00877 5.13716 5.85950 0.61958 0.77255 0.48576 0.95510
140 2.45935 5.44246 2.62444 1.89768 3.74812 3.70891 3.46653
3.72915 1.97372 3.74721 4.48344 2.74270 4.10237 2.75914 2.19896
2.71945 3.16588 3.83245 5.87585 3.77587 215 e - - -
2.68618 4.42225 2.77519 2.73123 3.46354 2.40513 3.72494
3.29354 2.67741 2.69355 4.24690 2.90347 2.73739 3.18146 2.89801
2.37887 2.77519 2.98518 4.58477 3.61503
0.00877 5.13716 5.85950 0.61958 0.77255 0.48576 0.95510
141 2.79782 4.42426 4.97726 4.36584 2.39012 4.21986 4.54703
2.25250 4.16020 0.97218 3.47742 4.37026 4.57511 4.28980 4.17864
3.09561 3.28077 2.46754 3.91443 3.85840 216 l - - -
2.68618 4.42225 2.77519 2.73123 3.46354 2.40513 3.72494
3.29354 2.67741 2.69355 4.24690 2.90347 2.73739 3.18146 2.89801
2.37887 2.77519 2.98518 4.58477 3.61503
0.00877 5.13716 5.85950 0.61958 0.77255 0.48576 0.95510
142 2.93486 5.44527 2.32722 1.92873 4.79027 3.70853 3.89562
4.27616 2.28753 3.36668 3.86405 2.22029 4.10205 2.36141 2.58528
2.65361 2.76580 3.83587 5.87796 3.76762 217 e - - -
2.68618 4.42225 2.77519 2.73123 3.46354 2.40513 3.72494
3.29354 2.67741 2.69355 4.24690 2.90347 2.73739 3.18146 2.89801
2.37887 2.77519 2.98518 4.58477 3.61503
0.00877 5.13716 5.85950 0.61958 0.77255 0.48576 0.95510
143 2.61695 5.51756 2.46804 2.74082 4.94114 1.33375 4.08840
4.42374 2.88243 3.92246 4.68283 2.61905 4.23149 3.20487 3.07111
1.90244 3.34461 3.98134 6.07009 4.65920 218 g - - -

```
          2.68622  4.42229  2.77524  2.73110  3.46358  2.40517  3.72499
3.29358  2.67745  2.69359  4.24694  2.90330  2.73744  3.18151  2.89805
2.37879  2.77524  2.98523  4.58481  3.61465
          0.03355  3.50189  5.85950  1.01861  0.44800  0.48576  0.95510
    144   3.38947  4.72442  5.35085  4.74817  2.79861  4.61474  4.95465
1.70906  4.55505  0.93426  3.09204  4.77395  4.91707  4.64237  4.55957
3.39180  3.61803  2.11554  5.35474  4.22307    223 l - - -
          2.68618  4.42225  2.77519  2.73123  3.46354  2.40513  3.72494
3.29354  2.67741  2.69355  4.24690  2.90347  2.73739  3.18146  2.89801
2.37887  2.77519  2.98518  4.58477  3.61503
          0.00877  5.13716  5.85950  0.61958  0.77255  0.48576  0.95510
    145   2.93596  5.39849  3.20403  2.05422  3.96454  2.58297  3.19598
4.19621  2.09012  3.33259  4.44437  3.19272  4.11027  2.27602  2.69294
2.57454  3.16710  2.83753  5.84302  3.11537    224 e - - -
          2.68618  4.42225  2.77519  2.73123  3.46354  2.40513  3.72494
3.29354  2.67741  2.69355  4.24690  2.90347  2.73739  3.18146  2.89801
2.37887  2.77519  2.98518  4.58477  3.61503
          0.00877  5.13716  5.85950  0.61958  0.77255  0.48576  0.95510
    146   2.46819  5.41862  2.37149  2.49826  4.75238  3.37734  3.90147
3.25241  2.50305  3.29018  4.46227  2.00688  4.10641  2.80238  2.43783
2.28786  3.16635  3.38891  5.85813  3.73615    225 n - - -
          2.68618  4.42225  2.77519  2.73123  3.46354  2.40513  3.72494
3.29354  2.67741  2.69355  4.24690  2.90347  2.73739  3.18146  2.89801
2.37887  2.77519  2.98518  4.58477  3.61503
          0.00877  5.13716  5.85950  0.61958  0.77255  0.48576  0.95510
    147   2.10225  4.97817  3.46920  2.67974  4.17336  3.38484  4.03823
2.96937  2.52083  2.48343  3.55071  2.29721  4.20813  2.77010  3.33287
2.65176  3.17885  2.25002  5.51257  3.68666    226 a - - -
          2.68618  4.42225  2.77519  2.73123  3.46354  2.40513  3.72494
3.29354  2.67741  2.69355  4.24690  2.90347  2.73739  3.18146  2.89801
2.37887  2.77519  2.98518  4.58477  3.61503
          0.00877  5.13716  5.85950  0.61958  0.77255  0.48576  0.95510
    148   2.98828  4.36677  4.92265  4.30951  2.49980  3.55141  4.48634
2.44832  4.10102  1.47008  2.45480  4.30784  4.51782  4.23657  3.52738
3.46541  3.22032  2.04464  4.98617  2.30550    227 l - - -
          2.68618  4.42225  2.77519  2.73123  3.46354  2.40513  3.72494
3.29354  2.67741  2.69355  4.24690  2.90347  2.73739  3.18146  2.89801
2.37887  2.77519  2.98518  4.58477  3.61503
          0.00877  5.13716  5.85950  0.61958  0.77255  0.48576  0.95510
    149   2.74675  5.44610  2.36378  2.20358  4.79142  3.38415  3.89548
4.27756  2.13297  3.75147  4.48671  2.29383  4.10191  2.47418  2.33358
2.57397  2.93606  3.11620  5.87861  3.79847    228 k - - -
          2.68618  4.42225  2.77519  2.73123  3.46354  2.40513  3.72494
3.29354  2.67741  2.69355  4.24690  2.90347  2.73739  3.18146  2.89801
2.37887  2.77519  2.98518  4.58477  3.61503
          0.00877  5.13716  5.85950  0.61958  0.77255  0.48576  0.95510
    150   2.71260  5.31781  2.03868  2.41281  4.61234  3.73299  3.92625
3.26239  2.55518  2.84429  4.37212  2.77709  3.65498  2.81202  2.93339
2.73833  2.95201  2.71080  5.78199  2.51620    229 d - - -
          2.68618  4.42225  2.77519  2.73123  3.46354  2.40513  3.72494
3.29354  2.67741  2.69355  4.24690  2.90347  2.73739  3.18146  2.89801
2.37887  2.77519  2.98518  4.58477  3.61503
          0.00877  5.13716  5.85950  0.61958  0.77255  0.48576  0.95510
```

```
    151   5.35360  6.17544  6.04544  6.07149  1.61858  5.70264  4.25607
4.86843  5.83661  3.93936  5.36382  5.20095  5.94849  5.40202  5.57520
5.13664  5.53894  4.84858  4.32074  0.37003    230 Y - - -
          2.68620  4.42227  2.77522  2.73125  3.46356  2.40515  3.72497
3.29356  2.67743  2.69338  4.24692  2.90349  2.73742  3.18149  2.89803
2.37889  2.77522  2.98521  4.58479  3.61463
          0.06189  2.86181  5.85950  0.28861  1.38353  0.48576  0.95510
    152   2.76452  5.42105  2.96538  1.98491  4.75583  3.71278  3.08908
3.66846  2.49607  2.43622  4.46443  2.44740  4.10601  2.30612  2.57211
2.70139  3.16631  3.46542  5.85993  3.33092    232 e - - -
          2.68618  4.42225  2.77519  2.73123  3.46354  2.40513  3.72494
3.29354  2.67741  2.69355  4.24690  2.90347  2.73739  3.18146  2.89801
2.37887  2.77519  2.98518  4.58477  3.61503
          0.00877  5.13716  5.85950  0.61958  0.77255  0.48576  0.95510
    153   2.60513  5.44451  2.97958  2.18729  4.78923  3.70869  3.33690
4.27489  2.05681  3.33028  3.77661  2.05655  4.10215  2.65954  2.46257
2.29031  2.93432  3.83497  5.87735  4.46670    233 n - - -
          2.68618  4.42225  2.77519  2.73123  3.46354  2.40513  3.72494
3.29354  2.67741  2.69355  4.24690  2.90347  2.73739  3.18146  2.89801
2.37887  2.77519  2.98518  4.58477  3.61503
          0.00877  5.13716  5.85950  0.61958  0.77255  0.48576  0.95510
    154   1.35701  4.86046  3.59555  3.03417  3.19461  3.86616  3.59312
3.40939  2.45649  3.08844  3.95482  3.50089  4.25163  3.33688  3.41849
2.04603  3.18817  2.91325  5.41138  3.11638    234 a - - -
          2.68618  4.42225  2.77519  2.73123  3.46354  2.40513  3.72494
3.29354  2.67741  2.69355  4.24690  2.90347  2.73739  3.18146  2.89801
2.37887  2.77519  2.98518  4.58477  3.61503
          0.03657  5.13716  3.50547  0.61958  0.77255  0.48576  0.95510
    155   2.93407  4.63043  4.95215  4.35739  1.48766  4.39266  4.68894
2.60111  3.03918  1.11917  3.49681  4.46750  4.72459  4.33806  4.25682
3.70260  3.46508  2.75513  5.19165  4.00957    235 l - - -
          2.68618  4.42225  2.77519  2.73123  3.46354  2.40513  3.72494
3.29354  2.67741  2.69355  4.24690  2.90347  2.73739  3.18146  2.89801
2.37887  2.77519  2.98518  4.58477  3.61503
          0.00901  5.10960  5.83195  0.61958  0.77255  0.43646  1.03936
    156   2.48905  4.18377  2.04871  1.76422  4.79346  3.70995  3.89828
4.27935  2.26743  3.75371  4.48955  2.32816  4.10440  2.78860  2.54489
2.90026  2.96220  3.83918  5.88126  4.47025    236 e - - -
          2.68618  4.42225  2.77519  2.73123  3.46354  2.40513  3.72494
3.29354  2.67741  2.69355  4.24690  2.90347  2.73739  3.18146  2.89801
2.37887  2.77519  2.98518  4.58477  3.61503
          0.00877  5.13716  5.85950  0.61958  0.77255  0.48576  0.95510
    157   2.93515  5.42083  1.91589  2.25845  3.48858  3.71279  3.46609
3.66899  2.63983  3.72167  4.46423  2.24671  4.10604  2.79033  2.66346
2.32980  2.76361  3.41858  5.85979  3.78809    237 d - - -
          2.68618  4.42225  2.77519  2.73123  3.46354  2.40513  3.72494
3.29354  2.67741  2.69355  4.24690  2.90347  2.73739  3.18146  2.89801
2.37887  2.77519  2.98518  4.58477  3.61503
          0.00877  5.13716  5.85950  0.61958  0.77255  0.48576  0.95510
    158   4.85848  5.92117  5.84743  5.65698  2.72960  5.47971  4.32025
4.37152  5.30741  2.62816  4.90777  5.11030  5.75196  5.20335  5.21589
4.89307  5.06564  4.36612  0.40972  2.37736    238 W - - -
          2.68618  4.42225  2.77519  2.73123  3.46354  2.40513  3.72494
3.29354  2.67741  2.69355  4.24690  2.90347  2.73739  3.18146  2.89801
2.37887  2.77519  2.98518  4.58477  3.61503
```

```
          0.00877  5.13716  5.85950  0.61958  0.77255  0.48576  0.95510
    159   2.95206  5.38078  3.23362  1.92851  4.69727  3.73574  3.47299
2.46232  1.94130  2.36758  4.43008  2.47078  4.12775  2.63820  2.90243
2.92992  3.18210  3.75619  5.82837  4.44159    239 e - - -
          2.68618  4.42225  2.77519  2.73123  3.46354  2.40513  3.72494
3.29354  2.67741  2.69355  4.24690  2.90347  2.73739  3.18146  2.89801
2.37887  2.77519  2.98518  4.58477  3.61503
          0.00877  5.13716  5.85950  0.61958  0.77255  0.48576  0.95510
    160   2.60367  5.44400  2.52059  2.28445  4.78853  3.38678  3.89575
3.69381  2.01808  3.36571  3.84504  2.12641  4.10212  2.62539  2.39838
2.45237  3.16586  3.83435  5.87700  4.46641    240 k - - -
          2.68618  4.42225  2.77519  2.73123  3.46354  2.40513  3.72494
3.29354  2.67741  2.69355  4.24690  2.90347  2.73739  3.18146  2.89801
2.37887  2.77519  2.98518  4.58477  3.61503
          0.00877  5.13716  5.85950  0.61958  0.77255  0.48576  0.95510
    161   2.58629  5.44084  2.61413  2.49080  4.78274  3.08372  3.19638
4.26665  2.51302  3.34957  4.48247  1.51708  3.59343  2.99649  2.92645
2.45444  2.89261  3.82980  5.87535  4.46600    241 n - - -
          2.68618  4.42225  2.77519  2.73123  3.46354  2.40513  3.72494
3.29354  2.67741  2.69355  4.24690  2.90347  2.73739  3.18146  2.89801
2.37887  2.77519  2.98518  4.58477  3.61503
          0.00877  5.13716  5.85950  0.61958  0.77255  0.48576  0.95510
    162   2.93700  5.44338  2.95801  2.48807  4.78673  2.88529  3.89798
4.27137  2.14030  3.74777  4.48462  2.90246  1.81259  2.52938  2.59049
2.48124  2.96832  3.83312  5.87688  3.70998    242 p - - -
          2.68618  4.42225  2.77519  2.73123  3.46354  2.40513  3.72494
3.29354  2.67741  2.69355  4.24690  2.90347  2.73739  3.18146  2.89801
2.37887  2.77519  2.98518  4.58477  3.61503
          0.03657  5.13716  3.50547  0.61958  0.77255  0.48576  0.95510
    163   2.73462  5.42228  2.40690  2.62010  4.76264  3.32737  3.88592
4.24527  2.22358  3.72555  3.84613  2.05485  4.09172  2.78778  2.73772
2.30951  2.13213  3.46510  5.85781  4.44975    243 n - - -
          2.68618  4.42225  2.77519  2.73123  3.46354  2.40513  3.72494
3.29354  2.67741  2.69355  4.24690  2.90347  2.73739  3.18146  2.89801
2.37887  2.77519  2.98518  4.58477  3.61503
          0.00901  5.10960  5.83195  0.61958  0.77255  0.57002  0.83359
    164   2.73853  5.43526  1.90022  2.32571  4.77893  3.36118  3.88753
4.26427  2.62398  3.74000  4.47659  2.48127  2.58025  2.77758  2.70000
2.20661  2.92904  3.45120  5.86872  4.45798    244 d - - -
          2.68632  4.42239  2.77534  2.73138  3.46368  2.40514  3.72509
3.29368  2.67738  2.69334  4.24704  2.90361  2.73698  3.18132  2.89793
2.37875  2.77514  2.98533  4.58491  3.61517
          0.05809  3.43525  3.72082  1.83942  0.17306  0.43646  1.03936
    165   2.26020  5.39975  2.95480  2.48173  3.98082  3.06081  3.89437
4.20490  2.63527  2.84144  4.44447  2.55474  2.68009  2.64055  2.71563
2.24831  2.45249  3.78217  5.84170  4.44014    257 s - - -
          2.68618  4.42225  2.77519  2.73123  3.46354  2.40513  3.72494
3.29354  2.67741  2.69355  4.24690  2.90347  2.73739  3.18146  2.89801
2.37887  2.77519  2.98518  4.58477  3.61503
          0.03076  5.11623  3.71750  0.61958  0.77255  0.55058  0.85948
    166   2.44049  5.36256  2.77298  2.38096  4.68126  3.07588  3.89143
3.61080  2.63732  3.26002  4.41033  1.92775  4.09419  2.99696  2.48770
2.49779  2.57152  2.82399  5.81146  4.41703    258 n - - -
```

```
          2.68618  4.42225  2.77519  2.73123  3.46354  2.40513  3.72494
3.29354  2.67741  2.69355  4.24690  2.90347  2.73739  3.18146  2.89801
2.37887  2.77519  2.98518  4.58477  3.61503
          0.03525  5.09462  3.55757  0.61958  0.77255  0.61212  0.78133
    167   1.78960  5.27601  2.76765  2.51467  4.56652  2.63094  3.89879
3.45528  2.35073  2.62920  4.33151  3.19762  3.52800  3.01638  2.82766
2.74137  2.62049  3.27446  5.74288  4.36753    259 a - - -
          2.68618  4.42225  2.77519  2.73123  3.46354  2.40513  3.72494
3.29354  2.67741  2.69355  4.24690  2.90347  2.73739  3.18146  2.89801
2.37887  2.77519  2.98518  4.58477  3.61503
          0.00939  5.06876  5.79110  0.61958  0.77255  0.38125  1.14887
    168   2.93549  5.42705  2.56976  2.63577  4.76425  3.34002  3.89988
2.56944  2.14297  3.23735  4.46982  2.75744  3.51629  2.17603  2.26266
2.71906  2.62673  3.81346  5.86439  4.45839    260 k - - -
          2.68572  4.42262  2.77524  2.73038  3.46390  2.40549  3.72531
3.29390  2.67731  2.69303  4.24726  2.90301  2.73758  3.18126  2.89820
2.37903  2.77537  2.98555  4.58513  3.61540
          1.01925  0.73871  1.82384  0.50377  0.92696  0.48576  0.95510
    169   2.37426  5.32349  2.54252  1.90503  4.64852  3.64327  3.83259
3.55462  2.57547  3.01345  4.36951  3.11886  4.03633  2.69750  2.17938
2.53546  2.53176  3.38750  5.76836  4.36987    265 e - - -
          2.68618  4.42225  2.77519  2.73123  3.46354  2.40513  3.72494
3.29354  2.67741  2.69355  4.24690  2.90347  2.73739  3.18146  2.89801
2.37887  2.77519  2.98518  4.58477  3.61503
          0.01043  4.96452  5.68687  0.61958  0.77255  0.89973  0.52202
    170   2.70560  4.74304  5.43063  4.87607  3.87786  4.81787  5.26572
2.00678  4.73462  1.85074  2.83598  4.95872  5.10556  4.89311  4.80681
4.17055  3.69933  0.82941  5.68775  4.52077    266 v - - -
          2.68618  4.42225  2.77519  2.73123  3.46354  2.40513  3.72494
3.29354  2.67741  2.69355  4.24690  2.90347  2.73739  3.18146  2.89801
2.37887  2.77519  2.98518  4.58477  3.61503
          0.01043  4.96452  5.68687  0.61958  0.77255  0.39587  1.11809
    171   2.59431  5.29881  3.22876  2.67211  3.90385  3.38346  3.44967
3.54474  2.25286  3.24061  4.35352  2.97269  4.11071  2.80730  1.64454
2.33682  2.69593  3.24316  5.76374  4.38664    267 r - - -
          2.68618  4.42225  2.77519  2.73123  3.46354  2.40513  3.72494
3.29354  2.67741  2.69355  4.24690  2.90347  2.73739  3.18146  2.89801
2.37887  2.77519  2.98518  4.58477  3.61503
          0.03458  5.10263  3.57875  0.61958  0.77255  0.58992  0.80828
    172   2.72025  5.40481  2.59469  1.95951  4.74383  3.35928  3.87224
3.08877  2.33033  3.70750  4.44706  2.75061  4.07789  2.47408  2.70775
2.87364  2.12148  3.36688  5.84111  3.68122    268 e - - -
          2.68618  4.42225  2.77519  2.73123  3.46354  2.40513  3.72494
3.29354  2.67741  2.69355  4.24690  2.90347  2.73739  3.18146  2.89801
2.37887  2.77519  2.98518  4.58477  3.61503
          0.09486  5.07735  2.47381  0.61958  0.77255  0.65782  0.72976
    173   2.40770  5.28027  3.18127  2.33247  4.58345  3.67457  3.86282
3.43841  2.45285  3.26820  4.33288  3.16151  4.06623  2.26876  1.66940
2.86906  2.52572  3.64832  5.73718  3.77119    269 r - - -
          2.68618  4.42225  2.77519  2.73123  3.46354  2.40513  3.72494
3.29354  2.67741  2.69355  4.24690  2.90347  2.73739  3.18146  2.89801
2.37887  2.77519  2.98518  4.58477  3.61503
          0.01014  4.99263  5.71497  0.61958  0.77255  0.62409  0.76733
```

174 3.58927 4.90407 5.43081 4.87170 0.86774 4.76096 4.77298
2.06670 4.68065 1.95502 3.10264 4.84188 5.03517 4.70557 4.66667
4.09256 3.81118 2.95801 5.03333 2.75605 270 f - - -
2.68618 4.42225 2.77519 2.73123 3.46354 2.40513 3.72494
3.29354 2.67741 2.69355 4.24690 2.90347 2.73739 3.18146 2.89801
2.37887 2.77519 2.98518 4.58477 3.61503
0.00961 5.04608 5.76843 0.61958 0.77255 0.46019 0.99739
175 2.52148 5.42952 2.58257 2.16090 3.81965 3.01481 3.45132
3.14152 2.33378 3.73416 4.47057 2.61656 4.09013 2.38185 2.72675
2.21333 2.93873 3.81936 5.86309 4.45303 271 e - - -
2.68618 4.42225 2.77519 2.73123 3.46354 2.40513 3.72494
3.29354 2.67741 2.69355 4.24690 2.90347 2.73739 3.18146 2.89801
2.37887 2.77519 2.98518 4.58477 3.61503
0.00901 5.10960 5.83195 0.61958 0.77255 0.43646 1.03936
176 2.62329 5.42339 2.24312 2.33833 4.75899 3.71251 3.90069
3.08467 2.48047 2.88221 4.46656 2.42244 4.10586 2.83348 2.73334
2.03394 2.60622 3.80893 5.86175 4.45656 272 s - - -
2.68618 4.42225 2.77519 2.73123 3.46354 2.40513 3.72494
3.29354 2.67741 2.69355 4.24690 2.90347 2.73739 3.18146 2.89801
2.37887 2.77519 2.98518 4.58477 3.61503
0.00877 5.13716 5.85950 0.61958 0.77255 0.48576 0.95510
177 2.04772 4.52179 5.09963 4.49832 3.14053 4.36925 4.71927
2.30412 4.30481 1.38620 3.19586 4.51770 4.71410 4.44128 4.33363
3.68718 3.06599 1.45230 5.20123 4.03111 273 l - - -
2.68618 4.42225 2.77519 2.73123 3.46354 2.40513 3.72494
3.29354 2.67741 2.69355 4.24690 2.90347 2.73739 3.18146 2.89801
2.37887 2.77519 2.98518 4.58477 3.61503
0.00877 5.13716 5.85950 0.61958 0.77255 0.48576 0.95510
178 2.93716 5.36141 2.00991 2.38451 3.45226 3.72455 2.36386
3.61697 2.46627 2.98917 4.41134 2.06987 4.11735 3.02462 3.15202
2.91682 2.93786 3.31847 5.81530 4.42613 274 d - - -
2.68618 4.42225 2.77519 2.73123 3.46354 2.40513 3.72494
3.29354 2.67741 2.69355 4.24690 2.90347 2.73739 3.18146 2.89801
2.37887 2.77519 2.98518 4.58477 3.61503
0.03267 5.13716 3.63959 0.61958 0.77255 0.48576 0.95510
179 2.57696 5.30218 2.98432 2.67697 3.85060 2.71260 3.91683
4.04561 2.52696 3.07155 3.73688 2.74361 4.11536 2.84498 2.88211
1.76990 2.22596 3.66439 5.76752 3.78604 275 s - - -
2.68618 4.42225 2.77519 2.73123 3.46354 2.40513 3.72494
3.29354 2.67741 2.69355 4.24690 2.90347 2.73739 3.18146 2.89801
2.37887 2.77519 2.98518 4.58477 3.61503
0.00898 5.11347 5.83581 0.61958 0.77255 0.55875 0.84845
180 2.32630 5.22983 2.80731 2.71503 3.07807 3.73907 2.81283
3.41223 2.38792 2.49531 3.74181 2.83160 4.13021 2.50442 2.97119
2.50915 2.72181 3.26087 5.71166 4.35271 276 a - - -
2.68618 4.42225 2.77519 2.73123 3.46354 2.40513 3.72494
3.29354 2.67741 2.69355 4.24690 2.90347 2.73739 3.18146 2.89801
2.37887 2.77519 2.98518 4.58477 3.61503
0.03564 5.11347 3.54050 0.61958 0.77255 0.55875 0.84845
181 2.77133 3.54816 4.93302 4.32558 1.33213 4.18755 4.52491
2.12095 4.12454 1.67259 3.47960 4.33641 4.54817 4.26239 4.15153
3.05907 3.25105 2.57118 5.02263 3.84366 277 f - - -
2.68618 4.42225 2.77519 2.73123 3.46354 2.40513 3.72494
3.29354 2.67741 2.69355 4.24690 2.90347 2.73739 3.18146 2.89801
2.37887 2.77519 2.98518 4.58477 3.61503

```
         0.03712  5.08704  3.49792  0.61958  0.77255  0.50688  0.92223
    182  2.72426  5.25052  2.90186  1.89933  3.78642  3.71992  3.91564
2.54502  2.41187  3.52705  4.30927  2.96822  4.11151  2.84145  2.72611
2.91585  2.46709  2.30130  5.72502  4.35804    278 e - - -
         2.68618  4.42225  2.77519  2.73123  3.46354  2.40513  3.72494
3.29354  2.67741  2.69355  4.24690  2.90347  2.73739  3.18146  2.89801
2.37887  2.77519  2.98518  4.58477  3.61503
         0.00922  5.08656  5.80890  0.61958  0.77255  0.40320  1.10316
    183  2.05204  5.43155  2.91095  2.22209  4.02911  3.37828  3.89846
4.25262  2.34009  3.05776  4.47375  2.34097  4.10413  2.67408  2.57057
2.47495  2.76378  3.81904  5.86775  3.41028    279 a - - -
         2.68618  4.42225  2.77519  2.73123  3.46354  2.40513  3.72494
3.29354  2.67741  2.69355  4.24690  2.90347  2.73739  3.18146  2.89801
2.37887  2.77519  2.98518  4.58477  3.61503
         0.00877  5.13716  5.85950  0.61958  0.77255  0.48576  0.95510
    184  1.86497  3.99926  2.82331  2.39475  4.62573  3.73101  3.92391
4.07985  2.68025  3.27049  4.38102  2.57116  4.12332  2.85089  3.16370
1.83761  2.61254  3.35036  5.78958  3.75907    280 s - - -
         2.68618  4.42225  2.77519  2.73123  3.46354  2.40513  3.72494
3.29354  2.67741  2.69355  4.24690  2.90347  2.73739  3.18146  2.89801
2.37887  2.77519  2.98518  4.58477  3.61503
         0.00877  5.13716  5.85950  0.61958  0.77255  0.48576  0.95510
    185  2.77243  4.45667  4.53015  3.06410  3.55639  4.11720  4.42583
1.71132  3.34618  2.07300  1.49454  4.12331  4.48391  4.01857  3.96103
3.40857  2.95853  2.66736  5.07755  3.88944    281 m - - -
         2.68618  4.42225  2.77519  2.73123  3.46354  2.40513  3.72494
3.29354  2.67741  2.69355  4.24690  2.90347  2.73739  3.18146  2.89801
2.37887  2.77519  2.98518  4.58477  3.61503
         0.00877  5.13716  5.85950  0.61958  0.77255  0.48576  0.95510
    186  2.12081  4.10785  2.89271  2.38897  4.68065  3.72512  3.91789
3.66114  2.66694  3.65964  4.41838  2.57981  1.71045  3.02626  2.40082
2.91876  3.17144  3.74114  5.82148  4.43151    282 p - - -
         2.68618  4.42225  2.77519  2.73123  3.46354  2.40513  3.72494
3.29354  2.67741  2.69355  4.24690  2.90347  2.73739  3.18146  2.89801
2.37887  2.77519  2.98518  4.58477  3.61503
         0.00877  5.13716  5.85950  0.61958  0.77255  0.48576  0.95510
    187  2.74773  5.39753  2.94044  2.03370  3.14917  3.71715  3.90638
3.62118  2.38822  3.08309  4.44349  2.96416  4.11014  2.25628  2.74833
1.95007  3.16681  3.77740  5.84235  3.28832    283 s - - -
         2.68526  4.42205  2.77551  2.73192  3.46314  2.40569  3.72496
3.29331  2.67786  2.69252  4.24758  2.90313  2.73740  3.18104  2.89846
2.37860  2.77567  2.98509  4.58545  3.61512
         0.29648  1.37154  5.85950  1.68417  0.20530  0.48576  0.95510
    188  4.11245  5.36513  5.56043  5.18325  0.59151  5.05058  4.36577
3.74150  4.98141  1.82673  4.34889  4.89329  5.35815  4.92555  4.94093
3.50108  4.33587  3.66292  4.51721  2.45330    293 F - - -
         2.68618  4.42225  2.77519  2.73123  3.46354  2.40513  3.72494
3.29354  2.67741  2.69355  4.24690  2.90347  2.73739  3.18146  2.89801
2.37887  2.77519  2.98518  4.58477  3.61503
         0.00877  5.13716  5.85950  0.61958  0.77255  0.48576  0.95510
    189  2.07256  5.43537  2.97109  2.63529  4.77568  3.37562  3.90007
3.68552  1.95089  3.73828  4.47768  2.86876  4.10636  2.63992  2.24278
1.88010  3.16904  3.82354  5.87072  4.46364    294 s - - -
```

```
         2.68618  4.42225  2.77519  2.73123  3.46354  2.40513  3.72494
3.29354  2.67741  2.69355  4.24690  2.90347  2.73739  3.18146  2.89801
2.37887  2.77519  2.98518  4.58477  3.61503
         0.00877  5.13716  5.85950  0.61958  0.77255  0.48576  0.95510
    190  2.46251  3.85792  3.94894  3.37858  3.74801  3.46716  3.62811
2.71839  3.32181  2.46458  2.97124  3.75268  3.28186  2.53721  2.73380
2.72438  3.19419  1.68767  5.21073  3.98782    295 v - - -
         2.68618  4.42225  2.77519  2.73123  3.46354  2.40513  3.72494
3.29354  2.67741  2.69355  4.24690  2.90347  2.73739  3.18146  2.89801
2.37887  2.77519  2.98518  4.58477  3.61503
         0.00877  5.13716  5.85950  0.61958  0.77255  0.48576  0.95510
    191  2.42389  5.44635  2.59228  2.24159  4.79160  2.88275  3.89577
4.27773  2.35758  3.38202  4.48699  2.40437  2.38830  2.64581  2.72996
2.62219  2.79916  3.83709  5.87890  4.46777    296 e - - -
         2.68614  4.42238  2.77510  2.73120  3.46367  2.40488  3.72507
3.29367  2.67754  2.69368  4.24703  2.90334  2.73736  3.18159  2.89813
2.37873  2.77532  2.98508  4.58490  3.61516
         0.12587  2.50745  3.30249  1.12514  0.39246  0.48576  0.95510
    192  2.92134  5.41722  2.74905  2.47736  4.75607  1.96781  3.88469
4.23768  2.14044  3.71979  4.45963  2.12472  3.56241  2.98315  2.72454
2.88636  2.72706  3.33211  5.85355  3.40653    301 g - - -
         2.68618  4.42225  2.77519  2.73123  3.46354  2.40513  3.72494
3.29354  2.67741  2.69355  4.24690  2.90347  2.73739  3.18146  2.89801
2.37887  2.77519  2.98518  4.58477  3.61503
         0.00907  5.10263  5.82497  0.61958  0.77255  0.58992  0.80828
    193  2.73153  4.93043  3.19013  2.93553  2.47434  3.39218  2.99192
3.50431  2.92091  3.16444  4.01807  3.11157  3.62043  3.25626  3.02935
3.04464  3.17421  3.23333  5.45761  1.47031    302 y - - -
         2.68618  4.42225  2.77519  2.73123  3.46354  2.40513  3.72494
3.29354  2.67741  2.69355  4.24690  2.90347  2.73739  3.18146  2.89801
2.37887  2.77519  2.98518  4.58477  3.61503
         0.03500  5.10263  3.56425  0.61958  0.77255  0.42574  1.05925
    194  2.55162  5.48309  2.90104  1.15621  4.82483  3.71653  3.92296
4.31096  2.51353  3.78608  4.52693  2.95217  4.12417  2.27243  2.91769
2.93021  2.90573  3.87239  5.91510  4.50160    303 e - - -
         2.68618  4.42225  2.77519  2.73123  3.46354  2.40513  3.72494
3.29354  2.67741  2.69355  4.24690  2.90347  2.73739  3.18146  2.89801
2.37887  2.77519  2.98518  4.58477  3.61503
         0.00899  5.11235  5.83469  0.61958  0.77255  0.44086  1.03137
    195  2.59775  4.42937  4.65067  4.05659  3.53434  4.13691  4.45746
1.88824  3.90620  1.95994  3.54690  4.18915  3.33722  3.03938  4.02676
3.43472  2.52725  1.45554  5.05946  3.87430    304 v - - -
         2.68618  4.42225  2.77519  2.73123  3.46354  2.40513  3.72494
3.29354  2.67741  2.69355  4.24690  2.90347  2.73739  3.18146  2.89801
2.37887  2.77519  2.98518  4.58477  3.61503
         0.00877  5.13716  5.85950  0.61958  0.77255  0.48576  0.95510
    196  2.58901  4.47304  4.34776  3.76439  3.57960  4.05872  4.35566
2.25825  3.65743  1.51588  3.58680  4.00563  2.13658  3.07358  3.86578
3.04490  2.68353  2.55079  5.08200  3.26683    305 l - - -
         2.68618  4.42225  2.77519  2.73123  3.46354  2.40513  3.72494
3.29354  2.67741  2.69355  4.24690  2.90347  2.73739  3.18146  2.89801
2.37887  2.77519  2.98518  4.58477  3.61503
         0.00877  5.13716  5.85950  0.61958  0.77255  0.48576  0.95510
```

```
    197   3.31472  4.68166  5.15645  4.55229  2.64782  4.49756  4.81582
2.84011  4.36980  0.74883  3.07008  4.61956  4.81176  3.63518  4.40806
3.81291  2.99750  2.22829  5.25967  4.12382    306 l - - -
          2.68618  4.42225  2.77519  2.73123  3.46354  2.40513  3.72494
3.29354  2.67741  2.69355  4.24690  2.90347  2.73739  3.18146  2.89801
2.37887  2.77519  2.98518  4.58477  3.61503
          0.00877  5.13716  5.85950  0.61958  0.77255  0.48576  0.95510
    198   4.70091  5.82868  6.47726  5.99431  2.90475  6.02093  5.96311
3.04287  5.85576  0.25653  3.48305  6.17287  5.92538  5.54899  5.70846
5.53230  4.89005  3.42388  5.80649  4.59193    307 L - - -
          2.68618  4.42225  2.77519  2.73123  3.46354  2.40513  3.72494
3.29354  2.67741  2.69355  4.24690  2.90347  2.73739  3.18146  2.89801
2.37887  2.77519  2.98518  4.58477  3.61503
          0.00877  5.13716  5.85950  0.61958  0.77255  0.48576  0.95510
    199   2.50523  5.00797  3.58284  3.03951  4.40671  3.31352  4.18132
3.81192  3.02804  3.09407  4.27813  3.13283  1.45356  3.36526  3.46534
1.99416  2.29696  3.05120  5.72016  4.42125    308 p - - -
          2.68618  4.42225  2.77519  2.73123  3.46354  2.40513  3.72494
3.29354  2.67741  2.69355  4.24690  2.90347  2.73739  3.18146  2.89801
2.37887  2.77519  2.98518  4.58477  3.61503
          0.00877  5.13716  5.85950  0.61958  0.77255  0.48576  0.95510
    200   2.36626  3.68071  4.69745  4.09740  3.48864  3.53234  4.43628
2.40853  3.93250  2.17558  3.15280  3.14451  4.48244  4.10833  4.02560
2.88068  2.30601  1.48274  5.00980  3.22711    309 v - - -
          2.68618  4.42225  2.77519  2.73123  3.46354  2.40513  3.72494
3.29354  2.67741  2.69355  4.24690  2.90347  2.73739  3.18146  2.89801
2.37887  2.77519  2.98518  4.58477  3.61503
          0.00877  5.13716  5.85950  0.61958  0.77255  0.48576  0.95510
    201   5.10144  6.06834  5.82561  5.75477  1.99902  5.56488  4.24875
4.76825  5.57458  3.92331  5.28609  3.31951  5.84113  5.28131  5.41971
4.97973  5.30585  4.70260  4.32975  0.33953    310 Y - - -
          2.68618  4.42225  2.77519  2.73123  3.46354  2.40513  3.72494
3.29354  2.67741  2.69355  4.24690  2.90347  2.73739  3.18146  2.89801
2.37887  2.77519  2.98518  4.58477  3.61503
          0.00877  5.13716  5.85950  0.61958  0.77255  0.48576  0.95510
    202   0.82041  4.66677  4.44741  3.92706  4.06002  3.90803  4.65529
3.30284  3.84097  3.12479  3.30637  4.10039  4.47118  4.09571  4.08871
2.91701  2.30262  2.29326  5.54571  4.34951    311 a - - -
          2.68618  4.42225  2.77519  2.73123  3.46354  2.40513  3.72494
3.29354  2.67741  2.69355  4.24690  2.90347  2.73739  3.18146  2.89801
2.37887  2.77519  2.98518  4.58477  3.61503
          0.00877  5.13716  5.85950  0.61958  0.77255  0.48576  0.95510
    203   2.96519  5.33616  3.25095  2.40826  4.63176  3.75123  3.94638
4.08004  2.69559  3.30146  3.70920  2.53552  4.14715  1.12183  3.16763
2.77689  3.19633  3.33311  5.79933  4.42554    312 q - - -
          2.68618  4.42225  2.77519  2.73123  3.46354  2.40513  3.72494
3.29354  2.67741  2.69355  4.24690  2.90347  2.73739  3.18146  2.89801
2.37887  2.77519  2.98518  4.58477  3.61503
          0.00877  5.13716  5.85950  0.61958  0.77255  0.48576  0.95510
    204   0.79262  4.65414  4.71245  4.19833  4.01822  3.39460  4.79837
2.23813  4.09005  3.03089  3.99032  4.29644  4.59014  4.31626  4.27654
3.41663  3.00675  2.17143  5.55429  4.36584    313 a - - -
          2.68618  4.42225  2.77519  2.73123  3.46354  2.40513  3.72494
3.29354  2.67741  2.69355  4.24690  2.90347  2.73739  3.18146  2.89801
2.37887  2.77519  2.98518  4.58477  3.61503
```

```
         0.00877  5.13716  5.85950  0.61958  0.77255  0.48576  0.95510
    205  0.30362  4.92778  5.14961  4.99989  5.19861  3.82116  5.67385
4.04744  4.90939  4.12971  5.15105  4.60257  4.65336  5.10733  4.99214
3.32207  3.62980  2.73960  6.73016  5.59869    314 A - - -
         2.68618  4.42225  2.77519  2.73123  3.46354  2.40513  3.72494
3.29354  2.67741  2.69355  4.24690  2.90347  2.73739  3.18146  2.89801
2.37887  2.77519  2.98518  4.58477  3.61503
         0.00877  5.13716  5.85950  0.61958  0.77255  0.48576  0.95510
    206  3.14633  5.29317  3.55120  3.05124  4.68657  3.89660  4.18724
4.09351  2.85061  3.68141  3.60449  0.81572  4.35167  3.34371  2.69058
3.19258  2.68158  3.74850  5.88880  4.58701    315 n - - -
         2.68618  4.42225  2.77519  2.73123  3.46354  2.40513  3.72494
3.29354  2.67741  2.69355  4.24690  2.90347  2.73739  3.18146  2.89801
2.37887  2.77519  2.98518  4.58477  3.61503
         0.00877  5.13716  5.85950  0.61958  0.77255  0.48576  0.95510
    207  3.47926  4.81316  5.43102  4.82017  2.13551  4.68785  4.99514
2.25909  4.62198  0.81674  2.54811  4.84855  4.96634  4.67045  4.60694
4.01341  3.27664  2.90334  3.87694  4.23198    316 l - - -
         2.68618  4.42225  2.77519  2.73123  3.46354  2.40513  3.72494
3.29354  2.67741  2.69355  4.24690  2.90347  2.73739  3.18146  2.89801
2.37887  2.77519  2.98518  4.58477  3.61503
         0.00877  5.13716  5.85950  0.61958  0.77255  0.48576  0.95510
    208  3.50570  5.67816  2.86737  3.04887  4.13034  4.02878  0.62385
4.43071  3.35678  3.96331  4.87126  3.57058  4.56727  3.64864  3.80501
2.91092  3.77876  4.10710  5.62194  3.35340    317 H - - -
         2.68618  4.42225  2.77519  2.73123  3.46354  2.40513  3.72494
3.29354  2.67741  2.69355  4.24690  2.90347  2.73739  3.18146  2.89801
2.37887  2.77519  2.98518  4.58477  3.61503
         0.00877  5.13716  5.85950  0.61958  0.77255  0.48576  0.95510
    209  4.88721  6.05005  6.17537  5.93623  4.14965  5.56928  6.29945
3.48881  5.77186  0.14890  3.98794  6.16023  5.89043  5.79340  5.69986
5.54856  5.15610  3.80797  6.28958  5.27126    318 L - - -
         2.68618  4.42225  2.77519  2.73123  3.46354  2.40513  3.72494
3.29354  2.67741  2.69355  4.24690  2.90347  2.73739  3.18146  2.89801
2.37887  2.77519  2.98518  4.58477  3.61503
         0.00877  5.13716  5.85950  0.61958  0.77255  0.48576  0.95510
    210  2.39454  4.49925  4.26695  3.68775  3.60953  4.04371  4.33497
2.35657  3.21833  1.12695  3.61244  2.85598  4.41648  3.83794  3.82193
3.04647  2.95296  2.75532  5.10568  3.41794    319 l - - -
         2.68618  4.42225  2.77519  2.73123  3.46354  2.40513  3.72494
3.29354  2.67741  2.69355  4.24690  2.90347  2.73739  3.18146  2.89801
2.37887  2.77519  2.98518  4.58477  3.61503
         0.00877  5.13716  5.85950  0.61958  0.77255  0.48576  0.95510
    211  3.41667  4.76868  5.25080  4.65801  3.62366  4.62591  2.94237
2.15425  4.47000  0.79108  3.53807  4.73996  4.92933  4.59800  4.52040
3.94920  3.64584  1.94207  5.38020  4.22892    320 l - - -
         2.68618  4.42225  2.77519  2.73123  3.46354  2.40513  3.72494
3.29354  2.67741  2.69355  4.24690  2.90347  2.73739  3.18146  2.89801
2.37887  2.77519  2.98518  4.58477  3.61503
         0.00877  5.13716  5.85950  0.61958  0.77255  0.48576  0.95510
    212  2.89007  4.58794  4.91617  4.31581  3.54154  4.34529  4.65749
2.58309  3.47608  0.70294  2.99139  4.42410  4.68314  4.30034  4.21618
3.65219  3.41837  2.74430  5.18172  3.43690    321 l - - -
```

2.68618 4.42225 2.77519 2.73123 3.46354 2.40513 3.72494
3.29354 2.67741 2.69355 4.24690 2.90347 2.73739 3.18146 2.89801
2.37887 2.77519 2.98518 4.58477 3.61503
0.00877 5.13716 5.85950 0.61958 0.77255 0.48576 0.95510
213 3.85780 6.01671 4.37277 3.54359 5.60537 4.43466 2.93911
4.88581 1.85500 4.21411 5.07756 3.32415 4.75414 2.65162 0.68013
3.81656 3.97612 4.55156 6.17122 5.06101 322 R - - -
2.68618 4.42225 2.77519 2.73123 3.46354 2.40513 3.72494
3.29354 2.67741 2.69355 4.24690 2.90347 2.73739 3.18146 2.89801
2.37887 2.77519 2.98518 4.58477 3.61503
0.00877 5.13716 5.85950 0.61958 0.77255 0.48576 0.95510
214 3.04692 5.53771 1.13416 2.11086 4.86416 3.75489 3.99205
3.20561 2.75842 3.83401 4.58888 3.22340 4.18271 2.50443 2.97790
3.00476 2.98842 3.91908 5.97653 4.56356 323 d - - -
2.68618 4.42225 2.77519 2.73123 3.46354 2.40513 3.72494
3.29354 2.67741 2.69355 4.24690 2.90347 2.73739 3.18146 2.89801
2.37887 2.77519 2.98518 4.58477 3.61503
0.00877 5.13716 5.85950 0.61958 0.77255 0.48576 0.95510
215 1.71039 4.64247 3.93520 3.36832 3.35287 1.83085 4.24125
2.53656 2.92446 2.86411 3.76208 3.74557 4.34509 3.60634 3.64580
2.41422 3.20185 2.41208 5.24500 4.01969 324 a - - -
2.68618 4.42225 2.77519 2.73123 3.46354 2.40513 3.72494
3.29354 2.67741 2.69355 4.24690 2.90347 2.73739 3.18146 2.89801
2.37887 2.77519 2.98518 4.58477 3.61503
0.00877 5.13716 5.85950 0.61958 0.77255 0.48576 0.95510
216 2.55962 4.40995 4.60476 3.19550 3.14115 4.10245 4.41668
2.35680 3.86165 2.02420 3.11014 4.14783 4.46978 3.57854 3.98612
2.26251 2.58844 1.62749 5.02564 3.23606 325 v - - -
2.68618 4.42225 2.77519 2.73123 3.46354 2.40513 3.72494
3.29354 2.67741 2.69355 4.24690 2.90347 2.73739 3.18146 2.89801
2.37887 2.77519 2.98518 4.58477 3.61503
0.00877 5.13716 5.85950 0.61958 0.77255 0.48576 0.95510
217 2.56954 4.45610 4.41007 3.82400 3.18985 4.07083 4.37179
1.52869 2.76079 1.96958 3.18462 3.48240 4.44065 2.85691 3.89664
3.35508 2.77199 2.29076 5.06702 3.87399 326 i - - -
2.68618 4.42225 2.77519 2.73123 3.46354 2.40513 3.72494
3.29354 2.67741 2.69355 4.24690 2.90347 2.73739 3.18146 2.89801
2.37887 2.77519 2.98518 4.58477 3.61503
0.00877 5.13716 5.85950 0.61958 0.77255 0.48576 0.95510
218 4.71443 5.83927 5.66036 5.41396 1.05779 5.37655 2.88048
4.47011 5.24102 3.06732 5.02501 2.86453 5.65942 5.08977 5.19419
4.74372 4.91987 4.36115 4.35442 0.94532 327 y - - -
2.68618 4.42225 2.77519 2.73123 3.46354 2.40513 3.72494
3.29354 2.67741 2.69355 4.24690 2.90347 2.73739 3.18146 2.89801
2.37887 2.77519 2.98518 4.58477 3.61503
0.00877 5.13716 5.85950 0.61958 0.77255 0.48576 0.95510
219 2.09992 5.32692 3.26800 2.58316 4.61694 1.35258 3.97147
4.05844 2.55746 3.60747 4.39191 2.97257 4.17076 3.09563 2.70139
2.98523 3.22096 3.68977 5.79740 3.21751 328 g - - -
2.68618 4.42225 2.77519 2.73123 3.46354 2.40513 3.72494
3.29354 2.67741 2.69355 4.24690 2.90347 2.73739 3.18146 2.89801
2.37887 2.77519 2.98518 4.58477 3.61503
0.00877 5.13716 5.85950 0.61958 0.77255 0.48576 0.95510

```
    220   2.48845  5.44509  2.04242  2.00677  4.78974  3.04359  3.89617
4.27546  1.99432  3.36599  4.48590  2.74302  4.10245  2.81303  2.92675
2.74040  2.65090  3.42678  5.87799  4.46722    329 k - - -
         2.68618  4.42225  2.77519  2.73123  3.46354  2.40513  3.72494
3.29354  2.67741  2.69355  4.24690  2.90347  2.73739  3.18146  2.89801
2.37887  2.77519  2.98518  4.58477  3.61503
         0.00877  5.13716  5.85950  0.61958  0.77255  0.48576  0.95510
    221   2.43150  5.44469  2.76128  1.67008  4.78931  3.36334  3.89600
4.27496  2.28422  3.74976  4.48549  3.17637  3.54996  2.10973  2.61715
2.55868  3.16625  3.38554  5.87760  3.83798    330 e - - -
         2.68618  4.42225  2.77519  2.73123  3.46354  2.40513  3.72494
3.29354  2.67741  2.69355  4.24690  2.90347  2.73739  3.18146  2.89801
2.37887  2.77519  2.98518  4.58477  3.61503
         0.00877  5.13716  5.85950  0.61958  0.77255  0.48576  0.95510
    222   3.45934  4.90690  3.62313  4.26060  3.35893  4.51103  4.56497
2.77263  4.10809  1.99012  3.80204  4.43755  4.86207  4.33155  4.27398
3.83102  3.69367  3.02939  0.78453  3.56303    331 w - - -
         2.68618  4.42225  2.77519  2.73123  3.46354  2.40513  3.72494
3.29354  2.67741  2.69355  4.24690  2.90347  2.73739  3.18146  2.89801
2.37887  2.77519  2.98518  4.58477  3.61503
         0.00877  5.13716  5.85950  0.61958  0.77255  0.48576  0.95510
    223   2.59135  5.32212  2.56481  2.68855  4.61637  1.54287  3.93235
3.54814  2.55120  2.96654  4.37775  2.31741  4.13009  2.84052  3.17482
2.93323  3.17553  3.31868  5.78745  4.40940    332 g - - -
         2.68618  4.42225  2.77519  2.73123  3.46354  2.40513  3.72494
3.29354  2.67741  2.69355  4.24690  2.90347  2.73739  3.18146  2.89801
2.37887  2.77519  2.98518  4.58477  3.61503
         0.00877  5.13716  5.85950  0.61958  0.77255  0.48576  0.95510
    224   2.56904  4.40428  4.63452  3.47261  2.30624  4.10719  4.42311
2.34216  3.88459  1.47147  3.15997  2.91102  4.47415  4.07140  3.99908
2.81711  3.20716  2.65439  5.02023  2.58706    333 l - - -
         2.68618  4.42225  2.77519  2.73123  3.46354  2.40513  3.72494
3.29354  2.67741  2.69355  4.24690  2.90347  2.73739  3.18146  2.89801
2.37887  2.77519  2.98518  4.58477  3.61503
         0.03326  5.13716  3.61801  0.61958  0.77255  0.48576  0.95510
    225   2.21828  5.41508  2.14380  2.48434  3.86060  3.21611  3.88954
4.23165  2.48437  3.71673  4.45802  2.75073  2.99611  2.80961  3.11919
2.23914  2.34503  3.80113  5.85290  3.69508    334 d - - -
         2.68633  4.42306  2.77523  2.73128  3.46401  2.40567  3.72527
3.29211  2.67804  2.69366  4.24609  2.90281  2.73729  3.18087  2.89693
2.37878  2.77543  2.98599  4.58558  3.61457
         0.56365  0.91642  3.47614  1.44417  0.26911  0.44173  1.02981
    226   2.75212  5.41972  2.76345  2.13279  3.83007  2.97940  3.45453
3.67737  2.48011  3.34612  4.46187  3.16802  2.51597  2.41414  2.85138
1.96085  3.15370  3.80731  5.85580  4.44828    341 s - - -
         2.68637  4.42086  2.77536  2.73087  3.46328  2.40554  3.72548
3.29283  2.67778  2.69339  4.24744  2.90269  2.73704  3.18172  2.89831
2.37904  2.77499  2.98550  4.58531  3.61471
         0.39615  1.57015  2.12793  1.65837  0.21127  0.43622  1.03981
    227   2.01015  5.02359  3.04202  2.62798  3.63737  2.68141  3.94569
2.74842  2.45987  2.75777  3.60539  3.04441  3.52156  2.89660  3.22098
2.54077  2.87871  3.05028  5.53932  3.62363    366 a - - -
         2.68618  4.42225  2.77519  2.73123  3.46354  2.40513  3.72494
3.29354  2.67741  2.69355  4.24690  2.90347  2.73739  3.18146  2.89801
2.37887  2.77519  2.98518  4.58477  3.61503
```

0.03402 5.01727 3.61854 0.61958 0.77255 0.41137 1.08691
228 2.51595 5.42541 2.32981 2.04696 4.76714 3.69183 3.88280
4.25121 2.62081 3.72946 4.46762 2.75264 2.62998 2.59835 3.11381
2.29290 2.21045 3.44014 5.86028 4.45046 367 e - - -
2.68618 4.42225 2.77519 2.73123 3.46354 2.40513 3.72494
3.29354 2.67741 2.69355 4.24690 2.90347 2.73739 3.18146 2.89801
2.37887 2.77519 2.98518 4.58477 3.61503
0.00917 5.09179 5.81414 0.61958 0.77255 0.51537 0.90951
229 2.57956 4.78196 3.65633 2.83268 3.93851 2.89289 4.11409
2.19273 3.06228 2.80780 3.47739 3.54217 4.25972 2.53090 3.13883
2.23791 2.53511 2.14687 5.34725 4.09019 368 v - - -
2.68618 4.42225 2.77519 2.73123 3.46354 2.40513 3.72494
3.29354 2.67741 2.69355 4.24690 2.90347 2.73739 3.18146 2.89801
2.37887 2.77519 2.98518 4.58477 3.61503
0.00898 5.11347 5.83581 0.61958 0.77255 0.55875 0.84845
230 2.62624 5.45465 1.81688 2.15524 3.40510 3.02348 3.90395
4.28178 2.36817 3.75848 4.49717 2.07133 4.10681 2.79962 3.14078
2.56061 3.17825 3.84359 5.88850 4.47627 369 d - - -
2.68618 4.42225 2.77519 2.73123 3.46354 2.40513 3.72494
3.29354 2.67741 2.69355 4.24690 2.90347 2.73739 3.18146 2.89801
2.37887 2.77519 2.98518 4.58477 3.61503
0.00898 5.11347 5.83581 0.61958 0.77255 0.55875 0.84845
231 2.93330 5.06028 2.41209 2.54207 2.76936 3.03078 3.99127
3.33208 2.81358 2.37520 4.13694 3.05179 4.17072 3.15899 2.83887
2.12682 2.72927 2.80823 5.57729 3.66055 370 s - - -
2.68619 4.42226 2.77503 2.73124 3.46355 2.40514 3.72495
3.29355 2.67742 2.69356 4.24691 2.90348 2.73740 3.18147 2.89802
2.37888 2.77520 2.98519 4.58478 3.61504
0.03241 3.54159 5.83581 0.41186 1.08595 0.44268 1.02809
232 3.56732 5.15444 4.45604 3.93458 2.19344 4.48196 2.69093
3.68733 3.84801 2.65590 4.27965 2.18462 4.83904 4.09109 3.45963
3.75540 3.79379 3.49047 4.67531 1.02327 372 y - - -
2.68618 4.42225 2.77519 2.73123 3.46354 2.40513 3.72494
3.29354 2.67741 2.69355 4.24690 2.90347 2.73739 3.18146 2.89801
2.37887 2.77519 2.98518 4.58477 3.61503
0.00877 5.13716 5.85950 0.61958 0.77255 0.48576 0.95510
233 2.94540 5.32939 2.98984 2.11653 3.92293 3.73745 3.22021
4.07862 2.53195 3.24833 4.38349 3.22685 4.13028 2.61962 2.93293
2.93333 3.17639 3.69401 5.78865 1.44475 373 y - - -
2.68618 4.42225 2.77519 2.73123 3.46354 2.40513 3.72494
3.29354 2.67741 2.69355 4.24690 2.90347 2.73739 3.18146 2.89801
2.37887 2.77519 2.98518 4.58477 3.61503
0.00877 5.13716 5.85950 0.61958 0.77255 0.48576 0.95510
234 2.93485 3.69155 2.48446 2.32822 3.94704 2.98529 3.89738
4.26163 2.28577 3.32602 4.47845 1.75624 3.62780 2.51396 2.92566
2.72429 2.95104 3.82549 5.87169 4.46295 374 n - - -
2.68627 4.42234 2.77510 2.73132 3.46363 2.40509 3.72504
3.29363 2.67750 2.69364 4.24699 2.90356 2.73713 3.18128 2.89789
2.37871 2.77529 2.98527 4.58486 3.61512
0.03358 3.50089 5.85950 1.49804 0.25305 0.48576 0.95510
235 2.58908 5.43378 2.34571 1.91473 4.77393 3.34189 3.40357
3.65704 2.34507 3.34513 4.47574 3.17981 4.10382 2.82369 2.09627
2.71895 2.85769 2.92049 5.86941 4.46147 383 e - - -

```
        2.68618  4.42225  2.77519  2.73123  3.46354  2.40513  3.72494
3.29354  2.67741  2.69355  4.24690  2.90347  2.73739  3.18146  2.89801
2.37887  2.77519  2.98518  4.58477  3.61503
        0.00877  5.13716  5.85950  0.61958  0.77255  0.48576  0.95510
    236  3.65960  5.03981  5.21744  4.64383  1.77732  4.81623  5.00979
2.97031  4.48310  0.95539  3.50611  4.82560  5.07823  1.93810  4.59034
4.13504  3.88034  2.70807  5.40045  4.17266    384 l - - -
        2.68618  4.42225  2.77519  2.73123  3.46354  2.40513  3.72494
3.29354  2.67741  2.69355  4.24690  2.90347  2.73739  3.18146  2.89801
2.37887  2.77519  2.98518  4.58477  3.61503
        0.00877  5.13716  5.85950  0.61958  0.77255  0.48576  0.95510
    237  2.95554  4.85535  3.60011  3.03698  3.52348  3.86801  4.09633
2.65270  1.93340  1.71662  3.94791  2.94915  4.25196  2.67589  2.88620
3.09329  2.76782  2.74917  5.41058  4.14173    385 l - - -
        2.68618  4.42225  2.77519  2.73123  3.46354  2.40513  3.72494
3.29354  2.67741  2.69355  4.24690  2.90347  2.73739  3.18146  2.89801
2.37887  2.77519  2.98518  4.58477  3.61503
        0.00877  5.13716  5.85950  0.61958  0.77255  0.48576  0.95510
    238  2.25556  3.63314  2.46188  2.49590  4.75883  3.07709  3.39748
4.23823  2.04086  3.38551  4.46628  3.18343  4.10556  2.67097  2.57711
2.52935  2.55590  3.40827  5.86149  3.61749    386 k - - -
        2.68618  4.42225  2.77519  2.73123  3.46354  2.40513  3.72494
3.29354  2.67741  2.69355  4.24690  2.90347  2.73739  3.18146  2.89801
2.37887  2.77519  2.98518  4.58477  3.61503
        0.00877  5.13716  5.85950  0.61958  0.77255  0.48576  0.95510
    239  2.58685  5.02747  3.42989  2.87031  3.35951  3.39620  4.01956
3.63322  2.11230  2.19554  3.59469  3.08244  4.19509  2.95402  2.13320
2.78540  3.17839  3.34162  5.55270  2.49342    387 k - - -
        2.68618  4.42225  2.77519  2.73123  3.46354  2.40513  3.72494
3.29354  2.67741  2.69355  4.24690  2.90347  2.73739  3.18146  2.89801
2.37887  2.77519  2.98518  4.58477  3.61503
        0.00877  5.13716  5.85950  0.61958  0.77255  0.48576  0.95510
    240  2.58897  3.76391  4.52977  3.94005  3.54315  4.09132  4.41067
1.68050  3.80416  1.95946  3.55665  4.11154  4.46433  4.01266  3.06978
2.85081  1.66271  2.67974  5.05697  3.86862    388 t - - -
        2.68618  4.42225  2.77519  2.73123  3.46354  2.40513  3.72494
3.29354  2.67741  2.69355  4.24690  2.90347  2.73739  3.18146  2.89801
2.37887  2.77519  2.98518  4.58477  3.61503
        0.00877  5.13716  5.85950  0.61958  0.77255  0.48576  0.95510
    241  2.00821  5.44153  2.94903  2.37452  4.78456  2.87116  3.89725
3.25325  1.93215  3.74589  4.48279  2.35908  4.10339  2.66494  2.71957
2.43446  3.16692  3.83109  5.87535  4.46566    389 k - - -
        2.68618  4.42225  2.77519  2.73123  3.46354  2.40513  3.72494
3.29354  2.67741  2.69355  4.24690  2.90347  2.73739  3.18146  2.89801
2.37887  2.77519  2.98518  4.58477  3.61503
        0.00877  5.13716  5.85950  0.61958  0.77255  0.48576  0.95510
    242  2.74668  5.41112  2.43457  1.70252  3.82938  3.71456  3.90319
3.38361  2.37649  3.27471  3.73651  2.78153  4.10770  2.66544  3.13400
2.53824  2.31019  3.79398  5.85253  4.45043    390 e - - -
        2.68618  4.42225  2.77519  2.73123  3.46354  2.40513  3.72494
3.29354  2.67741  2.69355  4.24690  2.90347  2.73739  3.18146  2.89801
2.37887  2.77519  2.98518  4.58477  3.61503
        0.00877  5.13716  5.85950  0.61958  0.77255  0.48576  0.95510
```

243 5.16275 6.15801 5.60827 5.54749 2.72285 5.50934 2.85647
4.92620 5.28874 4.03125 5.41686 5.08256 5.83112 5.23215 5.22893
5.00324 5.37697 4.86322 4.39842 0.25917 391 Y - - -
2.68618 4.42225 2.77519 2.73123 3.46354 2.40513 3.72494
3.29354 2.67741 2.69355 4.24690 2.90347 2.73739 3.18146 2.89801
2.37887 2.77519 2.98518 4.58477 3.61503
0.00877 5.13716 5.85950 0.61958 0.77255 0.48576 0.95510
244 2.25231 4.60447 4.48974 3.94618 3.87566 4.01432 4.59603
1.67573 3.84924 2.92841 3.86150 4.13260 4.50573 4.09012 4.07000
2.26483 1.34627 2.55449 5.38577 4.19048 392 t - - -
2.68618 4.42225 2.77519 2.73123 3.46354 2.40513 3.72494
3.29354 2.67741 2.69355 4.24690 2.90347 2.73739 3.18146 2.89801
2.37887 2.77519 2.98518 4.58477 3.61503
0.00877 5.13716 5.85950 0.61958 0.77255 0.48576 0.95510
245 2.97128 5.99688 1.75562 2.18709 5.31639 3.85011 4.27788
4.83317 3.18927 4.30137 5.09254 1.05908 4.40168 3.41363 3.75302
3.31491 2.53229 4.38279 6.44225 4.95897 393 n - - -
2.68618 4.42225 2.77519 2.73123 3.46354 2.40513 3.72494
3.29354 2.67741 2.69355 4.24690 2.90347 2.73739 3.18146 2.89801
2.37887 2.77519 2.98518 4.58477 3.61503
0.00877 5.13716 5.85950 0.61958 0.77255 0.48576 0.95510
246 3.67351 5.76270 2.92167 3.21749 3.55973 4.20796 1.08237
4.45906 3.51248 3.91923 4.90007 3.01363 4.70607 3.77369 3.95507
3.64102 3.92472 4.17736 5.14601 1.34960 394 h - - -
2.68618 4.42225 2.77519 2.73123 3.46354 2.40513 3.72494
3.29354 2.67741 2.69355 4.24690 2.90347 2.73739 3.18146 2.89801
2.37887 2.77519 2.98518 4.58477 3.61503
0.00877 5.13716 5.85950 0.61958 0.77255 0.48576 0.95510
247 2.56770 0.54067 5.40255 5.07041 4.54474 4.09695 5.53165
2.36369 4.87318 3.33633 4.46060 4.76427 4.81581 5.07425 4.93573
3.56542 3.66765 2.84958 6.23129 5.05615 395 C - - -
2.68618 4.42225 2.77519 2.73123 3.46354 2.40513 3.72494
3.29354 2.67741 2.69355 4.24690 2.90347 2.73739 3.18146 2.89801
2.37887 2.77519 2.98518 4.58477 3.61503
0.00877 5.13716 5.85950 0.61958 0.77255 0.48576 0.95510
248 2.75269 4.50992 4.23254 2.70586 3.62186 4.03772 4.32551
2.29481 3.56315 2.31242 3.23526 3.93735 4.41019 3.81445 3.35868
2.58042 2.05752 1.60521 5.11572 3.91343 396 v - - -
2.68618 4.42225 2.77519 2.73123 3.46354 2.40513 3.72494
3.29354 2.67741 2.69355 4.24690 2.90347 2.73739 3.18146 2.89801
2.37887 2.77519 2.98518 4.58477 3.61503
0.00877 5.13716 5.85950 0.61958 0.77255 0.48576 0.95510
249 2.76700 5.44057 2.48484 2.36616 4.78331 3.71002 3.39230
4.26750 2.03596 3.36232 4.48190 2.24648 4.10356 2.99449 2.41863
2.89911 2.10300 3.82997 5.87452 3.33690 397 k - - -
2.68618 4.42225 2.77519 2.73123 3.46354 2.40513 3.72494
3.29354 2.67741 2.69355 4.24690 2.90347 2.73739 3.18146 2.89801
2.37887 2.77519 2.98518 4.58477 3.61503
0.00877 5.13716 5.85950 0.61958 0.77255 0.48576 0.95510
250 3.16940 4.82396 4.09871 3.54202 3.06302 4.14129 3.49076
3.32576 3.46427 3.00705 3.93480 3.88963 4.51351 2.59833 3.78003
3.39956 1.69122 3.11300 1.43803 3.06072 398 w - - -
2.68618 4.42225 2.77519 2.73123 3.46354 2.40513 3.72494
3.29354 2.67741 2.69355 4.24690 2.90347 2.73739 3.18146 2.89801
2.37887 2.77519 2.98518 4.58477 3.61503

```
         0.00877  5.13716  5.85950  0.61958  0.77255  0.48576  0.95510
    251   5.28560  6.16332  5.98137  5.98639  2.42907  5.64555  4.28842
4.83556  5.76200  3.92593  5.34759  5.20013  5.92133  5.39413  5.53905
5.10887  5.49102  4.81424  4.35771  0.22429    399 Y - - -
         2.68618  4.42225  2.77519  2.73123  3.46354  2.40513  3.72494
3.29354  2.67741  2.69355  4.24690  2.90347  2.73739  3.18146  2.89801
2.37887  2.77519  2.98518  4.58477  3.61503
         0.00877  5.13716  5.85950  0.61958  0.77255  0.48576  0.95510
    252   3.00522  5.51559  2.74017  2.50761  4.86389  3.74587  3.46955
4.34770  2.17367  3.81689  4.55918  1.38681  4.15229  2.10281  2.70832
2.96279  3.23648  3.90876  5.93939  4.53183    400 n - - -
         2.68618  4.42225  2.77519  2.73123  3.46354  2.40513  3.72494
3.29354  2.67741  2.69355  4.24690  2.90347  2.73739  3.18146  2.89801
2.37887  2.77519  2.98518  4.58477  3.61503
         0.00877  5.13716  5.85950  0.61958  0.77255  0.48576  0.95510
    253   2.30614  5.44316  2.93681  2.15482  4.78729  3.70889  3.89604
3.79847  1.89761  3.74801  4.48409  2.49253  4.10237  2.62942  2.54573
2.46509  2.44175  3.48521  5.87638  4.46606    401 k - - -
         2.68618  4.42225  2.77519  2.73123  3.46354  2.40513  3.72494
3.29354  2.67741  2.69355  4.24690  2.90347  2.73739  3.18146  2.89801
2.37887  2.77519  2.98518  4.58477  3.61503
         0.00877  5.13716  5.85950  0.61958  0.77255  0.48576  0.95510
    254   2.78313  5.05734  3.72105  2.99396  4.80681  0.62965  4.64984
3.22062  3.59683  3.87269  4.71865  3.80405  4.44087  3.88608  3.98835
2.60992  3.48069  3.78227  6.15027  4.88513    402 G - - -
         2.68618  4.42225  2.77519  2.73123  3.46354  2.40513  3.72494
3.29354  2.67741  2.69355  4.24690  2.90347  2.73739  3.18146  2.89801
2.37887  2.77519  2.98518  4.58477  3.61503
         0.00877  5.13716  5.85950  0.61958  0.77255  0.48576  0.95510
    255   4.33325  5.69321  5.38910  5.05930  3.83090  5.26758  5.61889
3.22198  4.65521  0.26804  3.68942  5.33425  5.55152  3.63047  4.75160
4.82467  4.58292  3.51000  5.93890  4.80017    403 L - - -
         2.68618  4.42225  2.77519  2.73123  3.46354  2.40513  3.72494
3.29354  2.67741  2.69355  4.24690  2.90347  2.73739  3.18146  2.89801
2.37887  2.77519  2.98518  4.58477  3.61503
         0.00877  5.13716  5.85950  0.61958  0.77255  0.48576  0.95510
    256   2.77657  5.45126  2.59797  2.15057  3.83041  3.37319  3.90157
4.28085  2.33296  3.75603  4.49255  1.70558  4.10687  2.26586  3.13189
2.44175  2.94033  3.84131  5.88438  4.47313    404 n - - -
         2.68618  4.42225  2.77519  2.73123  3.46354  2.40513  3.72494
3.29354  2.67741  2.69355  4.24690  2.90347  2.73739  3.18146  2.89801
2.37887  2.77519  2.98518  4.58477  3.61503
         0.00877  5.13716  5.85950  0.61958  0.77255  0.48576  0.95510
    257   2.76701  5.43317  2.78083  2.26123  4.77302  3.71082  3.46760
3.00984  2.29798  2.83371  4.47523  2.54876  4.10417  2.28217  2.02599
2.74812  3.16631  3.82101  5.86893  3.77310    405 r - - -
         2.68618  4.42225  2.77519  2.73123  3.46354  2.40513  3.72494
3.29354  2.67741  2.69355  4.24690  2.90347  2.73739  3.18146  2.89801
2.37887  2.77519  2.98518  4.58477  3.61503
         0.00877  5.13716  5.85950  0.61958  0.77255  0.48576  0.95510
    258   2.32257  4.51499  4.94787  4.34653  3.12675  4.30195  4.63146
1.79905  4.16837  1.15516  3.51547  4.41337  4.64873  2.96235  4.22861
3.61150  3.36176  2.26389  5.14322  3.97708    406 l - - -
```

2.68618 4.42225 2.77519 2.73123 3.46354 2.40513 3.72494
3.29354 2.67741 2.69355 4.24690 2.90347 2.73739 3.18146 2.89801
2.37887 2.77519 2.98518 4.58477 3.61503
0.00877 5.13716 5.85950 0.61958 0.77255 0.48576 0.95510
259 2.94390 5.43321 2.94758 2.64405 4.77186 3.71923 3.06609
4.25107 1.44790 3.73419 4.47613 2.61613 4.11205 2.80090 2.34091
2.59096 3.17438 3.82066 3.71603 3.07775 407 k - - -
2.68618 4.42225 2.77519 2.73123 3.46354 2.40513 3.72494
3.29354 2.67741 2.69355 4.24690 2.90347 2.73739 3.18146 2.89801
2.37887 2.77519 2.98518 4.58477 3.61503
0.03087 5.13716 3.70816 0.61958 0.77255 0.48576 0.95510
260 2.92637 5.43687 2.69539 2.26026 4.78176 2.38074 3.88693
4.26768 2.24301 3.03926 4.47765 2.09821 3.18174 2.48014 2.87975
2.72716 2.75171 3.82742 5.86957 4.45858 408 n - - -
2.68618 4.42225 2.77519 2.73123 3.46354 2.40513 3.72494
3.29354 2.67741 2.69355 4.24690 2.90347 2.73739 3.18146 2.89801
2.37887 2.77519 2.98518 4.58477 3.61503
0.06062 5.11525 2.94102 0.61958 0.77255 0.55350 0.85551
261 2.71806 5.25047 2.93211 2.67028 4.53273 3.32744 3.90288
3.51423 2.25254 2.56004 3.59573 3.20530 3.61416 2.80394 2.66490
1.87067 2.35712 3.28990 5.72270 4.35322 409 s - - -
2.68618 4.42225 2.77519 2.73123 3.46354 2.40513 3.72494
3.29354 2.67741 2.69355 4.24690 2.90347 2.73739 3.18146 2.89801
2.37887 2.77519 2.98518 4.58477 3.61503
0.06392 5.06406 2.88958 0.61958 0.77255 0.69112 0.69517
262 2.49015 5.37752 2.72187 2.43716 3.96813 2.78669 3.84535
4.19778 2.44564 3.68012 4.42006 2.28663 2.56270 2.70626 3.07510
2.36785 2.35656 3.76475 5.81411 4.40679 410 n - - -
2.68800 4.41891 2.77485 2.73245 3.46576 2.40642 3.72617
3.29442 2.67807 2.69494 4.24228 2.90019 2.73611 3.18137 2.89865
2.37855 2.77577 2.98649 4.57822 3.60621
0.53795 0.95415 3.47649 3.00877 0.05061 0.32523 1.28145
263 2.24177 5.30330 3.23226 2.51884 4.59663 2.87243 3.47166
3.47050 2.39874 3.23596 3.26412 2.75233 3.62368 2.84451 2.92238
2.10529 2.57414 3.30223 5.76826 3.46901 527 s - - -
2.68618 4.42225 2.77519 2.73123 3.46354 2.40513 3.72494
3.29354 2.67741 2.69355 4.24690 2.90347 2.73739 3.18146 2.89801
2.37887 2.77519 2.98518 4.58477 3.61503
0.00899 5.11248 5.83483 0.61958 0.77255 0.56165 0.84460
264 2.59807 5.36416 2.79998 2.15965 3.69843 3.06305 3.90062
2.82408 2.20770 3.04340 3.74034 2.54334 3.64671 3.00707 2.56174
2.57851 2.88204 3.37966 5.81451 4.42165 528 e - - -
2.68618 4.42225 2.77519 2.73123 3.46354 2.40513 3.72494
3.29354 2.67741 2.69355 4.24690 2.90347 2.73739 3.18146 2.89801
2.37887 2.77519 2.98518 4.58477 3.61503
0.00899 5.11248 5.83483 0.61958 0.77255 0.56165 0.84460
265 2.76737 5.43120 2.14859 2.46864 4.77475 3.36392 3.88552
3.68635 2.32968 3.73583 4.47228 2.25324 4.09169 2.30417 2.89193
2.22474 2.57263 3.37454 5.86477 4.45469 529 d - - -
2.68618 4.42225 2.77519 2.73123 3.46354 2.40513 3.72494
3.29354 2.67741 2.69355 4.24690 2.90347 2.73739 3.18146 2.89801
2.37887 2.77519 2.98518 4.58477 3.61503
0.00899 5.11248 5.83483 0.61958 0.77255 0.56165 0.84460

```
    266   5.13739  6.12606  5.96598  5.90679  2.51134  5.48188  4.50135
4.69602  5.65481  3.79844  5.21673  5.31223  5.84505  5.46173  5.49521
5.12069  5.38519  4.71356  0.27992  2.84340    530 W - - -
          2.68618  4.42225  2.77519  2.73123  3.46354  2.40513  3.72494
3.29354  2.67741  2.69355  4.24690  2.90347  2.73739  3.18146  2.89801
2.37887  2.77519  2.98518  4.58477  3.61503
          0.00899  5.11248  5.83483  0.61958  0.77255  0.56165  0.84460
    267   2.95078  4.63612  2.49820  3.31838  3.33700  3.94564  4.20476
2.39558  3.26704  2.15704  3.25290  1.86990  4.32374  3.56169  3.21086
3.19487  2.92429  2.19259  5.22354  3.99564    531 n - - -
          2.68618  4.42225  2.77519  2.73123  3.46354  2.40513  3.72494
3.29354  2.67741  2.69355  4.24690  2.90347  2.73739  3.18146  2.89801
2.37887  2.77519  2.98518  4.58477  3.61503
          0.00899  5.11248  5.83483  0.61958  0.77255  0.44108  1.03098
    268   2.30164  5.40806  2.56321  2.38794  3.94806  3.71515  3.90388
3.29194  1.91268  3.03530  4.45287  2.39878  4.10825  3.00545  2.35512
2.71620  2.95206  3.79026  5.85023  3.78628    532 k - - -
          2.68618  4.42225  2.77519  2.73123  3.46354  2.40513  3.72494
3.29354  2.67741  2.69355  4.24690  2.90347  2.73739  3.18146  2.89801
2.37887  2.77519  2.98518  4.58477  3.61503
          0.00877  5.13716  5.85950  0.61958  0.77255  0.48576  0.95510
    269   3.45504  4.83603  5.01030  4.48509  1.35240  3.59060  4.35203
2.58435  4.30367  2.96262  3.95660  4.47658  4.86511  4.43270  3.42349
3.82727  3.68482  3.11981  4.67293  1.14028    533 y - - -
          2.68618  4.42225  2.77519  2.73123  3.46354  2.40513  3.72494
3.29354  2.67741  2.69355  4.24690  2.90347  2.73739  3.18146  2.89801
2.37887  2.77519  2.98518  4.58477  3.61503
          0.00877  5.13716  5.85950  0.61958  0.77255  0.48576  0.95510
    270   3.27565  5.46552  3.34796  3.01657  4.62425  3.92426  2.61275
4.28089  3.02300  3.29486  4.69337  0.76024  4.42324  3.44832  3.41461
3.29391  3.04796  3.92750  5.91485  4.50605    534 n - - -
          2.68618  4.42225  2.77519  2.73123  3.46354  2.40513  3.72494
3.29354  2.67741  2.69355  4.24690  2.90347  2.73739  3.18146  2.89801
2.37887  2.77519  2.98518  4.58477  3.61503
          0.00877  5.13716  5.85950  0.61958  0.77255  0.48576  0.95510
    271   2.31425  5.43704  2.96809  2.48413  3.83409  3.71024  3.50598
4.26183  2.24516  3.74071  4.47868  2.44536  4.10364  2.13214  2.01943
2.89920  2.60194  3.41909  5.87181  4.46321    535 r - - -
          2.68618  4.42225  2.77519  2.73123  3.46354  2.40513  3.72494
3.29354  2.67741  2.69355  4.24690  2.90347  2.73739  3.18146  2.89801
2.37887  2.77519  2.98518  4.58477  3.61503
          0.00877  5.13716  5.85950  0.61958  0.77255  0.48576  0.95510
    272   4.83537  5.89836  5.83915  5.69655  1.13352  5.45168  4.27089
4.49033  5.48580  3.74501  5.05531  5.08257  5.74205  5.23196  5.34182
4.85000  3.32403  4.41509  4.35958  0.67480    536 Y - - -
          2.68618  4.42225  2.77519  2.73123  3.46354  2.40513  3.72494
3.29354  2.67741  2.69355  4.24690  2.90347  2.73739  3.18146  2.89801
2.37887  2.77519  2.98518  4.58477  3.61503
          0.00877  5.13716  5.85950  0.61958  0.77255  0.48576  0.95510
    273   3.34404  3.82476  3.15430  3.10677  4.98047  4.04230  4.15781
4.38553  2.63822  3.88353  4.70581  3.57976  4.44777  2.64464  0.69645
3.34390  3.56082  4.02801  5.99642  4.72712    537 r - - -
          2.68618  4.42225  2.77519  2.73123  3.46354  2.40513  3.72494
3.29354  2.67741  2.69355  4.24690  2.90347  2.73739  3.18146  2.89801
2.37887  2.77519  2.98518  4.58477  3.61503
```

0.00877 5.13716 5.85950 0.61958 0.77255 0.48576 0.95510
274 2.91917 5.50501 3.67293 3.05614 4.86895 4.01506 4.09517
4.26182 2.38221 3.21362 4.59253 3.01323 4.39387 3.22424 0.85002
3.27584 2.63720 3.91573 5.91072 4.63916 538 r - - -
2.68618 4.42225 2.77519 2.73123 3.46354 2.40513 3.72494
3.29354 2.67741 2.69355 4.24690 2.90347 2.73739 3.18146 2.89801
2.37887 2.77519 2.98518 4.58477 3.61503
0.00877 5.13716 5.85950 0.61958 0.77255 0.48576 0.95510
275 2.93778 5.40516 2.23397 1.48862 3.45595 3.71700 3.90702
4.20580 2.38280 3.33184 4.45068 2.32657 4.11076 3.00954 3.13940
2.90841 2.93662 3.27009 5.84863 3.72834 539 e - - -
2.68618 4.42225 2.77519 2.73123 3.46354 2.40513 3.72494
3.29354 2.67741 2.69355 4.24690 2.90347 2.73739 3.18146 2.89801
2.37887 2.77519 2.98518 4.58477 3.61503
0.00877 5.13716 5.85950 0.61958 0.77255 0.48576 0.95510
276 3.33680 5.30427 5.87593 5.30232 3.71959 5.33124 5.67985
2.54562 5.13155 1.58383 0.64921 5.47112 5.48044 5.13248 5.14400
4.70280 4.25062 2.93846 5.84526 4.83534 540 M - - -
2.68618 4.42225 2.77519 2.73123 3.46354 2.40513 3.72494
3.29354 2.67741 2.69355 4.24690 2.90347 2.73739 3.18146 2.89801
2.37887 2.77519 2.98518 4.58477 3.61503
0.00877 5.13716 5.85950 0.61958 0.77255 0.48576 0.95510
277 3.11374 4.92426 4.30361 3.97629 4.56380 3.90244 4.90705
2.81633 3.90105 3.60327 4.55873 3.31020 4.57475 4.25518 4.16164
3.34207 0.50511 3.37422 6.03767 4.80004 541 T - - -
2.68618 4.42225 2.77519 2.73123 3.46354 2.40513 3.72494
3.29354 2.67741 2.69355 4.24690 2.90347 2.73739 3.18146 2.89801
2.37887 2.77519 2.98518 4.58477 3.61503
0.00877 5.13716 5.85950 0.61958 0.77255 0.48576 0.95510
278 2.88034 4.51401 4.94950 4.34683 2.81056 4.29505 4.62164
1.83094 4.16408 0.99322 3.49660 4.40952 4.64157 3.41459 4.21988
3.60468 2.77888 2.67153 5.12811 3.96379 542 l - - -
2.68618 4.42225 2.77519 2.73123 3.46354 2.40513 3.72494
3.29354 2.67741 2.69355 4.24690 2.90347 2.73739 3.18146 2.89801
2.37887 2.77519 2.98518 4.58477 3.61503
0.00877 5.13716 5.85950 0.61958 0.77255 0.48576 0.95510
279 2.42664 4.46722 4.36452 3.78033 3.20116 3.51281 4.35921
2.24335 3.67131 2.37941 2.28005 3.07858 4.43197 3.90213 3.87415
2.31201 1.90135 2.72220 5.07641 3.33488 543 t - - -
2.68618 4.42225 2.77519 2.73123 3.46354 2.40513 3.72494
3.29354 2.67741 2.69355 4.24690 2.90347 2.73739 3.18146 2.89801
2.37887 2.77519 2.98518 4.58477 3.61503
0.00877 5.13716 5.85950 0.61958 0.77255 0.48576 0.95510
280 2.35100 4.72752 5.16494 4.62452 4.00689 3.42365 5.09495
2.27186 4.48947 2.61452 3.92594 4.71848 4.95151 4.69924 4.61535
3.90647 3.61319 0.65546 5.66689 4.47130 544 V - - -
2.68618 4.42225 2.77519 2.73123 3.46354 2.40513 3.72494
3.29354 2.67741 2.69355 4.24690 2.90347 2.73739 3.18146 2.89801
2.37887 2.77519 2.98518 4.58477 3.61503
0.00877 5.13716 5.85950 0.61958 0.77255 0.48576 0.95510
281 4.67192 5.81415 6.52036 5.98616 3.69138 6.06915 6.31016
2.97054 5.78175 0.24355 3.00235 6.28467 5.93403 5.54676 5.66588
5.57399 4.86286 3.34716 6.05999 5.14708 545 L - - -

2.68618 4.42225 2.77519 2.73123 3.46354 2.40513 3.72494
3.29354 2.67741 2.69355 4.24690 2.90347 2.73739 3.18146 2.89801
2.37887 2.77519 2.98518 4.58477 3.61503
0.00877 5.13716 5.85950 0.61958 0.77255 0.48576 0.95510
282 4.87418 6.72088 0.10529 3.94379 6.39691 4.69120 5.66440
6.38014 5.02020 5.74960 6.87136 4.51981 5.40044 4.99074 5.56490
4.79167 5.26021 5.92143 7.20684 6.24927 546 D - - -
2.68618 4.42225 2.77519 2.73123 3.46354 2.40513 3.72494
3.29354 2.67741 2.69355 4.24690 2.90347 2.73739 3.18146 2.89801
2.37887 2.77519 2.98518 4.58477 3.61503
0.00877 5.13716 5.85950 0.61958 0.77255 0.48576 0.95510
283 3.49756 4.80861 5.49006 4.89938 2.50731 4.76909 5.13424
1.32111 4.71714 1.34472 3.06568 4.93336 5.05410 4.80329 4.72434
4.10517 3.22013 1.78702 5.50950 4.38198 547 i - - -
2.68618 4.42225 2.77519 2.73123 3.46354 2.40513 3.72494
3.29354 2.67741 2.69355 4.24690 2.90347 2.73739 3.18146 2.89801
2.37887 2.77519 2.98518 4.58477 3.61503
0.00877 5.13716 5.85950 0.61958 0.77255 0.48576 0.95510
284 3.13520 3.24682 5.73666 5.22136 4.27041 5.13514 5.70798
0.91531 5.10967 2.91166 3.37668 5.30101 5.41683 5.32133 5.20986
4.51812 3.91379 1.10669 6.12565 4.91440 548 i - - -
2.68618 4.42225 2.77519 2.73123 3.46354 2.40513 3.72494
3.29354 2.67741 2.69355 4.24690 2.90347 2.73739 3.18146 2.89801
2.37887 2.77519 2.98518 4.58477 3.61503
0.00877 5.13716 5.85950 0.61958 0.77255 0.48576 0.95510
285 0.68845 4.85923 4.85008 4.52110 5.24284 3.69199 5.34465
4.63589 4.46278 4.36351 5.16950 4.34604 4.50082 4.68373 4.67583
1.42160 2.63774 3.33672 6.61154 5.47642 549 A - - -
2.68618 4.42225 2.77519 2.73123 3.46354 2.40513 3.72494
3.29354 2.67741 2.69355 4.24690 2.90347 2.73739 3.18146 2.89801
2.37887 2.77519 2.98518 4.58477 3.61503
0.00877 5.13716 5.85950 0.61958 0.77255 0.48576 0.95510
286 3.27288 4.62325 5.17818 4.58269 3.65366 4.49317 4.84640
1.84811 3.60289 0.96080 3.16164 4.62913 4.82256 4.53579 4.43975
3.81439 3.10482 1.87105 5.31609 4.15593 550 l - - -
2.68618 4.42225 2.77519 2.73123 3.46354 2.40513 3.72494
3.29354 2.67741 2.69355 4.24690 2.90347 2.73739 3.18146 2.89801
2.37887 2.77519 2.98518 4.58477 3.61503
0.00877 5.13716 5.85950 0.61958 0.77255 0.48576 0.95510
287 4.58745 3.57094 5.84510 5.63492 0.49412 5.32313 4.32390
4.22930 5.40369 3.54093 4.81428 5.07069 5.63386 5.19242 5.26512
4.72426 4.81865 4.15695 1.89822 2.69107 551 F - - -
2.68618 4.42225 2.77519 2.73123 3.46354 2.40513 3.72494
3.29354 2.67741 2.69355 4.24690 2.90347 2.73739 3.18146 2.89801
2.37887 2.77519 2.98518 4.58477 3.61503
0.00877 5.13716 5.85950 0.61958 0.77255 0.48576 0.95510
288 3.06321 4.99456 4.33626 4.09025 5.09385 3.77317 5.11586
4.58890 4.09886 3.29453 5.12253 4.19041 0.70606 4.41864 4.36970
1.48965 3.56740 4.03327 6.46851 5.22651 552 p - - -
2.68618 4.42225 2.77519 2.73123 3.46354 2.40513 3.72494
3.29354 2.67741 2.69355 4.24690 2.90347 2.73739 3.18146 2.89801
2.37887 2.77519 2.98518 4.58477 3.61503
0.00877 5.13716 5.85950 0.61958 0.77255 0.48576 0.95510

289 2.97030 4.54154 4.14647 3.57094 2.62451 4.01893 4.29771
2.56592 3.49169 2.37000 3.65241 2.08137 4.39321 3.75478 3.76160
2.45247 1.72261 2.80163 5.13658 3.03012 553 t - - -
2.68618 4.42225 2.77519 2.73123 3.46354 2.40513 3.72494
3.29354 2.67741 2.69355 4.24690 2.90347 2.73739 3.18146 2.89801
2.37887 2.77519 2.98518 4.58477 3.61503
0.00877 5.13716 5.85950 0.61958 0.77255 0.48576 0.95510
290 5.22727 6.10569 6.00061 5.96936 1.76861 5.65640 3.41503
4.80242 5.73227 3.18067 5.30876 5.16151 5.90068 5.34693 5.50595
5.06550 5.41168 4.75382 4.31140 0.39695 554 Y - - -
2.68618 4.42225 2.77519 2.73123 3.46354 2.40513 3.72494
3.29354 2.67741 2.69355 4.24690 2.90347 2.73739 3.18146 2.89801
2.37887 2.77519 2.98518 4.58477 3.61503
0.00877 5.13716 5.85950 0.61958 0.77255 0.48576 0.95510
291 4.02044 6.72387 0.39047 2.88108 6.05003 4.01025 4.75650
5.75178 4.03007 5.17457 6.14045 2.20479 4.74032 3.96289 4.79849
3.83378 4.37408 5.23219 7.24375 5.61221 555 D - - -
2.68618 4.42225 2.77519 2.73123 3.46354 2.40513 3.72494
3.29354 2.67741 2.69355 4.24690 2.90347 2.73739 3.18146 2.89801
2.37887 2.77519 2.98518 4.58477 3.61503
0.00877 5.13716 5.85950 0.61958 0.77255 0.48576 0.95510
292 2.33945 4.52709 4.17371 3.59658 3.25506 3.14664 4.30577
2.43708 3.15055 2.57017 3.63837 3.89957 1.87614 3.77263 3.77500
2.75625 2.53605 2.22607 5.12918 3.08071 556 p - - -
2.68618 4.42225 2.77519 2.73123 3.46354 2.40513 3.72494
3.29354 2.67741 2.69355 4.24690 2.90347 2.73739 3.18146 2.89801
2.37887 2.77519 2.98518 4.58477 3.61503
0.00877 5.13716 5.85950 0.61958 0.77255 0.48576 0.95510
293 2.93706 4.14732 2.78980 2.39357 3.87468 3.37639 3.56273
3.44079 2.15293 3.62331 4.38918 3.21583 4.12143 3.03385 1.56968
2.69889 2.91153 3.31379 5.79646 3.37614 557 r - - -
2.68618 4.42225 2.77519 2.73123 3.46354 2.40513 3.72494
3.29354 2.67741 2.69355 4.24690 2.90347 2.73739 3.18146 2.89801
2.37887 2.77519 2.98518 4.58477 3.61503
0.00877 5.13716 5.85950 0.61958 0.77255 0.48576 0.95510
294 2.96147 4.89295 3.57426 3.00998 4.06833 3.86238 4.08391
2.72598 2.00159 1.92289 3.22884 3.48267 4.24652 2.44149 2.37770
2.67062 3.19216 2.70128 5.44150 4.16724 558 l - - -
2.68618 4.42225 2.77519 2.73123 3.46354 2.40513 3.72494
3.29354 2.67741 2.69355 4.24690 2.90347 2.73739 3.18146 2.89801
2.37887 2.77519 2.98518 4.58477 3.61503
0.00877 5.13716 5.85950 0.61958 0.77255 0.48576 0.95510
295 5.33745 6.17170 6.03216 6.05240 1.97985 5.69104 4.26220
4.85715 5.81995 3.93243 5.35624 5.20027 5.94246 5.39938 5.56709
5.13007 5.52678 4.83774 4.32779 0.28736 559 Y - - -
2.68618 4.42225 2.77519 2.73123 3.46354 2.40513 3.72494
3.29354 2.67741 2.69355 4.24690 2.90347 2.73739 3.18146 2.89801
2.37887 2.77519 2.98518 4.58477 3.61503
0.00877 5.13716 5.85950 0.61958 0.77255 0.48576 0.95510
296 2.96067 5.42228 2.40596 2.65913 4.75003 3.72808 3.92718
4.22228 2.39832 3.33564 4.47259 2.97490 1.41387 3.03190 2.92168
2.48034 2.77908 3.80465 5.86873 4.46964 560 p - - -
2.68618 4.42225 2.77519 2.73123 3.46354 2.40513 3.72494
3.29354 2.67741 2.69355 4.24690 2.90347 2.73739 3.18146 2.89801
2.37887 2.77519 2.98518 4.58477 3.61503

```
        0.00877  5.13716  5.85950  0.61958  0.77255  0.48576  0.95510
    297   2.58927  4.93116  3.51330  2.77323  4.11566  3.83698  4.05840
2.04281  2.30680  2.42547  2.75610  2.69863  3.14689  3.00154  3.07433
3.05470  2.73617  3.23238  5.47384  3.60264    561 i - - -
        2.68618  4.42225  2.77519  2.73123  3.46354  2.40513  3.72494
3.29354  2.67741  2.69355  4.24690  2.90347  2.73739  3.18146  2.89801
2.37887  2.77519  2.98518  4.58477  3.61503
        0.00877  5.13716  5.85950  0.61958  0.77255  0.48576  0.95510
    298   2.42974  5.42705  2.97862  2.41361  3.82816  2.39895  3.89964
4.24479  2.24932  3.31804  4.46979  3.18222  2.26889  2.51229  2.75982
2.43614  2.92624  3.81349  5.86443  4.45825    562 k - - -
        2.68635  4.42263  2.77518  2.73093  3.46347  2.40511  3.72532
3.29317  2.67698  2.69357  4.24727  2.90359  2.73737  3.18150  2.89838
2.37897  2.77537  2.98512  4.58514  3.61449
        0.18508  1.79511  5.85950  1.49289  0.25453  0.48576  0.95510
    299   2.86219  4.50715  4.86224  4.27507  3.65224  4.27855  4.64287
1.95556  4.11769  2.21218  3.64313  4.37370  4.64564  4.30090  4.21768
2.86788  1.48384  1.56077  5.21135  4.02585    575 t - - -
        2.68618  4.42225  2.77519  2.73123  3.46354  2.40513  3.72494
3.29354  2.67741  2.69355  4.24690  2.90347  2.73739  3.18146  2.89801
2.37887  2.77519  2.98518  4.58477  3.61503
        0.00877  5.13716  5.85950  0.61958  0.77255  0.48576  0.95510
    300   2.94083  5.44052  3.19346  2.49449  4.78278  3.71501  3.47007
4.26502  1.43757  3.74351  4.48236  2.44410  3.59339  2.51039  2.71500
2.60604  2.72158  2.93263  5.87388  4.46712    576 k - - -
        2.68618  4.42225  2.77519  2.73123  3.46354  2.40513  3.72494
3.29354  2.67741  2.69355  4.24690  2.90347  2.73739  3.18146  2.89801
2.37887  2.77519  2.98518  4.58477  3.61503
        0.00877  5.13716  5.85950  0.61958  0.77255  0.48576  0.95510
    301   2.38504  4.41573  4.57942  3.98565  3.09682  3.16155  4.41170
2.32602  3.84225  2.29538  2.85970  4.13472  3.77665  4.03850  3.97540
2.10856  2.01321  2.02519  5.03118  3.84493    577 t - - -
        2.68618  4.42225  2.77519  2.73123  3.46354  2.40513  3.72494
3.29354  2.67741  2.69355  4.24690  2.90347  2.73739  3.18146  2.89801
2.37887  2.77519  2.98518  4.58477  3.61503
        0.00877  5.13716  5.85950  0.61958  0.77255  0.48576  0.95510
    302   3.66863  6.34598  2.46266  0.87723  5.63360  3.91771  4.45199
5.18469  3.45630  4.62833  5.46717  3.36093  4.54119  1.35204  4.05620
3.53032  3.95359  4.72038  6.76121  5.22270    578 e - - -
        2.68618  4.42225  2.77519  2.73123  3.46354  2.40513  3.72494
3.29354  2.67741  2.69355  4.24690  2.90347  2.73739  3.18146  2.89801
2.37887  2.77519  2.98518  4.58477  3.61503
        0.00877  5.13716  5.85950  0.61958  0.77255  0.48576  0.95510
    303   4.04864  5.31732  5.92987  5.38074  3.77503  5.36586  5.77804
2.15617  5.20970  0.44835  3.53812  5.53910  5.52837  5.21696  5.22109
4.75695  2.89407  2.84743  5.92375  4.91174    579 L - - -
        2.68624  4.42231  2.77525  2.73129  3.46360  2.40519  3.72500
3.29360  2.67747  2.69272  4.24696  2.90352  2.73745  3.18152  2.89807
2.37893  2.77525  2.98524  4.58483  3.61509
        0.14544  2.02110  5.85950  0.16249  1.89730  0.48576  0.95510
    304   3.01820  4.93484  4.80066  4.70359  5.51835  3.72178  5.57440
4.96420  4.72980  4.69914  5.50407  4.44376  4.57074  4.95038  4.86458
2.24369  0.35151  4.20902  6.87276  5.75590    581 T - - -
```

```
         2.68618  4.42225  2.77519  2.73123  3.46354  2.40513  3.72494
3.29354  2.67741  2.69355  4.24690  2.90347  2.73739  3.18146  2.89801
2.37887  2.77519  2.98518  4.58477  3.61503
         0.00877  5.13716  5.85950  0.61958  0.77255  0.48576  0.95510
    305  5.01913  6.46576  5.62225  5.05888  6.19005  5.05193  5.52378
5.99298  3.77885  5.27231  6.33721  5.29067  5.58856  4.78853  0.10314
5.12353  5.26619  5.67298  6.82299  6.01708    582 R - - -
         2.68618  4.42225  2.77519  2.73123  3.46354  2.40513  3.72494
3.29354  2.67741  2.69355  4.24690  2.90347  2.73739  3.18146  2.89801
2.37887  2.77519  2.98518  4.58477  3.61503
         0.00877  5.13716  5.85950  0.61958  0.77255  0.48576  0.95510
    306  2.93805  5.35354  2.69883  1.43441  4.66093  3.34678  3.91855
3.20785  2.07410  3.64253  3.25219  3.21041  4.11949  3.02867  3.15541
2.91955  2.57081  3.03201  5.80958  4.42261    583 e - - -
         2.68618  4.42225  2.77519  2.73123  3.46354  2.40513  3.72494
3.29354  2.67741  2.69355  4.24690  2.90347  2.73739  3.18146  2.89801
2.37887  2.77519  2.98518  4.58477  3.61503
         0.00877  5.13716  5.85950  0.61958  0.77255  0.48576  0.95510
    307  3.98111  5.14957  6.12862  5.67357  4.40762  5.67516  6.44975
0.91001  5.63905  1.84576  4.11409  5.84449  5.83222  5.84282  5.78415
5.13134  4.23676  1.09471  6.62836  5.45521    584 i - - -
         2.68618  4.42225  2.77519  2.73123  3.46354  2.40513  3.72494
3.29354  2.67741  2.69355  4.24690  2.90347  2.73739  3.18146  2.89801
2.37887  2.77519  2.98518  4.58477  3.61503
         0.00877  5.13716  5.85950  0.61958  0.77255  0.48576  0.95510
    308  4.95554  5.94945  5.92237  5.80766  1.85891  5.53149  4.24959
4.59167  5.58144  3.82096  5.13419  5.10955  5.79421  5.26956  5.39864
4.92478  3.45443  4.51306  3.23871  0.41387    585 Y - - -
         2.68618  4.42225  2.77519  2.73123  3.46354  2.40513  3.72494
3.29354  2.67741  2.69355  4.24690  2.90347  2.73739  3.18146  2.89801
2.37887  2.77519  2.98518  4.58477  3.61503
         0.00877  5.13716  5.85950  0.61958  0.77255  0.48576  0.95510
    309  2.98157  4.81245  3.79552  3.24854  4.05265  3.88782  4.24804
3.05387  3.19155  3.10818  3.45162  3.66168  4.32628  3.17720  3.56379
1.94654  1.05112  3.15039  5.46666  4.21817    586 t - - -
         2.68623  4.42262  2.77500  2.73112  3.46288  2.40550  3.72532
3.29322  2.67778  2.69298  4.24727  2.90324  2.73740  3.18150  2.89838
2.37911  2.77521  2.98491  4.58514  3.61445
         0.19563  1.74391  5.85950  1.43645  0.27151  0.48576  0.95510
    310  3.12925  5.66106  1.12567  2.07163  4.99081  3.77361  4.05631
4.48487  2.85477  3.96100  4.71695  2.54908  4.23168  2.89340  3.37029
2.47912  3.06414  3.38772  6.09623  4.66247    593 d - - -
         2.68618  4.42225  2.77519  2.73123  3.46354  2.40513  3.72494
3.29354  2.67741  2.69355  4.24690  2.90347  2.73739  3.18146  2.89801
2.37887  2.77519  2.98518  4.58477  3.61503
         0.17597  5.13716  1.86124  0.61958  0.77255  0.48576  0.95510
    311  2.80629  4.58169  4.53046  3.95704  3.64031  4.22581  4.56995
2.46235  3.84620  2.11247  3.14144  4.19816  1.09691  4.07934  4.05251
3.52391  3.34840  2.23015  5.26039  4.07952    594 p - - -
         2.68618  4.42225  2.77519  2.73123  3.46354  2.40513  3.72494
3.29354  2.67741  2.69355  4.24690  2.90347  2.73739  3.18146  2.89801
2.37887  2.77519  2.98518  4.58477  3.61503
         0.03653  4.97154  3.54255  0.61958  0.77255  0.88698  0.53085
```

312 2.42097 3.54656 4.84977 4.24199 3.40955 4.10669 4.44574
1.70473 4.04156 2.29904 3.05065 4.25309 4.46727 4.18474 4.07019
3.41855 2.81091 1.46531 4.95136 2.76748 595 v - - -
2.68618 4.42225 2.77519 2.73123 3.46354 2.40513 3.72494
3.29354 2.67741 2.69355 4.24690 2.90347 2.73739 3.18146 2.89801
2.37887 2.77519 2.98518 4.58477 3.61503
0.01063 4.94563 5.66798 0.61958 0.77255 0.49691 0.93754
313 2.44094 5.06199 3.34303 2.78514 3.75362 1.87617 3.96170
3.69673 2.61618 2.79447 4.13783 2.47205 3.56949 2.86117 3.23378
2.43003 3.14243 2.51983 5.57539 4.25163 596 g - - -
2.68618 4.42225 2.77519 2.73123 3.46354 2.40513 3.72494
3.29354 2.67741 2.69355 4.24690 2.90347 2.73739 3.18146 2.89801
2.37887 2.77519 2.98518 4.58477 3.61503
0.00943 5.06420 5.78654 0.61958 0.77255 0.69080 0.69550
314 2.45524 4.48861 4.14155 2.93894 2.40502 3.98702 3.14290
2.68798 3.48084 2.38710 2.95440 3.38201 4.36036 3.30337 2.99088
3.25648 2.71147 2.44735 3.96864 2.35198 597 y - - -
2.68618 4.42225 2.77519 2.73123 3.46354 2.40513 3.72494
3.29354 2.67741 2.69355 4.24690 2.90347 2.73739 3.18146 2.89801
2.37887 2.77519 2.98518 4.58477 3.61503
0.08569 5.06420 2.57958 0.61958 0.77255 0.69080 0.69550
315 2.24962 5.07186 3.27997 2.37986 3.66954 3.33352 3.48605
2.52187 2.71522 2.84090 4.14301 2.36062 4.09869 2.79270 3.17849
2.29374 2.53364 3.40277 5.57562 4.24166 598 a - - -
2.68618 4.42225 2.77519 2.73123 3.46354 2.40513 3.72494
3.29354 2.67741 2.69355 4.24690 2.90347 2.73739 3.18146 2.89801
2.37887 2.77519 2.98518 4.58477 3.61503
0.10006 4.98868 2.42582 0.61958 0.77255 0.85475 0.55406
316 2.83699 5.18908 2.86089 2.59801 3.79478 2.58449 3.00939
3.22346 2.59648 3.46825 4.24616 2.05666 3.54958 2.95283 2.63030
2.64506 2.59871 3.19665 5.65969 3.63151 599 n - - -
2.68618 4.42225 2.77519 2.73123 3.46354 2.40513 3.72494
3.29354 2.67741 2.69355 4.24690 2.90347 2.73739 3.18146 2.89801
2.37887 2.77519 2.98518 4.58477 3.61503
0.01113 4.89974 5.62209 0.61958 0.77255 0.61488 0.77806
317 2.55984 5.38031 2.68606 2.29115 4.72131 2.79390 3.84210
4.20442 2.14087 3.35404 4.42204 2.89866 3.53745 2.59945 2.51743
2.31071 2.67209 3.44126 4.22623 3.74173 600 k - - -
2.68618 4.42225 2.77519 2.73123 3.46354 2.40513 3.72494
3.29354 2.67741 2.69355 4.24690 2.90347 2.73739 3.18146 2.89801
2.37887 2.77519 2.98518 4.58477 3.61503
0.10445 5.00920 2.38047 0.61958 0.77255 0.70608 0.68038
318 2.68370 5.34570 2.84766 2.40962 4.68346 2.48402 3.38264
4.16433 2.55430 3.32843 4.38830 2.33860 3.23297 2.42838 2.80997
2.63015 2.42831 3.73241 5.78276 3.23491 601 n - - -
2.68618 4.42225 2.77519 2.73123 3.46354 2.40513 3.72494
3.29354 2.67741 2.69355 4.24690 2.90347 2.73739 3.18146 2.89801
2.37887 2.77519 2.98518 4.58477 3.61503
0.04112 4.94238 3.40682 0.61958 0.77255 0.62020 0.77184
319 2.88063 5.04815 2.71620 2.74404 2.99148 2.99876 3.92597
2.80448 2.73593 2.75831 3.57226 2.49236 3.62311 2.54369 2.93833
2.45268 2.69245 3.37471 5.55740 3.28110 602 s - - -
2.68720 4.42086 2.77545 2.73207 3.46320 2.40531 3.72546
3.29281 2.67837 2.69376 4.24749 2.90277 2.73687 3.18167 2.89791
2.37861 2.77535 2.98587 4.57547 3.61277

```
          1.46156  0.76623  1.19283  1.92326  0.15798  0.69201  0.69429
    320   2.05918  4.98122  3.14879  2.59092  4.22547  2.79873  3.78904
2.82342  2.38455  3.24220  4.05037  2.84927  3.97711  2.69976  2.42829
2.55299  2.99164  3.00986  5.47951  3.57514    624 a - - -
          2.68618  4.42225  2.77519  2.73123  3.46354  2.40513  3.72494
3.29354  2.67741  2.69355  4.24690  2.90347  2.73739  3.18146  2.89801
2.37887  2.77519  2.98518  4.58477  3.61503
          0.05280  4.68856  3.16468  0.61958  0.77255  1.03997  0.43613
    321   2.24641  3.73354  3.22660  2.48990  4.10917  3.24480  3.83316
3.21139  2.65770  2.87194  3.96995  2.68933  2.24390  2.68013  3.11063
2.63744  3.00375  2.89286  5.41103  4.09448    625 p - - -
          2.68618  4.42225  2.77519  2.73123  3.46354  2.40513  3.72494
3.29354  2.67741  2.69355  4.24690  2.90347  2.73739  3.18146  2.89801
2.37887  2.77519  2.98518  4.58477  3.61503
          0.01329  4.72353  5.44588  0.61958  0.77255  0.30560  1.33439
    322   2.72960  5.41514  2.43884  2.61013  4.75550  3.03107  3.87844
4.23823  2.46696  3.41173  4.45784  2.27635  3.49954  2.61571  3.10871
1.85094  2.26864  3.80342  5.85122  4.44278    626 s - - -
          2.68618  4.42225  2.77519  2.73123  3.46354  2.40513  3.72494
3.29354  2.67741  2.69355  4.24690  2.90347  2.73739  3.18146  2.89801
2.37887  2.77519  2.98518  4.58477  3.61503
          0.00924  5.08413  5.80647  0.61958  0.77255  0.64024  0.74901
    323   2.77451  4.38050  4.86139  4.25453  1.49506  4.15944  4.48972
2.00442  4.06230  1.99404  3.47140  4.28894  3.59765  3.55678  4.10566
3.46806  3.22686  1.91364  5.00638  3.83006    627 f - - -
          2.68619  4.42226  2.77521  2.73124  3.46355  2.40514  3.72496
3.29355  2.67742  2.69356  4.24691  2.90322  2.73741  3.18148  2.89802
2.37888  2.77521  2.98520  4.58478  3.61504
          0.04136  3.28314  5.80647  0.37170  1.16977  0.50598  0.92360
    324   2.32586  5.42992  2.41228  2.26833  4.77259  3.69866  3.88644
3.59250  2.45204  3.73418  4.47125  2.05983  3.53677  2.50220  3.11482
2.12114  2.91191  3.81928  5.86400  3.90252    629 n - - -
          2.68618  4.42225  2.77519  2.73123  3.46354  2.40513  3.72494
3.29354  2.67741  2.69355  4.24690  2.90347  2.73739  3.18146  2.89801
2.37887  2.77519  2.98518  4.58477  3.61503
          0.00898  5.11254  5.83488  0.61958  0.77255  0.56147  0.84483
    325   2.34234  5.43051  2.37699  1.82394  4.77379  3.35974  3.88563
3.73086  2.49831  3.73502  4.47166  3.16639  4.09179  2.37348  2.53004
2.31451  2.76421  3.82019  5.86424  3.82609    630 e - - -
          2.68618  4.42225  2.77519  2.73123  3.46354  2.40513  3.72494
3.29354  2.67741  2.69355  4.24690  2.90347  2.73739  3.18146  2.89801
2.37887  2.77519  2.98518  4.58477  3.61503
          0.00898  5.11254  5.83488  0.61958  0.77255  0.56147  0.84483
    326   2.96193  4.40237  3.00842  3.97621  3.14480  4.08693  4.39975
1.54112  3.83221  1.97454  2.71810  3.21191  4.45447  3.51548  3.96421
3.38101  2.90861  2.10148  5.01769  3.29552    631 i - - -
          2.68618  4.42225  2.77519  2.73123  3.46354  2.40513  3.72494
3.29354  2.67741  2.69355  4.24690  2.90347  2.73739  3.18146  2.89801
2.37887  2.77519  2.98518  4.58477  3.61503
          0.00898  5.11254  5.83488  0.61958  0.77255  0.44117  1.03081
    327   3.64055  6.25150  2.06183  0.57592  5.51420  3.92004  4.45054
5.03863  3.46165  3.01814  5.37929  3.37313  4.53682  3.61206  4.06265
3.51763  3.92547  4.60817  6.68609  5.16552    632 E - - -
```

```
          2.68618  4.42225  2.77519  2.73123  3.46354  2.40513  3.72494
3.29354  2.67741  2.69355  4.24690  2.90347  2.73739  3.18146  2.89801
2.37887  2.77519  2.98518  4.58477  3.61503
          0.03121  5.13716  3.69488  0.61958  0.77255  0.48576  0.95510
    328   2.35541  5.39896  2.60787  2.33537  3.79972  3.70613  3.89592
4.20244  2.46878  3.36608  4.44406  1.58349  3.58709  2.99784  3.12793
2.32390  3.15873  3.78081  5.84150  3.71960    633 n - - -
          2.68618  4.42225  2.77519  2.73123  3.46354  2.40513  3.72494
3.29354  2.67741  2.69355  4.24690  2.90347  2.73739  3.18146  2.89801
2.37887  2.77519  2.98518  4.58477  3.61503
          0.06531  5.11491  2.86085  0.61958  0.77255  0.44506  1.02383
    329   2.15883  5.38226  2.93628  2.47848  4.71100  3.69126  3.04700
3.24096  2.14346  3.68055  3.30961  2.78044  4.08433  2.78251  2.71827
2.22363  2.56621  3.76409  5.82483  3.66690    634 k - - -
          2.68618  4.42225  2.77519  2.73123  3.46354  2.40513  3.72494
3.29354  2.67741  2.69355  4.24690  2.90347  2.73739  3.18146  2.89801
2.37887  2.77519  2.98518  4.58477  3.61503
          0.03091  5.08258  3.72021  0.61958  0.77255  0.39805  1.11362
    330   2.42175  4.38233  4.68170  3.46204  2.92636  3.52854  4.42456
2.45219  3.91798  1.34887  3.49962  4.18206  4.47114  4.09471  4.01280
2.62396  2.92490  2.19677  4.99993  2.78171    635 l - - -
          2.68618  4.42225  2.77519  2.73123  3.46354  2.40513  3.72494
3.29354  2.67741  2.69355  4.24690  2.90347  2.73739  3.18146  2.89801
2.37887  2.77519  2.98518  4.58477  3.61503
          0.00895  5.11686  5.83921  0.61958  0.77255  0.44832  1.01805
    331   2.98259  3.60387  4.45063  3.86519  3.57133  4.08367  4.39698
1.61844  3.18021  2.65768  3.58044  3.43260  3.64935  3.96564  3.92371
3.37223  1.65458  1.92920  5.08148  3.88947    636 i - - -
          2.68618  4.42225  2.77519  2.73123  3.46354  2.40513  3.72494
3.29354  2.67741  2.69355  4.24690  2.90347  2.73739  3.18146  2.89801
2.37887  2.77519  2.98518  4.58477  3.61503
          0.00877  5.13716  5.85950  0.61958  0.77255  0.48576  0.95510
    332   2.75114  5.31342  3.25044  2.69314  4.60610  3.73809  3.17413
3.48965  2.34229  2.98213  4.36861  2.76461  3.00794  3.04712  1.48181
2.73659  2.89247  3.30984  5.77826  4.40311    637 r - - -
          2.68618  4.42225  2.77519  2.73123  3.46354  2.40513  3.72494
3.29354  2.67741  2.69355  4.24690  2.90347  2.73739  3.18146  2.89801
2.37887  2.77519  2.98518  4.58477  3.61503
          0.00877  5.13716  5.85950  0.61958  0.77255  0.48576  0.95510
    333   2.73108  5.45012  2.96847  2.04648  4.79471  3.07558  3.89959
4.28067  2.29150  3.75518  4.49117  2.74634  1.99730  2.72912  2.92085
2.12281  2.96955  3.84055  5.88291  4.47165    638 p - - -
          2.68618  4.42225  2.77519  2.73123  3.46354  2.40513  3.72494
3.29354  2.67741  2.69355  4.24690  2.90347  2.73739  3.18146  2.89801
2.37887  2.77519  2.98518  4.58477  3.61503
          0.00877  5.13716  5.85950  0.61958  0.77255  0.48576  0.95510
    334   2.77480  5.14193  3.55800  2.84856  4.74168  3.81985  4.47449
4.13986  3.35025  3.15169  4.63459  3.67014  0.73520  3.68146  3.75589
2.51424  3.46378  3.76087  6.05469  4.76398    639 p - - -
          2.68618  4.42225  2.77519  2.73123  3.46354  2.40513  3.72494
3.29354  2.67741  2.69355  4.24690  2.90347  2.73739  3.18146  2.89801
2.37887  2.77519  2.98518  4.58477  3.61503
          0.00877  5.13716  5.85950  0.61958  0.77255  0.48576  0.95510
```

335 2.93609 5.42134 2.54123 2.47122 4.75579 2.68574 1.98804
4.23437 2.64083 3.72207 3.69897 3.18485 4.10677 2.60379 2.93274
2.10444 2.57295 3.80627 5.86038 3.79873 640 h - - -
2.68618 4.42225 2.77519 2.73123 3.46354 2.40513 3.72494
3.29354 2.67741 2.69355 4.24690 2.90347 2.73739 3.18146 2.89801
2.37887 2.77519 2.98518 4.58477 3.61503
0.00877 5.13716 5.85950 0.61958 0.77255 0.48576 0.95510
336 2.98067 4.40753 4.64885 4.05184 2.64837 4.11350 3.69167
2.46275 3.89352 1.01062 3.52297 3.56167 4.48007 4.08051 3.44396
3.41109 2.71952 2.65743 5.01605 3.35998 641 l - - -
2.68618 4.42225 2.77519 2.73123 3.46354 2.40513 3.72494
3.29354 2.67741 2.69355 4.24690 2.90347 2.73739 3.18146 2.89801
2.37887 2.77519 2.98518 4.58477 3.61503
0.00877 5.13716 5.85950 0.61958 0.77255 0.48576 0.95510
337 3.04695 4.47930 4.68984 4.10566 1.11353 4.16891 4.47816
2.86937 3.94549 2.60851 3.21169 4.22837 4.54157 3.43187 4.06241
2.82204 3.28399 1.76929 5.05511 3.83057 642 f - - -
2.68618 4.42225 2.77519 2.73123 3.46354 2.40513 3.72494
3.29354 2.67741 2.69355 4.24690 2.90347 2.73739 3.18146 2.89801
2.37887 2.77519 2.98518 4.58477 3.61503
0.00877 5.13716 5.85950 0.61958 0.77255 0.48576 0.95510
338 2.96067 5.46912 2.30740 2.18335 4.81169 3.71968 3.91690
4.29694 2.35781 3.77289 4.51187 2.90205 4.12057 3.01552 2.41456
2.40178 1.59867 3.85871 5.90208 4.49060 643 t - - -
2.68618 4.42225 2.77519 2.73123 3.46354 2.40513 3.72494
3.29354 2.67741 2.69355 4.24690 2.90347 2.73739 3.18146 2.89801
2.37887 2.77519 2.98518 4.58477 3.61503
0.00877 5.13716 5.85950 0.61958 0.77255 0.48576 0.95510
339 2.96350 4.61935 3.95471 3.09548 2.74601 3.97123 3.56969
2.55336 3.32522 2.82587 3.72590 3.29366 4.34835 2.99368 2.81705
3.22475 2.89132 2.68744 1.59280 3.24050 644 w - - -
2.68618 4.42225 2.77519 2.73123 3.46354 2.40513 3.72494
3.29354 2.67741 2.69355 4.24690 2.90347 2.73739 3.18146 2.89801
2.37887 2.77519 2.98518 4.58477 3.61503
0.00877 5.13716 5.85950 0.61958 0.77255 0.48576 0.95510
340 3.40397 5.42979 6.13289 5.55597 3.14015 5.56878 5.85987
2.22013 5.41470 0.40431 3.45190 5.72937 5.62557 5.29207 5.36202
4.95577 4.41334 3.03124 5.88784 4.89736 645 L - - -
2.68618 4.42225 2.77519 2.73123 3.46354 2.40513 3.72494
3.29354 2.67741 2.69355 4.24690 2.90347 2.73739 3.18146 2.89801
2.37887 2.77519 2.98518 4.58477 3.61503
0.00877 5.13716 5.85950 0.61958 0.77255 0.48576 0.95510
341 2.93522 5.43338 2.49197 2.49114 3.84293 3.71076 3.89839
4.25552 2.03689 3.73641 4.47544 2.15980 4.10417 2.99644 2.23790
2.10540 2.75341 3.41390 5.86915 3.85390 646 k - - -
2.68618 4.42225 2.77519 2.73123 3.46354 2.40513 3.72494
3.29354 2.67741 2.69355 4.24690 2.90347 2.73739 3.18146 2.89801
2.37887 2.77519 2.98518 4.58477 3.61503
0.00877 5.13716 5.85950 0.61958 0.77255 0.48576 0.95510
342 2.93582 5.44639 2.94893 2.07107 4.79136 2.71505 3.89639
4.27731 2.27767 3.75160 4.48714 2.45058 4.10270 2.47590 2.73785
2.01784 2.57599 3.83699 5.87905 3.40349 647 s - - -
2.68618 4.42225 2.77519 2.73123 3.46354 2.40513 3.72494
3.29354 2.67741 2.69355 4.24690 2.90347 2.73739 3.18146 2.89801
2.37887 2.77519 2.98518 4.58477 3.61503

```
          0.00877  5.13716  5.85950  0.61958  0.77255  0.48576  0.95510
    343   2.82969  4.46817  5.01697  4.40985  3.18627  4.28098  4.61855
1.52947  4.21173  1.30306  2.84286  4.42699  4.63176  4.34579  4.23843
2.59753  3.33033  2.30835  5.10759  3.93862    648 l - - -
          2.68618  4.42225  2.77519  2.73123  3.46354  2.40513  3.72494
3.29354  2.67741  2.69355  4.24690  2.90347  2.73739  3.18146  2.89801
2.37887  2.77519  2.98518  4.58477  3.61503
          0.00877  5.13716  5.85950  0.61958  0.77255  0.48576  0.95510
    344   2.93583  5.44361  2.36774  2.08113  4.78743  3.34333  3.89694
4.27254  2.11604  3.36552  4.48466  2.43589  4.10313  2.99375  2.54774
2.68707  2.08057  3.83359  5.87696  3.50319    649 t - - -
          2.68618  4.42225  2.77519  2.73123  3.46354  2.40513  3.72494
3.29354  2.67741  2.69355  4.24690  2.90347  2.73739  3.18146  2.89801
2.37887  2.77519  2.98518  4.58477  3.61503
          0.00877  5.13716  5.85950  0.61958  0.77255  0.48576  0.95510
    345   2.80292  4.46360  5.04122  4.43314  1.63420  3.61042  4.62409
1.49748  4.23017  1.85654  3.13364  4.43849  4.63554  4.36084  4.24896
3.59932  3.33015  2.28014  5.10684  3.93667    650 i - - -
          2.68618  4.42225  2.77519  2.73123  3.46354  2.40513  3.72494
3.29354  2.67741  2.69355  4.24690  2.90347  2.73739  3.18146  2.89801
2.37887  2.77519  2.98518  4.58477  3.61503
          0.00877  5.13716  5.85950  0.61958  0.77255  0.48576  0.95510
    346   2.94155  5.17462  3.06905  2.76505  3.32544  3.36906  2.77392
3.84417  2.75948  3.43706  4.24256  3.07882  3.61629  2.66682  2.96763
2.47813  2.93340  3.51154  4.30884  1.64064    651 y - - -
          2.68618  4.42225  2.77519  2.73123  3.46354  2.40513  3.72494
3.29354  2.67741  2.69355  4.24690  2.90347  2.73739  3.18146  2.89801
2.37887  2.77519  2.98518  4.58477  3.61503
          0.00877  5.13716  5.85950  0.61958  0.77255  0.48576  0.95510
    347   2.74837  4.68843  3.82324  2.79933  2.86038  3.93508  4.18718
2.93129  3.21196  2.90034  3.28851  3.28202  4.31472  3.51522  3.24517
2.55474  1.31826  2.75286  5.26917  3.43183    652 t - - -
          2.68618  4.42225  2.77519  2.73123  3.46354  2.40513  3.72494
3.29354  2.67741  2.69355  4.24690  2.90347  2.73739  3.18146  2.89801
2.37887  2.77519  2.98518  4.58477  3.61503
          0.00877  5.13716  5.85950  0.61958  0.77255  0.48576  0.95510
    348   2.72725  5.42257  2.80230  2.37174  4.75793  2.42491  3.90067
3.30439  2.13106  3.01973  4.46579  2.46675  4.10584  3.00020  2.45791
2.24286  3.16639  3.80798  5.86108  3.40888    653 k - - -
          2.68618  4.42225  2.77519  2.73123  3.46354  2.40513  3.72494
3.29354  2.67741  2.69355  4.24690  2.90347  2.73739  3.18146  2.89801
2.37887  2.77519  2.98518  4.58477  3.61503
          0.06234  5.13716  2.90847  0.61958  0.77255  0.48576  0.95510
    349   2.30509  5.22706  3.02928  2.69612  3.76054  3.72354  3.92053
3.19458  2.39783  3.22899  3.68047  2.52571  3.53840  2.50581  2.49603
2.56709  2.59748  2.97032  5.70657  3.31889    654 a - - -
          2.68618  4.42225  2.77519  2.73123  3.46354  2.40513  3.72494
3.29354  2.67741  2.69355  4.24690  2.90347  2.73739  3.18146  2.89801
2.37887  2.77519  2.98518  4.58477  3.61503
          0.04049  5.08406  3.39650  0.61958  0.77255  0.51983  0.90293
    350   2.72053  4.96311  3.43194  2.52322  3.02645  3.45005  3.54457
3.55501  2.69452  2.21172  4.04609  3.36870  3.18222  2.58379  2.67308
2.74833  3.15154  2.41097  5.49535  2.79258    655 l - - -
```

```
          2.68618  4.42225  2.77519  2.73123  3.46354  2.40513  3.72494
3.29354  2.67741  2.69355  4.24690  2.90347  2.73739  3.18146  2.89801
2.37887  2.77519  2.98518  4.58477  3.61503
          0.11933  5.07927  2.24185  0.61958  0.77255  0.65288  0.73511
    351   2.67640  5.32232  2.71320  2.43237  3.84124  2.65582  3.83549
4.11716  2.17031  3.61844  4.36866  2.91138  3.22730  2.93912  2.67503
2.46664  2.68859  3.24583  5.76802  2.54925    656 k - - -
          2.68618  4.42225  2.77519  2.73123  3.46354  2.40513  3.72494
3.29354  2.67741  2.69355  4.24690  2.90347  2.73739  3.18146  2.89801
2.37887  2.77519  2.98518  4.58477  3.61503
          0.01037  4.97031  5.69265  0.61958  0.77255  0.44775  1.01906
    352   2.47996  5.02652  3.38719  2.35699  2.96551  2.72491  3.98751
3.00806  2.63764  3.00505  4.10493  2.81043  3.65249  2.71499  3.26797
2.43221  2.92899  2.45887  5.54774  3.28368    657 e - - -
          2.68618  4.42225  2.77519  2.73123  3.46354  2.40513  3.72494
3.29354  2.67741  2.69355  4.24690  2.90347  2.73739  3.18146  2.89801
2.37887  2.77519  2.98518  4.58477  3.61503
          0.29180  5.08787  1.39874  0.61958  0.77255  0.63034  0.76016
    353   2.80227  5.18261  2.88007  2.54937  3.33959  2.11014  3.79092
3.92919  2.54933  3.46626  4.23719  2.84290  3.50032  2.46363  2.78489
2.30031  2.61118  3.54597  5.64694  3.34159    658 g - - -
          2.68636  4.42243  2.77515  2.73126  3.46372  2.40515  3.72472
3.29337  2.67759  2.69360  4.24708  2.90349  2.73739  3.18060  2.89819
2.37884  2.77512  2.98509  4.58495  3.61521
          0.51065  1.25353  2.16807  0.65561  0.73215  0.44192  1.02946
    354   2.40005  5.24825  2.88119  2.42118  4.54680  2.30475  3.37598
3.55782  2.59884  3.18748  4.30147  1.98110  3.52370  2.75662  3.08240
2.84343  3.08789  3.24408  3.72483  4.32881    662 n - - -
          2.68618  4.42225  2.77519  2.73123  3.46354  2.40513  3.72494
3.29354  2.67741  2.69355  4.24690  2.90347  2.73739  3.18146  2.89801
2.37887  2.77519  2.98518  4.58477  3.61503
          0.03954  4.94954  3.45197  0.61958  0.77255  0.41291  1.08389
    355   2.52869  4.14397  2.60915  2.47380  4.72930  3.68174  3.86978
4.20888  2.24052  3.28309  4.43643  2.59412  2.52373  2.62761  2.90445
1.99990  3.13576  3.09194  5.83154  4.42617    663 s - - -
          2.68618  4.42225  2.77519  2.73123  3.46354  2.40513  3.72494
3.29354  2.67741  2.69355  4.24690  2.90347  2.73739  3.18146  2.89801
2.37887  2.77519  2.98518  4.58477  3.61503
          0.00940  5.06791  5.79025  0.61958  0.77255  0.56558  0.83940
    356   2.76864  5.09647  3.10183  2.57680  2.63853  2.84443  3.96584
3.32251  2.77391  2.80501  4.16936  2.82572  3.17688  3.12204  2.62886
2.10541  2.75649  3.08905  5.60446  3.34188    664 s - - -
          2.68618  4.42225  2.77519  2.73123  3.46354  2.40513  3.72494
3.29354  2.67741  2.69355  4.24690  2.90347  2.73739  3.18146  2.89801
2.37887  2.77519  2.98518  4.58477  3.61503
          0.44052  5.09269  1.04938  0.61958  0.77255  0.61738  0.77513
    357   2.39485  4.95703  2.90863  2.59085  3.20989  3.58157  3.78515
3.61412  2.41657  3.21599  4.02733  3.11114  3.05486  2.51978  2.76988
2.33689  2.98261  2.74741  5.45869  2.86816    665 s - - -
          2.68618  4.42225  2.77519  2.73123  3.46354  2.40513  3.72494
3.29354  2.67741  2.69355  4.24690  2.90347  2.73739  3.18146  2.89801
2.37887  2.77519  2.98518  4.58477  3.61503
          0.01407  4.66625  5.38859  0.61958  0.77255  1.06437  0.42303
```

```
    358   2.78271  4.32234  4.04343  3.46434  2.81945  3.29772  4.13623
2.28124  3.37427  2.22513  2.82088  3.25047  2.78227  3.22217  3.14973
3.12178  2.51285  2.24495  4.92661  3.27685    666 l - - -
          2.68618  4.42225  2.77519  2.73123  3.46354  2.40513  3.72494
3.29354  2.67741  2.69355  4.24690  2.90347  2.73739  3.18146  2.89801
2.37887  2.77519  2.98518  4.58477  3.61503
          0.01310  4.73730  5.45964  0.61958  0.77255  0.94345  0.49312
    359   2.45725  5.11223  2.50396  2.45002  2.87385  3.61653  3.01427
3.81724  2.31768  2.82346  4.17373  2.42630  4.00779  2.94278  3.06385
2.81391  2.81086  3.14269  5.59315  3.23006    667 k - - -
          2.68618  4.42225  2.77519  2.73123  3.46354  2.40513  3.72494
3.29354  2.67741  2.69355  4.24690  2.90347  2.73739  3.18146  2.89801
2.37887  2.77519  2.98518  4.58477  3.61503
          0.01205  4.82088  5.54323  0.61958  0.77255  1.11541  0.39717
    360   2.37652  4.45517  3.84179  3.27058  2.44908  3.00633  4.09999
2.93557  3.20961  2.47601  3.56321  3.63264  3.59068  3.10784  3.52349
2.58116  2.57550  2.52841  5.04808  2.19352    668 y - - -
          2.68618  4.42225  2.77519  2.73123  3.46354  2.40513  3.72494
3.29354  2.67741  2.69355  4.24690  2.90347  2.73739  3.18146  2.89801
2.37887  2.77519  2.98518  4.58477  3.61503
          0.01205  4.82088  5.54323  0.61958  0.77255  0.22941  1.58475
    361   2.75403  3.65904  4.93699  4.32274  2.46671  4.15264  4.48375
2.43951  4.11006  1.69181  3.47497  4.31126  4.51582  4.24256  4.12176
3.46363  2.92584  2.41133  1.80251  2.73273    669 l - - -
          2.68618  4.42225  2.77519  2.73123  3.46354  2.40513  3.72494
3.29354  2.67741  2.69355  4.24690  2.90347  2.73739  3.18146  2.89801
2.37887  2.77519  2.98518  4.58477  3.61503
          0.08337  5.13716  2.60210  0.61958  0.77255  0.48576  0.95510
    362   2.54571  5.27599  2.87942  2.65701  4.56739  3.70207  3.89595
3.18217  2.50399  3.55814  3.29136  2.57948  4.09416  2.35954  3.13778
1.63414  2.70578  3.27949  5.74241  4.36648    670 s - - -
          2.68627  4.42255  2.77523  2.73110  3.46384  2.40542  3.72458
3.29346  2.67747  2.69385  4.24622  2.90300  2.73769  3.18142  2.89830
2.37882  2.77475  2.98484  4.58507  3.61481
          0.08082  2.59609  5.78557  1.97101  0.15003  0.69318  0.69312
    363   2.46482  5.02893  3.80320  3.49782  5.13819  0.67614  4.71215
4.59693  3.58971  4.20218  4.99370  3.18863  4.38416  3.91136  3.24652
2.27610  3.43920  4.01850  6.39027  5.12472    682 G - - -
          2.68621  4.42228  2.77523  2.73126  3.46357  2.40516  3.72498
3.29357  2.67744  2.69358  4.24693  2.90350  2.73743  3.18149  2.89804
2.37878  2.77505  2.98521  4.58480  3.61465
          0.06890  3.45676  3.35100  0.84755  0.55942  0.69318  0.69312
    364   2.89457  5.18721  2.89059  2.51980  3.30654  3.70552  1.82539
3.12219  2.68102  3.14195  4.24995  1.99696  4.09706  3.03536  2.92011
2.90443  2.89104  3.53423  5.67039  4.31339    686 h - - -
          2.68618  4.42225  2.77519  2.73123  3.46354  2.40513  3.72494
3.29354  2.67741  2.69355  4.24690  2.90347  2.73739  3.18146  2.89801
2.37887  2.77519  2.98518  4.58477  3.61503
          0.00975  5.03072  5.75307  0.61958  0.77255  0.76834  0.62321
    365   2.70370  5.25501  2.95060  2.51595  4.54388  3.68938  3.88344
3.36272  2.51445  3.18950  3.22907  2.73599  4.08129  2.23098  2.17613
2.88389  2.35594  3.20167  5.72286  3.10359    687 r - - -
          2.68618  4.42225  2.77519  2.73123  3.46354  2.40513  3.72494
3.29354  2.67741  2.69355  4.24690  2.90347  2.73739  3.18146  2.89801
2.37887  2.77519  2.98518  4.58477  3.61503
```

```
          0.00975  5.03072  5.75307  0.61958  0.77255  0.76834  0.62321
    366   2.69883  4.51107  4.02750  3.10828  3.62953  3.94979  3.59129
2.11352  3.38145  2.06927  3.22027  1.64496  4.32472  3.65378  3.66985
3.21251  3.14568  2.47192  5.10863  3.39745    688 n - - -
          2.68618  4.42225  2.77519  2.73123  3.46354  2.40513  3.72494
3.29354  2.67741  2.69355  4.24690  2.90347  2.73739  3.18146  2.89801
2.37887  2.77519  2.98518  4.58477  3.61503
          0.00975  5.03072  5.75307  0.61958  0.77255  0.54767  0.86345
    367   2.72216  4.85659  3.53573  2.63760  3.20021  3.42805  4.05394
2.82148  2.62230  3.08554  3.18917  3.12580  4.21234  3.28263  2.48294
2.36175  2.28749  2.61854  5.40748  2.76003    689 t - - -
          2.68618  4.42225  2.77519  2.73123  3.46354  2.40513  3.72494
3.29354  2.67741  2.69355  4.24690  2.90347  2.73739  3.18146  2.89801
2.37887  2.77519  2.98518  4.58477  3.61503
          0.00927  5.08165  5.80399  0.61958  0.77255  0.64672  0.74183
    368   2.74011  5.21242  2.99825  2.41518  2.30876  2.72191  3.40068
3.91367  2.41624  3.14954  3.68162  2.95265  4.11667  3.05351  2.95577
2.56819  2.94751  3.56033  5.69494  2.44955    690 f - - -
          2.68618  4.42225  2.77519  2.73123  3.46354  2.40513  3.72494
3.29354  2.67741  2.69355  4.24690  2.90347  2.73739  3.18146  2.89801
2.37887  2.77519  2.98518  4.58477  3.61503
          0.00927  5.08165  5.80399  0.61958  0.77255  0.64672  0.74183
    369   2.74960  5.29613  2.77675  2.65928  4.59179  3.70783  3.48486
2.85879  2.27969  3.58004  4.35016  3.19769  4.09995  2.80661  2.11657
2.05163  2.55375  2.78945  5.75954  3.73663    691 s - - -
          2.68618  4.42225  2.77519  2.73123  3.46354  2.40513  3.72494
3.29354  2.67741  2.69355  4.24690  2.90347  2.73739  3.18146  2.89801
2.37887  2.77519  2.98518  4.58477  3.61503
          0.06437  5.08165  2.88012  0.61958  0.77255  0.52652  0.89320
    370   2.89901  5.36452  3.16573  2.19964  3.53675  3.25125  3.86895
4.16415  2.24527  3.29381  4.41009  2.30384  3.30542  2.56668  2.92190
2.51619  2.56538  3.74517  5.80842  2.57278    692 e - - -
          2.68656  4.42263  2.77557  2.73136  3.46392  2.40535  3.72436
3.29392  2.67778  2.69322  4.24727  2.90291  2.73777  3.18066  2.89726
2.37924  2.77432  2.98509  4.58378  3.61541
          0.20252  1.71354  5.77618  1.49642  0.25351  0.59544  0.80145
    371   2.69830  4.39218  4.52020  3.92782  3.07216  3.49316  4.37504
2.34267  3.78942  2.31589  3.14409  4.08918  3.70808  3.99056  3.93230
2.65724  1.37231  2.35214  5.00667  3.35046    698 t - - -
          2.68618  4.42225  2.77519  2.73123  3.46354  2.40513  3.72494
3.29354  2.67741  2.69355  4.24690  2.90347  2.73739  3.18146  2.89801
2.37887  2.77519  2.98518  4.58477  3.61503
          0.00928  5.07992  5.80227  0.61958  0.77255  0.52342  0.89769
    372   2.77397  5.34292  2.92553  2.52638  3.89838  2.32878  3.90288
3.66651  2.50768  2.59012  4.39327  1.96250  3.54176  2.60645  3.13942
2.59742  2.92220  3.71398  5.79788  3.70155    699 n - - -
          2.68618  4.42225  2.77519  2.73123  3.46354  2.40513  3.72494
3.29354  2.67741  2.69355  4.24690  2.90347  2.73739  3.18146  2.89801
2.37887  2.77519  2.98518  4.58477  3.61503
          0.08976  5.10701  2.52828  0.61958  0.77255  0.57748  0.82396
    373   2.55229  5.24910  2.63096  2.44076  3.34447  2.49761  3.88295
3.45231  2.64542  3.14357  4.30542  2.76385  3.24733  2.82010  3.12617
2.10381  2.71506  3.24902  5.71798  3.70435    700 s - - -
```

```
        2.68618  4.42225  2.77519  2.73123  3.46354  2.40513  3.72494
3.29354  2.67741  2.69355  4.24690  2.90347  2.73739  3.18146  2.89801
2.37887  2.77519  2.98518  4.58477  3.61503
        0.06786  5.02704  2.82939  0.61958  0.77255  0.77638  0.61632
    374  2.88014  5.26579  2.92947  2.63113  4.60787  2.80775  3.88718
3.43140  2.64890  3.59495  4.36132  1.83973  3.51527  3.00192  3.13365
1.77858  2.43873  3.66634  5.76704  4.38407    701 s - - -
        2.68618  4.42225  2.77519  2.73123  3.46354  2.40513  3.72494
3.29354  2.67741  2.69355  4.24690  2.90347  2.73739  3.18146  2.89801
2.37887  2.77519  2.98518  4.58477  3.61503
        0.03669  4.96955  3.53784  0.61958  0.77255  0.89062  0.52831
    375  2.85396  5.27624  2.51478  2.58267  4.58604  3.29643  3.39263
2.70258  2.45241  3.56636  4.32636  2.27086  3.16584  2.56114  3.06946
2.83406  2.19934  3.31805  5.73061  3.60391    702 t - - -
        2.68618  4.42225  2.77519  2.73123  3.46354  2.40513  3.72494
3.29354  2.67741  2.69355  4.24690  2.90347  2.73739  3.18146  2.89801
2.37887  2.77519  2.98518  4.58477  3.61503
        0.22885  4.94351  1.62239  0.61958  0.77255  0.82825  0.57414
    376  2.60663  4.78469  3.06169  2.77579  3.12349  3.66967  3.37658
3.36102  2.75871  2.28237  3.87089  2.71283  3.14067  2.80408  3.19147
2.66005  2.36373  2.62263  5.32472  3.46179    703 l - - -
        2.68618  4.42225  2.77519  2.73123  3.46354  2.40513  3.72494
3.29354  2.67741  2.69355  4.24690  2.90347  2.73739  3.18146  2.89801
2.37887  2.77519  2.98518  4.58477  3.61503
        0.05089  4.76009  3.19294  0.61958  0.77255  0.64959  0.73869
    377  2.85948  4.33571  4.32413  3.30636  2.84154  3.32786  2.81742
1.93759  3.61683  2.22481  3.45058  3.94385  4.33163  3.83641  3.16472
2.95810  2.49766  2.29890  3.02331  3.33836    704 i - - -
        2.68635  4.42242  2.77537  2.73141  3.46305  2.40530  3.72512
3.29249  2.67758  2.69331  4.24707  2.90335  2.73757  3.18113  2.89818
2.37892  2.77518  2.98513  4.58494  3.61484
        0.99000  0.74960  1.85874  0.06746  2.72973  0.57454  0.82774
    378  2.66007  5.23289  2.87438  2.28664  3.66627  3.61989  3.81191
3.53299  2.42819  3.16143  3.64783  2.86543  3.34614  2.43566  2.78155
2.35731  2.26270  3.60080  5.69151  3.34420    706 t - - -
        2.68618  4.42225  2.77519  2.73123  3.46354  2.40513  3.72494
3.29354  2.67741  2.69355  4.24690  2.90347  2.73739  3.18146  2.89801
2.37887  2.77519  2.98518  4.58477  3.61503
        0.01135  4.88024  5.60259  0.61958  0.77255  0.47121  0.97881
    379  2.89891  5.39279  2.91470  2.13098  3.95814  2.10598  3.35919
4.21174  2.32888  3.69501  4.43543  2.91080  4.06808  2.76192  3.09243
1.87637  2.77704  3.42634  5.82974  4.42294    707 s - - -
        2.68618  4.42225  2.77519  2.73123  3.46354  2.40513  3.72494
3.29354  2.67741  2.69355  4.24690  2.90347  2.73739  3.18146  2.89801
2.37887  2.77519  2.98518  4.58477  3.61503
        0.00956  5.05122  5.77357  0.61958  0.77255  0.57681  0.82482
    380  2.76336  5.34184  2.75349  2.49382  4.65440  2.99461  3.88998
3.02195  2.63876  3.21749  4.39119  2.54383  1.99125  2.63626  2.92130
2.58243  2.87336  3.33312  5.79449  3.82544    708 p - - -
        2.68618  4.42225  2.77519  2.73123  3.46354  2.40513  3.72494
3.29354  2.67741  2.69355  4.24690  2.90347  2.73739  3.18146  2.89801
2.37887  2.77519  2.98518  4.58477  3.61503
        0.00925  5.08307  5.80542  0.61958  0.77255  0.64300  0.74594
```

```
    381   2.94805  4.35248  4.73292  4.12919  2.69902  4.09670  3.58422
1.91500  3.95191  1.92388  3.47027  3.30908  4.46224  4.11640  4.02307
2.49519  2.24316  1.96134  3.89830  3.79051    709 i - - -
          2.68618  4.42225  2.77519  2.73123  3.46354  2.40513  3.72494
3.29354  2.67741  2.69355  4.24690  2.90347  2.73739  3.18146  2.89801
2.37887  2.77519  2.98518  4.58477  3.61503
          0.06185  5.08307  2.92289  0.61958  0.77255  0.64300  0.74594
    382   2.91329  4.59198  3.86051  2.98426  2.44216  3.91011  3.57986
2.63275  3.23770  2.79978  3.69740  3.31360  4.28808  2.45565  3.57211
3.16057  2.88255  2.61894  5.17200  1.65374    710 y - - -
          2.68618  4.42225  2.77519  2.73123  3.46354  2.40513  3.72494
3.29354  2.67741  2.69355  4.24690  2.90347  2.73739  3.18146  2.89801
2.37887  2.77519  2.98518  4.58477  3.61503
          0.00975  5.03097  5.75332  0.61958  0.77255  0.76780  0.62368
    383   3.04045  5.07485  3.55957  3.24882  4.79963  0.71511  4.47225
4.21601  2.83120  3.84893  4.68179  3.16981  4.35571  3.67603  3.69928
2.80939  3.41860  2.86073  6.08587  4.80418    711 g - - -
          2.68618  4.42225  2.77519  2.73123  3.46354  2.40513  3.72494
3.29354  2.67741  2.69355  4.24690  2.90347  2.73739  3.18146  2.89801
2.37887  2.77519  2.98518  4.58477  3.61503
          0.03439  5.03097  3.60198  0.61958  0.77255  0.43439  1.04316
    384   2.91408  5.37829  2.80359  2.48193  4.70430  3.69523  3.88436
3.21290  2.06798  3.67542  3.76026  2.43005  3.57805  2.49131  2.58618
2.50342  2.72302  3.37895  4.37476  2.83066    712 k - - -
          2.68618  4.42225  2.77519  2.73123  3.46354  2.40513  3.72494
3.29354  2.67741  2.69355  4.24690  2.90347  2.73739  3.18146  2.89801
2.37887  2.77519  2.98518  4.58477  3.61503
          0.00920  5.08887  5.81122  0.61958  0.77255  0.62766  0.76323
    385   2.55460  4.97643  3.08878  2.86889  3.66253  3.79208  3.13854
2.82273  2.53746  2.78236  3.16756  3.36708  4.17942  2.96960  2.34498
2.41275  2.29947  3.01396  5.50707  3.24466    713 t - - -
          2.68618  4.42225  2.77519  2.73123  3.46354  2.40513  3.72494
3.29354  2.67741  2.69355  4.24690  2.90347  2.73739  3.18146  2.89801
2.37887  2.77519  2.98518  4.58477  3.61503
          0.03705  5.08887  3.49975  0.61958  0.77255  0.51977  0.90302
    386   2.72385  5.30004  2.81437  2.65872  4.59671  2.52555  3.45429
4.04992  2.65816  3.24072  3.72724  2.62415  4.10034  2.81093  2.90954
2.10087  2.19828  3.00295  5.76285  3.29257    714 s - - -
          2.68618  4.42225  2.77519  2.73123  3.46354  2.40513  3.72494
3.29354  2.67741  2.69355  4.24690  2.90347  2.73739  3.18146  2.89801
2.37887  2.77519  2.98518  4.58477  3.61503
          0.06051  5.08439  2.94655  0.61958  0.77255  0.63954  0.74979
    387   2.70928  5.32196  2.66918  2.23848  3.84885  3.06382  3.86854
3.21865  2.61716  3.61359  4.37129  2.57582  4.07040  2.97626  2.87585
1.70080  2.56373  3.33995  5.77441  4.38360    715 s - - -
          2.68618  4.42225  2.77519  2.73123  3.46354  2.40513  3.72494
3.29354  2.67741  2.69355  4.24690  2.90347  2.73739  3.18146  2.89801
2.37887  2.77519  2.98518  4.58477  3.61503
          0.29709  5.03361  1.38425  0.61958  0.77255  0.43932  1.03416
    388   2.83623  5.30366  2.64834  2.53163  4.63498  2.27406  3.80498
4.10962  2.55391  3.22046  4.35611  2.02070  2.86876  2.69074  3.04561
2.43809  2.64325  3.68863  5.75271  4.35062    716 n - - -
          2.68618  4.42225  2.77519  2.73123  3.46354  2.40513  3.72494
3.29354  2.67741  2.69355  4.24690  2.90347  2.73739  3.18146  2.89801
2.37887  2.77519  2.98518  4.58477  3.61503
```

0.04235 4.85088 3.39196 0.61958 0.77255 0.64008 0.74919
389 2.46484 5.16053 2.94173 2.36640 3.78630 3.28066 3.86785
3.40865 2.64320 3.43221 3.68704 2.56691 3.51418 2.80194 2.87626
2.38644 2.50588 2.71738 5.64244 2.93472 717 e - - -
2.68618 4.42225 2.77519 2.73123 3.46354 2.40513 3.72494
3.29354 2.67741 2.69355 4.24690 2.90347 2.73739 3.18146 2.89801
2.37887 2.77519 2.98518 4.58477 3.61503
0.09927 4.95407 2.43675 0.61958 0.77255 0.61639 0.77628
390 2.40502 5.35047 2.47826 2.17621 4.68892 3.29666 3.81955
2.77251 2.18050 3.30021 4.39289 2.70474 3.51796 2.71173 2.64100
2.82097 2.50653 3.73755 5.78712 4.38020 718 e - - -
2.68620 4.42227 2.77485 2.73125 3.46356 2.40515 3.72497
3.29356 2.67743 2.69357 4.24692 2.90349 2.73742 3.18148 2.89803
2.37889 2.77522 2.98520 4.58479 3.61505
0.17522 2.72040 2.35515 0.29634 1.36075 0.52003 0.90264
391 2.86647 5.29133 3.15056 2.59425 4.60245 3.28992 3.84467
4.06488 2.01315 3.26496 4.34121 2.94476 2.99917 2.57248 2.84865
2.15262 2.11719 3.28913 5.74483 3.31980 720 k - - -
2.68618 4.42225 2.77519 2.73123 3.46354 2.40513 3.72494
3.29354 2.67741 2.69355 4.24690 2.90347 2.73739 3.18146 2.89801
2.37887 2.77519 2.98518 4.58477 3.61503
0.16525 4.97309 1.92829 0.61958 0.77255 0.76296 0.62790
392 2.81834 5.20721 2.66738 2.56213 4.50480 3.23792 3.02928
2.91222 2.16939 2.70704 4.26068 2.64211 4.00450 2.54820 3.04489
2.34215 2.83952 3.20323 5.66940 3.64121 721 k - - -
2.68664 4.42287 2.77563 2.73113 3.46341 2.40535 3.72392
3.29285 2.67767 2.69299 4.24671 2.90387 2.73730 3.18174 2.89792
2.37897 2.77394 2.98580 4.58421 3.61565
0.69792 0.84389 2.62630 1.36577 0.29462 1.07355 0.41823
393 2.79771 5.30222 2.79776 2.21333 4.64429 3.24134 3.75812
4.12842 2.49522 3.60611 4.34423 2.60889 3.45022 2.24059 2.16320
2.43206 2.33232 3.69136 5.73642 4.32668 736 r - - -
2.68618 4.42225 2.77519 2.73123 3.46354 2.40513 3.72494
3.29354 2.67741 2.69355 4.24690 2.90347 2.73739 3.18146 2.89801
2.37887 2.77519 2.98518 4.58477 3.61503
0.01253 4.78198 5.50433 0.61958 0.77255 0.71220 0.67445
394 2.51914 5.22975 2.92864 2.59351 4.52521 3.64196 3.11348
2.93161 2.21946 3.19590 4.28381 2.69151 3.57586 2.95027 2.51192
2.38715 2.22926 3.59345 4.01026 4.31450 737 k - - -
2.68618 4.42225 2.77519 2.73123 3.46354 2.40513 3.72494
3.29354 2.67741 2.69355 4.24690 2.90347 2.73739 3.18146 2.89801
2.37887 2.77519 2.98518 4.58477 3.61503
0.11060 4.92540 2.32848 0.61958 0.77255 0.49985 0.93298
395 2.68913 4.35385 3.64013 3.80688 2.17478 3.10253 4.30425
1.92038 3.68005 2.26272 3.46919 3.12135 3.20896 3.89265 3.84588
3.28671 2.86624 2.44104 3.73693 2.80951 738 i - - -
2.68629 4.42236 2.77512 2.73099 3.46365 2.40524 3.72462
3.29365 2.67752 2.69349 4.24604 2.90358 2.73751 3.18102 2.89812
2.37898 2.77531 2.98488 4.58488 3.61514
0.25409 1.70979 3.13557 0.52379 0.89716 0.64936 0.73894
396 2.55999 4.64563 2.77237 3.14445 3.01585 3.29824 4.10142
2.39351 3.10460 2.33775 3.74708 2.54516 4.23369 3.41261 3.47293
2.26207 2.86735 2.23742 5.22292 3.98172 741 v - - -

```
         2.68618  4.42225  2.77519  2.73123  3.46354  2.40513  3.72494
3.29354  2.67741  2.69355  4.24690  2.90347  2.73739  3.18146  2.89801
2.37887  2.77519  2.98518  4.58477  3.61503
         0.03907  4.99089  3.45728  0.61958  0.77255  0.60992  0.78393
    397  2.70975  4.71753  3.64167  2.78999  2.53549  3.84280  4.08259
2.93336  2.76728  2.19082  3.81531  3.17736  2.77321  2.64142  3.09294
3.07595  2.87753  2.09175  5.28599  4.03333    742 v - - -
         2.68618  4.42225  2.77519  2.73123  3.46354  2.40513  3.72494
3.29354  2.67741  2.69355  4.24690  2.90347  2.73739  3.18146  2.89801
2.37887  2.77519  2.98518  4.58477  3.61503
         0.00984  5.02206  5.74441  0.61958  0.77255  0.62289  0.76871
    398  2.71412  5.31914  2.57340  2.38497  4.62697  3.31957  3.49617
3.25978  2.45678  2.82616  4.36977  2.94194  2.97160  2.78952  3.11946
2.27353  2.41102  3.36177  3.24903  4.38747    743 s - - -
         2.68618  4.42225  2.77519  2.73123  3.46354  2.40513  3.72494
3.29354  2.67741  2.69355  4.24690  2.90347  2.73739  3.18146  2.89801
2.37887  2.77519  2.98518  4.58477  3.61503
         0.00948  5.05944  5.78178  0.61958  0.77255  0.70237  0.68401
    399  2.42600  5.30542  2.62716  2.64007  4.60810  2.42714  3.88620
3.55569  2.49192  3.18607  3.71641  1.95677  2.87979  2.99824  3.12457
2.66390  2.89287  3.67309  5.76443  3.80155    744 n - - -
         2.68618  4.42225  2.77519  2.73123  3.46354  2.40513  3.72494
3.29354  2.67741  2.69355  4.24690  2.90347  2.73739  3.18146  2.89801
2.37887  2.77519  2.98518  4.58477  3.61503
         0.00948  5.05944  5.78178  0.61958  0.77255  0.70237  0.68401
    400  2.30877  5.29556  3.20162  2.64513  4.59458  3.69626  3.88875
3.21555  2.34346  3.58094  4.34867  2.79106  3.55280  2.51389  2.22393
2.15927  2.50945  3.02364  5.75681  3.73083    745 s - - -
         2.68618  4.42225  2.77519  2.73123  3.46354  2.40513  3.72494
3.29354  2.67741  2.69355  4.24690  2.90347  2.73739  3.18146  2.89801
2.37887  2.77519  2.98518  4.58477  3.61503
         0.04212  5.05944  3.35527  0.61958  0.77255  0.59530  0.80162
    401  2.99057  5.50807  1.72673  2.26243  4.84372  2.80871  3.93522
4.33249  2.38662  3.80944  4.55558  2.03455  4.12650  3.03957  3.20390
2.54802  3.22694  3.89450  5.94201  2.77988    746 d - - -
         2.68618  4.42225  2.77519  2.73123  3.46354  2.40513  3.72494
3.29354  2.67741  2.69355  4.24690  2.90347  2.73739  3.18146  2.89801
2.37887  2.77519  2.98518  4.58477  3.61503
         0.00956  5.05082  5.77316  0.61958  0.77255  0.57895  0.82209
    402  2.45568  4.43561  3.59028  3.89628  3.16581  4.09053  4.40305
1.28035  3.77221  2.62289  3.55425  4.08884  4.45883  3.44239  3.94085
3.38096  2.53145  1.82767  5.06637  3.87538    747 i - - -
         2.68618  4.42225  2.77519  2.73123  3.46354  2.40513  3.72494
3.29354  2.67741  2.69355  4.24690  2.90347  2.73739  3.18146  2.89801
2.37887  2.77519  2.98518  4.58477  3.61503
         0.00926  5.08240  5.80475  0.61958  0.77255  0.64475  0.74400
    403  3.01963  4.95012  2.91259  3.03152  2.18960  2.85504  3.50679
3.51403  3.04613  3.17114  4.04653  3.14062  4.28681  3.36409  3.46604
2.78238  3.25316  3.25532  5.30232  1.43412    748 y - - -
         2.68618  4.42225  2.77519  2.73123  3.46354  2.40513  3.72494
3.29354  2.67741  2.69355  4.24690  2.90347  2.73739  3.18146  2.89801
2.37887  2.77519  2.98518  4.58477  3.61503
         0.00926  5.08240  5.80475  0.61958  0.77255  0.64475  0.74400
```

404 2.91082 5.41384 2.91964 2.36742 3.91841 3.31736 3.87300
4.23946 1.60642 3.36411 4.45534 2.93696 3.49151 2.62406 2.12413
2.54483 2.93710 3.39404 5.84835 4.43941 749 k - - -
2.68618 4.42225 2.77519 2.73123 3.46354 2.40513 3.72494
3.29354 2.67741 2.69355 4.24690 2.90347 2.73739 3.18146 2.89801
2.37887 2.77519 2.98518 4.58477 3.61503
0.03553 5.08240 3.55093 0.61958 0.77255 0.50329 0.92771
405 3.00128 4.37896 4.90585 4.29858 3.47958 4.17619 4.51318
1.83651 4.10097 1.81754 3.16086 4.31726 4.53660 4.24615 4.13459
3.00286 2.44990 1.39915 5.02268 3.34821 750 v - - -
2.68618 4.42225 2.77519 2.73123 3.46354 2.40513 3.72494
3.29354 2.67741 2.69355 4.24690 2.90347 2.73739 3.18146 2.89801
2.37887 2.77519 2.98518 4.58477 3.61503
0.09896 5.08724 2.42988 0.61958 0.77255 0.63201 0.75827
406 2.87876 5.15188 3.00171 2.16181 3.72997 3.30146 3.89413
2.85392 2.67600 2.92616 4.21628 2.31769 3.60048 3.02905 2.70467
2.55338 2.21621 3.49547 4.28454 3.68201 751 e - - -
2.68618 4.42225 2.77519 2.73123 3.46354 2.40513 3.72494
3.29354 2.67741 2.69355 4.24690 2.90347 2.73739 3.18146 2.89801
2.37887 2.77519 2.98518 4.58477 3.61503
0.01008 4.99835 5.72070 0.61958 0.77255 0.61515 0.77774
407 2.59033 4.77120 3.16679 2.61733 3.93160 3.38742 4.07535
3.04251 3.01236 2.59455 2.63593 3.49565 4.22353 3.33467 3.41300
1.67045 2.92471 2.20231 5.33406 4.07313 752 s - - -
2.68618 4.42225 2.77519 2.73123 3.46354 2.40513 3.72494
3.29354 2.67741 2.69355 4.24690 2.90347 2.73739 3.18146 2.89801
2.37887 2.77519 2.98518 4.58477 3.61503
0.03335 5.05091 3.63443 0.61958 0.77255 0.60204 0.79339
408 2.73738 4.82245 3.54616 2.55571 3.43720 3.37879 4.05129
2.51440 2.77603 2.24752 3.36869 2.71537 4.20685 2.95292 3.03952
2.64905 2.19777 2.86381 5.37685 3.53207 753 t - - -
2.68618 4.42225 2.77519 2.73123 3.46354 2.40513 3.72494
3.29354 2.67741 2.69355 4.24690 2.90347 2.73739 3.18146 2.89801
2.37887 2.77519 2.98518 4.58477 3.61503
0.03767 5.05419 3.48704 0.61958 0.77255 0.71490 0.67186
409 1.90610 5.16766 2.58618 2.69012 4.42839 3.31283 3.90533
2.84043 2.68632 3.43639 3.65664 2.72988 3.29648 2.82063 3.15895
2.43645 2.42351 2.95556 5.65504 4.30190 754 a - - -
2.68618 4.42225 2.77519 2.73123 3.46354 2.40513 3.72494
3.29354 2.67741 2.69355 4.24690 2.90347 2.73739 3.18146 2.89801
2.37887 2.77519 2.98518 4.58477 3.61503
0.04357 5.02631 3.32215 0.61958 0.77255 0.61378 0.77937
410 2.74202 4.64220 2.80315 3.19684 2.99377 2.87030 3.52112
2.68082 3.15360 2.22545 3.74463 3.26221 4.26137 3.45771 3.15659
2.69425 2.69712 2.34376 5.22223 2.49659 755 l - - -
2.68618 4.42225 2.77519 2.73123 3.46354 2.40513 3.72494
3.29354 2.67741 2.69355 4.24690 2.90347 2.73739 3.18146 2.89801
2.37887 2.77519 2.98518 4.58477 3.61503
0.04073 5.03133 3.39964 0.61958 0.77255 0.76702 0.62436
411 2.68656 4.15799 2.32484 2.66128 4.45892 3.32492 3.88503
3.15382 2.65843 3.46126 3.65024 2.75683 3.56507 2.43759 2.72049
2.62630 2.40416 2.97990 5.66843 3.02763 756 d - - -
2.68618 4.42225 2.77519 2.73123 3.46354 2.40513 3.72494
3.29354 2.67741 2.69355 4.24690 2.90347 2.73739 3.18146 2.89801
2.37887 2.77519 2.98518 4.58477 3.61503

```
         0.03884  5.00065  3.46226  0.61958  0.77255  0.68738  0.69895
    412  2.56695  5.15195  2.93492  2.49302  3.78370  3.00025  2.88741
2.77330  2.39888  2.93147  4.21665  2.33359  4.08973  3.03406  2.92929
2.72291  3.11349  2.72423  5.64049  3.28206    757 n - - -
         2.68634  4.42293  2.77535  2.73157  3.46303  2.40528  3.72516
3.29131  2.67759  2.69374  4.24758  2.90319  2.73768  3.18215  2.89869
2.37881  2.77533  2.98564  4.58427  3.61140
         0.66801  0.80892  3.17162  1.28203  0.32500  0.81991  0.58066
    413  2.38578  5.12483  2.96610  2.67476  3.79478  2.99845  3.88493
3.41238  2.67023  3.03084  4.19060  2.28529  2.99984  3.02264  2.58249
2.52372  2.50056  2.69665  5.61572  4.26685    768 n - - -
         2.68618  4.42225  2.77519  2.73123  3.46354  2.40513  3.72494
3.29354  2.67741  2.69355  4.24690  2.90347  2.73739  3.18146  2.89801
2.37887  2.77519  2.98518  4.58477  3.61503
         0.36017  4.96683  1.21916  0.61958  0.77255  0.66826  0.71866
    414  2.59129  5.03710  2.87105  2.56608  4.30128  2.52478  3.78244
3.26814  2.56534  3.31007  4.10662  2.62966  2.50724  2.91759  3.03772
2.60219  2.35898  3.38482  5.52970  3.25233    769 t - - -
         2.68618  4.42225  2.77519  2.73123  3.46354  2.40513  3.72494
3.29354  2.67741  2.69355  4.24690  2.90347  2.73739  3.18146  2.89801
2.37887  2.77519  2.98518  4.58477  3.61503
         0.04876  4.69956  3.25729  0.61958  0.77255  0.61932  0.77286
    415  2.37044  3.93241  3.04772  2.55853  4.17717  3.21407  3.90184
3.58144  2.72657  2.53966  4.03807  2.75623  2.92672  2.79121  3.17962
2.68589  2.83362  3.28028  4.08773  2.35253    770 y - - -
         2.68618  4.42225  2.77519  2.73123  3.46354  2.40513  3.72494
3.29354  2.67741  2.69355  4.24690  2.90347  2.73739  3.18146  2.89801
2.37887  2.77519  2.98518  4.58477  3.61503
         0.01110  4.90218  5.62453  0.61958  0.77255  1.00294  0.45697
    416  2.64987  5.22519  2.69181  2.42585  4.52235  2.62502  3.39430
3.97589  2.58130  2.93817  3.64564  2.66135  3.50968  2.93897  2.83091
2.47948  2.38358  2.93615  5.68766  3.40143    771 t - - -
         2.68618  4.42225  2.77519  2.73123  3.46354  2.40513  3.72494
3.29354  2.67741  2.69355  4.24690  2.90347  2.73739  3.18146  2.89801
2.37887  2.77519  2.98518  4.58477  3.61503
         0.01110  4.90218  5.62453  0.61958  0.77255  0.63576  0.75403
    417  2.87892  5.26472  2.57847  2.62417  3.10712  3.29153  3.86642
4.01429  2.36045  3.21168  4.31849  2.41770  3.19235  2.81665  2.36412
2.48324  2.90809  3.03115  4.11482  3.36216    772 k - - -
         2.68618  4.42225  2.77519  2.73123  3.46354  2.40513  3.72494
3.29354  2.67741  2.69355  4.24690  2.90347  2.73739  3.18146  2.89801
2.37887  2.77519  2.98518  4.58477  3.61503
         0.12229  5.00361  2.22194  0.61958  0.77255  0.60742  0.78692
    418  2.85548  5.32913  2.86758  2.56302  3.90235  3.29759  3.12984
3.26463  2.34105  2.98844  4.37383  2.49455  3.18171  2.27263  2.47734
2.49824  2.63221  3.71154  5.77106  4.36952    773 q - - -
         2.68618  4.42225  2.77519  2.73123  3.46354  2.40513  3.72494
3.29354  2.67741  2.69355  4.24690  2.90347  2.73739  3.18146  2.89801
2.37887  2.77519  2.98518  4.58477  3.61503
         0.01058  4.94999  5.67233  0.61958  0.77255  0.51478  0.91039
    419  2.75585  4.63337  3.36186  2.75796  2.36042  3.90602  4.16084
2.37631  3.20108  2.62760  3.73706  2.82639  2.54294  3.50078  3.55084
2.68322  3.15417  2.90584  3.32268  2.65445    774 f - - -
```

```
          2.68618  4.42225  2.77519  2.73123  3.46354  2.40513  3.72494
3.29354  2.67741  2.69355  4.24690  2.90347  2.73739  3.18146  2.89801
2.37887  2.77519  2.98518  4.58477  3.61503
          0.00947  5.06014  5.78249  0.61958  0.77255  0.56640  0.83832
    420   2.94612  4.44606  4.32805  3.74433  2.42892  3.16670  4.33143
2.52957  3.63697  2.04637  3.55995  3.08105  4.40539  3.86969  3.34692
2.42845  2.65143  2.54800  5.05461  2.00787    775 y - - -
          2.68618  4.42225  2.77519  2.73123  3.46354  2.40513  3.72494
3.29354  2.67741  2.69355  4.24690  2.90347  2.73739  3.18146  2.89801
2.37887  2.77519  2.98518  4.58477  3.61503
          0.00920  5.08924  5.81158  0.61958  0.77255  0.62668  0.76435
    421   2.70456  4.93962  3.46148  2.69952  3.42523  1.73659  4.02261
2.88727  2.69333  2.57869  4.02474  3.11080  4.19071  3.22266  2.66321
2.56021  2.70647  2.97848  5.47695  4.18366    776 g - - -
          2.68618  4.42225  2.77519  2.73123  3.46354  2.40513  3.72494
3.29354  2.67741  2.69355  4.24690  2.90347  2.73739  3.18146  2.89801
2.37887  2.77519  2.98518  4.58477  3.61503
          0.00920  5.08924  5.81158  0.61958  0.77255  0.51529  0.90964
    422   2.77234  4.40214  4.59581  3.49560  3.17957  4.09600  4.41098
1.48771  3.37248  2.14208  3.51911  4.14063  2.67786  4.04636  3.97892
3.06544  3.19919  1.76754  5.02075  3.83522    777 i - - -
          2.68618  4.42225  2.77519  2.73123  3.46354  2.40513  3.72494
3.29354  2.67741  2.69355  4.24690  2.90347  2.73739  3.18146  2.89801
2.37887  2.77519  2.98518  4.58477  3.61503
          0.00899  5.11248  5.83483  0.61958  0.77255  0.56165  0.84460
    423   2.70005  5.41503  3.18050  2.50237  4.75191  3.34296  3.43442
3.67256  2.33732  3.71680  4.45785  2.06658  2.89126  2.45417  2.64300
2.23446  2.96128  3.35949  5.85263  3.34813    778 n - - -
          2.68618  4.42225  2.77519  2.73123  3.46354  2.40513  3.72494
3.29354  2.67741  2.69355  4.24690  2.90347  2.73739  3.18146  2.89801
2.37887  2.77519  2.98518  4.58477  3.61503
          0.00899  5.11248  5.83483  0.61958  0.77255  0.56165  0.84460
    424   2.92420  5.43300  2.76285  2.34912  4.77738  3.40138  3.88514
3.52415  1.76106  3.73791  4.47388  2.60213  4.09155  2.16166  2.54532
2.67562  2.46285  3.45441  5.86601  4.45561    779 k - - -
          2.68618  4.42225  2.77519  2.73123  3.46354  2.40513  3.72494
3.29354  2.67741  2.69355  4.24690  2.90347  2.73739  3.18146  2.89801
2.37887  2.77519  2.98518  4.58477  3.61503
          0.00899  5.11248  5.83483  0.61958  0.77255  0.56165  0.84460
    425   2.05806  4.43478  4.42605  3.83871  2.81528  3.50321  4.36525
2.07255  3.71838  2.30317  2.58026  3.40571  3.69545  2.88570  3.89748
2.64918  3.19119  2.08068  5.04618  3.85446    780 a - - -
          2.68618  4.42225  2.77519  2.73123  3.46354  2.40513  3.72494
3.29354  2.67741  2.69355  4.24690  2.90347  2.73739  3.18146  2.89801
2.37887  2.77519  2.98518  4.58477  3.61503
          0.06978  5.11248  2.79068  0.61958  0.77255  0.56165  0.84460
    426   2.89789  5.38001  2.48050  1.79412  4.71323  3.30453  3.45809
3.25836  2.31609  3.68023  4.42380  2.83429  4.06933  2.63657  3.09465
2.40968  2.73244  3.07191  4.44243  3.41034    781 e - - -
          2.68618  4.42225  2.77519  2.73123  3.46354  2.40513  3.72494
3.29354  2.67741  2.69355  4.24690  2.90347  2.73739  3.18146  2.89801
2.37887  2.77519  2.98518  4.58477  3.61503
          0.00955  5.05225  5.77459  0.61958  0.77255  0.59891  0.79719
```

```
    427   3.06516  4.49401  4.65736  4.07960  1.05288  3.51305  4.37410
2.92886  3.92899  1.97208  3.17418  3.12453  4.54006  4.11141  4.05019
3.47523  3.29704  2.74675  4.89531  3.16252    782 f - - -
          2.68618  4.42225  2.77519  2.73123  3.46354  2.40513  3.72494
3.29354  2.67741  2.69355  4.24690  2.90347  2.73739  3.18146  2.89801
2.37887  2.77519  2.98518  4.58477  3.61503
          0.07074  5.07843  2.77953  0.61958  0.77255  0.65505  0.73276
    428   2.69222  4.85305  3.47994  2.91880  2.40806  3.39667  3.01502
3.08043  2.69002  2.80729  3.45154  2.83571  4.17464  2.95922  2.80449
2.51055  2.69432  3.15142  5.39963  2.54239    783 f - - -
          2.68618  4.42225  2.77519  2.73123  3.46354  2.40513  3.72494
3.29354  2.67741  2.69355  4.24690  2.90347  2.73739  3.18146  2.89801
2.37887  2.77519  2.98518  4.58477  3.61503
          0.00988  5.01758  5.73992  0.61958  0.77255  0.79657  0.59943
    429   2.69007  4.54061  3.93592  3.36429  2.75962  2.67738  3.54817
2.57338  3.02864  2.28710  3.01659  3.22208  4.29865  2.93703  2.71284
3.17865  2.50598  2.34060  3.96155  3.91457    784 l - - -
          2.68618  4.42225  2.77519  2.73123  3.46354  2.40513  3.72494
3.29354  2.67741  2.69355  4.24690  2.90347  2.73739  3.18146  2.89801
2.37887  2.77519  2.98518  4.58477  3.61503
          0.00988  5.01758  5.73992  0.61958  0.77255  0.79657  0.59943
    430   2.49483  4.44802  3.11938  3.59119  3.17635  3.97507  4.26248
2.90781  3.50107  2.18661  3.21232  3.37747  4.34760  3.75190  3.74545
2.53983  2.10016  1.73580  4.01032  3.43445    785 v - - -
          2.68618  4.42225  2.77519  2.73123  3.46354  2.40513  3.72494
3.29354  2.67741  2.69355  4.24690  2.90347  2.73739  3.18146  2.89801
2.37887  2.77519  2.98518  4.58477  3.61503
          0.09603  5.01758  2.46582  0.61958  0.77255  0.63240  0.75783
    431   2.66753  5.38691  2.21222  2.56426  4.72780  2.77096  3.84154
4.21207  2.01733  3.29451  4.43006  1.84484  3.51907  2.73510  3.07625
2.67780  3.11465  3.77559  5.82184  4.41110    786 n - - -
          2.68618  4.42225  2.77519  2.73123  3.46354  2.40513  3.72494
3.29354  2.67741  2.69355  4.24690  2.90347  2.73739  3.18146  2.89801
2.37887  2.77519  2.98518  4.58477  3.61503
          0.03997  4.97313  3.43393  0.61958  0.77255  0.88406  0.53290
    432   2.86427  5.34470  2.55629  2.56129  4.67740  1.85269  3.82889
4.15468  2.39548  3.64483  4.39015  2.36891  4.03234  2.93027  2.54297
2.62534  2.49111  3.72901  5.78527  3.70683    787 g - - -
          2.68618  4.42225  2.77519  2.73123  3.46354  2.40513  3.72494
3.29354  2.67741  2.69355  4.24690  2.90347  2.73739  3.18146  2.89801
2.37887  2.77519  2.98518  4.58477  3.61503
          0.01065  4.94380  5.66615  0.61958  0.77255  0.81031  0.58829
    433   2.86487  5.37037  2.69131  2.43436  4.71328  3.34281  3.14608
3.62259  2.24714  3.67473  4.41160  2.51276  3.52955  2.31263  2.67531
2.22286  2.56387  3.34978  5.80435  3.72922    788 s - - -
          2.68618  4.42225  2.77519  2.73123  3.46354  2.40513  3.72494
3.29354  2.67741  2.69355  4.24690  2.90347  2.73739  3.18146  2.89801
2.37887  2.77519  2.98518  4.58477  3.61503
          0.13841  4.97424  2.10094  0.61958  0.77255  0.76084  0.62975
    434   2.64083  5.29649  2.49285  2.40699  4.62410  3.23737  3.34668
3.12203  2.30480  3.59428  4.34181  2.12344  4.00164  2.89980  2.64012
2.79886  2.37588  3.03766  5.73973  4.33956    789 n - - -
          2.68618  4.42225  2.77519  2.73123  3.46354  2.40513  3.72494
3.29354  2.67741  2.69355  4.24690  2.90347  2.73739  3.18146  2.89801
2.37887  2.77519  2.98518  4.58477  3.61503
```

0.01135 4.87977 5.60211 0.61958 0.77255 0.78527 0.60880
435 2.50895 5.32553 2.90604 2.15890 3.48411 3.63464 3.13726
4.12872 2.56497 2.78498 4.37054 2.39052 2.93198 2.72418 2.82842
2.22337 2.85452 3.70731 5.76816 4.36732 790 e - - -
2.68618 4.42225 2.77519 2.73123 3.46354 2.40513 3.72494
3.29354 2.67741 2.69355 4.24690 2.90347 2.73739 3.18146 2.89801
2.37887 2.77519 2.98518 4.58477 3.61503
0.22532 4.94759 1.63661 0.61958 0.77255 0.82130 0.57957
436 2.45089 5.16718 2.84981 2.40825 3.71268 3.20289 3.77486
3.45962 2.38225 2.86455 4.22143 2.82633 3.97376 2.25551 2.78095
2.43234 2.61428 3.53048 5.63121 3.26947 791 q - - -
2.68618 4.42225 2.77519 2.73123 3.46354 2.40513 3.72494
3.29354 2.67741 2.69355 4.24690 2.90347 2.73739 3.18146 2.89801
2.37887 2.77519 2.98518 4.58477 3.61503
0.01271 4.76736 5.48971 0.61958 0.77255 1.17881 0.36766
437 2.60389 5.19019 2.83321 2.38585 4.49326 2.96358 3.76951
3.12401 2.34632 3.47737 4.24215 2.81980 3.49576 2.67558 2.60367
2.77027 2.22438 3.17433 5.64886 2.99921 792 t - - -
2.68618 4.42225 2.77519 2.73123 3.46354 2.40513 3.72494
3.29354 2.67741 2.69355 4.24690 2.90347 2.73739 3.18146 2.89801
2.37887 2.77519 2.98518 4.58477 3.61503
0.08131 4.76736 2.66514 0.61958 0.77255 0.97669 0.47250
438 2.78674 5.15706 3.09581 2.53898 4.44898 2.74218 3.77672
3.46521 2.14537 3.13280 4.21262 3.07603 3.45294 2.13800 2.77971
2.44947 2.32194 3.15474 5.62309 4.24779 793 q - - -
2.68613 4.42237 2.77498 2.73100 3.46366 2.40525 3.72506
3.29335 2.67738 2.69367 4.24702 2.90338 2.73752 3.18158 2.89813
2.37899 2.77478 2.98530 4.58489 3.61515
0.60831 0.96246 2.60669 0.09263 2.42509 0.82915 0.57345
439 2.63931 3.55284 4.29449 3.70658 2.79414 3.40827 4.22400
1.94469 3.58420 2.09527 3.40225 2.73506 3.55732 3.36406 3.28121
3.20677 2.80152 2.54054 3.68544 2.90017 795 i - - -
2.68632 4.42239 2.77514 2.73118 3.46368 2.40526 3.72508
3.29368 2.67754 2.69369 4.24703 2.90323 2.73698 3.18160 2.89749
2.37890 2.77514 2.98532 4.58491 3.61517
0.20526 2.17430 2.63283 1.17655 0.36866 0.96452 0.47992
440 2.80108 4.55899 2.66239 3.05571 2.90844 3.33810 4.01330
2.37798 3.01591 2.77311 3.66019 3.10235 4.14591 3.32407 3.04868
2.77986 2.33509 2.25510 5.13582 2.81265 801 v - - -
2.68618 4.42225 2.77519 2.73123 3.46354 2.40513 3.72494
3.29354 2.67741 2.69355 4.24690 2.90347 2.73739 3.18146 2.89801
2.37887 2.77519 2.98518 4.58477 3.61503
0.04292 4.78910 3.39037 0.61958 0.77255 0.80810 0.59005
441 2.28911 4.32176 4.28653 3.28619 2.62756 3.43577 3.10117
2.49213 3.58303 2.38737 3.43641 3.91442 4.30978 3.80555 3.76892
2.86067 2.59384 2.25341 3.80675 2.18698 802 y - - -
2.68618 4.42225 2.77519 2.73123 3.46354 2.40513 3.72494
3.29354 2.67741 2.69355 4.24690 2.90347 2.73739 3.18146 2.89801
2.37887 2.77519 2.98518 4.58477 3.61503
0.01149 4.86760 5.58994 0.61958 0.77255 0.66688 0.72013
442 2.64435 5.20095 2.74569 2.45595 4.47814 1.82774 3.87255
3.16087 2.64227 3.14860 4.26067 2.59662 4.06754 2.99717 3.12037
2.39034 2.88769 3.55466 5.67701 3.10400 803 g - - -

```
         2.68618  4.42225  2.77519  2.73123  3.46354  2.40513  3.72494
3.29354  2.67741  2.69355  4.24690  2.90347  2.73739  3.18146  2.89801
2.37887  2.77519  2.98518  4.58477  3.61503
         0.16579  4.97874  1.92487  0.61958  0.77255  0.87364  0.54030
    443  2.61795  5.16915  3.11981  2.40025  4.50751  2.88227  3.84810
3.95672  2.61899  3.12591  4.27965  2.31556  4.02110  2.97025  3.09916
1.52790  2.61023  3.57043  5.68964  4.31695    804 s - - -
         2.68618  4.42225  2.77519  2.73123  3.46354  2.40513  3.72494
3.29354  2.67741  2.69355  4.24690  2.90347  2.73739  3.18146  2.89801
2.37887  2.77519  2.98518  4.58477  3.61503
         0.01200  4.82494  5.54729  0.61958  0.77255  0.96521  0.47949
    444  2.63188  5.02310  2.92068  2.66954  3.61874  2.81879  3.86142
3.67669  2.40737  2.75603  4.09404  3.18889  3.42549  2.78296  3.12629
2.22950  2.18457  2.94481  5.52639  3.24429    805 t - - -
         2.68618  4.42225  2.77519  2.73123  3.46354  2.40513  3.72494
3.29354  2.67741  2.69355  4.24690  2.90347  2.73739  3.18146  2.89801
2.37887  2.77519  2.98518  4.58477  3.61503
         0.03858  4.86472  3.50235  0.61958  0.77255  0.41593  1.07800
    445  2.89720  5.33567  2.69699  2.61834  4.65174  3.30754  3.87281
3.63119  2.62030  3.26779  4.38438  1.73478  3.55804  2.60139  3.10781
2.40237  2.13630  3.71070  5.78638  3.77358    806 n - - -
         2.68618  4.42225  2.77519  2.73123  3.46354  2.40513  3.72494
3.29354  2.67741  2.69355  4.24690  2.90347  2.73739  3.18146  2.89801
2.37887  2.77519  2.98518  4.58477  3.61503
         0.00961  5.04561  5.76796  0.61958  0.77255  0.73491  0.65306
    446  2.54241  5.37344  3.15686  2.00767  4.70510  3.67408  2.94258
3.25746  2.20000  3.33268  4.41764  2.74159  4.06722  2.44553  2.67792
2.39766  2.89403  3.36018  5.81420  3.36253    807 e - - -
         2.68618  4.42225  2.77519  2.73123  3.46354  2.40513  3.72494
3.29354  2.67741  2.69355  4.24690  2.90347  2.73739  3.18146  2.89801
2.37887  2.77519  2.98518  4.58477  3.61503
         0.37608  5.04561  1.18087  0.61958  0.77255  0.51566  0.90909
    447  2.61495  3.95476  2.85801  2.55749  4.38954  2.93010  3.78590
3.83007  2.40510  3.09239  4.17224  3.09027  2.39610  2.91064  2.76795
2.25650  2.26951  3.46537  5.58868  4.22352    808 s - - -
         2.68615  4.42241  2.77518  2.73102  3.46329  2.40516  3.72510
3.29303  2.67757  2.69334  4.24706  2.90339  2.73734  3.18162  2.89817
2.37903  2.77536  2.98509  4.58493  3.61519
         0.22980  2.07160  2.53427  1.27123  0.32918  0.86497  0.54656
    448  2.47285  5.17609  2.59636  2.55434  3.71037  2.57966  3.80279
3.95692  2.56409  3.49102  4.26012  2.83309  2.50502  2.91864  3.04748
1.96271  2.71079  3.56461  5.66816  4.28847    814 s - - -
         2.68618  4.42225  2.77519  2.73123  3.46354  2.40513  3.72494
3.29354  2.67741  2.69355  4.24690  2.90347  2.73739  3.18146  2.89801
2.37887  2.77519  2.98518  4.58477  3.61503
         0.04834  4.78696  3.24806  0.61958  0.77255  0.28220  1.40293
    449  2.73985  5.35572  2.97789  2.34366  3.96818  3.05176  3.88568
3.23272  2.38096  2.52351  4.40351  2.71109  2.29644  2.78756  2.73802
2.56748  2.75524  3.73189  5.80478  4.41058    815 p - - -
         2.68618  4.42225  2.77519  2.73123  3.46354  2.40513  3.72494
3.29354  2.67741  2.69355  4.24690  2.90347  2.73739  3.18146  2.89801
2.37887  2.77519  2.98518  4.58477  3.61503
         0.00927  5.08141  5.80376  0.61958  0.77255  0.64733  0.74116
```

```
    450   2.93864  4.51101  4.11243  3.20634  3.23104  2.63710  4.26745
2.53303  3.45768  2.52540  3.62160  3.10456  4.36132  3.05988  3.72862
3.25425  2.22961  2.44517  2.35678  2.52235    816 t - - -
          2.68618  4.42225  2.77519  2.73123  3.46354  2.40513  3.72494
3.29354  2.67741  2.69355  4.24690  2.90347  2.73739  3.18146  2.89801
2.37887  2.77519  2.98518  4.58477  3.61503
          0.21962  5.08141  1.65566  0.61958  0.77255  0.50226  0.92928
    451   2.84290  5.34071  2.47091  2.28467  4.68006  2.57716  3.80564
4.16225  2.37147  3.26356  4.38303  2.22868  3.55772  2.73224  2.80189
2.62536  3.07421  3.72831  5.77663  2.58027    817 n - - -
          2.68618  4.42225  2.77519  2.73123  3.46354  2.40513  3.72494
3.29354  2.67741  2.69355  4.24690  2.90347  2.73739  3.18146  2.89801
2.37887  2.77519  2.98518  4.58477  3.61503
          0.01100  4.91116  5.63351  0.61958  0.77255  0.54217  0.87104
    452   2.68062  4.75236  3.61234  3.04882  2.79378  2.06254  4.07481
3.03431  2.82270  2.77777  3.84855  3.50028  3.59011  3.03766  2.88198
2.32834  2.88671  2.16342  5.31671  4.05836    818 g - - -
          2.68632  4.41983  2.77533  2.73137  3.46331  2.40515  3.72508
3.29313  2.67722  2.69348  4.24704  2.90339  2.73753  3.18160  2.89815
2.37901  2.77533  2.98532  4.58491  3.61445
          0.76385  1.09788  1.60669  0.08209  2.54074  0.75392  0.63586
    453   2.80598  5.10835  2.92280  2.59174  4.37701  3.61598  3.81357
3.35780  2.58813  2.67235  4.17031  2.88628  2.84489  2.75578  2.37226
2.08559  2.39945  3.10620  5.58999  4.23084    820 s - - -
          2.68618  4.42225  2.77519  2.73123  3.46354  2.40513  3.72494
3.29354  2.67741  2.69355  4.24690  2.90347  2.73739  3.18146  2.89801
2.37887  2.77519  2.98518  4.58477  3.61503
          0.08134  4.81734  2.65884  0.61958  0.77255  0.99860  0.45949
    454   2.79213  4.03538  2.60428  2.36951  4.61916  2.69401  3.36859
4.09916  2.00960  3.25644  4.32536  2.83727  3.96222  2.37902  2.54826
2.75833  2.62298  3.26619  5.72011  4.31443    821 k - - -
          2.68618  4.42225  2.77519  2.73123  3.46354  2.40513  3.72494
3.29354  2.67741  2.69355  4.24690  2.90347  2.73739  3.18146  2.89801
2.37887  2.77519  2.98518  4.58477  3.61503
          0.01250  4.78446  5.50681  0.61958  0.77255  1.15935  0.37643
    455   2.62131  4.39616  3.92625  3.35193  3.51505  3.84052  4.11703
2.00348  3.27832  2.43698  3.50627  2.88292  4.21566  3.54973  3.14850
2.82123  1.93087  2.19251  4.99502  3.28667    822 t - - -
          2.68618  4.42225  2.77519  2.73123  3.46354  2.40513  3.72494
3.29354  2.67741  2.69355  4.24690  2.90347  2.73739  3.18146  2.89801
2.37887  2.77519  2.98518  4.58477  3.61503
          0.01250  4.78446  5.50681  0.61958  0.77255  0.50811  0.92038
    456   2.89962  4.53413  3.37288  3.36144  2.78253  3.04510  4.18542
2.41509  3.29960  2.39976  3.24130  2.71280  3.61748  3.00026  3.61120
2.89075  3.13145  2.30458  5.12709  2.21160    823 y - - -
          2.68618  4.42225  2.77519  2.73123  3.46354  2.40513  3.72494
3.29354  2.67741  2.69355  4.24690  2.90347  2.73739  3.18146  2.89801
2.37887  2.77519  2.98518  4.58477  3.61503
          0.01000  5.00636  5.72871  0.61958  0.77255  0.59111  0.80679
    457   2.40742  5.31496  2.26368  2.37944  2.96102  3.25225  3.44861
3.51672  2.63634  2.98333  3.72716  2.54258  4.08513  2.59045  3.12165
2.42389  2.71196  3.68446  5.77173  4.38561    824 d - - -
          2.68618  4.42225  2.77519  2.73123  3.46354  2.40513  3.72494
3.29354  2.67741  2.69355  4.24690  2.90347  2.73739  3.18146  2.89801
2.37887  2.77519  2.98518  4.58477  3.61503
```

```
        0.04148  5.06056  3.37300  0.61958  0.77255  0.55457  0.85406
    458  2.90216  5.39354  2.92224  2.36707  4.73053  2.85478  3.86673
3.20212  2.46135  3.69536  4.43635  2.38006  4.07207  2.43895  2.87479
1.90628  2.41150  3.77984  5.83106  3.66322    825 s - - -
        2.68702  4.42151  2.77579  2.73131  3.46247  2.40567  3.72462
3.29135  2.67734  2.69374  4.24807  2.90286  2.73595  3.18180  2.89834
2.37891  2.77562  2.98542  4.58594  3.61488
        0.93125  0.67631  2.32844  1.63075  0.21789  0.69758  0.68873
    459  2.67500  4.06479  2.71611  2.60819  4.54059  3.27685  3.38881
2.98788  2.22400  2.99098  4.29904  2.71275  3.58665  2.75772  2.83379
2.55499  2.37883  3.60877  5.70853  2.94601    840 k - - -
        2.68618  4.42225  2.77519  2.73123  3.46354  2.40513  3.72494
3.29354  2.67741  2.69355  4.24690  2.90347  2.73739  3.18146  2.89801
2.37887  2.77519  2.98518  4.58477  3.61503
        0.17414  4.96229  1.87849  0.61958  0.77255  0.90374  0.51928
    460  2.79859  5.18188  2.19147  2.54489  4.47750  3.59348  3.78660
3.03963  2.54419  3.08990  4.23592  2.48928  3.51573  2.70877  2.44545
2.46293  2.63091  3.16117  5.64536  3.64029    841 d - - -
        2.68618  4.42225  2.77519  2.73123  3.46354  2.40513  3.72494
3.29354  2.67741  2.69355  4.24690  2.90347  2.73739  3.18146  2.89801
2.37887  2.77519  2.98518  4.58477  3.61503
        0.05531  4.80045  3.08823  0.61958  0.77255  0.90229  0.52027
    461  2.46659  5.29494  2.36228  1.99026  3.30016  3.24065  3.33956
4.10809  2.37163  3.59570  4.33843  2.79798  3.98009  2.17775  3.00531
2.77656  2.83511  3.67990  4.15366  4.32934    842 e - - -
        2.68636  4.42243  2.77538  2.73142  3.46372  2.40531  3.72513
3.29261  2.67743  2.69210  4.24708  2.90365  2.73758  3.18165  2.89819
2.37905  2.77520  2.98537  4.58495  3.61479
        1.47814  0.61058  1.47447  0.06388  2.78248  0.98442  0.46786
    462  2.83177  4.67911  3.47944  2.68563  3.84549  3.71842  4.01507
2.77084  2.90262  2.81554  3.20371  3.41580  1.31750  3.27118  3.27096
2.99560  3.08078  2.88737  5.28469  4.02675    844 p - - -
        2.68618  4.42225  2.77519  2.73123  3.46354  2.40513  3.72494
3.29354  2.67741  2.69355  4.24690  2.90347  2.73739  3.18146  2.89801
2.37887  2.77519  2.98518  4.58477  3.61503
        0.01480  4.61657  5.33891  0.61958  0.77255  1.32412  0.30930
    463  2.73830  4.86826  3.18824  2.63960  4.11668  2.14487  3.81039
3.52223  2.43437  2.70409  3.97183  2.85192  2.46931  2.97691  3.07558
2.79461  2.53042  2.89918  5.40905  4.09099    845 g - - -
        2.68618  4.42225  2.77519  2.73123  3.46354  2.40513  3.72494
3.29354  2.67741  2.69355  4.24690  2.90347  2.73739  3.18146  2.89801
2.37887  2.77519  2.98518  4.58477  3.61503
        0.01480  4.61657  5.33891  0.61958  0.77255  0.38069  1.15009
    464  2.69805  5.30677  2.62607  1.88339  4.61569  3.24212  3.86666
3.25513  2.46910  2.95755  4.35712  2.59588  4.06793  2.78952  3.10320
2.86750  2.27579  3.02935  5.76172  3.31679    846 e - - -
        2.68618  4.42225  2.77519  2.73123  3.46354  2.40513  3.72494
3.29354  2.67741  2.69355  4.24690  2.90347  2.73739  3.18146  2.89801
2.37887  2.77519  2.98518  4.58477  3.61503
        0.00983  5.02352  5.74587  0.61958  0.77255  0.64006  0.74921
    465  2.70553  5.37238  3.16356  1.97666  4.70179  2.85280  3.86822
3.62456  2.33097  2.99435  4.41720  2.39893  2.99007  2.96979  2.72871
2.41018  2.59620  3.75458  5.81455  3.66545    847 e - - -
```

2.68618 4.42225 2.77519 2.73123 3.46354 2.40513 3.72494
3.29354 2.67741 2.69355 4.24690 2.90347 2.73739 3.18146 2.89801
2.37887 2.77519 2.98518 4.58477 3.61503
0.03673 5.05597 3.51677 0.61958 0.77255 0.59867 0.79748
466 2.90022 5.31696 2.93416 2.34678 4.62480 3.02429 3.88070
3.56526 2.49469 2.74460 4.36775 2.75333 3.05928 2.99059 3.11768
1.69393 2.48332 3.31194 5.77275 4.38546 848 s - - -
2.68631 4.42238 2.77479 2.73114 3.46366 2.40525 3.72459
3.29367 2.67753 2.69368 4.24702 2.90318 2.73698 3.18159 2.89813
2.37899 2.77514 2.98531 4.58490 3.61516
0.37619 1.67464 2.07023 0.50763 0.92110 0.55161 0.85808
467 2.67140 5.21735 2.90126 2.25952 3.76773 3.05327 3.85770
3.48136 2.46358 2.64962 4.27419 2.54534 2.69214 2.97770 3.10155
2.45169 2.51177 3.03543 5.68742 4.31554 852 e - - -
2.68618 4.42225 2.77519 2.73123 3.46354 2.40513 3.72494
3.29354 2.67741 2.69355 4.24690 2.90347 2.73739 3.18146 2.89801
2.37887 2.77519 2.98518 4.58477 3.61503
0.22150 4.96371 1.65183 0.61958 0.77255 0.90120 0.52102
468 2.78278 3.92333 2.89399 2.43287 3.23775 3.21137 3.80926
3.26030 2.60136 3.27736 4.08301 2.60216 3.09465 2.52769 2.64964
2.80847 2.49934 2.79255 5.51135 3.55082 853 e - - -
2.68618 4.42225 2.77519 2.73123 3.46354 2.40513 3.72494
3.29354 2.67741 2.69355 4.24690 2.90347 2.73739 3.18146 2.89801
2.37887 2.77519 2.98518 4.58477 3.61503
0.01287 4.75508 5.47742 0.61958 0.77255 0.42590 1.05896
469 2.89904 4.98682 3.07428 2.47190 3.52046 3.34049 3.96971
3.23339 2.80511 2.42825 4.06657 3.32038 1.62776 2.89281 3.25339
2.58394 3.13049 3.30184 5.51102 3.45105 854 p - - -
2.68618 4.42225 2.77519 2.73123 3.46354 2.40513 3.72494
3.29354 2.67741 2.69355 4.24690 2.90347 2.73739 3.18146 2.89801
2.37887 2.77519 2.98518 4.58477 3.61503
0.00973 5.03357 5.75592 0.61958 0.77255 0.76207 0.62867
470 2.72872 5.29270 2.38341 2.63007 4.59504 3.32874 3.87599
3.21348 2.48067 3.01882 4.34538 2.15940 3.59659 2.98835 3.11469
2.16510 2.20609 3.36165 5.75249 4.36912 855 n - - -
2.68618 4.42225 2.77519 2.73123 3.46354 2.40513 3.72494
3.29354 2.67741 2.69355 4.24690 2.90347 2.73739 3.18146 2.89801
2.37887 2.77519 2.98518 4.58477 3.61503
0.00973 5.03357 5.75592 0.61958 0.77255 0.55727 0.85043
471 2.34805 5.33504 2.95117 2.25898 2.98892 3.69906 3.18365
3.27921 2.49341 3.62543 4.38497 2.90169 3.21482 2.60534 2.91727
2.21928 3.14237 3.33178 5.78905 2.93240 856 s - - -
2.68625 4.42232 2.77508 2.73130 3.46361 2.40507 3.72501
3.29361 2.67747 2.69345 4.24696 2.90333 2.73746 3.18153 2.89807
2.37881 2.77526 2.98502 4.58484 3.61510
0.03537 3.45031 5.80276 1.28473 0.32397 0.50486 0.92530
472 2.92331 5.42561 2.95738 2.01398 3.93065 3.36308 3.88576
4.25068 2.16235 3.07406 4.46720 2.22172 4.09175 2.22115 2.70105
2.56881 2.67085 3.81437 4.24520 4.45157 863 e - - -
2.68618 4.42225 2.77519 2.73123 3.46354 2.40513 3.72494
3.29354 2.67741 2.69355 4.24690 2.90347 2.73739 3.18146 2.89801
2.37887 2.77519 2.98518 4.58477 3.61503
0.00900 5.11136 5.83370 0.61958 0.77255 0.56492 0.84027

```
    473   2.45814  5.43465  2.13235  2.33838  4.77929  3.08492  3.45523
4.26510  2.26455  2.94367  4.47548  2.14724  3.58226  2.63031  3.11344
2.55810  2.94734  3.82507  5.86752  4.45660    864 d - - -
          2.68618  4.42225  2.77519  2.73123  3.46354  2.40513  3.72494
3.29354  2.67741  2.69355  4.24690  2.90347  2.73739  3.18146  2.89801
2.37887  2.77519  2.98518  4.58477  3.61503
          0.00900  5.11136  5.83370  0.61958  0.77255  0.56492  0.84027
    474   2.70060  3.79016  4.27423  3.69409  2.56817  4.03741  3.24366
2.94189  3.59618  2.47654  3.59799  3.47493  2.86329  3.83938  3.82431
3.31422  2.92367  2.74225  5.07700  1.36902    865 y - - -
          2.68618  4.42225  2.77519  2.73123  3.46354  2.40513  3.72494
3.29354  2.67741  2.69355  4.24690  2.90347  2.73739  3.18146  2.89801
2.37887  2.77519  2.98518  4.58477  3.61503
          0.00900  5.11136  5.83370  0.61958  0.77255  0.56492  0.84027
    475   2.97501  5.33399  3.21768  2.36395  4.63109  3.74546  3.96231
4.07942  2.55962  3.62337  4.40304  2.98603  4.15336  3.08441  3.20370
1.35409  1.83487  3.70213  5.80972  3.63411    866 s - - -
          2.68618  4.42225  2.77519  2.73123  3.46354  2.40513  3.72494
3.29354  2.67741  2.69355  4.24690  2.90347  2.73739  3.18146  2.89801
2.37887  2.77519  2.98518  4.58477  3.61503
          0.00900  5.11136  5.83370  0.61958  0.77255  0.43927  1.03425
    476   4.05676  3.53947  5.13829  4.92479  4.52323  4.55669  0.20500
5.11902  4.54712  4.64311  5.70918  4.99426  5.24818  5.08013  4.68455
4.24385  4.51805  4.74054  6.00207  4.50475    867 H - - -
          2.68618  4.42225  2.77519  2.73123  3.46354  2.40513  3.72494
3.29354  2.67741  2.69355  4.24690  2.90347  2.73739  3.18146  2.89801
2.37887  2.77519  2.98518  4.58477  3.61503
          0.00877  5.13716  5.85950  0.61958  0.77255  0.48576  0.95510
    477   2.95030  5.01695  3.44364  2.69484  3.63883  3.81357  3.55745
2.67274  2.86347  2.75971  4.09818  3.09163  4.20092  2.71925  1.44370
2.77730  3.18141  3.32954  5.54336  2.94131    868 r - - -
          2.68618  4.42225  2.77519  2.73123  3.46354  2.40513  3.72494
3.29354  2.67741  2.69355  4.24690  2.90347  2.73739  3.18146  2.89801
2.37887  2.77519  2.98518  4.58477  3.61503
          0.00877  5.13716  5.85950  0.61958  0.77255  0.48576  0.95510
    478   3.78596  5.10886  5.53964  4.97483  3.01649  4.99659  5.21173
2.53593  4.80092  0.45048  3.50809  3.72366  5.22926  4.85777  4.83492
4.33931  4.00852  2.99127  5.49322  4.28339    869 L - - -
          2.68618  4.42225  2.77519  2.73123  3.46354  2.40513  3.72494
3.29354  2.67741  2.69355  4.24690  2.90347  2.73739  3.18146  2.89801
2.37887  2.77519  2.98518  4.58477  3.61503
          0.08565  5.13716  2.57429  0.61958  0.77255  0.48576  0.95510
    479   2.90298  3.31345  3.91801  3.39926  3.48427  3.20657  4.42236
3.73192  3.37225  3.41493  4.26975  2.85613  4.30399  3.68190  3.75001
0.84042  2.69160  3.39425  5.72556  4.47899    870 s - - -
          2.68618  4.42225  2.77519  2.73123  3.46354  2.40513  3.72494
3.29354  2.67741  2.69355  4.24690  2.90347  2.73739  3.18146  2.89801
2.37887  2.77519  2.98518  4.58477  3.61503
          0.05699  5.06097  3.01471  0.61958  0.77255  0.69866  0.68767
    480   2.89108  5.16857  2.56905  2.68892  3.34704  3.70518  2.49032
3.86047  2.68439  3.43599  4.23278  2.75285  4.09605  2.80373  2.65445
2.67140  3.12230  3.51390  3.11334  2.14742    871 y - - -
          2.68618  4.42225  2.77519  2.73123  3.46354  2.40513  3.72494
3.29354  2.67741  2.69355  4.24690  2.90347  2.73739  3.18146  2.89801
2.37887  2.77519  2.98518  4.58477  3.61503
```

```
          0.00992  5.01390  5.73624  0.61958  0.77255  0.69720  0.68911
    481   2.29086  3.60884  4.92605  4.31370  2.79877  4.14192  4.47957
1.56596  4.10239  2.17142  2.75965  4.30262  4.50232  4.23506  4.11397
3.45473  3.19684  1.66975  4.97118  3.29475    872 i - - -
          2.68618  4.42225  2.77519  2.73123  3.46354  2.40513  3.72494
3.29354  2.67741  2.69355  4.24690  2.90347  2.73739  3.18146  2.89801
2.37887  2.77519  2.98518  4.58477  3.61503
          0.00968  5.03792  5.76027  0.61958  0.77255  0.75238  0.63723
    482   2.55662  5.22950  2.78351  2.53506  4.50847  3.31468  3.89408
2.88095  2.26569  3.50612  3.64619  3.19796  3.20713  2.76528  3.14024
2.03501  2.25464  3.20106  4.17369  4.33680    873 s - - -
          2.68618  4.42225  2.77519  2.73123  3.46354  2.40513  3.72494
3.29354  2.67741  2.69355  4.24690  2.90347  2.73739  3.18146  2.89801
2.37887  2.77519  2.98518  4.58477  3.61503
          0.00968  5.03792  5.76027  0.61958  0.77255  0.75238  0.63723
    483   1.95740  3.33991  4.17410  3.20219  2.93692  3.03955  4.27064
2.92021  3.50590  2.08984  2.93572  2.77616  4.35632  3.75761  3.75212
2.79964  2.63146  2.55623  5.06403  2.93670    874 a - - -
          2.68618  4.42225  2.77519  2.73123  3.46354  2.40513  3.72494
3.29354  2.67741  2.69355  4.24690  2.90347  2.73739  3.18146  2.89801
2.37887  2.77519  2.98518  4.58477  3.61503
          0.05989  5.03792  2.96342  0.61958  0.77255  0.75238  0.63723
    484   2.29797  3.14788  4.38134  3.29431  2.68044  4.00308  3.72684
2.46286  3.67015  2.15919  3.48337  3.43917  4.37232  3.03932  3.84360
3.01069  2.63334  1.91468  4.98015  2.99594    875 v - - -
          2.68618  4.42225  2.77519  2.73123  3.46354  2.40513  3.72494
3.29354  2.67741  2.69355  4.24690  2.90347  2.73739  3.18146  2.89801
2.37887  2.77519  2.98518  4.58477  3.61503
          0.01018  4.98822  5.71056  0.61958  0.77255  0.65352  0.73441
    485   2.53781  5.01916  3.35212  2.62750  3.70358  3.04806  3.95869
2.94308  2.78306  3.03798  4.09632  2.97676  2.77222  2.62541  2.80428
2.47914  2.51650  2.81358  5.53738  2.78280    876 s - - -
          2.68618  4.42225  2.77519  2.73123  3.46354  2.40513  3.72494
3.29354  2.67741  2.69355  4.24690  2.90347  2.73739  3.18146  2.89801
2.37887  2.77519  2.98518  4.58477  3.61503
          0.03699  5.03648  3.51260  0.61958  0.77255  0.75560  0.63437
    486   2.22623  4.91474  3.41504  2.62450  3.30163  3.04611  3.14283
3.14905  2.67547  3.15295  2.99910  2.78218  3.64399  3.17811  3.27753
2.44029  2.38287  2.71986  5.45003  3.49046    877 a - - -
          2.68618  4.42225  2.77519  2.73123  3.46354  2.40513  3.72494
3.29354  2.67741  2.69355  4.24690  2.90347  2.73739  3.18146  2.89801
2.37887  2.77519  2.98518  4.58477  3.61503
          0.10427  5.00946  2.38222  0.61958  0.77255  0.67918  0.70732
    487   2.85647  5.34679  2.50174  2.55686  4.68365  2.35870  3.82077
4.16361  2.16975  3.64843  4.38977  3.10361  4.02629  2.39412  2.34956
2.40040  2.54060  3.30893  5.78431  3.65502    878 k - - -
          2.68618  4.42225  2.77519  2.73123  3.46354  2.40513  3.72494
3.29354  2.67741  2.69355  4.24690  2.90347  2.73739  3.18146  2.89801
2.37887  2.77519  2.98518  4.58477  3.61503
          0.17110  4.94966  1.89593  0.61958  0.77255  0.80573  0.59197
    488   2.80776  4.04652  2.84191  1.98689  3.80860  3.59748  3.78942
3.98385  2.14169  2.89762  4.27030  2.67213  3.99012  2.90017  3.02684
2.21633  2.25865  3.58832  5.67601  4.29012    879 e - - -
```

2.68618 4.42225 2.77519 2.73123 3.46354 2.40513 3.72494
3.29354 2.67741 2.69355 4.24690 2.90347 2.73739 3.18146 2.89801
2.37887 2.77519 2.98518 4.58477 3.61503
0.22879 4.82474 1.62721 0.61958 0.77255 0.90285 0.51989
489 2.58861 4.98588 2.85863 2.58284 4.23219 2.52677 3.78445
2.92042 2.41379 2.73434 3.42536 2.27709 3.49443 2.92842 3.04467
2.60304 2.76621 3.32231 5.48317 4.14140 880 n - - -
2.68618 4.42225 2.77519 2.73123 3.46354 2.40513 3.72494
3.29354 2.67741 2.69355 4.24690 2.90347 2.73739 3.18146 2.89801
2.37887 2.77519 2.98518 4.58477 3.61503
0.01385 4.68252 5.40487 0.61958 0.77255 1.14452 0.38328
490 2.42756 5.20155 2.83951 2.49414 4.51525 2.73916 2.97240
3.49755 2.49487 3.49343 4.25082 2.81757 3.94858 2.21690 2.76122
2.29615 2.72607 3.07607 5.65371 4.26264 881 q - - -
2.68618 4.42225 2.77519 2.73123 3.46354 2.40513 3.72494
3.29354 2.67741 2.69355 4.24690 2.90347 2.73739 3.18146 2.89801
2.37887 2.77519 2.98518 4.58477 3.61503
0.15537 4.72044 2.00254 0.61958 0.77255 0.63232 0.75791
491 2.62984 3.70820 3.04086 2.61028 3.98133 2.74285 2.84602
2.43927 2.79211 2.35497 3.88308 3.29590 3.48313 3.12739 2.93235
2.48043 3.04175 2.83980 5.33813 4.05087 882 l - - -
2.68618 4.42225 2.77519 2.73123 3.46354 2.40513 3.72494
3.29354 2.67741 2.69355 4.24690 2.90347 2.73739 3.18146 2.89801
2.37887 2.77519 2.98518 4.58477 3.61503
0.01206 4.82001 5.54235 0.61958 0.77255 0.79416 0.60141
492 2.64946 4.88587 3.08299 2.80418 2.93389 3.33570 3.93632
3.12941 2.56622 3.12535 3.09212 2.72527 2.67361 3.12893 2.85945
2.42748 2.61814 3.19416 4.12245 3.14225 883 s - - -
2.68608 4.42281 2.77531 2.73162 3.46373 2.40496 3.72504
3.29410 2.67797 2.69364 4.24746 2.90319 2.73680 3.18175 2.89837
2.37920 2.77423 2.98553 4.58429 3.61273
0.40195 1.11650 5.63708 1.46567 0.26257 0.69335 0.69295
493 2.53770 5.26965 2.69363 2.61464 3.81219 2.62436 3.34371
2.91545 2.46511 3.24544 4.32252 2.25197 2.74600 2.97249 3.09852
2.29586 3.10528 3.63587 5.73035 4.34833 899 n - - -
2.68618 4.42225 2.77519 2.73123 3.46354 2.40513 3.72494
3.29354 2.67741 2.69355 4.24690 2.90347 2.73739 3.18146 2.89801
2.37887 2.77519 2.98518 4.58477 3.61503
0.03894 4.99168 3.46094 0.61958 0.77255 0.46342 0.99190
494 2.60794 5.38880 2.11588 2.46363 4.72341 3.07646 3.86905
4.20209 2.30976 3.04443 4.43223 2.69715 3.56187 2.70138 3.09898
2.19395 2.92011 3.13196 5.82779 3.06881 900 d - - -
2.68618 4.42225 2.77519 2.73123 3.46354 2.40513 3.72494
3.29354 2.67741 2.69355 4.24690 2.90347 2.73739 3.18146 2.89801
2.37887 2.77519 2.98518 4.58477 3.61503
0.11923 5.06436 2.24355 0.61958 0.77255 0.69040 0.69590
495 2.65935 4.12745 2.67716 2.30065 4.68321 2.90833 3.82261
4.16299 2.41326 3.64850 4.38998 2.41237 3.51609 2.70267 2.67533
2.36617 2.63346 3.33643 5.78501 2.87893 901 e - - -
2.68618 4.42225 2.77519 2.73123 3.46354 2.40513 3.72494
3.29354 2.67741 2.69355 4.24690 2.90347 2.73739 3.18146 2.89801
2.37887 2.77519 2.98518 4.58477 3.61503
0.01052 4.95564 5.67799 0.61958 0.77255 0.28817 1.38484

```
    496   2.49611  5.31867  3.24130  2.39880  3.45515  3.07498  3.92599
3.20638  2.32558  3.28357  4.37289  2.46807  3.60187  2.59485  2.32910
2.92653  2.89711  3.31550  5.78264  2.73800    902 k - - -
          2.68618  4.42225  2.77519  2.73123  3.46354  2.40513  3.72494
3.29354  2.67741  2.69355  4.24690  2.90347  2.73739  3.18146  2.89801
2.37887  2.77519  2.98518  4.58477  3.61503
          0.00877  5.13716  5.85950  0.61958  0.77255  0.48576  0.95510
    497   2.60965  5.30576  2.99793  2.56122  4.59597  2.47567  3.48319
2.72504  2.55890  2.80445  4.36128  2.33876  3.24126  3.04708  2.94548
2.44859  2.75961  3.24254  5.77276  3.66170    903 n - - -
          2.68618  4.42225  2.77519  2.73123  3.46354  2.40513  3.72494
3.29354  2.67741  2.69355  4.24690  2.90347  2.73739  3.18146  2.89801
2.37887  2.77519  2.98518  4.58477  3.61503
          0.00877  5.13716  5.85950  0.61958  0.77255  0.48576  0.95510
    498   2.93580  5.39123  2.94123  2.65078  3.91676  2.65277  3.15271
3.70216  2.18481  3.32212  3.38676  3.19503  4.11129  2.48236  2.33762
2.10840  2.49923  3.76972  5.83761  4.44068    904 s - - -
          2.68618  4.42225  2.77519  2.73123  3.46354  2.40513  3.72494
3.29354  2.67741  2.69355  4.24690  2.90347  2.73739  3.18146  2.89801
2.37887  2.77519  2.98518  4.58477  3.61503
          0.00877  5.13716  5.85950  0.61958  0.77255  0.48576  0.95510
    499   2.10406  3.76457  4.51816  3.45809  3.23933  2.93288  4.39700
2.15205  3.79409  2.34344  3.16502  3.52790  3.73618  4.00041  3.48536
2.38976  3.20511  1.85781  3.95438  3.85354    905 v - - -
          2.68618  4.42225  2.77519  2.73123  3.46354  2.40513  3.72494
3.29354  2.67741  2.69355  4.24690  2.90347  2.73739  3.18146  2.89801
2.37887  2.77519  2.98518  4.58477  3.61503
          0.00877  5.13716  5.85950  0.61958  0.77255  0.48576  0.95510
    500   2.97661  4.38012  4.76628  4.16230  2.65308  4.12582  4.44936
2.58300  3.98405  2.01675  3.13155  3.59994  2.27473  3.56876  4.05347
3.09243  3.20874  2.21374  3.45443  1.95997    906 y - - -
          2.68618  4.42225  2.77519  2.73123  3.46354  2.40513  3.72494
3.29354  2.67741  2.69355  4.24690  2.90347  2.73739  3.18146  2.89801
2.37887  2.77519  2.98518  4.58477  3.61503
          0.00877  5.13716  5.85950  0.61958  0.77255  0.48576  0.95510
    501   2.11269  3.30000  4.79401  4.18851  3.17665  3.19651  4.45625
2.31638  4.00502  2.25392  2.91086  4.24298  4.49546  4.16382  4.06527
2.20924  2.40125  1.82716  4.99636  3.81673    907 v - - -
          2.68618  4.42225  2.77519  2.73123  3.46354  2.40513  3.72494
3.29354  2.67741  2.69355  4.24690  2.90347  2.73739  3.18146  2.89801
2.37887  2.77519  2.98518  4.58477  3.61503
          0.00877  5.13716  5.85950  0.61958  0.77255  0.48576  0.95510
    502   3.39763  3.18928  5.14613  4.61000  1.04954  4.49350  2.77649
2.75502  4.41303  2.44151  3.86489  4.52447  4.84206  4.49393  4.42697
3.81183  3.62674  3.02367  4.68237  1.87168    908 f - - -
          2.68618  4.42225  2.77519  2.73123  3.46354  2.40513  3.72494
3.29354  2.67741  2.69355  4.24690  2.90347  2.73739  3.18146  2.89801
2.37887  2.77519  2.98518  4.58477  3.61503
          0.00877  5.13716  5.85950  0.61958  0.77255  0.48576  0.95510
    503   1.80141  5.21001  3.30867  2.59434  4.48453  1.73291  3.42317
3.91308  2.76467  3.49514  4.29394  3.28773  4.16070  2.86084  3.23494
2.35442  2.98011  2.82416  5.71739  4.36775    909 g - - -
          2.68618  4.42225  2.77519  2.73123  3.46354  2.40513  3.72494
3.29354  2.67741  2.69355  4.24690  2.90347  2.73739  3.18146  2.89801
2.37887  2.77519  2.98518  4.58477  3.61503
```

```
          0.00877  5.13716  5.85950  0.61958  0.77255  0.48576  0.95510
    504   5.83012  6.65118  6.16933  6.19225  4.80926  5.37105  6.03326
6.19234  6.10274  5.32469  6.71356  6.26417  5.97799  6.39470  5.97511
6.05574  6.20415  6.06373  0.05941  4.77909    910 W - - -
          2.68618  4.42225  2.77519  2.73123  3.46354  2.40513  3.72494
3.29354  2.67741  2.69355  4.24690  2.90347  2.73739  3.18146  2.89801
2.37887  2.77519  2.98518  4.58477  3.61503
          0.00877  5.13716  5.85950  0.61958  0.77255  0.48576  0.95510
    505   2.56367  4.80256  3.66355  2.82343  3.96135  3.88914  4.12578
2.60279  2.81608  2.81946  3.15898  2.94548  4.27250  3.39101  3.46217
3.12045  1.21882  3.08538  5.36784  4.10991    911 t - - -
          2.68618  4.42225  2.77519  2.73123  3.46354  2.40513  3.72494
3.29354  2.67741  2.69355  4.24690  2.90347  2.73739  3.18146  2.89801
2.37887  2.77519  2.98518  4.58477  3.61503
          0.00877  5.13716  5.85950  0.61958  0.77255  0.48576  0.95510
    506   3.38902  5.47453  3.59127  3.16898  4.24547  4.07603  0.76629
4.18557  2.56675  3.73712  4.61540  3.63873  4.51303  3.45842  3.21988
2.57043  3.62074  3.88227  5.61466  3.36697    912 h - - -
          2.68618  4.42225  2.77519  2.73123  3.46354  2.40513  3.72494
3.29354  2.67741  2.69355  4.24690  2.90347  2.73739  3.18146  2.89801
2.37887  2.77519  2.98518  4.58477  3.61503
          0.00877  5.13716  5.85950  0.61958  0.77255  0.48576  0.95510
    507   2.50223  5.24666  3.27959  2.58642  3.81042  3.74852  3.94574
3.49265  2.10429  3.51927  3.68731  2.49022  4.13974  2.83470  2.39117
2.62281  2.25368  2.60277  5.72713  3.73614    913 k - - -
          2.68618  4.42225  2.77519  2.73123  3.46354  2.40513  3.72494
3.29354  2.67741  2.69355  4.24690  2.90347  2.73739  3.18146  2.89801
2.37887  2.77519  2.98518  4.58477  3.61503
          0.00877  5.13716  5.85950  0.61958  0.77255  0.48576  0.95510
    508   3.15623  5.42952  2.65438  2.96779  5.12880  3.16236  4.36864
4.61066  3.23947  4.14525  4.92638  2.92207  4.35886  3.51413  3.73944
0.77536  2.38522  4.11821  6.31125  4.92302    914 s - - -
          2.68618  4.42225  2.77519  2.73123  3.46354  2.40513  3.72494
3.29354  2.67741  2.69355  4.24690  2.90347  2.73739  3.18146  2.89801
2.37887  2.77519  2.98518  4.58477  3.61503
          0.00877  5.13716  5.85950  0.61958  0.77255  0.48576  0.95510
    509   2.02769  4.83632  5.56534  5.01746  4.05473  4.93995  5.41052
1.67472  4.87823  2.03312  3.32418  5.08906  5.23390  5.05193  4.95283
4.29684  3.80299  0.95225  5.84340  4.65995    915 v - - -
          2.68618  4.42225  2.77519  2.73123  3.46354  2.40513  3.72494
3.29354  2.67741  2.69355  4.24690  2.90347  2.73739  3.18146  2.89801
2.37887  2.77519  2.98518  4.58477  3.61503
          0.00877  5.13716  5.85950  0.61958  0.77255  0.48576  0.95510
    510   2.95917  5.47458  1.63358  2.15172  4.81883  3.33565  3.91529
4.30637  2.35531  3.77946  4.51649  2.46145  4.11831  2.76695  2.90489
2.32843  2.58752  3.86498  5.90662  4.49260    916 d - - -
          2.68618  4.42225  2.77519  2.73123  3.46354  2.40513  3.72494
3.29354  2.67741  2.69355  4.24690  2.90347  2.73739  3.18146  2.89801
2.37887  2.77519  2.98518  4.58477  3.61503
          0.03358  5.13716  3.60642  0.61958  0.77255  0.48576  0.95510
    511   2.67096  5.22470  2.77120  2.52853  2.95195  3.74027  3.93820
3.92486  2.55852  2.40228  4.28708  3.24826  2.14555  3.06879  2.20125
2.56609  3.16025  3.57227  5.70761  3.79292    917 p - - -
```

2.68618 4.42225 2.77519 2.73123 3.46354 2.40513 3.72494
3.29354 2.67741 2.69355 4.24690 2.90347 2.73739 3.18146 2.89801
2.37887 2.77519 2.98518 4.58477 3.61503
0.00898 5.11256 5.83491 0.61958 0.77255 0.44121 1.03075
512 2.93755 5.42233 2.49324 2.07282 4.03697 3.71391 3.90315
4.23485 2.35876 3.33423 4.46599 1.97773 4.10787 2.80369 3.13376
2.90470 2.08228 3.80707 5.86150 2.95050 918 n - - -
2.68609 4.42233 2.77509 2.73131 3.46362 2.40495 3.72502
3.29362 2.67749 2.69346 4.24698 2.90355 2.73748 3.18154 2.89809
2.37895 2.77527 2.98503 4.58485 3.61468
0.03380 3.49401 5.85950 1.40067 0.28294 0.48576 0.95510
513 3.68796 6.27621 2.50686 2.80992 5.60815 2.86496 3.39616
5.19062 3.57183 4.65944 5.51701 0.54882 4.57161 3.69035 4.18690
3.56484 3.99642 4.72581 6.79028 5.25013 926 N - - -
2.68618 4.42225 2.77519 2.73123 3.46354 2.40513 3.72494
3.29354 2.67741 2.69355 4.24690 2.90347 2.73739 3.18146 2.89801
2.37887 2.77519 2.98518 4.58477 3.61503
0.00877 5.13716 5.85950 0.61958 0.77255 0.48576 0.95510
514 2.93716 5.33087 2.94092 2.27027 4.63019 3.33144 3.92284
2.99591 2.67842 3.01787 4.38385 2.77739 3.60694 2.85015 2.16731
2.73001 2.05623 3.09648 5.79195 3.27949 927 t - - -
2.68620 4.42227 2.77522 2.73125 3.46356 2.40515 3.72497
3.29356 2.67743 2.69357 4.24692 2.90349 2.73742 3.18148 2.89803
2.37889 2.77522 2.98520 4.58479 3.61419
0.05936 2.90451 5.85950 0.29591 1.36200 0.48576 0.95510
515 3.71517 4.97286 5.73292 5.18797 3.98127 5.11945 5.56406
0.73867 5.04946 1.78494 3.81693 5.27205 5.36645 5.17337 5.10261
4.48706 3.95503 1.74193 5.89736 3.59084 929 i - - -
2.68618 4.42225 2.77519 2.73123 3.46354 2.40513 3.72494
3.29354 2.67741 2.69355 4.24690 2.90347 2.73739 3.18146 2.89801
2.37887 2.77519 2.98518 4.58477 3.61503
0.00877 5.13716 5.85950 0.61958 0.77255 0.48576 0.95510
516 2.46859 5.31997 2.38631 2.40452 3.14125 3.73252 3.49479
4.06763 2.36992 3.26101 4.37406 3.22252 4.12465 3.04095 2.71404
2.19308 2.76476 3.00561 5.78363 2.63356 930 s - - -
2.68618 4.42225 2.77519 2.73123 3.46354 2.40513 3.72494
3.29354 2.67741 2.69355 4.24690 2.90347 2.73739 3.18146 2.89801
2.37887 2.77519 2.98518 4.58477 3.61503
0.00877 5.13716 5.85950 0.61958 0.77255 0.48576 0.95510
517 2.49292 5.42760 2.55873 2.36946 4.76466 3.71205 3.90044
3.62854 2.32101 3.72949 4.47046 2.58346 2.30854 2.99944 3.13007
2.11886 2.36504 3.81395 5.86512 3.85467 931 s - - -
2.68626 4.42250 2.77482 2.73113 3.46342 2.40538 3.72520
3.29379 2.67691 2.69363 4.24715 2.90306 2.73747 3.18172 2.89805
2.37899 2.77545 2.98498 4.58502 3.61485
0.09446 2.43864 5.85950 1.65219 0.21273 0.48576 0.95510
518 2.96063 5.47009 1.39707 2.51366 4.81164 3.21933 3.49795
4.29742 2.12342 3.77384 4.51277 2.45487 3.59277 3.01653 3.15547
2.73154 2.94890 3.85905 5.90372 3.73562 946 d - - -
2.68618 4.42225 2.77519 2.73123 3.46354 2.40513 3.72494
3.29354 2.67741 2.69355 4.24690 2.90347 2.73739 3.18146 2.89801
2.37887 2.77519 2.98518 4.58477 3.61503
0.00877 5.13716 5.85950 0.61958 0.77255 0.48576 0.95510

519 2.94068 5.38030 2.99904 2.49923 4.69804 3.72500 3.91349
3.02031 1.49188 2.77864 4.42854 3.20425 4.11750 2.49516 2.30586
2.74775 2.85904 3.36988 5.82892 4.43703 947 k - - -
2.68618 4.42225 2.77519 2.73123 3.46354 2.40513 3.72494
3.29354 2.67741 2.69355 4.24690 2.90347 2.73739 3.18146 2.89801
2.37887 2.77519 2.98518 4.58477 3.61503
0.00877 5.13716 5.85950 0.61958 0.77255 0.48576 0.95510
520 3.41623 4.93832 5.22227 4.89944 4.44987 4.43160 5.54789
0.42186 4.75536 3.15116 4.32441 4.87050 5.03951 5.04812 4.88768
3.25050 3.23835 2.47145 6.22510 5.00991 948 I - - -
2.68618 4.42225 2.77519 2.73123 3.46354 2.40513 3.72494
3.29354 2.67741 2.69355 4.24690 2.90347 2.73739 3.18146 2.89801
2.37887 2.77519 2.98518 4.58477 3.61503
0.00877 5.13716 5.85950 0.61958 0.77255 0.48576 0.95510
521 2.98893 4.84139 3.83868 3.32039 4.16860 3.86313 4.33404
3.04893 3.27954 2.90520 4.11128 2.88487 4.35003 3.60820 3.65086
2.46654 0.87890 3.23475 5.57652 4.32597 949 t - - -
2.68618 4.42225 2.77519 2.73123 3.46354 2.40513 3.72494
3.29354 2.67741 2.69355 4.24690 2.90347 2.73739 3.18146 2.89801
2.37887 2.77519 2.98518 4.58477 3.61503
0.00877 5.13716 5.85950 0.61958 0.77255 0.48576 0.95510
522 3.10252 5.30894 3.46799 2.90946 4.56817 3.88908 3.53964
3.37977 2.55482 3.18656 4.38340 3.41307 4.27870 0.89623 3.08542
2.78206 3.32443 3.66455 5.76192 4.44587 950 q - - -
2.68618 4.42225 2.77519 2.73123 3.46354 2.40513 3.72494
3.29354 2.67741 2.69355 4.24690 2.90347 2.73739 3.18146 2.89801
2.37887 2.77519 2.98518 4.58477 3.61503
0.00877 5.13716 5.85950 0.61958 0.77255 0.48576 0.95510
523 3.94862 5.15107 6.02353 5.51606 2.88980 5.48941 6.01644
0.56874 5.42567 2.17823 3.87077 5.65026 5.65602 5.52020 5.49027
4.89965 4.19106 1.86069 6.20671 5.06740 951 I - - -
2.68618 4.42225 2.77519 2.73123 3.46354 2.40513 3.72494
3.29354 2.67741 2.69355 4.24690 2.90347 2.73739 3.18146 2.89801
2.37887 2.77519 2.98518 4.58477 3.61503
0.00877 5.13716 5.85950 0.61958 0.77255 0.48576 0.95510
524 2.62403 4.96947 4.57630 4.54766 5.63922 3.72095 5.56924
5.13081 4.73473 4.82712 5.61390 4.38979 0.34259 4.92810 4.89209
2.80768 3.61278 4.30359 6.96983 5.85475 952 P - - -
2.68618 4.42225 2.77519 2.73123 3.46354 2.40513 3.72494
3.29354 2.67741 2.69355 4.24690 2.90347 2.73739 3.18146 2.89801
2.37887 2.77519 2.98518 4.58477 3.61503
0.00877 5.13716 5.85950 0.61958 0.77255 0.48576 0.95510
525 0.49967 4.92026 5.00585 4.61837 4.37451 4.10061 5.28836
3.20300 4.47372 2.26557 4.28549 4.58146 4.77421 4.73083 4.64163
3.54611 3.66126 2.74528 6.08600 4.90297 953 A - - -
2.68618 4.42225 2.77519 2.73123 3.46354 2.40513 3.72494
3.29354 2.67741 2.69355 4.24690 2.90347 2.73739 3.18146 2.89801
2.37887 2.77519 2.98518 4.58477 3.61503
0.00877 5.13716 5.85950 0.61958 0.77255 0.48576 0.95510
526 3.57159 4.89058 5.55568 5.06383 4.29365 4.89836 5.60323
1.77034 4.94589 2.97060 4.13050 5.13081 5.28264 5.17789 5.07201
3.37434 3.26518 0.54539 6.10764 4.89567 954 V - - -
2.68618 4.42225 2.77519 2.73123 3.46354 2.40513 3.72494
3.29354 2.67741 2.69355 4.24690 2.90347 2.73739 3.18146 2.89801
2.37887 2.77519 2.98518 4.58477 3.61503

```
          0.00877  5.13716  5.85950  0.61958  0.77255  0.48576  0.95510
    527   4.04191  6.10274  4.06431  3.65188  5.73721  4.43556  4.50309
5.15114  0.33704  4.49659  5.40792  3.22510  4.90126  3.65164  2.90242
4.01473  4.23423  4.79396  6.39674  5.27421    955 K - - -
          2.68618  4.42225  2.77519  2.73123  3.46354  2.40513  3.72494
3.29354  2.67741  2.69355  4.24690  2.90347  2.73739  3.18146  2.89801
2.37887  2.77519  2.98518  4.58477  3.61503
          0.00877  5.13716  5.85950  0.61958  0.77255  0.48576  0.95510
    528   1.43384  5.09560  3.49859  2.69783  4.53276  2.20899  4.15096
3.95306  2.96825  3.56206  3.72790  3.44610  4.22949  3.31049  3.42044
1.75644  2.68354  3.58901  5.80250  4.48128    956 a - - -
          2.68618  4.42225  2.77519  2.73123  3.46354  2.40513  3.72494
3.29354  2.67741  2.69355  4.24690  2.90347  2.73739  3.18146  2.89801
2.37887  2.77519  2.98518  4.58477  3.61503
          0.00877  5.13716  5.85950  0.61958  0.77255  0.48576  0.95510
    529   2.95089  4.92835  3.02285  2.95777  2.81558  3.83967  2.89318
3.15483  2.93921  3.16145  4.01600  2.28823  4.22553  3.27373  2.82751
2.24935  3.18237  2.92993  3.50213  2.26739    957 s - - -
          2.68618  4.42225  2.77519  2.73123  3.46354  2.40513  3.72494
3.29354  2.67741  2.69355  4.24690  2.90347  2.73739  3.18146  2.89801
2.37887  2.77519  2.98518  4.58477  3.61503
          0.00877  5.13716  5.85950  0.61958  0.77255  0.48576  0.95510
    530   2.61507  5.42941  2.95428  2.24629  3.99681  3.06245  3.16604
4.24898  2.16237  3.39095  3.91738  2.92367  2.99592  2.82069  2.89604
1.99124  2.93386  3.44943  5.86615  3.45845    958 s - - -
          2.68618  4.42225  2.77519  2.73123  3.46354  2.40513  3.72494
3.29354  2.67741  2.69355  4.24690  2.90347  2.73739  3.18146  2.89801
2.37887  2.77519  2.98518  4.58477  3.61503
          0.00877  5.13716  5.85950  0.61958  0.77255  0.48576  0.95510
    531   2.96879  4.53293  3.13886  3.58605  3.64719  3.55674  4.30389
2.12572  3.50496  1.23497  2.96787  3.04517  4.39536  3.37298  3.76972
3.29029  2.93409  2.60599  5.13485  3.92846    959 l - - -
          2.68618  4.42225  2.77519  2.73123  3.46354  2.40513  3.72494
3.29354  2.67741  2.69355  4.24690  2.90347  2.73739  3.18146  2.89801
2.37887  2.77519  2.98518  4.58477  3.61503
          0.08299  5.13716  2.60684  0.61958  0.77255  0.48576  0.95510
    532   2.90378  5.33215  2.97571  2.32964  3.86355  2.54269  3.88173
4.10763  2.45902  2.95929  4.38161  2.75891  3.22557  2.65865  3.11734
2.18901  2.25928  3.70495  4.26871  3.41265    960 s - - -
          2.68633  4.42177  2.77525  2.73152  3.46403  2.40483  3.72445
3.29372  2.67790  2.69385  4.24739  2.90251  2.73739  3.18167  2.89786
2.37893  2.77510  2.98522  4.58369  3.61462
          0.51146  0.99112  3.53287  1.08496  0.41236  0.37540  1.16159
    533   2.93193  5.09330  3.11021  2.79927  3.33648  2.31288  3.54874
3.73107  2.79087  2.37701  4.16723  2.50955  4.16165  2.93552  2.76658
2.32896  2.74291  2.57568  5.60374  3.74088    966 g - - -
          2.68618  4.42225  2.77519  2.73123  3.46354  2.40513  3.72494
3.29354  2.67741  2.69355  4.24690  2.90347  2.73739  3.18146  2.89801
2.37887  2.77519  2.98518  4.58477  3.61503
          0.05499  5.11256  3.04731  0.61958  0.77255  0.56141  0.84491
    534   2.58898  5.37602  2.91710  2.32679  3.89202  2.38238  3.87314
4.17976  2.44192  3.67442  4.42094  2.44046  3.01158  2.64388  3.10437
2.28283  2.65131  3.36078  4.37571  3.76131    967 s - - -
```

```
          2.68618  4.42225  2.77519  2.73123  3.46354  2.40513  3.72494
3.29354  2.67741  2.69355  4.24690  2.90347  2.73739  3.18146  2.89801
2.37887  2.77519  2.98518  4.58477  3.61503
          0.06377  5.06697  2.89178  0.61958  0.77255  0.37920  1.15330
    535   1.98786  4.61413  3.88840  3.31904  3.31736  2.05561  4.19556
2.42064  3.26590  2.29189  3.71975  3.70354  4.31299  3.19318  3.20303
2.30016  2.92551  2.88369  5.20185  3.51516    968 a - - -
          2.68618  4.42225  2.77519  2.73123  3.46354  2.40513  3.72494
3.29354  2.67741  2.69355  4.24690  2.90347  2.73739  3.18146  2.89801
2.37887  2.77519  2.98518  4.58477  3.61503
          0.00922  5.08706  5.80940  0.61958  0.77255  0.51220  0.91423
    536   2.57029  5.41151  3.18270  2.47919  3.92117  3.37889  3.88988
4.22584  2.17511  3.71252  4.45478  2.93770  4.09522  2.22857  2.17410
2.23129  2.20595  3.79693  5.84994  3.85127    969 r - - -
          2.68618  4.42225  2.77519  2.73123  3.46354  2.40513  3.72494
3.29354  2.67741  2.69355  4.24690  2.90347  2.73739  3.18146  2.89801
2.37887  2.77519  2.98518  4.58477  3.61503
          0.00899  5.11230  5.83465  0.61958  0.77255  0.56217  0.84391
    537   2.75740  4.77989  5.44871  4.89831  4.02361  4.84028  5.29834
1.80741  4.75578  2.54469  3.91165  4.97723  5.15080  4.94488  4.84304
4.19119  3.73187  0.65426  5.76612  3.70169    970 V - - -
          2.68618  4.42225  2.77519  2.73123  3.46354  2.40513  3.72494
3.29354  2.67741  2.69355  4.24690  2.90347  2.73739  3.18146  2.89801
2.37887  2.77519  2.98518  4.58477  3.61503
          0.00899  5.11230  5.83465  0.61958  0.77255  0.56217  0.84391
    538   2.74151  4.46856  4.33171  3.05061  3.57679  4.05086  4.34534
1.33001  3.28391  2.66382  3.20096  3.99262  4.42133  3.87527  2.81570
3.33134  3.19653  1.79803  5.07883  3.40204    971 i - - -
          2.68618  4.42225  2.77519  2.73123  3.46354  2.40513  3.72494
3.29354  2.67741  2.69355  4.24690  2.90347  2.73739  3.18146  2.89801
2.37887  2.77519  2.98518  4.58477  3.61503
          0.00899  5.11230  5.83465  0.61958  0.77255  0.56217  0.84391
    539   2.60302  5.42068  2.89810  2.50443  4.75992  3.70022  3.46149
3.72613  1.51907  3.32628  4.46292  3.16998  3.58244  2.54158  2.71076
2.08632  3.15546  3.40498  5.85677  4.44960    972 k - - -
          2.68618  4.42225  2.77519  2.73123  3.46354  2.40513  3.72494
3.29354  2.67741  2.69355  4.24690  2.90347  2.73739  3.18146  2.89801
2.37887  2.77519  2.98518  4.58477  3.61503
          0.00899  5.11230  5.83465  0.61958  0.77255  0.44079  1.03150
    540   3.28458  5.43072  3.34534  3.06713  4.86635  0.67855  3.42032
4.32186  2.82853  3.90667  4.74799  3.08077  4.44764  3.53217  3.48848
3.32085  3.58128  3.34322  6.09574  4.76778    973 G - - -
          2.68618  4.42225  2.77519  2.73123  3.46354  2.40513  3.72494
3.29354  2.67741  2.69355  4.24690  2.90347  2.73739  3.18146  2.89801
2.37887  2.77519  2.98518  4.58477  3.61503
          0.00877  5.13716  5.85950  0.61958  0.77255  0.48576  0.95510
    541   2.96444  4.81177  3.67142  3.11495  3.98163  3.46911  4.14065
3.00113  3.07805  2.81040  3.92079  3.56247  1.13221  3.03950  3.47006
2.88514  2.73126  3.10781  5.38649  3.53929    974 p - - -
          2.68618  4.42225  2.77519  2.73123  3.46354  2.40513  3.72494
3.29354  2.67741  2.69355  4.24690  2.90347  2.73739  3.18146  2.89801
2.37887  2.77519  2.98518  4.58477  3.61503
          0.03918  5.13716  3.42494  0.61958  0.77255  0.48576  0.95510
```

```
    542   3.17157  5.54379  2.57528  2.76240  4.87424  0.88437  4.14603
4.34307  2.97757  3.88237  4.67905  3.00783  4.28820  3.28459  3.04682
2.83974  3.43012  3.95150  6.06350  3.72302    975 g - - -
         2.68618  4.42225  2.77519  2.73123  3.46354  2.40513  3.72494
3.29354  2.67741  2.69355  4.24690  2.90347  2.73739  3.18146  2.89801
2.37887  2.77519  2.98518  4.58477  3.61503
         0.03637  5.10701  3.51788  0.61958  0.77255  0.43240  1.04682
    543   3.44604  5.18866  4.04001  3.63738  1.24262  4.25714  1.42534
3.82186  3.63830  3.41238  4.38565  3.30612  4.69994  3.92740  3.96813
2.30741  3.71020  3.58914  4.88375  3.26303    976 f - - -
         2.68618  4.42225  2.77519  2.73123  3.46354  2.40513  3.72494
3.29354  2.67741  2.69355  4.24690  2.90347  2.73739  3.18146  2.89801
2.37887  2.77519  2.98518  4.58477  3.61503
         0.00900  5.11088  5.83323  0.61958  0.77255  0.56629  0.83846
    544   3.09627  4.89265  4.29880  3.95952  4.50037  3.89624  4.87528
2.71373  3.87757  3.53164  4.49272  3.35952  4.55879  4.22980  4.13655
3.33108  0.53009  3.30060  5.98222  4.74639    977 T - - -
         2.68618  4.42225  2.77519  2.73123  3.46354  2.40513  3.72494
3.29354  2.67741  2.69355  4.24690  2.90347  2.73739  3.18146  2.89801
2.37887  2.77519  2.98518  4.58477  3.61503
         0.00900  5.11088  5.83323  0.61958  0.77255  0.56629  0.83846
    545   4.71247  6.21848  5.56440  5.62428  6.49824  0.06004  6.49884
6.52155  5.91708  5.92455  7.00567  5.73534  5.60294  6.15740  5.89584
4.93475  5.25563  5.85278  7.15854  6.67975    978 G - - -
         2.68618  4.42225  2.77519  2.73123  3.46354  2.40513  3.72494
3.29354  2.67741  2.69355  4.24690  2.90347  2.73739  3.18146  2.89801
2.37887  2.77519  2.98518  4.58477  3.61503
         0.00900  5.11088  5.83323  0.61958  0.77255  0.43851  1.03563
    546   4.09700  6.00796  3.94826  3.10567  6.14305  0.19130  5.54141
5.84722  4.66464  5.36463  6.31643  4.44404  5.13174  4.84789  5.03187
4.17811  4.57721  5.22956  7.09878  6.08834    979 G - - -
         2.68618  4.42225  2.77519  2.73123  3.46354  2.40513  3.72494
3.29354  2.67741  2.69355  4.24690  2.90347  2.73739  3.18146  2.89801
2.37887  2.77519  2.98518  4.58477  3.61503
         0.00877  5.13716  5.85950  0.61958  0.77255  0.48576  0.95510
    547   2.94903  5.81658  1.01119  2.72574  5.14745  3.29884  4.20754
4.64701  3.07802  4.13600  4.91927  1.73335  3.45514  3.33823  3.61758
3.22723  3.55571  4.21286  6.28607  3.76028    980 d - - -
         2.68618  4.42225  2.77519  2.73123  3.46354  2.40513  3.72494
3.29354  2.67741  2.69355  4.24690  2.90347  2.73739  3.18146  2.89801
2.37887  2.77519  2.98518  4.58477  3.61503
         0.00877  5.13716  5.85950  0.61958  0.77255  0.48576  0.95510
    548   3.66936  4.97556  5.62523  5.02454  3.12212  4.91697  5.21641
2.40616  4.83946  0.72289  3.45948  5.07552  5.15408  4.85767  4.81894
4.25398  3.88981  1.66049  3.87060  4.39587    981 l - - -
         2.68618  4.42225  2.77519  2.73123  3.46354  2.40513  3.72494
3.29354  2.67741  2.69355  4.24690  2.90347  2.73739  3.18146  2.89801
2.37887  2.77519  2.98518  4.58477  3.61503
         0.00877  5.13716  5.85950  0.61958  0.77255  0.48576  0.95510
    549   3.30188  4.63089  5.27427  4.68885  3.76551  4.56346  4.94763
1.45127  4.50811  2.08456  3.20637  4.71617  4.89473  4.65548  4.54593
3.89284  2.86640  1.07653  5.41566  3.53311    982 v - - -
         2.68618  4.42225  2.77519  2.73123  3.46354  2.40513  3.72494
3.29354  2.67741  2.69355  4.24690  2.90347  2.73739  3.18146  2.89801
2.37887  2.77519  2.98518  4.58477  3.61503
```

```
         0.00877  5.13716  5.85950  0.61958  0.77255  0.48576  0.95510
    550  2.46641  5.36487  2.99322  2.51645  3.53296  3.35116  3.91426
4.14045  1.85142  2.73859  4.41431  2.99023  4.11609  2.66056  2.23749
2.75429  2.93443  3.08146  5.81775  3.30363    983 k - - -
         2.68618  4.42225  2.77519  2.73123  3.46354  2.40513  3.72494
3.29354  2.67741  2.69355  4.24690  2.90347  2.73739  3.18146  2.89801
2.37887  2.77519  2.98518  4.58477  3.61503
         0.00877  5.13716  5.85950  0.61958  0.77255  0.48576  0.95510
    551  2.57257  4.43531  4.51929  3.92881  2.69319  4.09206  4.40206
2.87726  3.79407  1.18734  2.54599  4.10464  3.71120  4.00263  3.21640
2.43980  3.21081  2.68654  5.04879  3.86046    984 l - - -
         2.68618  4.42225  2.77519  2.73123  3.46354  2.40513  3.72494
3.29354  2.67741  2.69355  4.24690  2.90347  2.73739  3.18146  2.89801
2.37887  2.77519  2.98518  4.58477  3.61503
         0.11363  5.13716  2.28733  0.61958  0.77255  0.48576  0.95510
    552  2.71519  5.34448  2.90813  2.43207  4.66655  3.06283  3.86265
3.04509  2.04738  3.04125  4.39125  2.73655  3.40663  2.96687  2.68448
2.06514  2.70497  3.38338  5.79118  3.71819    985 k - - -
         2.68618  4.42225  2.77519  2.73123  3.46354  2.40513  3.72494
3.29354  2.67741  2.69355  4.24690  2.90347  2.73739  3.18146  2.89801
2.37887  2.77519  2.98518  4.58477  3.61503
         0.06393  5.03326  2.89298  0.61958  0.77255  0.34486  1.23211
    553  2.72692  5.42190  2.40957  2.24676  4.76557  3.04755  3.87561
4.25064  2.48588  3.72659  4.46299  2.34898  2.87023  2.77775  2.90988
2.13229  2.59242  3.03857  5.85540  4.44515    986 s - - -
         2.68618  4.42225  2.77519  2.73123  3.46354  2.40513  3.72494
3.29354  2.67741  2.69355  4.24690  2.90347  2.73739  3.18146  2.89801
2.37887  2.77519  2.98518  4.58477  3.61503
         0.03323  5.08892  3.63001  0.61958  0.77255  0.62753  0.76338
    554  2.90764  5.18374  3.26460  2.70732  3.75587  3.33004  3.39599
3.43543  2.54447  3.12400  4.24787  2.19735  3.67737  2.47462  3.17586
2.03035  2.30934  2.94983  5.67093  3.71195    987 s - - -
         2.68618  4.42225  2.77519  2.73123  3.46354  2.40513  3.72494
3.29354  2.67741  2.69355  4.24690  2.90347  2.73739  3.18146  2.89801
2.37887  2.77519  2.98518  4.58477  3.61503
         0.00942  5.06512  5.78746  0.61958  0.77255  0.68854  0.69778
    555  2.90362  4.14001  2.48677  2.48886  4.70422  2.69715  3.87236
3.62331  2.30871  3.30488  4.42017  2.43661  3.48164  2.97408  2.90386
2.00677  2.49306  3.35988  5.81769  4.41665    988 s - - -
         2.68618  4.42225  2.77519  2.73123  3.46354  2.40513  3.72494
3.29354  2.67741  2.69355  4.24690  2.90347  2.73739  3.18146  2.89801
2.37887  2.77519  2.98518  4.58477  3.61503
         0.08154  5.06512  2.63117  0.61958  0.77255  0.57715  0.82439
    556  2.31651  5.37454  2.54800  2.19790  4.71131  2.99257  3.44496
3.57401  2.58650  3.00381  4.41738  2.75584  3.15673  2.74101  2.48492
2.66865  2.68664  3.76071  5.81222  3.38856    989 e - - -
         2.68618  4.42225  2.77519  2.73123  3.46354  2.40513  3.72494
3.29354  2.67741  2.69355  4.24690  2.90347  2.73739  3.18146  2.89801
2.37887  2.77519  2.98518  4.58477  3.61503
         0.15243  5.01924  2.00420  0.61958  0.77255  0.60386  0.79119
    557  2.18433  5.11378  2.73450  2.65594  4.37019  2.97867  3.86750
3.12013  2.65155  2.47758  3.57073  2.94620  4.05834  2.46697  2.68006
2.51744  2.64104  3.45583  5.60352  3.70238    990 a - - -
```

```
          2.68618  4.42225  2.77519  2.73123  3.46354  2.40513  3.72494
3.29354  2.67741  2.69355  4.24690  2.90347  2.73739  3.18146  2.89801
2.37887  2.77519  2.98518  4.58477  3.61503
          0.22481  4.92824  1.63943  0.61958  0.77255  0.74067  0.64778
    558   2.45071  5.06834  2.88552  2.59465  4.32789  2.97250  3.27496
2.68378  2.37503  3.08797  4.13267  2.29954  4.00069  2.94381  3.06301
2.32151  2.82099  3.07958  5.55594  3.63809    991 n - - -
          2.68655  4.42262  2.77482  2.73127  3.46391  2.40514  3.72531
3.29269  2.67778  2.69360  4.24727  2.90301  2.73735  3.18068  2.89801
2.37913  2.77540  2.98489  4.58514  3.61501
          1.01021  0.94500  1.39765  1.03320  0.43985  0.45694  1.00298
    559   2.41602  4.28721  4.37299  3.78407  3.04612  2.25153  4.26103
2.34508  3.65230  1.58057  3.40258  3.37870  4.31649  3.86030  3.80806
3.23874  3.06445  2.07454  4.90832  3.71940    998 l - - -
          2.68618  4.42225  2.77519  2.73123  3.46354  2.40513  3.72494
3.29354  2.67741  2.69355  4.24690  2.90347  2.73739  3.18146  2.89801
2.37887  2.77519  2.98518  4.58477  3.61503
          0.01210  4.81671  5.53905  0.61958  0.77255  0.84107  0.56431
    560   2.64725  5.26686  3.11765  2.42810  3.83830  2.86083  3.81300
4.04406  1.77460  3.55835  4.31610  3.10362  4.01526  2.68301  2.50923
2.10427  2.83551  3.25974  5.71891  3.61702    999 k - - -
          2.68636  4.42243  2.77521  2.73142  3.46372  2.40502  3.72513
3.29372  2.67719  2.69356  4.24461  2.90340  2.73758  3.18137  2.89798
2.37905  2.77499  2.98537  4.58495  3.61471
          0.22289  1.78246  3.45558  1.04368  0.43410  0.40179  1.10601
    561   2.72160  4.34009  3.93174  4.21794  2.64338  4.11425  4.44298
1.32616  4.02314  1.82651  2.98042  4.24637  4.47706  4.17035  4.06325
3.42210  2.95804  2.57057  4.96123  2.71632   1005 i - - -
          2.68623  4.42230  2.77524  2.73128  3.46358  2.40517  3.72499
3.29359  2.67727  2.69359  4.24694  2.90328  2.73744  3.18151  2.89764
2.37891  2.77524  2.98523  4.58482  3.61508
          0.70290  2.16080  0.94258  0.19130  1.74802  0.58460  0.81492
    562   2.74091  4.84157  3.20556  2.64739  3.40138  3.59285  3.80182
3.45669  2.14147  2.27377  3.92123  2.89896  3.98158  2.52949  3.06539
2.61476  2.09154  3.16000  5.36158  4.05050   1007 t - - -
          2.68620  4.41504  2.77538  2.73142  3.46298  2.40531  3.72513
3.29301  2.67743  2.69373  4.24708  2.90365  2.73758  3.18165  2.89819
2.37905  2.77498  2.98537  4.58495  3.61522
          1.25982  0.39147  3.21301  0.06384  2.78316  0.39455  1.12081
    563   2.53745  5.21601  3.19962  2.64254  3.89596  3.67852  3.42091
2.95524  2.06243  3.16211  4.27420  2.92521  2.25374  2.36165  2.53722
2.87431  3.10700  3.22054  5.68877  4.32075   1009 k - - -
          2.68618  4.42225  2.77519  2.73123  3.46354  2.40513  3.72494
3.29354  2.67741  2.69355  4.24690  2.90347  2.73739  3.18146  2.89801
2.37887  2.77519  2.98518  4.58477  3.61503
          0.15866  4.99096  1.96670  0.61958  0.77255  0.61101  0.78264
    564   2.31805  4.29068  4.52527  3.37795  2.52983  3.46692  4.31126
2.19837  3.34741  1.95941  3.40754  4.05243  4.36184  3.96073  3.88783
3.29191  2.45161  1.92746  4.90718  2.94804   1010 v - - -
          2.68618  4.42225  2.77519  2.73123  3.46354  2.40513  3.72494
3.29354  2.67741  2.69355  4.24690  2.90347  2.73739  3.18146  2.89801
2.37887  2.77519  2.98518  4.58477  3.61503
          0.22062  4.91074  1.65751  0.61958  0.77255  0.82557  0.57622
```

```
    565   2.61021  4.68963  3.42564  2.86345  3.85847  3.25404  3.44584
2.46599  2.38885  2.66331  3.30378  3.33086  3.14052  3.16673  3.25129
2.37570  2.16604  2.78949  5.24511  3.97495   1011 t - - -
          2.68633  4.42240  2.77534  2.73138  3.46336  2.40527  3.72509
3.29277  2.67755  2.69338  4.24704  2.90361  2.73736  3.18161  2.89794
2.37901  2.77466  2.98501  4.58491  3.61518
          0.70547  0.76656  3.18173  0.07862  2.58216  0.37829  1.15528
    566   2.55014  5.24882  2.73829  2.64705  3.10426  3.68882  2.95531
3.49492  2.46795  3.52907  4.30519  2.42556  3.25905  2.79060  3.12645
2.28449  2.46696  2.88189  5.71779  3.76451   1013 s - - -
          2.68618  4.42225  2.77519  2.73123  3.46354  2.40513  3.72494
3.29354  2.67741  2.69355  4.24690  2.90347  2.73739  3.18146  2.89801
2.37887  2.77519  2.98518  4.58477  3.61503
          0.03537  5.02736  3.56844  0.61958  0.77255  0.63061  0.75986
    567   2.68143  5.13365  3.27381  2.71621  2.61113  3.06870  3.92032
3.25374  2.54789  3.12682  4.20114  2.52044  3.60080  2.50465  2.92185
2.17225  2.40309  3.47138  5.62877  3.73459   1014 s - - -
          2.68618  4.42225  2.77519  2.73123  3.46354  2.40513  3.72494
3.29354  2.67741  2.69355  4.24690  2.90347  2.73739  3.18146  2.89801
2.37887  2.77519  2.98518  4.58477  3.61503
          0.09302  5.03545  2.49717  0.61958  0.77255  0.34687  1.22724
    568   2.45606  5.30389  2.98609  2.31077  3.42342  3.39167  3.88835
4.06181  2.64276  3.18222  3.70198  2.59028  3.20395  2.77304  2.89568
1.80943  2.90313  2.98159  5.76356  4.38050   1015 s - - -
          2.68618  4.42225  2.77519  2.73123  3.46354  2.40513  3.72494
3.29354  2.67741  2.69355  4.24690  2.90347  2.73739  3.18146  2.89801
2.37887  2.77519  2.98518  4.58477  3.61503
          0.00944  5.06297  5.78531  0.61958  0.77255  0.69381  0.69249
    569   2.17211  5.31701  2.68221  2.50458  3.84875  3.31775  3.88559
4.08292  2.63763  2.99445  4.36821  2.29521  2.84810  2.99609  3.12303
2.01662  2.74849  3.68672  5.77375  3.73648   1016 s - - -
          2.68618  4.42225  2.77519  2.73123  3.46354  2.40513  3.72494
3.29354  2.67741  2.69355  4.24690  2.90347  2.73739  3.18146  2.89801
2.37887  2.77519  2.98518  4.58477  3.61503
          0.00944  5.06297  5.78531  0.61958  0.77255  0.69381  0.69249
    570   2.73649  5.14021  3.03576  2.73167  3.76655  3.00797  3.93414
3.44078  2.72618  2.22193  4.20824  2.41419  3.58328  2.88107  2.94419
1.90386  2.65896  3.19035  5.63654  4.29395   1017 s - - -
          2.68618  4.42225  2.77519  2.73123  3.46354  2.40513  3.72494
3.29354  2.67741  2.69355  4.24690  2.90347  2.73739  3.18146  2.89801
2.37887  2.77519  2.98518  4.58477  3.61503
          0.00944  5.06297  5.78531  0.61958  0.77255  0.37470  1.16314
    571   2.45242  5.40117  3.20238  2.51748  4.72776  3.38324  3.90553
3.63540  2.36978  3.10666  4.44674  2.60655  2.82792  2.63510  2.69275
1.86924  2.60495  3.38972  5.84508  4.44558   1018 s - - -
          2.68618  4.42225  2.77519  2.73123  3.46354  2.40513  3.72494
3.29354  2.67741  2.69355  4.24690  2.90347  2.73739  3.18146  2.89801
2.37887  2.77519  2.98518  4.58477  3.61503
          0.00877  5.13716  5.85950  0.61958  0.77255  0.48576  0.95510
    572   2.74760  5.33541  3.23504  2.67840  4.63634  3.35641  3.92253
3.50045  2.19659  3.31744  4.38807  3.21786  4.12289  1.68375  2.46958
2.29259  2.53609  3.33006  5.79529  3.76614   1019 q - - -
          2.68618  4.42225  2.77519  2.73123  3.46354  2.40513  3.72494
3.29354  2.67741  2.69355  4.24690  2.90347  2.73739  3.18146  2.89801
2.37887  2.77519  2.98518  4.58477  3.61503
```

0.00877 5.13716 5.85950 0.61958 0.77255 0.48576 0.95510
573 2.93619 5.44610 2.75195 2.48177 4.79109 2.85234 3.89651
4.27677 1.84379 3.30153 4.48688 2.59668 4.10310 2.51433 2.26538
2.08555 3.16729 3.83672 5.87863 3.80588 1020 k - - -
2.68618 4.42225 2.77519 2.73123 3.46354 2.40513 3.72494
3.29354 2.67741 2.69355 4.24690 2.90347 2.73739 3.18146 2.89801
2.37887 2.77519 2.98518 4.58477 3.61503
0.00877 5.13716 5.85950 0.61958 0.77255 0.48576 0.95510
574 5.01548 5.99233 5.91186 5.80458 1.68117 5.55561 3.20720
4.64999 5.58311 3.85467 5.18422 5.11222 5.81565 5.27409 5.40835
4.95125 5.21127 3.37480 4.32668 0.45259 1021 Y - - -
2.68618 4.42225 2.77519 2.73123 3.46354 2.40513 3.72494
3.29354 2.67741 2.69355 4.24690 2.90347 2.73739 3.18146 2.89801
2.37887 2.77519 2.98518 4.58477 3.61503
0.00877 5.13716 5.85950 0.61958 0.77255 0.48576 0.95510
575 2.57479 5.41570 2.88130 2.64426 3.55848 3.10036 3.39467
3.63663 1.94319 3.71486 4.46003 2.95379 4.11001 2.79792 1.52933
2.90732 3.16960 3.79939 5.85569 4.45402 1022 r - - -
2.68618 4.42225 2.77519 2.73123 3.46354 2.40513 3.72494
3.29354 2.67741 2.69355 4.24690 2.90347 2.73739 3.18146 2.89801
2.37887 2.77519 2.98518 4.58477 3.61503
0.00877 5.13716 5.85950 0.61958 0.77255 0.48576 0.95510
576 3.94237 5.12403 6.07160 5.59764 4.33786 5.58504 6.28196
0.94767 5.54352 2.11111 3.36623 5.75116 5.75695 5.72468 5.66549
5.02119 4.19458 1.01694 6.49667 5.33633 1023 i - - -
2.68618 4.42225 2.77519 2.73123 3.46354 2.40513 3.72494
3.29354 2.67741 2.69355 4.24690 2.90347 2.73739 3.18146 2.89801
2.37887 2.77519 2.98518 4.58477 3.61503
0.00877 5.13716 5.85950 0.61958 0.77255 0.48576 0.95510
577 3.87391 5.78510 4.72806 4.08642 5.63136 4.39546 4.65917
5.12200 2.83047 4.51031 5.43093 4.30371 4.93099 3.82821 0.31284
3.22261 4.17481 4.70109 6.39716 5.30716 1024 R - - -
2.68618 4.42225 2.77519 2.73123 3.46354 2.40513 3.72494
3.29354 2.67741 2.69355 4.24690 2.90347 2.73739 3.18146 2.89801
2.37887 2.77519 2.98518 4.58477 3.61503
0.00877 5.13716 5.85950 0.61958 0.77255 0.48576 0.95510
578 3.86834 5.12060 5.88688 5.31467 2.90014 5.23702 5.61830
0.96768 5.16387 1.50303 2.52911 5.40721 5.41886 5.17041 5.15273
4.60283 4.09347 1.86361 5.82912 4.77082 1025 i - - -
2.68618 4.42225 2.77519 2.73123 3.46354 2.40513 3.72494
3.29354 2.67741 2.69355 4.24690 2.90347 2.73739 3.18146 2.89801
2.37887 2.77519 2.98518 4.58477 3.61503
0.00877 5.13716 5.85950 0.61958 0.77255 0.48576 0.95510
579 3.30432 3.71200 4.11389 3.52730 3.09919 4.20740 3.50208
3.57735 3.22719 3.21558 4.14893 3.88959 4.57687 3.68485 0.77554
3.47524 3.52712 3.35473 5.04018 2.78957 1026 r - - -
2.68618 4.42225 2.77519 2.73123 3.46354 2.40513 3.72494
3.29354 2.67741 2.69355 4.24690 2.90347 2.73739 3.18146 2.89801
2.37887 2.77519 2.98518 4.58477 3.61503
0.00877 5.13716 5.85950 0.61958 0.77255 0.48576 0.95510
580 5.29261 6.16410 5.98897 5.99571 2.39372 5.65250 4.28416
4.83697 5.77019 3.92522 5.34721 5.19990 5.92436 5.39447 5.54300
5.11170 5.49554 4.81622 4.35288 0.22816 1027 Y - - -

```
         2.68618  4.42225  2.77519  2.73123  3.46354  2.40513  3.72494
3.29354  2.67741  2.69355  4.24690  2.90347  2.73739  3.18146  2.89801
2.37887  2.77519  2.98518  4.58477  3.61503
         0.00877  5.13716  5.85950  0.61958  0.77255  0.48576  0.95510
    581  0.41670  4.83845  4.98332  4.73818  5.17321  3.08408  5.45312
4.50509  4.68346  4.29375  5.12621  4.43277  4.52807  4.86225  4.82015
2.82118  3.50284  2.43164  6.58244  5.46450   1028 A - - -
         2.68618  4.42225  2.77519  2.73123  3.46354  2.40513  3.72494
3.29354  2.67741  2.69355  4.24690  2.90347  2.73739  3.18146  2.89801
2.37887  2.77519  2.98518  4.58477  3.61503
         0.00877  5.13716  5.85950  0.61958  0.77255  0.48576  0.95510
    582  1.44267  2.94250  4.44105  3.99263  4.98511  3.12182  4.96366
4.41289  3.95534  4.08205  4.89335  3.32464  4.43738  4.23200  4.27895
0.96843  2.49065  3.87349  6.33316  5.13265   1029 s - - -
         2.68618  4.42225  2.77519  2.73123  3.46354  2.40513  3.72494
3.29354  2.67741  2.69355  4.24690  2.90347  2.73739  3.18146  2.89801
2.37887  2.77519  2.98518  4.58477  3.61503
         0.00877  5.13716  5.85950  0.61958  0.77255  0.48576  0.95510
    583  2.45983  5.34839  2.64071  2.67039  3.77908  3.72672  3.91840
4.11348  2.51744  3.63680  3.36356  1.69429  3.45404  2.65880  3.15580
2.59762  2.50489  3.37426  5.80529  3.76651   1030 n - - -
         2.68618  4.42225  2.77519  2.73123  3.46354  2.40513  3.72494
3.29354  2.67741  2.69355  4.24690  2.90347  2.73739  3.18146  2.89801
2.37887  2.77519  2.98518  4.58477  3.61503
         0.00877  5.13716  5.85950  0.61958  0.77255  0.48576  0.95510
    584  2.74824  5.40684  2.97762  2.64382  3.92172  2.55235  2.87483
4.21046  2.39037  3.70521  4.45178  2.30096  4.10845  2.79890  2.90150
2.18740  2.29344  3.17625  5.84933  3.79476   1031 s - - -
         2.68618  4.42225  2.77519  2.73123  3.46354  2.40513  3.72494
3.29354  2.67741  2.69355  4.24690  2.90347  2.73739  3.18146  2.89801
2.37887  2.77519  2.98518  4.58477  3.61503
         0.00877  5.13716  5.85950  0.61958  0.77255  0.48576  0.95510
    585  2.35332  5.42866  2.50447  2.35543  3.93504  2.80017  3.90001
4.24714  2.63786  3.34051  4.47142  2.12794  2.98348  2.99881  2.90520
2.06414  2.93580  3.81536  5.86591  4.45940   1032 s - - -
         2.68619  4.42226  2.77502  2.73124  3.46355  2.40514  3.72495
3.29355  2.67742  2.69356  4.24691  2.90348  2.73741  3.18147  2.89802
2.37888  2.77521  2.98519  4.58478  3.61504
         0.03381  3.49354  5.85950  0.39938  1.11089  0.48576  0.95510
    586  2.29496  4.43573  4.48893  3.89946  2.58009  1.90883  4.39060
2.24255  3.77120  2.36670  3.55134  2.87402  4.45267  3.98239  3.93416
3.07743  2.93271  2.11726  5.04874  3.34190   1034 g - - -
         2.68618  4.42225  2.77519  2.73123  3.46354  2.40513  3.72494
3.29354  2.67741  2.69355  4.24690  2.90347  2.73739  3.18146  2.89801
2.37887  2.77519  2.98518  4.58477  3.61503
         0.00877  5.13716  5.85950  0.61958  0.77255  0.48576  0.95510
    587  2.46293  5.42780  2.94683  2.52320  4.76543  3.28555  3.09290
4.24620  2.48621  3.72991  3.77764  2.47095  3.30294  2.65715  2.33813
2.56498  2.47573  3.02413  5.86495  3.17980   1035 r - - -
         2.68618  4.42225  2.77519  2.73123  3.46354  2.40513  3.72494
3.29354  2.67741  2.69355  4.24690  2.90347  2.73739  3.18146  2.89801
2.37887  2.77519  2.98518  4.58477  3.61503
         0.00877  5.13716  5.85950  0.61958  0.77255  0.48576  0.95510
```

588 2.59131 3.65731 4.88827 4.27658 2.74814 3.52541 3.65155
1.93619 4.07314 1.45128 3.13322 4.28604 4.50538 4.21437 4.10009
2.85077 2.75353 2.18486 4.98164 3.80445 1036 l - - -
2.68618 4.42225 2.77519 2.73123 3.46354 2.40513 3.72494
3.29354 2.67741 2.69355 4.24690 2.90347 2.73739 3.18146 2.89801
2.37887 2.77519 2.98518 4.58477 3.61503
0.02924 5.13716 3.77478 0.61958 0.77255 0.48576 0.95510
589 2.92648 5.40178 2.73701 2.48920 4.73225 3.33213 3.89436
3.24108 2.63487 3.33167 4.44631 2.61993 4.09887 2.40418 1.94875
2.32196 2.49518 3.78456 5.84327 3.15568 1037 r - - -
2.68618 4.42225 2.77519 2.73123 3.46354 2.40513 3.72494
3.29354 2.67741 2.69355 4.24690 2.90347 2.73739 3.18146 2.89801
2.37887 2.77519 2.98518 4.58477 3.61503
0.13875 5.11686 2.09106 0.61958 0.77255 0.54872 0.86202
590 2.68572 4.29494 4.77273 4.16416 2.73039 4.06476 4.39296
1.97553 3.96997 1.72976 3.41189 3.53531 4.42902 4.11946 3.36120
3.37198 3.13762 1.69233 4.91551 2.65289 1038 v - - -
2.68618 4.42225 2.77519 2.73123 3.46354 2.40513 3.72494
3.29354 2.67741 2.69355 4.24690 2.90347 2.73739 3.18146 2.89801
2.37887 2.77519 2.98518 4.58477 3.61503
0.03938 4.98829 3.44830 0.61958 0.77255 0.47337 0.97525
591 2.72734 4.95338 3.42371 2.25730 2.65635 3.06315 3.41802
3.16388 2.84951 2.70000 4.03643 2.86028 3.64967 3.18923 3.03060
2.15678 2.87376 2.78616 5.48580 3.10392 1039 s - - -
2.68618 4.42225 2.77519 2.73123 3.46354 2.40513 3.72494
3.29354 2.67741 2.69355 4.24690 2.90347 2.73739 3.18146 2.89801
2.37887 2.77519 2.98518 4.58477 3.61503
0.04150 5.05992 3.37235 0.61958 0.77255 0.70121 0.68515
592 2.90994 4.54624 3.93982 3.36822 3.22040 2.58025 4.19454
2.64467 2.73470 2.18456 3.65376 3.72907 4.30345 2.94604 2.97179
2.72344 2.37185 2.34674 4.04125 2.77472 1040 l - - -
2.68618 4.42225 2.77519 2.73123 3.46354 2.40513 3.72494
3.29354 2.67741 2.69355 4.24690 2.90347 2.73739 3.18146 2.89801
2.37887 2.77519 2.98518 4.58477 3.61503
0.00978 5.02819 5.75054 0.61958 0.77255 0.54140 0.87211
593 2.54219 5.09335 3.33418 2.59904 4.32441 2.88560 3.96059
2.69181 2.56488 3.05185 4.16611 2.64417 3.01283 2.45211 3.22890
2.58031 2.91039 2.71427 2.93278 4.27166 1041 q - - -
2.68618 4.42225 2.77519 2.73123 3.46354 2.40513 3.72494
3.29354 2.67741 2.69355 4.24690 2.90347 2.73739 3.18146 2.89801
2.37887 2.77519 2.98518 4.58477 3.61503
0.38770 5.08222 1.15463 0.61958 0.77255 0.52385 0.89706
594 2.77999 5.18811 2.40628 2.51424 4.49320 3.56951 3.76164
3.46635 2.38216 3.09046 4.23997 1.87924 3.96235 2.87291 2.57123
2.57652 2.58671 3.18008 5.64569 4.26075 1042 n - - -
2.68618 4.42225 2.77519 2.73123 3.46354 2.40513 3.72494
3.29354 2.67741 2.69355 4.24690 2.90347 2.73739 3.18146 2.89801
2.37887 2.77519 2.98518 4.58477 3.61503
0.12601 4.74503 2.20998 0.61958 0.77255 1.20319 0.35701
595 2.74218 4.48144 3.59249 3.02681 3.62348 3.09044 3.96643
2.36182 2.75455 2.31935 3.58613 3.43948 4.09425 3.28832 3.34048
1.87475 2.39161 2.75681 5.06311 3.29602 1043 s - - -
2.68618 4.42225 2.77519 2.73123 3.46354 2.40513 3.72494
3.29354 2.67741 2.69355 4.24690 2.90347 2.73739 3.18146 2.89801
2.37887 2.77519 2.98518 4.58477 3.61503

```
          0.04672  4.63356  3.32618  0.61958  0.77255  0.59917  0.79688
    596   2.83443  5.14953  2.91254  2.45798  3.28613  2.17562  3.83895
3.45415  2.61111  3.42327  4.21013  2.72402  3.09566  2.77178  3.08765
2.25867  2.63923  3.14584  5.62836  3.54096   1044 g - - -
          2.68618  4.42225  2.77519  2.73123  3.46354  2.40513  3.72494
3.29354  2.67741  2.69355  4.24690  2.90347  2.73739  3.18146  2.89801
2.37887  2.77519  2.98518  4.58477  3.61503
          0.01122  4.89178  5.61413  0.61958  0.77255  0.77210  0.61998
    597   2.63222  4.99628  2.60862  2.75125  4.21590  3.71191  3.92080
2.17700  2.38680  2.97196  4.07263  2.34808  4.10068  3.08727  3.19628
2.37361  2.51359  3.02511  4.20965  4.19306   1045 i - - -
          2.68618  4.42225  2.77519  2.73123  3.46354  2.40513  3.72494
3.29354  2.67741  2.69355  4.24690  2.90347  2.73739  3.18146  2.89801
2.37887  2.77519  2.98518  4.58477  3.61503
          0.10131  4.95753  2.41554  0.61958  0.77255  0.91218  0.51357
    598   2.60425  5.30489  2.61729  2.39936  4.63794  3.25383  3.35749
4.11553  2.38590  2.56255  4.34873  2.36425  3.20640  2.89004  2.63747
2.42034  2.63438  3.68905  5.74481  3.67584   1046 n - - -
          2.68618  4.42225  2.77519  2.73123  3.46354  2.40513  3.72494
3.29354  2.67741  2.69355  4.24690  2.90347  2.73739  3.18146  2.89801
2.37887  2.77519  2.98518  4.58477  3.61503
          0.01149  4.86771  5.59006  0.61958  0.77255  0.74518  0.64369
    599   2.86584  4.84849  3.14315  2.86836  3.57098  1.89119  3.97480
2.92324  2.65198  2.79516  3.93577  3.35358  4.13932  2.72975  3.27995
2.28371  2.64408  2.62931  5.39113  3.53511   1047 g - - -
          2.68618  4.42225  2.77519  2.73123  3.46354  2.40513  3.72494
3.29354  2.67741  2.69355  4.24690  2.90347  2.73739  3.18146  2.89801
2.37887  2.77519  2.98518  4.58477  3.61503
          0.26474  4.95377  1.48927  0.61958  0.77255  0.58069  0.81987
    600   2.80336  5.22097  2.84651  2.38620  3.73164  3.19573  3.78366
3.98974  2.33482  3.51034  3.68608  2.33704  3.98481  2.50036  3.02042
2.06358  2.63878  2.96014  5.67646  3.63756   1048 s - - -
          2.68625  4.42149  2.77560  2.73126  3.46395  2.40501  3.72482
3.29265  2.67762  2.69375  4.24731  2.90387  2.73748  3.18133  2.89802
2.37846  2.77526  2.98534  4.58518  3.61409
          0.94480  0.70718  2.13528  0.88132  0.53484  0.46460  0.98990
    601   2.19607  5.15489  2.95291  2.51492  3.08938  3.03253  3.38819
3.33724  2.50060  2.92412  4.21666  2.71066  4.05420  2.32789  3.11086
2.66827  2.51377  3.50354  4.12290  4.27736   1054 a - - -
          2.68618  4.42225  2.77519  2.73123  3.46354  2.40513  3.72494
3.29354  2.67741  2.69355  4.24690  2.90347  2.73739  3.18146  2.89801
2.37887  2.77519  2.98518  4.58477  3.61503
          0.04004  4.93723  3.43966  0.61958  0.77255  0.48582  0.95502
    602   2.89649  5.17371  3.25265  2.36986  2.88941  2.86180  3.91071
3.86451  2.56622  3.44211  4.23776  2.93911  3.57428  2.81640  2.91159
1.84406  2.85281  2.54408  5.66066  3.30016   1055 s - - -
          2.68618  4.42225  2.77519  2.73123  3.46354  2.40513  3.72494
3.29354  2.67741  2.69355  4.24690  2.90347  2.73739  3.18146  2.89801
2.37887  2.77519  2.98518  4.58477  3.61503
          0.03688  5.03938  3.51550  0.61958  0.77255  0.62854  0.76222
    603   2.31310  4.42574  4.28711  3.70401  2.64293  4.00679  4.30238
2.23553  3.59908  2.07901  2.93903  3.18217  3.65367  2.77428  3.38377
2.93003  2.64917  2.12275  5.03394  3.83747   1056 l - - -
```

2.68618 4.42225 2.77519 2.73123 3.46354 2.40513 3.72494
3.29354 2.67741 2.69355 4.24690 2.90347 2.73739 3.18146 2.89801
2.37887 2.77519 2.98518 4.58477 3.61503
0.00967 5.03967 5.76201 0.61958 0.77255 0.64403 0.74481
604 2.53588 5.32244 2.68103 2.50367 4.63102 3.69218 3.47006
3.20416 2.36204 3.26067 3.72657 2.12105 3.52227 2.78751 2.93802
2.53918 2.92254 2.66417 5.77760 3.40672 1057 n - - -
2.68618 4.42225 2.77519 2.73123 3.46354 2.40513 3.72494
3.29354 2.67741 2.69355 4.24690 2.90347 2.73739 3.18146 2.89801
2.37887 2.77519 2.98518 4.58477 3.61503
0.06671 5.06249 2.84381 0.61958 0.77255 0.57416 0.82823
605 2.92122 4.36264 4.52548 3.93172 2.65303 4.04551 4.35809
2.17823 3.78836 1.87487 3.47890 2.87638 3.23821 3.98474 3.92171
3.05932 1.86123 2.30416 4.97771 3.24405 1058 t - - -
2.68618 4.42225 2.77519 2.73123 3.46354 2.40513 3.72494
3.29354 2.67741 2.69355 4.24690 2.90347 2.73739 3.18146 2.89801
2.37887 2.77519 2.98518 4.58477 3.61503
0.00973 5.03326 5.75560 0.61958 0.77255 0.76277 0.62806
606 2.89010 5.36206 2.94122 2.13450 4.69117 3.29619 3.85873
2.98836 2.19829 2.80629 4.40695 2.63086 2.97387 2.26034 3.08990
2.52147 2.92156 3.10179 5.80441 4.40329 1059 e - - -
2.68618 4.42225 2.77519 2.73123 3.46354 2.40513 3.72494
3.29354 2.67741 2.69355 4.24690 2.90347 2.73739 3.18146 2.89801
2.37887 2.77519 2.98518 4.58477 3.61503
0.31129 5.03326 1.34330 0.61958 0.77255 0.76277 0.62806
607 2.77843 5.14362 3.08905 2.53241 4.43393 3.57557 3.29562
3.44590 2.25747 3.42466 4.19951 2.64364 2.18916 2.31075 3.00404
2.60007 2.54446 3.50461 4.08278 4.23632 1060 p - - -
2.68618 4.42225 2.77519 2.73123 3.46354 2.40513 3.72494
3.29354 2.67741 2.69355 4.24690 2.90347 2.73739 3.18146 2.89801
2.37887 2.77519 2.98518 4.58477 3.61503
0.01313 4.73510 5.45744 0.61958 0.77255 0.37549 1.16140
608 2.72808 5.32712 3.18317 2.36936 4.63928 3.30858 3.87797
4.10220 2.29011 2.97838 4.37701 2.92694 3.54739 2.98627 2.52509
2.53692 1.58666 3.36497 5.78024 4.39072 1061 t - - -
2.68618 4.42225 2.77519 2.73123 3.46354 2.40513 3.72494
3.29354 2.67741 2.69355 4.24690 2.90347 2.73739 3.18146 2.89801
2.37887 2.77519 2.98518 4.58477 3.61503
0.43557 5.05219 1.05927 0.61958 0.77255 0.71962 0.66735
609 2.75970 4.21058 4.31237 3.72381 1.67340 3.87387 3.43568
2.44159 3.58722 2.40018 3.32620 3.88808 4.24180 3.78991 3.73513
2.84037 2.65757 2.11001 4.77911 2.76633 1062 f - - -
2.68618 4.42225 2.77519 2.73123 3.46354 2.40513 3.72494
3.29354 2.67741 2.69355 4.24690 2.90347 2.73739 3.18146 2.89801
2.37887 2.77519 2.98518 4.58477 3.61503
0.05839 4.63119 3.05822 0.61958 0.77255 1.19246 0.36165
610 2.76628 5.21464 2.72565 2.27320 4.53341 3.52536 3.73460
4.00246 2.49061 3.16745 4.26697 2.71095 3.39097 2.61219 2.98135
1.56791 2.74734 3.59203 5.66543 4.26754 1063 s - - -
2.68633 4.42240 2.77535 2.73139 3.46369 2.40515 3.72510
3.29369 2.67739 2.69370 4.24705 2.90341 2.73709 3.18134 2.89756
2.37877 2.77516 2.98511 4.58492 3.61518
0.35137 1.59905 2.36244 0.96559 0.47926 0.22465 1.60342

611 2.90789 5.03961 3.35567 2.79746 3.70999 2.50232 3.96691
3.66939 2.78725 2.72427 4.11968 2.48642 3.56983 3.13333 2.75427
2.55187 1.67367 3.06824 5.55925 4.23999 1068 t - - -
2.68618 4.42225 2.77519 2.73123 3.46354 2.40513 3.72494
3.29354 2.67741 2.69355 4.24690 2.90347 2.73739 3.18146 2.89801
2.37887 2.77519 2.98518 4.58477 3.61503
0.15042 5.05679 2.01523 0.61958 0.77255 0.70871 0.67782
612 2.25628 5.06060 3.23835 2.68060 4.30254 3.25558 3.39136
3.35932 2.67438 3.32074 3.05825 2.39223 3.51853 2.72040 3.13994
2.38530 2.20392 3.39456 5.56062 3.25521 1069 t - - -
2.68618 4.42225 2.77519 2.73123 3.46354 2.40513 3.72494
3.29354 2.67741 2.69355 4.24690 2.90347 2.73739 3.18146 2.89801
2.37887 2.77519 2.98518 4.58477 3.61503
0.01093 4.91730 5.63965 0.61958 0.77255 0.33192 1.26424
613 2.76336 5.25658 3.05302 2.25881 3.10019 2.52174 3.92870
3.21895 2.69773 3.24672 4.31585 2.59845 3.59465 2.63295 3.17561
2.11133 2.61875 3.61007 4.23433 3.71433 1070 s - - -
2.68618 4.42225 2.77519 2.73123 3.46354 2.40513 3.72494
3.29354 2.67741 2.69355 4.24690 2.90347 2.73739 3.18146 2.89801
2.37887 2.77519 2.98518 4.58477 3.61503
0.00898 5.11275 5.83510 0.61958 0.77255 0.56085 0.84565
614 2.73918 5.33845 2.45781 2.40152 4.64453 2.86970 3.91133
3.06005 2.66489 3.62767 4.39123 2.31128 4.11105 3.02256 3.15028
1.87122 2.67089 2.69969 5.79705 4.41131 1071 s - - -
2.68618 4.42225 2.77519 2.73123 3.46354 2.40513 3.72494
3.29354 2.67741 2.69355 4.24690 2.90347 2.73739 3.18146 2.89801
2.37887 2.77519 2.98518 4.58477 3.61503
0.05957 5.11275 2.96020 0.61958 0.77255 0.56085 0.84565
615 2.90639 5.19307 2.84823 2.51000 4.45645 2.26609 3.91817
3.12162 2.69683 2.70359 4.25626 2.59391 2.99996 3.05047 2.93072
2.55976 2.90031 3.14646 5.67803 2.67729 1072 g - - -
2.68618 4.42225 2.77519 2.73123 3.46354 2.40513 3.72494
3.29354 2.67741 2.69355 4.24690 2.90347 2.73739 3.18146 2.89801
2.37887 2.77519 2.98518 4.58477 3.61503
0.03427 5.06263 3.59872 0.61958 0.77255 0.46791 0.98433
616 2.62581 5.42104 2.75736 2.35090 3.92786 2.82443 3.87536
4.24958 2.36221 3.34443 4.46209 2.43463 2.94611 2.60959 2.63135
2.29571 2.45072 3.81089 5.85460 4.44452 1073 s - - -
2.68618 4.42225 2.77519 2.73123 3.46354 2.40513 3.72494
3.29354 2.67741 2.69355 4.24690 2.90347 2.73739 3.18146 2.89801
2.37887 2.77519 2.98518 4.58477 3.61503
0.00919 5.09011 5.81246 0.61958 0.77255 0.51234 0.91403
617 2.92825 5.42650 1.87490 2.49520 4.02001 3.70143 3.89093
4.24826 2.62883 3.72937 4.46882 1.85829 3.26890 2.63838 2.89641
2.30990 2.92559 3.81414 5.86240 3.78178 1074 n - - -
2.68618 4.42225 2.77519 2.73123 3.46354 2.40513 3.72494
3.29354 2.67741 2.69355 4.24690 2.90347 2.73739 3.18146 2.89801
2.37887 2.77519 2.98518 4.58477 3.61503
0.00898 5.11347 5.83581 0.61958 0.77255 0.55875 0.84845
618 2.54734 4.50901 4.23777 3.30746 3.63605 4.01818 4.33894
2.97250 3.57623 1.08954 3.63572 3.94203 3.31097 3.82815 3.81789
3.02675 2.46648 2.28450 5.13048 3.92915 1075 l - - -
2.68618 4.42225 2.77519 2.73123 3.46354 2.40513 3.72494
3.29354 2.67741 2.69355 4.24690 2.90347 2.73739 3.18146 2.89801
2.37887 2.77519 2.98518 4.58477 3.61503

```
         0.00898  5.11347  5.83581  0.61958  0.77255  0.55875  0.84845
    619   2.73723  5.40981  3.18438  2.50150  4.74425  3.70302  3.89095
3.25047  1.90937  3.71041  4.45334  2.43726  3.25212  2.47244  2.54454
2.53838  2.18912  3.32463  5.84879  4.44468   1076 k - - -
         2.68618  4.42225  2.77519  2.73123  3.46354  2.40513  3.72494
3.29354  2.67741  2.69355  4.24690  2.90347  2.73739  3.18146  2.89801
2.37887  2.77519  2.98518  4.58477  3.61503
         0.00898  5.11347  5.83581  0.61958  0.77255  0.44268  1.02809
    620   3.68019  5.11579  4.85007  4.36470  2.03271  4.63642  3.45298
3.62800  4.25491  3.23235  4.25404  3.52893  4.98769  4.40330  4.42293
2.91975  3.90892  3.00223  4.58894  0.71901   1077 y - - -
         2.68618  4.42225  2.77519  2.73123  3.46354  2.40513  3.72494
3.29354  2.67741  2.69355  4.24690  2.90347  2.73739  3.18146  2.89801
2.37887  2.77519  2.98518  4.58477  3.61503
         0.00877  5.13716  5.85950  0.61958  0.77255  0.48576  0.95510
    621   2.96875  5.48682  2.57596  1.53468  4.83130  3.07855  3.92259
4.31963  2.19801  3.79164  4.52907  2.20222  4.12462  2.39750  2.92208
2.59359  3.20150  3.87742  5.91815  4.50291   1078 e - - -
         2.68618  4.42225  2.77519  2.73123  3.46354  2.40513  3.72494
3.29354  2.67741  2.69355  4.24690  2.90347  2.73739  3.18146  2.89801
2.37887  2.77519  2.98518  4.58477  3.61503
         0.00877  5.13716  5.85950  0.61958  0.77255  0.48576  0.95510
    622   2.76542  5.47252  2.02845  2.05973  4.81590  3.71688  2.84430
4.30293  2.65766  3.77714  4.51470  2.44440  3.65195  2.80623  3.15303
1.92807  2.66621  3.86246  5.90524  4.49156   1079 s - - -
         2.68618  4.42225  2.77519  2.73123  3.46354  2.40513  3.72494
3.29354  2.67741  2.69355  4.24690  2.90347  2.73739  3.18146  2.89801
2.37887  2.77519  2.98518  4.58477  3.61503
         0.00877  5.13716  5.85950  0.61958  0.77255  0.48576  0.95510
    623   4.95512  5.95321  5.95703  5.83661  0.35158  5.56576  4.30555
4.37723  5.60694  3.52210  3.66146  5.15920  5.81203  5.29497  5.42263
4.96671  5.15430  4.39385  4.38237  1.98976   1080 F - - -
         2.68618  4.42225  2.77519  2.73123  3.46354  2.40513  3.72494
3.29354  2.67741  2.69355  4.24690  2.90347  2.73739  3.18146  2.89801
2.37887  2.77519  2.98518  4.58477  3.61503
         0.00877  5.13716  5.85950  0.61958  0.77255  0.48576  0.95510
    624   2.46470  5.45100  2.61150  2.63364  4.79591  2.41482  3.90185
4.28077  1.91026  3.75566  4.49258  3.18155  3.59908  1.89173  2.42743
2.72234  3.17439  3.84174  5.88339  4.47375   1081 q - - -
         2.68618  4.42225  2.77519  2.73123  3.46354  2.40513  3.72494
3.29354  2.67741  2.69355  4.24690  2.90347  2.73739  3.18146  2.89801
2.37887  2.77519  2.98518  4.58477  3.61503
         0.00877  5.13716  5.85950  0.61958  0.77255  0.48576  0.95510
    625   3.74081  5.06111  5.33708  4.86370  1.87347  4.78245  4.41747
2.97894  4.67776  2.97185  4.09094  4.72689  5.11225  4.71532  4.69114
4.11473  3.35376  2.86802  4.64020  0.68413   1082 Y - - -
         2.68618  4.42225  2.77519  2.73123  3.46354  2.40513  3.72494
3.29354  2.67741  2.69355  4.24690  2.90347  2.73739  3.18146  2.89801
2.37887  2.77519  2.98518  4.58477  3.61503
         0.00877  5.13716  5.85950  0.61958  0.77255  0.48576  0.95510
    626   1.82872  4.74958  3.28425  2.92235  2.87645  3.90696  3.21578
2.35857  2.71357  2.29128  3.84873  3.26924  4.28845  3.44100  3.50834
2.52906  3.18796  2.64469  5.32144  3.54225   1083 a - - -
```

2.68618 4.42225 2.77519 2.73123 3.46354 2.40513 3.72494
3.29354 2.67741 2.69355 4.24690 2.90347 2.73739 3.18146 2.89801
2.37887 2.77519 2.98518 4.58477 3.61503
0.00877 5.13716 5.85950 0.61958 0.77255 0.48576 0.95510
627 2.77652 5.43652 1.86787 1.80660 4.77418 3.71367 3.90473
4.25554 2.64415 3.73866 3.62286 2.41859 4.10924 2.80583 3.13645
2.60209 2.48427 3.82323 5.87341 3.36772 1084 e - - -
2.68618 4.42225 2.77519 2.73123 3.46354 2.40513 3.72494
3.29354 2.67741 2.69355 4.24690 2.90347 2.73739 3.18146 2.89801
2.37887 2.77519 2.98518 4.58477 3.61503
0.00877 5.13716 5.85950 0.61958 0.77255 0.48576 0.95510
628 2.58016 4.36101 4.90962 4.29688 1.81067 4.14722 4.47913
2.19140 4.08976 1.74669 3.15949 4.29798 4.51093 4.22754 4.11051
2.91977 2.55680 2.16855 4.98293 3.07432 1085 l - - -
2.68618 4.42225 2.77519 2.73123 3.46354 2.40513 3.72494
3.29354 2.67741 2.69355 4.24690 2.90347 2.73739 3.18146 2.89801
2.37887 2.77519 2.98518 4.58477 3.61503
0.00877 5.13716 5.85950 0.61958 0.77255 0.48576 0.95510
629 2.59291 5.27664 2.76570 2.52312 3.89323 2.55436 3.93733
3.61444 2.70439 2.48529 4.33508 2.78903 2.26327 3.06017 2.97584
2.36866 2.85611 3.24593 5.75040 4.38251 1086 p - - -
2.68618 4.42225 2.77519 2.73123 3.46354 2.40513 3.72494
3.29354 2.67741 2.69355 4.24690 2.90347 2.73739 3.18146 2.89801
2.37887 2.77519 2.98518 4.58477 3.61503
0.00877 5.13716 5.85950 0.61958 0.77255 0.48576 0.95510
630 2.93680 5.34451 2.81299 2.36748 3.38091 2.42716 3.91933
2.98485 2.54045 3.63229 4.39609 2.62197 3.60807 2.63601 2.94216
2.60449 2.44849 2.69706 5.80233 3.76446 1087 e - - -
2.68618 4.42225 2.77519 2.73123 3.46354 2.40513 3.72494
3.29354 2.67741 2.69355 4.24690 2.90347 2.73739 3.18146 2.89801
2.37887 2.77519 2.98518 4.58477 3.61503
0.00877 5.13716 5.85950 0.61958 0.77255 0.48576 0.95510
631 2.62050 5.39130 2.58705 2.40362 4.71387 3.71831 3.47276
2.85851 2.65194 3.00666 4.43793 2.22730 3.59614 2.81000 2.91661
2.43491 2.12651 3.42676 5.83768 4.44071 1088 t - - -
2.68607 4.42238 2.77533 2.73121 3.46367 2.40526 3.72508
3.29308 2.67730 2.69353 4.24703 2.90330 2.73721 3.18159 2.89814
2.37900 2.77514 2.98512 4.58490 3.61516
0.20434 2.23785 2.54942 0.93146 0.50084 0.48576 0.95510
632 2.38500 4.32999 4.79809 4.18991 1.95890 4.09704 4.42526
2.30506 3.99756 2.06657 2.31027 3.59031 4.46156 4.14848 3.47397
3.40381 2.69858 1.95727 4.95097 3.77255 1092 v - - -
2.68618 4.42225 2.77519 2.73123 3.46354 2.40513 3.72494
3.29354 2.67741 2.69355 4.24690 2.90347 2.73739 3.18146 2.89801
2.37887 2.77519 2.98518 4.58477 3.61503
0.08776 5.05890 2.55535 0.61958 0.77255 0.70366 0.68274
633 2.66090 5.36190 3.12440 2.10502 4.69982 2.78312 3.83307
3.61287 2.06070 3.66400 4.40464 3.11575 3.57940 2.62626 2.69230
1.93279 2.54031 3.74874 5.79884 4.39268 1093 s - - -
2.68618 4.42225 2.77519 2.73123 3.46354 2.40513 3.72494
3.29354 2.67741 2.69355 4.24690 2.90347 2.73739 3.18146 2.89801
2.37887 2.77519 2.98518 4.58477 3.61503
0.01025 4.98139 5.70374 0.61958 0.77255 0.86865 0.54389

```
     634   2.59905  4.43409  4.14281  3.10544  1.97128  3.42250  4.24225
 2.45927  3.47529  1.89786  2.67511  3.35627  3.68701  3.26773  3.72271
 2.88280  2.85351  2.69257  5.03801  3.83520   1094 l - - -
           2.68618  4.42225  2.77519  2.73123  3.46354  2.40513  3.72494
 3.29354  2.67741  2.69355  4.24690  2.90347  2.73739  3.18146  2.89801
 2.37887  2.77519  2.98518  4.58477  3.61503
           0.07723  4.98139  2.69621  0.61958  0.77255  0.45564  1.00524
     635   2.38692  5.27916  2.94438  2.47872  3.81943  3.28459  3.43812
 3.52592  2.63315  3.25749  4.33260  2.76065  2.45155  2.99087  2.56416
 2.57507  2.16932  3.24238  5.74130  4.36087   1095 t - - -
           2.68618  4.42225  2.77519  2.73123  3.46354  2.40513  3.72494
 3.29354  2.67741  2.69355  4.24690  2.90347  2.73739  3.18146  2.89801
 2.37887  2.77519  2.98518  4.58477  3.61503
           0.00976  5.02974  5.75209  0.61958  0.77255  0.60632  0.78824
     636   2.71796  5.38953  2.95732  2.36712  4.72373  2.49356  3.87100
 3.64379  2.48746  3.69023  4.43321  2.56919  2.34499  2.74986  3.10130
 2.06926  2.67931  3.37898  5.82888  4.42451   1096 s - - -
           2.68618  4.42225  2.77519  2.73123  3.46354  2.40513  3.72494
 3.29354  2.67741  2.69355  4.24690  2.90347  2.73739  3.18146  2.89801
 2.37887  2.77519  2.98518  4.58477  3.61503
           0.00941  5.06683  5.78918  0.61958  0.77255  0.68431  0.70206
     637   2.54830  5.16010  3.05346  2.58272  2.58751  3.03316  3.93078
 3.10675  2.71828  3.42509  4.22670  2.13405  4.12047  2.83456  3.18809
 2.07758  2.58309  3.21779  5.65281  4.30588   1097 s - - -
           2.68618  4.42225  2.77519  2.73123  3.46354  2.40513  3.72494
 3.29354  2.67741  2.69355  4.24690  2.90347  2.73739  3.18146  2.89801
 2.37887  2.77519  2.98518  4.58477  3.61503
           0.00941  5.06683  5.78918  0.61958  0.77255  0.68431  0.70206
     638   2.71990  5.39550  3.16015  2.22727  4.73225  2.64532  3.16469
 4.21238  2.47008  2.80594  4.43842  2.75291  3.48130  2.27171  3.09875
 2.24568  2.30856  3.78168  5.83318  4.42729   1098 e - - -
           2.68618  4.42225  2.77519  2.73123  3.46354  2.40513  3.72494
 3.29354  2.67741  2.69355  4.24690  2.90347  2.73739  3.18146  2.89801
 2.37887  2.77519  2.98518  4.58477  3.61503
           0.03352  5.06683  3.62456  0.61958  0.77255  0.37904  1.15365
     639   2.75650  5.39846  2.63047  2.06810  4.72795  3.70517  3.89396
 2.97805  2.63500  3.69709  4.44325  2.35603  2.93838  2.64044  2.92906
 2.31793  2.90194  3.10546  5.84058  3.05988   1099 e - - -
           2.68618  4.42225  2.77519  2.73123  3.46354  2.40513  3.72494
 3.29354  2.67741  2.69355  4.24690  2.90347  2.73739  3.18146  2.89801
 2.37887  2.77519  2.98518  4.58477  3.61503
           0.20894  5.11491  1.70072  0.61958  0.77255  0.55449  0.85417
     640   2.48058  4.39117  2.73499  3.57227  3.08430  3.93392  4.22374
 2.07859  3.47825  1.81861  3.50378  3.39217  4.30600  3.72484  3.71374
 2.90006  2.46937  2.26199  4.99689  3.31123   1100 l - - -
           2.68618  4.42225  2.77519  2.73123  3.46354  2.40513  3.72494
 3.29354  2.67741  2.69355  4.24690  2.90347  2.73739  3.18146  2.89801
 2.37887  2.77519  2.98518  4.58477  3.61503
           0.09344  4.91691  2.50245  0.61958  0.77255  0.26750  1.44941
     641   2.71867  5.38747  2.59887  2.23587  4.72018  2.81607  3.44089
 2.95718  2.38990  3.68747  4.43139  3.15731  3.56924  2.64442  2.87815
 2.44838  2.21465  2.84176  5.82762  4.42423   1101 t - - -
           2.68618  4.42225  2.77519  2.73123  3.46354  2.40513  3.72494
 3.29354  2.67741  2.69355  4.24690  2.90347  2.73739  3.18146  2.89801
 2.37887  2.77519  2.98518  4.58477  3.61503
```

0.07517 5.07225 2.71589 0.61958 0.77255 0.52933 0.88916
642 2.91991 4.45086 4.20539 2.89199 3.23698 3.99089 4.28026
1.71425 3.53204 2.12265 3.56336 3.09331 3.66063 3.77938 3.76903
2.75447 2.19710 2.27846 5.05622 3.36831 1102 i - - -
2.68618 4.42225 2.77519 2.73123 3.46354 2.40513 3.72494
3.29354 2.67741 2.69355 4.24690 2.90347 2.73739 3.18146 2.89801
2.37887 2.77519 2.98518 4.58477 3.61503
0.10122 5.03946 2.41033 0.61958 0.77255 0.74892 0.64032
643 2.68764 4.58829 3.75380 3.18604 3.72452 3.85161 2.87779
2.50183 2.87573 2.05922 3.69165 3.59032 4.23083 3.44073 2.88142
2.34687 2.12719 2.49228 5.17052 3.46954 1103 l - - -
2.68618 4.42225 2.77519 2.73123 3.46354 2.40513 3.72494
3.29354 2.67741 2.69355 4.24690 2.90347 2.73739 3.18146 2.89801
2.37887 2.77519 2.98518 4.58477 3.61503
0.13095 4.94883 2.15723 0.61958 0.77255 0.92735 0.50352
644 2.82576 4.21703 4.68556 4.07719 2.72496 3.45644 4.31144
2.06288 3.88459 1.76532 2.98426 3.40354 4.34820 4.03530 3.92947
2.70775 2.78409 1.95649 3.77212 3.13258 1104 l - - -
2.68618 4.42225 2.77519 2.73123 3.46354 2.40513 3.72494
3.29354 2.67741 2.69355 4.24690 2.90347 2.73739 3.18146 2.89801
2.37887 2.77519 2.98518 4.58477 3.61503
0.14603 4.82982 2.05666 0.61958 0.77255 1.10406 0.40275
645 2.76262 4.95591 2.69339 2.31151 3.19965 2.97913 3.38418
3.60740 2.60258 2.18943 4.02724 3.12826 3.98531 2.95185 2.60104
2.64013 2.75691 3.28645 5.45995 3.18826 1105 l - - -
2.68618 4.42225 2.77519 2.73123 3.46354 2.40513 3.72494
3.29354 2.67741 2.69355 4.24690 2.90347 2.73739 3.18146 2.89801
2.37887 2.77519 2.98518 4.58477 3.61503
0.01364 4.69743 5.41978 0.61958 0.77255 0.41730 1.07535
646 2.72821 4.62789 3.77716 2.45410 2.59968 3.88252 3.54773
2.89904 2.90074 1.94979 3.38518 3.21524 4.26195 3.08881 3.52003
2.65325 2.69529 2.38399 5.20937 3.19043 1106 l - - -
2.68658 4.42265 2.77521 2.73129 3.46160 2.40528 3.72535
3.29230 2.67781 2.69395 4.24640 2.90367 2.73762 3.18134 2.89841
2.37888 2.77544 2.98514 4.58517 3.61266
0.35179 1.22630 5.74532 1.15405 0.37886 0.43975 1.03337
647 2.92430 5.29034 2.98335 2.43321 4.57986 3.41527 3.52495
2.58102 2.51728 2.70779 3.71848 2.37616 3.54281 2.66930 2.58882
2.75403 2.57142 3.65076 3.69494 3.65708 1115 n - - -
2.68618 4.42225 2.77519 2.73123 3.46354 2.40513 3.72494
3.29354 2.67741 2.69355 4.24690 2.90347 2.73739 3.18146 2.89801
2.37887 2.77519 2.98518 4.58477 3.61503
0.08637 5.10728 2.56796 0.61958 0.77255 0.57671 0.82495
648 2.57787 4.99211 2.71142 2.62773 3.00894 3.37699 3.96497
2.37180 2.59796 2.56303 3.53655 3.05337 4.14145 2.89686 2.80469
2.60836 2.49931 3.02373 5.51498 4.20380 1116 i - - -
2.68618 4.42225 2.77519 2.73123 3.46354 2.40513 3.72494
3.29354 2.67741 2.69355 4.24690 2.90347 2.73739 3.18146 2.89801
2.37887 2.77519 2.98518 4.58477 3.61503
0.38603 5.03066 1.15927 0.61958 0.77255 0.76848 0.62310
649 2.56058 5.19410 2.39667 2.29236 3.65521 3.53212 3.72227
3.98076 2.46807 3.11379 4.24177 2.79837 3.92507 2.30419 2.54191
2.41270 2.58353 3.57055 5.64280 3.58769 1117 e - - -

```
          2.68618  4.42225  2.77519  2.73123  3.46354  2.40513  3.72494
3.29354  2.67741  2.69355  4.24690  2.90347  2.73739  3.18146  2.89801
2.37887  2.77519  2.98518  4.58477  3.61503
          0.14175  4.65881  2.09817  0.61958  0.77255  0.32925  1.27104
    650   2.67822  5.27006  2.44864  2.40417  3.22088  3.22435  3.85148
3.57739  2.30263  2.60077  4.32218  2.40258  4.05171  2.96360  2.81467
2.38304  2.86688  3.63758  5.72914  3.35544   1118 k - - -
          2.68618  4.42225  2.77519  2.73123  3.46354  2.40513  3.72494
3.29354  2.67741  2.69355  4.24690  2.90347  2.73739  3.18146  2.89801
2.37887  2.77519  2.98518  4.58477  3.61503
          0.01029  4.97744  5.69978  0.61958  0.77255  0.30169  1.34541
    651   2.60410  5.29481  2.99968  2.30389  3.91369  3.35671  3.93231
2.39761  2.56475  3.25246  4.35142  2.08803  3.58979  2.86380  2.96326
2.38086  3.16924  3.09566  5.76435  3.37090   1119 n - - -
          2.68618  4.42225  2.77519  2.73123  3.46354  2.40513  3.72494
3.29354  2.67741  2.69355  4.24690  2.90347  2.73739  3.18146  2.89801
2.37887  2.77519  2.98518  4.58477  3.61503
          0.20533  5.13716  1.71621  0.61958  0.77255  0.48576  0.95510
    652   2.68668  4.86483  3.04899  2.64789  3.43115  3.31982  3.96111
2.16069  2.82751  2.50028  3.95058  3.06154  3.18118  2.67741  3.26149
2.14918  3.09195  2.94131  5.40379  3.57566   1120 s - - -
          2.68618  4.42225  2.77519  2.73123  3.46354  2.40513  3.72494
3.29354  2.67741  2.69355  4.24690  2.90347  2.73739  3.18146  2.89801
2.37887  2.77519  2.98518  4.58477  3.61503
          0.03983  4.94249  3.44492  0.61958  0.77255  0.28070  1.40753
    653   2.29516  5.15371  3.31868  2.42925  4.39989  1.96066  3.96143
3.82052  2.75610  2.88205  4.22441  3.05325  3.26824  3.10670  3.22264
2.07879  2.74532  2.95434  5.65378  3.64272   1121 g - - -
          2.68618  4.42225  2.77519  2.73123  3.46354  2.40513  3.72494
3.29354  2.67741  2.69355  4.24690  2.90347  2.73739  3.18146  2.89801
2.37887  2.77519  2.98518  4.58477  3.61503
          0.00898  5.11347  5.83581  0.61958  0.77255  0.44268  1.02809
    654   2.60474  5.35299  2.41933  2.66907  3.51090  2.42656  3.91845
4.12079  2.50563  3.64239  4.40422  1.85397  4.11919  3.02858  3.15580
2.45434  2.56945  3.35195  5.80936  3.71647   1122 n - - -
          2.68618  4.42225  2.77519  2.73123  3.46354  2.40513  3.72494
3.29354  2.67741  2.69355  4.24690  2.90347  2.73739  3.18146  2.89801
2.37887  2.77519  2.98518  4.58477  3.61503
          0.00877  5.13716  5.85950  0.61958  0.77255  0.48576  0.95510
    655   2.60149  5.28467  3.25883  2.57426  3.74831  2.31954  3.54131
3.50931  2.70063  3.56310  4.34244  2.34636  4.13189  2.53574  3.18013
1.91901  2.59164  3.05689  5.75671  4.38684   1123 s - - -
          2.68618  4.42225  2.77519  2.73123  3.46354  2.40513  3.72494
3.29354  2.67741  2.69355  4.24690  2.90347  2.73739  3.18146  2.89801
2.37887  2.77519  2.98518  4.58477  3.61503
          0.00877  5.13716  5.85950  0.61958  0.77255  0.48576  0.95510
    656   2.93491  5.43751  2.40697  1.98750  4.77916  3.09858  3.52047
3.05969  2.51517  3.36220  4.47907  2.46039  2.95251  2.51129  3.12564
2.74255  2.47295  3.82631  5.87223  3.79412   1124 e - - -
          2.68618  4.42225  2.77519  2.73123  3.46354  2.40513  3.72494
3.29354  2.67741  2.69355  4.24690  2.90347  2.73739  3.18146  2.89801
2.37887  2.77519  2.98518  4.58477  3.61503
          0.03836  5.13716  3.44959  0.61958  0.77255  0.48576  0.95510
```

```
    657   3.44036  3.69415  5.44469  4.86097  2.24422  4.72535  5.10736
1.95115  4.68177  1.34074  3.63974  4.88980  5.02373  4.79143  4.69968
4.06308  3.67478  1.27337  5.50756  4.35768   1125 v - - -
          2.68618  4.42225  2.77519  2.73123  3.46354  2.40513  3.72494
3.29354  2.67741  2.69355  4.24690  2.90347  2.73739  3.18146  2.89801
2.37887  2.77519  2.98518  4.58477  3.61503
          0.00903  5.10782  5.83017  0.61958  0.77255  0.57514  0.82697
    658   2.97612  4.36331  4.85151  3.58745  3.13498  4.13778  4.46646
1.33641  4.04755  1.85331  3.12356  3.67962  4.50097  4.19507  4.08730
3.44567  3.20830  2.26655  4.98484  2.32413   1126 i - - -
          2.68618  4.42225  2.77519  2.73123  3.46354  2.40513  3.72494
3.29354  2.67741  2.69355  4.24690  2.90347  2.73739  3.18146  2.89801
2.37887  2.77519  2.98518  4.58477  3.61503
          0.00903  5.10782  5.83017  0.61958  0.77255  0.57514  0.82697
    659   4.05446  5.22896  6.15334  5.66365  4.11592  5.67346  6.28510
0.76085  5.59656  1.33255  3.83541  5.84526  5.77898  5.66254  5.66438
5.11528  4.29711  1.86820  6.35908  5.28988   1127 i - - -
          2.68618  4.42225  2.77519  2.73123  3.46354  2.40513  3.72494
3.29354  2.67741  2.69355  4.24690  2.90347  2.73739  3.18146  2.89801
2.37887  2.77519  2.98518  4.58477  3.61503
          0.00903  5.10782  5.83017  0.61958  0.77255  0.57514  0.82697
    660   4.82335  6.68413  0.11091  3.89363  6.35158  4.64874  5.61608
6.32527  4.96712  5.70100  6.81892  4.46981  5.35811  4.93926  5.51530
4.73992  5.20877  5.86773  7.17172  6.20113   1128 D - - -
          2.68618  4.42225  2.77519  2.73123  3.46354  2.40513  3.72494
3.29354  2.67741  2.69355  4.24690  2.90347  2.73739  3.18146  2.89801
2.37887  2.77519  2.98518  4.58477  3.61503
          0.03482  5.10782  3.56944  0.61958  0.77255  0.43367  1.04448
    661   3.95527  6.03343  4.56281  3.67060  5.68604  4.49348  4.26637
4.93536  0.72926  4.24954  5.13285  3.99824  4.81411  3.38784  1.35849
2.95895  4.06249  4.61387  6.18827  5.11818   1129 k - - -
          2.68618  4.42225  2.77519  2.73123  3.46354  2.40513  3.72494
3.29354  2.67741  2.69355  4.24690  2.90347  2.73739  3.18146  2.89801
2.37887  2.77519  2.98518  4.58477  3.61503
          0.03934  5.11235  3.42478  0.61958  0.77255  0.56204  0.84408
    662   3.91607  5.11462  6.00004  5.49490  3.40246  5.47333  6.03213
0.67208  5.41011  1.85633  3.84201  5.63693  5.63499  5.50930  5.47954
4.88726  4.15910  1.67033  6.21826  5.10071   1130 I - - -
          2.68618  4.42225  2.77519  2.73123  3.46354  2.40513  3.72494
3.29354  2.67741  2.69355  4.24690  2.90347  2.73739  3.18146  2.89801
2.37887  2.77519  2.98518  4.58477  3.61503
          0.03454  5.08227  3.58470  0.61958  0.77255  0.50365  0.92716
    663   3.91129  6.10650  3.30473  0.33668  5.25482  4.19633  4.76916
4.75663  3.66948  3.22895  5.37606  3.79803  4.81691  3.98535  4.09382
3.86693  4.21890  4.48463  6.54139  5.16555   1131 E - - -
          2.68618  4.42225  2.77519  2.73123  3.46354  2.40513  3.72494
3.29354  2.67741  2.69355  4.24690  2.90347  2.73739  3.18146  2.89801
2.37887  2.77519  2.98518  4.58477  3.61503
          0.00921  5.08805  5.81040  0.61958  0.77255  0.62984  0.76074
    664   3.15760  3.53884  5.01419  4.42492  0.92285  4.31334  4.59775
2.58340  4.23381  1.92156  3.54562  4.44496  4.67097  4.36528  4.27322
3.11647  3.39516  2.67321  5.05014  3.78627   1132 f - - -
          2.68618  4.42225  2.77519  2.73123  3.46354  2.40513  3.72494
3.29354  2.67741  2.69355  4.24690  2.90347  2.73739  3.18146  2.89801
2.37887  2.77519  2.98518  4.58477  3.61503
```

```
         0.00921  5.08805  5.81040  0.61958  0.77255  0.62984  0.76074
    665  2.99376  4.39265  4.78228  4.18105  3.07843  4.14988  4.47275
0.92492  3.44721  2.27841  3.50043  4.25097  4.51214  4.16866  4.07384
2.98581  3.22633  2.40969  5.01650  3.34536   1133 i - - -
         2.68618  4.42225  2.77519  2.73123  3.46354  2.40513  3.72494
3.29354  2.67741  2.69355  4.24690  2.90347  2.73739  3.18146  2.89801
2.37887  2.77519  2.98518  4.58477  3.61503
         0.00921  5.08805  5.81040  0.61958  0.77255  0.62984  0.76074
    666  2.99940  4.90546  4.60376  4.46409  5.24021  3.70834  5.37887
4.61192  4.47629  4.39030  5.26072  4.33579  0.40418  4.74482  4.65288
2.72734  3.54759  3.00922  6.64118  5.46045   1134 P - - -
         2.68618  4.42225  2.77519  2.73123  3.46354  2.40513  3.72494
3.29354  2.67741  2.69355  4.24690  2.90347  2.73739  3.18146  2.89801
2.37887  2.77519  2.98518  4.58477  3.61503
         0.00921  5.08805  5.81040  0.61958  0.77255  0.62984  0.76074
    667  2.72505  4.51849  2.88811  3.13238  3.63449  3.98869  4.26804
1.90906  3.45261  2.21085  2.95493  3.33658  4.36282  3.71748  3.72664
2.42254  3.17395  1.86175  5.11869  3.34767   1135 v - - -
         2.68630  4.42237  2.77532  2.73135  3.46327  2.40507  3.72507
3.29297  2.67753  2.69343  4.24616  2.90336  2.73732  3.18120  2.89813
2.37899  2.77532  2.98531  4.58489  3.61515
         0.13496  2.32690  3.55250  0.98868  0.46532  0.62984  0.76074
    668  2.90227  3.97302  2.12854  2.44526  4.72270  3.01772  3.86714
4.20169  2.61033  3.68977  4.43383  2.12400  3.07047  2.79628  3.10263
2.67057  1.98430  3.77348  5.82872  4.42302   1139 t - - -
         2.68618  4.42225  2.77519  2.73123  3.46354  2.40513  3.72494
3.29354  2.67741  2.69355  4.24690  2.90347  2.73739  3.18146  2.89801
2.37887  2.77519  2.98518  4.58477  3.61503
         0.03253  5.03216  3.66978  0.61958  0.77255  0.69601  0.69030
    669  2.70876  5.17440  3.23214  2.28553  4.43921  3.01592  3.89394
2.49829  2.67145  2.84582  4.23721  2.98361  2.73087  2.48680  2.68911
2.53021  2.71297  2.96534  5.65841  4.30192   1140 e - - -
         2.68618  4.42225  2.77519  2.73123  3.46354  2.40513  3.72494
3.29354  2.67741  2.69355  4.24690  2.90347  2.73739  3.18146  2.89801
2.37887  2.77519  2.98518  4.58477  3.61503
         0.04969  5.00959  3.17492  0.61958  0.77255  0.74732  0.64176
    670  2.85950  4.78333  3.47925  2.91781  3.36198  3.32456  3.99302
2.61292  2.67750  2.56120  3.87537  2.68184  2.72266  3.22489  3.31215
2.31885  1.94105  3.07824  5.33635  4.06212   1141 t - - -
         2.68618  4.42225  2.77519  2.73123  3.46354  2.40513  3.72494
3.29354  2.67741  2.69355  4.24690  2.90347  2.73739  3.18146  2.89801
2.37887  2.77519  2.98518  4.58477  3.61503
         0.01073  4.93552  5.65786  0.61958  0.77255  0.82845  0.57399
    671  2.65533  4.70935  3.56295  2.40566  3.44313  3.33732  4.02754
2.60346  2.70084  2.25634  3.80525  2.80516  4.17457  3.29138  3.07780
2.78339  2.49807  2.42874  5.27318  2.95276   1142 l - - -
         2.68618  4.42225  2.77519  2.73123  3.46354  2.40513  3.72494
3.29354  2.67741  2.69355  4.24690  2.90347  2.73739  3.18146  2.89801
2.37887  2.77519  2.98518  4.58477  3.61503
         0.01073  4.93552  5.65786  0.61958  0.77255  0.82845  0.57399
    672  2.52568  5.28491  2.86307  1.95386  3.81046  3.63622  3.82673
4.06442  2.31024  2.46913  4.33371  2.87695  4.02892  2.71883  2.48848
2.65581  2.66023  3.27812  5.73630  3.59490   1143 e - - -
```

```
          2.68618  4.42225  2.77519  2.73123  3.46354  2.40513  3.72494
3.29354  2.67741  2.69355  4.24690  2.90347  2.73739  3.18146  2.89801
2.37887  2.77519  2.98518  4.58477  3.61503
          0.01073  4.93552  5.65786  0.61958  0.77255  0.82845  0.57399
    673   2.20187  5.35393  2.68176  1.63823  4.69335  3.62684  3.81738
4.17580  2.31488  2.94876  4.39640  2.86088  3.53941  2.51544  3.04802
2.81940  2.63322  3.74159  5.78972  4.38141   1144 e - - -
          2.68618  4.42225  2.77519  2.73123  3.46354  2.40513  3.72494
3.29354  2.67741  2.69355  4.24690  2.90347  2.73739  3.18146  2.89801
2.37887  2.77519  2.98518  4.58477  3.61503
          0.01073  4.93552  5.65786  0.61958  0.77255  0.82845  0.57399
    674   2.82781  3.57057  2.87945  2.19836  4.58552  3.61239  3.28955
4.05205  2.42374  3.56176  4.31684  2.84283  3.52085  2.69428  2.84010
2.45076  2.82659  3.64404  5.71847  2.00482   1145 y - - -
          2.68618  4.42225  2.77519  2.73123  3.46354  2.40513  3.72494
3.29354  2.67741  2.69355  4.24690  2.90347  2.73739  3.18146  2.89801
2.37887  2.77519  2.98518  4.58477  3.61503
          0.16190  4.87719  1.95297  0.61958  0.77255  0.82845  0.57399
    675   2.77269  5.26039  2.75806  1.64006  4.59471  3.53739  3.73383
4.07392  2.34329  3.15732  4.30510  2.38133  3.93686  2.83494  2.75055
2.54822  2.44332  3.64548  5.69858  4.29261   1146 e - - -
          2.68618  4.42225  2.77519  2.73123  3.46354  2.40513  3.72494
3.29354  2.67741  2.69355  4.24690  2.90347  2.73739  3.18146  2.89801
2.37887  2.77519  2.98518  4.58477  3.61503
          0.01384  4.68320  5.40555  0.61958  0.77255  1.04724  0.43218
    676   2.39446  3.96034  3.16452  1.79896  4.18360  2.71051  3.79656
3.59864  2.60002  2.88698  4.02009  2.80439  3.46477  2.94905  3.06254
2.79459  2.74765  2.97065  5.45327  3.48214   1147 e - - -
          2.68618  4.42225  2.77519  2.73123  3.46354  2.40513  3.72494
3.29354  2.67741  2.69355  4.24690  2.90347  2.73739  3.18146  2.89801
2.37887  2.77519  2.98518  4.58477  3.61503
          0.04854  4.68320  3.26672  0.61958  0.77255  1.04724  0.43218
    677   2.82896  5.32625  2.93309  2.16023  4.66676  2.59901  3.76345
4.14710  1.67865  3.62481  4.37574  2.16701  3.37095  2.65536  2.97806
2.78600  3.06245  3.71603  5.75506  4.34806   1148 k - - -
          2.68722  4.42294  2.77509  2.73180  3.46316  2.40683  3.72295
3.29318  2.67661  2.69333  4.24876  2.90215  2.73862  3.18007  2.89885
2.37823  2.77513  2.98510  4.58525  3.61199
          0.15685  1.92987        *  3.54358  0.02934  0.00000        *
//
HMMER3/f [3.3 | Nov 2019]
NAME  CryM2
DESC  Cry toxin (IDOPS Model 2)
LENG  656
ALPH  amino
RF    no
MM    no
CONS  yes
CS    no
MAP   yes
DATE  Wed Feb  3 11:08:28 2021
NSEQ  12
EFFN  0.758789
CKSUM 2954108072
GA 691.0 691.0
```

```
STATS LOCAL MSV       -12.3278  0.69687
STATS LOCAL VITERBI   -13.1321  0.69687
STATS LOCAL FORWARD    -6.4933  0.69687
HMM          A        C        D        E        F        G        H
I        K        L        M        N        P        Q        R        S
T        V        W        Y
            m->m     m->i     m->d     i->m     i->i     d->m     d->d
  COMPO   2.60394  4.47006  2.99097  2.78567  3.15897  2.94590  3.81464
2.76016  2.81821  2.37796  3.67247  2.84642  3.36228  3.09107  3.00327
2.60883  2.76490  2.62695  4.56732  3.28728
          2.68664  4.42176  2.77606  2.73228  3.46304  2.40488  3.72519
3.29244  2.67814  2.69381  4.24348  2.89820  2.73823  3.18254  2.89865
2.37902  2.77498  2.98580  4.58601  3.61558
          0.69704  1.31983  1.44919  3.13201  0.04461  0.00000        *
      1   2.39834  3.36506  3.43893  2.92416  3.55460  3.27843  3.85776
2.78439  2.82479  2.39238  3.52050  2.89440  3.80275  3.17061  3.15121
2.61271  2.17690  2.52485  5.00042  3.75808     67 t - - -
          2.68618  4.42225  2.77519  2.73123  3.46354  2.40513  3.72494
3.29354  2.67741  2.69355  4.24690  2.90347  2.73739  3.18146  2.89801
2.37887  2.77519  2.98518  4.58477  3.61503
          0.03406  3.79240  4.51475  0.61958  0.77255  0.70612  0.68034
      2   1.88166  2.78507  3.79131  3.30624  3.52155  3.26789  4.10645
2.48744  3.18029  2.23301  3.48341  3.48594  3.86229  3.49303  3.44030
2.65678  2.74904  2.26227  5.06909  3.84745     68 a - - -
          2.68618  4.42225  2.77519  2.73123  3.46354  2.40513  3.72494
3.29354  2.67741  2.69355  4.24690  2.90347  2.73739  3.18146  2.89801
2.37887  2.77519  2.98518  4.58477  3.61503
          0.03406  3.79240  4.51475  0.61958  0.77255  0.70612  0.68034
      3   2.94188  4.47786  3.90743  3.43514  2.75555  3.89055  3.94359
2.45491  3.22660  1.33859  3.18321  3.72188  4.25439  3.57201  3.45840
3.24481  3.18705  2.43049  4.48682  2.61106     69 l - - -
          2.68618  4.42225  2.77519  2.73123  3.46354  2.40513  3.72494
3.29354  2.67741  2.69355  4.24690  2.90347  2.73739  3.18146  2.89801
2.37887  2.77519  2.98518  4.58477  3.61503
          0.03406  3.79240  4.51475  0.61958  0.77255  0.70612  0.68034
      4   2.59559  4.81742  2.07098  2.25443  4.24234  3.16932  3.67984
3.66143  2.58084  3.28544  4.12574  2.50475  3.73305  2.85459  3.06455
2.60765  2.21980  3.28822  5.50636  4.12835     70 d - - -
          2.68618  4.42225  2.77519  2.73123  3.46354  2.40513  3.72494
3.29354  2.67741  2.69355  4.24690  2.90347  2.73739  3.18146  2.89801
2.37887  2.77519  2.98518  4.58477  3.61503
          0.03406  3.79240  4.51475  0.61958  0.77255  0.70612  0.68034
      5   2.65215  4.42045  3.25108  3.08030  4.07847  3.11260  4.16273
3.60515  3.13106  3.24002  4.28797  3.39402  0.99098  3.52240  3.38395
2.81764  3.08792  3.25949  5.28519  4.19877     71 p - - -
          2.68618  4.42225  2.77519  2.73123  3.46354  2.40513  3.72494
3.29354  2.67741  2.69355  4.24690  2.90347  2.73739  3.18146  2.89801
2.37887  2.77519  2.98518  4.58477  3.61503
          0.03406  3.79240  4.51475  0.61958  0.77255  0.70612  0.68034
      6   2.64141  4.26721  3.79907  3.34080  3.48771  3.60400  4.22100
1.71451  3.20813  2.22578  3.39611  3.63215  4.10214  3.56892  3.48506
2.98095  2.21465  1.99957  5.16819  3.93126     72 i - - -
          2.68633  4.42240  2.77523  2.73128  3.46369  2.40528  3.72510
3.29352  2.67746  2.69370  4.24705  2.90178  2.73755  3.18162  2.89816
2.37902  2.77535  2.98497  4.58492  3.61490
```

0.35711 1.24008 4.51475 1.33163 0.30659 0.70612 0.68034
7 2.60476 4.35002 3.37487 2.87869 2.89389 3.52234 3.78727
2.27918 2.82243 2.42455 3.43827 2.48393 3.94167 3.15647 3.16914
2.81514 2.86062 2.45024 4.77992 3.39680 80 i - - -
2.68618 4.42225 2.77519 2.73123 3.46354 2.40513 3.72494
3.29354 2.67741 2.69355 4.24690 2.90347 2.73739 3.18146 2.89801
2.37887 2.77519 2.98518 4.58477 3.61503
0.03406 3.79240 4.51475 0.61958 0.77255 0.70612 0.68034
8 2.63836 4.90605 2.47000 2.31461 4.21509 3.31127 3.57775
3.65212 2.29245 3.20907 4.02771 2.83768 3.76752 2.51250 2.21779
2.44370 2.88112 3.29900 5.39352 4.04982 81 r - - -
2.68618 4.42225 2.77519 2.73123 3.46354 2.40513 3.72494
3.29354 2.67741 2.69355 4.24690 2.90347 2.73739 3.18146 2.89801
2.37887 2.77519 2.98518 4.58477 3.61503
0.03406 3.79240 4.51475 0.61958 0.77255 0.70612 0.68034
9 1.91161 4.14429 3.11283 2.93217 4.19083 1.52553 4.09777
3.54267 3.07485 3.29508 4.17966 3.15996 3.62097 3.38077 3.38159
2.38106 2.69424 3.05537 5.52387 4.30828 82 g - - -
2.68618 4.42225 2.77519 2.73123 3.46354 2.40513 3.72494
3.29354 2.67741 2.69355 4.24690 2.90347 2.73739 3.18146 2.89801
2.37887 2.77519 2.98518 4.58477 3.61503
0.03406 3.79240 4.51475 0.61958 0.77255 0.70612 0.68034
10 2.58508 4.72725 1.97074 2.33850 4.04190 2.85962 3.67862
2.91951 2.56880 3.08749 3.95766 2.85518 3.76025 2.86391 3.03525
2.62555 2.86993 3.06837 5.35905 4.00245 83 d - - -
2.68618 4.42225 2.77519 2.73123 3.46354 2.40513 3.72494
3.29354 2.67741 2.69355 4.24690 2.90347 2.73739 3.18146 2.89801
2.37887 2.77519 2.98518 4.58477 3.61503
0.03406 3.79240 4.51475 0.61958 0.77255 0.70612 0.68034
11 2.77283 3.33332 4.15153 3.60490 2.68018 3.85559 4.16855
2.19747 3.47954 1.46991 3.07628 3.84120 4.22487 3.71420 3.68919
3.18120 3.02920 2.12050 4.75357 3.44842 84 l - - -
2.68618 4.42225 2.77519 2.73123 3.46354 2.40513 3.72494
3.29354 2.67741 2.69355 4.24690 2.90347 2.73739 3.18146 2.89801
2.37887 2.77519 2.98518 4.58477 3.61503
0.03406 3.79240 4.51475 0.61958 0.77255 0.70612 0.68034
12 2.58391 4.39569 2.82630 2.72595 3.41392 3.45748 3.75738
2.73969 2.74325 2.55441 3.51581 3.16414 3.89035 3.06740 3.12135
2.76028 2.83874 1.98952 4.90360 3.12326 85 v - - -
2.68618 4.42225 2.77519 2.73123 3.46354 2.40513 3.72494
3.29354 2.67741 2.69355 4.24690 2.90347 2.73739 3.18146 2.89801
2.37887 2.77519 2.98518 4.58477 3.61503
0.03406 3.79240 4.51475 0.61958 0.77255 0.43411 1.04368
13 2.60689 4.40402 3.32266 2.59583 3.58491 3.50285 3.83412
2.81208 2.74147 2.55182 2.29689 3.24775 3.93226 3.11339 3.08873
2.79691 2.22420 2.59986 5.04061 3.79626 86 t - - -
2.68618 4.42225 2.77519 2.73123 3.46354 2.40513 3.72494
3.29354 2.67741 2.69355 4.24690 2.90347 2.73739 3.18146 2.89801
2.37887 2.77519 2.98518 4.58477 3.61503
0.02765 3.99793 4.72027 0.61958 0.77255 0.53854 0.87610
14 2.71436 4.72868 2.90847 2.64169 3.82516 3.34993 3.83240
3.41390 2.66707 2.67109 4.07057 1.43905 3.90912 3.08787 3.02277
2.79388 3.03398 3.12614 5.24043 3.81788 87 n - - -

```
        2.68618  4.42225  2.77519  2.73123  3.46354  2.40513  3.72494
3.29354  2.67741  2.69355  4.24690  2.90347  2.73739  3.18146  2.89801
2.37887  2.77519  2.98518  4.58477  3.61503
        0.02765  3.99793  4.72027  0.61958  0.77255  0.53854  0.87610
     15  2.39203  4.44602  2.95789  2.66548  4.30170  1.91325  3.92588
3.74049  2.82114  3.36400  4.17959  2.46648  3.73173  3.12478  3.23491
2.01170  2.53035  3.26250  5.58406  4.29053     88 g - - -
        2.68618  4.42225  2.77519  2.73123  3.46354  2.40513  3.72494
3.29354  2.67741  2.69355  4.24690  2.90347  2.73739  3.18146  2.89801
2.37887  2.77519  2.98518  4.58477  3.61503
        0.02765  3.99793  4.72027  0.61958  0.77255  0.53854  0.87610
     16  3.07456  4.46991  4.55732  4.05399  3.31561  4.26287  4.72894
1.96668  3.89464  1.34972  3.17261  4.30256  4.59642  4.16078  4.09020
3.63901  3.33999  1.37138  5.26154  4.06407     89 l - - -
        2.68618  4.42225  2.77519  2.73123  3.46354  2.40513  3.72494
3.29354  2.67741  2.69355  4.24690  2.90347  2.73739  3.18146  2.89801
2.37887  2.77519  2.98518  4.58477  3.61503
        0.02765  3.99793  4.72027  0.61958  0.77255  0.53854  0.87610
     17  2.56496  4.31501  3.52650  3.03923  3.54390  3.45844  3.97016
2.72793  2.93366  1.77354  3.49035  3.38406  3.33570  3.29029  3.24712
2.79584  2.12125  2.52908  5.06104  3.82765     90 l - - -
        2.68618  4.42225  2.77519  2.73123  3.46354  2.40513  3.72494
3.29354  2.67741  2.69355  4.24690  2.90347  2.73739  3.18146  2.89801
2.37887  2.77519  2.98518  4.58477  3.61503
        0.02765  3.99793  4.72027  0.61958  0.77255  0.53854  0.87610
     18  2.61052  4.71642  2.73570  2.52419  4.30134  3.20019  3.86405
3.81513  2.72238  3.42309  4.27737  1.78516  2.02155  3.06401  3.13124
2.67074  2.97025  3.39605  5.56512  4.22377     91 n - - -
        2.68618  4.42225  2.77519  2.73123  3.46354  2.40513  3.72494
3.29354  2.67741  2.69355  4.24690  2.90347  2.73739  3.18146  2.89801
2.37887  2.77519  2.98518  4.58477  3.61503
        0.02765  3.99793  4.72027  0.61958  0.77255  0.53854  0.87610
     19  2.50416  4.29991  3.37858  2.89984  3.61581  3.38259  3.88295
2.03652  2.85163  2.66795  3.57384  2.96736  3.87981  3.18690  3.19496
2.14313  2.59925  2.60516  5.05306  3.80858     92 i - - -
        2.68618  4.42225  2.77519  2.73123  3.46354  2.40513  3.72494
3.29354  2.67741  2.69355  4.24690  2.90347  2.73739  3.18146  2.89801
2.37887  2.77519  2.98518  4.58477  3.61503
        0.02765  3.99793  4.72027  0.61958  0.77255  0.53854  0.87610
     20  2.86966  5.34492  1.56821  1.97708  4.69192  2.07697  3.75834
4.18978  2.74724  3.71189  4.55073  2.71862  3.83158  2.92306  3.32012
2.78547  3.16160  3.76988  5.88285  4.41336     93 d - - -
        2.68618  4.42225  2.77519  2.73123  3.46354  2.40513  3.72494
3.29354  2.67741  2.69355  4.24690  2.90347  2.73739  3.18146  2.89801
2.37887  2.77519  2.98518  4.58477  3.61503
        0.02765  3.99793  4.72027  0.61958  0.77255  0.53854  0.87610
     21  2.80933  4.96838  3.00056  2.57874  4.33980  3.44092  3.67783
3.82146  2.20383  3.35044  4.20267  1.97574  3.90994  2.83596  1.71680
2.82563  3.05819  3.46887  5.44069  4.14873     94 r - - -
        2.68618  4.42225  2.77519  2.73123  3.46354  2.40513  3.72494
3.29354  2.67741  2.69355  4.24690  2.90347  2.73739  3.18146  2.89801
2.37887  2.77519  2.98518  4.58477  3.61503
        0.02765  3.99793  4.72027  0.61958  0.77255  0.53854  0.87610
```

```
     22   2.51578  4.56279  2.88941  2.62513  4.20353  3.17746  3.88710
3.59441  2.73418  3.28489  4.14887  1.78629  3.79649  3.09477  3.12009
2.61253  1.92968  3.20044  5.50956  4.19081      95 n - - -
          2.68618  4.42225  2.77519  2.73123  3.46354  2.40513  3.72494
3.29354  2.67741  2.69355  4.24690  2.90347  2.73739  3.18146  2.89801
2.37887  2.77519  2.98518  4.58477  3.61503
          0.02765  3.99793  4.72027  0.61958  0.77255  0.53854  0.87610
     23   2.57142  4.24296  3.56514  3.01066  2.87532  3.55974  3.87893
2.45256  2.94718  2.42351  3.36691  3.05879  3.96349  3.24757  3.27294
2.83296  2.07114  2.27816  4.86669  3.62247      96 t - - -
          2.68618  4.42225  2.77519  2.73123  3.46354  2.40513  3.72494
3.29354  2.67741  2.69355  4.24690  2.90347  2.73739  3.18146  2.89801
2.37887  2.77519  2.98518  4.58477  3.61503
          0.02765  3.99793  4.72027  0.61958  0.77255  0.53854  0.87610
     24   2.73228  4.33643  3.74330  3.25043  3.52556  3.72248  4.10624
1.46302  2.73466  2.37744  3.44750  3.59034  4.14478  3.45563  3.29661
3.05886  2.74042  2.13763  5.10004  3.87562      97 i - - -
          2.68618  4.42225  2.77519  2.73123  3.46354  2.40513  3.72494
3.29354  2.67741  2.69355  4.24690  2.90347  2.73739  3.18146  2.89801
2.37887  2.77519  2.98518  4.58477  3.61503
          0.02765  3.99793  4.72027  0.61958  0.77255  0.53854  0.87610
     25   2.33061  1.20325  3.97435  3.67492  4.05479  2.22577  4.43636
3.35708  3.56755  3.18400  4.10450  3.62285  3.80055  3.86101  3.74621
2.56464  2.82188  2.95205  5.43993  4.30288      98 c - - -
          2.68618  4.42225  2.77519  2.73123  3.46354  2.40513  3.72494
3.29354  2.67741  2.69355  4.24690  2.90347  2.73739  3.18146  2.89801
2.37887  2.77519  2.98518  4.58477  3.61503
          0.02765  3.99793  4.72027  0.61958  0.77255  0.53854  0.87610
     26   2.74442  5.11544  1.72207  2.22947  4.41374  3.26583  3.21907
3.93903  2.60040  3.48214  4.30230  2.78534  3.81070  2.86030  3.11596
2.01206  3.02138  3.54443  5.63981  4.20919      99 d - - -
          2.68631  4.42238  2.77510  2.73136  3.46262  2.40505  3.72508
3.29317  2.67754  2.69368  4.24655  2.90325  2.73753  3.18159  2.89814
2.37893  2.77508  2.98531  4.58490  3.61516
          0.28039  1.44561  4.72027  1.19812  0.35920  0.53854  0.87610
     27   1.46315  4.25600  3.33720  3.04145  4.18984  3.03750  4.14205
3.50594  3.08323  3.24554  4.13353  3.27957  1.90486  3.40683  3.40762
2.49306  2.77761  3.06901  5.54964  4.32292     104 a - - -
          2.68618  4.42225  2.77519  2.73123  3.46354  2.40513  3.72494
3.29354  2.67741  2.69355  4.24690  2.90347  2.73739  3.18146  2.89801
2.37887  2.77519  2.98518  4.58477  3.61503
          0.02765  3.99793  4.72027  0.61958  0.77255  0.53854  0.87610
     28   2.64577  4.35846  3.49634  2.93043  3.19278  3.59301  3.33964
2.32212  2.75142  2.53643  3.45732  3.31325  3.96962  3.13873  2.66089
2.85526  2.87373  2.63739  4.68787  2.37841     105 i - - -
          2.68618  4.42225  2.77519  2.73123  3.46354  2.40513  3.72494
3.29354  2.67741  2.69355  4.24690  2.90347  2.73739  3.18146  2.89801
2.37887  2.77519  2.98518  4.58477  3.61503
          0.02765  3.99793  4.72027  0.61958  0.77255  0.53854  0.87610
     29   2.83139  4.82958  2.78429  2.68318  4.21318  3.27125  4.02828
3.95211  2.94333  3.58515  4.54076  0.95375  3.92992  3.30245  3.30121
2.89455  3.21813  3.56157  5.50482  4.15344     106 n - - -
          2.68618  4.42225  2.77519  2.73123  3.46354  2.40513  3.72494
3.29354  2.67741  2.69355  4.24690  2.90347  2.73739  3.18146  2.89801
2.37887  2.77519  2.98518  4.58477  3.61503
```

0.02765 3.99793 4.72027 0.61958 0.77255 0.53854 0.87610
30 2.43479 4.29851 4.15384 3.77932 3.71463 3.67664 4.61704
2.06529 3.67677 2.40126 3.59798 3.93884 4.25928 4.00594 3.90810
3.11503 3.08364 1.05014 5.46920 4.23575 107 v - - -
2.68618 4.42225 2.77519 2.73123 3.46354 2.40513 3.72494
3.29354 2.67741 2.69355 4.24690 2.90347 2.73739 3.18146 2.89801
2.37887 2.77519 2.98518 4.58477 3.61503
0.02765 3.99793 4.72027 0.61958 0.77255 0.53854 0.87610
31 2.32773 4.29413 3.48338 2.92888 3.49324 3.53142 3.86037
2.65648 2.65859 2.50863 3.07145 3.32012 3.94305 3.17988 3.19224
2.80278 2.36057 2.02822 4.95657 3.72336 108 v - - -
2.68618 4.42225 2.77519 2.73123 3.46354 2.40513 3.72494
3.29354 2.67741 2.69355 4.24690 2.90347 2.73739 3.18146 2.89801
2.37887 2.77519 2.98518 4.58477 3.61503
0.02765 3.99793 4.72027 0.61958 0.77255 0.53854 0.87610
32 1.73718 4.58481 3.27136 2.79166 4.12501 3.32722 3.81169
3.46612 2.42982 3.12037 4.00929 3.17690 3.86441 3.00662 1.82454
2.70600 2.91917 3.13076 5.37087 4.12254 109 a - - -
2.68618 4.42225 2.77519 2.73123 3.46354 2.40513 3.72494
3.29354 2.67741 2.69355 4.24690 2.90347 2.73739 3.18146 2.89801
2.37887 2.77519 2.98518 4.58477 3.61503
0.02765 3.99793 4.72027 0.61958 0.77255 0.53854 0.87610
33 2.76853 4.94095 3.00751 2.52864 4.21439 3.46781 2.24807
3.66762 1.78401 3.21973 4.06176 3.01222 3.89007 2.78611 2.51373
2.78532 2.69724 3.34132 5.36367 4.06083 110 k - - -
2.68618 4.42225 2.77519 2.73123 3.46354 2.40513 3.72494
3.29354 2.67741 2.69355 4.24690 2.90347 2.73739 3.18146 2.89801
2.37887 2.77519 2.98518 4.58477 3.61503
0.02765 3.99793 4.72027 0.61958 0.77255 0.53854 0.87610
34 2.93985 5.38448 1.10453 2.15042 4.70618 3.20814 3.82441
4.28793 2.87994 3.82885 4.70914 2.41350 3.85776 3.01245 3.46697
2.84864 3.25875 3.86153 5.93638 4.44116 111 d - - -
2.68618 4.42225 2.77519 2.73123 3.46354 2.40513 3.72494
3.29354 2.67741 2.69355 4.24690 2.90347 2.73739 3.18146 2.89801
2.37887 2.77519 2.98518 4.58477 3.61503
0.02765 3.99793 4.72027 0.61958 0.77255 0.53854 0.87610
35 2.92532 4.66031 3.54630 3.40447 4.38658 3.34079 4.47051
3.96561 3.47276 3.57831 4.63444 3.69789 0.62421 3.85773 3.70295
3.09705 3.37704 3.59646 5.53742 4.50940 112 P - - -
2.68618 4.42225 2.77519 2.73123 3.46354 2.40513 3.72494
3.29354 2.67741 2.69355 4.24690 2.90347 2.73739 3.18146 2.89801
2.37887 2.77519 2.98518 4.58477 3.61503
0.02765 3.99793 4.72027 0.61958 0.77255 0.53854 0.87610
36 3.42143 4.78689 4.41072 4.15034 0.82680 4.07918 3.93811
2.93113 4.05455 2.27292 3.64360 4.16226 4.53595 4.18829 4.14323
3.68403 3.72313 2.93081 4.15245 2.50475 113 f - - -
2.68625 4.42232 2.77526 2.73130 3.46249 2.40520 3.72501
3.29361 2.67748 2.69362 4.24697 2.90354 2.73746 3.18153 2.89808
2.37856 2.77526 2.98525 4.58484 3.61510
0.29924 1.38748 4.72027 0.67115 0.71564 0.53854 0.87610
37 2.82898 5.11921 2.94933 2.07175 4.47820 3.48133 3.60256
3.88793 2.11206 3.37446 4.19704 2.97523 3.89501 2.26660 1.80407
2.80604 3.03767 3.53100 5.48802 4.19929 116 r - - -

```
        2.68618  4.42225  2.77519  2.73123  3.46354  2.40513  3.72494
3.29354  2.67741  2.69355  4.24690  2.90347  2.73739  3.18146  2.89801
2.37887  2.77519  2.98518  4.58477  3.61503
        0.02765  3.99793  4.72027  0.61958  0.77255  0.53854  0.87610
     38  2.78501  4.98023  2.95575  2.50856  4.23267  3.45878  2.22591
3.73024  1.76125  3.26758  4.10676  2.99693  3.89110  2.78729  2.51900
2.57044  3.01110  3.39475  5.37914  4.05962    117 k - - -
        2.68618  4.42225  2.77519  2.73123  3.46354  2.40513  3.72494
3.29354  2.67741  2.69355  4.24690  2.90347  2.73739  3.18146  2.89801
2.37887  2.77519  2.98518  4.58477  3.61503
        0.02765  3.99793  4.72027  0.61958  0.77255  0.53854  0.87610
     39  3.05830  5.16749  3.46918  2.80280  4.62409  3.66882  3.61368
3.97133  1.28442  3.41173  4.28434  3.20269  4.03815  2.74893  1.62130
3.05351  3.22419  3.65373  5.45073  4.28573    118 k - - -
        2.68618  4.42225  2.77519  2.73123  3.46354  2.40513  3.72494
3.29354  2.67741  2.69355  4.24690  2.90347  2.73739  3.18146  2.89801
2.37887  2.77519  2.98518  4.58477  3.61503
        0.02765  3.99793  4.72027  0.61958  0.77255  0.53854  0.87610
     40  2.26996  4.21237  3.37537  3.06444  4.20308  2.99726  4.14227
3.55561  3.07862  3.29163  4.14911  3.27313  3.71752  3.40639  3.39690
1.41311  1.84809  3.08277  5.56093  4.32383    119 s - - -
        2.68618  4.42225  2.77519  2.73123  3.46354  2.40513  3.72494
3.29354  2.67741  2.69355  4.24690  2.90347  2.73739  3.18146  2.89801
2.37887  2.77519  2.98518  4.58477  3.61503
        0.02765  3.99793  4.72027  0.61958  0.77255  0.53854  0.87610
     41  2.70614  4.37274  3.78917  3.32647  3.45970  3.62380  4.18824
2.46522  3.16824  1.56386  3.39906  3.62780  4.11897  3.53975  3.43932
2.99775  1.95139  2.33583  5.12917  3.89265    120 l - - -
        2.68618  4.42225  2.77519  2.73123  3.46354  2.40513  3.72494
3.29354  2.67741  2.69355  4.24690  2.90347  2.73739  3.18146  2.89801
2.37887  2.77519  2.98518  4.58477  3.61503
        0.02765  3.99793  4.72027  0.61958  0.77255  0.53854  0.87610
     42  2.82612  5.31232  1.73927  2.16858  4.60427  3.26600  3.69892
4.11409  2.59231  3.62010  4.43861  2.51708  3.82306  1.92551  3.11512
2.74910  3.09391  3.70244  5.77133  4.31833    121 d - - -
        2.68618  4.42225  2.77519  2.73123  3.46354  2.40513  3.72494
3.29354  2.67741  2.69355  4.24690  2.90347  2.73739  3.18146  2.89801
2.37887  2.77519  2.98518  4.58477  3.61503
        0.02765  3.99793  4.72027  0.61958  0.77255  0.53854  0.87610
     43  2.68540  4.84129  2.68010  1.73253  4.25077  3.30059  3.76575
3.57113  2.58703  3.24921  4.12321  2.93402  3.84120  2.94280  3.01127
2.71879  1.93237  3.23910  5.51715  4.17906    122 e - - -
        2.68618  4.42225  2.77519  2.73123  3.46354  2.40513  3.72494
3.29354  2.67741  2.69355  4.24690  2.90347  2.73739  3.18146  2.89801
2.37887  2.77519  2.98518  4.58477  3.61503
        0.02765  3.99793  4.72027  0.61958  0.77255  0.53854  0.87610
     44  3.05745  4.39661  4.70679  4.14770  2.36771  4.31864  4.63023
1.52513  4.03043  1.61388  3.05067  4.34612  4.58041  4.17992  4.16554
3.65223  3.29029  1.75771  5.04970  3.85289    123 i - - -
        2.68618  4.42225  2.77519  2.73123  3.46354  2.40513  3.72494
3.29354  2.67741  2.69355  4.24690  2.90347  2.73739  3.18146  2.89801
2.37887  2.77519  2.98518  4.58477  3.61503
        0.02765  3.99793  4.72027  0.61958  0.77255  0.53854  0.87610
```

45 2.59570 4.52237 3.18570 2.50448 3.69528 3.48357 3.70621
2.52427 2.55441 2.70540 3.26560 3.10534 3.87214 2.28703 2.94613
2.72126 2.63291 2.77069 5.07648 3.80579 124 q - - -
2.68618 4.42225 2.77519 2.73123 3.46354 2.40513 3.72494
3.29354 2.67741 2.69355 4.24690 2.90347 2.73739 3.18146 2.89801
2.37887 2.77519 2.98518 4.58477 3.61503
0.02765 3.99793 4.72027 0.61958 0.77255 0.53854 0.87610
46 3.02913 5.18789 3.41695 2.74746 4.63804 3.65993 3.59099
3.98203 1.47328 3.41258 4.26852 3.16351 4.01774 2.47025 1.54942
3.01436 3.19076 3.65555 5.45649 4.27906 125 k - - -
2.68618 4.42225 2.77519 2.73123 3.46354 2.40513 3.72494
3.29354 2.67741 2.69355 4.24690 2.90347 2.73739 3.18146 2.89801
2.37887 2.77519 2.98518 4.58477 3.61503
0.02765 3.99793 4.72027 0.61958 0.77255 0.53854 0.87610
47 3.06454 5.19687 2.54665 0.85936 4.55950 3.35806 3.95765
4.03851 2.85429 3.63614 4.60617 3.01017 3.96907 3.17167 3.27500
3.03510 3.37024 3.70998 5.70746 4.43156 126 e - - -
2.68618 4.42225 2.77519 2.73123 3.46354 2.40513 3.72494
3.29354 2.67741 2.69355 4.24690 2.90347 2.73739 3.18146 2.89801
2.37887 2.77519 2.98518 4.58477 3.61503
0.02765 3.99793 4.72027 0.61958 0.77255 0.53854 0.87610
48 3.55149 4.88026 4.28783 4.02590 2.80470 3.87473 4.06200
3.58182 3.74609 2.95445 4.20960 4.14994 4.40324 4.12722 3.85192
3.75394 3.85855 3.47561 0.66625 2.80047 127 W - - -
2.68618 4.42225 2.77519 2.73123 3.46354 2.40513 3.72494
3.29354 2.67741 2.69355 4.24690 2.90347 2.73739 3.18146 2.89801
2.37887 2.77519 2.98518 4.58477 3.61503
0.02765 3.99793 4.72027 0.61958 0.77255 0.53854 0.87610
49 2.59161 4.42622 3.34508 2.82670 3.66548 3.46561 3.82376
2.89573 2.49535 2.64445 2.92696 3.23630 3.91400 3.08393 2.99815
2.76670 1.81579 2.66568 5.09057 3.84246 128 t - - -
2.68618 4.42225 2.77519 2.73123 3.46354 2.40513 3.72494
3.29354 2.67741 2.69355 4.24690 2.90347 2.73739 3.18146 2.89801
2.37887 2.77519 2.98518 4.58477 3.61503
0.02765 3.99793 4.72027 0.61958 0.77255 0.53854 0.87610
50 3.06454 5.19687 2.54665 0.85936 4.55950 3.35806 3.95765
4.03851 2.85429 3.63614 4.60617 3.01017 3.96907 3.17167 3.27500
3.03510 3.37024 3.70998 5.70746 4.43156 129 e - - -
2.68618 4.42225 2.77519 2.73123 3.46354 2.40513 3.72494
3.29354 2.67741 2.69355 4.24690 2.90347 2.73739 3.18146 2.89801
2.37887 2.77519 2.98518 4.58477 3.61503
0.02765 3.99793 4.72027 0.61958 0.77255 0.53854 0.87610
51 3.55149 4.88026 4.28783 4.02590 2.80470 3.87473 4.06200
3.58182 3.74609 2.95445 4.20960 4.14994 4.40324 4.12722 3.85192
3.75394 3.85855 3.47561 0.66625 2.80047 130 W - - -
2.68618 4.42225 2.77519 2.73123 3.46354 2.40513 3.72494
3.29354 2.67741 2.69355 4.24690 2.90347 2.73739 3.18146 2.89801
2.37887 2.77519 2.98518 4.58477 3.61503
0.02765 3.99793 4.72027 0.61958 0.77255 0.53854 0.87610
52 3.08987 5.05542 3.27400 2.87388 4.47057 3.56418 3.83370
3.90807 0.87871 3.44655 4.40268 3.29229 4.05684 3.01910 2.46228
3.12448 3.33454 3.60930 5.47479 4.30625 131 k - - -
2.68618 4.42225 2.77519 2.73123 3.46354 2.40513 3.72494
3.29354 2.67741 2.69355 4.24690 2.90347 2.73739 3.18146 2.89801
2.37887 2.77519 2.98518 4.58477 3.61503

```
          0.02765  3.99793  4.72027  0.61958  0.77255  0.53854  0.87610
     53   2.82871  5.13808  2.93321  1.82960  4.50768  3.47677  3.59981
3.91448  1.83514  3.39596  4.21352  2.96612  3.89137  2.72694  2.03555
2.80111  3.03741  3.55095  5.50373  4.21207    132 e - - -
          2.68618  4.42225  2.77519  2.73123  3.46354  2.40513  3.72494
3.29354  2.67741  2.69355  4.24690  2.90347  2.73739  3.18146  2.89801
2.37887  2.77519  2.98518  4.58477  3.61503
          0.02765  3.99793  4.72027  0.61958  0.77255  0.53854  0.87610
     54   2.66983  4.86710  2.78872  2.43285  4.26694  3.33590  3.68848
3.67688  2.13942  3.26737  4.09786  1.95752  3.82940  2.84662  2.79967
2.69249  2.34847  3.31923  5.46605  4.13758    133 n - - -
          2.68618  4.42225  2.77519  2.73123  3.46354  2.40513  3.72494
3.29354  2.67741  2.69355  4.24690  2.90347  2.73739  3.18146  2.89801
2.37887  2.77519  2.98518  4.58477  3.61503
          0.02765  3.99793  4.72027  0.61958  0.77255  0.53854  0.87610
     55   2.72219  5.08084  1.97440  2.23360  4.50668  3.23091  3.73035
3.99542  2.64493  3.53920  4.35530  2.23920  3.80092  2.88760  3.16902
1.85934  3.01965  3.57549  5.71503  4.29027    134 s - - -
          2.68618  4.42225  2.77519  2.73123  3.46354  2.40513  3.72494
3.29354  2.67741  2.69355  4.24690  2.90347  2.73739  3.18146  2.89801
2.37887  2.77519  2.98518  4.58477  3.61503
          0.02765  3.99793  4.72027  0.61958  0.77255  0.53854  0.87610
     56   2.25847  4.63311  2.95251  2.53150  4.02446  3.29972  2.29013
3.46742  2.54038  3.09058  3.92711  3.00250  2.91672  2.91279  2.95214
2.44168  2.84275  3.12309  5.32112  4.00230    135 a - - -
          2.68618  4.42225  2.77519  2.73123  3.46354  2.40513  3.72494
3.29354  2.67741  2.69355  4.24690  2.90347  2.73739  3.18146  2.89801
2.37887  2.77519  2.98518  4.58477  3.61503
          0.02765  3.99793  4.72027  0.61958  0.77255  0.53854  0.87610
     57   2.38041  4.30170  3.31938  3.14242  4.20312  3.02945  4.24809
3.74339  3.22467  3.45444  4.37097  3.34608  3.77891  3.56443  3.50584
0.91648  2.87020  3.25089  5.56584  4.28769    136 s - - -
          2.68618  4.42225  2.77519  2.73123  3.46354  2.40513  3.72494
3.29354  2.67741  2.69355  4.24690  2.90347  2.73739  3.18146  2.89801
2.37887  2.77519  2.98518  4.58477  3.61503
          0.02765  3.99793  4.72027  0.61958  0.77255  0.53854  0.87610
     58   3.29808  4.72398  4.39047  4.03234  3.18722  4.14052  4.64880
2.39966  3.79884  0.73488  3.17359  4.29559  4.55524  4.13708  3.95380
3.72815  3.58715  2.46929  5.11054  3.86235    137 l - - -
          2.68618  4.42225  2.77519  2.73123  3.46354  2.40513  3.72494
3.29354  2.67741  2.69355  4.24690  2.90347  2.73739  3.18146  2.89801
2.37887  2.77519  2.98518  4.58477  3.61503
          0.02765  3.99793  4.72027  0.61958  0.77255  0.53854  0.87610
     59   3.59231  4.84577  4.57821  4.25365  1.27300  4.35335  3.46933
3.28403  4.10359  2.62319  3.88488  4.04170  4.66564  4.09002  4.15103
3.72002  3.81563  3.20841  3.61620  1.21745    138 y - - -
          2.68618  4.42225  2.77519  2.73123  3.46354  2.40513  3.72494
3.29354  2.67741  2.69355  4.24690  2.90347  2.73739  3.18146  2.89801
2.37887  2.77519  2.98518  4.58477  3.61503
          0.02765  3.99793  4.72027  0.61958  0.77255  0.53854  0.87610
     60   2.79878  4.29527  4.10010  3.55090  3.44292  3.92377  4.33308
1.93870  3.45183  1.96641  3.32742  3.85461  4.29863  3.74020  3.72058
3.23590  2.20527  1.57942  5.13382  3.92945    139 v - - -
```

```
          2.68618  4.42225  2.77519  2.73123  3.46354  2.40513  3.72494
3.29354  2.67741  2.69355  4.24690  2.90347  2.73739  3.18146  2.89801
2.37887  2.77519  2.98518  4.58477  3.61503
          0.02765  3.99793  4.72027  0.61958  0.77255  0.53854  0.87610
     61   1.90788  4.69229  2.32520  2.44477  4.20885  3.23611  3.73179
3.63084  2.58451  3.23747  4.04951  2.93459  3.20744  2.89453  3.05090
2.38774  2.60960  3.23948  5.46692  4.12803    140 a - - -
          2.68618  4.42225  2.77519  2.73123  3.46354  2.40513  3.72494
3.29354  2.67741  2.69355  4.24690  2.90347  2.73739  3.18146  2.89801
2.37887  2.77519  2.98518  4.58477  3.61503
          0.02765  3.99793  4.72027  0.61958  0.77255  0.53854  0.87610
     62   2.09387  4.32735  3.26342  3.04077  4.22603  3.07177  4.17926
3.55144  3.10448  3.28353  4.21852  3.29902  1.27362  3.45427  3.41064
2.56782  2.86193  3.13097  5.58173  4.35386    141 p - - -
          2.68618  4.42225  2.77519  2.73123  3.46354  2.40513  3.72494
3.29354  2.67741  2.69355  4.24690  2.90347  2.73739  3.18146  2.89801
2.37887  2.77519  2.98518  4.58477  3.61503
          0.02765  3.99793  4.72027  0.61958  0.77255  0.53854  0.87610
     63   3.09442  4.45389  4.68083  4.21602  3.47436  4.34014  4.92955
1.04254  4.06483  1.97126  3.31298  4.43863  4.69385  4.35528  4.26101
3.74772  3.37488  1.62015  5.45320  4.23197    142 i - - -
          2.68618  4.42225  2.77519  2.73123  3.46354  2.40513  3.72494
3.29354  2.67741  2.69355  4.24690  2.90347  2.73739  3.18146  2.89801
2.37887  2.77519  2.98518  4.58477  3.61503
          0.02765  3.99793  4.72027  0.61958  0.77255  0.53854  0.87610
     64   2.90782  4.41731  4.29764  3.96605  3.64426  3.85212  4.74687
2.05149  3.83573  2.31043  3.59174  4.13534  4.40666  4.18714  4.03593
3.36465  3.26697  0.88040  5.44942  4.18821    143 v - - -
          2.68618  4.42225  2.77519  2.73123  3.46354  2.40513  3.72494
3.29354  2.67741  2.69355  4.24690  2.90347  2.73739  3.18146  2.89801
2.37887  2.77519  2.98518  4.58477  3.61503
          0.02765  3.99793  4.72027  0.61958  0.77255  0.53854  0.87610
     65   2.82762  4.58920  3.52696  3.44302  4.58034  0.55934  4.55436
4.21170  3.64127  3.84973  4.82747  3.69145  3.98430  3.95562  3.86231
3.00335  3.31863  3.71582  5.64106  4.68693    144 G - - -
          2.68618  4.42225  2.77519  2.73123  3.46354  2.40513  3.72494
3.29354  2.67741  2.69355  4.24690  2.90347  2.73739  3.18146  2.89801
2.37887  2.77519  2.98518  4.58477  3.61503
          0.02765  3.99793  4.72027  0.61958  0.77255  0.53854  0.87610
     66   2.56162  4.25275  3.87420  3.44066  3.70769  3.46456  4.31952
2.35397  3.33864  2.52330  3.61819  3.65373  4.05429  3.67166  3.61042
2.86836  1.83679  1.53339  5.32152  4.09677    145 v - - -
          2.68618  4.42225  2.77519  2.73123  3.46354  2.40513  3.72494
3.29354  2.67741  2.69355  4.24690  2.90347  2.73739  3.18146  2.89801
2.37887  2.77519  2.98518  4.58477  3.61503
          0.02765  3.99793  4.72027  0.61958  0.77255  0.53854  0.87610
     67   3.06888  4.40576  4.72598  4.23522  3.56311  4.37789  4.96508
1.47095  4.11773  2.08109  3.37950  4.45935  4.71292  4.39538  4.32556
3.76716  3.34102  1.06997  5.51384  4.29924    146 v - - -
          2.68618  4.42225  2.77519  2.73123  3.46354  2.40513  3.72494
3.29354  2.67741  2.69355  4.24690  2.90347  2.73739  3.18146  2.89801
2.37887  2.77519  2.98518  4.58477  3.61503
          0.02765  3.99793  4.72027  0.61958  0.77255  0.53854  0.87610
```

```
     68   1.77163  4.17855  3.43264  3.06604  4.17240  2.39832  4.12227
3.55395  3.08974  3.25405  4.08529  3.26901  3.69757  3.38400  3.42620
2.22439  1.70150  3.07099  5.52399  4.30462    147 t - - -
          2.68618  4.42225  2.77519  2.73123  3.46354  2.40513  3.72494
3.29354  2.67741  2.69355  4.24690  2.90347  2.73739  3.18146  2.89801
2.37887  2.77519  2.98518  4.58477  3.61503
          0.02765  3.99793  4.72027  0.61958  0.77255  0.53854  0.87610
     69   2.38041  4.30170  3.31938  3.14242  4.20312  3.02945  4.24809
3.74339  3.22467  3.45444  4.37097  3.34608  3.77891  3.56443  3.50584
0.91648  2.87020  3.25089  5.56584  4.28769    148 s - - -
          2.68618  4.42225  2.77519  2.73123  3.46354  2.40513  3.72494
3.29354  2.67741  2.69355  4.24690  2.90347  2.73739  3.18146  2.89801
2.37887  2.77519  2.98518  4.58477  3.61503
          0.02765  3.99793  4.72027  0.61958  0.77255  0.53854  0.87610
     70   2.70282  4.32794  3.86125  3.41318  1.75575  3.61708  4.02771
2.61525  3.32790  2.27558  3.41593  3.64712  4.11139  3.60463  3.57683
2.98903  2.09450  2.47163  4.68621  3.24400    149 f - - -
          2.68618  4.42225  2.77519  2.73123  3.46354  2.40513  3.72494
3.29354  2.67741  2.69355  4.24690  2.90347  2.73739  3.18146  2.89801
2.37887  2.77519  2.98518  4.58477  3.61503
          0.02765  3.99793  4.72027  0.61958  0.77255  0.53854  0.87610
     71   3.29808  4.72398  4.39047  4.03234  3.18722  4.14052  4.64880
2.39966  3.79884  0.73488  3.17359  4.29559  4.55524  4.13708  3.95380
3.72815  3.58715  2.46929  5.11054  3.86235    150 l - - -
          2.68618  4.42225  2.77519  2.73123  3.46354  2.40513  3.72494
3.29354  2.67741  2.69355  4.24690  2.90347  2.73739  3.18146  2.89801
2.37887  2.77519  2.98518  4.58477  3.61503
          0.02765  3.99793  4.72027  0.61958  0.77255  0.53854  0.87610
     72   3.29808  4.72398  4.39047  4.03234  3.18722  4.14052  4.64880
2.39966  3.79884  0.73488  3.17359  4.29559  4.55524  4.13708  3.95380
3.72815  3.58715  2.46929  5.11054  3.86235    151 l - - -
          2.68618  4.42225  2.77519  2.73123  3.46354  2.40513  3.72494
3.29354  2.67741  2.69355  4.24690  2.90347  2.73739  3.18146  2.89801
2.37887  2.77519  2.98518  4.58477  3.61503
          0.02765  3.99793  4.72027  0.61958  0.77255  0.53854  0.87610
     73   2.80302  5.18729  2.69803  1.97480  4.53188  3.39980  3.61827
3.96900  1.67804  3.45450  4.26242  2.64015  3.85565  2.50556  2.61089
2.75831  3.02884  3.58611  5.57770  4.23363    152 k - - -
          2.68618  4.42225  2.77519  2.73123  3.46354  2.40513  3.72494
3.29354  2.67741  2.69355  4.24690  2.90347  2.73739  3.18146  2.89801
2.37887  2.77519  2.98518  4.58477  3.61503
          0.02765  3.99793  4.72027  0.61958  0.77255  0.53854  0.87610
     74   1.98697  4.69035  3.00412  2.57889  4.20448  3.30931  3.73601
3.58006  1.74298  3.20375  4.04264  3.03222  3.82384  2.90578  2.77358
2.42120  2.89314  3.21814  5.42152  4.13194    153 k - - -
          2.68618  4.42225  2.77519  2.73123  3.46354  2.40513  3.72494
3.29354  2.67741  2.69355  4.24690  2.90347  2.73739  3.18146  2.89801
2.37887  2.77519  2.98518  4.58477  3.61503
          0.02765  3.99793  4.72027  0.61958  0.77255  0.53854  0.87610
     75   3.12413  4.46518  4.70721  4.16765  3.31415  4.41185  4.82213
1.77688  4.04117  1.33194  3.12775  4.42223  4.67624  4.25428  4.22448
3.76409  3.36839  1.42597  5.29493  4.14460    154 l - - -
          2.68618  4.42225  2.77519  2.73123  3.46354  2.40513  3.72494
3.29354  2.67741  2.69355  4.24690  2.90347  2.73739  3.18146  2.89801
2.37887  2.77519  2.98518  4.58477  3.61503
```

```
          0.02765  3.99793  4.72027  0.61958  0.77255  0.53854  0.87610
     76   2.39787  4.75134  2.90242  2.53461  4.29536  2.20074  3.74203
3.69315  1.74985  3.29053  4.12465  3.00092  3.82433  2.90755  2.79128
2.67141  2.92486  3.31229  5.48617  4.18773    155 k - - -
          2.68618  4.42225  2.77519  2.73123  3.46354  2.40513  3.72494
3.29354  2.67741  2.69355  4.24690  2.90347  2.73739  3.18146  2.89801
2.37887  2.77519  2.98518  4.58477  3.61503
          0.02765  3.99793  4.72027  0.61958  0.77255  0.53854  0.87610
     77   2.67196  4.91009  2.83721  2.42251  4.29105  3.00701  3.64792
3.71667  1.87281  3.27190  4.08744  2.92989  3.82542  2.58641  2.71863
2.04914  2.92569  3.35285  5.45404  4.12754    156 k - - -
          2.68618  4.42225  2.77519  2.73123  3.46354  2.40513  3.72494
3.29354  2.67741  2.69355  4.24690  2.90347  2.73739  3.18146  2.89801
2.37887  2.77519  2.98518  4.58477  3.61503
          0.02765  3.99793  4.72027  0.61958  0.77255  0.53854  0.87610
     78   2.81308  4.48546  3.66958  3.15030  3.33489  3.76069  4.02824
2.58120  2.93496  1.41959  3.26919  3.53555  4.14195  2.66012  3.22024
3.06970  3.05595  2.33510  5.00654  3.76813    157 l - - -
          2.68618  4.42225  2.77519  2.73123  3.46354  2.40513  3.72494
3.29354  2.67741  2.69355  4.24690  2.90347  2.73739  3.18146  2.89801
2.37887  2.77519  2.98518  4.58477  3.61503
          0.02765  3.99793  4.72027  0.61958  0.77255  0.53854  0.87610
     79   2.42294  4.20632  3.98428  3.46162  3.48246  3.21220  4.23079
1.85460  3.37264  2.37070  3.40858  3.72344  4.15262  3.65766  3.63409
3.03719  2.93973  1.49872  5.07524  3.86303    158 v - - -
          2.68618  4.42225  2.77519  2.73123  3.46354  2.40513  3.72494
3.29354  2.67741  2.69355  4.24690  2.90347  2.73739  3.18146  2.89801
2.37887  2.77519  2.98518  4.58477  3.61503
          0.02765  3.99793  4.72027  0.61958  0.77255  0.53854  0.87610
     80   2.16666  4.54458  2.96345  2.59553  4.14853  2.02939  3.81255
3.55044  2.64708  3.18581  4.01951  3.03213  3.77473  2.47460  3.05291
2.57508  2.53760  3.16255  5.43505  4.13885    159 g - - -
          2.68618  4.42225  2.77519  2.73123  3.46354  2.40513  3.72494
3.29354  2.67741  2.69355  4.24690  2.90347  2.73739  3.18146  2.89801
2.37887  2.77519  2.98518  4.58477  3.61503
          0.02765  3.99793  4.72027  0.61958  0.77255  0.53854  0.87610
     81   2.63715  4.80703  2.88705  2.50243  4.32398  2.71331  3.71014
3.74668  1.62854  3.31463  4.13830  2.97932  3.82437  2.86662  2.74275
2.25399  2.92931  3.35977  5.48994  4.18240    160 k - - -
          2.68618  4.42225  2.77519  2.73123  3.46354  2.40513  3.72494
3.29354  2.67741  2.69355  4.24690  2.90347  2.73739  3.18146  2.89801
2.37887  2.77519  2.98518  4.58477  3.61503
          0.02765  3.99793  4.72027  0.61958  0.77255  0.53854  0.87610
     82   2.83313  4.72277  3.44778  2.89458  2.95710  3.59013  3.75348
3.25401  2.42089  2.79968  3.82569  3.29409  4.01711  3.03634  1.39693
2.94305  3.07810  3.04597  4.96871  3.56409    161 r - - -
          2.68618  4.42225  2.77519  2.73123  3.46354  2.40513  3.72494
3.29354  2.67741  2.69355  4.24690  2.90347  2.73739  3.18146  2.89801
2.37887  2.77519  2.98518  4.58477  3.61503
          0.02765  3.99793  4.72027  0.61958  0.77255  0.53854  0.87610
     83   3.15210  4.46286  4.81375  4.27775  3.35212  4.49080  4.92121
1.34972  4.15777  1.48516  3.15261  4.51936  4.73790  4.35932  4.32906
3.85097  3.39548  1.57948  5.35597  4.21063    162 i - - -
```

2.68618 4.42225 2.77519 2.73123 3.46354 2.40513 3.72494
3.29354 2.67741 2.69355 4.24690 2.90347 2.73739 3.18146 2.89801
2.37887 2.77519 2.98518 4.58477 3.61503
0.02765 3.99793 4.72027 0.61958 0.77255 0.53854 0.87610
84 3.29808 4.72398 4.39047 4.03234 3.18722 4.14052 4.64880
2.39966 3.79884 0.73488 3.17359 4.29559 4.55524 4.13708 3.95380
3.72815 3.58715 2.46929 5.11054 3.86235 163 l - - -
2.68618 4.42225 2.77519 2.73123 3.46354 2.40513 3.72494
3.29354 2.67741 2.69355 4.24690 2.90347 2.73739 3.18146 2.89801
2.37887 2.77519 2.98518 4.58477 3.61503
0.02765 3.99793 4.72027 0.61958 0.77255 0.53854 0.87610
85 2.56605 4.46085 3.23362 2.68530 3.67146 3.45412 3.72991
3.01140 2.43062 2.54568 3.02679 3.13423 3.86540 2.96989 2.97551
2.05484 2.63767 2.75720 5.05595 3.79210 164 s - - -
2.68618 4.42225 2.77519 2.73123 3.46354 2.40513 3.72494
3.29354 2.67741 2.69355 4.24690 2.90347 2.73739 3.18146 2.89801
2.37887 2.77519 2.98518 4.58477 3.61503
0.02765 3.99793 4.72027 0.61958 0.77255 0.53854 0.87610
86 2.61595 4.74117 2.87627 1.87084 4.02356 3.37189 3.67889
3.38444 2.48684 2.60774 3.88845 2.95987 3.82314 2.85222 2.91696
2.37896 2.63792 3.07692 5.31491 3.99276 165 e - - -
2.68618 4.42225 2.77519 2.73123 3.46354 2.40513 3.72494
3.29354 2.67741 2.69355 4.24690 2.90347 2.73739 3.18146 2.89801
2.37887 2.77519 2.98518 4.58477 3.61503
0.02765 3.99793 4.72027 0.61958 0.77255 0.45412 1.00788
87 3.33343 4.70014 4.75567 4.21282 3.07153 4.51244 4.80019
2.22787 4.00904 0.86036 2.29869 4.49772 4.72110 4.19770 4.15495
3.87551 3.56161 2.36593 5.15413 4.04889 166 l - - -
2.68618 4.42225 2.77519 2.73123 3.46354 2.40513 3.72494
3.29354 2.67741 2.69355 4.24690 2.90347 2.73739 3.18146 2.89801
2.37887 2.77519 2.98518 4.58477 3.61503
0.02625 4.04896 4.77131 0.61958 0.77255 0.48576 0.95510
88 2.63068 4.67342 3.14495 2.56807 3.88580 3.47822 3.65332
3.27811 2.27484 2.75576 3.75726 3.05147 3.86100 2.50708 2.51077
2.70775 2.19293 2.99464 4.24050 3.89325 167 t - - -
2.68618 4.42225 2.77519 2.73123 3.46354 2.40513 3.72494
3.29354 2.67741 2.69355 4.24690 2.90347 2.73739 3.18146 2.89801
2.37887 2.77519 2.98518 4.58477 3.61503
0.02625 4.04896 4.77131 0.61958 0.77255 0.48576 0.95510
89 2.84760 5.26538 1.94546 2.20106 4.66689 2.90044 3.79062
4.18213 2.76276 3.71061 4.54613 1.46867 3.84818 2.95763 3.31539
2.78412 3.15195 3.75210 5.86861 4.41060 168 n - - -
2.68618 4.42225 2.77519 2.73123 3.46354 2.40513 3.72494
3.29354 2.67741 2.69355 4.24690 2.90347 2.73739 3.18146 2.89801
2.37887 2.77519 2.98518 4.58477 3.61503
0.02625 4.04896 4.77131 0.61958 0.77255 0.48576 0.95510
90 3.32423 4.69320 4.72703 4.22660 3.11588 4.48513 4.81213
2.02877 4.00514 0.82600 2.97616 4.49619 4.73591 4.23789 4.15846
3.87953 3.56961 2.24097 5.17891 3.99937 169 l - - -
2.68618 4.42225 2.77519 2.73123 3.46354 2.40513 3.72494
3.29354 2.67741 2.69355 4.24690 2.90347 2.73739 3.18146 2.89801
2.37887 2.77519 2.98518 4.58477 3.61503
0.02625 4.04896 4.77131 0.61958 0.77255 0.48576 0.95510

```
     91   3.25890  4.57257  4.86068  4.33820  3.25313  4.55290  4.93385
1.31899  4.17287  1.25211  3.07372  4.58367  4.78322  4.37140  4.32196
3.92984  3.49973  1.93788  5.30185  4.15119    170 l - - -
          2.68618  4.42225  2.77519  2.73123  3.46354  2.40513  3.72494
3.29354  2.67741  2.69355  4.24690  2.90347  2.73739  3.18146  2.89801
2.37887  2.77519  2.98518  4.58477  3.61503
          0.02625  4.04896  4.77131  0.61958  0.77255  0.48576  0.95510
     92   3.49106  4.84211  4.48107  4.23211  0.76177  4.13322  3.96743
2.99649  4.13553  2.32725  3.70244  4.22219  4.59014  4.25557  4.21149
3.74980  3.79345  2.99963  4.16985  2.51707    171 f - - -
          2.68618  4.42225  2.77519  2.73123  3.46354  2.40513  3.72494
3.29354  2.67741  2.69355  4.24690  2.90347  2.73739  3.18146  2.89801
2.37887  2.77519  2.98518  4.58477  3.61503
          0.02625  4.04896  4.77131  0.61958  0.77255  0.48576  0.95510
     93   3.00224  4.72657  3.62906  3.49603  4.47053  3.40426  4.55621
4.06484  3.56912  3.67110  4.73068  3.78339  0.55633  3.95247  3.79183
3.17599  3.45834  3.68948  5.60532  4.59466    172 P - - -
          2.68618  4.42225  2.77519  2.73123  3.46354  2.40513  3.72494
3.29354  2.67741  2.69355  4.24690  2.90347  2.73739  3.18146  2.89801
2.37887  2.77519  2.98518  4.58477  3.61503
          0.02625  4.04896  4.77131  0.61958  0.77255  0.48576  0.95510
     94   2.53582  4.63135  2.78779  2.58097  4.34640  3.15234  3.91908
3.86901  2.80186  3.48255  4.31809  1.72727  3.79909  3.12136  3.21472
1.69340  2.92692  3.40338  5.62983  4.27733    173 s - - -
          2.68618  4.42225  2.77519  2.73123  3.46354  2.40513  3.72494
3.29354  2.67741  2.69355  4.24690  2.90347  2.73739  3.18146  2.89801
2.37887  2.77519  2.98518  4.58477  3.61503
          0.02625  4.04896  4.77131  0.61958  0.77255  0.48576  0.95510
     95   2.38068  3.31244  3.24471  2.85077  4.12450  1.90751  3.97673
3.54211  2.87471  3.21248  4.04831  1.96908  3.76582  3.20405  3.25245
2.52294  2.78311  3.11681  5.45458  4.18717    174 g - - -
          2.68618  4.42225  2.77519  2.73123  3.46354  2.40513  3.72494
3.29354  2.67741  2.69355  4.24690  2.90347  2.73739  3.18146  2.89801
2.37887  2.77519  2.98518  4.58477  3.61503
          0.02625  4.04896  4.77131  0.61958  0.77255  0.48576  0.95510
     96   2.46344  4.50057  2.92440  2.71597  4.30967  3.10579  3.99914
3.83399  2.89764  3.47097  4.31106  2.40179  3.78496  3.22333  3.27961
1.24797  2.88634  3.34820  5.61902  4.29005    175 s - - -
          2.68618  4.42225  2.77519  2.73123  3.46354  2.40513  3.72494
3.29354  2.67741  2.69355  4.24690  2.90347  2.73739  3.18146  2.89801
2.37887  2.77519  2.98518  4.58477  3.61503
          0.02625  4.04896  4.77131  0.61958  0.77255  0.48576  0.95510
     97   2.48898  4.27816  3.55972  3.08941  3.70771  3.35170  4.03646
2.35865  3.00839  2.70345  3.65827  3.39165  3.91211  3.34995  3.32672
2.45445  1.60463  2.54172  5.18088  3.94256    176 t - - -
          2.68618  4.42225  2.77519  2.73123  3.46354  2.40513  3.72494
3.29354  2.67741  2.69355  4.24690  2.90347  2.73739  3.18146  2.89801
2.37887  2.77519  2.98518  4.58477  3.61503
          0.02625  4.04896  4.77131  0.61958  0.77255  0.48576  0.95510
     98   2.64910  4.92521  2.57728  2.21919  4.19630  3.36047  3.64515
3.62932  2.46369  2.68041  4.02204  2.34011  3.80862  2.79242  2.94064
2.15139  2.89343  3.28198  5.43128  4.06789    177 s - - -
          2.68618  4.42225  2.77519  2.73123  3.46354  2.40513  3.72494
3.29354  2.67741  2.69355  4.24690  2.90347  2.73739  3.18146  2.89801
2.37887  2.77519  2.98518  4.58477  3.61503
```

0.02625 4.04896 4.77131 0.61958 0.77255 0.48576 0.95510
99 2.59754 4.27783 3.61968 3.07215 3.43501 3.56749 3.94209
2.62728 2.97026 1.91693 3.06230 3.42603 3.99311 3.29680 3.28235
2.64375 2.07339 2.43456 4.94627 3.72145 178 l - - -
2.68618 4.42225 2.77519 2.73123 3.46354 2.40513 3.72494
3.29354 2.67741 2.69355 4.24690 2.90347 2.73739 3.18146 2.89801
2.37887 2.77519 2.98518 4.58477 3.61503
0.02625 4.04896 4.77131 0.61958 0.77255 0.48576 0.95510
100 3.11652 4.63928 4.24324 3.85342 3.29692 3.96808 4.54782
2.49068 3.61082 1.86793 1.07604 4.10809 4.43260 3.98278 3.79473
3.48249 3.42685 2.51659 5.15930 3.93501 179 m - - -
2.68618 4.42225 2.77519 2.73123 3.46354 2.40513 3.72494
3.29354 2.67741 2.69355 4.24690 2.90347 2.73739 3.18146 2.89801
2.37887 2.77519 2.98518 4.58477 3.61503
0.02625 4.04896 4.77131 0.61958 0.77255 0.48576 0.95510
101 2.87265 5.26005 2.50306 1.56267 4.55979 3.35592 3.71023
4.03128 2.45970 3.53921 4.38230 2.84616 3.88120 1.89234 2.87716
2.81573 3.12474 3.65556 5.68834 4.30215 180 e - - -
2.68618 4.42225 2.77519 2.73123 3.46354 2.40513 3.72494
3.29354 2.67741 2.69355 4.24690 2.90347 2.73739 3.18146 2.89801
2.37887 2.77519 2.98518 4.58477 3.61503
0.02625 4.04896 4.77131 0.61958 0.77255 0.48576 0.95510
102 2.98347 5.56126 1.57800 1.31528 4.84824 3.25043 3.79703
4.37015 2.81658 3.86836 4.72293 2.71341 3.87377 2.96353 3.41420
2.86354 3.27246 3.94081 6.01628 4.51042 181 e - - -
2.68618 4.42225 2.77519 2.73123 3.46354 2.40513 3.72494
3.29354 2.67741 2.69355 4.24690 2.90347 2.73739 3.18146 2.89801
2.37887 2.77519 2.98518 4.58477 3.61503
0.02625 4.04896 4.77131 0.61958 0.77255 0.48576 0.95510
103 3.17037 4.54472 4.57938 4.20466 3.49871 4.25159 4.90309
0.88271 4.03620 2.05240 3.41046 4.42573 4.66901 4.36848 4.21304
3.76696 3.46771 1.86689 5.42361 4.17486 182 i - - -
2.68618 4.42225 2.77519 2.73123 3.46354 2.40513 3.72494
3.29354 2.67741 2.69355 4.24690 2.90347 2.73739 3.18146 2.89801
2.37887 2.77519 2.98518 4.58477 3.61503
0.02625 4.04896 4.77131 0.61958 0.77255 0.48576 0.95510
104 3.32545 4.68725 4.74837 4.24177 3.12054 4.50219 4.82783
1.98929 4.02804 0.83685 2.97222 4.51184 4.74422 4.25066 4.17986
3.89209 3.56838 2.22279 5.18844 4.01891 183 l - - -
2.68618 4.42225 2.77519 2.73123 3.46354 2.40513 3.72494
3.29354 2.67741 2.69355 4.24690 2.90347 2.73739 3.18146 2.89801
2.37887 2.77519 2.98518 4.58477 3.61503
0.02625 4.04896 4.77131 0.61958 0.77255 0.48576 0.95510
105 3.21415 5.03434 3.72375 3.20215 4.43412 3.66389 3.96639
3.99095 2.32798 3.47493 4.47340 3.54687 4.16051 3.18049 0.73952
3.29160 3.46976 3.70799 5.44719 4.32628 184 r - - -
2.68618 4.42225 2.77519 2.73123 3.46354 2.40513 3.72494
3.29354 2.67741 2.69355 4.24690 2.90347 2.73739 3.18146 2.89801
2.37887 2.77519 2.98518 4.58477 3.61503
0.02625 4.04896 4.77131 0.61958 0.77255 0.48576 0.95510
106 1.76153 4.78169 2.84240 2.12662 4.16458 2.99277 3.71018
3.57226 2.50232 3.18115 4.00952 2.95876 3.82376 2.87623 2.70734
2.66444 2.89763 3.22535 5.41603 4.09123 185 a - - -

2.68618 4.42225 2.77519 2.73123 3.46354 2.40513 3.72494
3.29354 2.67741 2.69355 4.24690 2.90347 2.73739 3.18146 2.89801
2.37887 2.77519 2.98518 4.58477 3.61503
0.02625 4.04896 4.77131 0.61958 0.77255 0.48576 0.95510
107 2.27654 4.49194 2.68209 2.69334 4.13911 3.19621 3.93835
3.41061 2.85471 3.17319 4.04589 3.10557 3.81116 3.15710 3.26355
2.61028 1.44383 3.05018 5.48662 4.19662 186 t - - -
2.68618 4.42225 2.77519 2.73123 3.46354 2.40513 3.72494
3.29354 2.67741 2.69355 4.24690 2.90347 2.73739 3.18146 2.89801
2.37887 2.77519 2.98518 4.58477 3.61503
0.02625 4.04896 4.77131 0.61958 0.77255 0.48576 0.95510
108 2.82260 5.17189 2.49302 1.33609 4.49362 3.33146 3.71967
3.92749 2.51800 3.47752 4.32137 2.83926 3.86417 2.61469 2.96077
2.78185 2.78632 3.56018 5.66970 4.28104 187 e - - -
2.68618 4.42225 2.77519 2.73123 3.46354 2.40513 3.72494
3.29354 2.67741 2.69355 4.24690 2.90347 2.73739 3.18146 2.89801
2.37887 2.77519 2.98518 4.58477 3.61503
0.02625 4.04896 4.77131 0.61958 0.77255 0.48576 0.95510
109 2.88144 5.18826 2.83213 2.28524 4.54394 3.47324 3.64431
3.95736 1.87478 3.44439 4.27479 2.96291 3.91820 1.66480 2.49766
2.84736 3.09702 3.59841 5.55910 4.25822 188 q - - -
2.68618 4.42225 2.77519 2.73123 3.46354 2.40513 3.72494
3.29354 2.67741 2.69355 4.24690 2.90347 2.73739 3.18146 2.89801
2.37887 2.77519 2.98518 4.58477 3.61503
0.02625 4.04896 4.77131 0.61958 0.77255 0.48576 0.95510
110 3.12686 4.56911 4.17801 3.71020 1.57509 4.03899 3.58785
3.05382 3.59591 2.38898 3.67964 3.16221 4.38518 3.75442 3.77903
3.34783 3.35461 2.89331 3.87870 1.53740 189 y - - -
2.68618 4.42225 2.77519 2.73123 3.46354 2.40513 3.72494
3.29354 2.67741 2.69355 4.24690 2.90347 2.73739 3.18146 2.89801
2.37887 2.77519 2.98518 4.58477 3.61503
0.02625 4.04896 4.77131 0.61958 0.77255 0.48576 0.95510
111 2.83965 4.26432 4.32301 3.76692 3.37360 4.02346 4.41043
1.74397 3.64973 1.75730 3.27694 4.00907 4.37196 3.90213 3.85992
2.99589 3.08656 1.50911 5.07579 3.87876 190 v - - -
2.68618 4.42225 2.77519 2.73123 3.46354 2.40513 3.72494
3.29354 2.67741 2.69355 4.24690 2.90347 2.73739 3.18146 2.89801
2.37887 2.77519 2.98518 4.58477 3.61503
0.02625 4.04896 4.77131 0.61958 0.77255 0.48576 0.95510
112 2.64938 3.97811 2.94404 2.44068 4.17941 3.41102 3.62848
3.60200 2.19647 3.17596 3.98924 2.26609 3.82853 2.10966 2.73630
2.66780 2.88606 3.25954 5.37725 4.04830 191 q - - -
2.68618 4.42225 2.77519 2.73123 3.46354 2.40513 3.72494
3.29354 2.67741 2.69355 4.24690 2.90347 2.73739 3.18146 2.89801
2.37887 2.77519 2.98518 4.58477 3.61503
0.02625 4.04896 4.77131 0.61958 0.77255 0.48576 0.95510
113 2.72027 4.99164 2.79677 1.93857 4.25166 3.40966 3.64836
3.26734 2.36317 3.22931 4.06968 2.92050 3.85041 1.91228 2.77116
2.71749 2.95514 3.33075 5.45016 4.10287 192 q - - -
2.68618 4.42225 2.77520 2.73124 3.46354 2.40513 3.72495
3.29354 2.67732 2.69355 4.24690 2.90347 2.73740 3.18147 2.89801
2.37887 2.77520 2.98519 4.58477 3.61504
0.06718 2.87349 4.77131 0.47769 0.96815 0.48576 0.95510

```
    114   2.77537  5.09289  2.66729  2.43192  4.42592  3.44522  3.62317
3.86474  2.21947  3.36999  4.18150  2.94542  3.87206  2.00894  1.87302
2.75478  2.99870  3.49770  5.50144  4.17604    194 r - - -
          2.68618  4.42225  2.77519  2.73123  3.46354  2.40513  3.72494
3.29354  2.67741  2.69355  4.24690  2.90347  2.73739  3.18146  2.89801
2.37887  2.77519  2.98518  4.58477  3.61503
          0.02625  4.04896  4.77131  0.61958  0.77255  0.48576  0.95510
    115   3.35841  4.77029  4.47216  4.11619  3.21164  4.20451  4.71683
2.42879  3.88223  0.68484  3.18550  4.37450  4.61168  4.20806  4.02805
3.80108  3.64584  2.51132  5.15144  3.91438    195 L - - -
          2.68618  4.42225  2.77519  2.73123  3.46354  2.40513  3.72494
3.29354  2.67741  2.69355  4.24690  2.90347  2.73739  3.18146  2.89801
2.37887  2.77519  2.98518  4.58477  3.61503
          0.02625  4.04896  4.77131  0.61958  0.77255  0.48576  0.95510
    116   2.75196  5.11188  1.63223  2.26241  4.38904  3.30712  3.70745
3.32373  2.58969  3.43400  4.26094  2.19491  3.83145  2.86210  3.10021
2.71882  3.01907  3.47584  5.62101  4.20055    196 d - - -
          2.68618  4.42225  2.77519  2.73123  3.46354  2.40513  3.72494
3.29354  2.67741  2.69355  4.24690  2.90347  2.73739  3.18146  2.89801
2.37887  2.77519  2.98518  4.58477  3.61503
          0.02625  4.04896  4.77131  0.61958  0.77255  0.48576  0.95510
    117   2.17031  4.71640  2.59511  2.46927  4.10718  3.31460  3.73198
3.49958  2.57974  3.13555  3.96969  2.96621  3.81281  2.69214  3.02940
2.64196  1.91402  3.15813  5.39462  4.06860    197 t - - -
          2.68618  4.42225  2.77519  2.73123  3.46354  2.40513  3.72494
3.29354  2.67741  2.69355  4.24690  2.90347  2.73739  3.18146  2.89801
2.37887  2.77519  2.98518  4.58477  3.61503
          0.02625  4.04896  4.77131  0.61958  0.77255  0.48576  0.95510
    118   2.59177  4.65387  2.16039  2.52309  3.92640  3.40277  3.71474
3.20354  2.56806  2.70475  3.80485  3.01891  3.84159  2.90042  3.01295
2.51529  2.67242  2.43642  5.26282  3.95026    198 d - - -
          2.68618  4.42225  2.77519  2.73123  3.46354  2.40513  3.72494
3.29354  2.67741  2.69355  4.24690  2.90347  2.73739  3.18146  2.89801
2.37887  2.77519  2.98518  4.58477  3.61503
          0.02625  4.04896  4.77131  0.61958  0.77255  0.48576  0.95510
    119   2.56606  4.36967  3.60906  3.22315  3.76830  3.36335  4.17511
2.81141  3.07955  2.41600  3.76718  3.48992  3.97720  3.48684  3.36133
2.78903  1.27770  2.59177  5.31630  4.04351    199 t - - -
          2.68618  4.42225  2.77519  2.73123  3.46354  2.40513  3.72494
3.29354  2.67741  2.69355  4.24690  2.90347  2.73739  3.18146  2.89801
2.37887  2.77519  2.98518  4.58477  3.61503
          0.02625  4.04896  4.77131  0.61958  0.77255  0.48576  0.95510
    120   2.54180  4.24743  4.21507  3.65691  2.33663  3.87900  3.95721
2.53245  3.51891  1.64145  3.17801  3.82322  4.22423  3.71225  3.68193
3.17910  3.03188  2.42697  2.64041  2.99748    200 l - - -
          2.68618  4.42225  2.77519  2.73123  3.46354  2.40513  3.72494
3.29354  2.67741  2.69355  4.24690  2.90347  2.73739  3.18146  2.89801
2.37887  2.77519  2.98518  4.58477  3.61503
          0.02625  4.04896  4.77131  0.61958  0.77255  0.48576  0.95510
    121   2.04160  4.60563  2.85672  2.56058  4.32088  2.83773  3.86057
3.75544  2.73144  3.36667  4.18340  1.73206  3.21845  3.04171  3.16838
2.57465  2.86243  3.31351  5.58367  4.26305    201 n - - -
          2.68618  4.42225  2.77519  2.73123  3.46354  2.40513  3.72494
3.29354  2.67741  2.69355  4.24690  2.90347  2.73739  3.18146  2.89801
2.37887  2.77519  2.98518  4.58477  3.61503
```

0.02625 4.04896 4.77131 0.61958 0.77255 0.48576 0.95510
122 2.86031 4.80478 3.52818 2.94200 4.10515 3.56278 3.78927
3.44747 2.22246 3.05444 4.04066 3.31002 4.02425 2.98072 1.21267
2.95736 3.12244 2.81572 5.33114 4.08519 202 r - - -
2.68618 4.42225 2.77519 2.73123 3.46354 2.40513 3.72494
3.29354 2.67741 2.69355 4.24690 2.90347 2.73739 3.18146 2.89801
2.37887 2.77519 2.98518 4.58477 3.61503
0.02625 4.04896 4.77131 0.61958 0.77255 0.48576 0.95510
123 2.87353 4.37686 4.11539 3.65303 3.23206 3.90994 4.28105
2.20045 3.50511 2.23216 3.41539 3.90001 4.33565 3.82493 3.73285
3.27974 3.15307 1.13487 4.92910 3.07331 203 v - - -
2.68618 4.42225 2.77519 2.73123 3.46354 2.40513 3.72494
3.29354 2.67741 2.69355 4.24690 2.90347 2.73739 3.18146 2.89801
2.37887 2.77519 2.98518 4.58477 3.61503
0.02625 4.04896 4.77131 0.61958 0.77255 0.48576 0.95510
124 2.61800 4.80699 2.85795 2.29006 4.11213 3.36543 3.66793
3.53057 2.46681 3.13636 3.48539 2.24675 3.81606 2.82702 2.91110
2.00786 2.87294 3.19431 5.36949 4.03203 204 s - - -
2.68618 4.42225 2.77519 2.73123 3.46354 2.40513 3.72494
3.29354 2.67741 2.69355 4.24690 2.90347 2.73739 3.18146 2.89801
2.37887 2.77519 2.98518 4.58477 3.61503
0.02625 4.04896 4.77131 0.61958 0.77255 0.48576 0.95510
125 1.89637 4.78967 3.00980 2.55258 4.16493 3.39714 3.70011
3.56584 2.37022 3.15902 4.00572 3.02890 3.86144 2.04678 2.53475
2.71554 2.92974 3.23328 5.38254 4.08538 205 a - - -
2.68618 4.42225 2.77519 2.73123 3.46354 2.40513 3.72494
3.29354 2.67741 2.69355 4.24690 2.90347 2.73739 3.18146 2.89801
2.37887 2.77519 2.98518 4.58477 3.61503
0.02625 4.04896 4.77131 0.61958 0.77255 0.48576 0.95510
126 2.92967 5.31191 2.33699 1.11791 4.63459 3.29094 3.79989
4.13922 2.69190 3.68892 4.55702 2.52560 3.89152 2.97452 3.16870
2.86147 3.22047 3.74824 5.82084 4.39092 206 e - - -
2.68618 4.42225 2.77519 2.73123 3.46354 2.40513 3.72494
3.29354 2.67741 2.69355 4.24690 2.90347 2.73739 3.18146 2.89801
2.37887 2.77519 2.98518 4.58477 3.61503
0.02625 4.04896 4.77131 0.61958 0.77255 0.48576 0.95510
127 3.15431 4.57874 4.42804 3.90689 2.81420 4.16936 4.20599
2.51800 3.64601 0.93413 3.11179 4.10689 4.48244 3.91800 3.80335
3.54050 3.38749 2.58021 3.51454 3.16332 207 l - - -
2.68618 4.42225 2.77519 2.73123 3.46354 2.40513 3.72494
3.29354 2.67741 2.69355 4.24690 2.90347 2.73739 3.18146 2.89801
2.37887 2.77519 2.98518 4.58477 3.61503
0.02625 4.04896 4.77131 0.61958 0.77255 0.48576 0.95510
128 2.46423 4.51070 3.18075 2.28916 3.69047 3.47552 3.37248
2.71772 2.58072 2.73928 3.60475 3.09828 3.86365 2.93018 2.83046
2.70531 2.69123 2.35183 5.06356 3.78953 208 e - - -
2.68618 4.42225 2.77519 2.73123 3.46354 2.40513 3.72494
3.29354 2.67741 2.69355 4.24690 2.90347 2.73739 3.18146 2.89801
2.37887 2.77519 2.98518 4.58477 3.61503
0.02625 4.04896 4.77131 0.61958 0.77255 0.48576 0.95510
129 2.89902 4.65351 3.60866 3.53220 4.66473 0.50220 4.63853
4.31037 3.73728 3.94135 4.92090 3.77344 4.05011 4.04773 3.95090
3.07653 3.39400 3.80462 5.71003 4.77372 209 G - - -

2.68618 4.42225 2.77519 2.73123 3.46354 2.40513 3.72494
3.29354 2.67741 2.69355 4.24690 2.90347 2.73739 3.18146 2.89801
2.37887 2.77519 2.98518 4.58477 3.61503
0.02625 4.04896 4.77131 0.61958 0.77255 0.48576 0.95510
130 3.35841 4.77029 4.47216 4.11619 3.21164 4.20451 4.71683
2.42879 3.88223 0.68484 3.18550 4.37450 4.61168 4.20806 4.02805
3.80108 3.64584 2.51132 5.15144 3.91438 210 L - - -
2.68618 4.42225 2.77519 2.73123 3.46354 2.40513 3.72494
3.29354 2.67741 2.69355 4.24690 2.90347 2.73739 3.18146 2.89801
2.37887 2.77519 2.98518 4.58477 3.61503
0.02625 4.04896 4.77131 0.61958 0.77255 0.48576 0.95510
131 2.85294 5.20020 2.83592 2.13011 4.56427 3.46496 3.62763
3.98402 1.66387 3.45883 4.27451 2.94609 3.90052 2.02861 2.50338
2.81394 3.06777 3.61080 5.56612 4.25454 211 k - - -
2.68618 4.42225 2.77519 2.73123 3.46354 2.40513 3.72494
3.29354 2.67741 2.69355 4.24690 2.90347 2.73739 3.18146 2.89801
2.37887 2.77519 2.98518 4.58477 3.61503
0.02625 4.04896 4.77131 0.61958 0.77255 0.48576 0.95510
132 1.63357 4.52725 2.92527 2.68961 4.28208 3.14710 3.96936
3.67617 2.85768 3.37128 4.22876 1.73524 3.80022 3.18412 3.24991
2.59908 2.89266 3.24832 5.59301 4.28051 212 a - - -
2.68618 4.42225 2.77519 2.73123 3.46354 2.40513 3.72494
3.29354 2.67741 2.69355 4.24690 2.90347 2.73739 3.18146 2.89801
2.37887 2.77519 2.98518 4.58477 3.61503
0.02625 4.04896 4.77131 0.61958 0.77255 0.48576 0.95510
133 2.83111 5.25677 1.88933 2.19896 4.64050 3.24443 3.77298
4.15532 2.73087 3.68375 4.51363 1.56757 3.84176 2.93615 3.27984
2.50904 3.13008 3.72800 5.84397 4.38352 213 n - - -
2.68618 4.42225 2.77519 2.73123 3.46354 2.40513 3.72494
3.29354 2.67741 2.69355 4.24690 2.90347 2.73739 3.18146 2.89801
2.37887 2.77519 2.98518 4.58477 3.61503
0.02625 4.04896 4.77131 0.61958 0.77255 0.48576 0.95510
134 3.15981 4.47433 4.82529 4.28499 3.35782 4.48961 4.91291
1.66227 4.16698 1.36318 3.16061 4.52111 4.73907 4.36020 4.33314
3.84584 3.40198 1.39552 5.34873 4.20840 214 l - - -
2.68618 4.42225 2.77519 2.73123 3.46354 2.40513 3.72494
3.29354 2.67741 2.69355 4.24690 2.90347 2.73739 3.18146 2.89801
2.37887 2.77519 2.98518 4.58477 3.61503
0.02625 4.04896 4.77131 0.61958 0.77255 0.48576 0.95510
135 2.53505 5.01126 2.88197 2.08735 4.32786 3.41360 3.60131
3.76005 2.27324 3.29031 4.08457 2.74576 3.82459 2.56385 2.07567
2.66884 2.68585 3.39133 5.45677 4.11247 215 r - - -
2.68618 4.42225 2.77519 2.73123 3.46354 2.40513 3.72494
3.29354 2.67741 2.69355 4.24690 2.90347 2.73739 3.18146 2.89801
2.37887 2.77519 2.98518 4.58477 3.61503
0.02625 4.04896 4.77131 0.61958 0.77255 0.48576 0.95510
136 2.70316 4.86481 2.69906 1.72186 4.27765 3.31922 3.78379
3.60379 2.60608 3.27699 4.14759 2.95147 3.85923 2.95943 3.03304
2.73515 1.85341 3.26852 5.54088 4.20141 216 e - - -
2.68618 4.42225 2.77519 2.73123 3.46354 2.40513 3.72494
3.29354 2.67741 2.69355 4.24690 2.90347 2.73739 3.18146 2.89801
2.37887 2.77519 2.98518 4.58477 3.61503
0.02625 4.04896 4.77131 0.61958 0.77255 0.48576 0.95510

137 3.49106 4.84211 4.48107 4.23211 0.76177 4.13322 3.96743
2.99649 4.13553 2.32725 3.70244 4.22219 4.59014 4.25557 4.21149
3.74980 3.79345 2.99963 4.16985 2.51707 217 f - - -
2.68618 4.42225 2.77519 2.73123 3.46354 2.40513 3.72494
3.29354 2.67741 2.69355 4.24690 2.90347 2.73739 3.18146 2.89801
2.37887 2.77519 2.98518 4.58477 3.61503
0.02625 4.04896 4.77131 0.61958 0.77255 0.48576 0.95510
138 2.87931 4.87574 2.82111 2.72621 4.26801 3.31004 4.07758
4.02015 2.99819 3.64783 4.60465 0.87973 3.97208 3.35350 3.35640
2.94155 3.26875 3.62429 5.55383 4.20588 218 n - - -
2.68618 4.42225 2.77519 2.73123 3.46354 2.40513 3.72494
3.29354 2.67741 2.69355 4.24690 2.90347 2.73739 3.18146 2.89801
2.37887 2.77519 2.98518 4.58477 3.61503
0.02625 4.04896 4.77131 0.61958 0.77255 0.48576 0.95510
139 2.73136 5.19097 1.95780 2.12004 4.50804 3.34734 3.62920
3.97834 2.39090 3.47521 4.25881 2.81745 3.81477 2.42487 2.26747
2.68129 2.97149 3.57143 5.62198 4.21905 219 d - - -
2.68618 4.42225 2.77519 2.73123 3.46354 2.40513 3.72494
3.29354 2.67741 2.69355 4.24690 2.90347 2.73739 3.18146 2.89801
2.37887 2.77519 2.98518 4.58477 3.61503
0.02625 4.04896 4.77131 0.61958 0.77255 0.48576 0.95510
140 2.93599 5.11666 2.91293 2.56557 4.43599 3.49536 3.70403
3.89104 2.02296 3.39526 4.27975 3.04794 3.96714 1.38251 2.49019
2.92796 3.16628 3.56391 5.51164 4.22650 220 q - - -
2.68618 4.42225 2.77519 2.73123 3.46354 2.40513 3.72494
3.29354 2.67741 2.69355 4.24690 2.90347 2.73739 3.18146 2.89801
2.37887 2.77519 2.98518 4.58477 3.61503
0.02625 4.04896 4.77131 0.61958 0.77255 0.48576 0.95510
141 3.10202 4.42712 4.79481 4.30647 3.60272 4.43081 5.03127
1.47909 4.19086 2.11567 3.41397 4.52265 4.76081 4.46620 4.39392
3.82399 3.37400 1.01310 5.56315 4.34806 221 v - - -
2.68618 4.42225 2.77519 2.73123 3.46354 2.40513 3.72494
3.29354 2.67741 2.69355 4.24690 2.90347 2.73739 3.18146 2.89801
2.37887 2.77519 2.98518 4.58477 3.61503
0.02625 4.04896 4.77131 0.61958 0.77255 0.48576 0.95510
142 2.99655 5.59118 1.15636 1.92302 4.87221 3.23602 3.80346
4.40597 2.84976 3.89954 4.75916 2.42715 3.87324 2.97430 3.46268
2.87017 3.29168 3.97151 6.05689 4.52976 222 d - - -
2.68618 4.42225 2.77519 2.73123 3.46354 2.40513 3.72494
3.29354 2.67741 2.69355 4.24690 2.90347 2.73739 3.18146 2.89801
2.37887 2.77519 2.98518 4.58477 3.61503
0.02625 4.04896 4.77131 0.61958 0.77255 0.48576 0.95510
143 2.93665 5.43022 1.42783 2.15855 4.74950 3.24130 3.80851
4.30935 2.82577 3.82481 4.67926 1.73535 3.86841 2.98167 3.40847
2.84118 3.23949 3.87676 5.95275 4.45651 223 d - - -
2.68618 4.42225 2.77519 2.73123 3.46354 2.40513 3.72494
3.29354 2.67741 2.69355 4.24690 2.90347 2.73739 3.18146 2.89801
2.37887 2.77519 2.98518 4.58477 3.61503
0.02625 4.04896 4.77131 0.61958 0.77255 0.48576 0.95510
144 2.92383 4.50488 3.99557 3.65616 1.13106 3.74653 3.84499
2.93894 3.58286 2.50285 3.67069 3.77117 4.26056 3.80592 3.77484
2.74448 3.25275 2.79310 4.22955 2.60064 224 f - - -
2.68618 4.42225 2.77519 2.73123 3.46354 2.40513 3.72494
3.29354 2.67741 2.69355 4.24690 2.90347 2.73739 3.18146 2.89801
2.37887 2.77519 2.98518 4.58477 3.61503

0.02625 4.04896 4.77131 0.61958 0.77255 0.48576 0.95510
145 3.35841 4.77029 4.47216 4.11619 3.21164 4.20451 4.71683
2.42879 3.88223 0.68484 3.18550 4.37450 4.61168 4.20806 4.02805
3.80108 3.64584 2.51132 5.15144 3.91438 225 L - - -
2.68618 4.42225 2.77519 2.73123 3.46354 2.40513 3.72494
3.29354 2.67741 2.69355 4.24690 2.90347 2.73739 3.18146 2.89801
2.37887 2.77519 2.98518 4.58477 3.61503
0.02625 4.04896 4.77131 0.61958 0.77255 0.48576 0.95510
146 2.82216 5.10621 2.61981 2.38060 4.40651 3.35638 3.72396
3.94846 2.44593 3.47615 4.32346 1.90189 3.88375 1.79590 2.83164
2.79938 3.09070 3.57304 5.58402 4.20338 226 q - - -
2.68618 4.42225 2.77519 2.73123 3.46354 2.40513 3.72494
3.29354 2.67741 2.69355 4.24690 2.90347 2.73739 3.18146 2.89801
2.37887 2.77519 2.98518 4.58477 3.61503
0.02625 4.04896 4.77131 0.61958 0.77255 0.48576 0.95510
147 2.63951 4.75255 2.75012 2.54220 4.34469 3.22431 3.88974
3.86239 2.74966 3.46575 4.31803 1.69767 1.99204 3.08794 3.16178
2.69695 2.99963 3.43838 5.60354 4.26024 227 n - - -
2.68618 4.42225 2.77519 2.73123 3.46354 2.40513 3.72494
3.29354 2.67741 2.69355 4.24690 2.90347 2.73739 3.18146 2.89801
2.37887 2.77519 2.98518 4.58477 3.61503
0.02625 4.04896 4.77131 0.61958 0.77255 0.48576 0.95510
148 2.74920 4.92816 3.03038 2.56250 4.34541 3.43605 3.67474
3.76211 2.23386 3.30937 4.14314 2.13726 3.89153 2.82293 1.74631
2.77188 2.77471 3.40584 5.45603 4.16714 228 r - - -
2.68618 4.42225 2.77519 2.73123 3.46354 2.40513 3.72494
3.29354 2.67741 2.69355 4.24690 2.90347 2.73739 3.18146 2.89801
2.37887 2.77519 2.98518 4.58477 3.61503
0.02625 4.04896 4.77131 0.61958 0.77255 0.48576 0.95510
149 2.64255 4.50452 3.32450 2.75216 3.66844 3.54065 3.74954
2.72186 2.54963 2.66786 3.58583 3.19489 3.92628 2.49078 2.45987
2.79347 2.87244 2.09026 5.06049 3.80685 229 v - - -
2.68618 4.42225 2.77519 2.73123 3.46354 2.40513 3.72494
3.29354 2.67741 2.69355 4.24690 2.90347 2.73739 3.18146 2.89801
2.37887 2.77519 2.98518 4.58477 3.61503
0.02625 4.04896 4.77131 0.61958 0.77255 0.48576 0.95510
150 2.79364 5.15876 2.41190 1.93241 4.55303 2.22358 3.75137
4.02779 2.64060 3.56849 4.39444 1.86582 3.84154 2.91059 3.14068
2.75209 3.08157 3.62241 5.74511 4.32994 230 n - - -
2.68618 4.42225 2.77519 2.73123 3.46354 2.40513 3.72494
3.29354 2.67741 2.69355 4.24690 2.90347 2.73739 3.18146 2.89801
2.37887 2.77519 2.98518 4.58477 3.61503
0.02625 4.04896 4.77131 0.61958 0.77255 0.48576 0.95510
151 2.15248 4.26947 3.66811 3.18216 3.56724 3.50574 4.07709
1.73605 3.11279 2.49826 3.50569 3.49338 2.78314 3.42938 3.41811
2.84927 2.88553 2.33705 5.09719 3.87415 231 i - - -
2.68618 4.42225 2.77519 2.73123 3.46354 2.40513 3.72494
3.29354 2.67741 2.69355 4.24690 2.90347 2.73739 3.18146 2.89801
2.37887 2.77519 2.98518 4.58477 3.61503
0.02625 4.04896 4.77131 0.61958 0.77255 0.48576 0.95510
152 2.15297 4.20074 3.64534 3.22350 3.84580 3.20563 4.15905
2.91000 3.17920 2.85739 3.79184 3.43138 3.85177 3.48465 3.48064
1.75694 2.79060 1.72226 5.31043 4.08872 232 v - - -

2.68618 4.42225 2.77519 2.73123 3.46354 2.40513 3.72494
3.29354 2.67741 2.69355 4.24690 2.90347 2.73739 3.18146 2.89801
2.37887 2.77519 2.98518 4.58477 3.61503
0.02625 4.04896 4.77131 0.61958 0.77255 0.48576 0.95510
153 3.00224 4.72657 3.62906 3.49603 4.47053 3.40426 4.55621
4.06484 3.56912 3.67110 4.73068 3.78339 0.55633 3.95247 3.79183
3.17599 3.45834 3.68948 5.60532 4.59466 233 P - - -
2.68618 4.42225 2.77519 2.73123 3.46354 2.40513 3.72494
3.29354 2.67741 2.69355 4.24690 2.90347 2.73739 3.18146 2.89801
2.37887 2.77519 2.98518 4.58477 3.61503
0.02625 4.04896 4.77131 0.61958 0.77255 0.48576 0.95510
154 2.23159 4.32025 3.85400 3.38562 3.49139 3.61279 4.22500
2.44742 3.25737 1.38458 3.40003 3.65983 4.11610 3.59334 3.52195
2.98058 2.68496 2.30208 5.14311 3.93958 234 l - - -
2.68618 4.42225 2.77519 2.73123 3.46354 2.40513 3.72494
3.29354 2.67741 2.69355 4.24690 2.90347 2.73739 3.18146 2.89801
2.37887 2.77519 2.98518 4.58477 3.61503
0.02625 4.04896 4.77131 0.61958 0.77255 0.48576 0.95510
155 1.27066 4.17648 3.50006 3.21863 4.29591 2.96803 4.27015
3.64992 3.26179 3.39521 4.24273 3.35064 3.72182 3.55344 3.56091
1.59682 2.73522 3.13180 5.65969 4.44472 235 a - - -
2.68618 4.42225 2.77519 2.73123 3.46354 2.40513 3.72494
3.29354 2.67741 2.69355 4.24690 2.90347 2.73739 3.18146 2.89801
2.37887 2.77519 2.98518 4.58477 3.61503
0.02625 4.04896 4.77131 0.61958 0.77255 0.48576 0.95510
156 3.16860 4.50896 4.74622 4.23284 3.35835 4.44020 4.88473
1.05689 4.06124 1.81882 2.79749 4.47629 4.72397 4.31632 4.23846
3.81766 3.42217 1.83947 5.35226 4.17688 236 i - - -
2.68618 4.42225 2.77519 2.73123 3.46354 2.40513 3.72494
3.29354 2.67741 2.69355 4.24690 2.90347 2.73739 3.18146 2.89801
2.37887 2.77519 2.98518 4.58477 3.61503
0.02625 4.04896 4.77131 0.61958 0.77255 0.48576 0.95510
157 2.82199 4.34230 4.17345 3.70656 3.56116 3.87793 4.49895
1.29158 3.57196 2.25421 3.44540 3.95277 4.34177 3.90089 3.81379
3.25833 2.25714 1.91014 5.29944 4.08160 237 i - - -
2.68618 4.42225 2.77519 2.73123 3.46354 2.40513 3.72494
3.29354 2.67741 2.69355 4.24690 2.90347 2.73739 3.18146 2.89801
2.37887 2.77519 2.98518 4.58477 3.61503
0.02625 4.04896 4.77131 0.61958 0.77255 0.48576 0.95510
158 2.79221 5.11616 1.33183 2.26053 4.60791 3.21429 3.83718
4.11824 2.82912 3.68775 4.53572 2.80997 3.84354 3.01591 3.37316
2.02132 3.12568 3.68147 5.84574 4.40153 238 d - - -
2.68618 4.42225 2.77519 2.73123 3.46354 2.40513 3.72494
3.29354 2.67741 2.69355 4.24690 2.90347 2.73739 3.18146 2.89801
2.37887 2.77519 2.98518 4.58477 3.61503
0.02625 4.04896 4.77131 0.61958 0.77255 0.48576 0.95510
159 2.41035 4.33126 3.36913 3.19663 4.25684 3.05832 4.29982
3.80508 3.28244 3.51284 4.42630 3.39050 3.81243 3.61846 3.56056
0.85292 2.90434 3.30106 5.61579 4.34228 239 s - - -
2.68618 4.42225 2.77519 2.73123 3.46354 2.40513 3.72494
3.29354 2.67741 2.69355 4.24690 2.90347 2.73739 3.18146 2.89801
2.37887 2.77519 2.98518 4.58477 3.61503
0.02625 4.04896 4.77131 0.61958 0.77255 0.48576 0.95510

```
    160   3.12039  4.42879  4.84926  4.35703  3.60170  4.47920  5.07080
1.30640  4.24635  2.10167  3.40410  4.56991  4.79110  4.51087  4.44230
3.87149  3.38779  1.12301  5.57567  4.36881    240 v - - -
          2.68618  4.42225  2.77519  2.73123  3.46354  2.40513  3.72494
3.29354  2.67741  2.69355  4.24690  2.90347  2.73739  3.18146  2.89801
2.37887  2.77519  2.98518  4.58477  3.61503
          0.02625  4.04896  4.77131  0.61958  0.77255  0.48576  0.95510
    161   2.87931  4.87574  2.82111  2.72621  4.26801  3.31004  4.07758
4.02015  2.99819  3.64783  4.60465  0.87973  3.97208  3.35350  3.35640
2.94155  3.26875  3.62429  5.55383  4.20588    241 n - - -
          2.68618  4.42225  2.77519  2.73123  3.46354  2.40513  3.72494
3.29354  2.67741  2.69355  4.24690  2.90347  2.73739  3.18146  2.89801
2.37887  2.77519  2.98518  4.58477  3.61503
          0.02625  4.04896  4.77131  0.61958  0.77255  0.48576  0.95510
    162   2.51696  4.36119  3.59478  3.35937  4.13457  3.19354  4.36194
3.28365  3.30583  3.14078  4.18273  3.53737  3.91252  3.69255  3.55365
2.72057  0.90433  2.95928  5.55663  4.35230    242 t - - -
          2.68618  4.42225  2.77519  2.73123  3.46354  2.40513  3.72494
3.29354  2.67741  2.69355  4.24690  2.90347  2.73739  3.18146  2.89801
2.37887  2.77519  2.98518  4.58477  3.61503
          0.02625  4.04896  4.77131  0.61958  0.77255  0.48576  0.95510
    163   3.20174  4.63261  4.51654  3.99101  3.11760  4.30545  4.62279
2.26611  3.76267  1.40546  1.42350  4.27680  4.59212  4.03540  3.94354
3.67071  3.44593  2.34776  5.10874  3.93779    243 l - - -
          2.68618  4.42225  2.77519  2.73123  3.46354  2.40513  3.72494
3.29354  2.67741  2.69355  4.24690  2.90347  2.73739  3.18146  2.89801
2.37887  2.77519  2.98518  4.58477  3.61503
          0.02625  4.04896  4.77131  0.61958  0.77255  0.48576  0.95510
    164   3.03267  5.01648  3.03125  2.79233  4.20781  3.49003  3.95095
3.86917  2.60165  3.35356  4.37991  3.25232  4.04822  0.94997  2.88086
3.07456  3.33113  3.58887  5.44998  4.15689    244 q - - -
          2.68618  4.42225  2.77519  2.73123  3.46354  2.40513  3.72494
3.29354  2.67741  2.69355  4.24690  2.90347  2.73739  3.18146  2.89801
2.37887  2.77519  2.98518  4.58477  3.61503
          0.02625  4.04896  4.77131  0.61958  0.77255  0.48576  0.95510
    165   2.90693  5.07017  2.87497  2.55012  4.06867  3.47936  3.16170
3.84181  2.31821  3.34139  4.23663  3.03877  3.95921  1.37406  2.62428
2.90415  3.14720  3.52050  5.32410  3.92265    245 q - - -
          2.68618  4.42225  2.77519  2.73123  3.46354  2.40513  3.72494
3.29354  2.67741  2.69355  4.24690  2.90347  2.73739  3.18146  2.89801
2.37887  2.77519  2.98518  4.58477  3.61503
          0.02625  4.04896  4.77131  0.61958  0.77255  0.48576  0.95510
    166   3.19102  4.59169  4.63429  4.13655  3.20732  4.34074  4.77300
2.11324  3.95255  0.96128  3.05642  4.38887  4.65157  4.19353  4.12463
3.72788  3.45367  1.83309  5.22296  4.05254    246 l - - -
          2.68618  4.42225  2.77519  2.73123  3.46354  2.40513  3.72494
3.29354  2.67741  2.69355  4.24690  2.90347  2.73739  3.18146  2.89801
2.37887  2.77519  2.98518  4.58477  3.61503
          0.02625  4.04896  4.77131  0.61958  0.77255  0.48576  0.95510
    167   3.49106  4.84211  4.48107  4.23211  0.76177  4.13322  3.96743
2.99649  4.13553  2.32725  3.70244  4.22219  4.59014  4.25557  4.21149
3.74980  3.79345  2.99963  4.16985  2.51707    247 f - - -
          2.68618  4.42225  2.77519  2.73123  3.46354  2.40513  3.72494
3.29354  2.67741  2.69355  4.24690  2.90347  2.73739  3.18146  2.89801
2.37887  2.77519  2.98518  4.58477  3.61503
```

0.02625 4.04896 4.77131 0.61958 0.77255 0.48576 0.95510
168 3.10739 4.48805 4.63061 4.12619 3.34465 4.31542 4.78654
1.96689 3.96883 1.34850 3.19531 4.36572 4.64139 4.22562 4.15635
3.69313 3.37136 1.31504 5.29812 4.10633 248 v - - -
2.68618 4.42225 2.77519 2.73123 3.46354 2.40513 3.72494
3.29354 2.67741 2.69355 4.24690 2.90347 2.73739 3.18146 2.89801
2.37887 2.77519 2.98518 4.58477 3.61503
0.02625 4.04896 4.77131 0.61958 0.77255 0.48576 0.95510
169 2.57210 4.67578 2.73262 2.55538 4.35650 3.16173 3.91959
3.89873 2.80604 3.51230 4.35636 1.44827 3.81229 3.12550 3.21798
2.03360 2.96164 3.43718 5.64291 4.27693 249 n - - -
2.68618 4.42225 2.77519 2.73123 3.46354 2.40513 3.72494
3.29354 2.67741 2.69355 4.24690 2.90347 2.73739 3.18146 2.89801
2.37887 2.77519 2.98518 4.58477 3.61503
0.02625 4.04896 4.77131 0.61958 0.77255 0.48576 0.95510
170 3.21415 5.03434 3.72375 3.20215 4.43412 3.66389 3.96639
3.99095 2.32798 3.47493 4.47340 3.54687 4.16051 3.18049 0.73952
3.29160 3.46976 3.70799 5.44719 4.32628 250 r - - -
2.68618 4.42225 2.77519 2.73123 3.46354 2.40513 3.72494
3.29354 2.67741 2.69355 4.24690 2.90347 2.73739 3.18146 2.89801
2.37887 2.77519 2.98518 4.58477 3.61503
0.02625 4.04896 4.77131 0.61958 0.77255 0.48576 0.95510
171 3.35841 4.77029 4.47216 4.11619 3.21164 4.20451 4.71683
2.42879 3.88223 0.68484 3.18550 4.37450 4.61168 4.20806 4.02805
3.80108 3.64584 2.51132 5.15144 3.91438 251 L - - -
2.68618 4.42225 2.77519 2.73123 3.46354 2.40513 3.72494
3.29354 2.67741 2.69355 4.24690 2.90347 2.73739 3.18146 2.89801
2.37887 2.77519 2.98518 4.58477 3.61503
0.02625 4.04896 4.77131 0.61958 0.77255 0.48576 0.95510
172 3.00224 4.72657 3.62906 3.49603 4.47053 3.40426 4.55621
4.06484 3.56912 3.67110 4.73068 3.78339 0.55633 3.95247 3.79183
3.17599 3.45834 3.68948 5.60532 4.59466 252 P - - -
2.68618 4.42225 2.77519 2.73123 3.46354 2.40513 3.72494
3.29354 2.67741 2.69355 4.24690 2.90347 2.73739 3.18146 2.89801
2.37887 2.77519 2.98518 4.58477 3.61503
0.02625 4.04896 4.77131 0.61958 0.77255 0.48576 0.95510
173 3.03267 5.01648 3.03125 2.79233 4.20781 3.49003 3.95095
3.86917 2.60165 3.35356 4.37991 3.25232 4.04822 0.94997 2.88086
3.07456 3.33113 3.58887 5.44998 4.15689 253 q - - -
2.68618 4.42225 2.77519 2.73123 3.46354 2.40513 3.72494
3.29354 2.67741 2.69355 4.24690 2.90347 2.73739 3.18146 2.89801
2.37887 2.77519 2.98518 4.58477 3.61503
0.02625 4.04896 4.77131 0.61958 0.77255 0.48576 0.95510
174 3.49106 4.84211 4.48107 4.23211 0.76177 4.13322 3.96743
2.99649 4.13553 2.32725 3.70244 4.22219 4.59014 4.25557 4.21149
3.74980 3.79345 2.99963 4.16985 2.51707 254 f - - -
2.68618 4.42225 2.77519 2.73123 3.46354 2.40513 3.72494
3.29354 2.67741 2.69355 4.24690 2.90347 2.73739 3.18146 2.89801
2.37887 2.77519 2.98518 4.58477 3.61503
0.02625 4.04896 4.77131 0.61958 0.77255 0.48576 0.95510
175 2.92227 5.05364 3.08377 2.65252 4.35192 3.52818 3.70136
3.84104 2.17296 3.33313 4.22707 3.11103 3.97935 1.37763 2.20510
2.93315 3.15033 3.52319 5.44374 4.16942 255 q - - -

```
          2.68618  4.42225  2.77519  2.73123  3.46354  2.40513  3.72494
3.29354  2.67741  2.69355  4.24690  2.90347  2.73739  3.18146  2.89801
2.37887  2.77519  2.98518  4.58477  3.61503
          0.02625  4.04896  4.77131  0.61958  0.77255  0.48576  0.95510
    176   3.13651  4.44532  4.83871  4.28693  3.33726  4.47298  4.87792
1.53129  4.17729  1.50116  2.86427  4.50907  4.71511  4.34740  4.32784
3.82030  3.37314  1.45008  5.30523  4.18045    256 v - - -
          2.68618  4.42225  2.77519  2.73123  3.46354  2.40513  3.72494
3.29354  2.67741  2.69355  4.24690  2.90347  2.73739  3.18146  2.89801
2.37887  2.77519  2.98518  4.58477  3.61503
          0.02625  4.04896  4.77131  0.61958  0.77255  0.48576  0.95510
    177   2.69029  4.96187  2.85588  2.41923  4.31168  3.39204  3.64293
3.74996  2.32425  3.29460  4.10344  2.67113  3.83761  2.02217  2.53538
2.11409  2.93476  3.38411  5.46550  4.12888    257 q - - -
          2.68618  4.42225  2.77519  2.73123  3.46354  2.40513  3.72494
3.29354  2.67741  2.69355  4.24690  2.90347  2.73739  3.18146  2.89801
2.37887  2.77519  2.98518  4.58477  3.61503
          0.02625  4.04896  4.77131  0.61958  0.77255  0.48576  0.95510
    178   2.72690  5.01466  2.04730  2.32503  4.44963  1.75912  3.75855
3.92004  2.62955  3.47975  4.30246  2.86206  3.83235  2.91964  2.76577
2.71620  3.02191  3.51912  5.65635  4.27350    258 g - - -
          2.68618  4.42225  2.77519  2.73123  3.46354  2.40513  3.72494
3.29354  2.67741  2.69355  4.24690  2.90347  2.73739  3.18146  2.89801
2.37887  2.77519  2.98518  4.58477  3.61503
          0.02625  4.04896  4.77131  0.61958  0.77255  0.48576  0.95510
    179   2.94587  4.82471  2.43353  2.76959  2.94651  3.58118  3.71315
3.42740  2.97376  3.00840  4.00693  3.21717  4.07340  3.24833  3.36426
3.01170  3.21149  3.18954  4.52443  1.34307    259 y - - -
          2.68618  4.42225  2.77519  2.73123  3.46354  2.40513  3.72494
3.29354  2.67741  2.69355  4.24690  2.90347  2.73739  3.18146  2.89801
2.37887  2.77519  2.98518  4.58477  3.61503
          0.02625  4.04896  4.77131  0.61958  0.77255  0.48576  0.95510
    180   2.89344  5.20533  2.59184  2.01031  4.50386  3.38998  3.71972
3.96725  2.40929  3.48404  4.34662  2.90092  3.90730  1.48479  2.78203
2.85230  3.14251  3.61277  5.63240  4.27789    260 q - - -
          2.68618  4.42225  2.77519  2.73123  3.46354  2.40513  3.72494
3.29354  2.67741  2.69355  4.24690  2.90347  2.73739  3.18146  2.89801
2.37887  2.77519  2.98518  4.58477  3.61503
          0.02625  4.04896  4.77131  0.61958  0.77255  0.48576  0.95510
    181   3.12290  4.50532  4.63619  4.12950  3.31107  4.32368  4.78177
1.99575  3.96964  1.24525  3.15846  4.37155  4.64266  4.21799  4.15273
3.70048  3.38489  1.41546  5.27772  4.09537    261 l - - -
          2.68618  4.42225  2.77519  2.73123  3.46354  2.40513  3.72494
3.29354  2.67741  2.69355  4.24690  2.90347  2.73739  3.18146  2.89801
2.37887  2.77519  2.98518  4.58477  3.61503
          0.02625  4.04896  4.77131  0.61958  0.77255  0.48576  0.95510
    182   3.35841  4.77029  4.47216  4.11619  3.21164  4.20451  4.71683
2.42879  3.88223  0.68484  3.18550  4.37450  4.61168  4.20806  4.02805
3.80108  3.64584  2.51132  5.15144  3.91438    262 L - - -
          2.68618  4.42225  2.77519  2.73123  3.46354  2.40513  3.72494
3.29354  2.67741  2.69355  4.24690  2.90347  2.73739  3.18146  2.89801
2.37887  2.77519  2.98518  4.58477  3.61503
          0.02625  4.04896  4.77131  0.61958  0.77255  0.48576  0.95510
```

183 3.35841 4.77029 4.47216 4.11619 3.21164 4.20451 4.71683
2.42879 3.88223 0.68484 3.18550 4.37450 4.61168 4.20806 4.02805
3.80108 3.64584 2.51132 5.15144 3.91438 263 L - - -
2.68618 4.42225 2.77519 2.73123 3.46354 2.40513 3.72494
3.29354 2.67741 2.69355 4.24690 2.90347 2.73739 3.18146 2.89801
2.37887 2.77519 2.98518 4.58477 3.61503
0.02625 4.04896 4.77131 0.61958 0.77255 0.48576 0.95510
184 3.35841 4.77029 4.47216 4.11619 3.21164 4.20451 4.71683
2.42879 3.88223 0.68484 3.18550 4.37450 4.61168 4.20806 4.02805
3.80108 3.64584 2.51132 5.15144 3.91438 264 L - - -
2.68618 4.42225 2.77519 2.73123 3.46354 2.40513 3.72494
3.29354 2.67741 2.69355 4.24690 2.90347 2.73739 3.18146 2.89801
2.37887 2.77519 2.98518 4.58477 3.61503
0.02625 4.04896 4.77131 0.61958 0.77255 0.48576 0.95510
185 3.00224 4.72657 3.62906 3.49603 4.47053 3.40426 4.55621
4.06484 3.56912 3.67110 4.73068 3.78339 0.55633 3.95247 3.79183
3.17599 3.45834 3.68948 5.60532 4.59466 265 P - - -
2.68618 4.42225 2.77519 2.73123 3.46354 2.40513 3.72494
3.29354 2.67741 2.69355 4.24690 2.90347 2.73739 3.18146 2.89801
2.37887 2.77519 2.98518 4.58477 3.61503
0.02625 4.04896 4.77131 0.61958 0.77255 0.48576 0.95510
186 3.35841 4.77029 4.47216 4.11619 3.21164 4.20451 4.71683
2.42879 3.88223 0.68484 3.18550 4.37450 4.61168 4.20806 4.02805
3.80108 3.64584 2.51132 5.15144 3.91438 266 L - - -
2.68618 4.42225 2.77519 2.73123 3.46354 2.40513 3.72494
3.29354 2.67741 2.69355 4.24690 2.90347 2.73739 3.18146 2.89801
2.37887 2.77519 2.98518 4.58477 3.61503
0.02625 4.04896 4.77131 0.61958 0.77255 0.48576 0.95510
187 3.56199 4.84520 4.57261 4.24293 0.92189 4.35073 3.57373
3.17219 4.09362 2.49562 3.78796 4.08541 4.67443 4.11535 4.15545
3.73114 3.79405 3.12850 3.73201 1.80594 267 f - - -
2.68618 4.42225 2.77519 2.73123 3.46354 2.40513 3.72494
3.29354 2.67741 2.69355 4.24690 2.90347 2.73739 3.18146 2.89801
2.37887 2.77519 2.98518 4.58477 3.61503
0.02625 4.04896 4.77131 0.61958 0.77255 0.48576 0.95510
188 1.09381 4.21071 3.76821 3.42498 3.97671 3.16698 4.35510
2.87701 3.36443 2.87965 3.92093 3.53931 3.87622 3.68294 3.62926
2.61585 2.83822 2.27190 5.50903 4.28638 268 a - - -
2.68618 4.42225 2.77519 2.73123 3.46354 2.40513 3.72494
3.29354 2.67741 2.69355 4.24690 2.90347 2.73739 3.18146 2.89801
2.37887 2.77519 2.98518 4.58477 3.61503
0.02625 4.04896 4.77131 0.61958 0.77255 0.48576 0.95510
189 3.03267 5.01648 3.03125 2.79233 4.20781 3.49003 3.95095
3.86917 2.60165 3.35356 4.37991 3.25232 4.04822 0.94997 2.88086
3.07456 3.33113 3.58887 5.44998 4.15689 269 q - - -
2.68618 4.42225 2.77519 2.73123 3.46354 2.40513 3.72494
3.29354 2.67741 2.69355 4.24690 2.90347 2.73739 3.18146 2.89801
2.37887 2.77519 2.98518 4.58477 3.61503
0.02625 4.04896 4.77131 0.61958 0.77255 0.48576 0.95510
190 0.93026 4.20038 3.50871 3.28895 4.33518 2.53686 4.34617
3.64906 3.36359 3.43221 4.30442 3.40455 3.75395 3.65234 3.63793
2.47878 2.78907 3.14491 5.69056 4.50484 270 a - - -
2.68618 4.42225 2.77519 2.73123 3.46354 2.40513 3.72494
3.29354 2.67741 2.69355 4.24690 2.90347 2.73739 3.18146 2.89801
2.37887 2.77519 2.98518 4.58477 3.61503

```
          0.02625  4.04896  4.77131  0.61958  0.77255  0.48576  0.95510
    191   1.17269  4.21706  3.79777  3.43160  3.92510  3.21531  4.35007
2.76618  3.36699  2.80869  3.85845  3.56138  3.90620  3.68216  3.63538
2.65514  2.85263  2.09611  5.47327  4.25245    271 a - - -
          2.68618  4.42225  2.77519  2.73123  3.46354  2.40513  3.72494
3.29354  2.67741  2.69355  4.24690  2.90347  2.73739  3.18146  2.89801
2.37887  2.77519  2.98518  4.58477  3.61503
          0.02625  4.04896  4.77131  0.61958  0.77255  0.48576  0.95510
    192   2.53421  4.58300  2.91737  2.64961  4.23274  3.19799  3.91103
3.62597  2.75928  3.31292  4.17401  1.76427  3.81738  3.11753  3.14705
2.63107  1.84683  3.22835  5.53656  4.21876    272 n - - -
          2.68618  4.42225  2.77519  2.73123  3.46354  2.40513  3.72494
3.29354  2.67741  2.69355  4.24690  2.90347  2.73739  3.18146  2.89801
2.37887  2.77519  2.98518  4.58477  3.61503
          0.02625  4.04896  4.77131  0.61958  0.77255  0.48576  0.95510
    193   3.34930  4.69244  4.82275  4.26818  2.67918  4.55611  4.74643
2.24509  4.12801  0.83100  2.61738  4.53027  4.73539  4.23244  4.25578
3.90039  3.56776  2.39920  5.03597  3.85232    273 l - - -
          2.68618  4.42225  2.77519  2.73123  3.46354  2.40513  3.72494
3.29354  2.67741  2.69355  4.24690  2.90347  2.73739  3.18146  2.89801
2.37887  2.77519  2.98518  4.58477  3.61503
          0.02625  4.04896  4.77131  0.61958  0.77255  0.48576  0.95510
    194   2.89476  4.94469  2.86613  2.62984  3.60150  3.44462  1.37592
3.79301  2.57459  3.33099  4.25708  2.73319  3.98324  3.06743  2.89612
2.92271  3.18182  3.47060  5.03736  3.51268    274 h - - -
          2.68618  4.42225  2.77519  2.73123  3.46354  2.40513  3.72494
3.29354  2.67741  2.69355  4.24690  2.90347  2.73739  3.18146  2.89801
2.37887  2.77519  2.98518  4.58477  3.61503
          0.02625  4.04896  4.77131  0.61958  0.77255  0.48576  0.95510
    195   3.35841  4.77029  4.47216  4.11619  3.21164  4.20451  4.71683
2.42879  3.88223  0.68484  3.18550  4.37450  4.61168  4.20806  4.02805
3.80108  3.64584  2.51132  5.15144  3.91438    275 L - - -
          2.68618  4.42225  2.77519  2.73123  3.46354  2.40513  3.72494
3.29354  2.67741  2.69355  4.24690  2.90347  2.73739  3.18146  2.89801
2.37887  2.77519  2.98518  4.58477  3.61503
          0.02625  4.04896  4.77131  0.61958  0.77255  0.48576  0.95510
    196   2.29694  4.23184  3.42813  3.11148  4.20948  3.02879  4.17541
3.55384  3.11733  3.29636  4.15647  3.31254  3.74818  3.44472  3.43147
1.69131  1.45720  3.08959  5.57316  4.34003    276 t - - -
          2.68618  4.42225  2.77519  2.73123  3.46354  2.40513  3.72494
3.29354  2.67741  2.69355  4.24690  2.90347  2.73739  3.18146  2.89801
2.37887  2.77519  2.98518  4.58477  3.61503
          0.02625  4.04896  4.77131  0.61958  0.77255  0.48576  0.95510
    197   3.49106  4.84211  4.48107  4.23211  0.76177  4.13322  3.96743
2.99649  4.13553  2.32725  3.70244  4.22219  4.59014  4.25557  4.21149
3.74980  3.79345  2.99963  4.16985  2.51707    277 f - - -
          2.68618  4.42225  2.77519  2.73123  3.46354  2.40513  3.72494
3.29354  2.67741  2.69355  4.24690  2.90347  2.73739  3.18146  2.89801
2.37887  2.77519  2.98518  4.58477  3.61503
          0.02625  4.04896  4.77131  0.61958  0.77255  0.48576  0.95510
    198   3.17113  4.46972  4.87590  4.35644  3.47175  4.54068  5.02842
1.11922  4.23445  1.79041  3.27192  4.58874  4.80235  4.46560  4.41533
3.91549  3.42273  1.51509  5.47722  4.30307    278 i - - -
```

```
          2.68618  4.42225  2.77519  2.73123  3.46354  2.40513  3.72494
 3.29354  2.67741  2.69355  4.24690  2.90347  2.73739  3.18146  2.89801
 2.37887  2.77519  2.98518  4.58477  3.61503
          0.02625  4.04896  4.77131  0.61958  0.77255  0.48576  0.95510
    199   3.21415  5.03434  3.72375  3.20215  4.43412  3.66389  3.96639
 3.99095  2.32798  3.47493  4.47340  3.54687  4.16051  3.18049  0.73952
 3.29160  3.46976  3.70799  5.44719  4.32628    279 r - - -
          2.68618  4.42225  2.77519  2.73123  3.46354  2.40513  3.72494
 3.29354  2.67741  2.69355  4.24690  2.90347  2.73739  3.18146  2.89801
 2.37887  2.77519  2.98518  4.58477  3.61503
          0.02625  4.04896  4.77131  0.61958  0.77255  0.48576  0.95510
    200   2.97976  5.28862  0.94499  2.28815  4.78347  2.75392  3.94392
 4.34022  3.01601  3.89296  4.78384  2.85296  3.91324  3.14417  3.58647
 2.91958  3.32065  3.90189  5.95492  4.55805    280 d - - -
          2.68618  4.42225  2.77519  2.73123  3.46354  2.40513  3.72494
 3.29354  2.67741  2.69355  4.24690  2.90347  2.73739  3.18146  2.89801
 2.37887  2.77519  2.98518  4.58477  3.61503
          0.02625  4.04896  4.77131  0.61958  0.77255  0.48576  0.95510
    201   3.10202  4.42712  4.79481  4.30647  3.60272  4.43081  5.03127
 1.47909  4.19086  2.11567  3.41397  4.52265  4.76081  4.46620  4.39392
 3.82399  3.37400  1.01310  5.56315  4.34806    281 v - - -
          2.68618  4.42225  2.77519  2.73123  3.46354  2.40513  3.72494
 3.29354  2.67741  2.69355  4.24690  2.90347  2.73739  3.18146  2.89801
 2.37887  2.77519  2.98518  4.58477  3.61503
          0.02625  4.04896  4.77131  0.61958  0.77255  0.48576  0.95510
    202   3.17037  4.54472  4.57938  4.20466  3.49871  4.25159  4.90309
 0.88271  4.03620  2.05240  3.41046  4.42573  4.66901  4.36848  4.21304
 3.76696  3.46771  1.86689  5.42361  4.17486    282 i - - -
          2.68618  4.42225  2.77519  2.73123  3.46354  2.40513  3.72494
 3.29354  2.67741  2.69355  4.24690  2.90347  2.73739  3.18146  2.89801
 2.37887  2.77519  2.98518  4.58477  3.61503
          0.02625  4.04896  4.77131  0.61958  0.77255  0.48576  0.95510
    203   3.26307  4.57714  4.86258  4.33898  3.24464  4.55497  4.93212
 1.34671  4.17354  1.22226  3.06422  4.58519  4.78297  4.36862  4.32114
 3.93133  3.50320  1.95074  5.29612  4.14868    283 l - - -
          2.68618  4.42225  2.77519  2.73123  3.46354  2.40513  3.72494
 3.29354  2.67741  2.69355  4.24690  2.90347  2.73739  3.18146  2.89801
 2.37887  2.77519  2.98518  4.58477  3.61503
          0.02625  4.04896  4.77131  0.61958  0.77255  0.48576  0.95510
    204   2.83361  5.03776  2.65223  2.43361  3.99099  3.36856  2.72527
 3.88063  2.55377  3.41909  4.29300  1.47116  3.90778  2.96028  2.94545
 2.82657  3.11303  3.52366  5.33288  3.85904    284 n - - -
          2.68618  4.42225  2.77519  2.73123  3.46354  2.40513  3.72494
 3.29354  2.67741  2.69355  4.24690  2.90347  2.73739  3.18146  2.89801
 2.37887  2.77519  2.98518  4.58477  3.61503
          0.02625  4.04896  4.77131  0.61958  0.77255  0.48576  0.95510
    205   0.78651  4.28895  3.64987  3.45337  4.23595  3.10799  4.45342
 3.38847  3.47438  3.26160  4.27686  3.55577  3.86427  3.79994  3.71033
 2.63917  2.92896  3.01205  5.64533  4.46640    285 a - - -
          2.68618  4.42225  2.77519  2.73123  3.46354  2.40513  3.72494
 3.29354  2.67741  2.69355  4.24690  2.90347  2.73739  3.18146  2.89801
 2.37887  2.77519  2.98518  4.58477  3.61503
          0.02625  4.04896  4.77131  0.61958  0.77255  0.48576  0.95510
```

```
    206   2.97159  5.28389  0.96088  2.28457  4.78149  2.70976  3.93868
4.33553  3.00792  3.88709  4.77502  2.84909  3.90967  3.13755  3.57848
2.91203  3.31213  3.89587  5.95452  4.55483    286 d - - -
          2.68618  4.42225  2.77519  2.73123  3.46354  2.40513  3.72494
3.29354  2.67741  2.69355  4.24690  2.90347  2.73739  3.18146  2.89801
2.37887  2.77519  2.98518  4.58477  3.61503
          0.02625  4.04896  4.77131  0.61958  0.77255  0.48576  0.95510
    207   2.97292  5.46496  2.01419  1.09206  4.76493  3.26907  3.80807
4.26693  2.79703  3.79562  4.65932  2.75304  3.88426  2.97896  3.35838
2.87193  3.26179  3.85995  5.94081  4.47378    287 e - - -
          2.68618  4.42225  2.77519  2.73123  3.46354  2.40513  3.72494
3.29354  2.67741  2.69355  4.24690  2.90347  2.73739  3.18146  2.89801
2.37887  2.77519  2.98518  4.58477  3.61503
          0.02625  4.04896  4.77131  0.61958  0.77255  0.48576  0.95510
    208   3.64516  4.95409  4.37154  4.12363  2.86887  3.93677  4.13419
3.67984  3.84388  3.04277  4.30491  4.23792  4.46991  4.22231  3.94004
3.84703  3.95524  3.57496  0.59379  2.86228    288 W - - -
          2.68618  4.42225  2.77519  2.73123  3.46354  2.40513  3.72494
3.29354  2.67741  2.69355  4.24690  2.90347  2.73739  3.18146  2.89801
2.37887  2.77519  2.98518  4.58477  3.61503
          0.02625  4.04896  4.77131  0.61958  0.77255  0.48576  0.95510
    209   2.49901  4.59438  2.77236  2.57903  4.43357  1.73243  3.94469
3.91129  2.85770  3.51550  4.33716  2.01598  3.77879  3.14205  3.28809
2.29407  2.90296  3.41752  5.70031  4.37053    289 g - - -
          2.68618  4.42225  2.77519  2.73123  3.46354  2.40513  3.72494
3.29354  2.67741  2.69355  4.24690  2.90347  2.73739  3.18146  2.89801
2.37887  2.77519  2.98518  4.58477  3.61503
          0.02625  4.04896  4.77131  0.61958  0.77255  0.48576  0.95510
    210   3.19952  4.53012  4.75869  4.27928  3.38547  4.46829  4.93002
1.01981  4.09330  1.75395  3.24708  4.51778  4.76559  4.37786  4.27188
3.87300  3.46054  1.79750  5.38410  4.15523    290 i - - -
          2.68618  4.42225  2.77519  2.73123  3.46354  2.40513  3.72494
3.29354  2.67741  2.69355  4.24690  2.90347  2.73739  3.18146  2.89801
2.37887  2.77519  2.98518  4.58477  3.61503
          0.02625  4.04896  4.77131  0.61958  0.77255  0.48576  0.95510
    211   2.36122  4.34021  3.20894  2.94011  4.29542  3.05310  4.10864
3.75417  3.02633  3.41534  4.25910  3.22644  1.75808  3.35233  3.37381
1.57625  2.81396  3.25312  5.61627  4.35279    291 s - - -
          2.68618  4.42225  2.77519  2.73123  3.46354  2.40513  3.72494
3.29354  2.67741  2.69355  4.24690  2.90347  2.73739  3.18146  2.89801
2.37887  2.77519  2.98518  4.58477  3.61503
          0.02625  4.04896  4.77131  0.61958  0.77255  0.48576  0.95510
    212   1.83969  4.82028  2.81746  2.07187  4.16041  3.35865  3.69562
3.54402  2.33653  3.16869  4.00088  2.94566  3.83141  2.85906  2.91445
2.68006  2.65677  3.20986  5.41179  4.08245    292 a - - -
          2.68618  4.42225  2.77519  2.73123  3.46354  2.40513  3.72494
3.29354  2.67741  2.69355  4.24690  2.90347  2.73739  3.18146  2.89801
2.37887  2.77519  2.98518  4.58477  3.61503
          0.02625  4.04896  4.77131  0.61958  0.77255  0.48576  0.95510
    213   1.17655  4.54522  2.49104  2.68336  4.25252  3.17281  4.00635
3.54921  2.96645  3.31181  4.20024  3.10262  3.82641  3.23404  3.38484
2.64539  2.93063  3.16644  5.59233  4.29233    293 a - - -
          2.68618  4.42225  2.77519  2.73123  3.46354  2.40513  3.72494
3.29354  2.67741  2.69355  4.24690  2.90347  2.73739  3.18146  2.89801
2.37887  2.77519  2.98518  4.58477  3.61503
```

```
          0.02625  4.04896  4.77131  0.61958  0.77255  0.48576  0.95510
    214   2.65623  4.73058  2.98558  2.60872  4.14847  3.35152  3.78356
3.53025  2.49371  3.15099  4.04242  3.06717  3.87203  1.85475  2.84342
2.72630  1.95081  3.20477  5.41938  4.11690    294 q - - -
          2.68618  4.42225  2.77519  2.73123  3.46354  2.40513  3.72494
3.29354  2.67741  2.69355  4.24690  2.90347  2.73739  3.18146  2.89801
2.37887  2.77519  2.98518  4.58477  3.61503
          0.02625  4.04896  4.77131  0.61958  0.77255  0.48576  0.95510
    215   3.19269  4.59401  4.63367  4.13656  3.20522  4.34068  4.77267
2.11621  3.95159  0.95622  3.05481  4.38904  4.65173  4.19297  4.12347
3.72837  3.45552  1.84354  5.22179  4.05102    295 l - - -
          2.68618  4.42225  2.77519  2.73123  3.46354  2.40513  3.72494
3.29354  2.67741  2.69355  4.24690  2.90347  2.73739  3.18146  2.89801
2.37887  2.77519  2.98518  4.58477  3.61503
          0.02625  4.04896  4.77131  0.61958  0.77255  0.48576  0.95510
    216   2.74142  5.08322  2.51259  2.35392  4.41556  3.37357  3.65333
3.87901  2.36198  3.40375  4.21059  2.03612  3.84270  2.79067  1.97473
2.71735  2.98671  3.49772  5.55048  4.18557    296 r - - -
          2.68618  4.42225  2.77519  2.73123  3.46354  2.40513  3.72494
3.29354  2.67741  2.69355  4.24690  2.90347  2.73739  3.18146  2.89801
2.37887  2.77519  2.98518  4.58477  3.61503
          0.02625  4.04896  4.77131  0.61958  0.77255  0.48576  0.95510
    217   2.51696  4.36119  3.59478  3.35937  4.13457  3.19354  4.36194
3.28365  3.30583  3.14078  4.18273  3.53737  3.91252  3.69255  3.55365
2.72057  0.90433  2.95928  5.55663  4.35230    297 t - - -
          2.68618  4.42225  2.77519  2.73123  3.46354  2.40513  3.72494
3.29354  2.67741  2.69355  4.24690  2.90347  2.73739  3.18146  2.89801
2.37887  2.77519  2.98518  4.58477  3.61503
          0.02625  4.04896  4.77131  0.61958  0.77255  0.48576  0.95510
    218   2.91747  3.35659  4.06261  3.61366  2.58456  3.77263  3.79870
2.94708  3.43894  2.59977  3.68070  3.74955  4.24070  3.71330  3.63965
3.16297  3.21305  2.76863  4.21453  1.16038    298 y - - -
          2.68618  4.42225  2.77519  2.73123  3.46354  2.40513  3.72494
3.29354  2.67741  2.69355  4.24690  2.90347  2.73739  3.18146  2.89801
2.37887  2.77519  2.98518  4.58477  3.61503
          0.02625  4.04896  4.77131  0.61958  0.77255  0.48576  0.95510
    219   2.94469  5.10026  3.36353  2.71965  4.52953  3.61877  3.62268
3.87997  1.50584  3.36134  4.21050  3.15037  3.99434  2.75591  1.62369
2.94797  2.76926  3.55118  5.45071  4.24490    299 k - - -
          2.68618  4.42225  2.77519  2.73123  3.46354  2.40513  3.72494
3.29354  2.67741  2.69355  4.24690  2.90347  2.73739  3.18146  2.89801
2.37887  2.77519  2.98518  4.58477  3.61503
          0.02625  4.04896  4.77131  0.61958  0.77255  0.48576  0.95510
    220   2.72564  5.15695  2.01238  2.13728  4.47601  2.95837  3.64296
3.94207  2.42813  3.45502  4.24472  2.81943  3.81458  2.77236  2.09690
2.68184  2.97133  3.54222  5.61466  4.21341    300 d - - -
          2.68618  4.42225  2.77519  2.73123  3.46354  2.40513  3.72494
3.29354  2.67741  2.69355  4.24690  2.90347  2.73739  3.18146  2.89801
2.37887  2.77519  2.98518  4.58477  3.61503
          0.02625  4.04896  4.77131  0.61958  0.77255  0.48576  0.95510
    221   3.33031  4.89506  3.83241  3.46354  2.20491  4.02883  2.32850
3.51801  3.28993  2.93258  4.05992  3.65883  4.41305  3.60765  3.52484
3.39444  3.56214  3.34465  3.85184  1.11495    301 y - - -
```

2.68618 4.42225 2.77519 2.73123 3.46354 2.40513 3.72494
3.29354 2.67741 2.69355 4.24690 2.90347 2.73739 3.18146 2.89801
2.37887 2.77519 2.98518 4.58477 3.61503
0.02625 4.04896 4.77131 0.61958 0.77255 0.48576 0.95510
222 3.36441 4.71948 4.74225 4.24871 2.53072 4.48761 4.59854
2.29882 4.09577 0.81928 2.95604 4.47037 4.72220 4.22929 4.22516
3.87237 3.59862 2.43091 4.87270 3.52389 302 l - - -
2.68618 4.42225 2.77519 2.73123 3.46354 2.40513 3.72494
3.29354 2.67741 2.69355 4.24690 2.90347 2.73739 3.18146 2.89801
2.37887 2.77519 2.98518 4.58477 3.61503
0.02625 4.04896 4.77131 0.61958 0.77255 0.48576 0.95510
223 3.08730 5.19693 3.48341 2.82665 4.65824 3.69028 3.63996
4.00768 1.14294 3.44498 4.31905 3.22660 4.06307 2.77542 1.77375
3.08131 3.25410 3.68885 5.47961 4.31534 303 k - - -
2.68618 4.42225 2.77519 2.73123 3.46354 2.40513 3.72494
3.29354 2.67741 2.69355 4.24690 2.90347 2.73739 3.18146 2.89801
2.37887 2.77519 2.98518 4.58477 3.61503
0.02625 4.04896 4.77131 0.61958 0.77255 0.48576 0.95510
224 2.73811 5.13408 2.83634 2.11646 4.47923 3.42342 3.59748
3.92027 2.12029 3.40705 4.19425 2.32695 3.84128 2.28003 2.27919
2.70428 2.96003 3.52936 5.53331 4.18582 304 e - - -
2.68618 4.42225 2.77519 2.73123 3.46354 2.40513 3.72494
3.29354 2.67741 2.69355 4.24690 2.90347 2.73739 3.18146 2.89801
2.37887 2.77519 2.98518 4.58477 3.61503
0.02625 4.04896 4.77131 0.61958 0.77255 0.48576 0.95510
225 3.59516 4.90432 4.45994 4.13688 1.84118 4.32018 3.51579
3.34420 3.99129 2.69983 3.96519 4.01659 4.66070 4.05329 4.08798
3.70388 3.83071 3.26020 3.68423 0.85669 305 y - - -
2.68618 4.42225 2.77519 2.73123 3.46354 2.40513 3.72494
3.29354 2.67741 2.69355 4.24690 2.90347 2.73739 3.18146 2.89801
2.37887 2.77519 2.98518 4.58477 3.61503
0.02625 4.04896 4.77131 0.61958 0.77255 0.48576 0.95510
226 2.59963 4.25656 3.61860 3.06833 3.41094 3.58993 3.46604
2.40651 2.97339 2.43195 3.38596 3.42525 3.99786 3.29372 3.28392
2.87264 2.18143 1.85985 4.90676 3.67398 306 v - - -
2.68618 4.42225 2.77519 2.73123 3.46354 2.40513 3.72494
3.29354 2.67741 2.69355 4.24690 2.90347 2.73739 3.18146 2.89801
2.37887 2.77519 2.98518 4.58477 3.61503
0.02625 4.04896 4.77131 0.61958 0.77255 0.48576 0.95510
227 1.88096 4.53133 3.31152 2.77810 3.94931 3.37990 3.80712
3.22629 2.52515 2.94153 3.83683 3.18363 3.87459 3.01423 1.97461
2.70001 2.87305 2.63726 5.27414 4.01528 307 a - - -
2.68618 4.42225 2.77519 2.73123 3.46354 2.40513 3.72494
3.29354 2.67741 2.69355 4.24690 2.90347 2.73739 3.18146 2.89801
2.37887 2.77519 2.98518 4.58477 3.61503
0.02625 4.04896 4.77131 0.61958 0.77255 0.48576 0.95510
228 2.81670 5.34989 1.73221 1.91882 4.65340 3.29849 3.68328
4.14753 2.32551 3.63281 4.42949 2.75599 3.82862 2.25634 3.06316
2.73638 3.07243 3.72349 5.78085 4.33057 308 d - - -
2.68618 4.42225 2.77519 2.73123 3.46354 2.40513 3.72494
3.29354 2.67741 2.69355 4.24690 2.90347 2.73739 3.18146 2.89801
2.37887 2.77519 2.98518 4.58477 3.61503
0.02625 4.04896 4.77131 0.61958 0.77255 0.48576 0.95510

229 3.39982 4.90783 3.91400 3.58021 2.15696 4.07627 2.80990
3.53108 3.40014 2.92897 4.08237 3.72551 4.46466 3.69851 3.60753
3.46580 3.63573 3.36950 3.80900 0.95736 309 y - - -
2.68618 4.42225 2.77519 2.73123 3.46354 2.40513 3.72494
3.29354 2.67741 2.69355 4.24690 2.90347 2.73739 3.18146 2.89801
2.37887 2.77519 2.98518 4.58477 3.61503
0.02625 4.04896 4.77131 0.61958 0.77255 0.48576 0.95510
230 2.41035 4.33126 3.36913 3.19663 4.25684 3.05832 4.29982
3.80508 3.28244 3.51284 4.42630 3.39050 3.81243 3.61846 3.56056
0.85292 2.90434 3.30106 5.61579 4.34228 310 s - - -
2.68618 4.42225 2.77519 2.73123 3.46354 2.40513 3.72494
3.29354 2.67741 2.69355 4.24690 2.90347 2.73739 3.18146 2.89801
2.37887 2.77519 2.98518 4.58477 3.61503
0.02625 4.04896 4.77131 0.61958 0.77255 0.48576 0.95510
231 2.87931 4.87574 2.82111 2.72621 4.26801 3.31004 4.07758
4.02015 2.99819 3.64783 4.60465 0.87973 3.97208 3.35350 3.35640
2.94155 3.26875 3.62429 5.55383 4.20588 311 n - - -
2.68618 4.42225 2.77519 2.73123 3.46354 2.40513 3.72494
3.29354 2.67741 2.69355 4.24690 2.90347 2.73739 3.18146 2.89801
2.37887 2.77519 2.98518 4.58477 3.61503
0.02625 4.04896 4.77131 0.61958 0.77255 0.48576 0.95510
232 3.51130 4.92842 4.10319 3.86868 2.32121 4.03918 3.71497
3.49696 3.72133 2.90449 4.15421 3.94722 4.51032 3.98439 3.86748
3.62915 3.79964 3.37626 3.97068 0.71613 312 y - - -
2.68618 4.42225 2.77519 2.73123 3.46354 2.40513 3.72494
3.29354 2.67741 2.69355 4.24690 2.90347 2.73739 3.18146 2.89801
2.37887 2.77519 2.98518 4.58477 3.61503
0.02625 4.04896 4.77131 0.61958 0.77255 0.48576 0.95510
233 1.47218 1.70069 4.07458 3.71362 3.95887 3.06757 4.44223
3.02457 3.58852 2.97781 3.93463 3.62481 3.81314 3.85590 3.77585
2.52821 2.75993 2.68273 5.44666 4.27043 313 a - - -
2.68618 4.42225 2.77519 2.73123 3.46354 2.40513 3.72494
3.29354 2.67741 2.69355 4.24690 2.90347 2.73739 3.18146 2.89801
2.37887 2.77519 2.98518 4.58477 3.61503
0.02625 4.04896 4.77131 0.61958 0.77255 0.48576 0.95510
234 3.27464 4.59046 4.86492 4.33899 3.22250 4.55949 4.92665
1.42608 4.17244 1.14571 3.04017 4.58774 4.78204 4.35995 4.31680
3.93471 3.51311 1.98650 5.28117 4.14101 314 l - - -
2.68618 4.42225 2.77519 2.73123 3.46354 2.40513 3.72494
3.29354 2.67741 2.69355 4.24690 2.90347 2.73739 3.18146 2.89801
2.37887 2.77519 2.98518 4.58477 3.61503
0.02625 4.04896 4.77131 0.61958 0.77255 0.48576 0.95510
235 2.47895 4.56337 2.90941 2.61484 4.31003 3.15670 3.89067
3.74885 2.75134 3.37349 4.19575 1.90887 3.77777 3.07995 3.16681
1.76644 2.52467 3.30056 5.58188 4.26632 315 s - - -
2.68618 4.42225 2.77519 2.73123 3.46354 2.40513 3.72494
3.29354 2.67741 2.69355 4.24690 2.90347 2.73739 3.18146 2.89801
2.37887 2.77519 2.98518 4.58477 3.61503
0.02625 4.04896 4.77131 0.61958 0.77255 0.48576 0.95510
236 2.39062 4.29882 3.41374 3.11583 4.15079 3.10913 4.17906
3.42518 3.09751 3.19212 4.11275 3.34835 3.10681 3.45753 3.39800
2.57045 1.14026 3.03206 5.53026 4.30678 316 t - - -
2.68618 4.42225 2.77519 2.73123 3.46354 2.40513 3.72494
3.29354 2.67741 2.69355 4.24690 2.90347 2.73739 3.18146 2.89801
2.37887 2.77519 2.98518 4.58477 3.61503

```
          0.02625  4.04896  4.77131  0.61958  0.77255  0.48576  0.95510
    237   3.51130  4.92842  4.10319  3.86868  2.32121  4.03918  3.71497
3.49696  3.72133  2.90449  4.15421  3.94722  4.51032  3.98439  3.86748
3.62915  3.79964  3.37626  3.97068  0.71613    317 y - - -
          2.68618  4.42225  2.77519  2.73123  3.46354  2.40513  3.72494
3.29354  2.67741  2.69355  4.24690  2.90347  2.73739  3.18146  2.89801
2.37887  2.77519  2.98518  4.58477  3.61503
          0.02625  4.04896  4.77131  0.61958  0.77255  0.48576  0.95510
    238   2.84902  5.38499  1.87059  1.81944  4.67977  3.28701  3.70673
4.17941  2.59853  3.66948  4.47807  2.74567  3.83797  1.91396  3.13227
2.76216  3.11122  3.75756  5.82198  4.36109    318 e - - -
          2.68618  4.42225  2.77519  2.73123  3.46354  2.40513  3.72494
3.29354  2.67741  2.69355  4.24690  2.90347  2.73739  3.18146  2.89801
2.37887  2.77519  2.98518  4.58477  3.61503
          0.02625  4.04896  4.77131  0.61958  0.77255  0.48576  0.95510
    239   2.65253  4.99843  2.50474  2.10890  4.29977  3.04438  3.63340
3.74684  2.31343  3.30008  4.09490  2.85013  3.79768  2.76832  2.92906
2.38653  2.18082  3.37362  5.49726  4.11648    319 e - - -
          2.68618  4.42225  2.77519  2.73123  3.46354  2.40513  3.72494
3.29354  2.67741  2.69355  4.24690  2.90347  2.73739  3.18146  2.89801
2.37887  2.77519  2.98518  4.58477  3.61503
          0.02625  4.04896  4.77131  0.61958  0.77255  0.48576  0.95510
    240   1.50448  4.66160  2.79296  2.12952  4.23120  2.81012  3.84622
3.61082  2.72648  3.26844  4.11438  3.00028  3.81688  3.04013  3.15685
2.64940  2.91589  3.23364  5.52206  4.20705    320 a - - -
          2.68618  4.42225  2.77519  2.73123  3.46354  2.40513  3.72494
3.29354  2.67741  2.69355  4.24690  2.90347  2.73739  3.18146  2.89801
2.37887  2.77519  2.98518  4.58477  3.61503
          0.02625  4.04896  4.77131  0.61958  0.77255  0.48576  0.95510
    241   3.49106  4.84211  4.48107  4.23211  0.76177  4.13322  3.96743
2.99649  4.13553  2.32725  3.70244  4.22219  4.59014  4.25557  4.21149
3.74980  3.79345  2.99963  4.16985  2.51707    321 f - - -
          2.68618  4.42225  2.77519  2.73123  3.46354  2.40513  3.72494
3.29354  2.67741  2.69355  4.24690  2.90347  2.73739  3.18146  2.89801
2.37887  2.77519  2.98518  4.58477  3.61503
          0.02625  4.04896  4.77131  0.61958  0.77255  0.48576  0.95510
    242   3.11684  5.19453  3.55895  2.87281  4.66100  3.71431  3.64325
4.00275  1.57142  3.43936  4.32069  3.25734  4.08186  2.78082  1.23126
3.11585  3.27770  3.69184  5.47062  4.32066    322 r - - -
          2.68618  4.42225  2.77519  2.73123  3.46354  2.40513  3.72494
3.29354  2.67741  2.69355  4.24690  2.90347  2.73739  3.18146  2.89801
2.37887  2.77519  2.98518  4.58477  3.61503
          0.02625  4.04896  4.77131  0.61958  0.77255  0.48576  0.95510
    243   1.47301  4.18210  3.47333  3.17248  4.30657  1.70904  4.24008
3.66000  3.22838  3.38904  4.22273  3.32726  3.71590  3.51141  3.54424
2.42265  2.36743  3.13836  5.65674  4.44975    323 a - - -
          2.68618  4.42225  2.77519  2.73123  3.46354  2.40513  3.72494
3.29354  2.67741  2.69355  4.24690  2.90347  2.73739  3.18146  2.89801
2.37887  2.77519  2.98518  4.58477  3.61503
          0.02625  4.04896  4.77131  0.61958  0.77255  0.48576  0.95510
    244   2.89372  4.70816  3.61378  3.01285  3.69814  3.69340  3.83720
3.05904  2.39375  1.76216  3.67972  3.39479  4.08962  3.07738  1.69338
3.04407  3.12062  2.91063  5.13448  3.87274    324 r - - -
```

```
          2.68618  4.42225  2.77519  2.73123  3.46354  2.40513  3.72494
3.29354  2.67741  2.69355  4.24690  2.90347  2.73739  3.18146  2.89801
2.37887  2.77519  2.98518  4.58477  3.61503
          0.28203  4.04896  1.47705  0.61958  0.77255  0.48576  0.95510
    245   3.16951  4.59310  4.13961  3.84197  1.13707  3.87308  3.84981
2.70376  3.75114  2.09133  3.44575  3.93927  4.33454  3.93945  3.88858
3.44217  3.47125  2.68886  4.12308  2.50029    325 f - - -
          2.68618  4.42225  2.77519  2.73123  3.46354  2.40513  3.72494
3.29354  2.67741  2.69355  4.24690  2.90347  2.73739  3.18146  2.89801
2.37887  2.77519  2.98518  4.58477  3.61503
          0.03377  3.80070  4.52305  0.61958  0.77255  0.70047  0.68588
    246   3.14671  4.66477  3.73360  3.45110  2.30123  3.74753  3.59023
3.16623  3.31068  2.63830  3.84926  3.63737  4.22387  3.63398  3.50845
3.29000  3.43352  3.03363  3.94169  1.09413    326 y - - -
          2.68618  4.42225  2.77519  2.73123  3.46354  2.40513  3.72494
3.29354  2.67741  2.69355  4.24690  2.90347  2.73739  3.18146  2.89801
2.37887  2.77519  2.98518  4.58477  3.61503
          0.03377  3.80070  4.52305  0.61958  0.77255  0.70047  0.68588
    247   2.66198  4.42931  3.26174  3.09185  4.08999  3.12097  4.17388
3.61850  3.14328  3.25257  4.30061  3.40492  0.97259  3.53434  3.39552
2.82764  3.09832  3.27192  5.29473  4.21028    327 p - - -
          2.68618  4.42225  2.77519  2.73123  3.46354  2.40513  3.72494
3.29354  2.67741  2.69355  4.24690  2.90347  2.73739  3.18146  2.89801
2.37887  2.77519  2.98518  4.58477  3.61503
          0.03377  3.80070  4.52305  0.61958  0.77255  0.37168  1.16980
    248   2.84037  5.00547  3.03498  2.60443  4.38444  3.47028  3.69449
3.86351  2.20646  3.38458  4.23531  1.95609  3.93525  2.84991  1.64277
2.85373  3.08604  3.50889  5.46880  4.18108    328 r - - -
          2.68618  4.42225  2.77519  2.73123  3.46354  2.40513  3.72494
3.29354  2.67741  2.69355  4.24690  2.90347  2.73739  3.18146  2.89801
2.37887  2.77519  2.98518  4.58477  3.61503
          0.02625  4.04896  4.77131  0.61958  0.77255  0.48576  0.95510
    249   2.11122  4.65196  2.96235  2.53017  4.04224  3.32312  3.31369
3.45536  2.55735  3.08554  3.91952  2.41036  3.81332  2.91441  2.98218
2.63317  2.13893  3.11662  5.33673  4.02424    329 a - - -
          2.68618  4.42225  2.77519  2.73123  3.46354  2.40513  3.72494
3.29354  2.67741  2.69355  4.24690  2.90347  2.73739  3.18146  2.89801
2.37887  2.77519  2.98518  4.58477  3.61503
          0.02625  4.04896  4.77131  0.61958  0.77255  0.48576  0.95510
    250   2.67070  4.66848  3.30952  2.79372  4.14534  3.40696  3.78892
3.48827  2.36227  3.12625  4.01526  3.19211  3.90889  2.97575  1.79614
2.76968  1.82213  3.16829  5.36793  4.12246    330 r - - -
          2.68618  4.42225  2.77519  2.73123  3.46354  2.40513  3.72494
3.29354  2.67741  2.69355  4.24690  2.90347  2.73739  3.18146  2.89801
2.37887  2.77519  2.98518  4.58477  3.61503
          0.02625  4.04896  4.77131  0.61958  0.77255  0.48576  0.95510
    251   3.19711  4.60020  4.63180  4.13651  3.19973  4.34031  4.77168
2.12417  3.94885  0.94311  3.05074  4.38935  4.65211  4.19145  4.12017
3.72959  3.46047  1.87140  5.21862  4.04673    331 l - - -
          2.68618  4.42225  2.77519  2.73123  3.46354  2.40513  3.72494
3.29354  2.67741  2.69355  4.24690  2.90347  2.73739  3.18146  2.89801
2.37887  2.77519  2.98518  4.58477  3.61503
          0.02625  4.04896  4.77131  0.61958  0.77255  0.48576  0.95510
```

```
    252   2.74712  5.19687  2.31027  1.72433  4.48342  3.32020  2.42731
3.96576  2.49483  3.48268  4.27786  2.79332  3.35166  2.79414  3.00053
2.69474  2.99670  3.56729  5.64707  4.22616    332 e - - -
          2.68618  4.42225  2.77519  2.73123  3.46354  2.40513  3.72494
3.29354  2.67741  2.69355  4.24690  2.90347  2.73739  3.18146  2.89801
2.37887  2.77519  2.98518  4.58477  3.61503
          0.02625  4.04896  4.77131  0.61958  0.77255  0.48576  0.95510
    253   2.93895  5.43435  1.40110  2.15726  4.75317  3.24075  3.80952
4.31363  2.82884  3.82872  4.68381  1.76750  3.86885  2.98293  3.41282
2.84268  3.24211  3.88067  5.95654  4.45917    333 d - - -
          2.68618  4.42225  2.77519  2.73123  3.46354  2.40513  3.72494
3.29354  2.67741  2.69355  4.24690  2.90347  2.73739  3.18146  2.89801
2.37887  2.77519  2.98518  4.58477  3.61503
          0.02625  4.04896  4.77131  0.61958  0.77255  0.48576  0.95510
    254   3.16377  4.62535  4.42781  3.92419  3.14240  4.22022  4.57197
2.29565  3.67496  1.53670  1.34300  4.20576  4.54803  3.98762  3.86379
3.60223  3.42130  2.35878  5.10302  3.89966    334 m - - -
          2.68618  4.42225  2.77519  2.73123  3.46354  2.40513  3.72494
3.29354  2.67741  2.69355  4.24690  2.90347  2.73739  3.18146  2.89801
2.37887  2.77519  2.98518  4.58477  3.61503
          0.02625  4.04896  4.77131  0.61958  0.77255  0.48576  0.95510
    255   3.21617  4.62834  4.61851  4.13429  3.17626  4.33443  4.76396
2.16154  3.93223  0.88994  3.03719  4.38773  4.65228  4.18376  4.10092
3.73291  3.48282  1.99789  5.20271  4.02150    335 l - - -
          2.68618  4.42225  2.77519  2.73123  3.46354  2.40513  3.72494
3.29354  2.67741  2.69355  4.24690  2.90347  2.73739  3.18146  2.89801
2.37887  2.77519  2.98518  4.58477  3.61503
          0.02625  4.04896  4.77131  0.61958  0.77255  0.48576  0.95510
    256   2.88806  5.28362  2.46112  1.37364  4.57844  3.34454  3.72374
4.05497  2.49783  3.56442  4.41321  2.83421  3.88334  2.17263  2.92598
2.82610  3.14483  3.67836  5.71616  4.32087    336 e - - -
          2.68618  4.42225  2.77519  2.73123  3.46354  2.40513  3.72494
3.29354  2.67741  2.69355  4.24690  2.90347  2.73739  3.18146  2.89801
2.37887  2.77519  2.98518  4.58477  3.61503
          0.02625  4.04896  4.77131  0.61958  0.77255  0.48576  0.95510
    257   3.49106  4.84211  4.48107  4.23211  0.76177  4.13322  3.96743
2.99649  4.13553  2.32725  3.70244  4.22219  4.59014  4.25557  4.21149
3.74980  3.79345  2.99963  4.16985  2.51707    337 f - - -
          2.68618  4.42225  2.77519  2.73123  3.46354  2.40513  3.72494
3.29354  2.67741  2.69355  4.24690  2.90347  2.73739  3.18146  2.89801
2.37887  2.77519  2.98518  4.58477  3.61503
          0.02625  4.04896  4.77131  0.61958  0.77255  0.48576  0.95510
    258   3.10579  5.21336  3.53954  2.84670  4.68695  3.71037  3.63167
4.02554  1.31012  3.45221  4.32524  3.23911  4.07225  2.76480  1.48962
3.09755  3.26442  3.70764  5.47933  4.32509    338 k - - -
          2.68618  4.42225  2.77519  2.73123  3.46354  2.40513  3.72494
3.29354  2.67741  2.69355  4.24690  2.90347  2.73739  3.18146  2.89801
2.37887  2.77519  2.98518  4.58477  3.61503
          0.02625  4.04896  4.77131  0.61958  0.77255  0.48576  0.95510
    259   2.51696  4.36119  3.59478  3.35937  4.13457  3.19354  4.36194
3.28365  3.30583  3.14078  4.18273  3.53737  3.91252  3.69255  3.55365
2.72057  0.90433  2.95928  5.55663  4.35230    339 t - - -
          2.68618  4.42225  2.77519  2.73123  3.46354  2.40513  3.72494
3.29354  2.67741  2.69355  4.24690  2.90347  2.73739  3.18146  2.89801
2.37887  2.77519  2.98518  4.58477  3.61503
```

```
          0.02625  4.04896  4.77131  0.61958  0.77255  0.48576  0.95510
    260   3.66068  4.89203  4.65910  4.34988  1.09457  4.41284  3.49624
3.33041  4.19382  2.65569  3.92691  4.09845  4.72064  4.15596  4.22079
3.78383  3.88275  3.26089  3.63423  1.33749    340 f - - -
          2.68618  4.42225  2.77519  2.73123  3.46354  2.40513  3.72494
3.29354  2.67741  2.69355  4.24690  2.90347  2.73739  3.18146  2.89801
2.37887  2.77519  2.98518  4.58477  3.61503
          0.02625  4.04896  4.77131  0.61958  0.77255  0.48576  0.95510
    261   3.16377  4.62535  4.42781  3.92419  3.14240  4.22022  4.57197
2.29565  3.67496  1.53670  1.34300  4.20576  4.54803  3.98762  3.86379
3.60223  3.42130  2.35878  5.10302  3.89966    341 m - - -
          2.68618  4.42225  2.77519  2.73123  3.46354  2.40513  3.72494
3.29354  2.67741  2.69355  4.24690  2.90347  2.73739  3.18146  2.89801
2.37887  2.77519  2.98518  4.58477  3.61503
          0.02625  4.04896  4.77131  0.61958  0.77255  0.48576  0.95510
    262   2.68375  4.33017  3.83302  3.37568  1.95897  3.59485  4.04793
2.65463  3.29521  2.33783  3.45224  3.62400  4.09589  3.57886  3.55669
2.96220  1.80856  2.49697  4.76674  3.35324    342 t - - -
          2.68618  4.42225  2.77519  2.73123  3.46354  2.40513  3.72494
3.29354  2.67741  2.69355  4.24690  2.90347  2.73739  3.18146  2.89801
2.37887  2.77519  2.98518  4.58477  3.61503
          0.02625  4.04896  4.77131  0.61958  0.77255  0.48576  0.95510
    263   3.32545  4.68725  4.74837  4.24177  3.12054  4.50219  4.82783
1.98929  4.02804  0.83685  2.97222  4.51184  4.74422  4.25066  4.17986
3.89209  3.56838  2.22279  5.18844  4.01891    343 l - - -
          2.68618  4.42225  2.77519  2.73123  3.46354  2.40513  3.72494
3.29354  2.67741  2.69355  4.24690  2.90347  2.73739  3.18146  2.89801
2.37887  2.77519  2.98518  4.58477  3.61503
          0.02625  4.04896  4.77131  0.61958  0.77255  0.48576  0.95510
    264   2.87931  4.87574  2.82111  2.72621  4.26801  3.31004  4.07758
4.02015  2.99819  3.64783  4.60465  0.87973  3.97208  3.35350  3.35640
2.94155  3.26875  3.62429  5.55383  4.20588    344 n - - -
          2.68618  4.42225  2.77519  2.73123  3.46354  2.40513  3.72494
3.29354  2.67741  2.69355  4.24690  2.90347  2.73739  3.18146  2.89801
2.37887  2.77519  2.98518  4.58477  3.61503
          0.02625  4.04896  4.77131  0.61958  0.77255  0.48576  0.95510
    265   2.41944  4.31554  4.20877  3.83337  3.74798  3.70710  4.66202
2.07302  3.73281  2.43418  3.62658  3.98202  4.29085  4.05630  3.96028
3.14574  3.10771  1.00745  5.50386  4.27371    345 v - - -
          2.68618  4.42225  2.77519  2.73123  3.46354  2.40513  3.72494
3.29354  2.67741  2.69355  4.24690  2.90347  2.73739  3.18146  2.89801
2.37887  2.77519  2.98518  4.58477  3.61503
          0.02625  4.04896  4.77131  0.61958  0.77255  0.48576  0.95510
    266   3.34945  4.69014  4.76668  4.26763  2.01218  4.47154  4.45428
2.34743  4.12169  0.95980  2.97719  4.43631  4.70238  4.21861  4.22991
3.83993  3.57754  2.47543  4.69926  3.27435    346 l - - -
          2.68618  4.42225  2.77519  2.73123  3.46354  2.40513  3.72494
3.29354  2.67741  2.69355  4.24690  2.90347  2.73739  3.18146  2.89801
2.37887  2.77519  2.98518  4.58477  3.61503
          0.02625  4.04896  4.77131  0.61958  0.77255  0.48576  0.95510
    267   3.01148  5.59618  1.22889  1.63595  4.88004  3.24423  3.81470
4.40705  2.86106  3.90720  4.77366  2.70791  3.88114  2.98653  3.47214
2.88469  3.30629  3.97719  6.05493  4.53968    347 d - - -
```

```
         2.68618  4.42225  2.77519  2.73123  3.46354  2.40513  3.72494
3.29354  2.67741  2.69355  4.24690  2.90347  2.73739  3.18146  2.89801
2.37887  2.77519  2.98518  4.58477  3.61503
         0.02625  4.04896  4.77131  0.61958  0.77255  0.48576  0.95510
    268  3.22419  4.58956  4.45763  3.99090  1.89644  4.19186  3.72209
2.83197  3.84913  1.63690  3.45535  4.00092  4.50268  3.94797  3.96769
3.51484  3.44767  2.77923  3.96040  1.54215    348 y - - -
         2.68618  4.42225  2.77519  2.73123  3.46354  2.40513  3.72494
3.29354  2.67741  2.69355  4.24690  2.90347  2.73739  3.18146  2.89801
2.37887  2.77519  2.98518  4.58477  3.61503
         0.02625  4.04896  4.77131  0.61958  0.77255  0.48576  0.95510
    269  2.94938  4.44697  4.36685  4.03872  3.68500  3.90326  4.81184
2.05807  3.90924  2.34345  3.62647  4.19932  4.45635  4.25783  4.10427
3.42051  3.30815  0.82833  5.49818  4.23935    349 v - - -
         2.68618  4.42225  2.77519  2.73123  3.46354  2.40513  3.72494
3.29354  2.67741  2.69355  4.24690  2.90347  2.73739  3.18146  2.89801
2.37887  2.77519  2.98518  4.58477  3.61503
         0.02625  4.04896  4.77131  0.61958  0.77255  0.48576  0.95510
    270  2.41035  4.33126  3.36913  3.19663  4.25684  3.05832  4.29982
3.80508  3.28244  3.51284  4.42630  3.39050  3.81243  3.61846  3.56056
0.85292  2.90434  3.30106  5.61579  4.34228    350 s - - -
         2.68618  4.42225  2.77519  2.73123  3.46354  2.40513  3.72494
3.29354  2.67741  2.69355  4.24690  2.90347  2.73739  3.18146  2.89801
2.37887  2.77519  2.98518  4.58477  3.61503
         0.02625  4.04896  4.77131  0.61958  0.77255  0.48576  0.95510
    271  3.16453  4.50552  4.74646  4.22042  3.31942  4.43425  4.85363
1.12094  4.05744  1.77259  2.55924  4.46380  4.70632  4.29014  4.22764
3.80142  3.41232  1.88263  5.30986  4.15410    351 i - - -
         2.68618  4.42225  2.77519  2.73123  3.46354  2.40513  3.72494
3.29354  2.67741  2.69355  4.24690  2.90347  2.73739  3.18146  2.89801
2.37887  2.77519  2.98518  4.58477  3.61503
         0.02625  4.04896  4.77131  0.61958  0.77255  0.48576  0.95510
    272  3.64516  4.95409  4.37154  4.12363  2.86887  3.93677  4.13419
3.67984  3.84388  3.04277  4.30491  4.23792  4.46991  4.22231  3.94004
3.84703  3.95524  3.57496  0.59379  2.86228    352 W - - -
         2.68618  4.42225  2.77519  2.73123  3.46354  2.40513  3.72494
3.29354  2.67741  2.69355  4.24690  2.90347  2.73739  3.18146  2.89801
2.37887  2.77519  2.98518  4.58477  3.61503
         0.02625  4.04896  4.77131  0.61958  0.77255  0.48576  0.95510
    273  2.41035  4.33126  3.36913  3.19663  4.25684  3.05832  4.29982
3.80508  3.28244  3.51284  4.42630  3.39050  3.81243  3.61846  3.56056
0.85292  2.90434  3.30106  5.61579  4.34228    353 s - - -
         2.68618  4.42225  2.77519  2.73123  3.46354  2.40513  3.72494
3.29354  2.67741  2.69355  4.24690  2.90347  2.73739  3.18146  2.89801
2.37887  2.77519  2.98518  4.58477  3.61503
         0.02625  4.04896  4.77131  0.61958  0.77255  0.48576  0.95510
    274  3.35841  4.77029  4.47216  4.11619  3.21164  4.20451  4.71683
2.42879  3.88223  0.68484  3.18550  4.37450  4.61168  4.20806  4.02805
3.80108  3.64584  2.51132  5.15144  3.91438    354 L - - -
         2.68618  4.42225  2.77519  2.73123  3.46354  2.40513  3.72494
3.29354  2.67741  2.69355  4.24690  2.90347  2.73739  3.18146  2.89801
2.37887  2.77519  2.98518  4.58477  3.61503
         0.02625  4.04896  4.77131  0.61958  0.77255  0.48576  0.95510
```

```
   275   3.33961  4.68007  4.74263  4.25301  1.72079  4.44028  4.34920
2.38685  4.10530  1.10013  3.02187  4.39066  4.68409  4.19951  4.20965
3.80708  3.56869  2.49988  4.58721  3.11033    355 l - - -
         2.68618  4.42225  2.77519  2.73123  3.46354  2.40513  3.72494
3.29354  2.67741  2.69355  4.24690  2.90347  2.73739  3.18146  2.89801
2.37887  2.77519  2.98518  4.58477  3.61503
         0.02625  4.04896  4.77131  0.61958  0.77255  0.48576  0.95510
   276   3.14130  5.10446  3.32852  2.92133  4.53144  3.60779  3.86884
3.97270  0.81982  3.50099  4.45624  3.33675  4.09839  3.05270  2.48089
3.17368  3.38308  3.67174  5.51602  4.35580    356 k - - -
         2.68618  4.42225  2.77519  2.73123  3.46354  2.40513  3.72494
3.29354  2.67741  2.69355  4.24690  2.90347  2.73739  3.18146  2.89801
2.37887  2.77519  2.98518  4.58477  3.61503
         0.02625  4.04896  4.77131  0.61958  0.77255  0.48576  0.95510
   277   3.51130  4.92842  4.10319  3.86868  2.32121  4.03918  3.71497
3.49696  3.72133  2.90449  4.15421  3.94722  4.51032  3.98439  3.86748
3.62915  3.79964  3.37626  3.97068  0.71613    357 y - - -
         2.68618  4.42225  2.77519  2.73123  3.46354  2.40513  3.72494
3.29354  2.67741  2.69355  4.24690  2.90347  2.73739  3.18146  2.89801
2.37887  2.77519  2.98518  4.58477  3.61503
         0.02625  4.04896  4.77131  0.61958  0.77255  0.48576  0.95510
   278   2.62813  4.60287  3.14653  2.44350  3.78443  3.48798  3.71002
3.09756  2.52168  2.77772  3.30058  3.09182  3.88342  2.17269  2.91335
2.73345  2.86072  2.27788  5.14796  3.86704    358 q - - -
         2.68618  4.42225  2.77519  2.73123  3.46354  2.40513  3.72494
3.29354  2.67741  2.69355  4.24690  2.90347  2.73739  3.18146  2.89801
2.37887  2.77519  2.98518  4.58477  3.61503
         0.02625  4.04896  4.77131  0.61958  0.77255  0.48576  0.95510
   279   2.53582  4.63135  2.78779  2.58097  4.34640  3.15234  3.91908
3.86901  2.80186  3.48255  4.31809  1.72727  3.79909  3.12136  3.21472
1.69340  2.92692  3.40338  5.62983  4.27733    359 s - - -
         2.68618  4.42225  2.77519  2.73123  3.46354  2.40513  3.72494
3.29354  2.67741  2.69355  4.24690  2.90347  2.73739  3.18146  2.89801
2.37887  2.77519  2.98518  4.58477  3.61503
         0.02625  4.04896  4.77131  0.61958  0.77255  0.48576  0.95510
   280   3.35841  4.77029  4.47216  4.11619  3.21164  4.20451  4.71683
2.42879  3.88223  0.68484  3.18550  4.37450  4.61168  4.20806  4.02805
3.80108  3.64584  2.51132  5.15144  3.91438    360 L - - -
         2.68618  4.42225  2.77519  2.73123  3.46354  2.40513  3.72494
3.29354  2.67741  2.69355  4.24690  2.90347  2.73739  3.18146  2.89801
2.37887  2.77519  2.98518  4.58477  3.61503
         0.02625  4.04896  4.77131  0.61958  0.77255  0.48576  0.95510
   281   2.90858  4.34313  4.28493  3.73159  2.47228  3.99000  4.00682
2.51933  3.60612  1.60126  2.60278  3.90717  4.31112  3.78020  3.77197
3.28982  3.13483  2.46902  4.43642  2.01257    361 l - - -
         2.68618  4.42225  2.77519  2.73123  3.46354  2.40513  3.72494
3.29354  2.67741  2.69355  4.24690  2.90347  2.73739  3.18146  2.89801
2.37887  2.77519  2.98518  4.58477  3.61503
         0.02625  4.04896  4.77131  0.61958  0.77255  0.48576  0.95510
   282   3.09232  4.42753  4.76522  4.28012  3.60092  4.40258  5.00997
1.53899  4.16079  2.12008  3.41769  4.49716  4.74353  4.44204  4.36684
3.79720  3.36740  0.98646  5.55520  4.33560    362 v - - -
         2.68618  4.42225  2.77519  2.73123  3.46354  2.40513  3.72494
3.29354  2.67741  2.69355  4.24690  2.90347  2.73739  3.18146  2.89801
2.37887  2.77519  2.98518  4.58477  3.61503
```

```
          0.02625  4.04896  4.77131  0.61958  0.77255  0.48576  0.95510
    283   2.29139  4.23659  3.38288  3.13482  4.30353  2.99636  4.23168
3.68192  3.17729  3.41815  4.28235  3.31721  3.74313  3.50541  3.47847
1.05360  2.42365  3.17342  5.66234  4.41860    363 s - - -
          2.68618  4.42225  2.77519  2.73123  3.46354  2.40513  3.72494
3.29354  2.67741  2.69355  4.24690  2.90347  2.73739  3.18146  2.89801
2.37887  2.77519  2.98518  4.58477  3.61503
          0.02625  4.04896  4.77131  0.61958  0.77255  0.48576  0.95510
    284   2.29479  4.23363  3.42286  3.10356  4.21922  3.02621  4.17177
3.56997  3.11214  3.30659  4.16329  3.30734  3.74537  3.43797  3.42952
1.56582  1.56905  3.10017  5.57917  4.34529    364 s - - -
          2.68618  4.42225  2.77519  2.73123  3.46354  2.40513  3.72494
3.29354  2.67741  2.69355  4.24690  2.90347  2.73739  3.18146  2.89801
2.37887  2.77519  2.98518  4.58477  3.61503
          0.02625  4.04896  4.77131  0.61958  0.77255  0.48576  0.95510
    285   2.29375  4.26383  3.26221  3.04966  4.42406  1.81889  4.22623
3.89872  3.20494  3.56096  4.38159  3.26857  3.72809  3.48598  3.53784
1.29293  2.78373  3.31225  5.73729  4.50157    365 s - - -
          2.68618  4.42225  2.77519  2.73123  3.46354  2.40513  3.72494
3.29354  2.67741  2.69355  4.24690  2.90347  2.73739  3.18146  2.89801
2.37887  2.77519  2.98518  4.58477  3.61503
          0.02625  4.04896  4.77131  0.61958  0.77255  0.48576  0.95510
    286   0.78651  4.28895  3.64987  3.45337  4.23595  3.10799  4.45342
3.38847  3.47438  3.26160  4.27686  3.55577  3.86427  3.79994  3.71033
2.63917  2.92896  3.01205  5.64533  4.46640    366 a - - -
          2.68618  4.42225  2.77519  2.73123  3.46354  2.40513  3.72494
3.29354  2.67741  2.69355  4.24690  2.90347  2.73739  3.18146  2.89801
2.37887  2.77519  2.98518  4.58477  3.61503
          0.02625  4.04896  4.77131  0.61958  0.77255  0.48576  0.95510
    287   2.87931  4.87574  2.82111  2.72621  4.26801  3.31004  4.07758
4.02015  2.99819  3.64783  4.60465  0.87973  3.97208  3.35350  3.35640
2.94155  3.26875  3.62429  5.55383  4.20588    367 n - - -
          2.68618  4.42225  2.77519  2.73123  3.46354  2.40513  3.72494
3.29354  2.67741  2.69355  4.24690  2.90347  2.73739  3.18146  2.89801
2.37887  2.77519  2.98518  4.58477  3.61503
          0.02625  4.04896  4.77131  0.61958  0.77255  0.48576  0.95510
    288   3.35841  4.77029  4.47216  4.11619  3.21164  4.20451  4.71683
2.42879  3.88223  0.68484  3.18550  4.37450  4.61168  4.20806  4.02805
3.80108  3.64584  2.51132  5.15144  3.91438    368 L - - -
          2.68618  4.42225  2.77519  2.73123  3.46354  2.40513  3.72494
3.29354  2.67741  2.69355  4.24690  2.90347  2.73739  3.18146  2.89801
2.37887  2.77519  2.98518  4.58477  3.61503
          0.02625  4.04896  4.77131  0.61958  0.77255  0.48576  0.95510
    289   2.99242  4.65351  3.65152  3.33838  2.51155  3.72692  3.68463
3.29102  3.24564  2.85230  3.92865  3.56966  4.22537  3.57138  3.49078
2.73036  3.29786  3.08260  4.14157  1.10949    369 y - - -
          2.68618  4.42225  2.77519  2.73123  3.46354  2.40513  3.72494
3.29354  2.67741  2.69355  4.24690  2.90347  2.73739  3.18146  2.89801
2.37887  2.77519  2.98518  4.58477  3.61503
          0.02625  4.04896  4.77131  0.61958  0.77255  0.48576  0.95510
    290   1.63357  4.52725  2.92527  2.68961  4.28208  3.14710  3.96936
3.67617  2.85768  3.37128  4.22876  1.73524  3.80022  3.18412  3.24991
2.59908  2.89266  3.24832  5.59301  4.28051    370 a - - -
```

```
          2.68618  4.42225  2.77519  2.73123  3.46354  2.40513  3.72494
3.29354  2.67741  2.69355  4.24690  2.90347  2.73739  3.18146  2.89801
2.37887  2.77519  2.98518  4.58477  3.61503
          0.02625  4.04896  4.77131  0.61958  0.77255  0.48576  0.95510
    291   2.53269  3.54512  3.89745  3.37728  3.47568  3.49474  4.12965
1.70132  3.27137  2.46698  3.45036  3.60011  4.01139  3.55831  3.52781
2.04366  2.85338  2.27106  5.01038  3.79245    371 i - - -
          2.68618  4.42225  2.77519  2.73123  3.46354  2.40513  3.72494
3.29354  2.67741  2.69355  4.24690  2.90347  2.73739  3.18146  2.89801
2.37887  2.77519  2.98518  4.58477  3.61503
          0.02625  4.04896  4.77131  0.61958  0.77255  0.48576  0.95510
    292   2.89902  4.65351  3.60866  3.53220  4.66473  0.50220  4.63853
4.31037  3.73728  3.94135  4.92090  3.77344  4.05011  4.04773  3.95090
3.07653  3.39400  3.80462  5.71003  4.77372    372 G - - -
          2.68618  4.42225  2.77519  2.73123  3.46354  2.40513  3.72494
3.29354  2.67741  2.69355  4.24690  2.90347  2.73739  3.18146  2.89801
2.37887  2.77519  2.98518  4.58477  3.61503
          0.02625  4.04896  4.77131  0.61958  0.77255  0.48576  0.95510
    293   2.77934  5.18383  1.70447  2.22090  4.59522  3.24835  3.75370
4.09382  2.68563  3.62366  4.44042  2.32138  3.82704  2.90990  3.22581
1.93534  3.07507  3.66571  5.79236  4.34897    373 d - - -
          2.68618  4.42225  2.77519  2.73123  3.46354  2.40513  3.72494
3.29354  2.67741  2.69355  4.24690  2.90347  2.73739  3.18146  2.89801
2.37887  2.77519  2.98518  4.58477  3.61503
          0.02625  4.04896  4.77131  0.61958  0.77255  0.48576  0.95510
    294   2.53370  4.65054  2.71981  2.53534  4.44661  1.99177  3.92158
3.93078  2.82940  3.52846  4.35197  1.72004  3.78813  3.11460  3.26685
2.39305  2.92730  3.44532  5.70604  4.36347    374 n - - -
          2.68618  4.42225  2.77519  2.73123  3.46354  2.40513  3.72494
3.29354  2.67741  2.69355  4.24690  2.90347  2.73739  3.18146  2.89801
2.37887  2.77519  2.98518  4.58477  3.61503
          0.02625  4.04896  4.77131  0.61958  0.77255  0.48576  0.95510
    295   2.61996  4.79613  2.96507  2.50309  4.23132  3.35471  3.68885
3.64095  1.82819  3.22489  4.04339  2.98890  2.51489  2.84104  2.77569
2.47144  2.63013  3.27688  5.42885  4.11604    375 k - - -
          2.68618  4.42225  2.77519  2.73123  3.46354  2.40513  3.72494
3.29354  2.67741  2.69355  4.24690  2.90347  2.73739  3.18146  2.89801
2.37887  2.77519  2.98518  4.58477  3.61503
          0.11326  4.04896  2.41196  0.61958  0.77255  0.48576  0.95510
    296   2.59887  4.39534  3.32763  2.80111  3.55899  3.51421  3.79380
2.56389  2.69615  2.57908  3.51084  2.99118  3.92171  2.55236  3.04697
2.78719  2.84333  1.96611  4.99919  3.74024    376 v - - -
          2.68618  4.42225  2.77519  2.73123  3.46354  2.40513  3.72494
3.29354  2.67741  2.69355  4.24690  2.90347  2.73739  3.18146  2.89801
2.37887  2.77519  2.98518  4.58477  3.61503
          0.06827  3.96430  3.05729  0.61958  0.77255  0.57050  0.83297
    297   2.77261  5.01639  2.54750  2.33984  4.30377  3.29060  3.69873
3.86093  2.44852  3.40724  4.26978  1.72031  3.83717  2.24577  2.83436
2.75913  3.05282  3.49409  5.51835  4.13308    377 n - - -
          2.68618  4.42225  2.77519  2.73123  3.46354  2.40513  3.72494
3.29354  2.67741  2.69355  4.24690  2.90347  2.73739  3.18146  2.89801
2.37887  2.77519  2.98518  4.58477  3.61503
          0.02974  3.92577  4.64812  0.61958  0.77255  0.41800  1.07400
```

```
    298   2.72213  5.00514  2.58785  1.64127  4.37006  3.31520  3.71614
3.79069  2.54194  3.37813  4.20493  2.61453  3.83521  2.87267  3.00258
2.71360  2.15013  3.42233  5.58409  4.20975    378 e - - -
          2.68618  4.42225  2.77519  2.73123  3.46354  2.40513  3.72494
3.29354  2.67741  2.69355  4.24690  2.90347  2.73739  3.18146  2.89801
2.37887  2.77519  2.98518  4.58477  3.61503
          0.02625  4.04896  4.77131  0.61958  0.77255  0.48576  0.95510
    299   2.70982  4.83355  2.78655  2.54123  4.35913  1.78440  3.84186
3.85223  2.61007  3.42580  4.28462  3.02071  3.87070  1.82895  2.98533
2.75332  3.03718  3.45810  5.57203  4.25518    379 g - - -
          2.68618  4.42225  2.77519  2.73123  3.46354  2.40513  3.72494
3.29354  2.67741  2.69355  4.24690  2.90347  2.73739  3.18146  2.89801
2.37887  2.77519  2.98518  4.58477  3.61503
          0.02625  4.04896  4.77131  0.61958  0.77255  0.48576  0.95510
    300   2.40374  4.83683  2.72774  1.91495  4.29067  3.28382  3.72285
3.72136  2.55580  3.30802  4.12108  2.90957  3.27776  2.87900  3.02052
1.88398  2.90484  3.33578  5.52308  4.16818    380 s - - -
          2.68618  4.42225  2.77519  2.73123  3.46354  2.40513  3.72494
3.29354  2.67741  2.69355  4.24690  2.90347  2.73739  3.18146  2.89801
2.37887  2.77519  2.98518  4.58477  3.61503
          0.02625  4.04896  4.77131  0.61958  0.77255  0.48576  0.95510
    301   3.68026  4.90609  4.66170  4.35677  1.25234  4.42094  3.48542
3.36150  4.20093  2.68562  3.95609  4.09735  4.72794  4.15858  4.22669
3.79199  3.90154  3.28817  3.62154  1.14849    381 y - - -
          2.68618  4.42225  2.77519  2.73123  3.46354  2.40513  3.72494
3.29354  2.67741  2.69355  4.24690  2.90347  2.73739  3.18146  2.89801
2.37887  2.77519  2.98518  4.58477  3.61503
          0.02625  4.04896  4.77131  0.61958  0.77255  0.48576  0.95510
    302   2.33508  4.31027  3.30347  2.91338  4.17431  3.08349  4.02478
3.57049  2.93055  3.24426  4.07712  3.21224  2.63717  3.25500  3.29786
1.82623  1.76379  3.12045  5.50458  4.25376    382 t - - -
          2.68618  4.42225  2.77519  2.73123  3.46354  2.40513  3.72494
3.29354  2.67741  2.69355  4.24690  2.90347  2.73739  3.18146  2.89801
2.37887  2.77519  2.98518  4.58477  3.61503
          0.02625  4.04896  4.77131  0.61958  0.77255  0.48576  0.95510
    303   1.96831  4.22194  3.74227  3.27040  3.59134  3.45287  4.12847
1.70110  3.19713  2.55573  3.54242  3.53213  3.99237  3.50411  3.48283
2.42811  2.86611  2.31548  5.11614  3.89484    383 i - - -
          2.68618  4.42225  2.77519  2.73123  3.46354  2.40513  3.72494
3.29354  2.67741  2.69355  4.24690  2.90347  2.73739  3.18146  2.89801
2.37887  2.77519  2.98518  4.58477  3.61503
          0.02625  4.04896  4.77131  0.61958  0.77255  0.48576  0.95510
    304   2.64064  4.77087  2.85428  2.53981  4.25250  3.29045  3.78669
3.74003  2.53265  3.32356  4.17326  3.00985  3.84378  1.92647  2.90661
1.72900  2.95919  3.35573  5.49589  4.15897    384 s - - -
          2.68618  4.42225  2.77519  2.73123  3.46354  2.40513  3.72494
3.29354  2.67741  2.69355  4.24690  2.90347  2.73739  3.18146  2.89801
2.37887  2.77519  2.98518  4.58477  3.61503
          0.02625  4.04896  4.77131  0.61958  0.77255  0.48576  0.95510
    305   2.75653  4.96271  2.07200  2.36930  3.80566  3.37872  3.68547
3.63981  2.62573  3.22410  4.08492  2.51427  3.87112  2.91137  3.10506
2.75717  3.01090  3.31864  5.20534  2.13752    385 d - - -
          2.68618  4.42225  2.77519  2.73123  3.46354  2.40513  3.72494
3.29354  2.67741  2.69355  4.24690  2.90347  2.73739  3.18146  2.89801
2.37887  2.77519  2.98518  4.58477  3.61503
```

```
         0.02625  4.04896  4.77131  0.61958  0.77255  0.48576  0.95510
    306   3.02207  4.62906  3.86725  3.52154  3.13778  2.69063  4.05230
3.43358  3.32900  3.01928  4.05376  3.76209  4.18287  3.73161  3.53767
3.22605  3.35191  3.20172  0.97091  3.15857    386 w - - -
         2.68618  4.42225  2.77519  2.73123  3.46354  2.40513  3.72494
3.29354  2.67741  2.69355  4.24690  2.90347  2.73739  3.18146  2.89801
2.37887  2.77519  2.98518  4.58477  3.61503
         0.02625  4.04896  4.77131  0.61958  0.77255  0.48576  0.95510
    307   3.00224  4.72657  3.62906  3.49603  4.47053  3.40426  4.55621
4.06484  3.56912  3.67110  4.73068  3.78339  0.55633  3.95247  3.79183
3.17599  3.45834  3.68948  5.60532  4.59466    387 P - - -
         2.68618  4.42225  2.77519  2.73123  3.46354  2.40513  3.72494
3.29354  2.67741  2.69355  4.24690  2.90347  2.73739  3.18146  2.89801
2.37887  2.77519  2.98518  4.58477  3.61503
         0.02625  4.04896  4.77131  0.61958  0.77255  0.48576  0.95510
    308   3.49106  4.84211  4.48107  4.23211  0.76177  4.13322  3.96743
2.99649  4.13553  2.32725  3.70244  4.22219  4.59014  4.25557  4.21149
3.74980  3.79345  2.99963  4.16985  2.51707    388 f - - -
         2.68618  4.42225  2.77519  2.73123  3.46354  2.40513  3.72494
3.29354  2.67741  2.69355  4.24690  2.90347  2.73739  3.18146  2.89801
2.37887  2.77519  2.98518  4.58477  3.61503
         0.02625  4.04896  4.77131  0.61958  0.77255  0.48576  0.95510
    309   3.33804  4.67950  4.72597  4.24316  1.60475  4.42503  4.29901
2.40819  4.09423  1.17563  3.04918  4.36724  4.67657  4.18974  4.19855
3.79259  3.56831  2.51281  4.53431  3.03358    389 l - - -
         2.68618  4.42225  2.77519  2.73123  3.46354  2.40513  3.72494
3.29354  2.67741  2.69355  4.24690  2.90347  2.73739  3.18146  2.89801
2.37887  2.77519  2.98518  4.58477  3.61503
         0.02625  4.04896  4.77131  0.61958  0.77255  0.48576  0.95510
    310   2.86290  4.78063  3.08787  2.76727  3.00254  3.55297  3.68135
3.44288  2.80072  3.01849  3.98441  1.84368  4.03170  3.16054  3.16234
2.93263  3.13079  3.18340  4.56248  1.87836    390 n - - -
         2.68618  4.42225  2.77519  2.73123  3.46354  2.40513  3.72494
3.29354  2.67741  2.69355  4.24690  2.90347  2.73739  3.18146  2.89801
2.37887  2.77519  2.98518  4.58477  3.61503
         0.02625  4.04896  4.77131  0.61958  0.77255  0.48576  0.95510
    311   2.28706  4.23253  3.39998  3.12472  4.28772  3.00148  4.21230
3.65538  3.15511  3.38953  4.24704  3.31243  3.74008  3.48091  3.46337
1.12790  2.23117  3.15434  5.64304  4.40534    391 s - - -
         2.68618  4.42225  2.77519  2.73123  3.46354  2.40513  3.72494
3.29354  2.67741  2.69355  4.24690  2.90347  2.73739  3.18146  2.89801
2.37887  2.77519  2.98518  4.58477  3.61503
         0.02625  4.04896  4.77131  0.61958  0.77255  0.48576  0.95510
    312   3.13257  4.59313  4.18590  3.72122  2.49809  4.06958  3.83173
2.75066  3.52518  1.49495  3.42775  3.88463  4.41773  3.78432  3.71319
3.41346  3.36869  2.69702  4.18641  1.57940    392 l - - -
         2.68618  4.42225  2.77519  2.73123  3.46354  2.40513  3.72494
3.29354  2.67741  2.69355  4.24690  2.90347  2.73739  3.18146  2.89801
2.37887  2.77519  2.98518  4.58477  3.61503
         0.02625  4.04896  4.77131  0.61958  0.77255  0.48576  0.95510
    313   3.15715  4.50391  4.70290  4.19290  1.85784  4.33672  4.46082
1.44497  4.06041  1.76177  3.11323  4.34441  4.61944  4.19995  4.18438
3.69319  3.39759  2.13190  4.78668  3.38088    393 i - - -
```

```
          2.68618  4.42225  2.77519  2.73123  3.46354  2.40513  3.72494
3.29354  2.67741  2.69355  4.24690  2.90347  2.73739  3.18146  2.89801
2.37887  2.77519  2.98518  4.58477  3.61503
          0.02625  4.04896  4.77131  0.61958  0.77255  0.48576  0.95510
    314   3.03267  5.01648  3.03125  2.79233  4.20781  3.49003  3.95095
3.86917  2.60165  3.35356  4.37991  3.25232  4.04822  0.94997  2.88086
3.07456  3.33113  3.58887  5.44998  4.15689    394 q - - -
          2.68618  4.42225  2.77519  2.73123  3.46354  2.40513  3.72494
3.29354  2.67741  2.69355  4.24690  2.90347  2.73739  3.18146  2.89801
2.37887  2.77519  2.98518  4.58477  3.61503
          0.02625  4.04896  4.77131  0.61958  0.77255  0.48576  0.95510
    315   2.26837  4.22682  3.85418  3.39755  3.74409  3.41266  4.27428
2.45987  3.31945  2.62823  3.65372  3.60693  4.00795  3.62761  3.60194
2.80417  1.90256  1.57801  5.30153  4.08919    395 v - - -
          2.68618  4.42225  2.77519  2.73123  3.46354  2.40513  3.72494
3.29354  2.67741  2.69355  4.24690  2.90347  2.73739  3.18146  2.89801
2.37887  2.77519  2.98518  4.58477  3.61503
          0.02625  4.04896  4.77131  0.61958  0.77255  0.48576  0.95510
    316   2.83995  5.11163  2.93422  2.49642  4.48260  3.47816  3.63860
3.90388  1.73370  3.40139  4.22673  1.89147  3.91120  2.77378  2.30743
2.82243  3.05989  3.54370  5.51311  4.21902    396 k - - -
          2.68618  4.42225  2.77519  2.73123  3.46354  2.40513  3.72494
3.29354  2.67741  2.69355  4.24690  2.90347  2.73739  3.18146  2.89801
2.37887  2.77519  2.98518  4.58477  3.61503
          0.02625  4.04896  4.77131  0.61958  0.77255  0.48576  0.95510
    317   2.05417  4.22230  3.38679  3.16404  4.33338  2.97760  4.26854
3.71840  3.24025  3.45848  4.32130  3.32807  3.73695  3.54967  3.53741
1.02496  2.77423  3.19117  5.69507  4.45406    397 s - - -
          2.68618  4.42225  2.77519  2.73123  3.46354  2.40513  3.72494
3.29354  2.67741  2.69355  4.24690  2.90347  2.73739  3.18146  2.89801
2.37887  2.77519  2.98518  4.58477  3.61503
          0.02625  4.04896  4.77131  0.61958  0.77255  0.48576  0.95510
    318   2.87931  4.87574  2.82111  2.72621  4.26801  3.31004  4.07758
4.02015  2.99819  3.64783  4.60465  0.87973  3.97208  3.35350  3.35640
2.94155  3.26875  3.62429  5.55383  4.20588    398 n - - -
          2.68618  4.42225  2.77519  2.73123  3.46354  2.40513  3.72494
3.29354  2.67741  2.69355  4.24690  2.90347  2.73739  3.18146  2.89801
2.37887  2.77519  2.98518  4.58477  3.61503
          0.02625  4.04896  4.77131  0.61958  0.77255  0.48576  0.95510
    319   3.51130  4.92842  4.10319  3.86868  2.32121  4.03918  3.71497
3.49696  3.72133  2.90449  4.15421  3.94722  4.51032  3.98439  3.86748
3.62915  3.79964  3.37626  3.97068  0.71613    399 y - - -
          2.68618  4.42225  2.77519  2.73123  3.46354  2.40513  3.72494
3.29354  2.67741  2.69355  4.24690  2.90347  2.73739  3.18146  2.89801
2.37887  2.77519  2.98518  4.58477  3.61503
          0.02625  4.04896  4.77131  0.61958  0.77255  0.48576  0.95510
    320   3.10002  4.42719  4.78860  4.30088  3.60237  4.42502  5.02678
1.49267  4.18454  2.11661  3.41473  4.51731  4.75724  4.46109  4.38826
3.81845  3.37261  1.00661  5.56150  4.34549    400 v - - -
          2.68618  4.42225  2.77519  2.73123  3.46354  2.40513  3.72494
3.29354  2.67741  2.69355  4.24690  2.90347  2.73739  3.18146  2.89801
2.37887  2.77519  2.98518  4.58477  3.61503
          0.02625  4.04896  4.77131  0.61958  0.77255  0.48576  0.95510
```

```
    321   3.33869  4.71410  4.72862  4.20009  3.07995  4.49754  4.79269
2.22634  3.97396  0.80941  2.58151  4.48491  4.72271  4.19277  4.12569
3.87294  3.57315  2.35470  5.15854  4.02507    401 l - - -
          2.68618  4.42225  2.77519  2.73123  3.46354  2.40513  3.72494
3.29354  2.67741  2.69355  4.24690  2.90347  2.73739  3.18146  2.89801
2.37887  2.77519  2.98518  4.58477  3.61503
          0.02625  4.04896  4.77131  0.61958  0.77255  0.48576  0.95510
    322   2.47936  4.53044  2.89181  2.67995  4.31735  3.11784  3.97595
3.83849  2.86934  3.46907  4.30707  2.27196  3.78755  3.19336  3.26032
1.31144  2.89365  3.35842  5.61949  4.28590    402 s - - -
          2.68618  4.42225  2.77519  2.73123  3.46354  2.40513  3.72494
3.29354  2.67741  2.69355  4.24690  2.90347  2.73739  3.18146  2.89801
2.37887  2.77519  2.98518  4.58477  3.61503
          0.02625  4.04896  4.77131  0.61958  0.77255  0.48576  0.95510
    323   2.52399  4.51418  2.93622  2.82189  4.44035  1.07077  4.14785
3.99260  3.10773  3.63580  4.49697  2.64198  3.82449  3.40573  3.46521
2.64647  2.98060  3.46953  5.71613  4.44239    403 g - - -
          2.68618  4.42225  2.77519  2.73123  3.46354  2.40513  3.72494
3.29354  2.67741  2.69355  4.24690  2.90347  2.73739  3.18146  2.89801
2.37887  2.77519  2.98518  4.58477  3.61503
          0.02625  4.04896  4.77131  0.61958  0.77255  0.48576  0.95510
    324   3.04089  4.39184  4.66627  4.10829  2.42042  4.28889  4.60008
1.92430  3.99159  1.63168  3.10424  4.30880  4.56607  4.15704  4.13803
3.61942  3.27587  1.39298  5.04895  3.83340    404 v - - -
          2.68618  4.42225  2.77519  2.73123  3.46354  2.40513  3.72494
3.29354  2.67741  2.69355  4.24690  2.90347  2.73739  3.18146  2.89801
2.37887  2.77519  2.98518  4.58477  3.61503
          0.02625  4.04896  4.77131  0.61958  0.77255  0.48576  0.95510
    325   2.30916  4.26714  3.28448  3.08832  4.40094  2.54094  4.24947
3.88595  3.22726  3.56199  4.39553  3.29447  3.74136  3.52259  3.54212
1.00417  2.80272  3.30984  5.72173  4.48440    405 s - - -
          2.68618  4.42225  2.77519  2.73123  3.46354  2.40513  3.72494
3.29354  2.67741  2.69355  4.24690  2.90347  2.73739  3.18146  2.89801
2.37887  2.77519  2.98518  4.58477  3.61503
          0.02625  4.04896  4.77131  0.61958  0.77255  0.48576  0.95510
    326   2.38566  4.28126  3.46872  3.22332  4.16073  1.11739  4.27705
3.44617  3.26104  3.25694  4.19833  3.40858  3.81652  3.58696  3.54668
2.57434  2.85881  2.51849  5.56240  4.32335    406 g - - -
          2.68618  4.42225  2.77519  2.73123  3.46354  2.40513  3.72494
3.29354  2.67741  2.69355  4.24690  2.90347  2.73739  3.18146  2.89801
2.37887  2.77519  2.98518  4.58477  3.61503
          0.02625  4.04896  4.77131  0.61958  0.77255  0.48576  0.95510
    327   2.26340  4.43520  3.25330  2.69802  3.60576  3.48408  3.73170
2.76706  2.64752  2.67097  3.54433  2.92139  3.87712  2.98842  2.80433
2.59739  2.67494  2.73237  5.00310  2.74926    407 a - - -
          2.68618  4.42225  2.77519  2.73123  3.46354  2.40513  3.72494
3.29354  2.67741  2.69355  4.24690  2.90347  2.73739  3.18146  2.89801
2.37887  2.77519  2.98518  4.58477  3.61503
          0.02625  4.04896  4.77131  0.61958  0.77255  0.48576  0.95510
    328   2.00823  4.53923  3.21374  2.68898  3.98148  2.95461  3.77144
3.34091  2.53445  3.00716  3.85833  3.11623  3.83130  2.96766  2.03244
2.64167  2.83247  2.71523  5.28411  4.01187    408 a - - -
          2.68618  4.42225  2.77519  2.73123  3.46354  2.40513  3.72494
3.29354  2.67741  2.69355  4.24690  2.90347  2.73739  3.18146  2.89801
2.37887  2.77519  2.98518  4.58477  3.61503
```

```
          0.02625  4.04896  4.77131  0.61958  0.77255  0.48576  0.95510
    329   2.40834  4.13046  3.73551  2.92137  3.24371  3.60873  3.88914
2.32616  3.07403  2.07457  2.82998  3.47274  3.98245  3.34195  3.34327
2.72622  2.64090  2.37888  3.82087  3.52487    409 l - - -
          2.68618  4.42225  2.77519  2.73123  3.46354  2.40513  3.72494
3.29354  2.67741  2.69355  4.24690  2.90347  2.73739  3.18146  2.89801
2.37887  2.77519  2.98518  4.58477  3.61503
          0.02625  4.04896  4.77131  0.61958  0.77255  0.48576  0.95510
    330   2.58937  4.65313  3.18731  2.67466  4.16725  3.34514  3.75015
3.55754  2.39827  3.16931  4.00870  3.10242  3.84616  2.92296  1.82000
2.22324  2.30975  3.19980  5.38476  4.11368    410 r - - -
          2.68618  4.42225  2.77519  2.73123  3.46354  2.40513  3.72494
3.29354  2.67741  2.69355  4.24690  2.90347  2.73739  3.18146  2.89801
2.37887  2.77519  2.98518  4.58477  3.61503
          0.02625  4.04896  4.77131  0.61958  0.77255  0.48576  0.95510
    331   2.44636  4.16297  3.79007  3.22311  2.83506  3.64526  3.87666
2.25128  3.12679  2.32611  3.26569  2.99045  4.02124  3.39244  3.38440
2.92106  2.83840  2.40374  2.65255  3.32645    411 i - - -
          2.68618  4.42225  2.77519  2.73123  3.46354  2.40513  3.72494
3.29354  2.67741  2.69355  4.24690  2.90347  2.73739  3.18146  2.89801
2.37887  2.77519  2.98518  4.58477  3.61503
          0.02625  4.04896  4.77131  0.61958  0.77255  0.48576  0.95510
    332   2.52157  4.39332  3.28640  2.81253  3.63869  3.36272  3.83338
3.09520  2.77468  2.79669  3.68339  3.20707  3.86487  3.11894  3.14008
2.20873  1.87972  2.81457  5.06233  2.88491    412 t - - -
          2.68618  4.42225  2.77519  2.73123  3.46354  2.40513  3.72494
3.29354  2.67741  2.69355  4.24690  2.90347  2.73739  3.18146  2.89801
2.37887  2.77519  2.98518  4.58477  3.61503
          0.02625  4.04896  4.77131  0.61958  0.77255  0.48576  0.95510
    333   3.51557  4.79402  4.60460  4.23106  1.40972  4.34974  3.54089
3.17457  4.07556  2.30220  3.78409  4.06225  4.65327  4.08359  4.13597
3.69521  3.73518  3.10302  3.70207  1.23133    413 y - - -
          2.68618  4.42225  2.77519  2.73123  3.46354  2.40513  3.72494
3.29354  2.67741  2.69355  4.24690  2.90347  2.73739  3.18146  2.89801
2.37887  2.77519  2.98518  4.58477  3.61503
          0.22621  4.04896  1.68735  0.61958  0.77255  0.48576  0.95510
    334   2.52908  4.57607  2.96952  2.49306  3.85714  3.01408  3.18511
3.26567  2.47931  2.69080  3.75176  2.75241  3.78005  2.84561  2.89495
2.42708  2.46854  2.96195  5.17683  3.87494    414 s - - -
          2.68618  4.42225  2.77519  2.73123  3.46354  2.40513  3.72494
3.29354  2.67741  2.69355  4.24690  2.90347  2.73739  3.18146  2.89801
2.37887  2.77519  2.98518  4.58477  3.61503
          0.09394  3.85472  2.68112  0.61958  0.77255  0.66167  0.72565
    335   2.49725  4.47578  2.90207  2.62641  3.83556  3.17785  3.81262
3.21514  2.66952  2.99802  3.94589  1.82241  3.77514  3.06145  3.02625
2.61363  2.85475  2.54750  5.24400  3.87429    415 n - - -
          2.68618  4.42225  2.77519  2.73123  3.46354  2.40513  3.72494
3.29354  2.67741  2.69355  4.24690  2.90347  2.73739  3.18146  2.89801
2.37887  2.77519  2.98518  4.58477  3.61503
          0.03398  3.79475  4.51710  0.61958  0.77255  0.36986  1.17386
    336   2.41023  4.34448  3.31328  3.03611  4.21292  3.10878  4.15063
3.52954  3.04947  3.25994  4.17359  3.30351  1.37170  3.41205  3.36448
2.57533  2.19127  3.11677  5.56924  4.33463    416 p - - -
```

2.68618 4.42225 2.77519 2.73123 3.46354 2.40513 3.72494
3.29354 2.67741 2.69355 4.24690 2.90347 2.73739 3.18146 2.89801
2.37887 2.77519 2.98518 4.58477 3.61503
0.02625 4.04896 4.77131 0.61958 0.77255 0.48576 0.95510
337 2.65444 4.22346 3.79700 3.23997 2.58819 3.70281 3.98278
2.05335 3.16125 2.28936 3.29425 2.62745 4.07966 3.43593 3.44130
2.98250 2.89388 2.04695 4.80271 3.53681 417 v - - -
2.68618 4.42225 2.77519 2.73123 3.46354 2.40513 3.72494
3.29354 2.67741 2.69355 4.24690 2.90347 2.73739 3.18146 2.89801
2.37887 2.77519 2.98518 4.58477 3.61503
0.02625 4.04896 4.77131 0.61958 0.77255 0.48576 0.95510
338 3.18083 4.51241 4.79242 4.24186 1.93061 4.40481 4.56980
1.63481 4.10934 1.42762 2.99128 4.41596 4.64040 4.21533 4.21568
3.74290 3.40707 2.18475 4.88913 3.59236 418 l - - -
2.68618 4.42225 2.77519 2.73123 3.46354 2.40513 3.72494
3.29354 2.67741 2.69355 4.24690 2.90347 2.73739 3.18146 2.89801
2.37887 2.77519 2.98518 4.58477 3.61503
0.02625 4.04896 4.77131 0.61958 0.77255 0.48576 0.95510
339 2.38799 4.27400 3.49159 3.22074 4.10879 1.19786 4.25851
3.34444 3.24349 3.18217 4.12931 3.41145 3.82337 3.56851 3.53287
2.58124 2.85131 2.34217 5.52537 4.28636 419 g - - -
2.68618 4.42225 2.77519 2.73123 3.46354 2.40513 3.72494
3.29354 2.67741 2.69355 4.24690 2.90347 2.73739 3.18146 2.89801
2.37887 2.77519 2.98518 4.58477 3.61503
0.02625 4.04896 4.77131 0.61958 0.77255 0.48576 0.95510
340 2.67706 5.02741 2.77872 2.03989 4.34315 2.43014 3.61605
3.78566 2.33952 3.31861 4.11160 2.88001 3.43631 2.54092 2.42672
2.66011 2.91293 3.40999 5.49161 4.12895 420 e - - -
2.68618 4.42225 2.77519 2.73123 3.46354 2.40513 3.72494
3.29354 2.67741 2.69355 4.24690 2.90347 2.73739 3.18146 2.89801
2.37887 2.77519 2.98518 4.58477 3.61503
0.02625 4.04896 4.77131 0.61958 0.77255 0.48576 0.95510
341 3.25251 4.61639 4.45083 3.99244 1.91279 4.20238 3.69334
2.87529 3.85064 1.72287 3.50174 3.99460 4.51441 3.94858 3.97145
3.52661 3.47598 2.81705 3.92507 1.42679 421 y - - -
2.68628 4.42236 2.77530 2.73134 3.46364 2.40499 3.72505
3.29364 2.67751 2.69365 4.24700 2.90357 2.73707 3.18157 2.89811
2.37879 2.77486 2.98517 4.58487 3.61491
0.22621 1.63999 4.77131 1.13162 0.38936 0.48576 0.95510
342 2.57677 4.25391 3.53793 2.98271 3.39193 3.55924 3.44709
2.09015 2.89181 2.31268 3.37530 3.35556 3.95525 3.21400 3.21054
2.61744 2.31825 2.48452 4.85929 3.62599 426 i - - -
2.68618 4.42225 2.77519 2.73123 3.46354 2.40513 3.72494
3.29354 2.67741 2.69355 4.24690 2.90347 2.73739 3.18146 2.89801
2.37887 2.77519 2.98518 4.58477 3.61503
0.02625 4.04896 4.77131 0.61958 0.77255 0.48576 0.95510
343 2.62188 4.77684 2.98946 2.46667 4.01415 3.42552 3.24557
3.13077 2.39502 3.04013 3.86773 2.79602 3.82923 2.15699 2.81298
2.66394 2.36438 3.11909 5.27736 3.95582 427 q - - -
2.68618 4.42225 2.77519 2.73123 3.46354 2.40513 3.72494
3.29354 2.67741 2.69355 4.24690 2.90347 2.73739 3.18146 2.89801
2.37887 2.77519 2.98518 4.58477 3.61503
0.02625 4.04896 4.77131 0.61958 0.77255 0.48576 0.95510

```
    344   2.76960  5.24118  1.72080  2.21138  4.52493  3.30728  2.76710
4.02100  2.53012  3.53138  4.32871  2.55392  3.81960  2.62124  3.04845
2.52254  3.02373  3.61524  5.68981  4.25486    428 d - - -
          2.68618  4.42225  2.77519  2.73123  3.46354  2.40513  3.72494
3.29354  2.67741  2.69355  4.24690  2.90347  2.73739  3.18146  2.89801
2.37887  2.77519  2.98518  4.58477  3.61503
          0.02625  4.04896  4.77131  0.61958  0.77255  0.48576  0.95510
    345   2.38113  4.69049  2.81920  2.47154  3.50250  3.42346  3.13038
3.03689  2.46134  2.94043  3.77036  2.80225  3.81411  2.80756  2.75117
2.53209  2.56164  3.01105  5.21071  3.89497    429 a - - -
          2.68618  4.42225  2.77519  2.73123  3.46354  2.40513  3.72494
3.29354  2.67741  2.69355  4.24690  2.90347  2.73739  3.18146  2.89801
2.37887  2.77519  2.98518  4.58477  3.61503
          0.02625  4.04896  4.77131  0.61958  0.77255  0.48576  0.95510
    346   3.35841  4.77029  4.47216  4.11619  3.21164  4.20451  4.71683
2.42879  3.88223  0.68484  3.18550  4.37450  4.61168  4.20806  4.02805
3.80108  3.64584  2.51132  5.15144  3.91438    430 L - - -
          2.68618  4.42225  2.77519  2.73123  3.46354  2.40513  3.72494
3.29354  2.67741  2.69355  4.24690  2.90347  2.73739  3.18146  2.89801
2.37887  2.77519  2.98518  4.58477  3.61503
          0.02625  4.04896  4.77131  0.61958  0.77255  0.48576  0.95510
    347   2.63948  4.57584  3.19220  2.65045  3.54706  3.50752  3.09320
3.14363  2.38882  2.37969  3.66914  2.73335  3.89554  2.93812  2.92549
2.75054  2.86769  2.88384  4.95702  2.60491    431 l - - -
          2.68618  4.42225  2.77519  2.73123  3.46354  2.40513  3.72494
3.29354  2.67741  2.69355  4.24690  2.90347  2.73739  3.18146  2.89801
2.37887  2.77519  2.98518  4.58477  3.61503
          0.02625  4.04896  4.77131  0.61958  0.77255  0.48576  0.95510
    348   2.25672  4.56431  3.02666  2.59919  3.42831  3.29707  3.77789
3.38631  2.64992  3.04623  3.89309  1.90573  3.81234  2.98228  3.07467
2.30680  2.83260  3.05075  5.29155  3.96988    432 n - - -
          2.68618  4.42225  2.77519  2.73123  3.46354  2.40513  3.72494
3.29354  2.67741  2.69355  4.24690  2.90347  2.73739  3.18146  2.89801
2.37887  2.77519  2.98518  4.58477  3.61503
          0.02625  4.04896  4.77131  0.61958  0.77255  0.48576  0.95510
    349   2.05038  4.28602  4.17186  3.63902  2.96164  3.90528  4.33271
1.52956  3.54538  2.13375  3.29201  3.89744  4.29720  3.80189  3.78167
3.23212  3.06251  1.99476  5.04338  3.81509    433 i - - -
          2.68618  4.42225  2.77519  2.73123  3.46354  2.40513  3.72494
3.29354  2.67741  2.69355  4.24690  2.90347  2.73739  3.18146  2.89801
2.37887  2.77519  2.98518  4.58477  3.61503
          0.02625  4.04896  4.77131  0.61958  0.77255  0.48576  0.95510
    350   2.63357  4.58972  3.22720  2.66793  3.82450  3.48394  3.71093
2.52165  2.45465  2.84662  3.71942  2.83738  3.88985  2.91953  2.07875
2.74500  2.53442  2.89133  5.15178  3.88441    434 r - - -
          2.68618  4.42225  2.77519  2.73123  3.46354  2.40513  3.72494
3.29354  2.67741  2.69355  4.24690  2.90347  2.73739  3.18146  2.89801
2.37887  2.77519  2.98518  4.58477  3.61503
          0.02625  4.04896  4.77131  0.61958  0.77255  0.48576  0.95510
    351   2.04942  4.24631  3.93074  3.39670  3.54068  3.68766  4.21808
2.00492  3.31998  2.41997  3.45499  3.68420  4.14334  3.60949  3.60799
3.01487  2.42313  1.68830  5.12450  3.91025    435 v - - -
          2.68618  4.42225  2.77519  2.73123  3.46354  2.40513  3.72494
3.29354  2.67741  2.69355  4.24690  2.90347  2.73739  3.18146  2.89801
2.37887  2.77519  2.98518  4.58477  3.61503
```

```
         0.02625  4.04896  4.77131  0.61958  0.77255  0.48576  0.95510
    352  2.47940  4.56392  2.90747  2.61460  4.31176  3.15616  3.89154
3.75118  2.75251  3.37571  4.19807  1.90746  3.77792  3.08096  3.16788
1.76065  2.53521  3.30231  5.58355  4.26769    436 s - - -
         2.68618  4.42225  2.77519  2.73123  3.46354  2.40513  3.72494
3.29354  2.67741  2.69355  4.24690  2.90347  2.73739  3.18146  2.89801
2.37887  2.77519  2.98518  4.58477  3.61503
         0.02625  4.04896  4.77131  0.61958  0.77255  0.48576  0.95510
    353  3.51130  4.92842  4.10319  3.86868  2.32121  4.03918  3.71497
3.49696  3.72133  2.90449  4.15421  3.94722  4.51032  3.98439  3.86748
3.62915  3.79964  3.37626  3.97068  0.71613    437 y - - -
         2.68618  4.42225  2.77519  2.73123  3.46354  2.40513  3.72494
3.29354  2.67741  2.69355  4.24690  2.90347  2.73739  3.18146  2.89801
2.37887  2.77519  2.98518  4.58477  3.61503
         0.02625  4.04896  4.77131  0.61958  0.77255  0.48576  0.95510
    354  2.56888  4.29703  3.48655  2.92703  3.48758  3.52768  3.84706
2.44135  2.83561  2.53090  3.44266  3.31567  3.93828  3.17116  2.77091
2.40041  2.63122  2.02304  4.93853  3.70567    438 v - - -
         2.68618  4.42225  2.77519  2.73123  3.46354  2.40513  3.72494
3.29354  2.67741  2.69355  4.24690  2.90347  2.73739  3.18146  2.89801
2.37887  2.77519  2.98518  4.58477  3.61503
         0.02625  4.04896  4.77131  0.61958  0.77255  0.48576  0.95510
    355  2.89902  4.65351  3.60866  3.53220  4.66473  0.50220  4.63853
4.31037  3.73728  3.94135  4.92090  3.77344  4.05011  4.04773  3.95090
3.07653  3.39400  3.80462  5.71003  4.77372    439 G - - -
         2.68618  4.42225  2.77519  2.73123  3.46354  2.40513  3.72494
3.29354  2.67741  2.69355  4.24690  2.90347  2.73739  3.18146  2.89801
2.37887  2.77519  2.98518  4.58477  3.61503
         0.02625  4.04896  4.77131  0.61958  0.77255  0.48576  0.95510
    356  2.89902  4.65351  3.60866  3.53220  4.66473  0.50220  4.63853
4.31037  3.73728  3.94135  4.92090  3.77344  4.05011  4.04773  3.95090
3.07653  3.39400  3.80462  5.71003  4.77372    440 G - - -
         2.68618  4.42225  2.77519  2.73123  3.46354  2.40513  3.72494
3.29354  2.67741  2.69355  4.24690  2.90347  2.73739  3.18146  2.89801
2.37887  2.77519  2.98518  4.58477  3.61503
         0.02625  4.04896  4.77131  0.61958  0.77255  0.48576  0.95510
    357  2.72676  4.26111  3.93192  3.37970  3.45797  3.81299  4.16741
1.82706  2.88817  2.34473  3.38154  3.69825  4.19377  3.57356  3.52284
3.11172  2.69787  1.54300  5.04013  3.82652    441 v - - -
         2.68618  4.42225  2.77519  2.73123  3.46354  2.40513  3.72494
3.29354  2.67741  2.69355  4.24690  2.90347  2.73739  3.18146  2.89801
2.37887  2.77519  2.98518  4.58477  3.61503
         0.02625  4.04896  4.77131  0.61958  0.77255  0.48576  0.95510
    358  2.63226  4.81847  2.87663  2.44240  4.12062  3.37211  3.66935
3.56054  2.44064  2.84934  3.98017  2.41764  3.82492  2.45862  2.86510
1.98693  2.88782  3.22148  5.36998  4.03168    442 s - - -
         2.68618  4.42225  2.77519  2.73123  3.46354  2.40513  3.72494
3.29354  2.67741  2.69355  4.24690  2.90347  2.73739  3.18146  2.89801
2.37887  2.77519  2.98518  4.58477  3.61503
         0.02625  4.04896  4.77131  0.61958  0.77255  0.48576  0.95510
    359  2.29436  4.26346  3.25535  3.05221  4.43120  1.54319  4.23374
3.90702  3.21734  3.57002  4.39130  3.26968  3.72856  3.49554  3.54862
1.49058  2.78630  3.31731  5.74479  4.51006    443 s - - -
```

```
          2.68618  4.42225  2.77519  2.73123  3.46354  2.40513  3.72494
3.29354  2.67741  2.69355  4.24690  2.90347  2.73739  3.18146  2.89801
2.37887  2.77519  2.98518  4.58477  3.61503
          0.02625  4.04896  4.77131  0.61958  0.77255  0.48576  0.95510
    360   2.07043  4.30087  3.22881  2.91700  4.32417  1.92328  4.08689
3.75273  3.02118  3.40362  4.21818  3.20104  2.00453  3.31468  3.39465
2.24408  2.75875  3.23151  5.63436  4.38478    444 g - - -
          2.68618  4.42225  2.77519  2.73123  3.46354  2.40513  3.72494
3.29354  2.67741  2.69355  4.24690  2.90347  2.73739  3.18146  2.89801
2.37887  2.77519  2.98518  4.58477  3.61503
          0.02625  4.04896  4.77131  0.61958  0.77255  0.48576  0.95510
    361   2.61351  4.94656  2.61608  2.35664  4.23247  3.39085  3.58781
3.67153  2.14548  2.70350  4.00826  2.69893  3.79089  2.46343  2.64595
2.60936  2.65522  3.30595  5.41130  4.05018    445 k - - -
          2.68618  4.42225  2.77519  2.73123  3.46354  2.40513  3.72494
3.29354  2.67741  2.69355  4.24690  2.90347  2.73739  3.18146  2.89801
2.37887  2.77519  2.98518  4.58477  3.61503
          0.02625  4.04896  4.77131  0.61958  0.77255  0.48576  0.95510
    362   3.17678  4.48159  4.85971  4.34385  3.45703  4.53154  5.01750
1.08220  4.21192  1.78740  3.26275  4.57900  4.79796  4.45042  4.39406
3.90906  3.43009  1.58455  5.46739  4.28825    446 i - - -
          2.68618  4.42225  2.77519  2.73123  3.46354  2.40513  3.72494
3.29354  2.67741  2.69355  4.24690  2.90347  2.73739  3.18146  2.89801
2.37887  2.77519  2.98518  4.58477  3.61503
          0.02625  4.04896  4.77131  0.61958  0.77255  0.48576  0.95510
    363   2.89902  4.65351  3.60866  3.53220  4.66473  0.50220  4.63853
4.31037  3.73728  3.94135  4.92090  3.77344  4.05011  4.04773  3.95090
3.07653  3.39400  3.80462  5.71003  4.77372    447 G - - -
          2.68618  4.42225  2.77519  2.73123  3.46354  2.40513  3.72494
3.29354  2.67741  2.69355  4.24690  2.90347  2.73739  3.18146  2.89801
2.37887  2.77519  2.98518  4.58477  3.61503
          0.02625  4.04896  4.77131  0.61958  0.77255  0.48576  0.95510
    364   2.17738  4.41311  3.27524  2.78827  3.73183  3.39624  3.83488
3.00523  2.73127  2.76643  3.66108  3.20013  3.87715  2.47865  3.09781
2.54592  2.83134  1.97700  5.13323  3.87689    448 v - - -
          2.68618  4.42225  2.77519  2.73123  3.46354  2.40513  3.72494
3.29354  2.67741  2.69355  4.24690  2.90347  2.73739  3.18146  2.89801
2.37887  2.77519  2.98518  4.58477  3.61503
          0.02625  4.04896  4.77131  0.61958  0.77255  0.48576  0.95510
    365   2.49727  4.90928  2.78636  2.24927  4.25339  3.35652  3.66103
3.67869  2.42617  3.25207  4.06367  2.90920  3.81798  1.96120  2.86349
2.27562  2.73203  3.31902  5.45906  4.11130    449 q - - -
          2.68618  4.42225  2.77519  2.73123  3.46354  2.40513  3.72494
3.29354  2.67741  2.69355  4.24690  2.90347  2.73739  3.18146  2.89801
2.37887  2.77519  2.98518  4.58477  3.61503
          0.02625  4.04896  4.77131  0.61958  0.77255  0.48576  0.95510
    366   2.57130  4.50649  3.10478  2.66358  3.78375  3.03729  3.77847
3.16889  2.66172  1.95534  3.72382  2.53664  3.12191  3.01549  3.05130
2.69828  2.84860  2.88838  5.15854  3.87705    450 l - - -
          2.68618  4.42225  2.77519  2.73123  3.46354  2.40513  3.72494
3.29354  2.67741  2.69355  4.24690  2.90347  2.73739  3.18146  2.89801
2.37887  2.77519  2.98518  4.58477  3.61503
          0.02625  4.04896  4.77131  0.61958  0.77255  0.48576  0.95510
```

```
    367   2.65378  4.27330  3.72078  3.16663  2.78444  3.66274  3.94581
2.40221  3.08905  1.99059  3.30899  3.50578  4.05326  3.37493  3.38789
2.00760  2.90009  2.40264  4.79993  3.50993    451 l - - -
          2.68618  4.42225  2.77519  2.73123  3.46354  2.40513  3.72494
3.29354  2.67741  2.69355  4.24690  2.90347  2.73739  3.18146  2.89801
2.37887  2.77519  2.98518  4.58477  3.61503
          0.02625  4.04896  4.77131  0.61958  0.77255  0.48576  0.95510
    368   2.46538  4.52465  2.98485  2.64947  4.22725  3.17228  3.88325
3.64547  2.74230  3.28619  4.11384  2.08595  3.77940  3.07785  3.15097
2.13928  1.90175  3.22274  5.51605  4.21427    452 t - - -
          2.68618  4.42225  2.77519  2.73123  3.46354  2.40513  3.72494
3.29354  2.67741  2.69355  4.24690  2.90347  2.73739  3.18146  2.89801
2.37887  2.77519  2.98518  4.58477  3.61503
          0.28203  4.04896  1.47705  0.61958  0.77255  0.48576  0.95510
    369   2.36895  4.32660  2.98907  2.72175  3.99739  3.06068  3.89059
3.32901  2.75175  3.09184  3.98879  2.58519  3.71113  3.13765  3.08759
2.51032  1.69290  2.95755  5.35657  4.06525    453 t - - -
          2.68618  4.42225  2.77519  2.73123  3.46354  2.40513  3.72494
3.29354  2.67741  2.69355  4.24690  2.90347  2.73739  3.18146  2.89801
2.37887  2.77519  2.98518  4.58477  3.61503
          0.03377  3.80070  4.52305  0.61958  0.77255  0.70047  0.68588
    370   2.89384  4.98523  2.46301  1.13222  4.32300  3.23008  3.81306
3.76268  2.68475  3.39227  4.37139  2.89759  3.83183  3.03148  3.08465
2.88480  3.19818  3.45469  5.50832  4.23263    454 e - - -
          2.68618  4.42225  2.77519  2.73123  3.46354  2.40513  3.72494
3.29354  2.67741  2.69355  4.24690  2.90347  2.73739  3.18146  2.89801
2.37887  2.77519  2.98518  4.58477  3.61503
          0.03377  3.80070  4.52305  0.61958  0.77255  0.70047  0.68588
    371   3.08678  4.50931  4.37806  3.87344  3.08535  4.21583  4.54803
1.84679  3.66778  1.14830  2.97970  4.17038  4.51580  3.96292  3.87180
3.58835  3.33831  2.03663  5.06470  3.83725    455 l - - -
          2.68618  4.42225  2.77519  2.73123  3.46354  2.40513  3.72494
3.29354  2.67741  2.69355  4.24690  2.90347  2.73739  3.18146  2.89801
2.37887  2.77519  2.98518  4.58477  3.61503
          0.03377  3.80070  4.52305  0.61958  0.77255  0.70047  0.68588
    372   2.79184  5.15635  1.50315  2.13628  4.46009  3.18064  3.68225
3.93786  2.28159  3.50968  4.37719  2.70014  3.76981  2.85675  3.10171
2.73133  3.07782  3.56212  5.66593  4.24550    456 d - - -
          2.68618  4.42225  2.77519  2.73123  3.46354  2.40513  3.72494
3.29354  2.67741  2.69355  4.24690  2.90347  2.73739  3.18146  2.89801
2.37887  2.77519  2.98518  4.58477  3.61503
          0.03377  3.80070  4.52305  0.61958  0.77255  0.70047  0.68588
    373   2.77798  5.08383  2.61459  1.95736  4.40553  3.34101  3.57866
3.82584  1.92402  3.34076  4.17741  2.83216  3.81397  2.31443  2.54129
2.74361  3.00581  3.47319  5.48611  4.16121    457 k - - -
          2.68632  4.42239  2.77533  2.73137  3.46368  2.40527  3.72508
3.29333  2.67713  2.69339  4.24704  2.90360  2.73753  3.18002  2.89814
2.37901  2.77533  2.98508  4.58491  3.61517
          0.35357  1.24839  4.52305  1.24463  0.33974  0.70047  0.68588
    374   2.82737  4.80749  2.85931  2.61494  3.96593  3.32051  3.77192
3.58744  2.44125  3.09678  4.12865  3.07328  3.87421  1.36763  2.72603
2.87909  3.12533  3.32221  5.25174  3.94129    463 q - - -
          2.68618  4.42225  2.77519  2.73123  3.46354  2.40513  3.72494
3.29354  2.67741  2.69355  4.24690  2.90347  2.73739  3.18146  2.89801
2.37887  2.77519  2.98518  4.58477  3.61503
```

```
          0.03377  3.80070  4.52305  0.61958  0.77255  0.70047  0.68588
    375   1.12251  4.15936  3.39209  3.18208  3.98303  2.98539  4.21228
3.09807  3.19651  2.98271  4.02407  3.34909  3.71801  3.54475  3.45090
2.51109  2.78356  2.76506  5.41748  4.21010    464 a - - -
          2.68618  4.42225  2.77519  2.73123  3.46354  2.40513  3.72494
3.29354  2.67741  2.69355  4.24690  2.90347  2.73739  3.18146  2.89801
2.37887  2.77519  2.98518  4.58477  3.61503
          0.03377  3.80070  4.52305  0.61958  0.77255  0.70047  0.68588
    376   2.65833  4.63714  3.24368  2.74312  4.08039  3.35315  3.68883
3.42054  2.18312  3.04378  3.96958  3.12849  3.85274  2.87675  1.61824
2.75257  2.55301  3.11922  5.27746  4.04065    465 r - - -
          2.68618  4.42225  2.77519  2.73123  3.46354  2.40513  3.72494
3.29354  2.67741  2.69355  4.24690  2.90347  2.73739  3.18146  2.89801
2.37887  2.77519  2.98518  4.58477  3.61503
          0.03377  3.80070  4.52305  0.61958  0.77255  0.70047  0.68588
    377   1.56283  4.33768  3.19288  2.81637  3.93915  3.14008  3.86375
3.23826  2.61991  2.96343  3.89090  3.15104  3.75107  3.10474  2.50285
2.56484  2.79114  2.90715  5.28539  4.03394    466 a - - -
          2.68618  4.42225  2.77519  2.73123  3.46354  2.40513  3.72494
3.29354  2.67741  2.69355  4.24690  2.90347  2.73739  3.18146  2.89801
2.37887  2.77519  2.98518  4.58477  3.61503
          0.03377  3.80070  4.52305  0.61958  0.77255  0.70047  0.68588
    378   2.71509  4.95616  1.49945  2.20173  4.46976  2.29712  3.76822
3.97538  2.77629  3.56128  4.42992  2.74635  3.74382  2.96308  3.30280
2.69575  3.05327  3.55625  5.67903  4.30344    467 d - - -
          2.68618  4.42225  2.77519  2.73123  3.46354  2.40513  3.72494
3.29354  2.67741  2.69355  4.24690  2.90347  2.73739  3.18146  2.89801
2.37887  2.77519  2.98518  4.58477  3.61503
          0.03377  3.80070  4.52305  0.61958  0.77255  0.70047  0.68588
    379   2.65753  4.23644  3.81810  3.28816  3.31969  3.68622  4.08848
1.73981  3.15231  2.14084  2.89276  3.60351  4.09869  3.48214  3.42589
2.67451  2.92397  2.07238  4.95610  3.72246    468 i - - -
          2.68618  4.42225  2.77519  2.73123  3.46354  2.40513  3.72494
3.29354  2.67741  2.69355  4.24690  2.90347  2.73739  3.18146  2.89801
2.37887  2.77519  2.98518  4.58477  3.61503
          0.03377  3.80070  4.52305  0.61958  0.77255  0.70047  0.68588
    380   2.55116  4.39803  3.25743  2.90475  3.63380  3.27136  3.92786
2.98124  2.80485  2.27422  3.72859  3.26009  1.83316  3.22676  3.11015
2.72223  2.90647  2.76022  5.12066  3.81519    469 p - - -
          2.68618  4.42225  2.77519  2.73123  3.46354  2.40513  3.72494
3.29354  2.67741  2.69355  4.24690  2.90347  2.73739  3.18146  2.89801
2.37887  2.77519  2.98518  4.58477  3.61503
          0.03377  3.80070  4.52305  0.61958  0.77255  0.70047  0.68588
    381   2.77491  4.26823  4.06555  3.51669  3.35445  3.90269  4.30923
1.87419  3.41012  2.01806  2.79657  3.82791  4.26793  3.70071  3.67850
3.21719  2.71445  1.56337  5.09576  3.90278    470 v - - -
          2.68618  4.42225  2.77519  2.73123  3.46354  2.40513  3.72494
3.29354  2.67741  2.69355  4.24690  2.90347  2.73739  3.18146  2.89801
2.37887  2.77519  2.98518  4.58477  3.61503
          0.03377  3.80070  4.52305  0.61958  0.77255  0.70047  0.68588
    382   2.71509  4.95616  1.49945  2.20173  4.46976  2.29712  3.76822
3.97538  2.77629  3.56128  4.42992  2.74635  3.74382  2.96308  3.30280
2.69575  3.05327  3.55625  5.67903  4.30344    471 d - - -
```

2.68618 4.42225 2.77519 2.73123 3.46354 2.40513 3.72494
3.29354 2.67741 2.69355 4.24690 2.90347 2.73739 3.18146 2.89801
2.37887 2.77519 2.98518 4.58477 3.61503
0.03377 3.80070 4.52305 0.61958 0.77255 0.70047 0.68588
383 3.12642 4.53993 4.32586 3.86377 1.70462 4.13361 4.02602
2.35636 3.72190 1.46886 3.07884 4.03306 4.45089 3.90312 3.89026
3.50281 3.37228 2.39493 4.33596 2.78585 472 l - - -
2.68618 4.42225 2.77519 2.73123 3.46354 2.40513 3.72494
3.29354 2.67741 2.69355 4.24690 2.90347 2.73739 3.18146 2.89801
2.37887 2.77519 2.98518 4.58477 3.61503
0.03377 3.80070 4.52305 0.61958 0.77255 0.70047 0.68588
384 2.65872 4.95171 1.93043 2.20975 4.28309 3.21277 3.64431
3.71602 2.51833 3.30454 4.14531 2.75480 3.75042 2.51341 3.00378
2.64241 2.44768 3.35381 5.52243 4.13135 473 d - - -
2.68618 4.42225 2.77519 2.73123 3.46354 2.40513 3.72494
3.29354 2.67741 2.69355 4.24690 2.90347 2.73739 3.18146 2.89801
2.37887 2.77519 2.98518 4.58477 3.61503
0.03377 3.80070 4.52305 0.61958 0.77255 0.70047 0.68588
385 2.60423 4.83740 2.33334 2.29240 3.38519 3.30216 3.59173
3.51329 2.43398 3.10620 3.93621 2.58216 3.75847 2.42068 2.90399
2.61416 2.84950 3.18310 5.29700 3.91438 474 e - - -
2.68618 4.42225 2.77519 2.73123 3.46354 2.40513 3.72494
3.29354 2.67741 2.69355 4.24690 2.90347 2.73739 3.18146 2.89801
2.37887 2.77519 2.98518 4.58477 3.61503
0.03377 3.80070 4.52305 0.61958 0.77255 0.37168 1.16980
386 2.58552 4.38189 3.33981 2.77876 3.13849 3.52691 3.43963
2.42671 2.71698 2.33744 3.47474 2.96753 3.90864 2.47525 3.08836
2.76738 2.81652 2.65797 4.90851 3.63113 475 l - - -
2.68618 4.42225 2.77519 2.73123 3.46354 2.40513 3.72494
3.29354 2.67741 2.69355 4.24690 2.90347 2.73739 3.18146 2.89801
2.37887 2.77519 2.98518 4.58477 3.61503
0.02625 4.04896 4.77131 0.61958 0.77255 0.48576 0.95510
387 2.50096 4.63252 2.90245 2.53926 4.27274 3.20758 3.80422
3.70848 2.64308 3.31016 4.12074 1.98813 2.92603 2.97386 3.08189
2.05396 2.56145 3.28462 5.52560 4.20114 476 n - - -
2.68618 4.42225 2.77519 2.73123 3.46354 2.40513 3.72494
3.29354 2.67741 2.69355 4.24690 2.90347 2.73739 3.18146 2.89801
2.37887 2.77519 2.98518 4.58477 3.61503
0.02625 4.04896 4.77131 0.61958 0.77255 0.48576 0.95510
388 3.21580 4.56383 4.70025 4.17738 1.48283 4.37006 4.35776
1.97233 4.03866 1.56693 3.05690 4.32631 4.62505 4.15444 4.15852
3.71020 3.44475 2.31147 4.64819 3.20213 477 f - - -
2.68618 4.42225 2.77519 2.73123 3.46354 2.40513 3.72494
3.29354 2.67741 2.69355 4.24690 2.90347 2.73739 3.18146 2.89801
2.37887 2.77519 2.98518 4.58477 3.61503
0.02625 4.04896 4.77131 0.61958 0.77255 0.48576 0.95510
389 2.87931 4.87574 2.82111 2.72621 4.26801 3.31004 4.07758
4.02015 2.99819 3.64783 4.60465 0.87973 3.97208 3.35350 3.35640
2.94155 3.26875 3.62429 5.55383 4.20588 478 n - - -
2.68618 4.42225 2.77519 2.73123 3.46354 2.40513 3.72494
3.29354 2.67741 2.69355 4.24690 2.90347 2.73739 3.18146 2.89801
2.37887 2.77519 2.98518 4.58477 3.61503
0.02625 4.04896 4.77131 0.61958 0.77255 0.48576 0.95510

```
    390   2.69646  1.53360  4.32926  3.85994  3.49315  3.66500  4.47916
1.94865  3.67472  2.30736  3.47505  3.95555  4.21913  3.97177  3.85794
3.08556  3.05019  2.07962  5.18374  3.94029    479 c - - -
          2.68618  4.42225  2.77519  2.73123  3.46354  2.40513  3.72494
3.29354  2.67741  2.69355  4.24690  2.90347  2.73739  3.18146  2.89801
2.37887  2.77519  2.98518  4.58477  3.61503
          0.02625  4.04896  4.77131  0.61958  0.77255  0.48576  0.95510
    391   2.51299  4.40425  3.28395  2.74911  3.70784  3.38789  3.79428
3.04867  2.70861  2.76226  3.19637  2.89834  3.85064  3.04421  3.09790
2.18591  2.12267  2.55751  5.09929  3.83879    480 t - - -
          2.68621  4.42228  2.77522  2.73126  3.46357  2.40515  3.72497
3.29357  2.67743  2.69358  4.24692  2.90349  2.73734  3.18149  2.89803
2.37889  2.77486  2.98521  4.58480  3.61506
          0.22621  1.63999  4.77131  0.26614  1.45384  0.48576  0.95510
    392   2.79268  4.25543  4.19452  3.62522  2.67898  3.91121  4.20097
2.18834  3.51131  1.39220  3.11426  3.86859  4.25254  3.73127  3.71104
2.88720  3.02853  2.10974  4.82364  3.59913    482 l - - -
          2.68618  4.42225  2.77519  2.73123  3.46354  2.40513  3.72494
3.29354  2.67741  2.69355  4.24690  2.90347  2.73739  3.18146  2.89801
2.37887  2.77519  2.98518  4.58477  3.61503
          0.07172  4.04896  2.96096  0.61958  0.77255  0.48576  0.95510
    393   2.62446  4.44924  3.35161  2.78549  2.96491  3.53736  3.75045
2.92704  2.63202  2.22985  3.50948  3.21682  3.38882  3.03150  2.19231
2.79069  2.85799  2.71285  4.94269  3.64886    483 r - - -
          2.68618  4.42225  2.77519  2.73123  3.46354  2.40513  3.72494
3.29354  2.67741  2.69355  4.24690  2.90347  2.73739  3.18146  2.89801
2.37887  2.77519  2.98518  4.58477  3.61503
          0.02746  4.00469  4.72704  0.61958  0.77255  0.53186  0.88555
    394   2.63258  4.75072  2.70538  2.50785  4.32631  3.20608  3.86551
3.84934  2.72650  3.45268  4.30849  1.47960  2.55302  3.06448  3.13901
2.68581  2.99085  3.42819  5.58565  4.23729    484 n - - -
          2.68618  4.42225  2.77519  2.73123  3.46354  2.40513  3.72494
3.29354  2.67741  2.69355  4.24690  2.90347  2.73739  3.18146  2.89801
2.37887  2.77519  2.98518  4.58477  3.61503
          0.02746  4.00469  4.72704  0.61958  0.77255  0.45798  1.00119
    395   2.42010  4.37109  3.19963  3.00184  4.27918  3.07677  4.17256
3.75802  3.09512  3.44042  4.32598  3.27642  1.19844  3.44538  3.40808
2.26236  2.88435  3.27683  5.61584  4.34878    485 p - - -
          2.68618  4.42225  2.77519  2.73123  3.46354  2.40513  3.72494
3.29354  2.67741  2.69355  4.24690  2.90347  2.73739  3.18146  2.89801
2.37887  2.77519  2.98518  4.58477  3.61503
          0.02625  4.04896  4.77131  0.61958  0.77255  0.48576  0.95510
    396   3.04227  4.44480  4.50436  3.96308  3.28588  4.23520  4.62311
1.67978  3.81140  1.22874  3.12324  4.22509  4.54165  4.05818  4.00567
3.57462  2.89165  1.99535  5.18505  4.03384    486 l - - -
          2.68618  4.42225  2.77519  2.73123  3.46354  2.40513  3.72494
3.29354  2.67741  2.69355  4.24690  2.90347  2.73739  3.18146  2.89801
2.37887  2.77519  2.98518  4.58477  3.61503
          0.02625  4.04896  4.77131  0.61958  0.77255  0.48576  0.95510
    397   2.61146  4.84325  2.66010  2.19011  4.09608  3.38791  3.62645
3.51619  2.42985  2.63922  3.92395  2.91218  3.80403  2.63085  2.89573
2.39076  2.39778  3.18399  5.34639  4.00102    487 e - - -
          2.68618  4.42225  2.77519  2.73123  3.46354  2.40513  3.72494
3.29354  2.67741  2.69355  4.24690  2.90347  2.73739  3.18146  2.89801
2.37887  2.77519  2.98518  4.58477  3.61503
```

0.02625 4.04896 4.77131 0.61958 0.77255 0.48576 0.95510
398 2.27200 4.22749 3.84282 3.38675 3.74892 3.40366 4.26772
2.47656 3.30898 2.63559 3.65906 3.59754 4.00094 3.61823 3.59308
2.79536 1.86013 1.61076 5.30293 4.09018 488 v - - -
2.68618 4.42225 2.77519 2.73123 3.46354 2.40513 3.72494
3.29354 2.67741 2.69355 4.24690 2.90347 2.73739 3.18146 2.89801
2.37887 2.77519 2.98518 4.58477 3.61503
0.02625 4.04896 4.77131 0.61958 0.77255 0.48576 0.95510
399 3.00224 4.72657 3.62906 3.49603 4.47053 3.40426 4.55621
4.06484 3.56912 3.67110 4.73068 3.78339 0.55633 3.95247 3.79183
3.17599 3.45834 3.68948 5.60532 4.59466 489 P - - -
2.68618 4.42225 2.77519 2.73123 3.46354 2.40513 3.72494
3.29354 2.67741 2.69355 4.24690 2.90347 2.73739 3.18146 2.89801
2.37887 2.77519 2.98518 4.58477 3.61503
0.02625 4.04896 4.77131 0.61958 0.77255 0.48576 0.95510
400 3.26055 4.62328 4.43857 4.00017 1.56495 4.18133 3.63061
2.96195 3.86433 2.43299 3.62026 3.96922 4.50989 3.95050 3.97915
3.51077 3.48965 2.51692 3.85224 1.37277 490 y - - -
2.68618 4.42225 2.77519 2.73123 3.46354 2.40513 3.72494
3.29354 2.67741 2.69355 4.24690 2.90347 2.73739 3.18146 2.89801
2.37887 2.77519 2.98518 4.58477 3.61503
0.02625 4.04896 4.77131 0.61958 0.77255 0.48576 0.95510
401 2.79075 4.21155 4.29778 3.73450 2.26957 3.90993 4.04646
2.12430 3.60367 2.06317 3.17242 3.88717 4.25053 3.78369 3.75127
3.21294 3.02232 1.83915 4.52487 2.40750 491 v - - -
2.68618 4.42225 2.77519 2.73123 3.46354 2.40513 3.72494
3.29354 2.67741 2.69355 4.24690 2.90347 2.73739 3.18146 2.89801
2.37887 2.77519 2.98518 4.58477 3.61503
0.02625 4.04896 4.77131 0.61958 0.77255 0.48576 0.95510
402 1.82423 4.57473 3.21202 2.72576 4.14770 3.29783 3.80142
3.53127 2.48457 3.16440 4.00892 3.13055 3.83380 2.98709 1.90907
2.39804 2.86998 3.16462 5.39418 4.12831 492 a - - -
2.68618 4.42225 2.77519 2.73123 3.46354 2.40513 3.72494
3.29354 2.67741 2.69355 4.24690 2.90347 2.73739 3.18146 2.89801
2.37887 2.77519 2.98518 4.58477 3.61503
0.02625 4.04896 4.77131 0.61958 0.77255 0.48576 0.95510
403 2.25351 3.25225 3.70398 3.26545 4.03513 3.03718 4.19048
3.36252 3.19826 3.11751 3.97701 3.39258 3.74303 3.49574 3.49632
1.49592 1.83025 2.93954 5.43314 4.22805 493 s - - -
2.68618 4.42225 2.77519 2.73123 3.46354 2.40513 3.72494
3.29354 2.67741 2.69355 4.24690 2.90347 2.73739 3.18146 2.89801
2.37887 2.77519 2.98518 4.58477 3.61503
0.02625 4.04896 4.77131 0.61958 0.77255 0.48576 0.95510
404 2.66259 4.62423 3.21958 2.66860 3.72369 3.49797 3.68662
3.24168 2.43214 2.88101 3.74860 2.67897 3.90043 2.91389 2.29050
2.55631 2.89611 2.96925 2.61726 3.74887 494 r - - -
2.68618 4.42225 2.77519 2.73123 3.46354 2.40513 3.72494
3.29354 2.67741 2.69355 4.24690 2.90347 2.73739 3.18146 2.89801
2.37887 2.77519 2.98518 4.58477 3.61503
0.02625 4.04896 4.77131 0.61958 0.77255 0.48576 0.95510
405 3.34056 4.68081 4.74730 4.25584 1.75982 4.44506 4.36482
2.38059 4.10848 1.07763 3.01412 4.39784 4.68662 4.20253 4.21302
3.81181 3.56936 2.49613 4.60369 3.13437 495 l - - -

2.68618 4.42225 2.77519 2.73123 3.46354 2.40513 3.72494
3.29354 2.67741 2.69355 4.24690 2.90347 2.73739 3.18146 2.89801
2.37887 2.77519 2.98518 4.58477 3.61503
0.02625 4.04896 4.77131 0.61958 0.77255 0.48576 0.95510
406 2.67692 5.09537 2.00208 2.27218 4.35006 3.34615 3.18542
3.85223 2.41850 3.37602 4.16063 2.50168 3.79727 2.43291 2.91178
2.64258 2.91713 3.46149 5.52867 3.49839 496 d - - -
2.68618 4.42225 2.77519 2.73123 3.46354 2.40513 3.72494
3.29354 2.67741 2.69355 4.24690 2.90347 2.73739 3.18146 2.89801
2.37887 2.77519 2.98518 4.58477 3.61503
0.02625 4.04896 4.77131 0.61958 0.77255 0.48576 0.95510
407 2.73723 4.99761 2.53281 1.56728 4.44573 3.27197 3.77920
3.89634 2.63895 3.48084 4.32116 2.87362 3.84315 2.94772 3.10075
1.82229 3.04221 3.50334 5.66734 4.28327 497 e - - -
2.68618 4.42225 2.77519 2.73123 3.46354 2.40513 3.72494
3.29354 2.67741 2.69355 4.24690 2.90347 2.73739 3.18146 2.89801
2.37887 2.77519 2.98518 4.58477 3.61503
0.02625 4.04896 4.77131 0.61958 0.77255 0.48576 0.95510
408 2.54222 4.30546 3.46294 2.95195 3.54296 2.25606 3.89419
2.57084 2.88377 2.05639 3.51773 3.31963 3.92074 3.21570 3.22043
2.53518 2.82825 2.58082 5.00414 3.75627 498 l - - -
2.68618 4.42225 2.77519 2.73123 3.46354 2.40513 3.72494
3.29354 2.67741 2.69355 4.24690 2.90347 2.73739 3.18146 2.89801
2.37887 2.77519 2.98518 4.58477 3.61503
0.07670 4.04896 2.87536 0.61958 0.77255 0.48576 0.95510
409 2.45929 4.26019 3.52717 3.02334 3.68429 3.33328 3.97911
2.59280 2.95253 2.68800 3.62388 3.34231 3.87765 3.28184 3.28789
2.33679 1.76251 2.34324 5.14330 3.90706 499 t - - -
2.68618 4.42225 2.77519 2.73123 3.46354 2.40513 3.72494
3.29354 2.67741 2.69355 4.24690 2.90347 2.73739 3.18146 2.89801
2.37887 2.77519 2.98518 4.58477 3.61503
0.02759 3.99985 4.72219 0.61958 0.77255 0.53666 0.87875
410 2.64264 4.91562 2.04057 2.31775 4.21702 2.93307 3.66640
3.68511 2.52107 3.26435 4.07878 2.85355 3.79440 2.82185 3.01069
2.08905 2.90691 3.32222 5.46589 3.46594 500 d - - -
2.68618 4.42225 2.77519 2.73123 3.46354 2.40513 3.72494
3.29354 2.67741 2.69355 4.24690 2.90347 2.73739 3.18146 2.89801
2.37887 2.77519 2.98518 4.58477 3.61503
0.08280 3.99985 2.79441 0.61958 0.77255 0.53666 0.87875
411 2.66546 4.64950 3.25130 2.69502 3.91191 3.45274 3.68497
3.30900 2.29855 2.50730 3.83190 3.12504 3.88637 2.88227 1.85768
2.37283 2.91593 3.02901 5.19120 3.91540 501 r - - -
2.68618 4.42225 2.77519 2.73123 3.46354 2.40513 3.72494
3.29354 2.67741 2.69355 4.24690 2.90347 2.73739 3.18146 2.89801
2.37887 2.77519 2.98518 4.58477 3.61503
0.02913 3.94618 4.66852 0.61958 0.77255 0.42735 1.05622
412 2.74653 5.02969 2.64214 1.81417 4.40459 2.01687 3.70716
3.84683 2.47212 3.40007 4.22348 2.89049 3.84890 2.85957 2.60629
2.73513 3.01198 3.47274 5.57785 4.22194 502 e - - -
2.68618 4.42225 2.77519 2.73123 3.46354 2.40513 3.72494
3.29354 2.67741 2.69355 4.24690 2.90347 2.73739 3.18146 2.89801
2.37887 2.77519 2.98518 4.58477 3.61503
0.02625 4.04896 4.77131 0.61958 0.77255 0.48576 0.95510

```
    413   2.58311  4.74370  2.75552  2.44309  4.00926  2.67294  3.64622
3.42376  2.46099  3.03729  3.85917  2.95451  3.80693  2.80798  2.74581
2.46540  2.30404  3.10240  5.28854  3.46258    503 t - - -
          2.68618  4.42225  2.77519  2.73123  3.46354  2.40513  3.72494
3.29354  2.67741  2.69355  4.24690  2.90347  2.73739  3.18146  2.89801
2.37887  2.77519  2.98518  4.58477  3.61503
          0.02625  4.04896  4.77131  0.61958  0.77255  0.48576  0.95510
    414   1.85889  4.23496  3.70076  3.18360  3.55292  3.47740  4.05331
2.56731  3.10723  2.22089  3.50185  3.48226  3.98052  3.41387  3.41300
2.55374  2.85044  1.91184  5.06275  3.83953    504 a - - -
          2.68618  4.42225  2.77519  2.73123  3.46354  2.40513  3.72494
3.29354  2.67741  2.69355  4.24690  2.90347  2.73739  3.18146  2.89801
2.37887  2.77519  2.98518  4.58477  3.61503
          0.07172  4.04896  2.96096  0.61958  0.77255  0.48576  0.95510
    415   1.52291  4.16670  3.37574  3.11003  4.33278  1.70480  4.21603
3.74210  3.21090  3.43375  4.25387  3.27755  3.68225  3.48350  3.53325
2.11023  2.70490  3.18194  5.67029  4.45687    505 a - - -
          2.68618  4.42225  2.77519  2.73123  3.46354  2.40513  3.72494
3.29354  2.67741  2.69355  4.24690  2.90347  2.73739  3.18146  2.89801
2.37887  2.77519  2.98518  4.58477  3.61503
          0.02746  4.00469  4.72704  0.61958  0.77255  0.53186  0.88555
    416   2.36194  4.26113  3.77674  3.25632  3.59555  3.55887  4.14574
2.17471  3.17863  2.47175  3.50452  3.56068  4.05246  3.49021  3.48960
2.89514  1.86233  1.92398  5.15768  3.93676    506 t - - -
          2.68618  4.42225  2.77519  2.73123  3.46354  2.40513  3.72494
3.29354  2.67741  2.69355  4.24690  2.90347  2.73739  3.18146  2.89801
2.37887  2.77519  2.98518  4.58477  3.61503
          0.02746  4.00469  4.72704  0.61958  0.77255  0.45798  1.00119
    417   2.47055  4.46478  3.14413  2.69537  3.97686  2.01299  3.83097
3.36436  2.69949  3.02857  3.87398  3.11076  3.80026  2.75301  3.09950
2.28924  2.60988  2.58922  5.31171  4.03483    507 g - - -
          2.68618  4.42225  2.77519  2.73123  3.46354  2.40513  3.72494
3.29354  2.67741  2.69355  4.24690  2.90347  2.73739  3.18146  2.89801
2.37887  2.77519  2.98518  4.58477  3.61503
          0.02625  4.04896  4.77131  0.61958  0.77255  0.48576  0.95510
    418   2.50839  4.52541  3.02892  2.67155  4.06674  1.91467  3.23434
3.50918  2.70886  3.15371  4.00116  3.09209  3.81082  3.07055  3.09609
2.61693  2.02546  3.13776  5.38800  4.08897    508 g - - -
          2.68618  4.42225  2.77519  2.73123  3.46354  2.40513  3.72494
3.29354  2.67741  2.69355  4.24690  2.90347  2.73739  3.18146  2.89801
2.37887  2.77519  2.98518  4.58477  3.61503
          0.02625  4.04896  4.77131  0.61958  0.77255  0.48576  0.95510
    419   2.92589  4.59936  3.62304  3.15281  1.90718  3.78828  3.61779
3.13114  3.11304  2.71010  3.72046  2.06299  4.17352  3.38114  3.43138
3.07784  3.16255  2.93044  4.14946  2.09003    509 f - - -
          2.68618  4.42225  2.77519  2.73123  3.46354  2.40513  3.72494
3.29354  2.67741  2.69355  4.24690  2.90347  2.73739  3.18146  2.89801
2.37887  2.77519  2.98518  4.58477  3.61503
          0.02625  4.04896  4.77131  0.61958  0.77255  0.48576  0.95510
    420   2.82396  4.25340  4.31422  3.73834  3.03502  3.95737  4.19296
2.05433  3.58969  1.68529  3.08680  3.93739  4.28519  3.79676  3.75177
3.26105  3.05494  2.00256  2.41701  3.46439    510 l - - -
          2.68618  4.42225  2.77519  2.73123  3.46354  2.40513  3.72494
3.29354  2.67741  2.69355  4.24690  2.90347  2.73739  3.18146  2.89801
2.37887  2.77519  2.98518  4.58477  3.61503
```

```
          0.02625  4.04896  4.77131  0.61958  0.77255  0.48576  0.95510
     421  2.85661  4.96295  3.31600  2.68957  4.24873  3.58938  3.63735
 3.64074  2.08180  3.16091  3.49971  3.13445  3.96499  2.31376  1.51883
 2.88695  3.05588  3.34820  5.34846  4.10403    511 r - - -
          2.68618  4.42225  2.77519  2.73123  3.46354  2.40513  3.72494
 3.29354  2.67741  2.69355  4.24690  2.90347  2.73739  3.18146  2.89801
 2.37887  2.77519  2.98518  4.58477  3.61503
          0.02625  4.04896  4.77131  0.61958  0.77255  0.48576  0.95510
     422  2.49506  4.52172  3.09385  2.68751  4.11877  3.23843  3.84458
 3.50166  2.40382  3.15828  4.00048  3.10096  3.80563  3.04295  3.02309
 1.74607  2.02004  3.12714  5.41293  4.13226    512 s - - -
          2.68618  4.42225  2.77519  2.73123  3.46354  2.40513  3.72494
 3.29354  2.67741  2.69355  4.24690  2.90347  2.73739  3.18146  2.89801
 2.37887  2.77519  2.98518  4.58477  3.61503
          0.02625  4.04896  4.77131  0.61958  0.77255  0.48576  0.95510
     423  2.93996  5.50067  1.36198  1.77634  4.81084  2.60595  3.78017
 4.32733  2.78887  3.82346  4.66344  2.71207  3.85969  2.94267  3.38458
 2.83075  3.22811  3.89430  5.98426  4.48301    513 d - - -
          2.68618  4.42225  2.77519  2.73123  3.46354  2.40513  3.72494
 3.29354  2.67741  2.69355  4.24690  2.90347  2.73739  3.18146  2.89801
 2.37887  2.77519  2.98518  4.58477  3.61503
          0.02625  4.04896  4.77131  0.61958  0.77255  0.48576  0.95510
     424  2.32728  4.22765  3.57225  3.05465  3.58006  2.96329  3.96261
 2.56198  3.00141  2.62671  3.53695  3.37088  3.89806  3.30253  3.32794
 2.15594  2.79263  1.87807  5.03817  3.81325    514 v - - -
          2.68618  4.42225  2.77519  2.73123  3.46354  2.40513  3.72494
 3.29354  2.67741  2.69355  4.24690  2.90347  2.73739  3.18146  2.89801
 2.37887  2.77519  2.98518  4.58477  3.61503
          0.02625  4.04896  4.77131  0.61958  0.77255  0.48576  0.95510
     425  3.49106  4.84211  4.48107  4.23211  0.76177  4.13322  3.96743
 2.99649  4.13553  2.32725  3.70244  4.22219  4.59014  4.25557  4.21149
 3.74980  3.79345  2.99963  4.16985  2.51707    515 f - - -
          2.68638  4.42245  2.77540  2.73108  3.46374  2.40514  3.72515
 3.29341  2.67761  2.69300  4.24662  2.90354  2.73760  3.18154  2.89808
 2.37901  2.77438  2.98525  4.58497  3.61523
          0.28203  1.43851  4.77131  1.52819  0.24453  0.48576  0.95510
     426  2.73503  4.79740  3.23135  2.63628  4.06780  3.52723  3.65287
 2.86590  2.09613  3.03846  3.89612  3.09794  3.38904  2.82012  1.73192
 2.79471  2.95180  3.14323  5.27067  4.01033    522 r - - -
          2.68618  4.42225  2.77519  2.73123  3.46354  2.40513  3.72494
 3.29354  2.67741  2.69355  4.24690  2.90347  2.73739  3.18146  2.89801
 2.37887  2.77519  2.98518  4.58477  3.61503
          0.02625  4.04896  4.77131  0.61958  0.77255  0.48576  0.95510
     427  2.44298  3.07173  3.71746  3.20957  2.79813  3.33206  4.00791
 2.86463  3.13623  2.62290  3.55145  3.44562  3.89183  3.41954  3.42617
 1.60945  2.79031  2.60445  4.95102  3.66527    523 s - - -
          2.68618  4.42225  2.77519  2.73123  3.46354  2.40513  3.72494
 3.29354  2.67741  2.69355  4.24690  2.90347  2.73739  3.18146  2.89801
 2.37887  2.77519  2.98518  4.58477  3.61503
          0.02625  4.04896  4.77131  0.61958  0.77255  0.48576  0.95510
     428  2.72659  5.08751  2.49689  1.87983  4.47968  2.36800  3.71288
 3.94581  2.57776  3.48625  4.29463  2.22323  3.81848  2.86136  3.07933
 2.35295  3.00518  3.54154  5.67069  4.26541    524 e - - -
```

```
          2.68618  4.42225  2.77519  2.73123  3.46354  2.40513  3.72494
3.29354  2.67741  2.69355  4.24690  2.90347  2.73739  3.18146  2.89801
2.37887  2.77519  2.98518  4.58477  3.61503
          0.02625  4.04896  4.77131  0.61958  0.77255  0.48576  0.95510
    429   2.10898  4.73065  1.94142  2.48861  4.05091  3.36887  3.76182
2.76140  2.62982  3.03883  3.92637  2.99404  3.85411  2.94173  3.08892
2.69657  2.90681  2.96130  5.39041  4.05618    525 d - - -
          2.68618  4.42225  2.77519  2.73123  3.46354  2.40513  3.72494
3.29354  2.67741  2.69355  4.24690  2.90347  2.73739  3.18146  2.89801
2.37887  2.77519  2.98518  4.58477  3.61503
          0.02625  4.04896  4.77131  0.61958  0.77255  0.48576  0.95510
    430   2.51602  4.35834  3.28061  2.83596  2.23872  2.94854  3.84482
3.07174  2.85350  2.76512  3.65041  2.90055  3.86822  3.16126  3.22134
2.07079  2.82196  2.79372  5.00275  3.68213    526 s - - -
          2.68618  4.42225  2.77519  2.73123  3.46354  2.40513  3.72494
3.29354  2.67741  2.69355  4.24690  2.90347  2.73739  3.18146  2.89801
2.37887  2.77519  2.98518  4.58477  3.61503
          0.02625  4.04896  4.77131  0.61958  0.77255  0.48576  0.95510
    431   2.60502  4.22988  3.67400  3.10843  3.38055  3.62795  3.94307
2.36676  3.03592  2.25115  3.06509  3.46120  4.01713  3.32839  3.34568
2.57966  2.14644  2.10746  4.88748  3.66913    527 v - - -
          2.68618  4.42225  2.77519  2.73123  3.46354  2.40513  3.72494
3.29354  2.67741  2.69355  4.24690  2.90347  2.73739  3.18146  2.89801
2.37887  2.77519  2.98518  4.58477  3.61503
          0.22621  4.04896  1.68735  0.61958  0.77255  0.48576  0.95510
    432   2.34941  2.05357  3.75631  3.29633  3.54020  2.69444  4.07419
2.73734  3.15542  2.32751  3.56417  3.45451  3.81887  3.47975  3.40137
2.60898  2.73594  2.47508  5.03545  3.80344    528 c - - -
          2.68618  4.42225  2.77519  2.73123  3.46354  2.40513  3.72494
3.29354  2.67741  2.69355  4.24690  2.90347  2.73739  3.18146  2.89801
2.37887  2.77519  2.98518  4.58477  3.61503
          0.03197  3.85472  4.57706  0.61958  0.77255  0.66167  0.72565
    433   2.24150  4.19703  3.06089  2.90210  4.25624  1.39622  4.10660
3.73546  3.08431  3.42456  4.27761  3.14979  3.64451  3.38389  3.40236
2.09470  2.72732  3.19302  5.58013  4.33156    529 g - - -
          2.68618  4.42225  2.77519  2.73123  3.46354  2.40513  3.72494
3.29354  2.67741  2.69355  4.24690  2.90347  2.73739  3.18146  2.89801
2.37887  2.77519  2.98518  4.58477  3.61503
          0.09638  3.85472  2.64928  0.61958  0.77255  0.66167  0.72565
    434   3.06727  4.55452  4.05968  3.70142  3.10885  3.87826  4.38233
2.31131  3.46985  0.98072  3.16092  3.98436  4.32894  3.85747  3.65813
3.44295  3.36606  2.32740  4.95684  3.66097    530 l - - -
          2.68618  4.42225  2.77519  2.73123  3.46354  2.40513  3.72494
3.29354  2.67741  2.69355  4.24690  2.90347  2.73739  3.18146  2.89801
2.37887  2.77519  2.98518  4.58477  3.61503
          0.03406  3.79240  4.51475  0.61958  0.77255  0.70612  0.68034
    435   1.93866  4.14901  3.10661  2.92989  4.19218  1.50204  4.09844
3.54719  3.07522  3.29879  4.18560  3.16003  3.62300  3.38226  3.38127
2.38589  2.69964  3.06047  5.52352  4.30817    531 g - - -
          2.68618  4.42225  2.77519  2.73123  3.46354  2.40513  3.72494
3.29354  2.67741  2.69355  4.24690  2.90347  2.73739  3.18146  2.89801
2.37887  2.77519  2.98518  4.58477  3.61503
          0.03406  3.79240  4.51475  0.61958  0.77255  0.62169  0.77010
```

```
    436   2.47207  4.41800  3.13330  2.64121  3.76395  3.30179  3.73123
3.11013  2.59325  2.80782  3.21153  2.76543  3.21832  2.95798  2.97990
2.60325  2.13476  2.81934  5.12966  3.85635    532 t - - -
          2.68618  4.42225  2.77519  2.73123  3.46354  2.40513  3.72494
3.29354  2.67741  2.69355  4.24690  2.90347  2.73739  3.18146  2.89801
2.37887  2.77519  2.98518  4.58477  3.61503
          0.09394  3.85472  2.68112  0.61958  0.77255  0.66167  0.72565
    437   2.43802  4.42187  2.96626  2.57105  3.20676  2.61549  3.72629
3.24668  2.63516  2.91219  3.77095  2.51647  3.73988  2.96095  3.04253
2.32406  2.75514  2.92143  5.14574  3.82291    533 s - - -
          2.68618  4.42225  2.77519  2.73123  3.46354  2.40513  3.72494
3.29354  2.67741  2.69355  4.24690  2.90347  2.73739  3.18146  2.89801
2.37887  2.77519  2.98518  4.58477  3.61503
          0.03398  3.79475  4.51710  0.61958  0.77255  0.43251  1.04662
    438   2.03898  4.28904  3.49415  2.95911  2.89513  3.53645  3.79729
2.79001  2.90697  2.49214  3.42072  3.32974  3.41934  3.20667  3.24023
2.81244  2.83516  2.57202  4.72747  2.48437    534 a - - -
          2.68618  4.42225  2.77519  2.73123  3.46354  2.40513  3.72494
3.29354  2.67741  2.69355  4.24690  2.90347  2.73739  3.18146  2.89801
2.37887  2.77519  2.98518  4.58477  3.61503
          0.02759  3.99985  4.72219  0.61958  0.77255  0.45520  1.00599
    439   2.34742  4.80533  2.75942  2.42021  4.09238  3.38735  3.62429
3.51188  2.40142  2.85860  3.91363  2.93623  3.36856  2.77037  2.28155
2.37949  2.82816  3.17340  5.33377  3.99658    535 r - - -
          2.68618  4.42225  2.77519  2.73123  3.46354  2.40513  3.72494
3.29354  2.67741  2.69355  4.24690  2.90347  2.73739  3.18146  2.89801
2.37887  2.77519  2.98518  4.58477  3.61503
          0.02625  4.04896  4.77131  0.61958  0.77255  0.48576  0.95510
    440   2.28384  4.23230  3.39697  3.06507  4.21706  1.60790  4.14861
3.61213  3.10701  3.31598  4.15429  3.28337  3.73291  3.40901  3.44333
1.75202  2.74461  2.69115  5.56980  4.33233    536 g - - -
          2.68618  4.42225  2.77519  2.73123  3.46354  2.40513  3.72494
3.29354  2.67741  2.69355  4.24690  2.90347  2.73739  3.18146  2.89801
2.37887  2.77519  2.98518  4.58477  3.61503
          0.02625  4.04896  4.77131  0.61958  0.77255  0.48576  0.95510
    441   2.19624  5.03079  2.40613  2.29055  4.37981  2.78222  3.66716
3.83496  2.33946  3.38203  4.17983  2.14769  3.80330  2.80652  2.99473
2.65677  2.93798  3.44548  5.57282  4.18115    537 n - - -
          2.68618  4.42225  2.77519  2.73123  3.46354  2.40513  3.72494
3.29354  2.67741  2.69355  4.24690  2.90347  2.73739  3.18146  2.89801
2.37887  2.77519  2.98518  4.58477  3.61503
          0.02625  4.04896  4.77131  0.61958  0.77255  0.48576  0.95510
    442   2.63682  4.52803  3.24183  2.73989  3.51176  3.47959  3.73140
3.14815  2.60913  2.79810  3.69020  3.17276  3.91169  2.60353  2.96014
2.00724  2.89077  2.88732  2.72816  3.56781    538 s - - -
          2.68618  4.42225  2.77519  2.73123  3.46354  2.40513  3.72494
3.29354  2.67741  2.69355  4.24690  2.90347  2.73739  3.18146  2.89801
2.37887  2.77519  2.98518  4.58477  3.61503
          0.02625  4.04896  4.77131  0.61958  0.77255  0.48576  0.95510
    443   2.52459  4.56322  2.94237  2.66807  4.21646  3.19575  3.91753
3.60495  2.76771  3.29648  4.15851  1.88957  3.81627  3.12701  3.15200
2.62658  1.72665  3.20949  5.52535  4.21244    539 t - - -
          2.68618  4.42225  2.77519  2.73123  3.46354  2.40513  3.72494
3.29354  2.67741  2.69355  4.24690  2.90347  2.73739  3.18146  2.89801
2.37887  2.77519  2.98518  4.58477  3.61503
```

```
          0.02625  4.04896  4.77131  0.61958  0.77255  0.48576  0.95510
    444   2.92146  4.47731  3.89944  3.41400  1.98366  3.83899  3.65620
2.98717  3.33499  2.59107  3.61251  3.62626  4.22517  3.55871  3.58514
2.28961  3.16580  2.80061  4.08363  1.65975    540 y - - -
          2.68618  4.42225  2.77519  2.73123  3.46354  2.40513  3.72494
3.29354  2.67741  2.69355  4.24690  2.90347  2.73739  3.18146  2.89801
2.37887  2.77519  2.98518  4.58477  3.61503
          0.02625  4.04896  4.77131  0.61958  0.77255  0.48576  0.95510
    445   3.68112  4.90986  4.65165  4.34601  1.34478  4.41840  3.48408
3.36818  4.19131  2.69313  3.96341  4.09289  4.72658  4.15346  4.22122
3.78964  3.90279  3.29361  3.62126  1.07075    541 y - - -
          2.68618  4.42225  2.77519  2.73123  3.46354  2.40513  3.72494
3.29354  2.67741  2.69355  4.24690  2.90347  2.73739  3.18146  2.89801
2.37887  2.77519  2.98518  4.58477  3.61503
          0.02625  4.04896  4.77131  0.61958  0.77255  0.48576  0.95510
    446   2.42655  4.37551  3.20086  3.00638  4.27679  3.08051  4.17647
3.75755  3.09881  3.44111  4.33058  3.28096  1.18139  3.45124  3.40971
2.29173  2.89108  3.27895  5.61412  4.34674    542 p - - -
          2.68618  4.42225  2.77519  2.73123  3.46354  2.40513  3.72494
3.29354  2.67741  2.69355  4.24690  2.90347  2.73739  3.18146  2.89801
2.37887  2.77519  2.98518  4.58477  3.61503
          0.02625  4.04896  4.77131  0.61958  0.77255  0.48576  0.95510
    447   3.18840  5.33296  0.69448  2.46210  4.73586  3.35266  4.09910
4.36616  3.21856  3.94434  4.92964  3.02608  4.01823  3.34089  3.75621
3.13313  3.53189  3.98763  5.86922  4.58391    543 d - - -
          2.68618  4.42225  2.77519  2.73123  3.46354  2.40513  3.72494
3.29354  2.67741  2.69355  4.24690  2.90347  2.73739  3.18146  2.89801
2.37887  2.77519  2.98518  4.58477  3.61503
          0.02625  4.04896  4.77131  0.61958  0.77255  0.48576  0.95510
    448   3.51130  4.92842  4.10319  3.86868  2.32121  4.03918  3.71497
3.49696  3.72133  2.90449  4.15421  3.94722  4.51032  3.98439  3.86748
3.62915  3.79964  3.37626  3.97068  0.71613    544 y - - -
          2.68618  4.42225  2.77519  2.73123  3.46354  2.40513  3.72494
3.29354  2.67741  2.69355  4.24690  2.90347  2.73739  3.18146  2.89801
2.37887  2.77519  2.98518  4.58477  3.61503
          0.02625  4.04896  4.77131  0.61958  0.77255  0.48576  0.95510
    449   3.68026  4.90609  4.66170  4.35677  1.25234  4.42094  3.48542
3.36150  4.20093  2.68562  3.95609  4.09735  4.72794  4.15858  4.22669
3.79199  3.90154  3.28817  3.62154  1.14849    545 y - - -
          2.68618  4.42225  2.77519  2.73123  3.46354  2.40513  3.72494
3.29354  2.67741  2.69355  4.24690  2.90347  2.73739  3.18146  2.89801
2.37887  2.77519  2.98518  4.58477  3.61503
          0.02625  4.04896  4.77131  0.61958  0.77255  0.48576  0.95510
    450   3.17037  4.54472  4.57938  4.20466  3.49871  4.25159  4.90309
0.88271  4.03620  2.05240  3.41046  4.42573  4.66901  4.36848  4.21304
3.76696  3.46771  1.86689  5.42361  4.17486    546 i - - -
          2.68618  4.42225  2.77519  2.73123  3.46354  2.40513  3.72494
3.29354  2.67741  2.69355  4.24690  2.90347  2.73739  3.18146  2.89801
2.37887  2.77519  2.98518  4.58477  3.61503
          0.02625  4.04896  4.77131  0.61958  0.77255  0.48576  0.95510
    451   2.67070  4.66848  3.30952  2.79372  4.14534  3.40696  3.78892
3.48827  2.36227  3.12625  4.01526  3.19211  3.90889  2.97575  1.79614
2.76968  1.82213  3.16829  5.36793  4.12246    547 r - - -
```

2.68618 4.42225 2.77519 2.73123 3.46354 2.40513 3.72494
3.29354 2.67741 2.69355 4.24690 2.90347 2.73739 3.18146 2.89801
2.37887 2.77519 2.98518 4.58477 3.61503
0.02625 4.04896 4.77131 0.61958 0.77255 0.48576 0.95510
452 2.87931 4.87574 2.82111 2.72621 4.26801 3.31004 4.07758
4.02015 2.99819 3.64783 4.60465 0.87973 3.97208 3.35350 3.35640
2.94155 3.26875 3.62429 5.55383 4.20588 548 n - - -
2.68618 4.42225 2.77519 2.73123 3.46354 2.40513 3.72494
3.29354 2.67741 2.69355 4.24690 2.90347 2.73739 3.18146 2.89801
2.37887 2.77519 2.98518 4.58477 3.61503
0.02625 4.04896 4.77131 0.61958 0.77255 0.48576 0.95510
453 3.17037 4.54472 4.57938 4.20466 3.49871 4.25159 4.90309
0.88271 4.03620 2.05240 3.41046 4.42573 4.66901 4.36848 4.21304
3.76696 3.46771 1.86689 5.42361 4.17486 549 i - - -
2.68618 4.42225 2.77519 2.73123 3.46354 2.40513 3.72494
3.29354 2.67741 2.69355 4.24690 2.90347 2.73739 3.18146 2.89801
2.37887 2.77519 2.98518 4.58477 3.61503
0.02625 4.04896 4.77131 0.61958 0.77255 0.48576 0.95510
454 2.41035 4.33126 3.36913 3.19663 4.25684 3.05832 4.29982
3.80508 3.28244 3.51284 4.42630 3.39050 3.81243 3.61846 3.56056
0.85292 2.90434 3.30106 5.61579 4.34228 550 s - - -
2.68618 4.42225 2.77519 2.73123 3.46354 2.40513 3.72494
3.29354 2.67741 2.69355 4.24690 2.90347 2.73739 3.18146 2.89801
2.37887 2.77519 2.98518 4.58477 3.61503
0.02625 4.04896 4.77131 0.61958 0.77255 0.48576 0.95510
455 1.30836 4.20475 3.43894 3.20853 4.37373 1.63719 4.30429
3.73902 3.31386 3.47468 4.32092 3.35423 3.73355 3.58997 3.61367
2.45220 2.76895 3.19910 5.71308 4.51748 551 a - - -
2.68618 4.42225 2.77519 2.73123 3.46354 2.40513 3.72494
3.29354 2.67741 2.69355 4.24690 2.90347 2.73739 3.18146 2.89801
2.37887 2.77519 2.98518 4.58477 3.61503
0.02625 4.04896 4.77131 0.61958 0.77255 0.48576 0.95510
456 2.73131 4.30109 4.02917 3.51136 3.57498 3.77264 4.34254
2.00234 3.41254 2.35014 3.46524 3.79199 4.23199 3.72119 3.69287
3.11690 1.89188 1.55878 5.23146 4.01421 552 v - - -
2.68618 4.42225 2.77519 2.73123 3.46354 2.40513 3.72494
3.29354 2.67741 2.69355 4.24690 2.90347 2.73739 3.18146 2.89801
2.37887 2.77519 2.98518 4.58477 3.61503
0.02625 4.04896 4.77131 0.61958 0.77255 0.48576 0.95510
457 2.70800 4.29320 3.93784 3.42677 3.51170 3.72881 4.25019
2.08861 3.32734 2.33266 3.43122 3.71862 2.88790 3.63814 3.60598
3.06755 2.99801 1.36720 5.13990 3.91680 553 v - - -
2.68618 4.42225 2.77519 2.73123 3.46354 2.40513 3.72494
3.29354 2.67741 2.69355 4.24690 2.90347 2.73739 3.18146 2.89801
2.37887 2.77519 2.98518 4.58477 3.61503
0.02625 4.04896 4.77131 0.61958 0.77255 0.48576 0.95510
458 2.70567 4.82736 3.10220 2.55658 4.08556 3.16181 3.64523
3.49173 2.27541 2.55127 3.92467 3.03967 3.88216 1.97284 2.33239
2.74894 2.92996 3.18717 5.29851 4.00791 554 q - - -
2.68618 4.42225 2.77519 2.73123 3.46354 2.40513 3.72494
3.29354 2.67741 2.69355 4.24690 2.90347 2.73739 3.18146 2.89801
2.37887 2.77519 2.98518 4.58477 3.61503
0.02625 4.04896 4.77131 0.61958 0.77255 0.48576 0.95510

```
    459   2.60177  4.34480  3.13679  2.89874  3.55438  3.54708  3.88325
2.46334  2.87441  2.57080  3.49840  3.00649  3.96490  3.19009  3.23631
2.82665  2.67585  1.70677  5.01769  3.77157    555 v - - -
          2.68618  4.42225  2.77519  2.73123  3.46354  2.40513  3.72494
3.29354  2.67741  2.69355  4.24690  2.90347  2.73739  3.18146  2.89801
2.37887  2.77519  2.98518  4.58477  3.61503
          0.02625  4.04896  4.77131  0.61958  0.77255  0.48576  0.95510
    460   2.59930  4.58228  2.72157  2.58381  3.77815  3.46741  3.71382
2.55904  2.58362  2.66460  3.68085  2.38884  3.86786  2.92114  3.01305
2.70590  2.83670  2.52650  5.14953  3.85337    556 n - - -
          2.68618  4.42225  2.77519  2.73123  3.46354  2.40513  3.72494
3.29354  2.67741  2.69355  4.24690  2.90347  2.73739  3.18146  2.89801
2.37887  2.77519  2.98518  4.58477  3.61503
          0.02625  4.04896  4.77131  0.61958  0.77255  0.48576  0.95510
    461   2.67327  5.03174  2.51126  2.16987  4.35359  2.32288  3.63621
3.80370  2.41578  3.34364  4.13745  2.85379  3.80612  2.76903  2.32128
2.50210  2.91976  3.42218  5.52601  4.14687    557 e - - -
          2.68618  4.42225  2.77519  2.73123  3.46354  2.40513  3.72494
3.29354  2.67741  2.69355  4.24690  2.90347  2.73739  3.18146  2.89801
2.37887  2.77519  2.98518  4.58477  3.61503
          0.02625  4.04896  4.77131  0.61958  0.77255  0.48576  0.95510
    462   2.61961  4.74538  2.91132  2.31526  4.02773  3.38862  3.68579
3.07551  2.49452  3.04918  3.89024  2.15528  3.83286  2.85787  2.92757
2.67189  2.24946  3.08291  5.31687  3.99561    558 n - - -
          2.68618  4.42225  2.77519  2.73123  3.46354  2.40513  3.72494
3.29354  2.67741  2.69355  4.24690  2.90347  2.73739  3.18146  2.89801
2.37887  2.77519  2.98518  4.58477  3.61503
          0.02625  4.04896  4.77131  0.61958  0.77255  0.48576  0.95510
    463   2.48143  4.75077  2.96614  2.11117  3.98777  3.41636  3.64600
3.04966  2.44927  3.00380  3.42656  2.26522  3.82232  2.80938  2.89441
2.65066  2.83909  3.05486  5.27452  3.95048    559 e - - -
          2.68618  4.42225  2.77519  2.73123  3.46354  2.40513  3.72494
3.29354  2.67741  2.69355  4.24690  2.90347  2.73739  3.18146  2.89801
2.37887  2.77519  2.98518  4.58477  3.61503
          0.02625  4.04896  4.77131  0.61958  0.77255  0.48576  0.95510
    464   2.62872  4.91804  2.20588  2.34676  4.19673  3.36534  3.62928
3.26431  2.43224  3.20550  4.00960  2.69079  3.79978  2.57410  2.90842
2.63200  2.28952  3.27376  5.42143  4.05767    560 d - - -
          2.68618  4.42225  2.77519  2.73123  3.46354  2.40513  3.72494
3.29354  2.67741  2.69355  4.24690  2.90347  2.73739  3.18146  2.89801
2.37887  2.77519  2.98518  4.58477  3.61503
          0.02625  4.04896  4.77131  0.61958  0.77255  0.48576  0.95510
    465   2.71942  4.87577  1.91250  2.39450  4.04781  3.36774  3.73412
3.46930  2.62675  2.09555  4.00830  2.69478  3.86419  2.92506  3.09377
2.74617  2.98046  3.17253  5.38566  4.00976    561 d - - -
          2.68618  4.42225  2.77519  2.73123  3.46354  2.40513  3.72494
3.29354  2.67741  2.69355  4.24690  2.90347  2.73739  3.18146  2.89801
2.37887  2.77519  2.98518  4.58477  3.61503
          0.02625  4.04896  4.77131  0.61958  0.77255  0.48576  0.95510
    466   2.42642  4.41515  3.31812  2.76714  3.65879  3.45477  3.77969
2.45444  2.66505  2.70326  3.58693  3.19264  3.88385  3.03734  2.54083
2.54802  2.21724  2.71984  5.05456  3.80230    562 t - - -
          2.68618  4.42225  2.77519  2.73123  3.46354  2.40513  3.72494
3.29354  2.67741  2.69355  4.24690  2.90347  2.73739  3.18146  2.89801
2.37887  2.77519  2.98518  4.58477  3.61503
```

0.02625 4.04896 4.77131 0.61958 0.77255 0.48576 0.95510
467 2.70028 4.87903 3.10047 2.54324 4.23416 3.45834 3.63666
3.63187 2.00658 3.19438 4.01785 3.02463 3.86950 2.78113 1.99819
2.40968 2.36372 3.29393 5.37810 4.09079 563 r - - -
2.68618 4.42225 2.77519 2.73123 3.46354 2.40513 3.72494
3.29354 2.67741 2.69355 4.24690 2.90347 2.73739 3.18146 2.89801
2.37887 2.77519 2.98518 4.58477 3.61503
0.02625 4.04896 4.77131 0.61958 0.77255 0.48576 0.95510
468 3.00224 4.72657 3.62906 3.49603 4.47053 3.40426 4.55621
4.06484 3.56912 3.67110 4.73068 3.78339 0.55633 3.95247 3.79183
3.17599 3.45834 3.68948 5.60532 4.59466 564 P - - -
2.68618 4.42225 2.77519 2.73123 3.46354 2.40513 3.72494
3.29354 2.67741 2.69355 4.24690 2.90347 2.73739 3.18146 2.89801
2.37887 2.77519 2.98518 4.58477 3.61503
0.02625 4.04896 4.77131 0.61958 0.77255 0.48576 0.95510
469 3.35841 4.77029 4.47216 4.11619 3.21164 4.20451 4.71683
2.42879 3.88223 0.68484 3.18550 4.37450 4.61168 4.20806 4.02805
3.80108 3.64584 2.51132 5.15144 3.91438 565 L - - -
2.68618 4.42225 2.77519 2.73123 3.46354 2.40513 3.72494
3.29354 2.67741 2.69355 4.24690 2.90347 2.73739 3.18146 2.89801
2.37887 2.77519 2.98518 4.58477 3.61503
0.02625 4.04896 4.77131 0.61958 0.77255 0.48576 0.95510
470 2.80764 3.57696 3.62263 3.09919 2.73425 3.70494 2.18645
3.03957 2.97201 2.66661 3.63220 3.42878 4.09559 3.30184 3.27839
2.98759 3.04730 2.82538 4.31860 1.90409 566 y - - -
2.68618 4.42225 2.77519 2.73123 3.46354 2.40513 3.72494
3.29354 2.67741 2.69355 4.24690 2.90347 2.73739 3.18146 2.89801
2.37887 2.77519 2.98518 4.58477 3.61503
0.02625 4.04896 4.77131 0.61958 0.77255 0.48576 0.95510
471 3.61838 4.86975 4.62763 4.30782 0.98233 4.38817 3.52227
3.26950 4.15416 2.59657 3.87243 4.09175 4.70104 4.13846 4.19432
3.76065 3.84358 3.20819 3.66766 1.56386 567 f - - -
2.68618 4.42225 2.77519 2.73123 3.46354 2.40513 3.72494
3.29354 2.67741 2.69355 4.24690 2.90347 2.73739 3.18146 2.89801
2.37887 2.77519 2.98518 4.58477 3.61503
0.02625 4.04896 4.77131 0.61958 0.77255 0.48576 0.95510
472 2.70970 4.94627 2.61669 2.38449 4.42826 2.07292 3.76257
3.88617 2.33113 3.44986 4.27757 1.90970 3.83924 2.92687 2.99390
2.71665 3.01171 3.48640 5.62620 4.26640 568 n - - -
2.68618 4.42225 2.77519 2.73123 3.46354 2.40513 3.72494
3.29354 2.67741 2.69355 4.24690 2.90347 2.73739 3.18146 2.89801
2.37887 2.77519 2.98518 4.58477 3.61503
0.02625 4.04896 4.77131 0.61958 0.77255 0.48576 0.95510
473 2.94188 5.31129 2.39419 1.13725 4.59265 3.32735 3.77776
4.08224 2.60477 3.60699 4.48999 2.83367 3.90372 2.67412 3.04410
2.87569 3.21531 3.71611 5.76602 4.36074 569 e - - -
2.68618 4.42225 2.77519 2.73123 3.46354 2.40513 3.72494
3.29354 2.67741 2.69355 4.24690 2.90347 2.73739 3.18146 2.89801
2.37887 2.77519 2.98518 4.58477 3.61503
0.02625 4.04896 4.77131 0.61958 0.77255 0.48576 0.95510
474 2.61443 4.62485 2.63470 2.55198 3.83989 3.44149 3.72440
2.35870 2.59545 2.86654 3.74347 2.33582 3.86592 2.92686 3.03098
2.70893 2.85998 2.64833 5.20366 3.89695 570 n - - -

```
          2.68618  4.42225  2.77519  2.73123  3.46354  2.40513  3.72494
3.29354  2.67741  2.69355  4.24690  2.90347  2.73739  3.18146  2.89801
2.37887  2.77519  2.98518  4.58477  3.61503
          0.02625  4.04896  4.77131  0.61958  0.77255  0.48576  0.95510
    475   3.21415  5.03434  3.72375  3.20215  4.43412  3.66389  3.96639
3.99095  2.32798  3.47493  4.47340  3.54687  4.16051  3.18049  0.73952
3.29160  3.46976  3.70799  5.44719  4.32628    571 r - - -
          2.68618  4.42225  2.77519  2.73123  3.46354  2.40513  3.72494
3.29354  2.67741  2.69355  4.24690  2.90347  2.73739  3.18146  2.89801
2.37887  2.77519  2.98518  4.58477  3.61503
          0.02625  4.04896  4.77131  0.61958  0.77255  0.48576  0.95510
    476   2.07507  4.96049  2.29619  2.31818  4.36947  3.28794  3.69565
3.82112  2.54503  3.38166  4.18315  2.24791  2.87865  2.84097  3.04149
2.64466  2.92867  3.42423  5.57869  4.19306    572 a - - -
          2.68618  4.42225  2.77519  2.73123  3.46354  2.40513  3.72494
3.29354  2.67741  2.69355  4.24690  2.90347  2.73739  3.18146  2.89801
2.37887  2.77519  2.98518  4.58477  3.61503
          0.02625  4.04896  4.77131  0.61958  0.77255  0.48576  0.95510
    477   3.20097  4.53052  4.76276  4.28154  3.38296  4.47224  4.93107
1.02376  4.09630  1.74382  3.24309  4.52040  4.76704  4.37864  4.27441
3.87569  3.46124  1.79858  5.38295  4.15604    573 i - - -
          2.68618  4.42225  2.77519  2.73123  3.46354  2.40513  3.72494
3.29354  2.67741  2.69355  4.24690  2.90347  2.73739  3.18146  2.89801
2.37887  2.77519  2.98518  4.58477  3.61503
          0.02625  4.04896  4.77131  0.61958  0.77255  0.48576  0.95510
    478   2.30479  4.64917  3.00606  2.40552  4.03173  2.85098  3.70996
3.43767  2.52430  3.06230  3.89017  3.00628  3.80589  2.88447  2.72737
2.46906  2.07353  3.10046  5.31836  4.00986    574 t - - -
          2.68618  4.42225  2.77519  2.73123  3.46354  2.40513  3.72494
3.29354  2.67741  2.69355  4.24690  2.90347  2.73739  3.18146  2.89801
2.37887  2.77519  2.98518  4.58477  3.61503
          0.02625  4.04896  4.77131  0.61958  0.77255  0.48576  0.95510
    479   2.49369  4.57829  2.56982  2.58182  4.27371  3.16886  3.88391
3.68414  2.78262  3.33071  4.16098  3.02334  3.78254  3.07619  3.22121
1.44132  2.41433  3.26026  5.56482  4.24515    575 s - - -
          2.68618  4.42225  2.77519  2.73123  3.46354  2.40513  3.72494
3.29354  2.67741  2.69355  4.24690  2.90347  2.73739  3.18146  2.89801
2.37887  2.77519  2.98518  4.58477  3.61503
          0.02625  4.04896  4.77131  0.61958  0.77255  0.48576  0.95510
    480   2.38918  4.31644  3.36062  3.03232  4.14290  3.11637  4.11625
3.44941  3.02446  3.18895  4.08721  3.29936  2.51827  3.37739  3.34804
2.55862  1.31796  3.05071  5.50815  4.27476    576 t - - -
          2.68618  4.42225  2.77519  2.73123  3.46354  2.40513  3.72494
3.29354  2.67741  2.69355  4.24690  2.90347  2.73739  3.18146  2.89801
2.37887  2.77519  2.98518  4.58477  3.61503
          0.02625  4.04896  4.77131  0.61958  0.77255  0.48576  0.95510
    481   2.48317  4.88270  2.90609  2.26895  4.14933  3.39208  3.06797
3.58201  2.36460  3.15329  3.94930  2.61477  3.48108  2.72966  2.65335
2.31906  2.82783  3.23218  5.36434  3.51167    577 e - - -
          2.68618  4.42225  2.77519  2.73123  3.46354  2.40513  3.72494
3.29354  2.67741  2.69355  4.24690  2.90347  2.73739  3.18146  2.89801
2.37887  2.77519  2.98518  4.58477  3.61503
          0.15293  4.04896  2.08450  0.61958  0.77255  0.48576  0.95510
```

```
    482   2.73164  4.50192  3.41633  3.32244  4.46401  0.65140  4.43957
4.07648  3.51098  3.72396  4.70006  3.58058  3.89477  3.83090  3.74128
2.90506  3.21701  3.59451  5.54519  4.56767    578 G - - -
          2.68602  4.42233  2.77528  2.73131  3.46362  2.40484  3.72503
3.29362  2.67749  2.69363  4.24698  2.90355  2.73722  3.18154  2.89809
2.37895  2.77501  2.98526  4.58485  3.61511
          0.15885  1.98563  4.64812  1.24729  0.33867  0.41800  1.07400
    483   2.72347  4.42343  3.70288  3.18562  3.63418  3.61518  4.03507
2.60369  2.82233  2.54303  3.59724  3.51532  4.08433  3.34499  2.40430
2.97856  3.00998  1.39639  5.14556  3.90085    584 v - - -
          2.68618  4.42225  2.77519  2.73123  3.46354  2.40513  3.72494
3.29354  2.67741  2.69355  4.24690  2.90347  2.73739  3.18146  2.89801
2.37887  2.77519  2.98518  4.58477  3.61503
          0.02625  4.04896  4.77131  0.61958  0.77255  0.48576  0.95510
    484   1.94394  4.88592  2.38029  2.35582  4.36951  3.25339  3.75151
3.81522  2.63058  3.39700  4.21125  1.99420  3.80347  2.91154  3.12095
2.47709  2.94339  3.41229  5.60423  4.22869    585 a - - -
          2.68618  4.42225  2.77519  2.73123  3.46354  2.40513  3.72494
3.29354  2.67741  2.69355  4.24690  2.90347  2.73739  3.18146  2.89801
2.37887  2.77519  2.98518  4.58477  3.61503
          0.02625  4.04896  4.77131  0.61958  0.77255  0.48576  0.95510
    485   2.64900  4.74411  3.05998  2.53753  3.97092  3.44679  3.65905
3.40127  1.79083  3.01942  3.86177  3.02552  3.85813  2.83527  2.75832
2.43238  2.68187  3.09856  5.24467  3.00056    586 k - - -
          2.68618  4.42225  2.77519  2.73123  3.46354  2.40513  3.72494
3.29354  2.67741  2.69355  4.24690  2.90347  2.73739  3.18146  2.89801
2.37887  2.77519  2.98518  4.58477  3.61503
          0.02625  4.04896  4.77131  0.61958  0.77255  0.48576  0.95510
    486   3.11411  4.47485  4.66475  4.11659  3.29234  4.36686  4.75499
1.98343  3.97947  1.37085  2.84453  4.37054  4.63977  4.19398  4.16032
3.71062  3.35619  1.35658  5.24626  4.10270    587 v - - -
          2.68618  4.42225  2.77519  2.73123  3.46354  2.40513  3.72494
3.29354  2.67741  2.69355  4.24690  2.90347  2.73739  3.18146  2.89801
2.37887  2.77519  2.98518  4.58477  3.61503
          0.02625  4.04896  4.77131  0.61958  0.77255  0.48576  0.95510
    487   3.12588  4.43216  4.85865  4.36643  3.59553  4.48744  5.07734
1.22651  4.25526  2.09015  3.39660  4.57882  4.79627  4.51733  4.44913
3.88025  3.39272  1.19624  5.57485  4.37049    588 v - - -
          2.68618  4.42225  2.77519  2.73123  3.46354  2.40513  3.72494
3.29354  2.67741  2.69355  4.24690  2.90347  2.73739  3.18146  2.89801
2.37887  2.77519  2.98518  4.58477  3.61503
          0.02625  4.04896  4.77131  0.61958  0.77255  0.48576  0.95510
    488   1.50180  4.19161  3.47694  3.12087  4.27977  2.54104  4.18372
3.68604  3.15512  3.36810  4.18597  3.30089  3.70794  3.44062  3.49115
1.65084  2.43390  3.15738  5.61793  4.40226    589 a - - -
          2.68618  4.42225  2.77519  2.73123  3.46354  2.40513  3.72494
3.29354  2.67741  2.69355  4.24690  2.90347  2.73739  3.18146  2.89801
2.37887  2.77519  2.98518  4.58477  3.61503
          0.02625  4.04896  4.77131  0.61958  0.77255  0.48576  0.95510
    489   3.08459  4.42790  4.74284  4.26070  3.59952  4.38010  4.99399
1.57795  4.13826  2.12363  3.42085  4.47781  4.72996  4.42414  4.34625
3.77623  3.36234  0.97176  5.54913  4.32587    590 v - - -
          2.68618  4.42225  2.77519  2.73123  3.46354  2.40513  3.72494
3.29354  2.67741  2.69355  4.24690  2.90347  2.73739  3.18146  2.89801
2.37887  2.77519  2.98518  4.58477  3.61503
```

0.02625 4.04896 4.77131 0.61958 0.77255 0.48576 0.95510
490 3.25066 4.88181 3.72521 3.33407 2.28379 3.96154 1.88213
3.50542 3.16665 2.93850 4.03959 3.58244 4.35018 3.51034 3.42683
3.31399 3.48234 3.31950 3.92320 1.37895 591 y - - -
2.68618 4.42225 2.77519 2.73123 3.46354 2.40513 3.72494
3.29354 2.67741 2.69355 4.24690 2.90347 2.73739 3.18146 2.89801
2.37887 2.77519 2.98518 4.58477 3.61503
0.02625 4.04896 4.77131 0.61958 0.77255 0.48576 0.95510
491 2.87931 4.87574 2.82111 2.72621 4.26801 3.31004 4.07758
4.02015 2.99819 3.64783 4.60465 0.87973 3.97208 3.35350 3.35640
2.94155 3.26875 3.62429 5.55383 4.20588 592 n - - -
2.68618 4.42225 2.77519 2.73123 3.46354 2.40513 3.72494
3.29354 2.67741 2.69355 4.24690 2.90347 2.73739 3.18146 2.89801
2.37887 2.77519 2.98518 4.58477 3.61503
0.02625 4.04896 4.77131 0.61958 0.77255 0.48576 0.95510
492 3.21415 5.03434 3.72375 3.20215 4.43412 3.66389 3.96639
3.99095 2.32798 3.47493 4.47340 3.54687 4.16051 3.18049 0.73952
3.29160 3.46976 3.70799 5.44719 4.32628 593 r - - -
2.68618 4.42225 2.77519 2.73123 3.46354 2.40513 3.72494
3.29354 2.67741 2.69355 4.24690 2.90347 2.73739 3.18146 2.89801
2.37887 2.77519 2.98518 4.58477 3.61503
0.02625 4.04896 4.77131 0.61958 0.77255 0.48576 0.95510
493 3.14130 5.10446 3.32852 2.92133 4.53144 3.60779 3.86884
3.97270 0.81982 3.50099 4.45624 3.33675 4.09839 3.05270 2.48089
3.17368 3.38308 3.67174 5.51602 4.35580 594 k - - -
2.68618 4.42225 2.77519 2.73123 3.46354 2.40513 3.72494
3.29354 2.67741 2.69355 4.24690 2.90347 2.73739 3.18146 2.89801
2.37887 2.77519 2.98518 4.58477 3.61503
0.02625 4.04896 4.77131 0.61958 0.77255 0.48576 0.95510
494 1.94163 4.48724 3.04103 2.67414 4.21767 3.15991 3.88895
3.62211 2.75683 3.26736 4.08993 1.95829 3.76711 3.08208 3.17134
2.29970 2.36719 3.19533 5.50857 4.21560 595 a - - -
2.68618 4.42225 2.77519 2.73123 3.46354 2.40513 3.72494
3.29354 2.67741 2.69355 4.24690 2.90347 2.73739 3.18146 2.89801
2.37887 2.77519 2.98518 4.58477 3.61503
0.02625 4.04896 4.77131 0.61958 0.77255 0.48576 0.95510
495 2.87931 4.87574 2.82111 2.72621 4.26801 3.31004 4.07758
4.02015 2.99819 3.64783 4.60465 0.87973 3.97208 3.35350 3.35640
2.94155 3.26875 3.62429 5.55383 4.20588 596 n - - -
2.68618 4.42225 2.77519 2.73123 3.46354 2.40513 3.72494
3.29354 2.67741 2.69355 4.24690 2.90347 2.73739 3.18146 2.89801
2.37887 2.77519 2.98518 4.58477 3.61503
0.02625 4.04896 4.77131 0.61958 0.77255 0.48576 0.95510
496 2.95975 4.39859 4.25934 3.75949 3.01109 4.04942 4.21580
1.27649 3.59053 2.06372 3.28574 3.98294 4.40159 3.86847 3.78623
3.39141 3.20585 2.13445 4.73037 2.65579 597 i - - -
2.68618 4.42225 2.77519 2.73123 3.46354 2.40513 3.72494
3.29354 2.67741 2.69355 4.24690 2.90347 2.73739 3.18146 2.89801
2.37887 2.77519 2.98518 4.58477 3.61503
0.02625 4.04896 4.77131 0.61958 0.77255 0.48576 0.95510
497 2.10657 4.72183 2.59603 2.47488 3.81241 3.41979 3.09887
3.34811 2.52558 2.97520 3.82078 2.98252 3.84049 2.86070 2.97247
2.68127 2.86385 3.05154 5.16450 2.75517 598 a - - -

```
         2.68618  4.42225  2.77519  2.73123  3.46354  2.40513  3.72494
3.29354  2.67741  2.69355  4.24690  2.90347  2.73739  3.18146  2.89801
2.37887  2.77519  2.98518  4.58477  3.61503
         0.02625  4.04896  4.77131  0.61958  0.77255  0.48576  0.95510
    498  1.85650  4.76072  2.10459  2.43087  4.40337  2.11687  3.83747
3.83519  2.75713  3.44375  4.26543  2.92527  3.79471  3.01283  3.24290
2.62770  2.93384  3.40276  5.65832  4.30039    599 a - - -
         2.68619  4.42226  2.77521  2.73124  3.46315  2.40514  3.72496
3.29355  2.67742  2.69356  4.24691  2.90348  2.73741  3.18147  2.89802
2.37888  2.77521  2.98519  4.58478  3.61504
         0.10917  2.35442  4.77131  0.39046  1.12931  0.48576  0.95510
    499  2.08888  4.22105  3.72738  3.27768  3.82788  3.28113  4.20124
2.71386  3.20997  2.76153  3.74427  3.49152  3.90866  3.52315  3.51079
2.67725  1.62488  2.00121  5.33084  4.11567    601 t - - -
         2.68618  4.42225  2.77519  2.73123  3.46354  2.40513  3.72494
3.29354  2.67741  2.69355  4.24690  2.90347  2.73739  3.18146  2.89801
2.37887  2.77519  2.98518  4.58477  3.61503
         0.02625  4.04896  4.77131  0.61958  0.77255  0.48576  0.95510
    500  2.72277  4.95482  2.69439  2.39888  4.14866  3.34455  2.35008
3.77604  2.49803  3.33593  4.17089  1.93009  3.85247  2.88178  2.91824
2.47358  2.99447  3.41183  5.42265  4.01267    602 n - - -
         2.68618  4.42225  2.77519  2.73123  3.46354  2.40513  3.72494
3.29354  2.67741  2.69355  4.24690  2.90347  2.73739  3.18146  2.89801
2.37887  2.77519  2.98518  4.58477  3.61503
         0.02625  4.04896  4.77131  0.61958  0.77255  0.48576  0.95510
    501  2.69291  4.92419  2.83447  1.85576  4.16719  3.41455  3.65133
2.95644  2.39190  3.14927  3.99722  2.93387  3.84631  2.16453  2.80984
2.70464  2.92754  3.24006  5.40074  4.06027    603 e - - -
         2.68618  4.42225  2.77519  2.73123  3.46354  2.40513  3.72494
3.29354  2.67741  2.69355  4.24690  2.90347  2.73739  3.18146  2.89801
2.37887  2.77519  2.98518  4.58477  3.61503
         0.02625  4.04896  4.77131  0.61958  0.77255  0.48576  0.95510
    502  2.79937  4.95422  2.85864  2.54764  4.31964  3.38875  3.75503
3.85313  2.38908  3.40543  4.26592  1.45165  3.91051  2.93015  2.27796
2.81983  3.08031  3.48812  5.49385  4.16414    604 n - - -
         2.68618  4.42225  2.77519  2.73123  3.46354  2.40513  3.72494
3.29354  2.67741  2.69355  4.24690  2.90347  2.73739  3.18146  2.89801
2.37887  2.77519  2.98518  4.58477  3.61503
         0.02625  4.04896  4.77131  0.61958  0.77255  0.48576  0.95510
    503  2.89902  4.65351  3.60866  3.53220  4.66473  0.50220  4.63853
4.31037  3.73728  3.94135  4.92090  3.77344  4.05011  4.04773  3.95090
3.07653  3.39400  3.80462  5.71003  4.77372    605 G - - -
         2.68618  4.42225  2.77519  2.73123  3.46354  2.40513  3.72494
3.29354  2.67741  2.69355  4.24690  2.90347  2.73739  3.18146  2.89801
2.37887  2.77519  2.98518  4.58477  3.61503
         0.02625  4.04896  4.77131  0.61958  0.77255  0.48576  0.95510
    504  2.30281  4.22851  3.43905  3.13663  4.19556  3.03283  4.19203
3.52076  3.13639  3.28190  4.15269  3.32797  3.75588  3.46968  3.44003
1.93924  1.28880  3.06783  5.56871  4.33634    606 t - - -
         2.68618  4.42225  2.77519  2.73123  3.46354  2.40513  3.72494
3.29354  2.67741  2.69355  4.24690  2.90347  2.73739  3.18146  2.89801
2.37887  2.77519  2.98518  4.58477  3.61503
         0.02625  4.04896  4.77131  0.61958  0.77255  0.48576  0.95510
```

```
    505   3.18886  4.54714  4.71189  4.15051  3.16200  4.42211  4.74425
1.72227  3.99846  1.49881  1.66296  4.41266  4.65483  4.17626  4.15494
3.75991  3.41808  2.13118  5.16596  4.05899    607 l - - -
         2.68618  4.42225  2.77519  2.73123  3.46354  2.40513  3.72494
3.29354  2.67741  2.69355  4.24690  2.90347  2.73739  3.18146  2.89801
2.37887  2.77519  2.98518  4.58477  3.61503
         0.02625  4.04896  4.77131  0.61958  0.77255  0.48576  0.95510
    506   3.13301  4.45830  4.81284  4.33138  3.54434  4.44896  5.04103
1.03963  4.20231  2.02626  3.35642  4.54726  4.77301  4.47127  4.39439
3.84902  3.40523  1.48342  5.53667  4.33163    608 i - - -
         2.68618  4.42225  2.77519  2.73123  3.46354  2.40513  3.72494
3.29354  2.67741  2.69355  4.24690  2.90347  2.73739  3.18146  2.89801
2.37887  2.77519  2.98518  4.58477  3.61503
         0.02625  4.04896  4.77131  0.61958  0.77255  0.48576  0.95510
    507   3.20631  4.98652  3.31515  3.09613  3.42193  3.60842  0.81890
3.93661  2.93499  3.40685  4.46625  3.49698  4.17233  3.46562  3.18840
3.27638  3.51707  3.66854  4.88390  3.36851    609 h - - -
         2.68618  4.42225  2.77519  2.73123  3.46354  2.40513  3.72494
3.29354  2.67741  2.69355  4.24690  2.90347  2.73739  3.18146  2.89801
2.37887  2.77519  2.98518  4.58477  3.61503
         0.02625  4.04896  4.77131  0.61958  0.77255  0.48576  0.95510
    508   2.63973  4.54150  3.20209  2.40647  3.24685  3.52016  3.73945
3.01662  2.61214  2.00234  3.57596  3.14609  3.91110  2.44750  2.99707
2.77150  2.87074  2.79692  5.04533  3.75502    610 l - - -
         2.68618  4.42225  2.77519  2.73123  3.46354  2.40513  3.72494
3.29354  2.67741  2.69355  4.24690  2.90347  2.73739  3.18146  2.89801
2.37887  2.77519  2.98518  4.58477  3.61503
         0.02625  4.04896  4.77131  0.61958  0.77255  0.48576  0.95510
    509   1.18955  4.29722  3.66323  3.25244  3.79267  3.30075  4.19648
2.82317  3.15097  2.67180  3.19207  3.48657  3.93131  3.50796  3.44225
2.71403  2.88619  2.58829  5.33710  4.09630    611 a - - -
         2.68618  4.42225  2.77519  2.73123  3.46354  2.40513  3.72494
3.29354  2.67741  2.69355  4.24690  2.90347  2.73739  3.18146  2.89801
2.37887  2.77519  2.98518  4.58477  3.61503
         0.02625  4.04896  4.77131  0.61958  0.77255  0.48576  0.95510
    510   3.00224  4.72657  3.62906  3.49603  4.47053  3.40426  4.55621
4.06484  3.56912  3.67110  4.73068  3.78339  0.55633  3.95247  3.79183
3.17599  3.45834  3.68948  5.60532  4.59466    612 P - - -
         2.68618  4.42225  2.77519  2.73123  3.46354  2.40513  3.72494
3.29354  2.67741  2.69355  4.24690  2.90347  2.73739  3.18146  2.89801
2.37887  2.77519  2.98518  4.58477  3.61503
         0.02625  4.04896  4.77131  0.61958  0.77255  0.48576  0.95510
    511   2.67199  5.00957  2.70487  2.11055  4.31965  3.35502  3.63733
3.76215  2.30590  3.31232  4.11096  2.14154  3.30708  2.77310  2.88072
2.65750  2.41600  3.39038  5.50333  4.13072    613 e - - -
         2.68618  4.42225  2.77519  2.73123  3.46354  2.40513  3.72494
3.29354  2.67741  2.69355  4.24690  2.90347  2.73739  3.18146  2.89801
2.37887  2.77519  2.98518  4.58477  3.61503
         0.02625  4.04896  4.77131  0.61958  0.77255  0.48576  0.95510
    512   2.96935  5.51116  1.17307  1.93555  4.83336  2.79337  3.80810
4.35101  2.84542  3.85781  4.71371  2.71452  3.87091  2.97904  3.44898
2.85793  3.26714  3.92040  6.01833  4.51514    614 d - - -
         2.68618  4.42225  2.77519  2.73123  3.46354  2.40513  3.72494
3.29354  2.67741  2.69355  4.24690  2.90347  2.73739  3.18146  2.89801
2.37887  2.77519  2.98518  4.58477  3.61503
```

0.02625 4.04896 4.77131 0.61958 0.77255 0.48576 0.95510
513 2.55680 4.45056 3.18374 2.78816 3.54107 2.25202 3.82486
3.22642 2.81436 2.89491 3.78283 3.18748 3.87562 3.14468 3.18481
2.32839 2.87252 2.92986 4.99659 2.17632 615 y - - -
2.68618 4.42225 2.77519 2.73123 3.46354 2.40513 3.72494
3.29354 2.67741 2.69355 4.24690 2.90347 2.73739 3.18146 2.89801
2.37887 2.77519 2.98518 4.58477 3.61503
0.02625 4.04896 4.77131 0.61958 0.77255 0.48576 0.95510
514 2.51696 4.36119 3.59478 3.35937 4.13457 3.19354 4.36194
3.28365 3.30583 3.14078 4.18273 3.53737 3.91252 3.69255 3.55365
2.72057 0.90433 2.95928 5.55663 4.35230 616 t - - -
2.68618 4.42225 2.77519 2.73123 3.46354 2.40513 3.72494
3.29354 2.67741 2.69355 4.24690 2.90347 2.73739 3.18146 2.89801
2.37887 2.77519 2.98518 4.58477 3.61503
0.02625 4.04896 4.77131 0.61958 0.77255 0.48576 0.95510
515 2.89902 4.65351 3.60866 3.53220 4.66473 0.50220 4.63853
4.31037 3.73728 3.94135 4.92090 3.77344 4.05011 4.04773 3.95090
3.07653 3.39400 3.80462 5.71003 4.77372 617 G - - -
2.68618 4.42225 2.77519 2.73123 3.46354 2.40513 3.72494
3.29354 2.67741 2.69355 4.24690 2.90347 2.73739 3.18146 2.89801
2.37887 2.77519 2.98518 4.58477 3.61503
0.02625 4.04896 4.77131 0.61958 0.77255 0.48576 0.95510
516 3.49106 4.84211 4.48107 4.23211 0.76177 4.13322 3.96743
2.99649 4.13553 2.32725 3.70244 4.22219 4.59014 4.25557 4.21149
3.74980 3.79345 2.99963 4.16985 2.51707 618 f - - -
2.68618 4.42225 2.77519 2.73123 3.46354 2.40513 3.72494
3.29354 2.67741 2.69355 4.24690 2.90347 2.73739 3.18146 2.89801
2.37887 2.77519 2.98518 4.58477 3.61503
0.02625 4.04896 4.77131 0.61958 0.77255 0.48576 0.95510
517 2.51696 4.36119 3.59478 3.35937 4.13457 3.19354 4.36194
3.28365 3.30583 3.14078 4.18273 3.53737 3.91252 3.69255 3.55365
2.72057 0.90433 2.95928 5.55663 4.35230 619 t - - -
2.68618 4.42225 2.77519 2.73123 3.46354 2.40513 3.72494
3.29354 2.67741 2.69355 4.24690 2.90347 2.73739 3.18146 2.89801
2.37887 2.77519 2.98518 4.58477 3.61503
0.02625 4.04896 4.77131 0.61958 0.77255 0.48576 0.95510
518 3.12918 4.43667 4.85750 4.36618 3.58665 4.48687 5.07597
1.16599 4.25300 2.07697 3.38809 4.57904 4.79586 4.51443 4.44588
3.88052 3.39630 1.26588 5.57007 4.36719 620 i - - -
2.68618 4.42225 2.77519 2.73123 3.46354 2.40513 3.72494
3.29354 2.67741 2.69355 4.24690 2.90347 2.73739 3.18146 2.89801
2.37887 2.77519 2.98518 4.58477 3.61503
0.02625 4.04896 4.77131 0.61958 0.77255 0.48576 0.95510
519 2.41035 4.33126 3.36913 3.19663 4.25684 3.05832 4.29982
3.80508 3.28244 3.51284 4.42630 3.39050 3.81243 3.61846 3.56056
0.85292 2.90434 3.30106 5.61579 4.34228 621 s - - -
2.68618 4.42225 2.77519 2.73123 3.46354 2.40513 3.72494
3.29354 2.67741 2.69355 4.24690 2.90347 2.73739 3.18146 2.89801
2.37887 2.77519 2.98518 4.58477 3.61503
0.02625 4.04896 4.77131 0.61958 0.77255 0.48576 0.95510
520 3.00224 4.72657 3.62906 3.49603 4.47053 3.40426 4.55621
4.06484 3.56912 3.67110 4.73068 3.78339 0.55633 3.95247 3.79183
3.17599 3.45834 3.68948 5.60532 4.59466 622 P - - -

2.68618 4.42225 2.77519 2.73123 3.46354 2.40513 3.72494
3.29354 2.67741 2.69355 4.24690 2.90347 2.73739 3.18146 2.89801
2.37887 2.77519 2.98518 4.58477 3.61503
0.02625 4.04896 4.77131 0.61958 0.77255 0.48576 0.95510
521 3.27464 4.59046 4.86492 4.33899 3.22250 4.55949 4.92665
1.42608 4.17244 1.14571 3.04017 4.58774 4.78204 4.35995 4.31680
3.93471 3.51311 1.98650 5.28117 4.14101 623 l - - -
2.68618 4.42225 2.77519 2.73123 3.46354 2.40513 3.72494
3.29354 2.67741 2.69355 4.24690 2.90347 2.73739 3.18146 2.89801
2.37887 2.77519 2.98518 4.58477 3.61503
0.02625 4.04896 4.77131 0.61958 0.77255 0.48576 0.95510
522 3.07609 4.88560 3.38192 3.01103 2.66629 3.74826 1.36454
3.53252 2.82132 3.00589 4.05008 3.37760 4.18301 3.27339 3.11053
3.13236 3.31943 3.31294 4.26531 2.33551 624 h - - -
2.68618 4.42225 2.77519 2.73123 3.46354 2.40513 3.72494
3.29354 2.67741 2.69355 4.24690 2.90347 2.73739 3.18146 2.89801
2.37887 2.77519 2.98518 4.58477 3.61503
0.02625 4.04896 4.77131 0.61958 0.77255 0.48576 0.95510
523 1.79690 4.32993 3.45547 2.98004 3.67243 3.37458 3.96345
2.89425 2.91831 2.02277 3.61695 3.32754 2.84829 3.25863 3.25637
2.71915 2.84196 2.65360 5.14275 3.90109 625 a - - -
2.68618 4.42225 2.77519 2.73123 3.46354 2.40513 3.72494
3.29354 2.67741 2.69355 4.24690 2.90347 2.73739 3.18146 2.89801
2.37887 2.77519 2.98518 4.58477 3.61503
0.02625 4.04896 4.77131 0.61958 0.77255 0.48576 0.95510
524 2.32877 4.61608 2.43597 2.53971 4.19361 3.22267 3.82128
3.57923 2.70545 3.23294 4.06380 3.00302 3.79098 3.00588 3.15428
2.37009 1.71698 3.19407 5.48692 4.16769 626 t - - -
2.68618 4.42225 2.77519 2.73123 3.46354 2.40513 3.72494
3.29354 2.67741 2.69355 4.24690 2.90347 2.73739 3.18146 2.89801
2.37887 2.77519 2.98518 4.58477 3.61503
0.02625 4.04896 4.77131 0.61958 0.77255 0.48576 0.95510
525 2.43727 4.68523 3.03406 2.49848 3.92666 3.41905 3.36115
3.02534 2.45908 2.95753 3.79236 2.99766 3.82469 2.24363 2.88866
2.39548 2.53910 3.02524 5.22555 3.91669 627 q - - -
2.68618 4.42225 2.77519 2.73123 3.46354 2.40513 3.72494
3.29354 2.67741 2.69355 4.24690 2.90347 2.73739 3.18146 2.89801
2.37887 2.77519 2.98518 4.58477 3.61503
0.02625 4.04896 4.77131 0.61958 0.77255 0.48576 0.95510
526 2.45113 4.21014 3.78703 3.26296 2.76710 3.62752 4.02611
2.44718 3.18838 2.38157 3.36601 3.11250 4.05726 3.46991 3.46408
2.93811 2.88349 1.56814 4.87495 3.61698 628 v - - -
2.68618 4.42225 2.77519 2.73123 3.46354 2.40513 3.72494
3.29354 2.67741 2.69355 4.24690 2.90347 2.73739 3.18146 2.89801
2.37887 2.77519 2.98518 4.58477 3.61503
0.02625 4.04896 4.77131 0.61958 0.77255 0.48576 0.95510
527 2.50037 4.57862 2.89918 2.60877 4.27947 3.17625 3.88229
3.70279 2.73729 3.34182 4.17261 1.70190 3.78954 3.07464 3.14705
2.32740 2.16363 3.27525 5.55848 4.24346 629 n - - -
2.68618 4.42225 2.77519 2.73123 3.46354 2.40513 3.72494
3.29354 2.67741 2.69355 4.24690 2.90347 2.73739 3.18146 2.89801
2.37887 2.77519 2.98518 4.58477 3.61503
0.02625 4.04896 4.77131 0.61958 0.77255 0.48576 0.95510

528 2.64304 4.70934 3.06705 2.54145 3.88345 3.46029 3.03189
2.64954 2.41674 2.95530 3.80412 2.21471 3.86073 2.84620 2.59868
2.70992 2.87334 3.03725 5.18915 3.87274 630 n - - -
2.68618 4.42225 2.77519 2.73123 3.46354 2.40513 3.72494
3.29354 2.67741 2.69355 4.24690 2.90347 2.73739 3.18146 2.89801
2.37887 2.77519 2.98518 4.58477 3.61503
0.02625 4.04896 4.77131 0.61958 0.77255 0.48576 0.95510
529 2.65093 4.91541 2.79254 2.39231 4.28384 3.34896 3.66377
3.72094 2.41697 3.28345 4.09181 2.53500 2.74950 2.03338 2.84867
2.65928 2.66183 3.35027 5.47724 4.12692 631 q - - -
2.68618 4.42225 2.77519 2.73123 3.46354 2.40513 3.72494
3.29354 2.67741 2.69355 4.24690 2.90347 2.73739 3.18146 2.89801
2.37887 2.77519 2.98518 4.58477 3.61503
0.02625 4.04896 4.77131 0.61958 0.77255 0.48576 0.95510
530 2.45897 4.40893 3.24061 2.76826 3.90042 3.26681 3.85851
2.94094 2.74533 2.95903 3.81803 2.89406 3.81160 3.08959 3.13119
1.88827 2.00429 2.92435 5.26231 3.99373 632 s - - -
2.68618 4.42225 2.77519 2.73123 3.46354 2.40513 3.72494
3.29354 2.67741 2.69355 4.24690 2.90347 2.73739 3.18146 2.89801
2.37887 2.77519 2.98518 4.58477 3.61503
0.07670 4.04896 2.87536 0.61958 0.77255 0.48576 0.95510
531 2.64664 4.72379 3.09063 2.28444 3.91219 3.46448 3.63672
3.33510 2.35098 2.95636 3.80776 3.02652 3.85548 2.81594 2.05607
2.70872 2.87158 2.78717 5.19555 3.16090 633 r - - -
2.68618 4.42225 2.77519 2.73123 3.46354 2.40513 3.72494
3.29354 2.67741 2.69355 4.24690 2.90347 2.73739 3.18146 2.89801
2.37887 2.77519 2.98518 4.58477 3.61503
0.02759 3.99985 4.72219 0.61958 0.77255 0.53666 0.87875
532 2.21620 4.27024 3.65941 3.14121 3.59181 3.48321 4.05590
2.52118 3.05987 2.18975 3.51191 3.46205 3.98437 3.38286 3.38056
2.81590 1.95625 2.13755 5.12139 3.89691 634 t - - -
2.68618 4.42225 2.77519 2.73123 3.46354 2.40513 3.72494
3.29354 2.67741 2.69355 4.24690 2.90347 2.73739 3.18146 2.89801
2.37887 2.77519 2.98518 4.58477 3.61503
0.02759 3.99985 4.72219 0.61958 0.77255 0.45520 1.00599
533 3.44460 4.81757 4.32116 3.91494 1.33440 4.23508 2.21970
3.38551 3.79197 2.80266 3.95730 3.89127 4.56025 3.89127 3.94505
3.55966 3.66400 3.24168 3.68112 1.53647 635 f - - -
2.68618 4.42225 2.77519 2.73123 3.46354 2.40513 3.72494
3.29354 2.67741 2.69355 4.24690 2.90347 2.73739 3.18146 2.89801
2.37887 2.77519 2.98518 4.58477 3.61503
0.02625 4.04896 4.77131 0.61958 0.77255 0.48576 0.95510
534 3.17037 4.54472 4.57938 4.20466 3.49871 4.25159 4.90309
0.88271 4.03620 2.05240 3.41046 4.42573 4.66901 4.36848 4.21304
3.76696 3.46771 1.86689 5.42361 4.17486 636 i - - -
2.68618 4.42225 2.77519 2.73123 3.46354 2.40513 3.72494
3.29354 2.67741 2.69355 4.24690 2.90347 2.73739 3.18146 2.89801
2.37887 2.77519 2.98518 4.58477 3.61503
0.02625 4.04896 4.77131 0.61958 0.77255 0.48576 0.95510
535 2.72922 4.98565 2.96874 2.46962 4.32347 3.44871 3.62278
3.74086 2.09491 3.27589 4.08959 2.97229 3.86391 2.17371 2.39026
2.00252 2.95549 3.38840 5.43754 4.12752 637 s - - -
2.68618 4.42225 2.77519 2.73123 3.46354 2.40513 3.72494
3.29354 2.67741 2.69355 4.24690 2.90347 2.73739 3.18146 2.89801
2.37887 2.77519 2.98518 4.58477 3.61503

0.02625 4.04896 4.77131 0.61958 0.77255 0.48576 0.95510
536 3.11468 5.25160 2.57626 0.79811 4.62159 3.39551 4.00220
4.11115 2.90580 3.70141 4.67133 3.04724 4.00959 3.21651 3.33029
3.08124 3.42146 3.77812 5.75983 4.48656 638 e - - -
2.68618 4.42225 2.77519 2.73123 3.46354 2.40513 3.72494
3.29354 2.67741 2.69355 4.24690 2.90347 2.73739 3.18146 2.89801
2.37887 2.77519 2.98518 4.58477 3.61503
0.02625 4.04896 4.77131 0.61958 0.77255 0.48576 0.95510
537 2.85573 5.11521 2.77448 2.46063 4.49057 3.41992 3.69702
3.94870 1.63479 3.46783 4.30953 1.82850 3.91171 2.84726 2.61695
2.83724 3.10369 3.58039 5.57152 4.24984 639 k - - -
2.68618 4.42225 2.77519 2.73123 3.46354 2.40513 3.72494
3.29354 2.67741 2.69355 4.24690 2.90347 2.73739 3.18146 2.89801
2.37887 2.77519 2.98518 4.58477 3.61503
0.02625 4.04896 4.77131 0.61958 0.77255 0.48576 0.95510
538 3.68119 4.90978 4.65209 4.34650 1.34179 4.41855 3.48409
3.36805 4.19175 2.69295 3.96325 4.09307 4.72668 4.15369 4.22148
3.78978 3.90284 3.29352 3.62121 1.07299 640 y - - -
2.68618 4.42225 2.77519 2.73123 3.46354 2.40513 3.72494
3.29354 2.67741 2.69355 4.24690 2.90347 2.73739 3.18146 2.89801
2.37887 2.77519 2.98518 4.58477 3.61503
0.02625 4.04896 4.77131 0.61958 0.77255 0.48576 0.95510
539 2.89902 4.65351 3.60866 3.53220 4.66473 0.50220 4.63853
4.31037 3.73728 3.94135 4.92090 3.77344 4.05011 4.04773 3.95090
3.07653 3.39400 3.80462 5.71003 4.77372 641 G - - -
2.68618 4.42225 2.77519 2.73123 3.46354 2.40513 3.72494
3.29354 2.67741 2.69355 4.24690 2.90347 2.73739 3.18146 2.89801
2.37887 2.77519 2.98518 4.58477 3.61503
0.02625 4.04896 4.77131 0.61958 0.77255 0.48576 0.95510
540 2.87931 4.87574 2.82111 2.72621 4.26801 3.31004 4.07758
4.02015 2.99819 3.64783 4.60465 0.87973 3.97208 3.35350 3.35640
2.94155 3.26875 3.62429 5.55383 4.20588 642 n - - -
2.68618 4.42225 2.77519 2.73123 3.46354 2.40513 3.72494
3.29354 2.67741 2.69355 4.24690 2.90347 2.73739 3.18146 2.89801
2.37887 2.77519 2.98518 4.58477 3.61503
0.02625 4.04896 4.77131 0.61958 0.77255 0.48576 0.95510
541 2.68322 4.93641 2.71660 2.39495 4.34186 3.32188 3.70499
3.80872 2.46579 3.36521 4.18241 2.48837 3.83044 1.97646 2.88676
2.01084 2.96119 3.42372 5.53630 4.17710 643 q - - -
2.68618 4.42225 2.77519 2.73123 3.46354 2.40513 3.72494
3.29354 2.67741 2.69355 4.24690 2.90347 2.73739 3.18146 2.89801
2.37887 2.77519 2.98518 4.58477 3.61503
0.02625 4.04896 4.77131 0.61958 0.77255 0.48576 0.95510
542 2.89902 4.65351 3.60866 3.53220 4.66473 0.50220 4.63853
4.31037 3.73728 3.94135 4.92090 3.77344 4.05011 4.04773 3.95090
3.07653 3.39400 3.80462 5.71003 4.77372 644 G - - -
2.68618 4.42225 2.77519 2.73123 3.46354 2.40513 3.72494
3.29354 2.67741 2.69355 4.24690 2.90347 2.73739 3.18146 2.89801
2.37887 2.77519 2.98518 4.58477 3.61503
0.02625 4.04896 4.77131 0.61958 0.77255 0.48576 0.95510
543 3.18840 5.33296 0.69448 2.46210 4.73586 3.35266 4.09910
4.36616 3.21856 3.94434 4.92964 3.02608 4.01823 3.34089 3.75621
3.13313 3.53189 3.98763 5.86922 4.58391 645 d - - -

2.68618 4.42225 2.77519 2.73123 3.46354 2.40513 3.72494
3.29354 2.67741 2.69355 4.24690 2.90347 2.73739 3.18146 2.89801
2.37887 2.77519 2.98518 4.58477 3.61503
0.02625 4.04896 4.77131 0.61958 0.77255 0.48576 0.95510
544 2.41035 4.33126 3.36913 3.19663 4.25684 3.05832 4.29982
3.80508 3.28244 3.51284 4.42630 3.39050 3.81243 3.61846 3.56056
0.85292 2.90434 3.30106 5.61579 4.34228 646 s - - -
2.68618 4.42225 2.77519 2.73123 3.46354 2.40513 3.72494
3.29354 2.67741 2.69355 4.24690 2.90347 2.73739 3.18146 2.89801
2.37887 2.77519 2.98518 4.58477 3.61503
0.02625 4.04896 4.77131 0.61958 0.77255 0.48576 0.95510
545 3.35841 4.77029 4.47216 4.11619 3.21164 4.20451 4.71683
2.42879 3.88223 0.68484 3.18550 4.37450 4.61168 4.20806 4.02805
3.80108 3.64584 2.51132 5.15144 3.91438 647 L - - -
2.68618 4.42225 2.77519 2.73123 3.46354 2.40513 3.72494
3.29354 2.67741 2.69355 4.24690 2.90347 2.73739 3.18146 2.89801
2.37887 2.77519 2.98518 4.58477 3.61503
0.02625 4.04896 4.77131 0.61958 0.77255 0.48576 0.95510
546 3.03745 5.07659 3.43394 2.84270 4.23505 3.65530 3.02558
3.87292 2.02866 3.34652 4.24260 3.24292 4.05662 2.84998 1.16099
3.05880 3.23098 3.57036 5.31599 4.03842 648 r - - -
2.68618 4.42225 2.77519 2.73123 3.46354 2.40513 3.72494
3.29354 2.67741 2.69355 4.24690 2.90347 2.73739 3.18146 2.89801
2.37887 2.77519 2.98518 4.58477 3.61503
0.02625 4.04896 4.77131 0.61958 0.77255 0.48576 0.95510
547 3.08269 4.42875 4.72175 4.15693 1.96926 4.31589 4.58195
1.84395 4.03254 1.48506 3.00966 4.34341 4.57313 4.16051 4.15137
3.64523 3.31137 1.86157 4.97437 3.76239 649 l - - -
2.68618 4.42225 2.77519 2.73123 3.46354 2.40513 3.72494
3.29354 2.67741 2.69355 4.24690 2.90347 2.73739 3.18146 2.89801
2.37887 2.77519 2.98518 4.58477 3.61503
0.02625 4.04896 4.77131 0.61958 0.77255 0.48576 0.95510
548 2.60464 4.69954 3.02062 1.98158 3.91347 3.43702 3.65193
2.87372 2.32591 2.94032 3.78185 2.99630 3.83394 2.82804 2.88667
2.66743 2.50351 3.00357 5.21807 3.51586 650 e - - -
2.68618 4.42225 2.77519 2.73123 3.46354 2.40513 3.72494
3.29354 2.67741 2.69355 4.24690 2.90347 2.73739 3.18146 2.89801
2.37887 2.77519 2.98518 4.58477 3.61503
0.02625 4.04896 4.77131 0.61958 0.77255 0.48576 0.95510
549 2.63346 4.87070 2.82653 2.40244 4.20148 2.55972 3.65797
3.63714 2.42925 2.96326 4.02679 2.55840 3.81369 2.15738 2.86820
2.49787 2.88844 3.28168 5.42278 4.07660 651 q - - -
2.68620 4.42227 2.77521 2.73125 3.46356 2.40514 3.72496
3.29356 2.67742 2.69347 4.24691 2.90348 2.73741 3.18148 2.89802
2.37875 2.77521 2.98520 4.58479 3.61505
0.27385 2.88422 1.69470 0.78006 0.61318 0.48576 0.95510
550 2.37892 4.43145 2.91675 2.57761 4.13655 3.09017 3.80568
3.55856 2.66898 3.19231 4.01910 2.48097 3.10157 3.00225 3.07683
1.88302 2.47250 3.13611 5.42741 4.13186 655 s - - -
2.68618 4.42225 2.77519 2.73123 3.46354 2.40513 3.72494
3.29354 2.67741 2.69355 4.24690 2.90347 2.73739 3.18146 2.89801
2.37887 2.77519 2.98518 4.58477 3.61503
0.03192 3.85636 4.57871 0.61958 0.77255 0.39025 1.12976

```
    551   2.65071  4.91816  2.88677  2.41210  4.25240  3.38931  3.63024
3.68144  2.33957  3.23887  4.04325  2.13857  3.82030  2.56623  2.44848
2.46995  2.53682  3.32124  5.42892  4.09051    656 n - - -
          2.68618  4.42225  2.77519  2.73123  3.46354  2.40513  3.72494
3.29354  2.67741  2.69355  4.24690  2.90347  2.73739  3.18146  2.89801
2.37887  2.77519  2.98518  4.58477  3.61503
          0.02625  4.04896  4.77131  0.61958  0.77255  0.48576  0.95510
    552   2.11701  4.33498  3.33257  2.85685  3.89398  2.83296  3.91466
3.24116  2.83367  2.69437  3.81176  3.20944  3.28481  3.16233  3.20808
2.57312  1.76205  2.90254  5.26858  4.01828    657 t - - -
          2.68618  4.42225  2.77519  2.73123  3.46354  2.40513  3.72494
3.29354  2.67741  2.69355  4.24690  2.90347  2.73739  3.18146  2.89801
2.37887  2.77519  2.98518  4.58477  3.61503
          0.02625  4.04896  4.77131  0.61958  0.77255  0.48576  0.95510
    553   2.41093  3.55864  3.58065  3.01661  3.36405  3.54578  3.86074
2.41163  2.94947  2.43814  3.34458  3.10589  3.94584  3.24235  3.26564
2.81479  2.10979  2.46359  4.83212  3.21400    658 t - - -
          2.68618  4.42225  2.77519  2.73123  3.46354  2.40513  3.72494
3.29354  2.67741  2.69355  4.24690  2.90347  2.73739  3.18146  2.89801
2.37887  2.77519  2.98518  4.58477  3.61503
          0.02625  4.04896  4.77131  0.61958  0.77255  0.48576  0.95510
    554   1.92860  4.32953  4.12667  3.59545  3.43590  3.91952  4.36779
1.67625  3.48573  1.89196  3.32465  3.88517  4.31413  3.77747  3.74376
3.24755  3.08874  1.97553  5.15074  3.94669    659 i - - -
          2.68618  4.42225  2.77519  2.73123  3.46354  2.40513  3.72494
3.29354  2.67741  2.69355  4.24690  2.90347  2.73739  3.18146  2.89801
2.37887  2.77519  2.98518  4.58477  3.61503
          0.02625  4.04896  4.77131  0.61958  0.77255  0.48576  0.95510
    555   2.63544  4.95418  2.72145  2.38748  4.24304  3.41297  3.22480
3.68050  2.11028  3.22480  4.01595  2.91208  3.80666  2.71287  2.10467
2.63267  2.68952  3.31856  5.40428  3.61102    660 r - - -
          2.68618  4.42225  2.77519  2.73123  3.46354  2.40513  3.72494
3.29354  2.67741  2.69355  4.24690  2.90347  2.73739  3.18146  2.89801
2.37887  2.77519  2.98518  4.58477  3.61503
          0.02625  4.04896  4.77131  0.61958  0.77255  0.48576  0.95510
    556   3.51130  4.92842  4.10319  3.86868  2.32121  4.03918  3.71497
3.49696  3.72133  2.90449  4.15421  3.94722  4.51032  3.98439  3.86748
3.62915  3.79964  3.37626  3.97068  0.71613    661 y - - -
          2.68618  4.42225  2.77519  2.73123  3.46354  2.40513  3.72494
3.29354  2.67741  2.69355  4.24690  2.90347  2.73739  3.18146  2.89801
2.37887  2.77519  2.98518  4.58477  3.61503
          0.02625  4.04896  4.77131  0.61958  0.77255  0.48576  0.95510
    557   2.60857  4.45939  3.37081  2.81673  3.68013  3.48840  3.80084
2.97813  2.61429  2.66889  2.88232  3.23192  3.91932  3.05261  2.56130
2.77248  1.86582  2.74376  5.08705  3.83934    662 t - - -
          2.68618  4.42225  2.77519  2.73123  3.46354  2.40513  3.72494
3.29354  2.67741  2.69355  4.24690  2.90347  2.73739  3.18146  2.89801
2.37887  2.77519  2.98518  4.58477  3.61503
          0.02625  4.04896  4.77131  0.61958  0.77255  0.48576  0.95510
    558   3.36127  4.70789  4.76375  4.26415  2.32997  4.49058  4.54762
2.31556  4.11670  0.86113  2.95555  4.46558  4.71806  4.22937  4.23579
3.86600  3.59145  2.45008  4.80591  3.42930    663 l - - -
          2.68618  4.42225  2.77519  2.73123  3.46354  2.40513  3.72494
3.29354  2.67741  2.69355  4.24690  2.90347  2.73739  3.18146  2.89801
2.37887  2.77519  2.98518  4.58477  3.61503
```

```
         0.02625  4.04896  4.77131  0.61958  0.77255  0.48576  0.95510
    559  2.63738  4.69353  3.19304  2.72743  4.24097  3.34377  3.78371
3.68574  2.37792  3.27257  4.12426  3.13705  3.87343  2.96164  1.79908
1.72391  2.95243  3.30956  5.43120  4.15742    664 s - - -
         2.68618  4.42225  2.77519  2.73123  3.46354  2.40513  3.72494
3.29354  2.67741  2.69355  4.24690  2.90347  2.73739  3.18146  2.89801
2.37887  2.77519  2.98518  4.58477  3.61503
         0.02625  4.04896  4.77131  0.61958  0.77255  0.48576  0.95510
    560  1.87829  4.24709  3.35559  3.18587  4.43530  1.11726  4.33162
3.81175  3.34349  3.54914  4.40909  3.35311  3.75389  3.62214  3.63735
2.48927  2.81672  3.26153  5.75976  4.56627    665 g - - -
         2.68618  4.42225  2.77519  2.73123  3.46354  2.40513  3.72494
3.29354  2.67741  2.69355  4.24690  2.90347  2.73739  3.18146  2.89801
2.37887  2.77519  2.98518  4.58477  3.61503
         0.02625  4.04896  4.77131  0.61958  0.77255  0.48576  0.95510
    561  2.30520  5.00015  2.27278  2.30868  4.47171  3.24236  3.78977
3.92580  2.70781  3.51589  4.34919  1.53983  3.82920  2.95963  3.20991
2.71485  3.03705  3.51939  5.70913  4.30572    666 n - - -
         2.68618  4.42225  2.77519  2.73123  3.46354  2.40513  3.72494
3.29354  2.67741  2.69355  4.24690  2.90347  2.73739  3.18146  2.89801
2.37887  2.77519  2.98518  4.58477  3.61503
         0.02625  4.04896  4.77131  0.61958  0.77255  0.48576  0.95510
    562  2.66268  4.63252  3.18259  2.87513  4.33804  1.21076  4.00021
3.82218  2.71443  3.42884  4.30335  3.25406  3.89993  3.22279  2.30362
2.77367  3.04027  3.40908  5.53350  4.31798    667 g - - -
         2.68618  4.42225  2.77519  2.73123  3.46354  2.40513  3.72494
3.29354  2.67741  2.69355  4.24690  2.90347  2.73739  3.18146  2.89801
2.37887  2.77519  2.98518  4.58477  3.61503
         0.02625  4.04896  4.77131  0.61958  0.77255  0.48576  0.95510
    563  2.65416  4.89955  2.91737  2.42312  4.18573  3.41095  3.62303
3.61594  2.33386  2.89343  3.99570  2.16507  3.82662  2.43896  2.57340
2.39423  2.88807  3.27237  5.38251  4.04754    668 n - - -
         2.68618  4.42225  2.77519  2.73123  3.46354  2.40513  3.72494
3.29354  2.67741  2.69355  4.24690  2.90347  2.73739  3.18146  2.89801
2.37887  2.77519  2.98518  4.58477  3.61503
         0.28203  4.04896  1.47705  0.61958  0.77255  0.48576  0.95510
    564  2.54166  4.59002  2.13864  2.46394  3.96310  3.26025  3.74270
3.10433  2.63023  2.94669  3.86066  2.95121  3.78378  2.93845  3.07424
2.62796  2.57929  2.39335  5.33191  4.00391    669 d - - -
         2.68618  4.42225  2.77519  2.73123  3.46354  2.40513  3.72494
3.29354  2.67741  2.69355  4.24690  2.90347  2.73739  3.18146  2.89801
2.37887  2.77519  2.98518  4.58477  3.61503
         0.03377  3.80070  4.52305  0.61958  0.77255  0.37168  1.16980
    565  2.59353  4.61448  3.10982  2.58293  3.86852  3.42640  3.69524
2.62460  2.49166  2.90971  3.76132  2.61434  3.84864  2.89140  2.52373
2.13654  2.84215  2.96481  5.18962  3.89887    670 s - - -
         2.68618  4.42225  2.77519  2.73123  3.46354  2.40513  3.72494
3.29354  2.67741  2.69355  4.24690  2.90347  2.73739  3.18146  2.89801
2.37887  2.77519  2.98518  4.58477  3.61503
         0.02625  4.04896  4.77131  0.61958  0.77255  0.48576  0.95510
    566  3.51130  4.92842  4.10319  3.86868  2.32121  4.03918  3.71497
3.49696  3.72133  2.90449  4.15421  3.94722  4.51032  3.98439  3.86748
3.62915  3.79964  3.37626  3.97068  0.71613    671 y - - -
```

```
         2.68618  4.42225  2.77519  2.73123  3.46354  2.40513  3.72494
3.29354  2.67741  2.69355  4.24690  2.90347  2.73739  3.18146  2.89801
2.37887  2.77519  2.98518  4.58477  3.61503
         0.02625  4.04896  4.77131  0.61958  0.77255  0.48576  0.95510
    567  2.84839  5.08882  3.00951  2.53836  4.46316  3.49999  3.63739
3.87788  2.00729  3.37814  4.20831  1.92559  3.92358  2.77409  1.87122
2.83860  3.06467  3.52542  5.48833  4.20915    672 r - - -
         2.68618  4.42225  2.77519  2.73123  3.46354  2.40513  3.72494
3.29354  2.67741  2.69355  4.24690  2.90347  2.73739  3.18146  2.89801
2.37887  2.77519  2.98518  4.58477  3.61503
         0.02625  4.04896  4.77131  0.61958  0.77255  0.48576  0.95510
    568  3.32518  4.68925  4.74162  4.23691  3.11895  4.49691  4.82284
2.00234  4.02070  0.83314  2.97331  4.50689  4.74163  4.24652  4.17303
3.88818  3.56885  2.22872  5.18538  4.01270    673 l - - -
         2.68618  4.42225  2.77519  2.73123  3.46354  2.40513  3.72494
3.29354  2.67741  2.69355  4.24690  2.90347  2.73739  3.18146  2.89801
2.37887  2.77519  2.98518  4.58477  3.61503
         0.02625  4.04896  4.77131  0.61958  0.77255  0.48576  0.95510
    569  2.88215  4.40656  4.01462  3.53756  2.79473  3.85733  3.93326
2.54923  3.39506  2.33126  3.45503  3.75736  4.26330  3.67723  3.61924
3.19765  3.14358  1.70239  4.44167  1.74382    674 v - - -
         2.68618  4.42225  2.77519  2.73123  3.46354  2.40513  3.72494
3.29354  2.67741  2.69355  4.24690  2.90347  2.73739  3.18146  2.89801
2.37887  2.77519  2.98518  4.58477  3.61503
         0.02625  4.04896  4.77131  0.61958  0.77255  0.48576  0.95510
    570  3.35841  4.77029  4.47216  4.11619  3.21164  4.20451  4.71683
2.42879  3.88223  0.68484  3.18550  4.37450  4.61168  4.20806  4.02805
3.80108  3.64584  2.51132  5.15144  3.91438    675 L - - -
         2.68618  4.42225  2.77519  2.73123  3.46354  2.40513  3.72494
3.29354  2.67741  2.69355  4.24690  2.90347  2.73739  3.18146  2.89801
2.37887  2.77519  2.98518  4.58477  3.61503
         0.02625  4.04896  4.77131  0.61958  0.77255  0.48576  0.95510
    571  3.21415  5.03434  3.72375  3.20215  4.43412  3.66389  3.96639
3.99095  2.32798  3.47493  4.47340  3.54687  4.16051  3.18049  0.73952
3.29160  3.46976  3.70799  5.44719  4.32628    676 r - - -
         2.68618  4.42225  2.77519  2.73123  3.46354  2.40513  3.72494
3.29354  2.67741  2.69355  4.24690  2.90347  2.73739  3.18146  2.89801
2.37887  2.77519  2.98518  4.58477  3.61503
         0.02625  4.04896  4.77131  0.61958  0.77255  0.48576  0.95510
    572  3.11893  4.44801  4.75056  4.22477  3.45998  4.43950  4.90714
1.72405  4.10044  1.69021  3.27906  4.46756  4.72537  4.34653  4.29658
3.80269  3.37190  1.10688  5.41593  4.22931    677 v - - -
         2.68618  4.42225  2.77519  2.73123  3.46354  2.40513  3.72494
3.29354  2.67741  2.69355  4.24690  2.90347  2.73739  3.18146  2.89801
2.37887  2.77519  2.98518  4.58477  3.61503
         0.02625  4.04896  4.77131  0.61958  0.77255  0.48576  0.95510
    573  2.41035  4.33126  3.36913  3.19663  4.25684  3.05832  4.29982
3.80508  3.28244  3.51284  4.42630  3.39050  3.81243  3.61846  3.56056
0.85292  2.90434  3.30106  5.61579  4.34228    678 s - - -
         2.68618  4.42225  2.77519  2.73123  3.46354  2.40513  3.72494
3.29354  2.67741  2.69355  4.24690  2.90347  2.73739  3.18146  2.89801
2.37887  2.77519  2.98518  4.58477  3.61503
         0.02625  4.04896  4.77131  0.61958  0.77255  0.48576  0.95510
```

```
    574   2.29436  4.26346  3.25535  3.05221  4.43120  1.54319  4.23374
3.90702  3.21734  3.57002  4.39130  3.26968  3.72856  3.49554  3.54862
1.49058  2.78630  3.31731  5.74479  4.51006    679 s - - -
          2.68618  4.42225  2.77519  2.73123  3.46354  2.40513  3.72494
3.29354  2.67741  2.69355  4.24690  2.90347  2.73739  3.18146  2.89801
2.37887  2.77519  2.98518  4.58477  3.61503
          0.02625  4.04896  4.77131  0.61958  0.77255  0.48576  0.95510
    575   2.41706  4.31585  3.42612  2.86570  3.47928  3.53380  3.81212
2.29580  2.80664  2.41866  3.43293  3.27539  3.92672  2.72343  3.16316
2.59226  2.51616  2.40725  4.92445  3.68653    680 i - - -
          2.68618  4.42225  2.77519  2.73123  3.46354  2.40513  3.72494
3.29354  2.67741  2.69355  4.24690  2.90347  2.73739  3.18146  2.89801
2.37887  2.77519  2.98518  4.58477  3.61503
          0.02625  4.04896  4.77131  0.61958  0.77255  0.48576  0.95510
    576   1.75443  4.26476  3.43727  3.03811  3.97879  1.75199  4.07498
2.79996  3.03095  3.02036  3.91528  3.30674  3.79987  3.34952  3.36953
2.55308  2.77971  2.86283  5.38480  4.14631    681 g - - -
          2.68618  4.42225  2.77519  2.73123  3.46354  2.40513  3.72494
3.29354  2.67741  2.69355  4.24690  2.90347  2.73739  3.18146  2.89801
2.37887  2.77519  2.98518  4.58477  3.61503
          0.07868  4.04896  2.84353  0.61958  0.77255  0.48576  0.95510
    577   2.45971  4.55902  2.85409  2.56133  4.30679  2.79468  3.85476
3.74706  2.72494  3.35750  4.17337  1.86259  3.75086  3.03880  3.15467
2.02902  2.54787  3.29503  5.57057  4.25388    682 n - - -
          2.68618  4.42225  2.77519  2.73123  3.46354  2.40513  3.72494
3.29354  2.67741  2.69355  4.24690  2.90347  2.73739  3.18146  2.89801
2.37887  2.77519  2.98518  4.58477  3.61503
          0.02765  3.99793  4.72027  0.61958  0.77255  0.45412  1.00788
    578   2.61673  4.79624  2.92968  2.47224  4.17263  3.36176  3.67621
3.59311  2.41502  3.18023  3.99873  2.75757  3.82138  2.60327  2.61891
1.80791  2.63428  3.24108  5.39541  4.07412    683 s - - -
          2.68618  4.42225  2.77519  2.73123  3.46354  2.40513  3.72494
3.29354  2.67741  2.69355  4.24690  2.90347  2.73739  3.18146  2.89801
2.37887  2.77519  2.98518  4.58477  3.61503
          0.02625  4.04896  4.77131  0.61958  0.77255  0.48576  0.95510
    579   2.79029  4.31084  4.14237  3.62389  3.53575  3.87437  4.41618
1.54146  3.51525  2.26633  3.41685  3.89388  4.30704  3.81700  3.77657
3.21937  2.00531  1.80922  5.23443  4.02492    684 i - - -
          2.68618  4.42225  2.77519  2.73123  3.46354  2.40513  3.72494
3.29354  2.67741  2.69355  4.24690  2.90347  2.73739  3.18146  2.89801
2.37887  2.77519  2.98518  4.58477  3.61503
          0.02625  4.04896  4.77131  0.61958  0.77255  0.48576  0.95510
    580   2.68260  4.18272  3.99080  3.42176  2.99197  3.79103  4.11043
1.82728  2.96917  2.24693  3.26611  3.70452  4.15479  3.58431  3.56114
3.07901  2.69799  1.73003  4.88153  3.67662    685 v - - -
          2.68618  4.42225  2.77519  2.73123  3.46354  2.40513  3.72494
3.29354  2.67741  2.69355  4.24690  2.90347  2.73739  3.18146  2.89801
2.37887  2.77519  2.98518  4.58477  3.61503
          0.02625  4.04896  4.77131  0.61958  0.77255  0.48576  0.95510
    581   2.06421  4.54182  3.30935  2.74662  3.80425  3.47319  3.76786
3.07069  2.52160  2.56671  3.70315  3.18027  3.90445  2.98563  2.06266
2.75128  2.87743  2.63221  5.16799  3.90679    686 r - - -
          2.68618  4.42225  2.77519  2.73123  3.46354  2.40513  3.72494
3.29354  2.67741  2.69355  4.24690  2.90347  2.73739  3.18146  2.89801
2.37887  2.77519  2.98518  4.58477  3.61503
```

0.02625 4.04896 4.77131 0.61958 0.77255 0.48576 0.95510
582 2.58233 4.26594 3.51302 2.94620 3.39785 3.57235 3.82888
2.43791 2.67708 2.45539 3.36754 3.33352 3.95153 2.91361 3.18314
2.82806 2.61574 1.95913 4.85784 3.30436 687 v - - -
2.68618 4.42225 2.77519 2.73123 3.46354 2.40513 3.72494
3.29354 2.67741 2.69355 4.24690 2.90347 2.73739 3.18146 2.89801
2.37887 2.77519 2.98518 4.58477 3.61503
0.07670 4.04896 2.87536 0.61958 0.77255 0.48576 0.95510
583 2.59425 4.26531 3.61696 3.08939 3.36791 3.55850 3.93475
2.58471 2.99964 2.42726 3.41112 3.43233 3.99451 3.31968 3.30809
2.86125 2.02620 1.99160 4.89059 2.93082 688 v - - -
2.68618 4.42225 2.77519 2.73123 3.46354 2.40513 3.72494
3.29354 2.67741 2.69355 4.24690 2.90347 2.73739 3.18146 2.89801
2.37887 2.77519 2.98518 4.58477 3.61503
0.02759 3.99985 4.72219 0.61958 0.77255 0.53666 0.87875
584 2.62615 4.73391 2.51536 2.21993 3.95040 3.40698 3.66196
2.31568 2.48957 2.97212 3.82219 2.95905 3.83058 2.84045 2.72601
2.67986 2.86292 3.01905 5.25885 3.94026 689 e - - -
2.68618 4.42225 2.77519 2.73123 3.46354 2.40513 3.72494
3.29354 2.67741 2.69355 4.24690 2.90347 2.73739 3.18146 2.89801
2.37887 2.77519 2.98518 4.58477 3.61503
0.02759 3.99985 4.72219 0.61958 0.77255 0.53666 0.87875
585 2.61983 4.72041 2.65802 2.52153 4.41931 2.39363 3.93143
3.96162 2.84297 3.56628 4.42020 1.35065 3.81451 3.14247 3.26188
2.68034 3.00987 3.49788 5.67128 4.33045 690 n - - -
2.68618 4.42225 2.77519 2.73123 3.46354 2.40513 3.72494
3.29354 2.67741 2.69355 4.24690 2.90347 2.73739 3.18146 2.89801
2.37887 2.77519 2.98518 4.58477 3.61503
0.02759 3.99985 4.72219 0.61958 0.77255 0.45520 1.00599
586 2.42852 4.51284 3.13093 2.62739 3.74115 2.35289 3.73078
3.14790 2.62012 2.60653 3.67852 2.85555 3.84800 2.95912 3.03072
2.49929 2.81842 2.86962 5.11266 3.12893 691 g - - -
2.68618 4.42225 2.77519 2.73123 3.46354 2.40513 3.72494
3.29354 2.67741 2.69355 4.24690 2.90347 2.73739 3.18146 2.89801
2.37887 2.77519 2.98518 4.58477 3.61503
0.02625 4.04896 4.77131 0.61958 0.77255 0.48576 0.95510
587 2.64577 4.77154 3.09055 2.52188 4.01134 3.46041 3.63312
3.42654 2.33051 3.02912 3.86152 3.01460 2.89608 2.59676 2.06992
2.53707 2.87198 3.11937 4.15875 3.95358 692 r - - -
2.68618 4.42225 2.77519 2.73123 3.46354 2.40513 3.72494
3.29354 2.67741 2.69355 4.24690 2.90347 2.73739 3.18146 2.89801
2.37887 2.77519 2.98518 4.58477 3.61503
0.02625 4.04896 4.77131 0.61958 0.77255 0.48576 0.95510
588 2.69627 4.27139 3.88638 3.33685 3.46973 3.73742 4.13539
2.14536 3.17911 2.35597 3.39811 3.64968 4.14826 3.52498 3.09665
3.04766 2.67787 1.41112 5.04347 3.82836 693 v - - -
2.68618 4.42225 2.77519 2.73123 3.46354 2.40513 3.72494
3.29354 2.67741 2.69355 4.24690 2.90347 2.73739 3.18146 2.89801
2.37887 2.77519 2.98518 4.58477 3.61503
0.02625 4.04896 4.77131 0.61958 0.77255 0.48576 0.95510
589 2.56191 4.42127 3.24789 2.83504 3.52583 2.16055 3.84097
3.13773 2.82727 2.82714 3.72839 3.22374 3.89087 3.16827 3.18254
2.72277 2.58764 2.85863 4.98502 2.18520 694 g - - -

```
         2.68618  4.42225  2.77519  2.73123  3.46354  2.40513  3.72494
3.29354  2.67741  2.69355  4.24690  2.90347  2.73739  3.18146  2.89801
2.37887  2.77519  2.98518  4.58477  3.61503
         0.02625  4.04896  4.77131  0.61958  0.77255  0.48576  0.95510
    590  2.15468  4.37567  3.28362  2.79480  3.78620  3.30306  3.85134
3.15511  2.77253  2.85722  3.72505  3.18519  3.82689  3.10415  3.15381
2.14676  1.97100  2.85040  5.17320  3.34920    695 t - - -
         2.68618  4.42225  2.77519  2.73123  3.46354  2.40513  3.72494
3.29354  2.67741  2.69355  4.24690  2.90347  2.73739  3.18146  2.89801
2.37887  2.77519  2.98518  4.58477  3.61503
         0.02625  4.04896  4.77131  0.61958  0.77255  0.48576  0.95510
    591  1.94175  4.26615  3.92284  3.37070  3.45474  3.77124  4.17992
2.08196  3.28916  2.02581  3.36844  3.68722  4.17314  3.57690  3.57726
3.07320  2.73583  1.86467  5.05626  3.84667    696 v - - -
         2.68618  4.42225  2.77519  2.73123  3.46354  2.40513  3.72494
3.29354  2.67741  2.69355  4.24690  2.90347  2.73739  3.18146  2.89801
2.37887  2.77519  2.98518  4.58477  3.61503
         0.06591  4.04896  3.07176  0.61958  0.77255  0.48576  0.95510
    592  2.58373  4.71788  2.99770  2.34176  3.94535  3.41420  3.62562
3.35235  2.42362  2.80361  3.80041  2.65157  3.80950  2.79161  2.66896
2.40229  2.45775  3.04622  5.23119  3.45115    697 e - - -
         2.68618  4.42225  2.77519  2.73123  3.46354  2.40513  3.72494
3.29354  2.67741  2.69355  4.24690  2.90347  2.73739  3.18146  2.89801
2.37887  2.77519  2.98518  4.58477  3.61503
         0.02730  4.01035  4.73270  0.61958  0.77255  0.46129  0.99553
    593  2.45285  4.84364  2.89241  2.39879  4.11451  3.38789  3.62461
3.54151  2.40539  3.12884  3.93842  2.29619  3.43485  2.34812  2.85860
2.62922  2.68647  3.20272  5.35245  3.53467    698 n - - -
         2.68618  4.42225  2.77519  2.73123  3.46354  2.40513  3.72494
3.29354  2.67741  2.69355  4.24690  2.90347  2.73739  3.18146  2.89801
2.37887  2.77519  2.98518  4.58477  3.61503
         0.02625  4.04896  4.77131  0.61958  0.77255  0.48576  0.95510
    594  3.02585  4.36863  4.69529  4.14657  2.34067  4.28916  4.63185
1.65800  4.03014  1.88504  3.16741  4.33031  4.57973  4.20470  4.17462
3.62783  3.26676  1.40216  5.08478  3.85244    699 v - - -
         2.68618  4.42225  2.77519  2.73123  3.46354  2.40513  3.72494
3.29354  2.67741  2.69355  4.24690  2.90347  2.73739  3.18146  2.89801
2.37887  2.77519  2.98518  4.58477  3.61503
         0.02625  4.04896  4.77131  0.61958  0.77255  0.48576  0.95510
    595  2.68727  4.92084  2.46078  2.36952  4.18792  3.33952  3.70411
3.63755  2.54450  3.25230  3.50158  1.65333  3.83460  2.87019  3.00815
2.69729  2.95353  3.29619  5.45969  4.07965    700 n - - -
         2.68618  4.42225  2.77519  2.73123  3.46354  2.40513  3.72494
3.29354  2.67741  2.69355  4.24690  2.90347  2.73739  3.18146  2.89801
2.37887  2.77519  2.98518  4.58477  3.61503
         0.02625  4.04896  4.77131  0.61958  0.77255  0.48576  0.95510
    596  2.51696  4.36119  3.59478  3.35937  4.13457  3.19354  4.36194
3.28365  3.30583  3.14078  4.18273  3.53737  3.91252  3.69255  3.55365
2.72057  0.90433  2.95928  5.55663  4.35230    701 t - - -
         2.68618  4.42225  2.77519  2.73123  3.46354  2.40513  3.72494
3.29354  2.67741  2.69355  4.24690  2.90347  2.73739  3.18146  2.89801
2.37887  2.77519  2.98518  4.58477  3.61503
         0.02625  4.04896  4.77131  0.61958  0.77255  0.48576  0.95510
```

```
    597   2.31386  4.23860  3.44974  3.03011  4.05505  2.22547  4.06792
3.40705  3.01926  3.12734  3.97665  3.28139  3.75487  3.33037  3.36501
2.24004  1.65347  2.64578  5.42471  4.19366    702 t - - -
          2.68618  4.42225  2.77519  2.73123  3.46354  2.40513  3.72494
3.29354  2.67741  2.69355  4.24690  2.90347  2.73739  3.18146  2.89801
2.37887  2.77519  2.98518  4.58477  3.61503
          0.02625  4.04896  4.77131  0.61958  0.77255  0.48576  0.95510
    598   2.60342  4.71055  2.48695  2.48726  4.21863  3.24475  3.82934
3.61465  2.72502  3.27563  4.12423  2.69519  3.82288  3.02302  3.17039
2.66571  1.50957  3.24558  5.51857  4.18180    703 t - - -
          2.68618  4.42225  2.77519  2.73123  3.46354  2.40513  3.72494
3.29354  2.67741  2.69355  4.24690  2.90347  2.73739  3.18146  2.89801
2.37887  2.77519  2.98518  4.58477  3.61503
          0.02625  4.04896  4.77131  0.61958  0.77255  0.48576  0.95510
    599   2.91008  5.35291  1.77547  2.18843  4.68361  3.24699  3.80958
4.24216  2.80707  3.77221  4.62626  1.42872  3.86778  2.98467  3.36887
2.83020  3.21469  3.81754  5.89658  4.41884    704 n - - -
          2.68618  4.42225  2.77519  2.73123  3.46354  2.40513  3.72494
3.29354  2.67741  2.69355  4.24690  2.90347  2.73739  3.18146  2.89801
2.37887  2.77519  2.98518  4.58477  3.61503
          0.02625  4.04896  4.77131  0.61958  0.77255  0.48576  0.95510
    600   2.87931  4.87574  2.82111  2.72621  4.26801  3.31004  4.07758
4.02015  2.99819  3.64783  4.60465  0.87973  3.97208  3.35350  3.35640
2.94155  3.26875  3.62429  5.55383  4.20588    705 n - - -
          2.68618  4.42225  2.77519  2.73123  3.46354  2.40513  3.72494
3.29354  2.67741  2.69355  4.24690  2.90347  2.73739  3.18146  2.89801
2.37887  2.77519  2.98518  4.58477  3.61503
          0.02625  4.04896  4.77131  0.61958  0.77255  0.48576  0.95510
    601   2.92963  5.51919  1.55732  1.52632  4.80015  3.25831  3.75338
4.31935  2.72468  3.80168  4.63175  2.71069  3.85500  2.60309  3.30228
2.81763  3.20619  3.88683  5.95612  4.45618    706 e - - -
          2.68618  4.42225  2.77519  2.73123  3.46354  2.40513  3.72494
3.29354  2.67741  2.69355  4.24690  2.90347  2.73739  3.18146  2.89801
2.37887  2.77519  2.98518  4.58477  3.61503
          0.02625  4.04896  4.77131  0.61958  0.77255  0.48576  0.95510
    602   2.89902  4.65351  3.60866  3.53220  4.66473  0.50220  4.63853
4.31037  3.73728  3.94135  4.92090  3.77344  4.05011  4.04773  3.95090
3.07653  3.39400  3.80462  5.71003  4.77372    707 G - - -
          2.68618  4.42225  2.77519  2.73123  3.46354  2.40513  3.72494
3.29354  2.67741  2.69355  4.24690  2.90347  2.73739  3.18146  2.89801
2.37887  2.77519  2.98518  4.58477  3.61503
          0.02625  4.04896  4.77131  0.61958  0.77255  0.48576  0.95510
    603   3.09147  4.42758  4.76269  4.27790  3.60076  4.40009  5.00816
1.54362  4.15824  2.12046  3.41803  4.49498  4.74202  4.44000  4.36452
3.79487  3.36683  0.98461  5.55451  4.33452    708 v - - -
          2.68618  4.42225  2.77519  2.73123  3.46354  2.40513  3.72494
3.29354  2.67741  2.69355  4.24690  2.90347  2.73739  3.18146  2.89801
2.37887  2.77519  2.98518  4.58477  3.61503
          0.02625  4.04896  4.77131  0.61958  0.77255  0.48576  0.95510
    604   2.60921  4.37071  3.42075  2.89275  3.56200  3.54550  3.87066
2.12605  2.82309  2.42743  3.50959  2.54074  3.96285  3.16712  3.17751
2.82304  2.34144  2.47826  5.02544  3.77366    709 i - - -
          2.68618  4.42225  2.77519  2.73123  3.46354  2.40513  3.72494
3.29354  2.67741  2.69355  4.24690  2.90347  2.73739  3.18146  2.89801
2.37887  2.77519  2.98518  4.58477  3.61503
```

```
          0.02625  4.04896  4.77131  0.61958  0.77255  0.48576  0.95510
    605   3.18840  5.33296  0.69448  2.46210  4.73586  3.35266  4.09910
4.36616  3.21856  3.94434  4.92964  3.02608  4.01823  3.34089  3.75621
3.13313  3.53189  3.98763  5.86922  4.58391    710 d - - -
          2.68618  4.42225  2.77519  2.73123  3.46354  2.40513  3.72494
3.29354  2.67741  2.69355  4.24690  2.90347  2.73739  3.18146  2.89801
2.37887  2.77519  2.98518  4.58477  3.61503
          0.02625  4.04896  4.77131  0.61958  0.77255  0.48576  0.95510
    606   2.87931  4.87574  2.82111  2.72621  4.26801  3.31004  4.07758
4.02015  2.99819  3.64783  4.60465  0.87973  3.97208  3.35350  3.35640
2.94155  3.26875  3.62429  5.55383  4.20588    711 n - - -
          2.68618  4.42225  2.77519  2.73123  3.46354  2.40513  3.72494
3.29354  2.67741  2.69355  4.24690  2.90347  2.73739  3.18146  2.89801
2.37887  2.77519  2.98518  4.58477  3.61503
          0.02625  4.04896  4.77131  0.61958  0.77255  0.48576  0.95510
    607   2.83027  5.06856  1.66422  2.34966  4.66182  1.47817  3.92393
4.18077  2.94077  3.74864  4.60834  2.88929  3.87464  3.11757  3.47254
2.81645  3.18182  3.73266  5.85768  4.48494    712 g - - -
          2.68618  4.42225  2.77519  2.73123  3.46354  2.40513  3.72494
3.29354  2.67741  2.69355  4.24690  2.90347  2.73739  3.18146  2.89801
2.37887  2.77519  2.98518  4.58477  3.61503
          0.02625  4.04896  4.77131  0.61958  0.77255  0.48576  0.95510
    608   1.43188  4.18328  3.48346  3.20070  4.30544  2.96878  4.26122
3.66662  3.24697  3.40534  4.25027  3.34171  3.72101  3.53989  3.55075
1.41395  2.73640  3.14396  5.66475  4.44703    713 s - - -
          2.68618  4.42225  2.77519  2.73123  3.46354  2.40513  3.72494
3.29354  2.67741  2.69355  4.24690  2.90347  2.73739  3.18146  2.89801
2.37887  2.77519  2.98518  4.58477  3.61503
          0.02625  4.04896  4.77131  0.61958  0.77255  0.48576  0.95510
    609   2.99440  5.19932  3.37783  2.71032  4.62992  3.65139  3.19091
3.99210  1.45720  3.42637  4.26682  3.14461  4.00718  2.51511  1.66294
2.97565  3.16254  3.65371  5.47518  4.27359    714 k - - -
          2.68618  4.42225  2.77519  2.73123  3.46354  2.40513  3.72494
3.29354  2.67741  2.69355  4.24690  2.90347  2.73739  3.18146  2.89801
2.37887  2.77519  2.98518  4.58477  3.61503
          0.02625  4.04896  4.77131  0.61958  0.77255  0.48576  0.95510
    610   2.92383  4.50488  3.99557  3.65616  1.13106  3.74653  3.84499
2.93894  3.58286  2.50285  3.67069  3.77117  4.26056  3.80592  3.77484
2.74448  3.25275  2.79310  4.22955  2.60064    715 f - - -
          2.68618  4.42225  2.77519  2.73123  3.46354  2.40513  3.72494
3.29354  2.67741  2.69355  4.24690  2.90347  2.73739  3.18146  2.89801
2.37887  2.77519  2.98518  4.58477  3.61503
          0.02625  4.04896  4.77131  0.61958  0.77255  0.48576  0.95510
    611   2.64073  4.73958  3.05200  2.52810  3.99598  3.44295  3.65980
2.88454  2.03568  3.00942  3.85184  3.01913  3.85279  2.64462  2.77057
2.04224  2.87633  3.08424  5.26275  3.96362    716 k - - -
          2.68618  4.42225  2.77519  2.73123  3.46354  2.40513  3.72494
3.29354  2.67741  2.69355  4.24690  2.90347  2.73739  3.18146  2.89801
2.37887  2.77519  2.98518  4.58477  3.61503
          0.02625  4.04896  4.77131  0.61958  0.77255  0.48576  0.95510
    612   2.98156  5.43425  1.02925  2.17338  4.75781  3.23306  3.86013
4.34812  2.92799  3.88529  4.76995  2.45673  3.88865  3.04991  3.52238
2.88597  3.30333  3.91741  5.98673  4.48626    717 d - - -
```

```
         2.68618  4.42225  2.77519  2.73123  3.46354  2.40513  3.72494
3.29354  2.67741  2.69355  4.24690  2.90347  2.73739  3.18146  2.89801
2.37887  2.77519  2.98518  4.58477  3.61503
         0.02625  4.04896  4.77131  0.61958  0.77255  0.48576  0.95510
    613  3.17366  4.50336  4.79539  4.24550  2.04056  4.40677  4.59167
1.52564  4.11390  1.46989  3.00552  4.42185  4.64463  4.22602  4.22317
3.74613  3.40126  2.14321  4.91866  3.63089    718 l - - -
         2.68618  4.42225  2.77519  2.73123  3.46354  2.40513  3.72494
3.29354  2.67741  2.69355  4.24690  2.90347  2.73739  3.18146  2.89801
2.37887  2.77519  2.98518  4.58477  3.61503
         0.02625  4.04896  4.77131  0.61958  0.77255  0.48576  0.95510
    614  2.43269  4.51839  3.16022  2.47715  3.37332  3.46779  3.34292
2.73096  2.58502  2.74836  3.61085  2.42334  3.85648  2.92154  3.00296
2.69456  2.80744  2.62760  5.06530  3.78389    719 n - - -
         2.68618  4.42225  2.77519  2.73123  3.46354  2.40513  3.72494
3.29354  2.67741  2.69355  4.24690  2.90347  2.73739  3.18146  2.89801
2.37887  2.77519  2.98518  4.58477  3.61503
         0.02625  4.04896  4.77131  0.61958  0.77255  0.48576  0.95510
    615  3.17706  4.50539  4.80452  4.25299  2.08173  4.41383  4.60762
1.52431  4.12186  1.44099  2.99809  4.43153  4.64877  4.23168  4.22978
3.75327  3.40421  2.14330  4.93387  3.65836    720 l - - -
         2.68618  4.42225  2.77519  2.73123  3.46354  2.40513  3.72494
3.29354  2.67741  2.69355  4.24690  2.90347  2.73739  3.18146  2.89801
2.37887  2.77519  2.98518  4.58477  3.61503
         0.02625  4.04896  4.77131  0.61958  0.77255  0.48576  0.95510
    616  2.61989  4.88844  2.81902  2.38653  4.25267  2.29233  3.65097
3.68939  2.42056  3.25382  4.05499  2.55322  3.29091  2.61964  2.66656
2.44956  2.87790  3.31783  5.45444  4.10222    721 g - - -
         2.68618  4.42225  2.77519  2.73123  3.46354  2.40513  3.72494
3.29354  2.67741  2.69355  4.24690  2.90347  2.73739  3.18146  2.89801
2.37887  2.77519  2.98518  4.58477  3.61503
         0.02625  4.04896  4.77131  0.61958  0.77255  0.48576  0.95510
    617  2.50279  4.61451  2.91494  2.55793  4.24487  3.21032  3.81508
3.67201  2.65683  3.28543  4.10297  1.87829  3.22251  2.99120  3.08767
2.34554  2.22254  3.25819  5.50916  4.19139    722 n - - -
         2.68618  4.42225  2.77519  2.73123  3.46354  2.40513  3.72494
3.29354  2.67741  2.69355  4.24690  2.90347  2.73739  3.18146  2.89801
2.37887  2.77519  2.98518  4.58477  3.61503
         0.02625  4.04896  4.77131  0.61958  0.77255  0.48576  0.95510
    618  2.59603  4.27295  3.65715  3.11723  3.51122  3.57149  4.00838
2.23184  3.04774  2.46414  3.44768  3.46421  2.62267  3.35641  3.37069
2.87266  2.43852  1.91238  5.02703  3.80258    723 v - - -
         2.68618  4.42225  2.77519  2.73123  3.46354  2.40513  3.72494
3.29354  2.67741  2.69355  4.24690  2.90347  2.73739  3.18146  2.89801
2.37887  2.77519  2.98518  4.58477  3.61503
         0.02625  4.04896  4.77131  0.61958  0.77255  0.48576  0.95510
    619  3.01472  4.38457  4.60191  4.05906  2.00595  4.22280  4.49922
1.93160  3.93951  1.86550  3.15131  4.24342  4.52856  4.11522  4.09204
3.55843  3.25749  1.46800  4.94322  3.64161    724 v - - -
         2.68618  4.42225  2.77519  2.73123  3.46354  2.40513  3.72494
3.29354  2.67741  2.69355  4.24690  2.90347  2.73739  3.18146  2.89801
2.37887  2.77519  2.98518  4.58477  3.61503
         0.02625  4.04896  4.77131  0.61958  0.77255  0.48576  0.95510
```

```
    620   1.62387  4.18824  3.63480  3.17595  3.84685  3.17971  4.11126
3.01199  3.12944  2.88423  3.78455  3.39272  3.81920  3.42724  3.44390
2.18488  2.48654  2.03374  5.28457  4.06966    725 a - - -
          2.68618  4.42225  2.77519  2.73123  3.46354  2.40513  3.72494
3.29354  2.67741  2.69355  4.24690  2.90347  2.73739  3.18146  2.89801
2.37887  2.77519  2.98518  4.58477  3.61503
          0.02625  4.04896  4.77131  0.61958  0.77255  0.48576  0.95510
    621   2.66063  4.26505  3.78885  3.23705  3.40852  3.68823  4.06073
2.10341  3.15077  2.06071  3.35640  3.57065  4.09369  3.44951  3.44839
2.09204  2.91837  1.99925  4.97322  3.74942    726 v - - -
          2.68618  4.42225  2.77519  2.73123  3.46354  2.40513  3.72494
3.29354  2.67741  2.69355  4.24690  2.90347  2.73739  3.18146  2.89801
2.37887  2.77519  2.98518  4.58477  3.61503
          0.02625  4.04896  4.77131  0.61958  0.77255  0.48576  0.95510
    622   2.50143  5.02671  2.38368  2.29284  4.36420  3.32709  3.63625
3.82261  2.32083  3.35995  4.14602  2.44958  3.30003  2.76629  2.93928
2.06604  2.90225  3.42916  5.54248  4.15052    727 s - - -
          2.68618  4.42225  2.77519  2.73123  3.46354  2.40513  3.72494
3.29354  2.67741  2.69355  4.24690  2.90347  2.73739  3.18146  2.89801
2.37887  2.77519  2.98518  4.58477  3.61503
          0.02625  4.04896  4.77131  0.61958  0.77255  0.48576  0.95510
    623   2.28315  5.00558  2.55876  2.02535  4.40843  2.71280  3.71680
3.85765  2.57291  3.41909  4.23191  1.96372  3.81673  2.86921  3.06134
2.68482  2.97811  3.46564  5.61748  4.23084    728 n - - -
          2.68618  4.42225  2.77519  2.73123  3.46354  2.40513  3.72494
3.29354  2.67741  2.69355  4.24690  2.90347  2.73739  3.18146  2.89801
2.37887  2.77519  2.98518  4.58477  3.61503
          0.02625  4.04896  4.77131  0.61958  0.77255  0.48576  0.95510
    624   2.57419  4.69295  2.96254  2.52850  4.12964  3.31999  3.72894
3.53882  2.50601  3.15174  3.98151  3.00511  3.34213  2.15381  2.91344
2.49755  2.00666  3.18501  5.38940  4.07851    729 t - - -
          2.68618  4.42225  2.77519  2.73123  3.46354  2.40513  3.72494
3.29354  2.67741  2.69355  4.24690  2.90347  2.73739  3.18146  2.89801
2.37887  2.77519  2.98518  4.58477  3.61503
          0.02625  4.04896  4.77131  0.61958  0.77255  0.48576  0.95510
    625   2.36778  5.01778  2.31355  2.29735  4.34953  3.33218  3.63074
3.80537  2.28687  3.34486  4.13046  2.38831  3.78969  2.76000  2.92714
2.32094  2.69186  3.41468  5.52873  4.13919    730 k - - -
          2.68618  4.42225  2.77519  2.73123  3.46354  2.40513  3.72494
3.29354  2.67741  2.69355  4.24690  2.90347  2.73739  3.18146  2.89801
2.37887  2.77519  2.98518  4.58477  3.61503
          0.02625  4.04896  4.77131  0.61958  0.77255  0.48576  0.95510
    626   2.63761  4.69679  3.08599  2.39349  3.92652  3.46180  3.66169
3.30913  2.40459  2.94373  3.79137  3.03642  3.41987  2.67690  2.33883
2.70838  2.86706  2.21773  5.21399  3.92491    731 v - - -
          2.68618  4.42225  2.77519  2.73123  3.46354  2.40513  3.72494
3.29354  2.67741  2.69355  4.24690  2.90347  2.73739  3.18146  2.89801
2.37887  2.77519  2.98518  4.58477  3.61503
          0.02625  4.04896  4.77131  0.61958  0.77255  0.48576  0.95510
    627   2.59832  4.47193  3.23776  2.43220  3.64714  3.50187  3.75701
2.55418  2.65599  2.37147  3.56743  3.15913  2.65774  3.00317  3.04830
2.74932  2.69395  2.69076  5.05630  3.79232    732 l - - -
          2.68618  4.42225  2.77519  2.73123  3.46354  2.40513  3.72494
3.29354  2.67741  2.69355  4.24690  2.90347  2.73739  3.18146  2.89801
2.37887  2.77519  2.98518  4.58477  3.61503
```

```
         0.16823  4.04896  1.98485  0.61958  0.77255  0.48576  0.95510
    628  2.72401  4.44627  3.42311  3.04866  3.34010  3.54346  3.94778
2.71044  2.89267  1.43020  3.52065  2.85104  4.03446  3.32549  3.16981
2.94519  3.01742  2.55716  4.92579  3.55905    733 l - - -
         2.68618  4.42225  2.77519  2.73123  3.46354  2.40513  3.72494
3.29354  2.67741  2.69355  4.24690  2.90347  2.73739  3.18146  2.89801
2.37887  2.77519  2.98518  4.58477  3.61503
         0.03020  3.91092  4.63327  0.61958  0.77255  0.61719  0.77535
    629  2.70989  5.02355  1.80053  2.25631  4.23267  3.29672  3.64964
3.70499  2.49474  2.86593  4.12813  2.80452  3.79875  2.40837  2.97121
2.68753  2.96542  3.36469  5.48759  4.08572    734 d - - -
         2.68618  4.42225  2.77519  2.73123  3.46354  2.40513  3.72494
3.29354  2.67741  2.69355  4.24690  2.90347  2.73739  3.18146  2.89801
2.37887  2.77519  2.98518  4.58477  3.61503
         0.09087  3.91092  2.70549  0.61958  0.77255  0.61719  0.77535
    630  3.08756  4.45864  4.52409  4.01975  3.22601  4.30501  4.69486
1.33434  3.83847  1.52090  3.09506  4.29346  4.59890  4.12219  4.03698
3.68111  3.34060  1.82857  5.20091  3.98165    735 i - - -
         2.68619  4.42226  2.77521  2.73124  3.46355  2.40514  3.72496
3.29355  2.67742  2.69356  4.24691  2.90348  2.73741  3.18118  2.89802
2.37888  2.77521  2.98519  4.58478  3.61504
         0.13458  2.15757  4.57446  0.39046  1.12931  0.38872  1.13296
    631  2.59281  4.52622  3.16625  2.65099  3.74376  3.45610  3.73876
3.06173  2.59768  2.61886  3.66073  2.34641  3.87527  2.77677  2.99136
2.71842  2.62546  2.22540  5.11909  3.84147    737 v - - -
         2.68618  4.42225  2.77519  2.73123  3.46354  2.40513  3.72494
3.29354  2.67741  2.69355  4.24690  2.90347  2.73739  3.18146  2.89801
2.37887  2.77519  2.98518  4.58477  3.61503
         0.02625  4.04896  4.77131  0.61958  0.77255  0.48576  0.95510
    632  2.74622  4.24232  4.08387  3.52930  3.37150  3.85664  4.23926
2.01984  3.42075  1.86522  3.30360  3.80574  4.23602  3.69554  3.66561
2.66487  2.99849  1.54837  5.00337  3.79399    738 v - - -
         2.68618  4.42225  2.77519  2.73123  3.46354  2.40513  3.72494
3.29354  2.67741  2.69355  4.24690  2.90347  2.73739  3.18146  2.89801
2.37887  2.77519  2.98518  4.58477  3.61503
         0.02625  4.04896  4.77131  0.61958  0.77255  0.48576  0.95510
    633  2.57034  4.50660  3.15531  2.48265  3.70684  2.96143  3.71378
2.86520  2.60401  2.76191  3.62505  3.09317  3.85627  2.94235  3.01588
2.55702  2.13286  2.81858  5.08035  3.36204    739 t - - -
         2.68618  4.42225  2.77519  2.73123  3.46354  2.40513  3.72494
3.29354  2.67741  2.69355  4.24690  2.90347  2.73739  3.18146  2.89801
2.37887  2.77519  2.98518  4.58477  3.61503
         0.02625  4.04896  4.77131  0.61958  0.77255  0.48576  0.95510
    634  2.24278  4.28665  4.20920  3.64542  2.26818  3.93915  4.22976
2.16239  3.53702  1.60290  3.11547  3.89428  4.27967  3.75536  3.74140
3.24196  3.06043  2.21118  4.84408  3.60978    740 l - - -
         2.68618  4.42225  2.77519  2.73123  3.46354  2.40513  3.72494
3.29354  2.67741  2.69355  4.24690  2.90347  2.73739  3.18146  2.89801
2.37887  2.77519  2.98518  4.58477  3.61503
         0.02625  4.04896  4.77131  0.61958  0.77255  0.48576  0.95510
    635  2.64577  4.89278  2.68717  2.20397  4.30679  3.29978  3.71066
3.74242  2.53664  3.32177  4.13589  2.01596  3.81104  2.86534  2.99997
2.15143  2.51409  3.36278  5.53094  4.17010    741 n - - -
```

```
        2.68618  4.42225  2.77519  2.73123  3.46354  2.40513  3.72494
3.29354  2.67741  2.69355  4.24690  2.90347  2.73739  3.18146  2.89801
2.37887  2.77519  2.98518  4.58477  3.61503
        0.02625  4.04896  4.77131  0.61958  0.77255  0.48576  0.95510
    636  2.06097  4.22741  3.41087  3.04508  4.27505  2.34001  4.13450
3.68642  3.08369  3.35770  4.17404  3.26629  3.71388  3.37768  3.43580
1.54072  2.05002  3.16805  5.60355  4.37543    742 s - - -
        2.68618  4.42225  2.77519  2.73123  3.46354  2.40513  3.72494
3.29354  2.67741  2.69355  4.24690  2.90347  2.73739  3.18146  2.89801
2.37887  2.77519  2.98518  4.58477  3.61503
        0.02625  4.04896  4.77131  0.61958  0.77255  0.48576  0.95510
    637  2.41798  4.44916  3.15962  2.69855  4.08358  2.19188  3.85062
3.49504  2.72106  3.13610  3.96074  2.81815  3.26779  3.04768  3.13802
2.04944  2.53883  2.75433  5.39474  4.11105    743 s - - -
        2.68618  4.42225  2.77519  2.73123  3.46354  2.40513  3.72494
3.29354  2.67741  2.69355  4.24690  2.90347  2.73739  3.18146  2.89801
2.37887  2.77519  2.98518  4.58477  3.61503
        0.02625  4.04896  4.77131  0.61958  0.77255  0.48576  0.95510
    638  2.31125  4.50034  3.14198  2.65289  4.07681  2.86774  3.80340
3.48904  2.64287  3.12035  3.94284  3.07672  3.30585  2.98965  2.80047
1.96955  2.16118  3.10670  5.37384  4.08341    744 s - - -
        2.68618  4.42225  2.77519  2.73123  3.46354  2.40513  3.72494
3.29354  2.67741  2.69355  4.24690  2.90347  2.73739  3.18146  2.89801
2.37887  2.77519  2.98518  4.58477  3.61503
        0.02625  4.04896  4.77131  0.61958  0.77255  0.48576  0.95510
    639  2.66117  4.94204  2.73775  2.37223  4.32187  3.33387  3.67321
3.77108  2.44137  3.32635  4.13280  2.49768  2.58165  2.10432  2.88178
2.45692  2.92597  3.39087  5.51204  4.15199    745 q - - -
        2.68618  4.42225  2.77519  2.73123  3.46354  2.40513  3.72494
3.29354  2.67741  2.69355  4.24690  2.90347  2.73739  3.18146  2.89801
2.37887  2.77519  2.98518  4.58477  3.61503
        0.02625  4.04896  4.77131  0.61958  0.77255  0.48576  0.95510
    640  3.24597  4.58366  4.77700  4.23573  1.71370  4.43033  4.48883
2.06135  4.10030  1.25308  2.96573  4.40811  4.65556  4.19310  4.20733
3.76869  3.46880  2.34121  4.77237  3.41000    746 l - - -
        2.68618  4.42225  2.77519  2.73123  3.46354  2.40513  3.72494
3.29354  2.67741  2.69355  4.24690  2.90347  2.73739  3.18146  2.89801
2.37887  2.77519  2.98518  4.58477  3.61503
        0.02625  4.04896  4.77131  0.61958  0.77255  0.48576  0.95510
    641  3.02734  5.59082  1.10419  1.81127  4.87799  3.24443  3.83118
4.40382  2.89065  3.91508  4.79272  2.71509  3.88889  3.00815  3.50288
2.90128  3.32688  3.97965  6.06048  4.54993    747 d - - -
        2.68618  4.42225  2.77519  2.73123  3.46354  2.40513  3.72494
3.29354  2.67741  2.69355  4.24690  2.90347  2.73739  3.18146  2.89801
2.37887  2.77519  2.98518  4.58477  3.61503
        0.02625  4.04896  4.77131  0.61958  0.77255  0.48576  0.95510
    642  3.24013  4.55855  4.86087  4.31571  3.24035  4.54131  4.92120
1.65355  4.18197  1.08470  3.03539  4.56642  4.76089  4.34468  4.33094
3.89924  3.47617  1.81436  5.29238  4.18457    748 l - - -
        2.68618  4.42225  2.77519  2.73123  3.46354  2.40513  3.72494
3.29354  2.67741  2.69355  4.24690  2.90347  2.73739  3.18146  2.89801
2.37887  2.77519  2.98518  4.58477  3.61503
        0.02625  4.04896  4.77131  0.61958  0.77255  0.48576  0.95510
```

```
    643   3.11652  4.63928  4.24324  3.85342  3.29692  3.96808  4.54782
2.49068  3.61082  1.86793  1.07604  4.10809  4.43260  3.98278  3.79473
3.48249  3.42685  2.51659  5.15930  3.93501    749 m - - -
          2.68618  4.42225  2.77519  2.73123  3.46354  2.40513  3.72494
3.29354  2.67741  2.69355  4.24690  2.90347  2.73739  3.18146  2.89801
2.37887  2.77519  2.98518  4.58477  3.61503
          0.02625  4.04896  4.77131  0.61958  0.77255  0.48576  0.95510
    644   2.87931  4.87574  2.82111  2.72621  4.26801  3.31004  4.07758
4.02015  2.99819  3.64783  4.60465  0.87973  3.97208  3.35350  3.35640
2.94155  3.26875  3.62429  5.55383  4.20588    750 n - - -
          2.68618  4.42225  2.77519  2.73123  3.46354  2.40513  3.72494
3.29354  2.67741  2.69355  4.24690  2.90347  2.73739  3.18146  2.89801
2.37887  2.77519  2.98518  4.58477  3.61503
          0.02625  4.04896  4.77131  0.61958  0.77255  0.48576  0.95510
    645   3.03336  4.41498  4.54868  4.02395  3.41933  4.26597  4.71071
1.21948  3.87158  1.72246  3.25989  4.27529  4.58918  4.14877  4.07494
3.61923  2.91702  1.81233  5.30105  4.11740    751 i - - -
          2.68618  4.42225  2.77519  2.73123  3.46354  2.40513  3.72494
3.29354  2.67741  2.69355  4.24690  2.90347  2.73739  3.18146  2.89801
2.37887  2.77519  2.98518  4.58477  3.61503
          0.02625  4.04896  4.77131  0.61958  0.77255  0.48576  0.95510
    646   3.15416  4.50543  4.72089  4.18144  3.25228  4.40157  4.78743
1.31500  4.02286  1.70067  2.07416  4.42437  4.66602  4.23128  4.18353
3.75702  3.39580  1.97859  5.23417  4.09940    752 i - - -
          2.68618  4.42225  2.77519  2.73123  3.46354  2.40513  3.72494
3.29354  2.67741  2.69355  4.24690  2.90347  2.73739  3.18146  2.89801
2.37887  2.77519  2.98518  4.58477  3.61503
          0.02625  4.04896  4.77131  0.61958  0.77255  0.48576  0.95510
    647   3.34085  4.68106  4.74851  4.25658  1.77058  4.44635  4.36902
2.37894  4.10931  1.07166  3.01210  4.39975  4.68732  4.20334  4.21392
3.81310  3.56958  2.49513  4.60813  3.14086    753 l - - -
          2.68618  4.42225  2.77519  2.73123  3.46354  2.40513  3.72494
3.29354  2.67741  2.69355  4.24690  2.90347  2.73739  3.18146  2.89801
2.37887  2.77519  2.98518  4.58477  3.61503
          0.02625  4.04896  4.77131  0.61958  0.77255  0.48576  0.95510
    648   3.14749  4.45071  4.85103  4.31660  3.42636  4.50574  4.95718
1.53703  4.20462  1.55733  3.22795  4.54533  4.76080  4.41097  4.37597
3.86661  3.39263  1.28684  5.40581  4.25011    754 v - - -
          2.68618  4.42225  2.77519  2.73123  3.46354  2.40513  3.72494
3.29354  2.67741  2.69355  4.24690  2.90347  2.73739  3.18146  2.89801
2.37887  2.77519  2.98518  4.58477  3.61503
          0.02625  4.04896  4.77131  0.61958  0.77255  0.48576  0.95510
    649   2.41195  4.36559  3.19859  2.99601  4.28216  3.07221  4.16737
3.75850  3.09022  3.43920  4.31974  3.27071  1.22270  3.43757  3.40603
2.22063  2.87575  3.27409  5.61780  4.35129    755 p - - -
          2.68618  4.42225  2.77519  2.73123  3.46354  2.40513  3.72494
3.29354  2.67741  2.69355  4.24690  2.90347  2.73739  3.18146  2.89801
2.37887  2.77519  2.98518  4.58477  3.61503
          0.02625  4.04896  4.77131  0.61958  0.77255  0.48576  0.95510
    650   2.43340  4.21601  3.72705  3.16615  3.36831  3.64346  3.97453
2.22162  3.08891  2.36647  3.33383  3.50286  4.03678  3.37754  3.38601
2.92292  2.09399  2.04146  4.88554  3.26181    756 v - - -
          2.68618  4.42225  2.77519  2.73123  3.46354  2.40513  3.72494
3.29354  2.67741  2.69355  4.24690  2.90347  2.73739  3.18146  2.89801
2.37887  2.77519  2.98518  4.58477  3.61503
```

```
          0.02625  4.04896  4.77131  0.61958  0.77255  0.48576  0.95510
    651   2.76313  5.04903  2.33738  2.31277  4.12649  3.33584  3.70463
3.78147  2.59776  3.35439  4.19731  1.58717  3.85221  2.88922  3.08257
2.74425  3.02673  3.43035  5.43955  3.36071    757 n - - -
          2.68618  4.42225  2.77519  2.73123  3.46354  2.40513  3.72494
3.29354  2.67741  2.69355  4.24690  2.90347  2.73739  3.18146  2.89801
2.37887  2.77519  2.98518  4.58477  3.61503
          0.02625  4.04896  4.77131  0.61958  0.77255  0.48576  0.95510
    652   2.71787  4.60095  2.07796  2.73212  3.69865  3.58217  3.87456
2.16542  2.78076  2.31639  3.62516  3.21360  3.99809  3.10562  3.19755
2.87060  2.96359  2.52423  5.20620  3.89953    758 d - - -
          2.68618  4.42225  2.77519  2.73123  3.46354  2.40513  3.72494
3.29354  2.67741  2.69355  4.24690  2.90347  2.73739  3.18146  2.89801
2.37887  2.77519  2.98518  4.58477  3.61503
          0.02625  4.04896  4.77131  0.61958  0.77255  0.48576  0.95510
    653   2.39152  4.32229  3.35833  2.87613  3.92689  3.19925  3.93214
3.27272  2.85180  2.98459  3.84209  3.21614  2.66601  3.17658  3.22768
1.95700  2.15427  2.45823  5.29806  4.04737    759 s - - -
          2.68618  4.42225  2.77519  2.73123  3.46354  2.40513  3.72494
3.29354  2.67741  2.69355  4.24690  2.90347  2.73739  3.18146  2.89801
2.37887  2.77519  2.98518  4.58477  3.61503
          0.02625  4.04896  4.77131  0.61958  0.77255  0.48576  0.95510
    654   3.00224  4.72657  3.62906  3.49603  4.47053  3.40426  4.55621
4.06484  3.56912  3.67110  4.73068  3.78339  0.55633  3.95247  3.79183
3.17599  3.45834  3.68948  5.60532  4.59466    760 P - - -
          2.68618  4.42225  2.77519  2.73123  3.46354  2.40513  3.72494
3.29354  2.67741  2.69355  4.24690  2.90347  2.73739  3.18146  2.89801
2.37887  2.77519  2.98518  4.58477  3.61503
          0.02625  4.04896  4.77131  0.61958  0.77255  0.48576  0.95510
    655   3.32555  4.68608  4.75213  4.24450  3.12147  4.50508  4.83062
1.98176  4.03217  0.83906  2.97168  4.51459  4.74564  4.25300  4.18370
3.89424  3.56807  2.21940  5.19016  4.02237    761 l - - -
          2.68618  4.42225  2.77519  2.73123  3.46354  2.40513  3.72494
3.29354  2.67741  2.69355  4.24690  2.90347  2.73739  3.18146  2.89801
2.37887  2.77519  2.98518  4.58477  3.61503
          0.02625  4.04896  4.77131  0.61958  0.77255  0.48576  0.95510
    656   3.51130  4.92842  4.10319  3.86868  2.32121  4.03918  3.71497
3.49696  3.72133  2.90449  4.15421  3.94722  4.51032  3.98439  3.86748
3.62915  3.79964  3.37626  3.97068  0.71613    762 y - - -
          2.68618  4.42225  2.77519  2.73123  3.46354  2.40513  3.72494
3.29354  2.67741  2.69355  4.24690  2.90347  2.73739  3.18146  2.89801
2.37887  2.77519  2.98518  4.58477  3.61503
          0.01775  4.04045        *  0.61958  0.77255  0.00000        *
//
HMMER3/f [3.3 | Nov 2019]
NAME  CryM3
DESC  Cry toxin (IDOPS Model 3)
LENG  650
ALPH  amino
RF    no
MM    no
CONS  yes
CS    no
MAP   yes
DATE  Wed Feb  3 11:08:33 2021
```

```
NSEQ  8
EFFN  0.503906
CKSUM 2020791261
GA 1000.0 1000.0
STATS LOCAL MSV       -12.0417  0.69692
STATS LOCAL VITERBI  -13.1430  0.69692
STATS LOCAL FORWARD   -6.0221  0.69692
HMM          A        C        D        E        F        G        H
I        K        L        M        N        P        Q        R        S
T        V        W        Y
            m->m     m->i     m->d     i->m     i->i     d->m     d->d
  COMPO   2.51014  4.36044  2.95787  2.79533  3.11341  2.85606  3.88486
2.76391  2.79300  2.41080  3.71284  2.94838  3.36672  3.18945  3.01198
2.62128  2.77547  2.64696  4.45813  3.29091
          2.68618  4.42225  2.77519  2.73123  3.46354  2.40513  3.72494
3.29354  2.67741  2.69355  4.24690  2.90347  2.73739  3.18146  2.89801
2.37887  2.77519  2.98518  4.58477  3.61503
          0.03133  3.87472  4.59707  0.61958  0.77255  0.00000        *
      1   2.97087  4.51925  4.05178  3.64534  3.20855  3.83573  4.37996
2.38437  3.41332  1.80283  1.37362  3.92258  4.29629  3.79501  3.61839
3.32024  3.27619  2.39076  5.04971  3.81172       1 m - - -
          2.68618  4.42225  2.77519  2.73123  3.46354  2.40513  3.72494
3.29354  2.67741  2.69355  4.24690  2.90347  2.73739  3.18146  2.89801
2.37887  2.77519  2.98518  4.58477  3.61503
          0.03133  3.87472  4.59707  0.61958  0.77255  0.48576  0.95510
      2   2.82196  5.16163  2.40865  1.51281  4.44148  3.27942  3.66488
3.90731  2.43478  3.43785  4.30456  2.78266  3.82167  2.31290  2.84834
2.77145  3.08103  3.55099  5.60666  4.22452       2 e - - -
          2.68618  4.42225  2.77519  2.73123  3.46354  2.40513  3.72494
3.29354  2.67741  2.69355  4.24690  2.90347  2.73739  3.18146  2.89801
2.37887  2.77519  2.98518  4.58477  3.61503
          0.03133  3.87472  4.59707  0.61958  0.77255  0.48576  0.95510
      3   2.81676  5.23993  1.63562  2.11412  4.56066  3.16811  3.72343
4.10783  2.71559  3.65084  4.50633  1.84907  3.78432  2.89965  3.27130
2.74285  3.11960  3.69368  5.78369  4.31283       3 d - - -
          2.68618  4.42225  2.77519  2.73123  3.46354  2.40513  3.72494
3.29354  2.67741  2.69355  4.24690  2.90347  2.73739  3.18146  2.89801
2.37887  2.77519  2.98518  4.58477  3.61503
          0.03133  3.87472  4.59707  0.61958  0.77255  0.48576  0.95510
      4   2.45727  4.52953  2.72571  2.52285  4.20658  3.07664  3.84363
3.72669  2.72480  3.35743  4.20440  1.94532  3.72419  3.05619  3.12334
1.81642  2.84850  3.28006  5.50792  4.15835       4 s - - -
          2.68618  4.42225  2.77519  2.73123  3.46354  2.40513  3.72494
3.29354  2.67741  2.69355  4.24690  2.90347  2.73739  3.18146  2.89801
2.37887  2.77519  2.98518  4.58477  3.61503
          0.03133  3.87472  4.59707  0.61958  0.77255  0.48576  0.95510
      5   2.40464  4.44113  2.81463  2.60494  4.17808  3.04435  3.88728
3.69575  2.77734  3.34066  4.18928  2.33328  3.71054  3.11272  3.15779
1.53380  2.81519  3.23748  5.49445  4.16011       5 s - - -
          2.68618  4.42225  2.77519  2.73123  3.46354  2.40513  3.72494
3.29354  2.67741  2.69355  4.24690  2.90347  2.73739  3.18146  2.89801
2.37887  2.77519  2.98518  4.58477  3.61503
          0.03133  3.87472  4.59707  0.61958  0.77255  0.48576  0.95510
```

```
      6   3.18394  4.57158  4.46817  3.98536  1.81739  4.24176  4.17394
2.32886  3.84356  1.26758  3.01821  4.16005  4.52633  3.99840  3.99490
3.60398  3.42132  2.39767  4.47188  2.96978      6 l - - -
          2.68618  4.42225  2.77519  2.73123  3.46354  2.40513  3.72494
3.29354  2.67741  2.69355  4.24690  2.90347  2.73739  3.18146  2.89801
2.37887  2.77519  2.98518  4.58477  3.61503
          0.03133  3.87472  4.59707  0.61958  0.77255  0.48576  0.95510
      7   2.81676  5.23993  1.63562  2.11412  4.56066  3.16811  3.72343
4.10783  2.71559  3.65084  4.50633  1.84907  3.78432  2.89965  3.27130
2.74285  3.11960  3.69368  5.78369  4.31283      7 d - - -
          2.68618  4.42225  2.77519  2.73123  3.46354  2.40513  3.72494
3.29354  2.67741  2.69355  4.24690  2.90347  2.73739  3.18146  2.89801
2.37887  2.77519  2.98518  4.58477  3.61503
          0.03133  3.87472  4.59707  0.61958  0.77255  0.48576  0.95510
      8   2.43707  4.20945  3.65786  3.24839  3.72570  3.27746  4.18272
2.53455  3.14583  2.57524  3.65401  3.47285  3.90201  3.50093  3.43122
2.69163  1.68906  1.94360  5.29549  4.05906      8 t - - -
          2.68618  4.42225  2.77519  2.73123  3.46354  2.40513  3.72494
3.29354  2.67741  2.69355  4.24690  2.90347  2.73739  3.18146  2.89801
2.37887  2.77519  2.98518  4.58477  3.61503
          0.03133  3.87472  4.59707  0.61958  0.77255  0.48576  0.95510
      9   2.73394  4.37043  3.81025  3.36443  3.36202  3.65896  4.20877
2.34952  3.18139  1.40355  3.29790  3.66336  4.14199  3.56632  3.43764
3.04621  2.42368  2.25038  5.09470  3.85415      9 l - - -
          2.68618  4.42225  2.77519  2.73123  3.46354  2.40513  3.72494
3.29354  2.67741  2.69355  4.24690  2.90347  2.73739  3.18146  2.89801
2.37887  2.77519  2.98518  4.58477  3.61503
          0.03133  3.87472  4.59707  0.61958  0.77255  0.48576  0.95510
     10   2.14745  4.62524  2.71561  2.09018  4.15775  3.18152  3.74998
3.54129  2.60900  3.19209  4.03677  2.91729  3.75099  2.93774  3.03713
1.94761  2.85005  3.16999  5.44199  4.11915     10 s - - -
          2.68618  4.42225  2.77519  2.73123  3.46354  2.40513  3.72494
3.29354  2.67741  2.69355  4.24690  2.90347  2.73739  3.18146  2.89801
2.37887  2.77519  2.98518  4.58477  3.61503
          0.03133  3.87472  4.59707  0.61958  0.77255  0.48576  0.95510
     11   2.65123  4.35780  3.42140  3.02541  3.48130  3.52911  3.98183
1.67026  2.93710  2.47952  3.53579  2.65528  4.01436  3.32573  3.24081
2.90273  2.94328  2.29417  5.03524  3.72097     11 i - - -
          2.68618  4.42225  2.77519  2.73123  3.46354  2.40513  3.72494
3.29354  2.67741  2.69355  4.24690  2.90347  2.73739  3.18146  2.89801
2.37887  2.77519  2.98518  4.58477  3.61503
          0.03133  3.87472  4.59707  0.61958  0.77255  0.48576  0.95510
     12   2.52571  4.40844  3.13469  2.78903  3.74288  3.29253  3.89196
2.90161  2.78578  2.81162  3.78164  2.11939  3.85481  3.16273  3.13399
2.68999  2.86420  1.94017  5.19670  3.87228     12 v - - -
          2.68618  4.42225  2.77519  2.73123  3.46354  2.40513  3.72494
3.29354  2.67741  2.69355  4.24690  2.90347  2.73739  3.18146  2.89801
2.37887  2.77519  2.98518  4.58477  3.61503
          0.03133  3.87472  4.59707  0.61958  0.77255  0.48576  0.95510
     13   2.72515  4.72193  2.70930  2.59495  4.08579  3.18536  3.92257
3.79656  2.82640  3.44315  4.39831  1.15407  3.83729  3.19525  3.18120
2.79220  3.10703  3.41854  5.39143  4.03390     13 n - - -
          2.68618  4.42225  2.77519  2.73123  3.46354  2.40513  3.72494
3.29354  2.67741  2.69355  4.24690  2.90347  2.73739  3.18146  2.89801
2.37887  2.77519  2.98518  4.58477  3.61503
```

```
        0.03133  3.87472  4.59707  0.61958  0.77255  0.48576  0.95510
     14  2.67699  4.60369  3.03457  2.03445  3.70937  3.46298  3.76544
2.95762  2.58298  2.60492  2.40775  3.10507  3.90504  2.99016  2.94948
2.79545  2.92666  2.76245  5.15762  3.87279     14 e - - -
        2.68618  4.42225  2.77519  2.73123  3.46354  2.40513  3.72494
3.29354  2.67741  2.69355  4.24690  2.90347  2.73739  3.18146  2.89801
2.37887  2.77519  2.98518  4.58477  3.61503
        0.03133  3.87472  4.59707  0.61958  0.77255  0.48576  0.95510
     15  2.42819  4.22965  3.44727  2.97408  3.64617  3.28913  3.93976
2.31685  2.90033  2.64825  3.59459  3.29513  3.83947  3.24325  3.22824
2.24349  2.05877  2.50387  5.10923  3.86736     15 t - - -
        2.68618  4.42225  2.77519  2.73123  3.46354  2.40513  3.72494
3.29354  2.67741  2.69355  4.24690  2.90347  2.73739  3.18146  2.89801
2.37887  2.77519  2.98518  4.58477  3.61503
        0.03133  3.87472  4.59707  0.61958  0.77255  0.48576  0.95510
     16  2.72073  5.09855  1.74904  2.15445  4.49714  3.17423  3.69717
3.99493  2.64295  3.54018  4.36873  2.25013  3.76281  2.86142  3.17985
2.22701  3.02074  3.57811  5.71382  4.27318     16 d - - -
        2.68618  4.42225  2.77519  2.73123  3.46354  2.40513  3.72494
3.29354  2.67741  2.69355  4.24690  2.90347  2.73739  3.18146  2.89801
2.37887  2.77519  2.98518  4.58477  3.61503
        0.03133  3.87472  4.59707  0.61958  0.77255  0.48576  0.95510
     17  3.26109  4.66217  4.24182  3.95666  1.00804  3.95049  3.87804
2.78442  3.86362  2.15392  3.51419  4.02166  4.40896  4.03095  3.98279
3.53096  3.56220  2.77530  4.12630  2.49298     17 f - - -
        2.68618  4.42225  2.77519  2.73123  3.46354  2.40513  3.72494
3.29354  2.67741  2.69355  4.24690  2.90347  2.73739  3.18146  2.89801
2.37887  2.77519  2.98518  4.58477  3.61503
        0.03133  3.87472  4.59707  0.61958  0.77255  0.48576  0.95510
     18  2.75420  4.51129  3.36160  3.20088  4.19604  3.19867  4.27823
3.74193  3.25835  3.36857  4.41844  3.50740  0.82346  3.64706  3.50371
2.92174  3.19592  3.38706  5.38207  4.31670     18 p - - -
        2.68618  4.42225  2.77519  2.73123  3.46354  2.40513  3.72494
3.29354  2.67741  2.69355  4.24690  2.90347  2.73739  3.18146  2.89801
2.37887  2.77519  2.98518  4.58477  3.61503
        0.03133  3.87472  4.59707  0.61958  0.77255  0.48576  0.95510
     19  3.15227  4.57436  4.45355  3.91725  3.04025  4.28133  4.57378
2.16432  3.71140  1.04972  2.32726  4.22261  4.54238  3.96795  3.90108
3.63390  3.38864  2.25099  5.05399  3.89824     19 l - - -
        2.68618  4.42225  2.77519  2.73123  3.46354  2.40513  3.72494
3.29354  2.67741  2.69355  4.24690  2.90347  2.73739  3.18146  2.89801
2.37887  2.77519  2.98518  4.58477  3.61503
        0.03133  3.87472  4.59707  0.61958  0.77255  0.48576  0.95510
     20  3.37714  4.72141  4.30832  3.93856  1.62554  4.16789  3.44197
3.12517  3.80597  2.50855  3.74798  3.87666  4.50214  3.88759  3.92682
3.52620  3.60830  3.03819  3.63228  1.18511     20 y - - -
        2.68618  4.42225  2.77519  2.73123  3.46354  2.40513  3.72494
3.29354  2.67741  2.69355  4.24690  2.90347  2.73739  3.18146  2.89801
2.37887  2.77519  2.98518  4.58477  3.61503
        0.03133  3.87472  4.59707  0.61958  0.77255  0.48576  0.95510
     21  2.72515  4.72193  2.70930  2.59495  4.08579  3.18536  3.92257
3.79656  2.82640  3.44315  4.39831  1.15407  3.83729  3.19525  3.18120
2.79220  3.10703  3.41854  5.39143  4.03390     21 n - - -
```

```
         2.68618  4.42225  2.77519  2.73123  3.46354  2.40513  3.72494
3.29354  2.67741  2.69355  4.24690  2.90347  2.73739  3.18146  2.89801
2.37887  2.77519  2.98518  4.58477  3.61503
         0.03133  3.87472  4.59707  0.61958  0.77255  0.48576  0.95510
     22  2.42919  4.52067  2.70966  2.49433  4.29153  2.40162  3.83787
3.75959  2.73580  3.37079  4.19701  2.01111  3.70436  3.03498  3.16113
2.08217  2.81971  3.29394  5.56516  4.23794     22 n - - -
         2.68618  4.42225  2.77519  2.73123  3.46354  2.40513  3.72494
3.29354  2.67741  2.69355  4.24690  2.90347  2.73739  3.18146  2.89801
2.37887  2.77519  2.98518  4.58477  3.61503
         0.03133  3.87472  4.59707  0.61958  0.77255  0.48576  0.95510
     23  2.73009  4.72270  3.21278  2.64548  3.77095  3.51176  3.60527
3.34042  1.96320  2.94039  3.81962  3.09085  3.89779  2.81560  2.18042
2.80310  2.94227  3.06900  5.06490  2.53514     23 k - - -
         2.68618  4.42225  2.77519  2.73123  3.46354  2.40513  3.72494
3.29354  2.67741  2.69355  4.24690  2.90347  2.73739  3.18146  2.89801
2.37887  2.77519  2.98518  4.58477  3.61503
         0.03133  3.87472  4.59707  0.61958  0.77255  0.48576  0.95510
     24  2.74560  4.31321  3.90173  3.36986  3.38135  3.79340  4.20545
1.90114  3.23966  1.85037  3.27763  3.69976  4.19363  3.56991  3.52250
3.11331  2.19099  2.02586  5.07961  3.86206     24 l - - -
         2.68618  4.42225  2.77519  2.73123  3.46354  2.40513  3.72494
3.29354  2.67741  2.69355  4.24690  2.90347  2.73739  3.18146  2.89801
2.37887  2.77519  2.98518  4.58477  3.61503
         0.03133  3.87472  4.59707  0.61958  0.77255  0.48576  0.95510
     25  2.95406  5.06580  2.48830  1.02217  4.41227  3.27540  3.86227
3.86661  2.74307  3.48333  4.45717  2.93393  3.88006  3.07782  3.15222
2.93617  3.25828  3.55019  5.58341  4.30555     25 e - - -
         2.68618  4.42225  2.77519  2.73123  3.46354  2.40513  3.72494
3.29354  2.67741  2.69355  4.24690  2.90347  2.73739  3.18146  2.89801
2.37887  2.77519  2.98518  4.58477  3.61503
         0.03133  3.87472  4.59707  0.61958  0.77255  0.48576  0.95510
     26  2.75420  4.51129  3.36160  3.20088  4.19604  3.19867  4.27823
3.74193  3.25835  3.36857  4.41844  3.50740  0.82346  3.64706  3.50371
2.92174  3.19592  3.38706  5.38207  4.31670     26 p - - -
         2.68618  4.42225  2.77519  2.73123  3.46354  2.40513  3.72494
3.29354  2.67741  2.69355  4.24690  2.90347  2.73739  3.18146  2.89801
2.37887  2.77519  2.98518  4.58477  3.61503
         0.03133  3.87472  4.59707  0.61958  0.77255  0.48576  0.95510
     27  2.22657  4.16201  3.28998  2.98468  4.10060  2.95400  4.06371
3.43118  2.99359  3.18437  4.05455  3.20672  3.66653  3.33102  3.30912
1.65035  1.82092  2.98410  5.46782  4.22606     27 s - - -
         2.68618  4.42225  2.77519  2.73123  3.46354  2.40513  3.72494
3.29354  2.67741  2.69355  4.24690  2.90347  2.73739  3.18146  2.89801
2.37887  2.77519  2.98518  4.58477  3.61503
         0.03133  3.87472  4.59707  0.61958  0.77255  0.48576  0.95510
     28  3.10419  4.46755  4.56181  4.05612  3.23539  4.33261  4.72414
1.31201  3.87665  1.51353  3.09909  4.32619  4.62080  4.15359  4.07095
3.70916  3.35625  1.82976  5.21703  4.00327     28 i - - -
         2.68618  4.42225  2.77519  2.73123  3.46354  2.40513  3.72494
3.29354  2.67741  2.69355  4.24690  2.90347  2.73739  3.18146  2.89801
2.37887  2.77519  2.98518  4.58477  3.61503
         0.03133  3.87472  4.59707  0.61958  0.77255  0.48576  0.95510
```

29 1.01052 4.19303 3.46476 3.25606 4.05451 3.01753 4.27786
3.18035 3.27238 3.06187 4.09337 3.40514 3.75695 3.61331 3.52281
2.54327 2.82077 2.83436 5.48144 4.28192 29 a - - -
2.68618 4.42225 2.77519 2.73123 3.46354 2.40513 3.72494
3.29354 2.67741 2.69355 4.24690 2.90347 2.73739 3.18146 2.89801
2.37887 2.77519 2.98518 4.58477 3.61503
0.03133 3.87472 4.59707 0.61958 0.77255 0.48576 0.95510
30 2.75420 4.51129 3.36160 3.20088 4.19604 3.19867 4.27823
3.74193 3.25835 3.36857 4.41844 3.50740 0.82346 3.64706 3.50371
2.92174 3.19592 3.38706 5.38207 4.31670 30 p - - -
2.68618 4.42225 2.77519 2.73123 3.46354 2.40513 3.72494
3.29354 2.67741 2.69355 4.24690 2.90347 2.73739 3.18146 2.89801
2.37887 2.77519 2.98518 4.58477 3.61503
0.03133 3.87472 4.59707 0.61958 0.77255 0.48576 0.95510
31 1.01052 4.19303 3.46476 3.25606 4.05451 3.01753 4.27786
3.18035 3.27238 3.06187 4.09337 3.40514 3.75695 3.61331 3.52281
2.54327 2.82077 2.83436 5.48144 4.28192 31 a - - -
2.68618 4.42225 2.77519 2.73123 3.46354 2.40513 3.72494
3.29354 2.67741 2.69355 4.24690 2.90347 2.73739 3.18146 2.89801
2.37887 2.77519 2.98518 4.58477 3.61503
0.03133 3.87472 4.59707 0.61958 0.77255 0.48576 0.95510
32 3.15771 4.61926 4.19314 3.83291 3.13642 3.98506 4.48794
2.34049 3.60064 0.87242 3.15851 4.10818 4.41998 3.96843 3.77638
3.55606 3.45182 2.37843 5.01661 3.74048 32 l - - -
2.68618 4.42225 2.77519 2.73123 3.46354 2.40513 3.72494
3.29354 2.67741 2.69355 4.24690 2.90347 2.73739 3.18146 2.89801
2.37887 2.77519 2.98518 4.58477 3.61503
0.03133 3.87472 4.59707 0.61958 0.77255 0.48576 0.95510
33 3.03284 4.45083 4.33904 3.95485 3.37561 4.06909 4.68249
1.07512 3.78303 1.96109 3.31052 4.19987 4.50000 4.13104 3.97934
3.56453 3.33193 1.84577 5.26697 4.00552 33 i - - -
2.68618 4.42225 2.77519 2.73123 3.46354 2.40513 3.72494
3.29354 2.67741 2.69355 4.24690 2.90347 2.73739 3.18146 2.89801
2.37887 2.77519 2.98518 4.58477 3.61503
0.03133 3.87472 4.59707 0.61958 0.77255 0.48576 0.95510
34 1.01052 4.19303 3.46476 3.25606 4.05451 3.01753 4.27786
3.18035 3.27238 3.06187 4.09337 3.40514 3.75695 3.61331 3.52281
2.54327 2.82077 2.83436 5.48144 4.28192 34 a - - -
2.68618 4.42225 2.77519 2.73123 3.46354 2.40513 3.72494
3.29354 2.67741 2.69355 4.24690 2.90347 2.73739 3.18146 2.89801
2.37887 2.77519 2.98518 4.58477 3.61503
0.03133 3.87472 4.59707 0.61958 0.77255 0.48576 0.95510
35 2.80983 4.35172 4.12850 3.79161 3.55163 3.72629 4.59291
2.04988 3.65943 2.23820 3.51634 3.98111 4.28661 4.01903 3.87173
3.22990 3.17128 1.02205 5.33648 4.06827 35 v - - -
2.68618 4.42225 2.77519 2.73123 3.46354 2.40513 3.72494
3.29354 2.67741 2.69355 4.24690 2.90347 2.73739 3.18146 2.89801
2.37887 2.77519 2.98518 4.58477 3.61503
0.03133 3.87472 4.59707 0.61958 0.77255 0.48576 0.95510
36 1.01052 4.19303 3.46476 3.25606 4.05451 3.01753 4.27786
3.18035 3.27238 3.06187 4.09337 3.40514 3.75695 3.61331 3.52281
2.54327 2.82077 2.83436 5.48144 4.28192 36 a - - -
2.68618 4.42225 2.77519 2.73123 3.46354 2.40513 3.72494
3.29354 2.67741 2.69355 4.24690 2.90347 2.73739 3.18146 2.89801
2.37887 2.77519 2.98518 4.58477 3.61503

```
          0.03133  3.87472  4.59707  0.61958  0.77255  0.48576  0.95510
     37   2.75420  4.51129  3.36160  3.20088  4.19604  3.19867  4.27823
3.74193  3.25835  3.36857  4.41844  3.50740  0.82346  3.64706  3.50371
2.92174  3.19592  3.38706  5.38207  4.31670     37 p - - -
          2.68618  4.42225  2.77519  2.73123  3.46354  2.40513  3.72494
3.29354  2.67741  2.69355  4.24690  2.90347  2.73739  3.18146  2.89801
2.37887  2.77519  2.98518  4.58477  3.61503
          0.03133  3.87472  4.59707  0.61958  0.77255  0.48576  0.95510
     38   3.03284  4.45083  4.33904  3.95485  3.37561  4.06909  4.68249
1.07512  3.78303  1.96109  3.31052  4.19987  4.50000  4.13104  3.97934
3.56453  3.33193  1.84577  5.26697  4.00552     38 i - - -
          2.68618  4.42225  2.77519  2.73123  3.46354  2.40513  3.72494
3.29354  2.67741  2.69355  4.24690  2.90347  2.73739  3.18146  2.89801
2.37887  2.77519  2.98518  4.58477  3.61503
          0.03133  3.87472  4.59707  0.61958  0.77255  0.48576  0.95510
     39   1.01052  4.19303  3.46476  3.25606  4.05451  3.01753  4.27786
3.18035  3.27238  3.06187  4.09337  3.40514  3.75695  3.61331  3.52281
2.54327  2.82077  2.83436  5.48144  4.28192     39 a - - -
          2.68618  4.42225  2.77519  2.73123  3.46354  2.40513  3.72494
3.29354  2.67741  2.69355  4.24690  2.90347  2.73739  3.18146  2.89801
2.37887  2.77519  2.98518  4.58477  3.61503
          0.03133  3.87472  4.59707  0.61958  0.77255  0.48576  0.95510
     40   2.86996  5.05712  2.95165  2.52547  4.40412  3.46665  3.59634
3.81846  1.58986  3.31355  4.18129  2.99727  3.90252  2.01235  2.31441
2.86066  3.08071  3.49111  5.42333  4.16317     40 k - - -
          2.68618  4.42225  2.77519  2.73123  3.46354  2.40513  3.72494
3.29354  2.67741  2.69355  4.24690  2.90347  2.73739  3.18146  2.89801
2.37887  2.77519  2.98518  4.58477  3.61503
          0.03133  3.87472  4.59707  0.61958  0.77255  0.48576  0.95510
     41   3.25019  4.73827  3.84149  3.57122  2.29338  3.83270  3.61936
3.25908  3.42867  2.71244  3.93385  3.72612  4.30645  3.73417  3.61195
3.38669  3.53686  3.12972  3.93726  0.96205     41 y - - -
          2.68618  4.42225  2.77519  2.73123  3.46354  2.40513  3.72494
3.29354  2.67741  2.69355  4.24690  2.90347  2.73739  3.18146  2.89801
2.37887  2.77519  2.98518  4.58477  3.61503
          0.03133  3.87472  4.59707  0.61958  0.77255  0.48576  0.95510
     42   3.15771  4.61926  4.19314  3.83291  3.13642  3.98506  4.48794
2.34049  3.60064  0.87242  3.15851  4.10818  4.41998  3.96843  3.77638
3.55606  3.45182  2.37843  5.01661  3.74048     42 l - - -
          2.68618  4.42225  2.77519  2.73123  3.46354  2.40513  3.72494
3.29354  2.67741  2.69355  4.24690  2.90347  2.73739  3.18146  2.89801
2.37887  2.77519  2.98518  4.58477  3.61503
          0.03133  3.87472  4.59707  0.61958  0.77255  0.48576  0.95510
     43   1.01052  4.19303  3.46476  3.25606  4.05451  3.01753  4.27786
3.18035  3.27238  3.06187  4.09337  3.40514  3.75695  3.61331  3.52281
2.54327  2.82077  2.83436  5.48144  4.28192     43 a - - -
          2.68618  4.42225  2.77519  2.73123  3.46354  2.40513  3.72494
3.29354  2.67741  2.69355  4.24690  2.90347  2.73739  3.18146  2.89801
2.37887  2.77519  2.98518  4.58477  3.61503
          0.03133  3.87472  4.59707  0.61958  0.77255  0.48576  0.95510
     44   2.41377  4.25888  3.42992  3.18193  3.97070  3.09811  4.19910
3.09810  3.12495  2.96152  4.01182  3.39101  3.80201  3.51964  3.38358
2.61505  1.16810  2.79284  5.40831  4.18731     44 t - - -
```

```
         2.68618  4.42225  2.77519  2.73123  3.46354  2.40513  3.72494
3.29354  2.67741  2.69355  4.24690  2.90347  2.73739  3.18146  2.89801
2.37887  2.77519  2.98518  4.58477  3.61503
         0.03133  3.87472  4.59707  0.61958  0.77255  0.48576  0.95510
     45  1.01052  4.19303  3.46476  3.25606  4.05451  3.01753  4.27786
3.18035  3.27238  3.06187  4.09337  3.40514  3.75695  3.61331  3.52281
2.54327  2.82077  2.83436  5.48144  4.28192     45 a - - -
         2.68618  4.42225  2.77519  2.73123  3.46354  2.40513  3.72494
3.29354  2.67741  2.69355  4.24690  2.90347  2.73739  3.18146  2.89801
2.37887  2.77519  2.98518  4.58477  3.61503
         0.03133  3.87472  4.59707  0.61958  0.77255  0.48576  0.95510
     46  3.13260  4.49754  4.57868  4.06001  3.16843  4.35367  4.71105
1.51681  3.88418  1.27239  3.01918  4.33673  4.62015  4.13416  4.07017
3.72128  3.37745  1.92247  5.17278  3.98634     46 l - - -
         2.68618  4.42225  2.77519  2.73123  3.46354  2.40513  3.72494
3.29354  2.67741  2.69355  4.24690  2.90347  2.73739  3.18146  2.89801
2.37887  2.77519  2.98518  4.58477  3.61503
         0.03133  3.87472  4.59707  0.61958  0.77255  0.48576  0.95510
     47  1.47906  4.13431  3.26733  3.03926  4.22406  1.81134  4.15620
3.56680  3.14617  3.31697  4.17849  3.22640  3.64557  3.43748  3.45395
2.37864  2.68725  3.06492  5.56847  4.36006     47 a - - -
         2.68618  4.42225  2.77519  2.73123  3.46354  2.40513  3.72494
3.29354  2.67741  2.69355  4.24690  2.90347  2.73739  3.18146  2.89801
2.37887  2.77519  2.98518  4.58477  3.61503
         0.03133  3.87472  4.59707  0.61958  0.77255  0.48576  0.95510
     48  2.97404  4.93809  3.15019  2.77112  4.32503  3.46335  3.76023
3.75442  1.03520  3.31897  4.28021  3.19552  3.96346  2.95151  2.43176
3.01582  3.22709  3.46205  5.37869  4.19175     48 k - - -
         2.68618  4.42225  2.77519  2.73123  3.46354  2.40513  3.72494
3.29354  2.67741  2.69355  4.24690  2.90347  2.73739  3.18146  2.89801
2.37887  2.77519  2.98518  4.58477  3.61503
         0.03133  3.87472  4.59707  0.61958  0.77255  0.48576  0.95510
     49  3.33912  4.71305  4.09447  3.80105  2.66830  3.73256  3.90238
3.36273  3.52318  2.75727  3.99643  3.94963  4.25045  3.91067  3.65116
3.54280  3.63961  3.25312  0.88077  2.67027     49 w - - -
         2.68618  4.42225  2.77519  2.73123  3.46354  2.40513  3.72494
3.29354  2.67741  2.69355  4.24690  2.90347  2.73739  3.18146  2.89801
2.37887  2.77519  2.98518  4.58477  3.61503
         0.03133  3.87472  4.59707  0.61958  0.77255  0.48576  0.95510
     50  1.01052  4.19303  3.46476  3.25606  4.05451  3.01753  4.27786
3.18035  3.27238  3.06187  4.09337  3.40514  3.75695  3.61331  3.52281
2.54327  2.82077  2.83436  5.48144  4.28192     50 a - - -
         2.68618  4.42225  2.77519  2.73123  3.46354  2.40513  3.72494
3.29354  2.67741  2.69355  4.24690  2.90347  2.73739  3.18146  2.89801
2.37887  2.77519  2.98518  4.58477  3.61503
         0.03133  3.87472  4.59707  0.61958  0.77255  0.48576  0.95510
     51  1.97173  4.26959  3.83158  3.33413  3.48971  3.60751  4.20487
2.23091  3.22921  1.94945  3.39222  3.62770  4.09607  3.55528  3.51959
2.96133  2.94518  1.81628  5.14982  3.92481     51 v - - -
         2.68618  4.42225  2.77519  2.73123  3.46354  2.40513  3.72494
3.29354  2.67741  2.69355  4.24690  2.90347  2.73739  3.18146  2.89801
2.37887  2.77519  2.98518  4.58477  3.61503
         0.03133  3.87472  4.59707  0.61958  0.77255  0.48576  0.95510
```

```
     52   2.97404  4.93809  3.15019  2.77112  4.32503  3.46335  3.76023
3.75442  1.03520  3.31897  4.28021  3.19552  3.96346  2.95151  2.43176
3.01582  3.22709  3.46205  5.37869  4.19175      52 k - - -
          2.68618  4.42225  2.77519  2.73123  3.46354  2.40513  3.72494
3.29354  2.67741  2.69355  4.24690  2.90347  2.73739  3.18146  2.89801
2.37887  2.77519  2.98518  4.58477  3.61503
          0.03133  3.87472  4.59707  0.61958  0.77255  0.48576  0.95510
     53   1.90033  4.60225  2.89491  2.57035  4.06040  3.24131  3.76678
3.42994  2.51871  3.07303  3.98233  3.01532  3.79796  2.04163  2.86925
2.64820  2.88696  3.10380  5.36498  4.06287      53 a - - -
          2.68618  4.42225  2.77519  2.73123  3.46354  2.40513  3.72494
3.29354  2.67741  2.69355  4.24690  2.90347  2.73739  3.18146  2.89801
2.37887  2.77519  2.98518  4.58477  3.61503
          0.03133  3.87472  4.59707  0.61958  0.77255  0.48576  0.95510
     54   1.51825  4.13658  3.26063  3.03549  4.22837  1.75663  4.15612
3.57277  3.14577  3.32187  4.18341  3.22451  3.64576  3.43696  3.45398
2.37951  2.68894  3.06947  5.57160  4.36285      54 a - - -
          2.68618  4.42225  2.77519  2.73123  3.46354  2.40513  3.72494
3.29354  2.67741  2.69355  4.24690  2.90347  2.73739  3.18146  2.89801
2.37887  2.77519  2.98518  4.58477  3.61503
          0.03133  3.87472  4.59707  0.61958  0.77255  0.48576  0.95510
     55   3.26109  4.66217  4.24182  3.95666  1.00804  3.95049  3.87804
2.78442  3.86362  2.15392  3.51419  4.02166  4.40896  4.03095  3.98279
3.53096  3.56220  2.77530  4.12630  2.49298      55 f - - -
          2.68618  4.42225  2.77519  2.73123  3.46354  2.40513  3.72494
3.29354  2.67741  2.69355  4.24690  2.90347  2.73739  3.18146  2.89801
2.37887  2.77519  2.98518  4.58477  3.61503
          0.03133  3.87472  4.59707  0.61958  0.77255  0.48576  0.95510
     56   1.50879  4.11123  3.33050  3.05929  4.15834  2.89943  4.13453
3.49003  3.10781  3.25178  4.11504  3.23094  3.64092  3.41443  3.41277
1.63872  2.66000  3.00580  5.52886  4.29979      56 a - - -
          2.68618  4.42225  2.77519  2.73123  3.46354  2.40513  3.72494
3.29354  2.67741  2.69355  4.24690  2.90347  2.73739  3.18146  2.89801
2.37887  2.77519  2.98518  4.58477  3.61503
          0.03133  3.87472  4.59707  0.61958  0.77255  0.48576  0.95510
     57   2.97404  4.93809  3.15019  2.77112  4.32503  3.46335  3.76023
3.75442  1.03520  3.31897  4.28021  3.19552  3.96346  2.95151  2.43176
3.01582  3.22709  3.46205  5.37869  4.19175      57 k - - -
          2.68618  4.42225  2.77519  2.73123  3.46354  2.40513  3.72494
3.29354  2.67741  2.69355  4.24690  2.90347  2.73739  3.18146  2.89801
2.37887  2.77519  2.98518  4.58477  3.61503
          0.03133  3.87472  4.59707  0.61958  0.77255  0.48576  0.95510
     58   2.99209  4.42863  4.37275  3.87607  3.25911  4.12809  4.59007
1.97005  3.70968  1.38876  3.13713  4.14497  4.48592  4.00421  3.92546
3.50336  3.26357  1.49131  5.18215  3.96333      58 l - - -
          2.68618  4.42225  2.77519  2.73123  3.46354  2.40513  3.72494
3.29354  2.67741  2.69355  4.24690  2.90347  2.73739  3.18146  2.89801
2.37887  2.77519  2.98518  4.58477  3.61503
          0.03133  3.87472  4.59707  0.61958  0.77255  0.48576  0.95510
     59   2.78611  4.66799  3.30931  2.79714  3.74077  3.55875  3.74204
3.01833  1.77187  1.98128  3.70225  3.21898  3.97432  2.96372  2.59810
2.90985  3.01855  2.83207  5.13413  3.85937      59 k - - -
          2.68618  4.42225  2.77519  2.73123  3.46354  2.40513  3.72494
3.29354  2.67741  2.69355  4.24690  2.90347  2.73739  3.18146  2.89801
2.37887  2.77519  2.98518  4.58477  3.61503
```

```
          0.03133  3.87472  4.59707  0.61958  0.77255  0.48576  0.95510
     60   2.31597  4.23644  3.20823  3.02400  4.07846  2.96628  4.13384
3.60134  3.09793  3.32136  4.24719  3.24849  3.70524  3.44704  3.38396
1.08767  2.79625  3.13595  5.45075  4.16198     60 s - - -
          2.68618  4.42225  2.77519  2.73123  3.46354  2.40513  3.72494
3.29354  2.67741  2.69355  4.24690  2.90347  2.73739  3.18146  2.89801
2.37887  2.77519  2.98518  4.58477  3.61503
          0.03133  3.87472  4.59707  0.61958  0.77255  0.48576  0.95510
     61   2.74214  4.68879  2.94268  1.91768  3.74796  3.46211  3.78336
2.98957  2.58943  1.98383  3.71520  3.09109  3.92633  3.00893  2.95117
2.84367  2.99532  2.80064  5.20243  3.88918     61 e - - -
          2.68618  4.42225  2.77519  2.73123  3.46354  2.40513  3.72494
3.29354  2.67741  2.69355  4.24690  2.90347  2.73739  3.18146  2.89801
2.37887  2.77519  2.98518  4.58477  3.61503
          0.03133  3.87472  4.59707  0.61958  0.77255  0.48576  0.95510
     62   3.03284  4.45083  4.33904  3.95485  3.37561  4.06909  4.68249
1.07512  3.78303  1.96109  3.31052  4.19987  4.50000  4.13104  3.97934
3.56453  3.33193  1.84577  5.26697  4.00552     62 i - - -
          2.68618  4.42225  2.77519  2.73123  3.46354  2.40513  3.72494
3.29354  2.67741  2.69355  4.24690  2.90347  2.73739  3.18146  2.89801
2.37887  2.77519  2.98518  4.58477  3.61503
          0.03133  3.87472  4.59707  0.61958  0.77255  0.48576  0.95510
     63   3.26109  4.66217  4.24182  3.95666  1.00804  3.95049  3.87804
2.78442  3.86362  2.15392  3.51419  4.02166  4.40896  4.03095  3.98279
3.53096  3.56220  2.77530  4.12630  2.49298     63 f - - -
          2.68618  4.42225  2.77519  2.73123  3.46354  2.40513  3.72494
3.29354  2.67741  2.69355  4.24690  2.90347  2.73739  3.18146  2.89801
2.37887  2.77519  2.98518  4.58477  3.61503
          0.03133  3.87472  4.59707  0.61958  0.77255  0.48576  0.95510
     64   2.75420  4.51129  3.36160  3.20088  4.19604  3.19867  4.27823
3.74193  3.25835  3.36857  4.41844  3.50740  0.82346  3.64706  3.50371
2.92174  3.19592  3.38706  5.38207  4.31670     64 p - - -
          2.68618  4.42225  2.77519  2.73123  3.46354  2.40513  3.72494
3.29354  2.67741  2.69355  4.24690  2.90347  2.73739  3.18146  2.89801
2.37887  2.77519  2.98518  4.58477  3.61503
          0.03133  3.87472  4.59707  0.61958  0.77255  0.48576  0.95510
     65   2.66752  4.44283  3.34177  3.24141  4.38386  0.72564  4.36163
3.98394  3.42300  3.63781  4.61350  3.50605  3.83404  3.74696  3.65888
2.83948  3.14893  3.51182  5.47850  4.48580     65 g - - -
          2.68618  4.42225  2.77519  2.73123  3.46354  2.40513  3.72494
3.29354  2.67741  2.69355  4.24690  2.90347  2.73739  3.18146  2.89801
2.37887  2.77519  2.98518  4.58477  3.61503
          0.03133  3.87472  4.59707  0.61958  0.77255  0.48576  0.95510
     66   2.45727  4.52953  2.72571  2.52285  4.20658  3.07664  3.84363
3.72669  2.72480  3.35743  4.20440  1.94532  3.72419  3.05619  3.12334
1.81642  2.84850  3.28006  5.50792  4.15835     66 s - - -
          2.68618  4.42225  2.77519  2.73123  3.46354  2.40513  3.72494
3.29354  2.67741  2.69355  4.24690  2.90347  2.73739  3.18146  2.89801
2.37887  2.77519  2.98518  4.58477  3.61503
          0.03133  3.87472  4.59707  0.61958  0.77255  0.48576  0.95510
     67   2.66946  4.84829  2.73109  2.22808  4.16673  3.33116  3.64211
3.55839  2.36539  3.15770  4.00869  2.89719  3.80908  2.13330  2.74998
2.69051  2.29560  3.23232  5.38819  4.06571     67 q - - -
```

```
          2.68618  4.42225  2.77519  2.73123  3.46354  2.40513  3.72494
3.29354  2.67741  2.69355  4.24690  2.90347  2.73739  3.18146  2.89801
2.37887  2.77519  2.98518  4.58477  3.61503
          0.03133  3.87472  4.59707  0.61958  0.77255  0.48576  0.95510
     68   2.35733  4.28771  3.16751  2.91002  4.08782  3.04089  4.04004
3.40024  2.92699  3.12990  4.07055  3.19999  1.55876  3.30342  3.23946
2.51842  2.41750  3.01311  5.45241  4.20819      68 p - - -
          2.68618  4.42225  2.77519  2.73123  3.46354  2.40513  3.72494
3.29354  2.67741  2.69355  4.24690  2.90347  2.73739  3.18146  2.89801
2.37887  2.77519  2.98518  4.58477  3.61503
          0.03133  3.87472  4.59707  0.61958  0.77255  0.48576  0.95510
     69   1.16239  4.12519  3.33264  3.10573  4.18040  2.48648  4.18592
3.47192  3.17904  3.26691  4.15207  3.26850  3.66091  3.48330  3.46644
2.39867  2.69970  3.00297  5.54135  4.33999      69 a - - -
          2.68618  4.42225  2.77519  2.73123  3.46354  2.40513  3.72494
3.29354  2.67741  2.69355  4.24690  2.90347  2.73739  3.18146  2.89801
2.37887  2.77519  2.98518  4.58477  3.61503
          0.03133  3.87472  4.59707  0.61958  0.77255  0.48576  0.95510
     70   2.41377  4.25888  3.42992  3.18193  3.97070  3.09811  4.19910
3.09810  3.12495  2.96152  4.01182  3.39101  3.80201  3.51964  3.38358
2.61505  1.16810  2.79284  5.40831  4.18731      70 t - - -
          2.68618  4.42225  2.77519  2.73123  3.46354  2.40513  3.72494
3.29354  2.67741  2.69355  4.24690  2.90347  2.73739  3.18146  2.89801
2.37887  2.77519  2.98518  4.58477  3.61503
          0.03133  3.87472  4.59707  0.61958  0.77255  0.48576  0.95510
     71   2.97087  4.51925  4.05178  3.64534  3.20855  3.83573  4.37996
2.38437  3.41332  1.80283  1.37362  3.92258  4.29629  3.79501  3.61839
3.32024  3.27619  2.39076  5.04971  3.81172      71 m - - -
          2.68618  4.42225  2.77519  2.73123  3.46354  2.40513  3.72494
3.29354  2.67741  2.69355  4.24690  2.90347  2.73739  3.18146  2.89801
2.37887  2.77519  2.98518  4.58477  3.61503
          0.03133  3.87472  4.59707  0.61958  0.77255  0.48576  0.95510
     72   2.87078  5.35965  1.82463  1.37833  4.64961  3.18528  3.71260
4.14195  2.69839  3.67704  4.53636  2.66555  3.79426  2.88187  3.26079
2.77355  3.15667  3.74027  5.83576  4.36779      72 e - - -
          2.68618  4.42225  2.77519  2.73123  3.46354  2.40513  3.72494
3.29354  2.67741  2.69355  4.24690  2.90347  2.73739  3.18146  2.89801
2.37887  2.77519  2.98518  4.58477  3.61503
          0.03133  3.87472  4.59707  0.61958  0.77255  0.48576  0.95510
     73   2.97404  4.93809  3.15019  2.77112  4.32503  3.46335  3.76023
3.75442  1.03520  3.31897  4.28021  3.19552  3.96346  2.95151  2.43176
3.01582  3.22709  3.46205  5.37869  4.19175      73 k - - -
          2.68618  4.42225  2.77519  2.73123  3.46354  2.40513  3.72494
3.29354  2.67741  2.69355  4.24690  2.90347  2.73739  3.18146  2.89801
2.37887  2.77519  2.98518  4.58477  3.61503
          0.03133  3.87472  4.59707  0.61958  0.77255  0.48576  0.95510
     74   2.80983  4.35172  4.12850  3.79161  3.55163  3.72629  4.59291
2.04988  3.65943  2.23820  3.51634  3.98111  4.28661  4.01903  3.87173
3.22990  3.17128  1.02205  5.33648  4.06827      74 v - - -
          2.68618  4.42225  2.77519  2.73123  3.46354  2.40513  3.72494
3.29354  2.67741  2.69355  4.24690  2.90347  2.73739  3.18146  2.89801
2.37887  2.77519  2.98518  4.58477  3.61503
          0.03133  3.87472  4.59707  0.61958  0.77255  0.48576  0.95510
```

```
     75   2.58139  2.94850  3.49265  2.93120  3.82012  3.35670  3.81339
3.15992  2.46414  2.85959  3.79199  3.27059  3.86958  3.06046  1.63717
2.74093  2.89139  2.88375  5.14700  3.91403      75 r - - -
          2.68618  4.42225  2.77519  2.73123  3.46354  2.40513  3.72494
3.29354  2.67741  2.69355  4.24690  2.90347  2.73739  3.18146  2.89801
2.37887  2.77519  2.98518  4.58477  3.61503
          0.03133  3.87472  4.59707  0.61958  0.77255  0.48576  0.95510
     76   2.74562  4.31316  3.90188  3.37002  3.38145  3.79348  4.20559
1.90047  3.23983  1.85092  3.27771  3.69989  4.19371  3.57007  3.52265
3.11340  2.19099  2.02560  5.07971  3.86215      76 l - - -
          2.68618  4.42225  2.77519  2.73123  3.46354  2.40513  3.72494
3.29354  2.67741  2.69355  4.24690  2.90347  2.73739  3.18146  2.89801
2.37887  2.77519  2.98518  4.58477  3.61503
          0.03133  3.87472  4.59707  0.61958  0.77255  0.48576  0.95510
     77   2.79617  5.03072  2.38938  1.29037  4.46300  2.71365  3.76718
3.91825  2.65702  3.50900  4.38559  2.81503  3.81085  2.95355  3.11123
2.77355  3.10294  3.54050  5.64782  4.29771      77 e - - -
          2.68618  4.42225  2.77519  2.73123  3.46354  2.40513  3.72494
3.29354  2.67741  2.69355  4.24690  2.90347  2.73739  3.18146  2.89801
2.37887  2.77519  2.98518  4.58477  3.61503
          0.03133  3.87472  4.59707  0.61958  0.77255  0.48576  0.95510
     78   2.80983  4.35172  4.12850  3.79161  3.55163  3.72629  4.59291
2.04988  3.65943  2.23820  3.51634  3.98111  4.28661  4.01903  3.87173
3.22990  3.17128  1.02205  5.33648  4.06827      78 v - - -
          2.68618  4.42225  2.77519  2.73123  3.46354  2.40513  3.72494
3.29354  2.67741  2.69355  4.24690  2.90347  2.73739  3.18146  2.89801
2.37887  2.77519  2.98518  4.58477  3.61503
          0.03133  3.87472  4.59707  0.61958  0.77255  0.48576  0.95510
     79   2.80826  5.13391  2.47113  1.74377  4.41976  3.29985  3.65014
3.87894  2.38270  3.40687  4.26697  2.80430  3.82402  1.98888  2.77915
2.76478  3.06050  3.52472  5.57169  4.20330      79 e - - -
          2.68618  4.42225  2.77519  2.73123  3.46354  2.40513  3.72494
3.29354  2.67741  2.69355  4.24690  2.90347  2.73739  3.18146  2.89801
2.37887  2.77519  2.98518  4.58477  3.61503
          0.03133  3.87472  4.59707  0.61958  0.77255  0.48576  0.95510
     80   2.41377  4.25888  3.42992  3.18193  3.97070  3.09811  4.19910
3.09810  3.12495  2.96152  4.01182  3.39101  3.80201  3.51964  3.38358
2.61505  1.16810  2.79284  5.40831  4.18731      80 t - - -
          2.68618  4.42225  2.77519  2.73123  3.46354  2.40513  3.72494
3.29354  2.67741  2.69355  4.24690  2.90347  2.73739  3.18146  2.89801
2.37887  2.77519  2.98518  4.58477  3.61503
          0.03133  3.87472  4.59707  0.61958  0.77255  0.48576  0.95510
     81   3.15438  4.56174  4.47629  3.97273  3.08609  4.29083  4.61993
1.91344  3.76158  1.03206  2.97038  4.26143  4.57699  4.03957  3.95087
3.66938  3.40403  2.10140  5.09110  3.87786      81 l - - -
          2.68618  4.42225  2.77519  2.73123  3.46354  2.40513  3.72494
3.29354  2.67741  2.69355  4.24690  2.90347  2.73739  3.18146  2.89801
2.37887  2.77519  2.98518  4.58477  3.61503
          0.03133  3.87472  4.59707  0.61958  0.77255  0.48576  0.95510
     82   3.13260  4.49754  4.57868  4.06001  3.16843  4.35367  4.71105
1.51681  3.88418  1.27239  3.01918  4.33673  4.62015  4.13416  4.07017
3.72128  3.37745  1.92247  5.17278  3.98634      82 l - - -
          2.68618  4.42225  2.77519  2.73123  3.46354  2.40513  3.72494
3.29354  2.67741  2.69355  4.24690  2.90347  2.73739  3.18146  2.89801
2.37887  2.77519  2.98518  4.58477  3.61503
```

```
          0.03133  3.87472  4.59707  0.61958  0.77255  0.48576  0.95510
     83   2.79281  5.16311  1.88289  2.14880  4.49396  3.17296  3.72914
4.04176  2.70456  3.60065  4.45880  1.64015  3.78543  2.90947  3.23898
2.73533  3.09926  3.63563  5.72930  4.27548     83 n - - -
          2.68618  4.42225  2.77519  2.73123  3.46354  2.40513  3.72494
3.29354  2.67741  2.69355  4.24690  2.90347  2.73739  3.18146  2.89801
2.37887  2.77519  2.98518  4.58477  3.61503
          0.03133  3.87472  4.59707  0.61958  0.77255  0.48576  0.95510
     84   2.88236  4.86831  2.90423  2.65889  4.03720  3.36741  3.81726
3.66821  2.47950  3.17034  4.19804  3.11869  3.92083  1.23004  2.76368
2.92996  3.17928  3.39771  5.30876  4.00263     84 q - - -
          2.68618  4.42225  2.77519  2.73123  3.46354  2.40513  3.72494
3.29354  2.67741  2.69355  4.24690  2.90347  2.73739  3.18146  2.89801
2.37887  2.77519  2.98518  4.58477  3.61503
          0.03133  3.87472  4.59707  0.61958  0.77255  0.48576  0.95510
     85   2.76274  4.88530  3.15853  2.59302  4.25991  3.47509  3.59023
3.62277  1.67326  3.17531  4.02603  3.04409  3.88124  2.73882  2.04199
2.79246  2.48659  3.30386  5.33572  4.09252     85 k - - -
          2.68618  4.42225  2.77519  2.73123  3.46354  2.40513  3.72494
3.29354  2.67741  2.69355  4.24690  2.90347  2.73739  3.18146  2.89801
2.37887  2.77519  2.98518  4.58477  3.61503
          0.03133  3.87472  4.59707  0.61958  0.77255  0.48576  0.95510
     86   3.15771  4.61926  4.19314  3.83291  3.13642  3.98506  4.48794
2.34049  3.60064  0.87242  3.15851  4.10818  4.41998  3.96843  3.77638
3.55606  3.45182  2.37843  5.01661  3.74048     86 l - - -
          2.68618  4.42225  2.77519  2.73123  3.46354  2.40513  3.72494
3.29354  2.67741  2.69355  4.24690  2.90347  2.73739  3.18146  2.89801
2.37887  2.77519  2.98518  4.58477  3.61503
          0.03133  3.87472  4.59707  0.61958  0.77255  0.48576  0.95510
     87   2.58656  4.68898  2.78627  2.48866  4.13578  3.22911  3.72695
3.62371  2.47098  3.21887  4.08413  2.95414  3.78618  2.02709  2.83193
1.94647  2.90532  3.25609  5.39838  4.06369     87 s - - -
          2.68618  4.42225  2.77519  2.73123  3.46354  2.40513  3.72494
3.29354  2.67741  2.69355  4.24690  2.90347  2.73739  3.18146  2.89801
2.37887  2.77519  2.98518  4.58477  3.61503
          0.03133  3.87472  4.59707  0.61958  0.77255  0.48576  0.95510
     88   2.44923  4.93763  2.23014  2.26463  4.27531  3.26642  3.63363
3.70273  2.46194  3.28068  4.10031  2.80376  3.76532  2.14030  2.93589
2.63050  2.65855  3.33667  5.49256  4.11595     88 q - - -
          2.68618  4.42225  2.77519  2.73123  3.46354  2.40513  3.72494
3.29354  2.67741  2.69355  4.24690  2.90347  2.73739  3.18146  2.89801
2.37887  2.77519  2.98518  4.58477  3.61503
          0.03133  3.87472  4.59707  0.61958  0.77255  0.48576  0.95510
     89   2.99187  5.12073  0.91483  2.33607  4.49801  3.21224  3.93281
4.08666  3.01732  3.69455  4.67262  2.89258  3.86518  3.17190  3.53391
2.95391  3.33046  3.72558  5.66619  4.37043     89 d - - -
          2.68618  4.42225  2.77519  2.73123  3.46354  2.40513  3.72494
3.29354  2.67741  2.69355  4.24690  2.90347  2.73739  3.18146  2.89801
2.37887  2.77519  2.98518  4.58477  3.61503
          0.03133  3.87472  4.59707  0.61958  0.77255  0.48576  0.95510
     90   3.00784  4.84699  3.49870  3.00918  4.20214  3.49552  3.82565
3.74393  2.23793  3.25944  4.25948  3.36879  3.99792  3.04579  0.97495
3.09185  3.27359  3.46776  5.28422  4.12892     90 r - - -
```

```
          2.68618  4.42225  2.77519  2.73123  3.46354  2.40513  3.72494
  3.29354  2.67741  2.69355  4.24690  2.90347  2.73739  3.18146  2.89801
  2.37887  2.77519  2.98518  4.58477  3.61503
          0.03133  3.87472  4.59707  0.61958  0.77255  0.48576  0.95510
     91   2.80983  4.35172  4.12850  3.79161  3.55163  3.72629  4.59291
  2.04988  3.65943  2.23820  3.51634  3.98111  4.28661  4.01903  3.87173
  3.22990  3.17128  1.02205  5.33648  4.06827     91 v - - -
          2.68618  4.42225  2.77519  2.73123  3.46354  2.40513  3.72494
  3.29354  2.67741  2.69355  4.24690  2.90347  2.73739  3.18146  2.89801
  2.37887  2.77519  2.98518  4.58477  3.61503
          0.03133  3.87472  4.59707  0.61958  0.77255  0.48576  0.95510
     92   2.24782  4.80812  2.72340  2.40010  4.22621  3.27988  3.66693
  3.63099  2.07901  3.23234  4.07104  2.14075  3.79139  2.83217  2.77764
  2.66110  2.91344  3.27444  5.43454  4.10917     92 k - - -
          2.68618  4.42225  2.77519  2.73123  3.46354  2.40513  3.72494
  3.29354  2.67741  2.69355  4.24690  2.90347  2.73739  3.18146  2.89801
  2.37887  2.77519  2.98518  4.58477  3.61503
          0.03133  3.87472  4.59707  0.61958  0.77255  0.48576  0.95510
     93   2.82381  4.32998  4.13407  3.69375  3.47641  3.88099  4.50345
  1.28356  3.53986  2.10528  3.35664  3.94975  4.33865  3.89157  3.77742
  3.27892  2.74371  1.82572  5.28055  4.05083     93 i - - -
          2.68618  4.42225  2.77519  2.73123  3.46354  2.40513  3.72494
  3.29354  2.67741  2.69355  4.24690  2.90347  2.73739  3.18146  2.89801
  2.37887  2.77519  2.98518  4.58477  3.61503
          0.03133  3.87472  4.59707  0.61958  0.77255  0.48576  0.95510
     94   3.15771  4.61926  4.19314  3.83291  3.13642  3.98506  4.48794
  2.34049  3.60064  0.87242  3.15851  4.10818  4.41998  3.96843  3.77638
  3.55606  3.45182  2.37843  5.01661  3.74048     94 l - - -
          2.68618  4.42225  2.77519  2.73123  3.46354  2.40513  3.72494
  3.29354  2.67741  2.69355  4.24690  2.90347  2.73739  3.18146  2.89801
  2.37887  2.77519  2.98518  4.58477  3.61503
          0.03133  3.87472  4.59707  0.61958  0.77255  0.48576  0.95510
     95   2.78870  5.15083  2.41081  1.92754  4.46227  3.27392  3.64596
  3.91449  2.23137  3.44916  4.28546  1.90658  3.80629  2.80115  2.84576
  2.73864  3.04492  3.54249  5.61201  4.22722     95 n - - -
          2.68618  4.42225  2.77519  2.73123  3.46354  2.40513  3.72494
  3.29354  2.67741  2.69355  4.24690  2.90347  2.73739  3.18146  2.89801
  2.37887  2.77519  2.98518  4.58477  3.61503
          0.03133  3.87472  4.59707  0.61958  0.77255  0.48576  0.95510
     96   1.27998  4.12498  3.30266  3.06871  4.20059  2.17378  4.16581
  3.52575  3.15844  3.29089  4.15875  3.24337  3.64913  3.45335  3.45865
  2.38173  2.68624  3.03641  5.55278  4.34774     96 a - - -
          2.68618  4.42225  2.77519  2.73123  3.46354  2.40513  3.72494
  3.29354  2.67741  2.69355  4.24690  2.90347  2.73739  3.18146  2.89801
  2.37887  2.77519  2.98518  4.58477  3.61503
          0.03133  3.87472  4.59707  0.61958  0.77255  0.48576  0.95510
     97   2.95406  5.06580  2.48830  1.02217  4.41227  3.27540  3.86227
  3.86661  2.74307  3.48333  4.45717  2.93393  3.88006  3.07782  3.15222
  2.93617  3.25828  3.55019  5.58341  4.30555     97 e - - -
          2.68618  4.42225  2.77519  2.73123  3.46354  2.40513  3.72494
  3.29354  2.67741  2.69355  4.24690  2.90347  2.73739  3.18146  2.89801
  2.37887  2.77519  2.98518  4.58477  3.61503
          0.03133  3.87472  4.59707  0.61958  0.77255  0.48576  0.95510
```

```
     98   3.25019  4.73827  3.84149  3.57122  2.29338  3.83270  3.61936
3.25908  3.42867  2.71244  3.93385  3.72612  4.30645  3.73417  3.61195
3.38669  3.53686  3.12972  3.93726  0.96205     98 y - - -
          2.68618  4.42225  2.77519  2.73123  3.46354  2.40513  3.72494
3.29354  2.67741  2.69355  4.24690  2.90347  2.73739  3.18146  2.89801
2.37887  2.77519  2.98518  4.58477  3.61503
          0.03133  3.87472  4.59707  0.61958  0.77255  0.48576  0.95510
     99   2.85844  5.08037  3.09821  2.35349  4.46650  3.51503  3.55290
3.84231  1.75009  3.31819  4.15461  3.01295  3.90171  2.46782  1.77451
2.84398  3.04755  3.50413  5.41305  4.17465     99 k - - -
          2.68618  4.42225  2.77519  2.73123  3.46354  2.40513  3.72494
3.29354  2.67741  2.69355  4.24690  2.90347  2.73739  3.18146  2.89801
2.37887  2.77519  2.98518  4.58477  3.61503
          0.03133  3.87472  4.59707  0.61958  0.77255  0.48576  0.95510
    100   2.66752  4.44283  3.34177  3.24141  4.38386  0.72564  4.36163
3.98394  3.42300  3.63781  4.61350  3.50605  3.83404  3.74696  3.65888
2.83948  3.14893  3.51182  5.47850  4.48580    100 g - - -
          2.68618  4.42225  2.77519  2.73123  3.46354  2.40513  3.72494
3.29354  2.67741  2.69355  4.24690  2.90347  2.73739  3.18146  2.89801
2.37887  2.77519  2.98518  4.58477  3.61503
          0.03133  3.87472  4.59707  0.61958  0.77255  0.48576  0.95510
    101   3.05261  4.43045  4.50289  3.99502  2.39429  4.22494  4.44891
1.39381  3.87359  1.72344  3.07221  4.21147  4.52514  4.06624  4.04674
3.58139  3.29953  1.98693  4.85845  3.49858    101 i - - -
          2.68618  4.42225  2.77519  2.73123  3.46354  2.40513  3.72494
3.29354  2.67741  2.69355  4.24690  2.90347  2.73739  3.18146  2.89801
2.37887  2.77519  2.98518  4.58477  3.61503
          0.03133  3.87472  4.59707  0.61958  0.77255  0.48576  0.95510
    102   3.03284  4.45083  4.33904  3.95485  3.37561  4.06909  4.68249
1.07512  3.78303  1.96109  3.31052  4.19987  4.50000  4.13104  3.97934
3.56453  3.33193  1.84577  5.26697  4.00552    102 i - - -
          2.68618  4.42225  2.77519  2.73123  3.46354  2.40513  3.72494
3.29354  2.67741  2.69355  4.24690  2.90347  2.73739  3.18146  2.89801
2.37887  2.77519  2.98518  4.58477  3.61503
          0.03133  3.87472  4.59707  0.61958  0.77255  0.48576  0.95510
    103   2.76968  5.24130  2.13431  1.59869  4.50926  3.23088  3.12439
4.00632  2.53193  3.52211  4.33937  2.46782  3.77553  2.79169  3.05371
2.69705  3.03040  3.60827  5.68613  4.24273    103 e - - -
          2.68618  4.42225  2.77519  2.73123  3.46354  2.40513  3.72494
3.29354  2.67741  2.69355  4.24690  2.90347  2.73739  3.18146  2.89801
2.37887  2.77519  2.98518  4.58477  3.61503
          0.03133  3.87472  4.59707  0.61958  0.77255  0.48576  0.95510
    104   2.95114  4.39420  4.34347  3.86179  3.34681  4.08312  4.59930
1.91531  3.69652  1.67392  3.24019  4.12139  4.47151  4.01502  3.92138
3.46859  3.23383  1.27088  5.23680  3.99063    104 v - - -
          2.68618  4.42225  2.77519  2.73123  3.46354  2.40513  3.72494
3.29354  2.67741  2.69355  4.24690  2.90347  2.73739  3.18146  2.89801
2.37887  2.77519  2.98518  4.58477  3.61503
          0.03133  3.87472  4.59707  0.61958  0.77255  0.48576  0.95510
    105   2.22269  4.18735  3.12506  2.91414  4.25844  2.19303  4.09205
3.72521  3.05704  3.40027  4.23598  3.15615  3.64203  3.35505  3.38787
1.37080  2.70200  3.17957  5.58190  4.33518    105 s - - -
          2.68618  4.42225  2.77519  2.73123  3.46354  2.40513  3.72494
3.29354  2.67741  2.69355  4.24690  2.90347  2.73739  3.18146  2.89801
2.37887  2.77519  2.98518  4.58477  3.61503
```

0.03133 3.87472 4.59707 0.61958 0.77255 0.48576 0.95510
106 2.77886 5.15524 1.81598 2.20940 4.47024 3.25790 3.66037
3.93628 1.82110 3.47924 4.31690 2.76402 3.79768 2.81545 2.93056
2.72703 3.04281 3.55568 5.62852 4.23187 106 d - - -
2.68618 4.42225 2.77519 2.73123 3.46354 2.40513 3.72494
3.29354 2.67741 2.69355 4.24690 2.90347 2.73739 3.18146 2.89801
2.37887 2.77519 2.98518 4.58477 3.61503
0.03133 3.87472 4.59707 0.61958 0.77255 0.48576 0.95510
107 2.93636 4.35923 4.42224 3.94940 3.47392 4.10897 4.71822
1.69168 3.80984 2.04085 3.33487 4.19004 4.51289 4.12494 4.03997
3.50257 3.22852 1.12614 5.37156 4.12950 107 v - - -
2.68618 4.42225 2.77519 2.73123 3.46354 2.40513 3.72494
3.29354 2.67741 2.69355 4.24690 2.90347 2.73739 3.18146 2.89801
2.37887 2.77519 2.98518 4.58477 3.61503
0.03133 3.87472 4.59707 0.61958 0.77255 0.48576 0.95510
108 3.26109 4.66217 4.24182 3.95666 1.00804 3.95049 3.87804
2.78442 3.86362 2.15392 3.51419 4.02166 4.40896 4.03095 3.98279
3.53096 3.56220 2.77530 4.12630 2.49298 108 f - - -
2.68618 4.42225 2.77519 2.73123 3.46354 2.40513 3.72494
3.29354 2.67741 2.69355 4.24690 2.90347 2.73739 3.18146 2.89801
2.37887 2.77519 2.98518 4.58477 3.61503
0.03133 3.87472 4.59707 0.61958 0.77255 0.48576 0.95510
109 2.61294 4.78337 1.86154 2.33658 4.26033 3.18249 3.76276
3.60301 2.68368 3.29589 4.16868 2.85096 3.76968 2.94783 3.16192
2.64400 2.00652 3.24393 5.54930 4.18222 109 d - - -
2.68618 4.42225 2.77519 2.73123 3.46354 2.40513 3.72494
3.29354 2.67741 2.69355 4.24690 2.90347 2.73739 3.18146 2.89801
2.37887 2.77519 2.98518 4.58477 3.61503
0.03133 3.87472 4.59707 0.61958 0.77255 0.48576 0.95510
110 2.03562 4.80185 1.79171 2.29058 4.36847 2.76919 3.75644
3.78728 2.69828 3.40832 4.24510 2.80892 3.74313 2.93426 3.20007
2.60968 2.92686 3.37522 5.62479 4.24584 110 d - - -
2.68618 4.42225 2.77519 2.73123 3.46354 2.40513 3.72494
3.29354 2.67741 2.69355 4.24690 2.90347 2.73739 3.18146 2.89801
2.37887 2.77519 2.98518 4.58477 3.61503
0.03133 3.87472 4.59707 0.61958 0.77255 0.48576 0.95510
111 3.33854 4.72325 4.20961 3.83916 1.81690 4.11659 3.45878
3.12163 3.70797 2.52212 3.75852 3.83333 4.46803 3.83446 3.85640
3.48276 3.57668 3.02854 3.67058 1.11370 111 y - - -
2.68618 4.42225 2.77519 2.73123 3.46354 2.40513 3.72494
3.29354 2.67741 2.69355 4.24690 2.90347 2.73739 3.18146 2.89801
2.37887 2.77519 2.98518 4.58477 3.61503
0.03133 3.87472 4.59707 0.61958 0.77255 0.48576 0.95510
112 2.99963 4.36519 4.56916 4.07407 3.46301 4.26218 4.81725
1.39403 3.95119 1.98468 3.29021 4.31808 4.60623 4.23397 4.16938
3.64342 3.27184 1.28179 5.39949 4.18742 112 v - - -
2.68618 4.42225 2.77519 2.73123 3.46354 2.40513 3.72494
3.29354 2.67741 2.69355 4.24690 2.90347 2.73739 3.18146 2.89801
2.37887 2.77519 2.98518 4.58477 3.61503
0.03133 3.87472 4.59707 0.61958 0.77255 0.48576 0.95510
113 2.66968 4.88238 2.73762 2.39412 4.28922 3.30682 3.63920
3.72358 1.82591 3.28301 4.11305 2.42114 3.80202 2.79463 2.67519
2.28353 2.93626 3.35731 5.44956 4.12493 113 k - - -

```
          2.68618  4.42225  2.77519  2.73123  3.46354  2.40513  3.72494
 3.29354  2.67741  2.69355  4.24690  2.90347  2.73739  3.18146  2.89801
 2.37887  2.77519  2.98518  4.58477  3.61503
          0.03133  3.87472  4.59707  0.61958  0.77255  0.48576  0.95510
    114   2.88236  4.86831  2.90423  2.65889  4.03720  3.36741  3.81726
 3.66821  2.47950  3.17034  4.19804  3.11869  3.92083  1.23004  2.76368
 2.92996  3.17928  3.39771  5.30876  4.00263    114 q - - -
          2.68618  4.42225  2.77519  2.73123  3.46354  2.40513  3.72494
 3.29354  2.67741  2.69355  4.24690  2.90347  2.73739  3.18146  2.89801
 2.37887  2.77519  2.98518  4.58477  3.61503
          0.03133  3.87472  4.59707  0.61958  0.77255  0.48576  0.95510
    115   2.29066  4.26619  3.09152  2.82123  4.15546  2.98297  3.99018
 3.60389  2.90028  3.27334  4.12730  3.12364  2.12398  3.23519  3.24758
 1.66406  2.73311  3.13100  5.48492  4.21511    115 s - - -
          2.68618  4.42225  2.77519  2.73123  3.46354  2.40513  3.72494
 3.29354  2.67741  2.69355  4.24690  2.90347  2.73739  3.18146  2.89801
 2.37887  2.77519  2.98518  4.58477  3.61503
          0.03133  3.87472  4.59707  0.61958  0.77255  0.48576  0.95510
    116   2.51286  4.57590  2.90758  2.54547  4.12411  2.23291  3.74047
 3.50544  2.13059  3.14512  3.99077  2.99089  3.76558  2.92739  2.86119
 2.59586  2.35548  3.13961  5.38178  4.09387    116 k - - -
          2.68618  4.42225  2.77519  2.73123  3.46354  2.40513  3.72494
 3.29354  2.67741  2.69355  4.24690  2.90347  2.73739  3.18146  2.89801
 2.37887  2.77519  2.98518  4.58477  3.61503
          0.03133  3.87472  4.59707  0.61958  0.77255  0.48576  0.95510
    117   3.26109  4.66217  4.24182  3.95666  1.00804  3.95049  3.87804
 2.78442  3.86362  2.15392  3.51419  4.02166  4.40896  4.03095  3.98279
 3.53096  3.56220  2.77530  4.12630  2.49298    117 f - - -
          2.68618  4.42225  2.77519  2.73123  3.46354  2.40513  3.72494
 3.29354  2.67741  2.69355  4.24690  2.90347  2.73739  3.18146  2.89801
 2.37887  2.77519  2.98518  4.58477  3.61503
          0.03133  3.87472  4.59707  0.61958  0.77255  0.48576  0.95510
    118   2.54646  4.62261  2.76276  2.11831  4.08861  3.22445  3.74030
 3.43990  2.57482  3.11673  3.97478  2.94189  3.77313  2.93348  2.98247
 2.41079  1.96065  3.09877  5.38482  4.07300    118 t - - -
          2.68618  4.42225  2.77519  2.73123  3.46354  2.40513  3.72494
 3.29354  2.67741  2.69355  4.24690  2.90347  2.73739  3.18146  2.89801
 2.37887  2.77519  2.98518  4.58477  3.61503
          0.03133  3.87472  4.59707  0.61958  0.77255  0.48576  0.95510
    119   2.33538  4.27104  3.18947  2.89539  4.07093  3.04122  4.01348
 3.37371  2.90151  3.10891  4.02746  3.18762  1.76071  3.26939  3.22477
 2.49668  2.16447  2.98496  5.43412  4.19010    119 p - - -
          2.68618  4.42225  2.77519  2.73123  3.46354  2.40513  3.72494
 3.29354  2.67741  2.69355  4.24690  2.90347  2.73739  3.18146  2.89801
 2.37887  2.77519  2.98518  4.58477  3.61503
          0.03133  3.87472  4.59707  0.61958  0.77255  0.48576  0.95510
    120   1.27998  4.12498  3.30266  3.06871  4.20059  2.17378  4.16581
 3.52575  3.15844  3.29089  4.15875  3.24337  3.64913  3.45335  3.45865
 2.38173  2.68624  3.03641  5.55278  4.34774    120 a - - -
          2.68618  4.42225  2.77519  2.73123  3.46354  2.40513  3.72494
 3.29354  2.67741  2.69355  4.24690  2.90347  2.73739  3.18146  2.89801
 2.37887  2.77519  2.98518  4.58477  3.61503
          0.03133  3.87472  4.59707  0.61958  0.77255  0.48576  0.95510
```

```
    121   2.41377  4.25888  3.42992  3.18193  3.97070  3.09811  4.19910
 3.09810  3.12495  2.96152  4.01182  3.39101  3.80201  3.51964  3.38358
 2.61505  1.16810  2.79284  5.40831  4.18731    121 t - - -
          2.68618  4.42225  2.77519  2.73123  3.46354  2.40513  3.72494
 3.29354  2.67741  2.69355  4.24690  2.90347  2.73739  3.18146  2.89801
 2.37887  2.77519  2.98518  4.58477  3.61503
          0.03133  3.87472  4.59707  0.61958  0.77255  0.48576  0.95510
    122   1.01052  4.19303  3.46476  3.25606  4.05451  3.01753  4.27786
 3.18035  3.27238  3.06187  4.09337  3.40514  3.75695  3.61331  3.52281
 2.54327  2.82077  2.83436  5.48144  4.28192    122 a - - -
          2.68618  4.42225  2.77519  2.73123  3.46354  2.40513  3.72494
 3.29354  2.67741  2.69355  4.24690  2.90347  2.73739  3.18146  2.89801
 2.37887  2.77519  2.98518  4.58477  3.61503
          0.03133  3.87472  4.59707  0.61958  0.77255  0.48576  0.95510
    123   2.80563  4.99566  2.79945  2.46387  4.36826  3.37877  3.63505
 3.81299  1.49340  3.35016  4.20849  2.39114  3.86561  2.79307  2.47436
 2.80372  3.05176  3.46395  5.45336  4.15660    123 k - - -
          2.68618  4.42225  2.77519  2.73123  3.46354  2.40513  3.72494
 3.29354  2.67741  2.69355  4.24690  2.90347  2.73739  3.18146  2.89801
 2.37887  2.77519  2.98518  4.58477  3.61503
          0.03133  3.87472  4.59707  0.61958  0.77255  0.48576  0.95510
    124   2.45855  4.47827  3.07680  2.65000  4.06983  2.21240  3.76721
 3.45945  2.50637  3.10059  3.94795  3.05555  3.75608  2.96331  2.35396
 2.56589  2.32308  3.08817  5.34004  4.07141    124 g - - -
          2.68618  4.42225  2.77519  2.73123  3.46354  2.40513  3.72494
 3.29354  2.67741  2.69355  4.24690  2.90347  2.73739  3.18146  2.89801
 2.37887  2.77519  2.98518  4.58477  3.61503
          0.03133  3.87472  4.59707  0.61958  0.77255  0.48576  0.95510
    125   2.89020  4.54547  3.62202  3.12493  2.46989  3.75322  2.68923
 3.05481  2.95540  2.18049  3.63792  3.42714  4.12479  3.30194  3.23956
 3.04561  3.11819  2.86777  4.08453  1.84138    125 y - - -
          2.68618  4.42225  2.77519  2.73123  3.46354  2.40513  3.72494
 3.29354  2.67741  2.69355  4.24690  2.90347  2.73739  3.18146  2.89801
 2.37887  2.77519  2.98518  4.58477  3.61503
          0.03133  3.87472  4.59707  0.61958  0.77255  0.48576  0.95510
    126   3.26109  4.66217  4.24182  3.95666  1.00804  3.95049  3.87804
 2.78442  3.86362  2.15392  3.51419  4.02166  4.40896  4.03095  3.98279
 3.53096  3.56220  2.77530  4.12630  2.49298    126 f - - -
          2.68618  4.42225  2.77519  2.73123  3.46354  2.40513  3.72494
 3.29354  2.67741  2.69355  4.24690  2.90347  2.73739  3.18146  2.89801
 2.37887  2.77519  2.98518  4.58477  3.61503
          0.03133  3.87472  4.59707  0.61958  0.77255  0.48576  0.95510
    127   3.18917  4.58166  4.48360  3.98950  2.22386  4.27329  4.31803
 2.26539  3.84777  1.07668  2.95761  4.20821  4.54601  4.01572  4.00900
 3.63763  3.42632  2.35027  4.64524  3.21688    127 l - - -
          2.68618  4.42225  2.77519  2.73123  3.46354  2.40513  3.72494
 3.29354  2.67741  2.69355  4.24690  2.90347  2.73739  3.18146  2.89801
 2.37887  2.77519  2.98518  4.58477  3.61503
          0.03133  3.87472  4.59707  0.61958  0.77255  0.48576  0.95510
    128   2.72579  5.08993  1.95629  2.16097  4.48057  3.17464  3.70261
 3.98345  2.64724  3.53493  4.36879  1.83751  3.76663  2.87048  3.17677
 2.43052  3.02766  3.57096  5.70465  4.26536    128 n - - -
          2.68618  4.42225  2.77519  2.73123  3.46354  2.40513  3.72494
 3.29354  2.67741  2.69355  4.24690  2.90347  2.73739  3.18146  2.89801
 2.37887  2.77519  2.98518  4.58477  3.61503
```

```
          0.03133  3.87472  4.59707  0.61958  0.77255  0.48576  0.95510
    129   2.78463  4.34550  3.93483  3.38678  3.24443  3.83024  4.18179
2.27694  3.23870  1.53935  2.55715  3.71833  4.19925  3.55486  3.50472
3.13948  2.50441  2.20648  4.99076  3.80529    129 l - - -
          2.68618  4.42225  2.77519  2.73123  3.46354  2.40513  3.72494
3.29354  2.67741  2.69355  4.24690  2.90347  2.73739  3.18146  2.89801
2.37887  2.77519  2.98518  4.58477  3.61503
          0.03133  3.87472  4.59707  0.61958  0.77255  0.48576  0.95510
    130   2.31597  4.23644  3.20823  3.02400  4.07846  2.96628  4.13384
3.60134  3.09793  3.32136  4.24719  3.24849  3.70524  3.44704  3.38396
1.08767  2.79625  3.13595  5.45075  4.16198    130 s - - -
          2.68618  4.42225  2.77519  2.73123  3.46354  2.40513  3.72494
3.29354  2.67741  2.69355  4.24690  2.90347  2.73739  3.18146  2.89801
2.37887  2.77519  2.98518  4.58477  3.61503
          0.03133  3.87472  4.59707  0.61958  0.77255  0.48576  0.95510
    131   2.50777  4.58375  2.64966  2.49499  4.30634  1.95258  3.87290
3.82530  2.78351  3.44351  4.29517  1.85625  3.73530  3.08779  3.19369
2.58355  2.90353  3.36577  5.57328  4.24487    131 n - - -
          2.68618  4.42225  2.77519  2.73123  3.46354  2.40513  3.72494
3.29354  2.67741  2.69355  4.24690  2.90347  2.73739  3.18146  2.89801
2.37887  2.77519  2.98518  4.58477  3.61503
          0.03133  3.87472  4.59707  0.61958  0.77255  0.48576  0.95510
    132   2.00873  4.55489  3.06780  2.62606  3.82508  3.35874  3.73368
3.14716  2.50405  2.57673  3.75079  3.07382  3.83793  2.30481  2.85768
2.68888  2.85963  2.88630  5.19536  3.90416    132 a - - -
          2.68618  4.42225  2.77519  2.73123  3.46354  2.40513  3.72494
3.29354  2.67741  2.69355  4.24690  2.90347  2.73739  3.18146  2.89801
2.37887  2.77519  2.98518  4.58477  3.61503
          0.03133  3.87472  4.59707  0.61958  0.77255  0.48576  0.95510
    133   3.13260  4.49754  4.57868  4.06001  3.16843  4.35367  4.71105
1.51681  3.88418  1.27239  3.01918  4.33673  4.62015  4.13416  4.07017
3.72128  3.37745  1.92247  5.17278  3.98634    133 l - - -
          2.68618  4.42225  2.77519  2.73123  3.46354  2.40513  3.72494
3.29354  2.67741  2.69355  4.24690  2.90347  2.73739  3.18146  2.89801
2.37887  2.77519  2.98518  4.58477  3.61503
          0.03133  3.87472  4.59707  0.61958  0.77255  0.48576  0.95510
    134   3.03284  4.45083  4.33904  3.95485  3.37561  4.06909  4.68249
1.07512  3.78303  1.96109  3.31052  4.19987  4.50000  4.13104  3.97934
3.56453  3.33193  1.84577  5.26697  4.00552    134 i - - -
          2.68618  4.42225  2.77519  2.73123  3.46354  2.40513  3.72494
3.29354  2.67741  2.69355  4.24690  2.90347  2.73739  3.18146  2.89801
2.37887  2.77519  2.98518  4.58477  3.61503
          0.03133  3.87472  4.59707  0.61958  0.77255  0.48576  0.95510
    135   2.62771  4.73912  2.77104  2.48166  4.14712  3.25161  3.71642
3.64734  2.43167  3.23321  4.10763  2.95195  3.80259  1.78897  2.77973
2.22273  2.93779  3.28748  5.39959  4.06062    135 q - - -
          2.68618  4.42225  2.77519  2.73123  3.46354  2.40513  3.72494
3.29354  2.67741  2.69355  4.24690  2.90347  2.73739  3.18146  2.89801
2.37887  2.77519  2.98518  4.58477  3.61503
          0.03133  3.87472  4.59707  0.61958  0.77255  0.48576  0.95510
    136   3.00784  4.84699  3.49870  3.00918  4.20214  3.49552  3.82565
3.74393  2.23793  3.25944  4.25948  3.36879  3.99792  3.04579  0.97495
3.09185  3.27359  3.46776  5.28422  4.12892    136 r - - -
```

```
         2.68618  4.42225  2.77519  2.73123  3.46354  2.40513  3.72494
3.29354  2.67741  2.69355  4.24690  2.90347  2.73739  3.18146  2.89801
2.37887  2.77519  2.98518  4.58477  3.61503
         0.03133  3.87472  4.59707  0.61958  0.77255  0.48576  0.95510
    137  3.15771  4.61926  4.19314  3.83291  3.13642  3.98506  4.48794
2.34049  3.60064  0.87242  3.15851  4.10818  4.41998  3.96843  3.77638
3.55606  3.45182  2.37843  5.01661  3.74048    137 l - - -
         2.68618  4.42225  2.77519  2.73123  3.46354  2.40513  3.72494
3.29354  2.67741  2.69355  4.24690  2.90347  2.73739  3.18146  2.89801
2.37887  2.77519  2.98518  4.58477  3.61503
         0.03133  3.87472  4.59707  0.61958  0.77255  0.48576  0.95510
    138  2.75420  4.51129  3.36160  3.20088  4.19604  3.19867  4.27823
3.74193  3.25835  3.36857  4.41844  3.50740  0.82346  3.64706  3.50371
2.92174  3.19592  3.38706  5.38207  4.31670    138 p - - -
         2.68618  4.42225  2.77519  2.73123  3.46354  2.40513  3.72494
3.29354  2.67741  2.69355  4.24690  2.90347  2.73739  3.18146  2.89801
2.37887  2.77519  2.98518  4.58477  3.61503
         0.03133  3.87472  4.59707  0.61958  0.77255  0.48576  0.95510
    139  2.88236  4.86831  2.90423  2.65889  4.03720  3.36741  3.81726
3.66821  2.47950  3.17034  4.19804  3.11869  3.92083  1.23004  2.76368
2.92996  3.17928  3.39771  5.30876  4.00263    139 q - - -
         2.68618  4.42225  2.77519  2.73123  3.46354  2.40513  3.72494
3.29354  2.67741  2.69355  4.24690  2.90347  2.73739  3.18146  2.89801
2.37887  2.77519  2.98518  4.58477  3.61503
         0.03133  3.87472  4.59707  0.61958  0.77255  0.48576  0.95510
    140  3.26109  4.66217  4.24182  3.95666  1.00804  3.95049  3.87804
2.78442  3.86362  2.15392  3.51419  4.02166  4.40896  4.03095  3.98279
3.53096  3.56220  2.77530  4.12630  2.49298    140 f - - -
         2.68618  4.42225  2.77519  2.73123  3.46354  2.40513  3.72494
3.29354  2.67741  2.69355  4.24690  2.90347  2.73739  3.18146  2.89801
2.37887  2.77519  2.98518  4.58477  3.61503
         0.03133  3.87472  4.59707  0.61958  0.77255  0.48576  0.95510
    141  2.95406  5.06580  2.48830  1.02217  4.41227  3.27540  3.86227
3.86661  2.74307  3.48333  4.45717  2.93393  3.88006  3.07782  3.15222
2.93617  3.25828  3.55019  5.58341  4.30555    141 e - - -
         2.68618  4.42225  2.77519  2.73123  3.46354  2.40513  3.72494
3.29354  2.67741  2.69355  4.24690  2.90347  2.73739  3.18146  2.89801
2.37887  2.77519  2.98518  4.58477  3.61503
         0.03133  3.87472  4.59707  0.61958  0.77255  0.48576  0.95510
    142  3.00216  4.36665  4.57319  4.07815  3.46025  4.26584  4.82000
1.36984  3.95490  1.97965  3.28692  4.32195  4.60862  4.23687  4.17224
3.64730  3.27408  1.30447  5.39903  4.18788    142 v - - -
         2.68618  4.42225  2.77519  2.73123  3.46354  2.40513  3.72494
3.29354  2.67741  2.69355  4.24690  2.90347  2.73739  3.18146  2.89801
2.37887  2.77519  2.98518  4.58477  3.61503
         0.03133  3.87472  4.59707  0.61958  0.77255  0.48576  0.95510
    143  1.85624  4.58883  2.90246  2.57797  4.05310  3.23668  3.77227
3.42035  2.52981  3.06716  3.97654  3.01988  3.79607  2.08989  2.88055
2.64389  2.88204  3.09390  5.36185  4.06128    143 a - - -
         2.68618  4.42225  2.77519  2.73123  3.46354  2.40513  3.72494
3.29354  2.67741  2.69355  4.24690  2.90347  2.73739  3.18146  2.89801
2.37887  2.77519  2.98518  4.58477  3.61503
         0.03133  3.87472  4.59707  0.61958  0.77255  0.48576  0.95510
```

```
    144   2.24646  4.18296  3.22843  2.96877  4.16694  1.81410  4.08771
3.50569  3.03291  3.24930  4.11825  3.20232  3.66949  3.35644  3.35043
2.41700  1.77991  3.04208  5.51053  4.28720    144 t - - -
          2.68618  4.42225  2.77519  2.73123  3.46354  2.40513  3.72494
3.29354  2.67741  2.69355  4.24690  2.90347  2.73739  3.18146  2.89801
2.37887  2.77519  2.98518  4.58477  3.61503
          0.03133  3.87472  4.59707  0.61958  0.77255  0.48576  0.95510
    145   3.25019  4.73827  3.84149  3.57122  2.29338  3.83270  3.61936
3.25908  3.42867  2.71244  3.93385  3.72612  4.30645  3.73417  3.61195
3.38669  3.53686  3.12972  3.93726  0.96205    145 y - - -
          2.68618  4.42225  2.77519  2.73123  3.46354  2.40513  3.72494
3.29354  2.67741  2.69355  4.24690  2.90347  2.73739  3.18146  2.89801
2.37887  2.77519  2.98518  4.58477  3.61503
          0.03133  3.87472  4.59707  0.61958  0.77255  0.48576  0.95510
    146   2.95406  5.06580  2.48830  1.02217  4.41227  3.27540  3.86227
3.86661  2.74307  3.48333  4.45717  2.93393  3.88006  3.07782  3.15222
2.93617  3.25828  3.55019  5.58341  4.30555    146 e - - -
          2.68618  4.42225  2.77519  2.73123  3.46354  2.40513  3.72494
3.29354  2.67741  2.69355  4.24690  2.90347  2.73739  3.18146  2.89801
2.37887  2.77519  2.98518  4.58477  3.61503
          0.03133  3.87472  4.59707  0.61958  0.77255  0.48576  0.95510
    147   2.66752  4.44283  3.34177  3.24141  4.38386  0.72564  4.36163
3.98394  3.42300  3.63781  4.61350  3.50605  3.83404  3.74696  3.65888
2.83948  3.14893  3.51182  5.47850  4.48580    147 g - - -
          2.68618  4.42225  2.77519  2.73123  3.46354  2.40513  3.72494
3.29354  2.67741  2.69355  4.24690  2.90347  2.73739  3.18146  2.89801
2.37887  2.77519  2.98518  4.58477  3.61503
          0.03133  3.87472  4.59707  0.61958  0.77255  0.48576  0.95510
    148   2.80983  4.35172  4.12850  3.79161  3.55163  3.72629  4.59291
2.04988  3.65943  2.23820  3.51634  3.98111  4.28661  4.01903  3.87173
3.22990  3.17128  1.02205  5.33648  4.06827    148 v - - -
          2.68618  4.42225  2.77519  2.73123  3.46354  2.40513  3.72494
3.29354  2.67741  2.69355  4.24690  2.90347  2.73739  3.18146  2.89801
2.37887  2.77519  2.98518  4.58477  3.61503
          0.03133  3.87472  4.59707  0.61958  0.77255  0.48576  0.95510
    149   2.31597  4.23644  3.20823  3.02400  4.07846  2.96628  4.13384
3.60134  3.09793  3.32136  4.24719  3.24849  3.70524  3.44704  3.38396
1.08767  2.79625  3.13595  5.45075  4.16198    149 s - - -
          2.68618  4.42225  2.77519  2.73123  3.46354  2.40513  3.72494
3.29354  2.67741  2.69355  4.24690  2.90347  2.73739  3.18146  2.89801
2.37887  2.77519  2.98518  4.58477  3.61503
          0.03133  3.87472  4.59707  0.61958  0.77255  0.48576  0.95510
    150   3.05640  4.43536  4.49930  4.00215  2.71040  4.23955  4.53861
1.27855  3.87965  1.75697  3.10917  4.23685  4.54968  4.09843  4.06717
3.60768  3.31009  1.90659  4.98160  3.65132    150 i - - -
          2.68618  4.42225  2.77519  2.73123  3.46354  2.40513  3.72494
3.29354  2.67741  2.69355  4.24690  2.90347  2.73739  3.18146  2.89801
2.37887  2.77519  2.98518  4.58477  3.61503
          0.03133  3.87472  4.59707  0.61958  0.77255  0.48576  0.95510
    151   1.54708  4.11323  3.32564  3.05485  4.16099  2.89955  4.13258
3.49519  3.10452  3.25496  4.11764  3.22863  3.64079  3.41146  3.41044
1.59771  2.66061  3.00969  5.53041  4.30053    151 a - - -
          2.68618  4.42225  2.77519  2.73123  3.46354  2.40513  3.72494
3.29354  2.67741  2.69355  4.24690  2.90347  2.73739  3.18146  2.89801
2.37887  2.77519  2.98518  4.58477  3.61503
```

```
          0.03133  3.87472  4.59707  0.61958  0.77255  0.48576  0.95510
    152   3.15771  4.61926  4.19314  3.83291  3.13642  3.98506  4.48794
2.34049  3.60064  0.87242  3.15851  4.10818  4.41998  3.96843  3.77638
3.55606  3.45182  2.37843  5.01661  3.74048    152 l - - -
          2.68618  4.42225  2.77519  2.73123  3.46354  2.40513  3.72494
3.29354  2.67741  2.69355  4.24690  2.90347  2.73739  3.18146  2.89801
2.37887  2.77519  2.98518  4.58477  3.61503
          0.03133  3.87472  4.59707  0.61958  0.77255  0.48576  0.95510
    153   3.26109  4.66217  4.24182  3.95666  1.00804  3.95049  3.87804
2.78442  3.86362  2.15392  3.51419  4.02166  4.40896  4.03095  3.98279
3.53096  3.56220  2.77530  4.12630  2.49298    153 f - - -
          2.68618  4.42225  2.77519  2.73123  3.46354  2.40513  3.72494
3.29354  2.67741  2.69355  4.24690  2.90347  2.73739  3.18146  2.89801
2.37887  2.77519  2.98518  4.58477  3.61503
          0.03133  3.87472  4.59707  0.61958  0.77255  0.48576  0.95510
    154   2.41377  4.25888  3.42992  3.18193  3.97070  3.09811  4.19910
3.09810  3.12495  2.96152  4.01182  3.39101  3.80201  3.51964  3.38358
2.61505  1.16810  2.79284  5.40831  4.18731    154 t - - -
          2.68618  4.42225  2.77519  2.73123  3.46354  2.40513  3.72494
3.29354  2.67741  2.69355  4.24690  2.90347  2.73739  3.18146  2.89801
2.37887  2.77519  2.98518  4.58477  3.61503
          0.03133  3.87472  4.59707  0.61958  0.77255  0.48576  0.95510
    155   2.88236  4.86831  2.90423  2.65889  4.03720  3.36741  3.81726
3.66821  2.47950  3.17034  4.19804  3.11869  3.92083  1.23004  2.76368
2.92996  3.17928  3.39771  5.30876  4.00263    155 q - - -
          2.68618  4.42225  2.77519  2.73123  3.46354  2.40513  3.72494
3.29354  2.67741  2.69355  4.24690  2.90347  2.73739  3.18146  2.89801
2.37887  2.77519  2.98518  4.58477  3.61503
          0.03133  3.87472  4.59707  0.61958  0.77255  0.48576  0.95510
    156   2.97087  4.51925  4.05178  3.64534  3.20855  3.83573  4.37996
2.38437  3.41332  1.80283  1.37362  3.92258  4.29629  3.79501  3.61839
3.32024  3.27619  2.39076  5.04971  3.81172    156 m - - -
          2.68618  4.42225  2.77519  2.73123  3.46354  2.40513  3.72494
3.29354  2.67741  2.69355  4.24690  2.90347  2.73739  3.18146  2.89801
2.37887  2.77519  2.98518  4.58477  3.61503
          0.03133  3.87472  4.59707  0.61958  0.77255  0.48576  0.95510
    157   2.58861  0.93589  4.10823  3.84832  3.83906  3.21118  4.49490
2.98326  3.67524  2.89868  4.02000  3.83470  3.91832  4.02124  3.79925
2.86028  3.05555  2.72771  5.25480  4.12675    157 c - - -
          2.68618  4.42225  2.77519  2.73123  3.46354  2.40513  3.72494
3.29354  2.67741  2.69355  4.24690  2.90347  2.73739  3.18146  2.89801
2.37887  2.77519  2.98518  4.58477  3.61503
          0.03133  3.87472  4.59707  0.61958  0.77255  0.48576  0.95510
    158   2.25539  4.16708  3.31426  3.03279  4.04742  2.98041  4.09081
3.30680  3.01484  3.12057  4.02873  3.24317  3.69452  3.37510  3.30804
2.18832  1.42096  2.90244  5.44335  4.19675    158 t - - -
          2.68618  4.42225  2.77519  2.73123  3.46354  2.40513  3.72494
3.29354  2.67741  2.69355  4.24690  2.90347  2.73739  3.18146  2.89801
2.37887  2.77519  2.98518  4.58477  3.61503
          0.03133  3.87472  4.59707  0.61958  0.77255  0.48576  0.95510
    159   3.18917  4.58166  4.48360  3.98950  2.22386  4.27329  4.31803
2.26539  3.84777  1.07668  2.95761  4.20821  4.54601  4.01572  4.00900
3.63763  3.42632  2.35027  4.64524  3.21688    159 l - - -
```

2.68618 4.42225 2.77519 2.73123 3.46354 2.40513 3.72494
3.29354 2.67741 2.69355 4.24690 2.90347 2.73739 3.18146 2.89801
2.37887 2.77519 2.98518 4.58477 3.61503
0.03133 3.87472 4.59707 0.61958 0.77255 0.48576 0.95510
160 2.99708 4.81426 3.12768 2.88777 3.28613 3.44925 1.13776
3.70036 2.73075 3.19407 4.23713 3.29704 4.00186 3.25401 2.99637
3.06772 3.30049 3.43543 4.75116 3.23690 160 h - - -
2.68618 4.42225 2.77519 2.73123 3.46354 2.40513 3.72494
3.29354 2.67741 2.69355 4.24690 2.90347 2.73739 3.18146 2.89801
2.37887 2.77519 2.98518 4.58477 3.61503
0.03133 3.87472 4.59707 0.61958 0.77255 0.48576 0.95510
161 3.15771 4.61926 4.19314 3.83291 3.13642 3.98506 4.48794
2.34049 3.60064 0.87242 3.15851 4.10818 4.41998 3.96843 3.77638
3.55606 3.45182 2.37843 5.01661 3.74048 161 l - - -
2.68618 4.42225 2.77519 2.73123 3.46354 2.40513 3.72494
3.29354 2.67741 2.69355 4.24690 2.90347 2.73739 3.18146 2.89801
2.37887 2.77519 2.98518 4.58477 3.61503
0.03133 3.87472 4.59707 0.61958 0.77255 0.48576 0.95510
162 2.24646 4.18296 3.22843 2.96877 4.16694 1.81410 4.08771
3.50569 3.03291 3.24930 4.11825 3.20232 3.66949 3.35644 3.35043
2.41700 1.77991 3.04208 5.51053 4.28720 162 t - - -
2.68618 4.42225 2.77519 2.73123 3.46354 2.40513 3.72494
3.29354 2.67741 2.69355 4.24690 2.90347 2.73739 3.18146 2.89801
2.37887 2.77519 2.98518 4.58477 3.61503
0.03133 3.87472 4.59707 0.61958 0.77255 0.48576 0.95510
163 3.15771 4.61926 4.19314 3.83291 3.13642 3.98506 4.48794
2.34049 3.60064 0.87242 3.15851 4.10818 4.41998 3.96843 3.77638
3.55606 3.45182 2.37843 5.01661 3.74048 163 l - - -
2.68618 4.42225 2.77519 2.73123 3.46354 2.40513 3.72494
3.29354 2.67741 2.69355 4.24690 2.90347 2.73739 3.18146 2.89801
2.37887 2.77519 2.98518 4.58477 3.61503
0.03133 3.87472 4.59707 0.61958 0.77255 0.48576 0.95510
164 3.15771 4.61926 4.19314 3.83291 3.13642 3.98506 4.48794
2.34049 3.60064 0.87242 3.15851 4.10818 4.41998 3.96843 3.77638
3.55606 3.45182 2.37843 5.01661 3.74048 164 l - - -
2.68618 4.42225 2.77519 2.73123 3.46354 2.40513 3.72494
3.29354 2.67741 2.69355 4.24690 2.90347 2.73739 3.18146 2.89801
2.37887 2.77519 2.98518 4.58477 3.61503
0.03133 3.87472 4.59707 0.61958 0.77255 0.48576 0.95510
165 2.97404 4.93809 3.15019 2.77112 4.32503 3.46335 3.76023
3.75442 1.03520 3.31897 4.28021 3.19552 3.96346 2.95151 2.43176
3.01582 3.22709 3.46205 5.37869 4.19175 165 k - - -
2.68618 4.42225 2.77519 2.73123 3.46354 2.40513 3.72494
3.29354 2.67741 2.69355 4.24690 2.90347 2.73739 3.18146 2.89801
2.37887 2.77519 2.98518 4.58477 3.61503
0.03133 3.87472 4.59707 0.61958 0.77255 0.48576 0.95510
166 2.90809 5.37219 1.24767 1.90909 4.66886 3.16987 3.74890
4.16707 2.78244 3.72123 4.60622 2.66498 3.80382 2.93069 3.36155
2.80724 3.20795 3.76993 5.87153 4.40116 166 d - - -
2.68618 4.42225 2.77519 2.73123 3.46354 2.40513 3.72494
3.29354 2.67741 2.69355 4.24690 2.90347 2.73739 3.18146 2.89801
2.37887 2.77519 2.98518 4.58477 3.61503
0.03133 3.87472 4.59707 0.61958 0.77255 0.48576 0.95510

167 2.66752 4.44283 3.34177 3.24141 4.38386 0.72564 4.36163
3.98394 3.42300 3.63781 4.61350 3.50605 3.83404 3.74696 3.65888
2.83948 3.14893 3.51182 5.47850 4.48580 167 g - - -
2.68618 4.42225 2.77519 2.73123 3.46354 2.40513 3.72494
3.29354 2.67741 2.69355 4.24690 2.90347 2.73739 3.18146 2.89801
2.37887 2.77519 2.98518 4.58477 3.61503
0.03133 3.87472 4.59707 0.61958 0.77255 0.48576 0.95510
168 3.03284 4.45083 4.33904 3.95485 3.37561 4.06909 4.68249
1.07512 3.78303 1.96109 3.31052 4.19987 4.50000 4.13104 3.97934
3.56453 3.33193 1.84577 5.26697 4.00552 168 i - - -
2.68618 4.42225 2.77519 2.73123 3.46354 2.40513 3.72494
3.29354 2.67741 2.69355 4.24690 2.90347 2.73739 3.18146 2.89801
2.37887 2.77519 2.98518 4.58477 3.61503
0.03133 3.87472 4.59707 0.61958 0.77255 0.48576 0.95510
169 3.15771 4.61926 4.19314 3.83291 3.13642 3.98506 4.48794
2.34049 3.60064 0.87242 3.15851 4.10818 4.41998 3.96843 3.77638
3.55606 3.45182 2.37843 5.01661 3.74048 169 l - - -
2.68618 4.42225 2.77519 2.73123 3.46354 2.40513 3.72494
3.29354 2.67741 2.69355 4.24690 2.90347 2.73739 3.18146 2.89801
2.37887 2.77519 2.98518 4.58477 3.61503
0.03133 3.87472 4.59707 0.61958 0.77255 0.48576 0.95510
170 1.01052 4.19303 3.46476 3.25606 4.05451 3.01753 4.27786
3.18035 3.27238 3.06187 4.09337 3.40514 3.75695 3.61331 3.52281
2.54327 2.82077 2.83436 5.48144 4.28192 170 a - - -
2.68618 4.42225 2.77519 2.73123 3.46354 2.40513 3.72494
3.29354 2.67741 2.69355 4.24690 2.90347 2.73739 3.18146 2.89801
2.37887 2.77519 2.98518 4.58477 3.61503
0.03133 3.87472 4.59707 0.61958 0.77255 0.48576 0.95510
171 2.66752 4.44283 3.34177 3.24141 4.38386 0.72564 4.36163
3.98394 3.42300 3.63781 4.61350 3.50605 3.83404 3.74696 3.65888
2.83948 3.14893 3.51182 5.47850 4.48580 171 g - - -
2.68618 4.42225 2.77519 2.73123 3.46354 2.40513 3.72494
3.29354 2.67741 2.69355 4.24690 2.90347 2.73739 3.18146 2.89801
2.37887 2.77519 2.98518 4.58477 3.61503
0.03133 3.87472 4.59707 0.61958 0.77255 0.48576 0.95510
172 2.31597 4.23644 3.20823 3.02400 4.07846 2.96628 4.13384
3.60134 3.09793 3.32136 4.24719 3.24849 3.70524 3.44704 3.38396
1.08767 2.79625 3.13595 5.45075 4.16198 172 s - - -
2.68618 4.42225 2.77519 2.73123 3.46354 2.40513 3.72494
3.29354 2.67741 2.69355 4.24690 2.90347 2.73739 3.18146 2.89801
2.37887 2.77519 2.98518 4.58477 3.61503
0.03133 3.87472 4.59707 0.61958 0.77255 0.48576 0.95510
173 1.87330 4.78525 1.76738 2.32425 4.31811 3.15181 3.78828
3.66272 2.73994 3.35864 4.23321 2.83855 3.76327 2.97707 3.23124
2.64144 2.95332 3.28753 5.60566 4.23215 173 d - - -
2.68618 4.42225 2.77519 2.73123 3.46354 2.40513 3.72494
3.29354 2.67741 2.69355 4.24690 2.90347 2.73739 3.18146 2.89801
2.37887 2.77519 2.98518 4.58477 3.61503
0.03133 3.87472 4.59707 0.61958 0.77255 0.48576 0.95510
174 3.33912 4.71305 4.09447 3.80105 2.66830 3.73256 3.90238
3.36273 3.52318 2.75727 3.99643 3.94963 4.25045 3.91067 3.65116
3.54280 3.63961 3.25312 0.88077 2.67027 174 w - - -
2.68618 4.42225 2.77519 2.73123 3.46354 2.40513 3.72494
3.29354 2.67741 2.69355 4.24690 2.90347 2.73739 3.18146 2.89801
2.37887 2.77519 2.98518 4.58477 3.61503

```
        0.03133  3.87472  4.59707  0.61958  0.77255  0.48576  0.95510
    175   2.66752  4.44283  3.34177  3.24141  4.38386  0.72564  4.36163
3.98394  3.42300  3.63781  4.61350  3.50605  3.83404  3.74696  3.65888
2.83948  3.14893  3.51182  5.47850  4.48580    175 g - - -
        2.68618  4.42225  2.77519  2.73123  3.46354  2.40513  3.72494
3.29354  2.67741  2.69355  4.24690  2.90347  2.73739  3.18146  2.89801
2.37887  2.77519  2.98518  4.58477  3.61503
        0.03133  3.87472  4.59707  0.61958  0.77255  0.48576  0.95510
    176   3.26109  4.66217  4.24182  3.95666  1.00804  3.95049  3.87804
2.78442  3.86362  2.15392  3.51419  4.02166  4.40896  4.03095  3.98279
3.53096  3.56220  2.77530  4.12630  2.49298    176 f - - -
        2.68618  4.42225  2.77519  2.73123  3.46354  2.40513  3.72494
3.29354  2.67741  2.69355  4.24690  2.90347  2.73739  3.18146  2.89801
2.37887  2.77519  2.98518  4.58477  3.61503
        0.03133  3.87472  4.59707  0.61958  0.77255  0.48576  0.95510
    177   1.85423  4.13978  3.43371  3.12002  4.02632  3.00103  4.14524
3.10455  3.09284  3.01001  3.95754  3.30172  3.71314  3.43318  3.38419
2.45130  1.54820  2.74686  5.45742  4.23865    177 t - - -
        2.68618  4.42225  2.77519  2.73123  3.46354  2.40513  3.72494
3.29354  2.67741  2.69355  4.24690  2.90347  2.73739  3.18146  2.89801
2.37887  2.77519  2.98518  4.58477  3.61503
        0.03133  3.87472  4.59707  0.61958  0.77255  0.48576  0.95510
    178   2.65416  4.75935  2.79425  2.48615  4.13906  3.28052  3.71875
3.61434  2.44474  3.18456  4.07351  2.96536  2.25767  2.03259  2.80172
2.70064  2.95071  3.27511  5.38597  4.06994    178 q - - -
        2.68618  4.42225  2.77519  2.73123  3.46354  2.40513  3.72494
3.29354  2.67741  2.69355  4.24690  2.90347  2.73739  3.18146  2.89801
2.37887  2.77519  2.98518  4.58477  3.61503
        0.03133  3.87472  4.59707  0.61958  0.77255  0.48576  0.95510
    179   1.63150  4.60790  2.73637  2.09241  4.11655  3.20416  3.81151
3.37759  2.67001  3.12523  4.02800  2.97154  3.79090  3.01916  3.06754
2.65182  2.90513  3.05265  5.43818  4.13215    179 a - - -
        2.68618  4.42225  2.77519  2.73123  3.46354  2.40513  3.72494
3.29354  2.67741  2.69355  4.24690  2.90347  2.73739  3.18146  2.89801
2.37887  2.77519  2.98518  4.58477  3.61503
        0.03133  3.87472  4.59707  0.61958  0.77255  0.48576  0.95510
    180   2.99187  5.12073  0.91483  2.33607  4.49801  3.21224  3.93281
4.08666  3.01732  3.69455  4.67262  2.89258  3.86518  3.17190  3.53391
2.95391  3.33046  3.72558  5.66619  4.37043    180 d - - -
        2.68618  4.42225  2.77519  2.73123  3.46354  2.40513  3.72494
3.29354  2.67741  2.69355  4.24690  2.90347  2.73739  3.18146  2.89801
2.37887  2.77519  2.98518  4.58477  3.61503
        0.03133  3.87472  4.59707  0.61958  0.77255  0.48576  0.95510
    181   2.67728  4.55205  3.27661  2.79229  3.80802  3.47911  3.79410
2.87068  1.86218  2.73405  3.72559  3.20929  3.93514  3.01680  2.75250
2.82640  2.94321  1.99960  5.19668  3.93287    181 k - - -
        2.68618  4.42225  2.77519  2.73123  3.46354  2.40513  3.72494
3.29354  2.67741  2.69355  4.24690  2.90347  2.73739  3.18146  2.89801
2.37887  2.77519  2.98518  4.58477  3.61503
        0.03133  3.87472  4.59707  0.61958  0.77255  0.48576  0.95510
    182   2.99187  5.12073  0.91483  2.33607  4.49801  3.21224  3.93281
4.08666  3.01732  3.69455  4.67262  2.89258  3.86518  3.17190  3.53391
2.95391  3.33046  3.72558  5.66619  4.37043    182 d - - -
```

```
         2.68618  4.42225  2.77519  2.73123  3.46354  2.40513  3.72494
3.29354  2.67741  2.69355  4.24690  2.90347  2.73739  3.18146  2.89801
2.37887  2.77519  2.98518  4.58477  3.61503
         0.03133  3.87472  4.59707  0.61958  0.77255  0.48576  0.95510
    183  1.79284  4.12837  3.28700  3.03147  4.17519  2.90135  4.12712
3.52080  3.09089  3.27553  4.14101  3.21717  3.64347  3.40321  3.39815
1.39117  2.67087  3.03109  5.54189  4.30404    183 s - - -
         2.68618  4.42225  2.77519  2.73123  3.46354  2.40513  3.72494
3.29354  2.67741  2.69355  4.24690  2.90347  2.73739  3.18146  2.89801
2.37887  2.77519  2.98518  4.58477  3.61503
         0.03133  3.87472  4.59707  0.61958  0.77255  0.48576  0.95510
    184  3.18403  4.57154  4.47372  3.98795  1.87152  4.24783  4.19630
2.31815  3.84659  1.23466  3.00592  4.16891  4.52942  4.00186  3.99866
3.60949  3.42091  2.39020  4.49778  3.00668    184 l - - -
         2.68618  4.42225  2.77519  2.73123  3.46354  2.40513  3.72494
3.29354  2.67741  2.69355  4.24690  2.90347  2.73739  3.18146  2.89801
2.37887  2.77519  2.98518  4.58477  3.61503
         0.03133  3.87472  4.59707  0.61958  0.77255  0.48576  0.95510
    185  3.03284  4.45083  4.33904  3.95485  3.37561  4.06909  4.68249
1.07512  3.78303  1.96109  3.31052  4.19987  4.50000  4.13104  3.97934
3.56453  3.33193  1.84577  5.26697  4.00552    185 i - - -
         2.68618  4.42225  2.77519  2.73123  3.46354  2.40513  3.72494
3.29354  2.67741  2.69355  4.24690  2.90347  2.73739  3.18146  2.89801
2.37887  2.77519  2.98518  4.58477  3.61503
         0.03133  3.87472  4.59707  0.61958  0.77255  0.48576  0.95510
    186  2.55816  2.35965  3.40466  2.89795  3.77570  3.35109  3.83091
3.04219  1.87338  2.80922  3.73700  3.25255  3.86556  3.08952  2.83198
2.72864  2.86769  2.77510  5.13498  3.89905    186 k - - -
         2.68618  4.42225  2.77519  2.73123  3.46354  2.40513  3.72494
3.29354  2.67741  2.69355  4.24690  2.90347  2.73739  3.18146  2.89801
2.37887  2.77519  2.98518  4.58477  3.61503
         0.03133  3.87472  4.59707  0.61958  0.77255  0.48576  0.95510
    187  2.77842  4.64204  3.25275  2.80511  3.52047  3.56144  3.79764
2.94114  2.52974  1.85244  3.55848  3.23929  3.99018  2.11718  2.82488
2.91397  3.02469  2.81217  5.04816  3.72442    187 l - - -
         2.68618  4.42225  2.77519  2.73123  3.46354  2.40513  3.72494
3.29354  2.67741  2.69355  4.24690  2.90347  2.73739  3.18146  2.89801
2.37887  2.77519  2.98518  4.58477  3.61503
         0.03133  3.87472  4.59707  0.61958  0.77255  0.48576  0.95510
    188  3.26109  4.66217  4.24182  3.95666  1.00804  3.95049  3.87804
2.78442  3.86362  2.15392  3.51419  4.02166  4.40896  4.03095  3.98279
3.53096  3.56220  2.77530  4.12630  2.49298    188 f - - -
         2.68618  4.42225  2.77519  2.73123  3.46354  2.40513  3.72494
3.29354  2.67741  2.69355  4.24690  2.90347  2.73739  3.18146  2.89801
2.37887  2.77519  2.98518  4.58477  3.61503
         0.03133  3.87472  4.59707  0.61958  0.77255  0.48576  0.95510
    189  2.72515  4.72193  2.70930  2.59495  4.08579  3.18536  3.92257
3.79656  2.82640  3.44315  4.39831  1.15407  3.83729  3.19525  3.18120
2.79220  3.10703  3.41854  5.39143  4.03390    189 n - - -
         2.68618  4.42225  2.77519  2.73123  3.46354  2.40513  3.72494
3.29354  2.67741  2.69355  4.24690  2.90347  2.73739  3.18146  2.89801
2.37887  2.77519  2.98518  4.58477  3.61503
         0.03133  3.87472  4.59707  0.61958  0.77255  0.48576  0.95510
```

```
    190   2.87154  4.98989  3.16094  2.63265  4.31885  3.51165  3.59460
3.75403  2.00002  3.24453  4.12584  3.07307  3.92443  2.00617  1.70295
2.88434  3.07732  3.44183  5.35263  4.11135    190 r - - -
         2.68618  4.42225  2.77519  2.73123  3.46354  2.40513  3.72494
3.29354  2.67741  2.69355  4.24690  2.90347  2.73739  3.18146  2.89801
2.37887  2.77519  2.98518  4.58477  3.61503
         0.03133  3.87472  4.59707  0.61958  0.77255  0.48576  0.95510
    191   2.69251  4.48549  3.36205  2.83058  2.53100  3.57666  3.63963
2.98085  2.09046  2.60386  3.56486  3.23552  3.96055  3.07186  3.02017
2.84359  2.92260  2.76671  4.54186  2.55305    191 k - - -
         2.68618  4.42225  2.77519  2.73123  3.46354  2.40513  3.72494
3.29354  2.67741  2.69355  4.24690  2.90347  2.73739  3.18146  2.89801
2.37887  2.77519  2.98518  4.58477  3.61503
         0.03133  3.87472  4.59707  0.61958  0.77255  0.48576  0.95510
    192   2.80983  4.35172  4.12850  3.79161  3.55163  3.72629  4.59291
2.04988  3.65943  2.23820  3.51634  3.98111  4.28661  4.01903  3.87173
3.22990  3.17128  1.02205  5.33648  4.06827    192 v - - -
         2.68618  4.42225  2.77519  2.73123  3.46354  2.40513  3.72494
3.29354  2.67741  2.69355  4.24690  2.90347  2.73739  3.18146  2.89801
2.37887  2.77519  2.98518  4.58477  3.61503
         0.03133  3.87472  4.59707  0.61958  0.77255  0.48576  0.95510
    193   2.65697  4.54375  3.08884  2.73239  3.55206  3.40592  3.79893
3.00005  2.66368  1.98246  3.72766  2.12578  3.90853  3.08839  2.99856
2.79268  2.94765  2.78000  5.03376  3.65044    193 l - - -
         2.68618  4.42225  2.77519  2.73123  3.46354  2.40513  3.72494
3.29354  2.67741  2.69355  4.24690  2.90347  2.73739  3.18146  2.89801
2.37887  2.77519  2.98518  4.58477  3.61503
         0.03133  3.87472  4.59707  0.61958  0.77255  0.48576  0.95510
    194   2.71959  5.09695  1.97847  1.71328  4.38197  3.24654  3.65050
3.79351  2.53733  3.38801  4.22177  2.74171  3.77622  2.80396  3.05117
2.67743  2.98309  3.02682  5.60133  4.18526    194 e - - -
         2.68618  4.42225  2.77519  2.73123  3.46354  2.40513  3.72494
3.29354  2.67741  2.69355  4.24690  2.90347  2.73739  3.18146  2.89801
2.37887  2.77519  2.98518  4.58477  3.61503
         0.03133  3.87472  4.59707  0.61958  0.77255  0.48576  0.95510
    195   3.25019  4.73827  3.84149  3.57122  2.29338  3.83270  3.61936
3.25908  3.42867  2.71244  3.93385  3.72612  4.30645  3.73417  3.61195
3.38669  3.53686  3.12972  3.93726  0.96205    195 y - - -
         2.68618  4.42225  2.77519  2.73123  3.46354  2.40513  3.72494
3.29354  2.67741  2.69355  4.24690  2.90347  2.73739  3.18146  2.89801
2.37887  2.77519  2.98518  4.58477  3.61503
         0.03133  3.87472  4.59707  0.61958  0.77255  0.48576  0.95510
    196   2.74188  4.87680  2.92934  2.53062  4.23161  3.37421  3.64456
3.72100  2.20028  3.26982  4.12887  2.06203  3.85541  2.81153  1.87165
2.76750  2.99960  3.37467  5.37191  4.07217    196 r - - -
         2.68618  4.42225  2.77519  2.73123  3.46354  2.40513  3.72494
3.29354  2.67741  2.69355  4.24690  2.90347  2.73739  3.18146  2.89801
2.37887  2.77519  2.98518  4.58477  3.61503
         0.03133  3.87472  4.59707  0.61958  0.77255  0.48576  0.95510
    197   2.41377  4.25888  3.42992  3.18193  3.97070  3.09811  4.19910
3.09810  3.12495  2.96152  4.01182  3.39101  3.80201  3.51964  3.38358
2.61505  1.16810  2.79284  5.40831  4.18731    197 t - - -
         2.68618  4.42225  2.77519  2.73123  3.46354  2.40513  3.72494
3.29354  2.67741  2.69355  4.24690  2.90347  2.73739  3.18146  2.89801
2.37887  2.77519  2.98518  4.58477  3.61503
```

```
         0.03133  3.87472  4.59707  0.61958  0.77255  0.48576  0.95510
    198  3.00784  4.84699  3.49870  3.00918  4.20214  3.49552  3.82565
3.74393  2.23793  3.25944  4.25948  3.36879  3.99792  3.04579  0.97495
3.09185  3.27359  3.46776  5.28422  4.12892    198 r - - -
         2.68618  4.42225  2.77519  2.73123  3.46354  2.40513  3.72494
3.29354  2.67741  2.69355  4.24690  2.90347  2.73739  3.18146  2.89801
2.37887  2.77519  2.98518  4.58477  3.61503
         0.03133  3.87472  4.59707  0.61958  0.77255  0.48576  0.95510
    199  3.15232  4.57441  4.45356  3.91728  3.04025  4.28136  4.57382
2.16430  3.71139  1.04946  2.32819  4.22264  4.54243  3.96798  3.90107
3.63395  3.38870  2.25096  5.05401  3.89822    199 l - - -
         2.68618  4.42225  2.77519  2.73123  3.46354  2.40513  3.72494
3.29354  2.67741  2.69355  4.24690  2.90347  2.73739  3.18146  2.89801
2.37887  2.77519  2.98518  4.58477  3.61503
         0.03133  3.87472  4.59707  0.61958  0.77255  0.48576  0.95510
    200  2.97087  4.51925  4.05178  3.64534  3.20855  3.83573  4.37996
2.38437  3.41332  1.80283  1.37362  3.92258  4.29629  3.79501  3.61839
3.32024  3.27619  2.39076  5.04971  3.81172    200 m - - -
         2.68618  4.42225  2.77519  2.73123  3.46354  2.40513  3.72494
3.29354  2.67741  2.69355  4.24690  2.90347  2.73739  3.18146  2.89801
2.37887  2.77519  2.98518  4.58477  3.61503
         0.03133  3.87472  4.59707  0.61958  0.77255  0.48576  0.95510
    201  2.53041  4.49415  3.19502  2.68697  3.88314  2.68108  3.72838
3.22558  2.44839  2.90972  3.78915  3.10248  3.81381  2.93845  2.10024
2.64970  2.82351  2.45138  5.19680  3.93152    201 r - - -
         2.68618  4.42225  2.77519  2.73123  3.46354  2.40513  3.72494
3.29354  2.67741  2.69355  4.24690  2.90347  2.73739  3.18146  2.89801
2.37887  2.77519  2.98518  4.58477  3.61503
         0.03133  3.87472  4.59707  0.61958  0.77255  0.48576  0.95510
    202  3.12595  4.55548  4.42280  3.88366  3.04497  4.25029  4.54412
2.17463  3.68325  1.17096  2.02793  4.18871  4.51787  3.94082  3.87635
3.59802  3.36223  2.25546  5.04224  3.88701    202 l - - -
         2.68618  4.42225  2.77519  2.73123  3.46354  2.40513  3.72494
3.29354  2.67741  2.69355  4.24690  2.90347  2.73739  3.18146  2.89801
2.37887  2.77519  2.98518  4.58477  3.61503
         0.03133  3.87472  4.59707  0.61958  0.77255  0.48576  0.95510
    203  3.25019  4.73827  3.84149  3.57122  2.29338  3.83270  3.61936
3.25908  3.42867  2.71244  3.93385  3.72612  4.30645  3.73417  3.61195
3.38669  3.53686  3.12972  3.93726  0.96205    203 y - - -
         2.68618  4.42225  2.77519  2.73123  3.46354  2.40513  3.72494
3.29354  2.67741  2.69355  4.24690  2.90347  2.73739  3.18146  2.89801
2.37887  2.77519  2.98518  4.58477  3.61503
         0.03133  3.87472  4.59707  0.61958  0.77255  0.48576  0.95510
    204  2.22657  4.16201  3.28998  2.98468  4.10060  2.95400  4.06371
3.43118  2.99359  3.18437  4.05455  3.20672  3.66653  3.33102  3.30912
1.65035  1.82092  2.98410  5.46782  4.22606    204 s - - -
         2.68618  4.42225  2.77519  2.73123  3.46354  2.40513  3.72494
3.29354  2.67741  2.69355  4.24690  2.90347  2.73739  3.18146  2.89801
2.37887  2.77519  2.98518  4.58477  3.61503
         0.03133  3.87472  4.59707  0.61958  0.77255  0.48576  0.95510
    205  2.61872  4.74430  2.87838  2.09410  3.99192  3.07498  3.61986
2.81028  2.13210  3.00068  3.84505  2.93491  3.80646  2.79001  2.77645
2.66391  2.85428  3.06080  5.25952  3.94878    205 e - - -
```

2.68618 4.42225 2.77519 2.73123 3.46354 2.40513 3.72494
3.29354 2.67741 2.69355 4.24690 2.90347 2.73739 3.18146 2.89801
2.37887 2.77519 2.98518 4.58477 3.61503
0.03133 3.87472 4.59707 0.61958 0.77255 0.48576 0.95510
206 2.95406 5.06580 2.48830 1.02217 4.41227 3.27540 3.86227
3.86661 2.74307 3.48333 4.45717 2.93393 3.88006 3.07782 3.15222
2.93617 3.25828 3.55019 5.58341 4.30555 206 e - - -
2.68618 4.42225 2.77519 2.73123 3.46354 2.40513 3.72494
3.29354 2.67741 2.69355 4.24690 2.90347 2.73739 3.18146 2.89801
2.37887 2.77519 2.98518 4.58477 3.61503
0.03133 3.87472 4.59707 0.61958 0.77255 0.48576 0.95510
207 3.26109 4.66217 4.24182 3.95666 1.00804 3.95049 3.87804
2.78442 3.86362 2.15392 3.51419 4.02166 4.40896 4.03095 3.98279
3.53096 3.56220 2.77530 4.12630 2.49298 207 f - - -
2.68618 4.42225 2.77519 2.73123 3.46354 2.40513 3.72494
3.29354 2.67741 2.69355 4.24690 2.90347 2.73739 3.18146 2.89801
2.37887 2.77519 2.98518 4.58477 3.61503
0.03133 3.87472 4.59707 0.61958 0.77255 0.48576 0.95510
208 2.66752 4.44283 3.34177 3.24141 4.38386 0.72564 4.36163
3.98394 3.42300 3.63781 4.61350 3.50605 3.83404 3.74696 3.65888
2.83948 3.14893 3.51182 5.47850 4.48580 208 g - - -
2.68618 4.42225 2.77519 2.73123 3.46354 2.40513 3.72494
3.29354 2.67741 2.69355 4.24690 2.90347 2.73739 3.18146 2.89801
2.37887 2.77519 2.98518 4.58477 3.61503
0.03133 3.87472 4.59707 0.61958 0.77255 0.48576 0.95510
209 3.00784 4.84699 3.49870 3.00918 4.20214 3.49552 3.82565
3.74393 2.23793 3.25944 4.25948 3.36879 3.99792 3.04579 0.97495
3.09185 3.27359 3.46776 5.28422 4.12892 209 r - - -
2.68618 4.42225 2.77519 2.73123 3.46354 2.40513 3.72494
3.29354 2.67741 2.69355 4.24690 2.90347 2.73739 3.18146 2.89801
2.37887 2.77519 2.98518 4.58477 3.61503
0.03133 3.87472 4.59707 0.61958 0.77255 0.48576 0.95510
210 3.15771 4.61926 4.19314 3.83291 3.13642 3.98506 4.48794
2.34049 3.60064 0.87242 3.15851 4.10818 4.41998 3.96843 3.77638
3.55606 3.45182 2.37843 5.01661 3.74048 210 l - - -
2.68618 4.42225 2.77519 2.73123 3.46354 2.40513 3.72494
3.29354 2.67741 2.69355 4.24690 2.90347 2.73739 3.18146 2.89801
2.37887 2.77519 2.98518 4.58477 3.61503
0.03133 3.87472 4.59707 0.61958 0.77255 0.48576 0.95510
211 2.75848 2.77904 4.20314 3.65234 2.75515 3.84904 4.18794
2.22238 3.52144 1.52033 3.10620 3.86440 4.22559 3.74838 3.71410
3.17572 3.01575 2.12928 4.77190 3.48828 211 l - - -
2.68618 4.42225 2.77519 2.73123 3.46354 2.40513 3.72494
3.29354 2.67741 2.69355 4.24690 2.90347 2.73739 3.18146 2.89801
2.37887 2.77519 2.98518 4.58477 3.61503
0.03133 3.87472 4.59707 0.61958 0.77255 0.48576 0.95510
212 1.88320 4.62706 2.99898 2.61446 4.12355 3.28795 3.74107
3.41772 1.81624 3.10727 3.99583 3.04820 3.81974 2.92848 2.71073
2.68131 2.90406 3.09294 5.36721 4.09649 212 k - - -
2.68618 4.42225 2.77519 2.73123 3.46354 2.40513 3.72494
3.29354 2.67741 2.69355 4.24690 2.90347 2.73739 3.18146 2.89801
2.37887 2.77519 2.98518 4.58477 3.61503
0.03133 3.87472 4.59707 0.61958 0.77255 0.48576 0.95510

213 2.67728 4.55205 3.27661 2.79229 3.80802 3.47911 3.79410
2.87068 1.86218 2.73405 3.72559 3.20929 3.93514 3.01680 2.75250
2.82640 2.94321 1.99960 5.19668 3.93287 213 k - - -
2.68618 4.42225 2.77519 2.73123 3.46354 2.40513 3.72494
3.29354 2.67741 2.69355 4.24690 2.90347 2.73739 3.18146 2.89801
2.37887 2.77519 2.98518 4.58477 3.61503
0.03133 3.87472 4.59707 0.61958 0.77255 0.48576 0.95510
214 2.60419 4.76123 2.82444 2.45047 4.19513 3.28045 3.66236
3.64715 2.35780 3.22530 4.05721 2.11719 3.78694 2.82764 2.33662
2.28077 2.88966 3.27839 5.39881 4.07755 214 n - - -
2.68618 4.42225 2.77519 2.73123 3.46354 2.40513 3.72494
3.29354 2.67741 2.69355 4.24690 2.90347 2.73739 3.18146 2.89801
2.37887 2.77519 2.98518 4.58477 3.61503
0.03133 3.87472 4.59707 0.61958 0.77255 0.48576 0.95510
215 3.05452 4.51911 4.34091 3.86695 3.13648 4.11972 4.55369
2.11173 3.66069 1.07297 3.03872 4.14008 4.48165 3.96740 3.86231
3.51484 3.33075 1.95536 5.10006 3.87002 215 l - - -
2.68618 4.42225 2.77519 2.73123 3.46354 2.40513 3.72494
3.29354 2.67741 2.69355 4.24690 2.90347 2.73739 3.18146 2.89801
2.37887 2.77519 2.98518 4.58477 3.61503
0.03133 3.87472 4.59707 0.61958 0.77255 0.48576 0.95510
216 2.77719 4.99382 2.70185 2.40535 4.35358 3.34122 3.64440
3.81080 1.73740 3.35667 4.20969 2.02812 3.84361 2.80456 2.58136
2.77082 3.03266 3.45508 5.47468 4.15327 216 k - - -
2.68618 4.42225 2.77519 2.73123 3.46354 2.40513 3.72494
3.29354 2.67741 2.69355 4.24690 2.90347 2.73739 3.18146 2.89801
2.37887 2.77519 2.98518 4.58477 3.61503
0.03133 3.87472 4.59707 0.61958 0.77255 0.48576 0.95510
217 2.88231 5.40246 1.59432 1.52915 4.68875 3.17660 3.71384
4.19005 2.71727 3.71475 4.57364 2.64991 3.79244 2.88286 3.29553
2.77643 3.16991 3.78014 5.87265 4.38952 217 e - - -
2.68618 4.42225 2.77519 2.73123 3.46354 2.40513 3.72494
3.29354 2.67741 2.69355 4.24690 2.90347 2.73739 3.18146 2.89801
2.37887 2.77519 2.98518 4.58477 3.61503
0.03133 3.87472 4.59707 0.61958 0.77255 0.48576 0.95510
218 1.47906 4.13431 3.26733 3.03926 4.22406 1.81134 4.15620
3.56680 3.14617 3.31697 4.17849 3.22640 3.64557 3.43748 3.45395
2.37864 2.68725 3.06492 5.56847 4.36006 218 a - - -
2.68618 4.42225 2.77519 2.73123 3.46354 2.40513 3.72494
3.29354 2.67741 2.69355 4.24690 2.90347 2.73739 3.18146 2.89801
2.37887 2.77519 2.98518 4.58477 3.61503
0.03133 3.87472 4.59707 0.61958 0.77255 0.48576 0.95510
219 3.15771 4.61926 4.19314 3.83291 3.13642 3.98506 4.48794
2.34049 3.60064 0.87242 3.15851 4.10818 4.41998 3.96843 3.77638
3.55606 3.45182 2.37843 5.01661 3.74048 219 l - - -
2.68618 4.42225 2.77519 2.73123 3.46354 2.40513 3.72494
3.29354 2.67741 2.69355 4.24690 2.90347 2.73739 3.18146 2.89801
2.37887 2.77519 2.98518 4.58477 3.61503
0.03133 3.87472 4.59707 0.61958 0.77255 0.48576 0.95510
220 2.45902 4.48583 2.86064 2.59790 4.11341 3.12525 3.84388
3.49110 2.69080 3.19827 4.07153 1.99845 3.74685 3.05945 3.06596
2.56366 1.97562 3.10819 5.43255 4.11826 220 t - - -
2.68618 4.42225 2.77519 2.73123 3.46354 2.40513 3.72494
3.29354 2.67741 2.69355 4.24690 2.90347 2.73739 3.18146 2.89801
2.37887 2.77519 2.98518 4.58477 3.61503

0.03133 3.87472 4.59707 0.61958 0.77255 0.48576 0.95510
221 3.26109 4.66217 4.24182 3.95666 1.00804 3.95049 3.87804
2.78442 3.86362 2.15392 3.51419 4.02166 4.40896 4.03095 3.98279
3.53096 3.56220 2.77530 4.12630 2.49298 221 f - - -
2.68618 4.42225 2.77519 2.73123 3.46354 2.40513 3.72494
3.29354 2.67741 2.69355 4.24690 2.90347 2.73739 3.18146 2.89801
2.37887 2.77519 2.98518 4.58477 3.61503
0.03133 3.87472 4.59707 0.61958 0.77255 0.48576 0.95510
222 3.00784 4.84699 3.49870 3.00918 4.20214 3.49552 3.82565
3.74393 2.23793 3.25944 4.25948 3.36879 3.99792 3.04579 0.97495
3.09185 3.27359 3.46776 5.28422 4.12892 222 r - - -
2.68618 4.42225 2.77519 2.73123 3.46354 2.40513 3.72494
3.29354 2.67741 2.69355 4.24690 2.90347 2.73739 3.18146 2.89801
2.37887 2.77519 2.98518 4.58477 3.61503
0.03133 3.87472 4.59707 0.61958 0.77255 0.48576 0.95510
223 2.72515 4.72193 2.70930 2.59495 4.08579 3.18536 3.92257
3.79656 2.82640 3.44315 4.39831 1.15407 3.83729 3.19525 3.18120
2.79220 3.10703 3.41854 5.39143 4.03390 223 n - - -
2.68618 4.42225 2.77519 2.73123 3.46354 2.40513 3.72494
3.29354 2.67741 2.69355 4.24690 2.90347 2.73739 3.18146 2.89801
2.37887 2.77519 2.98518 4.58477 3.61503
0.03133 3.87472 4.59707 0.61958 0.77255 0.48576 0.95510
224 2.97087 4.51925 4.05178 3.64534 3.20855 3.83573 4.37996
2.38437 3.41332 1.80283 1.37362 3.92258 4.29629 3.79501 3.61839
3.32024 3.27619 2.39076 5.04971 3.81172 224 m - - -
2.68618 4.42225 2.77519 2.73123 3.46354 2.40513 3.72494
3.29354 2.67741 2.69355 4.24690 2.90347 2.73739 3.18146 2.89801
2.37887 2.77519 2.98518 4.58477 3.61503
0.03133 3.87472 4.59707 0.61958 0.77255 0.48576 0.95510
225 2.58861 0.93589 4.10823 3.84832 3.83906 3.21118 4.49490
2.98326 3.67524 2.89868 4.02000 3.83470 3.91832 4.02124 3.79925
2.86028 3.05555 2.72771 5.25480 4.12675 225 c - - -
2.68618 4.42225 2.77519 2.73123 3.46354 2.40513 3.72494
3.29354 2.67741 2.69355 4.24690 2.90347 2.73739 3.18146 2.89801
2.37887 2.77519 2.98518 4.58477 3.61503
0.03133 3.87472 4.59707 0.61958 0.77255 0.48576 0.95510
226 2.45071 4.51931 2.73642 2.53112 4.20366 3.07349 3.84727
3.72224 2.72905 3.35410 4.20067 1.99554 3.72235 3.06064 3.12626
1.77209 2.84366 3.27443 5.50609 4.15874 226 s - - -
2.68618 4.42225 2.77519 2.73123 3.46354 2.40513 3.72494
3.29354 2.67741 2.69355 4.24690 2.90347 2.73739 3.18146 2.89801
2.37887 2.77519 2.98518 4.58477 3.61503
0.03133 3.87472 4.59707 0.61958 0.77255 0.48576 0.95510
227 3.15771 4.61926 4.19314 3.83291 3.13642 3.98506 4.48794
2.34049 3.60064 0.87242 3.15851 4.10818 4.41998 3.96843 3.77638
3.55606 3.45182 2.37843 5.01661 3.74048 227 l - - -
2.68618 4.42225 2.77519 2.73123 3.46354 2.40513 3.72494
3.29354 2.67741 2.69355 4.24690 2.90347 2.73739 3.18146 2.89801
2.37887 2.77519 2.98518 4.58477 3.61503
0.03133 3.87472 4.59707 0.61958 0.77255 0.48576 0.95510
228 3.25019 4.73827 3.84149 3.57122 2.29338 3.83270 3.61936
3.25908 3.42867 2.71244 3.93385 3.72612 4.30645 3.73417 3.61195
3.38669 3.53686 3.12972 3.93726 0.96205 228 y - - -

```
          2.68618  4.42225  2.77519  2.73123  3.46354  2.40513  3.72494
3.29354  2.67741  2.69355  4.24690  2.90347  2.73739  3.18146  2.89801
2.37887  2.77519  2.98518  4.58477  3.61503
          0.03133  3.87472  4.59707  0.61958  0.77255  0.48576  0.95510
    229   2.80983  4.35172  4.12850  3.79161  3.55163  3.72629  4.59291
2.04988  3.65943  2.23820  3.51634  3.98111  4.28661  4.01903  3.87173
3.22990  3.17128  1.02205  5.33648  4.06827    229 v - - -
          2.68618  4.42225  2.77519  2.73123  3.46354  2.40513  3.72494
3.29354  2.67741  2.69355  4.24690  2.90347  2.73739  3.18146  2.89801
2.37887  2.77519  2.98518  4.58477  3.61503
          0.03133  3.87472  4.59707  0.61958  0.77255  0.48576  0.95510
    230   3.26109  4.66217  4.24182  3.95666  1.00804  3.95049  3.87804
2.78442  3.86362  2.15392  3.51419  4.02166  4.40896  4.03095  3.98279
3.53096  3.56220  2.77530  4.12630  2.49298    230 f - - -
          2.68618  4.42225  2.77519  2.73123  3.46354  2.40513  3.72494
3.29354  2.67741  2.69355  4.24690  2.90347  2.73739  3.18146  2.89801
2.37887  2.77519  2.98518  4.58477  3.61503
          0.03133  3.87472  4.59707  0.61958  0.77255  0.48576  0.95510
    231   2.33702  4.29147  3.07128  2.85140  4.12548  3.00094  4.02194
3.58505  2.92944  3.27280  4.16301  3.15092  1.54458  3.28734  3.25182
2.20458  2.78498  3.13476  5.46871  4.19219    231 p - - -
          2.68618  4.42225  2.77519  2.73123  3.46354  2.40513  3.72494
3.29354  2.67741  2.69355  4.24690  2.90347  2.73739  3.18146  2.89801
2.37887  2.77519  2.98518  4.58477  3.61503
          0.03133  3.87472  4.59707  0.61958  0.77255  0.48576  0.95510
    232   3.26109  4.66217  4.24182  3.95666  1.00804  3.95049  3.87804
2.78442  3.86362  2.15392  3.51419  4.02166  4.40896  4.03095  3.98279
3.53096  3.56220  2.77530  4.12630  2.49298    232 f - - -
          2.68618  4.42225  2.77519  2.73123  3.46354  2.40513  3.72494
3.29354  2.67741  2.69355  4.24690  2.90347  2.73739  3.18146  2.89801
2.37887  2.77519  2.98518  4.58477  3.61503
          0.03133  3.87472  4.59707  0.61958  0.77255  0.48576  0.95510
    233   1.54708  4.11323  3.32564  3.05485  4.16099  2.89955  4.13258
3.49519  3.10452  3.25496  4.11764  3.22863  3.64079  3.41146  3.41044
1.59771  2.66061  3.00969  5.53041  4.30053    233 a - - -
          2.68618  4.42225  2.77519  2.73123  3.46354  2.40513  3.72494
3.29354  2.67741  2.69355  4.24690  2.90347  2.73739  3.18146  2.89801
2.37887  2.77519  2.98518  4.58477  3.61503
          0.03133  3.87472  4.59707  0.61958  0.77255  0.48576  0.95510
    234   2.76483  5.00262  2.39793  1.39874  4.44798  2.45486  3.75757
3.90258  2.64927  3.48982  4.35633  2.81021  3.80048  2.94089  3.10927
2.74719  3.07291  3.51921  5.64091  4.28559    234 e - - -
          2.68618  4.42225  2.77519  2.73123  3.46354  2.40513  3.72494
3.29354  2.67741  2.69355  4.24690  2.90347  2.73739  3.18146  2.89801
2.37887  2.77519  2.98518  4.58477  3.61503
          0.03133  3.87472  4.59707  0.61958  0.77255  0.48576  0.95510
    235   1.01052  4.19303  3.46476  3.25606  4.05451  3.01753  4.27786
3.18035  3.27238  3.06187  4.09337  3.40514  3.75695  3.61331  3.52281
2.54327  2.82077  2.83436  5.48144  4.28192    235 a - - -
          2.68618  4.42225  2.77519  2.73123  3.46354  2.40513  3.72494
3.29354  2.67741  2.69355  4.24690  2.90347  2.73739  3.18146  2.89801
2.37887  2.77519  2.98518  4.58477  3.61503
          0.03133  3.87472  4.59707  0.61958  0.77255  0.48576  0.95510
```

```
    236   3.33912  4.71305  4.09447  3.80105  2.66830  3.73256  3.90238
3.36273  3.52318  2.75727  3.99643  3.94963  4.25045  3.91067  3.65116
3.54280  3.63961  3.25312  0.88077  2.67027    236 w - - -
          2.68618  4.42225  2.77519  2.73123  3.46354  2.40513  3.72494
3.29354  2.67741  2.69355  4.24690  2.90347  2.73739  3.18146  2.89801
2.37887  2.77519  2.98518  4.58477  3.61503
          0.03133  3.87472  4.59707  0.61958  0.77255  0.48576  0.95510
    237   2.55359  4.43291  3.17571  2.79506  3.27739  3.32134  3.74945
3.18003  2.76932  2.83777  3.77380  3.17414  3.85703  3.13219  3.11316
1.72646  2.87576  2.89826  4.78568  2.58397    237 s - - -
          2.68618  4.42225  2.77519  2.73123  3.46354  2.40513  3.72494
3.29354  2.67741  2.69355  4.24690  2.90347  2.73739  3.18146  2.89801
2.37887  2.77519  2.98518  4.58477  3.61503
          0.03133  3.87472  4.59707  0.61958  0.77255  0.48576  0.95510
    238   3.15771  4.61926  4.19314  3.83291  3.13642  3.98506  4.48794
2.34049  3.60064  0.87242  3.15851  4.10818  4.41998  3.96843  3.77638
3.55606  3.45182  2.37843  5.01661  3.74048    238 l - - -
          2.68618  4.42225  2.77519  2.73123  3.46354  2.40513  3.72494
3.29354  2.67741  2.69355  4.24690  2.90347  2.73739  3.18146  2.89801
2.37887  2.77519  2.98518  4.58477  3.61503
          0.03133  3.87472  4.59707  0.61958  0.77255  0.48576  0.95510
    239   3.12595  4.55548  4.42280  3.88366  3.04497  4.25029  4.54412
2.17463  3.68325  1.17096  2.02793  4.18871  4.51787  3.94082  3.87635
3.59802  3.36223  2.25546  5.04224  3.88701    239 l - - -
          2.68618  4.42225  2.77519  2.73123  3.46354  2.40513  3.72494
3.29354  2.67741  2.69355  4.24690  2.90347  2.73739  3.18146  2.89801
2.37887  2.77519  2.98518  4.58477  3.61503
          0.03133  3.87472  4.59707  0.61958  0.77255  0.48576  0.95510
    240   3.00784  4.84699  3.49870  3.00918  4.20214  3.49552  3.82565
3.74393  2.23793  3.25944  4.25948  3.36879  3.99792  3.04579  0.97495
3.09185  3.27359  3.46776  5.28422  4.12892    240 r - - -
          2.68618  4.42225  2.77519  2.73123  3.46354  2.40513  3.72494
3.29354  2.67741  2.69355  4.24690  2.90347  2.73739  3.18146  2.89801
2.37887  2.77519  2.98518  4.58477  3.61503
          0.03133  3.87472  4.59707  0.61958  0.77255  0.48576  0.95510
    241   2.65935  4.59479  2.63183  2.57185  3.36734  2.97413  3.68878
3.23046  2.71575  2.88074  3.78902  3.04052  3.86788  3.02320  3.12049
2.75462  2.92903  2.96148  4.84792  1.97747    241 y - - -
          2.68618  4.42225  2.77519  2.73123  3.46354  2.40513  3.72494
3.29354  2.67741  2.69355  4.24690  2.90347  2.73739  3.18146  2.89801
2.37887  2.77519  2.98518  4.58477  3.61503
          0.03133  3.87472  4.59707  0.61958  0.77255  0.48576  0.95510
    242   2.95406  5.06580  2.48830  1.02217  4.41227  3.27540  3.86227
3.86661  2.74307  3.48333  4.45717  2.93393  3.88006  3.07782  3.15222
2.93617  3.25828  3.55019  5.58341  4.30555    242 e - - -
          2.68618  4.42225  2.77519  2.73123  3.46354  2.40513  3.72494
3.29354  2.67741  2.69355  4.24690  2.90347  2.73739  3.18146  2.89801
2.37887  2.77519  2.98518  4.58477  3.61503
          0.03133  3.87472  4.59707  0.61958  0.77255  0.48576  0.95510
    243   2.66752  4.44283  3.34177  3.24141  4.38386  0.72564  4.36163
3.98394  3.42300  3.63781  4.61350  3.50605  3.83404  3.74696  3.65888
2.83948  3.14893  3.51182  5.47850  4.48580    243 g - - -
          2.68618  4.42225  2.77519  2.73123  3.46354  2.40513  3.72494
3.29354  2.67741  2.69355  4.24690  2.90347  2.73739  3.18146  2.89801
2.37887  2.77519  2.98518  4.58477  3.61503
```

```
          0.03133  3.87472  4.59707  0.61958  0.77255  0.48576  0.95510
    244   2.64162  4.33711  3.67321  3.22195  3.45706  3.53660  4.11446
2.44838  3.06113  1.70894  3.40872  3.53299  4.04555  3.44978  3.34034
2.91604  2.03149  2.30628  5.10747  3.85543    244 l - - -
          2.68618  4.42225  2.77519  2.73123  3.46354  2.40513  3.72494
3.29354  2.67741  2.69355  4.24690  2.90347  2.73739  3.18146  2.89801
2.37887  2.77519  2.98518  4.58477  3.61503
          0.03133  3.87472  4.59707  0.61958  0.77255  0.48576  0.95510
    245   2.97404  4.93809  3.15019  2.77112  4.32503  3.46335  3.76023
3.75442  1.03520  3.31897  4.28021  3.19552  3.96346  2.95151  2.43176
3.01582  3.22709  3.46205  5.37869  4.19175    245 k - - -
          2.68618  4.42225  2.77519  2.73123  3.46354  2.40513  3.72494
3.29354  2.67741  2.69355  4.24690  2.90347  2.73739  3.18146  2.89801
2.37887  2.77519  2.98518  4.58477  3.61503
          0.03133  3.87472  4.59707  0.61958  0.77255  0.48576  0.95510
    246   3.15771  4.61926  4.19314  3.83291  3.13642  3.98506  4.48794
2.34049  3.60064  0.87242  3.15851  4.10818  4.41998  3.96843  3.77638
3.55606  3.45182  2.37843  5.01661  3.74048    246 l - - -
          2.68618  4.42225  2.77519  2.73123  3.46354  2.40513  3.72494
3.29354  2.67741  2.69355  4.24690  2.90347  2.73739  3.18146  2.89801
2.37887  2.77519  2.98518  4.58477  3.61503
          0.03133  3.87472  4.59707  0.61958  0.77255  0.48576  0.95510
    247   2.80882  5.13889  2.46208  1.70743  4.42438  3.29685  3.65103
3.88447  2.38903  3.41215  4.27193  2.80024  3.82288  2.03176  2.78870
2.76401  3.06172  3.52917  5.57720  4.20640    247 e - - -
          2.68618  4.42225  2.77519  2.73123  3.46354  2.40513  3.72494
3.29354  2.67741  2.69355  4.24690  2.90347  2.73739  3.18146  2.89801
2.37887  2.77519  2.98518  4.58477  3.61503
          0.03133  3.87472  4.59707  0.61958  0.77255  0.48576  0.95510
    248   2.43478  4.52494  2.71119  2.49549  4.28216  2.69145  3.83549
3.75335  2.72831  3.36616  4.19434  1.96358  3.70760  3.03354  3.14982
1.96488  2.82375  3.29165  5.55722  4.22865    248 n - - -
          2.68618  4.42225  2.77519  2.73123  3.46354  2.40513  3.72494
3.29354  2.67741  2.69355  4.24690  2.90347  2.73739  3.18146  2.89801
2.37887  2.77519  2.98518  4.58477  3.61503
          0.03133  3.87472  4.59707  0.61958  0.77255  0.48576  0.95510
    249   2.22778  4.16188  3.29232  2.98618  4.09586  2.95594  4.06343
3.42375  2.99344  3.17892  4.05012  3.20814  3.66777  3.33144  3.30840
1.69273  1.77569  2.97923  5.46426  4.22276    249 s - - -
          2.68618  4.42225  2.77519  2.73123  3.46354  2.40513  3.72494
3.29354  2.67741  2.69355  4.24690  2.90347  2.73739  3.18146  2.89801
2.37887  2.77519  2.98518  4.58477  3.61503
          0.03133  3.87472  4.59707  0.61958  0.77255  0.48576  0.95510
    250   3.15771  4.61926  4.19314  3.83291  3.13642  3.98506  4.48794
2.34049  3.60064  0.87242  3.15851  4.10818  4.41998  3.96843  3.77638
3.55606  3.45182  2.37843  5.01661  3.74048    250 l - - -
          2.68618  4.42225  2.77519  2.73123  3.46354  2.40513  3.72494
3.29354  2.67741  2.69355  4.24690  2.90347  2.73739  3.18146  2.89801
2.37887  2.77519  2.98518  4.58477  3.61503
          0.03133  3.87472  4.59707  0.61958  0.77255  0.48576  0.95510
    251   1.99174  4.14901  3.24232  3.01175  4.17450  2.90789  4.12488
3.53696  3.08074  3.29029  4.16708  3.20949  3.65185  3.40305  3.38462
1.27555  2.69027  3.04957  5.54475  4.29270    251 s - - -
```

```
         2.68618  4.42225  2.77519  2.73123  3.46354  2.40513  3.72494
3.29354  2.67741  2.69355  4.24690  2.90347  2.73739  3.18146  2.89801
2.37887  2.77519  2.98518  4.58477  3.61503
         0.03133  3.87472  4.59707  0.61958  0.77255  0.48576  0.95510
    252  3.15771  4.61926  4.19314  3.83291  3.13642  3.98506  4.48794
2.34049  3.60064  0.87242  3.15851  4.10818  4.41998  3.96843  3.77638
3.55606  3.45182  2.37843  5.01661  3.74048    252 l - - -
         2.68618  4.42225  2.77519  2.73123  3.46354  2.40513  3.72494
3.29354  2.67741  2.69355  4.24690  2.90347  2.73739  3.18146  2.89801
2.37887  2.77519  2.98518  4.58477  3.61503
         0.03133  3.87472  4.59707  0.61958  0.77255  0.48576  0.95510
    253  3.33912  4.71305  4.09447  3.80105  2.66830  3.73256  3.90238
3.36273  3.52318  2.75727  3.99643  3.94963  4.25045  3.91067  3.65116
3.54280  3.63961  3.25312  0.88077  2.67027    253 w - - -
         2.68618  4.42225  2.77519  2.73123  3.46354  2.40513  3.72494
3.29354  2.67741  2.69355  4.24690  2.90347  2.73739  3.18146  2.89801
2.37887  2.77519  2.98518  4.58477  3.61503
         0.03133  3.87472  4.59707  0.61958  0.77255  0.48576  0.95510
    254  2.61606  4.81155  2.28338  2.32416  4.12588  3.26186  3.67637
3.51544  2.54892  3.17825  4.02554  2.10828  3.77623  2.85022  3.02091
2.63898  2.89480  2.62933  5.41505  4.04488    254 n - - -
         2.68618  4.42225  2.77519  2.73123  3.46354  2.40513  3.72494
3.29354  2.67741  2.69355  4.24690  2.90347  2.73739  3.18146  2.89801
2.37887  2.77519  2.98518  4.58477  3.61503
         0.03133  3.87472  4.59707  0.61958  0.77255  0.48576  0.95510
    255  3.38648  4.70935  4.36745  3.99964  1.37219  4.19064  3.44502
3.10291  3.86347  2.47649  3.72001  3.90558  4.51704  3.92098  3.96486
3.54749  3.61497  3.02298  3.62547  1.38418    255 f - - -
         2.68618  4.42225  2.77519  2.73123  3.46354  2.40513  3.72494
3.29354  2.67741  2.69355  4.24690  2.90347  2.73739  3.18146  2.89801
2.37887  2.77519  2.98518  4.58477  3.61503
         0.03133  3.87472  4.59707  0.61958  0.77255  0.48576  0.95510
    256  2.80983  4.35172  4.12850  3.79161  3.55163  3.72629  4.59291
2.04988  3.65943  2.23820  3.51634  3.98111  4.28661  4.01903  3.87173
3.22990  3.17128  1.02205  5.33648  4.06827    256 v - - -
         2.68618  4.42225  2.77519  2.73123  3.46354  2.40513  3.72494
3.29354  2.67741  2.69355  4.24690  2.90347  2.73739  3.18146  2.89801
2.37887  2.77519  2.98518  4.58477  3.61503
         0.03133  3.87472  4.59707  0.61958  0.77255  0.48576  0.95510
    257  2.66752  4.44283  3.34177  3.24141  4.38386  0.72564  4.36163
3.98394  3.42300  3.63781  4.61350  3.50605  3.83404  3.74696  3.65888
2.83948  3.14893  3.51182  5.47850  4.48580    257 g - - -
         2.68618  4.42225  2.77519  2.73123  3.46354  2.40513  3.72494
3.29354  2.67741  2.69355  4.24690  2.90347  2.73739  3.18146  2.89801
2.37887  2.77519  2.98518  4.58477  3.61503
         0.03133  3.87472  4.59707  0.61958  0.77255  0.48576  0.95510
    258  2.66608  4.59649  2.95118  1.94429  3.84139  3.41398  3.82319
2.85327  2.66620  2.76388  3.76181  3.09508  3.90478  3.04466  3.04890
2.79153  2.94298  1.98831  5.27485  3.97346    258 e - - -
         2.68618  4.42225  2.77519  2.73123  3.46354  2.40513  3.72494
3.29354  2.67741  2.69355  4.24690  2.90347  2.73739  3.18146  2.89801
2.37887  2.77519  2.98518  4.58477  3.61503
         0.03133  3.87472  4.59707  0.61958  0.77255  0.48576  0.95510
```

```
    259   2.57144  4.73772  2.07439  2.34211  4.30401  3.12246  3.78805
3.77957  2.73354  3.40372  4.25311  2.84375  3.74463  2.98115  3.21238
1.62053  2.92586  3.36209  5.58644  4.20661    259 s - - -
          2.68618  4.42225  2.77519  2.73123  3.46354  2.40513  3.72494
3.29354  2.67741  2.69355  4.24690  2.90347  2.73739  3.18146  2.89801
2.37887  2.77519  2.98518  4.58477  3.61503
          0.03133  3.87472  4.59707  0.61958  0.77255  0.48576  0.95510
    260   3.03284  4.45083  4.33904  3.95485  3.37561  4.06909  4.68249
1.07512  3.78303  1.96109  3.31052  4.19987  4.50000  4.13104  3.97934
3.56453  3.33193  1.84577  5.26697  4.00552    260 i - - -
          2.68618  4.42225  2.77519  2.73123  3.46354  2.40513  3.72494
3.29354  2.67741  2.69355  4.24690  2.90347  2.73739  3.18146  2.89801
2.37887  2.77519  2.98518  4.58477  3.61503
          0.03133  3.87472  4.59707  0.61958  0.77255  0.48576  0.95510
    261   2.45371  4.54267  2.74270  2.49607  4.24485  2.43449  3.81360
3.70054  2.69190  3.31420  4.14456  2.35082  2.27478  3.00825  3.11297
2.53405  2.82674  3.26275  5.51464  4.19699    261 p - - -
          2.68618  4.42225  2.77519  2.73123  3.46354  2.40513  3.72494
3.29354  2.67741  2.69355  4.24690  2.90347  2.73739  3.18146  2.89801
2.37887  2.77519  2.98518  4.58477  3.61503
          0.23773  3.87472  1.65641  0.61958  0.77255  0.48576  0.95510
    262   2.39809  4.42582  2.61577  2.46394  4.11599  2.19139  3.79890
3.63735  2.70361  3.28544  4.15544  2.15109  3.64080  3.03412  3.08198
2.49186  2.80160  3.19865  5.40694  4.09024    262 n - - -
          2.68618  4.42225  2.77519  2.73123  3.46354  2.40513  3.72494
3.29354  2.67741  2.69355  4.24690  2.90347  2.73739  3.18146  2.89801
2.37887  2.77519  2.98518  4.58477  3.61503
          0.03837  3.67537  4.39771  0.61958  0.77255  0.40072  1.10818
    263   3.04380  4.38669  4.61681  4.09075  3.38454  4.34685  4.80406
1.39110  3.96917  1.76022  3.20223  4.35274  4.63577  4.22433  4.17967
3.70582  3.29663  1.41343  5.34102  4.15448    263 i - - -
          2.68618  4.42225  2.77519  2.73123  3.46354  2.40513  3.72494
3.29354  2.67741  2.69355  4.24690  2.90347  2.73739  3.18146  2.89801
2.37887  2.77519  2.98518  4.58477  3.61503
          0.03133  3.87472  4.59707  0.61958  0.77255  0.48576  0.95510
    264   2.52844  4.53174  2.95155  2.57508  3.83955  3.27207  3.73680
3.28447  2.57800  2.46816  3.84651  2.20409  3.79415  2.96122  2.96026
2.24870  2.83401  2.97306  5.20125  3.86696    264 n - - -
          2.68618  4.42225  2.77519  2.73123  3.46354  2.40513  3.72494
3.29354  2.67741  2.69355  4.24690  2.90347  2.73739  3.18146  2.89801
2.37887  2.77519  2.98518  4.58477  3.61503
          0.03133  3.87472  4.59707  0.61958  0.77255  0.48576  0.95510
    265   2.69169  4.58141  3.16605  2.70241  3.06759  3.50473  2.83677
3.18010  2.62669  2.79025  3.71155  3.13670  2.96848  3.00686  2.98848
2.79619  2.93230  2.92949  4.58798  2.25094    265 y - - -
          2.68618  4.42225  2.77519  2.73123  3.46354  2.40513  3.72494
3.29354  2.67741  2.69355  4.24690  2.90347  2.73739  3.18146  2.89801
2.37887  2.77519  2.98518  4.58477  3.61503
          0.03133  3.87472  4.59707  0.61958  0.77255  0.48576  0.95510
    266   2.72515  4.72193  2.70930  2.59495  4.08579  3.18536  3.92257
3.79656  2.82640  3.44315  4.39831  1.15407  3.83729  3.19525  3.18120
2.79220  3.10703  3.41854  5.39143  4.03390    266 n - - -
          2.68618  4.42225  2.77519  2.73123  3.46354  2.40513  3.72494
3.29354  2.67741  2.69355  4.24690  2.90347  2.73739  3.18146  2.89801
2.37887  2.77519  2.98518  4.58477  3.61503
```

0.03133 3.87472 4.59707 0.61958 0.77255 0.48576 0.95510
267 2.88464 5.40637 1.55777 1.55880 4.69237 3.17548 3.71510
4.19436 2.72133 3.71892 4.57869 2.64865 3.79270 2.88454 3.30122
2.77795 3.17279 3.78411 5.87674 4.39235   267 d - - -
2.68618 4.42225 2.77519 2.73123 3.46354 2.40513 3.72494
3.29354 2.67741 2.69355 4.24690 2.90347 2.73739 3.18146 2.89801
2.37887 2.77519 2.98518 4.58477 3.61503
0.03133 3.87472 4.59707 0.61958 0.77255 0.48576 0.95510
268 3.33912 4.71305 4.09447 3.80105 2.66830 3.73256 3.90238
3.36273 3.52318 2.75727 3.99643 3.94963 4.25045 3.91067 3.65116
3.54280 3.63961 3.25312 0.88077 2.67027   268 w - - -
2.68618 4.42225 2.77519 2.73123 3.46354 2.40513 3.72494
3.29354 2.67741 2.69355 4.24690 2.90347 2.73739 3.18146 2.89801
2.37887 2.77519 2.98518 4.58477 3.61503
0.03133 3.87472 4.59707 0.61958 0.77255 0.48576 0.95510
269 2.67356 4.76527 2.84377 2.53165 4.29029 2.10265 3.73173
3.71384 1.69571 3.30316 4.16351 2.99496 3.82060 2.91102 2.70568
2.72194 2.97746 3.34473 5.44668 4.17763   269 k - - -
2.68618 4.42225 2.77519 2.73123 3.46354 2.40513 3.72494
3.29354 2.67741 2.69355 4.24690 2.90347 2.73739 3.18146 2.89801
2.37887 2.77519 2.98518 4.58477 3.61503
0.03133 3.87472 4.59707 0.61958 0.77255 0.48576 0.95510
270 2.66752 4.44283 3.34177 3.24141 4.38386 0.72564 4.36163
3.98394 3.42300 3.63781 4.61350 3.50605 3.83404 3.74696 3.65888
2.83948 3.14893 3.51182 5.47850 4.48580   270 g - - -
2.68618 4.42225 2.77519 2.73123 3.46354 2.40513 3.72494
3.29354 2.67741 2.69355 4.24690 2.90347 2.73739 3.18146 2.89801
2.37887 2.77519 2.98518 4.58477 3.61503
0.03133 3.87472 4.59707 0.61958 0.77255 0.48576 0.95510
271 1.91734 4.28111 3.78222 3.29329 3.47833 3.56649 4.17358
2.30863 3.18175 1.84422 3.38904 3.58915 4.06649 3.51647 3.47287
2.92484 2.93633 2.01150 5.13695 3.90838   271 l - - -
2.68618 4.42225 2.77519 2.73123 3.46354 2.40513 3.72494
3.29354 2.67741 2.69355 4.24690 2.90347 2.73739 3.18146 2.89801
2.37887 2.77519 2.98518 4.58477 3.61503
0.03133 3.87472 4.59707 0.61958 0.77255 0.48576 0.95510
272 2.99209 4.42863 4.37275 3.87607 3.25911 4.12809 4.59007
1.97005 3.70968 1.38876 3.13713 4.14497 4.48592 4.00421 3.92546
3.50336 3.26357 1.49131 5.18215 3.96333   272 l - - -
2.68618 4.42225 2.77519 2.73123 3.46354 2.40513 3.72494
3.29354 2.67741 2.69355 4.24690 2.90347 2.73739 3.18146 2.89801
2.37887 2.77519 2.98518 4.58477 3.61503
0.03133 3.87472 4.59707 0.61958 0.77255 0.48576 0.95510
273 3.25019 4.73827 3.84149 3.57122 2.29338 3.83270 3.61936
3.25908 3.42867 2.71244 3.93385 3.72612 4.30645 3.73417 3.61195
3.38669 3.53686 3.12972 3.93726 0.96205   273 y - - -
2.68618 4.42225 2.77519 2.73123 3.46354 2.40513 3.72494
3.29354 2.67741 2.69355 4.24690 2.90347 2.73739 3.18146 2.89801
2.37887 2.77519 2.98518 4.58477 3.61503
0.03133 3.87472 4.59707 0.61958 0.77255 0.48576 0.95510
274 2.97404 4.93809 3.15019 2.77112 4.32503 3.46335 3.76023
3.75442 1.03520 3.31897 4.28021 3.19552 3.96346 2.95151 2.43176
3.01582 3.22709 3.46205 5.37869 4.19175   274 k - - -

```
          2.68618  4.42225  2.77519  2.73123  3.46354  2.40513  3.72494
3.29354  2.67741  2.69355  4.24690  2.90347  2.73739  3.18146  2.89801
2.37887  2.77519  2.98518  4.58477  3.61503
          0.03133  3.87472  4.59707  0.61958  0.77255  0.48576  0.95510
    275   3.15771  4.61926  4.19314  3.83291  3.13642  3.98506  4.48794
2.34049  3.60064  0.87242  3.15851  4.10818  4.41998  3.96843  3.77638
3.55606  3.45182  2.37843  5.01661  3.74048    275 l - - -
          2.68618  4.42225  2.77519  2.73123  3.46354  2.40513  3.72494
3.29354  2.67741  2.69355  4.24690  2.90347  2.73739  3.18146  2.89801
2.37887  2.77519  2.98518  4.58477  3.61503
          0.03133  3.87472  4.59707  0.61958  0.77255  0.48576  0.95510
    276   3.15771  4.61926  4.19314  3.83291  3.13642  3.98506  4.48794
2.34049  3.60064  0.87242  3.15851  4.10818  4.41998  3.96843  3.77638
3.55606  3.45182  2.37843  5.01661  3.74048    276 l - - -
          2.68618  4.42225  2.77519  2.73123  3.46354  2.40513  3.72494
3.29354  2.67741  2.69355  4.24690  2.90347  2.73739  3.18146  2.89801
2.37887  2.77519  2.98518  4.58477  3.61503
          0.03133  3.87472  4.59707  0.61958  0.77255  0.48576  0.95510
    277   2.97087  4.51925  4.05178  3.64534  3.20855  3.83573  4.37996
2.38437  3.41332  1.80283  1.37362  3.92258  4.29629  3.79501  3.61839
3.32024  3.27619  2.39076  5.04971  3.81172    277 m - - -
          2.68618  4.42225  2.77519  2.73123  3.46354  2.40513  3.72494
3.29354  2.67741  2.69355  4.24690  2.90347  2.73739  3.18146  2.89801
2.37887  2.77519  2.98518  4.58477  3.61503
          0.03133  3.87472  4.59707  0.61958  0.77255  0.48576  0.95510
    278   2.66752  4.44283  3.34177  3.24141  4.38386  0.72564  4.36163
3.98394  3.42300  3.63781  4.61350  3.50605  3.83404  3.74696  3.65888
2.83948  3.14893  3.51182  5.47850  4.48580    278 g - - -
          2.68618  4.42225  2.77519  2.73123  3.46354  2.40513  3.72494
3.29354  2.67741  2.69355  4.24690  2.90347  2.73739  3.18146  2.89801
2.37887  2.77519  2.98518  4.58477  3.61503
          0.03133  3.87472  4.59707  0.61958  0.77255  0.48576  0.95510
    279   1.88097  4.74174  2.61868  1.80482  4.19887  3.22247  3.76101
3.49196  2.61310  3.20184  4.09097  2.90084  3.79239  2.95030  3.03642
2.67111  2.93950  3.16046  5.48787  4.15513    279 e - - -
          2.68618  4.42225  2.77519  2.73123  3.46354  2.40513  3.72494
3.29354  2.67741  2.69355  4.24690  2.90347  2.73739  3.18146  2.89801
2.37887  2.77519  2.98518  4.58477  3.61503
          0.03133  3.87472  4.59707  0.61958  0.77255  0.48576  0.95510
    280   2.40654  4.21324  3.47874  3.02197  3.69323  3.25527  3.99198
2.74301  2.95777  2.65802  3.62430  3.31941  2.82607  3.29354  3.28162
2.62691  2.29844  1.88065  5.16826  3.93620    280 v - - -
          2.68618  4.42225  2.77519  2.73123  3.46354  2.40513  3.72494
3.29354  2.67741  2.69355  4.24690  2.90347  2.73739  3.18146  2.89801
2.37887  2.77519  2.98518  4.58477  3.61503
          0.03133  3.87472  4.59707  0.61958  0.77255  0.48576  0.95510
    281   2.72515  4.72193  2.70930  2.59495  4.08579  3.18536  3.92257
3.79656  2.82640  3.44315  4.39831  1.15407  3.83729  3.19525  3.18120
2.79220  3.10703  3.41854  5.39143  4.03390    281 n - - -
          2.68618  4.42225  2.77519  2.73123  3.46354  2.40513  3.72494
3.29354  2.67741  2.69355  4.24690  2.90347  2.73739  3.18146  2.89801
2.37887  2.77519  2.98518  4.58477  3.61503
          0.03133  3.87472  4.59707  0.61958  0.77255  0.48576  0.95510
```

```
    282   2.88236  4.86831  2.90423  2.65889  4.03720  3.36741  3.81726
3.66821  2.47950  3.17034  4.19804  3.11869  3.92083  1.23004  2.76368
2.92996  3.17928  3.39771  5.30876  4.00263    282 q - - -
          2.68618  4.42225  2.77519  2.73123  3.46354  2.40513  3.72494
3.29354  2.67741  2.69355  4.24690  2.90347  2.73739  3.18146  2.89801
2.37887  2.77519  2.98518  4.58477  3.61503
          0.03133  3.87472  4.59707  0.61958  0.77255  0.48576  0.95510
    283   3.00784  4.84699  3.49870  3.00918  4.20214  3.49552  3.82565
3.74393  2.23793  3.25944  4.25948  3.36879  3.99792  3.04579  0.97495
3.09185  3.27359  3.46776  5.28422  4.12892    283 r - - -
          2.68618  4.42225  2.77519  2.73123  3.46354  2.40513  3.72494
3.29354  2.67741  2.69355  4.24690  2.90347  2.73739  3.18146  2.89801
2.37887  2.77519  2.98518  4.58477  3.61503
          0.03133  3.87472  4.59707  0.61958  0.77255  0.48576  0.95510
    284   3.15771  4.61926  4.19314  3.83291  3.13642  3.98506  4.48794
2.34049  3.60064  0.87242  3.15851  4.10818  4.41998  3.96843  3.77638
3.55606  3.45182  2.37843  5.01661  3.74048    284 l - - -
          2.68618  4.42225  2.77519  2.73123  3.46354  2.40513  3.72494
3.29354  2.67741  2.69355  4.24690  2.90347  2.73739  3.18146  2.89801
2.37887  2.77519  2.98518  4.58477  3.61503
          0.03133  3.87472  4.59707  0.61958  0.77255  0.48576  0.95510
    285   1.99151  4.33602  3.05283  2.71725  4.06246  3.08205  3.88798
3.38639  2.77019  3.11417  3.97117  2.43707  3.71352  3.11274  3.14375
2.49002  1.95983  2.99581  5.39834  4.12780    285 t - - -
          2.68618  4.42225  2.77519  2.73123  3.46354  2.40513  3.72494
3.29354  2.67741  2.69355  4.24690  2.90347  2.73739  3.18146  2.89801
2.37887  2.77519  2.98518  4.58477  3.61503
          0.03133  3.87472  4.59707  0.61958  0.77255  0.48576  0.95510
    286   2.45902  4.48583  2.86064  2.59790  4.11341  3.12525  3.84388
3.49110  2.69080  3.19827  4.07153  1.99845  3.74685  3.05945  3.06596
2.56366  1.97562  3.10819  5.43255  4.11826    286 t - - -
          2.68618  4.42225  2.77519  2.73123  3.46354  2.40513  3.72494
3.29354  2.67741  2.69355  4.24690  2.90347  2.73739  3.18146  2.89801
2.37887  2.77519  2.98518  4.58477  3.61503
          0.03133  3.87472  4.59707  0.61958  0.77255  0.48576  0.95510
    287   2.80983  4.35172  4.12850  3.79161  3.55163  3.72629  4.59291
2.04988  3.65943  2.23820  3.51634  3.98111  4.28661  4.01903  3.87173
3.22990  3.17128  1.02205  5.33648  4.06827    287 v - - -
          2.68618  4.42225  2.77519  2.73123  3.46354  2.40513  3.72494
3.29354  2.67741  2.69355  4.24690  2.90347  2.73739  3.18146  2.89801
2.37887  2.77519  2.98518  4.58477  3.61503
          0.03133  3.87472  4.59707  0.61958  0.77255  0.48576  0.95510
    288   2.86581  5.04245  3.12883  2.58703  4.43794  3.51236  3.56824
3.82233  1.49898  3.30954  4.15641  2.73468  3.91202  2.70630  1.93671
2.86271  3.06248  3.48875  5.39933  4.16760    288 k - - -
          2.68618  4.42225  2.77519  2.73123  3.46354  2.40513  3.72494
3.29354  2.67741  2.69355  4.24690  2.90347  2.73739  3.18146  2.89801
2.37887  2.77519  2.98518  4.58477  3.61503
          0.03133  3.87472  4.59707  0.61958  0.77255  0.48576  0.95510
    289   3.26109  4.66217  4.24182  3.95666  1.00804  3.95049  3.87804
2.78442  3.86362  2.15392  3.51419  4.02166  4.40896  4.03095  3.98279
3.53096  3.56220  2.77530  4.12630  2.49298    289 f - - -
          2.68618  4.42225  2.77519  2.73123  3.46354  2.40513  3.72494
3.29354  2.67741  2.69355  4.24690  2.90347  2.73739  3.18146  2.89801
2.37887  2.77519  2.98518  4.58477  3.61503
```

```
         0.03133  3.87472  4.59707  0.61958  0.77255  0.48576  0.95510
    290  2.72515  4.72193  2.70930  2.59495  4.08579  3.18536  3.92257
3.79656  2.82640  3.44315  4.39831  1.15407  3.83729  3.19525  3.18120
2.79220  3.10703  3.41854  5.39143  4.03390    290 n - - -
         2.68618  4.42225  2.77519  2.73123  3.46354  2.40513  3.72494
3.29354  2.67741  2.69355  4.24690  2.90347  2.73739  3.18146  2.89801
2.37887  2.77519  2.98518  4.58477  3.61503
         0.03133  3.87472  4.59707  0.61958  0.77255  0.48576  0.95510
    291  3.25019  4.73827  3.84149  3.57122  2.29338  3.83270  3.61936
3.25908  3.42867  2.71244  3.93385  3.72612  4.30645  3.73417  3.61195
3.38669  3.53686  3.12972  3.93726  0.96205    291 y - - -
         2.68618  4.42225  2.77519  2.73123  3.46354  2.40513  3.72494
3.29354  2.67741  2.69355  4.24690  2.90347  2.73739  3.18146  2.89801
2.37887  2.77519  2.98518  4.58477  3.61503
         0.03133  3.87472  4.59707  0.61958  0.77255  0.48576  0.95510
    292  2.31597  4.23644  3.20823  3.02400  4.07846  2.96628  4.13384
3.60134  3.09793  3.32136  4.24719  3.24849  3.70524  3.44704  3.38396
1.08767  2.79625  3.13595  5.45075  4.16198    292 s - - -
         2.68618  4.42225  2.77519  2.73123  3.46354  2.40513  3.72494
3.29354  2.67741  2.69355  4.24690  2.90347  2.73739  3.18146  2.89801
2.37887  2.77519  2.98518  4.58477  3.61503
         0.03133  3.87472  4.59707  0.61958  0.77255  0.48576  0.95510
    293  3.38852  4.71189  4.36458  3.99706  1.40423  4.19048  3.44249
3.10909  3.86117  2.48342  3.72613  3.90357  4.51714  3.91931  3.96352
3.54726  3.61700  3.02797  3.62301  1.34887    293 y - - -
         2.68618  4.42225  2.77519  2.73123  3.46354  2.40513  3.72494
3.29354  2.67741  2.69355  4.24690  2.90347  2.73739  3.18146  2.89801
2.37887  2.77519  2.98518  4.58477  3.61503
         0.03133  3.87472  4.59707  0.61958  0.77255  0.48576  0.95510
    294  2.62873  4.28585  3.72726  3.27618  2.08219  3.53344  3.98278
2.56928  3.19749  2.25542  3.38782  3.54240  4.03248  3.49209  3.46847
2.90157  2.07142  2.41813  4.72487  3.31059    294 t - - -
         2.68618  4.42225  2.77519  2.73123  3.46354  2.40513  3.72494
3.29354  2.67741  2.69355  4.24690  2.90347  2.73739  3.18146  2.89801
2.37887  2.77519  2.98518  4.58477  3.61503
         0.03133  3.87472  4.59707  0.61958  0.77255  0.48576  0.95510
    295  2.39898  4.43130  2.82472  2.61543  4.17476  3.04023  3.89338
3.69311  2.78479  3.34002  4.18923  2.37092  3.70913  3.12081  3.16261
1.51203  2.81207  3.23331  5.49324  4.16022    295 s - - -
         2.68618  4.42225  2.77519  2.73123  3.46354  2.40513  3.72494
3.29354  2.67741  2.69355  4.24690  2.90347  2.73739  3.18146  2.89801
2.37887  2.77519  2.98518  4.58477  3.61503
         0.03133  3.87472  4.59707  0.61958  0.77255  0.48576  0.95510
    296  2.88464  5.40637  1.55777  1.55880  4.69237  3.17548  3.71510
4.19436  2.72133  3.71892  4.57869  2.64865  3.79270  2.88454  3.30122
2.77795  3.17279  3.78411  5.87674  4.39235    296 d - - -
         2.68618  4.42225  2.77519  2.73123  3.46354  2.40513  3.72494
3.29354  2.67741  2.69355  4.24690  2.90347  2.73739  3.18146  2.89801
2.37887  2.77519  2.98518  4.58477  3.61503
         0.03133  3.87472  4.59707  0.61958  0.77255  0.48576  0.95510
    297  2.32545  4.26064  3.21462  2.88889  4.04222  3.04888  3.99307
3.34249  2.88511  3.08033  3.98575  3.18377  2.16061  3.24624  3.21593
2.48782  1.81759  2.95783  5.40672  4.16322    297 t - - -
```

2.68618 4.42225 2.77519 2.73123 3.46354 2.40513 3.72494
3.29354 2.67741 2.69355 4.24690 2.90347 2.73739 3.18146 2.89801
2.37887 2.77519 2.98518 4.58477 3.61503
0.03133 3.87472 4.59707 0.61958 0.77255 0.48576 0.95510
298 1.90033 4.60225 2.89491 2.57035 4.06040 3.24131 3.76678
3.42994 2.51871 3.07303 3.98233 3.01532 3.79796 2.04163 2.86925
2.64820 2.88696 3.10380 5.36498 4.06287 298 a - - -
2.68618 4.42225 2.77519 2.73123 3.46354 2.40513 3.72494
3.29354 2.67741 2.69355 4.24690 2.90347 2.73739 3.18146 2.89801
2.37887 2.77519 2.98518 4.58477 3.61503
0.03133 3.87472 4.59707 0.61958 0.77255 0.48576 0.95510
299 2.15745 4.80755 1.82841 2.28528 4.38167 2.47912 3.75346
3.81196 2.69444 3.42098 4.25375 2.80439 3.74040 2.92950 3.19843
2.60580 2.92522 3.39303 5.63269 4.25154 299 d - - -
2.68618 4.42225 2.77519 2.73123 3.46354 2.40513 3.72494
3.29354 2.67741 2.69355 4.24690 2.90347 2.73739 3.18146 2.89801
2.37887 2.77519 2.98518 4.58477 3.61503
0.03133 3.87472 4.59707 0.61958 0.77255 0.48576 0.95510
300 2.66336 4.28262 3.84497 3.37839 3.51443 3.63481 4.24797
1.69725 3.25170 2.26494 3.42173 3.66571 4.12942 3.60291 3.52809
3.00709 2.06156 2.02200 5.18516 3.95321 300 i - - -
2.68618 4.42225 2.77519 2.73123 3.46354 2.40513 3.72494
3.29354 2.67741 2.69355 4.24690 2.90347 2.73739 3.18146 2.89801
2.37887 2.77519 2.98518 4.58477 3.61503
0.03133 3.87472 4.59707 0.61958 0.77255 0.48576 0.95510
301 2.61644 4.32464 3.56554 3.12529 3.50064 3.49946 4.05578
1.82256 3.02665 2.35527 3.46910 3.46121 2.39145 3.38970 3.32713
2.87680 2.92890 2.32678 5.08872 3.83375 301 i - - -
2.68618 4.42225 2.77519 2.73123 3.46354 2.40513 3.72494
3.29354 2.67741 2.69355 4.24690 2.90347 2.73739 3.18146 2.89801
2.37887 2.77519 2.98518 4.58477 3.61503
0.03133 3.87472 4.59707 0.61958 0.77255 0.48576 0.95510
302 1.89703 4.53883 2.98309 2.63658 3.75598 3.26701 2.28422
3.35861 2.58486 3.00613 3.90780 3.06354 3.81323 3.01081 2.93241
2.66251 2.87882 3.04237 5.14291 3.76823 302 a - - -
2.68618 4.42225 2.77519 2.73123 3.46354 2.40513 3.72494
3.29354 2.67741 2.69355 4.24690 2.90347 2.73739 3.18146 2.89801
2.37887 2.77519 2.98518 4.58477 3.61503
0.03133 3.87472 4.59707 0.61958 0.77255 0.48576 0.95510
303 3.00784 4.84699 3.49870 3.00918 4.20214 3.49552 3.82565
3.74393 2.23793 3.25944 4.25948 3.36879 3.99792 3.04579 0.97495
3.09185 3.27359 3.46776 5.28422 4.12892 303 r - - -
2.68618 4.42225 2.77519 2.73123 3.46354 2.40513 3.72494
3.29354 2.67741 2.69355 4.24690 2.90347 2.73739 3.18146 2.89801
2.37887 2.77519 2.98518 4.58477 3.61503
0.03133 3.87472 4.59707 0.61958 0.77255 0.48576 0.95510
304 2.95406 5.06580 2.48830 1.02217 4.41227 3.27540 3.86227
3.86661 2.74307 3.48333 4.45717 2.93393 3.88006 3.07782 3.15222
2.93617 3.25828 3.55019 5.58341 4.30555 304 e - - -
2.68618 4.42225 2.77519 2.73123 3.46354 2.40513 3.72494
3.29354 2.67741 2.69355 4.24690 2.90347 2.73739 3.18146 2.89801
2.37887 2.77519 2.98518 4.58477 3.61503
0.03133 3.87472 4.59707 0.61958 0.77255 0.48576 0.95510

```
    305   2.72515  4.72193  2.70930  2.59495  4.08579  3.18536  3.92257
3.79656  2.82640  3.44315  4.39831  1.15407  3.83729  3.19525  3.18120
2.79220  3.10703  3.41854  5.39143  4.03390    305 n - - -
          2.68618  4.42225  2.77519  2.73123  3.46354  2.40513  3.72494
3.29354  2.67741  2.69355  4.24690  2.90347  2.73739  3.18146  2.89801
2.37887  2.77519  2.98518  4.58477  3.61503
          0.03133  3.87472  4.59707  0.61958  0.77255  0.48576  0.95510
    306   3.03284  4.45083  4.33904  3.95485  3.37561  4.06909  4.68249
1.07512  3.78303  1.96109  3.31052  4.19987  4.50000  4.13104  3.97934
3.56453  3.33193  1.84577  5.26697  4.00552    306 i - - -
          2.68618  4.42225  2.77519  2.73123  3.46354  2.40513  3.72494
3.29354  2.67741  2.69355  4.24690  2.90347  2.73739  3.18146  2.89801
2.37887  2.77519  2.98518  4.58477  3.61503
          0.03133  3.87472  4.59707  0.61958  0.77255  0.48576  0.95510
    307   2.74099  4.73243  3.25310  2.66802  3.76366  3.52164  2.85013
3.34451  2.18850  2.47283  3.82276  3.10701  3.90656  2.81781  1.95534
2.81404  2.95369  3.08014  5.06291  3.72980    307 r - - -
          2.68618  4.42225  2.77519  2.73123  3.46354  2.40513  3.72494
3.29354  2.67741  2.69355  4.24690  2.90347  2.73739  3.18146  2.89801
2.37887  2.77519  2.98518  4.58477  3.61503
          0.03133  3.87472  4.59707  0.61958  0.77255  0.48576  0.95510
    308   2.66752  4.44283  3.34177  3.24141  4.38386  0.72564  4.36163
3.98394  3.42300  3.63781  4.61350  3.50605  3.83404  3.74696  3.65888
2.83948  3.14893  3.51182  5.47850  4.48580    308 g - - -
          2.68618  4.42225  2.77519  2.73123  3.46354  2.40513  3.72494
3.29354  2.67741  2.69355  4.24690  2.90347  2.73739  3.18146  2.89801
2.37887  2.77519  2.98518  4.58477  3.61503
          0.03133  3.87472  4.59707  0.61958  0.77255  0.48576  0.95510
    309   1.97189  4.13316  3.58891  3.19027  3.74970  2.75357  4.12150
2.69707  3.14692  2.72394  3.69030  3.39702  3.81469  3.45756  3.43725
2.58489  2.74630  1.66280  5.24122  4.02153    309 v - - -
          2.68618  4.42225  2.77519  2.73123  3.46354  2.40513  3.72494
3.29354  2.67741  2.69355  4.24690  2.90347  2.73739  3.18146  2.89801
2.37887  2.77519  2.98518  4.58477  3.61503
          0.03133  3.87472  4.59707  0.61958  0.77255  0.48576  0.95510
    310   2.81382  4.64804  3.28720  2.85419  3.05837  3.56460  1.94627
3.14463  2.59682  2.21326  3.73747  3.25264  4.00350  3.09707  2.88671
2.92906  3.06449  2.95147  4.60494  3.07046    310 h - - -
          2.68618  4.42225  2.77519  2.73123  3.46354  2.40513  3.72494
3.29354  2.67741  2.69355  4.24690  2.90347  2.73739  3.18146  2.89801
2.37887  2.77519  2.98518  4.58477  3.61503
          0.03133  3.87472  4.59707  0.61958  0.77255  0.48576  0.95510
    311   2.75420  4.51129  3.36160  3.20088  4.19604  3.19867  4.27823
3.74193  3.25835  3.36857  4.41844  3.50740  0.82346  3.64706  3.50371
2.92174  3.19592  3.38706  5.38207  4.31670    311 p - - -
          2.68618  4.42225  2.77519  2.73123  3.46354  2.40513  3.72494
3.29354  2.67741  2.69355  4.24690  2.90347  2.73739  3.18146  2.89801
2.37887  2.77519  2.98518  4.58477  3.61503
          0.03133  3.87472  4.59707  0.61958  0.77255  0.48576  0.95510
    312   2.72930  4.30597  3.88840  3.36590  3.42170  3.76633  4.21248
1.80835  3.23466  2.01752  3.32116  3.69073  4.18394  3.57273  3.51916
3.09460  2.13388  2.00212  5.10804  3.88352    312 i - - -
          2.68618  4.42225  2.77519  2.73123  3.46354  2.40513  3.72494
3.29354  2.67741  2.69355  4.24690  2.90347  2.73739  3.18146  2.89801
2.37887  2.77519  2.98518  4.58477  3.61503
```

```
          0.03133  3.87472  4.59707  0.61958  0.77255  0.48576  0.95510
    313   3.25019  4.73827  3.84149  3.57122  2.29338  3.83270  3.61936
3.25908  3.42867  2.71244  3.93385  3.72612  4.30645  3.73417  3.61195
3.38669  3.53686  3.12972  3.93726  0.96205    313 y - - -
          2.68618  4.42225  2.77519  2.73123  3.46354  2.40513  3.72494
3.29354  2.67741  2.69355  4.24690  2.90347  2.73739  3.18146  2.89801
2.37887  2.77519  2.98518  4.58477  3.61503
          0.03133  3.87472  4.59707  0.61958  0.77255  0.48576  0.95510
    314   2.81676  5.23993  1.63562  2.11412  4.56066  3.16811  3.72343
4.10783  2.71559  3.65084  4.50633  1.84907  3.78432  2.89965  3.27130
2.74285  3.11960  3.69368  5.78369  4.31283    314 d - - -
          2.68618  4.42225  2.77519  2.73123  3.46354  2.40513  3.72494
3.29354  2.67741  2.69355  4.24690  2.90347  2.73739  3.18146  2.89801
2.37887  2.77519  2.98518  4.58477  3.61503
          0.03133  3.87472  4.59707  0.61958  0.77255  0.48576  0.95510
    315   2.37997  4.32935  3.07017  2.85289  4.27042  1.70470  4.05851
3.73902  3.00054  3.39050  4.25325  3.17102  1.91786  3.31722  3.34370
2.52375  2.82732  3.25201  5.52375  4.32782    315 g - - -
          2.68618  4.42225  2.77519  2.73123  3.46354  2.40513  3.72494
3.29354  2.67741  2.69355  4.24690  2.90347  2.73739  3.18146  2.89801
2.37887  2.77519  2.98518  4.58477  3.61503
          0.03133  3.87472  4.59707  0.61958  0.77255  0.48576  0.95510
    316   2.56374  4.72738  2.58290  2.02105  4.26726  2.52308  3.73912
3.70174  2.61351  3.30618  4.14043  2.85918  3.74880  2.91919  3.06336
1.93490  2.89166  3.30508  5.52346  4.17679    316 s - - -
          2.68618  4.42225  2.77519  2.73123  3.46354  2.40513  3.72494
3.29354  2.67741  2.69355  4.24690  2.90347  2.73739  3.18146  2.89801
2.37887  2.77519  2.98518  4.58477  3.61503
          0.03133  3.87472  4.59707  0.61958  0.77255  0.48576  0.95510
    317   2.52179  4.63303  2.81914  2.47084  4.12832  3.21498  3.71357
3.56615  2.50629  3.17165  4.00846  2.94088  2.85064  2.41079  2.90697
1.97403  2.83682  3.19220  5.39103  4.07198    317 s - - -
          2.68618  4.42225  2.77519  2.73123  3.46354  2.40513  3.72494
3.29354  2.67741  2.69355  4.24690  2.90347  2.73739  3.18146  2.89801
2.37887  2.77519  2.98518  4.58477  3.61503
          0.03133  3.87472  4.59707  0.61958  0.77255  0.48576  0.95510
    318   2.24726  4.18285  3.23236  2.96942  4.16083  1.87026  4.08563
3.49776  3.03020  3.24243  4.11178  3.20327  3.67046  3.35450  3.34775
2.41810  1.73379  3.03689  5.50551  4.28212    318 t - - -
          2.68618  4.42225  2.77519  2.73123  3.46354  2.40513  3.72494
3.29354  2.67741  2.69355  4.24690  2.90347  2.73739  3.18146  2.89801
2.37887  2.77519  2.98518  4.58477  3.61503
          0.03133  3.87472  4.59707  0.61958  0.77255  0.48576  0.95510
    319   2.70143  4.27159  3.83858  3.28680  3.33071  3.75867  4.10914
1.97880  3.18964  1.89461  3.24060  3.62695  3.06621  3.48983  3.48051
3.05324  2.95176  1.88583  4.97984  3.76937    319 v - - -
          2.68618  4.42225  2.77519  2.73123  3.46354  2.40513  3.72494
3.29354  2.67741  2.69355  4.24690  2.90347  2.73739  3.18146  2.89801
2.37887  2.77519  2.98518  4.58477  3.61503
          0.03133  3.87472  4.59707  0.61958  0.77255  0.48576  0.95510
    320   2.41377  4.25888  3.42992  3.18193  3.97070  3.09811  4.19910
3.09810  3.12495  2.96152  4.01182  3.39101  3.80201  3.51964  3.38358
2.61505  1.16810  2.79284  5.40831  4.18731    320 t - - -
```

```
          2.68618  4.42225  2.77519  2.73123  3.46354  2.40513  3.72494
3.29354  2.67741  2.69355  4.24690  2.90347  2.73739  3.18146  2.89801
2.37887  2.77519  2.98518  4.58477  3.61503
          0.03133  3.87472  4.59707  0.61958  0.77255  0.48576  0.95510
    321   2.66752  4.44283  3.34177  3.24141  4.38386  0.72564  4.36163
3.98394  3.42300  3.63781  4.61350  3.50605  3.83404  3.74696  3.65888
2.83948  3.14893  3.51182  5.47850  4.48580    321 g - - -
          2.68618  4.42225  2.77519  2.73123  3.46354  2.40513  3.72494
3.29354  2.67741  2.69355  4.24690  2.90347  2.73739  3.18146  2.89801
2.37887  2.77519  2.98518  4.58477  3.61503
          0.03133  3.87472  4.59707  0.61958  0.77255  0.48576  0.95510
    322   2.74713  4.42461  3.57854  3.18691  3.09973  2.36605  3.86723
3.17580  3.03172  2.79783  3.78929  3.47803  3.97578  3.42361  3.28375
2.94493  3.06441  2.93484  1.57978  3.14293    322 w - - -
          2.68618  4.42225  2.77519  2.73123  3.46354  2.40513  3.72494
3.29354  2.67741  2.69355  4.24690  2.90347  2.73739  3.18146  2.89801
2.37887  2.77519  2.98518  4.58477  3.61503
          0.03133  3.87472  4.59707  0.61958  0.77255  0.48576  0.95510
    323   3.03284  4.45083  4.33904  3.95485  3.37561  4.06909  4.68249
1.07512  3.78303  1.96109  3.31052  4.19987  4.50000  4.13104  3.97934
3.56453  3.33193  1.84577  5.26697  4.00552    323 i - - -
          2.68618  4.42225  2.77519  2.73123  3.46354  2.40513  3.72494
3.29354  2.67741  2.69355  4.24690  2.90347  2.73739  3.18146  2.89801
2.37887  2.77519  2.98518  4.58477  3.61503
          0.03133  3.87472  4.59707  0.61958  0.77255  0.48576  0.95510
    324   2.66752  4.44283  3.34177  3.24141  4.38386  0.72564  4.36163
3.98394  3.42300  3.63781  4.61350  3.50605  3.83404  3.74696  3.65888
2.83948  3.14893  3.51182  5.47850  4.48580    324 g - - -
          2.68618  4.42225  2.77519  2.73123  3.46354  2.40513  3.72494
3.29354  2.67741  2.69355  4.24690  2.90347  2.73739  3.18146  2.89801
2.37887  2.77519  2.98518  4.58477  3.61503
          0.03133  3.87472  4.59707  0.61958  0.77255  0.48576  0.95510
    325   2.72515  4.72193  2.70930  2.59495  4.08579  3.18536  3.92257
3.79656  2.82640  3.44315  4.39831  1.15407  3.83729  3.19525  3.18120
2.79220  3.10703  3.41854  5.39143  4.03390    325 n - - -
          2.68618  4.42225  2.77519  2.73123  3.46354  2.40513  3.72494
3.29354  2.67741  2.69355  4.24690  2.90347  2.73739  3.18146  2.89801
2.37887  2.77519  2.98518  4.58477  3.61503
          0.03133  3.87472  4.59707  0.61958  0.77255  0.48576  0.95510
    326   2.66752  4.44283  3.34177  3.24141  4.38386  0.72564  4.36163
3.98394  3.42300  3.63781  4.61350  3.50605  3.83404  3.74696  3.65888
2.83948  3.14893  3.51182  5.47850  4.48580    326 g - - -
          2.68618  4.42225  2.77519  2.73123  3.46354  2.40513  3.72494
3.29354  2.67741  2.69355  4.24690  2.90347  2.73739  3.18146  2.89801
2.37887  2.77519  2.98518  4.58477  3.61503
          0.03133  3.87472  4.59707  0.61958  0.77255  0.48576  0.95510
    327   3.00784  4.84699  3.49870  3.00918  4.20214  3.49552  3.82565
3.74393  2.23793  3.25944  4.25948  3.36879  3.99792  3.04579  0.97495
3.09185  3.27359  3.46776  5.28422  4.12892    327 r - - -
          2.68618  4.42225  2.77519  2.73123  3.46354  2.40513  3.72494
3.29354  2.67741  2.69355  4.24690  2.90347  2.73739  3.18146  2.89801
2.37887  2.77519  2.98518  4.58477  3.61503
          0.03133  3.87472  4.59707  0.61958  0.77255  0.48576  0.95510
```

```
    328   2.70181  4.29651  3.81198  3.28290  2.57590  3.70747  4.02908
2.39516  3.18462  1.82926  3.22330  3.60029  4.10939  3.47553  3.46424
3.01742  2.18353  2.28104  4.80629  3.49167    328 l - - -
          2.68618  4.42225  2.77519  2.73123  3.46354  2.40513  3.72494
3.29354  2.67741  2.69355  4.24690  2.90347  2.73739  3.18146  2.89801
2.37887  2.77519  2.98518  4.58477  3.61503
          0.03133  3.87472  4.59707  0.61958  0.77255  0.48576  0.95510
    329   2.42917  4.52064  2.70975  2.49436  4.29148  2.40269  3.83785
3.75953  2.73576  3.37073  4.19695  2.01115  3.70436  3.03496  3.16109
2.08142  2.81968  3.29389  5.56511  4.23790    329 n - - -
          2.68618  4.42225  2.77519  2.73123  3.46354  2.40513  3.72494
3.29354  2.67741  2.69355  4.24690  2.90347  2.73739  3.18146  2.89801
2.37887  2.77519  2.98518  4.58477  3.61503
          0.03133  3.87472  4.59707  0.61958  0.77255  0.48576  0.95510
    330   2.57127  4.67243  2.97002  2.48305  3.98093  2.78386  3.63518
3.06411  2.37730  3.00986  3.84853  2.43252  3.79241  2.80890  2.41229
2.63194  2.82698  3.06947  5.24846  3.94247    330 k - - -
          2.68618  4.42225  2.77519  2.73123  3.46354  2.40513  3.72494
3.29354  2.67741  2.69355  4.24690  2.90347  2.73739  3.18146  2.89801
2.37887  2.77519  2.98518  4.58477  3.61503
          0.03133  3.87472  4.59707  0.61958  0.77255  0.48576  0.95510
    331   3.19270  4.58486  4.42115  3.96590  1.53356  4.19931  4.03413
2.40943  3.82016  1.49515  3.11564  4.10143  4.50912  3.97760  3.96909
3.57192  3.43656  2.45574  4.31116  2.74481    331 l - - -
          2.68618  4.42225  2.77519  2.73123  3.46354  2.40513  3.72494
3.29354  2.67741  2.69355  4.24690  2.90347  2.73739  3.18146  2.89801
2.37887  2.77519  2.98518  4.58477  3.61503
          0.03133  3.87472  4.59707  0.61958  0.77255  0.48576  0.95510
    332   2.46971  4.47572  2.99029  2.59957  3.90122  3.22215  3.76278
2.93784  2.61644  2.99892  3.86334  2.35498  3.76606  2.98033  3.00756
1.93838  2.79553  2.97194  5.25015  3.93493    332 s - - -
          2.68618  4.42225  2.77519  2.73123  3.46354  2.40513  3.72494
3.29354  2.67741  2.69355  4.24690  2.90347  2.73739  3.18146  2.89801
2.37887  2.77519  2.98518  4.58477  3.61503
          0.03133  3.87472  4.59707  0.61958  0.77255  0.48576  0.95510
    333   2.19126  4.31362  3.82708  3.41634  1.72871  3.57962  4.00068
2.55643  3.35085  2.20200  3.38554  3.63513  4.09091  3.61657  3.59377
2.97306  3.01323  2.42675  4.61211  3.13621    333 f - - -
          2.68618  4.42225  2.77519  2.73123  3.46354  2.40513  3.72494
3.29354  2.67741  2.69355  4.24690  2.90347  2.73739  3.18146  2.89801
2.37887  2.77519  2.98518  4.58477  3.61503
          0.03133  3.87472  4.59707  0.61958  0.77255  0.48576  0.95510
    334   1.60926  4.21336  3.23413  2.94453  4.09999  2.99253  4.05603
3.40193  2.98648  3.14807  4.04860  3.20252  2.03506  3.31928  3.31230
2.44906  2.72837  2.98508  5.46484  4.23166    334 a - - -
          2.68618  4.42225  2.77519  2.73123  3.46354  2.40513  3.72494
3.29354  2.67741  2.69355  4.24690  2.90347  2.73739  3.18146  2.89801
2.37887  2.77519  2.98518  4.58477  3.61503
          0.03133  3.87472  4.59707  0.61958  0.77255  0.48576  0.95510
    335   2.55762  3.27814  2.35616  2.60505  3.54719  3.40157  3.68989
3.04876  2.62676  2.74497  3.63312  3.06795  3.83459  2.95235  3.03804
2.68001  2.81190  2.78822  4.97161  2.93322    335 d - - -
          2.68618  4.42225  2.77519  2.73123  3.46354  2.40513  3.72494
3.29354  2.67741  2.69355  4.24690  2.90347  2.73739  3.18146  2.89801
2.37887  2.77519  2.98518  4.58477  3.61503
```

```
         0.03133  3.87472  4.59707  0.61958  0.77255  0.48576  0.95510
    336  2.45071  4.51931  2.73642  2.53112  4.20366  3.07349  3.84727
3.72224  2.72905  3.35410  4.20067  1.99554  3.72235  3.06064  3.12626
1.77209  2.84366  3.27443  5.50609  4.15874    336 s - - -
         2.68618  4.42225  2.77519  2.73123  3.46354  2.40513  3.72494
3.29354  2.67741  2.69355  4.24690  2.90347  2.73739  3.18146  2.89801
2.37887  2.77519  2.98518  4.58477  3.61503
         0.03133  3.87472  4.59707  0.61958  0.77255  0.48576  0.95510
    337  2.69055  4.82460  2.77200  2.42207  3.89168  3.36429  3.63085
3.50557  2.23867  3.10468  3.96726  1.93590  3.83100  2.83087  2.77039
2.71883  2.93785  3.19539  5.20782  3.26940    337 n - - -
         2.68618  4.42225  2.77519  2.73123  3.46354  2.40513  3.72494
3.29354  2.67741  2.69355  4.24690  2.90347  2.73739  3.18146  2.89801
2.37887  2.77519  2.98518  4.58477  3.61503
         0.03133  3.87472  4.59707  0.61958  0.77255  0.48576  0.95510
    338  2.72937  4.93629  2.43418  1.61979  4.41214  2.03336  3.76580
3.86258  2.65814  3.45708  4.32089  2.82788  3.79370  2.95202  3.11289
2.72668  3.04524  3.47702  5.61750  4.27135    338 e - - -
         2.68618  4.42225  2.77519  2.73123  3.46354  2.40513  3.72494
3.29354  2.67741  2.69355  4.24690  2.90347  2.73739  3.18146  2.89801
2.37887  2.77519  2.98518  4.58477  3.61503
         0.03133  3.87472  4.59707  0.61958  0.77255  0.48576  0.95510
    339  2.65863  4.53159  3.11120  2.75126  3.53281  3.41501  3.80613
2.97258  2.67632  1.92854  3.70642  2.18220  3.91529  3.10115  3.00770
2.80075  2.94852  2.75784  5.02174  3.64131    339 l - - -
         2.68618  4.42225  2.77519  2.73123  3.46354  2.40513  3.72494
3.29354  2.67741  2.69355  4.24690  2.90347  2.73739  3.18146  2.89801
2.37887  2.77519  2.98518  4.58477  3.61503
         0.03133  3.87472  4.59707  0.61958  0.77255  0.48576  0.95510
    340  2.95406  5.06580  2.48830  1.02217  4.41227  3.27540  3.86227
3.86661  2.74307  3.48333  4.45717  2.93393  3.88006  3.07782  3.15222
2.93617  3.25828  3.55019  5.58341  4.30555    340 e - - -
         2.68618  4.42225  2.77519  2.73123  3.46354  2.40513  3.72494
3.29354  2.67741  2.69355  4.24690  2.90347  2.73739  3.18146  2.89801
2.37887  2.77519  2.98518  4.58477  3.61503
         0.03133  3.87472  4.59707  0.61958  0.77255  0.48576  0.95510
    341  3.03284  4.45083  4.33904  3.95485  3.37561  4.06909  4.68249
1.07512  3.78303  1.96109  3.31052  4.19987  4.50000  4.13104  3.97934
3.56453  3.33193  1.84577  5.26697  4.00552    341 i - - -
         2.68618  4.42225  2.77519  2.73123  3.46354  2.40513  3.72494
3.29354  2.67741  2.69355  4.24690  2.90347  2.73739  3.18146  2.89801
2.37887  2.77519  2.98518  4.58477  3.61503
         0.03133  3.87472  4.59707  0.61958  0.77255  0.48576  0.95510
    342  2.59312  4.29800  3.65288  3.16585  3.45500  3.50365  4.05677
2.50987  3.02039  2.23981  2.28819  3.48265  3.99994  3.38745  3.30989
2.85865  2.05839  2.35888  5.06940  3.84537    342 t - - -
         2.68618  4.42225  2.77519  2.73123  3.46354  2.40513  3.72494
3.29354  2.67741  2.69355  4.24690  2.90347  2.73739  3.18146  2.89801
2.37887  2.77519  2.98518  4.58477  3.61503
         0.03133  3.87472  4.59707  0.61958  0.77255  0.48576  0.95510
    343  2.82914  5.11986  2.61923  1.79503  4.46003  3.35762  3.62668
3.86821  1.70386  3.39756  4.24846  2.86538  3.84857  2.77590  2.58276
2.79398  3.06441  3.51773  5.53514  4.21473    343 k - - -
```

```
         2.68618  4.42225  2.77519  2.73123  3.46354  2.40513  3.72494
3.29354  2.67741  2.69355  4.24690  2.90347  2.73739  3.18146  2.89801
2.37887  2.77519  2.98518  4.58477  3.61503
         0.03133  3.87472  4.59707  0.61958  0.77255  0.48576  0.95510
    344  2.97470  4.36103  4.50976  4.02044  3.47167  4.20565  4.77669
1.55509  3.89314  2.01199  3.31015  4.26594  4.57094  4.18848  4.11842
3.58932  3.25322  1.17476  5.39067  4.16721    344 v - - -
         2.68618  4.42225  2.77519  2.73123  3.46354  2.40513  3.72494
3.29354  2.67741  2.69355  4.24690  2.90347  2.73739  3.18146  2.89801
2.37887  2.77519  2.98518  4.58477  3.61503
         0.03133  3.87472  4.59707  0.61958  0.77255  0.48576  0.95510
    345  2.84177  5.06748  3.02096  2.16969  4.44309  3.48769  3.56413
3.82466  1.78346  3.31460  4.15260  2.99135  3.89034  2.69847  1.80580
2.82795  3.03966  3.48592  5.42085  4.16931    345 k - - -
         2.68618  4.42225  2.77519  2.73123  3.46354  2.40513  3.72494
3.29354  2.67741  2.69355  4.24690  2.90347  2.73739  3.18146  2.89801
2.37887  2.77519  2.98518  4.58477  3.61503
         0.03133  3.87472  4.59707  0.61958  0.77255  0.48576  0.95510
    346  2.59414  4.63827  2.92657  2.56797  4.04099  3.28563  3.73590
3.41068  2.45166  3.04426  3.95386  3.01902  3.81445  2.05690  2.79207
2.67420  2.08829  3.09845  5.33473  4.03589    346 q - - -
         2.68618  4.42225  2.77519  2.73123  3.46354  2.40513  3.72494
3.29354  2.67741  2.69355  4.24690  2.90347  2.73739  3.18146  2.89801
2.37887  2.77519  2.98518  4.58477  3.61503
         0.03133  3.87472  4.59707  0.61958  0.77255  0.48576  0.95510
    347  2.80882  5.13889  2.46208  1.70743  4.42438  3.29685  3.65103
3.88447  2.38903  3.41215  4.27193  2.80024  3.82288  2.03176  2.78870
2.76401  3.06172  3.52917  5.57720  4.20640    347 e - - -
         2.68618  4.42225  2.77519  2.73123  3.46354  2.40513  3.72494
3.29354  2.67741  2.69355  4.24690  2.90347  2.73739  3.18146  2.89801
2.37887  2.77519  2.98518  4.58477  3.61503
         0.03133  3.87472  4.59707  0.61958  0.77255  0.48576  0.95510
    348  2.67539  4.28325  3.87029  3.40369  3.50918  3.65582  4.26638
1.65137  3.27564  2.25103  3.41376  3.68843  4.14610  3.62544  3.54889
3.02861  2.11172  2.00010  5.18896  3.95835    348 i - - -
         2.68618  4.42225  2.77519  2.73123  3.46354  2.40513  3.72494
3.29354  2.67741  2.69355  4.24690  2.90347  2.73739  3.18146  2.89801
2.37887  2.77519  2.98518  4.58477  3.61503
         0.03133  3.87472  4.59707  0.61958  0.77255  0.48576  0.95510
    349  2.61980  4.28398  3.71185  3.25944  2.14363  3.52244  3.98281
2.57454  3.18207  2.26745  3.39455  3.53017  4.02365  3.47926  3.45688
2.88980  2.02480  2.42001  4.74354  3.33645    349 t - - -
         2.68618  4.42225  2.77519  2.73123  3.46354  2.40513  3.72494
3.29354  2.67741  2.69355  4.24690  2.90347  2.73739  3.18146  2.89801
2.37887  2.77519  2.98518  4.58477  3.61503
         0.03133  3.87472  4.59707  0.61958  0.77255  0.48576  0.95510
    350  3.25019  4.73827  3.84149  3.57122  2.29338  3.83270  3.61936
3.25908  3.42867  2.71244  3.93385  3.72612  4.30645  3.73417  3.61195
3.38669  3.53686  3.12972  3.93726  0.96205    350 y - - -
         2.68618  4.42225  2.77519  2.73123  3.46354  2.40513  3.72494
3.29354  2.67741  2.69355  4.24690  2.90347  2.73739  3.18146  2.89801
2.37887  2.77519  2.98518  4.58477  3.61503
         0.03133  3.87472  4.59707  0.61958  0.77255  0.48576  0.95510
```

```
    351   2.74880  4.98697  2.56338  2.33377  4.27116  3.28478  3.66767
3.81597  2.39609  3.36250  4.22287  1.99891  3.81913  2.03819  2.77326
2.73798  3.02275  3.45421  5.47838  4.10180    351 n - - -
          2.68618  4.42225  2.77519  2.73123  3.46354  2.40513  3.72494
3.29354  2.67741  2.69355  4.24690  2.90347  2.73739  3.18146  2.89801
2.37887  2.77519  2.98518  4.58477  3.61503
          0.03133  3.87472  4.59707  0.61958  0.77255  0.48576  0.95510
    352   2.80790  5.21525  1.73354  2.12479  4.53903  3.17046  3.72363
4.08535  2.70886  3.63285  4.48820  1.75598  3.78427  2.90051  3.25760
2.73919  3.11116  3.67382  5.76512  4.30008    352 d - - -
          2.68618  4.42225  2.77519  2.73123  3.46354  2.40513  3.72494
3.29354  2.67741  2.69355  4.24690  2.90347  2.73739  3.18146  2.89801
2.37887  2.77519  2.98518  4.58477  3.61503
          0.03133  3.87472  4.59707  0.61958  0.77255  0.48576  0.95510
    353   2.69990  4.80128  2.91526  2.53841  4.23264  3.33756  3.68872
3.64054  1.69959  3.22449  4.09416  3.00510  2.32396  2.86037  2.61494
2.74251  2.97534  3.30073  5.39377  4.12117    353 k - - -
          2.68618  4.42225  2.77519  2.73123  3.46354  2.40513  3.72494
3.29354  2.67741  2.69355  4.24690  2.90347  2.73739  3.18146  2.89801
2.37887  2.77519  2.98518  4.58477  3.61503
          0.23773  3.87472  1.65641  0.61958  0.77255  0.48576  0.95510
    354   2.45167  4.23243  3.08270  2.96402  4.08319  1.11264  4.08559
3.64371  3.11585  3.32185  4.30595  3.25026  3.61727  3.45864  3.36327
2.62016  2.91812  3.21260  5.22045  4.18227    354 g - - -
          2.68618  4.42225  2.77519  2.73123  3.46354  2.40513  3.72494
3.29354  2.67741  2.69355  4.24690  2.90347  2.73739  3.18146  2.89801
2.37887  2.77519  2.98518  4.58477  3.61503
          0.03837  3.67537  4.39771  0.61958  0.77255  0.63884  0.75058
    355   2.59110  4.73225  1.82571  2.23209  4.28118  2.19013  3.73498
3.76779  2.72733  3.39168  4.26960  2.75164  3.67442  2.94509  3.21265
2.61075  2.94348  3.36386  5.51407  4.18105    355 d - - -
          2.68618  4.42225  2.77519  2.73123  3.46354  2.40513  3.72494
3.29354  2.67741  2.69355  4.24690  2.90347  2.73739  3.18146  2.89801
2.37887  2.77519  2.98518  4.58477  3.61503
          0.03837  3.67537  4.39771  0.61958  0.77255  0.40072  1.10818
    356   2.74770  4.92068  2.61671  2.38375  3.87115  3.30352  2.72199
3.74030  2.47227  3.29227  4.16995  1.77286  3.83595  2.88952  2.85510
2.75139  3.02534  3.39502  5.22171  3.75538    356 n - - -
          2.68618  4.42225  2.77519  2.73123  3.46354  2.40513  3.72494
3.29354  2.67741  2.69355  4.24690  2.90347  2.73739  3.18146  2.89801
2.37887  2.77519  2.98518  4.58477  3.61503
          0.03133  3.87472  4.59707  0.61958  0.77255  0.48576  0.95510
    357   2.53452  4.36332  3.25263  2.74201  2.83533  3.42747  3.73153
2.69438  2.71144  2.61784  3.52177  2.55223  3.85855  3.03969  3.08702
2.37513  2.79340  2.65800  4.87992  3.54740    357 s - - -
          2.68618  4.42225  2.77519  2.73123  3.46354  2.40513  3.72494
3.29354  2.67741  2.69355  4.24690  2.90347  2.73739  3.18146  2.89801
2.37887  2.77519  2.98518  4.58477  3.61503
          0.03133  3.87472  4.59707  0.61958  0.77255  0.48576  0.95510
    358   2.80639  5.16667  2.30323  1.66003  4.48263  3.22111  3.69549
3.97033  2.56615  3.52592  4.38186  1.90905  3.80129  2.86595  3.03527
2.75103  3.08912  3.58859  5.67831  4.26054    358 e - - -
          2.68618  4.42225  2.77519  2.73123  3.46354  2.40513  3.72494
3.29354  2.67741  2.69355  4.24690  2.90347  2.73739  3.18146  2.89801
2.37887  2.77519  2.98518  4.58477  3.61503
```

```
          0.03133  3.87472  4.59707  0.61958  0.77255  0.48576  0.95510
    359   2.29066  4.26619  3.09152  2.82123  4.15546  2.98297  3.99018
3.60389  2.90028  3.27334  4.12730  3.12364  2.12398  3.23519  3.24758
1.66406  2.73311  3.13100  5.48492  4.21511    359 s - - -
          2.68618  4.42225  2.77519  2.73123  3.46354  2.40513  3.72494
3.29354  2.67741  2.69355  4.24690  2.90347  2.73739  3.18146  2.89801
2.37887  2.77519  2.98518  4.58477  3.61503
          0.03133  3.87472  4.59707  0.61958  0.77255  0.48576  0.95510
    360   3.03284  4.45083  4.33904  3.95485  3.37561  4.06909  4.68249
1.07512  3.78303  1.96109  3.31052  4.19987  4.50000  4.13104  3.97934
3.56453  3.33193  1.84577  5.26697  4.00552    360 i - - -
          2.68618  4.42225  2.77519  2.73123  3.46354  2.40513  3.72494
3.29354  2.67741  2.69355  4.24690  2.90347  2.73739  3.18146  2.89801
2.37887  2.77519  2.98518  4.58477  3.61503
          0.03133  3.87472  4.59707  0.61958  0.77255  0.48576  0.95510
    361   1.97248  4.13458  3.52473  3.12743  3.78844  2.47773  4.08532
2.82930  3.09756  2.78863  3.72989  3.34476  3.77655  3.40625  3.40017
2.53822  2.72263  1.79507  5.25349  4.03217    361 v - - -
          2.68618  4.42225  2.77519  2.73123  3.46354  2.40513  3.72494
3.29354  2.67741  2.69355  4.24690  2.90347  2.73739  3.18146  2.89801
2.37887  2.77519  2.98518  4.58477  3.61503
          0.03133  3.87472  4.59707  0.61958  0.77255  0.48576  0.95510
    362   2.75420  4.51129  3.36160  3.20088  4.19604  3.19867  4.27823
3.74193  3.25835  3.36857  4.41844  3.50740  0.82346  3.64706  3.50371
2.92174  3.19592  3.38706  5.38207  4.31670    362 p - - -
          2.68618  4.42225  2.77519  2.73123  3.46354  2.40513  3.72494
3.29354  2.67741  2.69355  4.24690  2.90347  2.73739  3.18146  2.89801
2.37887  2.77519  2.98518  4.58477  3.61503
          0.03133  3.87472  4.59707  0.61958  0.77255  0.48576  0.95510
    363   1.83409  4.51861  3.21194  2.75114  4.04274  3.27351  3.77464
3.37456  2.40751  3.04196  3.94248  3.13365  3.81650  2.97690  1.94742
2.66007  2.87072  3.04827  5.30875  4.05838    363 a - - -
          2.68618  4.42225  2.77519  2.73123  3.46354  2.40513  3.72494
3.29354  2.67741  2.69355  4.24690  2.90347  2.73739  3.18146  2.89801
2.37887  2.77519  2.98518  4.58477  3.61503
          0.03133  3.87472  4.59707  0.61958  0.77255  0.48576  0.95510
    364   2.23523  4.95909  1.96957  2.21521  4.40311  2.83309  3.68886
3.86170  2.60208  3.42723  4.24669  2.30871  3.75099  2.84935  3.11856
2.63090  2.94702  3.45487  5.62641  4.22222    364 d - - -
          2.68618  4.42225  2.77519  2.73123  3.46354  2.40513  3.72494
3.29354  2.67741  2.69355  4.24690  2.90347  2.73739  3.18146  2.89801
2.37887  2.77519  2.98518  4.58477  3.61503
          0.03133  3.87472  4.59707  0.61958  0.77255  0.48576  0.95510
    365   2.66336  4.28262  3.84497  3.37839  3.51443  3.63481  4.24797
1.69725  3.25170  2.26494  3.42173  3.66571  4.12942  3.60291  3.52809
3.00709  2.06156  2.02200  5.18516  3.95321    365 i - - -
          2.68618  4.42225  2.77519  2.73123  3.46354  2.40513  3.72494
3.29354  2.67741  2.69355  4.24690  2.90347  2.73739  3.18146  2.89801
2.37887  2.77519  2.98518  4.58477  3.61503
          0.03133  3.87472  4.59707  0.61958  0.77255  0.48576  0.95510
    366   2.73133  4.49737  3.52446  2.95989  3.58130  3.59751  3.83322
2.04214  2.51328  2.47394  3.54197  3.34375  4.00847  3.09695  2.02287
2.92778  2.97646  2.55819  5.05696  3.79956    366 r - - -
```

```
          2.68618  4.42225  2.77519  2.73123  3.46354  2.40513  3.72494
3.29354  2.67741  2.69355  4.24690  2.90347  2.73739  3.18146  2.89801
2.37887  2.77519  2.98518  4.58477  3.61503
          0.03133  3.87472  4.59707  0.61958  0.77255  0.48576  0.95510
    367   2.72515  4.72193  2.70930  2.59495  4.08579  3.18536  3.92257
3.79656  2.82640  3.44315  4.39831  1.15407  3.83729  3.19525  3.18120
2.79220  3.10703  3.41854  5.39143  4.03390    367 n - - -
          2.68618  4.42225  2.77519  2.73123  3.46354  2.40513  3.72494
3.29354  2.67741  2.69355  4.24690  2.90347  2.73739  3.18146  2.89801
2.37887  2.77519  2.98518  4.58477  3.61503
          0.03133  3.87472  4.59707  0.61958  0.77255  0.48576  0.95510
    368   2.80882  5.13889  2.46208  1.70743  4.42438  3.29685  3.65103
3.88447  2.38903  3.41215  4.27193  2.80024  3.82288  2.03176  2.78870
2.76401  3.06172  3.52917  5.57720  4.20640    368 e - - -
          2.68618  4.42225  2.77519  2.73123  3.46354  2.40513  3.72494
3.29354  2.67741  2.69355  4.24690  2.90347  2.73739  3.18146  2.89801
2.37887  2.77519  2.98518  4.58477  3.61503
          0.03133  3.87472  4.59707  0.61958  0.77255  0.48576  0.95510
    369   3.03284  4.45083  4.33904  3.95485  3.37561  4.06909  4.68249
1.07512  3.78303  1.96109  3.31052  4.19987  4.50000  4.13104  3.97934
3.56453  3.33193  1.84577  5.26697  4.00552    369 i - - -
          2.68618  4.42225  2.77519  2.73123  3.46354  2.40513  3.72494
3.29354  2.67741  2.69355  4.24690  2.90347  2.73739  3.18146  2.89801
2.37887  2.77519  2.98518  4.58477  3.61503
          0.03133  3.87472  4.59707  0.61958  0.77255  0.48576  0.95510
    370   3.15771  4.61926  4.19314  3.83291  3.13642  3.98506  4.48794
2.34049  3.60064  0.87242  3.15851  4.10818  4.41998  3.96843  3.77638
3.55606  3.45182  2.37843  5.01661  3.74048    370 l - - -
          2.68618  4.42225  2.77519  2.73123  3.46354  2.40513  3.72494
3.29354  2.67741  2.69355  4.24690  2.90347  2.73739  3.18146  2.89801
2.37887  2.77519  2.98518  4.58477  3.61503
          0.03133  3.87472  4.59707  0.61958  0.77255  0.48576  0.95510
    371   2.41377  4.25888  3.42992  3.18193  3.97070  3.09811  4.19910
3.09810  3.12495  2.96152  4.01182  3.39101  3.80201  3.51964  3.38358
2.61505  1.16810  2.79284  5.40831  4.18731    371 t - - -
          2.68618  4.42225  2.77519  2.73123  3.46354  2.40513  3.72494
3.29354  2.67741  2.69355  4.24690  2.90347  2.73739  3.18146  2.89801
2.37887  2.77519  2.98518  4.58477  3.61503
          0.03133  3.87472  4.59707  0.61958  0.77255  0.48576  0.95510
    372   1.01052  4.19303  3.46476  3.25606  4.05451  3.01753  4.27786
3.18035  3.27238  3.06187  4.09337  3.40514  3.75695  3.61331  3.52281
2.54327  2.82077  2.83436  5.48144  4.28192    372 a - - -
          2.68618  4.42225  2.77519  2.73123  3.46354  2.40513  3.72494
3.29354  2.67741  2.69355  4.24690  2.90347  2.73739  3.18146  2.89801
2.37887  2.77519  2.98518  4.58477  3.61503
          0.23773  3.87472  1.65641  0.61958  0.77255  0.48576  0.95510
    373   2.32614  4.16623  3.26790  3.02488  3.80754  3.00808  4.05508
2.91224  2.96371  2.78125  3.85587  3.25951  3.70039  3.37225  3.22512
2.52646  1.56100  2.62960  5.26461  4.02761    373 t - - -
          2.68618  4.42225  2.77519  2.73123  3.46354  2.40513  3.72494
3.29354  2.67741  2.69355  4.24690  2.90347  2.73739  3.18146  2.89801
2.37887  2.77519  2.98518  4.58477  3.61503
          0.03837  3.67537  4.39771  0.61958  0.77255  0.63884  0.75058
```

```
    374   2.65219  4.26112  3.83823  3.50789  3.41605  3.50322  4.34649
2.10447  3.37156  2.14909  3.42613  3.72537  4.08342  3.75139  3.60014
3.00431  3.02431  1.32331  5.16185  3.87506    374 v - - -
          2.68618  4.42225  2.77519  2.73123  3.46354  2.40513  3.72494
3.29354  2.67741  2.69355  4.24690  2.90347  2.73739  3.18146  2.89801
2.37887  2.77519  2.98518  4.58477  3.61503
          0.03837  3.67537  4.39771  0.61958  0.77255  0.40072  1.10818
    375   2.75420  4.51129  3.36160  3.20088  4.19604  3.19867  4.27823
3.74193  3.25835  3.36857  4.41844  3.50740  0.82346  3.64706  3.50371
2.92174  3.19592  3.38706  5.38207  4.31670    375 p - - -
          2.68618  4.42225  2.77519  2.73123  3.46354  2.40513  3.72494
3.29354  2.67741  2.69355  4.24690  2.90347  2.73739  3.18146  2.89801
2.37887  2.77519  2.98518  4.58477  3.61503
          0.03133  3.87472  4.59707  0.61958  0.77255  0.48576  0.95510
    376   1.97664  4.20106  3.45327  2.97283  3.65797  2.89570  3.93800
2.34114  2.92256  2.67360  3.59489  3.28572  3.81625  3.24346  3.25706
2.61192  2.30961  2.52246  5.10735  3.87700    376 a - - -
          2.68618  4.42225  2.77519  2.73123  3.46354  2.40513  3.72494
3.29354  2.67741  2.69355  4.24690  2.90347  2.73739  3.18146  2.89801
2.37887  2.77519  2.98518  4.58477  3.61503
          0.03133  3.87472  4.59707  0.61958  0.77255  0.48576  0.95510
    377   2.27239  4.24300  3.17291  2.82358  4.09848  3.00665  3.95128
3.48206  2.85180  3.16826  4.01199  3.12739  2.22170  3.18639  3.20933
2.01312  2.18648  3.04004  5.43271  4.17962    377 s - - -
          2.68618  4.42225  2.77519  2.73123  3.46354  2.40513  3.72494
3.29354  2.67741  2.69355  4.24690  2.90347  2.73739  3.18146  2.89801
2.37887  2.77519  2.98518  4.58477  3.61503
          0.03133  3.87472  4.59707  0.61958  0.77255  0.48576  0.95510
    378   1.01052  4.19303  3.46476  3.25606  4.05451  3.01753  4.27786
3.18035  3.27238  3.06187  4.09337  3.40514  3.75695  3.61331  3.52281
2.54327  2.82077  2.83436  5.48144  4.28192    378 a - - -
          2.68618  4.42225  2.77519  2.73123  3.46354  2.40513  3.72494
3.29354  2.67741  2.69355  4.24690  2.90347  2.73739  3.18146  2.89801
2.37887  2.77519  2.98518  4.58477  3.61503
          0.03133  3.87472  4.59707  0.61958  0.77255  0.48576  0.95510
    379   2.99187  5.12073  0.91483  2.33607  4.49801  3.21224  3.93281
4.08666  3.01732  3.69455  4.67262  2.89258  3.86518  3.17190  3.53391
2.95391  3.33046  3.72558  5.66619  4.37043    379 d - - -
          2.68618  4.42225  2.77519  2.73123  3.46354  2.40513  3.72494
3.29354  2.67741  2.69355  4.24690  2.90347  2.73739  3.18146  2.89801
2.37887  2.77519  2.98518  4.58477  3.61503
          0.03133  3.87472  4.59707  0.61958  0.77255  0.48576  0.95510
    380   2.64207  4.41461  3.43481  3.02061  3.49163  3.44628  3.97970
2.77209  2.89814  1.74296  3.52411  3.38010  2.21988  3.29864  3.19980
2.84937  2.95855  2.61579  5.06002  3.77486    380 l - - -
          2.68618  4.42225  2.77519  2.73123  3.46354  2.40513  3.72494
3.29354  2.67741  2.69355  4.24690  2.90347  2.73739  3.18146  2.89801
2.37887  2.77519  2.98518  4.58477  3.61503
          0.03133  3.87472  4.59707  0.61958  0.77255  0.48576  0.95510
    381   3.26109  4.66217  4.24182  3.95666  1.00804  3.95049  3.87804
2.78442  3.86362  2.15392  3.51419  4.02166  4.40896  4.03095  3.98279
3.53096  3.56220  2.77530  4.12630  2.49298    381 f - - -
          2.68618  4.42225  2.77519  2.73123  3.46354  2.40513  3.72494
3.29354  2.67741  2.69355  4.24690  2.90347  2.73739  3.18146  2.89801
2.37887  2.77519  2.98518  4.58477  3.61503
```

```
         0.03133  3.87472  4.59707  0.61958  0.77255  0.48576  0.95510
    382   3.26109  4.66217  4.24182  3.95666  1.00804  3.95049  3.87804
2.78442  3.86362  2.15392  3.51419  4.02166  4.40896  4.03095  3.98279
3.53096  3.56220  2.77530  4.12630  2.49298    382 f - - -
         2.68618  4.42225  2.77519  2.73123  3.46354  2.40513  3.72494
3.29354  2.67741  2.69355  4.24690  2.90347  2.73739  3.18146  2.89801
2.37887  2.77519  2.98518  4.58477  3.61503
         0.03133  3.87472  4.59707  0.61958  0.77255  0.48576  0.95510
    383   2.97404  4.93809  3.15019  2.77112  4.32503  3.46335  3.76023
3.75442  1.03520  3.31897  4.28021  3.19552  3.96346  2.95151  2.43176
3.01582  3.22709  3.46205  5.37869  4.19175    383 k - - -
         2.68618  4.42225  2.77519  2.73123  3.46354  2.40513  3.72494
3.29354  2.67741  2.69355  4.24690  2.90347  2.73739  3.18146  2.89801
2.37887  2.77519  2.98518  4.58477  3.61503
         0.03133  3.87472  4.59707  0.61958  0.77255  0.48576  0.95510
    384   2.45388  4.47570  2.87366  2.60769  4.10590  3.12386  3.84748
3.48080  2.69540  3.19046  4.06415  2.05000  3.74620  3.06459  3.06875
2.56118  1.92748  3.09874  5.42744  4.11560    384 t - - -
         2.68618  4.42225  2.77519  2.73123  3.46354  2.40513  3.72494
3.29354  2.67741  2.69355  4.24690  2.90347  2.73739  3.18146  2.89801
2.37887  2.77519  2.98518  4.58477  3.61503
         0.03133  3.87472  4.59707  0.61958  0.77255  0.48576  0.95510
    385   1.01052  4.19303  3.46476  3.25606  4.05451  3.01753  4.27786
3.18035  3.27238  3.06187  4.09337  3.40514  3.75695  3.61331  3.52281
2.54327  2.82077  2.83436  5.48144  4.28192    385 a - - -
         2.68618  4.42225  2.77519  2.73123  3.46354  2.40513  3.72494
3.29354  2.67741  2.69355  4.24690  2.90347  2.73739  3.18146  2.89801
2.37887  2.77519  2.98518  4.58477  3.61503
         0.03133  3.87472  4.59707  0.61958  0.77255  0.48576  0.95510
    386   2.99187  5.12073  0.91483  2.33607  4.49801  3.21224  3.93281
4.08666  3.01732  3.69455  4.67262  2.89258  3.86518  3.17190  3.53391
2.95391  3.33046  3.72558  5.66619  4.37043    386 d - - -
         2.68618  4.42225  2.77519  2.73123  3.46354  2.40513  3.72494
3.29354  2.67741  2.69355  4.24690  2.90347  2.73739  3.18146  2.89801
2.37887  2.77519  2.98518  4.58477  3.61503
         0.03133  3.87472  4.59707  0.61958  0.77255  0.48576  0.95510
    387   3.03284  4.45083  4.33904  3.95485  3.37561  4.06909  4.68249
1.07512  3.78303  1.96109  3.31052  4.19987  4.50000  4.13104  3.97934
3.56453  3.33193  1.84577  5.26697  4.00552    387 i - - -
         2.68618  4.42225  2.77519  2.73123  3.46354  2.40513  3.72494
3.29354  2.67741  2.69355  4.24690  2.90347  2.73739  3.18146  2.89801
2.37887  2.77519  2.98518  4.58477  3.61503
         0.03133  3.87472  4.59707  0.61958  0.77255  0.48576  0.95510
    388   2.72515  4.72193  2.70930  2.59495  4.08579  3.18536  3.92257
3.79656  2.82640  3.44315  4.39831  1.15407  3.83729  3.19525  3.18120
2.79220  3.10703  3.41854  5.39143  4.03390    388 n - - -
         2.68618  4.42225  2.77519  2.73123  3.46354  2.40513  3.72494
3.29354  2.67741  2.69355  4.24690  2.90347  2.73739  3.18146  2.89801
2.37887  2.77519  2.98518  4.58477  3.61503
         0.03133  3.87472  4.59707  0.61958  0.77255  0.48576  0.95510
    389   2.76951  4.30249  3.98623  3.47148  2.83852  3.74354  3.92237
2.50012  3.26970  2.15038  3.29614  3.69233  4.15465  3.58258  3.48295
3.09154  3.03359  1.79431  2.40037  3.01654    389 v - - -
```

```
         2.68618  4.42225  2.77519  2.73123  3.46354  2.40513  3.72494
3.29354  2.67741  2.69355  4.24690  2.90347  2.73739  3.18146  2.89801
2.37887  2.77519  2.98518  4.58477  3.61503
         0.03133  3.87472  4.59707  0.61958  0.77255  0.48576  0.95510
    390  2.95505  5.05091  3.31047  2.71349  4.46579  3.57242  3.58193
3.83281  1.32950  3.31071  4.18849  3.12715  3.96442  2.72569  1.82789
2.96208  3.13969  3.52017  5.38076  4.19215    390 k - - -
         2.68618  4.42225  2.77519  2.73123  3.46354  2.40513  3.72494
3.29354  2.67741  2.69355  4.24690  2.90347  2.73739  3.18146  2.89801
2.37887  2.77519  2.98518  4.58477  3.61503
         0.03133  3.87472  4.59707  0.61958  0.77255  0.48576  0.95510
    391  3.38852  4.71189  4.36458  3.99706  1.40423  4.19048  3.44249
3.10909  3.86117  2.48342  3.72613  3.90357  4.51714  3.91931  3.96352
3.54726  3.61700  3.02797  3.62301  1.34887    391 y - - -
         2.68623  4.42230  2.77524  2.73128  3.46359  2.40517  3.72499
3.29359  2.67745  2.69360  4.24694  2.90351  2.73744  3.18093  2.89805
2.37891  2.77485  2.98523  4.58482  3.61508
         0.23773  1.60196  4.59707  0.66506  0.72205  0.48576  0.95510
    392  3.32648  4.64441  4.44357  4.04915  1.40705  4.16534  3.50322
3.01167  3.87418  2.36094  3.60690  3.94398  4.49343  3.94175  3.95614
3.53505  3.55611  2.94903  2.05950  1.99058    394 f - - -
         2.68618  4.42225  2.77519  2.73123  3.46354  2.40513  3.72494
3.29354  2.67741  2.69355  4.24690  2.90347  2.73739  3.18146  2.89801
2.37887  2.77519  2.98518  4.58477  3.61503
         0.03133  3.87472  4.59707  0.61958  0.77255  0.48576  0.95510
    393  2.55057  4.29465  3.51881  3.10945  2.08004  3.38328  3.83900
2.89441  3.07582  2.56263  3.58793  3.38480  3.92751  3.37174  3.37297
1.92586  2.88795  2.67127  4.60568  3.10889    395 s - - -
         2.68618  4.42225  2.77519  2.73123  3.46354  2.40513  3.72494
3.29354  2.67741  2.69355  4.24690  2.90347  2.73739  3.18146  2.89801
2.37887  2.77519  2.98518  4.58477  3.61503
         0.03133  3.87472  4.59707  0.61958  0.77255  0.48576  0.95510
    394  2.67509  4.79546  2.78911  2.47747  4.16200  3.29374  3.70721
3.64120  2.41267  3.20172  4.09104  2.96041  2.76232  1.75542  2.76431
2.71366  2.96479  3.30260  5.39424  4.07682    396 q - - -
         2.68618  4.42225  2.77519  2.73123  3.46354  2.40513  3.72494
3.29354  2.67741  2.69355  4.24690  2.90347  2.73739  3.18146  2.89801
2.37887  2.77519  2.98518  4.58477  3.61503
         0.03133  3.87472  4.59707  0.61958  0.77255  0.48576  0.95510
    395  2.22146  4.18839  3.11012  2.90581  4.27179  1.77987  4.09370
3.73831  3.06346  3.41077  4.24455  3.15053  3.64007  3.35557  3.39718
1.62230  2.70183  3.18739  5.59327  4.34740    397 s - - -
         2.68618  4.42225  2.77519  2.73123  3.46354  2.40513  3.72494
3.29354  2.67741  2.69355  4.24690  2.90347  2.73739  3.18146  2.89801
2.37887  2.77519  2.98518  4.58477  3.61503
         0.03133  3.87472  4.59707  0.61958  0.77255  0.48576  0.95510
    396  2.65306  4.33968  3.69152  3.23941  3.44265  3.55356  4.12504
2.43271  3.07640  1.66114  3.39154  3.54931  4.05793  3.46371  3.35294
2.93277  2.08231  2.29625  5.10325  3.85346    398 l - - -
         2.68618  4.42225  2.77519  2.73123  3.46354  2.40513  3.72494
3.29354  2.67741  2.69355  4.24690  2.90347  2.73739  3.18146  2.89801
2.37887  2.77519  2.98518  4.58477  3.61503
         0.03133  3.87472  4.59707  0.61958  0.77255  0.48576  0.95510
```

397 3.02444 4.46864 4.16552 3.66524 2.17771 4.01128 3.74224
2.63651 3.53609 1.57413 3.28707 3.81796 4.33471 3.72011 3.72597
3.32165 3.25199 2.58612 4.07851 1.91056 399 l - - -
2.68618 4.42225 2.77519 2.73123 3.46354 2.40513 3.72494
3.29354 2.67741 2.69355 4.24690 2.90347 2.73739 3.18146 2.89801
2.37887 2.77519 2.98518 4.58477 3.61503
0.03133 3.87472 4.59707 0.61958 0.77255 0.48576 0.95510
398 2.70010 4.46562 3.35116 2.98515 2.80153 3.47647 3.67769
3.11714 2.92429 2.74590 3.74029 3.31166 3.98133 3.27538 3.22511
2.30271 3.00356 2.87844 4.38597 1.70403 400 y - - -
2.68618 4.42225 2.77519 2.73123 3.46354 2.40513 3.72494
3.29354 2.67741 2.69355 4.24690 2.90347 2.73739 3.18146 2.89801
2.37887 2.77519 2.98518 4.58477 3.61503
0.03133 3.87472 4.59707 0.61958 0.77255 0.48576 0.95510
399 2.66752 4.44283 3.34177 3.24141 4.38386 0.72564 4.36163
3.98394 3.42300 3.63781 4.61350 3.50605 3.83404 3.74696 3.65888
2.83948 3.14893 3.51182 5.47850 4.48580 401 g - - -
2.68618 4.42225 2.77519 2.73123 3.46354 2.40513 3.72494
3.29354 2.67741 2.69355 4.24690 2.90347 2.73739 3.18146 2.89801
2.37887 2.77519 2.98518 4.58477 3.61503
0.03133 3.87472 4.59707 0.61958 0.77255 0.48576 0.95510
400 3.33912 4.71305 4.09447 3.80105 2.66830 3.73256 3.90238
3.36273 3.52318 2.75727 3.99643 3.94963 4.25045 3.91067 3.65116
3.54280 3.63961 3.25312 0.88077 2.67027 402 w - - -
2.68618 4.42225 2.77519 2.73123 3.46354 2.40513 3.72494
3.29354 2.67741 2.69355 4.24690 2.90347 2.73739 3.18146 2.89801
2.37887 2.77519 2.98518 4.58477 3.61503
0.03133 3.87472 4.59707 0.61958 0.77255 0.48576 0.95510
401 2.72515 4.72193 2.70930 2.59495 4.08579 3.18536 3.92257
3.79656 2.82640 3.44315 4.39831 1.15407 3.83729 3.19525 3.18120
2.79220 3.10703 3.41854 5.39143 4.03390 403 n - - -
2.68618 4.42225 2.77519 2.73123 3.46354 2.40513 3.72494
3.29354 2.67741 2.69355 4.24690 2.90347 2.73739 3.18146 2.89801
2.37887 2.77519 2.98518 4.58477 3.61503
0.03133 3.87472 4.59707 0.61958 0.77255 0.48576 0.95510
402 3.03284 4.45083 4.33904 3.95485 3.37561 4.06909 4.68249
1.07512 3.78303 1.96109 3.31052 4.19987 4.50000 4.13104 3.97934
3.56453 3.33193 1.84577 5.26697 4.00552 404 i - - -
2.68618 4.42225 2.77519 2.73123 3.46354 2.40513 3.72494
3.29354 2.67741 2.69355 4.24690 2.90347 2.73739 3.18146 2.89801
2.37887 2.77519 2.98518 4.58477 3.61503
0.03133 3.87472 4.59707 0.61958 0.77255 0.48576 0.95510
403 2.97404 4.93809 3.15019 2.77112 4.32503 3.46335 3.76023
3.75442 1.03520 3.31897 4.28021 3.19552 3.96346 2.95151 2.43176
3.01582 3.22709 3.46205 5.37869 4.19175 405 k - - -
2.68618 4.42225 2.77519 2.73123 3.46354 2.40513 3.72494
3.29354 2.67741 2.69355 4.24690 2.90347 2.73739 3.18146 2.89801
2.37887 2.77519 2.98518 4.58477 3.61503
0.03133 3.87472 4.59707 0.61958 0.77255 0.48576 0.95510
404 3.18394 4.57158 4.46817 3.98536 1.81739 4.24176 4.17394
2.32886 3.84356 1.26758 3.01821 4.16005 4.52633 3.99840 3.99490
3.60398 3.42132 2.39767 4.47188 2.96978 406 l - - -
2.68618 4.42225 2.77519 2.73123 3.46354 2.40513 3.72494
3.29354 2.67741 2.69355 4.24690 2.90347 2.73739 3.18146 2.89801
2.37887 2.77519 2.98518 4.58477 3.61503

0.03133 3.87472 4.59707 0.61958 0.77255 0.48576 0.95510
405 2.73325 4.97521 1.58858 2.24506 4.51725 1.95245 3.80581
4.02320 2.80916 3.60185 4.46286 2.78673 3.77670 2.99780 3.33578
2.71789 3.07452 3.59420 5.72413 4.34818 407 d - - -
2.68618 4.42225 2.77519 2.73123 3.46354 2.40513 3.72494
3.29354 2.67741 2.69355 4.24690 2.90347 2.73739 3.18146 2.89801
2.37887 2.77519 2.98518 4.58477 3.61503
0.03133 3.87472 4.59707 0.61958 0.77255 0.48576 0.95510
406 2.62132 4.80210 1.81157 2.32444 4.27588 3.18179 3.76058
3.62195 2.68411 3.31112 4.18358 2.84218 3.77011 2.94451 3.16628
2.64777 2.05450 3.26114 5.56162 4.19017 408 d - - -
2.68618 4.42225 2.77519 2.73123 3.46354 2.40513 3.72494
3.29354 2.67741 2.69355 4.24690 2.90347 2.73739 3.18146 2.89801
2.37887 2.77519 2.98518 4.58477 3.61503
0.03133 3.87472 4.59707 0.61958 0.77255 0.48576 0.95510
407 2.71526 4.88946 3.00607 2.49260 4.21429 3.43602 3.58387
3.60665 2.02456 3.16056 3.99690 2.97560 3.84547 2.12524 2.29736
2.73442 2.49494 3.28147 5.33799 4.05935 409 k - - -
2.68618 4.42225 2.77519 2.73123 3.46354 2.40513 3.72494
3.29354 2.67741 2.69355 4.24690 2.90347 2.73739 3.18146 2.89801
2.37887 2.77519 2.98518 4.58477 3.61503
0.03133 3.87472 4.59707 0.61958 0.77255 0.48576 0.95510
408 2.51312 4.28696 3.47895 2.97262 3.61366 3.39667 3.91098
2.67962 2.79547 2.57206 3.54760 3.31808 3.89790 3.20565 2.79972
2.73502 2.25248 1.88076 5.08260 3.84671 410 v - - -
2.68618 4.42225 2.77519 2.73123 3.46354 2.40513 3.72494
3.29354 2.67741 2.69355 4.24690 2.90347 2.73739 3.18146 2.89801
2.37887 2.77519 2.98518 4.58477 3.61503
0.03133 3.87472 4.59707 0.61958 0.77255 0.48576 0.95510
409 2.77071 4.26879 4.13289 3.61445 3.49962 3.88421 4.42199
1.71126 3.50859 2.19175 3.36424 3.89242 4.30250 3.81336 3.77420
3.22993 2.48741 1.44475 5.23217 4.01990 411 v - - -
2.68618 4.42225 2.77519 2.73123 3.46354 2.40513 3.72494
3.29354 2.67741 2.69355 4.24690 2.90347 2.73739 3.18146 2.89801
2.37887 2.77519 2.98518 4.58477 3.61503
0.03133 3.87472 4.59707 0.61958 0.77255 0.48576 0.95510
410 3.15771 4.61926 4.19314 3.83291 3.13642 3.98506 4.48794
2.34049 3.60064 0.87242 3.15851 4.10818 4.41998 3.96843 3.77638
3.55606 3.45182 2.37843 5.01661 3.74048 412 l - - -
2.68618 4.42225 2.77519 2.73123 3.46354 2.40513 3.72494
3.29354 2.67741 2.69355 4.24690 2.90347 2.73739 3.18146 2.89801
2.37887 2.77519 2.98518 4.58477 3.61503
0.03133 3.87472 4.59707 0.61958 0.77255 0.48576 0.95510
411 2.59570 4.80094 2.69562 2.36747 4.24485 2.91506 3.66165
3.67895 2.11615 3.25481 4.07628 2.41983 3.76672 2.82100 2.84610
2.14297 2.88166 3.30153 5.45430 4.11646 413 k - - -
2.68618 4.42225 2.77519 2.73123 3.46354 2.40513 3.72494
3.29354 2.67741 2.69355 4.24690 2.90347 2.73739 3.18146 2.89801
2.37887 2.77519 2.98518 4.58477 3.61503
0.03133 3.87472 4.59707 0.61958 0.77255 0.48576 0.95510
412 2.31597 4.23644 3.20823 3.02400 4.07846 2.96628 4.13384
3.60134 3.09793 3.32136 4.24719 3.24849 3.70524 3.44704 3.38396
1.08767 2.79625 3.13595 5.45075 4.16198 414 s - - -

```
         2.68618  4.42225  2.77519  2.73123  3.46354  2.40513  3.72494
3.29354  2.67741  2.69355  4.24690  2.90347  2.73739  3.18146  2.89801
2.37887  2.77519  2.98518  4.58477  3.61503
         0.03133  3.87472  4.59707  0.61958  0.77255  0.48576  0.95510
    413  3.00784  4.84699  3.49870  3.00918  4.20214  3.49552  3.82565
3.74393  2.23793  3.25944  4.25948  3.36879  3.99792  3.04579  0.97495
3.09185  3.27359  3.46776  5.28422  4.12892    415 r - - -
         2.68618  4.42225  2.77519  2.73123  3.46354  2.40513  3.72494
3.29354  2.67741  2.69355  4.24690  2.90347  2.73739  3.18146  2.89801
2.37887  2.77519  2.98518  4.58477  3.61503
         0.23773  3.87472  1.65641  0.61958  0.77255  0.48576  0.95510
    414  2.65219  4.26112  3.83823  3.50789  3.41605  3.50322  4.34649
2.10447  3.37156  2.14909  3.42613  3.72537  4.08342  3.75139  3.60014
3.00431  3.02431  1.32331  5.16185  3.87506    416 v - - -
         2.68618  4.42225  2.77519  2.73123  3.46354  2.40513  3.72494
3.29354  2.67741  2.69355  4.24690  2.90347  2.73739  3.18146  2.89801
2.37887  2.77519  2.98518  4.58477  3.61503
         0.03837  3.67537  4.39771  0.61958  0.77255  0.40072  1.10818
    415  2.32630  4.26218  3.21111  2.88787  4.04571  3.04818  3.99393
3.34664  2.88524  3.08377  3.98987  3.18311  2.09500  3.24689  3.21583
2.48828  1.86301  2.96127  5.40963  4.16587    417 t - - -
         2.68618  4.42225  2.77519  2.73123  3.46354  2.40513  3.72494
3.29354  2.67741  2.69355  4.24690  2.90347  2.73739  3.18146  2.89801
2.37887  2.77519  2.98518  4.58477  3.61503
         0.03133  3.87472  4.59707  0.61958  0.77255  0.48576  0.95510
    416  2.23056  4.19284  3.09138  2.91147  4.27778  1.50524  4.10921
3.74988  3.08578  3.42856  4.26897  3.15479  3.64464  3.37819  3.41221
1.89552  2.71511  3.19775  5.60059  4.35458    418 g - - -
         2.68618  4.42225  2.77519  2.73123  3.46354  2.40513  3.72494
3.29354  2.67741  2.69355  4.24690  2.90347  2.73739  3.18146  2.89801
2.37887  2.77519  2.98518  4.58477  3.61503
         0.03133  3.87472  4.59707  0.61958  0.77255  0.48576  0.95510
    417  2.41916  4.23556  3.40498  2.94579  3.67379  2.60849  3.93146
2.34427  2.88895  2.68064  3.61769  3.27217  3.82485  3.22824  3.22240
2.62740  2.04886  2.54668  5.12569  3.88517    419 t - - -
         2.68618  4.42225  2.77519  2.73123  3.46354  2.40513  3.72494
3.29354  2.67741  2.69355  4.24690  2.90347  2.73739  3.18146  2.89801
2.37887  2.77519  2.98518  4.58477  3.61503
         0.03133  3.87472  4.59707  0.61958  0.77255  0.48576  0.95510
    418  3.03284  4.45083  4.33904  3.95485  3.37561  4.06909  4.68249
1.07512  3.78303  1.96109  3.31052  4.19987  4.50000  4.13104  3.97934
3.56453  3.33193  1.84577  5.26697  4.00552    420 i - - -
         2.68618  4.42225  2.77519  2.73123  3.46354  2.40513  3.72494
3.29354  2.67741  2.69355  4.24690  2.90347  2.73739  3.18146  2.89801
2.37887  2.77519  2.98518  4.58477  3.61503
         0.03133  3.87472  4.59707  0.61958  0.77255  0.48576  0.95510
    419  2.75420  4.51129  3.36160  3.20088  4.19604  3.19867  4.27823
3.74193  3.25835  3.36857  4.41844  3.50740  0.82346  3.64706  3.50371
2.92174  3.19592  3.38706  5.38207  4.31670    421 p - - -
         2.68618  4.42225  2.77519  2.73123  3.46354  2.40513  3.72494
3.29354  2.67741  2.69355  4.24690  2.90347  2.73739  3.18146  2.89801
2.37887  2.77519  2.98518  4.58477  3.61503
         0.03133  3.87472  4.59707  0.61958  0.77255  0.48576  0.95510
```

```
    420   2.29066  4.26619  3.09152  2.82123  4.15546  2.98297  3.99018
3.60389  2.90028  3.27334  4.12730  3.12364  2.12398  3.23519  3.24758
1.66406  2.73311  3.13100  5.48492  4.21511    422 s - - -
          2.68618  4.42225  2.77519  2.73123  3.46354  2.40513  3.72494
3.29354  2.67741  2.69355  4.24690  2.90347  2.73739  3.18146  2.89801
2.37887  2.77519  2.98518  4.58477  3.61503
          0.03133  3.87472  4.59707  0.61958  0.77255  0.48576  0.95510
    421   2.72515  4.72193  2.70930  2.59495  4.08579  3.18536  3.92257
3.79656  2.82640  3.44315  4.39831  1.15407  3.83729  3.19525  3.18120
2.79220  3.10703  3.41854  5.39143  4.03390    423 n - - -
          2.68618  4.42225  2.77519  2.73123  3.46354  2.40513  3.72494
3.29354  2.67741  2.69355  4.24690  2.90347  2.73739  3.18146  2.89801
2.37887  2.77519  2.98518  4.58477  3.61503
          0.03133  3.87472  4.59707  0.61958  0.77255  0.48576  0.95510
    422   2.65496  4.36489  3.50013  2.93971  3.15538  3.58970  3.73306
2.40246  2.71260  2.45152  3.43053  3.31663  3.97060  3.13282  2.55149
2.86580  2.88617  2.58946  4.67452  2.40182    424 y - - -
          2.68618  4.42225  2.77519  2.73123  3.46354  2.40513  3.72494
3.29354  2.67741  2.69355  4.24690  2.90347  2.73739  3.18146  2.89801
2.37887  2.77519  2.98518  4.58477  3.61503
          0.03133  3.87472  4.59707  0.61958  0.77255  0.48576  0.95510
    423   3.15771  4.61926  4.19314  3.83291  3.13642  3.98506  4.48794
2.34049  3.60064  0.87242  3.15851  4.10818  4.41998  3.96843  3.77638
3.55606  3.45182  2.37843  5.01661  3.74048    425 l - - -
          2.68618  4.42225  2.77519  2.73123  3.46354  2.40513  3.72494
3.29354  2.67741  2.69355  4.24690  2.90347  2.73739  3.18146  2.89801
2.37887  2.77519  2.98518  4.58477  3.61503
          0.03133  3.87472  4.59707  0.61958  0.77255  0.48576  0.95510
    424   2.05867  4.86322  2.70317  2.07966  4.19891  3.32287  3.64304
3.57444  2.08093  3.18678  4.03035  2.88503  3.80552  2.80525  2.76449
2.68777  2.92667  3.24285  5.41103  4.08598    426 a - - -
          2.68618  4.42225  2.77519  2.73123  3.46354  2.40513  3.72494
3.29354  2.67741  2.69355  4.24690  2.90347  2.73739  3.18146  2.89801
2.37887  2.77519  2.98518  4.58477  3.61503
          0.03133  3.87472  4.59707  0.61958  0.77255  0.48576  0.95510
    425   3.25019  4.73827  3.84149  3.57122  2.29338  3.83270  3.61936
3.25908  3.42867  2.71244  3.93385  3.72612  4.30645  3.73417  3.61195
3.38669  3.53686  3.12972  3.93726  0.96205    427 y - - -
          2.68618  4.42225  2.77519  2.73123  3.46354  2.40513  3.72494
3.29354  2.67741  2.69355  4.24690  2.90347  2.73739  3.18146  2.89801
2.37887  2.77519  2.98518  4.58477  3.61503
          0.03133  3.87472  4.59707  0.61958  0.77255  0.48576  0.95510
    426   2.99187  5.12073  0.91483  2.33607  4.49801  3.21224  3.93281
4.08666  3.01732  3.69455  4.67262  2.89258  3.86518  3.17190  3.53391
2.95391  3.33046  3.72558  5.66619  4.37043    428 d - - -
          2.68618  4.42225  2.77519  2.73123  3.46354  2.40513  3.72494
3.29354  2.67741  2.69355  4.24690  2.90347  2.73739  3.18146  2.89801
2.37887  2.77519  2.98518  4.58477  3.61503
          0.03133  3.87472  4.59707  0.61958  0.77255  0.48576  0.95510
    427   2.67560  4.82680  1.93444  2.34013  4.44181  1.57922  3.85150
3.94026  2.84916  3.54453  4.41089  2.85624  3.77235  3.05738  3.34203
2.69681  3.03767  3.51012  5.66169  4.32858    429 g - - -
          2.68618  4.42225  2.77519  2.73123  3.46354  2.40513  3.72494
3.29354  2.67741  2.69355  4.24690  2.90347  2.73739  3.18146  2.89801
2.37887  2.77519  2.98518  4.58477  3.61503
```

0.03133 3.87472 4.59707 0.61958 0.77255 0.48576 0.95510
428 3.25019 4.73827 3.84149 3.57122 2.29338 3.83270 3.61936
3.25908 3.42867 2.71244 3.93385 3.72612 4.30645 3.73417 3.61195
3.38669 3.53686 3.12972 3.93726 0.96205 430 y - - -
2.68618 4.42225 2.77519 2.73123 3.46354 2.40513 3.72494
3.29354 2.67741 2.69355 4.24690 2.90347 2.73739 3.18146 2.89801
2.37887 2.77519 2.98518 4.58477 3.61503
0.03133 3.87472 4.59707 0.61958 0.77255 0.48576 0.95510
429 3.25019 4.73827 3.84149 3.57122 2.29338 3.83270 3.61936
3.25908 3.42867 2.71244 3.93385 3.72612 4.30645 3.73417 3.61195
3.38669 3.53686 3.12972 3.93726 0.96205 431 y - - -
2.68618 4.42225 2.77519 2.73123 3.46354 2.40513 3.72494
3.29354 2.67741 2.69355 4.24690 2.90347 2.73739 3.18146 2.89801
2.37887 2.77519 2.98518 4.58477 3.61503
0.03133 3.87472 4.59707 0.61958 0.77255 0.48576 0.95510
430 3.05640 4.43536 4.49930 4.00215 2.71040 4.23955 4.53861
1.27855 3.87965 1.75697 3.10917 4.23685 4.54968 4.09843 4.06717
3.60768 3.31009 1.90659 4.98160 3.65132 432 i - - -
2.68618 4.42225 2.77519 2.73123 3.46354 2.40513 3.72494
3.29354 2.67741 2.69355 4.24690 2.90347 2.73739 3.18146 2.89801
2.37887 2.77519 2.98518 4.58477 3.61503
0.03133 3.87472 4.59707 0.61958 0.77255 0.48576 0.95510
431 2.62060 3.40842 3.47561 2.90698 3.89644 3.37061 3.79158
3.24986 2.38651 2.92705 3.86221 3.25628 3.87793 3.02054 1.49170
2.76201 2.92502 2.96779 5.18691 3.95325 433 r - - -
2.68618 4.42225 2.77519 2.73123 3.46354 2.40513 3.72494
3.29354 2.67741 2.69355 4.24690 2.90347 2.73739 3.18146 2.89801
2.37887 2.77519 2.98518 4.58477 3.61503
0.03133 3.87472 4.59707 0.61958 0.77255 0.48576 0.95510
432 1.01052 4.19303 3.46476 3.25606 4.05451 3.01753 4.27786
3.18035 3.27238 3.06187 4.09337 3.40514 3.75695 3.61331 3.52281
2.54327 2.82077 2.83436 5.48144 4.28192 434 a - - -
2.68618 4.42225 2.77519 2.73123 3.46354 2.40513 3.72494
3.29354 2.67741 2.69355 4.24690 2.90347 2.73739 3.18146 2.89801
2.37887 2.77519 2.98518 4.58477 3.61503
0.03133 3.87472 4.59707 0.61958 0.77255 0.48576 0.95510
433 2.99963 4.36519 4.56916 4.07407 3.46301 4.26218 4.81725
1.39403 3.95119 1.98468 3.29021 4.31808 4.60623 4.23397 4.16938
3.64342 3.27184 1.28179 5.39949 4.18742 435 v - - -
2.68618 4.42225 2.77519 2.73123 3.46354 2.40513 3.72494
3.29354 2.67741 2.69355 4.24690 2.90347 2.73739 3.18146 2.89801
2.37887 2.77519 2.98518 4.58477 3.61503
0.03133 3.87472 4.59707 0.61958 0.77255 0.48576 0.95510
434 2.39817 4.29987 3.26270 2.92779 3.75942 3.13524 3.96072
3.17388 2.86687 2.56868 3.89309 3.22679 3.77881 3.25887 3.17859
1.44754 2.80269 2.85547 5.21464 3.87270 436 s - - -
2.68618 4.42225 2.77519 2.73123 3.46354 2.40513 3.72494
3.29354 2.67741 2.69355 4.24690 2.90347 2.73739 3.18146 2.89801
2.37887 2.77519 2.98518 4.58477 3.61503
0.03133 3.87472 4.59707 0.61958 0.77255 0.48576 0.95510
435 1.01052 4.19303 3.46476 3.25606 4.05451 3.01753 4.27786
3.18035 3.27238 3.06187 4.09337 3.40514 3.75695 3.61331 3.52281
2.54327 2.82077 2.83436 5.48144 4.28192 437 a - - -

```
          2.68618  4.42225  2.77519  2.73123  3.46354  2.40513  3.72494
 3.29354  2.67741  2.69355  4.24690  2.90347  2.73739  3.18146  2.89801
 2.37887  2.77519  2.98518  4.58477  3.61503
          0.03133  3.87472  4.59707  0.61958  0.77255  0.48576  0.95510
    436   2.58861  0.93589  4.10823  3.84832  3.83906  3.21118  4.49490
 2.98326  3.67524  2.89868  4.02000  3.83470  3.91832  4.02124  3.79925
 2.86028  3.05555  2.72771  5.25480  4.12675    438 c - - -
          2.68618  4.42225  2.77519  2.73123  3.46354  2.40513  3.72494
 3.29354  2.67741  2.69355  4.24690  2.90347  2.73739  3.18146  2.89801
 2.37887  2.77519  2.98518  4.58477  3.61503
          0.03133  3.87472  4.59707  0.61958  0.77255  0.48576  0.95510
    437   2.75420  4.51129  3.36160  3.20088  4.19604  3.19867  4.27823
 3.74193  3.25835  3.36857  4.41844  3.50740  0.82346  3.64706  3.50371
 2.92174  3.19592  3.38706  5.38207  4.31670    439 p - - -
          2.68618  4.42225  2.77519  2.73123  3.46354  2.40513  3.72494
 3.29354  2.67741  2.69355  4.24690  2.90347  2.73739  3.18146  2.89801
 2.37887  2.77519  2.98518  4.58477  3.61503
          0.03133  3.87472  4.59707  0.61958  0.77255  0.48576  0.95510
    438   2.97042  5.03658  3.36975  2.75160  4.45095  3.58742  3.58568
 3.81539  1.64665  3.29651  4.18095  3.15265  3.97676  2.73241  1.42580
 2.98437  3.15278  3.50961  5.36671  4.18791    440 r - - -
          2.68618  4.42225  2.77519  2.73123  3.46354  2.40513  3.72494
 3.29354  2.67741  2.69355  4.24690  2.90347  2.73739  3.18146  2.89801
 2.37887  2.77519  2.98518  4.58477  3.61503
          0.03133  3.87472  4.59707  0.61958  0.77255  0.48576  0.95510
    439   2.46337  4.49881  2.72263  2.57595  4.28798  1.58487  3.92635
 3.81000  2.85056  3.44330  4.29964  2.25083  3.72525  3.15713  3.23968
 2.56043  2.88130  3.33584  5.56671  4.25816    441 g - - -
          2.68618  4.42225  2.77519  2.73123  3.46354  2.40513  3.72494
 3.29354  2.67741  2.69355  4.24690  2.90347  2.73739  3.18146  2.89801
 2.37887  2.77519  2.98518  4.58477  3.61503
          0.03133  3.87472  4.59707  0.61958  0.77255  0.48576  0.95510
    440   2.80983  4.35172  4.12850  3.79161  3.55163  3.72629  4.59291
 2.04988  3.65943  2.23820  3.51634  3.98111  4.28661  4.01903  3.87173
 3.22990  3.17128  1.02205  5.33648  4.06827    442 v - - -
          2.68618  4.42225  2.77519  2.73123  3.46354  2.40513  3.72494
 3.29354  2.67741  2.69355  4.24690  2.90347  2.73739  3.18146  2.89801
 2.37887  2.77519  2.98518  4.58477  3.61503
          0.03133  3.87472  4.59707  0.61958  0.77255  0.48576  0.95510
    441   2.27916  4.25210  3.10663  2.83914  4.16248  2.97193  4.00340
 3.61273  2.91850  3.28339  4.13625  3.13126  2.56435  3.25098  3.26265
 1.44720  2.72672  3.13229  5.49314  4.22570    443 s - - -
          2.68618  4.42225  2.77519  2.73123  3.46354  2.40513  3.72494
 3.29354  2.67741  2.69355  4.24690  2.90347  2.73739  3.18146  2.89801
 2.37887  2.77519  2.98518  4.58477  3.61503
          0.03133  3.87472  4.59707  0.61958  0.77255  0.48576  0.95510
    442   3.15771  4.61926  4.19314  3.83291  3.13642  3.98506  4.48794
 2.34049  3.60064  0.87242  3.15851  4.10818  4.41998  3.96843  3.77638
 3.55606  3.45182  2.37843  5.01661  3.74048    444 l - - -
          2.68618  4.42225  2.77519  2.73123  3.46354  2.40513  3.72494
 3.29354  2.67741  2.69355  4.24690  2.90347  2.73739  3.18146  2.89801
 2.37887  2.77519  2.98518  4.58477  3.61503
          0.03133  3.87472  4.59707  0.61958  0.77255  0.48576  0.95510
```

```
    443   1.31340  4.10265  3.35185  3.09026  4.14267  2.90028  4.15332
3.44903  3.13365  3.23338  4.10708  3.24796  3.64555  3.44200  3.42858
1.90673  2.66305  2.97701  5.52371  4.29673    445 a - - -
          2.68618  4.42225  2.77519  2.73123  3.46354  2.40513  3.72494
3.29354  2.67741  2.69355  4.24690  2.90347  2.73739  3.18146  2.89801
2.37887  2.77519  2.98518  4.58477  3.61503
          0.03133  3.87472  4.59707  0.61958  0.77255  0.48576  0.95510
    444   3.25019  4.73827  3.84149  3.57122  2.29338  3.83270  3.61936
3.25908  3.42867  2.71244  3.93385  3.72612  4.30645  3.73417  3.61195
3.38669  3.53686  3.12972  3.93726  0.96205    446 y - - -
          2.68618  4.42225  2.77519  2.73123  3.46354  2.40513  3.72494
3.29354  2.67741  2.69355  4.24690  2.90347  2.73739  3.18146  2.89801
2.37887  2.77519  2.98518  4.58477  3.61503
          0.03133  3.87472  4.59707  0.61958  0.77255  0.48576  0.95510
    445   2.72515  4.72193  2.70930  2.59495  4.08579  3.18536  3.92257
3.79656  2.82640  3.44315  4.39831  1.15407  3.83729  3.19525  3.18120
2.79220  3.10703  3.41854  5.39143  4.03390    447 n - - -
          2.68618  4.42225  2.77519  2.73123  3.46354  2.40513  3.72494
3.29354  2.67741  2.69355  4.24690  2.90347  2.73739  3.18146  2.89801
2.37887  2.77519  2.98518  4.58477  3.61503
          0.03133  3.87472  4.59707  0.61958  0.77255  0.48576  0.95510
    446   2.99708  4.81426  3.12768  2.88777  3.28613  3.44925  1.13776
3.70036  2.73075  3.19407  4.23713  3.29704  4.00186  3.25401  2.99637
3.06772  3.30049  3.43543  4.75116  3.23690    448 h - - -
          2.68618  4.42225  2.77519  2.73123  3.46354  2.40513  3.72494
3.29354  2.67741  2.69355  4.24690  2.90347  2.73739  3.18146  2.89801
2.37887  2.77519  2.98518  4.58477  3.61503
          0.03133  3.87472  4.59707  0.61958  0.77255  0.48576  0.95510
    447   2.83788  5.26914  1.39590  2.10233  4.58688  3.15938  3.73578
4.14277  2.75097  3.68698  4.55113  2.16260  3.78764  2.91628  3.31799
2.75769  3.14586  3.72618  5.81451  4.33435    449 d - - -
          2.68618  4.42225  2.77519  2.73123  3.46354  2.40513  3.72494
3.29354  2.67741  2.69355  4.24690  2.90347  2.73739  3.18146  2.89801
2.37887  2.77519  2.98518  4.58477  3.61503
          0.03133  3.87472  4.59707  0.61958  0.77255  0.48576  0.95510
    448   3.03637  4.46016  4.23134  3.73019  2.00409  4.03806  3.76450
2.61115  3.60111  1.54136  3.24948  3.86171  4.35404  3.76426  3.77361
3.35046  3.26259  2.57407  4.08280  2.06984    450 l - - -
          2.68618  4.42225  2.77519  2.73123  3.46354  2.40513  3.72494
3.29354  2.67741  2.69355  4.24690  2.90347  2.73739  3.18146  2.89801
2.37887  2.77519  2.98518  4.58477  3.61503
          0.03133  3.87472  4.59707  0.61958  0.77255  0.48576  0.95510
    449   2.65306  4.33968  3.69152  3.23941  3.44265  3.55356  4.12504
2.43271  3.07640  1.66114  3.39154  3.54931  4.05793  3.46371  3.35294
2.93277  2.08231  2.29625  5.10325  3.85346    451 l - - -
          2.68618  4.42225  2.77519  2.73123  3.46354  2.40513  3.72494
3.29354  2.67741  2.69355  4.24690  2.90347  2.73739  3.18146  2.89801
2.37887  2.77519  2.98518  4.58477  3.61503
          0.03133  3.87472  4.59707  0.61958  0.77255  0.48576  0.95510
    450   2.41377  4.25888  3.42992  3.18193  3.97070  3.09811  4.19910
3.09810  3.12495  2.96152  4.01182  3.39101  3.80201  3.51964  3.38358
2.61505  1.16810  2.79284  5.40831  4.18731    452 t - - -
          2.68618  4.42225  2.77519  2.73123  3.46354  2.40513  3.72494
3.29354  2.67741  2.69355  4.24690  2.90347  2.73739  3.18146  2.89801
2.37887  2.77519  2.98518  4.58477  3.61503
```

```
          0.03133  3.87472  4.59707  0.61958  0.77255  0.48576  0.95510
    451   3.15771  4.61926  4.19314  3.83291  3.13642  3.98506  4.48794
2.34049  3.60064  0.87242  3.15851  4.10818  4.41998  3.96843  3.77638
3.55606  3.45182  2.37843  5.01661  3.74048    453 l - - -
          2.68618  4.42225  2.77519  2.73123  3.46354  2.40513  3.72494
3.29354  2.67741  2.69355  4.24690  2.90347  2.73739  3.18146  2.89801
2.37887  2.77519  2.98518  4.58477  3.61503
          0.03133  3.87472  4.59707  0.61958  0.77255  0.48576  0.95510
    452   2.41377  4.25888  3.42992  3.18193  3.97070  3.09811  4.19910
3.09810  3.12495  2.96152  4.01182  3.39101  3.80201  3.51964  3.38358
2.61505  1.16810  2.79284  5.40831  4.18731    454 t - - -
          2.68618  4.42225  2.77519  2.73123  3.46354  2.40513  3.72494
3.29354  2.67741  2.69355  4.24690  2.90347  2.73739  3.18146  2.89801
2.37887  2.77519  2.98518  4.58477  3.61503
          0.03133  3.87472  4.59707  0.61958  0.77255  0.48576  0.95510
    453   3.25019  4.73827  3.84149  3.57122  2.29338  3.83270  3.61936
3.25908  3.42867  2.71244  3.93385  3.72612  4.30645  3.73417  3.61195
3.38669  3.53686  3.12972  3.93726  0.96205    455 y - - -
          2.68618  4.42225  2.77519  2.73123  3.46354  2.40513  3.72494
3.29354  2.67741  2.69355  4.24690  2.90347  2.73739  3.18146  2.89801
2.37887  2.77519  2.98518  4.58477  3.61503
          0.03133  3.87472  4.59707  0.61958  0.77255  0.48576  0.95510
    454   2.72515  4.72193  2.70930  2.59495  4.08579  3.18536  3.92257
3.79656  2.82640  3.44315  4.39831  1.15407  3.83729  3.19525  3.18120
2.79220  3.10703  3.41854  5.39143  4.03390    456 n - - -
          2.68618  4.42225  2.77519  2.73123  3.46354  2.40513  3.72494
3.29354  2.67741  2.69355  4.24690  2.90347  2.73739  3.18146  2.89801
2.37887  2.77519  2.98518  4.58477  3.61503
          0.03133  3.87472  4.59707  0.61958  0.77255  0.48576  0.95510
    455   2.97042  5.03658  3.36975  2.75160  4.45095  3.58742  3.58568
3.81539  1.64665  3.29651  4.18095  3.15265  3.97676  2.73241  1.42580
2.98437  3.15278  3.50961  5.36671  4.18791    457 r - - -
          2.68618  4.42225  2.77519  2.73123  3.46354  2.40513  3.72494
3.29354  2.67741  2.69355  4.24690  2.90347  2.73739  3.18146  2.89801
2.37887  2.77519  2.98518  4.58477  3.61503
          0.03133  3.87472  4.59707  0.61958  0.77255  0.48576  0.95510
    456   3.13260  4.49754  4.57868  4.06001  3.16843  4.35367  4.71105
1.51681  3.88418  1.27239  3.01918  4.33673  4.62015  4.13416  4.07017
3.72128  3.37745  1.92247  5.17278  3.98634    458 l - - -
          2.68618  4.42225  2.77519  2.73123  3.46354  2.40513  3.72494
3.29354  2.67741  2.69355  4.24690  2.90347  2.73739  3.18146  2.89801
2.37887  2.77519  2.98518  4.58477  3.61503
          0.03133  3.87472  4.59707  0.61958  0.77255  0.48576  0.95510
    457   2.95406  5.06580  2.48830  1.02217  4.41227  3.27540  3.86227
3.86661  2.74307  3.48333  4.45717  2.93393  3.88006  3.07782  3.15222
2.93617  3.25828  3.55019  5.58341  4.30555    459 e - - -
          2.68618  4.42225  2.77519  2.73123  3.46354  2.40513  3.72494
3.29354  2.67741  2.69355  4.24690  2.90347  2.73739  3.18146  2.89801
2.37887  2.77519  2.98518  4.58477  3.61503
          0.03133  3.87472  4.59707  0.61958  0.77255  0.48576  0.95510
    458   3.25019  4.73827  3.84149  3.57122  2.29338  3.83270  3.61936
3.25908  3.42867  2.71244  3.93385  3.72612  4.30645  3.73417  3.61195
3.38669  3.53686  3.12972  3.93726  0.96205    460 y - - -
```

```
          2.68618  4.42225  2.77519  2.73123  3.46354  2.40513  3.72494
3.29354  2.67741  2.69355  4.24690  2.90347  2.73739  3.18146  2.89801
2.37887  2.77519  2.98518  4.58477  3.61503
          0.03133  3.87472  4.59707  0.61958  0.77255  0.48576  0.95510
    459   2.99187  5.12073  0.91483  2.33607  4.49801  3.21224  3.93281
4.08666  3.01732  3.69455  4.67262  2.89258  3.86518  3.17190  3.53391
2.95391  3.33046  3.72558  5.66619  4.37043    461 d - - -
          2.68618  4.42225  2.77519  2.73123  3.46354  2.40513  3.72494
3.29354  2.67741  2.69355  4.24690  2.90347  2.73739  3.18146  2.89801
2.37887  2.77519  2.98518  4.58477  3.61503
          0.03133  3.87472  4.59707  0.61958  0.77255  0.48576  0.95510
    460   1.50879  4.11123  3.33050  3.05929  4.15834  2.89943  4.13453
3.49003  3.10781  3.25178  4.11504  3.23094  3.64092  3.41443  3.41277
1.63872  2.66000  3.00580  5.52886  4.29979    462 a - - -
          2.68618  4.42225  2.77519  2.73123  3.46354  2.40513  3.72494
3.29354  2.67741  2.69355  4.24690  2.90347  2.73739  3.18146  2.89801
2.37887  2.77519  2.98518  4.58477  3.61503
          0.03133  3.87472  4.59707  0.61958  0.77255  0.48576  0.95510
    461   2.75420  4.51129  3.36160  3.20088  4.19604  3.19867  4.27823
3.74193  3.25835  3.36857  4.41844  3.50740  0.82346  3.64706  3.50371
2.92174  3.19592  3.38706  5.38207  4.31670    463 p - - -
          2.68618  4.42225  2.77519  2.73123  3.46354  2.40513  3.72494
3.29354  2.67741  2.69355  4.24690  2.90347  2.73739  3.18146  2.89801
2.37887  2.77519  2.98518  4.58477  3.61503
          0.03133  3.87472  4.59707  0.61958  0.77255  0.48576  0.95510
    462   2.25503  4.16691  3.31408  3.03235  4.04783  2.98011  4.09051
3.30800  3.01466  3.12113  4.02882  3.24281  3.69420  3.37467  3.30806
2.18531  1.42240  2.90322  5.44346  4.19697    464 t - - -
          2.68618  4.42225  2.77519  2.73123  3.46354  2.40513  3.72494
3.29354  2.67741  2.69355  4.24690  2.90347  2.73739  3.18146  2.89801
2.37887  2.77519  2.98518  4.58477  3.61503
          0.03133  3.87472  4.59707  0.61958  0.77255  0.48576  0.95510
    463   2.41377  4.25888  3.42992  3.18193  3.97070  3.09811  4.19910
3.09810  3.12495  2.96152  4.01182  3.39101  3.80201  3.51964  3.38358
2.61505  1.16810  2.79284  5.40831  4.18731    465 t - - -
          2.68618  4.42225  2.77519  2.73123  3.46354  2.40513  3.72494
3.29354  2.67741  2.69355  4.24690  2.90347  2.73739  3.18146  2.89801
2.37887  2.77519  2.98518  4.58477  3.61503
          0.03133  3.87472  4.59707  0.61958  0.77255  0.48576  0.95510
    464   2.80826  5.13391  2.47113  1.74377  4.41976  3.29985  3.65014
3.87894  2.38270  3.40687  4.26697  2.80430  3.82402  1.98888  2.77915
2.76478  3.06050  3.52472  5.57169  4.20330    466 e - - -
          2.68618  4.42225  2.77519  2.73123  3.46354  2.40513  3.72494
3.29354  2.67741  2.69355  4.24690  2.90347  2.73739  3.18146  2.89801
2.37887  2.77519  2.98518  4.58477  3.61503
          0.03133  3.87472  4.59707  0.61958  0.77255  0.48576  0.95510
    465   2.72515  4.72193  2.70930  2.59495  4.08579  3.18536  3.92257
3.79656  2.82640  3.44315  4.39831  1.15407  3.83729  3.19525  3.18120
2.79220  3.10703  3.41854  5.39143  4.03390    467 n - - -
          2.68618  4.42225  2.77519  2.73123  3.46354  2.40513  3.72494
3.29354  2.67741  2.69355  4.24690  2.90347  2.73739  3.18146  2.89801
2.37887  2.77519  2.98518  4.58477  3.61503
          0.03133  3.87472  4.59707  0.61958  0.77255  0.48576  0.95510
```

```
    466   3.03284  4.45083  4.33904  3.95485  3.37561  4.06909  4.68249
1.07512  3.78303  1.96109  3.31052  4.19987  4.50000  4.13104  3.97934
3.56453  3.33193  1.84577  5.26697  4.00552    468 i - - -
          2.68618  4.42225  2.77519  2.73123  3.46354  2.40513  3.72494
3.29354  2.67741  2.69355  4.24690  2.90347  2.73739  3.18146  2.89801
2.37887  2.77519  2.98518  4.58477  3.61503
          0.03133  3.87472  4.59707  0.61958  0.77255  0.48576  0.95510
    467   3.03284  4.45083  4.33904  3.95485  3.37561  4.06909  4.68249
1.07512  3.78303  1.96109  3.31052  4.19987  4.50000  4.13104  3.97934
3.56453  3.33193  1.84577  5.26697  4.00552    469 i - - -
          2.68618  4.42225  2.77519  2.73123  3.46354  2.40513  3.72494
3.29354  2.67741  2.69355  4.24690  2.90347  2.73739  3.18146  2.89801
2.37887  2.77519  2.98518  4.58477  3.61503
          0.03133  3.87472  4.59707  0.61958  0.77255  0.48576  0.95510
    468   2.48070  4.20298  3.67213  3.32456  3.68371  2.80624  4.22657
2.48817  3.25601  2.61049  3.67224  3.53247  3.93603  3.59813  3.51535
2.75704  2.87384  1.31016  5.24085  3.99730    470 v - - -
          2.68618  4.42225  2.77519  2.73123  3.46354  2.40513  3.72494
3.29354  2.67741  2.69355  4.24690  2.90347  2.73739  3.18146  2.89801
2.37887  2.77519  2.98518  4.58477  3.61503
          0.03133  3.87472  4.59707  0.61958  0.77255  0.48576  0.95510
    469   2.66752  4.44283  3.34177  3.24141  4.38386  0.72564  4.36163
3.98394  3.42300  3.63781  4.61350  3.50605  3.83404  3.74696  3.65888
2.83948  3.14893  3.51182  5.47850  4.48580    471 g - - -
          2.68618  4.42225  2.77519  2.73123  3.46354  2.40513  3.72494
3.29354  2.67741  2.69355  4.24690  2.90347  2.73739  3.18146  2.89801
2.37887  2.77519  2.98518  4.58477  3.61503
          0.03133  3.87472  4.59707  0.61958  0.77255  0.48576  0.95510
    470   3.26109  4.66217  4.24182  3.95666  1.00804  3.95049  3.87804
2.78442  3.86362  2.15392  3.51419  4.02166  4.40896  4.03095  3.98279
3.53096  3.56220  2.77530  4.12630  2.49298    472 f - - -
          2.68618  4.42225  2.77519  2.73123  3.46354  2.40513  3.72494
3.29354  2.67741  2.69355  4.24690  2.90347  2.73739  3.18146  2.89801
2.37887  2.77519  2.98518  4.58477  3.61503
          0.03133  3.87472  4.59707  0.61958  0.77255  0.48576  0.95510
    471   1.54708  4.11323  3.32564  3.05485  4.16099  2.89955  4.13258
3.49519  3.10452  3.25496  4.11764  3.22863  3.64079  3.41146  3.41044
1.59771  2.66061  3.00969  5.53041  4.30053    473 a - - -
          2.68618  4.42225  2.77519  2.73123  3.46354  2.40513  3.72494
3.29354  2.67741  2.69355  4.24690  2.90347  2.73739  3.18146  2.89801
2.37887  2.77519  2.98518  4.58477  3.61503
          0.03133  3.87472  4.59707  0.61958  0.77255  0.48576  0.95510
    472   2.75420  4.51129  3.36160  3.20088  4.19604  3.19867  4.27823
3.74193  3.25835  3.36857  4.41844  3.50740  0.82346  3.64706  3.50371
2.92174  3.19592  3.38706  5.38207  4.31670    474 p - - -
          2.68618  4.42225  2.77519  2.73123  3.46354  2.40513  3.72494
3.29354  2.67741  2.69355  4.24690  2.90347  2.73739  3.18146  2.89801
2.37887  2.77519  2.98518  4.58477  3.61503
          0.03133  3.87472  4.59707  0.61958  0.77255  0.48576  0.95510
    473   2.83788  5.26914  1.39590  2.10233  4.58688  3.15938  3.73578
4.14277  2.75097  3.68698  4.55113  2.16260  3.78764  2.91628  3.31799
2.75769  3.14586  3.72618  5.81451  4.33435    475 d - - -
          2.68618  4.42225  2.77519  2.73123  3.46354  2.40513  3.72494
3.29354  2.67741  2.69355  4.24690  2.90347  2.73739  3.18146  2.89801
2.37887  2.77519  2.98518  4.58477  3.61503
```

0.03133 3.87472 4.59707 0.61958 0.77255 0.48576 0.95510
474 2.72515 4.72193 2.70930 2.59495 4.08579 3.18536 3.92257
3.79656 2.82640 3.44315 4.39831 1.15407 3.83729 3.19525 3.18120
2.79220 3.10703 3.41854 5.39143 4.03390 476 n - - -
2.68618 4.42225 2.77519 2.73123 3.46354 2.40513 3.72494
3.29354 2.67741 2.69355 4.24690 2.90347 2.73739 3.18146 2.89801
2.37887 2.77519 2.98518 4.58477 3.61503
0.03133 3.87472 4.59707 0.61958 0.77255 0.48576 0.95510
475 2.41377 4.25888 3.42992 3.18193 3.97070 3.09811 4.19910
3.09810 3.12495 2.96152 4.01182 3.39101 3.80201 3.51964 3.38358
2.61505 1.16810 2.79284 5.40831 4.18731 477 t - - -
2.68618 4.42225 2.77519 2.73123 3.46354 2.40513 3.72494
3.29354 2.67741 2.69355 4.24690 2.90347 2.73739 3.18146 2.89801
2.37887 2.77519 2.98518 4.58477 3.61503
0.03133 3.87472 4.59707 0.61958 0.77255 0.48576 0.95510
476 2.97404 4.93809 3.15019 2.77112 4.32503 3.46335 3.76023
3.75442 1.03520 3.31897 4.28021 3.19552 3.96346 2.95151 2.43176
3.01582 3.22709 3.46205 5.37869 4.19175 478 k - - -
2.68618 4.42225 2.77519 2.73123 3.46354 2.40513 3.72494
3.29354 2.67741 2.69355 4.24690 2.90347 2.73739 3.18146 2.89801
2.37887 2.77519 2.98518 4.58477 3.61503
0.03133 3.87472 4.59707 0.61958 0.77255 0.48576 0.95510
477 2.63838 4.87811 1.76633 2.25760 4.38796 3.13839 3.75925
3.87285 2.71672 3.47437 4.32142 2.78584 3.75414 2.94255 3.22446
1.85129 2.97592 3.45508 5.64984 4.24224 479 d - - -
2.68618 4.42225 2.77519 2.73123 3.46354 2.40513 3.72494
3.29354 2.67741 2.69355 4.24690 2.90347 2.73739 3.18146 2.89801
2.37887 2.77519 2.98518 4.58477 3.61503
0.03133 3.87472 4.59707 0.61958 0.77255 0.48576 0.95510
478 3.26109 4.66217 4.24182 3.95666 1.00804 3.95049 3.87804
2.78442 3.86362 2.15392 3.51419 4.02166 4.40896 4.03095 3.98279
3.53096 3.56220 2.77530 4.12630 2.49298 480 f - - -
2.68618 4.42225 2.77519 2.73123 3.46354 2.40513 3.72494
3.29354 2.67741 2.69355 4.24690 2.90347 2.73739 3.18146 2.89801
2.37887 2.77519 2.98518 4.58477 3.61503
0.03133 3.87472 4.59707 0.61958 0.77255 0.48576 0.95510
479 3.33782 4.72325 4.20784 3.83745 1.81960 4.11559 3.45914
3.12156 3.70624 2.52238 3.75872 3.83257 4.46738 3.83355 3.85510
3.48197 3.57610 3.02835 3.67135 1.11302 481 y - - -
2.68618 4.42225 2.77519 2.73123 3.46354 2.40513 3.72494
3.29354 2.67741 2.69355 4.24690 2.90347 2.73739 3.18146 2.89801
2.37887 2.77519 2.98518 4.58477 3.61503
0.03133 3.87472 4.59707 0.61958 0.77255 0.48576 0.95510
480 2.07460 4.43246 3.11667 2.68049 4.04871 3.17290 3.78577
3.43857 2.54282 3.08897 3.93615 3.07051 3.74413 2.98719 2.37698
1.91490 2.77475 3.06161 5.33468 4.06517 482 s - - -
2.68618 4.42225 2.77519 2.73123 3.46354 2.40513 3.72494
3.29354 2.67741 2.69355 4.24690 2.90347 2.73739 3.18146 2.89801
2.37887 2.77519 2.98518 4.58477 3.61503
0.03133 3.87472 4.59707 0.61958 0.77255 0.48576 0.95510
481 2.75826 4.91282 3.08957 2.55811 4.30145 3.45208 3.58861
3.69204 1.67705 3.22475 4.06586 3.01766 3.86998 2.73423 2.05335
2.35465 2.98050 3.35745 5.36391 4.10896 483 k - - -

```
         2.68618  4.42225  2.77519  2.73123  3.46354  2.40513  3.72494
3.29354  2.67741  2.69355  4.24690  2.90347  2.73739  3.18146  2.89801
2.37887  2.77519  2.98518  4.58477  3.61503
         0.03133  3.87472  4.59707  0.61958  0.77255  0.48576  0.95510
    482  2.77719  4.99382  2.70185  2.40535  4.35358  3.34122  3.64440
3.81080  1.73740  3.35667  4.20969  2.02812  3.84361  2.80456  2.58136
2.77082  3.03266  3.45508  5.47468  4.15327    484 k - - -
         2.68618  4.42225  2.77519  2.73123  3.46354  2.40513  3.72494
3.29354  2.67741  2.69355  4.24690  2.90347  2.73739  3.18146  2.89801
2.37887  2.77519  2.98518  4.58477  3.61503
         0.03133  3.87472  4.59707  0.61958  0.77255  0.48576  0.95510
    483  2.31597  4.23644  3.20823  3.02400  4.07846  2.96628  4.13384
3.60134  3.09793  3.32136  4.24719  3.24849  3.70524  3.44704  3.38396
1.08767  2.79625  3.13595  5.45075  4.16198    485 s - - -
         2.68618  4.42225  2.77519  2.73123  3.46354  2.40513  3.72494
3.29354  2.67741  2.69355  4.24690  2.90347  2.73739  3.18146  2.89801
2.37887  2.77519  2.98518  4.58477  3.61503
         0.03133  3.87472  4.59707  0.61958  0.77255  0.48576  0.95510
    484  2.99708  4.81426  3.12768  2.88777  3.28613  3.44925  1.13776
3.70036  2.73075  3.19407  4.23713  3.29704  4.00186  3.25401  2.99637
3.06772  3.30049  3.43543  4.75116  3.23690    486 h - - -
         2.68618  4.42225  2.77519  2.73123  3.46354  2.40513  3.72494
3.29354  2.67741  2.69355  4.24690  2.90347  2.73739  3.18146  2.89801
2.37887  2.77519  2.98518  4.58477  3.61503
         0.03133  3.87472  4.59707  0.61958  0.77255  0.48576  0.95510
    485  3.25019  4.73827  3.84149  3.57122  2.29338  3.83270  3.61936
3.25908  3.42867  2.71244  3.93385  3.72612  4.30645  3.73417  3.61195
3.38669  3.53686  3.12972  3.93726  0.96205    487 y - - -
         2.68618  4.42225  2.77519  2.73123  3.46354  2.40513  3.72494
3.29354  2.67741  2.69355  4.24690  2.90347  2.73739  3.18146  2.89801
2.37887  2.77519  2.98518  4.58477  3.61503
         0.03133  3.87472  4.59707  0.61958  0.77255  0.48576  0.95510
    486  3.15771  4.61926  4.19314  3.83291  3.13642  3.98506  4.48794
2.34049  3.60064  0.87242  3.15851  4.10818  4.41998  3.96843  3.77638
3.55606  3.45182  2.37843  5.01661  3.74048    488 l - - -
         2.68618  4.42225  2.77519  2.73123  3.46354  2.40513  3.72494
3.29354  2.67741  2.69355  4.24690  2.90347  2.73739  3.18146  2.89801
2.37887  2.77519  2.98518  4.58477  3.61503
         0.03133  3.87472  4.59707  0.61958  0.77255  0.48576  0.95510
    487  2.31597  4.23644  3.20823  3.02400  4.07846  2.96628  4.13384
3.60134  3.09793  3.32136  4.24719  3.24849  3.70524  3.44704  3.38396
1.08767  2.79625  3.13595  5.45075  4.16198    489 s - - -
         2.68618  4.42225  2.77519  2.73123  3.46354  2.40513  3.72494
3.29354  2.67741  2.69355  4.24690  2.90347  2.73739  3.18146  2.89801
2.37887  2.77519  2.98518  4.58477  3.61503
         0.03133  3.87472  4.59707  0.61958  0.77255  0.48576  0.95510
    488  2.20362  4.68439  2.72512  1.88920  4.12701  3.24295  3.72111
3.42796  2.55599  3.12935  3.99057  2.91584  3.77688  2.90156  2.98357
2.62573  2.40227  3.09538  5.41497  4.09062    490 e - - -
         2.68618  4.42225  2.77519  2.73123  3.46354  2.40513  3.72494
3.29354  2.67741  2.69355  4.24690  2.90347  2.73739  3.18146  2.89801
2.37887  2.77519  2.98518  4.58477  3.61503
         0.03133  3.87472  4.59707  0.61958  0.77255  0.48576  0.95510
```

```
    489   2.41377  4.25888  3.42992  3.18193  3.97070  3.09811  4.19910
3.09810  3.12495  2.96152  4.01182  3.39101  3.80201  3.51964  3.38358
2.61505  1.16810  2.79284  5.40831  4.18731    491 t - - -
          2.68618  4.42225  2.77519  2.73123  3.46354  2.40513  3.72494
3.29354  2.67741  2.69355  4.24690  2.90347  2.73739  3.18146  2.89801
2.37887  2.77519  2.98518  4.58477  3.61503
          0.03133  3.87472  4.59707  0.61958  0.77255  0.48576  0.95510
    490   2.80819  5.21615  1.73040  2.12440  4.53982  3.17039  3.72358
4.08614  2.70902  3.63346  4.48879  1.75873  3.78426  2.90042  3.25801
2.73930  3.11142  3.67452  5.76577  4.30053    492 d - - -
          2.68618  4.42225  2.77519  2.73123  3.46354  2.40513  3.72494
3.29354  2.67741  2.69355  4.24690  2.90347  2.73739  3.18146  2.89801
2.37887  2.77519  2.98518  4.58477  3.61503
          0.03133  3.87472  4.59707  0.61958  0.77255  0.48576  0.95510
    491   2.99187  5.12073  0.91483  2.33607  4.49801  3.21224  3.93281
4.08666  3.01732  3.69455  4.67262  2.89258  3.86518  3.17190  3.53391
2.95391  3.33046  3.72558  5.66619  4.37043    493 d - - -
          2.68618  4.42225  2.77519  2.73123  3.46354  2.40513  3.72494
3.29354  2.67741  2.69355  4.24690  2.90347  2.73739  3.18146  2.89801
2.37887  2.77519  2.98518  4.58477  3.61503
          0.03133  3.87472  4.59707  0.61958  0.77255  0.48576  0.95510
    492   1.50879  4.11123  3.33050  3.05929  4.15834  2.89943  4.13453
3.49003  3.10781  3.25178  4.11504  3.23094  3.64092  3.41443  3.41277
1.63872  2.66000  3.00580  5.52886  4.29979    494 a - - -
          2.68618  4.42225  2.77519  2.73123  3.46354  2.40513  3.72494
3.29354  2.67741  2.69355  4.24690  2.90347  2.73739  3.18146  2.89801
2.37887  2.77519  2.98518  4.58477  3.61503
          0.03133  3.87472  4.59707  0.61958  0.77255  0.48576  0.95510
    493   3.25019  4.73827  3.84149  3.57122  2.29338  3.83270  3.61936
3.25908  3.42867  2.71244  3.93385  3.72612  4.30645  3.73417  3.61195
3.38669  3.53686  3.12972  3.93726  0.96205    495 y - - -
          2.68618  4.42225  2.77519  2.73123  3.46354  2.40513  3.72494
3.29354  2.67741  2.69355  4.24690  2.90347  2.73739  3.18146  2.89801
2.37887  2.77519  2.98518  4.58477  3.61503
          0.03133  3.87472  4.59707  0.61958  0.77255  0.48576  0.95510
    494   2.80983  4.35172  4.12850  3.79161  3.55163  3.72629  4.59291
2.04988  3.65943  2.23820  3.51634  3.98111  4.28661  4.01903  3.87173
3.22990  3.17128  1.02205  5.33648  4.06827    496 v - - -
          2.68618  4.42225  2.77519  2.73123  3.46354  2.40513  3.72494
3.29354  2.67741  2.69355  4.24690  2.90347  2.73739  3.18146  2.89801
2.37887  2.77519  2.98518  4.58477  3.61503
          0.03133  3.87472  4.59707  0.61958  0.77255  0.48576  0.95510
    495   3.03284  4.45083  4.33904  3.95485  3.37561  4.06909  4.68249
1.07512  3.78303  1.96109  3.31052  4.19987  4.50000  4.13104  3.97934
3.56453  3.33193  1.84577  5.26697  4.00552    497 i - - -
          2.68618  4.42225  2.77519  2.73123  3.46354  2.40513  3.72494
3.29354  2.67741  2.69355  4.24690  2.90347  2.73739  3.18146  2.89801
2.37887  2.77519  2.98518  4.58477  3.61503
          0.03133  3.87472  4.59707  0.61958  0.77255  0.48576  0.95510
    496   2.75420  4.51129  3.36160  3.20088  4.19604  3.19867  4.27823
3.74193  3.25835  3.36857  4.41844  3.50740  0.82346  3.64706  3.50371
2.92174  3.19592  3.38706  5.38207  4.31670    498 p - - -
          2.68618  4.42225  2.77519  2.73123  3.46354  2.40513  3.72494
3.29354  2.67741  2.69355  4.24690  2.90347  2.73739  3.18146  2.89801
2.37887  2.77519  2.98518  4.58477  3.61503
```

0.03133 3.87472 4.59707 0.61958 0.77255 0.48576 0.95510
497 1.01052 4.19303 3.46476 3.25606 4.05451 3.01753 4.27786
3.18035 3.27238 3.06187 4.09337 3.40514 3.75695 3.61331 3.52281
2.54327 2.82077 2.83436 5.48144 4.28192 499 a - - -
2.68618 4.42225 2.77519 2.73123 3.46354 2.40513 3.72494
3.29354 2.67741 2.69355 4.24690 2.90347 2.73739 3.18146 2.89801
2.37887 2.77519 2.98518 4.58477 3.61503
0.03133 3.87472 4.59707 0.61958 0.77255 0.48576 0.95510
498 2.84535 4.50116 3.71278 3.21680 3.29752 3.70927 4.00199
2.61412 2.81002 1.31596 3.33068 3.55932 4.13027 3.34801 2.68076
3.09849 3.10482 2.54705 4.95135 3.66562 500 l - - -
2.68618 4.42225 2.77519 2.73123 3.46354 2.40513 3.72494
3.29354 2.67741 2.69355 4.24690 2.90347 2.73739 3.18146 2.89801
2.37887 2.77519 2.98518 4.58477 3.61503
0.03133 3.87472 4.59707 0.61958 0.77255 0.48576 0.95510
499 2.82662 5.04434 2.60081 2.17367 4.32091 3.33874 3.66733
3.78050 2.32875 3.32053 4.21283 2.88381 3.85573 1.62478 2.67096
2.80950 3.07933 3.45420 5.48717 4.15463 501 q - - -
2.68618 4.42225 2.77519 2.73123 3.46354 2.40513 3.72494
3.29354 2.67741 2.69355 4.24690 2.90347 2.73739 3.18146 2.89801
2.37887 2.77519 2.98518 4.58477 3.61503
0.03133 3.87472 4.59707 0.61958 0.77255 0.48576 0.95510
500 3.26109 4.66217 4.24182 3.95666 1.00804 3.95049 3.87804
2.78442 3.86362 2.15392 3.51419 4.02166 4.40896 4.03095 3.98279
3.53096 3.56220 2.77530 4.12630 2.49298 502 f - - -
2.68618 4.42225 2.77519 2.73123 3.46354 2.40513 3.72494
3.29354 2.67741 2.69355 4.24690 2.90347 2.73739 3.18146 2.89801
2.37887 2.77519 2.98518 4.58477 3.61503
0.03133 3.87472 4.59707 0.61958 0.77255 0.48576 0.95510
501 1.31387 4.10266 3.35180 3.09016 4.14272 2.90027 4.15325
3.44918 3.13356 3.23344 4.10709 3.24791 3.64553 3.44190 3.42854
1.90594 2.66303 2.97711 5.52372 4.29674 503 a - - -
2.68618 4.42225 2.77519 2.73123 3.46354 2.40513 3.72494
3.29354 2.67741 2.69355 4.24690 2.90347 2.73739 3.18146 2.89801
2.37887 2.77519 2.98518 4.58477 3.61503
0.03133 3.87472 4.59707 0.61958 0.77255 0.48576 0.95510
502 2.63803 4.75875 2.65741 1.83244 4.16706 3.25655 3.73489
3.46428 2.55327 3.16416 4.05190 2.90892 3.80216 2.91982 2.96394
2.68287 2.05037 3.14380 5.44962 4.12107 504 e - - -
2.68618 4.42225 2.77519 2.73123 3.46354 2.40513 3.72494
3.29354 2.67741 2.69355 4.24690 2.90347 2.73739 3.18146 2.89801
2.37887 2.77519 2.98518 4.58477 3.61503
0.03133 3.87472 4.59707 0.61958 0.77255 0.48576 0.95510
503 2.80983 4.35172 4.12850 3.79161 3.55163 3.72629 4.59291
2.04988 3.65943 2.23820 3.51634 3.98111 4.28661 4.01903 3.87173
3.22990 3.17128 1.02205 5.33648 4.06827 505 v - - -
2.68618 4.42225 2.77519 2.73123 3.46354 2.40513 3.72494
3.29354 2.67741 2.69355 4.24690 2.90347 2.73739 3.18146 2.89801
2.37887 2.77519 2.98518 4.58477 3.61503
0.03133 3.87472 4.59707 0.61958 0.77255 0.48576 0.95510
504 2.31597 4.23644 3.20823 3.02400 4.07846 2.96628 4.13384
3.60134 3.09793 3.32136 4.24719 3.24849 3.70524 3.44704 3.38396
1.08767 2.79625 3.13595 5.45075 4.16198 506 s - - -

2.68618 4.42225 2.77519 2.73123 3.46354 2.40513 3.72494
3.29354 2.67741 2.69355 4.24690 2.90347 2.73739 3.18146 2.89801
2.37887 2.77519 2.98518 4.58477 3.61503
0.03133 3.87472 4.59707 0.61958 0.77255 0.48576 0.95510
505 2.78132 5.01409 1.47215 2.27511 3.90290 3.27546 3.69452
3.69108 2.67103 3.28434 4.19510 2.82047 3.82710 2.92201 3.16506
2.76060 3.05846 3.37250 5.29395 3.15233 507 d - - -
2.68618 4.42225 2.77519 2.73123 3.46354 2.40513 3.72494
3.29354 2.67741 2.69355 4.24690 2.90347 2.73739 3.18146 2.89801
2.37887 2.77519 2.98518 4.58477 3.61503
0.03133 3.87472 4.59707 0.61958 0.77255 0.48576 0.95510
506 3.00784 4.84699 3.49870 3.00918 4.20214 3.49552 3.82565
3.74393 2.23793 3.25944 4.25948 3.36879 3.99792 3.04579 0.97495
3.09185 3.27359 3.46776 5.28422 4.12892 508 r - - -
2.68618 4.42225 2.77519 2.73123 3.46354 2.40513 3.72494
3.29354 2.67741 2.69355 4.24690 2.90347 2.73739 3.18146 2.89801
2.37887 2.77519 2.98518 4.58477 3.61503
0.03133 3.87472 4.59707 0.61958 0.77255 0.48576 0.95510
507 2.31597 4.23644 3.20823 3.02400 4.07846 2.96628 4.13384
3.60134 3.09793 3.32136 4.24719 3.24849 3.70524 3.44704 3.38396
1.08767 2.79625 3.13595 5.45075 4.16198 509 s - - -
2.68618 4.42225 2.77519 2.73123 3.46354 2.40513 3.72494
3.29354 2.67741 2.69355 4.24690 2.90347 2.73739 3.18146 2.89801
2.37887 2.77519 2.98518 4.58477 3.61503
0.03133 3.87472 4.59707 0.61958 0.77255 0.48576 0.95510
508 3.26109 4.66217 4.24182 3.95666 1.00804 3.95049 3.87804
2.78442 3.86362 2.15392 3.51419 4.02166 4.40896 4.03095 3.98279
3.53096 3.56220 2.77530 4.12630 2.49298 510 f - - -
2.68618 4.42225 2.77519 2.73123 3.46354 2.40513 3.72494
3.29354 2.67741 2.69355 4.24690 2.90347 2.73739 3.18146 2.89801
2.37887 2.77519 2.98518 4.58477 3.61503
0.03133 3.87472 4.59707 0.61958 0.77255 0.48576 0.95510
509 3.15771 4.61926 4.19314 3.83291 3.13642 3.98506 4.48794
2.34049 3.60064 0.87242 3.15851 4.10818 4.41998 3.96843 3.77638
3.55606 3.45182 2.37843 5.01661 3.74048 511 l - - -
2.68618 4.42225 2.77519 2.73123 3.46354 2.40513 3.72494
3.29354 2.67741 2.69355 4.24690 2.90347 2.73739 3.18146 2.89801
2.37887 2.77519 2.98518 4.58477 3.61503
0.03133 3.87472 4.59707 0.61958 0.77255 0.48576 0.95510
510 2.95406 5.06580 2.48830 1.02217 4.41227 3.27540 3.86227
3.86661 2.74307 3.48333 4.45717 2.93393 3.88006 3.07782 3.15222
2.93617 3.25828 3.55019 5.58341 4.30555 512 e - - -
2.68618 4.42225 2.77519 2.73123 3.46354 2.40513 3.72494
3.29354 2.67741 2.69355 4.24690 2.90347 2.73739 3.18146 2.89801
2.37887 2.77519 2.98518 4.58477 3.61503
0.03133 3.87472 4.59707 0.61958 0.77255 0.48576 0.95510
511 2.99187 5.12073 0.91483 2.33607 4.49801 3.21224 3.93281
4.08666 3.01732 3.69455 4.67262 2.89258 3.86518 3.17190 3.53391
2.95391 3.33046 3.72558 5.66619 4.37043 513 d - - -
2.68618 4.42225 2.77519 2.73123 3.46354 2.40513 3.72494
3.29354 2.67741 2.69355 4.24690 2.90347 2.73739 3.18146 2.89801
2.37887 2.77519 2.98518 4.58477 3.61503
0.03133 3.87472 4.59707 0.61958 0.77255 0.48576 0.95510

```
     512   2.41377  4.25888  3.42992  3.18193  3.97070  3.09811  4.19910
 3.09810  3.12495  2.96152  4.01182  3.39101  3.80201  3.51964  3.38358
 2.61505  1.16810  2.79284  5.40831  4.18731    514 t - - -
           2.68618  4.42225  2.77519  2.73123  3.46354  2.40513  3.72494
 3.29354  2.67741  2.69355  4.24690  2.90347  2.73739  3.18146  2.89801
 2.37887  2.77519  2.98518  4.58477  3.61503
           0.03133  3.87472  4.59707  0.61958  0.77255  0.48576  0.95510
     513   2.75420  4.51129  3.36160  3.20088  4.19604  3.19867  4.27823
 3.74193  3.25835  3.36857  4.41844  3.50740  0.82346  3.64706  3.50371
 2.92174  3.19592  3.38706  5.38207  4.31670    515 p - - -
           2.68618  4.42225  2.77519  2.73123  3.46354  2.40513  3.72494
 3.29354  2.67741  2.69355  4.24690  2.90347  2.73739  3.18146  2.89801
 2.37887  2.77519  2.98518  4.58477  3.61503
           0.03133  3.87472  4.59707  0.61958  0.77255  0.48576  0.95510
     514   2.99187  5.12073  0.91483  2.33607  4.49801  3.21224  3.93281
 4.08666  3.01732  3.69455  4.67262  2.89258  3.86518  3.17190  3.53391
 2.95391  3.33046  3.72558  5.66619  4.37043    516 d - - -
           2.68618  4.42225  2.77519  2.73123  3.46354  2.40513  3.72494
 3.29354  2.67741  2.69355  4.24690  2.90347  2.73739  3.18146  2.89801
 2.37887  2.77519  2.98518  4.58477  3.61503
           0.03133  3.87472  4.59707  0.61958  0.77255  0.48576  0.95510
     515   2.88236  4.86831  2.90423  2.65889  4.03720  3.36741  3.81726
 3.66821  2.47950  3.17034  4.19804  3.11869  3.92083  1.23004  2.76368
 2.92996  3.17928  3.39771  5.30876  4.00263    517 q - - -
           2.68618  4.42225  2.77519  2.73123  3.46354  2.40513  3.72494
 3.29354  2.67741  2.69355  4.24690  2.90347  2.73739  3.18146  2.89801
 2.37887  2.77519  2.98518  4.58477  3.61503
           0.03133  3.87472  4.59707  0.61958  0.77255  0.48576  0.95510
     516   1.01052  4.19303  3.46476  3.25606  4.05451  3.01753  4.27786
 3.18035  3.27238  3.06187  4.09337  3.40514  3.75695  3.61331  3.52281
 2.54327  2.82077  2.83436  5.48144  4.28192    518 a - - -
           2.68618  4.42225  2.77519  2.73123  3.46354  2.40513  3.72494
 3.29354  2.67741  2.69355  4.24690  2.90347  2.73739  3.18146  2.89801
 2.37887  2.77519  2.98518  4.58477  3.61503
           0.03133  3.87472  4.59707  0.61958  0.77255  0.48576  0.95510
     517   2.41377  4.25888  3.42992  3.18193  3.97070  3.09811  4.19910
 3.09810  3.12495  2.96152  4.01182  3.39101  3.80201  3.51964  3.38358
 2.61505  1.16810  2.79284  5.40831  4.18731    519 t - - -
           2.68618  4.42225  2.77519  2.73123  3.46354  2.40513  3.72494
 3.29354  2.67741  2.69355  4.24690  2.90347  2.73739  3.18146  2.89801
 2.37887  2.77519  2.98518  4.58477  3.61503
           0.03133  3.87472  4.59707  0.61958  0.77255  0.48576  0.95510
     518   2.90799  5.37303  1.24917  1.90690  4.66947  3.16985  3.74851
 4.16772  2.78190  3.72149  4.60621  2.66460  3.80370  2.93016  3.36115
 2.80697  3.20773  3.77041  5.87198  4.40129    520 d - - -
           2.68618  4.42225  2.77519  2.73123  3.46354  2.40513  3.72494
 3.29354  2.67741  2.69355  4.24690  2.90347  2.73739  3.18146  2.89801
 2.37887  2.77519  2.98518  4.58477  3.61503
           0.03133  3.87472  4.59707  0.61958  0.77255  0.48576  0.95510
     519   2.66752  4.44283  3.34177  3.24141  4.38386  0.72564  4.36163
 3.98394  3.42300  3.63781  4.61350  3.50605  3.83404  3.74696  3.65888
 2.83948  3.14893  3.51182  5.47850  4.48580    521 g - - -
           2.68618  4.42225  2.77519  2.73123  3.46354  2.40513  3.72494
 3.29354  2.67741  2.69355  4.24690  2.90347  2.73739  3.18146  2.89801
 2.37887  2.77519  2.98518  4.58477  3.61503
```

```
          0.03133  3.87472  4.59707  0.61958  0.77255  0.48576  0.95510
    520   2.31597  4.23644  3.20823  3.02400  4.07846  2.96628  4.13384
3.60134  3.09793  3.32136  4.24719  3.24849  3.70524  3.44704  3.38396
1.08767  2.79625  3.13595  5.45075  4.16198    522 s - - -
          2.68618  4.42225  2.77519  2.73123  3.46354  2.40513  3.72494
3.29354  2.67741  2.69355  4.24690  2.90347  2.73739  3.18146  2.89801
2.37887  2.77519  2.98518  4.58477  3.61503
          0.03133  3.87472  4.59707  0.61958  0.77255  0.48576  0.95510
    521   3.03284  4.45083  4.33904  3.95485  3.37561  4.06909  4.68249
1.07512  3.78303  1.96109  3.31052  4.19987  4.50000  4.13104  3.97934
3.56453  3.33193  1.84577  5.26697  4.00552    523 i - - -
          2.68618  4.42225  2.77519  2.73123  3.46354  2.40513  3.72494
3.29354  2.67741  2.69355  4.24690  2.90347  2.73739  3.18146  2.89801
2.37887  2.77519  2.98518  4.58477  3.61503
          0.03133  3.87472  4.59707  0.61958  0.77255  0.48576  0.95510
    522   2.97404  4.93809  3.15019  2.77112  4.32503  3.46335  3.76023
3.75442  1.03520  3.31897  4.28021  3.19552  3.96346  2.95151  2.43176
3.01582  3.22709  3.46205  5.37869  4.19175    524 k - - -
          2.68618  4.42225  2.77519  2.73123  3.46354  2.40513  3.72494
3.29354  2.67741  2.69355  4.24690  2.90347  2.73739  3.18146  2.89801
2.37887  2.77519  2.98518  4.58477  3.61503
          0.03133  3.87472  4.59707  0.61958  0.77255  0.48576  0.95510
    523   3.26109  4.66217  4.24182  3.95666  1.00804  3.95049  3.87804
2.78442  3.86362  2.15392  3.51419  4.02166  4.40896  4.03095  3.98279
3.53096  3.56220  2.77530  4.12630  2.49298    525 f - - -
          2.68618  4.42225  2.77519  2.73123  3.46354  2.40513  3.72494
3.29354  2.67741  2.69355  4.24690  2.90347  2.73739  3.18146  2.89801
2.37887  2.77519  2.98518  4.58477  3.61503
          0.03133  3.87472  4.59707  0.61958  0.77255  0.48576  0.95510
    524   1.60213  4.12530  3.45121  3.12718  4.03243  2.98732  4.14588
3.12800  3.10413  3.02608  3.96015  3.30011  3.70175  3.43419  3.39814
2.43424  1.77348  2.75963  5.45747  4.24212    526 a - - -
          2.68618  4.42225  2.77519  2.73123  3.46354  2.40513  3.72494
3.29354  2.67741  2.69355  4.24690  2.90347  2.73739  3.18146  2.89801
2.37887  2.77519  2.98518  4.58477  3.61503
          0.03133  3.87472  4.59707  0.61958  0.77255  0.48576  0.95510
    525   2.67888  5.07915  2.20919  1.98003  4.38681  2.96296  3.59945
3.84513  2.39476  3.37316  4.17263  2.77044  3.76635  2.73524  2.33503
2.64007  2.92597  3.45835  5.54544  4.15080    527 e - - -
          2.68618  4.42225  2.77519  2.73123  3.46354  2.40513  3.72494
3.29354  2.67741  2.69355  4.24690  2.90347  2.73739  3.18146  2.89801
2.37887  2.77519  2.98518  4.58477  3.61503
          0.03133  3.87472  4.59707  0.61958  0.77255  0.48576  0.95510
    526   2.41377  4.25888  3.42992  3.18193  3.97070  3.09811  4.19910
3.09810  3.12495  2.96152  4.01182  3.39101  3.80201  3.51964  3.38358
2.61505  1.16810  2.79284  5.40831  4.18731    528 t - - -
          2.68618  4.42225  2.77519  2.73123  3.46354  2.40513  3.72494
3.29354  2.67741  2.69355  4.24690  2.90347  2.73739  3.18146  2.89801
2.37887  2.77519  2.98518  4.58477  3.61503
          0.03133  3.87472  4.59707  0.61958  0.77255  0.48576  0.95510
    527   2.92215  4.37268  4.29745  3.80734  2.04840  3.99777  4.25024
2.15527  3.69660  1.87454  3.16740  4.00660  4.37486  3.91073  3.89080
3.35765  3.19142  1.57758  4.71275  3.28732    529 v - - -
```

```
         2.68618  4.42225  2.77519  2.73123  3.46354  2.40513  3.72494
3.29354  2.67741  2.69355  4.24690  2.90347  2.73739  3.18146  2.89801
2.37887  2.77519  2.98518  4.58477  3.61503
         0.03133  3.87472  4.59707  0.61958  0.77255  0.48576  0.95510
    528  3.13260  4.49754  4.57868  4.06001  3.16843  4.35367  4.71105
1.51681  3.88418  1.27239  3.01918  4.33673  4.62015  4.13416  4.07017
3.72128  3.37745  1.92247  5.17278  3.98634    530 l - - -
         2.68618  4.42225  2.77519  2.73123  3.46354  2.40513  3.72494
3.29354  2.67741  2.69355  4.24690  2.90347  2.73739  3.18146  2.89801
2.37887  2.77519  2.98518  4.58477  3.61503
         0.03133  3.87472  4.59707  0.61958  0.77255  0.48576  0.95510
    529  2.22146  4.18839  3.11012  2.90581  4.27179  1.77987  4.09370
3.73831  3.06346  3.41077  4.24455  3.15053  3.64007  3.35557  3.39718
1.62230  2.70183  3.18739  5.59327  4.34740    531 s - - -
         2.68618  4.42225  2.77519  2.73123  3.46354  2.40513  3.72494
3.29354  2.67741  2.69355  4.24690  2.90347  2.73739  3.18146  2.89801
2.37887  2.77519  2.98518  4.58477  3.61503
         0.03133  3.87472  4.59707  0.61958  0.77255  0.48576  0.95510
    530  2.72515  4.72193  2.70930  2.59495  4.08579  3.18536  3.92257
3.79656  2.82640  3.44315  4.39831  1.15407  3.83729  3.19525  3.18120
2.79220  3.10703  3.41854  5.39143  4.03390    532 n - - -
         2.68618  4.42225  2.77519  2.73123  3.46354  2.40513  3.72494
3.29354  2.67741  2.69355  4.24690  2.90347  2.73739  3.18146  2.89801
2.37887  2.77519  2.98518  4.58477  3.61503
         0.03133  3.87472  4.59707  0.61958  0.77255  0.48576  0.95510
    531  2.95406  5.06580  2.48830  1.02217  4.41227  3.27540  3.86227
3.86661  2.74307  3.48333  4.45717  2.93393  3.88006  3.07782  3.15222
2.93617  3.25828  3.55019  5.58341  4.30555    533 e - - -
         2.68618  4.42225  2.77519  2.73123  3.46354  2.40513  3.72494
3.29354  2.67741  2.69355  4.24690  2.90347  2.73739  3.18146  2.89801
2.37887  2.77519  2.98518  4.58477  3.61503
         0.03133  3.87472  4.59707  0.61958  0.77255  0.48576  0.95510
    532  1.01052  4.19303  3.46476  3.25606  4.05451  3.01753  4.27786
3.18035  3.27238  3.06187  4.09337  3.40514  3.75695  3.61331  3.52281
2.54327  2.82077  2.83436  5.48144  4.28192    534 a - - -
         2.68618  4.42225  2.77519  2.73123  3.46354  2.40513  3.72494
3.29354  2.67741  2.69355  4.24690  2.90347  2.73739  3.18146  2.89801
2.37887  2.77519  2.98518  4.58477  3.61503
         0.03133  3.87472  4.59707  0.61958  0.77255  0.48576  0.95510
    533  2.97404  4.93809  3.15019  2.77112  4.32503  3.46335  3.76023
3.75442  1.03520  3.31897  4.28021  3.19552  3.96346  2.95151  2.43176
3.01582  3.22709  3.46205  5.37869  4.19175    535 k - - -
         2.68618  4.42225  2.77519  2.73123  3.46354  2.40513  3.72494
3.29354  2.67741  2.69355  4.24690  2.90347  2.73739  3.18146  2.89801
2.37887  2.77519  2.98518  4.58477  3.61503
         0.03133  3.87472  4.59707  0.61958  0.77255  0.48576  0.95510
    534  3.25019  4.73827  3.84149  3.57122  2.29338  3.83270  3.61936
3.25908  3.42867  2.71244  3.93385  3.72612  4.30645  3.73417  3.61195
3.38669  3.53686  3.12972  3.93726  0.96205    536 y - - -
         2.68618  4.42225  2.77519  2.73123  3.46354  2.40513  3.72494
3.29354  2.67741  2.69355  4.24690  2.90347  2.73739  3.18146  2.89801
2.37887  2.77519  2.98518  4.58477  3.61503
         0.03133  3.87472  4.59707  0.61958  0.77255  0.48576  0.95510
```

```
    535   2.31597  4.23644  3.20823  3.02400  4.07846  2.96628  4.13384
3.60134  3.09793  3.32136  4.24719  3.24849  3.70524  3.44704  3.38396
1.08767  2.79625  3.13595  5.45075  4.16198    537 s - - -
          2.68618  4.42225  2.77519  2.73123  3.46354  2.40513  3.72494
3.29354  2.67741  2.69355  4.24690  2.90347  2.73739  3.18146  2.89801
2.37887  2.77519  2.98518  4.58477  3.61503
          0.03133  3.87472  4.59707  0.61958  0.77255  0.48576  0.95510
    536   3.03284  4.45083  4.33904  3.95485  3.37561  4.06909  4.68249
1.07512  3.78303  1.96109  3.31052  4.19987  4.50000  4.13104  3.97934
3.56453  3.33193  1.84577  5.26697  4.00552    538 i - - -
          2.68618  4.42225  2.77519  2.73123  3.46354  2.40513  3.72494
3.29354  2.67741  2.69355  4.24690  2.90347  2.73739  3.18146  2.89801
2.37887  2.77519  2.98518  4.58477  3.61503
          0.03133  3.87472  4.59707  0.61958  0.77255  0.48576  0.95510
    537   3.00784  4.84699  3.49870  3.00918  4.20214  3.49552  3.82565
3.74393  2.23793  3.25944  4.25948  3.36879  3.99792  3.04579  0.97495
3.09185  3.27359  3.46776  5.28422  4.12892    539 r - - -
          2.68618  4.42225  2.77519  2.73123  3.46354  2.40513  3.72494
3.29354  2.67741  2.69355  4.24690  2.90347  2.73739  3.18146  2.89801
2.37887  2.77519  2.98518  4.58477  3.61503
          0.03133  3.87472  4.59707  0.61958  0.77255  0.48576  0.95510
    538   3.15771  4.61926  4.19314  3.83291  3.13642  3.98506  4.48794
2.34049  3.60064  0.87242  3.15851  4.10818  4.41998  3.96843  3.77638
3.55606  3.45182  2.37843  5.01661  3.74048    540 l - - -
          2.68618  4.42225  2.77519  2.73123  3.46354  2.40513  3.72494
3.29354  2.67741  2.69355  4.24690  2.90347  2.73739  3.18146  2.89801
2.37887  2.77519  2.98518  4.58477  3.61503
          0.03133  3.87472  4.59707  0.61958  0.77255  0.48576  0.95510
    539   2.72515  4.72193  2.70930  2.59495  4.08579  3.18536  3.92257
3.79656  2.82640  3.44315  4.39831  1.15407  3.83729  3.19525  3.18120
2.79220  3.10703  3.41854  5.39143  4.03390    541 n - - -
          2.68618  4.42225  2.77519  2.73123  3.46354  2.40513  3.72494
3.29354  2.67741  2.69355  4.24690  2.90347  2.73739  3.18146  2.89801
2.37887  2.77519  2.98518  4.58477  3.61503
          0.03133  3.87472  4.59707  0.61958  0.77255  0.48576  0.95510
    540   2.41377  4.25888  3.42992  3.18193  3.97070  3.09811  4.19910
3.09810  3.12495  2.96152  4.01182  3.39101  3.80201  3.51964  3.38358
2.61505  1.16810  2.79284  5.40831  4.18731    542 t - - -
          2.68618  4.42225  2.77519  2.73123  3.46354  2.40513  3.72494
3.29354  2.67741  2.69355  4.24690  2.90347  2.73739  3.18146  2.89801
2.37887  2.77519  2.98518  4.58477  3.61503
          0.03133  3.87472  4.59707  0.61958  0.77255  0.48576  0.95510
    541   2.66752  4.44283  3.34177  3.24141  4.38386  0.72564  4.36163
3.98394  3.42300  3.63781  4.61350  3.50605  3.83404  3.74696  3.65888
2.83948  3.14893  3.51182  5.47850  4.48580    543 g - - -
          2.68618  4.42225  2.77519  2.73123  3.46354  2.40513  3.72494
3.29354  2.67741  2.69355  4.24690  2.90347  2.73739  3.18146  2.89801
2.37887  2.77519  2.98518  4.58477  3.61503
          0.03133  3.87472  4.59707  0.61958  0.77255  0.48576  0.95510
    542   3.26109  4.66217  4.24182  3.95666  1.00804  3.95049  3.87804
2.78442  3.86362  2.15392  3.51419  4.02166  4.40896  4.03095  3.98279
3.53096  3.56220  2.77530  4.12630  2.49298    544 f - - -
          2.68618  4.42225  2.77519  2.73123  3.46354  2.40513  3.72494
3.29354  2.67741  2.69355  4.24690  2.90347  2.73739  3.18146  2.89801
2.37887  2.77519  2.98518  4.58477  3.61503
```

0.03133 3.87472 4.59707 0.61958 0.77255 0.48576 0.95510
543 2.72515 4.72193 2.70930 2.59495 4.08579 3.18536 3.92257
3.79656 2.82640 3.44315 4.39831 1.15407 3.83729 3.19525 3.18120
2.79220 3.10703 3.41854 5.39143 4.03390 545 n - - -
2.68618 4.42225 2.77519 2.73123 3.46354 2.40513 3.72494
3.29354 2.67741 2.69355 4.24690 2.90347 2.73739 3.18146 2.89801
2.37887 2.77519 2.98518 4.58477 3.61503
0.03133 3.87472 4.59707 0.61958 0.77255 0.48576 0.95510
544 2.41377 4.25888 3.42992 3.18193 3.97070 3.09811 4.19910
3.09810 3.12495 2.96152 4.01182 3.39101 3.80201 3.51964 3.38358
2.61505 1.16810 2.79284 5.40831 4.18731 546 t - - -
2.68618 4.42225 2.77519 2.73123 3.46354 2.40513 3.72494
3.29354 2.67741 2.69355 4.24690 2.90347 2.73739 3.18146 2.89801
2.37887 2.77519 2.98518 4.58477 3.61503
0.03133 3.87472 4.59707 0.61958 0.77255 0.48576 0.95510
545 1.01052 4.19303 3.46476 3.25606 4.05451 3.01753 4.27786
3.18035 3.27238 3.06187 4.09337 3.40514 3.75695 3.61331 3.52281
2.54327 2.82077 2.83436 5.48144 4.28192 547 a - - -
2.68618 4.42225 2.77519 2.73123 3.46354 2.40513 3.72494
3.29354 2.67741 2.69355 4.24690 2.90347 2.73739 3.18146 2.89801
2.37887 2.77519 2.98518 4.58477 3.61503
0.03133 3.87472 4.59707 0.61958 0.77255 0.48576 0.95510
546 2.41377 4.25888 3.42992 3.18193 3.97070 3.09811 4.19910
3.09810 3.12495 2.96152 4.01182 3.39101 3.80201 3.51964 3.38358
2.61505 1.16810 2.79284 5.40831 4.18731 548 t - - -
2.68618 4.42225 2.77519 2.73123 3.46354 2.40513 3.72494
3.29354 2.67741 2.69355 4.24690 2.90347 2.73739 3.18146 2.89801
2.37887 2.77519 2.98518 4.58477 3.61503
0.03133 3.87472 4.59707 0.61958 0.77255 0.48576 0.95510
547 3.00784 4.84699 3.49870 3.00918 4.20214 3.49552 3.82565
3.74393 2.23793 3.25944 4.25948 3.36879 3.99792 3.04579 0.97495
3.09185 3.27359 3.46776 5.28422 4.12892 549 r - - -
2.68618 4.42225 2.77519 2.73123 3.46354 2.40513 3.72494
3.29354 2.67741 2.69355 4.24690 2.90347 2.73739 3.18146 2.89801
2.37887 2.77519 2.98518 4.58477 3.61503
0.03133 3.87472 4.59707 0.61958 0.77255 0.48576 0.95510
548 3.25019 4.73827 3.84149 3.57122 2.29338 3.83270 3.61936
3.25908 3.42867 2.71244 3.93385 3.72612 4.30645 3.73417 3.61195
3.38669 3.53686 3.12972 3.93726 0.96205 550 y - - -
2.68618 4.42225 2.77519 2.73123 3.46354 2.40513 3.72494
3.29354 2.67741 2.69355 4.24690 2.90347 2.73739 3.18146 2.89801
2.37887 2.77519 2.98518 4.58477 3.61503
0.03133 3.87472 4.59707 0.61958 0.77255 0.48576 0.95510
549 2.96903 5.06024 3.36062 2.73298 4.48250 3.59025 3.57502
3.84188 1.48134 3.31283 4.18959 3.13975 3.97260 2.71698 1.58875
2.97553 3.14711 3.53066 5.37786 4.19791 551 k - - -
2.68618 4.42225 2.77519 2.73123 3.46354 2.40513 3.72494
3.29354 2.67741 2.69355 4.24690 2.90347 2.73739 3.18146 2.89801
2.37887 2.77519 2.98518 4.58477 3.61503
0.03133 3.87472 4.59707 0.61958 0.77255 0.48576 0.95510
550 3.15771 4.61926 4.19314 3.83291 3.13642 3.98506 4.48794
2.34049 3.60064 0.87242 3.15851 4.10818 4.41998 3.96843 3.77638
3.55606 3.45182 2.37843 5.01661 3.74048 552 l - - -

```
         2.68618  4.42225  2.77519  2.73123  3.46354  2.40513  3.72494
3.29354  2.67741  2.69355  4.24690  2.90347  2.73739  3.18146  2.89801
2.37887  2.77519  2.98518  4.58477  3.61503
         0.03133  3.87472  4.59707  0.61958  0.77255  0.48576  0.95510
    551  3.01032  4.38075  4.56430  4.07535  3.43460  4.25825  4.81343
1.25130  3.94304  1.94502  3.26584  4.31892  4.60504  4.22798  4.15709
3.64442  3.28439  1.45898  5.38407  4.17226    553 i - - -
         2.68618  4.42225  2.77519  2.73123  3.46354  2.40513  3.72494
3.29354  2.67741  2.69355  4.24690  2.90347  2.73739  3.18146  2.89801
2.37887  2.77519  2.98518  4.58477  3.61503
         0.03133  3.87472  4.59707  0.61958  0.77255  0.48576  0.95510
    552  3.03284  4.45083  4.33904  3.95485  3.37561  4.06909  4.68249
1.07512  3.78303  1.96109  3.31052  4.19987  4.50000  4.13104  3.97934
3.56453  3.33193  1.84577  5.26697  4.00552    554 i - - -
         2.68618  4.42225  2.77519  2.73123  3.46354  2.40513  3.72494
3.29354  2.67741  2.69355  4.24690  2.90347  2.73739  3.18146  2.89801
2.37887  2.77519  2.98518  4.58477  3.61503
         0.03133  3.87472  4.59707  0.61958  0.77255  0.48576  0.95510
    553  3.00784  4.84699  3.49870  3.00918  4.20214  3.49552  3.82565
3.74393  2.23793  3.25944  4.25948  3.36879  3.99792  3.04579  0.97495
3.09185  3.27359  3.46776  5.28422  4.12892    555 r - - -
         2.68618  4.42225  2.77519  2.73123  3.46354  2.40513  3.72494
3.29354  2.67741  2.69355  4.24690  2.90347  2.73739  3.18146  2.89801
2.37887  2.77519  2.98518  4.58477  3.61503
         0.03133  3.87472  4.59707  0.61958  0.77255  0.48576  0.95510
    554  2.92657  4.37793  4.29627  3.80772  1.98883  3.99679  4.23434
2.17182  3.69631  1.87011  3.16529  4.00366  4.37391  3.90790  3.88881
3.35700  3.19581  1.61845  4.68799  3.25322    556 v - - -
         2.68618  4.42225  2.77519  2.73123  3.46354  2.40513  3.72494
3.29354  2.67741  2.69355  4.24690  2.90347  2.73739  3.18146  2.89801
2.37887  2.77519  2.98518  4.58477  3.61503
         0.03133  3.87472  4.59707  0.61958  0.77255  0.48576  0.95510
    555  2.96767  5.06119  3.35625  2.73010  4.48346  3.58906  3.57473
3.84312  1.45617  3.31382  4.19005  3.13783  3.97160  2.71647  1.62065
2.97368  3.14595  3.53134  5.37886  4.19810    557 k - - -
         2.68618  4.42225  2.77519  2.73123  3.46354  2.40513  3.72494
3.29354  2.67741  2.69355  4.24690  2.90347  2.73739  3.18146  2.89801
2.37887  2.77519  2.98518  4.58477  3.61503
         0.03133  3.87472  4.59707  0.61958  0.77255  0.48576  0.95510
    556  1.73936  4.18266  3.78494  3.38798  3.71665  3.31955  4.29089
2.37527  3.31223  2.53016  3.62936  3.57205  3.95065  3.63486  3.58388
2.74178  2.85362  1.62014  5.32860  4.10237    558 v - - -
         2.68618  4.42225  2.77519  2.73123  3.46354  2.40513  3.72494
3.29354  2.67741  2.69355  4.24690  2.90347  2.73739  3.18146  2.89801
2.37887  2.77519  2.98518  4.58477  3.61503
         0.03133  3.87472  4.59707  0.61958  0.77255  0.48576  0.95510
    557  2.48125  4.48013  3.07812  2.67293  4.03307  3.21106  3.78336
3.41050  2.52007  3.05936  3.93424  3.07456  2.02054  2.98875  2.56066
2.59397  2.53829  3.06005  5.32466  4.05583    559 p - - -
         2.68618  4.42225  2.77519  2.73123  3.46354  2.40513  3.72494
3.29354  2.67741  2.69355  4.24690  2.90347  2.73739  3.18146  2.89801
2.37887  2.77519  2.98518  4.58477  3.61503
         0.03133  3.87472  4.59707  0.61958  0.77255  0.48576  0.95510
```

```
     558   1.90986  3.48942  3.59027  3.06934  3.32127  3.39054  3.88281
 2.70656  2.97841  2.48643  3.42791  3.36768  3.88433  3.28851  3.27775
 2.71852  2.77201  2.47547  4.82352  2.61835    560 a - - -
           2.68618  4.42225  2.77519  2.73123  3.46354  2.40513  3.72494
 3.29354  2.67741  2.69355  4.24690  2.90347  2.73739  3.18146  2.89801
 2.37887  2.77519  2.98518  4.58477  3.61503
           0.03133  3.87472  4.59707  0.61958  0.77255  0.48576  0.95510
     559   3.00784  4.84699  3.49870  3.00918  4.20214  3.49552  3.82565
 3.74393  2.23793  3.25944  4.25948  3.36879  3.99792  3.04579  0.97495
 3.09185  3.27359  3.46776  5.28422  4.12892    561 r - - -
           2.68618  4.42225  2.77519  2.73123  3.46354  2.40513  3.72494
 3.29354  2.67741  2.69355  4.24690  2.90347  2.73739  3.18146  2.89801
 2.37887  2.77519  2.98518  4.58477  3.61503
           0.03133  3.87472  4.59707  0.61958  0.77255  0.48576  0.95510
     560   3.15771  4.61926  4.19314  3.83291  3.13642  3.98506  4.48794
 2.34049  3.60064  0.87242  3.15851  4.10818  4.41998  3.96843  3.77638
 3.55606  3.45182  2.37843  5.01661  3.74048    562 l - - -
           2.68618  4.42225  2.77519  2.73123  3.46354  2.40513  3.72494
 3.29354  2.67741  2.69355  4.24690  2.90347  2.73739  3.18146  2.89801
 2.37887  2.77519  2.98518  4.58477  3.61503
           0.03133  3.87472  4.59707  0.61958  0.77255  0.48576  0.95510
     561   1.56963  4.20968  3.24007  2.94819  4.09818  2.99107  4.05703
 3.39962  2.98902  3.14653  4.04590  3.20400  2.09992  3.32073  3.31471
 2.44666  2.72563  2.98227  5.46361  4.23107    563 a - - -
           2.68618  4.42225  2.77519  2.73123  3.46354  2.40513  3.72494
 3.29354  2.67741  2.69355  4.24690  2.90347  2.73739  3.18146  2.89801
 2.37887  2.77519  2.98518  4.58477  3.61503
           0.03133  3.87472  4.59707  0.61958  0.77255  0.48576  0.95510
     562   1.01052  4.19303  3.46476  3.25606  4.05451  3.01753  4.27786
 3.18035  3.27238  3.06187  4.09337  3.40514  3.75695  3.61331  3.52281
 2.54327  2.82077  2.83436  5.48144  4.28192    564 a - - -
           2.68618  4.42225  2.77519  2.73123  3.46354  2.40513  3.72494
 3.29354  2.67741  2.69355  4.24690  2.90347  2.73739  3.18146  2.89801
 2.37887  2.77519  2.98518  4.58477  3.61503
           0.03133  3.87472  4.59707  0.61958  0.77255  0.48576  0.95510
     563   2.66752  4.44283  3.34177  3.24141  4.38386  0.72564  4.36163
 3.98394  3.42300  3.63781  4.61350  3.50605  3.83404  3.74696  3.65888
 2.83948  3.14893  3.51182  5.47850  4.48580    565 g - - -
           2.68618  4.42225  2.77519  2.73123  3.46354  2.40513  3.72494
 3.29354  2.67741  2.69355  4.24690  2.90347  2.73739  3.18146  2.89801
 2.37887  2.77519  2.98518  4.58477  3.61503
           0.03133  3.87472  4.59707  0.61958  0.77255  0.48576  0.95510
     564   3.03284  4.45083  4.33904  3.95485  3.37561  4.06909  4.68249
 1.07512  3.78303  1.96109  3.31052  4.19987  4.50000  4.13104  3.97934
 3.56453  3.33193  1.84577  5.26697  4.00552    566 i - - -
           2.68618  4.42225  2.77519  2.73123  3.46354  2.40513  3.72494
 3.29354  2.67741  2.69355  4.24690  2.90347  2.73739  3.18146  2.89801
 2.37887  2.77519  2.98518  4.58477  3.61503
           0.03133  3.87472  4.59707  0.61958  0.77255  0.48576  0.95510
     565   3.00784  4.84699  3.49870  3.00918  4.20214  3.49552  3.82565
 3.74393  2.23793  3.25944  4.25948  3.36879  3.99792  3.04579  0.97495
 3.09185  3.27359  3.46776  5.28422  4.12892    567 r - - -
           2.68618  4.42225  2.77519  2.73123  3.46354  2.40513  3.72494
 3.29354  2.67741  2.69355  4.24690  2.90347  2.73739  3.18146  2.89801
 2.37887  2.77519  2.98518  4.58477  3.61503
```

```
          0.03133  3.87472  4.59707  0.61958  0.77255  0.48576  0.95510
    566   2.80983  4.35172  4.12850  3.79161  3.55163  3.72629  4.59291
2.04988  3.65943  2.23820  3.51634  3.98111  4.28661  4.01903  3.87173
3.22990  3.17128  1.02205  5.33648  4.06827    568 v - - -
          2.68618  4.42225  2.77519  2.73123  3.46354  2.40513  3.72494
3.29354  2.67741  2.69355  4.24690  2.90347  2.73739  3.18146  2.89801
2.37887  2.77519  2.98518  4.58477  3.61503
          0.03133  3.87472  4.59707  0.61958  0.77255  0.48576  0.95510
    567   2.87154  4.98989  3.16094  2.63265  4.31885  3.51165  3.59460
3.75403  2.00002  3.24453  4.12584  3.07307  3.92443  2.00617  1.70295
2.88434  3.07732  3.44183  5.35263  4.11135    569 r - - -
          2.68618  4.42225  2.77519  2.73123  3.46354  2.40513  3.72494
3.29354  2.67741  2.69355  4.24690  2.90347  2.73739  3.18146  2.89801
2.37887  2.77519  2.98518  4.58477  3.61503
          0.03133  3.87472  4.59707  0.61958  0.77255  0.48576  0.95510
    568   2.31597  4.23644  3.20823  3.02400  4.07846  2.96628  4.13384
3.60134  3.09793  3.32136  4.24719  3.24849  3.70524  3.44704  3.38396
1.08767  2.79625  3.13595  5.45075  4.16198    570 s - - -
          2.68618  4.42225  2.77519  2.73123  3.46354  2.40513  3.72494
3.29354  2.67741  2.69355  4.24690  2.90347  2.73739  3.18146  2.89801
2.37887  2.77519  2.98518  4.58477  3.61503
          0.03133  3.87472  4.59707  0.61958  0.77255  0.48576  0.95510
    569   2.88236  4.86831  2.90423  2.65889  4.03720  3.36741  3.81726
3.66821  2.47950  3.17034  4.19804  3.11869  3.92083  1.23004  2.76368
2.92996  3.17928  3.39771  5.30876  4.00263    571 q - - -
          2.68618  4.42225  2.77519  2.73123  3.46354  2.40513  3.72494
3.29354  2.67741  2.69355  4.24690  2.90347  2.73739  3.18146  2.89801
2.37887  2.77519  2.98518  4.58477  3.61503
          0.03133  3.87472  4.59707  0.61958  0.77255  0.48576  0.95510
    570   2.76373  5.04934  2.10028  2.21042  4.39780  3.17410  3.75131
3.95796  2.70972  3.54314  4.41213  1.50956  3.79007  2.94328  3.21050
2.73231  3.08192  3.56107  5.65531  4.22364    572 n - - -
          2.68618  4.42225  2.77519  2.73123  3.46354  2.40513  3.72494
3.29354  2.67741  2.69355  4.24690  2.90347  2.73739  3.18146  2.89801
2.37887  2.77519  2.98518  4.58477  3.61503
          0.03133  3.87472  4.59707  0.61958  0.77255  0.48576  0.95510
    571   2.31597  4.23644  3.20823  3.02400  4.07846  2.96628  4.13384
3.60134  3.09793  3.32136  4.24719  3.24849  3.70524  3.44704  3.38396
1.08767  2.79625  3.13595  5.45075  4.16198    573 s - - -
          2.68618  4.42225  2.77519  2.73123  3.46354  2.40513  3.72494
3.29354  2.67741  2.69355  4.24690  2.90347  2.73739  3.18146  2.89801
2.37887  2.77519  2.98518  4.58477  3.61503
          0.03133  3.87472  4.59707  0.61958  0.77255  0.48576  0.95510
    572   2.66752  4.44283  3.34177  3.24141  4.38386  0.72564  4.36163
3.98394  3.42300  3.63781  4.61350  3.50605  3.83404  3.74696  3.65888
2.83948  3.14893  3.51182  5.47850  4.48580    574 g - - -
          2.68618  4.42225  2.77519  2.73123  3.46354  2.40513  3.72494
3.29354  2.67741  2.69355  4.24690  2.90347  2.73739  3.18146  2.89801
2.37887  2.77519  2.98518  4.58477  3.61503
          0.03133  3.87472  4.59707  0.61958  0.77255  0.48576  0.95510
    573   2.72515  4.72193  2.70930  2.59495  4.08579  3.18536  3.92257
3.79656  2.82640  3.44315  4.39831  1.15407  3.83729  3.19525  3.18120
2.79220  3.10703  3.41854  5.39143  4.03390    575 n - - -
```

```
        2.68618  4.42225  2.77519  2.73123  3.46354  2.40513  3.72494
3.29354  2.67741  2.69355  4.24690  2.90347  2.73739  3.18146  2.89801
2.37887  2.77519  2.98518  4.58477  3.61503
        0.03133  3.87472  4.59707  0.61958  0.77255  0.48576  0.95510
    574  2.72515  4.72193  2.70930  2.59495  4.08579  3.18536  3.92257
3.79656  2.82640  3.44315  4.39831  1.15407  3.83729  3.19525  3.18120
2.79220  3.10703  3.41854  5.39143  4.03390    576 n - - -
        2.68618  4.42225  2.77519  2.73123  3.46354  2.40513  3.72494
3.29354  2.67741  2.69355  4.24690  2.90347  2.73739  3.18146  2.89801
2.37887  2.77519  2.98518  4.58477  3.61503
        0.03133  3.87472  4.59707  0.61958  0.77255  0.48576  0.95510
    575  2.97042  5.03658  3.36975  2.75160  4.45095  3.58742  3.58568
3.81539  1.64665  3.29651  4.18095  3.15265  3.97676  2.73241  1.42580
2.98437  3.15278  3.50961  5.36671  4.18791    577 r - - -
        2.68618  4.42225  2.77519  2.73123  3.46354  2.40513  3.72494
3.29354  2.67741  2.69355  4.24690  2.90347  2.73739  3.18146  2.89801
2.37887  2.77519  2.98518  4.58477  3.61503
        0.03133  3.87472  4.59707  0.61958  0.77255  0.48576  0.95510
    576  3.12595  4.55548  4.42280  3.88366  3.04497  4.25029  4.54412
2.17463  3.68325  1.17096  2.02793  4.18871  4.51787  3.94082  3.87635
3.59802  3.36223  2.25546  5.04224  3.88701    578 l - - -
        2.68618  4.42225  2.77519  2.73123  3.46354  2.40513  3.72494
3.29354  2.67741  2.69355  4.24690  2.90347  2.73739  3.18146  2.89801
2.37887  2.77519  2.98518  4.58477  3.61503
        0.03133  3.87472  4.59707  0.61958  0.77255  0.48576  0.95510
    577  3.15771  4.61926  4.19314  3.83291  3.13642  3.98506  4.48794
2.34049  3.60064  0.87242  3.15851  4.10818  4.41998  3.96843  3.77638
3.55606  3.45182  2.37843  5.01661  3.74048    579 l - - -
        2.68618  4.42225  2.77519  2.73123  3.46354  2.40513  3.72494
3.29354  2.67741  2.69355  4.24690  2.90347  2.73739  3.18146  2.89801
2.37887  2.77519  2.98518  4.58477  3.61503
        0.03133  3.87472  4.59707  0.61958  0.77255  0.48576  0.95510
    578  2.66752  4.44283  3.34177  3.24141  4.38386  0.72564  4.36163
3.98394  3.42300  3.63781  4.61350  3.50605  3.83404  3.74696  3.65888
2.83948  3.14893  3.51182  5.47850  4.48580    580 g - - -
        2.68618  4.42225  2.77519  2.73123  3.46354  2.40513  3.72494
3.29354  2.67741  2.69355  4.24690  2.90347  2.73739  3.18146  2.89801
2.37887  2.77519  2.98518  4.58477  3.61503
        0.03133  3.87472  4.59707  0.61958  0.77255  0.48576  0.95510
    579  2.22146  4.18839  3.11012  2.90581  4.27179  1.77987  4.09370
3.73831  3.06346  3.41077  4.24455  3.15053  3.64007  3.35557  3.39718
1.62230  2.70183  3.18739  5.59327  4.34740    581 s - - -
        2.68618  4.42225  2.77519  2.73123  3.46354  2.40513  3.72494
3.29354  2.67741  2.69355  4.24690  2.90347  2.73739  3.18146  2.89801
2.37887  2.77519  2.98518  4.58477  3.61503
        0.03133  3.87472  4.59707  0.61958  0.77255  0.48576  0.95510
    580  3.05456  4.43781  4.48293  3.97525  2.01718  4.19721  4.33431
1.59407  3.85097  1.71009  3.05698  4.17022  4.49766  4.02613  4.01333
3.54830  3.29830  2.09185  4.71110  3.30029    582 i - - -
        2.68618  4.42225  2.77519  2.73123  3.46354  2.40513  3.72494
3.29354  2.67741  2.69355  4.24690  2.90347  2.73739  3.18146  2.89801
2.37887  2.77519  2.98518  4.58477  3.61503
        0.03133  3.87472  4.59707  0.61958  0.77255  0.48576  0.95510
```

```
    581   2.32630  4.26218  3.21111  2.88787  4.04571  3.04818  3.99393
3.34664  2.88524  3.08377  3.98987  3.18311  2.09500  3.24689  3.21583
2.48828  1.86301  2.96127  5.40963  4.16587    583 t - - -
          2.68618  4.42225  2.77519  2.73123  3.46354  2.40513  3.72494
3.29354  2.67741  2.69355  4.24690  2.90347  2.73739  3.18146  2.89801
2.37887  2.77519  2.98518  4.58477  3.61503
          0.03133  3.87472  4.59707  0.61958  0.77255  0.48576  0.95510
    582   1.78770  4.18601  3.80221  3.40442  3.70854  3.33953  4.30292
2.34520  3.32725  2.51429  3.61910  3.58899  3.96611  3.64981  3.59712
2.76134  2.86369  1.57341  5.32856  4.10240    584 v - - -
          2.68618  4.42225  2.77519  2.73123  3.46354  2.40513  3.72494
3.29354  2.67741  2.69355  4.24690  2.90347  2.73739  3.18146  2.89801
2.37887  2.77519  2.98518  4.58477  3.61503
          0.03133  3.87472  4.59707  0.61958  0.77255  0.48576  0.95510
    583   2.80737  5.17108  2.30067  1.62634  4.48641  3.22085  3.69493
3.97416  2.56646  3.52876  4.38432  1.95238  3.80104  2.86502  3.03680
2.75103  3.08980  3.59188  5.68112  4.26238    585 e - - -
          2.68618  4.42225  2.77519  2.73123  3.46354  2.40513  3.72494
3.29354  2.67741  2.69355  4.24690  2.90347  2.73739  3.18146  2.89801
2.37887  2.77519  2.98518  4.58477  3.61503
          0.03133  3.87472  4.59707  0.61958  0.77255  0.48576  0.95510
    584   1.51825  4.13658  3.26063  3.03549  4.22837  1.75663  4.15612
3.57277  3.14577  3.32187  4.18341  3.22451  3.64576  3.43696  3.45398
2.37951  2.68894  3.06947  5.57160  4.36285    586 a - - -
          2.68618  4.42225  2.77519  2.73123  3.46354  2.40513  3.72494
3.29354  2.67741  2.69355  4.24690  2.90347  2.73739  3.18146  2.89801
2.37887  2.77519  2.98518  4.58477  3.61503
          0.03133  3.87472  4.59707  0.61958  0.77255  0.48576  0.95510
    585   2.72515  4.72193  2.70930  2.59495  4.08579  3.18536  3.92257
3.79656  2.82640  3.44315  4.39831  1.15407  3.83729  3.19525  3.18120
2.79220  3.10703  3.41854  5.39143  4.03390    587 n - - -
          2.68618  4.42225  2.77519  2.73123  3.46354  2.40513  3.72494
3.29354  2.67741  2.69355  4.24690  2.90347  2.73739  3.18146  2.89801
2.37887  2.77519  2.98518  4.58477  3.61503
          0.03133  3.87472  4.59707  0.61958  0.77255  0.48576  0.95510
    586   2.29066  4.26619  3.09152  2.82123  4.15546  2.98297  3.99018
3.60389  2.90028  3.27334  4.12730  3.12364  2.12398  3.23519  3.24758
1.66406  2.73311  3.13100  5.48492  4.21511    588 s - - -
          2.68618  4.42225  2.77519  2.73123  3.46354  2.40513  3.72494
3.29354  2.67741  2.69355  4.24690  2.90347  2.73739  3.18146  2.89801
2.37887  2.77519  2.98518  4.58477  3.61503
          0.03133  3.87472  4.59707  0.61958  0.77255  0.48576  0.95510
    587   2.72459  4.92283  2.44214  1.65892  4.40556  1.97373  3.76999
3.85506  2.66252  3.45202  4.31666  2.83337  3.79342  2.95761  3.11490
2.72522  3.04264  3.46954  5.61301  4.27009    589 e - - -
          2.68618  4.42225  2.77519  2.73123  3.46354  2.40513  3.72494
3.29354  2.67741  2.69355  4.24690  2.90347  2.73739  3.18146  2.89801
2.37887  2.77519  2.98518  4.58477  3.61503
          0.03133  3.87472  4.59707  0.61958  0.77255  0.48576  0.95510
    588   3.33912  4.71305  4.09447  3.80105  2.66830  3.73256  3.90238
3.36273  3.52318  2.75727  3.99643  3.94963  4.25045  3.91067  3.65116
3.54280  3.63961  3.25312  0.88077  2.67027    590 w - - -
          2.68618  4.42225  2.77519  2.73123  3.46354  2.40513  3.72494
3.29354  2.67741  2.69355  4.24690  2.90347  2.73739  3.18146  2.89801
2.37887  2.77519  2.98518  4.58477  3.61503
```

```
         0.03133  3.87472  4.59707  0.61958  0.77255  0.48576  0.95510
    589  2.93646  4.35924  4.42245  3.94955  3.47391  4.10922  4.71836
1.69140  3.81003  2.04078  3.33480  4.19022  4.51304  4.12508  4.04015
3.50279  3.22859  1.12621  5.37160  4.12959    591 v - - -
         2.68618  4.42225  2.77519  2.73123  3.46354  2.40513  3.72494
3.29354  2.67741  2.69355  4.24690  2.90347  2.73739  3.18146  2.89801
2.37887  2.77519  2.98518  4.58477  3.61503
         0.03133  3.87472  4.59707  0.61958  0.77255  0.48576  0.95510
    590  2.99187  5.12073  0.91483  2.33607  4.49801  3.21224  3.93281
4.08666  3.01732  3.69455  4.67262  2.89258  3.86518  3.17190  3.53391
2.95391  3.33046  3.72558  5.66619  4.37043    592 d - - -
         2.68618  4.42225  2.77519  2.73123  3.46354  2.40513  3.72494
3.29354  2.67741  2.69355  4.24690  2.90347  2.73739  3.18146  2.89801
2.37887  2.77519  2.98518  4.58477  3.61503
         0.03133  3.87472  4.59707  0.61958  0.77255  0.48576  0.95510
    591  3.38852  4.71189  4.36458  3.99706  1.40423  4.19048  3.44249
3.10909  3.86117  2.48342  3.72613  3.90357  4.51714  3.91931  3.96352
3.54726  3.61700  3.02797  3.62301  1.34887    593 y - - -
         2.68618  4.42225  2.77519  2.73123  3.46354  2.40513  3.72494
3.29354  2.67741  2.69355  4.24690  2.90347  2.73739  3.18146  2.89801
2.37887  2.77519  2.98518  4.58477  3.61503
         0.03133  3.87472  4.59707  0.61958  0.77255  0.48576  0.95510
    592  3.00216  4.36665  4.57319  4.07815  3.46025  4.26584  4.82000
1.36984  3.95490  1.97965  3.28692  4.32195  4.60862  4.23687  4.17224
3.64730  3.27408  1.30447  5.39903  4.18788    594 v - - -
         2.68618  4.42225  2.77519  2.73123  3.46354  2.40513  3.72494
3.29354  2.67741  2.69355  4.24690  2.90347  2.73739  3.18146  2.89801
2.37887  2.77519  2.98518  4.58477  3.61503
         0.03133  3.87472  4.59707  0.61958  0.77255  0.48576  0.95510
    593  2.41377  4.25888  3.42992  3.18193  3.97070  3.09811  4.19910
3.09810  3.12495  2.96152  4.01182  3.39101  3.80201  3.51964  3.38358
2.61505  1.16810  2.79284  5.40831  4.18731    595 t - - -
         2.68618  4.42225  2.77519  2.73123  3.46354  2.40513  3.72494
3.29354  2.67741  2.69355  4.24690  2.90347  2.73739  3.18146  2.89801
2.37887  2.77519  2.98518  4.58477  3.61503
         0.03133  3.87472  4.59707  0.61958  0.77255  0.48576  0.95510
    594  2.99187  5.12073  0.91483  2.33607  4.49801  3.21224  3.93281
4.08666  3.01732  3.69455  4.67262  2.89258  3.86518  3.17190  3.53391
2.95391  3.33046  3.72558  5.66619  4.37043    596 d - - -
         2.68618  4.42225  2.77519  2.73123  3.46354  2.40513  3.72494
3.29354  2.67741  2.69355  4.24690  2.90347  2.73739  3.18146  2.89801
2.37887  2.77519  2.98518  4.58477  3.61503
         0.03133  3.87472  4.59707  0.61958  0.77255  0.48576  0.95510
    595  1.67886  4.11855  3.27582  2.99514  4.21411  2.50880  4.10348
3.61394  3.07837  3.31059  4.13828  3.19093  3.62272  3.36663  3.40634
1.59968  2.64484  3.08486  5.55451  4.33179    597 s - - -
         2.68618  4.42225  2.77519  2.73123  3.46354  2.40513  3.72494
3.29354  2.67741  2.69355  4.24690  2.90347  2.73739  3.18146  2.89801
2.37887  2.77519  2.98518  4.58477  3.61503
         0.03133  3.87472  4.59707  0.61958  0.77255  0.48576  0.95510
    596  3.26109  4.66217  4.24182  3.95666  1.00804  3.95049  3.87804
2.78442  3.86362  2.15392  3.51419  4.02166  4.40896  4.03095  3.98279
3.53096  3.56220  2.77530  4.12630  2.49298    598 f - - -
```

```
          2.68618  4.42225  2.77519  2.73123  3.46354  2.40513  3.72494
3.29354  2.67741  2.69355  4.24690  2.90347  2.73739  3.18146  2.89801
2.37887  2.77519  2.98518  4.58477  3.61503
          0.03133  3.87472  4.59707  0.61958  0.77255  0.48576  0.95510
    597   2.41377  4.25888  3.42992  3.18193  3.97070  3.09811  4.19910
3.09810  3.12495  2.96152  4.01182  3.39101  3.80201  3.51964  3.38358
2.61505  1.16810  2.79284  5.40831  4.18731     599 t - - -
          2.68618  4.42225  2.77519  2.73123  3.46354  2.40513  3.72494
3.29354  2.67741  2.69355  4.24690  2.90347  2.73739  3.18146  2.89801
2.37887  2.77519  2.98518  4.58477  3.61503
          0.03133  3.87472  4.59707  0.61958  0.77255  0.48576  0.95510
    598   3.26109  4.66217  4.24182  3.95666  1.00804  3.95049  3.87804
2.78442  3.86362  2.15392  3.51419  4.02166  4.40896  4.03095  3.98279
3.53096  3.56220  2.77530  4.12630  2.49298     600 f - - -
          2.68618  4.42225  2.77519  2.73123  3.46354  2.40513  3.72494
3.29354  2.67741  2.69355  4.24690  2.90347  2.73739  3.18146  2.89801
2.37887  2.77519  2.98518  4.58477  3.61503
          0.03133  3.87472  4.59707  0.61958  0.77255  0.48576  0.95510
    599   2.79281  5.16311  1.88289  2.14880  4.49396  3.17296  3.72914
4.04176  2.70456  3.60065  4.45880  1.64015  3.78543  2.90947  3.23898
2.73533  3.09926  3.63563  5.72930  4.27548     601 n - - -
          2.68618  4.42225  2.77519  2.73123  3.46354  2.40513  3.72494
3.29354  2.67741  2.69355  4.24690  2.90347  2.73739  3.18146  2.89801
2.37887  2.77519  2.98518  4.58477  3.61503
          0.03133  3.87472  4.59707  0.61958  0.77255  0.48576  0.95510
    600   2.99187  5.12073  0.91483  2.33607  4.49801  3.21224  3.93281
4.08666  3.01732  3.69455  4.67262  2.89258  3.86518  3.17190  3.53391
2.95391  3.33046  3.72558  5.66619  4.37043     602 d - - -
          2.68618  4.42225  2.77519  2.73123  3.46354  2.40513  3.72494
3.29354  2.67741  2.69355  4.24690  2.90347  2.73739  3.18146  2.89801
2.37887  2.77519  2.98518  4.58477  3.61503
          0.03133  3.87472  4.59707  0.61958  0.77255  0.48576  0.95510
    601   3.15771  4.61926  4.19314  3.83291  3.13642  3.98506  4.48794
2.34049  3.60064  0.87242  3.15851  4.10818  4.41998  3.96843  3.77638
3.55606  3.45182  2.37843  5.01661  3.74048     603 l - - -
          2.68618  4.42225  2.77519  2.73123  3.46354  2.40513  3.72494
3.29354  2.67741  2.69355  4.24690  2.90347  2.73739  3.18146  2.89801
2.37887  2.77519  2.98518  4.58477  3.61503
          0.03133  3.87472  4.59707  0.61958  0.77255  0.48576  0.95510
    602   2.66752  4.44283  3.34177  3.24141  4.38386  0.72564  4.36163
3.98394  3.42300  3.63781  4.61350  3.50605  3.83404  3.74696  3.65888
2.83948  3.14893  3.51182  5.47850  4.48580     604 g - - -
          2.68618  4.42225  2.77519  2.73123  3.46354  2.40513  3.72494
3.29354  2.67741  2.69355  4.24690  2.90347  2.73739  3.18146  2.89801
2.37887  2.77519  2.98518  4.58477  3.61503
          0.03133  3.87472  4.59707  0.61958  0.77255  0.48576  0.95510
    603   3.03284  4.45083  4.33904  3.95485  3.37561  4.06909  4.68249
1.07512  3.78303  1.96109  3.31052  4.19987  4.50000  4.13104  3.97934
3.56453  3.33193  1.84577  5.26697  4.00552     605 i - - -
          2.68618  4.42225  2.77519  2.73123  3.46354  2.40513  3.72494
3.29354  2.67741  2.69355  4.24690  2.90347  2.73739  3.18146  2.89801
2.37887  2.77519  2.98518  4.58477  3.61503
          0.03133  3.87472  4.59707  0.61958  0.77255  0.48576  0.95510
```

```
    604   2.41377  4.25888  3.42992  3.18193  3.97070  3.09811  4.19910
3.09810  3.12495  2.96152  4.01182  3.39101  3.80201  3.51964  3.38358
2.61505  1.16810  2.79284  5.40831  4.18731    606 t - - -
         2.68618  4.42225  2.77519  2.73123  3.46354  2.40513  3.72494
3.29354  2.67741  2.69355  4.24690  2.90347  2.73739  3.18146  2.89801
2.37887  2.77519  2.98518  4.58477  3.61503
         0.03133  3.87472  4.59707  0.61958  0.77255  0.48576  0.95510
    605   2.41377  4.25888  3.42992  3.18193  3.97070  3.09811  4.19910
3.09810  3.12495  2.96152  4.01182  3.39101  3.80201  3.51964  3.38358
2.61505  1.16810  2.79284  5.40831  4.18731    607 t - - -
         2.68618  4.42225  2.77519  2.73123  3.46354  2.40513  3.72494
3.29354  2.67741  2.69355  4.24690  2.90347  2.73739  3.18146  2.89801
2.37887  2.77519  2.98518  4.58477  3.61503
         0.03133  3.87472  4.59707  0.61958  0.77255  0.48576  0.95510
    606   1.79356  4.12842  3.28686  3.03141  4.17522  2.90136  4.12712
3.52086  3.09086  3.27559  4.14110  3.21714  3.64348  3.40321  3.39812
1.39068  2.67092  3.03115  5.54192  4.30404    608 s - - -
         2.68618  4.42225  2.77519  2.73123  3.46354  2.40513  3.72494
3.29354  2.67741  2.69355  4.24690  2.90347  2.73739  3.18146  2.89801
2.37887  2.77519  2.98518  4.58477  3.61503
         0.03133  3.87472  4.59707  0.61958  0.77255  0.48576  0.95510
    607   2.31597  4.23644  3.20823  3.02400  4.07846  2.96628  4.13384
3.60134  3.09793  3.32136  4.24719  3.24849  3.70524  3.44704  3.38396
1.08767  2.79625  3.13595  5.45075  4.16198    609 s - - -
         2.68618  4.42225  2.77519  2.73123  3.46354  2.40513  3.72494
3.29354  2.67741  2.69355  4.24690  2.90347  2.73739  3.18146  2.89801
2.37887  2.77519  2.98518  4.58477  3.61503
         0.03133  3.87472  4.59707  0.61958  0.77255  0.48576  0.95510
    608   2.41377  4.25888  3.42992  3.18193  3.97070  3.09811  4.19910
3.09810  3.12495  2.96152  4.01182  3.39101  3.80201  3.51964  3.38358
2.61505  1.16810  2.79284  5.40831  4.18731    610 t - - -
         2.68618  4.42225  2.77519  2.73123  3.46354  2.40513  3.72494
3.29354  2.67741  2.69355  4.24690  2.90347  2.73739  3.18146  2.89801
2.37887  2.77519  2.98518  4.58477  3.61503
         0.03133  3.87472  4.59707  0.61958  0.77255  0.48576  0.95510
    609   2.72515  4.72193  2.70930  2.59495  4.08579  3.18536  3.92257
3.79656  2.82640  3.44315  4.39831  1.15407  3.83729  3.19525  3.18120
2.79220  3.10703  3.41854  5.39143  4.03390    611 n - - -
         2.68618  4.42225  2.77519  2.73123  3.46354  2.40513  3.72494
3.29354  2.67741  2.69355  4.24690  2.90347  2.73739  3.18146  2.89801
2.37887  2.77519  2.98518  4.58477  3.61503
         0.03133  3.87472  4.59707  0.61958  0.77255  0.48576  0.95510
    610   1.01052  4.19303  3.46476  3.25606  4.05451  3.01753  4.27786
3.18035  3.27238  3.06187  4.09337  3.40514  3.75695  3.61331  3.52281
2.54327  2.82077  2.83436  5.48144  4.28192    612 a - - -
         2.68618  4.42225  2.77519  2.73123  3.46354  2.40513  3.72494
3.29354  2.67741  2.69355  4.24690  2.90347  2.73739  3.18146  2.89801
2.37887  2.77519  2.98518  4.58477  3.61503
         0.03133  3.87472  4.59707  0.61958  0.77255  0.48576  0.95510
    611   3.18394  4.57158  4.46817  3.98536  1.81739  4.24176  4.17394
2.32886  3.84356  1.26758  3.01821  4.16005  4.52633  3.99840  3.99490
3.60398  3.42132  2.39767  4.47188  2.96978    613 l - - -
         2.68618  4.42225  2.77519  2.73123  3.46354  2.40513  3.72494
3.29354  2.67741  2.69355  4.24690  2.90347  2.73739  3.18146  2.89801
2.37887  2.77519  2.98518  4.58477  3.61503
```

```
        0.03133  3.87472  4.59707  0.61958  0.77255  0.48576  0.95510
    612  3.26109  4.66217  4.24182  3.95666  1.00804  3.95049  3.87804
2.78442  3.86362  2.15392  3.51419  4.02166  4.40896  4.03095  3.98279
3.53096  3.56220  2.77530  4.12630  2.49298    614 f - - -
        2.68618  4.42225  2.77519  2.73123  3.46354  2.40513  3.72494
3.29354  2.67741  2.69355  4.24690  2.90347  2.73739  3.18146  2.89801
2.37887  2.77519  2.98518  4.58477  3.61503
        0.03133  3.87472  4.59707  0.61958  0.77255  0.48576  0.95510
    613  2.31597  4.23644  3.20823  3.02400  4.07846  2.96628  4.13384
3.60134  3.09793  3.32136  4.24719  3.24849  3.70524  3.44704  3.38396
1.08767  2.79625  3.13595  5.45075  4.16198    615 s - - -
        2.68618  4.42225  2.77519  2.73123  3.46354  2.40513  3.72494
3.29354  2.67741  2.69355  4.24690  2.90347  2.73739  3.18146  2.89801
2.37887  2.77519  2.98518  4.58477  3.61503
        0.03133  3.87472  4.59707  0.61958  0.77255  0.48576  0.95510
    614  3.03284  4.45083  4.33904  3.95485  3.37561  4.06909  4.68249
1.07512  3.78303  1.96109  3.31052  4.19987  4.50000  4.13104  3.97934
3.56453  3.33193  1.84577  5.26697  4.00552    616 i - - -
        2.68618  4.42225  2.77519  2.73123  3.46354  2.40513  3.72494
3.29354  2.67741  2.69355  4.24690  2.90347  2.73739  3.18146  2.89801
2.37887  2.77519  2.98518  4.58477  3.61503
        0.03133  3.87472  4.59707  0.61958  0.77255  0.48576  0.95510
    615  2.64807  4.89688  1.71962  2.24756  4.39979  3.13921  3.75772
3.88649  2.71858  3.48583  4.33356  2.77878  3.75567  2.94056  3.23004
1.89458  2.98428  3.46853  5.65988  4.24825    617 d - - -
        2.68618  4.42225  2.77519  2.73123  3.46354  2.40513  3.72494
3.29354  2.67741  2.69355  4.24690  2.90347  2.73739  3.18146  2.89801
2.37887  2.77519  2.98518  4.58477  3.61503
        0.03133  3.87472  4.59707  0.61958  0.77255  0.48576  0.95510
    616  2.31597  4.23644  3.20823  3.02400  4.07846  2.96628  4.13384
3.60134  3.09793  3.32136  4.24719  3.24849  3.70524  3.44704  3.38396
1.08767  2.79625  3.13595  5.45075  4.16198    618 s - - -
        2.68618  4.42225  2.77519  2.73123  3.46354  2.40513  3.72494
3.29354  2.67741  2.69355  4.24690  2.90347  2.73739  3.18146  2.89801
2.37887  2.77519  2.98518  4.58477  3.61503
        0.03133  3.87472  4.59707  0.61958  0.77255  0.48576  0.95510
    617  2.99187  5.12073  0.91483  2.33607  4.49801  3.21224  3.93281
4.08666  3.01732  3.69455  4.67262  2.89258  3.86518  3.17190  3.53391
2.95391  3.33046  3.72558  5.66619  4.37043    619 d - - -
        2.68618  4.42225  2.77519  2.73123  3.46354  2.40513  3.72494
3.29354  2.67741  2.69355  4.24690  2.90347  2.73739  3.18146  2.89801
2.37887  2.77519  2.98518  4.58477  3.61503
        0.03133  3.87472  4.59707  0.61958  0.77255  0.48576  0.95510
    618  2.22146  4.18839  3.11012  2.90581  4.27179  1.77987  4.09370
3.73831  3.06346  3.41077  4.24455  3.15053  3.64007  3.35557  3.39718
1.62230  2.70183  3.18739  5.59327  4.34740    620 s - - -
        2.68618  4.42225  2.77519  2.73123  3.46354  2.40513  3.72494
3.29354  2.67741  2.69355  4.24690  2.90347  2.73739  3.18146  2.89801
2.37887  2.77519  2.98518  4.58477  3.61503
        0.03133  3.87472  4.59707  0.61958  0.77255  0.48576  0.95510
    619  2.98631  4.42233  4.37089  3.87504  3.27174  4.12445  4.59189
1.95970  3.70975  1.42739  3.15091  4.14292  4.48500  4.00662  3.92688
3.50014  3.25865  1.45423  5.18982  3.96810    621 l - - -
```

```
          2.68618  4.42225  2.77519  2.73123  3.46354  2.40513  3.72494
 3.29354  2.67741  2.69355  4.24690  2.90347  2.73739  3.18146  2.89801
 2.37887  2.77519  2.98518  4.58477  3.61503
          0.03133  3.87472  4.59707  0.61958  0.77255  0.48576  0.95510
     620  2.72515  4.72193  2.70930  2.59495  4.08579  3.18536  3.92257
 3.79656  2.82640  3.44315  4.39831  1.15407  3.83729  3.19525  3.18120
 2.79220  3.10703  3.41854  5.39143  4.03390    622 n - - -
          2.68618  4.42225  2.77519  2.73123  3.46354  2.40513  3.72494
 3.29354  2.67741  2.69355  4.24690  2.90347  2.73739  3.18146  2.89801
 2.37887  2.77519  2.98518  4.58477  3.61503
          0.03133  3.87472  4.59707  0.61958  0.77255  0.48576  0.95510
     621  1.50879  4.11123  3.33050  3.05929  4.15834  2.89943  4.13453
 3.49003  3.10781  3.25178  4.11504  3.23094  3.64092  3.41443  3.41277
 1.63872  2.66000  3.00580  5.52886  4.29979    623 a - - -
          2.68618  4.42225  2.77519  2.73123  3.46354  2.40513  3.72494
 3.29354  2.67741  2.69355  4.24690  2.90347  2.73739  3.18146  2.89801
 2.37887  2.77519  2.98518  4.58477  3.61503
          0.03133  3.87472  4.59707  0.61958  0.77255  0.48576  0.95510
     622  2.22103  4.18821  3.11256  2.90573  4.27031  1.83408  4.09219
 3.73644  3.06097  3.40859  4.24202  3.15053  3.63994  3.35354  3.39511
 1.58085  2.70094  3.18609  5.59176  4.34580    624 s - - -
          2.68618  4.42225  2.77519  2.73123  3.46354  2.40513  3.72494
 3.29354  2.67741  2.69355  4.24690  2.90347  2.73739  3.18146  2.89801
 2.37887  2.77519  2.98518  4.58477  3.61503
          0.03133  3.87472  4.59707  0.61958  0.77255  0.48576  0.95510
     623  2.80826  5.13391  2.47113  1.74377  4.41976  3.29985  3.65014
 3.87894  2.38270  3.40687  4.26697  2.80430  3.82402  1.98888  2.77915
 2.76478  3.06050  3.52472  5.57169  4.20330    625 e - - -
          2.68618  4.42225  2.77519  2.73123  3.46354  2.40513  3.72494
 3.29354  2.67741  2.69355  4.24690  2.90347  2.73739  3.18146  2.89801
 2.37887  2.77519  2.98518  4.58477  3.61503
          0.03133  3.87472  4.59707  0.61958  0.77255  0.48576  0.95510
     624  2.80826  5.13391  2.47113  1.74377  4.41976  3.29985  3.65014
 3.87894  2.38270  3.40687  4.26697  2.80430  3.82402  1.98888  2.77915
 2.76478  3.06050  3.52472  5.57169  4.20330    626 e - - -
          2.68618  4.42225  2.77519  2.73123  3.46354  2.40513  3.72494
 3.29354  2.67741  2.69355  4.24690  2.90347  2.73739  3.18146  2.89801
 2.37887  2.77519  2.98518  4.58477  3.61503
          0.03133  3.87472  4.59707  0.61958  0.77255  0.48576  0.95510
     625  2.83905  4.46713  3.67180  3.29688  2.99682  2.70958  3.87345
 3.21149  3.10528  2.81034  3.83548  3.56170  4.02483  3.50930  3.33156
 3.04123  3.15503  2.98555  1.33919  3.02549    627 w - - -
          2.68618  4.42225  2.77519  2.73123  3.46354  2.40513  3.72494
 3.29354  2.67741  2.69355  4.24690  2.90347  2.73739  3.18146  2.89801
 2.37887  2.77519  2.98518  4.58477  3.61503
          0.03133  3.87472  4.59707  0.61958  0.77255  0.48576  0.95510
     626  3.25019  4.73827  3.84149  3.57122  2.29338  3.83270  3.61936
 3.25908  3.42867  2.71244  3.93385  3.72612  4.30645  3.73417  3.61195
 3.38669  3.53686  3.12972  3.93726  0.96205    628 y - - -
          2.68618  4.42225  2.77519  2.73123  3.46354  2.40513  3.72494
 3.29354  2.67741  2.69355  4.24690  2.90347  2.73739  3.18146  2.89801
 2.37887  2.77519  2.98518  4.58477  3.61503
          0.03133  3.87472  4.59707  0.61958  0.77255  0.48576  0.95510
```

627 3.15771 4.61926 4.19314 3.83291 3.13642 3.98506 4.48794
2.34049 3.60064 0.87242 3.15851 4.10818 4.41998 3.96843 3.77638
3.55606 3.45182 2.37843 5.01661 3.74048 629 l - - -
2.68618 4.42225 2.77519 2.73123 3.46354 2.40513 3.72494
3.29354 2.67741 2.69355 4.24690 2.90347 2.73739 3.18146 2.89801
2.37887 2.77519 2.98518 4.58477 3.61503
0.03133 3.87472 4.59707 0.61958 0.77255 0.48576 0.95510
628 2.31597 4.23644 3.20823 3.02400 4.07846 2.96628 4.13384
3.60134 3.09793 3.32136 4.24719 3.24849 3.70524 3.44704 3.38396
1.08767 2.79625 3.13595 5.45075 4.16198 630 s - - -
2.68618 4.42225 2.77519 2.73123 3.46354 2.40513 3.72494
3.29354 2.67741 2.69355 4.24690 2.90347 2.73739 3.18146 2.89801
2.37887 2.77519 2.98518 4.58477 3.61503
0.03133 3.87472 4.59707 0.61958 0.77255 0.48576 0.95510
629 2.86996 5.05712 2.95165 2.52547 4.40412 3.46665 3.59634
3.81846 1.58986 3.31355 4.18129 2.99727 3.90252 2.01235 2.31441
2.86066 3.08071 3.49111 5.42333 4.16317 631 k - - -
2.68618 4.42225 2.77519 2.73123 3.46354 2.40513 3.72494
3.29354 2.67741 2.69355 4.24690 2.90347 2.73739 3.18146 2.89801
2.37887 2.77519 2.98518 4.58477 3.61503
0.03133 3.87472 4.59707 0.61958 0.77255 0.48576 0.95510
630 3.15771 4.61926 4.19314 3.83291 3.13642 3.98506 4.48794
2.34049 3.60064 0.87242 3.15851 4.10818 4.41998 3.96843 3.77638
3.55606 3.45182 2.37843 5.01661 3.74048 632 l - - -
2.68618 4.42225 2.77519 2.73123 3.46354 2.40513 3.72494
3.29354 2.67741 2.69355 4.24690 2.90347 2.73739 3.18146 2.89801
2.37887 2.77519 2.98518 4.58477 3.61503
0.03133 3.87472 4.59707 0.61958 0.77255 0.48576 0.95510
631 3.05456 4.43781 4.48293 3.97525 2.01718 4.19721 4.33431
1.59407 3.85097 1.71009 3.05698 4.17022 4.49766 4.02613 4.01333
3.54830 3.29830 2.09185 4.71110 3.30029 633 i - - -
2.68618 4.42225 2.77519 2.73123 3.46354 2.40513 3.72494
3.29354 2.67741 2.69355 4.24690 2.90347 2.73739 3.18146 2.89801
2.37887 2.77519 2.98518 4.58477 3.61503
0.03133 3.87472 4.59707 0.61958 0.77255 0.48576 0.95510
632 3.15771 4.61926 4.19314 3.83291 3.13642 3.98506 4.48794
2.34049 3.60064 0.87242 3.15851 4.10818 4.41998 3.96843 3.77638
3.55606 3.45182 2.37843 5.01661 3.74048 634 l - - -
2.68618 4.42225 2.77519 2.73123 3.46354 2.40513 3.72494
3.29354 2.67741 2.69355 4.24690 2.90347 2.73739 3.18146 2.89801
2.37887 2.77519 2.98518 4.58477 3.61503
0.03133 3.87472 4.59707 0.61958 0.77255 0.48576 0.95510
633 2.80983 4.35172 4.12850 3.79161 3.55163 3.72629 4.59291
2.04988 3.65943 2.23820 3.51634 3.98111 4.28661 4.01903 3.87173
3.22990 3.17128 1.02205 5.33648 4.06827 635 v - - -
2.68618 4.42225 2.77519 2.73123 3.46354 2.40513 3.72494
3.29354 2.67741 2.69355 4.24690 2.90347 2.73739 3.18146 2.89801
2.37887 2.77519 2.98518 4.58477 3.61503
0.03133 3.87472 4.59707 0.61958 0.77255 0.48576 0.95510
634 2.97404 4.93809 3.15019 2.77112 4.32503 3.46335 3.76023
3.75442 1.03520 3.31897 4.28021 3.19552 3.96346 2.95151 2.43176
3.01582 3.22709 3.46205 5.37869 4.19175 636 k - - -
2.68618 4.42225 2.77519 2.73123 3.46354 2.40513 3.72494
3.29354 2.67741 2.69355 4.24690 2.90347 2.73739 3.18146 2.89801
2.37887 2.77519 2.98518 4.58477 3.61503

0.03133 3.87472 4.59707 0.61958 0.77255 0.48576 0.95510
635 2.87075 5.35947 1.82529 1.37797 4.64945 3.18531 3.71261
4.14176 2.69835 3.67690 4.53624 2.66562 3.79428 2.88189 3.26068
2.77356 3.15664 3.74011 5.83562 4.36772 637 e - - -
2.68618 4.42225 2.77519 2.73123 3.46354 2.40513 3.72494
3.29354 2.67741 2.69355 4.24690 2.90347 2.73739 3.18146 2.89801
2.37887 2.77519 2.98518 4.58477 3.61503
0.13780 3.87472 2.22593 0.61958 0.77255 0.48576 0.95510
636 2.47502 4.26460 3.39048 2.99138 2.44191 3.28699 3.81883
2.89455 2.96496 2.58302 3.59947 3.28942 3.84866 3.27920 3.27865
1.90151 2.82320 2.65666 4.69418 3.22449 638 s - - -
2.68622 4.42229 2.77523 2.73127 3.46358 2.40516 3.72498
3.29358 2.67745 2.69359 4.24693 2.90303 2.73743 3.18150 2.89804
2.37891 2.77523 2.98496 4.58481 3.61507
0.41979 2.03038 1.55340 0.86733 0.54485 0.43562 1.04090
637 2.23758 4.14860 3.05073 2.86839 3.87940 2.88277 3.97810
3.38006 2.92698 3.12079 4.07355 3.11910 3.60738 3.29590 3.21162
1.43611 2.70471 2.96102 5.27125 3.96408 642 s - - -
2.68618 4.42225 2.77519 2.73123 3.46354 2.40513 3.72494
3.29354 2.67741 2.69355 4.24690 2.90347 2.73739 3.18146 2.89801
2.37887 2.77519 2.98518 4.58477 3.61503
0.03837 3.67537 4.39771 0.61958 0.77255 0.40072 1.10818
638 1.54808 4.11339 3.28070 3.00051 4.21297 2.21858 4.10763
3.61352 3.08917 3.30883 4.13557 3.19293 3.62085 3.37223 3.41748
1.89970 2.64179 3.08305 5.55455 4.33413 643 a - - -
2.68618 4.42225 2.77519 2.73123 3.46354 2.40513 3.72494
3.29354 2.67741 2.69355 4.24690 2.90347 2.73739 3.18146 2.89801
2.37887 2.77519 2.98518 4.58477 3.61503
0.03133 3.87472 4.59707 0.61958 0.77255 0.48576 0.95510
639 3.26109 4.66217 4.24182 3.95666 1.00804 3.95049 3.87804
2.78442 3.86362 2.15392 3.51419 4.02166 4.40896 4.03095 3.98279
3.53096 3.56220 2.77530 4.12630 2.49298 644 f - - -
2.68618 4.42225 2.77519 2.73123 3.46354 2.40513 3.72494
3.29354 2.67741 2.69355 4.24690 2.90347 2.73739 3.18146 2.89801
2.37887 2.77519 2.98518 4.58477 3.61503
0.03133 3.87472 4.59707 0.61958 0.77255 0.48576 0.95510
640 2.47436 4.45795 3.16161 2.70038 3.98953 3.23444 3.76434
3.35600 2.48135 3.02075 3.88336 3.09335 3.77791 2.97061 2.33879
2.38500 1.92766 3.01292 5.27977 4.01845 645 t - - -
2.68618 4.42225 2.77519 2.73123 3.46354 2.40513 3.72494
3.29354 2.67741 2.69355 4.24690 2.90347 2.73739 3.18146 2.89801
2.37887 2.77519 2.98518 4.58477 3.61503
0.03133 3.87472 4.59707 0.61958 0.77255 0.48576 0.95510
641 2.41801 4.40132 2.96548 2.69207 4.06063 3.10529 3.88919
3.41502 2.74661 3.14959 4.03221 2.39577 3.74256 3.12231 3.10059
2.54593 1.67141 3.03678 5.40247 4.10582 646 t - - -
2.68618 4.42225 2.77519 2.73123 3.46354 2.40513 3.72494
3.29354 2.67741 2.69355 4.24690 2.90347 2.73739 3.18146 2.89801
2.37887 2.77519 2.98518 4.58477 3.61503
0.03133 3.87472 4.59707 0.61958 0.77255 0.48576 0.95510
642 2.88236 4.86831 2.90423 2.65889 4.03720 3.36741 3.81726
3.66821 2.47950 3.17034 4.19804 3.11869 3.92083 1.23004 2.76368
2.92996 3.17928 3.39771 5.30876 4.00263 647 q - - -

2.68618 4.42225 2.77519 2.73123 3.46354 2.40513 3.72494
3.29354 2.67741 2.69355 4.24690 2.90347 2.73739 3.18146 2.89801
2.37887 2.77519 2.98518 4.58477 3.61503
0.03133 3.87472 4.59707 0.61958 0.77255 0.48576 0.95510
643 3.01032 4.38075 4.56430 4.07535 3.43460 4.25825 4.81343
1.25130 3.94304 1.94502 3.26584 4.31892 4.60504 4.22798 4.15709
3.64442 3.28439 1.45898 5.38407 4.17226 648 i - - -
2.68618 4.42225 2.77519 2.73123 3.46354 2.40513 3.72494
3.29354 2.67741 2.69355 4.24690 2.90347 2.73739 3.18146 2.89801
2.37887 2.77519 2.98518 4.58477 3.61503
0.03133 3.87472 4.59707 0.61958 0.77255 0.48576 0.95510
644 2.54403 4.62847 2.70593 2.48988 4.19951 3.14304 3.81102
3.70610 2.66714 3.32641 4.18743 1.97409 2.14592 3.01738 3.06601
2.61245 2.90510 3.29796 5.47649 4.14036 649 n - - -
2.68618 4.42225 2.77519 2.73123 3.46354 2.40513 3.72494
3.29354 2.67741 2.69355 4.24690 2.90347 2.73739 3.18146 2.89801
2.37887 2.77519 2.98518 4.58477 3.61503
0.03133 3.87472 4.59707 0.61958 0.77255 0.48576 0.95510
645 2.64837 4.41197 3.45120 3.03435 3.47606 3.45891 3.98612
2.75179 2.90984 1.69449 3.50431 3.39235 2.29131 3.30854 3.20944
2.86031 2.96255 2.60130 5.05271 3.77019 650 l - - -
2.68618 4.42225 2.77519 2.73123 3.46354 2.40513 3.72494
3.29354 2.67741 2.69355 4.24690 2.90347 2.73739 3.18146 2.89801
2.37887 2.77519 2.98518 4.58477 3.61503
0.23773 3.87472 1.65641 0.61958 0.77255 0.48576 0.95510
646 2.06982 4.51006 2.90234 2.57819 3.99701 3.19567 3.71309
3.24980 1.97983 2.98497 3.91108 2.99901 3.75516 2.92027 2.68917
2.62860 2.85054 2.94460 5.28374 4.01395 651 k - - -
2.68618 4.42225 2.77519 2.73123 3.46354 2.40513 3.72494
3.29354 2.67741 2.69355 4.24690 2.90347 2.73739 3.18146 2.89801
2.37887 2.77519 2.98518 4.58477 3.61503
0.03837 3.67537 4.39771 0.61958 0.77255 0.63884 0.75058
647 2.52470 4.30168 3.11257 2.93304 3.92244 3.00090 4.01821
3.42601 2.97460 3.07154 4.12202 3.25385 1.29517 3.37062 3.23327
2.68892 2.95379 3.09390 5.15462 4.04463 652 p - - -
2.68618 4.42225 2.77519 2.73123 3.46354 2.40513 3.72494
3.29354 2.67741 2.69355 4.24690 2.90347 2.73739 3.18146 2.89801
2.37887 2.77519 2.98518 4.58477 3.61503
0.10309 3.67537 2.62255 0.61958 0.77255 0.40072 1.10818
648 2.95137 4.48057 3.90690 3.43329 2.55640 3.87888 3.77705
2.61265 3.24158 1.58141 3.31904 3.67901 4.24402 3.55703 3.47024
3.21851 3.19214 2.54718 4.23995 1.99864 653 l - - -
2.68618 4.42225 2.77519 2.73123 3.46354 2.40513 3.72494
3.29354 2.67741 2.69355 4.24690 2.90347 2.73739 3.18146 2.89801
2.37887 2.77519 2.98518 4.58477 3.61503
0.03298 3.82405 4.54639 0.61958 0.77255 0.52999 0.88821
649 2.95986 4.41679 4.29393 3.80178 3.23268 4.06981 4.53064
1.98131 3.63011 1.39485 3.12237 4.07847 4.43911 3.93760 3.85311
3.44625 3.23472 1.56021 5.14696 3.91725 654 l - - -
2.68618 4.42225 2.77519 2.73123 3.46354 2.40513 3.72494
3.29354 2.67741 2.69355 4.24690 2.90347 2.73739 3.18146 2.89801
2.37887 2.77519 2.98518 4.58477 3.61503
0.03298 3.82405 4.54639 0.61958 0.77255 0.52999 0.88821

```
    650   2.71385  4.54408  3.32487  2.81583  3.68947  3.54575  3.78269
2.16488  1.89802  2.58856  3.62097  3.23610  3.96411  3.01693  2.73480
2.87773  2.95667  2.56886  5.12105  3.85651    655 k - - -
          2.68811  4.41941  2.77555  2.73176  3.46431  2.40498  3.72643
3.29539  2.67915  2.69557  4.24847  2.89673  2.73882  3.17894  2.89932
2.37815  2.77502  2.98656  4.58679  3.60573
          0.19537  1.72897        *  4.07114  0.01721  0.00000        *
//
HMMER3/f [3.3 | Nov 2019]
NAME  CryM4
DESC  Cry toxin (IDOPS Model 4)
LENG  752
ALPH  amino
RF    no
MM    no
CONS  yes
CS    no
MAP   yes
DATE  Wed Feb  3 11:08:34 2021
NSEQ  4
EFFN  1.074219
CKSUM 2838223019
GA 750.0 750.0
STATS LOCAL MSV      -12.0702  0.69640
STATS LOCAL VITERBI  -13.3201  0.69640
STATS LOCAL FORWARD   -6.6747  0.69640
HMM          A        C        D        E        F        G        H
I        K        L        M        N        P        Q        R        S
T        V        W        Y
            m->m     m->i     m->d     i->m     i->i     d->m     d->d
  COMPO   2.57796  4.59977  2.88406  2.69168  3.28302  2.94349  3.84579
2.75056  2.65959  2.51852  3.69257  2.83201  3.27577  3.03415  3.07229
2.60805  2.76554  2.71728  4.74673  3.29808
          2.68618  4.42225  2.77519  2.73123  3.46354  2.40513  3.72494
3.29354  2.67741  2.69355  4.24690  2.90347  2.73739  3.18146  2.89801
2.37887  2.77519  2.98518  4.58477  3.61503
          0.31255  4.22952  1.37097  0.61958  0.77255  0.00000        *
      1   3.02787  4.56524  4.12873  3.72802  3.24111  3.88912  4.44613
2.42411  3.49198  1.82499  1.24367  3.99634  4.35039  3.86917  3.68860
3.38453  3.33475  2.43869  5.09158  3.85968      1 m - - -
          2.68618  4.42225  2.77519  2.73123  3.46354  2.40513  3.72494
3.29354  2.67741  2.69355  4.24690  2.90347  2.73739  3.18146  2.89801
2.37887  2.77519  2.98518  4.58477  3.61503
          0.02914  3.94610  4.66845  0.61958  0.77255  0.76495  0.62616
      2   2.35195  4.27323  3.27114  3.09048  4.14993  3.00177  4.19823
3.68256  3.16920  3.39718  4.31724  3.30338  3.74668  3.51281  3.45286
0.98525  2.83762  3.20155  5.51657  4.23388      2 s - - -
          2.68618  4.42225  2.77519  2.73123  3.46354  2.40513  3.72494
3.29354  2.67741  2.69355  4.24690  2.90347  2.73739  3.18146  2.89801
2.37887  2.77519  2.98518  4.58477  3.61503
          0.02914  3.94610  4.66845  0.61958  0.77255  0.76495  0.62616
      3   2.86626  4.38870  4.22694  3.89254  3.60433  3.79972  4.68167
2.04821  3.76143  2.27872  3.55851  4.07049  4.35627  4.11602  3.96680
3.30803  3.22611  0.93699  5.40114  4.13724      3 v - - -
```

```
          2.68618  4.42225  2.77519  2.73123  3.46354  2.40513  3.72494
3.29354  2.67741  2.69355  4.24690  2.90347  2.73739  3.18146  2.89801
2.37887  2.77519  2.98518  4.58477  3.61503
          0.02914  3.94610  4.66845  0.61958  0.77255  0.76495  0.62616
      4   2.86626  4.38870  4.22694  3.89254  3.60433  3.79972  4.68167
2.04821  3.76143  2.27872  3.55851  4.07049  4.35627  4.11602  3.96680
3.30803  3.22611  0.93699  5.40114  4.13724      4 v - - -
          2.68618  4.42225  2.77519  2.73123  3.46354  2.40513  3.72494
3.29354  2.67741  2.69355  4.24690  2.90347  2.73739  3.18146  2.89801
2.37887  2.77519  2.98518  4.58477  3.61503
          0.02914  3.94610  4.66845  0.61958  0.77255  0.76495  0.62616
      5   3.35426  4.81340  3.94723  3.69060  2.29772  3.91613  3.65425
3.35327  3.54600  2.78817  4.02071  3.81466  4.38835  3.83435  3.71455
3.48357  3.64134  3.22733  3.94424  0.85163      5 y - - -
          2.68618  4.42225  2.77519  2.73123  3.46354  2.40513  3.72494
3.29354  2.67741  2.69355  4.24690  2.90347  2.73739  3.18146  2.89801
2.37887  2.77519  2.98518  4.58477  3.61503
          0.02914  3.94610  4.66845  0.61958  0.77255  0.76495  0.62616
      6   3.35426  4.81340  3.94723  3.69060  2.29772  3.91613  3.65425
3.35327  3.54600  2.78817  4.02071  3.81466  4.38835  3.83435  3.71455
3.48357  3.64134  3.22733  3.94424  0.85163      6 y - - -
          2.68618  4.42225  2.77519  2.73123  3.46354  2.40513  3.72494
3.29354  2.67741  2.69355  4.24690  2.90347  2.73739  3.18146  2.89801
2.37887  2.77519  2.98518  4.58477  3.61503
          0.02914  3.94610  4.66845  0.61958  0.77255  0.76495  0.62616
      7   2.86626  4.38870  4.22694  3.89254  3.60433  3.79972  4.68167
2.04821  3.76143  2.27872  3.55851  4.07049  4.35627  4.11602  3.96680
3.30803  3.22611  0.93699  5.40114  4.13724      7 v - - -
          2.68618  4.42225  2.77519  2.73123  3.46354  2.40513  3.72494
3.29354  2.67741  2.69355  4.24690  2.90347  2.73739  3.18146  2.89801
2.37887  2.77519  2.98518  4.58477  3.61503
          0.02914  3.94610  4.66845  0.61958  0.77255  0.76495  0.62616
      8   3.03982  5.00607  3.22069  2.82865  4.40932  3.52113  3.80083
3.84324  0.94188  3.39238  4.35009  3.24980  4.01646  2.98829  2.44687
3.07710  3.28773  3.54695  5.43390  4.25734      8 k - - -
          2.68618  4.42225  2.77519  2.73123  3.46354  2.40513  3.72494
3.29354  2.67741  2.69355  4.24690  2.90347  2.73739  3.18146  2.89801
2.37887  2.77519  2.98518  4.58477  3.61503
          0.02914  3.94610  4.66845  0.61958  0.77255  0.76495  0.62616
      9   2.75806  4.52607  3.44690  3.35573  4.49643  0.62402  4.47138
4.11406  3.54701  3.75893  4.73537  3.61119  3.91956  3.86534  3.77485
2.93211  3.24503  3.62817  5.57201  4.60085      9 G - - -
          2.68618  4.42225  2.77519  2.73123  3.46354  2.40513  3.72494
3.29354  2.67741  2.69355  4.24690  2.90347  2.73739  3.18146  2.89801
2.37887  2.77519  2.98518  4.58477  3.61503
          0.02914  3.94610  4.66845  0.61958  0.77255  0.76495  0.62616
     10   2.75806  4.52607  3.44690  3.35573  4.49643  0.62402  4.47138
4.11406  3.54701  3.75893  4.73537  3.61119  3.91956  3.86534  3.77485
2.93211  3.24503  3.62817  5.57201  4.60085     10 G - - -
          2.68618  4.42225  2.77519  2.73123  3.46354  2.40513  3.72494
3.29354  2.67741  2.69355  4.24690  2.90347  2.73739  3.18146  2.89801
2.37887  2.77519  2.98518  4.58477  3.61503
          0.02914  3.94610  4.66845  0.61958  0.77255  0.76495  0.62616
```

```
     11   3.06939  5.20812  0.81795  2.38255  4.59519  3.26706  3.99695
4.20027  3.09517  3.79558  4.77549  2.94253  3.92509  3.23637  3.62123
3.02359  3.40961  3.83168  5.74944  4.45619      11 d - - -
          2.68618  4.42225  2.77519  2.73123  3.46354  2.40513  3.72494
3.29354  2.67741  2.69355  4.24690  2.90347  2.73739  3.18146  2.89801
2.37887  2.77519  2.98518  4.58477  3.61503
          0.02914  3.94610  4.66845  0.61958  0.77255  0.76495  0.62616
     12   3.08851  4.48711  4.43964  4.05840  3.42425  4.14544  4.77306
0.99118  3.88795  1.99552  3.34844  4.29344  4.56981  4.22881  4.07612
3.64805  3.38646  1.84989  5.33013  4.07461      12 i - - -
          2.68618  4.42225  2.77519  2.73123  3.46354  2.40513  3.72494
3.29354  2.67741  2.69355  4.24690  2.90347  2.73739  3.18146  2.89801
2.37887  2.77519  2.98518  4.58477  3.61503
          0.02914  3.94610  4.66845  0.61958  0.77255  0.76495  0.62616
     13   2.94302  4.39650  4.34900  3.82565  3.27873  4.07084  4.52797
2.05462  3.67406  1.82052  2.01876  4.08767  4.43216  3.94675  3.88784
3.42422  3.20943  1.51205  5.15221  3.97604      13 v - - -
          2.68618  4.42225  2.77519  2.73123  3.46354  2.40513  3.72494
3.29354  2.67741  2.69355  4.24690  2.90347  2.73739  3.18146  2.89801
2.37887  2.77519  2.98518  4.58477  3.61503
          0.02914  3.94610  4.66845  0.61958  0.77255  0.76495  0.62616
     14   3.06939  5.20812  0.81795  2.38255  4.59519  3.26706  3.99695
4.20027  3.09517  3.79558  4.77549  2.94253  3.92509  3.23637  3.62123
3.02359  3.40961  3.83168  5.74944  4.45619      14 d - - -
          2.68618  4.42225  2.77519  2.73123  3.46354  2.40513  3.72494
3.29354  2.67741  2.69355  4.24690  2.90347  2.73739  3.18146  2.89801
2.37887  2.77519  2.98518  4.58477  3.61503
          0.02914  3.94610  4.66845  0.61958  0.77255  0.76495  0.62616
     15   2.85075  4.59570  3.46590  3.31567  4.30429  3.27905  4.38697
3.86874  3.37928  3.48757  4.54066  3.61489  0.70144  3.76583  3.61637
3.02058  3.29814  3.50570  5.47055  4.42601      15 p - - -
          2.68618  4.42225  2.77519  2.73123  3.46354  2.40513  3.72494
3.29354  2.67741  2.69355  4.24690  2.90347  2.73739  3.18146  2.89801
2.37887  2.77519  2.98518  4.58477  3.61503
          0.02914  3.94610  4.66845  0.61958  0.77255  0.76495  0.62616
     16   3.35268  4.73293  4.33961  4.06829  0.89854  4.02483  3.91094
2.86751  3.97351  2.22071  3.58693  4.10246  4.48192  4.12126  4.07505
3.61871  3.65393  2.86358  4.13857  2.49652      16 f - - -
          2.68618  4.42225  2.77519  2.73123  3.46354  2.40513  3.72494
3.29354  2.67741  2.69355  4.24690  2.90347  2.73739  3.18146  2.89801
2.37887  2.77519  2.98518  4.58477  3.61503
          0.02914  3.94610  4.66845  0.61958  0.77255  0.76495  0.62616
     17   2.35195  4.27323  3.27114  3.09048  4.14993  3.00177  4.19823
3.68256  3.16920  3.39718  4.31724  3.30338  3.74668  3.51281  3.45286
0.98525  2.83762  3.20155  5.51657  4.23388      17 s - - -
          2.68618  4.42225  2.77519  2.73123  3.46354  2.40513  3.72494
3.29354  2.67741  2.69355  4.24690  2.90347  2.73739  3.18146  2.89801
2.37887  2.77519  2.98518  4.58477  3.61503
          0.02914  3.94610  4.66845  0.61958  0.77255  0.76495  0.62616
     18   2.78516  4.78380  2.75034  2.64333  4.15892  3.23388  3.98153
3.88535  2.89149  3.52393  4.47889  1.03405  3.88944  3.25457  3.24851
2.84964  3.16955  3.50011  5.45642  4.10209      18 n - - -
          2.68618  4.42225  2.77519  2.73123  3.46354  2.40513  3.72494
3.29354  2.67741  2.69355  4.24690  2.90347  2.73739  3.18146  2.89801
2.37887  2.77519  2.98518  4.58477  3.61503
```

```
         0.02914  3.94610  4.66845  0.61958  0.77255  0.76495  0.62616
     19  2.68440  2.19611  3.93171  3.44190  2.83860  3.60273  3.86827
2.71229  3.28111  2.44060  3.46552  3.62668  4.07623  3.57037  3.49636
2.97146  2.97852  2.52131  4.43415  1.94103     19 y - - -
         2.68618  4.42225  2.77519  2.73123  3.46354  2.40513  3.72494
3.29354  2.67741  2.69355  4.24690  2.90347  2.73739  3.18146  2.89801
2.37887  2.77519  2.98518  4.58477  3.61503
         0.02914  3.94610  4.66845  0.61958  0.77255  0.76495  0.62616
     20  2.35195  4.27323  3.27114  3.09048  4.14993  3.00177  4.19823
3.68256  3.16920  3.39718  4.31724  3.30338  3.74668  3.51281  3.45286
0.98525  2.83762  3.20155  5.51657  4.23388     20 s - - -
         2.68618  4.42225  2.77519  2.73123  3.46354  2.40513  3.72494
3.29354  2.67741  2.69355  4.24690  2.90347  2.73739  3.18146  2.89801
2.37887  2.77519  2.98518  4.58477  3.61503
         0.02914  3.94610  4.66845  0.61958  0.77255  0.76495  0.62616
     21  3.01634  5.14177  2.51979  0.92511  4.49734  3.32204  3.91546
3.96587  2.80529  3.57126  4.54223  2.97580  3.93017  3.12970  3.22162
2.99138  3.32120  3.64219  5.65506  4.37755     21 e - - -
         2.68618  4.42225  2.77519  2.73123  3.46354  2.40513  3.72494
3.29354  2.67741  2.69355  4.24690  2.90347  2.73739  3.18146  2.89801
2.37887  2.77519  2.98518  4.58477  3.61503
         0.02914  3.94610  4.66845  0.61958  0.77255  0.76495  0.62616
     22  2.90350  5.10661  2.99994  2.55550  4.46833  3.50285  3.61502
3.87948  1.53899  3.36224  4.22393  3.02630  3.93143  1.94007  2.31689
2.88936  3.10992  3.54623  5.46029  4.20422     22 k - - -
         2.68618  4.42225  2.77519  2.73123  3.46354  2.40513  3.72494
3.29354  2.67741  2.69355  4.24690  2.90347  2.73739  3.18146  2.89801
2.37887  2.77519  2.98518  4.58477  3.61503
         0.02914  3.94610  4.66845  0.61958  0.77255  0.76495  0.62616
     23  3.03982  5.00607  3.22069  2.82865  4.40932  3.52113  3.80083
3.84324  0.94188  3.39238  4.35009  3.24980  4.01646  2.98829  2.44687
3.07710  3.28773  3.54695  5.43390  4.25734     23 k - - -
         2.68618  4.42225  2.77519  2.73123  3.46354  2.40513  3.72494
3.29354  2.67741  2.69355  4.24690  2.90347  2.73739  3.18146  2.89801
2.37887  2.77519  2.98518  4.58477  3.61503
         0.02914  3.94610  4.66845  0.61958  0.77255  0.76495  0.62616
     24  3.35426  4.81340  3.94723  3.69060  2.29772  3.91613  3.65425
3.35327  3.54600  2.78817  4.02071  3.81466  4.38835  3.83435  3.71455
3.48357  3.64134  3.22733  3.94424  0.85163     24 y - - -
         2.68618  4.42225  2.77519  2.73123  3.46354  2.40513  3.72494
3.29354  2.67741  2.69355  4.24690  2.90347  2.73739  3.18146  2.89801
2.37887  2.77519  2.98518  4.58477  3.61503
         0.02914  3.94610  4.66845  0.61958  0.77255  0.76495  0.62616
     25  2.31747  4.29589  3.13814  2.86488  4.21137  3.01087  4.03374
3.66359  2.94612  3.32859  4.17712  3.16224  2.01041  3.27685  3.29520
1.59634  2.76323  3.17934  5.53641  4.26895     25 s - - -
         2.68618  4.42225  2.77519  2.73123  3.46354  2.40513  3.72494
3.29354  2.67741  2.69355  4.24690  2.90347  2.73739  3.18146  2.89801
2.37887  2.77519  2.98518  4.58477  3.61503
         0.02914  3.94610  4.66845  0.61958  0.77255  0.76495  0.62616
     26  3.06939  5.20812  0.81795  2.38255  4.59519  3.26706  3.99695
4.20027  3.09517  3.79558  4.77549  2.94253  3.92509  3.23637  3.62123
3.02359  3.40961  3.83168  5.74944  4.45619     26 d - - -
```

```
          2.68618  4.42225  2.77519  2.73123  3.46354  2.40513  3.72494
3.29354  2.67741  2.69355  4.24690  2.90347  2.73739  3.18146  2.89801
2.37887  2.77519  2.98518  4.58477  3.61503
          0.02914  3.94610  4.66845  0.61958  0.77255  0.76495  0.62616
     27   2.35195  4.27323  3.27114  3.09048  4.14993  3.00177  4.19823
3.68256  3.16920  3.39718  4.31724  3.30338  3.74668  3.51281  3.45286
0.98525  2.83762  3.20155  5.51657  4.23388     27 s - - -
          2.68618  4.42225  2.77519  2.73123  3.46354  2.40513  3.72494
3.29354  2.67741  2.69355  4.24690  2.90347  2.73739  3.18146  2.89801
2.37887  2.77519  2.98518  4.58477  3.61503
          0.02914  3.94610  4.66845  0.61958  0.77255  0.76495  0.62616
     28   2.78516  4.78380  2.75034  2.64333  4.15892  3.23388  3.98153
3.88535  2.89149  3.52393  4.47889  1.03405  3.88944  3.25457  3.24851
2.84964  3.16955  3.50011  5.45642  4.10209     28 n - - -
          2.68618  4.42225  2.77519  2.73123  3.46354  2.40513  3.72494
3.29354  2.67741  2.69355  4.24690  2.90347  2.73739  3.18146  2.89801
2.37887  2.77519  2.98518  4.58477  3.61503
          0.02914  3.94610  4.66845  0.61958  0.77255  0.76495  0.62616
     29   2.78516  4.78380  2.75034  2.64333  4.15892  3.23388  3.98153
3.88535  2.89149  3.52393  4.47889  1.03405  3.88944  3.25457  3.24851
2.84964  3.16955  3.50011  5.45642  4.10209     29 n - - -
          2.68618  4.42225  2.77519  2.73123  3.46354  2.40513  3.72494
3.29354  2.67741  2.69355  4.24690  2.90347  2.73739  3.18146  2.89801
2.37887  2.77519  2.98518  4.58477  3.61503
          0.02914  3.94610  4.66845  0.61958  0.77255  0.76495  0.62616
     30   2.78516  4.78380  2.75034  2.64333  4.15892  3.23388  3.98153
3.88535  2.89149  3.52393  4.47889  1.03405  3.88944  3.25457  3.24851
2.84964  3.16955  3.50011  5.45642  4.10209     30 n - - -
          2.68618  4.42225  2.77519  2.73123  3.46354  2.40513  3.72494
3.29354  2.67741  2.69355  4.24690  2.90347  2.73739  3.18146  2.89801
2.37887  2.77519  2.98518  4.58477  3.61503
          0.02914  3.94610  4.66845  0.61958  0.77255  0.76495  0.62616
     31   2.94003  4.92756  2.95244  2.70844  4.10582  3.41514  3.86735
3.74797  2.52418  3.24301  4.26893  3.16877  3.96973  1.10868  2.80686
2.98473  3.23700  3.47311  5.36488  4.06354     31 q - - -
          2.68618  4.42225  2.77519  2.73123  3.46354  2.40513  3.72494
3.29354  2.67741  2.69355  4.24690  2.90347  2.73739  3.18146  2.89801
2.37887  2.77519  2.98518  4.58477  3.61503
          0.02914  3.94610  4.66845  0.61958  0.77255  0.76495  0.62616
     32   3.01634  5.14177  2.51979  0.92511  4.49734  3.32204  3.91546
3.96587  2.80529  3.57126  4.54223  2.97580  3.93017  3.12970  3.22162
2.99138  3.32120  3.64219  5.65506  4.37755     32 e - - -
          2.68618  4.42225  2.77519  2.73123  3.46354  2.40513  3.72494
3.29354  2.67741  2.69355  4.24690  2.90347  2.73739  3.18146  2.89801
2.37887  2.77519  2.98518  4.58477  3.61503
          0.02914  3.94610  4.66845  0.61958  0.77255  0.76495  0.62616
     33   3.23817  4.67872  4.30758  3.94796  3.16445  4.07544  4.58060
2.37275  3.71498  0.78962  3.16477  4.21629  4.49826  4.06570  3.87893
3.65516  3.52914  2.42916  5.07026  3.81051     33 l - - -
          2.68618  4.42225  2.77519  2.73123  3.46354  2.40513  3.72494
3.29354  2.67741  2.69355  4.24690  2.90347  2.73739  3.18146  2.89801
2.37887  2.77519  2.98518  4.58477  3.61503
          0.02914  3.94610  4.66845  0.61958  0.77255  0.76495  0.62616
```

```
     34   2.69692  4.29939  3.91259  3.44140  3.53312  3.68321  4.29435
1.63721  3.31667  2.28355  3.43779  3.72121  4.17214  3.65983  3.58810
3.05395  1.98593  2.02084  5.20718  3.97974     34 i - - -
          2.68618  4.42225  2.77519  2.73123  3.46354  2.40513  3.72494
3.29354  2.67741  2.69355  4.24690  2.90347  2.73739  3.18146  2.89801
2.37887  2.77519  2.98518  4.58477  3.61503
          0.02914  3.94610  4.66845  0.61958  0.77255  0.76495  0.62616
     35   2.54458  4.23663  3.84379  3.41281  3.68596  3.44634  4.29724
2.32858  3.30941  2.49504  3.59579  3.63012  4.03516  3.64620  3.58219
2.85076  1.93493  1.53262  5.30380  4.07729     35 v - - -
          2.68618  4.42225  2.77519  2.73123  3.46354  2.40513  3.72494
3.29354  2.67741  2.69355  4.24690  2.90347  2.73739  3.18146  2.89801
2.37887  2.77519  2.98518  4.58477  3.61503
          0.02914  3.94610  4.66845  0.61958  0.77255  0.76495  0.62616
     36   3.01634  5.14177  2.51979  0.92511  4.49734  3.32204  3.91546
3.96587  2.80529  3.57126  4.54223  2.97580  3.93017  3.12970  3.22162
2.99138  3.32120  3.64219  5.65506  4.37755     36 e - - -
          2.68618  4.42225  2.77519  2.73123  3.46354  2.40513  3.72494
3.29354  2.67741  2.69355  4.24690  2.90347  2.73739  3.18146  2.89801
2.37887  2.77519  2.98518  4.58477  3.61503
          0.02914  3.94610  4.66845  0.61958  0.77255  0.76495  0.62616
     37   2.35195  4.27323  3.27114  3.09048  4.14993  3.00177  4.19823
3.68256  3.16920  3.39718  4.31724  3.30338  3.74668  3.51281  3.45286
0.98525  2.83762  3.20155  5.51657  4.23388     37 s - - -
          2.68618  4.42225  2.77519  2.73123  3.46354  2.40513  3.72494
3.29354  2.67741  2.69355  4.24690  2.90347  2.73739  3.18146  2.89801
2.37887  2.77519  2.98518  4.58477  3.61503
          0.02914  3.94610  4.66845  0.61958  0.77255  0.76495  0.62616
     38   2.35195  4.27323  3.27114  3.09048  4.14993  3.00177  4.19823
3.68256  3.16920  3.39718  4.31724  3.30338  3.74668  3.51281  3.45286
0.98525  2.83762  3.20155  5.51657  4.23388     38 s - - -
          2.68618  4.42225  2.77519  2.73123  3.46354  2.40513  3.72494
3.29354  2.67741  2.69355  4.24690  2.90347  2.73739  3.18146  2.89801
2.37887  2.77519  2.98518  4.58477  3.61503
          0.02914  3.94610  4.66845  0.61958  0.77255  0.76495  0.62616
     39   2.35195  4.27323  3.27114  3.09048  4.14993  3.00177  4.19823
3.68256  3.16920  3.39718  4.31724  3.30338  3.74668  3.51281  3.45286
0.98525  2.83762  3.20155  5.51657  4.23388     39 s - - -
          2.68618  4.42225  2.77519  2.73123  3.46354  2.40513  3.72494
3.29354  2.67741  2.69355  4.24690  2.90347  2.73739  3.18146  2.89801
2.37887  2.77519  2.98518  4.58477  3.61503
          0.02914  3.94610  4.66845  0.61958  0.77255  0.76495  0.62616
     40   3.35268  4.73293  4.33961  4.06829  0.89854  4.02483  3.91094
2.86751  3.97351  2.22071  3.58693  4.10246  4.48192  4.12126  4.07505
3.61871  3.65393  2.86358  4.13857  2.49652     40 f - - -
          2.68618  4.42225  2.77519  2.73123  3.46354  2.40513  3.72494
3.29354  2.67741  2.69355  4.24690  2.90347  2.73739  3.18146  2.89801
2.37887  2.77519  2.98518  4.58477  3.61503
          0.02914  3.94610  4.66845  0.61958  0.77255  0.76495  0.62616
     41   3.35426  4.81340  3.94723  3.69060  2.29772  3.91613  3.65425
3.35327  3.54600  2.78817  4.02071  3.81466  4.38835  3.83435  3.71455
3.48357  3.64134  3.22733  3.94424  0.85163     41 y - - -
          2.68618  4.42225  2.77519  2.73123  3.46354  2.40513  3.72494
3.29354  2.67741  2.69355  4.24690  2.90347  2.73739  3.18146  2.89801
2.37887  2.77519  2.98518  4.58477  3.61503
```

```
          0.02914  3.94610  4.66845  0.61958  0.77255  0.76495  0.62616
     42   2.35195  4.27323  3.27114  3.09048  4.14993  3.00177  4.19823
3.68256  3.16920  3.39718  4.31724  3.30338  3.74668  3.51281  3.45286
0.98525  2.83762  3.20155  5.51657  4.23388     42 s - - -
          2.68618  4.42225  2.77519  2.73123  3.46354  2.40513  3.72494
3.29354  2.67741  2.69355  4.24690  2.90347  2.73739  3.18146  2.89801
2.37887  2.77519  2.98518  4.58477  3.61503
          0.02914  3.94610  4.66845  0.61958  0.77255  0.76495  0.62616
     43   2.86258  5.31554  1.57359  2.13071  4.63612  3.19750  3.75497
4.18767  2.75496  3.71863  4.57227  1.77175  3.81677  2.92909  3.32108
2.77992  3.16464  3.76584  5.84971  4.36913     43 d - - -
          2.68618  4.42225  2.77519  2.73123  3.46354  2.40513  3.72494
3.29354  2.67741  2.69355  4.24690  2.90347  2.73739  3.18146  2.89801
2.37887  2.77519  2.98518  4.58477  3.61503
          0.02914  3.94610  4.66845  0.61958  0.77255  0.76495  0.62616
     44   2.45306  4.29820  3.49417  3.24987  4.03458  3.13497  4.26148
3.17071  3.19429  3.03158  4.07757  3.44727  3.84461  3.58549  3.44930
2.65518  1.05298  2.85779  5.46592  4.25128     44 t - - -
          2.68618  4.42225  2.77519  2.73123  3.46354  2.40513  3.72494
3.29354  2.67741  2.69355  4.24690  2.90347  2.73739  3.18146  2.89801
2.37887  2.77519  2.98518  4.58477  3.61503
          0.02914  3.94610  4.66845  0.61958  0.77255  0.76495  0.62616
     45   2.45306  4.29820  3.49417  3.24987  4.03458  3.13497  4.26148
3.17071  3.19429  3.03158  4.07757  3.44727  3.84461  3.58549  3.44930
2.65518  1.05298  2.85779  5.46592  4.25128     45 t - - -
          2.68618  4.42225  2.77519  2.73123  3.46354  2.40513  3.72494
3.29354  2.67741  2.69355  4.24690  2.90347  2.73739  3.18146  2.89801
2.37887  2.77519  2.98518  4.58477  3.61503
          0.02914  3.94610  4.66845  0.61958  0.77255  0.76495  0.62616
     46   2.78516  4.78380  2.75034  2.64333  4.15892  3.23388  3.98153
3.88535  2.89149  3.52393  4.47889  1.03405  3.88944  3.25457  3.24851
2.84964  3.16955  3.50011  5.45642  4.10209     46 n - - -
          2.68618  4.42225  2.77519  2.73123  3.46354  2.40513  3.72494
3.29354  2.67741  2.69355  4.24690  2.90347  2.73739  3.18146  2.89801
2.37887  2.77519  2.98518  4.58477  3.61503
          0.02914  3.94610  4.66845  0.61958  0.77255  0.76495  0.62616
     47   3.01634  5.14177  2.51979  0.92511  4.49734  3.32204  3.91546
3.96587  2.80529  3.57126  4.54223  2.97580  3.93017  3.12970  3.22162
2.99138  3.32120  3.64219  5.65506  4.37755     47 e - - -
          2.68618  4.42225  2.77519  2.73123  3.46354  2.40513  3.72494
3.29354  2.67741  2.69355  4.24690  2.90347  2.73739  3.18146  2.89801
2.37887  2.77519  2.98518  4.58477  3.61503
          0.02914  3.94610  4.66845  0.61958  0.77255  0.76495  0.62616
     48   2.78516  4.78380  2.75034  2.64333  4.15892  3.23388  3.98153
3.88535  2.89149  3.52393  4.47889  1.03405  3.88944  3.25457  3.24851
2.84964  3.16955  3.50011  5.45642  4.10209     48 n - - -
          2.68618  4.42225  2.77519  2.73123  3.46354  2.40513  3.72494
3.29354  2.67741  2.69355  4.24690  2.90347  2.73739  3.18146  2.89801
2.37887  2.77519  2.98518  4.58477  3.61503
          0.02914  3.94610  4.66845  0.61958  0.77255  0.34396  1.23429
     49   3.29360  4.79306  4.45849  4.09440  3.42170  4.11655  4.74704
2.63604  3.83948  1.97436  0.82679  4.32314  4.59174  4.20428  3.99959
3.67297  3.61269  2.68118  5.30086  4.08847     49 m - - -
```

2.68618 4.42225 2.77519 2.73123 3.46354 2.40513 3.72494
3.29354 2.67741 2.69355 4.24690 2.90347 2.73739 3.18146 2.89801
2.37887 2.77519 2.98518 4.58477 3.61503
0.02187 4.22952 4.95186 0.61958 0.77255 0.48576 0.95510
50 2.75398 5.14401 2.22456 2.37041 4.48766 3.41133 3.69553
3.93335 1.61522 3.46280 4.25338 2.91239 3.87258 2.82315 2.90241
2.72537 2.36537 3.53991 5.62685 4.24324 50 k - - -
2.68618 4.42225 2.77519 2.73123 3.46354 2.40513 3.72494
3.29354 2.67741 2.69355 4.24690 2.90347 2.73739 3.18146 2.89801
2.37887 2.77519 2.98518 4.58477 3.61503
0.02187 4.22952 4.95186 0.61958 0.77255 0.48576 0.95510
51 2.85512 4.97035 3.22498 2.74294 4.45309 3.53359 3.79541
3.78284 1.23807 3.37727 4.24171 3.18703 4.00381 2.94895 2.58280
2.89725 2.16527 3.44379 5.55111 4.29659 51 k - - -
2.68618 4.42225 2.77519 2.73123 3.46354 2.40513 3.72494
3.29354 2.67741 2.69355 4.24690 2.90347 2.73739 3.18146 2.89801
2.37887 2.77519 2.98518 4.58477 3.61503
0.02187 4.22952 4.95186 0.61958 0.77255 0.48576 0.95510
52 2.78454 4.99939 3.01466 2.53367 4.10404 3.51656 3.68462
3.70067 1.70683 3.25827 4.08929 2.24680 3.93017 2.84983 2.73348
2.79530 3.01070 3.37018 5.34843 2.62160 52 k - - -
2.68618 4.42225 2.77519 2.73123 3.46354 2.40513 3.72494
3.29354 2.67741 2.69355 4.24690 2.90347 2.73739 3.18146 2.89801
2.37887 2.77519 2.98518 4.58477 3.61503
0.31255 4.22952 1.37097 0.61958 0.77255 0.48576 0.95510
53 3.12101 4.79605 3.58771 3.18627 2.31565 3.84636 2.01350
3.41067 3.02363 2.87009 3.94704 3.47088 4.24142 3.38605 3.29820
3.19306 3.35422 3.21600 3.94690 1.53374 53 y - - -
2.68618 4.42225 2.77519 2.73123 3.46354 2.40513 3.72494
3.29354 2.67741 2.69355 4.24690 2.90347 2.73739 3.18146 2.89801
2.37887 2.77519 2.98518 4.58477 3.61503
0.02914 3.94610 4.66845 0.61958 0.77255 0.34396 1.23429
54 2.87626 4.89234 3.08522 2.79874 4.01454 3.48172 2.35869
3.83252 2.77507 3.40355 4.30379 3.25055 1.25678 3.21922 3.11863
2.94437 3.19290 3.48842 5.36377 3.96460 54 p - - -
2.68618 4.42225 2.77519 2.73123 3.46354 2.40513 3.72494
3.29354 2.67741 2.69355 4.24690 2.90347 2.73739 3.18146 2.89801
2.37887 2.77519 2.98518 4.58477 3.61503
0.02187 4.22952 4.95186 0.61958 0.77255 0.48576 0.95510
55 2.71849 4.72313 3.22700 2.68878 3.96233 3.54278 3.76545
2.40168 1.78353 2.95243 3.83870 3.15782 2.58563 2.95694 2.83886
2.81022 2.95616 2.99552 5.27467 3.99710 55 k - - -
2.68618 4.42225 2.77519 2.73123 3.46354 2.40513 3.72494
3.29354 2.67741 2.69355 4.24690 2.90347 2.73739 3.18146 2.89801
2.37887 2.77519 2.98518 4.58477 3.61503
0.02187 4.22952 4.95186 0.61958 0.77255 0.48576 0.95510
56 2.75639 4.92569 3.04582 2.04399 4.18595 3.52025 3.70437
2.46784 1.74061 3.15511 4.00344 3.05110 3.92584 2.85745 2.75632
2.78694 2.98167 3.21201 5.40602 4.09747 56 k - - -
2.68618 4.42225 2.77519 2.73123 3.46354 2.40513 3.72494
3.29354 2.67741 2.69355 4.24690 2.90347 2.73739 3.18146 2.89801
2.37887 2.77519 2.98518 4.58477 3.61503
0.02187 4.22952 4.95186 0.61958 0.77255 0.48576 0.95510

```
     57   2.71511  4.70626  3.11100  2.64978  3.88929  3.52274  3.77837
 2.08748  2.58092  2.92738  3.81578  2.30412  3.94695  2.27628  2.96378
 2.80918  2.95599  2.97596  5.24153  3.94141      57 i - - -
          2.68618  4.42225  2.77519  2.73123  3.46354  2.40513  3.72494
 3.29354  2.67741  2.69355  4.24690  2.90347  2.73739  3.18146  2.89801
 2.37887  2.77519  2.98518  4.58477  3.61503
          0.02187  4.22952  4.95186  0.61958  0.77255  0.48576  0.95510
     58   2.73704  4.95374  2.22764  2.46346  4.18325  3.46418  3.71510
 3.57907  1.97693  2.07804  4.03060  2.98861  3.89885  2.87072  2.92227
 2.75396  2.97421  3.26582  5.43895  4.09470      58 k - - -
          2.68618  4.42225  2.77519  2.73123  3.46354  2.40513  3.72494
 3.29354  2.67741  2.69355  4.24690  2.90347  2.73739  3.18146  2.89801
 2.37887  2.77519  2.98518  4.58477  3.61503
          0.02187  4.22952  4.95186  0.61958  0.77255  0.48576  0.95510
     59   2.60580  4.55371  3.20285  2.75190  3.88547  3.41968  3.86847
 2.32663  2.75634  2.95256  3.82734  2.26904  3.91408  3.10067  3.15302
 1.84055  2.90106  2.93553  5.26285  3.97325      59 s - - -
          2.68618  4.42225  2.77519  2.73123  3.46354  2.40513  3.72494
 3.29354  2.67741  2.69355  4.24690  2.90347  2.73739  3.18146  2.89801
 2.37887  2.77519  2.98518  4.58477  3.61503
          0.02187  4.22952  4.95186  0.61958  0.77255  0.48576  0.95510
     60   3.36114  4.68358  5.00293  4.43077  2.02468  4.57813  4.75647
 2.37251  4.28896  1.20345  1.71881  4.61447  4.76522  4.33200  4.35443
 3.91586  3.57547  2.53373  5.01474  3.82671      60 l - - -
          2.68618  4.42225  2.77519  2.73123  3.46354  2.40513  3.72494
 3.29354  2.67741  2.69355  4.24690  2.90347  2.73739  3.18146  2.89801
 2.37887  2.77519  2.98518  4.58477  3.61503
          0.02187  4.22952  4.95186  0.61958  0.77255  0.48576  0.95510
     61   2.63564  4.76174  3.09458  2.62446  4.24890  3.38612  3.79299
 3.65018  1.96892  3.25798  4.08118  3.08978  3.88193  2.95567  2.90846
 2.03219  1.90009  3.28273  5.47838  4.17489      61 t - - -
          2.68618  4.42225  2.77519  2.73123  3.46354  2.40513  3.72494
 3.29354  2.67741  2.69355  4.24690  2.90347  2.73739  3.18146  2.89801
 2.37887  2.77519  2.98518  4.58477  3.61503
          0.02187  4.22952  4.95186  0.61958  0.77255  0.48576  0.95510
     62   2.87380  4.43970  3.97782  3.40497  2.24700  3.88977  4.04293
 2.67327  3.18338  1.51964  3.28865  3.71544  4.23966  3.53424  2.31603
 3.17837  3.10272  2.58966  4.76578  3.41859      62 l - - -
          2.68618  4.42225  2.77519  2.73123  3.46354  2.40513  3.72494
 3.29354  2.67741  2.69355  4.24690  2.90347  2.73739  3.18146  2.89801
 2.37887  2.77519  2.98518  4.58477  3.61503
          0.02187  4.22952  4.95186  0.61958  0.77255  0.48576  0.95510
     63   1.16640  4.53643  3.12722  2.88785  4.39102  3.20191  4.13336
 3.78607  3.04381  3.48651  4.33853  2.01035  3.88159  3.36067  3.42147
 2.65758  2.95579  3.33168  5.71124  4.42017      63 a - - -
          2.68618  4.42225  2.77519  2.73123  3.46354  2.40513  3.72494
 3.29354  2.67741  2.69355  4.24690  2.90347  2.73739  3.18146  2.89801
 2.37887  2.77519  2.98518  4.58477  3.61503
          0.02187  4.22952  4.95186  0.61958  0.77255  0.48576  0.95510
     64   2.53866  4.53215  3.23789  2.75064  3.99875  3.34828  3.87494
 3.36258  2.75046  3.04834  3.89523  2.29425  3.86901  3.08531  3.16185
 2.01457  2.24894  2.34459  5.34135  4.06087      64 s - - -
          2.68618  4.42225  2.77519  2.73123  3.46354  2.40513  3.72494
 3.29354  2.67741  2.69355  4.24690  2.90347  2.73739  3.18146  2.89801
 2.37887  2.77519  2.98518  4.58477  3.61503
```

```
          0.02187  4.22952  4.95186  0.61958  0.77255  0.48576  0.95510
     65   2.79327  5.11111  2.65907  2.40256  4.53890  1.95411  3.79149
4.01479  2.59282  3.55053  4.36205  2.08570  3.90275  2.12850  3.04478
2.77717  3.07604  3.60741  5.71262  4.32863     65 g - - -
          2.68618  4.42225  2.77519  2.73123  3.46354  2.40513  3.72494
3.29354  2.67741  2.69355  4.24690  2.90347  2.73739  3.18146  2.89801
2.37887  2.77519  2.98518  4.58477  3.61503
          0.20483  4.22952  1.76812  0.61958  0.77255  0.48576  0.95510
     66   2.73444  4.42590  3.53023  2.21892  3.60208  3.71947  4.02673
1.95545  2.97550  2.45330  3.50020  3.44837  4.10905  3.31588  3.33247
3.00218  2.97854  1.83020  5.15519  3.90769     66 v - - -
          2.68618  4.42225  2.77519  2.73123  3.46354  2.40513  3.72494
3.29354  2.67741  2.69355  4.24690  2.90347  2.73739  3.18146  2.89801
2.37887  2.77519  2.98518  4.58477  3.61503
          0.02620  4.05089  4.77324  0.61958  0.77255  0.67925  0.70724
     67   3.34538  4.68971  4.68624  4.22381  1.38741  4.39428  4.18822
2.46613  4.07144  1.35901  3.12433  4.31702  4.66536  4.17172  4.17685
3.76704  3.57968  2.55112  4.41588  2.86473     67 l - - -
          2.68618  4.42225  2.77519  2.73123  3.46354  2.40513  3.72494
3.29354  2.67741  2.69355  4.24690  2.90347  2.73739  3.18146  2.89801
2.37887  2.77519  2.98518  4.58477  3.61503
          0.02620  4.05089  4.77324  0.61958  0.77255  0.67925  0.70724
     68   1.75976  4.27073  3.39256  2.99568  4.12958  3.08504  4.06979
3.45515  2.99676  3.18273  4.03689  3.26240  2.16640  3.31662  3.34872
2.49253  1.98628  3.03304  5.48660  4.25054     68 a - - -
          2.68618  4.42225  2.77519  2.73123  3.46354  2.40513  3.72494
3.29354  2.67741  2.69355  4.24690  2.90347  2.73739  3.18146  2.89801
2.37887  2.77519  2.98518  4.58477  3.61503
          0.02620  4.05089  4.77324  0.61958  0.77255  0.38139  1.14858
     69   3.01386  5.33720  1.22179  2.38739  4.90572  1.62575  4.02850
4.45114  3.08080  3.98463  4.84816  2.94619  3.99535  3.21781  3.65220
2.96470  3.36007  3.98475  6.07885  4.66675     69 d - - -
          2.68618  4.42225  2.77519  2.73123  3.46354  2.40513  3.72494
3.29354  2.67741  2.69355  4.24690  2.90347  2.73739  3.18146  2.89801
2.37887  2.77519  2.98518  4.58477  3.61503
          0.02187  4.22952  4.95186  0.61958  0.77255  0.48576  0.95510
     70   2.58367  4.59981  3.09208  2.82289  4.31539  3.26039  4.05467
3.70607  2.91806  3.40273  4.26428  2.09317  3.90164  3.27375  3.29179
2.69961  1.29093  3.29593  5.63175  4.33080     70 t - - -
          2.68618  4.42225  2.77519  2.73123  3.46354  2.40513  3.72494
3.29354  2.67741  2.69355  4.24690  2.90347  2.73739  3.18146  2.89801
2.37887  2.77519  2.98518  4.58477  3.61503
          0.02187  4.22952  4.95186  0.61958  0.77255  0.48576  0.95510
     71   3.05735  4.54979  4.21017  3.75765  2.70726  4.00934  3.95863
2.72228  3.60181  2.46510  3.60717  3.91492  4.41588  3.85800  3.79689
3.36353  3.32154  1.81905  4.36423  1.29476     71 y - - -
          2.68618  4.42225  2.77519  2.73123  3.46354  2.40513  3.72494
3.29354  2.67741  2.69355  4.24690  2.90347  2.73739  3.18146  2.89801
2.37887  2.77519  2.98518  4.58477  3.61503
          0.02187  4.22952  4.95186  0.61958  0.77255  0.48576  0.95510
     72   2.99381  4.38663  4.53552  3.97421  3.03685  4.14801  4.31935
1.58415  3.81633  1.53721  3.17494  4.13837  4.45910  4.00134  3.95276
3.46319  3.22387  2.27215  4.77199  2.31011     72 l - - -
```

2.68618 4.42225 2.77519 2.73123 3.46354 2.40513 3.72494
3.29354 2.67741 2.69355 4.24690 2.90347 2.73739 3.18146 2.89801
2.37887 2.77519 2.98518 4.58477 3.61503
0.02187 4.22952 4.95186 0.61958 0.77255 0.48576 0.95510
73 2.83408 5.28194 1.91398 2.29900 4.60807 3.37211 3.75622
4.09480 2.61024 3.60788 4.40857 2.86089 2.09662 2.13300 3.12163
2.77971 3.09568 3.68502 5.76970 4.34264 73 d - - -
2.68618 4.42225 2.77519 2.73123 3.46354 2.40513 3.72494
3.29354 2.67741 2.69355 4.24690 2.90347 2.73739 3.18146 2.89801
2.37887 2.77519 2.98518 4.58477 3.61503
0.02187 4.22952 4.95186 0.61958 0.77255 0.48576 0.95510
74 2.67668 4.36413 3.59029 3.06067 3.46986 3.63511 2.62240
1.80856 2.93721 2.53493 3.48429 3.43366 4.04431 3.29384 3.24702
2.92321 2.33365 2.54256 4.95467 3.69484 74 i - - -
2.68618 4.42225 2.77519 2.73123 3.46354 2.40513 3.72494
3.29354 2.67741 2.69355 4.24690 2.90347 2.73739 3.18146 2.89801
2.37887 2.77519 2.98518 4.58477 3.61503
0.02187 4.22952 4.95186 0.61958 0.77255 0.48576 0.95510
75 2.03760 4.34460 3.53021 3.01792 3.74145 3.39417 3.98766
3.03961 2.97019 2.06115 3.68693 3.35876 3.92303 3.28643 3.31996
2.06207 2.23034 2.76273 5.16924 3.93445 75 a - - -
2.68618 4.42225 2.77519 2.73123 3.46354 2.40513 3.72494
3.29354 2.67741 2.69355 4.24690 2.90347 2.73739 3.18146 2.89801
2.37887 2.77519 2.98518 4.58477 3.61503
0.31255 4.22952 1.37097 0.61958 0.77255 0.48576 0.95510
76 2.86258 5.31554 1.57359 2.13071 4.63612 3.19750 3.75497
4.18767 2.75496 3.71863 4.57227 1.77175 3.81677 2.92909 3.32108
2.77992 3.16464 3.76584 5.84971 4.36913 76 d - - -
2.68618 4.42225 2.77519 2.73123 3.46354 2.40513 3.72494
3.29354 2.67741 2.69355 4.24690 2.90347 2.73739 3.18146 2.89801
2.37887 2.77519 2.98518 4.58477 3.61503
0.02914 3.94610 4.66845 0.61958 0.77255 0.76495 0.62616
77 3.05255 4.39064 4.69374 4.19871 3.51459 4.35871 4.92545
1.31337 4.08080 2.02390 3.33002 4.42919 4.68640 4.35318 4.28795
3.74408 3.32178 1.24878 5.46935 4.26219 77 v - - -
2.68627 4.42234 2.77528 2.73132 3.46362 2.40521 3.72503
3.29363 2.67749 2.69363 4.24698 2.90302 2.73748 3.18155 2.89809
2.37833 2.77528 2.98527 4.58485 3.61512
0.26241 1.50770 4.66845 0.95654 0.48486 0.34396 1.23429
78 2.78933 4.59986 3.48532 3.10444 3.06377 3.56579 3.86103
3.34703 3.07601 2.97044 3.93412 3.43607 4.09227 3.40774 3.39828
1.60907 3.10961 3.08024 4.63345 1.66610 81 s - - -
2.68618 4.42225 2.77519 2.73123 3.46354 2.40513 3.72494
3.29354 2.67741 2.69355 4.24690 2.90347 2.73739 3.18146 2.89801
2.37887 2.77519 2.98518 4.58477 3.61503
0.02187 4.22952 4.95186 0.61958 0.77255 0.48576 0.95510
79 2.72982 4.82411 3.05579 2.67383 4.26550 3.42155 3.85429
3.65796 2.55745 3.26741 4.14863 3.13453 3.94284 1.65190 2.91028
2.79381 1.83023 3.31986 5.51846 4.21339 82 q - - -
2.68618 4.42225 2.77519 2.73123 3.46354 2.40513 3.72494
3.29354 2.67741 2.69355 4.24690 2.90347 2.73739 3.18146 2.89801
2.37887 2.77519 2.98518 4.58477 3.61503
0.02187 4.22952 4.95186 0.61958 0.77255 0.48576 0.95510

```
     80   1.53267  4.61641  3.01917  2.79005  4.42641  3.22444  4.08057
3.83541  2.97590  3.51718  4.36578  1.51460  3.88778  3.29253  3.37295
2.68027  2.98315  3.38372  5.72736  4.41527     83 n - - -
          2.68618  4.42225  2.77519  2.73123  3.46354  2.40513  3.72494
3.29354  2.67741  2.69355  4.24690  2.90347  2.73739  3.18146  2.89801
2.37887  2.77519  2.98518  4.58477  3.61503
          0.20483  4.22952  1.76812  0.61958  0.77255  0.48576  0.95510
     81   3.21866  4.57194  4.75968  4.19238  2.12237  4.40959  4.58889
2.29213  4.05964  1.21598  1.97292  4.40629  4.62370  4.15166  4.16917
3.73839  3.43659  2.42126  4.91146  3.69815     84 l - - -
          2.68618  4.42225  2.77519  2.73123  3.46354  2.40513  3.72494
3.29354  2.67741  2.69355  4.24690  2.90347  2.73739  3.18146  2.89801
2.37887  2.77519  2.98518  4.58477  3.61503
          0.02620  4.05089  4.77324  0.61958  0.77255  0.67925  0.70724
     82   1.61620  4.68301  3.08484  2.68244  4.20715  3.34331  3.80930
3.52012  1.81554  3.19560  4.07036  3.11440  3.87650  2.99296  2.79635
2.72570  2.95300  3.18170  5.44417  4.17255     85 a - - -
          2.68618  4.42225  2.77519  2.73123  3.46354  2.40513  3.72494
3.29354  2.67741  2.69355  4.24690  2.90347  2.73739  3.18146  2.89801
2.37887  2.77519  2.98518  4.58477  3.61503
          0.02620  4.05089  4.77324  0.61958  0.77255  0.67925  0.70724
     83   2.72556  4.92559  2.64797  1.54147  4.33245  3.31761  3.77557
3.66892  2.59961  3.32782  4.19470  2.92610  3.85944  2.94559  3.03601
2.74249  2.07667  3.32641  5.57992  4.22768     86 e - - -
          2.68618  4.42225  2.77519  2.73123  3.46354  2.40513  3.72494
3.29354  2.67741  2.69355  4.24690  2.90347  2.73739  3.18146  2.89801
2.37887  2.77519  2.98518  4.58477  3.61503
          0.02620  4.05089  4.77324  0.61958  0.77255  0.67925  0.70724
     84   2.69407  4.83092  2.72875  1.82358  4.25049  3.32004  3.79526
3.56849  2.61775  3.25198  4.12696  2.97011  3.86231  2.97559  3.03770
2.73556  1.74706  3.23758  5.52383  4.19212     87 t - - -
          2.68618  4.42225  2.77519  2.73123  3.46354  2.40513  3.72494
3.29354  2.67741  2.69355  4.24690  2.90347  2.73739  3.18146  2.89801
2.37887  2.77519  2.98518  4.58477  3.61503
          0.02620  4.05089  4.77324  0.61958  0.77255  0.67925  0.70724
     85   2.76761  4.68992  3.04215  1.71719  3.84585  3.54278  3.87387
2.06859  2.71540  2.73193  3.75853  3.17021  3.99577  3.09214  3.10459
2.89313  3.02092  2.65009  5.30477  4.00307     88 e - - -
          2.68618  4.42225  2.77519  2.73123  3.46354  2.40513  3.72494
3.29354  2.67741  2.69355  4.24690  2.90347  2.73739  3.18146  2.89801
2.37887  2.77519  2.98518  4.58477  3.61503
          0.02620  4.05089  4.77324  0.61958  0.77255  0.38139  1.14858
     86   2.65691  4.82851  2.98191  2.49872  4.11169  3.43930  3.70704
3.52119  2.49705  3.13307  3.95758  2.29764  2.81891  2.35097  2.93652
2.69681  2.90417  2.42427  5.37629  4.04701     89 n - - -
          2.68618  4.42225  2.77519  2.73123  3.46354  2.40513  3.72494
3.29354  2.67741  2.69355  4.24690  2.90347  2.73739  3.18146  2.89801
2.37887  2.77519  2.98518  4.58477  3.61503
          0.02187  4.22952  4.95186  0.61958  0.77255  0.48576  0.95510
     87   2.89438  5.34550  2.59893  1.91174  4.67859  3.42420  3.73878
4.14455  1.99076  3.63190  4.43671  1.66845  3.92485  2.87283  2.90511
2.83221  3.13842  3.74084  5.76440  4.36954     90 n - - -
          2.68618  4.42225  2.77519  2.73123  3.46354  2.40513  3.72494
3.29354  2.67741  2.69355  4.24690  2.90347  2.73739  3.18146  2.89801
2.37887  2.77519  2.98518  4.58477  3.61503
```

```
          0.02187  4.22952  4.95186  0.61958  0.77255  0.48576  0.95510
     88   2.63933  4.61072  3.15929  2.21987  3.83009  3.49845  3.76982
2.37626  2.63854  2.85993  3.73042  3.12698  2.85736  2.98272  3.05694
2.74983  2.27932  2.88054  5.19470  3.91003     91 e - - -
          2.68618  4.42225  2.77519  2.73123  3.46354  2.40513  3.72494
3.29354  2.67741  2.69355  4.24690  2.90347  2.73739  3.18146  2.89801
2.37887  2.77519  2.98518  4.58477  3.61503
          0.15591  4.22952  2.04170  0.61958  0.77255  0.48576  0.95510
     89   3.24906  5.39346  0.64098  2.50539  4.80473  3.39693  4.15234
4.44792  3.28260  4.01801  5.00685  3.07098  4.06615  3.39589  3.82517
3.18981  3.59442  4.06482  5.92753  4.64773     92 D - - -
          2.68618  4.42225  2.77519  2.73123  3.46354  2.40513  3.72494
3.29354  2.67741  2.69355  4.24690  2.90347  2.73739  3.18146  2.89801
2.37887  2.77519  2.98518  4.58477  3.61503
          0.02496  4.09857  4.82092  0.61958  0.77255  0.63433  0.75564
     90   3.33162  4.63896  4.94136  4.41266  3.21585  4.62113  4.97957
1.50690  4.24501  1.03120  3.02545  4.65848  4.82687  4.40947  4.37505
3.99872  3.56714  2.05391  5.29698  4.17418     93 l - - -
          2.68618  4.42225  2.77519  2.73123  3.46354  2.40513  3.72494
3.29354  2.67741  2.69355  4.24690  2.90347  2.73739  3.18146  2.89801
2.37887  2.77519  2.98518  4.58477  3.61503
          0.02496  4.09857  4.82092  0.61958  0.77255  0.63433  0.75564
     91   2.62708  4.52200  3.18778  2.74766  2.33299  3.45082  3.78437
3.15531  2.76862  2.81806  3.72247  2.38665  2.50366  3.09480  3.15811
2.76197  2.90223  2.89162  4.95384  3.55216     94 f - - -
          2.68618  4.42225  2.77519  2.73123  3.46354  2.40513  3.72494
3.29354  2.67741  2.69355  4.24690  2.90347  2.73739  3.18146  2.89801
2.37887  2.77519  2.98518  4.58477  3.61503
          0.02496  4.09857  4.82092  0.61958  0.77255  0.63433  0.75564
     92   2.04659  4.67583  2.97172  2.09953  3.81788  3.43966  3.72657
3.27194  2.59462  2.93438  3.80357  3.04454  3.88067  2.94297  3.01414
2.73556  2.90215  2.99319  5.18375  2.57646     95 a - - -
          2.68618  4.42225  2.77519  2.73123  3.46354  2.40513  3.72494
3.29354  2.67741  2.69355  4.24690  2.90347  2.73739  3.18146  2.89801
2.37887  2.77519  2.98518  4.58477  3.61503
          0.18160  4.09857  1.90066  0.61958  0.77255  0.63433  0.75564
     93   2.58089  4.67668  2.72503  2.51090  4.25656  3.17543  3.84177
3.76687  2.69908  3.38041  4.23751  1.88882  2.03821  3.04458  3.10338
2.64510  2.94141  3.35253  5.52631  4.18772     96 n - - -
          2.68618  4.42225  2.77519  2.73123  3.46354  2.40513  3.72494
3.29354  2.67741  2.69355  4.24690  2.90347  2.73739  3.18146  2.89801
2.37887  2.77519  2.98518  4.58477  3.61503
          0.02914  3.94610  4.66845  0.61958  0.77255  0.76495  0.62616
     94   2.64031  4.39402  3.43477  2.99688  3.13380  3.48496  3.80664
2.91135  2.89870  2.61256  3.60532  3.34198  3.96986  3.26208  3.20627
2.83553  2.01728  2.69240  4.68245  2.07191     97 t - - -
          2.68618  4.42225  2.77519  2.73123  3.46354  2.40513  3.72494
3.29354  2.67741  2.69355  4.24690  2.90347  2.73739  3.18146  2.89801
2.37887  2.77519  2.98518  4.58477  3.61503
          0.02914  3.94610  4.66845  0.61958  0.77255  0.76495  0.62616
     95   2.78516  4.78380  2.75034  2.64333  4.15892  3.23388  3.98153
3.88535  2.89149  3.52393  4.47889  1.03405  3.88944  3.25457  3.24851
2.84964  3.16955  3.50011  5.45642  4.10209     98 n - - -
```

```
          2.68618  4.42225  2.77519  2.73123  3.46354  2.40513  3.72494
3.29354  2.67741  2.69355  4.24690  2.90347  2.73739  3.18146  2.89801
2.37887  2.77519  2.98518  4.58477  3.61503
          0.02914  3.94610  4.66845  0.61958  0.77255  0.76495  0.62616
     96   2.78516  4.78380  2.75034  2.64333  4.15892  3.23388  3.98153
3.88535  2.89149  3.52393  4.47889  1.03405  3.88944  3.25457  3.24851
2.84964  3.16955  3.50011  5.45642  4.10209     99 n - - -
          2.68618  4.42225  2.77519  2.73123  3.46354  2.40513  3.72494
3.29354  2.67741  2.69355  4.24690  2.90347  2.73739  3.18146  2.89801
2.37887  2.77519  2.98518  4.58477  3.61503
          0.02914  3.94610  4.66845  0.61958  0.77255  0.76495  0.62616
     97   3.18881  4.53128  4.69923  4.17706  3.19171  4.43937  4.80173
1.46712  4.00607  1.22638  3.02720  4.44157  4.68743  4.23031  4.17528
3.80963  3.43084  1.94058  5.21849  4.05198    100 l - - -
          2.68618  4.42225  2.77519  2.73123  3.46354  2.40513  3.72494
3.29354  2.67741  2.69355  4.24690  2.90347  2.73739  3.18146  2.89801
2.37887  2.77519  2.98518  4.58477  3.61503
          0.02914  3.94610  4.66845  0.61958  0.77255  0.53136  0.88626
     98   2.80534  5.11979  2.84365  2.43534  4.39422  3.45456  2.37818
3.88664  1.86959  3.39567  4.21105  2.16930  3.89302  2.78699  2.62572
2.78235  3.03116  3.52183  5.50477  4.15821    101 k - - -
          2.68618  4.42225  2.77519  2.73123  3.46354  2.40513  3.72494
3.29354  2.67741  2.69355  4.24690  2.90347  2.73739  3.18146  2.89801
2.37887  2.77519  2.98518  4.58477  3.61503
          0.02496  4.09857  4.82092  0.61958  0.77255  0.40295  1.10367
     99   2.66526  4.63999  3.22552  2.70928  3.91133  3.49573  3.79465
2.50078  2.12926  2.93784  3.81269  3.16699  3.93233  3.00255  2.94850
1.76072  2.92266  2.95737  5.24977  3.97013    102 s - - -
          2.68618  4.42225  2.77519  2.73123  3.46354  2.40513  3.72494
3.29354  2.67741  2.69355  4.24690  2.90347  2.73739  3.18146  2.89801
2.37887  2.77519  2.98518  4.58477  3.61503
          0.02187  4.22952  4.95186  0.61958  0.77255  0.48576  0.95510
    100   2.64805  4.29980  3.64824  3.09044  3.44099  3.65103  3.94934
2.09666  3.01529  2.46174  2.50038  2.64641  4.04079  3.32027  3.33257
2.91978  2.34902  2.45237  4.92973  3.70672    103 i - - -
          2.68618  4.42225  2.77519  2.73123  3.46354  2.40513  3.72494
3.29354  2.67741  2.69355  4.24690  2.90347  2.73739  3.18146  2.89801
2.37887  2.77519  2.98518  4.58477  3.61503
          0.02187  4.22952  4.95186  0.61958  0.77255  0.48576  0.95510
    101   3.26759  5.36652  3.75315  2.99511  4.89070  3.84844  3.71250
4.20486  1.21624  3.59292  4.46971  3.36637  4.19376  2.84111  1.32958
3.24972  3.40691  3.88674  5.58668  4.46210    104 k - - -
          2.68618  4.42225  2.77519  2.73123  3.46354  2.40513  3.72494
3.29354  2.67741  2.69355  4.24690  2.90347  2.73739  3.18146  2.89801
2.37887  2.77519  2.98518  4.58477  3.61503
          0.02187  4.22952  4.95186  0.61958  0.77255  0.48576  0.95510
    102   2.94412  5.43641  1.72669  2.25258  4.82898  2.32441  3.85676
4.34757  2.82997  3.84772  4.67045  1.61675  3.92750  3.01407  3.40272
2.86345  3.24028  3.90083  6.00309  4.52406    105 n - - -
          2.68618  4.42225  2.77519  2.73123  3.46354  2.40513  3.72494
3.29354  2.67741  2.69355  4.24690  2.90347  2.73739  3.18146  2.89801
2.37887  2.77519  2.98518  4.58477  3.61503
          0.31255  4.22952  1.37097  0.61958  0.77255  0.48576  0.95510
```

```
    103   2.67009  4.82151  2.65711  1.73473  4.22784  3.28170  3.74854
3.54209  2.56889  3.22499  4.10302  2.91557  3.82384  2.92700  2.99042
2.70414  2.02621  3.21314  5.49681  4.15950    106 e - - -
          2.68618  4.42225  2.77519  2.73123  3.46354  2.40513  3.72494
3.29354  2.67741  2.69355  4.24690  2.90347  2.73739  3.18146  2.89801
2.37887  2.77519  2.98518  4.58477  3.61503
          0.28223  3.94610  1.48471  0.61958  0.77255  0.34396  1.23429
    104   2.70460  4.27619  3.88517  3.34742  3.34336  3.73836  4.11540
1.92391  3.23063  1.75100  3.30562  3.65487  4.14279  3.53492  3.49824
2.31018  2.96390  2.21611  4.95333  3.72530    107 l - - -
          2.68618  4.42225  2.77519  2.73123  3.46354  2.40513  3.72494
3.29354  2.67741  2.69355  4.24690  2.90347  2.73739  3.18146  2.89801
2.37887  2.77519  2.98518  4.58477  3.61503
          0.02620  4.05089  4.77324  0.61958  0.77255  0.67925  0.70724
    105   2.56207  2.55646  3.70242  3.17561  3.44359  3.52578  3.99720
2.06532  3.07706  2.48221  3.43576  2.50077  3.99151  3.39016  3.36725
2.84574  2.84865  2.36221  4.94689  3.71095    108 i - - -
          2.68618  4.42225  2.77519  2.73123  3.46354  2.40513  3.72494
3.29354  2.67741  2.69355  4.24690  2.90347  2.73739  3.18146  2.89801
2.37887  2.77519  2.98518  4.58477  3.61503
          0.02620  4.05089  4.77324  0.61958  0.77255  0.67925  0.70724
    106   2.82234  5.14597  2.92769  1.99170  4.50423  3.47970  3.61543
3.92305  2.15190  3.40614  4.21923  2.96813  3.89560  2.15207  1.91411
2.79461  3.03462  3.55547  5.51982  4.21544    109 r - - -
          2.68618  4.42225  2.77519  2.73123  3.46354  2.40513  3.72494
3.29354  2.67741  2.69355  4.24690  2.90347  2.73739  3.18146  2.89801
2.37887  2.77519  2.98518  4.58477  3.61503
          0.02620  4.05089  4.77324  0.61958  0.77255  0.67925  0.70724
    107   2.73946  5.10965  1.85302  2.25508  4.49312  3.27559  3.72514
3.95529  2.61650  3.51002  4.32336  2.13274  3.82142  2.87635  3.13470
2.70583  2.24150  3.55197  5.69571  4.27794    110 d - - -
          2.68618  4.42225  2.77519  2.73123  3.46354  2.40513  3.72494
3.29354  2.67741  2.69355  4.24690  2.90347  2.73739  3.18146  2.89801
2.37887  2.77519  2.98518  4.58477  3.61503
          0.02620  4.05089  4.77324  0.61958  0.77255  0.67925  0.70724
    108   2.72860  4.64293  3.11346  2.76263  3.65928  3.46634  3.85771
3.12246  2.72017  1.97387  3.82349  1.78146  3.96896  3.13485  3.06698
2.85154  3.01773  2.89716  5.13083  3.74669    111 n - - -
          2.68618  4.42225  2.77519  2.73123  3.46354  2.40513  3.72494
3.29354  2.67741  2.69355  4.24690  2.90347  2.73739  3.18146  2.89801
2.37887  2.77519  2.98518  4.58477  3.61503
          0.02620  4.05089  4.77324  0.61958  0.77255  0.67925  0.70724
    109   3.00522  4.72913  3.63226  3.49957  4.47376  3.40671  4.55952
4.06867  3.57285  3.67467  4.73439  3.78670  0.55391  3.95613  3.79526
3.17904  3.46148  3.69306  5.60793  4.59794    112 P - - -
          2.68618  4.42225  2.77519  2.73123  3.46354  2.40513  3.72494
3.29354  2.67741  2.69355  4.24690  2.90347  2.73739  3.18146  2.89801
2.37887  2.77519  2.98518  4.58477  3.61503
          0.02620  4.05089  4.77324  0.61958  0.77255  0.67925  0.70724
    110   2.59768  4.76406  2.86156  2.48520  4.23866  3.29621  3.73715
3.68038  2.51391  3.26671  4.08926  2.97210  2.42046  2.21758  2.92911
2.07449  2.89695  3.29937  5.47258  4.14120    113 s - - -
          2.68618  4.42225  2.77519  2.73123  3.46354  2.40513  3.72494
3.29354  2.67741  2.69355  4.24690  2.90347  2.73739  3.18146  2.89801
2.37887  2.77519  2.98518  4.58477  3.61503
```

```
         0.02620  4.05089  4.77324  0.61958  0.77255  0.67925  0.70724
    111  2.79195  4.56494  3.25040  2.06591  3.70444  3.64136  3.98692
1.66223  2.87262  2.55709  3.62617  3.33281  4.08110  3.25053  3.22701
2.99638  3.04571  2.40618  5.23612  3.96311    114 i - - -
         2.68618  4.42225  2.77519  2.73123  3.46354  2.40513  3.72494
3.29354  2.67741  2.69355  4.24690  2.90347  2.73739  3.18146  2.89801
2.37887  2.77519  2.98518  4.58477  3.61503
         0.02620  4.05089  4.77324  0.61958  0.77255  0.67925  0.70724
    112  2.72955  4.97032  1.51663  2.34411  4.44871  3.25000  3.82543
3.82613  2.76097  3.48155  4.34283  2.87633  3.84397  3.00148  3.26542
2.73297  2.02396  3.44537  5.70872  4.31565    115 d - - -
         2.68618  4.42225  2.77519  2.73123  3.46354  2.40513  3.72494
3.29354  2.67741  2.69355  4.24690  2.90347  2.73739  3.18146  2.89801
2.37887  2.77519  2.98518  4.58477  3.61503
         0.02620  4.05089  4.77324  0.61958  0.77255  0.67925  0.70724
    113  2.57701  4.57152  3.04934  2.63414  3.86930  3.35147  3.78173
3.29358  2.63544  2.26593  3.84417  2.20672  3.85141  3.00370  3.02852
2.11757  2.87074  2.99088  5.23162  3.91297    116 s - - -
         2.68618  4.42225  2.77519  2.73123  3.46354  2.40513  3.72494
3.29354  2.67741  2.69355  4.24690  2.90347  2.73739  3.18146  2.89801
2.37887  2.77519  2.98518  4.58477  3.61503
         0.02620  4.05089  4.77324  0.61958  0.77255  0.38139  1.14858
    114  2.74286  4.61269  3.43181  2.82542  3.77226  3.62635  3.77852
3.12238  2.08075  2.13390  2.57368  3.25998  3.99747  3.00818  2.27868
2.88223  2.96071  2.89315  5.12943  3.89100    117 k - - -
         2.68618  4.42225  2.77519  2.73123  3.46354  2.40513  3.72494
3.29354  2.67741  2.69355  4.24690  2.90347  2.73739  3.18146  2.89801
2.37887  2.77519  2.98518  4.58477  3.61503
         0.02187  4.22952  4.95186  0.61958  0.77255  0.48576  0.95510
    115  2.07984  4.73644  3.00502  2.57965  4.31143  2.45469  3.81966
3.74597  2.63201  3.33675  4.14055  3.05091  3.84232  2.15339  3.07497
2.02280  2.89620  3.33588  5.55070  4.22236    118 s - - -
         2.68618  4.42225  2.77519  2.73123  3.46354  2.40513  3.72494
3.29354  2.67741  2.69355  4.24690  2.90347  2.73739  3.18146  2.89801
2.37887  2.77519  2.98518  4.58477  3.61503
         0.02187  4.22952  4.95186  0.61958  0.77255  0.48576  0.95510
    116  3.05481  4.46019  4.51715  3.99066  2.92230  4.12806  4.21476
1.41492  3.76155  2.09069  3.31170  4.12167  4.47476  4.00293  3.89624
3.48030  3.29632  2.39034  2.01390  3.17301    119 i - - -
         2.68618  4.42225  2.77519  2.73123  3.46354  2.40513  3.72494
3.29354  2.67741  2.69355  4.24690  2.90347  2.73739  3.18146  2.89801
2.37887  2.77519  2.98518  4.58477  3.61503
         0.02187  4.22952  4.95186  0.61958  0.77255  0.48576  0.95510
    117  2.73897  4.28149  3.92218  3.35495  2.35417  3.77378  3.90354
2.19387  3.22958  2.37942  3.35585  3.62755  4.13868  3.50037  2.40360
3.05093  2.96922  2.52372  4.56152  2.32470    120 i - - -
         2.68618  4.42225  2.77519  2.73123  3.46354  2.40513  3.72494
3.29354  2.67741  2.69355  4.24690  2.90347  2.73739  3.18146  2.89801
2.37887  2.77519  2.98518  4.58477  3.61503
         0.02187  4.22952  4.95186  0.61958  0.77255  0.48576  0.95510
    118  2.70327  5.04142  2.19595  2.01423  4.30400  3.41082  3.67855
3.75578  2.49071  3.31750  4.11550  2.90715  3.85209  2.81526  2.98018
2.69098  2.25899  3.39307  5.51626  2.90308    121 e - - -
```

```
          2.68618  4.42225  2.77519  2.73123  3.46354  2.40513  3.72494
3.29354  2.67741  2.69355  4.24690  2.90347  2.73739  3.18146  2.89801
2.37887  2.77519  2.98518  4.58477  3.61503
          0.02187  4.22952  4.95186  0.61958  0.77255  0.48576  0.95510
    119   2.68206  4.60529  3.21080  2.89025  3.98193  3.42071  4.04286
3.15297  2.93240  3.05772  4.01609  1.44134  3.99584  3.29726  3.29698
2.82813  3.02770  1.98080  5.41640  4.08289    122 n - - -
          2.68618  4.42225  2.77519  2.73123  3.46354  2.40513  3.72494
3.29354  2.67741  2.69355  4.24690  2.90347  2.73739  3.18146  2.89801
2.37887  2.77519  2.98518  4.58477  3.61503
          0.02187  4.22952  4.95186  0.61958  0.77255  0.48576  0.95510
    120   2.86371  4.40414  4.05786  3.51595  3.37557  3.92403  4.27678
1.83568  3.39669  1.69185  3.29129  3.82935  2.16071  3.68945  3.65608
3.23217  3.11381  2.27790  5.06519  3.86372    123 l - - -
          2.68618  4.42225  2.77519  2.73123  3.46354  2.40513  3.72494
3.29354  2.67741  2.69355  4.24690  2.90347  2.73739  3.18146  2.89801
2.37887  2.77519  2.98518  4.58477  3.61503
          0.02187  4.22952  4.95186  0.61958  0.77255  0.48576  0.95510
    121   2.75135  4.72376  3.27800  2.73835  3.69187  3.57069  3.74323
3.34727  2.50877  2.96934  3.84941  3.19194  2.54675  2.98892  2.17728
2.84381  2.98635  3.07537  5.07523  2.16488    124 y - - -
          2.68618  4.42225  2.77519  2.73123  3.46354  2.40513  3.72494
3.29354  2.67741  2.69355  4.24690  2.90347  2.73739  3.18146  2.89801
2.37887  2.77519  2.98518  4.58477  3.61503
          0.02187  4.22952  4.95186  0.61958  0.77255  0.48576  0.95510
    122   2.71528  5.04659  2.87855  2.42928  4.41117  2.43382  3.68199
3.85452  2.01532  3.38550  4.17633  2.95441  3.86841  2.28631  2.81846
2.13570  2.96057  3.46868  5.55291  4.19636    125 k - - -
          2.68618  4.42225  2.77519  2.73123  3.46354  2.40513  3.72494
3.29354  2.67741  2.69355  4.24690  2.90347  2.73739  3.18146  2.89801
2.37887  2.77519  2.98518  4.58477  3.61503
          0.02187  4.22952  4.95186  0.61958  0.77255  0.48576  0.95510
    123   2.41201  4.34900  3.53331  3.31502  4.52014  1.10000  4.42257
3.90018  3.41011  3.62455  4.47307  3.47292  3.86485  3.70514  3.70593
2.59051  1.82460  3.35432  5.84757  4.65193    126 g - - -
          2.68618  4.42225  2.77519  2.73123  3.46354  2.40513  3.72494
3.29354  2.67741  2.69355  4.24690  2.90347  2.73739  3.18146  2.89801
2.37887  2.77519  2.98518  4.58477  3.61503
          0.02187  4.22952  4.95186  0.61958  0.77255  0.48576  0.95510
    124   3.10274  4.56397  4.61000  4.29848  3.84091  4.08289  5.04845
2.10527  4.17294  2.47397  3.76444  4.42777  4.63408  4.51346  4.34955
3.62054  3.46208  0.66803  5.68088  4.42926    127 V - - -
          2.68618  4.42225  2.77519  2.73123  3.46354  2.40513  3.72494
3.29354  2.67741  2.69355  4.24690  2.90347  2.73739  3.18146  2.89801
2.37887  2.77519  2.98518  4.58477  3.61503
          0.02187  4.22952  4.95186  0.61958  0.77255  0.48576  0.95510
    125   2.56567  4.31083  3.67590  3.17728  3.67632  3.46598  4.08207
2.04387  3.10890  2.69259  3.63134  3.48064  3.99424  3.42356  3.42403
2.18992  1.76143  2.56046  5.15243  3.92582    128 t - - -
          2.68618  4.42225  2.77519  2.73123  3.46354  2.40513  3.72494
3.29354  2.67741  2.69355  4.24690  2.90347  2.73739  3.18146  2.89801
2.37887  2.77519  2.98518  4.58477  3.61503
          0.02187  4.22952  4.95186  0.61958  0.77255  0.48576  0.95510
```

```
    126   2.78516  4.27565  4.24571  3.70627  1.84617  3.86268  4.26037
2.35965  3.59341  2.20947  3.31408  3.90442  4.27203  3.82286  3.78875
3.19086  2.32093  1.75830  4.89584  3.62313    129 v - - -
          2.68618  4.42225  2.77519  2.73123  3.46354  2.40513  3.72494
3.29354  2.67741  2.69355  4.24690  2.90347  2.73739  3.18146  2.89801
2.37887  2.77519  2.98518  4.58477  3.61503
          0.02187  4.22952  4.95186  0.61958  0.77255  0.48576  0.95510
    127   2.68309  4.98998  2.13970  2.40578  4.51388  2.44724  3.79084
3.98191  2.64467  3.52565  4.31551  2.93142  2.48230  2.93328  3.14482
2.00106  2.98509  3.54538  5.70631  4.31669    130 s - - -
          2.68618  4.42225  2.77519  2.73123  3.46354  2.40513  3.72494
3.29354  2.67741  2.69355  4.24690  2.90347  2.73739  3.18146  2.89801
2.37887  2.77519  2.98518  4.58477  3.61503
          0.20483  4.22952  1.76812  0.61958  0.77255  0.48576  0.95510
    128   2.66285  4.80383  2.84426  2.53094  4.26575  3.30477  3.77762
3.75692  2.50818  3.33413  4.18556  3.00551  3.85204  1.72339  2.87720
1.93373  2.97492  3.37583  5.49970  4.16076    131 q - - -
          2.68618  4.42225  2.77519  2.73123  3.46354  2.40513  3.72494
3.29354  2.67741  2.69355  4.24690  2.90347  2.73739  3.18146  2.89801
2.37887  2.77519  2.98518  4.58477  3.61503
          0.02620  4.05089  4.77324  0.61958  0.77255  0.67925  0.70724
    129   3.49373  4.84424  4.48374  4.23523  0.75941  4.13528  3.96859
2.99901  4.13862  2.32936  3.70472  4.22448  4.59221  4.25814  4.21409
3.75232  3.79615  3.00228  4.17058  2.51762    132 f - - -
          2.68618  4.42225  2.77519  2.73123  3.46354  2.40513  3.72494
3.29354  2.67741  2.69355  4.24690  2.90347  2.73739  3.18146  2.89801
2.37887  2.77519  2.98518  4.58477  3.61503
          0.02620  4.05089  4.77324  0.61958  0.77255  0.67925  0.70724
    130   2.68391  4.28246  3.81863  3.28342  3.37103  3.69922  4.08261
1.95342  3.17343  1.86292  3.33801  3.60346  4.11292  3.48476  3.45440
2.17975  2.94652  2.24800  4.96202  3.72781    133 l - - -
          2.68618  4.42225  2.77519  2.73123  3.46354  2.40513  3.72494
3.29354  2.67741  2.69355  4.24690  2.90347  2.73739  3.18146  2.89801
2.37887  2.77519  2.98518  4.58477  3.61503
          0.02620  4.05089  4.77324  0.61958  0.77255  0.67925  0.70724
    131   2.91599  5.37213  1.71436  2.18171  4.70001  3.24699  3.80860
4.25805  2.80927  3.78400  4.63718  1.46806  3.86825  2.98263  3.37582
2.83235  3.21954  3.83145  5.90969  4.42781    134 n - - -
          2.68618  4.42225  2.77519  2.73123  3.46354  2.40513  3.72494
3.29354  2.67741  2.69355  4.24690  2.90347  2.73739  3.18146  2.89801
2.37887  2.77519  2.98518  4.58477  3.61503
          0.02620  4.05089  4.77324  0.61958  0.77255  0.67925  0.70724
    132   1.58942  4.63797  2.99330  2.65267  4.14343  3.29006  3.84583
3.51840  2.61437  3.16280  4.05483  3.09043  3.85287  2.05932  2.97417
2.68759  2.92976  3.17604  5.44373  4.14531    135 a - - -
          2.68618  4.42225  2.77519  2.73123  3.46354  2.40513  3.72494
3.29354  2.67741  2.69355  4.24690  2.90347  2.73739  3.18146  2.89801
2.37887  2.77519  2.98518  4.58477  3.61503
          0.02620  4.05089  4.77324  0.61958  0.77255  0.38139  1.14858
    133   3.17590  4.56654  4.68945  4.21794  1.31698  4.26121  4.36033
2.36614  4.08557  2.01012  3.32241  4.30694  4.61999  4.22951  4.20714
3.63522  3.44142  1.67999  4.67970  3.18011    136 f - - -
          2.68618  4.42225  2.77519  2.73123  3.46354  2.40513  3.72494
3.29354  2.67741  2.69355  4.24690  2.90347  2.73739  3.18146  2.89801
2.37887  2.77519  2.98518  4.58477  3.61503
```

```
          0.02187  4.22952  4.95186  0.61958  0.77255  0.48576  0.95510
    134   2.81144  5.20933  1.93064  2.31679  4.59507  3.34849  3.78745
4.06621  2.67215  3.60585  4.41163  1.81654  3.88979  2.93355  3.19401
2.77175  2.15642  3.65317  5.78243  4.35819    137 n - - -
          2.68618  4.42225  2.77519  2.73123  3.46354  2.40513  3.72494
3.29354  2.67741  2.69355  4.24690  2.90347  2.73739  3.18146  2.89801
2.37887  2.77519  2.98518  4.58477  3.61503
          0.02187  4.22952  4.95186  0.61958  0.77255  0.48576  0.95510
    135   2.60946  4.73551  2.87130  2.63197  4.51618  1.66345  3.98251
3.98491  2.86920  3.57484  4.38845  2.19915  2.29501  3.16445  3.31208
2.67979  2.99012  3.50728  5.75984  4.42871    138 g - - -
          2.68618  4.42225  2.77519  2.73123  3.46354  2.40513  3.72494
3.29354  2.67741  2.69355  4.24690  2.90347  2.73739  3.18146  2.89801
2.37887  2.77519  2.98518  4.58477  3.61503
          0.02187  4.22952  4.95186  0.61958  0.77255  0.48576  0.95510
    136   2.76892  4.64153  3.24818  2.89053  3.80412  3.54769  3.99816
2.10859  2.88593  2.86568  3.86772  1.48970  4.05613  3.26873  3.24349
2.92496  3.06672  2.74800  5.28114  3.93563    139 n - - -
          2.68618  4.42225  2.77519  2.73123  3.46354  2.40513  3.72494
3.29354  2.67741  2.69355  4.24690  2.90347  2.73739  3.18146  2.89801
2.37887  2.77519  2.98518  4.58477  3.61503
          0.31255  4.22952  1.37097  0.61958  0.77255  0.48576  0.95510
    137   3.03982  5.00607  3.22069  2.82865  4.40932  3.52113  3.80083
3.84324  0.94188  3.39238  4.35009  3.24980  4.01646  2.98829  2.44687
3.07710  3.28773  3.54695  5.43390  4.25734    140 k - - -
          2.68618  4.42225  2.77519  2.73123  3.46354  2.40513  3.72494
3.29354  2.67741  2.69355  4.24690  2.90347  2.73739  3.18146  2.89801
2.37887  2.77519  2.98518  4.58477  3.61503
          0.02914  3.94610  4.66845  0.61958  0.77255  0.76495  0.62616
    138   3.35268  4.73293  4.33961  4.06829  0.89854  4.02483  3.91094
2.86751  3.97351  2.22071  3.58693  4.10246  4.48192  4.12126  4.07505
3.61871  3.65393  2.86358  4.13857  2.49652    141 f - - -
          2.68618  4.42225  2.77519  2.73123  3.46354  2.40513  3.72494
3.29354  2.67741  2.69355  4.24690  2.90347  2.73739  3.18146  2.89801
2.37887  2.77519  2.98518  4.58477  3.61503
          0.02914  3.94610  4.66845  0.61958  0.77255  0.76495  0.62616
    139   3.03738  4.44870  4.47988  3.97913  3.29669  4.20587  4.67171
1.96139  3.81779  1.38197  3.16210  4.23607  4.55007  4.09648  4.02266
3.58135  3.30535  1.40379  5.23115  4.02421    142 l - - -
          2.68618  4.42225  2.77519  2.73123  3.46354  2.40513  3.72494
3.29354  2.67741  2.69355  4.24690  2.90347  2.73739  3.18146  2.89801
2.37887  2.77519  2.98518  4.58477  3.61503
          0.02914  3.94610  4.66845  0.61958  0.77255  0.76495  0.62616
    140   2.82018  5.18010  1.67750  2.22363  4.23623  3.27420  2.18634
3.96786  2.62300  3.49837  4.35219  2.78292  3.83059  2.88755  3.12388
2.76618  3.09221  3.59268  5.52441  4.05166    143 d - - -
          2.68618  4.42225  2.77519  2.73123  3.46354  2.40513  3.72494
3.29354  2.67741  2.69355  4.24690  2.90347  2.73739  3.18146  2.89801
2.37887  2.77519  2.98518  4.58477  3.61503
          0.02914  3.94610  4.66845  0.61958  0.77255  0.76495  0.62616
    141   2.67695  4.93729  1.70254  2.27312  4.45198  3.16901  3.78491
3.94217  2.74311  3.53206  4.37441  2.80552  3.78416  2.96434  3.25811
1.78582  3.01255  3.51688  5.70360  4.28997    144 d - - -
```

```
         2.68618  4.42225  2.77519  2.73123  3.46354  2.40513  3.72494
3.29354  2.67741  2.69355  4.24690  2.90347  2.73739  3.18146  2.89801
2.37887  2.77519  2.98518  4.58477  3.61503
         0.02914  3.94610  4.66845  0.61958  0.77255  0.76495  0.62616
    142  2.76016  4.57004  3.40603  2.88257  3.71979  3.60899  3.83482
1.99534  1.83606  2.60729  3.64085  3.29925  4.01845  3.07196  2.78380
2.93508  2.99913  2.56475  5.15700  3.90106    145 k - - -
         2.68618  4.42225  2.77519  2.73123  3.46354  2.40513  3.72494
3.29354  2.67741  2.69355  4.24690  2.90347  2.73739  3.18146  2.89801
2.37887  2.77519  2.98518  4.58477  3.61503
         0.02914  3.94610  4.66845  0.61958  0.77255  0.76495  0.62616
    143  2.75286  4.61598  3.06820  1.92746  2.10375  3.51529  3.76873
3.02506  2.78905  2.64402  3.67479  3.17757  3.96958  3.12011  3.16845
2.87752  3.00721  2.83110  4.84452  3.38891    146 e - - -
         2.68618  4.42225  2.77519  2.73123  3.46354  2.40513  3.72494
3.29354  2.67741  2.69355  4.24690  2.90347  2.73739  3.18146  2.89801
2.37887  2.77519  2.98518  4.58477  3.61503
         0.02914  3.94610  4.66845  0.61958  0.77255  0.76495  0.62616
    144  2.69692  4.29939  3.91259  3.44140  3.53312  3.68321  4.29435
1.63721  3.31667  2.28355  3.43779  3.72121  4.17214  3.65983  3.58810
3.05395  1.98593  2.02084  5.20718  3.97974    147 i - - -
         2.68618  4.42225  2.77519  2.73123  3.46354  2.40513  3.72494
3.29354  2.67741  2.69355  4.24690  2.90347  2.73739  3.18146  2.89801
2.37887  2.77519  2.98518  4.58477  3.61503
         0.02914  3.94610  4.66845  0.61958  0.77255  0.76495  0.62616
    145  0.91266  4.22954  3.53793  3.33284  4.12623  3.05206  4.34611
3.26281  3.35100  3.14100  4.16493  3.46366  3.79826  3.68547  3.59632
2.57929  2.86175  2.90448  5.54604  4.35453    148 a - - -
         2.68618  4.42225  2.77519  2.73123  3.46354  2.40513  3.72494
3.29354  2.67741  2.69355  4.24690  2.90347  2.73739  3.18146  2.89801
2.37887  2.77519  2.98518  4.58477  3.61503
         0.02914  3.94610  4.66845  0.61958  0.77255  0.76495  0.62616
    146  3.08851  4.48711  4.43964  4.05840  3.42425  4.14544  4.77306
0.99118  3.88795  1.99552  3.34844  4.29344  4.56981  4.22881  4.07612
3.64805  3.38646  1.84989  5.33013  4.07461    149 i - - -
         2.68618  4.42225  2.77519  2.73123  3.46354  2.40513  3.72494
3.29354  2.67741  2.69355  4.24690  2.90347  2.73739  3.18146  2.89801
2.37887  2.77519  2.98518  4.58477  3.61503
         0.02914  3.94610  4.66845  0.61958  0.77255  0.76495  0.62616
    147  2.66500  4.80687  2.67014  1.77437  4.21524  3.28207  3.75160
3.52666  2.57175  3.21328  4.09247  2.92256  3.82424  2.93168  2.99076
2.70290  1.98019  3.19936  5.48813  4.15391    150 e - - -
         2.68618  4.42225  2.77519  2.73123  3.46354  2.40513  3.72494
3.29354  2.67741  2.69355  4.24690  2.90347  2.73739  3.18146  2.89801
2.37887  2.77519  2.98518  4.58477  3.61503
         0.02914  3.94610  4.66845  0.61958  0.77255  0.76495  0.62616
    148  2.64031  4.39402  3.43477  2.99688  3.13380  3.48496  3.80664
2.91135  2.89870  2.61256  3.60532  3.34198  3.96986  3.26208  3.20627
2.83553  2.01728  2.69240  4.68245  2.07191    151 t - - -
         2.68618  4.42225  2.77519  2.73123  3.46354  2.40513  3.72494
3.29354  2.67741  2.69355  4.24690  2.90347  2.73739  3.18146  2.89801
2.37887  2.77519  2.98518  4.58477  3.61503
         0.02914  3.94610  4.66845  0.61958  0.77255  0.76495  0.62616
```

```
    149   1.41712  4.16185  3.33448  3.10433  4.28404  1.72119  4.21354
3.63702  3.21109  3.38009  4.23462  3.27592  3.67972  3.49590  3.51669
2.40659  2.71856  3.11936  5.62600  4.42240    152 a - - -
          2.68618  4.42225  2.77519  2.73123  3.46354  2.40513  3.72494
3.29354  2.67741  2.69355  4.24690  2.90347  2.73739  3.18146  2.89801
2.37887  2.77519  2.98518  4.58477  3.61503
          0.02914  3.94610  4.66845  0.61958  0.77255  0.34396  1.23429
    150   1.54676  4.30582  3.66276  3.18456  3.78759  3.36985  4.11770
2.22049  3.13588  2.81564  3.73797  3.46175  3.94923  3.44206  3.45813
2.00541  2.87017  2.67615  5.24547  4.01867    153 a - - -
          2.68618  4.42225  2.77519  2.73123  3.46354  2.40513  3.72494
3.29354  2.67741  2.69355  4.24690  2.90347  2.73739  3.18146  2.89801
2.37887  2.77519  2.98518  4.58477  3.61503
          0.02187  4.22952  4.95186  0.61958  0.77255  0.48576  0.95510
    151   3.11157  4.46419  4.66525  4.14967  1.87899  4.18776  3.98491
1.88400  3.97904  2.12995  3.32794  4.14294  4.50356  4.07014  4.04427
3.51143  3.33837  2.59711  1.97921  2.69865    154 f - - -
          2.68618  4.42225  2.77519  2.73123  3.46354  2.40513  3.72494
3.29354  2.67741  2.69355  4.24690  2.90347  2.73739  3.18146  2.89801
2.37887  2.77519  2.98518  4.58477  3.61503
          0.02187  4.22952  4.95186  0.61958  0.77255  0.48576  0.95510
    152   2.66346  4.93310  2.92758  2.46077  4.31901  3.40445  3.70123
3.75374  2.06104  3.31456  4.11139  2.38210  3.85498  2.83781  2.88705
2.12464  2.22274  3.37592  5.50767  4.15918    155 k - - -
          2.68618  4.42225  2.77519  2.73123  3.46354  2.40513  3.72494
3.29354  2.67741  2.69355  4.24690  2.90347  2.73739  3.18146  2.89801
2.37887  2.77519  2.98518  4.58477  3.61503
          0.02187  4.22952  4.95186  0.61958  0.77255  0.48576  0.95510
    153   2.03792  4.80700  3.05436  2.60641  4.28954  3.39549  3.79116
3.68715  1.92162  3.28915  4.11468  3.07933  2.13181  2.95047  2.89438
2.71696  2.95379  3.31929  5.50513  4.19831    156 k - - -
          2.68618  4.42225  2.77519  2.73123  3.46354  2.40513  3.72494
3.29354  2.67741  2.69355  4.24690  2.90347  2.73739  3.18146  2.89801
2.37887  2.77519  2.98518  4.58477  3.61503
          0.02187  4.22952  4.95186  0.61958  0.77255  0.48576  0.95510
    154   3.58296  4.94778  4.76280  4.41894  3.31381  4.43210  4.96505
2.55063  4.18445  0.53374  3.25075  4.66084  4.81524  4.46461  4.29594
4.06845  3.86586  2.67792  5.30656  4.10752    157 L - - -
          2.68618  4.42225  2.77519  2.73123  3.46354  2.40513  3.72494
3.29354  2.67741  2.69355  4.24690  2.90347  2.73739  3.18146  2.89801
2.37887  2.77519  2.98518  4.58477  3.61503
          0.02187  4.22952  4.95186  0.61958  0.77255  0.48576  0.95510
    155   2.80171  4.45077  3.95563  3.48862  3.53547  3.72901  4.31611
2.56588  3.33294  1.49949  3.46983  3.76775  4.22690  3.68682  3.59231
3.10509  1.61731  2.44167  5.21237  3.98936    158 l - - -
          2.68618  4.42225  2.77519  2.73123  3.46354  2.40513  3.72494
3.29354  2.67741  2.69355  4.24690  2.90347  2.73739  3.18146  2.89801
2.37887  2.77519  2.98518  4.58477  3.61503
          0.02187  4.22952  4.95186  0.61958  0.77255  0.48576  0.95510
    156   2.59637  4.60965  3.18348  2.66385  3.93637  3.42860  3.78808
3.30359  2.62867  2.97414  3.82527  3.12604  3.88364  2.29025  3.04202
2.10439  2.32584  2.39556  5.27104  3.98289    159 s - - -
          2.68618  4.42225  2.77519  2.73123  3.46354  2.40513  3.72494
3.29354  2.67741  2.69355  4.24690  2.90347  2.73739  3.18146  2.89801
2.37887  2.77519  2.98518  4.58477  3.61503
```

0.02187 4.22952 4.95186 0.61958 0.77255 0.48576 0.95510
157 2.77417 5.14263 2.11020 2.34426 4.43963 3.37518 2.63449
3.95622 2.59492 3.49482 4.29834 2.89328 3.88004 2.89194 3.09450
1.67109 3.03873 3.56245 5.64999 4.23429 160 s - - -
2.68618 4.42225 2.77519 2.73123 3.46354 2.40513 3.72494
3.29354 2.67741 2.69355 4.24690 2.90347 2.73739 3.18146 2.89801
2.37887 2.77519 2.98518 4.58477 3.61503
0.02187 4.22952 4.95186 0.61958 0.77255 0.48576 0.95510
158 2.79700 4.37009 3.86753 3.33983 3.41681 3.83583 4.14350
1.69744 3.22776 1.82235 3.39124 2.48374 4.21604 3.54839 3.50916
3.13346 3.04061 2.26619 5.01436 3.77114 161 i - - -
2.68618 4.42225 2.77519 2.73123 3.46354 2.40513 3.72494
3.29354 2.67741 2.69355 4.24690 2.90347 2.73739 3.18146 2.89801
2.37887 2.77519 2.98518 4.58477 3.61503
0.02187 4.22952 4.95186 0.61958 0.77255 0.48576 0.95510
159 2.66024 4.43376 3.45160 2.87796 3.56155 3.60340 2.82339
2.26267 2.26945 2.61021 2.57489 3.29855 3.98166 3.11500 3.06434
2.85278 2.88843 2.69434 4.98821 3.74245 162 i - - -
2.68618 4.42225 2.77519 2.73123 3.46354 2.40513 3.72494
3.29354 2.67741 2.69355 4.24690 2.90347 2.73739 3.18146 2.89801
2.37887 2.77519 2.98518 4.58477 3.61503
0.02187 4.22952 4.95186 0.61958 0.77255 0.48576 0.95510
160 2.63428 4.70343 2.95837 2.71892 4.39733 3.27446 4.01409
3.81239 2.86726 3.48383 4.33975 1.35139 3.90701 3.21640 3.26167
2.72311 1.98401 3.39153 5.68632 4.35911 163 n - - -
2.68618 4.42225 2.77519 2.73123 3.46354 2.40513 3.72494
3.29354 2.67741 2.69355 4.24690 2.90347 2.73739 3.18146 2.89801
2.37887 2.77519 2.98518 4.58477 3.61503
0.02187 4.22952 4.95186 0.61958 0.77255 0.48576 0.95510
161 2.13146 5.09201 2.88164 2.41783 4.42634 3.44717 3.66234
3.86876 2.00015 3.39033 4.17877 2.37345 3.87207 2.19312 2.77275
2.71491 2.96657 3.48604 5.54948 4.19177 164 k - - -
2.68618 4.42225 2.77519 2.73123 3.46354 2.40513 3.72494
3.29354 2.67741 2.69355 4.24690 2.90347 2.73739 3.18146 2.89801
2.37887 2.77519 2.98518 4.58477 3.61503
0.02187 4.22952 4.95186 0.61958 0.77255 0.48576 0.95510
162 1.55038 4.30993 3.59911 3.41178 4.59260 1.08619 4.50829
3.98201 3.54774 3.70958 4.54671 3.51063 3.85057 3.80559 3.82800
2.56133 2.89369 3.38751 5.92019 4.74419 165 g - - -
2.68618 4.42225 2.77519 2.73123 3.46354 2.40513 3.72494
3.29354 2.67741 2.69355 4.24690 2.90347 2.73739 3.18146 2.89801
2.37887 2.77519 2.98518 4.58477 3.61503
0.02187 4.22952 4.95186 0.61958 0.77255 0.48576 0.95510
163 2.02531 5.11218 2.07642 2.34803 4.56134 2.31295 3.78307
4.03292 2.65547 3.56895 4.36456 2.23458 3.87037 2.92599 3.17158
2.72849 3.03854 3.60740 5.74818 4.33852 166 a - - -
2.68618 4.42225 2.77519 2.73123 3.46354 2.40513 3.72494
3.29354 2.67741 2.69355 4.24690 2.90347 2.73739 3.18146 2.89801
2.37887 2.77519 2.98518 4.58477 3.61503
0.02187 4.22952 4.95186 0.61958 0.77255 0.48576 0.95510
164 1.38990 4.27007 3.75750 3.42463 4.28969 3.12650 4.40225
3.46299 3.39995 3.32007 4.21824 3.52791 3.86601 3.70255 3.68156
2.57664 1.35793 3.04135 5.69786 4.50061 167 t - - -

```
          2.68618  4.42225  2.77519  2.73123  3.46354  2.40513  3.72494
3.29354  2.67741  2.69355  4.24690  2.90347  2.73739  3.18146  2.89801
2.37887  2.77519  2.98518  4.58477  3.61503
          0.02187  4.22952  4.95186  0.61958  0.77255  0.48576  0.95510
    165   1.90876  4.91295  1.93577  2.47095  4.56746  1.83546  3.91309
4.01757  2.83754  3.60277  4.41727  2.97806  3.87935  3.08150  3.33890
2.72077  3.04139  3.56626  5.80118  4.42425    168 g - - -
          2.68618  4.42225  2.77519  2.73123  3.46354  2.40513  3.72494
3.29354  2.67741  2.69355  4.24690  2.90347  2.73739  3.18146  2.89801
2.37887  2.77519  2.98518  4.58477  3.61503
          0.02187  4.22952  4.95186  0.61958  0.77255  0.48576  0.95510
    166   2.66359  4.34599  3.84512  3.41243  3.65568  3.56862  4.26433
1.48304  3.32595  2.60383  3.64653  3.66527  4.11995  3.65185  3.59829
1.76728  3.00561  2.38411  5.22111  3.96819    169 i - - -
          2.68623  4.42230  2.77524  2.73128  3.46358  2.40465  3.72499
3.29359  2.67745  2.69359  4.24694  2.90351  2.73744  3.18151  2.89805
2.37891  2.77524  2.98523  4.58481  3.61508
          0.56564  1.34189  1.76812  0.19269  1.74147  0.48576  0.95510
    167   1.58745  4.19449  3.58892  3.25540  4.14549  3.05506  4.25632
3.28139  3.23118  3.15783  4.07311  3.40227  3.77846  3.54886  3.52228
2.50135  1.46173  2.89034  5.56118  4.35321    171 t - - -
          2.68618  4.42225  2.77519  2.73123  3.46354  2.40513  3.72494
3.29354  2.67741  2.69355  4.24690  2.90347  2.73739  3.18146  2.89801
2.37887  2.77519  2.98518  4.58477  3.61503
          0.02620  4.05089  4.77324  0.61958  0.77255  0.67925  0.70724
    168   2.77591  4.35263  3.89013  3.35108  3.33724  3.81131  4.15797
1.91246  3.23530  1.72719  3.25939  3.68674  2.50750  3.54470  3.51449
3.11527  3.02752  2.24808  5.00635  3.79307    172 l - - -
          2.68618  4.42225  2.77519  2.73123  3.46354  2.40513  3.72494
3.29354  2.67741  2.69355  4.24690  2.90347  2.73739  3.18146  2.89801
2.37887  2.77519  2.98518  4.58477  3.61503
          0.02620  4.05089  4.77324  0.61958  0.77255  0.67925  0.70724
    169   2.10761  4.26943  4.02484  3.48812  3.41223  3.76201  4.24881
2.25656  3.38521  2.19173  2.25567  3.76152  4.19126  3.66632  3.64295
3.08958  2.99328  1.72889  5.06288  3.86834    173 v - - -
          2.68618  4.42225  2.77519  2.73123  3.46354  2.40513  3.72494
3.29354  2.67741  2.69355  4.24690  2.90347  2.73739  3.18146  2.89801
2.37887  2.77519  2.98518  4.58477  3.61503
          0.02620  4.05089  4.77324  0.61958  0.77255  0.67925  0.70724
    170   3.34097  4.68351  4.70814  4.23461  1.48852  4.41049  4.24448
2.43571  4.08402  1.26413  3.08450  4.34320  4.67115  4.18134  4.18851
3.78006  3.57296  2.53087  4.47593  2.94952    174 l - - -
          2.68618  4.42225  2.77519  2.73123  3.46354  2.40513  3.72494
3.29354  2.67741  2.69355  4.24690  2.90347  2.73739  3.18146  2.89801
2.37887  2.77519  2.98518  4.58477  3.61503
          0.02620  4.05089  4.77324  0.61958  0.77255  0.38139  1.14858
    171   3.47313  4.75650  5.14178  4.61008  3.23959  4.78406  5.13343
1.61003  4.44064  0.85782  3.03271  4.84731  4.95195  4.55418  4.53747
4.17093  3.70344  2.18503  5.36838  4.27921    175 l - - -
          2.68618  4.42225  2.77519  2.73123  3.46354  2.40513  3.72494
3.29354  2.67741  2.69355  4.24690  2.90347  2.73739  3.18146  2.89801
2.37887  2.77519  2.98518  4.58477  3.61503
          0.02187  4.22952  4.95186  0.61958  0.77255  0.48576  0.95510
```

```
    172   2.67981  4.88680  2.96315  2.55825  4.43500  2.28184  3.78975
3.87147  1.69085  3.42775  4.23363  3.04219  3.88444  2.93700  2.89201
2.04488  2.97756  3.46149  5.60066  4.27515    176 k - - -
          2.68618  4.42225  2.77519  2.73123  3.46354  2.40513  3.72494
3.29354  2.67741  2.69355  4.24690  2.90347  2.73739  3.18146  2.89801
2.37887  2.77519  2.98518  4.58477  3.61503
          0.02187  4.22952  4.95186  0.61958  0.77255  0.48576  0.95510
    173   2.41597  4.40289  3.37428  3.02961  4.38468  1.68803  4.17288
3.80010  3.09281  3.45925  4.28013  3.31347  2.15789  3.40055  3.46200
2.56617  1.93308  3.30041  5.69669  4.45092    177 g - - -
          2.68618  4.42225  2.77519  2.73123  3.46354  2.40513  3.72494
3.29354  2.67741  2.69355  4.24690  2.90347  2.73739  3.18146  2.89801
2.37887  2.77519  2.98518  4.58477  3.61503
          0.02187  4.22952  4.95186  0.61958  0.77255  0.48576  0.95510
    174   3.20496  4.53842  4.91345  4.33776  2.14004  4.43426  4.69862
1.70936  4.19477  1.62696  1.78202  4.49769  4.67005  4.27769  4.26897
3.76700  3.42803  2.32710  5.03450  3.87294    178 l - - -
          2.68621  4.42228  2.77522  2.73126  3.46357  2.40481  3.72497
3.29357  2.67744  2.69358  4.24693  2.90350  2.73743  3.18149  2.89804
2.37890  2.77522  2.98521  4.58480  3.61506
          0.20483  1.72517  4.95186  0.25055  1.50674  0.48576  0.95510
    175   2.79868  4.29518  4.16718  3.60561  2.29506  3.87744  4.19476
2.48179  3.48938  2.04354  1.98202  3.84749  4.24606  3.71990  3.69522
3.18150  2.19151  2.38419  4.84841  3.61955    180 m - - -
          2.68618  4.42225  2.77519  2.73123  3.46354  2.40513  3.72494
3.29354  2.67741  2.69355  4.24690  2.90347  2.73739  3.18146  2.89801
2.37887  2.77519  2.98518  4.58477  3.61503
          0.02187  4.22952  4.95186  0.61958  0.77255  0.48576  0.95510
    176   2.65053  4.77318  3.06683  2.62865  4.27872  2.46995  3.81092
3.67913  1.95940  3.28643  4.11219  3.09341  3.89036  2.97601  2.92272
2.71303  1.77693  3.30810  5.50436  4.20302    181 t - - -
          2.68618  4.42225  2.77519  2.73123  3.46354  2.40513  3.72494
3.29354  2.67741  2.69355  4.24690  2.90347  2.73739  3.18146  2.89801
2.37887  2.77519  2.98518  4.58477  3.61503
          0.02187  4.22952  4.95186  0.61958  0.77255  0.48576  0.95510
    177   2.91583  4.35597  4.41748  3.88447  3.60311  4.08956  4.59832
1.58154  3.78304  2.34091  3.47935  4.12293  4.48090  4.05400  4.01456
3.42981  1.89884  1.49385  5.31640  4.11185    182 v - - -
          2.68618  4.42225  2.77519  2.73123  3.46354  2.40513  3.72494
3.29354  2.67741  2.69355  4.24690  2.90347  2.73739  3.18146  2.89801
2.37887  2.77519  2.98518  4.58477  3.61503
          0.02187  4.22952  4.95186  0.61958  0.77255  0.48576  0.95510
    178   1.58961  4.31242  3.59475  3.41152  4.59696  1.05922  4.51101
3.98670  3.55106  3.71471  4.55263  3.51131  3.85215  3.80883  3.83069
2.56377  2.89677  3.39138  5.92373  4.74813    183 g - - -
          2.68618  4.42225  2.77519  2.73123  3.46354  2.40513  3.72494
3.29354  2.67741  2.69355  4.24690  2.90347  2.73739  3.18146  2.89801
2.37887  2.77519  2.98518  4.58477  3.61503
          0.02187  4.22952  4.95186  0.61958  0.77255  0.48576  0.95510
    179   2.74032  4.50981  3.64564  3.29574  3.78253  1.48698  4.24381
3.09697  3.23727  1.53427  3.84360  3.59400  4.09557  3.59887  3.52130
2.95707  3.10411  2.88186  5.29530  4.03337    184 g - - -
          2.68618  4.42225  2.77519  2.73123  3.46354  2.40513  3.72494
3.29354  2.67741  2.69355  4.24690  2.90347  2.73739  3.18146  2.89801
2.37887  2.77519  2.98518  4.58477  3.61503
```

```
          0.02187  4.22952  4.95186  0.61958  0.77255  0.48576  0.95510
    180   1.57807  4.27506  3.64443  3.37558  4.49174  3.05218  4.42714
3.87744  3.42750  3.60258  4.43425  3.47642  3.82170  3.70892  3.72136
1.05705  2.83882  3.30797  5.84192  4.63495    185 s - - -
          2.68618  4.42225  2.77519  2.73123  3.46354  2.40513  3.72494
3.29354  2.67741  2.69355  4.24690  2.90347  2.73739  3.18146  2.89801
2.37887  2.77519  2.98518  4.58477  3.61503
          0.02187  4.22952  4.95186  0.61958  0.77255  0.48576  0.95510
    181   3.46812  4.75044  5.14338  4.61162  3.24539  4.78282  5.13535
1.57646  4.44365  0.87676  3.03821  4.84719  4.95179  4.55772  4.54065
4.16941  3.69872  2.17211  5.37218  4.28233    186 l - - -
          2.68618  4.42225  2.77519  2.73123  3.46354  2.40513  3.72494
3.29354  2.67741  2.69355  4.24690  2.90347  2.73739  3.18146  2.89801
2.37887  2.77519  2.98518  4.58477  3.61503
          0.02187  4.22952  4.95186  0.61958  0.77255  0.48576  0.95510
    182   2.70877  4.41908  3.76542  3.34976  3.52896  3.57250  4.19216
2.82244  3.22642  1.25870  3.57882  3.62464  4.11430  3.58378  3.49112
2.01680  3.04465  2.65716  5.12547  3.84237    187 l - - -
          2.68618  4.42225  2.77519  2.73123  3.46354  2.40513  3.72494
3.29354  2.67741  2.69355  4.24690  2.90347  2.73739  3.18146  2.89801
2.37887  2.77519  2.98518  4.58477  3.61503
          0.02187  4.22952  4.95186  0.61958  0.77255  0.48576  0.95510
    183   2.63975  4.57137  3.41445  3.23932  4.66231  1.74653  4.44139
4.16900  3.42236  3.80628  4.66404  3.51082  1.05236  3.71949  3.74354
2.79404  3.11723  3.61747  5.87630  4.73108    188 p - - -
          2.68618  4.42225  2.77519  2.73123  3.46354  2.40513  3.72494
3.29354  2.67741  2.69355  4.24690  2.90347  2.73739  3.18146  2.89801
2.37887  2.77519  2.98518  4.58477  3.61503
          0.02187  4.22952  4.95186  0.61958  0.77255  0.48576  0.95510
    184   2.88551  4.58826  3.79704  3.41597  1.99100  3.66595  4.10453
3.12679  3.38471  2.67595  3.79427  3.69183  1.29806  3.67917  3.66421
3.10031  3.21910  2.95409  4.80033  3.30322    189 p - - -
          2.68621  4.42228  2.77522  2.73126  3.46357  2.40516  3.72497
3.29273  2.67744  2.69358  4.24693  2.90350  2.73743  3.18149  2.89804
2.37890  2.77522  2.98521  4.58480  3.61506
          0.20483  1.72517  4.95186  0.25055  1.50674  0.48576  0.95510
    185   1.62083  4.37568  3.52912  3.27366  4.42123  3.14558  4.36929
3.75626  3.33171  3.49040  4.37532  3.46477  1.23370  3.64610  3.63664
2.62163  2.92446  3.27723  5.77170  4.56241    191 p - - -
          2.68618  4.42225  2.77519  2.73123  3.46354  2.40513  3.72494
3.29354  2.67741  2.69355  4.24690  2.90347  2.73739  3.18146  2.89801
2.37887  2.77519  2.98518  4.58477  3.61503
          0.02187  4.22952  4.95186  0.61958  0.77255  0.48576  0.95510
    186   2.11384  4.58414  3.15181  2.69183  3.95439  2.54159  3.83678
3.33172  2.71105  2.24875  3.86005  2.26920  3.88641  3.04865  3.12528
2.70014  2.88293  3.02123  5.30168  4.01414    192 a - - -
          2.68618  4.42225  2.77519  2.73123  3.46354  2.40513  3.72494
3.29354  2.67741  2.69355  4.24690  2.90347  2.73739  3.18146  2.89801
2.37887  2.77519  2.98518  4.58477  3.61503
          0.02187  4.22952  4.95186  0.61958  0.77255  0.48576  0.95510
    187   1.57807  4.27506  3.64443  3.37558  4.49174  3.05218  4.42714
3.87744  3.42750  3.60258  4.43425  3.47642  3.82170  3.70892  3.72136
1.05705  2.83882  3.30797  5.84192  4.63495    193 s - - -
```

2.68618 4.42225 2.77519 2.73123 3.46354 2.40513 3.72494
3.29354 2.67741 2.69355 4.24690 2.90347 2.73739 3.18146 2.89801
2.37887 2.77519 2.98518 4.58477 3.61503
0.02187 4.22952 4.95186 0.61958 0.77255 0.48576 0.95510
188 2.71645 4.42063 3.77914 3.36480 3.51942 3.58244 4.20007
2.81026 3.23814 1.23189 3.56905 3.63697 4.12271 3.59576 3.49952
2.06301 3.05160 2.64959 5.12227 3.83946 194 l - - -
2.68618 4.42225 2.77519 2.73123 3.46354 2.40513 3.72494
3.29354 2.67741 2.69355 4.24690 2.90347 2.73739 3.18146 2.89801
2.37887 2.77519 2.98518 4.58477 3.61503
0.02187 4.22952 4.95186 0.61958 0.77255 0.48576 0.95510
189 3.53420 4.83210 5.05087 4.55174 1.90090 4.68841 4.63052
2.43901 4.39976 0.83660 3.02069 4.68264 4.87709 4.42969 4.45782
4.06726 3.75537 2.61148 4.80761 3.39488 195 l - - -
2.68618 4.42225 2.77519 2.73123 3.46354 2.40513 3.72494
3.29354 2.67741 2.69355 4.24690 2.90347 2.73739 3.18146 2.89801
2.37887 2.77519 2.98518 4.58477 3.61503
0.02187 4.22952 4.95186 0.61958 0.77255 0.48576 0.95510
190 1.70069 4.32090 3.58117 3.41029 4.60963 0.99031 4.51899
4.00058 3.56063 3.72991 4.57063 3.51358 3.85753 3.81846 3.83837
2.57217 2.90701 3.40339 5.93374 4.75935 196 g - - -
2.68618 4.42225 2.77519 2.73123 3.46354 2.40513 3.72494
3.29354 2.67741 2.69355 4.24690 2.90347 2.73739 3.18146 2.89801
2.37887 2.77519 2.98518 4.58477 3.61503
0.02187 4.22952 4.95186 0.61958 0.77255 0.48576 0.95510
191 1.90037 4.35359 3.55150 3.08130 3.81491 3.35589 4.05898
3.09066 3.03261 2.02774 3.77053 3.39336 3.92839 3.35761 3.36993
1.72786 2.87173 2.80543 5.25448 4.01321 197 s - - -
2.68618 4.42225 2.77519 2.73123 3.46354 2.40513 3.72494
3.29354 2.67741 2.69355 4.24690 2.90347 2.73739 3.18146 2.89801
2.37887 2.77519 2.98518 4.58477 3.61503
0.02187 4.22952 4.95186 0.61958 0.77255 0.48576 0.95510
192 3.37644 4.65384 5.09944 4.58639 3.39419 4.73397 5.15900
1.04314 4.42574 1.40411 3.20096 4.80567 4.94848 4.60637 4.55372
4.12744 3.61960 1.93012 5.47128 4.32444 198 i - - -
2.68618 4.42225 2.77519 2.73123 3.46354 2.40513 3.72494
3.29354 2.67741 2.69355 4.24690 2.90347 2.73739 3.18146 2.89801
2.37887 2.77519 2.98518 4.58477 3.61503
0.02187 4.22952 4.95186 0.61958 0.77255 0.48576 0.95510
193 1.98104 4.37750 3.54314 3.04385 3.56924 3.46534 3.95406
3.05256 2.95735 2.74547 3.65667 3.38765 2.44513 3.29930 3.28319
2.79626 2.89866 2.79386 2.21783 3.72920 199 a - - -
2.68618 4.42225 2.77519 2.73123 3.46354 2.40513 3.72494
3.29354 2.67741 2.69355 4.24690 2.90347 2.73739 3.18146 2.89801
2.37887 2.77519 2.98518 4.58477 3.61503
0.02187 4.22952 4.95186 0.61958 0.77255 0.48576 0.95510
194 2.63123 4.87248 2.20137 2.47145 4.43921 2.26615 3.81077
3.89213 2.66026 3.45899 4.25340 2.97633 3.84543 2.95958 3.14475
2.02232 2.26963 3.46115 5.65501 4.28899 200 s - - -
2.68618 4.42225 2.77519 2.73123 3.46354 2.40513 3.72494
3.29354 2.67741 2.69355 4.24690 2.90347 2.73739 3.18146 2.89801
2.37887 2.77519 2.98518 4.58477 3.61503
0.02187 4.22952 4.95186 0.61958 0.77255 0.48576 0.95510

```
    195   3.06232  4.40192  4.83695  4.25539  2.21648  4.30242  4.63152
1.78929  4.11009  1.77112  2.12288  4.38922  4.57612  4.22327  4.18625
3.62998  3.29082  1.77012  5.03908  3.90332    201 v - - -
          2.68618  4.42225  2.77519  2.73123  3.46354  2.40513  3.72494
3.29354  2.67741  2.69355  4.24690  2.90347  2.73739  3.18146  2.89801
2.37887  2.77519  2.98518  4.58477  3.61503
          0.20483  4.22952  1.76812  0.61958  0.77255  0.48576  0.95510
    196   3.66592  4.89546  4.66328  4.35530  1.10167  4.41641  3.49582
3.33610  4.19895  2.66068  3.93211  4.10075  4.72384  4.15918  4.22465
3.78754  3.88780  3.26633  3.63314  1.32259    202 f - - -
          2.68618  4.42225  2.77519  2.73123  3.46354  2.40513  3.72494
3.29354  2.67741  2.69355  4.24690  2.90347  2.73739  3.18146  2.89801
2.37887  2.77519  2.98518  4.58477  3.61503
          0.02620  4.05089  4.77324  0.61958  0.77255  0.38139  1.14858
    197   2.74298  4.30744  3.88254  3.30833  3.41029  3.80787  4.09127
2.03506  2.31939  2.31611  2.54710  3.64525  4.16947  3.49586  3.46343
3.08246  2.97640  1.85206  4.97051  3.76511    203 v - - -
          2.68618  4.42225  2.77519  2.73123  3.46354  2.40513  3.72494
3.29354  2.67741  2.69355  4.24690  2.90347  2.73739  3.18146  2.89801
2.37887  2.77519  2.98518  4.58477  3.61503
          0.02187  4.22952  4.95186  0.61958  0.77255  0.48576  0.95510
    198   2.48594  4.41966  3.47594  3.18873  4.34821  3.19193  4.28618
3.67777  3.20010  3.40429  4.30025  3.42943  1.25295  3.55046  3.51288
2.65562  1.88905  3.24071  5.69880  4.47425    204 p - - -
          2.68618  4.42225  2.77519  2.73123  3.46354  2.40513  3.72494
3.29354  2.67741  2.69355  4.24690  2.90347  2.73739  3.18146  2.89801
2.37887  2.77519  2.98518  4.58477  3.61503
          0.02187  4.22952  4.95186  0.61958  0.77255  0.48576  0.95510
    199   2.66305  4.84839  2.21386  2.46860  4.16960  2.58118  3.74466
3.58900  2.58994  2.33330  4.02473  2.98506  3.86604  2.90806  3.05983
2.20728  2.92798  3.24886  5.44433  4.10053    205 s - - -
          2.68618  4.42225  2.77519  2.73123  3.46354  2.40513  3.72494
3.29354  2.67741  2.69355  4.24690  2.90347  2.73739  3.18146  2.89801
2.37887  2.77519  2.98518  4.58477  3.61503
          0.31255  4.22952  1.37097  0.61958  0.77255  0.48576  0.95510
    200   2.25372  4.19050  3.34395  3.03048  4.14530  2.98373  4.10451
3.48415  3.03864  3.23001  4.09421  3.24622  3.69788  3.37103  3.35572
1.62707  1.69995  3.02890  5.50942  4.27107    206 s - - -
          2.68618  4.42225  2.77519  2.73123  3.46354  2.40513  3.72494
3.29354  2.67741  2.69355  4.24690  2.90347  2.73739  3.18146  2.89801
2.37887  2.77519  2.98518  4.58477  3.61503
          0.02914  3.94610  4.66845  0.61958  0.77255  0.76495  0.62616
    201   2.68592  4.80509  2.81873  2.50624  4.19127  3.31126  3.74227
3.66875  2.46384  3.23365  4.11668  2.98833  2.15235  1.95301  2.82344
2.72775  2.98048  3.32414  5.42728  4.10986    207 q - - -
          2.68618  4.42225  2.77519  2.73123  3.46354  2.40513  3.72494
3.29354  2.67741  2.69355  4.24690  2.90347  2.73739  3.18146  2.89801
2.37887  2.77519  2.98518  4.58477  3.61503
          0.02914  3.94610  4.66845  0.61958  0.77255  0.34396  1.23429
    202   2.70580  4.75044  3.09970  2.73019  4.20994  3.40641  3.89607
3.59071  2.62548  3.22311  4.11515  3.17267  3.94627  2.10058  2.97217
2.79110  1.44324  3.25929  5.49633  4.20168    208 t - - -
          2.68618  4.42225  2.77519  2.73123  3.46354  2.40513  3.72494
3.29354  2.67741  2.69355  4.24690  2.90347  2.73739  3.18146  2.89801
2.37887  2.77519  2.98518  4.58477  3.61503
```

```
        0.02187  4.22952  4.95186  0.61958  0.77255  0.48576  0.95510
    203   2.78007  5.18555  2.58255  1.80925  4.57209  2.34244  3.75502
4.04671  2.60497  3.56947  4.36322  2.24167  3.87552  2.89298  3.11376
2.17368  3.05100  3.63008  5.74055  4.32586    209 e - - -
        2.68618  4.42225  2.77519  2.73123  3.46354  2.40513  3.72494
3.29354  2.67741  2.69355  4.24690  2.90347  2.73739  3.18146  2.89801
2.37887  2.77519  2.98518  4.58477  3.61503
        0.02187  4.22952  4.95186  0.61958  0.77255  0.48576  0.95510
    204   2.03237  4.24700  3.89826  3.36340  3.51568  2.59458  4.14720
1.92063  3.28971  2.50566  3.47193  3.63825  4.09856  3.56557  3.56675
2.95593  2.92275  1.89929  5.04019  3.82972    210 v - - -
        2.68618  4.42225  2.77519  2.73123  3.46354  2.40513  3.72494
3.29354  2.67741  2.69355  4.24690  2.90347  2.73739  3.18146  2.89801
2.37887  2.77519  2.98518  4.58477  3.61503
        0.02187  4.22952  4.95186  0.61958  0.77255  0.48576  0.95510
    205   2.64159  4.36951  3.49849  2.94295  3.50082  2.72596  3.87175
2.20624  2.86321  2.52772  2.54436  3.34773  3.99359  2.53972  3.20615
2.86304  2.87793  2.60842  4.95996  3.72587    211 i - - -
        2.68618  4.42225  2.77519  2.73123  3.46354  2.40513  3.72494
3.29354  2.67741  2.69355  4.24690  2.90347  2.73739  3.18146  2.89801
2.37887  2.77519  2.98518  4.58477  3.61503
        0.02187  4.22952  4.95186  0.61958  0.77255  0.48576  0.95510
    206   2.09172  5.03082  2.06663  2.37790  4.43625  3.35806  3.73889
3.88534  2.57283  3.44113  4.23444  2.30697  3.85410  2.87708  3.06998
2.69154  2.33314  3.48449  5.63248  4.24443    212 d - - -
        2.68618  4.42225  2.77519  2.73123  3.46354  2.40513  3.72494
3.29354  2.67741  2.69355  4.24690  2.90347  2.73739  3.18146  2.89801
2.37887  2.77519  2.98518  4.58477  3.61503
        0.02187  4.22952  4.95186  0.61958  0.77255  0.48576  0.95510
    207   2.76032  5.00440  1.84015  2.40295  4.25271  3.41601  3.75993
2.43284  2.62591  3.28263  4.12490  2.21190  3.89899  2.92269  3.11372
2.76587  3.01519  3.29804  5.52997  4.15365    213 d - - -
        2.68618  4.42225  2.77519  2.73123  3.46354  2.40513  3.72494
3.29354  2.67741  2.69355  4.24690  2.90347  2.73739  3.18146  2.89801
2.37887  2.77519  2.98518  4.58477  3.61503
        0.02187  4.22952  4.95186  0.61958  0.77255  0.48576  0.95510
    208   2.11050  4.33579  4.41728  3.84313  3.27810  4.08006  4.41610
1.85756  3.72333  1.59409  2.37683  4.07077  4.40409  3.92965  3.90134
3.38675  3.14761  2.15642  5.01406  3.85874    214 l - - -
        2.68618  4.42225  2.77519  2.73123  3.46354  2.40513  3.72494
3.29354  2.67741  2.69355  4.24690  2.90347  2.73739  3.18146  2.89801
2.37887  2.77519  2.98518  4.58477  3.61503
        0.02187  4.22952  4.95186  0.61958  0.77255  0.48576  0.95510
    209   3.00247  4.49148  4.32406  3.83207  2.89975  3.96628  4.10515
2.66755  3.59730  2.31510  3.48770  3.97707  4.38737  3.89117  3.76777
3.34437  3.27513  1.79394  1.48638  3.05086    215 w - - -
        2.68618  4.42225  2.77519  2.73123  3.46354  2.40513  3.72494
3.29354  2.67741  2.69355  4.24690  2.90347  2.73739  3.18146  2.89801
2.37887  2.77519  2.98518  4.58477  3.61503
        0.02187  4.22952  4.95186  0.61958  0.77255  0.48576  0.95510
    210   2.81807  4.99407  3.14735  2.01665  4.26282  3.56501  3.69509
3.65057  2.27331  2.46794  4.06485  3.09313  3.95906  2.84244  1.74748
2.83944  3.03337  3.34228  5.42350  4.13371    216 r - - -
```

```
          2.68618  4.42225  2.77519  2.73123  3.46354  2.40513  3.72494
3.29354  2.67741  2.69355  4.24690  2.90347  2.73739  3.18146  2.89801
2.37887  2.77519  2.98518  4.58477  3.61503
          0.02187  4.22952  4.95186  0.61958  0.77255  0.48576  0.95510
    211   3.22690  5.18921  3.19996  2.97711  4.40446  3.64385  4.13254
4.10826  2.77604  3.57227  4.60598  3.43403  4.21247  0.71080  3.04598
3.26690  3.53111  3.81985  5.61755  4.34378    217 q - - -
          2.68618  4.42225  2.77519  2.73123  3.46354  2.40513  3.72494
3.29354  2.67741  2.69355  4.24690  2.90347  2.73739  3.18146  2.89801
2.37887  2.77519  2.98518  4.58477  3.61503
          0.02187  4.22952  4.95186  0.61958  0.77255  0.48576  0.95510
    212   2.74175  4.24088  4.08187  3.50736  3.27042  3.83719  4.14977
2.02631  3.39527  1.82449  2.36143  3.77253  4.19751  3.64215  3.61761
2.27957  2.97785  2.29279  4.87287  3.68453    218 l - - -
          2.68618  4.42225  2.77519  2.73123  3.46354  2.40513  3.72494
3.29354  2.67741  2.69355  4.24690  2.90347  2.73739  3.18146  2.89801
2.37887  2.77519  2.98518  4.58477  3.61503
          0.02187  4.22952  4.95186  0.61958  0.77255  0.48576  0.95510
    213   2.74612  4.98804  2.93692  1.78992  4.27375  3.47924  3.69734
3.62886  1.92686  3.24719  4.07571  2.99903  3.90236  2.84057  2.80767
2.75664  2.97857  2.42868  5.47158  4.13871    219 e - - -
          2.68618  4.42225  2.77519  2.73123  3.46354  2.40513  3.72494
3.29354  2.67741  2.69355  4.24690  2.90347  2.73739  3.18146  2.89801
2.37887  2.77519  2.98518  4.58477  3.61503
          0.02187  4.22952  4.95186  0.61958  0.77255  0.48576  0.95510
    214   2.66795  4.90080  2.32068  2.44665  4.15938  3.44553  3.67760
3.56022  2.10766  2.40216  3.98261  2.97139  3.85975  2.82424  2.91666
2.68844  2.39213  3.23073  5.40375  4.05995    220 k - - -
          2.68618  4.42225  2.77519  2.73123  3.46354  2.40513  3.72494
3.29354  2.67741  2.69355  4.24690  2.90347  2.73739  3.18146  2.89801
2.37887  2.77519  2.98518  4.58477  3.61503
          0.02187  4.22952  4.95186  0.61958  0.77255  0.48576  0.95510
    215   3.80424  5.14643  4.38673  4.19600  2.40294  4.26283  3.84731
3.76969  4.04504  3.12739  4.40880  4.19212  4.73410  4.26097  4.14895
3.89915  4.09590  3.65865  4.05572  0.53072    221 Y - - -
          2.68618  4.42225  2.77519  2.73123  3.46354  2.40513  3.72494
3.29354  2.67741  2.69355  4.24690  2.90347  2.73739  3.18146  2.89801
2.37887  2.77519  2.98518  4.58477  3.61503
          0.02187  4.22952  4.95186  0.61958  0.77255  0.48576  0.95510
    216   2.96290  4.36785  4.54037  4.01151  3.63726  4.18061  4.70615
1.61339  3.90830  2.34626  3.50007  4.23335  4.55888  4.17531  4.12696
3.52865  2.18199  1.22951  5.38284  4.17709    222 v - - -
          2.68618  4.42225  2.77519  2.73123  3.46354  2.40513  3.72494
3.29354  2.67741  2.69355  4.24690  2.90347  2.73739  3.18146  2.89801
2.37887  2.77519  2.98518  4.58477  3.61503
          0.02187  4.22952  4.95186  0.61958  0.77255  0.48576  0.95510
    217   3.15018  5.78697  1.04721  1.60774  5.06709  3.33443  3.93009
4.61562  3.01072  4.09504  4.96980  2.79124  3.98733  3.10468  3.64841
3.00692  3.45084  4.16940  6.23535  4.69362    223 d - - -
          2.68618  4.42225  2.77519  2.73123  3.46354  2.40513  3.72494
3.29354  2.67741  2.69355  4.24690  2.90347  2.73739  3.18146  2.89801
2.37887  2.77519  2.98518  4.58477  3.61503
          0.02187  4.22952  4.95186  0.61958  0.77255  0.48576  0.95510
```

218 2.97815 5.37220 2.65303 1.25979 4.72354 3.46889 3.77566
4.16705 1.84073 3.65920 4.49110 2.95532 3.97714 2.91721 2.82445
2.91782 3.21951 3.78207 5.77559 4.41697 224 e - - -
2.68618 4.42225 2.77519 2.73123 3.46354 2.40513 3.72494
3.29354 2.67741 2.69355 4.24690 2.90347 2.73739 3.18146 2.89801
2.37887 2.77519 2.98518 4.58477 3.61503
0.02187 4.22952 4.95186 0.61958 0.77255 0.48576 0.95510
219 3.13227 5.33383 3.53049 2.83474 4.81430 3.76399 3.67842
4.15416 1.61659 3.55695 4.40295 3.25429 4.11367 1.99291 1.42496
3.10586 3.28958 3.81326 5.57949 4.40257 225 r - - -
2.68618 4.42225 2.77519 2.73123 3.46354 2.40513 3.72494
3.29354 2.67741 2.69355 4.24690 2.90347 2.73739 3.18146 2.89801
2.37887 2.77519 2.98518 4.58477 3.61503
0.02187 4.22952 4.95186 0.61958 0.77255 0.48576 0.95510
220 3.32169 4.66102 4.81585 4.45732 3.64239 4.43292 5.13400
0.71984 4.29347 2.17033 3.53769 4.65564 4.84343 4.61411 4.45169
3.97643 3.61986 1.92373 5.59674 4.35691 226 i - - -
2.68618 4.42225 2.77519 2.73123 3.46354 2.40513 3.72494
3.29354 2.67741 2.69355 4.24690 2.90347 2.73739 3.18146 2.89801
2.37887 2.77519 2.98518 4.58477 3.61503
0.02187 4.22952 4.95186 0.61958 0.77255 0.48576 0.95510
221 2.85628 5.13656 1.22784 2.39659 4.63343 3.32718 3.92748
4.03447 2.88865 3.66879 4.52944 2.93827 3.93829 3.10310 3.41272
2.84280 2.05178 3.63527 5.87838 4.46592 227 d - - -
2.68618 4.42225 2.77519 2.73123 3.46354 2.40513 3.72494
3.29354 2.67741 2.69355 4.24690 2.90347 2.73739 3.18146 2.89801
2.37887 2.77519 2.98518 4.58477 3.61503
0.02187 4.22952 4.95186 0.61958 0.77255 0.48576 0.95510
222 2.58470 4.66237 2.94301 2.73536 4.47787 3.21270 4.06009
4.00551 2.95421 3.61748 4.44535 1.99653 3.88055 3.26621 3.36146
1.21952 2.99674 3.50659 5.76058 4.41973 228 s - - -
2.68618 4.42225 2.77519 2.73123 3.46354 2.40513 3.72494
3.29354 2.67741 2.69355 4.24690 2.90347 2.73739 3.18146 2.89801
2.37887 2.77519 2.98518 4.58477 3.61503
0.02187 4.22952 4.95186 0.61958 0.77255 0.48576 0.95510
223 2.99227 4.95438 3.48209 2.91206 4.08640 3.72335 3.82672
3.39111 1.25023 2.05685 3.98782 3.33478 4.12064 3.01186 2.54917
3.07227 3.20600 3.18573 5.36598 4.11230 229 k - - -
2.68618 4.42225 2.77519 2.73123 3.46354 2.40513 3.72494
3.29354 2.67741 2.69355 4.24690 2.90347 2.73739 3.18146 2.89801
2.37887 2.77519 2.98518 4.58477 3.61503
0.02187 4.22952 4.95186 0.61958 0.77255 0.48576 0.95510
224 3.25990 4.52873 5.10955 4.62873 3.72790 4.69219 5.33080
0.98617 4.52526 2.19064 3.50863 4.81764 4.97720 4.77709 4.70171
4.10163 3.52618 1.27572 5.75776 4.55531 230 i - - -
2.68618 4.42225 2.77519 2.73123 3.46354 2.40513 3.72494
3.29354 2.67741 2.69355 4.24690 2.90347 2.73739 3.18146 2.89801
2.37887 2.77519 2.98518 4.58477 3.61503
0.02187 4.22952 4.95186 0.61958 0.77255 0.48576 0.95510
225 2.77051 4.43086 3.66941 3.14933 3.48022 3.75470 4.04734
1.97903 3.05605 1.91788 3.46163 2.11165 4.14637 3.39609 3.37600
3.04292 3.01521 2.39388 5.04269 3.77958 231 l - - -
2.68618 4.42225 2.77519 2.73123 3.46354 2.40513 3.72494
3.29354 2.67741 2.69355 4.24690 2.90347 2.73739 3.18146 2.89801
2.37887 2.77519 2.98518 4.58477 3.61503

```
          0.20483  4.22952  1.76812  0.61958  0.77255  0.48576  0.95510
    226   3.19072  5.33531  0.69232  2.46373  4.73852  3.35434  4.10113
4.36932  3.22100  3.94719  4.93261  3.02777  4.02005  3.34297  3.75885
3.13529  3.53428  3.99061  5.87148  4.58636    232 D - - -
          2.68618  4.42225  2.77519  2.73123  3.46354  2.40513  3.72494
3.29354  2.67741  2.69355  4.24690  2.90347  2.73739  3.18146  2.89801
2.37887  2.77519  2.98518  4.58477  3.61503
          0.02620  4.05089  4.77324  0.61958  0.77255  0.67925  0.70724
    227   3.51433  4.93065  4.10616  3.87209  2.32183  4.04152  3.71622
3.49974  3.72469  2.90675  4.15681  3.94976  4.51265  3.98727  3.87040
3.63195  3.80269  3.37914  3.97135  0.71382    233 y - - -
          2.68618  4.42225  2.77519  2.73123  3.46354  2.40513  3.72494
3.29354  2.67741  2.69355  4.24690  2.90347  2.73739  3.18146  2.89801
2.37887  2.77519  2.98518  4.58477  3.61503
          0.02620  4.05089  4.77324  0.61958  0.77255  0.67925  0.70724
    228   3.20889  4.98860  3.31748  3.09874  3.42365  3.61036  0.81584
3.93950  2.93756  3.40945  4.46907  3.49947  4.17442  3.46827  3.19081
3.27897  3.51975  3.67139  4.88556  3.37019    234 h - - -
          2.68618  4.42225  2.77519  2.73123  3.46354  2.40513  3.72494
3.29354  2.67741  2.69355  4.24690  2.90347  2.73739  3.18146  2.89801
2.37887  2.77519  2.98518  4.58477  3.61503
          0.02620  4.05089  4.77324  0.61958  0.77255  0.67925  0.70724
    229   2.81931  5.16148  2.79713  2.41065  4.51134  3.43796  3.63595
3.94917  1.82380  3.44006  4.25343  2.12726  3.88426  2.15558  2.57566
2.78577  3.04390  3.57411  5.55832  4.22995    235 k - - -
          2.68618  4.42225  2.77519  2.73123  3.46354  2.40513  3.72494
3.29354  2.67741  2.69355  4.24690  2.90347  2.73739  3.18146  2.89801
2.37887  2.77519  2.98518  4.58477  3.61503
          0.02620  4.05089  4.77324  0.61958  0.77255  0.38139  1.14858
    230   2.94340  4.44387  4.11125  3.60076  1.93942  3.92561  3.81820
2.87689  3.49584  2.50652  3.53799  3.77842  4.30004  3.70439  3.70670
3.22901  2.13743  2.70794  4.24563  1.68753    236 y - - -
          2.68618  4.42225  2.77519  2.73123  3.46354  2.40513  3.72494
3.29354  2.67741  2.69355  4.24690  2.90347  2.73739  3.18146  2.89801
2.37887  2.77519  2.98518  4.58477  3.61503
          0.02187  4.22952  4.95186  0.61958  0.77255  0.48576  0.95510
    231   2.78867  4.38371  3.81772  3.28904  3.40551  3.81112  4.10948
1.96010  3.17991  1.63895  3.38069  2.42823  4.19210  3.50267  3.46889
3.10490  3.03134  2.33687  5.00060  3.75676    237 l - - -
          2.68618  4.42225  2.77519  2.73123  3.46354  2.40513  3.72494
3.29354  2.67741  2.69355  4.24690  2.90347  2.73739  3.18146  2.89801
2.37887  2.77519  2.98518  4.58477  3.61503
          0.02187  4.22952  4.95186  0.61958  0.77255  0.48576  0.95510
    232   1.91969  4.38849  3.97991  3.52271  3.55957  3.63496  4.33014
2.62868  3.38878  2.31005  1.47709  3.75760  4.17125  3.71435  3.63783
3.02135  3.06226  2.49060  5.21825  4.01795    238 m - - -
          2.68618  4.42225  2.77519  2.73123  3.46354  2.40513  3.72494
3.29354  2.67741  2.69355  4.24690  2.90347  2.73739  3.18146  2.89801
2.37887  2.77519  2.98518  4.58477  3.61503
          0.02187  4.22952  4.95186  0.61958  0.77255  0.48576  0.95510
    233   2.41113  4.34830  3.53419  3.31147  4.51394  1.12807  4.41741
3.89343  3.40367  3.61738  4.46550  3.47090  3.86402  3.69912  3.70077
2.58950  1.77859  3.34983  5.84201  4.64554    239 g - - -
```

```
         2.68618  4.42225  2.77519  2.73123  3.46354  2.40513  3.72494
3.29354  2.67741  2.69355  4.24690  2.90347  2.73739  3.18146  2.89801
2.37887  2.77519  2.98518  4.58477  3.61503
         0.02187  4.22952  4.95186  0.61958  0.77255  0.48576  0.95510
    234  1.00639  4.25662  3.69645  3.42594  4.46189  3.05061  4.44879
3.83155  3.46657  3.57070  4.40783  3.50021  3.82300  3.74304  3.74821
1.65793  2.83366  3.27550  5.82375  4.62509    240 a - - -
         2.68618  4.42225  2.77519  2.73123  3.46354  2.40513  3.72494
3.29354  2.67741  2.69355  4.24690  2.90347  2.73739  3.18146  2.89801
2.37887  2.77519  2.98518  4.58477  3.61503
         0.02187  4.22952  4.95186  0.61958  0.77255  0.48576  0.95510
    235  3.31529  5.45321  2.70580  0.60645  4.85294  3.54554  4.18442
4.38266  3.11533  3.94788  4.92308  3.20387  4.17210  3.40321  3.54988
3.27009  3.62767  4.03530  5.95474  4.69895    241 E - - -
         2.68618  4.42225  2.77519  2.73123  3.46354  2.40513  3.72494
3.29354  2.67741  2.69355  4.24690  2.90347  2.73739  3.18146  2.89801
2.37887  2.77519  2.98518  4.58477  3.61503
         0.02187  4.22952  4.95186  0.61958  0.77255  0.48576  0.95510
    236  3.30023  4.63658  4.70559  4.22411  1.87464  4.32933  3.94632
2.78200  4.06850  1.21599  3.37532  4.20753  4.62100  4.13231  4.14209
3.65789  3.52176  2.77881  4.17336  1.93316    242 l - - -
         2.68618  4.42225  2.77519  2.73123  3.46354  2.40513  3.72494
3.29354  2.67741  2.69355  4.24690  2.90347  2.73739  3.18146  2.89801
2.37887  2.77519  2.98518  4.58477  3.61503
         0.02187  4.22952  4.95186  0.61958  0.77255  0.48576  0.95510
    237  1.65835  4.90952  2.92203  2.52602  4.34254  3.40508  3.76704
3.75769  2.03718  3.34222  4.16137  2.33082  3.89440  2.92012  2.89925
2.73709  2.98506  3.38776  5.54163  4.21303    243 a - - -
         2.68618  4.42225  2.77519  2.73123  3.46354  2.40513  3.72494
3.29354  2.67741  2.69355  4.24690  2.90347  2.73739  3.18146  2.89801
2.37887  2.77519  2.98518  4.58477  3.61503
         0.02187  4.22952  4.95186  0.61958  0.77255  0.48576  0.95510
    238  1.62490  4.28379  3.58295  3.28733  4.53120  1.57056  4.37530
3.96819  3.36995  3.63514  4.43625  3.42707  3.80239  3.63437  3.69622
1.66840  2.82018  3.36074  5.85351  4.64697    244 g - - -
         2.68618  4.42225  2.77519  2.73123  3.46354  2.40513  3.72494
3.29354  2.67741  2.69355  4.24690  2.90347  2.73739  3.18146  2.89801
2.37887  2.77519  2.98518  4.58477  3.61503
         0.02187  4.22952  4.95186  0.61958  0.77255  0.48576  0.95510
    239  2.17095  4.24331  3.87479  3.32195  3.40799  3.69210  4.07938
1.96781  3.23314  1.92801  3.37444  3.62018  4.10549  3.51234  3.50384
2.36447  2.91950  2.31674  4.93805  3.72872    245 l - - -
         2.68618  4.42225  2.77519  2.73123  3.46354  2.40513  3.72494
3.29354  2.67741  2.69355  4.24690  2.90347  2.73739  3.18146  2.89801
2.37887  2.77519  2.98518  4.58477  3.61503
         0.02187  4.22952  4.95186  0.61958  0.77255  0.48576  0.95510
    240  2.73774  4.82215  3.12875  2.61188  4.05973  3.52650  3.72281
3.44758  1.92977  3.06665  2.26656  2.33406  3.93413  2.89688  2.77174
2.79580  2.96807  3.15586  5.32122  4.02745    246 k - - -
         2.68618  4.42225  2.77519  2.73123  3.46354  2.40513  3.72494
3.29354  2.67741  2.69355  4.24690  2.90347  2.73739  3.18146  2.89801
2.37887  2.77519  2.98518  4.58477  3.61503
         0.02187  4.22952  4.95186  0.61958  0.77255  0.48576  0.95510
```

```
    241   0.93086  4.25342  3.70610  3.44580  4.46074  3.05000  4.46330
3.82245  3.48712  3.57038  4.41158  3.50993  3.82558  3.76307  3.76198
1.80535  2.83642  3.26939  5.82704  4.63007    247 a - - -
          2.68618  4.42225  2.77519  2.73123  3.46354  2.40513  3.72494
3.29354  2.67741  2.69355  4.24690  2.90347  2.73739  3.18146  2.89801
2.37887  2.77519  2.98518  4.58477  3.61503
          0.02187  4.22952  4.95186  0.61958  0.77255  0.48576  0.95510
    242   2.20347  4.66399  3.16495  2.62118  3.92979  3.47036  3.74056
3.30795  2.05016  2.96060  2.81358  3.09992  3.88918  2.93082  2.95492
2.18025  2.87752  3.01520  5.24860  3.95746    248 k - - -
          2.68618  4.42225  2.77519  2.73123  3.46354  2.40513  3.72494
3.29354  2.67741  2.69355  4.24690  2.90347  2.73739  3.18146  2.89801
2.37887  2.77519  2.98518  4.58477  3.61503
          0.02187  4.22952  4.95186  0.61958  0.77255  0.48576  0.95510
    243   3.41860  4.69539  5.13598  4.60926  3.31239  4.76138  5.14800
1.27321  4.44955  1.10707  3.10805  4.83252  4.94877  4.58562  4.55639
4.14853  3.65411  2.04583  5.41524  4.30575    249 l - - -
          2.68618  4.42225  2.77519  2.73123  3.46354  2.40513  3.72494
3.29354  2.67741  2.69355  4.24690  2.90347  2.73739  3.18146  2.89801
2.37887  2.77519  2.98518  4.58477  3.61503
          0.02187  4.22952  4.95186  0.61958  0.77255  0.48576  0.95510
    244   2.89494  5.29311  2.87017  1.89784  4.66518  3.51283  3.68480
4.09852  1.54032  3.55879  4.35999  2.98605  3.94830  2.21602  2.60261
2.84817  3.11253  3.70637  5.65762  4.32731    250 k - - -
          2.68618  4.42225  2.77519  2.73123  3.46354  2.40513  3.72494
3.29354  2.67741  2.69355  4.24690  2.90347  2.73739  3.18146  2.89801
2.37887  2.77519  2.98518  4.58477  3.61503
          0.02187  4.22952  4.95186  0.61958  0.77255  0.48576  0.95510
    245   3.10510  5.72105  1.61494  1.09479  5.00801  3.33989  3.90300
4.54626  2.94340  4.02762  4.88535  2.79789  3.97382  3.07059  3.55785
2.97376  3.39764  4.10367  6.16789  4.64436    251 e - - -
          2.68618  4.42225  2.77519  2.73123  3.46354  2.40513  3.72494
3.29354  2.67741  2.69355  4.24690  2.90347  2.73739  3.18146  2.89801
2.37887  2.77519  2.98518  4.58477  3.61503
          0.02187  4.22952  4.95186  0.61958  0.77255  0.48576  0.95510
    246   3.98789  5.12769  4.92363  4.68751  1.37439  4.65031  3.56786
3.63623  4.51386  2.90744  4.21157  4.28522  4.94301  4.38481  4.47528
4.03732  4.20332  3.57233  3.67794  0.84206    252 y - - -
          2.68618  4.42225  2.77519  2.73123  3.46354  2.40513  3.72494
3.29354  2.67741  2.69355  4.24690  2.90347  2.73739  3.18146  2.89801
2.37887  2.77519  2.98518  4.58477  3.61503
          0.02187  4.22952  4.95186  0.61958  0.77255  0.48576  0.95510
    247   2.08413  5.00097  2.80620  2.45023  4.40473  3.39272  3.75183
3.85069  2.51506  3.40975  4.21987  2.16448  3.88485  1.89073  2.94909
2.73322  2.99985  3.46648  5.59323  4.23379    253 q - - -
          2.68618  4.42225  2.77519  2.73123  3.46354  2.40513  3.72494
3.29354  2.67741  2.69355  4.24690  2.90347  2.73739  3.18146  2.89801
2.37887  2.77519  2.98518  4.58477  3.61503
          0.02187  4.22952  4.95186  0.61958  0.77255  0.48576  0.95510
    248   2.80055  4.86311  3.36130  2.85672  4.43334  3.48531  3.84669
3.87442  2.33862  3.42628  4.28078  3.25438  3.99762  3.01367  1.29377
1.97571  3.10018  3.49501  5.54408  4.29462    254 r - - -
          2.68618  4.42225  2.77519  2.73123  3.46354  2.40513  3.72494
3.29354  2.67741  2.69355  4.24690  2.90347  2.73739  3.18146  2.89801
2.37887  2.77519  2.98518  4.58477  3.61503
```

```
          0.02187  4.22952  4.95186  0.61958  0.77255  0.48576  0.95510
    249   2.80500  4.55066  3.54022  3.10633  3.71218  3.66891  4.07859
2.72847  2.92438  2.61357  3.67886  3.49186  4.13821  2.22561  3.22114
3.02978  3.08619  1.35375  5.23800  3.97008    255 v - - -
          2.68618  4.42225  2.77519  2.73123  3.46354  2.40513  3.72494
3.29354  2.67741  2.69355  4.24690  2.90347  2.73739  3.18146  2.89801
2.37887  2.77519  2.98518  4.58477  3.61503
          0.02187  4.22952  4.95186  0.61958  0.77255  0.48576  0.95510
    250   3.31039  4.65737  4.90964  4.39917  3.31579  4.53857  4.98211
2.11947  4.23196  0.93616  3.13659  4.62152  4.81327  4.42126  4.37163
3.92527  3.56489  1.60812  5.35727  4.22077    256 l - - -
          2.68618  4.42225  2.77519  2.73123  3.46354  2.40513  3.72494
3.29354  2.67741  2.69355  4.24690  2.90347  2.73739  3.18146  2.89801
2.37887  2.77519  2.98518  4.58477  3.61503
          0.02187  4.22952  4.95186  0.61958  0.77255  0.48576  0.95510
    251   2.89875  5.25894  2.93451  2.50265  4.63471  3.53012  3.68631
4.06635  1.52693  3.53282  4.33959  2.16829  3.95911  2.20980  2.56285
2.86080  3.11504  3.68253  5.62922  4.31307    257 k - - -
          2.68618  4.42225  2.77519  2.73123  3.46354  2.40513  3.72494
3.29354  2.67741  2.69355  4.24690  2.90347  2.73739  3.18146  2.89801
2.37887  2.77519  2.98518  4.58477  3.61503
          0.02187  4.22952  4.95186  0.61958  0.77255  0.48576  0.95510
    252   3.27567  4.58930  4.93591  4.42733  2.04297  4.50993  4.68250
1.13305  4.29170  1.84404  3.20415  4.55713  4.77593  4.41230  4.39067
3.87776  3.51659  2.11557  4.97839  3.60348    258 i - - -
          2.68618  4.42225  2.77519  2.73123  3.46354  2.40513  3.72494
3.29354  2.67741  2.69355  4.24690  2.90347  2.73739  3.18146  2.89801
2.37887  2.77519  2.98518  4.58477  3.61503
          0.02187  4.22952  4.95186  0.61958  0.77255  0.48576  0.95510
    253   2.03999  4.44685  4.11762  3.61410  1.93047  3.92190  3.82023
2.88413  3.51570  2.51579  3.54883  3.78477  4.30295  3.71787  3.72446
3.22972  3.18908  2.71415  4.24107  1.67691    259 y - - -
          2.68618  4.42225  2.77519  2.73123  3.46354  2.40513  3.72494
3.29354  2.67741  2.69355  4.24690  2.90347  2.73739  3.18146  2.89801
2.37887  2.77519  2.98518  4.58477  3.61503
          0.02187  4.22952  4.95186  0.61958  0.77255  0.48576  0.95510
    254   2.76767  4.99433  2.78660  1.94524  4.22443  3.44524  3.73965
3.64246  2.54092  2.31520  4.09429  1.95404  3.90784  2.90152  2.98390
2.77673  3.01268  3.32106  5.48371  4.12548    260 e - - -
          2.68618  4.42225  2.77519  2.73123  3.46354  2.40513  3.72494
3.29354  2.67741  2.69355  4.24690  2.90347  2.73739  3.18146  2.89801
2.37887  2.77519  2.98518  4.58477  3.61503
          0.02187  4.22952  4.95186  0.61958  0.77255  0.48576  0.95510
    255   3.06112  5.59279  1.41769  2.23718  4.91493  3.33184  3.91423
4.48782  2.95171  3.98647  4.84392  1.43667  3.96920  3.08778  3.55128
2.95319  3.36679  4.04224  6.10817  4.59549    261 d - - -
          2.68618  4.42225  2.77519  2.73123  3.46354  2.40513  3.72494
3.29354  2.67741  2.69355  4.24690  2.90347  2.73739  3.18146  2.89801
2.37887  2.77519  2.98518  4.58477  3.61503
          0.02187  4.22952  4.95186  0.61958  0.77255  0.48576  0.95510
    256   2.78493  5.03515  1.84240  2.43328  4.63300  3.28676  3.94257
4.13613  2.90196  3.71261  4.54558  2.95710  3.91345  3.12054  3.41653
1.26855  3.13492  3.68199  5.87330  4.46359    262 s - - -
```

```
          2.68618  4.42225  2.77519  2.73123  3.46354  2.40513  3.72494
3.29354  2.67741  2.69355  4.24690  2.90347  2.73739  3.18146  2.89801
2.37887  2.77519  2.98518  4.58477  3.61503
          0.02187  4.22952  4.95186  0.61958  0.77255  0.48576  0.95510
    257   2.90501  4.89208  3.42204  2.84265  4.07979  3.66939  3.79758
3.38287  1.29885  2.95932  2.47554  3.27786  4.06583  2.97736  2.58656
2.98525  3.12471  3.15871  5.35301  4.10556    263 k - - -
          2.68618  4.42225  2.77519  2.73123  3.46354  2.40513  3.72494
3.29354  2.67741  2.69355  4.24690  2.90347  2.73739  3.18146  2.89801
2.37887  2.77519  2.98518  4.58477  3.61503
          0.02187  4.22952  4.95186  0.61958  0.77255  0.48576  0.95510
    258   2.95473  5.22427  2.76255  2.51317  4.60921  3.47172  3.81368
4.09286  1.91451  3.61256  4.46017  1.31665  3.99551  2.97084  2.79450
2.93028  3.21787  3.71356  5.70809  4.36815    264 n - - -
          2.68618  4.42225  2.77519  2.73123  3.46354  2.40513  3.72494
3.29354  2.67741  2.69355  4.24690  2.90347  2.73739  3.18146  2.89801
2.37887  2.77519  2.98518  4.58477  3.61503
          0.15591  4.22952  2.04170  0.61958  0.77255  0.48576  0.95510
    259   2.69194  5.05075  2.04121  2.33604  4.40981  3.34954  3.66745
3.87225  2.44258  3.40591  4.19923  2.87412  3.82393  2.80079  2.18212
2.15092  2.94832  3.47680  5.57325  4.18954    265 d - - -
          2.68624  4.42231  2.77526  2.73129  3.46360  2.40519  3.72501
3.29360  2.67747  2.69315  4.24696  2.90296  2.73746  3.18152  2.89807
2.37893  2.77526  2.98524  4.58483  3.61509
          0.48554  1.59422  1.70611  0.66933  0.71754  0.40295  1.10367
    260   3.25859  4.65562  4.63992  4.09935  3.09817  4.40582  4.70406
2.25357  3.89129  1.23269  1.53933  4.38203  4.65235  4.11502  4.05459
3.76303  3.49238  2.36174  5.12750  3.99532    268 l - - -
          2.68618  4.42225  2.77519  2.73123  3.46354  2.40513  3.72494
3.29354  2.67741  2.69355  4.24690  2.90347  2.73739  3.18146  2.89801
2.37887  2.77519  2.98518  4.58477  3.61503
          0.02584  4.06439  4.78674  0.61958  0.77255  0.66695  0.72005
    261   2.70846  4.98623  2.76002  2.40435  4.39198  3.35623  3.68513
3.83790  1.93790  3.38085  4.19234  2.09078  3.84333  2.82892  2.79696
2.12816  2.97237  3.45365  5.54490  4.19679    269 k - - -
          2.68618  4.42225  2.77519  2.73123  3.46354  2.40513  3.72494
3.29354  2.67741  2.69355  4.24690  2.90347  2.73739  3.18146  2.89801
2.37887  2.77519  2.98518  4.58477  3.61503
          0.02584  4.06439  4.78674  0.61958  0.77255  0.38715  1.13628
    262   2.94215  4.75205  3.70729  3.09086  3.87430  3.76084  3.92039
2.13384  2.45705  2.75294  3.80616  3.46882  4.16069  3.14865  1.35491
3.10408  3.17312  2.85854  5.26787  4.02730    270 r - - -
          2.68618  4.42225  2.77519  2.73123  3.46354  2.40513  3.72494
3.29354  2.67741  2.69355  4.24690  2.90347  2.73739  3.18146  2.89801
2.37887  2.77519  2.98518  4.58477  3.61503
          0.02187  4.22952  4.95186  0.61958  0.77255  0.48576  0.95510
    263   2.11636  4.37977  4.61543  4.09694  3.65905  4.21282  4.78428
1.27773  3.99859  2.33640  3.51030  4.30032  4.59780  4.25766  4.20835
3.57247  3.26005  1.46991  5.43440  4.23106    271 i - - -
          2.68618  4.42225  2.77519  2.73123  3.46354  2.40513  3.72494
3.29354  2.67741  2.69355  4.24690  2.90347  2.73739  3.18146  2.89801
2.37887  2.77519  2.98518  4.58477  3.61503
          0.20483  4.22952  1.76812  0.61958  0.77255  0.48576  0.95510
```

```
    264   3.11663  5.25369  2.57744  0.79585  4.62396  3.39697  4.00395
4.11392  2.90782  3.70391  4.67384  3.04870  4.01117  3.21827  3.33244
3.08305  3.42346  3.78073  5.76183  4.48868    272 e - - -
          2.68618  4.42225  2.77519  2.73123  3.46354  2.40513  3.72494
3.29354  2.67741  2.69355  4.24690  2.90347  2.73739  3.18146  2.89801
2.37887  2.77519  2.98518  4.58477  3.61503
          0.02620  4.05089  4.77324  0.61958  0.77255  0.67925  0.70724
    265   2.06372  4.81913  2.73578  1.94369  4.25579  3.29564  3.74343
3.65598  2.57883  3.27161  4.10247  2.93037  2.41485  2.90873  3.03108
2.66321  2.92401  3.29084  5.50692  4.16275    273 e - - -
          2.68618  4.42225  2.77519  2.73123  3.46354  2.40513  3.72494
3.29354  2.67741  2.69355  4.24690  2.90347  2.73739  3.18146  2.89801
2.37887  2.77519  2.98518  4.58477  3.61503
          0.02620  4.05089  4.77324  0.61958  0.77255  0.67925  0.70724
    266   3.00522  4.72913  3.63226  3.49957  4.47376  3.40671  4.55952
4.06867  3.57285  3.67467  4.73439  3.78670  0.55391  3.95613  3.79526
3.17904  3.46148  3.69306  5.60793  4.59794    274 P - - -
          2.68618  4.42225  2.77519  2.73123  3.46354  2.40513  3.72494
3.29354  2.67741  2.69355  4.24690  2.90347  2.73739  3.18146  2.89801
2.37887  2.77519  2.98518  4.58477  3.61503
          0.02620  4.05089  4.77324  0.61958  0.77255  0.67925  0.70724
    267   2.29689  4.26434  3.25314  3.05857  4.43697  1.42676  4.24290
3.91442  3.23041  3.57891  4.40149  3.27375  3.73097  3.50728  3.55902
1.59946  2.79072  3.32254  5.75126  4.51745    275 g - - -
          2.68618  4.42225  2.77519  2.73123  3.46354  2.40513  3.72494
3.29354  2.67741  2.69355  4.24690  2.90347  2.73739  3.18146  2.89801
2.37887  2.77519  2.98518  4.58477  3.61503
          0.02620  4.05089  4.77324  0.61958  0.77255  0.67925  0.70724
    268   2.51824  4.36245  3.59676  3.36156  4.13655  3.19471  4.36394
3.28587  3.30806  3.14293  4.18483  3.53917  3.91387  3.69469  3.55572
2.72188  0.90172  2.96129  5.55843  4.35430    276 t - - -
          2.68618  4.42225  2.77519  2.73123  3.46354  2.40513  3.72494
3.29354  2.67741  2.69355  4.24690  2.90347  2.73739  3.18146  2.89801
2.37887  2.77519  2.98518  4.58477  3.61503
          0.02620  4.05089  4.77324  0.61958  0.77255  0.67925  0.70724
    269   2.48133  4.42762  3.22232  3.02445  4.45877  1.23298  4.23272
3.94428  3.19549  3.58787  4.44331  3.31816  2.02971  3.49760  3.53219
2.63011  2.94571  3.41880  5.69675  4.51974    277 g - - -
          2.68618  4.42225  2.77519  2.73123  3.46354  2.40513  3.72494
3.29354  2.67741  2.69355  4.24690  2.90347  2.73739  3.18146  2.89801
2.37887  2.77519  2.98518  4.58477  3.61503
          0.02620  4.05089  4.77324  0.61958  0.77255  0.67925  0.70724
    270   2.95097  4.44812  4.36946  4.04148  3.68657  3.90519  4.81432
2.05838  3.91203  2.34473  3.62783  4.20175  4.45823  4.26052  4.10686
3.42263  3.30973  0.82642  5.50005  4.24131    278 v - - -
          2.68618  4.42225  2.77519  2.73123  3.46354  2.40513  3.72494
3.29354  2.67741  2.69355  4.24690  2.90347  2.73739  3.18146  2.89801
2.37887  2.77519  2.98518  4.58477  3.61503
          0.02620  4.05089  4.77324  0.61958  0.77255  0.67925  0.70724
    271   3.17193  4.54586  4.58196  4.20738  3.50016  4.25356  4.90554
0.88079  4.03896  2.05355  3.41170  4.42820  4.67087  4.37110  4.21560
3.76919  3.46927  1.86732  5.42540  4.17676    279 i - - -
          2.68618  4.42225  2.77519  2.73123  3.46354  2.40513  3.72494
3.29354  2.67741  2.69355  4.24690  2.90347  2.73739  3.18146  2.89801
2.37887  2.77519  2.98518  4.58477  3.61503
```

0.02620 4.05089 4.77324 0.61958 0.77255 0.67925 0.70724
272 2.68405 4.79254 2.76134 1.93091 4.22188 3.31909 3.81053
3.52930 2.63369 3.22544 4.10649 2.99199 3.86581 2.99693 3.04480
2.73667 1.65306 3.20309 5.50677 4.18383 280 t - - -
2.68618 4.42225 2.77519 2.73123 3.46354 2.40513 3.72494
3.29354 2.67741 2.69355 4.24690 2.90347 2.73739 3.18146 2.89801
2.37887 2.77519 2.98518 4.58477 3.61503
0.02620 4.05089 4.77324 0.61958 0.77255 0.67925 0.70724
273 1.58942 4.63797 2.99330 2.65267 4.14343 3.29006 3.84583
3.51840 2.61437 3.16280 4.05483 3.09043 3.85287 2.05932 2.97417
2.68759 2.92976 3.17604 5.44373 4.14531 281 a - - -
2.68618 4.42225 2.77519 2.73123 3.46354 2.40513 3.72494
3.29354 2.67741 2.69355 4.24690 2.90347 2.73739 3.18146 2.89801
2.37887 2.77519 2.98518 4.58477 3.61503
0.02620 4.05089 4.77324 0.61958 0.77255 0.67925 0.70724
274 2.08970 4.23063 4.06419 3.53585 2.23580 3.73752 4.18909
2.31498 3.44401 2.19656 3.29897 3.76833 4.17149 3.69203 3.67562
3.06937 2.97284 1.76845 4.90236 3.63737 282 v - - -
2.68618 4.42225 2.77519 2.73123 3.46354 2.40513 3.72494
3.29354 2.67741 2.69355 4.24690 2.90347 2.73739 3.18146 2.89801
2.37887 2.77519 2.98518 4.58477 3.61503
0.02620 4.05089 4.77324 0.61958 0.77255 0.67925 0.70724
275 3.21660 5.03647 3.72641 3.20452 4.43676 3.66584 3.96816
3.99380 2.32926 3.47742 4.47593 3.54904 4.16244 3.18223 0.73721
3.29400 3.47212 3.71077 5.44908 4.32858 283 r - - -
2.68618 4.42225 2.77519 2.73123 3.46354 2.40513 3.72494
3.29354 2.67741 2.69355 4.24690 2.90347 2.73739 3.18146 2.89801
2.37887 2.77519 2.98518 4.58477 3.61503
0.02620 4.05089 4.77324 0.61958 0.77255 0.67925 0.70724
276 2.05615 4.38975 3.33084 2.87031 3.69027 3.43906 3.90618
2.08938 2.84404 2.70244 3.63549 2.40823 3.92562 3.18499 3.20545
2.76256 2.86371 2.57378 5.13692 3.87617 284 a - - -
2.68618 4.42225 2.77519 2.73123 3.46354 2.40513 3.72494
3.29354 2.67741 2.69355 4.24690 2.90347 2.73739 3.18146 2.89801
2.37887 2.77519 2.98518 4.58477 3.61503
0.02620 4.05089 4.77324 0.61958 0.77255 0.67925 0.70724
277 1.56876 4.35166 3.77529 3.34091 3.56536 3.52614 4.22592
2.55637 3.22845 1.71745 3.51433 3.61233 4.07624 3.57613 3.50609
2.91932 2.99105 2.39671 5.20396 3.96392 285 a - - -
2.68618 4.42225 2.77519 2.73123 3.46354 2.40513 3.72494
3.29354 2.67741 2.69355 4.24690 2.90347 2.73739 3.18146 2.89801
2.37887 2.77519 2.98518 4.58477 3.61503
0.02620 4.05089 4.77324 0.61958 0.77255 0.67925 0.70724
278 2.94578 5.44469 1.33949 2.15554 4.76268 3.24032 3.81354
4.32502 2.83824 3.83951 4.69647 1.84424 3.87112 2.98779 3.42529
2.84768 3.24995 3.89120 5.96670 4.46671 286 d - - -
2.68618 4.42225 2.77519 2.73123 3.46354 2.40513 3.72494
3.29354 2.67741 2.69355 4.24690 2.90347 2.73739 3.18146 2.89801
2.37887 2.77519 2.98518 4.58477 3.61503
0.02620 4.05089 4.77324 0.61958 0.77255 0.67925 0.70724
279 2.65788 4.96312 2.11896 2.35897 4.30554 3.35229 3.65083
3.73758 2.41718 3.30132 4.10568 2.88874 3.81207 2.78885 2.27736
2.65465 2.29640 3.36600 5.49376 4.12930 287 d - - -

```
          2.68618  4.42225  2.77519  2.73123  3.46354  2.40513  3.72494
3.29354  2.67741  2.69355  4.24690  2.90347  2.73739  3.18146  2.89801
2.37887  2.77519  2.98518  4.58477  3.61503
          0.02620  4.05089  4.77324  0.61958  0.77255  0.38139  1.14858
    280   2.16328  5.31005  1.49961  2.28514  4.71351  3.33154  3.83016
4.19968  2.76526  3.72872  4.54425  2.04258  3.90922  2.98325  3.31392
2.81771  3.16704  3.77032  5.89961  4.44834    288 d - - -
          2.68618  4.42225  2.77519  2.73123  3.46354  2.40513  3.72494
3.29354  2.67741  2.69355  4.24690  2.90347  2.73739  3.18146  2.89801
2.37887  2.77519  2.98518  4.58477  3.61503
          0.02187  4.22952  4.95186  0.61958  0.77255  0.48576  0.95510
    281   3.51634  4.81781  4.98482  4.50912  1.40428  4.62217  4.43333
2.50942  4.35213  1.11352  3.11098  4.58298  4.84273  4.38869  4.41035
3.99957  3.74119  2.64973  4.61423  3.10895    289 l - - -
          2.68618  4.42225  2.77519  2.73123  3.46354  2.40513  3.72494
3.29354  2.67741  2.69355  4.24690  2.90347  2.73739  3.18146  2.89801
2.37887  2.77519  2.98518  4.58477  3.61503
          0.02187  4.22952  4.95186  0.61958  0.77255  0.48576  0.95510
    282   2.75535  4.94103  3.03309  2.00952  4.20550  3.51525  3.69960
2.52584  1.73913  3.17355  4.01775  3.04209  3.92152  2.84965  2.75377
2.78113  2.98076  3.23399  5.41741  4.10516    290 k - - -
          2.68618  4.42225  2.77519  2.73123  3.46354  2.40513  3.72494
3.29354  2.67741  2.69355  4.24690  2.90347  2.73739  3.18146  2.89801
2.37887  2.77519  2.98518  4.58477  3.61503
          0.02187  4.22952  4.95186  0.61958  0.77255  0.48576  0.95510
    283   2.13824  4.78010  3.13870  2.64347  4.24791  3.41863  3.78425
3.60329  1.65865  3.23679  4.06997  3.10671  3.89921  2.94479  2.84471
2.72396  2.15546  3.25289  5.46810  4.17345    291 k - - -
          2.68618  4.42225  2.77519  2.73123  3.46354  2.40513  3.72494
3.29354  2.67741  2.69355  4.24690  2.90347  2.73739  3.18146  2.89801
2.37887  2.77519  2.98518  4.58477  3.61503
          0.02187  4.22952  4.95186  0.61958  0.77255  0.48576  0.95510
    284   3.94352  5.09551  4.92444  4.67878  0.85564  4.63430  3.58494
3.56942  4.50405  2.84603  4.14825  4.28812  4.92774  4.38101  4.46545
4.02021  4.16030  3.51244  3.69711  1.38943    292 f - - -
          2.68618  4.42225  2.77519  2.73123  3.46354  2.40513  3.72494
3.29354  2.67741  2.69355  4.24690  2.90347  2.73739  3.18146  2.89801
2.37887  2.77519  2.98518  4.58477  3.61503
          0.02187  4.22952  4.95186  0.61958  0.77255  0.48576  0.95510
    285   3.38021  4.65727  5.10421  4.58937  3.38552  4.73703  5.15796
1.06260  4.42930  1.37208  3.19076  4.80876  4.94841  4.60461  4.55482
4.12954  3.62248  1.94082  5.46521  4.32285    293 i - - -
          2.68618  4.42225  2.77519  2.73123  3.46354  2.40513  3.72494
3.29354  2.67741  2.69355  4.24690  2.90347  2.73739  3.18146  2.89801
2.37887  2.77519  2.98518  4.58477  3.61503
          0.02187  4.22952  4.95186  0.61958  0.77255  0.48576  0.95510
    286   1.83769  4.27639  3.69105  3.20214  3.86148  3.29037  4.13388
3.09852  3.15670  2.91605  3.80488  3.44834  3.90196  3.45059  3.48206
2.04749  2.14102  1.96726  5.29374  4.07546    294 a - - -
          2.68618  4.42225  2.77519  2.73123  3.46354  2.40513  3.72494
3.29354  2.67741  2.69355  4.24690  2.90347  2.73739  3.18146  2.89801
2.37887  2.77519  2.98518  4.58477  3.61503
          0.02187  4.22952  4.95186  0.61958  0.77255  0.48576  0.95510
```

```
    287   2.71351  4.49493  3.48626  2.94026  3.70224  3.63176  3.90776
1.91123  2.13662  2.67629  3.62147  3.35565  4.03804  3.17712  3.06893
2.91232  2.21018  2.58522  5.13622  3.89492    295 i - - -
          2.68618  4.42225  2.77519  2.73123  3.46354  2.40513  3.72494
3.29354  2.67741  2.69355  4.24690  2.90347  2.73739  3.18146  2.89801
2.37887  2.77519  2.98518  4.58477  3.61503
          0.02187  4.22952  4.95186  0.61958  0.77255  0.48576  0.95510
    288   3.43391  4.71168  5.14217  4.61286  3.28927  4.76925  5.14458
1.36269  4.45156  1.02566  3.08307  4.83911  4.94964  4.57777  4.55379
4.15563  3.66753  2.08540  5.40019  4.29912    296 l - - -
          2.68618  4.42225  2.77519  2.73123  3.46354  2.40513  3.72494
3.29354  2.67741  2.69355  4.24690  2.90347  2.73739  3.18146  2.89801
2.37887  2.77519  2.98518  4.58477  3.61503
          0.02187  4.22952  4.95186  0.61958  0.77255  0.48576  0.95510
    289   2.96696  5.35680  2.60264  1.77479  4.66856  3.44655  3.79062
4.14429  2.51482  3.64052  4.48303  2.93763  3.96955  1.41483  2.91830
2.90810  3.21713  3.76434  5.77524  4.39555    297 q - - -
          2.68633  4.42240  2.77485  2.73139  3.46369  2.40494  3.72510
3.29285  2.67756  2.69370  4.24705  2.90305  2.73755  3.18162  2.89816
2.37902  2.77535  2.98534  4.58492  3.61402
          0.20483  1.72517  4.95186  1.37562  0.29127  0.48576  0.95510
    290   2.54582  4.31505  3.60698  3.11209  2.39226  3.42934  4.00498
2.98610  3.06877  2.71664  3.63257  3.42343  3.95674  3.36752  3.39412
2.03414  1.83744  2.72624  5.04233  3.75893    303 t - - -
          2.68618  4.42225  2.77519  2.73123  3.46354  2.40513  3.72494
3.29354  2.67741  2.69355  4.24690  2.90347  2.73739  3.18146  2.89801
2.37887  2.77519  2.98518  4.58477  3.61503
          0.02187  4.22952  4.95186  0.61958  0.77255  0.48576  0.95510
    291   2.55670  4.38841  3.47274  2.99668  3.76670  3.41093  3.99467
2.15351  2.95655  2.81204  3.72299  3.35176  2.10194  3.28646  3.30640
2.06077  2.88196  2.76829  5.20048  3.95153    304 s - - -
          2.68618  4.42225  2.77519  2.73123  3.46354  2.40513  3.72494
3.29354  2.67741  2.69355  4.24690  2.90347  2.73739  3.18146  2.89801
2.37887  2.77519  2.98518  4.58477  3.61503
          0.02187  4.22952  4.95186  0.61958  0.77255  0.48576  0.95510
    292   2.69580  4.64906  3.15534  1.88871  3.87697  3.53824  3.83154
2.25977  2.68827  2.84948  3.76676  3.16850  3.96313  3.04256  3.09914
2.81521  2.26347  2.77012  5.26853  3.98115    305 e - - -
          2.68618  4.42225  2.77519  2.73123  3.46354  2.40513  3.72494
3.29354  2.67741  2.69355  4.24690  2.90347  2.73739  3.18146  2.89801
2.37887  2.77519  2.98518  4.58477  3.61503
          0.02187  4.22952  4.95186  0.61958  0.77255  0.48576  0.95510
    293   2.98490  5.24483  2.96382  2.59872  4.65527  3.55133  3.75925
4.09932  1.20516  3.58312  4.42405  2.13616  4.02045  2.90109  2.55663
2.96102  3.21700  3.72808  5.65483  4.36394    306 k - - -
          2.68618  4.42225  2.77519  2.73123  3.46354  2.40513  3.72494
3.29354  2.67741  2.69355  4.24690  2.90347  2.73739  3.18146  2.89801
2.37887  2.77519  2.98518  4.58477  3.61503
          0.02187  4.22952  4.95186  0.61958  0.77255  0.48576  0.95510
    294   2.76405  5.00037  2.88858  1.77038  4.22607  3.47818  3.71009
3.64046  2.44358  2.32775  4.05928  2.99341  3.90995  2.20675  2.85674
2.77256  2.99703  3.32711  5.46122  4.12046    307 e - - -
          2.68618  4.42225  2.77519  2.73123  3.46354  2.40513  3.72494
3.29354  2.67741  2.69355  4.24690  2.90347  2.73739  3.18146  2.89801
2.37887  2.77519  2.98518  4.58477  3.61503
```

```
          0.02187  4.22952  4.95186  0.61958  0.77255  0.48576  0.95510
    295   2.54367  4.35168  3.54241  3.03182  3.71163  3.41939  3.98869
3.01221  2.96468  2.75060  2.38997  3.37321  3.93975  3.29343  3.30460
2.07579  1.85409  2.74824  5.15079  3.91551    308 t - - -
          2.68618  4.42225  2.77519  2.73123  3.46354  2.40513  3.72494
3.29354  2.67741  2.69355  4.24690  2.90347  2.73739  3.18146  2.89801
2.37887  2.77519  2.98518  4.58477  3.61503
          0.02187  4.22952  4.95186  0.61958  0.77255  0.48576  0.95510
    296   3.09053  5.63956  1.08924  2.22768  4.95668  3.32831  3.93007
4.53789  2.99284  4.03340  4.90029  1.86750  3.97744  3.10749  3.60759
2.97400  3.40075  4.08848  6.15336  4.62793    309 d - - -
          2.68618  4.42225  2.77519  2.73123  3.46354  2.40513  3.72494
3.29354  2.67741  2.69355  4.24690  2.90347  2.73739  3.18146  2.89801
2.37887  2.77519  2.98518  4.58477  3.61503
          0.02187  4.22952  4.95186  0.61958  0.77255  0.48576  0.95510
    297   2.74716  4.33595  3.89511  3.36482  2.20909  3.74035  3.99987
2.69928  3.27671  1.84628  3.38382  3.64638  4.15130  3.54242  3.53945
1.92368  3.00563  2.54174  4.70416  3.32104    310 l - - -
          2.68618  4.42225  2.77519  2.73123  3.46354  2.40513  3.72494
3.29354  2.67741  2.69355  4.24690  2.90347  2.73739  3.18146  2.89801
2.37887  2.77519  2.98518  4.58477  3.61503
          0.02187  4.22952  4.95186  0.61958  0.77255  0.48576  0.95510
    298   2.63647  4.44529  3.38840  2.84808  3.62974  2.52283  3.85161
2.36670  2.78891  2.66092  3.56969  3.27754  3.96751  2.38694  3.15976
2.82024  2.88274  2.22094  5.05945  3.80806    311 v - - -
          2.68618  4.42225  2.77519  2.73123  3.46354  2.40513  3.72494
3.29354  2.67741  2.69355  4.24690  2.90347  2.73739  3.18146  2.89801
2.37887  2.77519  2.98518  4.58477  3.61503
          0.02187  4.22952  4.95186  0.61958  0.77255  0.48576  0.95510
    299   3.80424  5.14643  4.38673  4.19600  2.40294  4.26283  3.84731
3.76969  4.04504  3.12739  4.40880  4.19212  4.73410  4.26097  4.14895
3.89915  4.09590  3.65865  4.05572  0.53072    312 Y - - -
          2.68618  4.42225  2.77519  2.73123  3.46354  2.40513  3.72494
3.29354  2.67741  2.69355  4.24690  2.90347  2.73739  3.18146  2.89801
2.37887  2.77519  2.98518  4.58477  3.61503
          0.02187  4.22952  4.95186  0.61958  0.77255  0.48576  0.95510
    300   2.66766  4.88476  2.91190  2.51474  4.35788  3.36856  3.77428
3.80655  2.53725  3.37333  4.18227  3.01246  2.51950  1.89413  2.96281
2.02868  2.95786  3.41274  5.56307  4.21981    313 q - - -
          2.68618  4.42225  2.77519  2.73123  3.46354  2.40513  3.72494
3.29354  2.67741  2.69355  4.24690  2.90347  2.73739  3.18146  2.89801
2.37887  2.77519  2.98518  4.58477  3.61503
          0.02187  4.22952  4.95186  0.61958  0.77255  0.48576  0.95510
    301   2.78717  4.94070  3.14753  2.60611  4.22211  3.54895  3.70219
3.58510  1.84885  3.18148  4.03140  3.09280  3.94820  1.93194  2.64308
2.81934  3.00907  2.39144  5.40558  4.11581    314 k - - -
          2.68618  4.42225  2.77519  2.73123  3.46354  2.40513  3.72494
3.29354  2.67741  2.69355  4.24690  2.90347  2.73739  3.18146  2.89801
2.37887  2.77519  2.98518  4.58477  3.61503
          0.02187  4.22952  4.95186  0.61958  0.77255  0.48576  0.95510
    302   2.89599  4.46984  3.94667  3.39073  3.36477  3.93564  4.19273
1.70244  3.21026  1.67856  3.27348  3.74657  4.28097  2.36665  3.48068
3.22550  3.12712  2.40024  5.05116  3.84878    315 l - - -
```

```
          2.68618  4.42225  2.77519  2.73123  3.46354  2.40513  3.72494
 3.29354  2.67741  2.69355  4.24690  2.90347  2.73739  3.18146  2.89801
 2.37887  2.77519  2.98518  4.58477  3.61503
          0.02187  4.22952  4.95186  0.61958  0.77255  0.48576  0.95510
     303  2.75919  4.45014  3.87556  3.41928  3.60880  3.65529  4.28323
 2.63343  3.26718  1.75811  3.55929  3.70273  4.17959  3.63564  3.53857
 3.03810  1.41105  2.48426  5.24768  4.00927    316 t - - -
          2.68618  4.42225  2.77519  2.73123  3.46354  2.40513  3.72494
 3.29354  2.67741  2.69355  4.24690  2.90347  2.73739  3.18146  2.89801
 2.37887  2.77519  2.98518  4.58477  3.61503
          0.02187  4.22952  4.95186  0.61958  0.77255  0.48576  0.95510
     304  1.67997  4.43455  4.02929  3.58527  3.53495  3.71778  4.39238
 2.54744  3.46077  1.32352  3.45976  3.82843  4.24013  3.78141  3.70755
 3.11017  3.12732  2.42917  5.24058  4.02915    317 l - - -
          2.68618  4.42225  2.77519  2.73123  3.46354  2.40513  3.72494
 3.29354  2.67741  2.69355  4.24690  2.90347  2.73739  3.18146  2.89801
 2.37887  2.77519  2.98518  4.58477  3.61503
          0.02187  4.22952  4.95186  0.61958  0.77255  0.48576  0.95510
     305  3.29869  4.97961  3.94636  3.84728  4.78758  3.64774  4.88149
 4.44313  3.93886  4.02289  5.09758  4.11130  0.36966  4.31567  4.13026
 3.48053  3.77053  4.04412  5.85897  4.91765    318 P - - -
          2.68618  4.42225  2.77519  2.73123  3.46354  2.40513  3.72494
 3.29354  2.67741  2.69355  4.24690  2.90347  2.73739  3.18146  2.89801
 2.37887  2.77519  2.98518  4.58477  3.61503
          0.02187  4.22952  4.95186  0.61958  0.77255  0.48576  0.95510
     306  3.37644  4.65384  5.09944  4.58639  3.39419  4.73397  5.15900
 1.04314  4.42574  1.40411  3.20096  4.80567  4.94848  4.60637  4.55372
 4.12744  3.61960  1.93012  5.47128  4.32444    319 i - - -
          2.68618  4.42225  2.77519  2.73123  3.46354  2.40513  3.72494
 3.29354  2.67741  2.69355  4.24690  2.90347  2.73739  3.18146  2.89801
 2.37887  2.77519  2.98518  4.58477  3.61503
          0.02187  4.22952  4.95186  0.61958  0.77255  0.48576  0.95510
     307  2.79997  4.33227  4.01221  3.44090  1.98472  3.84802  4.05018
 1.97478  3.25483  2.20601  3.29411  3.72259  4.20776  3.56937  2.42040
 3.13822  3.03293  2.44473  4.75709  3.43715    320 i - - -
          2.68618  4.42225  2.77519  2.73123  3.46354  2.40513  3.72494
 3.29354  2.67741  2.69355  4.24690  2.90347  2.73739  3.18146  2.89801
 2.37887  2.77519  2.98518  4.58477  3.61503
          0.02187  4.22952  4.95186  0.61958  0.77255  0.48576  0.95510
     308  1.88371  4.27164  3.96689  3.49522  3.79500  3.46367  4.33616
 2.58678  3.42316  2.72646  3.71866  3.68261  4.06227  3.71020  3.69860
 2.85180  2.01379  1.53042  5.33138  4.12483    321 v - - -
          2.68618  4.42225  2.77519  2.73123  3.46354  2.40513  3.72494
 3.29354  2.67741  2.69355  4.24690  2.90347  2.73739  3.18146  2.89801
 2.37887  2.77519  2.98518  4.58477  3.61503
          0.02187  4.22952  4.95186  0.61958  0.77255  0.48576  0.95510
     309  2.79466  4.72284  3.29578  2.85723  3.89941  3.57278  3.92933
 3.11583  2.67102  2.83055  3.85771  3.29548  4.04605  1.51690  2.99893
 2.92114  3.07149  2.06126  5.33097  4.03027    322 q - - -
          2.68618  4.42225  2.77519  2.73123  3.46354  2.40513  3.72494
 3.29354  2.67741  2.69355  4.24690  2.90347  2.73739  3.18146  2.89801
 2.37887  2.77519  2.98518  4.58477  3.61503
          0.02187  4.22952  4.95186  0.61958  0.77255  0.48576  0.95510
```

```
    310   1.01099  2.07494  4.21937  3.87071  4.14485  3.13355  4.59018
3.25729  3.75294  3.18330  4.11707  3.73403  3.89736  4.00291  3.93171
2.59828  2.85085  2.87849  5.61573  4.45132    323 a - - -
          2.68618  4.42225  2.77519  2.73123  3.46354  2.40513  3.72494
3.29354  2.67741  2.69355  4.24690  2.90347  2.73739  3.18146  2.89801
2.37887  2.77519  2.98518  4.58477  3.61503
          0.02187  4.22952  4.95186  0.61958  0.77255  0.48576  0.95510
    311   1.33812  1.46780  4.26037  3.92534  4.13413  3.14188  4.61702
3.22410  3.79628  3.16689  4.11158  3.76085  3.90836  4.04483  3.95904
2.61103  2.86043  2.85470  5.61420  4.45145    324 a - - -
          2.68618  4.42225  2.77519  2.73123  3.46354  2.40513  3.72494
3.29354  2.67741  2.69355  4.24690  2.90347  2.73739  3.18146  2.89801
2.37887  2.77519  2.98518  4.58477  3.61503
          0.02187  4.22952  4.95186  0.61958  0.77255  0.48576  0.95510
    312   2.58308  4.51010  3.29044  2.83910  3.94071  3.39785  3.94181
3.14768  2.83126  2.96815  3.85937  1.96417  3.92424  3.17630  3.21755
2.73142  2.10311  2.12131  5.32890  4.05505    325 n - - -
          2.68618  4.42225  2.77519  2.73123  3.46354  2.40513  3.72494
3.29354  2.67741  2.69355  4.24690  2.90347  2.73739  3.18146  2.89801
2.37887  2.77519  2.98518  4.58477  3.61503
          0.02187  4.22952  4.95186  0.61958  0.77255  0.48576  0.95510
    313   2.24044  4.31628  4.34699  3.77659  3.30432  4.02297  4.37565
2.25579  3.66026  1.62038  2.28373  4.01103  4.36475  3.88047  3.85218
3.33002  3.11386  1.79592  5.01037  3.84560    326 l - - -
          2.68618  4.42225  2.77519  2.73123  3.46354  2.40513  3.72494
3.29354  2.67741  2.69355  4.24690  2.90347  2.73739  3.18146  2.89801
2.37887  2.77519  2.98518  4.58477  3.61503
          0.02187  4.22952  4.95186  0.61958  0.77255  0.48576  0.95510
    314   2.94528  5.12784  2.79053  2.56049  3.96256  3.48299  1.48029
3.97414  2.62485  3.50054  4.38404  2.03354  4.01568  3.05951  2.99773
2.93990  3.22137  3.62266  5.33183  3.83540    327 h - - -
          2.68618  4.42225  2.77519  2.73123  3.46354  2.40513  3.72494
3.29354  2.67741  2.69355  4.24690  2.90347  2.73739  3.18146  2.89801
2.37887  2.77519  2.98518  4.58477  3.61503
          0.02187  4.22952  4.95186  0.61958  0.77255  0.48576  0.95510
    315   3.58296  4.94778  4.76280  4.41894  3.31381  4.43210  4.96505
2.55063  4.18445  0.53374  3.25075  4.66084  4.81524  4.46461  4.29594
4.06845  3.86586  2.67792  5.30656  4.10752    328 L - - -
          2.68618  4.42225  2.77519  2.73123  3.46354  2.40513  3.72494
3.29354  2.67741  2.69355  4.24690  2.90347  2.73739  3.18146  2.89801
2.37887  2.77519  2.98518  4.58477  3.61503
          0.02187  4.22952  4.95186  0.61958  0.77255  0.48576  0.95510
    316   2.74027  4.32381  3.91218  3.36069  3.32918  3.76144  4.10945
2.58401  3.23940  1.57973  2.35430  3.66853  4.15683  3.53382  3.50125
2.21937  2.99245  2.45664  4.93494  3.72672    329 l - - -
          2.68618  4.42225  2.77519  2.73123  3.46354  2.40513  3.72494
3.29354  2.67741  2.69355  4.24690  2.90347  2.73739  3.18146  2.89801
2.37887  2.77519  2.98518  4.58477  3.61503
          0.02187  4.22952  4.95186  0.61958  0.77255  0.48576  0.95510
    317   3.53420  4.83210  5.05087  4.55174  1.90090  4.68841  4.63052
2.43901  4.39976  0.83660  3.02069  4.68264  4.87709  4.42969  4.45782
4.06726  3.75537  2.61148  4.80761  3.39488    330 l - - -
          2.68618  4.42225  2.77519  2.73123  3.46354  2.40513  3.72494
3.29354  2.67741  2.69355  4.24690  2.90347  2.73739  3.18146  2.89801
2.37887  2.77519  2.98518  4.58477  3.61503
```

```
          0.02187  4.22952  4.95186  0.61958  0.77255  0.48576  0.95510
    318   3.03443  4.71870  3.72345  3.27142  3.43096  3.89867  4.14643
2.80805  2.96876  1.10089  3.40512  3.66044  4.29480  2.38814  3.22458
3.26590  3.27710  2.79300  5.12947  3.86482    331 l - - -
          2.68618  4.42225  2.77519  2.73123  3.46354  2.40513  3.72494
3.29354  2.67741  2.69355  4.24690  2.90347  2.73739  3.18146  2.89801
2.37887  2.77519  2.98518  4.58477  3.61503
          0.02187  4.22952  4.95186  0.61958  0.77255  0.48576  0.95510
    319   3.28256  5.35719  3.77447  3.01950  4.87750  3.85562  3.72245
4.19316  1.48274  3.58764  4.47095  3.38352  4.20458  2.85408  1.08242
3.26964  3.42227  3.88078  5.58315  4.46345    332 r - - -
          2.68618  4.42225  2.77519  2.73123  3.46354  2.40513  3.72494
3.29354  2.67741  2.69355  4.24690  2.90347  2.73739  3.18146  2.89801
2.37887  2.77519  2.98518  4.58477  3.61503
          0.02187  4.22952  4.95186  0.61958  0.77255  0.48576  0.95510
    320   3.41937  5.55540  0.51660  2.63395  4.99116  3.52339  4.30456
4.67065  3.46466  4.21929  5.21995  3.20306  4.20198  3.55427  4.01856
3.35108  3.77030  4.27597  6.08415  4.82387    333 D - - -
          2.68618  4.42225  2.77519  2.73123  3.46354  2.40513  3.72494
3.29354  2.67741  2.69355  4.24690  2.90347  2.73739  3.18146  2.89801
2.37887  2.77519  2.98518  4.58477  3.61503
          0.02187  4.22952  4.95186  0.61958  0.77255  0.48576  0.95510
    321   1.67205  4.32981  4.12741  3.57680  3.41768  3.88844  4.30214
1.90503  3.47265  2.19698  2.45374  3.85429  4.28183  3.74129  3.71981
3.20147  3.06801  2.17083  5.07319  3.88333    334 a - - -
          2.68618  4.42225  2.77519  2.73123  3.46354  2.40513  3.72494
3.29354  2.67741  2.69355  4.24690  2.90347  2.73739  3.18146  2.89801
2.37887  2.77519  2.98518  4.58477  3.61503
          0.02187  4.22952  4.95186  0.61958  0.77255  0.48576  0.95510
    322   2.92743  4.41324  4.37798  3.91334  3.65122  4.01579  4.66515
1.12517  3.77678  2.33145  3.52732  4.12917  4.47524  4.09220  3.99954
3.40322  2.17446  1.92806  5.40952  4.19718    335 i - - -
          2.68618  4.42225  2.77519  2.73123  3.46354  2.40513  3.72494
3.29354  2.67741  2.69355  4.24690  2.90347  2.73739  3.18146  2.89801
2.37887  2.77519  2.98518  4.58477  3.61503
          0.02187  4.22952  4.95186  0.61958  0.77255  0.48576  0.95510
    323   2.67769  4.35281  3.61189  3.05453  2.43402  3.66368  3.91426
2.76043  2.97296  2.03210  3.38917  3.43537  4.04359  2.55892  3.29617
2.92624  2.26425  2.57931  4.88424  3.62252    336 l - - -
          2.68618  4.42225  2.77519  2.73123  3.46354  2.40513  3.72494
3.29354  2.67741  2.69355  4.24690  2.90347  2.73739  3.18146  2.89801
2.37887  2.77519  2.98518  4.58477  3.61503
          0.02187  4.22952  4.95186  0.61958  0.77255  0.48576  0.95510
    324   2.71862  4.53063  3.39657  2.87521  2.47868  3.61721  3.75963
3.08371  2.85326  2.74454  3.65240  2.35946  4.01478  3.16298  3.23223
2.20843  2.95803  2.84485  4.71445  2.49468    337 s - - -
          2.68618  4.42225  2.77519  2.73123  3.46354  2.40513  3.72494
3.29354  2.67741  2.69355  4.24690  2.90347  2.73739  3.18146  2.89801
2.37887  2.77519  2.98518  4.58477  3.61503
          0.02187  4.22952  4.95186  0.61958  0.77255  0.48576  0.95510
    325   2.40293  4.30625  3.58384  3.19019  4.12033  1.72536  4.20800
3.45256  3.19537  3.20093  4.06176  3.41259  3.85646  3.49197  3.52683
1.79917  2.83672  2.02866  5.51060  4.28313    338 g - - -
```

2.68618 4.42225 2.77519 2.73123 3.46354 2.40513 3.72494
3.29354 2.67741 2.69355 4.24690 2.90347 2.73739 3.18146 2.89801
2.37887 2.77519 2.98518 4.58477 3.61503
0.02187 4.22952 4.95186 0.61958 0.77255 0.48576 0.95510
326 2.69942 5.07938 2.30849 2.01807 4.34909 3.41450 3.65790
3.81943 2.43922 3.35671 4.14290 2.90103 3.84521 2.18939 2.91981
2.67846 2.93580 3.44098 2.98079 4.14177 339 e - - -
2.68618 4.42225 2.77519 2.73123 3.46354 2.40513 3.72494
3.29354 2.67741 2.69355 4.24690 2.90347 2.73739 3.18146 2.89801
2.37887 2.77519 2.98518 4.58477 3.61503
0.02187 4.22952 4.95186 0.61958 0.77255 0.48576 0.95510
327 2.72154 4.98081 2.72150 1.65759 4.43465 2.31885 3.79954
3.86524 2.63978 3.44640 4.26030 2.95606 3.88103 2.95354 3.11249
2.73076 2.14352 3.47277 5.65187 4.28361 340 e - - -
2.68618 4.42225 2.77519 2.73123 3.46354 2.40513 3.72494
3.29354 2.67741 2.69355 4.24690 2.90347 2.73739 3.18146 2.89801
2.37887 2.77519 2.98518 4.58477 3.61503
0.02187 4.22952 4.95186 0.61958 0.77255 0.48576 0.95510
328 3.08405 4.61635 4.05195 3.54429 1.80906 4.00502 3.73469
3.08497 3.39251 2.60966 3.67813 3.75154 4.36068 2.27464 3.62882
3.30139 3.31193 2.92827 1.96191 2.51363 341 f - - -
2.68618 4.42225 2.77519 2.73123 3.46354 2.40513 3.72494
3.29354 2.67741 2.69355 4.24690 2.90347 2.73739 3.18146 2.89801
2.37887 2.77519 2.98518 4.58477 3.61503
0.02187 4.22952 4.95186 0.61958 0.77255 0.48576 0.95510
329 2.79327 5.11111 2.65907 2.40256 4.53890 1.95411 3.79149
4.01479 2.59282 3.55053 4.36205 2.08570 3.90275 2.12850 3.04478
2.77717 3.07604 3.60741 5.71262 4.32863 342 g - - -
2.68618 4.42225 2.77519 2.73123 3.46354 2.40513 3.72494
3.29354 2.67741 2.69355 4.24690 2.90347 2.73739 3.18146 2.89801
2.37887 2.77519 2.98518 4.58477 3.61503
0.02187 4.22952 4.95186 0.61958 0.77255 0.48576 0.95510
330 3.28440 4.59586 4.97123 4.43981 3.37807 4.59069 5.01253
1.03977 4.28020 1.80576 2.26534 4.65497 4.83752 4.46897 4.41616
3.96226 3.52780 1.97096 5.39544 4.26536 343 i - - -
2.68618 4.42225 2.77519 2.73123 3.46354 2.40513 3.72494
3.29354 2.67741 2.69355 4.24690 2.90347 2.73739 3.18146 2.89801
2.37887 2.77519 2.98518 4.58477 3.61503
0.02187 4.22952 4.95186 0.61958 0.77255 0.48576 0.95510
331 2.90411 5.25440 1.16748 2.33647 4.77159 3.30056 3.93969
4.29611 2.94765 3.84752 4.69336 2.89019 3.93940 3.11722 3.50615
1.91216 3.24184 3.84107 5.99703 4.53987 344 d - - -
2.68618 4.42225 2.77519 2.73123 3.46354 2.40513 3.72494
3.29354 2.67741 2.69355 4.24690 2.90347 2.73739 3.18146 2.89801
2.37887 2.77519 2.98518 4.58477 3.61503
0.02187 4.22952 4.95186 0.61958 0.77255 0.48576 0.95510
332 2.66567 4.83245 3.07716 2.59500 4.30676 3.40811 3.76300
3.71247 1.71370 3.29946 4.11615 3.07059 3.88730 2.91440 2.83356
2.08299 2.16303 3.33995 5.49972 4.19113 345 k - - -
2.68618 4.42225 2.77519 2.73123 3.46354 2.40513 3.72494
3.29354 2.67741 2.69355 4.24690 2.90347 2.73739 3.18146 2.89801
2.37887 2.77519 2.98518 4.58477 3.61503
0.02187 4.22952 4.95186 0.61958 0.77255 0.48576 0.95510

```
    333   2.91174  5.22651  2.67991  2.44535  4.54026  3.43395  3.80200
4.08845  2.52259  3.60094  4.44184  1.77947  3.96298  1.59754  2.91300
2.88013  3.17961  3.70099  5.69757  4.31157    346 q - - -
         2.68618  4.42225  2.77519  2.73123  3.46354  2.40513  3.72494
3.29354  2.67741  2.69355  4.24690  2.90347  2.73739  3.18146  2.89801
2.37887  2.77519  2.98518  4.58477  3.61503
         0.02187  4.22952  4.95186  0.61958  0.77255  0.48576  0.95510
    334   2.83291  4.98455  2.87437  2.62405  4.51549  2.07852  3.92656
4.02121  2.67003  3.57392  4.42718  3.10985  3.97351  1.31401  3.04186
2.86410  3.15371  3.61675  5.69540  4.37769    347 q - - -
         2.68618  4.42225  2.77519  2.73123  3.46354  2.40513  3.72494
3.29354  2.67741  2.69355  4.24690  2.90347  2.73739  3.18146  2.89801
2.37887  2.77519  2.98518  4.58477  3.61503
         0.02187  4.22952  4.95186  0.61958  0.77255  0.48576  0.95510
    335   2.78197  4.45725  3.70560  3.11379  3.34626  3.73095  3.87631
2.88244  2.09421  1.68887  3.48845  3.47392  4.09937  3.25301  3.03579
3.01297  3.00520  2.70379  2.92189  3.52717    348 l - - -
         2.68618  4.42225  2.77519  2.73123  3.46354  2.40513  3.72494
3.29354  2.67741  2.69355  4.24690  2.90347  2.73739  3.18146  2.89801
2.37887  2.77519  2.98518  4.58477  3.61503
         0.02187  4.22952  4.95186  0.61958  0.77255  0.48576  0.95510
    336   2.87581  5.19570  3.04217  2.54571  4.56507  3.54831  3.67583
3.99622  2.19956  3.47385  4.28270  2.23961  3.95910  1.85580  1.93990
2.85155  3.08953  3.62242  5.57427  4.27064    349 q - - -
         2.68618  4.42225  2.77519  2.73123  3.46354  2.40513  3.72494
3.29354  2.67741  2.69355  4.24690  2.90347  2.73739  3.18146  2.89801
2.37887  2.77519  2.98518  4.58477  3.61503
         0.02187  4.22952  4.95186  0.61958  0.77255  0.48576  0.95510
    337   2.50907  4.34868  3.72717  3.42369  4.11135  1.21848  4.39805
3.19589  3.42447  3.13773  4.10503  3.58782  3.96689  3.73368  3.70058
2.72695  2.95957  1.81953  5.56921  4.34532    350 g - - -
         2.68618  4.42225  2.77519  2.73123  3.46354  2.40513  3.72494
3.29354  2.67741  2.69355  4.24690  2.90347  2.73739  3.18146  2.89801
2.37887  2.77519  2.98518  4.58477  3.61503
         0.02187  4.22952  4.95186  0.61958  0.77255  0.48576  0.95510
    338   3.37890  4.78375  4.42475  3.99992  2.41859  4.26858  3.83802
2.98832  3.78631  1.66419  3.65518  4.06488  4.61170  4.00138  3.93561
3.63046  3.61452  2.93615  4.10450  1.11935    351 y - - -
         2.68618  4.42225  2.77519  2.73123  3.46354  2.40513  3.72494
3.29354  2.67741  2.69355  4.24690  2.90347  2.73739  3.18146  2.89801
2.37887  2.77519  2.98518  4.58477  3.61503
         0.02187  4.22952  4.95186  0.61958  0.77255  0.48576  0.95510
    339   2.84835  4.96315  3.22117  2.73950  4.44399  3.52970  3.79554
3.77458  1.26184  3.37119  4.23487  3.18440  4.00042  2.94954  2.59174
2.89144  2.12136  3.43557  5.54778  4.29163    352 k - - -
         2.68618  4.42225  2.77519  2.73123  3.46354  2.40513  3.72494
3.29354  2.67741  2.69355  4.24690  2.90347  2.73739  3.18146  2.89801
2.37887  2.77519  2.98518  4.58477  3.61503
         0.02187  4.22952  4.95186  0.61958  0.77255  0.48576  0.95510
    340   2.08067  5.43015  1.48291  1.81303  4.75320  3.34715  3.81288
4.23557  2.75195  3.75382  4.57110  2.82252  3.91722  2.96270  3.31232
2.84368  3.19967  3.81668  5.92558  4.45909    353 d - - -
         2.68618  4.42225  2.77519  2.73123  3.46354  2.40513  3.72494
3.29354  2.67741  2.69355  4.24690  2.90347  2.73739  3.18146  2.89801
2.37887  2.77519  2.98518  4.58477  3.61503
```

0.02187 4.22952 4.95186 0.61958 0.77255 0.48576 0.95510
341 2.74378 5.16056 2.71505 1.91123 4.47612 3.40601 3.67985
3.93679 2.45211 3.45569 4.23943 2.32575 3.86028 2.28079 2.92622
2.70939 2.24336 3.54115 5.62060 4.22694 354 e - - -
2.68618 4.42225 2.77519 2.73123 3.46354 2.40513 3.72494
3.29354 2.67741 2.69355 4.24690 2.90347 2.73739 3.18146 2.89801
2.37887 2.77519 2.98518 4.58477 3.61503
0.02187 4.22952 4.95186 0.61958 0.77255 0.48576 0.95510
342 2.89341 4.54788 3.76678 3.22949 2.19745 3.85686 3.97925
2.78372 3.10763 1.75069 3.36683 3.59967 4.21501 2.01822 3.41193
3.13446 3.12383 2.70623 4.76275 3.35483 355 l - - -
2.68618 4.42225 2.77519 2.73123 3.46354 2.40513 3.72494
3.29354 2.67741 2.69355 4.24690 2.90347 2.73739 3.18146 2.89801
2.37887 2.77519 2.98518 4.58477 3.61503
0.02187 4.22952 4.95186 0.61958 0.77255 0.48576 0.95510
343 2.75535 4.94103 3.03309 2.00952 4.20550 3.51525 3.69960
2.52584 1.73913 3.17355 4.01775 3.04209 3.92152 2.84965 2.75377
2.78113 2.98076 3.23399 5.41741 4.10516 356 k - - -
2.68618 4.42225 2.77519 2.73123 3.46354 2.40513 3.72494
3.29354 2.67741 2.69355 4.24690 2.90347 2.73739 3.18146 2.89801
2.37887 2.77519 2.98518 4.58477 3.61503
0.02187 4.22952 4.95186 0.61958 0.77255 0.48576 0.95510
344 3.01785 5.29334 3.25695 2.11134 4.72639 3.66139 3.67618
4.10177 1.70909 3.53527 4.36203 3.14727 4.04186 2.79739 1.45130
2.98720 3.20153 3.74238 5.59211 4.35720 357 r - - -
2.68618 4.42225 2.77519 2.73123 3.46354 2.40513 3.72494
3.29354 2.67741 2.69355 4.24690 2.90347 2.73739 3.18146 2.89801
2.37887 2.77519 2.98518 4.58477 3.61503
0.02187 4.22952 4.95186 0.61958 0.77255 0.48576 0.95510
345 3.01050 4.70791 3.87325 3.28277 3.58602 3.86411 4.04291
2.92707 2.68036 1.23325 3.55546 3.63319 4.25129 3.33032 1.99527
3.22684 3.24182 2.84491 5.15116 3.91155 358 l - - -
2.68618 4.42225 2.77519 2.73123 3.46354 2.40513 3.72494
3.29354 2.67741 2.69355 4.24690 2.90347 2.73739 3.18146 2.89801
2.37887 2.77519 2.98518 4.58477 3.61503
0.02187 4.22952 4.95186 0.61958 0.77255 0.48576 0.95510
346 2.91898 4.40991 4.36134 3.89503 3.64881 4.00245 4.64907
1.14831 3.75967 2.33563 3.52708 4.11296 4.46343 4.07493 3.98439
3.38831 2.12627 1.93750 5.40030 4.18788 359 i - - -
2.68618 4.42225 2.77519 2.73123 3.46354 2.40513 3.72494
3.29354 2.67741 2.69355 4.24690 2.90347 2.73739 3.18146 2.89801
2.37887 2.77519 2.98518 4.58477 3.61503
0.02187 4.22952 4.95186 0.61958 0.77255 0.48576 0.95510
347 2.81448 4.90732 3.23669 2.66793 4.15700 3.59156 3.71314
2.41885 1.60930 3.10768 3.98033 3.14087 3.97730 2.29185 2.60515
2.86249 3.02829 3.21841 5.36086 4.08973 360 k - - -
2.68618 4.42225 2.77519 2.73123 3.46354 2.40513 3.72494
3.29354 2.67741 2.69355 4.24690 2.90347 2.73739 3.18146 2.89801
2.37887 2.77519 2.98518 4.58477 3.61503
0.02187 4.22952 4.95186 0.61958 0.77255 0.48576 0.95510
348 2.17608 4.71563 3.04151 1.72785 4.03956 3.46211 3.83582
3.18856 2.68383 3.01869 3.91108 3.11326 3.93147 3.02561 3.11436
2.77054 2.95161 2.20845 5.39118 4.08371 361 e - - -

2.68618 4.42225 2.77519 2.73123 3.46354 2.40513 3.72494
3.29354 2.67741 2.69355 4.24690 2.90347 2.73739 3.18146 2.89801
2.37887 2.77519 2.98518 4.58477 3.61503
0.02187 4.22952 4.95186 0.61958 0.77255 0.48576 0.95510
349 3.80424 5.14643 4.38673 4.19600 2.40294 4.26283 3.84731
3.76969 4.04504 3.12739 4.40880 4.19212 4.73410 4.26097 4.14895
3.89915 4.09590 3.65865 4.05572 0.53072 362 Y - - -
2.68618 4.42225 2.77519 2.73123 3.46354 2.40513 3.72494
3.29354 2.67741 2.69355 4.24690 2.90347 2.73739 3.18146 2.89801
2.37887 2.77519 2.98518 4.58477 3.61503
0.02187 4.22952 4.95186 0.61958 0.77255 0.48576 0.95510
350 2.37816 4.31108 3.58940 3.28164 4.35734 3.10813 4.32951
3.71520 3.28840 3.45190 4.30439 3.44509 3.84430 3.60581 3.59225
1.71325 1.17569 3.22320 5.71906 4.49637 363 t - - -
2.68618 4.42225 2.77519 2.73123 3.46354 2.40513 3.72494
3.29354 2.67741 2.69355 4.24690 2.90347 2.73739 3.18146 2.89801
2.37887 2.77519 2.98518 4.58477 3.61503
0.02187 4.22952 4.95186 0.61958 0.77255 0.48576 0.95510
351 3.06773 5.60737 1.32457 2.23302 4.92733 3.33084 3.91616
4.50129 2.95892 3.99792 4.85668 1.53187 3.97037 3.09003 3.56265
2.95706 3.37378 4.05439 6.12001 4.60357 364 d - - -
2.68618 4.42225 2.77519 2.73123 3.46354 2.40513 3.72494
3.29354 2.67741 2.69355 4.24690 2.90347 2.73739 3.18146 2.89801
2.37887 2.77519 2.98518 4.58477 3.61503
0.02187 4.22952 4.95186 0.61958 0.77255 0.48576 0.95510
352 2.75387 5.04316 2.17524 2.41461 4.15457 3.44581 3.69481
3.74369 1.97119 3.30572 4.12561 2.94957 3.89068 2.85508 2.94788
2.74663 2.99223 3.39744 5.42259 2.26639 365 k - - -
2.68618 4.42225 2.77519 2.73123 3.46354 2.40513 3.72494
3.29354 2.67741 2.69355 4.24690 2.90347 2.73739 3.18146 2.89801
2.37887 2.77519 2.98518 4.58477 3.61503
0.02187 4.22952 4.95186 0.61958 0.77255 0.48576 0.95510
353 1.80079 4.35459 4.44263 3.91884 3.63093 4.07175 4.63420
1.57009 3.82530 2.36330 3.50327 4.14135 4.48287 4.09051 4.05466
3.42226 3.18870 1.48133 5.35057 4.14555 366 v - - -
2.68618 4.42225 2.77519 2.73123 3.46354 2.40513 3.72494
3.29354 2.67741 2.69355 4.24690 2.90347 2.73739 3.18146 2.89801
2.37887 2.77519 2.98518 4.58477 3.61503
0.02187 4.22952 4.95186 0.61958 0.77255 0.48576 0.95510
354 2.72929 4.33830 3.77228 3.22985 3.40833 3.74684 4.05106
1.99088 3.12650 2.33635 2.10021 2.49780 4.13217 3.44195 3.41894
3.03248 2.97171 2.38395 4.96416 3.73726 367 i - - -
2.68618 4.42225 2.77519 2.73123 3.46354 2.40513 3.72494
3.29354 2.67741 2.69355 4.24690 2.90347 2.73739 3.18146 2.89801
2.37887 2.77519 2.98518 4.58477 3.61503
0.02187 4.22952 4.95186 0.61958 0.77255 0.48576 0.95510
355 2.76115 5.03953 2.94107 2.49621 4.42633 3.45767 3.70766
3.84820 1.68393 3.39028 4.19783 2.20659 3.90545 2.84279 2.74159
2.76142 2.24648 3.47497 5.55298 4.22230 368 k - - -
2.68618 4.42225 2.77519 2.73123 3.46354 2.40513 3.72494
3.29354 2.67741 2.69355 4.24690 2.90347 2.73739 3.18146 2.89801
2.37887 2.77519 2.98518 4.58477 3.61503
0.02187 4.22952 4.95186 0.61958 0.77255 0.48576 0.95510

356 2.67912 4.58008 3.21979 2.10579 3.85591 3.52753 3.88831
2.94001 2.77211 2.83764 3.75699 3.22449 3.97753 3.11871 3.17032
2.82589 2.23141 1.85744 5.26539 3.98992 369 v - - -
2.68618 4.42225 2.77519 2.73123 3.46354 2.40513 3.72494
3.29354 2.67741 2.69355 4.24690 2.90347 2.73739 3.18146 2.89801
2.37887 2.77519 2.98518 4.58477 3.61503
0.02187 4.22952 4.95186 0.61958 0.77255 0.48576 0.95510
357 3.80424 5.14643 4.38673 4.19600 2.40294 4.26283 3.84731
3.76969 4.04504 3.12739 4.40880 4.19212 4.73410 4.26097 4.14895
3.89915 4.09590 3.65865 4.05572 0.53072 370 Y - - -
2.68618 4.42225 2.77519 2.73123 3.46354 2.40513 3.72494
3.29354 2.67741 2.69355 4.24690 2.90347 2.73739 3.18146 2.89801
2.37887 2.77519 2.98518 4.58477 3.61503
0.02187 4.22952 4.95186 0.61958 0.77255 0.48576 0.95510
358 2.63428 4.70343 2.95837 2.71892 4.39733 3.27446 4.01409
3.81239 2.86726 3.48383 4.33975 1.35139 3.90701 3.21640 3.26167
2.72311 1.98401 3.39153 5.68632 4.35911 371 n - - -
2.68618 4.42225 2.77519 2.73123 3.46354 2.40513 3.72494
3.29354 2.67741 2.69355 4.24690 2.90347 2.73739 3.18146 2.89801
2.37887 2.77519 2.98518 4.58477 3.61503
0.02187 4.22952 4.95186 0.61958 0.77255 0.48576 0.95510
359 2.76142 5.05812 1.87791 2.39312 4.27259 3.42907 3.72333
3.72087 2.54052 2.34298 4.12924 2.93505 3.89100 2.19910 3.01204
2.75283 3.00577 3.38866 5.52453 4.14394 372 d - - -
2.68618 4.42225 2.77519 2.73123 3.46354 2.40513 3.72494
3.29354 2.67741 2.69355 4.24690 2.90347 2.73739 3.18146 2.89801
2.37887 2.77519 2.98518 4.58477 3.61503
0.02187 4.22952 4.95186 0.61958 0.77255 0.48576 0.95510
360 2.72471 4.53130 3.59780 3.27074 3.88517 1.23141 4.25532
3.22537 3.22700 1.83015 3.96986 3.56823 4.07349 3.59530 3.51753
2.92449 3.10718 2.97795 5.36476 4.09439 373 g - - -
2.68618 4.42225 2.77519 2.73123 3.46354 2.40513 3.72494
3.29354 2.67741 2.69355 4.24690 2.90347 2.73739 3.18146 2.89801
2.37887 2.77519 2.98518 4.58477 3.61503
0.02187 4.22952 4.95186 0.61958 0.77255 0.48576 0.95510
361 3.53420 4.83210 5.05087 4.55174 1.90090 4.68841 4.63052
2.43901 4.39976 0.83660 3.02069 4.68264 4.87709 4.42969 4.45782
4.06726 3.75537 2.61148 4.80761 3.39488 374 l - - -
2.68618 4.42225 2.77519 2.73123 3.46354 2.40513 3.72494
3.29354 2.67741 2.69355 4.24690 2.90347 2.73739 3.18146 2.89801
2.37887 2.77519 2.98518 4.58477 3.61503
0.02187 4.22952 4.95186 0.61958 0.77255 0.48576 0.95510
362 2.89712 5.39647 2.49583 1.51139 4.70968 3.38823 3.75663
4.19945 2.57446 3.68610 4.48855 2.05445 3.91291 2.22303 3.05964
2.82313 3.15087 3.78288 5.82665 4.39487 375 e - - -
2.68618 4.42225 2.77519 2.73123 3.46354 2.40513 3.72494
3.29354 2.67741 2.69355 4.24690 2.90347 2.73739 3.18146 2.89801
2.37887 2.77519 2.98518 4.58477 3.61503
0.02187 4.22952 4.95186 0.61958 0.77255 0.48576 0.95510
363 2.79824 5.03039 3.01976 1.98452 4.30925 3.52469 3.69056
3.69538 1.68237 2.38375 4.09957 3.03762 3.93339 2.83215 2.67498
2.80527 3.01926 3.37178 5.46781 4.15584 376 k - - -
2.68618 4.42225 2.77519 2.73123 3.46354 2.40513 3.72494
3.29354 2.67741 2.69355 4.24690 2.90347 2.73739 3.18146 2.89801
2.37887 2.77519 2.98518 4.58477 3.61503

0.02187 4.22952 4.95186 0.61958 0.77255 0.48576 0.95510
364 2.74357 4.96293 3.10208 2.05853 4.24687 3.52216 3.66298
2.54694 1.96370 3.21347 4.03236 3.03912 3.90848 2.80261 2.21592
2.76052 2.96181 3.31450 5.41075 4.10408 377 k - - -
2.68618 4.42225 2.77519 2.73123 3.46354 2.40513 3.72494
3.29354 2.67741 2.69355 4.24690 2.90347 2.73739 3.18146 2.89801
2.37887 2.77519 2.98518 4.58477 3.61503
0.02187 4.22952 4.95186 0.61958 0.77255 0.48576 0.95510
365 2.92388 4.86425 3.45543 2.89425 4.06917 3.69806 3.84743
2.35114 1.25194 2.94960 3.94537 3.32416 4.10113 3.03456 2.64103
3.02712 3.14909 2.96921 5.37296 4.11899 378 k - - -
2.68618 4.42225 2.77519 2.73123 3.46354 2.40513 3.72494
3.29354 2.67741 2.69355 4.24690 2.90347 2.73739 3.18146 2.89801
2.37887 2.77519 2.98518 4.58477 3.61503
0.02187 4.22952 4.95186 0.61958 0.77255 0.48576 0.95510
366 2.14752 4.75859 3.09219 2.57497 4.00850 3.48517 3.72022
3.38859 2.10435 2.30657 3.87318 2.46578 3.89762 2.89668 2.89067
2.73710 2.91124 3.09142 5.29787 3.99583 379 k - - -
2.68618 4.42225 2.77519 2.73123 3.46354 2.40513 3.72494
3.29354 2.67741 2.69355 4.24690 2.90347 2.73739 3.18146 2.89801
2.37887 2.77519 2.98518 4.58477 3.61503
0.02187 4.22952 4.95186 0.61958 0.77255 0.48576 0.95510
367 2.97687 5.34191 2.78344 1.62278 4.71505 3.51232 3.74671
4.14215 1.41941 3.62010 4.44831 3.00033 3.98851 2.88164 2.67293
2.92531 3.20432 3.76246 5.71994 4.39404 380 k - - -
2.68618 4.42225 2.77519 2.73123 3.46354 2.40513 3.72494
3.29354 2.67741 2.69355 4.24690 2.90347 2.73739 3.18146 2.89801
2.37887 2.77519 2.98518 4.58477 3.61503
0.20483 4.22952 1.76812 0.61958 0.77255 0.48576 0.95510
368 1.30350 4.26948 3.39574 3.09653 4.22755 3.05561 4.18676
3.54829 3.13604 3.28705 4.16967 3.31974 2.03301 3.45435 3.45761
2.51097 2.79758 3.10200 5.58715 4.36494 381 a - - -
2.68618 4.42225 2.77519 2.73123 3.46354 2.40513 3.72494
3.29354 2.67741 2.69355 4.24690 2.90347 2.73739 3.18146 2.89801
2.37887 2.77519 2.98518 4.58477 3.61503
0.02620 4.05089 4.77324 0.61958 0.77255 0.38139 1.14858
369 2.80766 5.18589 2.10365 1.56654 4.44185 3.39876 3.74195
3.87236 2.59568 2.39858 4.26687 2.88437 3.89248 2.88687 3.10049
2.77217 3.05804 3.51290 5.65709 4.25170 382 e - - -
2.68618 4.42225 2.77519 2.73123 3.46354 2.40513 3.72494
3.29354 2.67741 2.69355 4.24690 2.90347 2.73739 3.18146 2.89801
2.37887 2.77519 2.98518 4.58477 3.61503
0.20483 4.22952 1.76812 0.61958 0.77255 0.48576 0.95510
370 2.85170 5.10577 2.71765 2.44182 4.47555 3.39691 3.71772
3.94562 1.83409 3.47707 4.32363 1.62428 3.90765 2.87437 2.69035
2.83440 3.11003 3.57639 5.58649 4.25214 383 n - - -
2.68618 4.42225 2.77519 2.73123 3.46354 2.40513 3.72494
3.29354 2.67741 2.69355 4.24690 2.90347 2.73739 3.18146 2.89801
2.37887 2.77519 2.98518 4.58477 3.61503
0.02620 4.05089 4.77324 0.61958 0.77255 0.67925 0.70724
371 2.94428 4.39565 4.20413 3.70205 2.83815 3.99825 4.05128
1.51259 3.53846 2.13859 3.32305 3.90392 4.35405 3.79925 3.73190
3.32965 3.18657 2.27254 4.52118 2.05976 384 i - - -

```
          2.68618  4.42225  2.77519  2.73123  3.46354  2.40513  3.72494
3.29354  2.67741  2.69355  4.24690  2.90347  2.73739  3.18146  2.89801
2.37887  2.77519  2.98518  4.58477  3.61503
          0.02620  4.05089  4.77324  0.61958  0.77255  0.67925  0.70724
    372   2.54840  4.64865  2.76928  2.57043  4.35276  3.15703  3.91751
3.88002  2.80092  3.49242  4.32970  1.61813  3.80369  3.11984  3.21485
1.80450  2.93799  3.41570  5.63564  4.27891    385 n - - -
          2.68618  4.42225  2.77519  2.73123  3.46354  2.40513  3.72494
3.29354  2.67741  2.69355  4.24690  2.90347  2.73739  3.18146  2.89801
2.37887  2.77519  2.98518  4.58477  3.61503
          0.02620  4.05089  4.77324  0.61958  0.77255  0.67925  0.70724
    373   2.64114  4.88203  1.88967  2.36078  4.42069  2.29669  3.78413
3.85929  2.68529  3.44949  4.26768  2.88067  3.80561  2.94799  3.18290
2.65675  2.17592  3.44332  5.65587  4.27758    386 d - - -
          2.68618  4.42225  2.77519  2.73123  3.46354  2.40513  3.72494
3.29354  2.67741  2.69355  4.24690  2.90347  2.73739  3.18146  2.89801
2.37887  2.77519  2.98518  4.58477  3.61503
          0.02620  4.05089  4.77324  0.61958  0.77255  0.67925  0.70724
    374   2.73352  4.83509  2.86788  2.57525  4.24741  3.34601  3.81618
3.73440  2.54581  3.29963  4.18853  3.05103  1.71041  2.10444  2.90107
2.78136  3.03948  3.38466  5.48738  4.17412    387 p - - -
          2.68618  4.42225  2.77519  2.73123  3.46354  2.40513  3.72494
3.29354  2.67741  2.69355  4.24690  2.90347  2.73739  3.18146  2.89801
2.37887  2.77519  2.98518  4.58477  3.61503
          0.02620  4.05089  4.77324  0.61958  0.77255  0.67925  0.70724
    375   2.88435  5.28008  2.47060  1.40716  4.57634  3.34748  3.72069
4.05190  2.48971  3.56049  4.40713  2.83672  3.88292  2.11209  2.91625
2.82324  3.13993  3.67480  5.71137  4.31758    388 e - - -
          2.68615  4.42299  2.77509  2.73117  3.46428  2.40451  3.72568
3.29286  2.67781  2.69343  4.24764  2.90421  2.73660  3.18150  2.89769
2.37898  2.77593  2.98535  4.58551  3.61382
          0.43786  1.06093  4.77324  2.42602  0.09254  0.38139  1.14858
    376   2.00820  4.70153  2.95199  2.64079  4.40390  3.26229  3.93182
3.83671  2.78755  3.45015  4.26736  1.62685  2.52094  3.10899  3.22188
2.65868  2.94544  3.39709  5.66130  4.33604    404 n - - -
          2.68618  4.42225  2.77519  2.73123  3.46354  2.40513  3.72494
3.29354  2.67741  2.69355  4.24690  2.90347  2.73739  3.18146  2.89801
2.37887  2.77519  2.98518  4.58477  3.61503
          0.02187  4.22952  4.95186  0.61958  0.77255  0.48576  0.95510
    377   2.08617  4.39799  3.54107  3.00820  2.36899  3.63955  3.82173
2.90821  2.96667  2.60184  3.51953  2.57093  4.03346  3.26142  3.30767
2.90497  2.92989  2.68688  4.70667  2.53512    405 a - - -
          2.68618  4.42225  2.77519  2.73123  3.46354  2.40513  3.72494
3.29354  2.67741  2.69355  4.24690  2.90347  2.73739  3.18146  2.89801
2.37887  2.77519  2.98518  4.58477  3.61503
          0.02187  4.22952  4.95186  0.61958  0.77255  0.48576  0.95510
    378   2.96358  5.14774  3.38490  2.74415  4.56505  3.66040  3.68723
3.92834  1.63995  3.41661  4.25398  3.18906  4.03688  2.81809  1.54575
2.96636  2.36440  3.59120  5.51662  4.29151    406 r - - -
          2.68618  4.42225  2.77519  2.73123  3.46354  2.40513  3.72494
3.29354  2.67741  2.69355  4.24690  2.90347  2.73739  3.18146  2.89801
2.37887  2.77519  2.98518  4.58477  3.61503
          0.20483  4.22952  1.76812  0.61958  0.77255  0.48576  0.95510
```

```
    379   2.89713  5.30998  2.34140  1.64238  4.63145  3.28949  3.77080
4.13342  2.65817  3.66667  4.51525  1.60693  3.87477  2.93643  3.14689
2.82760  3.17981  3.73503  5.80979  4.37476    407 n - - -
          2.68618  4.42225  2.77519  2.73123  3.46354  2.40513  3.72494
3.29354  2.67741  2.69355  4.24690  2.90347  2.73739  3.18146  2.89801
2.37887  2.77519  2.98518  4.58477  3.61503
          0.02620  4.05089  4.77324  0.61958  0.77255  0.67925  0.70724
    380   2.55994  4.26998  3.87019  3.43798  3.74594  3.44860  4.32269
2.43594  3.33793  2.57750  3.66101  3.64766  4.04862  3.67049  3.61215
2.85384  1.64295  1.63807  5.34447  4.11999    408 v - - -
          2.68618  4.42225  2.77519  2.73123  3.46354  2.40513  3.72494
3.29354  2.67741  2.69355  4.24690  2.90347  2.73739  3.18146  2.89801
2.37887  2.77519  2.98518  4.58477  3.61503
          0.02620  4.05089  4.77324  0.61958  0.77255  0.67925  0.70724
    381   2.64216  4.77476  2.32857  2.45214  3.90843  3.41582  2.69865
3.39485  2.50047  3.00734  2.62134  2.96640  3.84143  2.84765  2.94528
2.68672  2.88108  3.09717  5.23679  3.87868    409 d - - -
          2.68618  4.42225  2.77519  2.73123  3.46354  2.40513  3.72494
3.29354  2.67741  2.69355  4.24690  2.90347  2.73739  3.18146  2.89801
2.37887  2.77519  2.98518  4.58477  3.61503
          0.02620  4.05089  4.77324  0.61958  0.77255  0.67925  0.70724
    382   3.09653  5.20812  3.51210  2.83552  4.67650  3.70132  3.63522
4.02005  1.19740  3.45077  4.32339  3.23226  4.06773  2.76899  1.66038
3.08869  3.25884  3.70096  5.48107  4.32160    410 k - - -
          2.68618  4.42225  2.77519  2.73123  3.46354  2.40513  3.72494
3.29354  2.67741  2.69355  4.24690  2.90347  2.73739  3.18146  2.89801
2.37887  2.77519  2.98518  4.58477  3.61503
          0.02620  4.05089  4.77324  0.61958  0.77255  0.67925  0.70724
    383   3.64876  4.95693  4.37475  4.12738  2.87138  3.93916  4.13698
3.68363  3.84764  3.04618  4.30859  4.24130  4.47247  4.22597  3.94343
3.85062  3.95897  3.57880  0.59121  2.86471    411 W - - -
          2.68618  4.42225  2.77519  2.73123  3.46354  2.40513  3.72494
3.29354  2.67741  2.69355  4.24690  2.90347  2.73739  3.18146  2.89801
2.37887  2.77519  2.98518  4.58477  3.61503
          0.02620  4.05089  4.77324  0.61958  0.77255  0.67925  0.70724
    384   2.88117  4.87751  2.82257  2.72791  4.27011  3.31154  4.07951
4.02278  3.00034  3.65026  4.60713  0.87702  3.97372  3.35551  3.35855
2.94339  3.27072  3.62671  5.55571  4.20790    412 n - - -
          2.68618  4.42225  2.77519  2.73123  3.46354  2.40513  3.72494
3.29354  2.67741  2.69355  4.24690  2.90347  2.73739  3.18146  2.89801
2.37887  2.77519  2.98518  4.58477  3.61503
          0.02620  4.05089  4.77324  0.61958  0.77255  0.38139  1.14858
    385   2.68872  4.80476  3.10986  2.56105  3.96686  3.50737  2.77158
3.44102  2.01152  3.05154  3.88824  3.05850  3.89916  2.86138  2.82963
2.74018  2.26740  3.13944  5.25635  2.86730    413 k - - -
          2.68618  4.42225  2.77519  2.73123  3.46354  2.40513  3.72494
3.29354  2.67741  2.69355  4.24690  2.90347  2.73739  3.18146  2.89801
2.37887  2.77519  2.98518  4.58477  3.61503
          0.02187  4.22952  4.95186  0.61958  0.77255  0.48576  0.95510
    386   2.97588  4.37078  4.57334  4.04378  3.62818  4.20539  4.72550
1.36380  3.93873  2.32577  3.48828  4.26074  4.57626  4.20138  4.15081
3.55394  2.18424  1.43316  5.38389  4.18102    414 i - - -
          2.68618  4.42225  2.77519  2.73123  3.46354  2.40513  3.72494
3.29354  2.67741  2.69355  4.24690  2.90347  2.73739  3.18146  2.89801
2.37887  2.77519  2.98518  4.58477  3.61503
```

```
         0.02187  4.22952  4.95186  0.61958  0.77255  0.48576  0.95510
    387  2.03100  4.56932  3.21410  2.77354  3.87399  3.44427  3.88617
3.18026  2.76405  2.13624  3.81914  1.99058  3.93723  3.12120  3.15075
2.76689  2.92693  2.90785  5.26845  3.97866    415 n - - -
         2.68618  4.42225  2.77519  2.73123  3.46354  2.40513  3.72494
3.29354  2.67741  2.69355  4.24690  2.90347  2.73739  3.18146  2.89801
2.37887  2.77519  2.98518  4.58477  3.61503
         0.02187  4.22952  4.95186  0.61958  0.77255  0.48576  0.95510
    388  2.23671  5.26693  2.02108  1.92902  4.58455  3.38305  3.69202
4.06496  1.99190  3.56134  4.33656  2.85067  3.85962  2.81433  3.00829
2.71939  3.02007  3.64554  5.71214  4.28716    416 e - - -
         2.68618  4.42225  2.77519  2.73123  3.46354  2.40513  3.72494
3.29354  2.67741  2.69355  4.24690  2.90347  2.73739  3.18146  2.89801
2.37887  2.77519  2.98518  4.58477  3.61503
         0.02187  4.22952  4.95186  0.61958  0.77255  0.48576  0.95510
    389  3.98789  5.12769  4.92363  4.68751  1.37439  4.65031  3.56786
3.63623  4.51386  2.90744  4.21157  4.28522  4.94301  4.38481  4.47528
4.03732  4.20332  3.57233  3.67794  0.84206    417 y - - -
         2.68618  4.42225  2.77519  2.73123  3.46354  2.40513  3.72494
3.29354  2.67741  2.69355  4.24690  2.90347  2.73739  3.18146  2.89801
2.37887  2.77519  2.98518  4.58477  3.61503
         0.02187  4.22952  4.95186  0.61958  0.77255  0.48576  0.95510
    390  2.72163  4.62586  3.36975  2.77592  3.82176  3.60137  3.77168
2.46781  2.02580  2.82248  3.71466  3.22442  3.97878  2.99019  2.18446
2.85072  2.94403  2.32680  5.16422  3.91242    418 k - - -
         2.68618  4.42225  2.77519  2.73123  3.46354  2.40513  3.72494
3.29354  2.67741  2.69355  4.24690  2.90347  2.73739  3.18146  2.89801
2.37887  2.77519  2.98518  4.58477  3.61503
         0.02187  4.22952  4.95186  0.61958  0.77255  0.48576  0.95510
    391  2.83462  4.85175  3.46748  2.91147  4.36675  3.54137  3.84707
3.71351  2.31524  3.30923  4.19619  3.29931  4.02805  3.01816  1.29092
2.91657  2.07581  3.38365  5.49932  4.27586    419 r - - -
         2.68618  4.42225  2.77519  2.73123  3.46354  2.40513  3.72494
3.29354  2.67741  2.69355  4.24690  2.90347  2.73739  3.18146  2.89801
2.37887  2.77519  2.98518  4.58477  3.61503
         0.02187  4.22952  4.95186  0.61958  0.77255  0.48576  0.95510
    392  2.71019  4.77907  2.93121  1.78250  2.53046  2.43917  3.77836
3.41515  2.66679  3.05630  3.92214  3.05665  3.91794  2.98728  3.11038
2.77407  2.96636  3.12290  5.28424  3.92883    420 e - - -
         2.68618  4.42225  2.77519  2.73123  3.46354  2.40513  3.72494
3.29354  2.67741  2.69355  4.24690  2.90347  2.73739  3.18146  2.89801
2.37887  2.77519  2.98518  4.58477  3.61503
         0.02187  4.22952  4.95186  0.61958  0.77255  0.48576  0.95510
    393  3.05304  4.53376  4.22671  3.72376  2.76199  4.04425  4.00074
2.73691  3.53560  2.18721  1.66000  3.91851  4.40173  3.80141  3.72765
3.37245  3.29190  2.66846  4.44045  1.73231    421 m - - -
         2.68618  4.42225  2.77519  2.73123  3.46354  2.40513  3.72494
3.29354  2.67741  2.69355  4.24690  2.90347  2.73739  3.18146  2.89801
2.37887  2.77519  2.98518  4.58477  3.61503
         0.02187  4.22952  4.95186  0.61958  0.77255  0.48576  0.95510
    394  1.67947  4.28361  3.65012  3.26064  4.31619  3.10131  4.28451
3.68310  3.25595  3.39726  4.22880  3.42872  3.82114  3.54992  3.58346
1.72938  1.57425  3.19144  5.67088  4.45807    422 t - - -
```

2.68618 4.42225 2.77519 2.73123 3.46354 2.40513 3.72494
3.29354 2.67741 2.69355 4.24690 2.90347 2.73739 3.18146 2.89801
2.37887 2.77519 2.98518 4.58477 3.61503
0.02187 4.22952 4.95186 0.61958 0.77255 0.48576 0.95510
395 3.51917 4.83695 5.06598 4.51316 3.12527 4.74732 5.02848
2.31848 4.31708 0.78413 1.99323 4.77791 4.90000 4.42371 4.41262
4.11922 3.73826 2.50749 5.26474 4.21169 423 l - - -
2.68618 4.42225 2.77519 2.73123 3.46354 2.40513 3.72494
3.29354 2.67741 2.69355 4.24690 2.90347 2.73739 3.18146 2.89801
2.37887 2.77519 2.98518 4.58477 3.61503
0.02187 4.22952 4.95186 0.61958 0.77255 0.48576 0.95510
396 2.69481 4.81955 2.94599 2.64016 4.36583 3.34341 3.89393
3.86160 2.65336 3.44292 4.28703 3.10090 3.91552 2.07183 3.03091
1.35379 3.02967 3.45631 5.60840 4.27208 424 s - - -
2.68618 4.42225 2.77519 2.73123 3.46354 2.40513 3.72494
3.29354 2.67741 2.69355 4.24690 2.90347 2.73739 3.18146 2.89801
2.37887 2.77519 2.98518 4.58477 3.61503
0.02187 4.22952 4.95186 0.61958 0.77255 0.48576 0.95510
397 1.91106 4.34157 4.28101 3.88375 3.85906 3.67407 4.68316
2.24647 3.79776 2.61332 3.74396 3.99486 4.28334 4.09020 4.02323
3.10389 3.11116 1.04822 5.54881 4.33589 425 v - - -
2.68618 4.42225 2.77519 2.73123 3.46354 2.40513 3.72494
3.29354 2.67741 2.69355 4.24690 2.90347 2.73739 3.18146 2.89801
2.37887 2.77519 2.98518 4.58477 3.61503
0.02187 4.22952 4.95186 0.61958 0.77255 0.48576 0.95510
398 3.58296 4.94778 4.76280 4.41894 3.31381 4.43210 4.96505
2.55063 4.18445 0.53374 3.25075 4.66084 4.81524 4.46461 4.29594
4.06845 3.86586 2.67792 5.30656 4.10752 426 L - - -
2.68618 4.42225 2.77519 2.73123 3.46354 2.40513 3.72494
3.29354 2.67741 2.69355 4.24690 2.90347 2.73739 3.18146 2.89801
2.37887 2.77519 2.98518 4.58477 3.61503
0.02187 4.22952 4.95186 0.61958 0.77255 0.48576 0.95510
399 2.91104 5.26432 1.14162 2.33367 4.77889 3.30072 3.94250
4.30511 2.95486 3.85635 4.70400 2.88839 3.94159 3.12080 3.51565
1.95463 3.24924 3.84992 6.00497 4.54542 427 d - - -
2.68618 4.42225 2.77519 2.73123 3.46354 2.40513 3.72494
3.29354 2.67741 2.69355 4.24690 2.90347 2.73739 3.18146 2.89801
2.37887 2.77519 2.98518 4.58477 3.61503
0.02187 4.22952 4.95186 0.61958 0.77255 0.48576 0.95510
400 2.93172 4.51619 3.92038 3.38587 1.86899 3.92268 4.01496
2.72976 3.25411 1.69404 3.31997 3.71154 4.27278 2.32103 3.52480
3.21087 3.16179 2.66688 4.67841 3.25070 428 l - - -
2.68618 4.42225 2.77519 2.73123 3.46354 2.40513 3.72494
3.29354 2.67741 2.69355 4.24690 2.90347 2.73739 3.18146 2.89801
2.37887 2.77519 2.98518 4.58477 3.61503
0.02187 4.22952 4.95186 0.61958 0.77255 0.48576 0.95510
401 1.91106 4.34157 4.28101 3.88375 3.85906 3.67407 4.68316
2.24647 3.79776 2.61332 3.74396 3.99486 4.28334 4.09020 4.02323
3.10389 3.11116 1.04822 5.54881 4.33589 429 v - - -
2.68618 4.42225 2.77519 2.73123 3.46354 2.40513 3.72494
3.29354 2.67741 2.69355 4.24690 2.90347 2.73739 3.18146 2.89801
2.37887 2.77519 2.98518 4.58477 3.61503
0.02187 4.22952 4.95186 0.61958 0.77255 0.48576 0.95510

402 1.56453 5.01968 1.46883 2.45108 4.60490 3.30388 3.95532
3.99598 2.91954 3.64452 4.49928 2.97479 3.92560 3.13536 3.43370
2.80662 3.14039 3.58341 5.85991 4.46794 430 d - - -
2.68618 4.42225 2.77519 2.73123 3.46354 2.40513 3.72494
3.29354 2.67741 2.69355 4.24690 2.90347 2.73739 3.18146 2.89801
2.37887 2.77519 2.98518 4.58477 3.61503
0.02187 4.22952 4.95186 0.61958 0.77255 0.48576 0.95510
403 3.58296 4.94778 4.76280 4.41894 3.31381 4.43210 4.96505
2.55063 4.18445 0.53374 3.25075 4.66084 4.81524 4.46461 4.29594
4.06845 3.86586 2.67792 5.30656 4.10752 431 L - - -
2.68618 4.42225 2.77519 2.73123 3.46354 2.40513 3.72494
3.29354 2.67741 2.69355 4.24690 2.90347 2.73739 3.18146 2.89801
2.37887 2.77519 2.98518 4.58477 3.61503
0.02187 4.22952 4.95186 0.61958 0.77255 0.48576 0.95510
404 3.84852 5.02579 4.95073 4.67076 0.89690 4.58594 3.63722
3.45344 4.46749 2.72435 4.02119 4.30488 4.88684 4.37513 4.42988
3.98431 4.06897 3.41160 1.97277 2.02963 432 f - - -
2.68618 4.42225 2.77519 2.73123 3.46354 2.40513 3.72494
3.29354 2.67741 2.69355 4.24690 2.90347 2.73739 3.18146 2.89801
2.37887 2.77519 2.98518 4.58477 3.61503
0.02187 4.22952 4.95186 0.61958 0.77255 0.48576 0.95510
405 2.45378 4.42335 3.35806 3.12375 4.46555 3.13550 4.28923
3.94128 3.22705 3.60216 4.44425 3.37397 1.26169 3.54514 3.55818
1.70907 2.92694 3.40395 5.78475 4.53292 433 p - - -
2.68618 4.42225 2.77519 2.73123 3.46354 2.40513 3.72494
3.29354 2.67741 2.69355 4.24690 2.90347 2.73739 3.18146 2.89801
2.37887 2.77519 2.98518 4.58477 3.61503
0.02187 4.22952 4.95186 0.61958 0.77255 0.48576 0.95510
406 1.93007 4.27823 3.68682 3.19693 3.85609 3.29478 4.12944
3.09196 3.15133 2.91016 3.79955 3.44647 3.90353 3.44594 3.47739
2.05653 2.03124 1.96830 5.28873 4.06994 434 a - - -
2.68618 4.42225 2.77519 2.73123 3.46354 2.40513 3.72494
3.29354 2.67741 2.69355 4.24690 2.90347 2.73739 3.18146 2.89801
2.37887 2.77519 2.98518 4.58477 3.61503
0.02187 4.22952 4.95186 0.61958 0.77255 0.48576 0.95510
407 2.79358 4.40881 4.02158 3.57429 1.58734 3.70808 4.13417
2.74541 3.48517 2.40275 3.52814 3.77628 4.21261 3.74782 3.71781
3.08533 1.77269 2.59435 4.75840 3.30978 435 f - - -
2.68618 4.42225 2.77519 2.73123 3.46354 2.40513 3.72494
3.29354 2.67741 2.69355 4.24690 2.90347 2.73739 3.18146 2.89801
2.37887 2.77519 2.98518 4.58477 3.61503
0.02187 4.22952 4.95186 0.61958 0.77255 0.48576 0.95510
408 2.80231 5.29732 2.08640 2.28414 4.62672 2.38233 3.71567
4.11348 1.95737 3.60643 4.38700 2.25033 3.87123 2.84236 3.05164
2.74293 3.05431 3.68923 5.75556 4.32434 436 k - - -
2.68618 4.42225 2.77519 2.73123 3.46354 2.40513 3.72494
3.29354 2.67741 2.69355 4.24690 2.90347 2.73739 3.18146 2.89801
2.37887 2.77519 2.98518 4.58477 3.61503
0.02187 4.22952 4.95186 0.61958 0.77255 0.48576 0.95510
409 2.88886 4.58612 3.80613 3.42347 1.93765 3.67286 4.09937
3.11924 3.39099 2.66696 3.78592 3.69671 1.32874 3.68314 3.66852
3.10547 3.22064 2.94836 4.78406 3.28352 437 p - - -
2.68618 4.42225 2.77519 2.73123 3.46354 2.40513 3.72494
3.29354 2.67741 2.69355 4.24690 2.90347 2.73739 3.18146 2.89801
2.37887 2.77519 2.98518 4.58477 3.61503

```
          0.02187  4.22952  4.95186  0.61958  0.77255  0.48576  0.95510
    410   2.80897  5.18410  2.56897  1.90177  4.57442  3.35976  3.78274
4.04785  2.63456  3.58154  4.38859  2.11520  3.89424  2.92926  3.12936
1.68913  3.08616  3.63888  5.75414  4.34490    438 s - - -
          2.68618  4.42225  2.77519  2.73123  3.46354  2.40513  3.72494
3.29354  2.67741  2.69355  4.24690  2.90347  2.73739  3.18146  2.89801
2.37887  2.77519  2.98518  4.58477  3.61503
          0.02187  4.22952  4.95186  0.61958  0.77255  0.48576  0.95510
    411   2.69183  4.66926  3.17966  2.67714  3.74061  3.52282  3.75570
3.26713  2.59051  2.91077  3.78937  3.14703  3.93938  2.25224  2.97415
2.79073  1.99568  2.99790  5.12480  2.60514    439 t - - -
          2.68618  4.42225  2.77519  2.73123  3.46354  2.40513  3.72494
3.29354  2.67741  2.69355  4.24690  2.90347  2.73739  3.18146  2.89801
2.37887  2.77519  2.98518  4.58477  3.61503
          0.02187  4.22952  4.95186  0.61958  0.77255  0.48576  0.95510
    412   3.09723  4.60113  4.11508  3.63670  1.76264  4.01668  3.72790
3.08982  3.54681  2.65147  3.70809  3.79871  2.38609  3.74095  3.76754
3.32457  3.33438  2.92225  4.08935  1.53405    440 y - - -
          2.68618  4.42225  2.77519  2.73123  3.46354  2.40513  3.72494
3.29354  2.67741  2.69355  4.24690  2.90347  2.73739  3.18146  2.89801
2.37887  2.77519  2.98518  4.58477  3.61503
          0.02187  4.22952  4.95186  0.61958  0.77255  0.48576  0.95510
    413   2.74790  5.05007  2.83397  2.45372  4.46689  3.41162  3.73170
3.91707  1.92780  3.45029  4.25147  2.16972  3.89084  2.86879  2.85598
1.83600  3.01075  3.52216  5.60931  4.25500    441 s - - -
          2.68618  4.42225  2.77519  2.73123  3.46354  2.40513  3.72494
3.29354  2.67741  2.69355  4.24690  2.90347  2.73739  3.18146  2.89801
2.37887  2.77519  2.98518  4.58477  3.61503
          0.20483  4.22952  1.76812  0.61958  0.77255  0.48576  0.95510
    414   2.75227  4.97988  2.98781  2.49956  4.32463  3.46017  3.63724
3.72492  1.84424  3.26920  4.09588  2.99480  3.88134  2.09403  2.55792
2.75920  2.30281  3.38196  5.43851  4.13984    442 k - - -
          2.68618  4.42225  2.77519  2.73123  3.46354  2.40513  3.72494
3.29354  2.67741  2.69355  4.24690  2.90347  2.73739  3.18146  2.89801
2.37887  2.77519  2.98518  4.58477  3.61503
          0.02620  4.05089  4.77324  0.61958  0.77255  0.67925  0.70724
    415   1.50173  4.21424  3.41698  3.20194  4.39563  1.40178  4.31186
3.76466  3.32208  3.49911  4.34637  3.35249  3.73717  3.59738  3.62158
2.45835  2.77861  3.21831  5.73077  4.53527    443 g - - -
          2.68618  4.42225  2.77519  2.73123  3.46354  2.40513  3.72494
3.29354  2.67741  2.69355  4.24690  2.90347  2.73739  3.18146  2.89801
2.37887  2.77519  2.98518  4.58477  3.61503
          0.02620  4.05089  4.77324  0.61958  0.77255  0.67925  0.70724
    416   2.16978  4.34678  4.24886  3.70970  3.36998  4.01508  4.43107
1.70248  3.59192  1.58681  3.24464  3.98612  4.37824  3.86278  3.82637
3.34155  3.13679  1.97674  5.13405  3.94866    444 l - - -
          2.68618  4.42225  2.77519  2.73123  3.46354  2.40513  3.72494
3.29354  2.67741  2.69355  4.24690  2.90347  2.73739  3.18146  2.89801
2.37887  2.77519  2.98518  4.58477  3.61503
          0.02620  4.05089  4.77324  0.61958  0.77255  0.67925  0.70724
    417   2.78384  4.63219  3.41611  2.87413  3.73939  3.61097  3.81318
3.01749  1.87310  2.62402  1.98966  3.29089  4.01701  3.04489  2.73733
2.93089  3.01799  2.83325  5.15386  3.90972    445 k - - -
```

2.68618 4.42225 2.77519 2.73123 3.46354 2.40513 3.72494
3.29354 2.67741 2.69355 4.24690 2.90347 2.73739 3.18146 2.89801
2.37887 2.77519 2.98518 4.58477 3.61503
0.02620 4.05089 4.77324 0.61958 0.77255 0.38139 1.14858
418 2.79683 4.44518 3.73599 3.19056 3.53601 3.80282 4.09520
1.93199 3.06308 2.36901 3.45206 3.57882 4.18175 2.07986 3.37976
3.08438 3.03841 1.86462 5.11588 3.88694 446 v - - -
2.68618 4.42225 2.77519 2.73123 3.46354 2.40513 3.72494
3.29354 2.67741 2.69355 4.24690 2.90347 2.73739 3.18146 2.89801
2.37887 2.77519 2.98518 4.58477 3.61503
0.02187 4.22952 4.95186 0.61958 0.77255 0.48576 0.95510
419 2.75635 5.01193 3.09131 2.11910 4.31667 3.52152 3.65615
3.71975 1.94239 3.27019 4.08113 3.03061 3.90891 2.78859 2.07818
2.76242 2.97367 2.48336 5.44679 4.13664 447 k - - -
2.68618 4.42225 2.77519 2.73123 3.46354 2.40513 3.72494
3.29354 2.67741 2.69355 4.24690 2.90347 2.73739 3.18146 2.89801
2.37887 2.77519 2.98518 4.58477 3.61503
0.02187 4.22952 4.95186 0.61958 0.77255 0.48576 0.95510
420 2.92851 5.23321 2.64027 2.44083 4.54393 3.42247 3.82772
4.10843 2.57552 3.62667 4.47665 1.36653 3.97070 2.07389 2.97223
2.89654 3.20570 3.72036 5.72044 4.32561 448 n - - -
2.68618 4.42225 2.77519 2.73123 3.46354 2.40513 3.72494
3.29354 2.67741 2.69355 4.24690 2.90347 2.73739 3.18146 2.89801
2.37887 2.77519 2.98518 4.58477 3.61503
0.02187 4.22952 4.95186 0.61958 0.77255 0.48576 0.95510
421 2.37019 4.31647 3.57112 3.26627 4.41156 3.09462 4.33208
3.78970 3.28525 3.51005 4.35191 3.43294 3.83554 3.59875 3.59644
1.10413 1.82338 3.26977 5.75978 4.53457 449 s - - -
2.68618 4.42225 2.77519 2.73123 3.46354 2.40513 3.72494
3.29354 2.67741 2.69355 4.24690 2.90347 2.73739 3.18146 2.89801
2.37887 2.77519 2.98518 4.58477 3.61503
0.02187 4.22952 4.95186 0.61958 0.77255 0.48576 0.95510
422 3.45811 5.24133 3.98966 3.44371 4.68990 3.85675 4.15125
4.27051 2.47144 3.72137 4.72482 3.76921 4.35346 3.36496 0.54787
3.53317 3.70697 3.98190 5.63415 4.55403 450 R - - -
2.68618 4.42225 2.77519 2.73123 3.46354 2.40513 3.72494
3.29354 2.67741 2.69355 4.24690 2.90347 2.73739 3.18146 2.89801
2.37887 2.77519 2.98518 4.58477 3.61503
0.02187 4.22952 4.95186 0.61958 0.77255 0.48576 0.95510
423 3.06211 5.22702 3.34093 2.78546 4.60681 3.68466 3.73066
4.03550 2.09787 3.48707 4.35480 3.22532 4.08655 1.39187 1.74077
3.05602 3.25703 3.70478 5.55233 4.32413 451 q - - -
2.68618 4.42225 2.77519 2.73123 3.46354 2.40513 3.72494
3.29354 2.67741 2.69355 4.24690 2.90347 2.73739 3.18146 2.89801
2.37887 2.77519 2.98518 4.58477 3.61503
0.02187 4.22952 4.95186 0.61958 0.77255 0.48576 0.95510
424 3.38021 4.65727 5.10421 4.58937 3.38552 4.73703 5.15796
1.06260 4.42930 1.37208 3.19076 4.80876 4.94841 4.60461 4.55482
4.12954 3.62248 1.94082 5.46521 4.32285 452 i - - -
2.68618 4.42225 2.77519 2.73123 3.46354 2.40513 3.72494
3.29354 2.67741 2.69355 4.24690 2.90347 2.73739 3.18146 2.89801
2.37887 2.77519 2.98518 4.58477 3.61503
0.02187 4.22952 4.95186 0.61958 0.77255 0.48576 0.95510

```
    425   3.98523  5.12746  4.91807  4.68060  1.40805  4.64772  3.56856
 3.63519  4.50760  2.90716  4.21119  4.28321  4.94123  4.38194  4.47161
 4.03488  4.20103  3.57110  3.67929  0.82389    453 y - - -
          2.68618  4.42225  2.77519  2.73123  3.46354  2.40513  3.72494
 3.29354  2.67741  2.69355  4.24690  2.90347  2.73739  3.18146  2.89801
 2.37887  2.77519  2.98518  4.58477  3.61503
          0.02187  4.22952  4.95186  0.61958  0.77255  0.48576  0.95510
    426   1.67515  4.28673  3.64291  3.25506  4.34320  3.09499  4.28672
 3.71715  3.25362  3.42502  4.25206  3.42395  3.81745  3.54738  3.58399
 1.47728  1.84444  3.21297  5.69146  4.47759    454 s - - -
          2.68618  4.42225  2.77519  2.73123  3.46354  2.40513  3.72494
 3.29354  2.67741  2.69355  4.24690  2.90347  2.73739  3.18146  2.89801
 2.37887  2.77519  2.98518  4.58477  3.61503
          0.02187  4.22952  4.95186  0.61958  0.77255  0.48576  0.95510
    427   2.55160  4.39550  3.45539  2.97296  3.77887  3.40445  3.98052
 2.17109  2.93631  2.82676  3.73081  3.33460  2.49835  3.26496  3.29293
 1.79447  2.87560  2.78866  5.20534  3.95482    455 s - - -
          2.68618  4.42225  2.77519  2.73123  3.46354  2.40513  3.72494
 3.29354  2.67741  2.69355  4.24690  2.90347  2.73739  3.18146  2.89801
 2.37887  2.77519  2.98518  4.58477  3.61503
          0.02187  4.22952  4.95186  0.61958  0.77255  0.48576  0.95510
    428   2.84606  4.33429  4.22700  3.69397  3.51498  3.95951  4.41875
 1.51669  3.59181  2.30750  3.42252  3.95505  2.50541  3.86754  3.83343
 3.28783  3.11260  1.61073  5.17846  3.97345    456 i - - -
          2.68630  4.42237  2.77532  2.73136  3.46366  2.40525  3.72507
 3.29366  2.67753  2.69367  4.24702  2.90359  2.73752  3.18083  2.89813
 2.37865  2.77532  2.98531  4.58489  3.61283
          0.20483  1.72517  4.95186  1.19068  0.36242  0.48576  0.95510
    429   2.67531  4.86927  2.98675  2.02318  4.12295  3.46506  3.68133
 2.59142  2.09045  3.13075  3.95273  2.99860  3.87222  2.83407  2.87724
 2.26973  2.90811  3.19900  5.37080  4.04228    461 e - - -
          2.68618  4.42225  2.77519  2.73123  3.46354  2.40513  3.72494
 3.29354  2.67741  2.69355  4.24690  2.90347  2.73739  3.18146  2.89801
 2.37887  2.77519  2.98518  4.58477  3.61503
          0.02187  4.22952  4.95186  0.61958  0.77255  0.48576  0.95510
    430   2.69040  4.75956  2.85649  2.73945  4.63244  1.19287  4.15142
 4.17758  3.09935  3.78012  4.62433  1.89544  3.93477  3.37200  3.51391
 2.77386  3.11651  3.65751  5.88200  4.56467    462 g - - -
          2.68618  4.42225  2.77519  2.73123  3.46354  2.40513  3.72494
 3.29354  2.67741  2.69355  4.24690  2.90347  2.73739  3.18146  2.89801
 2.37887  2.77519  2.98518  4.58477  3.61503
          0.02187  4.22952  4.95186  0.61958  0.77255  0.48576  0.95510
    431   2.19205  4.62026  2.34446  2.65385  3.88874  3.48097  3.80327
 2.36745  2.68241  2.90195  3.77930  3.13276  3.91588  3.00958  3.11458
 2.74661  2.35411  2.84295  5.25790  3.96576    463 a - - -
          2.68618  4.42225  2.77519  2.73123  3.46354  2.40513  3.72494
 3.29354  2.67741  2.69355  4.24690  2.90347  2.73739  3.18146  2.89801
 2.37887  2.77519  2.98518  4.58477  3.61503
          0.02187  4.22952  4.95186  0.61958  0.77255  0.48576  0.95510
    432   2.68598  4.37626  3.92023  3.52574  3.79135  3.55849  4.39805
 2.58128  3.44698  2.67008  3.74589  3.74468  2.31130  3.77472  3.70905
 2.98121  3.05650  1.14638  5.37248  4.14476    464 v - - -
          2.68618  4.42225  2.77519  2.73123  3.46354  2.40513  3.72494
 3.29354  2.67741  2.69355  4.24690  2.90347  2.73739  3.18146  2.89801
 2.37887  2.77519  2.98518  4.58477  3.61503
```

```
          0.02187  4.22952  4.95186  0.61958  0.77255  0.48576  0.95510
    433   2.50593  4.37360  3.45897  2.95899  3.82284  2.32751  3.97240
2.36915  2.93230  2.88600  3.75951  3.31247  3.89664  3.24764  3.29945
1.99920  2.21803  2.85531  5.22679  3.98354    465 s - - -
          2.68618  4.42225  2.77519  2.73123  3.46354  2.40513  3.72494
3.29354  2.67741  2.69355  4.24690  2.90347  2.73739  3.18146  2.89801
2.37887  2.77519  2.98518  4.58477  3.61503
          0.02187  4.22952  4.95186  0.61958  0.77255  0.48576  0.95510
    434   2.71382  4.91330  2.94431  2.55643  4.41849  2.31771  3.79566
3.85116  1.89096  3.41368  4.22812  3.04663  2.07576  2.94726  2.90294
2.74410  3.00369  3.45779  5.59035  4.27010    466 k - - -
          2.68618  4.42225  2.77519  2.73123  3.46354  2.40513  3.72494
3.29354  2.67741  2.69355  4.24690  2.90347  2.73739  3.18146  2.89801
2.37887  2.77519  2.98518  4.58477  3.61503
          0.02187  4.22952  4.95186  0.61958  0.77255  0.48576  0.95510
    435   2.65786  4.38222  3.51439  2.95982  3.50030  3.62133  3.88437
2.21854  2.86655  2.02028  3.45993  3.36343  4.00862  2.52968  3.20471
2.35541  2.89410  2.59757  4.96877  3.73126    467 l - - -
          2.68618  4.42225  2.77519  2.73123  3.46354  2.40513  3.72494
3.29354  2.67741  2.69355  4.24690  2.90347  2.73739  3.18146  2.89801
2.37887  2.77519  2.98518  4.58477  3.61503
          0.15591  4.22952  2.04170  0.61958  0.77255  0.48576  0.95510
    436   2.65829  4.90334  1.87983  2.37953  4.44131  2.31610  3.79944
3.88170  2.69704  3.46883  4.28487  2.89968  3.82379  2.96167  3.19493
2.67320  2.08310  3.46438  5.67361  4.29482    468 d - - -
          2.68618  4.42225  2.77519  2.73123  3.46354  2.40513  3.72494
3.29354  2.67741  2.69355  4.24690  2.90347  2.73739  3.18146  2.89801
2.37887  2.77519  2.98518  4.58477  3.61503
          0.02496  4.09857  4.82092  0.61958  0.77255  0.63433  0.75564
    437   2.35192  4.36200  3.20584  2.89908  4.38477  2.08425  4.09267
3.83279  3.01405  3.46777  4.27715  3.20432  2.07522  3.31092  3.39717
1.72705  2.79863  3.30060  5.67965  4.41897    469 s - - -
          2.68618  4.42225  2.77519  2.73123  3.46354  2.40513  3.72494
3.29354  2.67741  2.69355  4.24690  2.90347  2.73739  3.18146  2.89801
2.37887  2.77519  2.98518  4.58477  3.61503
          0.02496  4.09857  4.82092  0.61958  0.77255  0.63433  0.75564
    438   2.65503  4.70159  3.03419  2.66090  4.13689  3.35839  3.82700
3.51292  2.55560  3.14697  4.04148  3.10821  3.89012  2.09278  2.90379
2.73815  1.64827  3.18855  5.42604  4.12970    470 t - - -
          2.68618  4.42225  2.77519  2.73123  3.46354  2.40513  3.72494
3.29354  2.67741  2.69355  4.24690  2.90347  2.73739  3.18146  2.89801
2.37887  2.77519  2.98518  4.58477  3.61503
          0.02496  4.09857  4.82092  0.61958  0.77255  0.63433  0.75564
    439   2.81585  5.27115  1.82676  2.22986  4.60941  2.35359  3.73180
4.10152  2.61862  3.61459  4.42281  2.79574  3.84954  2.01618  3.14368
2.75404  3.08632  3.68609  5.77652  4.33811    471 d - - -
          2.68618  4.42225  2.77519  2.73123  3.46354  2.40513  3.72494
3.29354  2.67741  2.69355  4.24690  2.90347  2.73739  3.18146  2.89801
2.37887  2.77519  2.98518  4.58477  3.61503
          0.02496  4.09857  4.82092  0.61958  0.77255  0.63433  0.75564
    440   2.67420  4.67926  3.04295  2.13105  3.81108  3.48389  2.56348
2.33818  2.53288  2.88742  3.76775  3.06885  3.89790  2.92590  2.92685
2.76038  2.90795  2.95687  5.16814  3.85050    472 e - - -
```

```
         2.68618  4.42225  2.77519  2.73123  3.46354  2.40513  3.72494
3.29354  2.67741  2.69355  4.24690  2.90347  2.73739  3.18146  2.89801
2.37887  2.77519  2.98518  4.58477  3.61503
         0.02496  4.09857  4.82092  0.61958  0.77255  0.40295  1.10367
    441  2.52006  4.63500  3.05588  2.67382  4.38379  2.34303  3.92145
3.82581  2.77321  3.42487  4.22529  2.12265  3.83188  3.09164  3.21374
1.94029  2.18932  3.36776  5.63595  4.31986    473 s - - -
         2.68618  4.42225  2.77519  2.73123  3.46354  2.40513  3.72494
3.29354  2.67741  2.69355  4.24690  2.90347  2.73739  3.18146  2.89801
2.37887  2.77519  2.98518  4.58477  3.61503
         0.02187  4.22952  4.95186  0.61958  0.77255  0.48576  0.95510
    442  2.62419  4.73160  2.99170  2.63779  4.31692  1.72689  3.87975
3.73758  2.67988  3.34743  4.17658  3.09501  3.88152  2.28145  3.08440
2.69616  2.22854  3.34205  5.57099  4.26024    474 g - - -
         2.68618  4.42225  2.77519  2.73123  3.46354  2.40513  3.72494
3.29354  2.67741  2.69355  4.24690  2.90347  2.73739  3.18146  2.89801
2.37887  2.77519  2.98518  4.58477  3.61503
         0.02187  4.22952  4.95186  0.61958  0.77255  0.48576  0.95510
    443  2.75931  4.93524  1.88492  2.50358  4.46472  3.32765  3.93516
3.83308  2.86144  3.50411  4.36449  3.01442  3.92385  3.11815  3.34467
2.79126  1.38582  3.45240  5.73998  4.37443    475 t - - -
         2.68618  4.42225  2.77519  2.73123  3.46354  2.40513  3.72494
3.29354  2.67741  2.69355  4.24690  2.90347  2.73739  3.18146  2.89801
2.37887  2.77519  2.98518  4.58477  3.61503
         0.02187  4.22952  4.95186  0.61958  0.77255  0.48576  0.95510
    444  3.79340  5.00644  4.87987  4.57558  1.47759  4.53654  3.66514
3.41622  4.37230  2.69707  3.99108  4.28152  4.85155  4.33404  4.36917
3.94300  4.01973  3.37417  1.16300  2.07550    476 w - - -
         2.68618  4.42225  2.77519  2.73123  3.46354  2.40513  3.72494
3.29354  2.67741  2.69355  4.24690  2.90347  2.73739  3.18146  2.89801
2.37887  2.77519  2.98518  4.58477  3.61503
         0.02187  4.22952  4.95186  0.61958  0.77255  0.48576  0.95510
    445  2.84430  5.23055  2.74015  1.96721  4.59046  2.37904  3.71738
4.03694  1.58525  3.53778  4.33987  2.94139  3.91849  2.84827  2.80541
2.80514  3.08447  3.64412  5.67504  4.31244    477 k - - -
         2.68618  4.42225  2.77519  2.73123  3.46354  2.40513  3.72494
3.29354  2.67741  2.69355  4.24690  2.90347  2.73739  3.18146  2.89801
2.37887  2.77519  2.98518  4.58477  3.61503
         0.19097  4.22952  1.83706  0.61958  0.77255  0.48576  0.95510
    446  2.70377  4.84423  2.17517  2.42677  2.42590  3.39388  3.69784
3.48764  2.62605  3.10565  3.97071  2.27764  3.86724  2.91875  3.09657
2.73455  2.95800  3.18350  5.19440  3.75346    478 d - - -
         2.68618  4.42225  2.77519  2.73123  3.46354  2.40513  3.72494
3.29354  2.67741  2.69355  4.24690  2.90347  2.73739  3.18146  2.89801
2.37887  2.77519  2.98518  4.58477  3.61503
         0.02584  4.06439  4.78674  0.61958  0.77255  0.66695  0.72005
    447  3.18289  4.55391  4.59995  4.22640  3.51034  4.26726  4.92265
0.86752  4.05827  2.06161  3.42045  4.44543  4.68386  4.38940  4.23345
3.78476  3.48021  1.87046  5.43794  4.19007    479 i - - -
         2.68618  4.42225  2.77519  2.73123  3.46354  2.40513  3.72494
3.29354  2.67741  2.69355  4.24690  2.90347  2.73739  3.18146  2.89801
2.37887  2.77519  2.98518  4.58477  3.61503
         0.02584  4.06439  4.78674  0.61958  0.77255  0.66695  0.72005
```

```
    448   2.83711  5.11685  2.59026  2.37856  4.41604  3.34999  3.74735
3.97087  2.49259  3.50225  4.35533  1.57932  3.89233  2.12228  2.88507
2.81358  3.11305  3.59367  5.60758  4.21897    480 n - - -
          2.68618  4.42225  2.77519  2.73123  3.46354  2.40513  3.72494
3.29354  2.67741  2.69355  4.24690  2.90347  2.73739  3.18146  2.89801
2.37887  2.77519  2.98518  4.58477  3.61503
          0.02584  4.06439  4.78674  0.61958  0.77255  0.66695  0.72005
    449   2.82492  5.16716  2.81865  2.42192  4.52005  3.44814  3.63749
3.95636  1.82187  3.44450  4.25707  2.24955  3.89049  2.01881  2.56483
2.79156  3.04789  3.58093  5.56002  4.23453    481 k - - -
          2.68618  4.42225  2.77519  2.73123  3.46354  2.40513  3.72494
3.29354  2.67741  2.69355  4.24690  2.90347  2.73739  3.18146  2.89801
2.37887  2.77519  2.98518  4.58477  3.61503
          0.02584  4.06439  4.78674  0.61958  0.77255  0.66695  0.72005
    450   2.82065  4.47377  3.77773  3.21698  3.54305  3.75156  4.02123
1.53470  2.77981  2.39572  3.49914  3.56384  4.15450  3.33516  2.22750
3.09130  3.06799  2.40777  5.08947  3.85018    482 i - - -
          2.68618  4.42225  2.77519  2.73123  3.46354  2.40513  3.72494
3.29354  2.67741  2.69355  4.24690  2.90347  2.73739  3.18146  2.89801
2.37887  2.77519  2.98518  4.58477  3.61503
          0.02584  4.06439  4.78674  0.61958  0.77255  0.66695  0.72005
    451   3.37692  4.78464  4.49690  4.14170  3.21941  4.22387  4.73759
2.43809  3.90762  0.67041  3.18970  4.39853  4.62883  4.22966  4.05061
3.82335  3.66389  2.52449  5.16406  3.93032    483 L - - -
          2.68618  4.42225  2.77519  2.73123  3.46354  2.40513  3.72494
3.29354  2.67741  2.69355  4.24690  2.90347  2.73739  3.18146  2.89801
2.37887  2.77519  2.98518  4.58477  3.61503
          0.02584  4.06439  4.78674  0.61958  0.77255  0.66695  0.72005
    452   2.65056  4.73698  2.27754  2.48142  3.83164  3.40439  3.70182
3.36235  2.59239  3.00826  3.86912  2.98841  3.85716  2.91148  3.04181
2.70848  2.29942  3.06784  5.19979  2.49494    484 d - - -
          2.68618  4.42225  2.77519  2.73123  3.46354  2.40513  3.72494
3.29354  2.67741  2.69355  4.24690  2.90347  2.73739  3.18146  2.89801
2.37887  2.77519  2.98518  4.58477  3.61503
          0.02584  4.06439  4.78674  0.61958  0.77255  0.66695  0.72005
    453   2.30078  4.27098  3.27469  3.06343  4.43991  1.75327  4.24012
3.91549  3.22068  3.57698  4.39651  3.27969  3.73645  3.50006  3.55320
1.30904  2.79202  3.32497  5.75242  4.51785    485 s - - -
          2.68618  4.42225  2.77519  2.73123  3.46354  2.40513  3.72494
3.29354  2.67741  2.69355  4.24690  2.90347  2.73739  3.18146  2.89801
2.37887  2.77519  2.98518  4.58477  3.61503
          0.02584  4.06439  4.78674  0.61958  0.77255  0.66695  0.72005
    454   2.85354  4.74141  3.31402  2.85364  3.62488  3.63452  3.85727
3.04973  2.57349  1.82124  3.64143  3.29512  4.05676  1.79987  2.87329
2.97810  3.09566  2.92253  5.14338  3.82569    486 q - - -
          2.68637  4.42244  2.77538  2.73142  3.46372  2.40497  3.72513
3.29288  2.67759  2.69373  4.24708  2.90308  2.73710  3.18165  2.89763
2.37872  2.77538  2.98537  4.58495  3.61522
          0.24650  1.56005  4.78674  1.53163  0.24358  0.66695  0.72005
    455   2.84799  5.12343  2.46647  1.37538  4.59667  1.95858  3.84637
4.06874  2.75255  3.63330  4.48610  2.88403  3.88491  3.02301  3.23298
2.82271  3.15889  3.66369  5.77775  4.40677    493 e - - -
          2.68618  4.42225  2.77519  2.73123  3.46354  2.40513  3.72494
3.29354  2.67741  2.69355  4.24690  2.90347  2.73739  3.18146  2.89801
2.37887  2.77519  2.98518  4.58477  3.61503
```

```
         0.02584  4.06439  4.78674  0.61958  0.77255  0.66695  0.72005
    456   3.70795  4.92724  4.68029  4.38102  1.31150  4.43994  3.48995
3.38975  4.22418  2.70969  3.98284  4.11135  4.74612  4.17601  4.24610
3.81245  3.92886  3.31636  3.62373  1.07382    494 y - - -
         2.68618  4.42225  2.77519  2.73123  3.46354  2.40513  3.72494
3.29354  2.67741  2.69355  4.24690  2.90347  2.73739  3.18146  2.89801
2.37887  2.77519  2.98518  4.58477  3.61503
         0.02584  4.06439  4.78674  0.61958  0.77255  0.66695  0.72005
    457   1.89979  4.78257  3.08624  2.66053  4.28724  3.39007  3.77256
3.60814  1.52633  3.25195  4.12098  3.10423  3.89843  2.94114  2.68470
2.76475  2.99266  3.26773  5.46994  4.19925    495 k - - -
         2.68618  4.42225  2.77519  2.73123  3.46354  2.40513  3.72494
3.29354  2.67741  2.69355  4.24690  2.90347  2.73739  3.18146  2.89801
2.37887  2.77519  2.98518  4.58477  3.61503
         0.02584  4.06439  4.78674  0.61958  0.77255  0.66695  0.72005
    458   2.92115  4.67335  3.63390  3.55975  4.69058  0.48611  4.66442
4.34068  3.76689  3.96945  4.94964  3.79878  4.07039  4.07616  3.97813
3.09921  3.41733  3.83193  5.73107  4.80033    496 G - - -
         2.68618  4.42225  2.77519  2.73123  3.46354  2.40513  3.72494
3.29354  2.67741  2.69355  4.24690  2.90347  2.73739  3.18146  2.89801
2.37887  2.77519  2.98518  4.58477  3.61503
         0.02584  4.06439  4.78674  0.61958  0.77255  0.66695  0.72005
    459   2.78050  5.20727  1.93878  1.81152  4.52249  3.30152  3.70692
3.97664  2.59246  3.52279  4.33470  2.79051  3.83216  2.85270  3.11750
2.72810  2.10751  3.58350  5.71010  4.28324    497 e - - -
         2.68618  4.42225  2.77519  2.73123  3.46354  2.40513  3.72494
3.29354  2.67741  2.69355  4.24690  2.90347  2.73739  3.18146  2.89801
2.37887  2.77519  2.98518  4.58477  3.61503
         0.02584  4.06439  4.78674  0.61958  0.77255  0.38715  1.13628
    460   2.88590  4.36471  4.30572  3.76556  3.45045  4.00101  4.45719
2.19242  3.64257  1.50550  3.34055  4.01415  4.39128  3.91032  3.86635
3.33251  2.22036  1.61946  5.16716  3.98138    498 l - - -
         2.68618  4.42225  2.77519  2.73123  3.46354  2.40513  3.72494
3.29354  2.67741  2.69355  4.24690  2.90347  2.73739  3.18146  2.89801
2.37887  2.77519  2.98518  4.58477  3.61503
         0.02187  4.22952  4.95186  0.61958  0.77255  0.48576  0.95510
    461   2.64356  4.58869  3.20682  2.14605  3.75298  3.52641  2.82402
3.12551  2.61929  2.79644  2.66747  3.14053  3.91701  2.97472  3.02603
2.76100  2.87548  2.38371  5.12850  3.84596    499 e - - -
         2.68618  4.42225  2.77519  2.73123  3.46354  2.40513  3.72494
3.29354  2.67741  2.69355  4.24690  2.90347  2.73739  3.18146  2.89801
2.37887  2.77519  2.98518  4.58477  3.61503
         0.02187  4.22952  4.95186  0.61958  0.77255  0.48576  0.95510
    462   2.25507  4.81641  3.26184  2.66869  4.06937  3.56888  3.70873
3.43781  1.97419  2.27913  3.90694  3.13844  3.95206  2.87952  2.10947
2.82101  2.97542  3.15086  5.30175  4.03368    500 k - - -
         2.68618  4.42225  2.77519  2.73123  3.46354  2.40513  3.72494
3.29354  2.67741  2.69355  4.24690  2.90347  2.73739  3.18146  2.89801
2.37887  2.77519  2.98518  4.58477  3.61503
         0.02187  4.22952  4.95186  0.61958  0.77255  0.48576  0.95510
    463   2.21311  4.27339  4.17699  3.62186  2.13386  3.87365  3.95647
2.63621  3.50261  1.88155  3.30943  3.80254  4.23222  3.70445  3.68376
3.16829  3.03987  2.48392  4.46081  2.16951    501 l - - -
```

```
          2.68618  4.42225  2.77519  2.73123  3.46354  2.40513  3.72494
 3.29354  2.67741  2.69355  4.24690  2.90347  2.73739  3.18146  2.89801
 2.37887  2.77519  2.98518  4.58477  3.61503
          0.02187  4.22952  4.95186  0.61958  0.77255  0.48576  0.95510
    464   2.97342  5.56627  1.47438  1.78646  4.85951  3.34955  3.80981
 4.37765  2.74533  3.85062  4.66367  2.80069  3.92541  2.03881  3.30915
 2.86837  3.24296  3.93823  5.99635  4.50582    502 d - - -
          2.68618  4.42225  2.77519  2.73123  3.46354  2.40513  3.72494
 3.29354  2.67741  2.69355  4.24690  2.90347  2.73739  3.18146  2.89801
 2.37887  2.77519  2.98518  4.58477  3.61503
          0.02187  4.22952  4.95186  0.61958  0.77255  0.48576  0.95510
    465   2.84606  4.33429  4.22700  3.69397  3.51498  3.95951  4.41875
 1.51669  3.59181  2.30750  3.42252  3.95505  2.50541  3.86754  3.83343
 3.28783  3.11260  1.61073  5.17846  3.97345    503 i - - -
          2.68618  4.42225  2.77519  2.73123  3.46354  2.40513  3.72494
 3.29354  2.67741  2.69355  4.24690  2.90347  2.73739  3.18146  2.89801
 2.37887  2.77519  2.98518  4.58477  3.61503
          0.02187  4.22952  4.95186  0.61958  0.77255  0.48576  0.95510
    466   2.89894  5.20465  3.08440  2.57402  4.58271  3.56838  3.67734
 4.00655  2.17332  3.47904  4.29210  2.19591  3.97445  2.11313  1.70768
 2.87554  3.10841  3.63657  5.57259  4.28155    504 r - - -
          2.68618  4.42225  2.77519  2.73123  3.46354  2.40513  3.72494
 3.29354  2.67741  2.69355  4.24690  2.90347  2.73739  3.18146  2.89801
 2.37887  2.77519  2.98518  4.58477  3.61503
          0.02187  4.22952  4.95186  0.61958  0.77255  0.48576  0.95510
    467   2.63796  4.78228  3.08008  2.60997  4.27784  3.38400  3.78584
 3.68423  1.93199  3.28312  4.10172  3.07914  3.87881  2.94371  2.89848
 1.81152  2.18541  3.30954  5.49521  4.18705    505 s - - -
          2.68618  4.42225  2.77519  2.73123  3.46354  2.40513  3.72494
 3.29354  2.67741  2.69355  4.24690  2.90347  2.73739  3.18146  2.89801
 2.37887  2.77519  2.98518  4.58477  3.61503
          0.02187  4.22952  4.95186  0.61958  0.77255  0.48576  0.95510
    468   3.15415  4.58224  4.36341  3.89608  2.63944  4.14761  3.97001
 1.85603  3.71778  2.33827  3.53141  4.02039  4.50401  3.94607  3.88439
 3.49127  3.39644  2.56461  4.31688  1.27843    506 y - - -
          2.68618  4.42225  2.77519  2.73123  3.46354  2.40513  3.72494
 3.29354  2.67741  2.69355  4.24690  2.90347  2.73739  3.18146  2.89801
 2.37887  2.77519  2.98518  4.58477  3.61503
          0.02187  4.22952  4.95186  0.61958  0.77255  0.48576  0.95510
    469   2.21008  5.08960  1.80114  2.38156  4.45383  3.39309  3.71592
 3.90199  2.49752  3.44511  4.24039  2.91996  3.86833  2.84915  2.19676
 2.71560  2.99102  3.50919  5.62107  4.23790    507 d - - -
          2.68618  4.42225  2.77519  2.73123  3.46354  2.40513  3.72494
 3.29354  2.67741  2.69355  4.24690  2.90347  2.73739  3.18146  2.89801
 2.37887  2.77519  2.98518  4.58477  3.61503
          0.02187  4.22952  4.95186  0.61958  0.77255  0.48576  0.95510
    470   3.15051  5.27025  3.50994  2.88690  4.68755  3.75460  3.73068
 4.08718  2.02228  3.51657  4.39554  3.29540  4.13770  2.01503  1.15954
 3.14497  3.32598  3.76717  5.55646  4.36889    508 r - - -
          2.68618  4.42225  2.77519  2.73123  3.46354  2.40513  3.72494
 3.29354  2.67741  2.69355  4.24690  2.90347  2.73739  3.18146  2.89801
 2.37887  2.77519  2.98518  4.58477  3.61503
          0.02187  4.22952  4.95186  0.61958  0.77255  0.48576  0.95510
```

```
    471   3.32169  4.66102  4.81585  4.45732  3.64239  4.43292  5.13400
0.71984  4.29347  2.17033  3.53769  4.65564  4.84343  4.61411  4.45169
3.97643  3.61986  1.92373  5.59674  4.35691    509 i - - -
          2.68618  4.42225  2.77519  2.73123  3.46354  2.40513  3.72494
3.29354  2.67741  2.69355  4.24690  2.90347  2.73739  3.18146  2.89801
2.37887  2.77519  2.98518  4.58477  3.61503
          0.02187  4.22952  4.95186  0.61958  0.77255  0.48576  0.95510
    472   3.09053  5.63956  1.08924  2.22768  4.95668  3.32831  3.93007
4.53789  2.99284  4.03340  4.90029  1.86750  3.97744  3.10749  3.60759
2.97400  3.40075  4.08848  6.15336  4.62793    510 d - - -
          2.68618  4.42225  2.77519  2.73123  3.46354  2.40513  3.72494
3.29354  2.67741  2.69355  4.24690  2.90347  2.73739  3.18146  2.89801
2.37887  2.77519  2.98518  4.58477  3.61503
          0.02187  4.22952  4.95186  0.61958  0.77255  0.48576  0.95510
    473   1.34192  4.73657  3.18556  2.78428  4.30561  3.40167  3.90905
3.62850  1.86272  3.29931  4.16893  3.20615  3.94907  3.09423  2.90295
2.78892  3.02304  3.27677  5.54135  4.27309    511 a - - -
          2.68618  4.42225  2.77519  2.73123  3.46354  2.40513  3.72494
3.29354  2.67741  2.69355  4.24690  2.90347  2.73739  3.18146  2.89801
2.37887  2.77519  2.98518  4.58477  3.61503
          0.02187  4.22952  4.95186  0.61958  0.77255  0.48576  0.95510
    474   3.48582  4.77258  5.13483  4.60397  3.22524  4.78567  5.12712
1.69929  4.42985  0.81214  3.01994  4.84568  4.95182  4.54393  4.52702
4.17365  3.71565  2.21884  5.35859  4.26964    512 l - - -
          2.68618  4.42225  2.77519  2.73123  3.46354  2.40513  3.72494
3.29354  2.67741  2.69355  4.24690  2.90347  2.73739  3.18146  2.89801
2.37887  2.77519  2.98518  4.58477  3.61503
          0.02187  4.22952  4.95186  0.61958  0.77255  0.48576  0.95510
    475   2.27971  5.15042  3.17709  2.58138  4.52518  3.57493  3.65085
3.92981  1.73644  3.41401  4.21942  3.07053  3.95445  2.22947  1.96137
2.83580  3.05537  3.56483  5.52545  4.24015    513 k - - -
          2.68618  4.42225  2.77519  2.73123  3.46354  2.40513  3.72494
3.29354  2.67741  2.69355  4.24690  2.90347  2.73739  3.18146  2.89801
2.37887  2.77519  2.98518  4.58477  3.61503
          0.02187  4.22952  4.95186  0.61958  0.77255  0.48576  0.95510
    476   1.97520  4.76191  3.00531  2.60113  4.25922  3.35756  3.82441
3.67326  2.60997  3.27896  4.11016  3.07332  2.10697  2.18594  3.02185
2.69561  2.94061  3.30089  5.51328  4.19690    514 a - - -
          2.68618  4.42225  2.77519  2.73123  3.46354  2.40513  3.72494
3.29354  2.67741  2.69355  4.24690  2.90347  2.73739  3.18146  2.89801
2.37887  2.77519  2.98518  4.58477  3.61503
          0.02187  4.22952  4.95186  0.61958  0.77255  0.48576  0.95510
    477   3.27265  4.66564  4.59205  4.08842  2.73851  4.26679  4.15736
2.70496  3.81634  1.14369  3.29829  4.20570  4.58702  4.05575  3.94261
3.64379  3.50532  2.73897  1.88330  2.95184    515 l - - -
          2.68618  4.42225  2.77519  2.73123  3.46354  2.40513  3.72494
3.29354  2.67741  2.69355  4.24690  2.90347  2.73739  3.18146  2.89801
2.37887  2.77519  2.98518  4.58477  3.61503
          0.02187  4.22952  4.95186  0.61958  0.77255  0.48576  0.95510
    478   3.17810  4.55189  4.54212  4.04810  1.89132  4.19275  3.85885
2.84202  3.90128  2.27663  2.19113  4.06566  4.51394  4.00577  4.00688
3.51057  3.40358  2.76575  4.12699  1.45686    516 y - - -
          2.68618  4.42225  2.77519  2.73123  3.46354  2.40513  3.72494
3.29354  2.67741  2.69355  4.24690  2.90347  2.73739  3.18146  2.89801
2.37887  2.77519  2.98518  4.58477  3.61503
```

```
          0.02187  4.22952  4.95186  0.61958  0.77255  0.48576  0.95510
    479   2.89494  5.39691  1.93233  1.75101  4.72680  3.34953  3.79337
4.22246  2.70905  3.72713  4.53401  2.82706  3.90610  2.93835  3.25889
1.66452  3.16658  3.79764  5.88982  4.43026    517 s - - -
          2.68618  4.42225  2.77519  2.73123  3.46354  2.40513  3.72494
3.29354  2.67741  2.69355  4.24690  2.90347  2.73739  3.18146  2.89801
2.37887  2.77519  2.98518  4.58477  3.61503
          0.02187  4.22952  4.95186  0.61958  0.77255  0.48576  0.95510
    480   2.68376  5.00853  2.36781  2.39924  4.28466  3.43165  3.65712
3.72935  2.41747  2.48979  4.08101  2.28164  3.84970  2.78859  2.37503
2.67847  2.91849  3.36743  5.47794  4.11124    518 n - - -
          2.68613  4.42263  2.77557  2.73161  3.46299  2.40550  3.72532
3.29235  2.67736  2.69350  4.24727  2.90384  2.73732  3.18114  2.89838
2.37893  2.77557  2.98556  4.58515  3.61109
          0.41163  1.79111  1.76812  2.21081  0.11610  0.48576  0.95510
    481   2.81582  5.24478  2.55888  1.77482  4.57432  3.36200  3.66348
4.03293  1.91468  3.52817  4.33497  2.17634  3.85514  2.79809  2.80889
2.75970  3.05758  3.63725  5.66536  4.28000    532 e - - -
          2.68618  4.42225  2.77519  2.73123  3.46354  2.40513  3.72494
3.29354  2.67741  2.69355  4.24690  2.90347  2.73739  3.18146  2.89801
2.37887  2.77519  2.98518  4.58477  3.61503
          0.02620  4.05089  4.77324  0.61958  0.77255  0.67925  0.70724
    482   2.65449  4.76085  2.96311  2.60196  4.29762  3.31964  3.78204
3.72468  1.89318  3.32151  4.16739  3.05391  3.86070  2.95577  2.78380
1.56762  2.96747  3.34453  5.49539  4.19650    533 s - - -
          2.68621  4.42228  2.77476  2.73126  3.46357  2.40515  3.72497
3.29357  2.67743  2.69358  4.24692  2.90349  2.73742  3.18149  2.89803
2.37890  2.77522  2.98521  4.58480  3.61506
          0.23300  1.61248  4.77324  0.26129  1.46993  0.67925  0.70724
    483   2.57751  4.64327  2.76222  2.62406  4.45554  1.42624  4.01142
3.98479  2.94175  3.59781  4.44502  1.99599  3.82287  3.23079  3.35115
2.66158  2.99234  3.49304  5.71651  4.39754    535 g - - -
          2.68599  4.42241  2.77535  2.73139  3.46370  2.40476  3.72510
3.29370  2.67714  2.69371  4.24705  2.90362  2.73755  3.18162  2.89764
2.37902  2.77535  2.98476  4.58493  3.61519
          0.44031  1.05639  4.77324  1.00816  0.45396  0.67925  0.70724
    484   1.77308  4.43749  3.11223  2.74647  4.16267  3.16077  3.92865
3.52010  2.80622  3.21324  4.05374  2.20606  3.77827  3.13818  3.20090
2.55053  2.07262  3.11563  5.48060  4.20164    539 a - - -
          2.68618  4.42225  2.77519  2.73123  3.46354  2.40513  3.72494
3.29354  2.67741  2.69355  4.24690  2.90347  2.73739  3.18146  2.89801
2.37887  2.77519  2.98518  4.58477  3.61503
          0.02620  4.05089  4.77324  0.61958  0.77255  0.38139  1.14858
    485   2.64390  4.54022  3.28935  2.78811  3.65671  3.49576  3.81737
3.17531  2.72032  2.84900  3.73086  2.32030  3.94307  3.08935  3.09355
2.78335  1.88036  2.90706  2.98553  3.73931    540 t - - -
          2.68618  4.42225  2.77519  2.73123  3.46354  2.40513  3.72494
3.29354  2.67741  2.69355  4.24690  2.90347  2.73739  3.18146  2.89801
2.37887  2.77519  2.98518  4.58477  3.61503
          0.02187  4.22952  4.95186  0.61958  0.77255  0.48576  0.95510
    486   2.71043  4.73663  3.01809  2.03285  3.82743  3.49514  3.75906
3.32861  2.62239  2.98225  3.85254  3.08801  3.92991  2.97975  3.03751
2.78813  2.35613  3.05108  5.20015  2.15476    541 e - - -
```

```
          2.68618  4.42225  2.77519  2.73123  3.46354  2.40513  3.72494
3.29354  2.67741  2.69355  4.24690  2.90347  2.73739  3.18146  2.89801
2.37887  2.77519  2.98518  4.58477  3.61503
          0.02187  4.22952  4.95186  0.61958  0.77255  0.48576  0.95510
    487   2.56911  4.44862  3.38286  2.92358  3.87840  3.40112  3.98132
3.07261  2.89939  2.90974  3.80975  2.42823  3.93578  3.23993  3.26790
2.74102  1.73786  2.07855  5.28849  4.02901    542 t - - -
          2.68618  4.42225  2.77519  2.73123  3.46354  2.40513  3.72494
3.29354  2.67741  2.69355  4.24690  2.90347  2.73739  3.18146  2.89801
2.37887  2.77519  2.98518  4.58477  3.61503
          0.02187  4.22952  4.95186  0.61958  0.77255  0.48576  0.95510
    488   2.57270  4.66446  3.03606  2.66610  4.28835  3.29019  3.90193
3.70578  2.74136  3.33269  4.15677  2.18153  2.56835  3.08314  3.16322
2.65711  1.70159  3.30077  5.56331  4.25220    543 t - - -
          2.68618  4.42225  2.77519  2.73123  3.46354  2.40513  3.72494
3.29354  2.67741  2.69355  4.24690  2.90347  2.73739  3.18146  2.89801
2.37887  2.77519  2.98518  4.58477  3.61503
          0.02187  4.22952  4.95186  0.61958  0.77255  0.48576  0.95510
    489   2.97029  5.20396  1.69190  2.47334  4.84976  1.15317  4.07630
4.38911  3.12132  3.94558  4.81082  3.01332  4.00120  3.27528  3.66309
2.95439  3.33458  3.91954  6.03066  4.66252    544 g - - -
          2.68618  4.42225  2.77519  2.73123  3.46354  2.40513  3.72494
3.29354  2.67741  2.69355  4.24690  2.90347  2.73739  3.18146  2.89801
2.37887  2.77519  2.98518  4.58477  3.61503
          0.02187  4.22952  4.95186  0.61958  0.77255  0.48576  0.95510
    490   3.93742  5.11290  4.84439  4.56739  2.01624  4.58966  3.56201
3.71186  4.32740  3.00828  4.26988  4.23616  4.89629  4.30874  4.33417
3.98341  4.15222  3.61334  1.59341  0.99292    545 y - - -
          2.68618  4.42225  2.77519  2.73123  3.46354  2.40513  3.72494
3.29354  2.67741  2.69355  4.24690  2.90347  2.73739  3.18146  2.89801
2.37887  2.77519  2.98518  4.58477  3.61503
          0.02187  4.22952  4.95186  0.61958  0.77255  0.48576  0.95510
    491   3.01015  4.38356  4.65538  4.12951  3.64986  4.26958  4.80224
1.28789  4.02358  2.32174  3.49911  4.33689  4.63041  4.28387  4.22769
3.62403  2.36094  1.38138  5.43242  4.22955    546 i - - -
          2.68618  4.42225  2.77519  2.73123  3.46354  2.40513  3.72494
3.29354  2.67741  2.69355  4.24690  2.90347  2.73739  3.18146  2.89801
2.37887  2.77519  2.98518  4.58477  3.61503
          0.02187  4.22952  4.95186  0.61958  0.77255  0.48576  0.95510
    492   3.17177  4.89616  3.91722  3.86891  4.97511  0.34319  4.95187
4.67693  4.09782  4.27988  5.26921  4.08332  4.29707  4.39439  4.28013
3.35620  3.68030  4.13588  5.96048  5.09425    547 G - - -
          2.68618  4.42225  2.77519  2.73123  3.46354  2.40513  3.72494
3.29354  2.67741  2.69355  4.24690  2.90347  2.73739  3.18146  2.89801
2.37887  2.77519  2.98518  4.58477  3.61503
          0.02187  4.22952  4.95186  0.61958  0.77255  0.48576  0.95510
    493   2.95838  5.23966  2.89838  2.55623  4.63803  3.52340  3.76082
4.09135  1.39600  3.58229  4.41810  1.81547  4.00129  2.90312  2.62117
2.93194  3.19665  3.71486  5.66499  4.35555    548 k - - -
          2.68618  4.42225  2.77519  2.73123  3.46354  2.40513  3.72494
3.29354  2.67741  2.69355  4.24690  2.90347  2.73739  3.18146  2.89801
2.37887  2.77519  2.98518  4.58477  3.61503
          0.02187  4.22952  4.95186  0.61958  0.77255  0.48576  0.95510
```

```
    494   2.70214  5.01813  2.16949  2.41473  4.36443  3.41035  3.69728
3.80055  2.45843  3.35763  4.15568  2.94442  3.86237  2.83137  2.24341
2.69820  1.99551  3.42405  5.54563  4.18005    549 t - - -
          2.68618  4.42225  2.77519  2.73123  3.46354  2.40513  3.72494
3.29354  2.67741  2.69355  4.24690  2.90347  2.73739  3.18146  2.89801
2.37887  2.77519  2.98518  4.58477  3.61503
          0.31255  4.22952  1.37097  0.61958  0.77255  0.48576  0.95510
    495   1.41712  4.16185  3.33448  3.10433  4.28404  1.72119  4.21354
3.63702  3.21109  3.38009  4.23462  3.27592  3.67972  3.49590  3.51669
2.40659  2.71856  3.11936  5.62600  4.42240    550 a - - -
          2.68618  4.42225  2.77519  2.73123  3.46354  2.40513  3.72494
3.29354  2.67741  2.69355  4.24690  2.90347  2.73739  3.18146  2.89801
2.37887  2.77519  2.98518  4.58477  3.61503
          0.02914  3.94610  4.66845  0.61958  0.77255  0.76495  0.62616
    496   2.48775  4.57038  2.75158  2.54503  4.26362  3.10719  3.87241
3.78340  2.75391  3.40648  4.24802  1.86561  3.75352  3.07999  3.15913
1.75269  2.87839  3.32910  5.55690  4.20676    551 s - - -
          2.68618  4.42225  2.77519  2.73123  3.46354  2.40513  3.72494
3.29354  2.67741  2.69355  4.24690  2.90347  2.73739  3.18146  2.89801
2.37887  2.77519  2.98518  4.58477  3.61503
          0.02914  3.94610  4.66845  0.61958  0.77255  0.76495  0.62616
    497   1.64583  4.46234  2.89632  2.65920  4.19937  3.10124  3.92516
3.58159  2.81326  3.29018  4.15481  1.92813  3.75418  3.14581  3.19744
2.55369  2.84181  3.16547  5.52075  4.21190    552 a - - -
          2.68618  4.42225  2.77519  2.73123  3.46354  2.40513  3.72494
3.29354  2.67741  2.69355  4.24690  2.90347  2.73739  3.18146  2.89801
2.37887  2.77519  2.98518  4.58477  3.61503
          0.02914  3.94610  4.66845  0.61958  0.77255  0.34396  1.23429
    498   2.58829  4.54223  3.23194  2.71321  3.83919  3.44026  3.80840
3.21340  2.68936  2.89133  2.55252  2.48202  3.89394  3.03199  3.09605
2.23364  2.22735  2.92652  5.20351  3.92731    553 t - - -
          2.68618  4.42225  2.77519  2.73123  3.46354  2.40513  3.72494
3.29354  2.67741  2.69355  4.24690  2.90347  2.73739  3.18146  2.89801
2.37887  2.77519  2.98518  4.58477  3.61503
          0.02187  4.22952  4.95186  0.61958  0.77255  0.48576  0.95510
    499   2.97387  5.21750  1.64973  2.46263  4.85465  1.18384  4.06943
4.39423  3.11441  3.94810  4.81271  3.00488  3.99986  3.26697  3.65949
2.95432  3.33588  3.92514  6.03486  4.66155    554 g - - -
          2.68618  4.42225  2.77519  2.73123  3.46354  2.40513  3.72494
3.29354  2.67741  2.69355  4.24690  2.90347  2.73739  3.18146  2.89801
2.37887  2.77519  2.98518  4.58477  3.61503
          0.02187  4.22952  4.95186  0.61958  0.77255  0.48576  0.95510
    500   1.62490  4.28379  3.58295  3.28733  4.53120  1.57056  4.37530
3.96819  3.36995  3.63514  4.43625  3.42707  3.80239  3.63437  3.69622
1.66840  2.82018  3.36074  5.85351  4.64697    555 g - - -
          2.68618  4.42225  2.77519  2.73123  3.46354  2.40513  3.72494
3.29354  2.67741  2.69355  4.24690  2.90347  2.73739  3.18146  2.89801
2.37887  2.77519  2.98518  4.58477  3.61503
          0.02187  4.22952  4.95186  0.61958  0.77255  0.48576  0.95510
    501   2.08297  4.29732  3.61650  3.07833  2.43710  3.58643  3.94186
2.74698  3.02788  2.52889  3.45341  2.60107  4.01073  3.32035  3.35103
2.87161  2.87570  2.11009  4.92179  3.67005    556 a - - -
          2.68621  4.42228  2.77472  2.73126  3.46357  2.40516  3.72497
3.29357  2.67744  2.69358  4.24693  2.90350  2.73743  3.18149  2.89804
2.37890  2.77522  2.98521  4.58480  3.61506
```

```
          0.20483  1.72517  4.95186  0.25055  1.50674  0.48576  0.95510
    502   2.74997  4.27558  4.03620  3.47148  3.15070  3.79646  4.05499
2.59685  3.30575  1.58967  3.26956  3.72523  4.17474  3.59215  3.52393
3.09584  2.18746  2.45842  2.71229  3.45068    558 l - - -
          2.68618  4.42225  2.77519  2.73123  3.46354  2.40513  3.72494
3.29354  2.67741  2.69355  4.24690  2.90347  2.73739  3.18146  2.89801
2.37887  2.77519  2.98518  4.58477  3.61503
          0.02187  4.22952  4.95186  0.61958  0.77255  0.48576  0.95510
    503   2.73015  5.05587  2.92363  2.44390  4.37184  2.50579  2.53336
3.82659  2.35013  3.35662  4.15125  2.38307  3.87799  2.79467  2.26651
2.72062  2.96399  3.45300  5.51352  4.16094    559 r - - -
          2.68618  4.42225  2.77519  2.73123  3.46354  2.40513  3.72494
3.29354  2.67741  2.69355  4.24690  2.90347  2.73739  3.18146  2.89801
2.37887  2.77519  2.98518  4.58477  3.61503
          0.02187  4.22952  4.95186  0.61958  0.77255  0.48576  0.95510
    504   2.70383  4.69315  3.23000  2.69810  3.92243  3.52995  3.77067
3.28209  2.09132  2.91941  2.59610  3.16234  2.13357  2.97043  2.86573
2.80144  2.94586  3.01630  5.24884  3.97466    560 k - - -
          2.68618  4.42225  2.77519  2.73123  3.46354  2.40513  3.72494
3.29354  2.67741  2.69355  4.24690  2.90347  2.73739  3.18146  2.89801
2.37887  2.77519  2.98518  4.58477  3.61503
          0.02187  4.22952  4.95186  0.61958  0.77255  0.48576  0.95510
    505   3.00096  4.38107  4.62314  4.04963  3.03424  4.16340  4.35753
1.50874  3.86681  1.56339  3.11570  4.18816  4.46720  4.03777  3.97504
3.48229  3.22955  2.31558  2.58297  3.49012    561 i - - -
          2.68618  4.42225  2.77519  2.73123  3.46354  2.40513  3.72494
3.29354  2.67741  2.69355  4.24690  2.90347  2.73739  3.18146  2.89801
2.37887  2.77519  2.98518  4.58477  3.61503
          0.02187  4.22952  4.95186  0.61958  0.77255  0.48576  0.95510
    506   2.59569  4.28476  3.78054  3.28422  3.61852  3.52608  4.13537
1.65445  3.20617  2.61524  3.57637  3.56340  4.04336  3.51200  3.49742
2.11416  2.24438  2.43694  5.12304  3.90378    562 i - - -
          2.68618  4.42225  2.77519  2.73123  3.46354  2.40513  3.72494
3.29354  2.67741  2.69355  4.24690  2.90347  2.73739  3.18146  2.89801
2.37887  2.77519  2.98518  4.58477  3.61503
          0.02187  4.22952  4.95186  0.61958  0.77255  0.48576  0.95510
    507   1.90911  4.53424  3.13685  2.81504  4.41841  3.18545  4.04972
3.85588  2.93684  3.48560  4.29712  2.23950  3.83825  3.24653  3.34699
1.42333  2.89323  3.36769  5.69950  4.40696    563 s - - -
          2.68618  4.42225  2.77519  2.73123  3.46354  2.40513  3.72494
3.29354  2.67741  2.69355  4.24690  2.90347  2.73739  3.18146  2.89801
2.37887  2.77519  2.98518  4.58477  3.61503
          0.02187  4.22952  4.95186  0.61958  0.77255  0.48576  0.95510
    508   2.85473  5.33458  1.61299  2.27974  4.65684  3.36678  3.76176
4.15084  2.62881  3.65407  4.45362  2.84645  2.61853  2.08486  3.15108
2.79049  3.11701  3.73305  5.81046  4.37079    564 d - - -
          2.68618  4.42225  2.77519  2.73123  3.46354  2.40513  3.72494
3.29354  2.67741  2.69355  4.24690  2.90347  2.73739  3.18146  2.89801
2.37887  2.77519  2.98518  4.58477  3.61503
          0.02187  4.22952  4.95186  0.61958  0.77255  0.48576  0.95510
    509   2.67382  4.62505  3.24259  2.68673  3.74537  3.54500  3.74143
3.18550  2.06293  2.34011  3.71338  3.15553  2.71841  2.96746  2.93982
2.78463  2.90299  2.92598  5.11447  2.82110    565 k - - -
```

```
         2.68618  4.42225  2.77519  2.73123  3.46354  2.40513  3.72494
3.29354  2.67741  2.69355  4.24690  2.90347  2.73739  3.18146  2.89801
2.37887  2.77519  2.98518  4.58477  3.61503
         0.02187  4.22952  4.95186  0.61958  0.77255  0.48576  0.95510
    510  2.60470  4.76419  2.88767  2.58495  4.45015  3.26809  3.90219
3.91601  2.75125  3.49884  4.30530  1.81374  2.32434  3.06998  3.19765
1.95797  2.95799  3.46237  5.68633  4.34274    566 n - - -
         2.68618  4.42225  2.77519  2.73123  3.46354  2.40513  3.72494
3.29354  2.67741  2.69355  4.24690  2.90347  2.73739  3.18146  2.89801
2.37887  2.77519  2.98518  4.58477  3.61503
         0.02187  4.22952  4.95186  0.61958  0.77255  0.48576  0.95510
    511  1.35814  4.58432  3.06184  2.82365  4.40878  3.21647  4.09529
3.81215  2.99484  3.49956  4.34816  1.71151  3.88379  3.31170  3.38606
2.66925  2.96913  3.36021  5.71638  4.41269    567 a - - -
         2.68618  4.42225  2.77519  2.73123  3.46354  2.40513  3.72494
3.29354  2.67741  2.69355  4.24690  2.90347  2.73739  3.18146  2.89801
2.37887  2.77519  2.98518  4.58477  3.61503
         0.02187  4.22952  4.95186  0.61958  0.77255  0.48576  0.95510
    512  2.81611  4.44140  3.92540  3.49222  3.61891  3.74386  4.34473
1.40996  3.38695  2.43238  3.57274  3.78014  1.96621  3.72808  3.65299
3.13739  3.12978  2.32492  5.25207  4.01829    568 i - - -
         2.68618  4.42225  2.77519  2.73123  3.46354  2.40513  3.72494
3.29354  2.67741  2.69355  4.24690  2.90347  2.73739  3.18146  2.89801
2.37887  2.77519  2.98518  4.58477  3.61503
         0.02187  4.22952  4.95186  0.61958  0.77255  0.48576  0.95510
    513  2.99166  4.38519  4.52503  3.96649  3.03778  4.14526  4.31042
1.49438  3.80779  1.64718  3.19801  4.13120  4.45937  3.99917  3.94813
3.46100  3.22262  2.25060  4.76558  2.30283    569 i - - -
         2.68618  4.42225  2.77519  2.73123  3.46354  2.40513  3.72494
3.29354  2.67741  2.69355  4.24690  2.90347  2.73739  3.18146  2.89801
2.37887  2.77519  2.98518  4.58477  3.61503
         0.02187  4.22952  4.95186  0.61958  0.77255  0.48576  0.95510
    514  2.58660  4.60728  3.08234  2.81240  4.31873  3.26225  4.04814
3.71097  2.91015  3.40494  4.26535  2.04545  3.90117  3.26517  3.28639
2.70024  1.31800  3.30074  5.63270  4.32997    570 t - - -
         2.68618  4.42225  2.77519  2.73123  3.46354  2.40513  3.72494
3.29354  2.67741  2.69355  4.24690  2.90347  2.73739  3.18146  2.89801
2.37887  2.77519  2.98518  4.58477  3.61503
         0.02187  4.22952  4.95186  0.61958  0.77255  0.48576  0.95510
    515  2.72846  4.89473  3.13338  2.56482  4.13468  3.52306  3.67430
3.56834  1.99418  3.15083  3.97746  3.05980  3.91126  2.82838  2.23681
2.19294  2.95026  3.25077  5.34685  2.66373    571 k - - -
         2.68618  4.42225  2.77519  2.73123  3.46354  2.40513  3.72494
3.29354  2.67741  2.69355  4.24690  2.90347  2.73739  3.18146  2.89801
2.37887  2.77519  2.98518  4.58477  3.61503
         0.02187  4.22952  4.95186  0.61958  0.77255  0.48576  0.95510
    516  1.39889  4.31070  4.11720  3.71346  3.88662  3.50337  4.54602
2.47251  3.63692  2.71144  3.79397  3.83147  4.14438  3.92847  3.88201
2.92974  3.02095  1.42520  5.50459  4.29469    572 a - - -
         2.68618  4.42225  2.77519  2.73123  3.46354  2.40513  3.72494
3.29354  2.67741  2.69355  4.24690  2.90347  2.73739  3.18146  2.89801
2.37887  2.77519  2.98518  4.58477  3.61503
         0.02187  4.22952  4.95186  0.61958  0.77255  0.48576  0.95510
```

```
    517   2.68110  4.84674  3.02598  2.51925  4.11508  3.46795  3.69646
3.52660  2.07293  2.39295  3.95690  2.44178  3.88321  2.85480  2.85454
2.14441  2.91918  3.20567  5.36375  4.04337    573 k - - -
          2.68621  4.42228  2.77522  2.73126  3.46357  2.40516  3.72368
3.29357  2.67744  2.69358  4.24693  2.90350  2.73743  3.18149  2.89804
2.37890  2.77522  2.98521  4.58480  3.61506
          0.20483  1.72517  4.95186  0.25055  1.50674  0.48576  0.95510
    518   2.11685  4.25244  3.88420  3.35084  3.52735  2.45285  4.14511
2.03972  3.27912  2.51679  3.48225  3.62931  4.09443  3.55708  3.56031
2.94923  2.92242  1.80173  5.05004  3.83843    575 v - - -
          2.68618  4.42225  2.77519  2.73123  3.46354  2.40513  3.72494
3.29354  2.67741  2.69355  4.24690  2.90347  2.73739  3.18146  2.89801
2.37887  2.77519  2.98518  4.58477  3.61503
          0.02187  4.22952  4.95186  0.61958  0.77255  0.48576  0.95510
    519   2.09348  4.52409  3.25429  2.74110  3.77738  2.64399  3.81690
2.32295  2.70858  2.82195  3.69762  3.18900  3.91859  2.43251  3.10326
2.75213  2.87498  2.85055  5.16059  3.89285    576 a - - -
          2.68618  4.42225  2.77519  2.73123  3.46354  2.40513  3.72494
3.29354  2.67741  2.69355  4.24690  2.90347  2.73739  3.18146  2.89801
2.37887  2.77519  2.98518  4.58477  3.61503
          0.02187  4.22952  4.95186  0.61958  0.77255  0.48576  0.95510
    520   2.04303  4.64855  2.40534  2.62145  4.04512  3.38157  3.81360
3.38837  2.68066  3.07949  3.92237  3.09266  3.87097  3.00242  3.12335
2.17922  2.88242  2.25033  5.36898  4.06387    577 a - - -
          2.68618  4.42225  2.77519  2.73123  3.46354  2.40513  3.72494
3.29354  2.67741  2.69355  4.24690  2.90347  2.73739  3.18146  2.89801
2.37887  2.77519  2.98518  4.58477  3.61503
          0.02187  4.22952  4.95186  0.61958  0.77255  0.48576  0.95510
    521   2.70436  5.02797  2.74884  2.40131  4.45825  2.38546  3.74143
3.91894  2.54172  3.45910  4.25122  2.16232  3.86061  2.27769  3.01368
2.17067  2.97960  3.51027  5.63630  4.25644    578 n - - -
          2.68618  4.42225  2.77519  2.73123  3.46354  2.40513  3.72494
3.29354  2.67741  2.69355  4.24690  2.90347  2.73739  3.18146  2.89801
2.37887  2.77519  2.98518  4.58477  3.61503
          0.02187  4.22952  4.95186  0.61958  0.77255  0.48576  0.95510
    522   2.82397  4.58068  3.61001  3.01988  3.31475  3.72346  3.80512
3.04761  2.66416  2.01306  3.62959  3.40490  4.09425  3.17233  1.91328
2.99986  3.04516  2.85054  4.81303  2.38774    579 r - - -
          2.68618  4.42225  2.77519  2.73123  3.46354  2.40513  3.72494
3.29354  2.67741  2.69355  4.24690  2.90347  2.73739  3.18146  2.89801
2.37887  2.77519  2.98518  4.58477  3.61503
          0.02187  4.22952  4.95186  0.61958  0.77255  0.48576  0.95510
    523   1.74669  4.34557  3.52407  3.11483  4.22985  3.16366  4.17585
3.57319  3.11131  3.29040  4.13707  3.36617  2.19425  3.42467  3.46247
2.57037  1.63763  3.13804  5.58518  4.35285    580 t - - -
          2.68618  4.42225  2.77519  2.73123  3.46354  2.40513  3.72494
3.29354  2.67741  2.69355  4.24690  2.90347  2.73739  3.18146  2.89801
2.37887  2.77519  2.98518  4.58477  3.61503
          0.02187  4.22952  4.95186  0.61958  0.77255  0.48576  0.95510
    524   3.29869  4.97961  3.94636  3.84728  4.78758  3.64774  4.88149
4.44313  3.93886  4.02289  5.09758  4.11130  0.36966  4.31567  4.13026
3.48053  3.77053  4.04412  5.85897  4.91765    581 P - - -
          2.68618  4.42225  2.77519  2.73123  3.46354  2.40513  3.72494
3.29354  2.67741  2.69355  4.24690  2.90347  2.73739  3.18146  2.89801
2.37887  2.77519  2.98518  4.58477  3.61503
```

```
         0.02187  4.22952  4.95186  0.61958  0.77255  0.48576  0.95510
    525  2.64170  4.61915  3.14815  2.71934  2.62210  3.43305  3.82412
3.35207  2.76485  3.00549  3.88064  1.89832  3.92424  3.09046  3.17677
2.05803  2.93282  3.05189  5.12379  3.72269    582 n - - -
         2.68618  4.42225  2.77519  2.73123  3.46354  2.40513  3.72494
3.29354  2.67741  2.69355  4.24690  2.90347  2.73739  3.18146  2.89801
2.37887  2.77519  2.98518  4.58477  3.61503
         0.02187  4.22952  4.95186  0.61958  0.77255  0.48576  0.95510
    526  2.66177  4.32812  3.68650  3.17087  3.25476  3.61014  3.93128
2.81583  3.09398  2.56887  3.50798  3.49178  4.05182  3.39423  3.39255
2.14944  2.93159  2.02139  4.77886  2.06215    583 v - - -
         2.68618  4.42225  2.77519  2.73123  3.46354  2.40513  3.72494
3.29354  2.67741  2.69355  4.24690  2.90347  2.73739  3.18146  2.89801
2.37887  2.77519  2.98518  4.58477  3.61503
         0.02187  4.22952  4.95186  0.61958  0.77255  0.48576  0.95510
    527  3.12258  4.43354  4.91221  4.36672  2.39453  4.43708  4.81860
1.61796  4.24535  2.01228  3.28653  4.51669  4.71904  4.41026  4.36452
3.78616  3.36472  1.19073  5.24007  4.02100    584 v - - -
         2.68627  4.42234  2.77529  2.73132  3.46363  2.40522  3.72504
3.29363  2.67750  2.69318  4.24699  2.90299  2.73749  3.18080  2.89810
2.37896  2.77529  2.98528  4.58486  3.61512
         0.20483  1.72517  4.95186  0.96361  0.48048  0.48576  0.95510
    528  2.72347  5.10752  2.19303  2.34440  4.45579  2.43285  3.70723
3.91698  2.52393  3.45174  4.23934  2.89118  3.85460  2.28487  3.02075
2.69906  2.22110  3.51848  5.63126  4.23419    588 d - - -
         2.68618  4.42225  2.77519  2.73123  3.46354  2.40513  3.72494
3.29354  2.67741  2.69355  4.24690  2.90347  2.73739  3.18146  2.89801
2.37887  2.77519  2.98518  4.58477  3.61503
         0.02187  4.22952  4.95186  0.61958  0.77255  0.48576  0.95510
    529  3.24549  4.57522  4.92327  4.36284  2.25203  4.48119  4.73054
2.23484  4.22818  1.10722  3.03741  4.53237  4.71709  4.32058  4.31766
3.82157  3.47295  1.72379  5.05538  3.84549    589 l - - -
         2.68618  4.42225  2.77519  2.73123  3.46354  2.40513  3.72494
3.29354  2.67741  2.69355  4.24690  2.90347  2.73739  3.18146  2.89801
2.37887  2.77519  2.98518  4.58477  3.61503
         0.02187  4.22952  4.95186  0.61958  0.77255  0.48576  0.95510
    530  2.77159  4.83206  3.28071  2.67869  4.08655  3.58079  3.70598
3.45467  1.95434  2.27773  3.91860  3.14581  3.95997  2.87502  2.08599
2.83413  2.38279  3.16795  5.30705  4.04315    590 k - - -
         2.68618  4.42225  2.77519  2.73123  3.46354  2.40513  3.72494
3.29354  2.67741  2.69355  4.24690  2.90347  2.73739  3.18146  2.89801
2.37887  2.77519  2.98518  4.58477  3.61503
         0.02187  4.22952  4.95186  0.61958  0.77255  0.48576  0.95510
    531  2.86689  4.78448  2.28863  2.83635  2.26718  3.69380  2.58963
3.38148  2.84502  2.95227  3.88783  3.27261  4.08119  3.14825  3.25427
2.94249  3.09788  3.13096  4.49086  2.14339    591 y - - -
         2.68618  4.42225  2.77519  2.73123  3.46354  2.40513  3.72494
3.29354  2.67741  2.69355  4.24690  2.90347  2.73739  3.18146  2.89801
2.37887  2.77519  2.98518  4.58477  3.61503
         0.02187  4.22952  4.95186  0.61958  0.77255  0.48576  0.95510
    532  2.75120  5.17589  2.14576  1.94350  4.50746  3.37528  3.70970
3.97491  2.54112  3.50037  4.28614  2.86903  3.85887  2.83995  3.05148
2.10968  2.22013  3.57074  5.67415  4.26236    592 e - - -
```

```
        2.68618  4.42225  2.77519  2.73123  3.46354  2.40513  3.72494
3.29354  2.67741  2.69355  4.24690  2.90347  2.73739  3.18146  2.89801
2.37887  2.77519  2.98518  4.58477  3.61503
        0.02187  4.22952  4.95186  0.61958  0.77255  0.48576  0.95510
    533  2.64573  4.51601  3.31044  2.83916  3.79882  3.50269  3.91116
2.18366  2.81089  2.81383  3.73228  1.96135  3.97101  3.16296  3.18925
2.80929  2.30370  2.71751  5.21939  3.94752    593 n - - -
        2.68618  4.42225  2.77519  2.73123  3.46354  2.40513  3.72494
3.29354  2.67741  2.69355  4.24690  2.90347  2.73739  3.18146  2.89801
2.37887  2.77519  2.98518  4.58477  3.61503
        0.02187  4.22952  4.95186  0.61958  0.77255  0.48576  0.95510
    534  3.17177  4.89616  3.91722  3.86891  4.97511  0.34319  4.95187
4.67693  4.09782  4.27988  5.26921  4.08332  4.29707  4.39439  4.28013
3.35620  3.68030  4.13588  5.96048  5.09425    594 G - - -
        2.68618  4.42225  2.77519  2.73123  3.46354  2.40513  3.72494
3.29354  2.67741  2.69355  4.24690  2.90347  2.73739  3.18146  2.89801
2.37887  2.77519  2.98518  4.58477  3.61503
        0.02187  4.22952  4.95186  0.61958  0.77255  0.48576  0.95510
    535  2.40649  4.41099  3.30813  3.02334  4.51399  1.55301  4.21794
3.96951  3.15553  3.60361  4.40778  3.30254  2.11478  3.44178  3.53111
1.88340  2.86916  3.40427  5.80696  4.55484    595 g - - -
        2.68618  4.42225  2.77519  2.73123  3.46354  2.40513  3.72494
3.29354  2.67741  2.69355  4.24690  2.90347  2.73739  3.18146  2.89801
2.37887  2.77519  2.98518  4.58477  3.61503
        0.02187  4.22952  4.95186  0.61958  0.77255  0.48576  0.95510
    536  2.11675  4.47765  3.29571  2.79771  3.81040  3.42618  3.86993
2.28214  2.78810  2.86486  3.73651  2.47078  3.90746  3.11634  3.18238
2.11721  2.86397  2.85813  5.19884  3.93423    596 a - - -
        2.68618  4.42225  2.77519  2.73123  3.46354  2.40513  3.72494
3.29354  2.67741  2.69355  4.24690  2.90347  2.73739  3.18146  2.89801
2.37887  2.77519  2.98518  4.58477  3.61503
        0.02187  4.22952  4.95186  0.61958  0.77255  0.48576  0.95510
    537  2.80431  4.32125  3.99588  3.45089  2.07703  3.82937  3.85685
2.18650  3.35530  2.44821  3.42186  2.59622  4.19443  3.58394  3.58613
3.11313  3.03567  2.57277  4.39901  2.15962    597 f - - -
        2.68618  4.42225  2.77519  2.73123  3.46354  2.40513  3.72494
3.29354  2.67741  2.69355  4.24690  2.90347  2.73739  3.18146  2.89801
2.37887  2.77519  2.98518  4.58477  3.61503
        0.02187  4.22952  4.95186  0.61958  0.77255  0.48576  0.95510
    538  2.48594  4.41966  3.47594  3.18873  4.34821  3.19193  4.28618
3.67777  3.20010  3.40429  4.30025  3.42943  1.25295  3.55046  3.51288
2.65562  1.88905  3.24071  5.69880  4.47425    598 p - - -
        2.68623  4.42230  2.77524  2.73128  3.46358  2.40517  3.72499
3.29359  2.67745  2.69359  4.24694  2.90351  2.73744  3.18094  2.89805
2.37866  2.77524  2.98523  4.58481  3.61508
        0.56564  1.34189  1.76812  0.19269  1.74147  0.48576  0.95510
    539  2.83101  4.58659  3.48035  3.00311  1.77563  3.66166  3.79220
3.01041  1.95780  2.60511  3.64892  3.38487  4.07938  3.22261  3.04844
2.99833  3.07602  2.83049  4.69498  3.21285    600 f - - -
        2.68618  4.42225  2.77519  2.73123  3.46354  2.40513  3.72494
3.29354  2.67741  2.69355  4.24690  2.90347  2.73739  3.18146  2.89801
2.37887  2.77519  2.98518  4.58477  3.61503
        0.02620  4.05089  4.77324  0.61958  0.77255  0.38139  1.14858
```

540 3.17177 4.89616 3.91722 3.86891 4.97511 0.34319 4.95187
4.67693 4.09782 4.27988 5.26921 4.08332 4.29707 4.39439 4.28013
3.35620 3.68030 4.13588 5.96048 5.09425 601 G - - -
2.68618 4.42225 2.77519 2.73123 3.46354 2.40513 3.72494
3.29354 2.67741 2.69355 4.24690 2.90347 2.73739 3.18146 2.89801
2.37887 2.77519 2.98518 4.58477 3.61503
0.02187 4.22952 4.95186 0.61958 0.77255 0.48576 0.95510
541 2.83673 5.04048 3.25043 2.62946 4.35963 3.59423 3.66360
3.75080 1.76057 3.28028 2.65019 3.10738 3.96457 2.25778 2.05639
2.84813 3.03974 3.42257 5.43937 4.16889 602 k - - -
2.68618 4.42225 2.77519 2.73123 3.46354 2.40513 3.72494
3.29354 2.67741 2.69355 4.24690 2.90347 2.73739 3.18146 2.89801
2.37887 2.77519 2.98518 4.58477 3.61503
0.02187 4.22952 4.95186 0.61958 0.77255 0.48576 0.95510
542 2.09993 4.76754 2.31473 2.56495 3.81518 3.45918 3.77896
3.40120 2.70464 3.05266 3.92226 3.06499 3.92605 3.01029 3.15187
2.78190 2.97428 3.11066 5.21177 2.05360 603 y - - -
2.68618 4.42225 2.77519 2.73123 3.46354 2.40513 3.72494
3.29354 2.67741 2.69355 4.24690 2.90347 2.73739 3.18146 2.89801
2.37887 2.77519 2.98518 4.58477 3.61503
0.15591 4.22952 2.04170 0.61958 0.77255 0.48576 0.95510
543 2.59894 4.32464 3.75057 3.34766 3.73360 3.45438 4.25860
2.61015 3.27398 2.62962 3.69262 3.59791 2.25076 3.61043 3.55532
2.86809 2.96699 1.35949 5.29246 4.05745 604 v - - -
2.68618 4.42225 2.77519 2.73123 3.46354 2.40513 3.72494
3.29354 2.67741 2.69355 4.24690 2.90347 2.73739 3.18146 2.89801
2.37887 2.77519 2.98518 4.58477 3.61503
0.02496 4.09857 4.82092 0.61958 0.77255 0.63433 0.75564
544 1.87377 4.24964 3.64432 3.16417 3.66101 3.38509 4.07158
1.96150 3.10728 2.66833 3.61391 3.44749 3.93834 3.41482 3.41875
2.09993 2.84007 2.50189 5.14327 3.91776 605 a - - -
2.68618 4.42225 2.77519 2.73123 3.46354 2.40513 3.72494
3.29354 2.67741 2.69355 4.24690 2.90347 2.73739 3.18146 2.89801
2.37887 2.77519 2.98518 4.58477 3.61503
0.02496 4.09857 4.82092 0.61958 0.77255 0.63433 0.75564
545 2.65703 4.76583 2.70935 2.57243 4.51202 2.08255 3.99144
4.04985 2.91144 3.64581 4.49130 1.34263 3.86050 3.19814 3.33964
2.71787 3.05206 3.56887 5.75658 4.41638 606 n - - -
2.68618 4.42225 2.77519 2.73123 3.46354 2.40513 3.72494
3.29354 2.67741 2.69355 4.24690 2.90347 2.73739 3.18146 2.89801
2.37887 2.77519 2.98518 4.58477 3.61503
0.02496 4.09857 4.82092 0.61958 0.77255 0.40295 1.10367
546 2.70603 5.11816 2.28423 2.34219 4.41790 3.40559 2.73836
3.88527 2.43536 3.40856 4.18718 2.88965 3.84317 2.17798 2.91856
2.67741 2.29892 3.49196 5.57507 4.18053 607 q - - -
2.68618 4.42225 2.77519 2.73123 3.46354 2.40513 3.72494
3.29354 2.67741 2.69355 4.24690 2.90347 2.73739 3.18146 2.89801
2.37887 2.77519 2.98518 4.58477 3.61503
0.02187 4.22952 4.95186 0.61958 0.77255 0.48576 0.95510
547 2.20045 4.98212 2.84337 2.42023 4.35848 3.39231 3.70379
3.80395 2.47784 3.35690 4.15054 2.26216 2.76745 2.17212 2.93752
2.68003 2.93730 3.41872 5.54601 4.18090 608 q - - -
2.68618 4.42225 2.77519 2.73123 3.46354 2.40513 3.72494
3.29354 2.67741 2.69355 4.24690 2.90347 2.73739 3.18146 2.89801
2.37887 2.77519 2.98518 4.58477 3.61503

```
         0.20483  4.22952  1.76812  0.61958  0.77255  0.48576  0.95510
    548  1.87147  4.53597  3.18276  2.70511  4.12926  3.26049  3.81425
3.52560  2.56106  3.15967  3.99513  3.11325  3.80773  3.00165  2.19186
2.02133  2.83399  3.14731  5.39779  4.12203    609 a - - -
         2.68618  4.42225  2.77519  2.73123  3.46354  2.40513  3.72494
3.29354  2.67741  2.69355  4.24690  2.90347  2.73739  3.18146  2.89801
2.37887  2.77519  2.98518  4.58477  3.61503
         0.02620  4.05089  4.77324  0.61958  0.77255  0.67925  0.70724
    549  3.18995  4.64793  4.18091  3.73702  2.40152  4.08515  3.75613
2.85849  3.53532  1.72662  3.54126  3.87524  4.44047  3.79335  3.72079
3.43695  3.42793  2.78891  4.08054  1.33288    610 y - - -
         2.68618  4.42225  2.77519  2.73123  3.46354  2.40513  3.72494
3.29354  2.67741  2.69355  4.24690  2.90347  2.73739  3.18146  2.89801
2.37887  2.77519  2.98518  4.58477  3.61503
         0.02620  4.05089  4.77324  0.61958  0.77255  0.67925  0.70724
    550  1.93419  4.78197  3.08019  2.65541  4.28413  3.38757  3.76617
3.60366  1.51732  3.24738  4.11759  3.09929  3.89513  2.93463  2.67294
2.76345  2.99100  3.26439  5.46492  4.19476    611 k - - -
         2.68618  4.42225  2.77519  2.73123  3.46354  2.40513  3.72494
3.29354  2.67741  2.69355  4.24690  2.90347  2.73739  3.18146  2.89801
2.37887  2.77519  2.98518  4.58477  3.61503
         0.02620  4.05089  4.77324  0.61958  0.77255  0.67925  0.70724
    551  2.75620  5.10292  2.69889  1.91910  4.43500  3.38380  3.65080
3.87137  1.92443  3.39807  4.20738  2.88406  2.47320  2.78664  2.76042
2.72880  2.99578  3.49707  5.55895  4.20205    612 e - - -
         2.68618  4.42225  2.77519  2.73123  3.46354  2.40513  3.72494
3.29354  2.67741  2.69355  4.24690  2.90347  2.73739  3.18146  2.89801
2.37887  2.77519  2.98518  4.58477  3.61503
         0.02620  4.05089  4.77324  0.61958  0.77255  0.67925  0.70724
    552  2.64662  4.73395  2.36288  2.50857  2.57368  3.44092  3.66801
3.34119  2.51755  2.95852  3.82711  3.01006  3.85949  2.87557  2.35095
2.70476  2.88565  3.05489  5.13574  3.75065    613 r - - -
         2.68618  4.42225  2.77519  2.73123  3.46354  2.40513  3.72494
3.29354  2.67741  2.69355  4.24690  2.90347  2.73739  3.18146  2.89801
2.37887  2.77519  2.98518  4.58477  3.61503
         0.02620  4.05089  4.77324  0.61958  0.77255  0.67925  0.70724
    553  2.64278  4.56218  3.13711  2.08085  3.78634  3.49292  3.79560
2.30397  2.65787  2.75616  3.68425  3.14264  3.92031  3.01776  3.05643
2.77657  2.29974  2.67454  5.19216  3.91245    614 e - - -
         2.68618  4.42225  2.77519  2.73123  3.46354  2.40513  3.72494
3.29354  2.67741  2.69355  4.24690  2.90347  2.73739  3.18146  2.89801
2.37887  2.77519  2.98518  4.58477  3.61503
         0.02620  4.05089  4.77324  0.61958  0.77255  0.67925  0.70724
    554  3.49373  4.84424  4.48374  4.23523  0.75941  4.13528  3.96859
2.99901  4.13862  2.32936  3.70472  4.22448  4.59221  4.25814  4.21409
3.75232  3.79615  3.00228  4.17058  2.51762    615 f - - -
         2.68618  4.42225  2.77519  2.73123  3.46354  2.40513  3.72494
3.29354  2.67741  2.69355  4.24690  2.90347  2.73739  3.18146  2.89801
2.37887  2.77519  2.98518  4.58477  3.61503
         0.02620  4.05089  4.77324  0.61958  0.77255  0.38139  1.14858
    555  2.78098  5.01529  2.96895  1.78510  4.28170  3.50515  3.69611
3.67007  1.91470  2.34250  4.08479  3.01893  3.92200  2.84043  2.74037
2.78895  3.00660  3.34805  5.46593  4.14364    616 e - - -
```

```
         2.68618  4.42225  2.77519  2.73123  3.46354  2.40513  3.72494
3.29354  2.67741  2.69355  4.24690  2.90347  2.73739  3.18146  2.89801
2.37887  2.77519  2.98518  4.58477  3.61503
         0.02187  4.22952  4.95186  0.61958  0.77255  0.48576  0.95510
    556  3.97526  5.12600  4.89912  4.65710  1.50197  4.63845  3.57131
3.63047  4.48614  2.90528  4.20888  4.27639  4.93481  4.37212  4.45884
4.02618  4.19241  3.56581  3.68430  0.77900    617 y - - -
         2.68618  4.42225  2.77519  2.73123  3.46354  2.40513  3.72494
3.29354  2.67741  2.69355  4.24690  2.90347  2.73739  3.18146  2.89801
2.37887  2.77519  2.98518  4.58477  3.61503
         0.02187  4.22952  4.95186  0.61958  0.77255  0.48576  0.95510
    557  3.29869  4.97961  3.94636  3.84728  4.78758  3.64774  4.88149
4.44313  3.93886  4.02289  5.09758  4.11130  0.36966  4.31567  4.13026
3.48053  3.77053  4.04412  5.85897  4.91765    618 P - - -
         2.68618  4.42225  2.77519  2.73123  3.46354  2.40513  3.72494
3.29354  2.67741  2.69355  4.24690  2.90347  2.73739  3.18146  2.89801
2.37887  2.77519  2.98518  4.58477  3.61503
         0.02187  4.22952  4.95186  0.61958  0.77255  0.48576  0.95510
    558  2.70662  4.79983  2.81538  2.68993  4.63356  1.41998  4.11270
4.17537  3.05115  3.76946  4.61051  1.59966  3.93287  3.32400  3.47797
2.77829  3.11993  3.66500  5.87601  4.54640    619 g - - -
         2.68618  4.42225  2.77519  2.73123  3.46354  2.40513  3.72494
3.29354  2.67741  2.69355  4.24690  2.90347  2.73739  3.18146  2.89801
2.37887  2.77519  2.98518  4.58477  3.61503
         0.20483  4.22952  1.76812  0.61958  0.77255  0.48576  0.95510
    559  2.86102  4.81370  3.16801  2.74614  3.20026  3.58959  3.66988
3.41180  2.56945  2.94830  3.91853  3.18951  4.01850  1.76877  2.89724
2.92557  3.09822  3.17011  4.71384  2.13633    620 q - - -
         2.68618  4.42225  2.77519  2.73123  3.46354  2.40513  3.72494
3.29354  2.67741  2.69355  4.24690  2.90347  2.73739  3.18146  2.89801
2.37887  2.77519  2.98518  4.58477  3.61503
         0.02620  4.05089  4.77324  0.61958  0.77255  0.67925  0.70724
    560  3.14330  5.10633  3.33062  2.92319  4.53376  3.60946  3.87022
3.97517  0.81765  3.50307  4.45830  3.33848  4.10000  3.05403  2.48165
3.17560  3.38497  3.67413  5.51760  4.35771    621 k - - -
         2.68618  4.42225  2.77519  2.73123  3.46354  2.40513  3.72494
3.29354  2.67741  2.69355  4.24690  2.90347  2.73739  3.18146  2.89801
2.37887  2.77519  2.98518  4.58477  3.61503
         0.02620  4.05089  4.77324  0.61958  0.77255  0.38139  1.14858
    561  3.48642  4.77337  5.13438  4.60359  3.22457  4.78568  5.12675
1.70370  4.42920  0.81004  3.01938  4.84551  4.95179  4.54339  4.52642
4.17372  3.71624  2.22050  5.35811  4.26911    622 l - - -
         2.68618  4.42225  2.77519  2.73123  3.46354  2.40513  3.72494
3.29354  2.67741  2.69355  4.24690  2.90347  2.73739  3.18146  2.89801
2.37887  2.77519  2.98518  4.58477  3.61503
         0.02187  4.22952  4.95186  0.61958  0.77255  0.48576  0.95510
    562  2.72342  4.97705  2.80719  2.45651  4.28105  3.39207  2.49356
3.82474  2.54985  3.38464  4.19911  2.16578  3.88621  2.91273  2.99323
1.82642  2.99692  3.44564  5.52303  4.13482    623 s - - -
         2.68618  4.42225  2.77519  2.73123  3.46354  2.40513  3.72494
3.29354  2.67741  2.69355  4.24690  2.90347  2.73739  3.18146  2.89801
2.37887  2.77519  2.98518  4.58477  3.61503
         0.02187  4.22952  4.95186  0.61958  0.77255  0.48576  0.95510
```

```
     563   2.79768  5.20788  2.06877  2.31974  4.56396  3.36445  3.75580
4.04612  2.60618  3.57034  4.36898  2.87455  3.88258  2.24768  3.11283
1.65155  3.06533  3.63605  5.73763  4.31950    624 s - - -
           2.68618  4.42225  2.77519  2.73123  3.46354  2.40513  3.72494
3.29354  2.67741  2.69355  4.24690  2.90347  2.73739  3.18146  2.89801
2.37887  2.77519  2.98518  4.58477  3.61503
           0.02187  4.22952  4.95186  0.61958  0.77255  0.48576  0.95510
     564   3.26099  4.53059  5.09218  4.56277  3.53517  4.67540  5.15784
1.53110  4.45446  1.52497  3.32415  4.75692  4.91138  4.63495  4.59461
4.04872  3.50567  1.17442  5.54045  4.39400    625 v - - -
           2.68618  4.42225  2.77519  2.73123  3.46354  2.40513  3.72494
3.29354  2.67741  2.69355  4.24690  2.90347  2.73739  3.18146  2.89801
2.37887  2.77519  2.98518  4.58477  3.61503
           0.02187  4.22952  4.95186  0.61958  0.77255  0.48576  0.95510
     565   2.67785  4.76275  3.09601  2.57039  3.94045  3.49786  2.77304
3.38945  2.48379  2.31067  3.85594  3.06595  3.89936  2.37399  2.88839
2.19818  2.91026  3.09551  5.24820  3.92596    626 s - - -
           2.68618  4.42225  2.77519  2.73123  3.46354  2.40513  3.72494
3.29354  2.67741  2.69355  4.24690  2.90347  2.73739  3.18146  2.89801
2.37887  2.77519  2.98518  4.58477  3.61503
           0.02187  4.22952  4.95186  0.61958  0.77255  0.48576  0.95510
     566   1.33440  4.86421  2.79589  1.84417  4.38855  3.35022  3.92773
3.71643  2.79342  3.40255  4.27186  3.06068  3.93051  3.11394  3.22784
2.79112  3.06838  3.35786  5.66640  4.33425    627 a - - -
           2.68618  4.42225  2.77519  2.73123  3.46354  2.40513  3.72494
3.29354  2.67741  2.69355  4.24690  2.90347  2.73739  3.18146  2.89801
2.37887  2.77519  2.98518  4.58477  3.61503
           0.02187  4.22952  4.95186  0.61958  0.77255  0.48576  0.95510
     567   3.99318  5.12490  4.94556  4.71393  1.16092  4.65842  3.56685
3.63325  4.53743  2.90247  4.20649  4.29328  4.94815  4.39563  4.48842
4.04482  4.20732  3.57058  3.67467  0.99031    628 y - - -
           2.68618  4.42225  2.77519  2.73123  3.46354  2.40513  3.72494
3.29354  2.67741  2.69355  4.24690  2.90347  2.73739  3.18146  2.89801
2.37887  2.77519  2.98518  4.58477  3.61503
           0.02187  4.22952  4.95186  0.61958  0.77255  0.48576  0.95510
     568   2.89411  5.31325  2.44930  1.72617  4.70283  1.79580  3.82835
4.18976  2.73135  3.71141  4.53162  2.15330  3.92091  2.98297  3.25022
2.83716  3.18015  3.77009  5.87823  4.44333    629 e - - -
           2.68618  4.42225  2.77519  2.73123  3.46354  2.40513  3.72494
3.29354  2.67741  2.69355  4.24690  2.90347  2.73739  3.18146  2.89801
2.37887  2.77519  2.98518  4.58477  3.61503
           0.02187  4.22952  4.95186  0.61958  0.77255  0.48576  0.95510
     569   2.75975  4.75293  3.29568  2.73727  3.99115  3.57624  3.77310
3.27717  1.65366  2.23193  3.85730  3.19418  3.98100  2.96169  2.75319
2.85251  2.35527  3.02113  5.29159  4.02653    630 k - - -
           2.68618  4.42225  2.77519  2.73123  3.46354  2.40513  3.72494
3.29354  2.67741  2.69355  4.24690  2.90347  2.73739  3.18146  2.89801
2.37887  2.77519  2.98518  4.58477  3.61503
           0.02187  4.22952  4.95186  0.61958  0.77255  0.48576  0.95510
     570   2.66722  4.43969  3.69390  3.28959  2.06832  3.48162  4.02180
3.16231  3.26683  2.81659  3.82052  3.54178  4.05823  3.55276  3.56588
1.31613  3.02779  2.91095  4.80005  3.29820    631 s - - -
           2.68618  4.42225  2.77519  2.73123  3.46354  2.40513  3.72494
3.29354  2.67741  2.69355  4.24690  2.90347  2.73739  3.18146  2.89801
2.37887  2.77519  2.98518  4.58477  3.61503
```

```
          0.02187  4.22952  4.95186  0.61958  0.77255  0.48576  0.95510
    571   1.98466  4.68278  3.17175  2.68840  4.16085  3.39066  3.82542
3.50970  1.99383  3.17109  4.01282  3.13606  3.89166  3.00353  2.95294
2.70482  1.88866  3.16713  5.43097  4.14353    632 t - - -
          2.68618  4.42225  2.77519  2.73123  3.46354  2.40513  3.72494
3.29354  2.67741  2.69355  4.24690  2.90347  2.73739  3.18146  2.89801
2.37887  2.77519  2.98518  4.58477  3.61503
          0.02187  4.22952  4.95186  0.61958  0.77255  0.48576  0.95510
    572   2.10535  4.22089  3.80449  3.24397  3.34967  3.66861  3.99580
2.04605  3.15883  2.40467  3.34610  3.55567  4.06403  3.43630  3.43373
2.95156  2.33836  2.38586  4.85472  2.69921    633 i - - -
          2.68618  4.42225  2.77519  2.73123  3.46354  2.40513  3.72494
3.29354  2.67741  2.69355  4.24690  2.90347  2.73739  3.18146  2.89801
2.37887  2.77519  2.98518  4.58477  3.61503
          0.02187  4.22952  4.95186  0.61958  0.77255  0.48576  0.95510
    573   3.29869  4.97961  3.94636  3.84728  4.78758  3.64774  4.88149
4.44313  3.93886  4.02289  5.09758  4.11130  0.36966  4.31567  4.13026
3.48053  3.77053  4.04412  5.85897  4.91765    634 P - - -
          2.68618  4.42225  2.77519  2.73123  3.46354  2.40513  3.72494
3.29354  2.67741  2.69355  4.24690  2.90347  2.73739  3.18146  2.89801
2.37887  2.77519  2.98518  4.58477  3.61503
          0.02187  4.22952  4.95186  0.61958  0.77255  0.48576  0.95510
    574   3.01386  5.33720  1.22179  2.38739  4.90572  1.62575  4.02850
4.45114  3.08080  3.98463  4.84816  2.94619  3.99535  3.21781  3.65220
2.96470  3.36007  3.98475  6.07885  4.66675    635 d - - -
          2.68618  4.42225  2.77519  2.73123  3.46354  2.40513  3.72494
3.29354  2.67741  2.69355  4.24690  2.90347  2.73739  3.18146  2.89801
2.37887  2.77519  2.98518  4.58477  3.61503
          0.19097  4.22952  1.83706  0.61958  0.77255  0.48576  0.95510
    575   2.94761  4.50643  3.90564  3.39246  1.96064  3.88939  2.22278
2.88845  3.26758  1.86642  3.49677  3.64530  4.24692  3.53448  3.52372
3.18113  3.17774  2.75663  4.20348  2.60741    636 l - - -
          2.68618  4.42225  2.77519  2.73123  3.46354  2.40513  3.72494
3.29354  2.67741  2.69355  4.24690  2.90347  2.73739  3.18146  2.89801
2.37887  2.77519  2.98518  4.58477  3.61503
          0.02584  4.06439  4.78674  0.61958  0.77255  0.66695  0.72005
    576   2.77282  5.22737  2.06853  2.27559  4.54921  3.35667  3.64900
4.01977  1.84227  3.51232  4.30478  2.82824  3.83623  2.17746  2.84553
2.71856  3.01414  3.61451  5.65170  4.25271    637 k - - -
          2.68618  4.42225  2.77519  2.73123  3.46354  2.40513  3.72494
3.29354  2.67741  2.69355  4.24690  2.90347  2.73739  3.18146  2.89801
2.37887  2.77519  2.98518  4.58477  3.61503
          0.02584  4.06439  4.78674  0.61958  0.77255  0.38715  1.13628
    577   1.58961  4.31242  3.59475  3.41152  4.59696  1.05922  4.51101
3.98670  3.55106  3.71471  4.55263  3.51131  3.85215  3.80883  3.83069
2.56377  2.89677  3.39138  5.92373  4.74813    638 g - - -
          2.68618  4.42225  2.77519  2.73123  3.46354  2.40513  3.72494
3.29354  2.67741  2.69355  4.24690  2.90347  2.73739  3.18146  2.89801
2.37887  2.77519  2.98518  4.58477  3.61503
          0.02187  4.22952  4.95186  0.61958  0.77255  0.48576  0.95510
    578   2.11949  4.37730  4.62739  4.10713  3.66119  4.22483  4.78980
1.34121  4.00949  2.34221  3.51264  4.30972  4.60503  4.26724  4.21775
3.58330  3.26143  1.38767  5.43517  4.23102    639 i - - -
```

2.68618 4.42225 2.77519 2.73123 3.46354 2.40513 3.72494
3.29354 2.67741 2.69355 4.24690 2.90347 2.73739 3.18146 2.89801
2.37887 2.77519 2.98518 4.58477 3.61503
0.02187 4.22952 4.95186 0.61958 0.77255 0.48576 0.95510
579 3.09053 5.63956 1.08924 2.22768 4.95668 3.32831 3.93007
4.53789 2.99284 4.03340 4.90029 1.86750 3.97744 3.10749 3.60759
2.97400 3.40075 4.08848 6.15336 4.62793 640 d - - -
2.68618 4.42225 2.77519 2.73123 3.46354 2.40513 3.72494
3.29354 2.67741 2.69355 4.24690 2.90347 2.73739 3.18146 2.89801
2.37887 2.77519 2.98518 4.58477 3.61503
0.02187 4.22952 4.95186 0.61958 0.77255 0.48576 0.95510
580 1.51483 4.26044 3.76291 3.32157 3.96606 3.24022 4.24465
3.13396 3.27943 3.00762 3.90977 3.50525 3.90100 3.56906 3.58336
1.88324 2.84321 1.91375 5.40759 4.19546 641 a - - -
2.68618 4.42225 2.77519 2.73123 3.46354 2.40513 3.72494
3.29354 2.67741 2.69355 4.24690 2.90347 2.73739 3.18146 2.89801
2.37887 2.77519 2.98518 4.58477 3.61503
0.02187 4.22952 4.95186 0.61958 0.77255 0.48576 0.95510
581 1.86661 4.33810 4.26791 3.86901 3.85870 3.66054 4.66986
2.26159 3.78382 2.61952 3.74559 3.98097 4.27173 4.07563 4.01083
3.08951 3.10307 1.07125 5.54190 4.32947 642 v - - -
2.68618 4.42225 2.77519 2.73123 3.46354 2.40513 3.72494
3.29354 2.67741 2.69355 4.24690 2.90347 2.73739 3.18146 2.89801
2.37887 2.77519 2.98518 4.58477 3.61503
0.02187 4.22952 4.95186 0.61958 0.77255 0.48576 0.95510
582 2.66157 4.26456 3.90916 3.37973 3.46131 2.40841 4.14381
2.52375 3.28336 2.41159 2.38116 3.65481 4.11509 3.57034 3.54761
2.98518 2.94432 1.64162 5.00963 3.80018 643 v - - -
2.68618 4.42225 2.77519 2.73123 3.46354 2.40513 3.72494
3.29354 2.67741 2.69355 4.24690 2.90347 2.73739 3.18146 2.89801
2.37887 2.77519 2.98518 4.58477 3.61503
0.02187 4.22952 4.95186 0.61958 0.77255 0.48576 0.95510
583 3.52359 4.82808 4.93408 4.48241 1.18947 4.58547 4.30871
2.57117 4.32144 1.32349 3.19428 4.52062 4.82985 4.36655 4.38344
3.96777 3.75291 2.68792 4.48887 2.93045 644 f - - -
2.68618 4.42225 2.77519 2.73123 3.46354 2.40513 3.72494
3.29354 2.67741 2.69355 4.24690 2.90347 2.73739 3.18146 2.89801
2.37887 2.77519 2.98518 4.58477 3.61503
0.02187 4.22952 4.95186 0.61958 0.77255 0.48576 0.95510
584 2.61447 4.55195 3.40797 3.23398 4.67007 1.00534 4.43999
4.17545 3.42672 3.81205 4.66354 3.49911 1.87923 3.71579 3.75086
2.76935 3.09572 3.61104 5.88859 4.73889 645 g - - -
2.68618 4.42225 2.77519 2.73123 3.46354 2.40513 3.72494
3.29354 2.67741 2.69355 4.24690 2.90347 2.73739 3.18146 2.89801
2.37887 2.77519 2.98518 4.58477 3.61503
0.02187 4.22952 4.95186 0.61958 0.77255 0.48576 0.95510
585 3.03247 4.70605 3.65295 3.33104 1.22327 3.79951 3.83364
3.25217 3.35509 2.82746 3.90281 2.11462 4.28304 3.62279 3.64086
3.19773 3.32430 3.06107 4.35403 2.71952 646 f - - -
2.68618 4.42225 2.77519 2.73123 3.46354 2.40513 3.72494
3.29354 2.67741 2.69355 4.24690 2.90347 2.73739 3.18146 2.89801
2.37887 2.77519 2.98518 4.58477 3.61503
0.02187 4.22952 4.95186 0.61958 0.77255 0.48576 0.95510

```
    586  2.81815  4.35402  4.02580  3.45173  3.49220  3.89571  4.20980
1.81465  3.23197  2.34899  3.41752  3.76726  4.26534  3.60832  2.41293
3.19425  3.06019  1.54650  5.08472  3.87414    647 v - - -
         2.68618  4.42225  2.77519  2.73123  3.46354  2.40513  3.72494
3.29354  2.67741  2.69355  4.24690  2.90347  2.73739  3.18146  2.89801
2.37887  2.77519  2.98518  4.58477  3.61503
         0.02187  4.22952  4.95186  0.61958  0.77255  0.48576  0.95510
    587  3.00740  5.35611  1.07845  2.37267  4.81429  3.36046  3.97596
4.34419  2.98302  3.88285  4.74563  2.93528  2.18494  3.15673  3.53778
2.95230  3.32866  3.91289  5.99718  4.58152    648 d - - -
         2.68618  4.42225  2.77519  2.73123  3.46354  2.40513  3.72494
3.29354  2.67741  2.69355  4.24690  2.90347  2.73739  3.18146  2.89801
2.37887  2.77519  2.98518  4.58477  3.61503
         0.02187  4.22952  4.95186  0.61958  0.77255  0.48576  0.95510
    588  2.70123  4.82110  3.16087  2.59282  4.10027  3.50950  3.69740
3.48846  2.02222  3.09849  3.93215  3.07963  3.90818  2.85867  2.26945
2.15406  2.93252  2.40497  5.33363  4.04044    649 k - - -
         2.68618  4.42225  2.77519  2.73123  3.46354  2.40513  3.72494
3.29354  2.67741  2.69355  4.24690  2.90347  2.73739  3.18146  2.89801
2.37887  2.77519  2.98518  4.58477  3.61503
         0.02187  4.22952  4.95186  0.61958  0.77255  0.48576  0.95510
    589  2.63870  4.66069  3.08869  2.66800  3.97138  3.41037  3.83243
3.39938  2.68179  2.24706  3.92863  1.97023  3.90661  3.04337  3.08652
2.05294  2.93023  3.08878  5.32046  3.99934    650 n - - -
         2.68618  4.42225  2.77519  2.73123  3.46354  2.40513  3.72494
3.29354  2.67741  2.69355  4.24690  2.90347  2.73739  3.18146  2.89801
2.37887  2.77519  2.98518  4.58477  3.61503
         0.02187  4.22952  4.95186  0.61958  0.77255  0.48576  0.95510
    590  3.24751  4.66399  4.44617  3.97050  2.67953  4.24235  4.07427
2.70504  3.76513  1.05941  3.34690  4.11515  4.56538  3.99190  3.91978
3.59247  3.48021  2.70911  4.40876  1.91653    651 l - - -
         2.68618  4.42225  2.77519  2.73123  3.46354  2.40513  3.72494
3.29354  2.67741  2.69355  4.24690  2.90347  2.73739  3.18146  2.89801
2.37887  2.77519  2.98518  4.58477  3.61503
         0.02187  4.22952  4.95186  0.61958  0.77255  0.48576  0.95510
    591  2.83621  5.27003  2.02230  1.80565  4.58914  3.36765  3.76491
4.04824  2.64377  3.58771  4.39462  2.85590  3.89313  2.90788  3.16848
2.78397  1.80353  3.65067  5.77055  4.34338    652 t - - -
         2.68618  4.42225  2.77519  2.73123  3.46354  2.40513  3.72494
3.29354  2.67741  2.69355  4.24690  2.90347  2.73739  3.18146  2.89801
2.37887  2.77519  2.98518  4.58477  3.61503
         0.02187  4.22952  4.95186  0.61958  0.77255  0.48576  0.95510
    592  2.73690  5.02751  2.75645  2.43291  4.44982  3.37599  3.76485
3.92044  2.54539  3.46612  4.27177  2.17780  3.88432  2.25990  2.98969
1.71427  3.01579  3.52035  5.63439  4.26226    653 s - - -
         2.68618  4.42225  2.77519  2.73123  3.46354  2.40513  3.72494
3.29354  2.67741  2.69355  4.24690  2.90347  2.73739  3.18146  2.89801
2.37887  2.77519  2.98518  4.58477  3.61503
         0.02187  4.22952  4.95186  0.61958  0.77255  0.48576  0.95510
    593  2.78891  5.15806  2.73945  1.90488  4.50532  3.42395  3.71073
3.95344  2.01601  3.47630  4.27523  2.92814  3.89306  2.84330  2.87279
1.76400  3.03483  3.56515  5.63637  4.26427    654 s - - -
         2.68621  4.42228  2.77522  2.73126  3.46357  2.40516  3.72497
3.29357  2.67744  2.69358  4.24693  2.90350  2.73743  3.18149  2.89804
2.37890  2.77472  2.98521  4.58480  3.61506
```

0.36810 1.72517 2.04170 0.25055 1.50674 0.48576 0.95510
594 1.87377 4.24964 3.64432 3.16417 3.66101 3.38509 4.07158
1.96150 3.10728 2.66833 3.61391 3.44749 3.93834 3.41482 3.41875
2.09993 2.84007 2.50189 5.14327 3.91776 656 a - - -
2.68618 4.42225 2.77519 2.73123 3.46354 2.40513 3.72494
3.29354 2.67741 2.69355 4.24690 2.90347 2.73739 3.18146 2.89801
2.37887 2.77519 2.98518 4.58477 3.61503
0.02496 4.09857 4.82092 0.61958 0.77255 0.40295 1.10367
595 2.74826 5.18477 2.05755 2.31290 4.48918 3.38599 2.69266
3.96814 2.50815 3.48684 4.26959 2.20865 3.85623 2.81999 3.01191
2.70822 2.37238 3.56587 5.65088 4.23830 657 d - - -
2.68618 4.42225 2.77519 2.73123 3.46354 2.40513 3.72494
3.29354 2.67741 2.69355 4.24690 2.90347 2.73739 3.18146 2.89801
2.37887 2.77519 2.98518 4.58477 3.61503
0.02187 4.22952 4.95186 0.61958 0.77255 0.48576 0.95510
596 2.72884 5.14049 2.23652 2.33232 4.38708 3.40299 3.67271
3.89616 2.47519 3.42236 4.20802 2.21010 3.85280 2.20141 2.96829
2.69653 2.96931 3.50721 5.57249 2.87623 658 q - - -
2.68618 4.42225 2.77519 2.73123 3.46354 2.40513 3.72494
3.29354 2.67741 2.69355 4.24690 2.90347 2.73739 3.18146 2.89801
2.37887 2.77519 2.98518 4.58477 3.61503
0.02187 4.22952 4.95186 0.61958 0.77255 0.48576 0.95510
597 3.58296 4.94778 4.76280 4.41894 3.31381 4.43210 4.96505
2.55063 4.18445 0.53374 3.25075 4.66084 4.81524 4.46461 4.29594
4.06845 3.86586 2.67792 5.30656 4.10752 659 L - - -
2.68618 4.42225 2.77519 2.73123 3.46354 2.40513 3.72494
3.29354 2.67741 2.69355 4.24690 2.90347 2.73739 3.18146 2.89801
2.37887 2.77519 2.98518 4.58477 3.61503
0.02187 4.22952 4.95186 0.61958 0.77255 0.48576 0.95510
598 2.85219 4.48895 3.85117 3.25231 3.40599 3.83885 4.04798
2.70229 2.92636 1.74769 2.02733 3.61379 4.19071 3.38183 2.25677
3.12333 3.07745 2.61649 5.01807 3.81974 660 l - - -
2.68618 4.42225 2.77519 2.73123 3.46354 2.40513 3.72494
3.29354 2.67741 2.69355 4.24690 2.90347 2.73739 3.18146 2.89801
2.37887 2.77519 2.98518 4.58477 3.61503
0.02187 4.22952 4.95186 0.61958 0.77255 0.48576 0.95510
599 2.62787 4.74754 3.00836 2.61183 4.26527 2.45143 3.83532
3.68142 2.62459 3.29097 4.11798 3.07960 3.87785 2.14129 3.03415
2.69382 1.78513 3.30356 5.51897 4.20594 661 t - - -
2.68618 4.42225 2.77519 2.73123 3.46354 2.40513 3.72494
3.29354 2.67741 2.69355 4.24690 2.90347 2.73739 3.18146 2.89801
2.37887 2.77519 2.98518 4.58477 3.61503
0.02187 4.22952 4.95186 0.61958 0.77255 0.48576 0.95510
600 3.04556 5.54246 1.67958 2.25554 4.87401 3.33493 3.91657
4.44758 2.94284 3.95600 4.81348 1.23621 3.96935 3.09161 3.52918
2.94746 3.35299 4.00681 6.07377 4.57343 662 n - - -
2.68618 4.42225 2.77519 2.73123 3.46354 2.40513 3.72494
3.29354 2.67741 2.69355 4.24690 2.90347 2.73739 3.18146 2.89801
2.37887 2.77519 2.98518 4.58477 3.61503
0.02187 4.22952 4.95186 0.61958 0.77255 0.48576 0.95510
601 3.20518 4.53951 4.91009 4.33450 2.09239 4.43207 4.69192
1.74339 4.19134 1.62518 1.78124 4.49418 4.66808 4.27394 4.26564
3.76452 3.42810 2.33591 5.02712 3.86131 663 l - - -

2.68618 4.42225 2.77519 2.73123 3.46354 2.40513 3.72494
3.29354 2.67741 2.69355 4.24690 2.90347 2.73739 3.18146 2.89801
2.37887 2.77519 2.98518 4.58477 3.61503
0.02187 4.22952 4.95186 0.61958 0.77255 0.48576 0.95510
602 2.13442 4.39828 4.50549 3.96712 3.47318 4.18024 4.62257
1.36235 3.84558 1.74642 3.34128 4.20103 4.53130 4.09631 4.04816
3.51723 3.24243 1.92990 5.25296 4.07049 664 i - - -
2.68618 4.42225 2.77519 2.73123 3.46354 2.40513 3.72494
3.29354 2.67741 2.69355 4.24690 2.90347 2.73739 3.18146 2.89801
2.37887 2.77519 2.98518 4.58477 3.61503
0.02187 4.22952 4.95186 0.61958 0.77255 0.48576 0.95510
603 2.55187 4.36240 3.55277 3.06556 3.74217 3.41325 4.02524
3.00726 2.99279 2.06113 3.70151 3.39564 3.95202 3.33109 3.32459
2.15609 1.72268 2.74432 5.19014 3.94891 665 t - - -
2.68618 4.42225 2.77519 2.73123 3.46354 2.40513 3.72494
3.29354 2.67741 2.69355 4.24690 2.90347 2.73739 3.18146 2.89801
2.37887 2.77519 2.98518 4.58477 3.61503
0.02187 4.22952 4.95186 0.61958 0.77255 0.48576 0.95510
604 2.05452 4.70419 3.04040 2.63878 4.29340 3.31340 3.86043
3.71772 2.66217 3.32586 4.14610 3.09270 3.86243 2.17790 3.07704
1.64950 2.91912 3.31686 5.54801 4.23352 666 s - - -
2.68618 4.42225 2.77519 2.73123 3.46354 2.40513 3.72494
3.29354 2.67741 2.69355 4.24690 2.90347 2.73739 3.18146 2.89801
2.37887 2.77519 2.98518 4.58477 3.61503
0.02187 4.22952 4.95186 0.61958 0.77255 0.48576 0.95510
605 2.91185 4.57713 3.74520 3.22496 3.71899 3.83403 4.09421
1.37350 2.02357 2.56042 3.64176 3.59915 4.23535 3.38187 3.07027
3.17856 3.15409 2.40231 5.24228 4.00706 667 i - - -
2.68618 4.42225 2.77519 2.73123 3.46354 2.40513 3.72494
3.29354 2.67741 2.69355 4.24690 2.90347 2.73739 3.18146 2.89801
2.37887 2.77519 2.98518 4.58477 3.61503
0.02187 4.22952 4.95186 0.61958 0.77255 0.48576 0.95510
606 3.29869 4.97961 3.94636 3.84728 4.78758 3.64774 4.88149
4.44313 3.93886 4.02289 5.09758 4.11130 0.36966 4.31567 4.13026
3.48053 3.77053 4.04412 5.85897 4.91765 668 P - - -
2.68618 4.42225 2.77519 2.73123 3.46354 2.40513 3.72494
3.29354 2.67741 2.69355 4.24690 2.90347 2.73739 3.18146 2.89801
2.37887 2.77519 2.98518 4.58477 3.61503
0.02187 4.22952 4.95186 0.61958 0.77255 0.48576 0.95510
607 1.32448 4.36811 4.07038 3.62574 3.72088 3.67331 4.46216
1.74175 3.53442 2.50500 3.63189 3.84074 4.22926 3.84405 3.79523
3.06912 3.08136 2.19487 5.37305 4.15327 669 a - - -
2.68618 4.42225 2.77519 2.73123 3.46354 2.40513 3.72494
3.29354 2.67741 2.69355 4.24690 2.90347 2.73739 3.18146 2.89801
2.37887 2.77519 2.98518 4.58477 3.61503
0.02187 4.22952 4.95186 0.61958 0.77255 0.48576 0.95510
608 2.20554 4.76059 3.01595 1.76300 3.99022 3.48980 3.79764
3.25624 2.62853 2.21260 3.87100 3.09299 3.93505 2.98745 3.05055
2.78805 2.96325 2.99459 5.34505 4.03738 670 e - - -
2.68618 4.42225 2.77519 2.73123 3.46354 2.40513 3.72494
3.29354 2.67741 2.69355 4.24690 2.90347 2.73739 3.18146 2.89801
2.37887 2.77519 2.98518 4.58477 3.61503
0.02187 4.22952 4.95186 0.61958 0.77255 0.48576 0.95510

609 2.64367 4.41743 3.43823 2.87782 3.57334 2.55440 3.84088
2.27575 2.30093 2.61168 2.70007 3.29920 3.97676 3.13452 3.12968
2.84059 2.87894 2.65524 5.00522 3.76356 671 i - - -
2.68618 4.42225 2.77519 2.73123 3.46354 2.40513 3.72494
3.29354 2.67741 2.69355 4.24690 2.90347 2.73739 3.18146 2.89801
2.37887 2.77519 2.98518 4.58477 3.61503
0.02187 4.22952 4.95186 0.61958 0.77255 0.48576 0.95510
610 1.97617 4.42075 3.41432 2.95158 3.60482 3.42240 3.92697
3.15120 2.93091 2.84965 3.74116 3.31904 3.94141 3.25349 3.28724
2.03280 2.89454 2.86967 5.05882 2.07162 672 a - - -
2.68618 4.42225 2.77519 2.73123 3.46354 2.40513 3.72494
3.29354 2.67741 2.69355 4.24690 2.90347 2.73739 3.18146 2.89801
2.37887 2.77519 2.98518 4.58477 3.61503
0.02187 4.22952 4.95186 0.61958 0.77255 0.48576 0.95510
611 2.66154 4.54923 3.27527 2.74137 3.70791 3.55214 3.79352
3.04049 2.66637 2.19343 3.64170 2.53697 3.94914 2.45421 3.04860
2.80314 2.89732 2.19495 5.10742 3.83540 673 l - - -
2.68618 4.42225 2.77519 2.73123 3.46354 2.40513 3.72494
3.29354 2.67741 2.69355 4.24690 2.90347 2.73739 3.18146 2.89801
2.37887 2.77519 2.98518 4.58477 3.61503
0.15591 4.22952 2.04170 0.61958 0.77255 0.48576 0.95510
612 2.65111 4.62616 3.33279 2.84128 4.12292 3.39304 3.84815
3.46343 2.46837 3.12073 4.01409 3.22826 3.91778 3.04755 2.05744
2.76494 1.53865 3.14180 5.38149 4.13424 674 t - - -
2.68618 4.42225 2.77519 2.73123 3.46354 2.40513 3.72494
3.29354 2.67741 2.69355 4.24690 2.90347 2.73739 3.18146 2.89801
2.37887 2.77519 2.98518 4.58477 3.61503
0.02496 4.09857 4.82092 0.61958 0.77255 0.40295 1.10367
613 2.11029 4.98355 2.90296 2.06179 4.33211 2.61995 3.68192
3.76712 2.41989 3.32016 4.11525 2.95916 3.85798 2.81454 2.23884
2.68690 2.93138 3.39373 5.50855 4.15521 675 e - - -
2.68618 4.42225 2.77519 2.73123 3.46354 2.40513 3.72494
3.29354 2.67741 2.69355 4.24690 2.90347 2.73739 3.18146 2.89801
2.37887 2.77519 2.98518 4.58477 3.61503
0.02187 4.22952 4.95186 0.61958 0.77255 0.48576 0.95510
614 2.64310 4.40163 3.45491 2.89383 2.52863 3.59609 3.83921
2.88283 2.33071 2.55975 2.68252 3.31115 2.73510 3.15091 3.16753
2.84557 2.87648 2.66603 4.95511 3.69977 676 k - - -
2.68618 4.42225 2.77519 2.73123 3.46354 2.40513 3.72494
3.29354 2.67741 2.69355 4.24690 2.90347 2.73739 3.18146 2.89801
2.37887 2.77519 2.98518 4.58477 3.61503
0.02187 4.22952 4.95186 0.61958 0.77255 0.48576 0.95510
615 2.73991 5.04506 2.75610 2.42414 4.45582 3.38177 3.75342
3.92633 2.52710 3.46717 4.27013 2.18438 3.88279 2.12090 2.97167
1.81683 3.01376 3.52639 5.63253 4.25858 677 s - - -
2.68621 4.42228 2.77522 2.73126 3.46357 2.40515 3.72497
3.29357 2.67743 2.69315 4.24692 2.90349 2.73742 3.18149 2.89803
2.37890 2.77522 2.98521 4.58480 3.61506
0.41163 1.79111 1.76812 0.26129 1.46993 0.48576 0.95510
616 2.88117 4.87751 2.82257 2.72791 4.27011 3.31154 4.07951
4.02278 3.00034 3.65026 4.60713 0.87702 3.97372 3.35551 3.35855
2.94339 3.27072 3.62671 5.55571 4.20790 679 n - - -
2.68618 4.42225 2.77519 2.73123 3.46354 2.40513 3.72494
3.29354 2.67741 2.69355 4.24690 2.90347 2.73739 3.18146 2.89801
2.37887 2.77519 2.98518 4.58477 3.61503

0.02620 4.05089 4.77324 0.61958 0.77255 0.38139 1.14858
617 2.76933 4.35274 3.84828 3.28771 2.04609 3.79314 4.01100
2.03940 2.26447 2.30448 3.35553 3.61667 4.16025 3.46160 3.39642
3.07533 3.00231 2.44716 4.82780 3.52477 680 i - - -
2.68618 4.42225 2.77519 2.73123 3.46354 2.40513 3.72494
3.29354 2.67741 2.69355 4.24690 2.90347 2.73739 3.18146 2.89801
2.37887 2.77519 2.98518 4.58477 3.61503
0.02187 4.22952 4.95186 0.61958 0.77255 0.48576 0.95510
618 2.79875 5.31402 1.99865 2.28678 4.63748 3.38702 3.69597
4.12572 2.05494 3.60578 4.37984 2.17056 3.86841 2.24816 2.98802
2.73627 3.04355 3.69762 5.74306 4.31480 681 d - - -
2.68618 4.42225 2.77519 2.73123 3.46354 2.40513 3.72494
3.29354 2.67741 2.69355 4.24690 2.90347 2.73739 3.18146 2.89801
2.37887 2.77519 2.98518 4.58477 3.61503
0.31255 4.22952 1.37097 0.61958 0.77255 0.48576 0.95510
619 2.74933 4.53035 3.37996 2.91118 3.53521 3.61178 3.89231
1.93163 2.69922 2.39294 3.50500 3.33155 4.03246 2.16431 3.00538
2.94157 2.99958 2.55577 5.07978 3.79876 682 i - - -
2.68618 4.42225 2.77519 2.73123 3.46354 2.40513 3.72494
3.29354 2.67741 2.69355 4.24690 2.90347 2.73739 3.18146 2.89801
2.37887 2.77519 2.98518 4.58477 3.61503
0.02914 3.94610 4.66845 0.61958 0.77255 0.34396 1.23429
620 2.84041 4.60386 3.67603 3.28997 3.69988 3.61445 4.23217
3.01009 3.16685 1.78684 3.74898 3.62307 1.33162 3.56746 3.45303
3.05307 3.17809 2.85779 5.27616 3.99753 683 p - - -
2.68618 4.42225 2.77519 2.73123 3.46354 2.40513 3.72494
3.29354 2.67741 2.69355 4.24690 2.90347 2.73739 3.18146 2.89801
2.37887 2.77519 2.98518 4.58477 3.61503
0.02187 4.22952 4.95186 0.61958 0.77255 0.48576 0.95510
621 2.75438 4.91183 2.99048 2.54184 4.10202 3.49566 3.72333
3.56145 2.44837 2.33515 4.00849 2.25025 3.92512 1.94692 2.83609
2.78683 2.99138 3.25679 5.37218 4.03265 684 q - - -
2.68618 4.42225 2.77519 2.73123 3.46354 2.40513 3.72494
3.29354 2.67741 2.69355 4.24690 2.90347 2.73739 3.18146 2.89801
2.37887 2.77519 2.98518 4.58477 3.61503
0.02187 4.22952 4.95186 0.61958 0.77255 0.48576 0.95510
622 2.81141 4.26024 4.27017 3.70199 3.14213 3.92997 4.18794
2.38417 3.56196 2.12269 2.43818 3.90650 4.28155 3.78794 3.73903
3.23266 3.04730 1.55077 4.77900 2.36726 685 v - - -
2.68621 4.42228 2.77522 2.73081 3.46357 2.40515 3.72497
3.29357 2.67743 2.69358 4.24692 2.90349 2.73742 3.18149 2.89803
2.37890 2.77522 2.98521 4.58480 3.61506
0.19097 1.79111 4.95186 0.26129 1.46993 0.48576 0.95510
623 3.31529 5.45321 2.70580 0.60645 4.85294 3.54554 4.18442
4.38266 3.11533 3.94788 4.92308 3.20387 4.17210 3.40321 3.54988
3.27009 3.62767 4.03530 5.95474 4.69895 687 E - - -
2.68618 4.42225 2.77519 2.73123 3.46354 2.40513 3.72494
3.29354 2.67741 2.69355 4.24690 2.90347 2.73739 3.18146 2.89801
2.37887 2.77519 2.98518 4.58477 3.61503
0.02187 4.22952 4.95186 0.61958 0.77255 0.48576 0.95510
624 2.61219 4.80558 2.99983 2.52198 4.23611 3.37515 3.74167
3.66326 2.52517 3.25139 4.05777 3.01699 2.75485 2.19071 2.96743
2.17974 2.22649 3.29021 5.46989 4.13646 688 s - - -

```
         2.68618  4.42225  2.77519  2.73123  3.46354  2.40513  3.72494
3.29354  2.67741  2.69355  4.24690  2.90347  2.73739  3.18146  2.89801
2.37887  2.77519  2.98518  4.58477  3.61503
         0.02187  4.22952  4.95186  0.61958  0.77255  0.48576  0.95510
    625  3.25990  4.52873  5.10955  4.62873  3.72790  4.69219  5.33080
0.98617  4.52526  2.19064  3.50863  4.81764  4.97720  4.77709  4.70171
4.10163  3.52618  1.27572  5.75776  4.55531    689 i - - -
         2.68618  4.42225  2.77519  2.73123  3.46354  2.40513  3.72494
3.29354  2.67741  2.69355  4.24690  2.90347  2.73739  3.18146  2.89801
2.37887  2.77519  2.98518  4.58477  3.61503
         0.02187  4.22952  4.95186  0.61958  0.77255  0.48576  0.95510
    626  2.93350  5.16178  2.72008  2.50718  4.09005  3.45486  2.46459
4.02070  2.63915  3.54478  4.41635  1.30229  3.99587  3.04265  3.03491
2.91764  3.21220  3.65523  5.42992  3.94529    690 n - - -
         2.68618  4.42225  2.77519  2.73123  3.46354  2.40513  3.72494
3.29354  2.67741  2.69355  4.24690  2.90347  2.73739  3.18146  2.89801
2.37887  2.77519  2.98518  4.58477  3.61503
         0.02187  4.22952  4.95186  0.61958  0.77255  0.48576  0.95510
    627  1.54137  4.55987  3.07123  2.78627  4.44636  2.09711  4.05573
3.88278  2.95903  3.51086  4.32448  2.04451  3.84502  3.25370  3.37741
2.61232  2.91373  3.39328  5.72607  4.43023    691 a - - -
         2.68618  4.42225  2.77519  2.73123  3.46354  2.40513  3.72494
3.29354  2.67741  2.69355  4.24690  2.90347  2.73739  3.18146  2.89801
2.37887  2.77519  2.98518  4.58477  3.61503
         0.02187  4.22952  4.95186  0.61958  0.77255  0.48576  0.95510
    628  3.06036  5.29558  3.19659  2.70202  4.70611  3.65681  3.71677
4.10617  1.36097  3.54858  4.39933  3.16473  4.06684  1.67963  2.35575
3.03347  3.25349  3.75841  5.60810  4.37049    692 k - - -
         2.68618  4.42225  2.77519  2.73123  3.46354  2.40513  3.72494
3.29354  2.67741  2.69355  4.24690  2.90347  2.73739  3.18146  2.89801
2.37887  2.77519  2.98518  4.58477  3.61503
         0.02187  4.22952  4.95186  0.61958  0.77255  0.48576  0.95510
    629  2.87894  5.35981  2.54270  1.78303  4.67732  3.39912  3.74240
4.16058  2.53229  3.64899  4.44880  2.11542  3.90971  1.83492  3.00131
2.81186  3.12853  3.74843  5.78797  4.36905    693 e - - -
         2.68618  4.42225  2.77519  2.73123  3.46354  2.40513  3.72494
3.29354  2.67741  2.69355  4.24690  2.90347  2.73739  3.18146  2.89801
2.37887  2.77519  2.98518  4.58477  3.61503
         0.02187  4.22952  4.95186  0.61958  0.77255  0.48576  0.95510
    630  2.14877  5.08444  3.03370  2.53710  4.45228  3.51316  3.68676
3.86289  1.63501  3.38532  4.19865  3.03782  3.93269  2.11790  2.61102
2.80395  3.03590  3.50119  5.53421  4.22592    694 k - - -
         2.68618  4.42225  2.77519  2.73123  3.46354  2.40513  3.72494
3.29354  2.67741  2.69355  4.24690  2.90347  2.73739  3.18146  2.89801
2.37887  2.77519  2.98518  4.58477  3.61503
         0.31255  4.22952  1.37097  0.61958  0.77255  0.48576  0.95510
    631  3.18447  4.52586  4.69948  4.17824  3.20117  4.43822  4.80480
1.43332  4.00773  1.25875  3.03737  4.44160  4.68829  4.23460  4.17796
3.80904  3.42717  1.92535  5.22505  4.05582    695 l - - -
         2.68618  4.42225  2.77519  2.73123  3.46354  2.40513  3.72494
3.29354  2.67741  2.69355  4.24690  2.90347  2.73739  3.18146  2.89801
2.37887  2.77519  2.98518  4.58477  3.61503
         0.02914  3.94610  4.66845  0.61958  0.77255  0.76495  0.62616
```

632 2.67390 4.52873 3.26015 2.82312 3.68107 3.48631 3.86032
2.85405 2.65109 2.60476 3.64927 3.24390 3.95711 2.12334 2.97153
2.83579 2.95189 1.89129 5.15689 3.86813 696 v - - -
2.68624 4.42231 2.77525 2.73129 3.46360 2.40518 3.72500
3.29360 2.67746 2.69318 4.24695 2.90352 2.73745 3.18152 2.89806
2.37892 2.77525 2.98524 4.58198 3.61509
0.26241 1.50770 4.66845 0.66816 0.71878 0.76495 0.62616
633 2.81456 5.09639 2.55007 1.71428 4.21826 3.34133 2.17864
3.83853 2.44130 3.37760 4.23442 2.85583 3.85358 2.84871 2.84638
2.78395 3.06546 3.49182 5.45525 4.04835 699 e - - -
2.68618 4.42225 2.77519 2.73123 3.46354 2.40513 3.72494
3.29354 2.67741 2.69355 4.24690 2.90347 2.73739 3.18146 2.89801
2.37887 2.77519 2.98518 4.58477 3.61503
0.02914 3.94610 4.66845 0.61958 0.77255 0.34396 1.23429
634 1.70668 4.38746 3.53798 3.05555 3.73725 3.43712 4.03213
2.98192 2.99863 1.99764 3.68205 3.39861 2.47831 3.33092 3.33778
2.77936 2.90206 2.73644 5.20311 3.96475 700 a - - -
2.68618 4.42225 2.77519 2.73123 3.46354 2.40513 3.72494
3.29354 2.67741 2.69355 4.24690 2.90347 2.73739 3.18146 2.89801
2.37887 2.77519 2.98518 4.58477 3.61503
0.02187 4.22952 4.95186 0.61958 0.77255 0.48576 0.95510
635 2.60188 2.59849 3.71261 3.17627 3.46945 3.55812 3.99711
2.76414 3.07189 2.52626 2.09976 2.44495 4.01534 3.38737 3.36379
2.86888 2.88154 2.54578 4.95586 3.72595 701 m - - -
2.68618 4.42225 2.77519 2.73123 3.46354 2.40513 3.72494
3.29354 2.67741 2.69355 4.24690 2.90347 2.73739 3.18146 2.89801
2.37887 2.77519 2.98518 4.58477 3.61503
0.02187 4.22952 4.95186 0.61958 0.77255 0.48576 0.95510
636 2.69970 4.70192 3.19406 2.67238 2.60499 3.52606 3.75288
3.30178 1.81993 2.93351 3.81623 3.14445 2.58949 2.96968 2.92716
2.79015 2.94062 3.03078 5.17201 3.83783 702 k - - -
2.68618 4.42225 2.77519 2.73123 3.46354 2.40513 3.72494
3.29354 2.67741 2.69355 4.24690 2.90347 2.73739 3.18146 2.89801
2.37887 2.77519 2.98518 4.58477 3.61503
0.02187 4.22952 4.95186 0.61958 0.77255 0.48576 0.95510
637 2.47380 4.40436 3.49296 3.16978 4.29039 3.19734 4.25021
3.61676 3.16785 3.34568 4.23336 3.41700 2.16576 3.51289 3.48869
2.64471 1.17728 3.19345 5.64712 4.42051 703 t - - -
2.68618 4.42225 2.77519 2.73123 3.46354 2.40513 3.72494
3.29354 2.67741 2.69355 4.24690 2.90347 2.73739 3.18146 2.89801
2.37887 2.77519 2.98518 4.58477 3.61503
0.02187 4.22952 4.95186 0.61958 0.77255 0.48576 0.95510
638 3.04477 5.39084 1.01765 2.37012 4.93719 1.99713 4.03060
4.48977 3.09627 4.01773 4.88711 2.93441 4.00249 3.22080 3.67865
2.98439 3.38862 4.02418 6.10681 4.68465 704 d - - -
2.68618 4.42225 2.77519 2.73123 3.46354 2.40513 3.72494
3.29354 2.67741 2.69355 4.24690 2.90347 2.73739 3.18146 2.89801
2.37887 2.77519 2.98518 4.58477 3.61503
0.02187 4.22952 4.95186 0.61958 0.77255 0.48576 0.95510
639 2.78645 5.14089 1.95912 2.34343 4.53697 3.34918 3.79067
3.99656 2.67092 3.55263 4.36201 2.22945 3.88708 2.94004 3.18356
2.76068 1.78007 3.59305 5.74017 4.33056 705 t - - -
2.68618 4.42225 2.77519 2.73123 3.46354 2.40513 3.72494
3.29354 2.67741 2.69355 4.24690 2.90347 2.73739 3.18146 2.89801
2.37887 2.77519 2.98518 4.58477 3.61503

0.02187 4.22952 4.95186 0.61958 0.77255 0.48576 0.95510
640 2.66037 4.88441 2.02325 2.47164 4.42311 3.31207 3.83068
3.85890 2.69748 3.44979 4.25867 2.97928 3.86230 2.98831 3.18085
2.02007 1.85855 3.44586 5.65819 4.29266 706 t - - -
2.68618 4.42225 2.77519 2.73123 3.46354 2.40513 3.72494
3.29354 2.67741 2.69355 4.24690 2.90347 2.73739 3.18146 2.89801
2.37887 2.77519 2.98518 4.58477 3.61503
0.02187 4.22952 4.95186 0.61958 0.77255 0.48576 0.95510
641 2.73490 5.01123 2.81981 2.44900 4.38883 3.40724 3.73807
3.83257 2.48856 3.39063 4.20131 1.93236 3.88713 2.13726 2.91702
2.73760 2.22990 3.45574 5.57405 4.21720 707 n - - -
2.68618 4.42225 2.77519 2.73123 3.46354 2.40513 3.72494
3.29354 2.67741 2.69355 4.24690 2.90347 2.73739 3.18146 2.89801
2.37887 2.77519 2.98518 4.58477 3.61503
0.02187 4.22952 4.95186 0.61958 0.77255 0.48576 0.95510
642 2.74868 5.05130 2.84031 2.45574 4.46755 3.41420 3.72956
3.91709 1.89526 3.44927 4.25022 2.21095 3.89142 2.86613 2.84686
1.83784 3.01049 3.52246 5.60722 4.25407 708 s - - -
2.68618 4.42225 2.77519 2.73123 3.46354 2.40513 3.72494
3.29354 2.67741 2.69355 4.24690 2.90347 2.73739 3.18146 2.89801
2.37887 2.77519 2.98518 4.58477 3.61503
0.02187 4.22952 4.95186 0.61958 0.77255 0.48576 0.95510
643 3.00133 4.40684 4.47662 3.92300 2.94144 4.12556 4.22136
1.85222 3.76545 1.56029 3.20906 4.08604 4.44222 3.95522 3.91159
3.43804 3.23085 2.35604 4.65723 1.90303 709 l - - -
2.68618 4.42225 2.77519 2.73123 3.46354 2.40513 3.72494
3.29354 2.67741 2.69355 4.24690 2.90347 2.73739 3.18146 2.89801
2.37887 2.77519 2.98518 4.58477 3.61503
0.02187 4.22952 4.95186 0.61958 0.77255 0.48576 0.95510
644 3.53669 4.83461 5.05410 4.55401 1.94845 4.69348 4.64571
2.43443 4.40229 0.81909 3.01582 4.68976 4.88022 4.43269 4.46107
4.07297 3.75780 2.60882 4.82259 3.41731 710 l - - -
2.68618 4.42225 2.77519 2.73123 3.46354 2.40513 3.72494
3.29354 2.67741 2.69355 4.24690 2.90347 2.73739 3.18146 2.89801
2.37887 2.77519 2.98518 4.58477 3.61503
0.02187 4.22952 4.95186 0.61958 0.77255 0.48576 0.95510
645 1.95674 4.56137 3.14441 2.75161 4.32884 2.14575 3.96395
3.75256 2.83173 3.37737 4.18748 2.16723 3.83285 3.14861 3.25763
2.59646 2.16638 3.30438 5.60757 4.31100 711 a - - -
2.68618 4.42225 2.77519 2.73123 3.46354 2.40513 3.72494
3.29354 2.67741 2.69355 4.24690 2.90347 2.73739 3.18146 2.89801
2.37887 2.77519 2.98518 4.58477 3.61503
0.02187 4.22952 4.95186 0.61958 0.77255 0.48576 0.95510
646 3.98789 5.12769 4.92363 4.68751 1.37439 4.65031 3.56786
3.63623 4.51386 2.90744 4.21157 4.28522 4.94301 4.38481 4.47528
4.03732 4.20332 3.57233 3.67794 0.84206 712 y - - -
2.68618 4.42225 2.77519 2.73123 3.46354 2.40513 3.72494
3.29354 2.67741 2.69355 4.24690 2.90347 2.73739 3.18146 2.89801
2.37887 2.77519 2.98518 4.58477 3.61503
0.02187 4.22952 4.95186 0.61958 0.77255 0.48576 0.95510
647 2.80838 5.12986 2.71927 2.42578 4.55509 2.45165 3.76845
4.01273 2.01098 3.53877 4.34841 1.67859 3.91246 2.91157 2.92476
2.79008 3.07818 3.61112 5.69126 4.32557 713 n - - -

```
         2.68618  4.42225  2.77519  2.73123  3.46354  2.40513  3.72494
3.29354  2.67741  2.69355  4.24690  2.90347  2.73739  3.18146  2.89801
2.37887  2.77519  2.98518  4.58477  3.61503
         0.02187  4.22952  4.95186  0.61958  0.77255  0.48576  0.95510
    648  3.25611  4.51985  5.12220  4.63987  3.74908  4.70263  5.34403
1.10037  4.54147  2.21854  3.52743  4.82680  4.98444  4.79462  4.71908
4.11095  3.52136  1.12543  5.77369  4.56788    714 i - - -
         2.68618  4.42225  2.77519  2.73123  3.46354  2.40513  3.72494
3.29354  2.67741  2.69355  4.24690  2.90347  2.73739  3.18146  2.89801
2.37887  2.77519  2.98518  4.58477  3.61503
         0.02187  4.22952  4.95186  0.61958  0.77255  0.48576  0.95510
    649  2.85025  5.26504  2.52909  1.88154  4.61535  3.37171  3.77859
4.09238  2.62951  3.61580  4.42513  1.73935  2.39617  2.92378  3.12648
2.80097  3.11857  3.68657  5.78160  4.36353    715 n - - -
         2.68618  4.42225  2.77519  2.73123  3.46354  2.40513  3.72494
3.29354  2.67741  2.69355  4.24690  2.90347  2.73739  3.18146  2.89801
2.37887  2.77519  2.98518  4.58477  3.61503
         0.02187  4.22952  4.95186  0.61958  0.77255  0.48576  0.95510
    650  1.72186  4.27748  3.71158  3.27613  4.04622  3.20730  4.23590
3.25779  3.24202  3.09474  3.98280  3.47027  3.87881  3.53738  3.55929
1.50289  2.83631  2.10112  5.46669  4.24946    716 s - - -
         2.68627  4.42372  2.77566  2.73126  3.46202  2.40521  3.72641
3.29164  2.67706  2.69363  4.24618  2.90379  2.73838  3.18143  2.89891
2.37831  2.77516  2.98665  4.58318  3.61186
         0.20483  1.72517  4.95186  3.46642  0.03173  0.48576  0.95510
    651  2.64457  4.77813  2.99212  2.52544  4.01392  3.42795  3.72762
3.49284  2.56010  3.10827  3.93849  2.28800  2.65679  2.90542  3.00654
2.22908  2.90173  3.16891  5.32182  2.83707    764 s - - -
         2.68618  4.42225  2.77519  2.73123  3.46354  2.40513  3.72494
3.29354  2.67741  2.69355  4.24690  2.90347  2.73739  3.18146  2.89801
2.37887  2.77519  2.98518  4.58477  3.61503
         0.02187  4.22952  4.95186  0.61958  0.77255  0.48576  0.95510
    652  2.11338  5.09195  3.27395  2.67454  4.49580  3.59951  3.68771
3.87568  1.53737  3.38500  4.21256  3.13815  3.99212  2.81953  1.90666
2.89143  3.09593  3.52967  5.50866  4.25405    765 k - - -
         2.68618  4.42225  2.77519  2.73123  3.46354  2.40513  3.72494
3.29354  2.67741  2.69355  4.24690  2.90347  2.73739  3.18146  2.89801
2.37887  2.77519  2.98518  4.58477  3.61503
         0.02187  4.22952  4.95186  0.61958  0.77255  0.48576  0.95510
    653  2.70738  4.83566  2.93477  1.75097  3.99025  3.46605  3.72979
3.46333  2.55934  3.09244  3.94213  3.02442  3.90217  2.91495  2.99616
2.75227  2.37054  3.16186  5.31176  2.60472    766 e - - -
         2.68618  4.42225  2.77519  2.73123  3.46354  2.40513  3.72494
3.29354  2.67741  2.69355  4.24690  2.90347  2.73739  3.18146  2.89801
2.37887  2.77519  2.98518  4.58477  3.61503
         0.02187  4.22952  4.95186  0.61958  0.77255  0.48576  0.95510
    654  1.93167  4.81774  3.01838  2.68052  4.32251  3.38803  3.89407
3.72143  2.61937  3.33116  4.21475  3.13688  3.94013  1.43058  2.97799
2.78699  3.04291  3.36475  5.57680  4.26620    767 q - - -
         2.68618  4.42225  2.77519  2.73123  3.46354  2.40513  3.72494
3.29354  2.67741  2.69355  4.24690  2.90347  2.73739  3.18146  2.89801
2.37887  2.77519  2.98518  4.58477  3.61503
         0.02187  4.22952  4.95186  0.61958  0.77255  0.48576  0.95510
```

```
     655   3.00407  5.47220  2.38518  1.46869  4.79598  3.36756  3.85987
 4.31228  2.76863  3.82355  4.66710  1.50502  3.96048  3.02214  3.27892
 2.91942  3.28744  3.89696  5.95821  4.50334    768 e - - -
           2.68618  4.42225  2.77519  2.73123  3.46354  2.40513  3.72494
 3.29354  2.67741  2.69355  4.24690  2.90347  2.73739  3.18146  2.89801
 2.37887  2.77519  2.98518  4.58477  3.61503
           0.02187  4.22952  4.95186  0.61958  0.77255  0.48576  0.95510
     656   3.80424  5.14643  4.38673  4.19600  2.40294  4.26283  3.84731
 3.76969  4.04504  3.12739  4.40880  4.19212  4.73410  4.26097  4.14895
 3.89915  4.09590  3.65865  4.05572  0.53072    769 Y - - -
           2.68618  4.42225  2.77519  2.73123  3.46354  2.40513  3.72494
 3.29354  2.67741  2.69355  4.24690  2.90347  2.73739  3.18146  2.89801
 2.37887  2.77519  2.98518  4.58477  3.61503
           0.02187  4.22952  4.95186  0.61958  0.77255  0.48576  0.95510
     657   3.34250  5.28507  3.54004  3.11313  4.75547  3.77450  4.01532
 4.21219  0.63480  3.70584  4.66231  3.51589  4.26127  3.19670  2.57270
 3.36959  3.57582  3.90514  5.67242  4.54523    770 K - - -
           2.68618  4.42225  2.77519  2.73123  3.46354  2.40513  3.72494
 3.29354  2.67741  2.69355  4.24690  2.90347  2.73739  3.18146  2.89801
 2.37887  2.77519  2.98518  4.58477  3.61503
           0.02187  4.22952  4.95186  0.61958  0.77255  0.48576  0.95510
     658   3.32169  4.66102  4.81585  4.45732  3.64239  4.43292  5.13400
 0.71984  4.29347  2.17033  3.53769  4.65564  4.84343  4.61411  4.45169
 3.97643  3.61986  1.92373  5.59674  4.35691    771 i - - -
           2.68618  4.42225  2.77519  2.73123  3.46354  2.40513  3.72494
 3.29354  2.67741  2.69355  4.24690  2.90347  2.73739  3.18146  2.89801
 2.37887  2.77519  2.98518  4.58477  3.61503
           0.02187  4.22952  4.95186  0.61958  0.77255  0.48576  0.95510
     659   2.78569  4.37673  3.86769  3.29310  2.29894  3.80171  4.00447
 2.05452  3.09477  2.27751  3.34954  3.62101  4.16626  3.45078  2.06340
 3.08330  3.01787  2.50006  4.82167  3.51202    772 i - - -
           2.68618  4.42225  2.77519  2.73123  3.46354  2.40513  3.72494
 3.29354  2.67741  2.69355  4.24690  2.90347  2.73739  3.18146  2.89801
 2.37887  2.77519  2.98518  4.58477  3.61503
           0.02187  4.22952  4.95186  0.61958  0.77255  0.48576  0.95510
     660   3.15415  4.58224  4.36341  3.89608  2.63944  4.14761  3.97001
 1.85603  3.71778  2.33827  3.53141  4.02039  4.50401  3.94607  3.88439
 3.49127  3.39644  2.56461  4.31688  1.27843    773 y - - -
           2.68618  4.42225  2.77519  2.73123  3.46354  2.40513  3.72494
 3.29354  2.67741  2.69355  4.24690  2.90347  2.73739  3.18146  2.89801
 2.37887  2.77519  2.98518  4.58477  3.61503
           0.02187  4.22952  4.95186  0.61958  0.77255  0.48576  0.95510
     661   3.26759  5.36652  3.75315  2.99511  4.89070  3.84844  3.71250
 4.20486  1.21624  3.59292  4.46971  3.36637  4.19376  2.84111  1.32958
 3.24972  3.40691  3.88674  5.58668  4.46210    774 k - - -
           2.68618  4.42225  2.77519  2.73123  3.46354  2.40513  3.72494
 3.29354  2.67741  2.69355  4.24690  2.90347  2.73739  3.18146  2.89801
 2.37887  2.77519  2.98518  4.58477  3.61503
           0.02187  4.22952  4.95186  0.61958  0.77255  0.48576  0.95510
     662   3.25611  4.51985  5.12220  4.63987  3.74908  4.70263  5.34403
 1.10037  4.54147  2.21854  3.52743  4.82680  4.98444  4.79462  4.71908
 4.11095  3.52136  1.12543  5.77369  4.56788    775 i - - -
           2.68618  4.42225  2.77519  2.73123  3.46354  2.40513  3.72494
 3.29354  2.67741  2.69355  4.24690  2.90347  2.73739  3.18146  2.89801
 2.37887  2.77519  2.98518  4.58477  3.61503
```

0.02187 4.22952 4.95186 0.61958 0.77255 0.48576 0.95510
663 0.60109 4.41262 3.86724 3.69364 4.44957 3.22442 4.66715
3.63204 3.72040 3.49556 4.49930 3.73948 3.99876 4.03009 3.93455
2.76579 3.06880 3.22148 5.83873 4.68571 776 A - - -
2.68618 4.42225 2.77519 2.73123 3.46354 2.40513 3.72494
3.29354 2.67741 2.69355 4.24690 2.90347 2.73739 3.18146 2.89801
2.37887 2.77519 2.98518 4.58477 3.61503
0.02187 4.22952 4.95186 0.61958 0.77255 0.48576 0.95510
664 1.72636 4.42033 3.39978 2.92962 3.67899 3.40310 3.92977
3.16867 2.91057 2.86769 3.75222 3.30183 3.92566 3.23370 3.27403
2.06296 2.88024 2.88023 5.11479 2.41757 777 a - - -
2.68618 4.42225 2.77519 2.73123 3.46354 2.40513 3.72494
3.29354 2.67741 2.69355 4.24690 2.90347 2.73739 3.18146 2.89801
2.37887 2.77519 2.98518 4.58477 3.61503
0.02187 4.22952 4.95186 0.61958 0.77255 0.48576 0.95510
665 2.95094 5.22959 2.79784 2.51967 4.61620 3.48528 3.79353
4.08966 1.77398 3.60010 4.44210 1.41833 3.99364 2.94501 2.74189
2.92503 3.20562 3.71072 5.69390 4.36164 778 n - - -
2.68627 4.42234 2.77529 2.73132 3.46363 2.40522 3.72504
3.29363 2.67750 2.69364 4.24699 2.90242 2.73700 3.18156 2.89810
2.37896 2.77529 2.98528 4.58486 3.61512
0.20483 1.72517 4.95186 0.96361 0.48048 0.48576 0.95510
666 2.80934 5.08624 2.61010 1.56735 4.56428 3.34408 3.86152
4.02636 2.72525 3.59712 4.42894 2.94965 3.91834 3.02598 3.19545
1.54342 3.11816 3.61733 5.77436 4.38476 782 s - - -
2.68618 4.42225 2.77519 2.73123 3.46354 2.40513 3.72494
3.29354 2.67741 2.69355 4.24690 2.90347 2.73739 3.18146 2.89801
2.37887 2.77519 2.98518 4.58477 3.61503
0.02187 4.22952 4.95186 0.61958 0.77255 0.48576 0.95510
667 2.63962 4.80609 2.81781 2.56870 4.52131 2.11916 3.93271
3.99214 2.80592 3.56951 4.37930 1.76467 2.33274 3.10415 3.26118
2.69171 3.00023 3.52658 5.74999 4.40302 783 n - - -
2.68618 4.42225 2.77519 2.73123 3.46354 2.40513 3.72494
3.29354 2.67741 2.69355 4.24690 2.90347 2.73739 3.18146 2.89801
2.37887 2.77519 2.98518 4.58477 3.61503
0.02187 4.22952 4.95186 0.61958 0.77255 0.48576 0.95510
668 1.82774 4.55703 3.11315 2.78199 4.40987 3.19774 4.02223
3.84689 2.90222 3.47206 4.28238 2.07215 3.83856 3.21357 3.31930
1.60515 2.89476 3.36699 5.68593 4.38762 784 s - - -
2.68618 4.42225 2.77519 2.73123 3.46354 2.40513 3.72494
3.29354 2.67741 2.69355 4.24690 2.90347 2.73739 3.18146 2.89801
2.37887 2.77519 2.98518 4.58477 3.61503
0.02187 4.22952 4.95186 0.61958 0.77255 0.48576 0.95510
669 3.09682 5.31085 3.25024 2.74142 4.73463 3.68289 3.72071
4.12541 1.13311 3.55984 4.41744 3.19323 4.08852 2.03519 2.31682
3.07050 3.28377 3.78292 5.60931 4.38787 785 k - - -
2.68618 4.42225 2.77519 2.73123 3.46354 2.40513 3.72494
3.29354 2.67741 2.69355 4.24690 2.90347 2.73739 3.18146 2.89801
2.37887 2.77519 2.98518 4.58477 3.61503
0.02187 4.22952 4.95186 0.61958 0.77255 0.48576 0.95510
670 3.25990 4.52873 5.10955 4.62873 3.72790 4.69219 5.33080
0.98617 4.52526 2.19064 3.50863 4.81764 4.97720 4.77709 4.70171
4.10163 3.52618 1.27572 5.75776 4.55531 786 i - - -

```
         2.68618  4.42225  2.77519  2.73123  3.46354  2.40513  3.72494
3.29354  2.67741  2.69355  4.24690  2.90347  2.73739  3.18146  2.89801
2.37887  2.77519  2.98518  4.58477  3.61503
         0.02187  4.22952  4.95186  0.61958  0.77255  0.48576  0.95510
    671  2.69481  4.81955  2.94599  2.64016  4.36583  3.34341  3.89393
3.86160  2.65336  3.44292  4.28703  3.10090  3.91552  2.07183  3.03091
1.35379  3.02967  3.45631  5.60840  4.27208    787 s - - -
         2.68618  4.42225  2.77519  2.73123  3.46354  2.40513  3.72494
3.29354  2.67741  2.69355  4.24690  2.90347  2.73739  3.18146  2.89801
2.37887  2.77519  2.98518  4.58477  3.61503
         0.02187  4.22952  4.95186  0.61958  0.77255  0.48576  0.95510
    672  3.58296  4.94778  4.76280  4.41894  3.31381  4.43210  4.96505
2.55063  4.18445  0.53374  3.25075  4.66084  4.81524  4.46461  4.29594
4.06845  3.86586  2.67792  5.30656  4.10752    788 L - - -
         2.68618  4.42225  2.77519  2.73123  3.46354  2.40513  3.72494
3.29354  2.67741  2.69355  4.24690  2.90347  2.73739  3.18146  2.89801
2.37887  2.77519  2.98518  4.58477  3.61503
         0.02187  4.22952  4.95186  0.61958  0.77255  0.48576  0.95510
    673  2.62337  4.73071  2.86792  2.66887  4.49552  3.23310  4.02332
4.02734  2.91242  3.62863  4.45658  1.51350  3.88773  3.22134  3.33300
1.58782  3.02104  3.53835  5.76707  4.41152    789 n - - -
         2.68618  4.42225  2.77519  2.73123  3.46354  2.40513  3.72494
3.29354  2.67741  2.69355  4.24690  2.90347  2.73739  3.18146  2.89801
2.37887  2.77519  2.98518  4.58477  3.61503
         0.02187  4.22952  4.95186  0.61958  0.77255  0.48576  0.95510
    674  2.68485  4.51098  3.38776  2.82390  3.50020  3.59781  2.77838
2.30642  2.23163  2.71572  3.60711  3.25729  3.97834  3.07436  3.03018
2.84467  2.91226  2.79942  4.93564  2.58524    790 k - - -
         2.68618  4.42225  2.77519  2.73123  3.46354  2.40513  3.72494
3.29354  2.67741  2.69355  4.24690  2.90347  2.73739  3.18146  2.89801
2.37887  2.77519  2.98518  4.58477  3.61503
         0.15591  4.22952  2.04170  0.61958  0.77255  0.48576  0.95510
    675  2.83701  5.01301  2.95487  2.58217  4.38679  3.44958  3.73806
3.89025  2.31113  3.42202  4.27236  1.56078  3.93929  2.89924  1.98833
2.84982  3.09781  3.52721  5.50978  4.19907    791 n - - -
         2.68618  4.42225  2.77519  2.73123  3.46354  2.40513  3.72494
3.29354  2.67741  2.69355  4.24690  2.90347  2.73739  3.18146  2.89801
2.37887  2.77519  2.98518  4.58477  3.61503
         0.22086  4.09857  1.70611  0.61958  0.77255  0.40295  1.10367
    676  2.80150  4.73576  3.35613  2.79625  3.88886  3.59659  3.76113
3.18080  1.58706  2.78130  2.35283  3.22990  3.99751  2.96059  2.62716
2.90785  3.02846  2.97110  5.22857  3.97745    792 k - - -
         2.68618  4.42225  2.77519  2.73123  3.46354  2.40513  3.72494
3.29354  2.67741  2.69355  4.24690  2.90347  2.73739  3.18146  2.89801
2.37887  2.77519  2.98518  4.58477  3.61503
         0.02584  4.06439  4.78674  0.61958  0.77255  0.66695  0.72005
    677  2.63547  4.82604  2.93007  2.49812  4.28491  3.34714  3.70345
3.70902  1.84027  3.28089  4.09745  2.98564  2.61017  2.85450  2.78911
2.11530  2.91444  3.33242  5.47028  4.15133    793 k - - -
         2.68618  4.42225  2.77519  2.73123  3.46354  2.40513  3.72494
3.29354  2.67741  2.69355  4.24690  2.90347  2.73739  3.18146  2.89801
2.37887  2.77519  2.98518  4.58477  3.61503
         0.02584  4.06439  4.78674  0.61958  0.77255  0.66695  0.72005
```

```
    678   2.85155  5.11054  1.57579  2.33909  4.69111  1.53477  3.92473
 4.21305  2.94455  3.77425  4.63315  2.88353  3.88386  3.11599  3.48396
 2.82978  3.19963  3.76353  5.88329  4.50090    794 g - - -
          2.68618  4.42225  2.77519  2.73123  3.46354  2.40513  3.72494
 3.29354  2.67741  2.69355  4.24690  2.90347  2.73739  3.18146  2.89801
 2.37887  2.77519  2.98518  4.58477  3.61503
          0.02584  4.06439  4.78674  0.61958  0.77255  0.66695  0.72005
    679   2.50308  4.31623  3.44850  2.98672  3.69221  2.37538  3.96520
 2.94228  2.92476  1.94479  3.64868  3.32491  3.89422  3.26753  3.25630
 2.70618  2.15302  2.68388  5.14273  3.90126    795 l - - -
          2.68618  4.42225  2.77519  2.73123  3.46354  2.40513  3.72494
 3.29354  2.67741  2.69355  4.24690  2.90347  2.73739  3.18146  2.89801
 2.37887  2.77519  2.98518  4.58477  3.61503
          0.02584  4.06439  4.78674  0.61958  0.77255  0.66695  0.72005
    680   2.54678  4.60180  2.90889  2.64701  4.25373  3.20485  3.91680
 3.65101  2.76466  3.33475  4.19517  1.67449  3.82513  3.12176  3.15438
 2.64065  1.91401  3.25049  5.55413  4.23324    796 n - - -
          2.68618  4.42225  2.77519  2.73123  3.46354  2.40513  3.72494
 3.29354  2.67741  2.69355  4.24690  2.90347  2.73739  3.18146  2.89801
 2.37887  2.77519  2.98518  4.58477  3.61503
          0.02584  4.06439  4.78674  0.61958  0.77255  0.66695  0.72005
    681   2.85096  4.44100  3.81670  3.31782  1.98754  3.77884  3.69406
 2.95102  3.24689  2.57562  3.57537  3.57072  4.17142  3.48964  3.51790
 2.05528  3.09700  2.75711  4.19393  1.99064    797 f - - -
          2.68618  4.42225  2.77519  2.73123  3.46354  2.40513  3.72494
 3.29354  2.67741  2.69355  4.24690  2.90347  2.73739  3.18146  2.89801
 2.37887  2.77519  2.98518  4.58477  3.61503
          0.02584  4.06439  4.78674  0.61958  0.77255  0.38715  1.13628
    682   2.08547  4.81820  2.84760  2.02197  4.36564  3.31856  3.84675
 3.78396  2.69336  3.39175  4.21027  3.02080  3.86946  3.01254  3.14791
 1.59266  2.96547  3.38475  5.61379  4.27166    798 s - - -
          2.68618  4.42225  2.77519  2.73123  3.46354  2.40513  3.72494
 3.29354  2.67741  2.69355  4.24690  2.90347  2.73739  3.18146  2.89801
 2.37887  2.77519  2.98518  4.58477  3.61503
          0.02187  4.22952  4.95186  0.61958  0.77255  0.48576  0.95510
    683   2.83139  4.99562  3.10047  2.67798  4.51619  3.48103  3.79699
 3.93791  1.26328  3.47774  4.31591  3.13609  3.97657  2.94799  2.64019
 2.01212  3.10748  3.55573  5.60048  4.31912    799 k - - -
          2.68618  4.42225  2.77519  2.73123  3.46354  2.40513  3.72494
 3.29354  2.67741  2.69355  4.24690  2.90347  2.73739  3.18146  2.89801
 2.37887  2.77519  2.98518  4.58477  3.61503
          0.02187  4.22952  4.95186  0.61958  0.77255  0.48576  0.95510
    684   3.27627  4.58930  4.93826  4.42995  2.09381  4.51408  4.69605
 1.11202  4.29506  1.84609  3.20663  4.56264  4.77990  4.41787  4.39568
 3.88273  3.51760  2.10482  4.99402  3.62383    800 i - - -
          2.68618  4.42225  2.77519  2.73123  3.46354  2.40513  3.72494
 3.29354  2.67741  2.69355  4.24690  2.90347  2.73739  3.18146  2.89801
 2.37887  2.77519  2.98518  4.58477  3.61503
          0.02187  4.22952  4.95186  0.61958  0.77255  0.48576  0.95510
    685   2.64030  4.42066  2.61569  2.85023  2.57086  2.71443  3.84417
 2.23972  2.84716  2.62289  3.52937  3.28030  3.97204  3.15181  3.22468
 2.83295  2.88037  2.66085  4.98323  3.71046    801 i - - -
          2.68578  4.42231  2.77526  2.73129  3.46360  2.40519  3.72501
 3.29360  2.67747  2.69361  4.24696  2.90353  2.73746  3.18152  2.89807
 2.37859  2.77526  2.98524  4.58483  3.61509
```

```
         0.20483  1.72517  4.95186  0.66933  0.71754  0.48576  0.95510
    686  2.69871  4.79708  1.95726  2.54164  4.04782  3.45180  3.79246
3.33869  2.65319  2.28288  3.93341  3.05035  3.91398  2.97492  3.10642
2.76022  2.20583  3.05818  5.39128  4.06135    804 d - - -
         2.68618  4.42225  2.77519  2.73123  3.46354  2.40513  3.72494
3.29354  2.67741  2.69355  4.24690  2.90347  2.73739  3.18146  2.89801
2.37887  2.77519  2.98518  4.58477  3.61503
         0.02187  4.22952  4.95186  0.61958  0.77255  0.48576  0.95510
    687  2.75919  4.45014  3.87556  3.41928  3.60880  3.65529  4.28323
2.63343  3.26718  1.75811  3.55929  3.70273  4.17959  3.63564  3.53857
3.03810  1.41105  2.48426  5.24768  4.00927    805 t - - -
         2.68618  4.42225  2.77519  2.73123  3.46354  2.40513  3.72494
3.29354  2.67741  2.69355  4.24690  2.90347  2.73739  3.18146  2.89801
2.37887  2.77519  2.98518  4.58477  3.61503
         0.02187  4.22952  4.95186  0.61958  0.77255  0.48576  0.95510
    688  2.82822  5.23758  1.90702  2.30812  4.62335  3.34590  3.79570
4.09866  2.68944  3.63489  4.44259  1.70035  3.89479  2.94301  3.21610
2.78345  2.31171  3.68229  5.80882  4.37813    806 n - - -
         2.68618  4.42225  2.77519  2.73123  3.46354  2.40513  3.72494
3.29354  2.67741  2.69355  4.24690  2.90347  2.73739  3.18146  2.89801
2.37887  2.77519  2.98518  4.58477  3.61503
         0.02187  4.22952  4.95186  0.61958  0.77255  0.48576  0.95510
    689  3.32169  4.66102  4.81585  4.45732  3.64239  4.43292  5.13400
0.71984  4.29347  2.17033  3.53769  4.65564  4.84343  4.61411  4.45169
3.97643  3.61986  1.92373  5.59674  4.35691    807 i - - -
         2.68618  4.42225  2.77519  2.73123  3.46354  2.40513  3.72494
3.29354  2.67741  2.69355  4.24690  2.90347  2.73739  3.18146  2.89801
2.37887  2.77519  2.98518  4.58477  3.61503
         0.02187  4.22952  4.95186  0.61958  0.77255  0.48576  0.95510
    690  2.66837  4.85273  2.91799  2.54439  4.34730  3.35993  3.80440
3.79460  2.57534  3.37130  4.18851  3.03336  2.00593  2.12787  2.99253
2.14341  2.96930  3.40250  5.56722  4.23122    808 p - - -
         2.68618  4.42225  2.77519  2.73123  3.46354  2.40513  3.72494
3.29354  2.67741  2.69355  4.24690  2.90347  2.73739  3.18146  2.89801
2.37887  2.77519  2.98518  4.58477  3.61503
         0.02187  4.22952  4.95186  0.61958  0.77255  0.48576  0.95510
    691  2.73974  4.85322  3.05712  2.57879  4.10134  3.50787  3.73210
2.59557  2.04514  3.11187  3.96666  1.85971  3.92960  2.90200  2.82057
2.78922  2.97602  3.15861  5.36359  4.05350    809 n - - -
         2.68618  4.42225  2.77519  2.73123  3.46354  2.40513  3.72494
3.29354  2.67741  2.69355  4.24690  2.90347  2.73739  3.18146  2.89801
2.37887  2.77519  2.98518  4.58477  3.61503
         0.02187  4.22952  4.95186  0.61958  0.77255  0.48576  0.95510
    692  2.65101  4.49174  3.79694  3.58534  4.33601  3.31449  4.56869
3.51006  3.53575  3.36005  4.39880  3.72205  4.05177  3.91501  3.76596
2.85779  0.68492  3.16327  5.73940  4.55729    810 T - - -
         2.68618  4.42225  2.77519  2.73123  3.46354  2.40513  3.72494
3.29354  2.67741  2.69355  4.24690  2.90347  2.73739  3.18146  2.89801
2.37887  2.77519  2.98518  4.58477  3.61503
         0.02187  4.22952  4.95186  0.61958  0.77255  0.48576  0.95510
    693  2.60348  4.56999  3.32171  2.79674  3.96702  3.42082  3.85490
3.31815  2.63794  3.00579  3.87304  3.21430  3.91276  3.06805  2.31976
1.67054  2.90212  2.33393  5.30068  4.02961    811 s - - -
```

```
         2.68618  4.42225  2.77519  2.73123  3.46354  2.40513  3.72494
3.29354  2.67741  2.69355  4.24690  2.90347  2.73739  3.18146  2.89801
2.37887  2.77519  2.98518  4.58477  3.61503
         0.02187  4.22952  4.95186  0.61958  0.77255  0.48576  0.95510
    694  2.76387  4.90800  2.21121  2.46687  4.09106  3.44018  3.78464
3.51191  2.67032  1.81256  4.02597  2.32857  3.92165  2.97358  3.13485
2.79656  3.01894  3.21549  5.42253  4.06261    812 l - - -
         2.68618  4.42225  2.77519  2.73123  3.46354  2.40513  3.72494
3.29354  2.67741  2.69355  4.24690  2.90347  2.73739  3.18146  2.89801
2.37887  2.77519  2.98518  4.58477  3.61503
         0.02187  4.22952  4.95186  0.61958  0.77255  0.48576  0.95510
    695  2.22018  4.46633  2.49124  2.83283  2.69358  3.58282  3.84593
2.89939  2.82657  2.07466  3.54132  3.27329  3.98021  3.13698  3.21332
2.83911  2.89840  2.68400  5.01508  3.73370    813 l - - -
         2.68618  4.42225  2.77519  2.73123  3.46354  2.40513  3.72494
3.29354  2.67741  2.69355  4.24690  2.90347  2.73739  3.18146  2.89801
2.37887  2.77519  2.98518  4.58477  3.61503
         0.02187  4.22952  4.95186  0.61958  0.77255  0.48576  0.95510
    696  2.15860  4.88233  3.04690  2.57809  4.34404  3.42261  3.75578
3.74638  1.61736  3.32531  4.14369  3.06097  2.53682  2.90338  2.80857
2.73106  2.96939  3.37501  5.51964  4.20859    814 k - - -
         2.68618  4.42225  2.77519  2.73123  3.46354  2.40513  3.72494
3.29354  2.67741  2.69355  4.24690  2.90347  2.73739  3.18146  2.89801
2.37887  2.77519  2.98518  4.58477  3.61503
         0.02187  4.22952  4.95186  0.61958  0.77255  0.48576  0.95510
    697  2.95909  5.46280  1.49889  2.24520  4.85423  2.12279  3.86419
4.37606  2.84960  3.87327  4.69900  1.98503  3.93143  3.02299  3.43002
2.87362  3.25710  3.92645  6.02849  4.54233    815 d - - -
         2.68618  4.42225  2.77519  2.73123  3.46354  2.40513  3.72494
3.29354  2.67741  2.69355  4.24690  2.90347  2.73739  3.18146  2.89801
2.37887  2.77519  2.98518  4.58477  3.61503
         0.02187  4.22952  4.95186  0.61958  0.77255  0.48576  0.95510
    698  2.41509  4.34567  3.55433  3.29568  4.43768  1.91673  4.37816
3.80179  3.35441  3.53286  4.38609  3.46606  3.86650  3.65913  3.65574
2.59488  1.10059  3.29290  5.77662  4.57544    816 t - - -
         2.68618  4.42225  2.77519  2.73123  3.46354  2.40513  3.72494
3.29354  2.67741  2.69355  4.24690  2.90347  2.73739  3.18146  2.89801
2.37887  2.77519  2.98518  4.58477  3.61503
         0.02187  4.22952  4.95186  0.61958  0.77255  0.48576  0.95510
    699  2.55625  4.29553  3.91178  3.53061  3.84644  3.40564  4.38590
2.70921  3.46205  2.81611  3.82600  3.68518  4.05081  3.77056  3.71642
1.90890  2.96187  1.19479  5.38912  4.15145    817 v - - -
         2.68618  4.42225  2.77519  2.73123  3.46354  2.40513  3.72494
3.29354  2.67741  2.69355  4.24690  2.90347  2.73739  3.18146  2.89801
2.37887  2.77519  2.98518  4.58477  3.61503
         0.02187  4.22952  4.95186  0.61958  0.77255  0.48576  0.95510
    700  3.00566  5.33833  2.85434  1.85305  4.72522  3.54407  3.74701
4.13905  1.24261  3.61280  4.44832  3.03728  4.01059  2.88238  2.59900
2.95848  3.22639  3.76793  5.70274  4.40044    818 k - - -
         2.68618  4.42225  2.77519  2.73123  3.46354  2.40513  3.72494
3.29354  2.67741  2.69355  4.24690  2.90347  2.73739  3.18146  2.89801
2.37887  2.77519  2.98518  4.58477  3.61503
         0.02187  4.22952  4.95186  0.61958  0.77255  0.48576  0.95510
```

```
    701   2.97387  5.21750  1.64973  2.46263  4.85465  1.18384  4.06943
4.39423  3.11441  3.94810  4.81271  3.00488  3.99986  3.26697  3.65949
2.95432  3.33588  3.92514  6.03486  4.66155    819 g - - -
          2.68618  4.42225  2.77519  2.73123  3.46354  2.40513  3.72494
3.29354  2.67741  2.69355  4.24690  2.90347  2.73739  3.18146  2.89801
2.37887  2.77519  2.98518  4.58477  3.61503
          0.02187  4.22952  4.95186  0.61958  0.77255  0.48576  0.95510
    702   2.79485  5.17308  2.61209  1.63577  4.52430  3.38006  3.75171
3.97684  2.57829  3.52078  4.32575  2.11293  3.88816  2.89315  3.06424
2.76218  2.22644  3.58344  5.70063  4.30020    820 e - - -
          2.68618  4.42225  2.77519  2.73123  3.46354  2.40513  3.72494
3.29354  2.67741  2.69355  4.24690  2.90347  2.73739  3.18146  2.89801
2.37887  2.77519  2.98518  4.58477  3.61503
          0.02187  4.22952  4.95186  0.61958  0.77255  0.48576  0.95510
    703   3.05735  4.54979  4.21017  3.75765  2.70726  4.00934  3.95863
2.72228  3.60181  2.46510  3.60717  3.91492  4.41588  3.85800  3.79689
3.36353  3.32154  1.81905  4.36423  1.29476    821 y - - -
          2.68618  4.42225  2.77519  2.73123  3.46354  2.40513  3.72494
3.29354  2.67741  2.69355  4.24690  2.90347  2.73739  3.18146  2.89801
2.37887  2.77519  2.98518  4.58477  3.61503
          0.02187  4.22952  4.95186  0.61958  0.77255  0.48576  0.95510
    704   2.85747  4.94982  3.02366  2.74050  4.57582  1.30531  3.95998
4.02117  1.76584  3.58951  4.44470  3.19634  4.00644  3.13973  2.93497
2.90743  3.18288  3.61784  5.70040  4.44301    822 g - - -
          2.68618  4.42225  2.77519  2.73123  3.46354  2.40513  3.72494
3.29354  2.67741  2.69355  4.24690  2.90347  2.73739  3.18146  2.89801
2.37887  2.77519  2.98518  4.58477  3.61503
          0.20483  4.22952  1.76812  0.61958  0.77255  0.48576  0.95510
    705   2.64104  4.50296  3.29371  2.90175  3.22778  3.41800  3.80195
3.25248  2.87765  2.89799  3.83615  3.27059  3.94630  3.22705  3.22048
1.57459  2.96003  2.97373  4.75612  2.26677    823 s - - -
          2.68618  4.42225  2.77519  2.73123  3.46354  2.40513  3.72494
3.29354  2.67741  2.69355  4.24690  2.90347  2.73739  3.18146  2.89801
2.37887  2.77519  2.98518  4.58477  3.61503
          0.02620  4.05089  4.77324  0.61958  0.77255  0.38139  1.14858
    706   3.98789  5.12769  4.92363  4.68751  1.37439  4.65031  3.56786
3.63623  4.51386  2.90744  4.21157  4.28522  4.94301  4.38481  4.47528
4.03732  4.20332  3.57233  3.67794  0.84206    824 y - - -
          2.68618  4.42225  2.77519  2.73123  3.46354  2.40513  3.72494
3.29354  2.67741  2.69355  4.24690  2.90347  2.73739  3.18146  2.89801
2.37887  2.77519  2.98518  4.58477  3.61503
          0.02187  4.22952  4.95186  0.61958  0.77255  0.48576  0.95510
    707   2.71725  4.61496  3.35453  2.80181  3.84533  3.58724  3.82889
2.24494  1.82704  2.81880  3.73727  3.24816  3.99303  3.04929  2.92631
2.85704  2.25674  2.77433  5.21722  3.95863    825 k - - -
          2.68618  4.42225  2.77519  2.73123  3.46354  2.40513  3.72494
3.29354  2.67741  2.69355  4.24690  2.90347  2.73739  3.18146  2.89801
2.37887  2.77519  2.98518  4.58477  3.61503
          0.02187  4.22952  4.95186  0.61958  0.77255  0.48576  0.95510
    708   2.94638  4.33973  4.50949  3.94961  3.44991  4.16838  4.56099
1.70144  3.83851  1.71875  3.33161  4.17365  4.49991  4.06915  4.03042
3.48841  2.20552  1.60217  5.18067  3.99954    826 v - - -
          2.68618  4.42225  2.77519  2.73123  3.46354  2.40513  3.72494
3.29354  2.67741  2.69355  4.24690  2.90347  2.73739  3.18146  2.89801
2.37887  2.77519  2.98518  4.58477  3.61503
```

0.02187 4.22952 4.95186 0.61958 0.77255 0.48576 0.95510
709 3.14178 4.45224 4.93343 4.38437 2.17950 4.44540 4.79964
1.33318 4.25760 1.93616 3.23211 4.52747 4.71821 4.40262 4.36252
3.79349 3.38103 1.58346 5.19248 3.97404 827 i - - -
2.68618 4.42225 2.77519 2.73123 3.46354 2.40513 3.72494
3.29354 2.67741 2.69355 4.24690 2.90347 2.73739 3.18146 2.89801
2.37887 2.77519 2.98518 4.58477 3.61503
0.02187 4.22952 4.95186 0.61958 0.77255 0.48576 0.95510
710 3.31529 5.45321 2.70580 0.60645 4.85294 3.54554 4.18442
4.38266 3.11533 3.94788 4.92308 3.20387 4.17210 3.40321 3.54988
3.27009 3.62767 4.03530 5.95474 4.69895 828 E - - -
2.68618 4.42225 2.77519 2.73123 3.46354 2.40513 3.72494
3.29354 2.67741 2.69355 4.24690 2.90347 2.73739 3.18146 2.89801
2.37887 2.77519 2.98518 4.58477 3.61503
0.02187 4.22952 4.95186 0.61958 0.77255 0.48576 0.95510
711 2.50907 4.34868 3.72717 3.42369 4.11135 1.21848 4.39805
3.19589 3.42447 3.13773 4.10503 3.58782 3.96689 3.73368 3.70058
2.72695 2.95957 1.81953 5.56921 4.34532 829 g - - -
2.68618 4.42225 2.77519 2.73123 3.46354 2.40513 3.72494
3.29354 2.67741 2.69355 4.24690 2.90347 2.73739 3.18146 2.89801
2.37887 2.77519 2.98518 4.58477 3.61503
0.02187 4.22952 4.95186 0.61958 0.77255 0.48576 0.95510
712 3.29869 4.97961 3.94636 3.84728 4.78758 3.64774 4.88149
4.44313 3.93886 4.02289 5.09758 4.11130 0.36966 4.31567 4.13026
3.48053 3.77053 4.04412 5.85897 4.91765 830 P - - -
2.68618 4.42225 2.77519 2.73123 3.46354 2.40513 3.72494
3.29354 2.67741 2.69355 4.24690 2.90347 2.73739 3.18146 2.89801
2.37887 2.77519 2.98518 4.58477 3.61503
0.02187 4.22952 4.95186 0.61958 0.77255 0.48576 0.95510
713 2.84476 4.39171 4.19936 3.72500 3.63972 3.87586 4.51289
1.42201 3.59886 2.37944 3.53666 3.96413 4.35648 3.91886 3.84400
3.25227 1.71611 2.04394 5.33636 4.12065 831 i - - -
2.68621 4.42228 2.77522 2.73126 3.46357 2.40516 3.72497
3.29357 2.67744 2.69358 4.24693 2.90293 2.73743 3.18149 2.89804
2.37890 2.77522 2.98521 4.58480 3.61506
0.20483 1.72517 4.95186 0.25055 1.50674 0.48576 0.95510
714 3.14178 4.45224 4.93343 4.38437 2.17950 4.44540 4.79964
1.33318 4.25760 1.93616 3.23211 4.52747 4.71821 4.40262 4.36252
3.79349 3.38103 1.58346 5.19248 3.97404 833 i - - -
2.68618 4.42225 2.77519 2.73123 3.46354 2.40513 3.72494
3.29354 2.67741 2.69355 4.24690 2.90347 2.73739 3.18146 2.89801
2.37887 2.77519 2.98518 4.58477 3.61503
0.02187 4.22952 4.95186 0.61958 0.77255 0.48576 0.95510
715 2.93737 5.17827 3.28400 2.67916 4.59293 3.62834 3.67814
3.97932 1.49857 3.45133 4.27568 3.14295 2.46446 2.80370 1.86075
2.92750 3.13602 3.62500 5.54162 4.29440 834 k - - -
2.68618 4.42225 2.77519 2.73123 3.46354 2.40513 3.72494
3.29354 2.67741 2.69355 4.24690 2.90347 2.73739 3.18146 2.89801
2.37887 2.77519 2.98518 4.58477 3.61503
0.02187 4.22952 4.95186 0.61958 0.77255 0.48576 0.95510
716 3.36420 4.65821 5.10581 4.54089 2.09294 4.63490 4.84998
1.66531 4.40197 1.09260 2.97714 4.70126 4.81810 4.43643 4.45195
3.98388 3.58316 2.33073 5.08793 3.90113 835 l - - -

```
         2.68618  4.42225  2.77519  2.73123  3.46354  2.40513  3.72494
3.29354  2.67741  2.69355  4.24690  2.90347  2.73739  3.18146  2.89801
2.37887  2.77519  2.98518  4.58477  3.61503
         0.02187  4.22952  4.95186  0.61958  0.77255  0.48576  0.95510
    717  2.51079  4.57726  3.11122  2.76038  4.36618  3.22261  3.98838
3.79871  2.85013  3.42483  4.23944  2.27188  3.84479  3.17759  3.26349
1.47186  2.14950  3.34172  5.64260  4.34267    836 s - - -
         2.68618  4.42225  2.77519  2.73123  3.46354  2.40513  3.72494
3.29354  2.67741  2.69355  4.24690  2.90347  2.73739  3.18146  2.89801
2.37887  2.77519  2.98518  4.58477  3.61503
         0.02187  4.22952  4.95186  0.61958  0.77255  0.48576  0.95510
    718  2.75590  5.15452  2.84565  2.40061  4.49335  3.44823  3.66006
3.94468  1.98133  3.44624  4.22931  2.22504  2.63358  2.27056  2.75893
2.72596  2.98716  3.55013  5.58837  4.22293    837 k - - -
         2.68618  4.42225  2.77519  2.73123  3.46354  2.40513  3.72494
3.29354  2.67741  2.69355  4.24690  2.90347  2.73739  3.18146  2.89801
2.37887  2.77519  2.98518  4.58477  3.61503
         0.02187  4.22952  4.95186  0.61958  0.77255  0.48576  0.95510
    719  2.69040  4.75956  2.85649  2.73945  4.63244  1.19287  4.15142
4.17758  3.09935  3.78012  4.62433  1.89544  3.93477  3.37200  3.51391
2.77386  3.11651  3.65751  5.88200  4.56467    838 g - - -
         2.68618  4.42225  2.77519  2.73123  3.46354  2.40513  3.72494
3.29354  2.67741  2.69355  4.24690  2.90347  2.73739  3.18146  2.89801
2.37887  2.77519  2.98518  4.58477  3.61503
         0.02187  4.22952  4.95186  0.61958  0.77255  0.48576  0.95510
    720  1.97824  4.61909  3.16174  2.71778  4.12410  3.35175  3.87330
3.48837  2.68614  3.14984  4.00033  3.15290  3.88266  2.33065  3.07964
2.68558  1.75181  3.14219  5.43099  4.14399    839 t - - -
         2.68618  4.42225  2.77519  2.73123  3.46354  2.40513  3.72494
3.29354  2.67741  2.69355  4.24690  2.90347  2.73739  3.18146  2.89801
2.37887  2.77519  2.98518  4.58477  3.61503
         0.02187  4.22952  4.95186  0.61958  0.77255  0.48576  0.95510
    721  2.87956  4.93507  3.10831  2.68775  3.19902  3.61488  2.56932
3.59564  2.67694  3.13929  4.04994  1.85815  4.03739  3.04976  3.07650
2.91008  3.11549  3.31151  4.72236  2.19464    840 n - - -
         2.68618  4.42225  2.77519  2.73123  3.46354  2.40513  3.72494
3.29354  2.67741  2.69355  4.24690  2.90347  2.73739  3.18146  2.89801
2.37887  2.77519  2.98518  4.58477  3.61503
         0.02187  4.22952  4.95186  0.61958  0.77255  0.48576  0.95510
    722  2.77426  5.09397  2.16305  1.61312  4.35277  3.41322  3.73715
2.52406  2.58536  3.34736  4.17888  2.91011  3.89008  2.88485  3.08241
2.75830  3.02189  3.37526  5.59099  4.20418    841 e - - -
         2.68630  4.42237  2.77532  2.73136  3.46366  2.40525  3.72507
3.29282  2.67753  2.69275  4.24702  2.90359  2.73752  3.18083  2.89813
2.37899  2.77532  2.98531  4.58489  3.61515
         0.20483  1.72517  4.95186  1.19068  0.36242  0.48576  0.95510
    723  3.33020  4.63789  5.01012  4.45886  1.55749  4.56215  4.67997
1.80709  4.31644  1.35218  3.02932  4.59646  4.77287  4.37251  4.38076
3.90684  3.55185  2.35204  4.93446  3.62764    846 l - - -
         2.68618  4.42225  2.77519  2.73123  3.46354  2.40513  3.72494
3.29354  2.67741  2.69355  4.24690  2.90347  2.73739  3.18146  2.89801
2.37887  2.77519  2.98518  4.58477  3.61503
         0.02187  4.22952  4.95186  0.61958  0.77255  0.48576  0.95510
```

```
    724  2.66567  4.83245  3.07716  2.59500  4.30676  3.40811  3.76300
3.71247  1.71370  3.29946  4.11615  3.07059  3.88730  2.91440  2.83356
2.08299  2.16303  3.33995  5.49972  4.19113    847 k - - -
         2.68618  4.42225  2.77519  2.73123  3.46354  2.40513  3.72494
3.29354  2.67741  2.69355  4.24690  2.90347  2.73739  3.18146  2.89801
2.37887  2.77519  2.98518  4.58477  3.61503
         0.02187  4.22952  4.95186  0.61958  0.77255  0.48576  0.95510
    725  2.78867  4.38371  3.81772  3.28904  3.40551  3.81112  4.10948
1.96010  3.17991  1.63895  3.38069  2.42823  4.19210  3.50267  3.46889
3.10490  3.03134  2.33687  5.00060  3.75676    848 l - - -
         2.68618  4.42225  2.77519  2.73123  3.46354  2.40513  3.72494
3.29354  2.67741  2.69355  4.24690  2.90347  2.73739  3.18146  2.89801
2.37887  2.77519  2.98518  4.58477  3.61503
         0.02187  4.22952  4.95186  0.61958  0.77255  0.48576  0.95510
    726  2.70386  4.82902  2.92942  1.79433  4.07789  3.45176  3.75518
3.45885  2.57273  2.28289  3.95949  3.03111  3.90330  2.93186  3.00574
2.14770  2.95603  3.15776  5.38154  4.05290    849 e - - -
         2.68618  4.42225  2.77519  2.73123  3.46354  2.40513  3.72494
3.29354  2.67741  2.69355  4.24690  2.90347  2.73739  3.18146  2.89801
2.37887  2.77519  2.98518  4.58477  3.61503
         0.02187  4.22952  4.95186  0.61958  0.77255  0.48576  0.95510
    727  2.75880  5.08123  2.72617  2.40982  4.48112  3.38453  3.75327
3.95564  2.52787  3.49071  4.29400  1.88649  3.88758  2.11432  2.97402
2.07887  3.03017  3.55443  5.65118  4.27171    850 n - - -
         2.68618  4.42225  2.77519  2.73123  3.46354  2.40513  3.72494
3.29354  2.67741  2.69355  4.24690  2.90347  2.73739  3.18146  2.89801
2.37887  2.77519  2.98518  4.58477  3.61503
         0.02187  4.22952  4.95186  0.61958  0.77255  0.48576  0.95510
    728  2.70259  5.06455  2.79897  1.95192  4.38906  3.40936  3.67452
3.83801  2.44126  3.37649  4.16396  2.91574  3.85160  2.17595  2.90808
2.22390  2.38402  3.45419  5.55650  4.17931    851 e - - -
         2.68618  4.42225  2.77519  2.73123  3.46354  2.40513  3.72494
3.29354  2.67741  2.69355  4.24690  2.90347  2.73739  3.18146  2.89801
2.37887  2.77519  2.98518  4.58477  3.61503
         0.02187  4.22952  4.95186  0.61958  0.77255  0.48576  0.95510
    729  2.71703  4.81670  3.03295  2.56705  4.02626  3.49475  3.73666
2.47334  2.50103  3.05800  3.92193  2.28446  3.91938  1.98417  2.89956
2.77266  2.95648  3.12411  5.32618  4.00511    852 q - - -
         2.68618  4.42225  2.77519  2.73123  3.46354  2.40513  3.72494
3.29354  2.67741  2.69355  4.24690  2.90347  2.73739  3.18146  2.89801
2.37887  2.77519  2.98518  4.58477  3.61503
         0.02187  4.22952  4.95186  0.61958  0.77255  0.48576  0.95510
    730  2.93336  5.40382  1.72991  2.26259  4.81681  1.82548  3.86332
4.33249  2.83814  3.83823  4.66190  1.99573  3.92661  3.02224  3.40844
2.85905  3.23395  3.88527  5.99656  4.52295    853 d - - -
         2.68618  4.42225  2.77519  2.73123  3.46354  2.40513  3.72494
3.29354  2.67741  2.69355  4.24690  2.90347  2.73739  3.18146  2.89801
2.37887  2.77519  2.98518  4.58477  3.61503
         0.02187  4.22952  4.95186  0.61958  0.77255  0.48576  0.95510
    731  2.75061  5.14914  2.08785  2.35723  4.48840  3.40359  3.69653
3.93783  1.75739  3.46754  4.25664  2.90231  3.86817  2.82427  2.93227
2.72015  2.26462  3.54247  5.63399  4.24402    854 k - - -
         2.68618  4.42225  2.77519  2.73123  3.46354  2.40513  3.72494
3.29354  2.67741  2.69355  4.24690  2.90347  2.73739  3.18146  2.89801
2.37887  2.77519  2.98518  4.58477  3.61503
```

```
         0.02187  4.22952  4.95186  0.61958  0.77255  0.48576  0.95510
    732  3.52359  4.82808  4.93408  4.48241  1.18947  4.58547  4.30871
2.57117  4.32144  1.32349  3.19428  4.52062  4.82985  4.36655  4.38344
3.96777  3.75291  2.68792  4.48887  2.93045    855 f - - -
         2.68618  4.42225  2.77519  2.73123  3.46354  2.40513  3.72494
3.29354  2.67741  2.69355  4.24690  2.90347  2.73739  3.18146  2.89801
2.37887  2.77519  2.98518  4.58477  3.61503
         0.02187  4.22952  4.95186  0.61958  0.77255  0.48576  0.95510
    733  1.62435  4.60763  3.26547  2.77621  4.22240  3.32484  3.88623
3.62390  2.63242  3.25112  4.08063  3.18337  3.87372  3.06937  2.10993
2.08933  2.89873  3.23599  5.48373  4.20667    856 a - - -
         2.68618  4.42225  2.77519  2.73123  3.46354  2.40513  3.72494
3.29354  2.67741  2.69355  4.24690  2.90347  2.73739  3.18146  2.89801
2.37887  2.77519  2.98518  4.58477  3.61503
         0.02187  4.22952  4.95186  0.61958  0.77255  0.48576  0.95510
    734  3.29086  4.55416  5.13339  4.60351  3.50725  4.71165  5.18884
1.23698  4.49396  1.48746  3.28950  4.79795  4.93368  4.65836  4.62417
4.08797  3.53379  1.46832  5.53958  4.40713    857 i - - -
         2.68618  4.42225  2.77519  2.73123  3.46354  2.40513  3.72494
3.29354  2.67741  2.69355  4.24690  2.90347  2.73739  3.18146  2.89801
2.37887  2.77519  2.98518  4.58477  3.61503
         0.02187  4.22952  4.95186  0.61958  0.77255  0.48576  0.95510
    735  3.09053  5.63956  1.08924  2.22768  4.95668  3.32831  3.93007
4.53789  2.99284  4.03340  4.90029  1.86750  3.97744  3.10749  3.60759
2.97400  3.40075  4.08848  6.15336  4.62793    858 d - - -
         2.68618  4.42225  2.77519  2.73123  3.46354  2.40513  3.72494
3.29354  2.67741  2.69355  4.24690  2.90347  2.73739  3.18146  2.89801
2.37887  2.77519  2.98518  4.58477  3.61503
         0.02187  4.22952  4.95186  0.61958  0.77255  0.48576  0.95510
    736  2.73467  5.13567  2.88181  1.94673  4.47048  3.45084  3.64496
3.92066  2.32192  3.42260  4.20131  2.93500  3.86654  2.27518  2.26720
2.16011  2.96320  3.52619  5.56569  4.20116    859 e - - -
         2.68618  4.42225  2.77519  2.73123  3.46354  2.40513  3.72494
3.29354  2.67741  2.69355  4.24690  2.90347  2.73739  3.18146  2.89801
2.37887  2.77519  2.98518  4.58477  3.61503
         0.02187  4.22952  4.95186  0.61958  0.77255  0.48576  0.95510
    737  3.38021  4.65727  5.10421  4.58937  3.38552  4.73703  5.15796
1.06260  4.42930  1.37208  3.19076  4.80876  4.94841  4.60461  4.55482
4.12954  3.62248  1.94082  5.46521  4.32285    860 i - - -
         2.68618  4.42225  2.77519  2.73123  3.46354  2.40513  3.72494
3.29354  2.67741  2.69355  4.24690  2.90347  2.73739  3.18146  2.89801
2.37887  2.77519  2.98518  4.58477  3.61503
         0.02187  4.22952  4.95186  0.61958  0.77255  0.48576  0.95510
    738  2.87120  4.67096  3.26032  1.82345  3.82124  3.70052  4.05021
1.58436  2.93328  2.66887  3.73481  3.37222  4.14553  3.30176  3.30135
3.06076  3.12508  2.49958  5.34498  4.06596    861 i - - -
         2.68618  4.42225  2.77519  2.73123  3.46354  2.40513  3.72494
3.29354  2.67741  2.69355  4.24690  2.90347  2.73739  3.18146  2.89801
2.37887  2.77519  2.98518  4.58477  3.61503
         0.02187  4.22952  4.95186  0.61958  0.77255  0.48576  0.95510
    739  3.53669  4.83461  5.05410  4.55401  1.94845  4.69348  4.64571
2.43443  4.40229  0.81909  3.01582  4.68976  4.88022  4.43269  4.46107
4.07297  3.75780  2.60882  4.82259  3.41731    862 l - - -
```

2.68618 4.42225 2.77519 2.73123 3.46354 2.40513 3.72494
3.29354 2.67741 2.69355 4.24690 2.90347 2.73739 3.18146 2.89801
2.37887 2.77519 2.98518 4.58477 3.61503
0.02187 4.22952 4.95186 0.61958 0.77255 0.48576 0.95510
740 2.78394 5.15640 2.67635 1.67509 4.44353 3.40610 2.45608
3.93216 2.51086 3.46481 4.26729 2.90570 3.88599 2.85921 2.97697
2.11252 3.03360 3.54866 5.62350 4.22708 863 e - - -
2.68618 4.42225 2.77519 2.73123 3.46354 2.40513 3.72494
3.29354 2.67741 2.69355 4.24690 2.90347 2.73739 3.18146 2.89801
2.37887 2.77519 2.98518 4.58477 3.61503
0.02187 4.22952 4.95186 0.61958 0.77255 0.48576 0.95510
741 3.29869 4.97961 3.94636 3.84728 4.78758 3.64774 4.88149
4.44313 3.93886 4.02289 5.09758 4.11130 0.36966 4.31567 4.13026
3.48053 3.77053 4.04412 5.85897 4.91765 864 P - - -
2.68618 4.42225 2.77519 2.73123 3.46354 2.40513 3.72494
3.29354 2.67741 2.69355 4.24690 2.90347 2.73739 3.18146 2.89801
2.37887 2.77519 2.98518 4.58477 3.61503
0.02187 4.22952 4.95186 0.61958 0.77255 0.48576 0.95510
742 3.26023 4.52303 5.11223 4.58841 3.58072 4.69448 5.19865
1.42064 4.48445 1.65729 3.36683 4.78016 4.93231 4.67583 4.62991
4.07226 3.50695 1.14987 5.58635 4.42951 865 v - - -
2.68618 4.42225 2.77519 2.73123 3.46354 2.40513 3.72494
3.29354 2.67741 2.69355 4.24690 2.90347 2.73739 3.18146 2.89801
2.37887 2.77519 2.98518 4.58477 3.61503
0.02187 4.22952 4.95186 0.61958 0.77255 0.48576 0.95510
743 2.77065 5.16652 2.59833 1.91901 4.55722 2.47928 3.75321
4.02986 2.59945 3.55531 4.34878 2.10908 3.87327 2.89111 3.10547
2.07561 3.04200 3.61453 5.72795 4.31701 866 e - - -
2.68618 4.42225 2.77519 2.73123 3.46354 2.40513 3.72494
3.29354 2.67741 2.69355 4.24690 2.90347 2.73739 3.18146 2.89801
2.37887 2.77519 2.98518 4.58477 3.61503
0.19097 4.22952 1.83706 0.61958 0.77255 0.48576 0.95510
744 2.82261 5.16828 2.79714 2.41206 4.51820 3.44081 3.64060
3.95699 1.83454 3.44732 4.25988 2.09163 3.88767 2.15608 2.58506
2.78844 3.04757 3.58080 5.56549 4.23548 867 k - - -
2.68618 4.42225 2.77519 2.73123 3.46354 2.40513 3.72494
3.29354 2.67741 2.69355 4.24690 2.90347 2.73739 3.18146 2.89801
2.37887 2.77519 2.98518 4.58477 3.61503
0.02584 4.06439 4.78674 0.61958 0.77255 0.66695 0.72005
745 2.96409 5.46792 1.24189 2.15845 4.78535 3.24459 3.82734
4.35227 2.86266 3.86590 4.72691 1.96360 3.88089 3.00349 3.45532
2.86283 3.27067 3.91664 5.99100 4.48629 868 d - - -
2.68618 4.42225 2.77519 2.73123 3.46354 2.40513 3.72494
3.29354 2.67741 2.69355 4.24690 2.90347 2.73739 3.18146 2.89801
2.37887 2.77519 2.98518 4.58477 3.61503
0.02584 4.06439 4.78674 0.61958 0.77255 0.66695 0.72005
746 2.68105 4.74737 2.94325 2.07765 2.50406 3.45445 3.68854
3.32773 2.52939 2.93468 3.82233 3.01777 3.88008 2.30332 2.94552
2.74179 2.91760 3.05522 5.16129 3.79345 869 e - - -
2.68618 4.42225 2.77519 2.73123 3.46354 2.40513 3.72494
3.29354 2.67741 2.69355 4.24690 2.90347 2.73739 3.18146 2.89801
2.37887 2.77519 2.98518 4.58477 3.61503
0.02584 4.06439 4.78674 0.61958 0.77255 0.66695 0.72005

```
    747   3.12909  4.43480  4.86727  4.37590  3.61551  4.49307  5.08936
1.32702  4.26596  2.11528  3.41674  4.58664  4.80425  4.53058  4.46112
3.88649  3.39665  1.09000  5.59121  4.38342    870 v - - -
          2.68618  4.42225  2.77519  2.73123  3.46354  2.40513  3.72494
3.29354  2.67741  2.69355  4.24690  2.90347  2.73739  3.18146  2.89801
2.37887  2.77519  2.98518  4.58477  3.61503
          0.02584  4.06439  4.78674  0.61958  0.77255  0.66695  0.72005
    748   2.71244  4.45749  3.39170  3.00187  3.60125  3.57255  4.00971
1.65476  2.95218  2.60799  3.63966  2.18643  4.05528  3.32923  3.27738
2.93869  2.99985  2.42978  5.12970  3.81807    871 i - - -
          2.68618  4.42225  2.77519  2.73123  3.46354  2.40513  3.72494
3.29354  2.67741  2.69355  4.24690  2.90347  2.73739  3.18146  2.89801
2.37887  2.77519  2.98518  4.58477  3.61503
          0.02584  4.06439  4.78674  0.61958  0.77255  0.66695  0.72005
    749   1.61361  4.31143  3.98459  3.53320  3.62268  3.65291  4.37988
1.65056  3.43967  2.38943  3.52861  3.77380  4.18431  3.75626  3.70630
3.04038  3.02744  2.08106  5.28781  4.06492    872 a - - -
          2.68618  4.42225  2.77519  2.73123  3.46354  2.40513  3.72494
3.29354  2.67741  2.69355  4.24690  2.90347  2.73739  3.18146  2.89801
2.37887  2.77519  2.98518  4.58477  3.61503
          0.02584  4.06439  4.78674  0.61958  0.77255  0.66695  0.72005
    750   2.85354  4.74141  3.31402  2.85364  3.62488  3.63452  3.85727
3.04973  2.57349  1.82124  3.64143  3.29512  4.05676  1.79987  2.87329
2.97810  3.09566  2.92253  5.14338  3.82569    873 q - - -
          2.68618  4.42225  2.77519  2.73123  3.46354  2.40513  3.72494
3.29354  2.67741  2.69355  4.24690  2.90347  2.73739  3.18146  2.89801
2.37887  2.77519  2.98518  4.58477  3.61503
          0.02584  4.06439  4.78674  0.61958  0.77255  0.66695  0.72005
    751   2.95355  5.45585  1.34595  2.16101  4.77405  3.24700  3.81999
4.33683  2.84493  3.84970  4.70622  1.80784  3.87785  2.99390  3.43285
2.85463  3.25754  3.90201  5.97671  4.47577    874 d - - -
          2.68618  4.42225  2.77519  2.73123  3.46354  2.40513  3.72494
3.29354  2.67741  2.69355  4.24690  2.90347  2.73739  3.18146  2.89801
2.37887  2.77519  2.98518  4.58477  3.61503
          0.02584  4.06439  4.78674  0.61958  0.77255  0.66695  0.72005
    752   1.93943  5.07028  1.94331  2.27630  4.49434  3.26650  3.74951
3.95307  2.65311  3.51633  4.33250  2.12926  3.82489  2.90501  3.17192
2.70553  3.02332  3.54511  5.70703  4.29409    875 a - - -
          2.68954  4.42838  2.77455  2.73339  3.46292  2.40475  3.72717
3.28580  2.67432  2.69204  4.24648  2.89848  2.74280  3.18193  2.90115
2.38144  2.77305  2.98295  4.58170  3.61418
          0.37232  1.16839        *  4.59150  0.01019  0.00000        *
//
HMMER3/f [3.3 | Nov 2019]
NAME  CryM5
DESC  Cry toxin (IDOPS Model 5)
LENG  746
ALPH  amino
RF    no
MM    no
CONS  yes
CS    no
MAP   yes
DATE  Wed Feb  3 11:08:23 2021
NSEQ  9
```

```
EFFN  2.245605
CKSUM 2797034133
GA 201.0 201.0
STATS LOCAL MSV       -12.2947  0.69642
STATS LOCAL VITERBI   -13.4591  0.69642
STATS LOCAL FORWARD    -6.3490  0.69642
HMM          A        C        D        E        F        G        H
I        K        L        M        N        P        Q        R        S
T        V        W        Y
            m->m     m->i     m->d     i->m     i->i     d->m     d->d
  COMPO   2.62490  4.48636  2.92618  2.71006  3.30191  2.94019  3.78686
2.74232  2.66996  2.49630  3.69236  2.80565  3.32802  3.00362  3.10445
2.59438  2.70631  2.74686  4.65087  3.29955
          2.68622  4.42230  2.77524  2.73128  3.46358  2.40517  3.72499
3.29358  2.67745  2.69359  4.24368  2.90351  2.73744  3.18151  2.89805
2.37891  2.77524  2.98523  4.58481  3.61508
          0.28860  1.82767  2.40904  0.19372  1.73662  0.00000        *
      1   2.84738  4.71087  2.57978  2.96874  3.93096  3.66988  4.09292
3.19651  3.04770  2.91017  1.27135  3.41335  4.14850  3.35750  3.43854
3.00558  2.48667  2.96703  5.38029  4.11361      2 m - - -
          2.68618  4.42225  2.77519  2.73123  3.46354  2.40513  3.72494
3.29354  2.67741  2.69355  4.24690  2.90347  2.73739  3.18146  2.89801
2.37887  2.77519  2.98518  4.58477  3.61503
          0.01477  4.61851  5.34086  0.61958  0.77255  0.39462  1.12067
      2   1.59059  2.58302  2.71683  3.05498  3.78077  3.68440  4.03665
3.12336  3.02130  2.84529  2.80258  3.46304  4.11422  3.33144  3.39497
2.95068  2.52277  2.87608  5.20649  3.96243      3 a - - -
          2.68618  4.42225  2.77519  2.73123  3.46354  2.40513  3.72494
3.29354  2.67741  2.69355  4.24690  2.90347  2.73739  3.18146  2.89801
2.37887  2.77519  2.98518  4.58477  3.61503
          0.01350  4.70791  5.43026  0.61958  0.77255  0.48576  0.95510
      3   2.78419  5.22930  2.53065  2.48830  4.54777  3.55458  3.75298
3.04071  2.18122  3.52268  4.27902  1.99979  3.95403  2.47561  2.98697
2.75781  2.23889  3.60578  5.67764  4.28283      4 n - - -
          2.68618  4.42225  2.77519  2.73123  3.46354  2.40513  3.72494
3.29354  2.67741  2.69355  4.24690  2.90347  2.73739  3.18146  2.89801
2.37887  2.77519  2.98518  4.58477  3.61503
          0.01350  4.70791  5.43026  0.61958  0.77255  0.48576  0.95510
      4   2.85962  4.35402  4.29228  3.72722  3.44143  3.94149  4.30176
1.95805  3.60179  1.27544  3.42325  3.94294  3.05317  3.83942  3.79326
2.51170  3.10624  2.50877  4.99864  3.80888      5 l - - -
          2.68618  4.42225  2.77519  2.73123  3.46354  2.40513  3.72494
3.29354  2.67741  2.69355  4.24690  2.90347  2.73739  3.18146  2.89801
2.37887  2.77519  2.98518  4.58477  3.61503
          0.01350  4.70791  5.43026  0.61958  0.77255  0.48576  0.95510
      5   2.79341  5.13577  3.06948  2.54478  4.42728  3.57382  3.78848
3.87916  2.55265  3.42826  4.21041  1.98765  3.97914  2.19456  3.02628
2.05751  2.57775  3.50588  5.61529  3.06791      6 n - - -
          2.68618  4.42225  2.77519  2.73123  3.46354  2.40513  3.72494
3.29354  2.67741  2.69355  4.24690  2.90347  2.73739  3.18146  2.89801
2.37887  2.77519  2.98518  4.58477  3.61503
          0.01350  4.70791  5.43026  0.61958  0.77255  0.48576  0.95510
      6   2.42710  5.28379  2.47103  1.73507  4.60696  3.55357  3.77663
3.10295  2.53987  3.58138  4.33666  2.15562  3.96940  2.88473  3.03597
2.39131  3.05109  3.66355  5.73155  4.32600      7 e - - -
```

```
         2.68618  4.42225  2.77519  2.73123  3.46354  2.40513  3.72494
3.29354  2.67741  2.69355  4.24690  2.90347  2.73739  3.18146  2.89801
2.37887  2.77519  2.98518  4.58477  3.61503
         0.01350  4.70791  5.43026  0.61958  0.77255  0.48576  0.95510
      7  2.82435  4.35079  4.10721  3.53013  3.45042  3.90010  4.19538
1.96303  3.42633  1.70817  3.43559  3.80885  4.26986  2.40375  3.66419
2.51729  3.05768  2.12970  4.97183  3.77293      8 l - - -
         2.68618  4.42225  2.77519  2.73123  3.46354  2.40513  3.72494
3.29354  2.67741  2.69355  4.24690  2.90347  2.73739  3.18146  2.89801
2.37887  2.77519  2.98518  4.58477  3.61503
         0.01350  4.70791  5.43026  0.61958  0.77255  0.48576  0.95510
      8  2.88346  4.88334  3.30687  2.84660  3.91114  3.68775  3.95223
3.53480  2.82609  3.17167  4.03744  3.32026  2.98175  2.56553  3.22691
2.37166  3.13619  3.24956  5.30835  1.40384      9 y - - -
         2.68620  4.42227  2.77522  2.73126  3.46356  2.40515  3.72497
3.29356  2.67743  2.69357  4.24692  2.90349  2.73703  3.18149  2.89803
2.37889  2.77522  2.98521  4.58479  3.61505
         0.10075  2.39187  5.43026  0.28170  1.40447  0.48576  0.95510
      9  2.79483  4.87138  3.29633  2.85424  4.33095  3.56165  4.04424
3.75501  2.88302  2.63805  4.21696  2.58283  1.50830  3.22950  3.30872
1.93938  3.10473  3.39805  5.63244  4.32151     11 p - - -
         2.68618  4.42225  2.77519  2.73123  3.46354  2.40513  3.72494
3.29354  2.67741  2.69355  4.24690  2.90347  2.73739  3.18146  2.89801
2.37887  2.77519  2.98518  4.58477  3.61503
         0.10417  4.70791  2.40904  0.61958  0.77255  0.48576  0.95510
     10  3.15093  4.51406  4.93239  4.41311  3.81111  4.40460  4.96935
1.13842  4.29108  2.56474  3.69348  4.54583  4.78589  4.52738  4.44539
2.70262  3.42191  1.23227  5.54515  4.33818     12 i - - -
         2.68618  4.42225  2.77519  2.73123  3.46354  2.40513  3.72494
3.29354  2.67741  2.69355  4.24690  2.90347  2.73739  3.18146  2.89801
2.37887  2.77519  2.98518  4.58477  3.61503
         0.01477  4.61851  5.34086  0.61958  0.77255  0.39462  1.12067
     11  2.82098  5.05959  3.11077  2.61268  4.25763  3.60415  2.88488
3.75934  2.63079  3.34241  4.15266  2.53480  1.74900  2.98591  3.09145
2.35677  3.05959  3.41969  5.52356  3.09944     13 p - - -
         2.68618  4.42225  2.77519  2.73123  3.46354  2.40513  3.72494
3.29354  2.67741  2.69355  4.24690  2.90347  2.73739  3.18146  2.89801
2.37887  2.77519  2.98518  4.58477  3.61503
         0.01350  4.70791  5.43026  0.61958  0.77255  0.48576  0.95510
     12  3.71301  5.39394  3.94279  3.63826  2.68820  4.35565  2.63234
4.06323  3.59559  3.46090  4.56830  2.47313  4.76665  3.90693  3.88076
3.73240  3.94785  3.86576  4.33040  0.74383     14 y - - -
         2.68618  4.42225  2.77519  2.73123  3.46354  2.40513  3.72494
3.29354  2.67741  2.69355  4.24690  2.90347  2.73739  3.18146  2.89801
2.37887  2.77519  2.98518  4.58477  3.61503
         0.01350  4.70791  5.43026  0.61958  0.77255  0.48576  0.95510
     13  3.72931  5.61857  3.58967  3.58841  5.15475  4.01520  4.98732
5.14398  4.02545  4.68902  5.69573  0.27417  4.72713  4.32625  4.35574
3.80131  4.17075  4.66430  6.33456  5.09264     15 N - - -
         2.68618  4.42225  2.77519  2.73123  3.46354  2.40513  3.72494
3.29354  2.67741  2.69355  4.24690  2.90347  2.73739  3.18146  2.89801
2.37887  2.77519  2.98518  4.58477  3.61503
         0.01350  4.70791  5.43026  0.61958  0.77255  0.48576  0.95510
```

```
     14   3.16068  4.67147  4.95782  4.59497  4.21019  4.20090  5.31494
2.29450  4.47230  2.85724  4.05058  4.61229  4.79322  4.76721  4.64158
3.65682  2.45192  0.59708  6.02398  4.80877      16 V - - -
          2.68618  4.42225  2.77519  2.73123  3.46354  2.40513  3.72494
3.29354  2.67741  2.69355  4.24690  2.90347  2.73739  3.18146  2.89801
2.37887  2.77519  2.98518  4.58477  3.61503
          0.01350  4.70791  5.43026  0.61958  0.77255  0.48576  0.95510
     15   3.87425  5.14135  5.65805  5.15486  3.49863  5.16809  5.60961
2.47642  4.97540  0.50938  3.27090  5.35007  5.32777  5.00242  5.00668
4.60035  4.11696  2.02016  5.70598  4.66590      17 L - - -
          2.68618  4.42225  2.77519  2.73123  3.46354  2.40513  3.72494
3.29354  2.67741  2.69355  4.24690  2.90347  2.73739  3.18146  2.89801
2.37887  2.77519  2.98518  4.58477  3.61503
          0.01350  4.70791  5.43026  0.61958  0.77255  0.48576  0.95510
     16   1.18630  4.53439  4.02665  3.56989  4.18547  3.53158  4.48449
2.39992  3.52401  3.23379  4.13569  3.76849  4.17704  3.81338  3.83044
1.52228  3.11404  3.07849  5.62857  4.41432      18 a - - -
          2.68618  4.42225  2.77519  2.73123  3.46354  2.40513  3.72494
3.29354  2.67741  2.69355  4.24690  2.90347  2.73739  3.18146  2.89801
2.37887  2.77519  2.98518  4.58477  3.61503
          0.01350  4.70791  5.43026  0.61958  0.77255  0.48576  0.95510
     17   2.76887  4.99116  2.60641  2.59641  4.23566  2.91131  2.60071
2.92314  2.59392  3.25360  4.06169  3.11881  3.98474  2.94393  3.06053
2.08561  2.49426  3.32745  5.49030  3.08874      19 s - - -
          2.68628  4.42235  2.77485  2.73133  3.46364  2.40523  3.72505
3.29364  2.67751  2.69365  4.24700  2.90357  2.73750  3.18093  2.89811
2.37897  2.77489  2.98483  4.58487  3.61513
          0.56346  1.07628  2.40904  0.10710  2.28709  0.48576  0.95510
     18   2.14392  4.65393  3.58968  3.18473  3.89575  3.58060  4.18764
3.47417  3.17526  3.15960  4.06399  3.53614  1.18400  3.51069  3.52216
2.95329  3.13035  3.17240  5.34956  2.63932      21 p - - -
          2.68618  4.42225  2.77519  2.73123  3.46354  2.40513  3.72494
3.29354  2.67741  2.69355  4.24690  2.90347  2.73739  3.18146  2.89801
2.37887  2.77519  2.98518  4.58477  3.61503
          0.01477  4.61851  5.34086  0.61958  0.77255  0.65133  0.73679
     19   2.74890  4.30726  3.95319  2.61864  2.23099  3.80567  4.09244
2.06934  3.29646  2.49672  3.41687  3.68281  2.98495  3.55673  3.55868
3.07646  2.49491  2.18470  4.91705  3.71080      22 i - - -
          2.68618  4.42225  2.77519  2.73123  3.46354  2.40513  3.72494
3.29354  2.67741  2.69355  4.24690  2.90347  2.73739  3.18146  2.89801
2.37887  2.77519  2.98518  4.58477  3.61503
          0.01477  4.61851  5.34086  0.61958  0.77255  0.65133  0.73679
     20   2.82953  5.20566  3.13225  2.56169  4.52976  3.59921  3.74866
3.97434  1.85278  3.48364  4.26069  3.08603  3.98620  2.36499  2.40439
1.85477  3.05167  3.58899  5.62999  3.01402      23 k - - -
          2.68618  4.42225  2.77519  2.73123  3.46354  2.40513  3.72494
3.29354  2.67741  2.69355  4.24690  2.90347  2.73739  3.18146  2.89801
2.37887  2.77519  2.98518  4.58477  3.61503
          0.01477  4.61851  5.34086  0.61958  0.77255  0.65133  0.73679
     21   2.75348  5.12758  3.04354  2.21955  3.15031  3.54124  3.73287
3.87238  2.13965  2.80513  4.18293  2.25411  3.93540  2.09333  2.95148
2.74258  2.98394  3.49163  5.58719  4.21278      24 q - - -
          2.68618  4.42225  2.77519  2.73123  3.46354  2.40513  3.72494
3.29354  2.67741  2.69355  4.24690  2.90347  2.73739  3.18146  2.89801
2.37887  2.77519  2.98518  4.58477  3.61503
```

0.18807 4.61851 1.82276 0.61958 0.77255 0.65133 0.73679
22 2.71100 4.23620 4.00727 3.43276 2.53501 3.78418 4.08265
2.61809 3.33587 1.74496 3.33016 2.72674 4.15825 3.58440 3.56991
3.06476 2.43023 2.08044 4.84466 3.63580 25 l - - -
2.68618 4.42225 2.77519 2.73123 3.46354 2.40513 3.72494
3.29354 2.67741 2.69355 4.24690 2.90347 2.73739 3.18146 2.89801
2.37887 2.77519 2.98518 4.58477 3.61503
0.12525 4.44798 2.24410 0.61958 0.77255 0.62241 0.76927
23 2.27293 5.20261 2.29424 2.40515 4.55048 2.71805 3.73375
4.02352 2.51782 3.53175 4.29681 2.95369 2.83919 1.96712 3.01597
2.72911 3.00670 3.60622 5.68896 4.28366 26 q - - -
2.68588 4.42271 2.77440 2.73169 3.46400 2.40505 3.72540
3.29338 2.67786 2.69362 4.24735 2.90344 2.73709 3.18131 2.89846
2.37908 2.77436 2.98512 4.58523 3.61549
1.10656 0.60300 2.10261 1.21799 0.35073 0.86748 0.54474
24 2.09827 4.60731 3.20974 2.75890 4.02925 1.98245 3.90490
3.41079 2.78347 2.33346 3.93444 2.46102 3.92216 3.11778 3.19558
2.72735 2.92158 3.08718 5.37440 4.08896 33 g - - -
2.68618 4.42225 2.77519 2.73123 3.46354 2.40513 3.72494
3.29354 2.67741 2.69355 4.24690 2.90347 2.73739 3.18146 2.89801
2.37887 2.77519 2.98518 4.58477 3.61503
0.01966 4.33461 5.05695 0.61958 0.77255 0.71957 0.66741
25 2.29112 4.33664 3.71209 2.50554 2.17981 3.70934 3.98978
2.28447 3.09245 2.52700 3.44457 3.51401 4.09129 3.37879 3.40724
2.97154 2.40050 2.55555 4.94258 3.72085 34 f - - -
2.68618 4.42225 2.77519 2.73123 3.46354 2.40513 3.72494
3.29354 2.67741 2.69355 4.24690 2.90347 2.73739 3.18146 2.89801
2.37887 2.77519 2.98518 4.58477 3.61503
0.14158 4.45849 2.11666 0.61958 0.77255 0.46824 0.98378
26 2.70683 4.89096 2.61142 2.56022 4.13438 2.84196 3.75270
2.74222 2.55972 3.15698 3.97297 2.53028 2.95770 2.90681 3.02194
2.74606 2.44953 2.70031 5.40652 4.07465 35 t - - -
2.68618 4.42225 2.77519 2.73123 3.46354 2.40513 3.72494
3.29354 2.67741 2.69355 4.24690 2.90347 2.73739 3.18146 2.89801
2.37887 2.77519 2.98518 4.58477 3.61503
0.01622 4.52538 5.24773 0.61958 0.77255 0.78682 0.60750
27 2.25943 5.20697 2.30234 2.48401 4.67346 3.46106 3.87757
4.14924 2.71679 3.67325 4.45428 1.98703 3.97678 3.01048 3.22423
1.61958 3.11731 3.71242 5.84012 4.43387 36 s - - -
2.68618 4.42225 2.77519 2.73123 3.46354 2.40513 3.72494
3.29354 2.67741 2.69355 4.24690 2.90347 2.73739 3.18146 2.89801
2.37887 2.77519 2.98518 4.58477 3.61503
0.01622 4.52538 5.24773 0.61958 0.77255 0.78682 0.60750
28 2.71492 5.07610 2.22783 2.46284 3.19739 3.50610 3.70401
3.81135 2.12458 3.35583 4.13313 2.99979 2.93911 2.43297 2.94759
2.70746 2.94653 3.43585 5.53900 3.03114 37 k - - -
2.68618 4.42225 2.77519 2.73123 3.46354 2.40513 3.72494
3.29354 2.67741 2.69355 4.24690 2.90347 2.73739 3.18146 2.89801
2.37887 2.77519 2.98518 4.58477 3.61503
0.01622 4.52538 5.24773 0.61958 0.77255 0.51971 0.90311
29 2.30435 4.38642 3.80509 3.23707 2.37291 3.77400 4.04791
2.84907 2.51647 1.67482 3.48490 3.58937 3.08882 3.44917 3.45684
3.03754 2.99251 2.64358 4.97765 3.75741 38 l - - -

```
         2.68618  4.42225  2.77519  2.73123  3.46354  2.40513  3.72494
3.29354  2.67741  2.69355  4.24690  2.90347  2.73739  3.18146  2.89801
2.37887  2.77519  2.98518  4.58477  3.61503
         0.01464  4.62738  5.34973  0.61958  0.77255  0.40178  1.10603
     30  2.81396  5.21736  2.22947  2.50969  4.52840  3.56119  3.79507
3.99122  2.56921  3.51868  4.28921  2.18476  3.98006  2.91249  3.05956
2.04706  2.18497  3.59603  5.69011  3.19967     39 s - - -
         2.68618  4.42225  2.77519  2.73123  3.46354  2.40513  3.72494
3.29354  2.67741  2.69355  4.24690  2.90347  2.73739  3.18146  2.89801
2.37887  2.77519  2.98518  4.58477  3.61503
         0.01350  4.70791  5.43026  0.61958  0.77255  0.48576  0.95510
     31  2.12389  5.27668  2.51351  2.47162  4.61443  3.54750  3.74879
4.09417  1.92160  3.57997  4.32480  3.01938  3.08165  2.13853  2.98184
2.41710  3.02135  3.66456  5.71699  4.31052     40 k - - -
         2.68618  4.42225  2.77519  2.73123  3.46354  2.40513  3.72494
3.29354  2.67741  2.69355  4.24690  2.90347  2.73739  3.18146  2.89801
2.37887  2.77519  2.98518  4.58477  3.61503
         0.01350  4.70791  5.43026  0.61958  0.77255  0.48576  0.95510
     32  2.97671  4.49861  4.18403  3.62627  1.80997  4.00730  4.05756
2.97382  2.59355  2.22717  3.61539  2.75434  4.37218  3.75909  3.75772
3.28608  3.20680  2.77780  3.10640  2.05113     41 f - - -
         2.68618  4.42225  2.77519  2.73123  3.46354  2.40513  3.72494
3.29354  2.67741  2.69355  4.24690  2.90347  2.73739  3.18146  2.89801
2.37887  2.77519  2.98518  4.58477  3.61503
         0.01350  4.70791  5.43026  0.61958  0.77255  0.48576  0.95510
     33  2.79660  5.27214  2.48367  1.75154  4.59996  3.55037  3.75788
4.07598  2.50892  3.57027  4.32041  2.49962  3.95666  2.86247  2.51032
2.41982  3.02983  2.50800  5.71483  4.31016     42 e - - -
         2.68618  4.42225  2.77519  2.73123  3.46354  2.40513  3.72494
3.29354  2.67741  2.69355  4.24690  2.90347  2.73739  3.18146  2.89801
2.37887  2.77519  2.98518  4.58477  3.61503
         0.01350  4.70791  5.43026  0.61958  0.77255  0.48576  0.95510
     34  2.90411  5.42735  2.04911  1.86022  4.75339  3.56191  3.83345
4.24615  2.62367  3.72486  4.48071  2.53889  4.01314  1.84730  3.13404
2.85024  3.14479  3.81037  5.86035  2.97102     43 q - - -
         2.68618  4.42225  2.77519  2.73123  3.46354  2.40513  3.72494
3.29354  2.67741  2.69355  4.24690  2.90347  2.73739  3.18146  2.89801
2.37887  2.77519  2.98518  4.58477  3.61503
         0.01350  4.70791  5.43026  0.61958  0.77255  0.48576  0.95510
     35  2.82747  4.36634  4.15438  3.58621  3.46852  3.88052  4.23782
2.75964  3.47421  1.45026  2.62555  3.84211  4.27785  3.72991  3.70183
2.51855  1.90837  2.58312  5.00396  3.81008     44 l - - -
         2.68618  4.42225  2.77519  2.73123  3.46354  2.40513  3.72494
3.29354  2.67741  2.69355  4.24690  2.90347  2.73739  3.18146  2.89801
2.37887  2.77519  2.98518  4.58477  3.61503
         0.01350  4.70791  5.43026  0.61958  0.77255  0.48576  0.95510
     36  2.48565  4.51609  5.08154  4.49359  2.63979  4.43019  4.78792
1.67451  4.32490  1.21389  3.34334  4.56268  4.73085  4.44095  4.36784
3.75888  3.41517  1.95634  5.21205  4.07030     45 l - - -
         2.68618  4.42225  2.77519  2.73123  3.46354  2.40513  3.72494
3.29354  2.67741  2.69355  4.24690  2.90347  2.73739  3.18146  2.89801
2.37887  2.77519  2.98518  4.58477  3.61503
         0.01350  4.70791  5.43026  0.61958  0.77255  0.48576  0.95510
```

```
     37   3.03472  5.57652  2.21474  2.06815  4.90679  2.71114  3.92153
4.40266  1.19054  3.87217  4.64610  3.05038  4.08674  3.04367  3.23601
2.96375  3.28286  3.96418  6.00327  4.56339      46 k - - -
          2.68618  4.42225  2.77519  2.73123  3.46354  2.40513  3.72494
3.29354  2.67741  2.69355  4.24690  2.90347  2.73739  3.18146  2.89801
2.37887  2.77519  2.98518  4.58477  3.61503
          0.01350  4.70791  5.43026  0.61958  0.77255  0.48576  0.95510
     38   2.36890  5.45195  1.80817  2.13495  4.78260  2.33089  3.84454
4.27706  2.19481  3.75207  4.50784  2.46380  4.02015  2.95630  3.15686
2.86137  3.16100  3.83703  5.88638  4.45240      47 d - - -
          2.68618  4.42225  2.77519  2.73123  3.46354  2.40513  3.72494
3.29354  2.67741  2.69355  4.24690  2.90347  2.73739  3.18146  2.89801
2.37887  2.77519  2.98518  4.58477  3.61503
          0.01350  4.70791  5.43026  0.61958  0.77255  0.48576  0.95510
     39   3.96142  5.17803  5.83091  5.26159  1.87683  5.30047  5.44217
2.11333  5.11889  0.66933  3.14597  5.41996  5.33945  4.97634  5.05982
4.68510  4.16194  2.84061  5.46341  4.34273      48 L - - -
          2.68618  4.42225  2.77519  2.73123  3.46354  2.40513  3.72494
3.29354  2.67741  2.69355  4.24690  2.90347  2.73739  3.18146  2.89801
2.37887  2.77519  2.98518  4.58477  3.61503
          0.01350  4.70791  5.43026  0.61958  0.77255  0.48576  0.95510
     40   2.92348  5.38089  3.04843  1.67233  4.73734  3.62946  3.81172
4.19792  1.52169  3.66894  4.43458  3.10066  4.04261  2.47592  2.88812
2.88302  2.50139  3.78084  5.78616  4.41242      49 k - - -
          2.68618  4.42225  2.77519  2.73123  3.46354  2.40513  3.72494
3.29354  2.67741  2.69355  4.24690  2.90347  2.73739  3.18146  2.89801
2.37887  2.77519  2.98518  4.58477  3.61503
          0.01350  4.70791  5.43026  0.61958  0.77255  0.48576  0.95510
     41   2.89120  5.29230  3.06215  1.75498  4.62039  2.85577  3.81261
4.06885  1.50350  2.71805  4.35457  3.10396  4.03137  2.92853  2.92950
2.86557  3.11991  3.67601  5.72611  4.35856      50 k - - -
          2.68618  4.42225  2.77519  2.73123  3.46354  2.40513  3.72494
3.29354  2.67741  2.69355  4.24690  2.90347  2.73739  3.18146  2.89801
2.37887  2.77519  2.98518  4.58477  3.61503
          0.01350  4.70791  5.43026  0.61958  0.77255  0.48576  0.95510
     42   1.85000  4.75986  3.37574  2.83174  4.03899  2.05078  3.94152
2.67634  2.32076  3.08343  3.93421  3.29839  4.05631  3.14787  3.22160
2.87434  2.47300  3.12258  5.38603  4.10137      51 a - - -
          2.68618  4.42225  2.77519  2.73123  3.46354  2.40513  3.72494
3.29354  2.67741  2.69355  4.24690  2.90347  2.73739  3.18146  2.89801
2.37887  2.77519  2.98518  4.58477  3.61503
          0.01350  4.70791  5.43026  0.61958  0.77255  0.48576  0.95510
     43   3.50385  4.83226  5.18763  4.62967  3.16639  4.67570  4.72916
2.12342  4.41209  1.38909  3.32075  4.71178  4.92638  4.51686  4.46474
4.01595  3.72635  2.74161  1.21201  3.63903      52 w - - -
          2.68618  4.42225  2.77519  2.73123  3.46354  2.40513  3.72494
3.29354  2.67741  2.69355  4.24690  2.90347  2.73739  3.18146  2.89801
2.37887  2.77519  2.98518  4.58477  3.61503
          0.11004  4.70791  2.35204  0.61958  0.77255  0.48576  0.95510
     44   2.90371  5.40075  2.83915  1.49211  4.74220  3.54971  3.81223
4.22275  1.83228  3.69962  4.46491  2.43080  4.00274  2.92635  3.03505
2.32022  3.14263  3.79431  5.82998  4.41914      53 e - - -
          2.68618  4.42225  2.77519  2.73123  3.46354  2.40513  3.72494
3.29354  2.67741  2.69355  4.24690  2.90347  2.73739  3.18146  2.89801
2.37887  2.77519  2.98518  4.58477  3.61503
```

```
          0.11139  4.61273  2.34877  0.61958  0.77255  0.66070  0.72668
     45   2.36809  5.33884  2.86026  1.40567  4.69477  3.54870  3.80751
4.14409  1.74798  3.64296  4.43111  3.03442  4.00727  2.93055  2.93620
2.87417  3.15443  3.74054  5.78128  4.40277     54 e - - -
          2.68618  4.42225  2.77519  2.73123  3.46354  2.40513  3.72494
3.29354  2.67741  2.69355  4.24690  2.90347  2.73739  3.18146  2.89801
2.37887  2.77519  2.98518  4.58477  3.61503
          0.01635  4.51768  5.24003  0.61958  0.77255  0.79672  0.59930
     46   2.86971  4.43621  3.97305  3.41613  1.77935  2.68221  3.95097
2.91947  2.50397  2.61310  3.55146  3.69928  4.24858  3.58159  3.58675
3.15220  3.10012  2.71794  3.04527  2.41082     55 f - - -
          2.68618  4.42225  2.77519  2.73123  3.46354  2.40513  3.72494
3.29354  2.67741  2.69355  4.24690  2.90347  2.73739  3.18146  2.89801
2.37887  2.77519  2.98518  4.58477  3.61503
          0.25863  4.51768  1.52795  0.61958  0.77255  0.79672  0.59930
     47   2.03422  4.78510  2.98257  2.62537  4.38796  1.99833  3.88652
3.81790  2.68813  3.41371  4.23189  3.09207  3.89322  1.87704  3.10771
2.70436  2.97399  3.40653  5.62430  4.30139     56 q - - -
          2.68618  4.42225  2.77519  2.73123  3.46354  2.40513  3.72494
3.29354  2.67741  2.69355  4.24690  2.90347  2.73739  3.18146  2.89801
2.37887  2.77519  2.98518  4.58477  3.61503
          0.02078  4.27983  5.00218  0.61958  0.77255  0.76517  0.62597
     48   2.81318  4.79669  3.37239  2.80919  4.05906  3.64465  3.85416
2.43751  1.43839  3.01472  3.91814  3.26779  4.04932  3.04254  2.86328
2.91429  2.37459  2.99316  5.36820  4.10147     57 k - - -
          2.68618  4.42225  2.77519  2.73123  3.46354  2.40513  3.72494
3.29354  2.67741  2.69355  4.24690  2.90347  2.73739  3.18146  2.89801
2.37887  2.77519  2.98518  4.58477  3.61503
          0.01822  4.41025  5.13260  0.61958  0.77255  0.46470  0.98973
     49   2.33373  5.14609  2.97601  2.13931  4.49640  3.52234  3.79085
3.95090  2.56948  3.48838  4.26455  2.13978  2.89362  2.91318  3.05395
2.77085  1.95253  3.55543  5.66794  4.28623     58 t - - -
          2.68618  4.42225  2.77519  2.73123  3.46354  2.40513  3.72494
3.29354  2.67741  2.69355  4.24690  2.90347  2.73739  3.18146  2.89801
2.37887  2.77519  2.98518  4.58477  3.61503
          0.01483  4.61419  5.33653  0.61958  0.77255  0.65835  0.72920
     50   3.82246  5.46379  4.63455  4.64458  5.66133  0.15509  5.65127
5.50364  4.91689  5.02901  6.05182  4.80240  4.86561  5.18391  5.01376
4.02315  4.35381  4.89086  6.50221  5.80734     59 G - - -
          2.68618  4.42225  2.77519  2.73123  3.46354  2.40513  3.72494
3.29354  2.67741  2.69355  4.24690  2.90347  2.73739  3.18146  2.89801
2.37887  2.77519  2.98518  4.58477  3.61503
          0.01483  4.61419  5.33653  0.61958  0.77255  0.65835  0.72920
     51   2.75787  5.09047  3.04237  2.51520  4.37889  3.54150  3.75523
3.82758  2.52056  3.38071  4.16508  2.17264  3.94580  2.13244  2.99294
2.10000  2.49926  3.45809  5.57187  3.13681     60 s - - -
          2.68618  4.42225  2.77519  2.73123  3.46354  2.40513  3.72494
3.29354  2.67741  2.69355  4.24690  2.90347  2.73739  3.18146  2.89801
2.37887  2.77519  2.98518  4.58477  3.61503
          0.01483  4.61419  5.33653  0.61958  0.77255  0.65835  0.72920
     52   2.75934  4.31003  3.98259  3.40287  2.29815  3.81477  4.07663
2.76719  2.69345  2.11957  2.73633  3.69515  4.18600  3.56380  2.71591
3.08702  2.99046  2.56829  4.86976  2.28553     61 l - - -
```

2.68618 4.42225 2.77519 2.73123 3.46354 2.40513 3.72494
3.29354 2.67741 2.69355 4.24690 2.90347 2.73739 3.18146 2.89801
2.37887 2.77519 2.98518 4.58477 3.61503
0.12153 4.61419 2.25831 0.61958 0.77255 0.65835 0.72920
53 2.76507 5.07177 2.47134 2.49575 4.36261 3.51315 3.76136
3.79925 2.20822 2.62384 4.16329 3.02880 3.93956 2.89597 3.00927
1.58509 3.00557 3.43871 5.56935 4.20472 62 s - - -
2.68618 4.42225 2.77519 2.73123 3.46354 2.40513 3.72494
3.29354 2.67741 2.69355 4.24690 2.90347 2.73739 3.18146 2.89801
2.37887 2.77519 2.98518 4.58477 3.61503
0.01649 4.50914 5.23149 0.61958 0.77255 0.54040 0.87350
54 1.98232 4.65614 3.39462 2.84147 3.86310 3.63651 3.90394
3.22975 2.34397 1.91476 3.78130 3.30001 3.02976 3.13933 3.19054
2.87852 2.44961 2.96884 5.24228 3.97422 63 l - - -
2.68618 4.42225 2.77519 2.73123 3.46354 2.40513 3.72494
3.29354 2.67741 2.69355 4.24690 2.90347 2.73739 3.18146 2.89801
2.37887 2.77519 2.98518 4.58477 3.61503
0.01485 4.61273 5.33507 0.61958 0.77255 0.66070 0.72668
55 1.79396 4.36521 3.79941 3.22716 2.81173 3.76450 4.03641
2.82678 2.52396 2.22033 2.70119 3.57933 4.14099 3.44047 3.46104
3.02424 2.48181 2.61883 4.96166 3.74544 64 a - - -
2.68618 4.42225 2.77519 2.73123 3.46354 2.40513 3.72494
3.29354 2.67741 2.69355 4.24690 2.90347 2.73739 3.18146 2.89801
2.37887 2.77519 2.98518 4.58477 3.61503
0.20048 4.61273 1.76178 0.61958 0.77255 0.39012 1.13004
56 2.72078 4.77042 3.21682 2.32621 3.97424 2.32723 3.79969
2.69462 2.29496 2.46033 3.85531 3.15992 3.97306 2.98812 3.07272
2.80124 2.44982 3.07710 5.30241 4.00251 65 k - - -
2.68584 4.42230 2.77525 2.73128 3.46359 2.40518 3.72500
3.29359 2.67706 2.69360 4.24695 2.90352 2.73745 3.18152 2.89806
2.37892 2.77525 2.98524 4.58482 3.61508
0.25119 1.52803 5.26566 0.17539 1.82716 0.76305 0.62781
57 2.33044 5.40841 1.66284 1.98907 4.73242 3.49569 3.80700
4.22453 2.62581 3.70835 4.47459 2.95953 3.97201 2.40214 3.14614
2.28060 3.13275 3.79156 5.85129 4.41492 67 d - - -
2.68618 4.42225 2.77519 2.73123 3.46354 2.40513 3.72494
3.29354 2.67741 2.69355 4.24690 2.90347 2.73739 3.18146 2.89801
2.37887 2.77519 2.98518 4.58477 3.61503
0.01593 4.54331 5.26566 0.61958 0.77255 0.34474 1.23239
58 2.13094 4.83037 2.67736 2.70517 4.02892 3.63312 2.60046
2.74882 2.69322 2.53402 3.91227 2.60274 4.02138 3.03432 3.13702
2.84617 3.00072 3.14165 5.35691 3.01680 68 a - - -
2.68618 4.42225 2.77519 2.73123 3.46354 2.40513 3.72494
3.29354 2.67741 2.69355 4.24690 2.90347 2.73739 3.18146 2.89801
2.37887 2.77519 2.98518 4.58477 3.61503
0.01350 4.70791 5.43026 0.61958 0.77255 0.48576 0.95510
59 3.59404 4.85790 5.54519 4.96988 2.47688 4.93464 5.28126
1.91368 4.82181 0.93746 3.26732 5.08260 5.11493 4.82926 4.82295
4.29457 3.81666 1.64829 5.49866 4.42857 69 l - - -
2.68618 4.42225 2.77519 2.73123 3.46354 2.40513 3.72494
3.29354 2.67741 2.69355 4.24690 2.90347 2.73739 3.18146 2.89801
2.37887 2.77519 2.98518 4.58477 3.61503
0.01350 4.70791 5.43026 0.61958 0.77255 0.48576 0.95510

```
     60   2.77717  5.24211  2.51742  2.20406  4.56789  3.54918  3.74374
4.04070  2.17565  2.40050  4.28876  3.02384  3.94649  2.19291  2.97635
2.35894  2.60003  3.62251  5.68566  4.28595     70 k - - -
         2.68618  4.42225  2.77519  2.73123  3.46354  2.40513  3.72494
3.29354  2.67741  2.69355  4.24690  2.90347  2.73739  3.18146  2.89801
2.37887  2.77519  2.98518  4.58477  3.61503
         0.01350  4.70791  5.43026  0.61958  0.77255  0.48576  0.95510
     61   2.77348  5.22233  2.54371  2.15841  4.54204  3.55103  3.74433
2.67557  1.97764  3.51631  4.27028  2.54272  3.94672  2.46251  2.97754
2.74727  3.00494  2.83011  5.66979  4.27437     71 k - - -
         2.68618  4.42225  2.77519  2.73123  3.46354  2.40513  3.72494
3.29354  2.67741  2.69355  4.24690  2.90347  2.73739  3.18146  2.89801
2.37887  2.77519  2.98518  4.58477  3.61503
         0.10075  4.70791  2.44396  0.61958  0.77255  0.48576  0.95510
     62   1.54525  4.43647  4.10283  3.65434  3.91558  2.09343  4.46303
1.52513  3.59992  2.92717  3.87667  3.83264  4.21219  3.86971  3.85961
3.00249  3.09732  2.71567  5.41852  4.21589     72 i - - -
         2.68618  4.42225  2.77519  2.73123  3.46354  2.40513  3.72494
3.29354  2.67741  2.69355  4.24690  2.90347  2.73739  3.18146  2.89801
2.37887  2.77519  2.98518  4.58477  3.61503
         0.01472  4.62188  5.34423  0.61958  0.77255  0.64581  0.74283
     63   3.72778  4.97633  5.15064  4.76986  1.57851  4.65361  3.92837
3.43409  4.57565  2.88373  2.48408  4.47218  4.96083  4.51437  4.53877
3.99775  3.94696  3.32171  1.32438  1.84760     73 w - - -
         2.68618  4.42225  2.77519  2.73123  3.46354  2.40513  3.72494
3.29354  2.67741  2.69355  4.24690  2.90347  2.73739  3.18146  2.89801
2.37887  2.77519  2.98518  4.58477  3.61503
         0.01472  4.62188  5.34423  0.61958  0.77255  0.39730  1.11515
     64   2.84172  5.36021  2.18000  2.10860  4.69704  2.79069  3.78641
4.18623  2.55260  3.66262  4.40857  2.20099  3.97715  2.41432  3.05604
2.38844  2.50725  3.74750  5.79480  4.37343     74 e - - -
         2.68618  4.42225  2.77519  2.73123  3.46354  2.40513  3.72494
3.29354  2.67741  2.69355  4.24690  2.90347  2.73739  3.18146  2.89801
2.37887  2.77519  2.98518  4.58477  3.61503
         0.01350  4.70791  5.43026  0.61958  0.77255  0.48576  0.95510
     65   1.85224  4.45630  2.80561  3.15197  3.58726  3.77200  4.03473
2.44982  3.10099  2.02513  3.56135  3.53861  4.15119  3.39478  3.43712
3.02347  2.54348  2.71153  5.04670  2.85397     75 a - - -
         2.68618  4.42225  2.77519  2.73123  3.46354  2.40513  3.72494
3.29354  2.67741  2.69355  4.24690  2.90347  2.73739  3.18146  2.89801
2.37887  2.77519  2.98518  4.58477  3.61503
         0.01350  4.70791  5.43026  0.61958  0.77255  0.48576  0.95510
     66   2.37078  4.21150  4.45160  3.85629  2.63237  3.91855  4.23465
1.91967  3.70219  2.39731  3.32779  3.97810  4.28582  3.88788  3.81479
2.26138  3.01757  2.07335  4.82541  2.37935     76 i - - -
         2.68618  4.42225  2.77519  2.73123  3.46354  2.40513  3.72494
3.29354  2.67741  2.69355  4.24690  2.90347  2.73739  3.18146  2.89801
2.37887  2.77519  2.98518  4.58477  3.61503
         0.01350  4.70791  5.43026  0.61958  0.77255  0.48576  0.95510
     67   2.79190  5.17686  2.54819  2.52293  4.47721  3.57199  3.76801
2.56078  1.96699  3.46027  4.23288  3.06093  3.96949  1.94803  2.98766
2.77803  2.50753  3.54156  5.63635  4.25917     77 q - - -
         2.68620  4.42227  2.77522  2.73125  3.46356  2.40489  3.72497
3.29356  2.67743  2.69357  4.24692  2.90349  2.73742  3.18148  2.89803
2.37889  2.77522  2.98520  4.58479  3.61505
```

```
          0.09189  2.48391  5.43026  0.29740  1.35769  0.48576  0.95510
     68   2.81603  5.28318  2.48263  2.48424  4.62410  2.43609  3.78313
4.10075  2.19229  3.59723  4.35075  2.56899  3.97139  2.89076  3.03933
2.33246  1.95118  3.67711  5.74391  4.33794     79 t - - -
          2.68618  4.42225  2.77519  2.73123  3.46354  2.40513  3.72494
3.29354  2.67741  2.69355  4.24690  2.90347  2.73739  3.18146  2.89801
2.37887  2.77519  2.98518  4.58477  3.61503
          0.01350  4.70791  5.43026  0.61958  0.77255  0.48576  0.95510
     69   3.05729  5.58574  1.88491  2.48887  4.91406  1.48437  3.94679
4.40903  2.21424  3.88749  4.66929  3.05640  4.10129  2.40219  3.28875
2.98628  3.31006  3.97569  6.02680  4.58467     80 g - - -
          2.68618  4.42225  2.77519  2.73123  3.46354  2.40513  3.72494
3.29354  2.67741  2.69355  4.24690  2.90347  2.73739  3.18146  2.89801
2.37887  2.77519  2.98518  4.58477  3.61503
          0.01350  4.70791  5.43026  0.61958  0.77255  0.48576  0.95510
     70   2.40695  5.22605  3.02952  1.91641  4.56257  3.55814  3.77058
4.02790  1.95818  3.53717  4.29613  3.04753  3.96566  2.87975  2.99519
2.09993  2.23861  3.61749  5.69226  4.30119     81 e - - -
          2.68618  4.42225  2.77519  2.73123  3.46354  2.40513  3.72494
3.29354  2.67741  2.69355  4.24690  2.90347  2.73739  3.18146  2.89801
2.37887  2.77519  2.98518  4.58477  3.61503
          0.01350  4.70791  5.43026  0.61958  0.77255  0.48576  0.95510
     71   3.45862  4.72271  5.44552  4.89252  2.14510  4.83913  5.25093
1.14327  4.75445  1.84200  3.46772  4.98473  5.07619  4.85251  4.79950
4.20190  3.69472  1.53390  5.56536  4.41788     82 i - - -
          2.68618  4.42225  2.77519  2.73123  3.46354  2.40513  3.72494
3.29354  2.67741  2.69355  4.24690  2.90347  2.73739  3.18146  2.89801
2.37887  2.77519  2.98518  4.58477  3.61503
          0.01350  4.70791  5.43026  0.61958  0.77255  0.48576  0.95510
     72   3.52855  6.17769  0.82759  2.51563  5.49349  3.64028  4.30974
5.12404  3.46810  4.57894  5.48171  1.49733  4.33018  3.49822  4.15231
3.37346  3.85636  4.63928  6.68236  5.09841     83 d - - -
          2.68618  4.42225  2.77519  2.73123  3.46354  2.40513  3.72494
3.29354  2.67741  2.69355  4.24690  2.90347  2.73739  3.18146  2.89801
2.37887  2.77519  2.98518  4.58477  3.61503
          0.01350  4.70791  5.43026  0.61958  0.77255  0.48576  0.95510
     73   3.71832  5.29790  4.35631  4.12717  2.96879  4.32134  4.25700
4.08431  4.06190  3.53625  4.67637  4.27650  2.48080  4.34301  4.25447
3.85164  4.05129  3.88162  4.60229  0.51682     84 Y - - -
          2.68618  4.42225  2.77519  2.73123  3.46354  2.40513  3.72494
3.29354  2.67741  2.69355  4.24690  2.90347  2.73739  3.18146  2.89801
2.37887  2.77519  2.98518  4.58477  3.61503
          0.01350  4.70791  5.43026  0.61958  0.77255  0.48576  0.95510
     74   3.38646  4.85677  4.58731  4.04652  2.54128  4.45532  4.59283
2.80710  3.87884  0.85392  3.33233  4.33141  4.74577  2.16259  4.08047
3.76714  3.60957  2.89129  5.11268  3.80549     85 l - - -
          2.68618  4.42225  2.77519  2.73123  3.46354  2.40513  3.72494
3.29354  2.67741  2.69355  4.24690  2.90347  2.73739  3.18146  2.89801
2.37887  2.77519  2.98518  4.58477  3.61503
          0.01350  4.70791  5.43026  0.61958  0.77255  0.48576  0.95510
     75   1.61685  4.79359  3.47726  3.00013  4.61362  2.60072  4.18226
4.05748  3.02304  3.65640  4.44505  2.46881  4.05159  3.34894  3.45766
1.82010  1.98296  3.57709  5.86456  4.56174     86 a - - -
```

```
         2.68618  4.42225  2.77519  2.73123  3.46354  2.40513  3.72494
3.29354  2.67741  2.69355  4.24690  2.90347  2.73739  3.18146  2.89801
2.37887  2.77519  2.98518  4.58477  3.61503
         0.01350  4.70791  5.43026  0.61958  0.77255  0.48576  0.95510
     76  3.30989  4.64780  5.09006  4.52137  2.33274  4.47944  4.56960
2.69124  4.34470  0.91344  3.31043  4.55154  4.76416  4.40925  4.36929
3.80476  3.53360  2.12670  4.88056  2.55711     87 l - - -
         2.68618  4.42225  2.77519  2.73123  3.46354  2.40513  3.72494
3.29354  2.67741  2.69355  4.24690  2.90347  2.73739  3.18146  2.89801
2.37887  2.77519  2.98518  4.58477  3.61503
         0.01350  4.70791  5.43026  0.61958  0.77255  0.48576  0.95510
     77  3.25634  4.57446  5.07678  4.52880  3.79891  4.58318  5.04308
1.76298  4.40725  2.07891  3.66592  4.67385  4.89836  4.61882  4.54493
3.92888  1.84261  1.11204  5.56398  4.37288     88 v - - -
         2.68618  4.42225  2.77519  2.73123  3.46354  2.40513  3.72494
3.29354  2.67741  2.69355  4.24690  2.90347  2.73739  3.18146  2.89801
2.37887  2.77519  2.98518  4.58477  3.61503
         0.01350  4.70791  5.43026  0.61958  0.77255  0.48576  0.95510
     78  2.87466  5.32258  3.10874  2.56220  4.67609  3.62090  2.82370
4.13556  1.53161  3.61104  4.37168  2.54522  4.01518  2.14562  2.84792
2.84103  2.53135  3.72059  5.73089  4.36132     89 k - - -
         2.68618  4.42225  2.77519  2.73123  3.46354  2.40513  3.72494
3.29354  2.67741  2.69355  4.24690  2.90347  2.73739  3.18146  2.89801
2.37887  2.77519  2.98518  4.58477  3.61503
         0.01350  4.70791  5.43026  0.61958  0.77255  0.48576  0.95510
     79  1.85065  4.78590  3.36503  2.81895  4.11453  2.72096  3.94487
2.72879  2.79100  3.15755  3.99702  3.28534  4.04335  3.13805  2.53857
1.80544  3.02466  3.19809  5.44191  4.14955     90 s - - -
         2.68618  4.42225  2.77519  2.73123  3.46354  2.40513  3.72494
3.29354  2.67741  2.69355  4.24690  2.90347  2.73739  3.18146  2.89801
2.37887  2.77519  2.98518  4.58477  3.61503
         0.01350  4.70791  5.43026  0.61958  0.77255  0.48576  0.95510
     80  2.22008  4.50009  3.83003  3.29733  3.85551  1.92951  4.20946
2.45686  3.25115  2.92367  3.81503  3.61695  4.14773  3.54411  3.58659
1.76679  3.04720  2.29725  5.29903  4.07435     91 s - - -
         2.68618  4.42225  2.77519  2.73123  3.46354  2.40513  3.72494
3.29354  2.67741  2.69355  4.24690  2.90347  2.73739  3.18146  2.89801
2.37887  2.77519  2.98518  4.58477  3.61503
         0.01350  4.70791  5.43026  0.61958  0.77255  0.48576  0.95510
     81  3.83140  5.06068  5.75286  5.18387  2.37943  5.19465  5.45005
1.26522  5.04346  0.93785  3.19924  5.33276  5.28376  4.96074  5.01064
4.57400  4.04272  2.62017  5.53014  4.45135     92 l - - -
         2.68618  4.42225  2.77519  2.73123  3.46354  2.40513  3.72494
3.29354  2.67741  2.69355  4.24690  2.90347  2.73739  3.18146  2.89801
2.37887  2.77519  2.98518  4.58477  3.61503
         0.01350  4.70791  5.43026  0.61958  0.77255  0.48576  0.95510
     82  2.63368  4.59424  4.05750  3.78331  5.01183  1.83197  4.83630
4.47456  3.85350  4.13182  4.91246  3.83504  4.13957  4.10501  4.15123
0.82624  2.21290  3.77906  6.32133  5.14055     93 s - - -
         2.68618  4.42225  2.77519  2.73123  3.46354  2.40513  3.72494
3.29354  2.67741  2.69355  4.24690  2.90347  2.73739  3.18146  2.89801
2.37887  2.77519  2.98518  4.58477  3.61503
         0.01350  4.70791  5.43026  0.61958  0.77255  0.48576  0.95510
```

```
     83   3.86883  5.13567  5.65580  5.15187  3.50147  5.16517  5.60744
2.47108  4.97366  0.51590  3.27368  5.34630  5.32570  5.00181  5.00573
4.59636  4.11150  1.99577  5.70675  4.66599     94 L - - -
          2.68618  4.42225  2.77519  2.73123  3.46354  2.40513  3.72494
3.29354  2.67741  2.69355  4.24690  2.90347  2.73739  3.18146  2.89801
2.37887  2.77519  2.98518  4.58477  3.61503
          0.01350  4.70791  5.43026  0.61958  0.77255  0.48576  0.95510
     84   2.41820  4.37781  4.55834  3.98465  3.54402  4.13906  4.49683
1.39946  3.85883  2.07638  3.50643  2.86683  4.49689  4.07797  4.02121
3.44608  3.20215  1.66494  5.12929  3.93552     95 i - - -
          2.68618  4.42225  2.77519  2.73123  3.46354  2.40513  3.72494
3.29354  2.67741  2.69355  4.24690  2.90347  2.73739  3.18146  2.89801
2.37887  2.77519  2.98518  4.58477  3.61503
          0.10856  4.70791  2.36608  0.61958  0.77255  0.48576  0.95510
     85   2.65665  4.56967  3.65299  3.17937  4.16643  1.55625  4.21928
3.54204  3.16052  2.39263  4.09331  3.50593  4.06075  3.47761  3.52868
2.19457  1.87185  3.18978  5.54625  4.30131     96 g - - -
          2.68618  4.42225  2.77519  2.73123  3.46354  2.40513  3.72494
3.29354  2.67741  2.69355  4.24690  2.90347  2.73739  3.18146  2.89801
2.37887  2.77519  2.98518  4.58477  3.61503
          0.30940  4.61419  1.36179  0.61958  0.77255  0.39124  1.12769
     86   2.74436  4.28901  4.03721  3.47636  3.44300  3.80181  4.17362
1.77047  3.37559  2.04176  3.41676  3.74946  4.20318  3.64280  3.62181
2.46089  2.00079  2.35626  4.97573  3.77208     97 i - - -
          2.68591  4.42232  2.77526  2.73130  3.46361  2.40494  3.72501
3.29361  2.67747  2.69328  4.24696  2.90353  2.73746  3.18153  2.89807
2.37893  2.77526  2.98525  4.58484  3.61510
          0.12088  2.22398  5.17032  0.93183  0.50060  0.30062  1.34846
     87   1.76404  4.41126  4.71514  4.13280  1.88728  4.16900  4.50810
2.52969  3.98168  1.66203  3.35120  4.25171  4.51805  4.14661  4.08474
3.48399  3.24761  2.00179  5.03287  3.84222    101 l - - -
          2.68618  4.42225  2.77519  2.73123  3.46354  2.40513  3.72494
3.29354  2.67741  2.69355  4.24690  2.90347  2.73739  3.18146  2.89801
2.37887  2.77519  2.98518  4.58477  3.61503
          0.01350  4.70791  5.43026  0.61958  0.77255  0.48576  0.95510
     88   3.61667  4.81303  5.69412  5.21479  4.05681  5.22373  5.89975
1.09898  5.14938  1.97530  3.79468  5.37431  5.42056  5.35579  5.28810
4.65010  3.87066  0.98764  6.17493  4.99437    102 v - - -
          2.68618  4.42225  2.77519  2.73123  3.46354  2.40513  3.72494
3.29354  2.67741  2.69355  4.24690  2.90347  2.73739  3.18146  2.89801
2.37887  2.77519  2.98518  4.58477  3.61503
          0.01350  4.70791  5.43026  0.61958  0.77255  0.48576  0.95510
     89   2.84533  4.75727  4.05672  3.89892  4.98482  3.51333  4.95712
4.36438  3.94528  4.08604  4.98440  3.98003  0.57434  4.27343  4.18276
3.03511  2.22975  3.80966  6.30987  5.15179    103 P - - -
          2.68618  4.42225  2.77519  2.73123  3.46354  2.40513  3.72494
3.29354  2.67741  2.69355  4.24690  2.90347  2.73739  3.18146  2.89801
2.37887  2.77519  2.98518  4.58477  3.61503
          0.01350  4.70791  5.43026  0.61958  0.77255  0.48576  0.95510
     90   2.96367  5.43269  2.85787  1.59649  4.78141  1.78767  3.88801
4.25861  2.18335  3.75042  4.52478  3.07073  2.85196  3.00887  3.14574
2.91578  3.21039  3.83641  5.89375  4.48298    104 e - - -
          2.68618  4.42225  2.77519  2.73123  3.46354  2.40513  3.72494
3.29354  2.67741  2.69355  4.24690  2.90347  2.73739  3.18146  2.89801
2.37887  2.77519  2.98518  4.58477  3.61503
```

```
          0.01350  4.70791  5.43026  0.61958  0.77255  0.48576  0.95510
     91   2.10269  4.34342  4.60731  4.02993  3.50479  2.92469  4.48019
1.53418  3.89191  2.08120  3.47717  4.17119  4.47215  4.09436  4.02673
3.41765  3.17627  1.67201  5.08219  3.89292    105 i - - -
          2.68618  4.42225  2.77519  2.73123  3.46354  2.40513  3.72494
3.29354  2.67741  2.69355  4.24690  2.90347  2.73739  3.18146  2.89801
2.37887  2.77519  2.98518  4.58477  3.61503
          0.01350  4.70791  5.43026  0.61958  0.77255  0.48576  0.95510
     92   2.36011  5.27224  2.46711  2.20096  4.64200  1.72192  3.84958
4.11255  2.64249  3.62841  4.39668  3.06439  4.00753  2.96860  3.14175
2.39610  2.55050  3.69521  5.79147  4.38988    106 g - - -
          2.68618  4.42225  2.77519  2.73123  3.46354  2.40513  3.72494
3.29354  2.67741  2.69355  4.24690  2.90347  2.73739  3.18146  2.89801
2.37887  2.77519  2.98518  4.58477  3.61503
          0.01350  4.70791  5.43026  0.61958  0.77255  0.48576  0.95510
     93   2.09702  4.24472  4.38307  3.79447  2.25199  3.91970  4.24830
1.91437  3.65752  2.42707  3.36398  3.95396  2.98433  3.85978  3.80174
3.21657  2.44476  2.12119  4.87961  3.69198    107 i - - -
          2.68618  4.42225  2.77519  2.73123  3.46354  2.40513  3.72494
3.29354  2.67741  2.69355  4.24690  2.90347  2.73739  3.18146  2.89801
2.37887  2.77519  2.98518  4.58477  3.61503
          0.01350  4.70791  5.43026  0.61958  0.77255  0.48576  0.95510
     94   2.05783  4.36168  4.52674  3.96949  2.65944  3.99977  4.47508
2.49964  3.84414  2.53788  3.53150  4.11081  4.42214  4.05627  4.00121
3.32989  2.43274  1.18728  5.12203  3.92785    108 v - - -
          2.68618  4.42225  2.77519  2.73123  3.46354  2.40513  3.72494
3.29354  2.67741  2.69355  4.24690  2.90347  2.73739  3.18146  2.89801
2.37887  2.77519  2.98518  4.58477  3.61503
          0.01350  4.70791  5.43026  0.61958  0.77255  0.48576  0.95510
     95   1.49355  4.45678  4.21118  3.73528  4.04263  3.56046  4.52538
3.23143  3.66653  3.10090  4.00711  3.86215  4.19904  3.92436  3.92568
2.25060  2.33888  1.40476  5.51297  4.31886    109 v - - -
          2.68618  4.42225  2.77519  2.73123  3.46354  2.40513  3.72494
3.29354  2.67741  2.69355  4.24690  2.90347  2.73739  3.18146  2.89801
2.37887  2.77519  2.98518  4.58477  3.61503
          0.01350  4.70791  5.43026  0.61958  0.77255  0.48576  0.95510
     96   2.80306  4.74257  3.91266  3.80357  5.06086  3.45813  4.94197
4.60776  3.97300  4.26795  5.10320  3.90727  0.58602  4.25323  4.23025
2.06709  3.33538  3.93236  6.36996  5.17242    110 P - - -
          2.68618  4.42225  2.77519  2.73123  3.46354  2.40513  3.72494
3.29354  2.67741  2.69355  4.24690  2.90347  2.73739  3.18146  2.89801
2.37887  2.77519  2.98518  4.58477  3.61503
          0.01350  4.70791  5.43026  0.61958  0.77255  0.48576  0.95510
     97   2.41228  4.56551  4.99047  4.40716  1.36127  4.40649  4.70275
2.09790  4.24508  1.52486  3.29309  4.50460  4.70756  4.35974  4.30760
3.73037  3.43825  2.48392  5.11816  3.92656    111 f - - -
          2.68618  4.42225  2.77519  2.73123  3.46354  2.40513  3.72494
3.29354  2.67741  2.69355  4.24690  2.90347  2.73739  3.18146  2.89801
2.37887  2.77519  2.98518  4.58477  3.61503
          0.01350  4.70791  5.43026  0.61958  0.77255  0.48576  0.95510
     98   2.44531  4.64610  5.28140  4.74061  3.79736  4.73970  5.22212
1.05815  4.61774  1.63969  3.62953  4.86336  5.02359  4.79810  4.72470
4.10046  3.60830  1.68235  5.65610  4.48671    112 i - - -
```

2.68618 4.42225 2.77519 2.73123 3.46354 2.40513 3.72494
3.29354 2.67741 2.69355 4.24690 2.90347 2.73739 3.18146 2.89801
2.37887 2.77519 2.98518 4.58477 3.61503
0.01350 4.70791 5.43026 0.61958 0.77255 0.48576 0.95510
99 2.79745 4.94336 3.20911 2.77840 4.43365 2.18443 4.01280
3.86821 2.84229 2.57394 4.28677 1.65517 4.05815 3.17775 3.29098
1.97353 3.10408 3.48590 5.69910 4.36910 113 n - - -
2.68618 4.42225 2.77519 2.73123 3.46354 2.40513 3.72494
3.29354 2.67741 2.69355 4.24690 2.90347 2.73739 3.18146 2.89801
2.37887 2.77519 2.98518 4.58477 3.61503
0.01350 4.70791 5.43026 0.61958 0.77255 0.48576 0.95510
100 3.36122 4.75272 4.96917 4.39915 3.41921 4.52574 4.88175
2.59453 4.22366 1.20378 1.34054 4.58973 4.81481 4.38425 4.33857
3.86060 2.52778 2.64424 5.32422 4.22737 114 l - - -
2.68618 4.42225 2.77519 2.73123 3.46354 2.40513 3.72494
3.29354 2.67741 2.69355 4.24690 2.90347 2.73739 3.18146 2.89801
2.37887 2.77519 2.98518 4.58477 3.61503
0.01350 4.70791 5.43026 0.61958 0.77255 0.48576 0.95510
101 3.45072 4.73629 5.37439 4.80688 1.30642 4.76382 5.08792
1.90037 4.65467 1.73104 3.35633 4.89406 5.00085 4.72245 4.68444
4.11407 3.68041 1.62464 5.38819 4.21638 115 f - - -
2.68618 4.42225 2.77519 2.73123 3.46354 2.40513 3.72494
3.29354 2.67741 2.69355 4.24690 2.90347 2.73739 3.18146 2.89801
2.37887 2.77519 2.98518 4.58477 3.61503
0.01350 4.70791 5.43026 0.61958 0.77255 0.48576 0.95510
102 3.04960 4.39953 4.92448 4.34350 2.05952 4.24801 4.48915
1.77798 4.15977 2.33118 3.41554 4.36177 4.58344 4.28741 4.19763
3.56923 3.28282 1.36146 4.90944 2.64081 116 v - - -
2.68618 4.42225 2.77519 2.73123 3.46354 2.40513 3.72494
3.29354 2.67741 2.69355 4.24690 2.90347 2.73739 3.18146 2.89801
2.37887 2.77519 2.98518 4.58477 3.61503
0.01350 4.70791 5.43026 0.61958 0.77255 0.48576 0.95510
103 2.37402 5.22469 2.25921 2.50791 4.59396 2.11688 3.78745
4.06601 2.54562 3.57121 4.32624 3.04867 2.91939 2.89638 2.51274
2.33077 3.03628 3.64285 5.72314 4.32596 117 g - - -
2.68618 4.42225 2.77519 2.73123 3.46354 2.40513 3.72494
3.29354 2.67741 2.69355 4.24690 2.90347 2.73739 3.18146 2.89801
2.37887 2.77519 2.98518 4.58477 3.61503
0.01350 4.70791 5.43026 0.61958 0.77255 0.48576 0.95510
104 2.02527 4.32081 4.07916 2.68411 1.78448 3.86071 4.13943
2.76873 3.40805 2.50579 3.43015 3.77347 4.23449 3.65522 3.64143
3.13868 3.02600 2.22801 3.21712 3.66338 118 f - - -
2.68618 4.42225 2.77519 2.73123 3.46354 2.40513 3.72494
3.29354 2.67741 2.69355 4.24690 2.90347 2.73739 3.18146 2.89801
2.37887 2.77519 2.98518 4.58477 3.61503
0.01350 4.70791 5.43026 0.61958 0.77255 0.48576 0.95510
105 3.42356 4.71326 5.39181 4.80654 2.58534 4.72462 5.08221
1.42263 4.64097 1.43766 2.43166 4.88070 4.95877 4.68687 4.64498
4.07053 3.64968 1.69083 5.38162 4.29236 119 i - - -
2.68618 4.42225 2.77519 2.73123 3.46354 2.40513 3.72494
3.29354 2.67741 2.69355 4.24690 2.90347 2.73739 3.18146 2.89801
2.37887 2.77519 2.98518 4.58477 3.61503
0.01350 4.70791 5.43026 0.61958 0.77255 0.48576 0.95510

```
    106   2.78209  4.70594  3.40020  2.84257  3.00158  2.83960  3.90847
3.26861  2.80612  2.94102  3.80557  2.68093  4.06995  3.14591  2.61263
2.41022  3.01604  3.00882  2.06964  3.94751    120 w - - -
          2.68618  4.42225  2.77519  2.73123  3.46354  2.40513  3.72494
3.29354  2.67741  2.69355  4.24690  2.90347  2.73739  3.18146  2.89801
2.37887  2.77519  2.98518  4.58477  3.61503
          0.01350  4.70791  5.43026  0.61958  0.77255  0.48576  0.95510
    107   2.83353  4.98496  3.25984  2.83997  4.58790  2.55884  4.05946
4.01584  2.26527  3.60005  4.40775  3.30269  1.31139  3.21750  3.23013
2.89369  2.42335  3.60226  5.80227  4.48680    121 p - - -
          2.68618  4.42225  2.77519  2.73123  3.46354  2.40513  3.72494
3.29354  2.67741  2.69355  4.24690  2.90347  2.73739  3.18146  2.89801
2.37887  2.77519  2.98518  4.58477  3.61503
          0.01350  4.70791  5.43026  0.61958  0.77255  0.48576  0.95510
    108   2.79485  4.87141  3.27606  2.71560  2.99783  2.83623  2.49848
3.48014  1.67646  3.11303  2.92253  3.21388  4.03566  3.03161  3.08665
2.86462  3.02482  3.18949  5.37405  4.05987    122 k - - -
          2.68618  4.42225  2.77519  2.73123  3.46354  2.40513  3.72494
3.29354  2.67741  2.69355  4.24690  2.90347  2.73739  3.18146  2.89801
2.37887  2.77519  2.98518  4.58477  3.61503
          0.01350  4.70791  5.43026  0.61958  0.77255  0.48576  0.95510
    109   2.77279  5.01255  2.61105  2.58658  4.26258  2.90796  3.79584
2.85534  2.27011  2.11364  4.08149  2.58258  3.98353  2.93814  3.05549
2.36442  3.00470  3.35110  5.50822  4.16360    123 l - - -
          2.68618  4.42225  2.77519  2.73123  3.46354  2.40513  3.72494
3.29354  2.67741  2.69355  4.24690  2.90347  2.73739  3.18146  2.89801
2.37887  2.77519  2.98518  4.58477  3.61503
          0.01350  4.70791  5.43026  0.61958  0.77255  0.48576  0.95510
    110   2.28138  4.55183  3.64295  3.11562  1.77665  3.65183  4.08998
3.19637  3.08951  2.90202  3.78354  2.71808  4.12005  3.39454  3.45426
2.35772  2.10265  2.93584  5.24728  4.00207    124 f - - -
          2.68618  4.42225  2.77519  2.73123  3.46354  2.40513  3.72494
3.29354  2.67741  2.69355  4.24690  2.90347  2.73739  3.18146  2.89801
2.37887  2.77519  2.98518  4.58477  3.61503
          0.30249  4.70791  1.37832  0.61958  0.77255  0.48576  0.95510
    111   2.72648  4.82472  3.15659  2.78339  4.47112  1.38440  3.98666
3.87821  2.13408  3.48499  4.31145  3.22363  3.98764  3.16019  3.09778
2.80262  2.25189  3.47017  5.69204  4.39906    125 g - - -
          2.68618  4.42225  2.77519  2.73123  3.46354  2.40513  3.72494
3.29354  2.67741  2.69355  4.24690  2.90347  2.73739  3.18146  2.89801
2.37887  2.77519  2.98518  4.58477  3.61503
          0.01798  4.42340  5.14574  0.61958  0.77255  0.90538  0.51817
    112   2.31435  5.03504  2.40117  2.45372  4.39541  3.45203  3.71022
3.84731  2.47767  3.38761  4.16197  2.98410  3.87762  2.83108  2.47669
1.94405  2.44203  3.45238  5.56819  4.19309    126 s - - -
          2.68618  4.42225  2.77519  2.73123  3.46354  2.40513  3.72494
3.29354  2.67741  2.69355  4.24690  2.90347  2.73739  3.18146  2.89801
2.37887  2.77519  2.98518  4.58477  3.61503
          0.01798  4.42340  5.14574  0.61958  0.77255  0.29157  1.37471
    113   2.40925  5.26192  2.52586  2.47009  4.60169  2.89853  3.74278
4.08136  2.16143  3.56853  4.31225  2.29738  3.01966  2.84374  2.97875
2.35466  2.50440  3.65150  5.70663  4.30047    127 k - - -
          2.68636  4.42243  2.77538  2.73108  3.46372  2.40507  3.72513
3.29372  2.67759  2.69373  4.24708  2.90365  2.73679  3.18056  2.89819
2.37881  2.77503  2.98537  4.58495  3.61521
```

```
          0.64722  1.44886  1.42020  0.91362  0.51261  0.48576  0.95510
    114   2.74386  4.90414  3.10900  2.59062  4.16909  3.53956  3.76164
3.57636  2.17330  3.18106  2.80974  2.53580  1.97682  2.92020  2.92191
2.78476  2.98034  3.25843  5.42091  4.10360    131 p - - -
          2.68618  4.42225  2.77519  2.73123  3.46354  2.40513  3.72494
3.29354  2.67741  2.69355  4.24690  2.90347  2.73739  3.18146  2.89801
2.37887  2.77519  2.98518  4.58477  3.61503
          0.11589  4.43704  2.32689  0.61958  0.77255  0.29650  1.36031
    115   2.42176  5.47069  2.28698  2.02606  4.79621  3.53783  3.84670
4.29166  2.19433  3.76782  4.53182  1.55628  4.01443  2.96373  3.17826
2.87093  3.17960  3.85367  5.90460  4.46543    132 n - - -
          2.68618  4.42225  2.77519  2.73123  3.46354  2.40513  3.72494
3.29354  2.67741  2.69355  4.24690  2.90347  2.73739  3.18146  2.89801
2.37887  2.77519  2.98518  4.58477  3.61503
          0.01452  4.63528  5.35762  0.61958  0.77255  0.40844  1.09270
    116   2.08603  5.05533  2.66699  2.22673  4.31925  3.57921  3.77893
2.88368  2.55766  3.32511  3.06168  3.08908  3.97113  2.23350  3.03128
2.77896  2.55811  3.40072  5.54044  4.18467    133 a - - -
          2.68618  4.42225  2.77519  2.73123  3.46354  2.40513  3.72494
3.29354  2.67741  2.69355  4.24690  2.90347  2.73739  3.18146  2.89801
2.37887  2.77519  2.98518  4.58477  3.61503
          0.01350  4.70791  5.43026  0.61958  0.77255  0.48576  0.95510
    117   2.42758  5.25054  3.02283  1.98170  4.58173  3.54910  3.74020
4.05672  1.93202  3.54902  4.29621  2.53801  2.96522  2.48726  2.96487
2.74512  3.00788  2.86927  5.69087  4.28998    134 k - - -
          2.68618  4.42225  2.77519  2.73123  3.46354  2.40513  3.72494
3.29354  2.67741  2.69355  4.24690  2.90347  2.73739  3.18146  2.89801
2.37887  2.77519  2.98518  4.58477  3.61503
          0.01350  4.70791  5.43026  0.61958  0.77255  0.48576  0.95510
    118   3.07356  5.62907  1.85333  2.47049  4.95552  3.58231  3.96472
4.46288  2.83196  3.93694  4.71782  1.49992  4.10530  2.11104  3.37141
2.35158  3.33164  4.01952  6.07629  4.61516    135 n - - -
          2.68618  4.42225  2.77519  2.73123  3.46354  2.40513  3.72494
3.29354  2.67741  2.69355  4.24690  2.90347  2.73739  3.18146  2.89801
2.37887  2.77519  2.98518  4.58477  3.61503
          0.01350  4.70791  5.43026  0.61958  0.77255  0.48576  0.95510
    119   3.74079  4.99194  5.67758  5.08815  2.51798  5.06966  5.36800
1.19855  4.93616  1.13601  2.33482  5.22288  5.18879  4.86611  4.89915
4.43364  3.95126  2.67528  5.49417  4.47903    136 l - - -
          2.68618  4.42225  2.77519  2.73123  3.46354  2.40513  3.72494
3.29354  2.67741  2.69355  4.24690  2.90347  2.73739  3.18146  2.89801
2.37887  2.77519  2.98518  4.58477  3.61503
          0.01350  4.70791  5.43026  0.61958  0.77255  0.48576  0.95510
    120   3.81327  5.07188  5.45434  5.02132  0.80246  4.95960  4.72720
1.75141  4.85953  2.13447  3.54666  4.97134  5.20233  4.85007  4.86373
4.36021  4.05167  2.71548  4.87224  3.31621    137 f - - -
          2.68618  4.42225  2.77519  2.73123  3.46354  2.40513  3.72494
3.29354  2.67741  2.69355  4.24690  2.90347  2.73739  3.18146  2.89801
2.37887  2.77519  2.98518  4.58477  3.61503
          0.01350  4.70791  5.43026  0.61958  0.77255  0.48576  0.95510
    121   2.91668  5.44534  2.33679  1.46607  4.77540  3.56373  3.84216
4.26791  2.24585  3.74414  4.50056  2.53320  4.01987  2.95391  3.14566
2.86056  2.16027  3.82989  5.87884  4.44764    138 e - - -
```

```
         2.68618  4.42225  2.77519  2.73123  3.46354  2.40513  3.72494
3.29354  2.67741  2.69355  4.24690  2.90347  2.73739  3.18146  2.89801
2.37887  2.77519  2.98518  4.58477  3.61503
         0.01350  4.70791  5.43026  0.61958  0.77255  0.48576  0.95510
    122  1.66613  4.75596  3.34464  2.34760  3.94309  3.66990  3.90143
3.30393  2.77224  2.21476  3.84628  3.27947  4.06181  2.55758  3.19236
2.89874  3.02188  2.56877  5.30807  4.02544    139 a - - -
         2.68618  4.42225  2.77519  2.73123  3.46354  2.40513  3.72494
3.29354  2.67741  2.69355  4.24690  2.90347  2.73739  3.18146  2.89801
2.37887  2.77519  2.98518  4.58477  3.61503
         0.01350  4.70791  5.43026  0.61958  0.77255  0.48576  0.95510
    123  3.85311  5.07121  5.79082  5.22472  3.47842  5.26725  5.61542
1.08690  5.09119  1.06359  2.37105  5.41230  5.33845  5.02706  5.07623
4.65707  4.06732  2.51165  5.67732  4.69208    140 l - - -
         2.68618  4.42225  2.77519  2.73123  3.46354  2.40513  3.72494
3.29354  2.67741  2.69355  4.24690  2.90347  2.73739  3.18146  2.89801
2.37887  2.77519  2.98518  4.58477  3.61503
         0.01350  4.70791  5.43026  0.61958  0.77255  0.48576  0.95510
    124  2.80666  5.02471  2.30194  2.62291  4.26755  3.61863  3.82118
2.92036  1.57350  3.26033  2.57689  3.14349  4.01329  2.96650  3.04370
2.83420  3.03761  3.33212  5.52148  4.18460    141 k - - -
         2.68618  4.42225  2.77519  2.73123  3.46354  2.40513  3.72494
3.29354  2.67741  2.69355  4.24690  2.90347  2.73739  3.18146  2.89801
2.37887  2.77519  2.98518  4.58477  3.61503
         0.01350  4.70791  5.43026  0.61958  0.77255  0.48576  0.95510
    125  2.93524  5.47242  2.04106  1.87819  4.80336  3.56758  3.85302
4.29819  2.26000  3.77019  4.52762  3.02749  2.01207  2.38146  3.15766
2.87563  3.17754  3.85753  5.90197  4.46784    142 e - - -
         2.68618  4.42225  2.77519  2.73123  3.46354  2.40513  3.72494
3.29354  2.67741  2.69355  4.24690  2.90347  2.73739  3.18146  2.89801
2.37887  2.77519  2.98518  4.58477  3.61503
         0.01350  4.70791  5.43026  0.61958  0.77255  0.48576  0.95510
    126  1.87826  5.35763  2.40720  1.92974  4.69384  3.55666  3.78830
4.17982  1.95451  3.65784  4.40617  3.02228  3.98193  2.39649  3.04293
2.80146  3.08271  3.74456  5.79051  4.37358    143 a - - -
         2.68618  4.42225  2.77519  2.73123  3.46354  2.40513  3.72494
3.29354  2.67741  2.69355  4.24690  2.90347  2.73739  3.18146  2.89801
2.37887  2.77519  2.98518  4.58477  3.61503
         0.01350  4.70791  5.43026  0.61958  0.77255  0.48576  0.95510
    127  3.54557  4.76828  5.58121  5.07639  3.95356  5.06995  5.65859
1.07857  4.98458  2.40883  2.52758  5.21553  5.29385  5.16866  5.09963
4.47041  3.79569  1.07818  5.98278  4.81608    144 v - - -
         2.68618  4.42225  2.77519  2.73123  3.46354  2.40513  3.72494
3.29354  2.67741  2.69355  4.24690  2.90347  2.73739  3.18146  2.89801
2.37887  2.77519  2.98518  4.58477  3.61503
         0.01350  4.70791  5.43026  0.61958  0.77255  0.48576  0.95510
    128  2.97784  5.46715  2.95942  1.84090  4.82457  3.63041  3.84812
4.29498  1.87702  3.75416  4.52185  2.55672  4.06817  1.60198  2.95391
2.92494  3.20768  3.86830  5.86435  4.47651    145 q - - -
         2.68618  4.42225  2.77519  2.73123  3.46354  2.40513  3.72494
3.29354  2.67741  2.69355  4.24690  2.90347  2.73739  3.18146  2.89801
2.37887  2.77519  2.98518  4.58477  3.61503
         0.01350  4.70791  5.43026  0.61958  0.77255  0.48576  0.95510
```

```
    129   2.37585  5.32636  3.04892  1.74366  4.67788  3.59233  3.77060
4.14807  1.73102  3.62131  4.37377  2.49658  3.99326  2.87350  2.47576
2.81202  3.07721  3.72227  5.74313  4.35586    146 k - - -
         2.68618  4.42225  2.77519  2.73123  3.46354  2.40513  3.72494
3.29354  2.67741  2.69355  4.24690  2.90347  2.73739  3.18146  2.89801
2.37887  2.77519  2.98518  4.58477  3.61503
         0.01350  4.70791  5.43026  0.61958  0.77255  0.48576  0.95510
    130   2.84846  4.41671  4.00817  2.67041  3.47538  3.89901  4.17444
2.01689  3.33752  1.84576  2.00250  3.75489  4.26411  2.80644  3.60593
3.16915  3.07910  2.62151  5.01164  3.81365    147 l - - -
         2.68618  4.42225  2.77519  2.73123  3.46354  2.40513  3.72494
3.29354  2.67741  2.69355  4.24690  2.90347  2.73739  3.18146  2.89801
2.37887  2.77519  2.98518  4.58477  3.61503
         0.01350  4.70791  5.43026  0.61958  0.77255  0.48576  0.95510
    131   3.03919  4.42138  4.74343  2.79359  3.48003  4.24114  4.58699
1.50853  4.01745  1.73767  2.57838  4.30147  4.57449  4.19922  4.13528
3.55293  3.27322  1.72861  5.13592  3.96572    148 i - - -
         2.68618  4.42225  2.77519  2.73123  3.46354  2.40513  3.72494
3.29354  2.67741  2.69355  4.24690  2.90347  2.73739  3.18146  2.89801
2.37887  2.77519  2.98518  4.58477  3.61503
         0.01350  4.70791  5.43026  0.61958  0.77255  0.48576  0.95510
    132   3.29574  5.95895  1.14116  1.97323  5.24869  3.60348  4.10734
4.79024  3.09523  4.24121  5.06060  1.88317  4.20578  2.36313  3.69788
3.16976  3.57382  4.32823  6.37983  4.85185    149 d - - -
         2.68618  4.42225  2.77519  2.73123  3.46354  2.40513  3.72494
3.29354  2.67741  2.69355  4.24690  2.90347  2.73739  3.18146  2.89801
2.37887  2.77519  2.98518  4.58477  3.61503
         0.01350  4.70791  5.43026  0.61958  0.77255  0.48576  0.95510
    133   2.77953  5.26780  3.03330  1.99307  4.60833  3.55225  2.91559
4.08640  2.18113  2.80708  4.31089  2.61057  3.94534  2.40297  2.49964
2.35858  3.00953  3.65627  5.70027  4.29881    150 e - - -
         2.68618  4.42225  2.77519  2.73123  3.46354  2.40513  3.72494
3.29354  2.67741  2.69355  4.24690  2.90347  2.73739  3.18146  2.89801
2.37887  2.77519  2.98518  4.58477  3.61503
         0.01350  4.70791  5.43026  0.61958  0.77255  0.48576  0.95510
    134   2.40432  5.24570  2.18799  2.49919  4.59400  3.55149  3.78102
4.06476  1.78798  3.56904  4.32514  3.04279  3.96832  2.88971  3.02446
2.35295  2.17045  3.64611  5.72054  4.32239    151 k - - -
         2.68618  4.42225  2.77519  2.73123  3.46354  2.40513  3.72494
3.29354  2.67741  2.69355  4.24690  2.90347  2.73739  3.18146  2.89801
2.37887  2.77519  2.98518  4.58477  3.61503
         0.01350  4.70791  5.43026  0.61958  0.77255  0.48576  0.95510
    135   3.91397  5.13887  5.78874  5.21948  1.67176  5.24356  5.39005
2.01005  5.07364  0.78178  3.15712  5.36302  5.30539  4.94911  5.02063
4.62300  4.11720  2.81037  5.43717  4.29974    152 l - - -
         2.68618  4.42225  2.77519  2.73123  3.46354  2.40513  3.72494
3.29354  2.67741  2.69355  4.24690  2.90347  2.73739  3.18146  2.89801
2.37887  2.77519  2.98518  4.58477  3.61503
         0.01350  4.70791  5.43026  0.61958  0.77255  0.48576  0.95510
    136   2.79648  4.92450  3.22903  2.28868  4.16073  3.62914  3.84358
3.56257  2.63613  2.32092  4.01367  3.18716  4.02555  3.00994  2.59485
2.44800  1.70124  3.25664  5.44578  4.13155    153 t - - -
         2.68618  4.42225  2.77519  2.73123  3.46354  2.40513  3.72494
3.29354  2.67741  2.69355  4.24690  2.90347  2.73739  3.18146  2.89801
2.37887  2.77519  2.98518  4.58477  3.61503
```

```
          0.01350  4.70791  5.43026  0.61958  0.77255  0.48576  0.95510
    137   2.04176  5.37545  2.11356  2.47595  4.71547  3.55784  3.82710
4.20092  2.61223  3.68847  4.44527  2.14187  4.00434  2.07414  3.11826
2.42410  3.12213  3.76928  5.83039  4.40979    154 a - - -
          2.68618  4.42225  2.77519  2.73123  3.46354  2.40513  3.72494
3.29354  2.67741  2.69355  4.24690  2.90347  2.73739  3.18146  2.89801
2.37887  2.77519  2.98518  4.58477  3.61503
          0.01350  4.70791  5.43026  0.61958  0.77255  0.48576  0.95510
    138   2.80819  5.13411  2.54424  1.92876  2.60973  3.58451  3.79343
3.84864  2.58275  3.40468  4.19657  3.08336  3.98997  2.19678  3.06266
2.80556  3.04142  3.48742  5.56452  2.57582    155 e - - -
          2.68618  4.42225  2.77519  2.73123  3.46354  2.40513  3.72494
3.29354  2.67741  2.69355  4.24690  2.90347  2.73739  3.18146  2.89801
2.37887  2.77519  2.98518  4.58477  3.61503
          0.01350  4.70791  5.43026  0.61958  0.77255  0.48576  0.95510
    139   2.80420  5.27883  2.52832  2.16403  4.60468  3.55222  3.76369
4.08044  2.51738  2.88658  4.32733  2.59389  3.96133  1.85813  3.01023
2.76983  2.23272  3.65959  5.72130  4.31629    156 q - - -
          2.68618  4.42225  2.77519  2.73123  3.46354  2.40513  3.72494
3.29354  2.67741  2.69355  4.24690  2.90347  2.73739  3.18146  2.89801
2.37887  2.77519  2.98518  4.58477  3.61503
          0.01350  4.70791  5.43026  0.61958  0.77255  0.48576  0.95510
    140   2.35277  5.07419  2.56662  2.22113  4.34337  3.57747  3.77557
3.00644  2.54843  2.14485  4.13595  3.08345  3.96970  2.47412  2.57219
2.77722  3.00161  3.42302  5.55476  4.19552    157 l - - -
          2.68618  4.42225  2.77519  2.73123  3.46354  2.40513  3.72494
3.29354  2.67741  2.69355  4.24690  2.90347  2.73739  3.18146  2.89801
2.37887  2.77519  2.98518  4.58477  3.61503
          0.01350  4.70791  5.43026  0.61958  0.77255  0.48576  0.95510
    141   2.04882  5.26373  2.48965  2.48528  4.60249  3.54987  3.77278
4.07708  2.22706  3.57589  4.32900  1.98328  3.96392  2.87970  3.02238
2.41815  2.53758  3.65582  5.72401  4.32102    158 n - - -
          2.68618  4.42225  2.77519  2.73123  3.46354  2.40513  3.72494
3.29354  2.67741  2.69355  4.24690  2.90347  2.73739  3.18146  2.89801
2.37887  2.77519  2.98518  4.58477  3.61503
          0.01350  4.70791  5.43026  0.61958  0.77255  0.48576  0.95510
    142   2.77840  4.50501  3.64218  3.07671  2.37217  3.75294  3.99916
2.46451  2.55208  2.71487  3.60798  2.08596  4.13278  3.33357  3.38103
2.99805  2.56369  2.77789  5.06819  2.84932    159 n - - -
          2.68618  4.42225  2.77519  2.73123  3.46354  2.40513  3.72494
3.29354  2.67741  2.69355  4.24690  2.90347  2.73739  3.18146  2.89801
2.37887  2.77519  2.98518  4.58477  3.61503
          0.01350  4.70791  5.43026  0.61958  0.77255  0.48576  0.95510
    143   3.08596  4.58845  4.30860  3.78397  3.15977  4.09983  4.20691
2.95190  3.57317  0.94481  3.58339  2.70068  4.47416  3.87946  3.76750
3.41651  3.32321  2.81902  2.98093  3.33508    160 l - - -
          2.68618  4.42225  2.77519  2.73123  3.46354  2.40513  3.72494
3.29354  2.67741  2.69355  4.24690  2.90347  2.73739  3.18146  2.89801
2.37887  2.77519  2.98518  4.58477  3.61503
          0.01350  4.70791  5.43026  0.61958  0.77255  0.48576  0.95510
    144   2.84385  5.27168  2.42309  2.50147  4.58118  3.56730  3.80626
4.04573  2.57970  2.85142  4.33431  1.57377  3.99319  2.41102  3.07027
2.81551  2.56403  3.64556  5.73008  4.33338    161 n - - -
```

2.68618 4.42225 2.77519 2.73123 3.46354 2.40513 3.72494
3.29354 2.67741 2.69355 4.24690 2.90347 2.73739 3.18146 2.89801
2.37887 2.77519 2.98518 4.58477 3.61503
0.01350 4.70791 5.43026 0.61958 0.77255 0.48576 0.95510
145 2.09303 5.29566 2.47362 2.47664 4.64019 2.80844 3.77401
4.12096 1.75182 3.60895 4.35743 2.56948 3.96581 2.87875 3.02449
2.38656 3.04868 3.69050 5.74851 4.33913 162 k - - -
2.68618 4.42225 2.77519 2.73123 3.46354 2.40513 3.72494
3.29354 2.67741 2.69355 4.24690 2.90347 2.73739 3.18146 2.89801
2.37887 2.77519 2.98518 4.58477 3.61503
0.01350 4.70791 5.43026 0.61958 0.77255 0.48576 0.95510
146 2.77434 5.13710 2.61413 1.94980 3.22645 3.56599 3.76478
3.87268 2.19130 3.41739 4.19392 3.05950 3.96140 2.88443 3.01007
2.76674 1.99646 3.49663 5.60417 3.19871 163 e - - -
2.68618 4.42225 2.77519 2.73123 3.46354 2.40513 3.72494
3.29354 2.67741 2.69355 4.24690 2.90347 2.73739 3.18146 2.89801
2.37887 2.77519 2.98518 4.58477 3.61503
0.01350 4.70791 5.43026 0.61958 0.77255 0.48576 0.95510
147 3.56949 4.82787 5.54188 4.97594 2.47299 4.93479 5.30906
1.20309 4.83345 1.26921 3.32934 5.08452 5.12639 4.86503 4.84694
4.29876 3.79670 1.88249 5.54453 4.45702 164 i - - -
2.68618 4.42225 2.77519 2.73123 3.46354 2.40513 3.72494
3.29354 2.67741 2.69355 4.24690 2.90347 2.73739 3.18146 2.89801
2.37887 2.77519 2.98518 4.58477 3.61503
0.01350 4.70791 5.43026 0.61958 0.77255 0.48576 0.95510
148 2.98454 5.54269 1.95621 1.62461 4.86974 3.56989 3.89057
4.37230 2.21021 3.84037 4.60280 2.07979 4.05430 3.00758 3.24012
2.36880 3.23179 3.92642 5.97229 4.52386 165 e - - -
2.68618 4.42225 2.77519 2.73123 3.46354 2.40513 3.72494
3.29354 2.67741 2.69355 4.24690 2.90347 2.73739 3.18146 2.89801
2.37887 2.77519 2.98518 4.58477 3.61503
0.01350 4.70791 5.43026 0.61958 0.77255 0.48576 0.95510
149 2.25249 4.90497 3.33195 2.91931 4.67661 1.35670 4.14643
4.12104 2.26044 3.70303 4.49565 3.35102 4.07773 3.30636 3.37336
1.87521 3.13364 3.65007 5.89620 4.58122 166 g - - -
2.68618 4.42225 2.77519 2.73123 3.46354 2.40513 3.72494
3.29354 2.67741 2.69355 4.24690 2.90347 2.73739 3.18146 2.89801
2.37887 2.77519 2.98518 4.58477 3.61503
0.01350 4.70791 5.43026 0.61958 0.77255 0.48576 0.95510
150 3.86951 5.10153 5.79867 5.20506 2.48517 5.21143 5.48245
1.68530 5.05815 0.77224 2.38571 5.36756 5.27389 4.93098 4.99689
4.58480 4.07162 2.80769 5.53198 4.55407 167 l - - -
2.68618 4.42225 2.77519 2.73123 3.46354 2.40513 3.72494
3.29354 2.67741 2.69355 4.24690 2.90347 2.73739 3.18146 2.89801
2.37887 2.77519 2.98518 4.58477 3.61503
0.01350 4.70791 5.43026 0.61958 0.77255 0.48576 0.95510
151 2.85870 5.11429 3.20261 2.64937 3.12558 3.64638 3.82028
3.81378 1.88233 3.37636 4.18676 3.16382 4.03998 1.53997 2.92457
2.87144 2.57892 3.47291 5.57256 4.23562 168 q - - -
2.68618 4.42225 2.77519 2.73123 3.46354 2.40513 3.72494
3.29354 2.67741 2.69355 4.24690 2.90347 2.73739 3.18146 2.89801
2.37887 2.77519 2.98518 4.58477 3.61503
0.01350 4.70791 5.43026 0.61958 0.77255 0.48576 0.95510

152 2.87473 5.37511 1.86262 2.47504 4.71307 2.84996 3.81801
4.19825 1.89490 3.68282 4.43786 2.49029 4.00059 2.92788 3.09593
2.82871 2.19806 3.76610 5.82184 4.40264 169 d - - -
2.68618 4.42225 2.77519 2.73123 3.46354 2.40513 3.72494
3.29354 2.67741 2.69355 4.24690 2.90347 2.73739 3.18146 2.89801
2.37887 2.77519 2.98518 4.58477 3.61503
0.01350 4.70791 5.43026 0.61958 0.77255 0.48576 0.95510
153 2.07827 5.00620 2.56183 2.61313 4.27868 3.58951 3.83454
3.69358 2.64026 2.32374 4.10721 2.26374 4.00306 2.98315 3.10803
2.81920 2.18006 3.35959 5.53516 4.19350 170 a - - -
2.68618 4.42225 2.77519 2.73123 3.46354 2.40513 3.72494
3.29354 2.67741 2.69355 4.24690 2.90347 2.73739 3.18146 2.89801
2.37887 2.77519 2.98518 4.58477 3.61503
0.01350 4.70791 5.43026 0.61958 0.77255 0.48576 0.95510
154 2.35704 4.26002 4.57951 3.98408 3.34327 4.00922 4.33991
2.13158 3.82334 1.44054 2.57261 4.08895 4.36819 3.99876 3.91989
2.64287 3.08674 2.06978 4.90350 3.72523 171 l - - -
2.68618 4.42225 2.77519 2.73123 3.46354 2.40513 3.72494
3.29354 2.67741 2.69355 4.24690 2.90347 2.73739 3.18146 2.89801
2.37887 2.77519 2.98518 4.58477 3.61503
0.01350 4.70791 5.43026 0.61958 0.77255 0.48576 0.95510
155 2.11429 5.08701 3.09779 2.54991 4.37670 2.92616 3.78019
3.81663 2.55214 3.37718 4.16249 2.54614 3.96826 2.43480 3.02860
2.11931 2.56958 3.45172 5.57885 3.08322 172 a - - -
2.68618 4.42225 2.77519 2.73123 3.46354 2.40513 3.72494
3.29354 2.67741 2.69355 4.24690 2.90347 2.73739 3.18146 2.89801
2.37887 2.77519 2.98518 4.58477 3.61503
0.01350 4.70791 5.43026 0.61958 0.77255 0.48576 0.95510
156 2.76980 5.02347 2.60116 2.58006 4.27736 3.58809 3.01613
2.85990 2.57129 2.35771 4.08979 2.58310 3.97901 2.92679 2.26098
2.78946 2.55689 3.36415 5.51433 4.16725 173 r - - -
2.68618 4.42225 2.77519 2.73123 3.46354 2.40513 3.72494
3.29354 2.67741 2.69355 4.24690 2.90347 2.73739 3.18146 2.89801
2.37887 2.77519 2.98518 4.58477 3.61503
0.01350 4.70791 5.43026 0.61958 0.77255 0.48576 0.95510
157 3.56389 4.86148 5.11924 4.66300 1.49467 4.59124 4.12946
3.13737 4.47980 2.09169 3.79698 4.50112 4.89973 4.48524 4.48056
3.92608 3.78738 2.31339 4.32519 1.19854 174 y - - -
2.68618 4.42225 2.77519 2.73123 3.46354 2.40513 3.72494
3.29354 2.67741 2.69355 4.24690 2.90347 2.73739 3.18146 2.89801
2.37887 2.77519 2.98518 4.58477 3.61503
0.01350 4.70791 5.43026 0.61958 0.77255 0.48576 0.95510
158 2.78169 5.13037 3.09448 2.24651 4.41958 3.57693 3.76817
2.92172 2.51745 3.40960 4.18930 3.07363 3.96954 1.98562 2.50774
2.40569 2.18511 3.49098 5.59773 4.23056 175 q - - -
2.68618 4.42225 2.77519 2.73123 3.46354 2.40513 3.72494
3.29354 2.67741 2.69355 4.24690 2.90347 2.73739 3.18146 2.89801
2.37887 2.77519 2.98518 4.58477 3.61503
0.01350 4.70791 5.43026 0.61958 0.77255 0.48576 0.95510
159 2.81773 5.18989 3.05357 2.22989 4.50107 3.58070 3.79289
3.95014 2.53718 3.48453 4.26151 1.61015 3.98939 2.91224 2.49135
2.38123 3.05219 2.76598 5.66017 4.28650 176 n - - -
2.68618 4.42225 2.77519 2.73123 3.46354 2.40513 3.72494
3.29354 2.67741 2.69355 4.24690 2.90347 2.73739 3.18146 2.89801
2.37887 2.77519 2.98518 4.58477 3.61503

```
          0.01350  4.70791  5.43026  0.61958  0.77255  0.48576  0.95510
    160   1.63834  4.46694  2.93592  3.22452  3.61684  3.81928  4.09882
2.32806  3.17213  2.24939  3.58146  3.60328  4.19987  3.46413  3.50324
3.07650  2.50259  2.25772  5.09348  3.86760    177 a - - -
          2.68618  4.42225  2.77519  2.73123  3.46354  2.40513  3.72494
3.29354  2.67741  2.69355  4.24690  2.90347  2.73739  3.18146  2.89801
2.37887  2.77519  2.98518  4.58477  3.61503
          0.01350  4.70791  5.43026  0.61958  0.77255  0.48576  0.95510
    161   3.38500  4.66704  5.35565  4.79131  2.52143  4.72906  5.13329
1.08230  4.64280  2.14582  2.52368  4.87457  4.98567  4.74685  4.68544
4.08155  3.62010  1.56109  5.48344  4.34766    178 i - - -
          2.68618  4.42225  2.77519  2.73123  3.46354  2.40513  3.72494
3.29354  2.67741  2.69355  4.24690  2.90347  2.73739  3.18146  2.89801
2.37887  2.77519  2.98518  4.58477  3.61503
          0.01350  4.70791  5.43026  0.61958  0.77255  0.48576  0.95510
    162   2.80865  5.24384  1.92316  2.49952  4.55833  3.56470  3.77100
2.59059  2.51896  3.53418  4.29628  2.50345  3.97097  2.20757  2.50158
2.78216  3.04113  3.61859  5.69190  4.29977    179 d - - -
          2.68618  4.42225  2.77519  2.73123  3.46354  2.40513  3.72494
3.29354  2.67741  2.69355  4.24690  2.90347  2.73739  3.18146  2.89801
2.37887  2.77519  2.98518  4.58477  3.61503
          0.01350  4.70791  5.43026  0.61958  0.77255  0.48576  0.95510
    163   2.37453  5.14000  2.03324  2.18051  4.43014  3.56496  3.76646
3.05569  2.53173  3.42209  4.19833  2.53731  3.96173  2.88568  3.01409
2.76709  2.52389  2.89296  5.60995  4.23444    180 d - - -
          2.68618  4.42225  2.77519  2.73123  3.46354  2.40513  3.72494
3.29354  2.67741  2.69355  4.24690  2.90347  2.73739  3.18146  2.89801
2.37887  2.77519  2.98518  4.58477  3.61503
          0.01350  4.70791  5.43026  0.61958  0.77255  0.48576  0.95510
    164   1.67692  4.40902  3.84212  3.28216  2.73895  3.77666  4.09715
2.05302  3.22074  2.63785  3.54443  2.74321  4.17700  3.50074  3.52524
2.48381  3.01760  2.67851  5.02895  3.80866    181 a - - -
          2.68618  4.42225  2.77519  2.73123  3.46354  2.40513  3.72494
3.29354  2.67741  2.69355  4.24690  2.90347  2.73739  3.18146  2.89801
2.37887  2.77519  2.98518  4.58477  3.61503
          0.01350  4.70791  5.43026  0.61958  0.77255  0.48576  0.95510
    165   2.81119  5.02256  2.53303  2.61676  4.27574  3.60600  3.84022
2.50293  2.64183  3.28641  4.10679  3.13947  4.01650  1.90622  3.10444
2.83986  1.93928  3.34369  5.53709  4.19643    182 q - - -
          2.68618  4.42225  2.77519  2.73123  3.46354  2.40513  3.72494
3.29354  2.67741  2.69355  4.24690  2.90347  2.73739  3.18146  2.89801
2.37887  2.77519  2.98518  4.58477  3.61503
          0.01350  4.70791  5.43026  0.61958  0.77255  0.48576  0.95510
    166   2.77980  2.57463  4.53232  3.93181  2.66022  2.87917  4.23531
2.21919  3.76035  2.38030  3.30749  4.01245  4.28905  3.92923  3.84161
2.59242  3.01209  2.43921  2.52701  2.72371    183 i - - -
          2.68618  4.42225  2.77519  2.73123  3.46354  2.40513  3.72494
3.29354  2.67741  2.69355  4.24690  2.90347  2.73739  3.18146  2.89801
2.37887  2.77519  2.98518  4.58477  3.61503
          0.10856  4.70791  2.36608  0.61958  0.77255  0.48576  0.95510
    167   2.34478  5.16817  3.00617  2.18408  4.47965  3.52329  2.90709
3.94207  2.46980  3.45913  4.21981  2.48914  3.91989  2.18995  2.95492
2.36190  2.97544  2.80500  5.62217  4.23275    184 e - - -
```

```
          2.68618  4.42225  2.77519  2.73123  3.46354  2.40513  3.72494
3.29354  2.67741  2.69355  4.24690  2.90347  2.73739  3.18146  2.89801
2.37887  2.77519  2.98518  4.58477  3.61503
          0.01483  4.61419  5.33653  0.61958  0.77255  0.65835  0.72920
    168   2.80823  5.10288  3.07915  2.56287  4.38151  1.87191  2.80415
3.81996  2.14645  2.66459  4.17588  2.57077  3.98417  2.91189  2.94526
2.81144  3.03897  3.46524  5.57086  4.22023    185 g - - -
          2.68618  4.42225  2.77519  2.73123  3.46354  2.40513  3.72494
3.29354  2.67741  2.69355  4.24690  2.90347  2.73739  3.18146  2.89801
2.37887  2.77519  2.98518  4.58477  3.61503
          0.01483  4.61419  5.33653  0.61958  0.77255  0.65835  0.72920
    169   2.32406  5.18933  2.99284  2.18043  4.51064  2.80896  2.95831
3.97801  2.46886  3.48646  4.24267  3.00045  3.91955  2.09716  2.95591
2.38814  2.53110  3.56850  5.64218  4.24776    186 q - - -
          2.68618  4.42225  2.77519  2.73123  3.46354  2.40513  3.72494
3.29354  2.67741  2.69355  4.24690  2.90347  2.73739  3.18146  2.89801
2.37887  2.77519  2.98518  4.58477  3.61503
          0.11501  4.61419  2.31532  0.61958  0.77255  0.65835  0.72920
    170   2.70217  4.76779  3.19589  2.63857  2.96664  2.74323  3.78226
3.37272  2.28124  3.01483  3.85375  3.13794  3.95407  2.51851  3.06488
2.34223  2.51407  2.57046  5.29972  3.99553    187 k - - -
          2.68618  4.42225  2.77519  2.73123  3.46354  2.40513  3.72494
3.29354  2.67741  2.69355  4.24690  2.90347  2.73739  3.18146  2.89801
2.37887  2.77519  2.98518  4.58477  3.61503
          0.01638  4.51556  5.23790  0.61958  0.77255  0.79943  0.59708
    171   2.74708  5.11974  2.46498  2.45309  2.99873  3.50224  3.72680
3.86263  2.14217  3.40389  4.18351  2.47393  2.20535  2.84951  2.98563
2.73330  2.98220  3.48338  5.58603  4.20410    188 k - - -
          2.68618  4.42225  2.77519  2.73123  3.46354  2.40513  3.72494
3.29354  2.67741  2.69355  4.24690  2.90347  2.73739  3.18146  2.89801
2.37887  2.77519  2.98518  4.58477  3.61503
          0.01638  4.51556  5.23790  0.61958  0.77255  0.54272  0.87028
    172   2.73466  4.81062  3.21866  2.33780  2.94648  2.89461  3.80894
3.41450  2.28105  2.52999  3.89152  2.59250  3.98358  2.99037  3.09301
2.80686  2.51438  2.62748  5.33708  4.03031    189 k - - -
          2.68618  4.42225  2.77519  2.73123  3.46354  2.40513  3.72494
3.29354  2.67741  2.69355  4.24690  2.90347  2.73739  3.18146  2.89801
2.37887  2.77519  2.98518  4.58477  3.61503
          0.10140  4.61419  2.44744  0.61958  0.77255  0.65835  0.72920
    173   2.24836  4.53484  3.45170  2.88945  3.71227  3.63418  3.90002
3.06707  2.84562  2.36721  3.65234  3.32842  2.91516  3.17228  2.66898
2.87555  2.10356  2.35897  5.12380  3.87041    190 t - - -
          2.68618  4.42225  2.77519  2.73123  3.46354  2.40513  3.72494
3.29354  2.67741  2.69355  4.24690  2.90347  2.73739  3.18146  2.89801
2.37887  2.77519  2.98518  4.58477  3.61503
          0.01616  4.52895  5.25130  0.61958  0.77255  0.78216  0.61141
    174   2.70935  4.53796  2.28597  2.86842  3.68893  3.64572  3.88455
2.46676  2.83882  2.75299  2.87117  2.68506  4.02879  3.15769  3.23126
2.87898  2.94122  2.41741  5.10399  2.87143    191 d - - -
          2.68637  4.42244  2.77467  2.73101  3.46304  2.40502  3.72513
3.29373  2.67759  2.69340  4.24708  2.90326  2.73758  3.18165  2.89819
2.37877  2.77538  2.98537  4.58496  3.61522
          0.50812  1.25686  2.17308  0.92884  0.50255  0.78216  0.61141
```

```
    175   2.78566  5.22866  3.01288  2.08661  4.58716  3.52495  3.67567
4.04238  1.97556  3.51418  4.27780  2.99817  3.91928  2.05586  2.28548
2.28459  3.00717  3.62917  5.62987  4.26689    195 k - - -
          2.68652  4.41310  2.77587  2.73150  3.46421  2.40521  3.72562
3.29421  2.67731  2.69383  4.24757  2.90287  2.73589  3.18099  2.89868
2.37925  2.77443  2.98586  4.58544  3.61570
          0.77543  0.86035  2.15005  1.85448  0.17024  0.68824  0.69807
    176   2.66820  2.97545  3.59286  3.02400  3.50128  3.65662  3.91774
2.33485  2.44738  2.21035  3.47087  2.74546  4.03741  3.26634  3.29648
2.90772  2.43303  2.61789  4.95422  3.72518    208 l - - -
          2.68608  4.41855  2.77548  2.73151  3.46382  2.40541  3.72523
3.29382  2.67769  2.69383  4.24718  2.90375  2.73657  3.18067  2.89829
2.37890  2.77471  2.98501  4.58505  3.61531
          0.57098  0.84610  5.12550  1.04355  0.43418  0.44551  1.02304
    177   2.78380  4.68422  3.42637  2.86377  3.84560  3.67941  3.90127
3.22025  1.82810  2.91195  3.78735  3.32124  4.06940  3.14068  3.13324
2.91920  2.18397  2.41681  5.22643  2.36756    212 k - - -
          2.68618  4.42225  2.77519  2.73123  3.46354  2.40513  3.72494
3.29354  2.67741  2.69355  4.24690  2.90347  2.73739  3.18146  2.89801
2.37887  2.77519  2.98518  4.58477  3.61503
          0.01472  4.62188  5.34423  0.61958  0.77255  0.39730  1.11515
    178   2.35201  5.24381  2.20705  2.51958  4.64420  2.76407  3.82467
4.12018  2.59827  3.62420  4.38092  2.12260  3.98489  2.93675  3.09348
2.08822  2.48433  3.68791  5.77515  4.37355    213 s - - -
          2.68618  4.42225  2.77519  2.73123  3.46354  2.40513  3.72494
3.29354  2.67741  2.69355  4.24690  2.90347  2.73739  3.18146  2.89801
2.37887  2.77519  2.98518  4.58477  3.61503
          0.01350  4.70791  5.43026  0.61958  0.77255  0.48576  0.95510
    179   2.05585  5.21366  1.95202  2.51565  4.50624  3.56935  2.44456
3.96572  2.58170  2.77234  4.27907  2.57518  3.98892  2.92401  3.07001
2.80797  3.06249  3.58069  5.68021  4.29308    214 d - - -
          2.68618  4.42225  2.77519  2.73123  3.46354  2.40513  3.72494
3.29354  2.67741  2.69355  4.24690  2.90347  2.73739  3.18146  2.89801
2.37887  2.77519  2.98518  4.58477  3.61503
          0.01350  4.70791  5.43026  0.61958  0.77255  0.48576  0.95510
    180   2.83244  4.31328  4.26032  3.67251  3.37127  3.93215  4.22320
1.94521  2.25029  1.87046  2.63721  3.89574  4.29474  3.77329  3.72196
3.21843  3.06341  2.52482  4.91163  2.40393    215 l - - -
          2.68618  4.42225  2.77519  2.73123  3.46354  2.40513  3.72494
3.29354  2.67741  2.69355  4.24690  2.90347  2.73739  3.18146  2.89801
2.37887  2.77519  2.98518  4.58477  3.61503
          0.21526  4.70791  1.68934  0.61958  0.77255  0.48576  0.95510
    181   2.70796  4.49995  2.80686  2.91266  3.64899  3.65885  3.90208
2.14113  2.39478  2.30720  2.83454  3.34800  4.04004  3.19090  3.25311
2.89584  2.42064  2.75063  5.07709  3.82905    216 i - - -
          2.68618  4.42225  2.77519  2.73123  3.46354  2.40513  3.72494
3.29354  2.67741  2.69355  4.24690  2.90347  2.73739  3.18146  2.89801
2.37887  2.77519  2.98518  4.58477  3.61503
          0.01649  4.50914  5.23149  0.61958  0.77255  0.80751  0.59053
    182   2.30559  5.01433  3.05624  2.50091  3.05216  3.51950  3.71467
3.72490  1.92393  3.29001  4.08140  2.54176  3.91254  2.84426  2.51573
2.35994  2.94700  3.36951  5.49455  4.13977    217 k - - -
          2.68618  4.42225  2.77519  2.73123  3.46354  2.40513  3.72494
3.29354  2.67741  2.69355  4.24690  2.90347  2.73739  3.18146  2.89801
2.37887  2.77519  2.98518  4.58477  3.61503
```

0.01649 4.50914 5.23149 0.61958 0.77255 0.80751 0.59053
183 3.30330 4.58518 5.22975 4.67151 2.47026 4.65323 5.05016
1.72903 4.53434 1.67998 3.34474 4.77925 4.90427 4.64777 4.59652
4.00699 3.53894 1.11931 5.40191 4.25084 218 v - - -
2.68618 4.42225 2.77519 2.73123 3.46354 2.40513 3.72494
3.29354 2.67741 2.69355 4.24690 2.90347 2.73739 3.18146 2.89801
2.37887 2.77519 2.98518 4.58477 3.61503
0.01649 4.50914 5.23149 0.61958 0.77255 0.80751 0.59053
184 2.37129 5.12463 2.53823 2.43212 4.43530 3.48960 3.68472
3.89613 2.43518 3.41584 4.17750 2.48222 3.88502 2.36903 2.50398
2.68660 2.47413 2.81961 5.58099 4.19329 219 q - - -
2.68618 4.42225 2.77519 2.73123 3.46354 2.40513 3.72494
3.29354 2.67741 2.69355 4.24690 2.90347 2.73739 3.18146 2.89801
2.37887 2.77519 2.98518 4.58477 3.61503
0.01649 4.50914 5.23149 0.61958 0.77255 0.54040 0.87350
185 2.76621 5.24009 2.47315 2.16091 4.57394 2.81926 3.72982
4.05058 2.48268 3.54454 4.29500 2.99128 3.92558 2.83492 2.47571
1.87098 2.47306 3.62707 5.68884 4.28376 220 s - - -
2.68618 4.42225 2.77519 2.73123 3.46354 2.40513 3.72494
3.29354 2.67741 2.69355 4.24690 2.90347 2.73739 3.18146 2.89801
2.37887 2.77519 2.98518 4.58477 3.61503
0.01485 4.61273 5.33507 0.61958 0.77255 0.66070 0.72668
186 2.39385 5.22732 3.01037 2.15098 4.56441 3.52632 3.70835
4.03773 1.90648 3.52512 4.27265 2.99923 3.91970 2.37067 2.51484
2.37568 2.49674 3.61422 5.66145 4.26537 221 k - - -
2.68618 4.42225 2.77519 2.73123 3.46354 2.40513 3.72494
3.29354 2.67741 2.69355 4.24690 2.90347 2.73739 3.18146 2.89801
2.37887 2.77519 2.98518 4.58477 3.61503
0.01485 4.61273 5.33507 0.61958 0.77255 0.66070 0.72668
187 3.02912 4.38739 4.86992 4.28259 1.55709 4.20537 4.42223
2.07782 4.09859 1.78072 3.27397 4.31102 4.52987 4.20722 4.13188
3.52296 3.25810 2.03398 4.82559 2.66019 222 f - - -
2.68618 4.42225 2.77519 2.73123 3.46354 2.40513 3.72494
3.29354 2.67741 2.69355 4.24690 2.90347 2.73739 3.18146 2.89801
2.37887 2.77519 2.98518 4.58477 3.61503
0.01485 4.61273 5.33507 0.61958 0.77255 0.66070 0.72668
188 2.75236 5.17965 1.98317 2.12973 3.24072 3.52399 3.72490
3.95255 2.19475 2.77954 4.23068 3.00483 3.92422 2.39930 2.96446
2.72950 2.98448 3.55146 5.63174 4.24062 223 d - - -
2.68618 4.42225 2.77519 2.73123 3.46354 2.40513 3.72494
3.29354 2.67741 2.69355 4.24690 2.90347 2.73739 3.18146 2.89801
2.37887 2.77519 2.98518 4.58477 3.61503
0.01485 4.61273 5.33507 0.61958 0.77255 0.66070 0.72668
189 2.32186 5.00335 2.59664 2.55223 4.29679 3.54013 3.77706
2.85336 2.56604 3.30678 4.10432 2.51088 3.95007 2.91423 3.03844
1.86307 2.54871 3.37306 5.52671 4.17471 224 s - - -
2.68618 4.42225 2.77519 2.73123 3.46354 2.40513 3.72494
3.29354 2.67741 2.69355 4.24690 2.90347 2.73739 3.18146 2.89801
2.37887 2.77519 2.98518 4.58477 3.61503
0.01485 4.61273 5.33507 0.61958 0.77255 0.66070 0.72668
190 1.77758 4.37724 4.38857 3.82307 3.48801 4.01753 4.41062
2.05062 3.70541 1.48583 3.43459 4.03569 4.40204 3.93760 3.89858
3.32835 2.51200 2.31529 5.09265 3.90808 225 l - - -

2.68618 4.42225 2.77519 2.73123 3.46354 2.40513 3.72494
3.29354 2.67741 2.69355 4.24690 2.90347 2.73739 3.18146 2.89801
2.37887 2.77519 2.98518 4.58477 3.61503
0.01485 4.61273 5.33507 0.61958 0.77255 0.39012 1.13004
191 2.87701 5.36461 1.81191 2.48231 4.70805 2.81274 3.82689
4.19132 2.60713 3.68073 4.43852 1.89215 4.00476 2.93862 2.57980
2.83360 2.51973 3.76136 5.82342 4.40629 226 d - - -
2.68618 4.42225 2.77519 2.73123 3.46354 2.40513 3.72494
3.29354 2.67741 2.69355 4.24690 2.90347 2.73739 3.18146 2.89801
2.37887 2.77519 2.98518 4.58477 3.61503
0.11004 4.70791 2.35204 0.61958 0.77255 0.48576 0.95510
192 2.72704 4.88420 3.21775 2.78927 4.54579 2.61222 4.02517
3.98839 2.83435 3.57135 4.36692 3.24611 4.01108 2.41074 3.27436
1.24004 2.43053 3.54899 5.77330 4.44658 227 s - - -
2.68618 4.42225 2.77519 2.73123 3.46354 2.40513 3.72494
3.29354 2.67741 2.69355 4.24690 2.90347 2.73739 3.18146 2.89801
2.37887 2.77519 2.98518 4.58477 3.61503
0.01485 4.61273 5.33507 0.61958 0.77255 0.39012 1.13004
193 2.78129 4.29494 4.12049 3.54023 2.69542 3.86194 4.16141
1.77538 3.43851 2.48632 3.40867 3.79583 4.23512 2.91136 3.65966
2.19794 2.53090 2.14003 4.90900 3.71048 228 i - - -
2.68618 4.42225 2.77519 2.73123 3.46354 2.40513 3.72494
3.29354 2.67741 2.69355 4.24690 2.90347 2.73739 3.18146 2.89801
2.37887 2.77519 2.98518 4.58477 3.61503
0.01350 4.70791 5.43026 0.61958 0.77255 0.48576 0.95510
194 3.15329 4.50007 5.04804 4.44804 1.77992 4.34339 4.60369
1.87294 4.25642 1.49557 2.42547 4.47658 4.64376 4.33292 4.26438
3.66448 3.37853 2.60195 4.98100 2.70154 229 l - - -
2.68618 4.42225 2.77519 2.73123 3.46354 2.40513 3.72494
3.29354 2.67741 2.69355 4.24690 2.90347 2.73739 3.18146 2.89801
2.37887 2.77519 2.98518 4.58477 3.61503
0.01350 4.70791 5.43026 0.61958 0.77255 0.48576 0.95510
195 2.82789 4.81394 3.27573 2.38633 4.03317 3.66618 3.94300
2.24717 2.81240 3.06985 3.93430 2.60846 4.08640 3.15370 3.23417
2.93006 1.53192 3.09234 5.39034 4.10089 230 t - - -
2.68618 4.42225 2.77519 2.73123 3.46354 2.40513 3.72494
3.29354 2.67741 2.69355 4.24690 2.90347 2.73739 3.18146 2.89801
2.37887 2.77519 2.98518 4.58477 3.61503
0.01350 4.70791 5.43026 0.61958 0.77255 0.48576 0.95510
196 2.79035 5.20545 2.53601 2.50091 4.51569 3.55814 3.76807
3.97658 2.52786 2.85341 4.26360 2.20103 3.96285 2.50220 3.01506
2.34720 1.98362 3.57891 5.66646 4.27728 231 t - - -
2.68618 4.42225 2.77519 2.73123 3.46354 2.40513 3.72494
3.29354 2.67741 2.69355 4.24690 2.90347 2.73739 3.18146 2.89801
2.37887 2.77519 2.98518 4.58477 3.61503
0.01350 4.70791 5.43026 0.61958 0.77255 0.48576 0.95510
197 2.13442 5.27646 2.51672 2.46340 4.61859 2.89864 3.02471
4.10204 2.14794 3.57982 4.31854 2.57119 3.93858 2.41838 2.51429
2.73648 3.00475 3.66566 5.71000 4.30129 232 a - - -
2.68618 4.42225 2.77519 2.73123 3.46354 2.40513 3.72494
3.29354 2.67741 2.69355 4.24690 2.90347 2.73739 3.18146 2.89801
2.37887 2.77519 2.98518 4.58477 3.61503
0.01350 4.70791 5.43026 0.61958 0.77255 0.48576 0.95510

```
    198   3.10675  4.49549  4.80074  2.90005  2.49048  4.27655  4.58402
2.11919  4.05401  1.08178  2.55263  4.34324  4.58649  4.19055  4.14418
3.58559  3.33309  2.57652  5.06816  3.93140    233 l - - -
          2.68618  4.42225  2.77519  2.73123  3.46354  2.40513  3.72494
3.29354  2.67741  2.69355  4.24690  2.90347  2.73739  3.18146  2.89801
2.37887  2.77519  2.98518  4.58477  3.61503
          0.01350  4.70791  5.43026  0.61958  0.77255  0.48576  0.95510
    199   2.27338  4.77754  3.37324  2.85575  4.15937  3.57630  3.99272
2.65723  2.85500  3.20566  4.04501  2.63394  1.83559  3.18957  3.28385
2.02123  3.03993  3.22938  5.49013  4.19877    234 p - - -
          2.68618  4.42225  2.77519  2.73123  3.46354  2.40513  3.72494
3.29354  2.67741  2.69355  4.24690  2.90347  2.73739  3.18146  2.89801
2.37887  2.77519  2.98518  4.58477  3.61503
          0.01350  4.70791  5.43026  0.61958  0.77255  0.48576  0.95510
    200   2.78310  5.15456  2.23215  2.24450  4.44419  3.56646  2.19067
2.93994  2.53350  3.43552  4.21113  3.05785  3.96526  2.47956  3.01535
2.77272  3.01520  2.81849  5.62030  4.24311    235 h - - -
          2.68618  4.42225  2.77519  2.73123  3.46354  2.40513  3.72494
3.29354  2.67741  2.69355  4.24690  2.90347  2.73739  3.18146  2.89801
2.37887  2.77519  2.98518  4.58477  3.61503
          0.01350  4.70791  5.43026  0.61958  0.77255  0.48576  0.95510
    201   3.82611  5.07206  5.58028  5.06120  0.87049  5.07193  4.98279
2.09151  4.90343  1.55110  3.30683  5.10923  5.22203  4.85051  4.88971
4.44281  4.04106  2.75685  5.10774  3.70018    236 f - - -
          2.68618  4.42225  2.77519  2.73123  3.46354  2.40513  3.72494
3.29354  2.67741  2.69355  4.24690  2.90347  2.73739  3.18146  2.89801
2.37887  2.77519  2.98518  4.58477  3.61503
          0.01350  4.70791  5.43026  0.61958  0.77255  0.48576  0.95510
    202   3.03739  4.52350  4.36876  3.78070  3.49291  4.14428  4.42563
1.59123  1.95983  1.69499  2.63812  4.05637  4.48110  3.88984  3.80804
3.43464  3.26611  2.50556  5.14793  3.97809    237 i - - -
          2.68618  4.42225  2.77519  2.73123  3.46354  2.40513  3.72494
3.29354  2.67741  2.69355  4.24690  2.90347  2.73739  3.18146  2.89801
2.37887  2.77519  2.98518  4.58477  3.61503
          0.01350  4.70791  5.43026  0.61958  0.77255  0.48576  0.95510
    203   2.85018  5.35142  2.41637  2.10876  4.68600  3.55352  3.79883
4.17101  2.57162  3.65604  4.40758  1.98184  2.90544  2.90635  3.07504
2.35403  2.21723  3.73903  5.79532  4.37708    238 n - - -
          2.68679  4.42325  2.77471  2.73116  3.46312  2.40583  3.72594
3.29383  2.67771  2.69382  4.24632  2.90093  2.73614  3.18194  2.89900
2.37915  2.77471  2.98566  4.58108  3.61603
          0.39265  1.13832  5.43026  2.22057  0.11490  0.48576  0.95510
    204   2.39108  5.26681  2.58282  2.14963  4.60470  2.87869  3.73559
4.08591  2.47578  3.56939  4.31079  3.01279  3.93943  2.42079  2.57575
2.11440  2.50655  3.65389  5.70448  4.29705    253 s - - -
          2.68618  4.42225  2.77519  2.73123  3.46354  2.40513  3.72494
3.29354  2.67741  2.69355  4.24690  2.90347  2.73739  3.18146  2.89801
2.37887  2.77519  2.98518  4.58477  3.61503
          0.01350  4.70791  5.43026  0.61958  0.77255  0.48576  0.95510
    205   2.82626  5.29028  2.17878  2.14549  4.61338  2.75722  3.78708
4.08714  2.55579  3.58950  4.34721  2.17286  3.97647  2.89693  3.05315
2.79200  2.27211  2.83329  5.74188  4.33598    254 e - - -
          2.68618  4.42225  2.77519  2.73123  3.46354  2.40513  3.72494
3.29354  2.67741  2.69355  4.24690  2.90347  2.73739  3.18146  2.89801
2.37887  2.77519  2.98518  4.58477  3.61503
```

```
          0.01350  4.70791  5.43026  0.61958  0.77255  0.48576  0.95510
    206   2.81035  4.56471  3.62452  3.06090  2.33050  3.76692  2.92236
3.07653  3.00586  2.77427  3.66809  3.47495  4.14695  3.32151  2.68444
2.55102  2.55373  2.84721  5.02710  2.06599    255 y - - -
          2.68618  4.42225  2.77519  2.73123  3.46354  2.40513  3.72494
3.29354  2.67741  2.69355  4.24690  2.90347  2.73739  3.18146  2.89801
2.37887  2.77519  2.98518  4.58477  3.61503
          0.01350  4.70791  5.43026  0.61958  0.77255  0.48576  0.95510
    207   2.77026  4.92625  2.66703  2.26129  4.15254  3.60862  2.89854
2.81945  2.63306  3.17937  4.00150  3.15160  3.99966  2.97939  3.09139
2.44264  2.52849  2.10098  5.43850  4.11334    256 v - - -
          2.68618  4.42225  2.77519  2.73123  3.46354  2.40513  3.72494
3.29354  2.67741  2.69355  4.24690  2.90347  2.73739  3.18146  2.89801
2.37887  2.77519  2.98518  4.58477  3.61503
          0.01350  4.70791  5.43026  0.61958  0.77255  0.48576  0.95510
    208   2.34090  4.82486  3.28186  2.38745  4.02934  3.64250  3.85848
3.40966  2.06115  2.25410  3.91019  3.22022  4.03128  3.04699  3.13288
2.85855  2.16363  2.61932  5.35889  4.05921    257 k - - -
          2.68618  4.42225  2.77519  2.73123  3.46354  2.40513  3.72494
3.29354  2.67741  2.69355  4.24690  2.90347  2.73739  3.18146  2.89801
2.37887  2.77519  2.98518  4.58477  3.61503
          0.01350  4.70791  5.43026  0.61958  0.77255  0.48576  0.95510
    209   2.37766  4.28175  4.46027  3.87040  2.56952  2.83191  4.29339
2.64809  3.72525  1.30318  2.59841  4.01734  4.33490  3.91442  3.85367
3.27080  3.07937  2.49272  4.89073  3.70917    258 l - - -
          2.68618  4.42225  2.77519  2.73123  3.46354  2.40513  3.72494
3.29354  2.67741  2.69355  4.24690  2.90347  2.73739  3.18146  2.89801
2.37887  2.77519  2.98518  4.58477  3.61503
          0.01350  4.70791  5.43026  0.61958  0.77255  0.48576  0.95510
    210   2.27821  2.99837  3.50702  2.96731  4.09806  2.20770  2.80414
3.49082  2.94581  3.15558  4.00308  3.38710  4.06446  3.27297  3.35697
2.32045  1.88470  3.17277  5.45565  4.18417    259 t - - -
          2.68618  4.42225  2.77519  2.73123  3.46354  2.40513  3.72494
3.29354  2.67741  2.69355  4.24690  2.90347  2.73739  3.18146  2.89801
2.37887  2.77519  2.98518  4.58477  3.61503
          0.01350  4.70791  5.43026  0.61958  0.77255  0.48576  0.95510
    211   2.41015  2.84422  4.77314  4.18637  2.62102  4.12689  4.49327
2.57958  4.01465  1.03462  3.31777  4.25788  4.48880  4.16144  4.08473
3.44903  3.22075  2.47084  4.99308  3.81359    260 l - - -
          2.68618  4.42225  2.77519  2.73123  3.46354  2.40513  3.72494
3.29354  2.67741  2.69355  4.24690  2.90347  2.73739  3.18146  2.89801
2.37887  2.77519  2.98518  4.58477  3.61503
          0.01350  4.70791  5.43026  0.61958  0.77255  0.48576  0.95510
    212   4.19156  5.72584  4.88068  4.87152  5.69429  4.37180  5.80197
5.55614  5.01783  5.03479  6.16112  5.07459  0.12859  5.36839  5.09978
4.39831  4.69765  5.08592  6.56371  5.84517    261 P - - -
          2.68618  4.42225  2.77519  2.73123  3.46354  2.40513  3.72494
3.29354  2.67741  2.69355  4.24690  2.90347  2.73739  3.18146  2.89801
2.37887  2.77519  2.98518  4.58477  3.61503
          0.01350  4.70791  5.43026  0.61958  0.77255  0.48576  0.95510
    213   3.41654  4.77629  4.97604  4.43331  3.02604  4.53080  4.47869
2.81379  4.24265  1.51645  2.11433  4.51020  4.81570  4.36411  4.32345
3.85610  3.63963  2.84119  4.78538  1.21877    262 y - - -
```

```
         2.68618  4.42225  2.77519  2.73123  3.46354  2.40513  3.72494
3.29354  2.67741  2.69355  4.24690  2.90347  2.73739  3.18146  2.89801
2.37887  2.77519  2.98518  4.58477  3.61503
         0.01350  4.70791  5.43026  0.61958  0.77255  0.48576  0.95510
    214  4.77206  5.70402  5.57146  5.51015  1.41603  5.23872  3.89445
4.32664  5.29295  3.46789  4.85181  4.78238  5.49858  4.95500  5.10020
4.65796  4.96935  4.29217  3.96904  0.54444    263 Y - - -
         2.68618  4.42225  2.77519  2.73123  3.46354  2.40513  3.72494
3.29354  2.67741  2.69355  4.24690  2.90347  2.73739  3.18146  2.89801
2.37887  2.77519  2.98518  4.58477  3.61503
         0.01350  4.70791  5.43026  0.61958  0.77255  0.48576  0.95510
    215  1.47512  4.49461  4.07663  3.59256  4.13038  3.51860  4.46748
3.40641  3.53833  3.20044  4.08451  3.77242  4.15970  3.81360  3.83730
2.19684  1.67031  1.96262  5.56919  4.36447    264 a - - -
         2.68618  4.42225  2.77519  2.73123  3.46354  2.40513  3.72494
3.29354  2.67741  2.69355  4.24690  2.90347  2.73739  3.18146  2.89801
2.37887  2.77519  2.98518  4.58477  3.61503
         0.01350  4.70791  5.43026  0.61958  0.77255  0.48576  0.95510
    216  3.23144  4.58246  5.01955  4.43095  3.41546  4.45259  4.79139
1.75609  4.27147  1.30708  2.01237  4.55012  4.74177  4.39235  4.34088
3.77639  2.20469  2.42854  5.22238  4.10130    265 l - - -
         2.68618  4.42225  2.77519  2.73123  3.46354  2.40513  3.72494
3.29354  2.67741  2.69355  4.24690  2.90347  2.73739  3.18146  2.89801
2.37887  2.77519  2.98518  4.58477  3.61503
         0.01350  4.70791  5.43026  0.61958  0.77255  0.48576  0.95510
    217  2.80857  4.25974  4.35963  3.77144  3.34100  2.14605  4.23752
2.64551  3.63436  2.07265  2.63392  3.94385  4.29453  3.84211  3.78618
3.22109  3.04190  1.65549  4.87244  2.74083    266 v - - -
         2.68618  4.42225  2.77519  2.73123  3.46354  2.40513  3.72494
3.29354  2.67741  2.69355  4.24690  2.90347  2.73739  3.18146  2.89801
2.37887  2.77519  2.98518  4.58477  3.61503
         0.01350  4.70791  5.43026  0.61958  0.77255  0.48576  0.95510
    218  0.47413  4.61406  4.31456  4.18358  5.13077  2.23116  5.13944
4.55487  4.29138  4.28333  5.09487  4.04775  4.22463  4.49380  4.47767
2.90344  3.25518  3.84465  6.45510  5.35227    267 A - - -
         2.68618  4.42225  2.77519  2.73123  3.46354  2.40513  3.72494
3.29354  2.67741  2.69355  4.24690  2.90347  2.73739  3.18146  2.89801
2.37887  2.77519  2.98518  4.58477  3.61503
         0.01350  4.70791  5.43026  0.61958  0.77255  0.48576  0.95510
    219  2.74656  4.75724  3.53547  3.24475  4.74175  3.44732  4.44946
4.17949  3.35959  3.82416  4.64042  2.41394  4.13866  3.66849  3.73836
2.18672  0.86294  3.66516  6.04036  4.77294    268 t - - -
         2.68618  4.42225  2.77519  2.73123  3.46354  2.40513  3.72494
3.29354  2.67741  2.69355  4.24690  2.90347  2.73739  3.18146  2.89801
2.37887  2.77519  2.98518  4.58477  3.61503
         0.01350  4.70791  5.43026  0.61958  0.77255  0.48576  0.95510
    220  2.45101  4.70079  5.15953  4.57223  3.40762  4.60260  4.93711
2.00249  4.41625  0.98219  2.04304  4.70479  4.85809  4.50431  4.47311
3.93382  3.59179  2.51357  5.30629  4.21594    269 l - - -
         2.68618  4.42225  2.77519  2.73123  3.46354  2.40513  3.72494
3.29354  2.67741  2.69355  4.24690  2.90347  2.73739  3.18146  2.89801
2.37887  2.77519  2.98518  4.58477  3.61503
         0.01350  4.70791  5.43026  0.61958  0.77255  0.48576  0.95510
```

221 2.83574 5.26953 3.09152 2.19219 4.60102 3.59847 1.89114
4.06296 2.16517 3.55688 4.31860 2.59948 3.99153 2.87396 2.46256
2.80806 3.06169 3.65597 5.69355 3.07517 270 h - - -
2.68618 4.42225 2.77519 2.73123 3.46354 2.40513 3.72494
3.29354 2.67741 2.69355 4.24690 2.90347 2.73739 3.18146 2.89801
2.37887 2.77519 2.98518 4.58477 3.61503
0.01350 4.70791 5.43026 0.61958 0.77255 0.48576 0.95510
222 4.04609 5.24777 5.88083 5.34708 3.44706 5.44059 5.75923
1.94368 5.17869 0.48116 3.19804 5.58776 5.45778 5.10234 5.16069
4.87555 4.25901 2.67380 5.72187 4.73410 271 L - - -
2.68618 4.42225 2.77519 2.73123 3.46354 2.40513 3.72494
3.29354 2.67741 2.69355 4.24690 2.90347 2.73739 3.18146 2.89801
2.37887 2.77519 2.98518 4.58477 3.61503
0.01350 4.70791 5.43026 0.61958 0.77255 0.48576 0.95510
223 2.33794 4.29210 4.37302 3.78677 3.36973 3.95405 4.27726
2.26139 3.65486 1.60034 2.19368 3.96895 4.32124 3.86357 3.81362
2.24386 3.07186 2.46645 4.91953 3.73611 272 l - - -
2.68618 4.42225 2.77519 2.73123 3.46354 2.40513 3.72494
3.29354 2.67741 2.69355 4.24690 2.90347 2.73739 3.18146 2.89801
2.37887 2.77519 2.98518 4.58477 3.61503
0.01350 4.70791 5.43026 0.61958 0.77255 0.48576 0.95510
224 2.45689 4.48197 4.69599 4.12923 3.51513 4.22189 4.62344
2.01142 3.98842 1.04096 3.42942 4.29239 4.58244 4.18875 4.13130
3.54529 2.51000 2.33004 5.20218 4.03842 273 l - - -
2.68618 4.42225 2.77519 2.73123 3.46354 2.40513 3.72494
3.29354 2.67741 2.69355 4.24690 2.90347 2.73739 3.18146 2.89801
2.37887 2.77519 2.98518 4.58477 3.61503
0.01350 4.70791 5.43026 0.61958 0.77255 0.48576 0.95510
225 3.12020 4.76367 3.94926 3.43737 3.00649 4.04270 2.83485
3.28017 3.33362 1.63651 3.87795 2.61959 4.41658 3.63981 3.63109
3.32108 3.34926 3.07421 4.59359 1.53109 274 y - - -
2.68618 4.42225 2.77519 2.73123 3.46354 2.40513 3.72494
3.29354 2.67741 2.69355 4.24690 2.90347 2.73739 3.18146 2.89801
2.37887 2.77519 2.98518 4.58477 3.61503
0.01350 4.70791 5.43026 0.61958 0.77255 0.48576 0.95510
226 3.05946 5.45334 3.35201 2.32833 4.86919 3.77030 2.86394
4.28884 1.96445 3.71828 4.50257 3.24258 4.14419 1.80059 1.62354
3.02189 3.25960 3.88769 5.78496 4.48705 275 r - - -
2.68618 4.42225 2.77519 2.73123 3.46354 2.40513 3.72494
3.29354 2.67741 2.69355 4.24690 2.90347 2.73739 3.18146 2.89801
2.37887 2.77519 2.98518 4.58477 3.61503
0.01350 4.70791 5.43026 0.61958 0.77255 0.48576 0.95510
227 3.13255 5.49175 1.21595 2.62357 5.16562 1.71746 4.22842
4.68738 3.23233 4.19954 5.01750 3.17077 4.20019 3.39552 3.80074
1.93039 3.48512 4.17433 6.35389 4.89487 276 d - - -
2.68618 4.42225 2.77519 2.73123 3.46354 2.40513 3.72494
3.29354 2.67741 2.69355 4.24690 2.90347 2.73739 3.18146 2.89801
2.37887 2.77519 2.98518 4.58477 3.61503
0.01350 4.70791 5.43026 0.61958 0.77255 0.48576 0.95510
228 2.31161 4.27191 4.29783 3.71284 3.36280 3.91136 4.22348
2.25142 3.58461 2.43035 2.61330 3.90833 4.28231 3.80356 3.75622
3.20278 2.46809 2.47708 4.88807 1.66666 277 y - - -
2.68618 4.42225 2.77519 2.73123 3.46354 2.40513 3.72494
3.29354 2.67741 2.69355 4.24690 2.90347 2.73739 3.18146 2.89801
2.37887 2.77519 2.98518 4.58477 3.61503

```
          0.01350  4.70791  5.43026  0.61958  0.77255  0.48576  0.95510
    229   3.78564  5.06018  5.41870  5.12075  4.10679  4.90985  5.77484
0.40676  4.98302  2.58609  3.97736  5.27361  5.32536  5.28456  5.09937
4.55894  4.09201  2.21758  6.11402  4.89494    278 I - - -
          2.68618  4.42225  2.77519  2.73123  3.46354  2.40513  3.72494
3.29354  2.67741  2.69355  4.24690  2.90347  2.73739  3.18146  2.89801
2.37887  2.77519  2.98518  4.58477  3.61503
          0.01350  4.70791  5.43026  0.61958  0.77255  0.48576  0.95510
    230   2.77928  4.99601  3.16506  2.30998  4.24226  3.60282  3.80213
2.82870  2.26601  3.25541  4.06591  3.12900  3.99321  1.98216  3.04122
2.80833  2.20042  2.67927  5.49309  4.15635    279 q - - -
          2.68618  4.42225  2.77519  2.73123  3.46354  2.40513  3.72494
3.29354  2.67741  2.69355  4.24690  2.90347  2.73739  3.18146  2.89801
2.37887  2.77519  2.98518  4.58477  3.61503
          0.01350  4.70791  5.43026  0.61958  0.77255  0.48576  0.95510
    231   2.93948  4.98788  3.31619  2.82021  1.62715  3.74220  2.78226
3.60783  2.28229  3.21331  4.08179  2.14980  4.14446  3.14299  3.13639
3.00012  3.16941  3.32439  5.19611  3.73984    280 f - - -
          2.68618  4.42225  2.77519  2.73123  3.46354  2.40513  3.72494
3.29354  2.67741  2.69355  4.24690  2.90347  2.73739  3.18146  2.89801
2.37887  2.77519  2.98518  4.58477  3.61503
          0.01350  4.70791  5.43026  0.61958  0.77255  0.48576  0.95510
    232   1.51950  2.83498  4.17634  3.68017  3.97140  1.46682  4.45744
3.29772  3.60042  3.05212  2.78434  3.82266  4.17341  3.86149  3.86076
2.93602  3.07308  2.99660  5.43278  4.23757    281 g - - -
          2.68618  4.42225  2.77519  2.73123  3.46354  2.40513  3.72494
3.29354  2.67741  2.69355  4.24690  2.90347  2.73739  3.18146  2.89801
2.37887  2.77519  2.98518  4.58477  3.61503
          0.01350  4.70791  5.43026  0.61958  0.77255  0.48576  0.95510
    233   2.77989  5.20888  3.04113  1.76319  4.52634  3.55732  3.75314
3.98944  2.49805  3.50302  4.26244  2.27898  3.02014  2.86185  2.48389
2.75747  2.59858  2.83927  5.66244  4.27286    282 e - - -
          2.68618  4.42225  2.77519  2.73123  3.46354  2.40513  3.72494
3.29354  2.67741  2.69355  4.24690  2.90347  2.73739  3.18146  2.89801
2.37887  2.77519  2.98518  4.58477  3.61503
          0.01350  4.70791  5.43026  0.61958  0.77255  0.48576  0.95510
    234   2.81299  5.27505  3.07177  2.18313  4.61664  3.58015  2.86081
4.08433  1.72823  3.56914  4.32129  3.05374  3.97220  2.85442  2.49842
2.40008  2.24330  3.66487  5.70109  4.31494    283 k - - -
          2.68618  4.42225  2.77519  2.73123  3.46354  2.40513  3.72494
3.29354  2.67741  2.69355  4.24690  2.90347  2.73739  3.18146  2.89801
2.37887  2.77519  2.98518  4.58477  3.61503
          0.01350  4.70791  5.43026  0.61958  0.77255  0.48576  0.95510
    235   4.04427  5.34243  5.20445  4.84122  2.88315  4.78680  4.39708
3.46873  4.44194  1.94981  4.06152  4.76207  5.15651  4.68716  4.50044
4.32008  4.29713  3.51887  0.61415  2.93800    284 W - - -
          2.68618  4.42225  2.77519  2.73123  3.46354  2.40513  3.72494
3.29354  2.67741  2.69355  4.24690  2.90347  2.73739  3.18146  2.89801
2.37887  2.77519  2.98518  4.58477  3.61503
          0.01350  4.70791  5.43026  0.61958  0.77255  0.48576  0.95510
    236   2.77406  5.15517  2.57841  2.51694  4.45158  2.09519  3.75837
3.04159  2.23266  2.83660  4.21008  2.62969  3.95844  2.87419  2.51649
2.76245  3.00514  3.51950  5.61757  4.24012    285 g - - -
```

```
         2.68618  4.42225  2.77519  2.73123  3.46354  2.40513  3.72494
3.29354  2.67741  2.69355  4.24690  2.90347  2.73739  3.18146  2.89801
2.37887  2.77519  2.98518  4.58477  3.61503
         0.01350  4.70791  5.43026  0.61958  0.77255  0.48576  0.95510
    237  2.78408  4.58593  2.81604  2.97266  2.08940  3.72573  3.95877
2.54463  2.94412  2.80215  3.68593  2.77467  4.10947  3.25707  3.32790
2.18063  3.01626  2.86844  5.11622  2.89560    286 f - - -
         2.68618  4.42225  2.77519  2.73123  3.46354  2.40513  3.72494
3.29354  2.67741  2.69355  4.24690  2.90347  2.73739  3.18146  2.89801
2.37887  2.77519  2.98518  4.58477  3.61503
         0.01350  4.70791  5.43026  0.61958  0.77255  0.48576  0.95510
    238  2.40857  5.19605  3.04770  2.18344  4.50920  3.55521  3.02936
3.97155  1.98071  3.48781  4.24758  3.03778  3.00563  2.85663  2.97863
2.05847  3.00272  2.91714  5.64988  4.26115    287 k - - -
         2.68618  4.42225  2.77519  2.73123  3.46354  2.40513  3.72494
3.29354  2.67741  2.69355  4.24690  2.90347  2.73739  3.18146  2.89801
2.37887  2.77519  2.98518  4.58477  3.61503
         0.42966  4.70791  1.07809  0.61958  0.77255  0.48576  0.95510
    239  2.16281  4.31627  3.83258  3.29062  3.53206  3.73605  4.12315
2.02772  3.22402  2.51487  3.48296  2.68437  4.14997  3.51557  3.52535
3.03071  2.96956  1.66259  5.05481  3.83398    288 v - - -
         2.68618  4.42225  2.77519  2.73123  3.46354  2.40513  3.72494
3.29354  2.67741  2.69355  4.24690  2.90347  2.73739  3.18146  2.89801
2.37887  2.77519  2.98518  4.58477  3.61503
         0.02039  4.29864  5.02099  0.61958  0.77255  0.80568  0.59201
    240  2.82031  5.18503  2.18539  2.40296  4.41329  2.60919  3.77777
3.93150  2.62595  3.47954  4.28265  2.00178  3.93522  2.92337  3.12989
2.79416  3.07038  3.55667  5.64063  2.35420    289 n - - -
         2.68653  4.42295  2.77442  2.73118  3.46267  2.40557  3.72565
3.29353  2.67777  2.69391  4.24600  2.90330  2.73769  3.18159  2.89871
2.37825  2.77351  2.98588  4.58547  3.61573
         0.80652  0.60217  5.12128  1.67648  0.20706  0.28330  1.39957
    241  2.77881  5.25386  2.19032  1.99854  4.58341  3.54653  3.74432
4.05929  2.20249  3.55298  4.30060  3.01986  3.02059  2.84711  2.98172
2.40019  2.52849  2.83416  5.69662  4.29338    300 e - - -
         2.68618  4.42225  2.77519  2.73123  3.46354  2.40513  3.72494
3.29354  2.67741  2.69355  4.24690  2.90347  2.73739  3.18146  2.89801
2.37887  2.77519  2.98518  4.58477  3.61503
         0.01350  4.70791  5.43026  0.61958  0.77255  0.48576  0.95510
    242  2.41008  5.24900  2.55491  2.15107  4.58096  3.54433  3.73547
4.05794  2.15952  3.54871  4.29369  3.01684  3.93944  2.21417  2.96724
2.40315  2.52938  2.87490  5.68963  4.28627    301 e - - -
         2.68618  4.42225  2.77519  2.73123  3.46354  2.40513  3.72494
3.29354  2.67741  2.69355  4.24690  2.90347  2.73739  3.18146  2.89801
2.37887  2.77519  2.98518  4.58477  3.61503
         0.01350  4.70791  5.43026  0.61958  0.77255  0.48576  0.95510
    243  2.10930  4.75262  3.32789  2.34227  2.51439  3.65542  3.87734
3.32391  2.75278  2.98643  3.84014  2.69571  4.04219  2.55668  3.18218
2.87440  3.00126  2.53631  5.29334  3.11421    302 a - - -
         2.68618  4.42225  2.77519  2.73123  3.46354  2.40513  3.72494
3.29354  2.67741  2.69355  4.24690  2.90347  2.73739  3.18146  2.89801
2.37887  2.77519  2.98518  4.58477  3.61503
         0.01350  4.70791  5.43026  0.61958  0.77255  0.48576  0.95510
```

```
    244   3.70174  4.91319  5.71298  5.19182  3.73588  5.20885  5.71526
1.11749  5.09043  1.13697  3.48437  5.35795  5.35704  5.16008  5.15126
4.61425  3.93964  1.71183  5.88572  4.81141    303 i - - -
          2.68618  4.42225  2.77519  2.73123  3.46354  2.40513  3.72494
3.29354  2.67741  2.69355  4.24690  2.90347  2.73739  3.18146  2.89801
2.37887  2.77519  2.98518  4.58477  3.61503
          0.01350  4.70791  5.43026  0.61958  0.77255  0.48576  0.95510
    245   1.68532  5.56328  1.76908  2.54237  5.01554  3.57606  4.09230
4.51424  3.02832  4.02695  4.83498  1.44772  4.16021  3.24270  3.58111
3.06318  3.41921  4.06300  6.18755  4.73061    304 n - - -
          2.68618  4.42225  2.77519  2.73123  3.46354  2.40513  3.72494
3.29354  2.67741  2.69355  4.24690  2.90347  2.73739  3.18146  2.89801
2.37887  2.77519  2.98518  4.58477  3.61503
          0.01350  4.70791  5.43026  0.61958  0.77255  0.48576  0.95510
    246   2.81563  5.24249  3.07382  2.53085  4.58345  3.58062  3.76998
4.04468  2.47798  3.54616  4.30694  2.24652  3.98054  2.13113  2.19795
2.37831  2.17056  3.63597  5.69193  4.31156    305 q - - -
          2.68618  4.42225  2.77519  2.73123  3.46354  2.40513  3.72494
3.29354  2.67741  2.69355  4.24690  2.90347  2.73739  3.18146  2.89801
2.37887  2.77519  2.98518  4.58477  3.61503
          0.01350  4.70791  5.43026  0.61958  0.77255  0.48576  0.95510
    247   2.39322  4.57296  3.53111  2.96592  2.88189  3.71786  2.92517
3.09037  2.10594  2.42416  2.83891  3.40316  4.09953  3.24482  3.30805
2.95312  2.22677  2.85368  5.14271  3.89285    306 k - - -
          2.68618  4.42225  2.77519  2.73123  3.46354  2.40513  3.72494
3.29354  2.67741  2.69355  4.24690  2.90347  2.73739  3.18146  2.89801
2.37887  2.77519  2.98518  4.58477  3.61503
          0.01350  4.70791  5.43026  0.61958  0.77255  0.48576  0.95510
    248   4.02758  5.84167  4.25453  3.79786  5.44262  4.32334  4.56654
4.96270  0.29498  4.36592  5.34636  4.15557  4.81637  3.75656  3.00462
4.05496  4.24597  4.64667  6.18479  5.17281    307 K - - -
          2.68618  4.42225  2.77519  2.73123  3.46354  2.40513  3.72494
3.29354  2.67741  2.69355  4.24690  2.90347  2.73739  3.18146  2.89801
2.37887  2.77519  2.98518  4.58477  3.61503
          0.01350  4.70791  5.43026  0.61958  0.77255  0.48576  0.95510
    249   2.34902  4.60098  3.49684  2.93251  3.75652  3.70816  3.94538
3.12507  2.41011  2.41512  3.69829  3.37848  4.09073  2.67830  3.28315
2.94086  2.15165  2.19725  5.16749  2.93915    308 t - - -
          2.68618  4.42225  2.77519  2.73123  3.46354  2.40513  3.72494
3.29354  2.67741  2.69355  4.24690  2.90347  2.73739  3.18146  2.89801
2.37887  2.77519  2.98518  4.58477  3.61503
          0.01350  4.70791  5.43026  0.61958  0.77255  0.48576  0.95510
    250   2.81845  5.31030  2.19009  1.93693  4.63838  3.55114  2.96768
4.12046  2.53189  3.60873  4.35819  2.15286  3.96712  2.87743  3.02964
2.77923  2.56694  3.69299  5.74894  3.05217    309 e - - -
          2.68618  4.42225  2.77519  2.73123  3.46354  2.40513  3.72494
3.29354  2.67741  2.69355  4.24690  2.90347  2.73739  3.18146  2.89801
2.37887  2.77519  2.98518  4.58477  3.61503
          0.01350  4.70791  5.43026  0.61958  0.77255  0.48576  0.95510
    251   3.89013  5.10860  5.81678  5.24085  3.43289  5.28452  5.60329
1.36266  5.10319  0.94705  1.95928  5.43199  5.33772  5.00761  5.07002
4.67167  4.09852  2.62674  5.64184  4.67475    310 l - - -
          2.68618  4.42225  2.77519  2.73123  3.46354  2.40513  3.72494
3.29354  2.67741  2.69355  4.24690  2.90347  2.73739  3.18146  2.89801
2.37887  2.77519  2.98518  4.58477  3.61503
```

0.01350 4.70791 5.43026 0.61958 0.77255 0.48576 0.95510
252 3.58712 5.72854 4.11966 3.27915 5.31876 4.15970 3.95521
4.59522 1.25938 3.92568 4.79333 3.64234 4.47820 1.99572 1.19918
3.54835 3.70307 4.26580 5.88336 4.77933 311 r - - -
2.68618 4.42225 2.77519 2.73123 3.46354 2.40513 3.72494
3.29354 2.67741 2.69355 4.24690 2.90347 2.73739 3.18146 2.89801
2.37887 2.77519 2.98518 4.58477 3.61503
0.01350 4.70791 5.43026 0.61958 0.77255 0.48576 0.95510
253 2.78705 5.25527 3.04991 2.17689 4.59053 3.56136 3.74132
4.06221 2.13711 3.55162 3.08686 2.55562 3.95360 1.96111 2.53096
2.75608 2.56749 3.64105 5.68942 4.29528 312 q - - -
2.68618 4.42225 2.77519 2.73123 3.46354 2.40513 3.72494
3.29354 2.67741 2.69355 4.24690 2.90347 2.73739 3.18146 2.89801
2.37887 2.77519 2.98518 4.58477 3.61503
0.01350 4.70791 5.43026 0.61958 0.77255 0.48576 0.95510
254 2.78510 5.19632 2.60874 2.51170 4.50709 3.56695 3.75542
3.96478 2.17528 2.42225 4.24846 2.56317 3.96096 2.86635 2.21774
2.76607 2.21432 3.56879 5.64782 4.26552 313 k - - -
2.68618 4.42225 2.77519 2.73123 3.46354 2.40513 3.72494
3.29354 2.67741 2.69355 4.24690 2.90347 2.73739 3.18146 2.89801
2.37887 2.77519 2.98518 4.58477 3.61503
0.01350 4.70791 5.43026 0.61958 0.77255 0.48576 0.95510
255 3.78564 5.06018 5.41870 5.12075 4.10679 4.90985 5.77484
0.40676 4.98302 2.58609 3.97736 5.27361 5.32536 5.28456 5.09937
4.55894 4.09201 2.21758 6.11402 4.89494 314 I - - -
2.68618 4.42225 2.77519 2.73123 3.46354 2.40513 3.72494
3.29354 2.67741 2.69355 4.24690 2.90347 2.73739 3.18146 2.89801
2.37887 2.77519 2.98518 4.58477 3.61503
0.01350 4.70791 5.43026 0.61958 0.77255 0.48576 0.95510
256 2.44451 5.31382 3.07677 2.54459 4.66047 3.60665 2.44896
4.12359 1.87674 3.60441 4.36399 2.56740 4.00558 1.86231 2.88396
2.82847 3.08803 3.70813 5.73049 4.35338 315 q - - -
2.68618 4.42225 2.77519 2.73123 3.46354 2.40513 3.72494
3.29354 2.67741 2.69355 4.24690 2.90347 2.73739 3.18146 2.89801
2.37887 2.77519 2.98518 4.58477 3.61503
0.01350 4.70791 5.43026 0.61958 0.77255 0.48576 0.95510
257 2.76709 5.19271 2.61346 1.96730 3.29233 3.55290 3.74508
3.96639 2.49505 2.81968 4.24272 2.53235 3.94640 2.85379 2.49407
2.42201 2.52725 3.56495 5.64630 4.25671 316 e - - -
2.68618 4.42225 2.77519 2.73123 3.46354 2.40513 3.72494
3.29354 2.67741 2.69355 4.24690 2.90347 2.73739 3.18146 2.89801
2.37887 2.77519 2.98518 4.58477 3.61503
0.01350 4.70791 5.43026 0.61958 0.77255 0.48576 0.95510
258 3.63721 5.37262 3.88061 3.54474 2.73338 4.29883 1.95753
4.04121 3.49507 3.45165 4.53694 2.52183 4.70675 3.82052 3.79948
3.65643 3.87035 3.83307 4.36953 0.92942 317 y - - -
2.68618 4.42225 2.77519 2.73123 3.46354 2.40513 3.72494
3.29354 2.67741 2.69355 4.24690 2.90347 2.73739 3.18146 2.89801
2.37887 2.77519 2.98518 4.58477 3.61503
0.01350 4.70791 5.43026 0.61958 0.77255 0.48576 0.95510
259 2.65530 4.59243 4.12453 3.86349 4.91783 3.37404 4.86461
4.33431 3.88253 4.04369 4.86040 3.88242 4.16468 4.15975 4.14709
0.98126 1.20699 3.70826 6.26099 5.07741 318 s - - -

2.68618 4.42225 2.77519 2.73123 3.46354 2.40513 3.72494
3.29354 2.67741 2.69355 4.24690 2.90347 2.73739 3.18146 2.89801
2.37887 2.77519 2.98518 4.58477 3.61503
0.01350 4.70791 5.43026 0.61958 0.77255 0.48576 0.95510
260 2.80674 5.30256 2.98231 1.73873 4.64105 3.55315 3.75814
4.12219 2.18063 3.60357 4.34894 2.51823 3.96038 2.45187 2.98622
2.08413 2.58735 3.69048 5.73705 4.32914 319 e - - -
2.68618 4.42225 2.77519 2.73123 3.46354 2.40513 3.72494
3.29354 2.67741 2.69355 4.24690 2.90347 2.73739 3.18146 2.89801
2.37887 2.77519 2.98518 4.58477 3.61503
0.01350 4.70791 5.43026 0.61958 0.77255 0.48576 0.95510
261 2.35827 5.10054 2.57777 2.54347 4.38097 3.57410 3.77190
2.90910 2.53657 3.37816 4.16264 3.07628 3.96760 2.50212 2.22785
2.77417 2.03550 3.45577 5.57734 4.21288 320 t - - -
2.68618 4.42225 2.77519 2.73123 3.46354 2.40513 3.72494
3.29354 2.67741 2.69355 4.24690 2.90347 2.73739 3.18146 2.89801
2.37887 2.77519 2.98518 4.58477 3.61503
0.01350 4.70791 5.43026 0.61958 0.77255 0.48576 0.95510
262 1.99340 4.40793 4.55065 4.00672 3.65934 2.73722 4.56681
1.73827 3.89317 2.61143 3.61465 4.16110 4.47826 4.11898 4.06879
3.38298 3.21412 1.31608 5.24221 4.04716 321 v - - -
2.68618 4.42225 2.77519 2.73123 3.46354 2.40513 3.72494
3.29354 2.67741 2.69355 4.24690 2.90347 2.73739 3.18146 2.89801
2.37887 2.77519 2.98518 4.58477 3.61503
0.01350 4.70791 5.43026 0.61958 0.77255 0.48576 0.95510
263 2.77114 4.45383 2.80787 3.14324 3.58294 3.76669 4.02557
2.42855 3.08983 2.01596 2.82864 3.53064 4.14475 3.38487 2.80980
2.55142 3.00289 2.72265 5.03965 2.42431 322 l - - -
2.68618 4.42225 2.77519 2.73123 3.46354 2.40513 3.72494
3.29354 2.67741 2.69355 4.24690 2.90347 2.73739 3.18146 2.89801
2.37887 2.77519 2.98518 4.58477 3.61503
0.01350 4.70791 5.43026 0.61958 0.77255 0.48576 0.95510
264 2.37549 5.26720 2.48672 2.49734 4.60181 3.55830 3.78988
4.07149 1.94220 3.57755 4.33734 2.56685 3.97859 2.90061 3.03800
2.79230 1.76058 3.65742 5.73138 4.33235 323 t - - -
2.68618 4.42225 2.77519 2.73123 3.46354 2.40513 3.72494
3.29354 2.67741 2.69355 4.24690 2.90347 2.73739 3.18146 2.89801
2.37887 2.77519 2.98518 4.58477 3.61503
0.01350 4.70791 5.43026 0.61958 0.77255 0.48576 0.95510
265 2.30626 4.28167 4.23842 3.65693 3.40288 3.88929 4.21671
2.66856 3.54231 2.09309 2.73066 3.87326 4.26831 3.76946 3.73448
2.63508 2.14999 1.66258 4.91764 3.72421 324 v - - -
2.68618 4.42225 2.77519 2.73123 3.46354 2.40513 3.72494
3.29354 2.67741 2.69355 4.24690 2.90347 2.73739 3.18146 2.89801
2.37887 2.77519 2.98518 4.58477 3.61503
0.01350 4.70791 5.43026 0.61958 0.77255 0.48576 0.95510
266 4.52873 5.69215 5.41913 5.33881 0.26038 4.88194 4.53878
4.05250 5.25383 3.24788 4.67964 5.07809 5.36224 5.20709 5.16724
4.70684 4.84949 4.09132 4.65325 2.96389 325 F - - -
2.68618 4.42225 2.77519 2.73123 3.46354 2.40513 3.72494
3.29354 2.67741 2.69355 4.24690 2.90347 2.73739 3.18146 2.89801
2.37887 2.77519 2.98518 4.58477 3.61503
0.01350 4.70791 5.43026 0.61958 0.77255 0.48576 0.95510

```
     267   2.78468  5.04606  3.14483  2.00036  4.30594  3.59736  3.79054
 2.89449  1.98524  2.35458  4.11100  3.11213  3.98776  2.48016  3.01146
 2.80131  2.57303  3.38812  5.53064  4.18505    326 k - - -
           2.68623  4.42230  2.77486  2.73128  3.46358  2.40517  3.72499
 3.29359  2.67745  2.69359  4.24694  2.90351  2.73744  3.18151  2.89805
 2.37866  2.77524  2.98523  4.58482  3.61508
           0.09517  2.44882  5.43026  0.66479  0.72233  0.48576  0.95510
     268   2.87968  5.33490  3.02813  2.54027  4.69307  2.84333  3.80243
 4.15973  1.37598  3.64076  4.40164  2.48712  4.01602  2.48241  2.93686
 2.36727  3.11165  3.73917  5.76862  4.38460    329 k - - -
           2.68618  4.42225  2.77519  2.73123  3.46354  2.40513  3.72494
 3.29354  2.67741  2.69355  4.24690  2.90347  2.73739  3.18146  2.89801
 2.37887  2.77519  2.98518  4.58477  3.61503
           0.01350  4.70791  5.43026  0.61958  0.77255  0.48576  0.95510
     269   2.79110  5.10775  2.52354  2.54699  2.71716  2.38945  3.78772
 3.81828  2.57105  3.38064  4.17099  2.28079  3.98026  2.45692  3.04988
 2.79263  3.02398  3.45933  5.57110  3.06275    330 n - - -
           2.68618  4.42225  2.77519  2.73123  3.46354  2.40513  3.72494
 3.29354  2.67741  2.69355  4.24690  2.90347  2.73739  3.18146  2.89801
 2.37887  2.77519  2.98518  4.58477  3.61503
           0.01350  4.70791  5.43026  0.61958  0.77255  0.48576  0.95510
     270   3.30223  4.77296  4.60152  4.00918  3.46985  4.38624  4.62485
 2.73063  2.69520  0.77450  2.52878  4.28967  4.68493  4.03609  3.83079
 3.70439  3.52391  2.76885  5.28842  4.16037    331 l - - -
           2.68618  4.42225  2.77519  2.73123  3.46354  2.40513  3.72494
 3.29354  2.67741  2.69355  4.24690  2.90347  2.73739  3.18146  2.89801
 2.37887  2.77519  2.98518  4.58477  3.61503
           0.10856  4.70791  2.36608  0.61958  0.77255  0.48576  0.95510
     271   2.29455  5.12367  2.98007  2.61028  4.57372  3.52955  3.91723
 4.02232  2.70428  3.57751  4.37650  2.39613  1.53282  2.39223  3.15832
 2.85922  3.12986  3.62215  5.76496  4.40283    332 p - - -
           2.68618  4.42225  2.77519  2.73123  3.46354  2.40513  3.72494
 3.29354  2.67741  2.69355  4.24690  2.90347  2.73739  3.18146  2.89801
 2.37887  2.77519  2.98518  4.58477  3.61503
           0.01483  4.61419  5.33653  0.61958  0.77255  0.39124  1.12769
     272   2.77971  5.08731  2.54740  2.55676  4.37084  3.57575  3.78820
 3.80672  2.21914  3.37231  3.01491  3.08756  3.97643  2.91727  3.03519
 2.12430  1.82030  3.44683  5.57816  4.21711    333 t - - -
           2.68618  4.42225  2.77519  2.73123  3.46354  2.40513  3.72494
 3.29354  2.67741  2.69355  4.24690  2.90347  2.73739  3.18146  2.89801
 2.37887  2.77519  2.98518  4.58477  3.61503
           0.01350  4.70791  5.43026  0.61958  0.77255  0.48576  0.95510
     273   2.84194  4.31185  4.29988  3.71265  3.38873  3.95232  4.25124
 1.93058  2.25443  1.68382  3.38509  3.92676  4.31451  3.80933  3.75511
 3.24112  3.07368  2.10144  4.93017  2.81285    334 l - - -
           2.68618  4.42225  2.77519  2.73123  3.46354  2.40513  3.72494
 3.29354  2.67741  2.69355  4.24690  2.90347  2.73739  3.18146  2.89801
 2.37887  2.77519  2.98518  4.58477  3.61503
           0.18053  4.70791  1.85696  0.61958  0.77255  0.48576  0.95510
     274   2.75710  4.50649  3.55569  3.03515  2.64570  2.75479  3.96534
 3.01652  3.00543  1.90249  3.62649  1.96917  4.10039  3.31343  3.36149
 2.96289  3.00255  2.79154  5.01502  3.70835    335 l - - -
           2.68618  4.42225  2.77519  2.73123  3.46354  2.40513  3.72494
 3.29354  2.67741  2.69355  4.24690  2.90347  2.73739  3.18146  2.89801
 2.37887  2.77519  2.98518  4.58477  3.61503
```

```
          0.01593  4.54331  5.26566  0.61958  0.77255  0.34474  1.23239
    275   2.78602  5.26419  3.00148  2.16100  4.59945  2.86691  3.75037
4.07677  2.16376  3.56776  4.31493  2.23419  3.08229  2.85319  2.98674
2.39976  2.20919  3.65115  5.70910  4.30474    336 e - - -
          2.68618  4.42225  2.77519  2.73123  3.46354  2.40513  3.72494
3.29354  2.67741  2.69355  4.24690  2.90347  2.73739  3.18146  2.89801
2.37887  2.77519  2.98518  4.58477  3.61503
          0.36896  4.70791  1.20556  0.61958  0.77255  0.48576  0.95510
    276   2.66556  4.82384  2.94387  2.65636  4.54782  3.31999  3.98415
4.01930  2.83912  3.59880  4.40352  1.75561  1.94939  3.15287  3.28595
2.03411  3.02734  3.55138  5.78062  4.43587    337 n - - -
          2.68621  4.42228  2.77522  2.73083  3.46356  2.40515  3.72497
3.29357  2.67743  2.69358  4.24692  2.90349  2.73742  3.18149  2.89803
2.37889  2.77522  2.98521  4.58480  3.61506
          0.15997  1.95466  5.08050  0.26705  1.45087  0.27090  1.43841
    277   2.31122  4.69386  3.45621  2.90353  3.95283  3.64517  3.96874
3.32741  2.85197  2.08980  3.86769  3.35152  4.07567  3.19997  2.68252
1.65530  2.51007  3.05258  5.32498  4.05634    339 s - - -
          2.68618  4.42225  2.77519  2.73123  3.46354  2.40513  3.72494
3.29354  2.67741  2.69355  4.24690  2.90347  2.73739  3.18146  2.89801
2.37887  2.77519  2.98518  4.58477  3.61503
          0.01350  4.70791  5.43026  0.61958  0.77255  0.48576  0.95510
    278   2.88531  5.40213  2.03864  2.10909  4.73580  3.55729  3.82339
4.22565  2.60977  3.70592  4.45959  1.93659  2.86830  2.93319  3.11955
2.42505  2.52333  3.78942  5.84292  4.41655    340 n - - -
          2.68618  4.42225  2.77519  2.73123  3.46354  2.40513  3.72494
3.29354  2.67741  2.69355  4.24690  2.90347  2.73739  3.18146  2.89801
2.37887  2.77519  2.98518  4.58477  3.61503
          0.01350  4.70791  5.43026  0.61958  0.77255  0.48576  0.95510
    279   2.88012  4.72495  3.59169  3.01738  3.90315  3.79102  4.01618
3.10948  1.48988  2.02360  3.80909  3.45772  4.17794  3.25992  3.17829
3.04341  2.50256  2.35562  5.31259  4.06378    341 k - - -
          2.68618  4.42225  2.77519  2.73123  3.46354  2.40513  3.72494
3.29354  2.67741  2.69355  4.24690  2.90347  2.73739  3.18146  2.89801
2.37887  2.77519  2.98518  4.58477  3.61503
          0.01350  4.70791  5.43026  0.61958  0.77255  0.48576  0.95510
    280   2.77024  5.04373  3.11701  2.01883  3.18493  2.83666  3.78547
3.73461  2.56897  3.31348  4.11042  2.55804  3.97544  2.92089  3.04153
2.13894  2.49657  3.38894  5.53271  3.16785    342 e - - -
          2.68618  4.42225  2.77519  2.73123  3.46354  2.40513  3.72494
3.29354  2.67741  2.69355  4.24690  2.90347  2.73739  3.18146  2.89801
2.37887  2.77519  2.98518  4.58477  3.61503
          0.01350  4.70791  5.43026  0.61958  0.77255  0.48576  0.95510
    281   2.43157  5.46858  2.22670  2.07519  4.84817  3.57171  3.97109
4.33277  2.83524  3.84133  4.62816  3.07413  4.09301  3.10488  3.36290
1.19178  3.28286  3.90323  6.00317  4.57004    343 s - - -
          2.68618  4.42225  2.77519  2.73123  3.46354  2.40513  3.72494
3.29354  2.67741  2.69355  4.24690  2.90347  2.73739  3.18146  2.89801
2.37887  2.77519  2.98518  4.58477  3.61503
          0.01350  4.70791  5.43026  0.61958  0.77255  0.48576  0.95510
    282   2.81413  4.22329  4.54450  3.94800  2.59798  3.95813  4.25553
2.24755  3.78126  2.04131  3.32880  2.85735  4.32103  3.95188  3.86949
3.26036  3.04627  1.69126  4.80241  2.25935    344 v - - -
```

2.68618 4.42225 2.77519 2.73123 3.46354 2.40513 3.72494
3.29354 2.67741 2.69355 4.24690 2.90347 2.73739 3.18146 2.89801
2.37887 2.77519 2.98518 4.58477 3.61503
0.01350 4.70791 5.43026 0.61958 0.77255 0.48576 0.95510
283 2.40280 5.52039 2.27950 2.49595 4.86077 3.58450 3.93651
4.34921 2.21789 3.84016 4.62090 1.24095 4.08701 3.06374 3.27940
2.96089 3.27662 3.92025 5.98729 4.55266 345 n - - -
2.68618 4.42225 2.77519 2.73123 3.46354 2.40513 3.72494
3.29354 2.67741 2.69355 4.24690 2.90347 2.73739 3.18146 2.89801
2.37887 2.77519 2.98518 4.58477 3.61503
0.01350 4.70791 5.43026 0.61958 0.77255 0.48576 0.95510
284 2.13899 5.19115 2.19710 2.53948 4.51380 3.57368 3.80575
3.94487 1.57401 3.49732 4.27608 3.07684 3.99134 2.92558 3.03993
2.80620 3.05756 2.74614 5.67955 4.30078 346 k - - -
2.68618 4.42225 2.77519 2.73123 3.46354 2.40513 3.72494
3.29354 2.67741 2.69355 4.24690 2.90347 2.73739 3.18146 2.89801
2.37887 2.77519 2.98518 4.58477 3.61503
0.01350 4.70791 5.43026 0.61958 0.77255 0.48576 0.95510
285 2.90383 5.29527 2.42040 2.55493 4.54093 3.61231 3.83117
4.05285 1.89481 3.57173 4.35734 3.09502 4.03823 2.42349 3.02038
2.87770 3.13633 3.66815 5.70494 1.73299 347 y - - -
2.68618 4.42225 2.77519 2.73123 3.46354 2.40513 3.72494
3.29354 2.67741 2.69355 4.24690 2.90347 2.73739 3.18146 2.89801
2.37887 2.77519 2.98518 4.58477 3.61503
0.01350 4.70791 5.43026 0.61958 0.77255 0.48576 0.95510
286 2.33729 4.77113 3.32461 2.76980 3.97433 3.65071 3.88554
2.68826 2.75046 3.01713 3.87038 1.88956 4.04446 2.56462 3.18074
2.87379 2.47726 2.53647 5.32609 4.03635 348 n - - -
2.68618 4.42225 2.77519 2.73123 3.46354 2.40513 3.72494
3.29354 2.67741 2.69355 4.24690 2.90347 2.73739 3.18146 2.89801
2.37887 2.77519 2.98518 4.58477 3.61503
0.01350 4.70791 5.43026 0.61958 0.77255 0.48576 0.95510
287 2.82218 5.24402 2.53533 2.52870 4.56739 3.58541 3.77050
4.02496 2.16376 3.53267 4.29731 2.51802 3.98376 2.88059 1.74518
2.79795 3.05120 2.47201 5.68396 4.30472 349 r - - -
2.68618 4.42225 2.77519 2.73123 3.46354 2.40513 3.72494
3.29354 2.67741 2.69355 4.24690 2.90347 2.73739 3.18146 2.89801
2.37887 2.77519 2.98518 4.58477 3.61503
0.01350 4.70791 5.43026 0.61958 0.77255 0.48576 0.95510
288 3.87018 5.17948 5.03166 4.68682 2.63678 4.73652 4.14321
2.25946 4.45588 2.85930 4.13612 4.56348 5.08347 4.57270 4.52119
4.13421 4.11708 3.23461 4.32500 0.59878 350 Y - - -
2.68618 4.42225 2.77519 2.73123 3.46354 2.40513 3.72494
3.29354 2.67741 2.69355 4.24690 2.90347 2.73739 3.18146 2.89801
2.37887 2.77519 2.98518 4.58477 3.61503
0.01350 4.70791 5.43026 0.61958 0.77255 0.48576 0.95510
289 2.88072 4.35305 4.31957 3.74709 3.48479 3.99508 2.36285
1.62528 3.61742 2.50124 3.47312 3.97147 4.36759 3.86614 3.81411
3.29190 2.54703 1.63012 5.02954 3.82830 351 i - - -
2.68618 4.42225 2.77519 2.73123 3.46354 2.40513 3.72494
3.29354 2.67741 2.69355 4.24690 2.90347 2.73739 3.18146 2.89801
2.37887 2.77519 2.98518 4.58477 3.61503
0.01350 4.70791 5.43026 0.61958 0.77255 0.48576 0.95510

290 2.96654 5.37534 3.25411 2.24650 4.75671 3.70257 2.77807
4.19277 1.78300 3.65007 4.42482 3.17759 4.08287 2.90919 1.61171
2.93401 2.53538 3.78955 5.74607 4.41769 352 r - - -
2.68618 4.42225 2.77519 2.73123 3.46354 2.40513 3.72494
3.29354 2.67741 2.69355 4.24690 2.90347 2.73739 3.18146 2.89801
2.37887 2.77519 2.98518 4.58477 3.61503
0.01350 4.70791 5.43026 0.61958 0.77255 0.48576 0.95510
291 2.34263 4.52826 3.65429 3.10717 3.75467 2.00021 4.06207
2.11432 3.07055 2.82469 3.71168 2.79932 4.12472 3.37520 3.43154
2.48026 3.02174 2.35492 5.18878 3.95129 353 g - - -
2.68618 4.42225 2.77519 2.73123 3.46354 2.40513 3.72494
3.29354 2.67741 2.69355 4.24690 2.90347 2.73739 3.18146 2.89801
2.37887 2.77519 2.98518 4.58477 3.61503
0.01350 4.70791 5.43026 0.61958 0.77255 0.48576 0.95510
292 2.45613 4.63883 4.99573 4.41621 3.45469 4.49625 4.84647
2.01040 4.26620 1.69553 1.20718 4.57185 4.78420 4.40625 4.36434
3.82278 3.51066 2.42108 5.29322 4.17437 354 m - - -
2.68618 4.42225 2.77519 2.73123 3.46354 2.40513 3.72494
3.29354 2.67741 2.69355 4.24690 2.90347 2.73739 3.18146 2.89801
2.37887 2.77519 2.98518 4.58477 3.61503
0.01350 4.70791 5.43026 0.61958 0.77255 0.48576 0.95510
293 2.82328 4.65172 3.68167 3.19696 3.92828 3.67684 2.75996
3.29167 3.09308 3.03247 3.94261 3.56461 4.18361 3.46841 3.42658
3.00875 1.07650 2.43462 5.36155 4.08195 355 t - - -
2.68618 4.42225 2.77519 2.73123 3.46354 2.40513 3.72494
3.29354 2.67741 2.69355 4.24690 2.90347 2.73739 3.18146 2.89801
2.37887 2.77519 2.98518 4.58477 3.61503
0.01350 4.70791 5.43026 0.61958 0.77255 0.48576 0.95510
294 2.79652 4.29918 4.16002 2.72359 3.39452 3.88294 4.17901
2.71487 3.46523 1.52924 3.39693 3.82521 4.25278 3.70750 3.67577
3.16517 2.48718 1.92660 3.08593 3.71122 356 l - - -
2.68618 4.42225 2.77519 2.73123 3.46354 2.40513 3.72494
3.29354 2.67741 2.69355 4.24690 2.90347 2.73739 3.18146 2.89801
2.37887 2.77519 2.98518 4.58477 3.61503
0.01350 4.70791 5.43026 0.61958 0.77255 0.48576 0.95510
295 2.79394 4.92919 3.22014 2.66954 2.96923 3.63131 2.93900
3.55912 2.64644 3.17402 2.95555 1.84582 4.02186 2.21025 3.09262
2.84452 3.02489 3.25470 5.38934 4.03954 357 n - - -
2.68618 4.42225 2.77519 2.73123 3.46354 2.40513 3.72494
3.29354 2.67741 2.69355 4.24690 2.90347 2.73739 3.18146 2.89801
2.37887 2.77519 2.98518 4.58477 3.61503
0.01350 4.70791 5.43026 0.61958 0.77255 0.48576 0.95510
296 2.65366 1.46023 4.48006 4.04788 4.10736 1.94646 4.68859
3.40823 3.94307 3.20033 4.09362 3.98619 4.19186 4.15953 4.12096
2.26532 3.09699 1.83359 5.58029 4.40936 358 c - - -
2.68618 4.42225 2.77519 2.73123 3.46354 2.40513 3.72494
3.29354 2.67741 2.69355 4.24690 2.90347 2.73739 3.18146 2.89801
2.37887 2.77519 2.98518 4.58477 3.61503
0.01350 4.70791 5.43026 0.61958 0.77255 0.48576 0.95510
297 3.47562 5.00904 4.66701 4.30175 3.71248 4.34103 4.98539
3.00359 4.12347 0.53900 3.62626 4.52390 2.57628 4.43175 4.28803
3.83522 3.79373 3.06081 5.57667 4.41105 359 L - - -
2.68618 4.42225 2.77519 2.73123 3.46354 2.40513 3.72494
3.29354 2.67741 2.69355 4.24690 2.90347 2.73739 3.18146 2.89801
2.37887 2.77519 2.98518 4.58477 3.61503

```
         0.01350  4.70791  5.43026  0.61958  0.77255  0.48576  0.95510
    298  4.14564  6.17171  0.22568  3.25148  5.72363  4.09097  4.97558
5.55962  4.24873  5.02011  6.08237  3.82748  4.79485  4.26004  4.82119
4.05906  4.51846  5.12596  6.68028  5.54848    360 D - - -
         2.68618  4.42225  2.77519  2.73123  3.46354  2.40513  3.72494
3.29354  2.67741  2.69355  4.24690  2.90347  2.73739  3.18146  2.89801
2.37887  2.77519  2.98518  4.58477  3.61503
         0.01350  4.70791  5.43026  0.61958  0.77255  0.48576  0.95510
    299  3.40905  4.70402  5.36642  4.77887  2.03564  4.69878  5.04735
1.31833  4.61125  1.62400  2.48497  4.85203  4.93676  4.65787  4.61564
4.04212  3.63460  1.94142  5.35255  4.25861    361 i - - -
         2.68618  4.42225  2.77519  2.73123  3.46354  2.40513  3.72494
3.29354  2.67741  2.69355  4.24690  2.90347  2.73739  3.18146  2.89801
2.37887  2.77519  2.98518  4.58477  3.61503
         0.01350  4.70791  5.43026  0.61958  0.77255  0.48576  0.95510
    300  1.82378  4.34245  4.58515  4.00755  3.47461  4.09574  4.45286
2.04281  3.86706  2.46440  3.46328  4.15251  4.46036  4.07083  4.00304
3.40601  3.17060  1.33877  5.05190  2.73886    362 v - - -
         2.68618  4.42225  2.77519  2.73123  3.46354  2.40513  3.72494
3.29354  2.67741  2.69355  4.24690  2.90347  2.73739  3.18146  2.89801
2.37887  2.77519  2.98518  4.58477  3.61503
         0.01350  4.70791  5.43026  0.61958  0.77255  0.48576  0.95510
    301  0.98675  4.94621  3.84964  3.36087  4.72669  3.67289  4.31186
4.09807  2.86282  3.71805  4.58936  3.67357  4.27139  3.50596  1.50801
3.09339  3.34097  3.69081  5.90347  4.69494    363 a - - -
         2.68618  4.42225  2.77519  2.73123  3.46354  2.40513  3.72494
3.29354  2.67741  2.69355  4.24690  2.90347  2.73739  3.18146  2.89801
2.37887  2.77519  2.98518  4.58477  3.61503
         0.01350  4.70791  5.43026  0.61958  0.77255  0.48576  0.95510
    302  3.25231  4.65416  4.84721  4.29027  3.53828  4.42903  4.83032
2.03438  4.14114  1.14838  3.40056  4.48540  4.75741  4.34166  4.29074
3.76357  1.59733  2.38088  5.35984  4.21845    364 l - - -
         2.68618  4.42225  2.77519  2.73123  3.46354  2.40513  3.72494
3.29354  2.67741  2.69355  4.24690  2.90347  2.73739  3.18146  2.89801
2.37887  2.77519  2.98518  4.58477  3.61503
         0.01350  4.70791  5.43026  0.61958  0.77255  0.48576  0.95510
    303  4.52187  5.59275  5.48202  5.32126  1.84758  5.07773  4.02688
4.06653  5.08233  3.23898  4.60839  4.79979  5.39181  4.91555  4.97402
4.56399  4.74870  4.06219  0.56855  2.38063    365 W - - -
         2.68618  4.42225  2.77519  2.73123  3.46354  2.40513  3.72494
3.29354  2.67741  2.69355  4.24690  2.90347  2.73739  3.18146  2.89801
2.37887  2.77519  2.98518  4.58477  3.61503
         0.01350  4.70791  5.43026  0.61958  0.77255  0.48576  0.95510
    304  2.81279  4.75049  3.91301  3.81068  5.06386  3.46468  4.95057
4.61541  3.98262  4.27627  5.11505  3.91462  0.56303  4.26486  4.23670
2.15307  3.34589  3.94108  6.37270  5.17522    366 P - - -
         2.68618  4.42225  2.77519  2.73123  3.46354  2.40513  3.72494
3.29354  2.67741  2.69355  4.24690  2.90347  2.73739  3.18146  2.89801
2.37887  2.77519  2.98518  4.58477  3.61503
         0.01350  4.70791  5.43026  0.61958  0.77255  0.48576  0.95510
    305  2.73593  4.53539  3.93813  3.46932  2.65655  3.58228  4.35089
3.36819  3.42805  3.09436  4.00020  3.71594  4.17865  3.71697  3.73883
1.88651  1.12101  3.07040  5.40053  4.12294    367 t - - -
```

2.68618 4.42225 2.77519 2.73123 3.46354 2.40513 3.72494
3.29354 2.67741 2.69355 4.24690 2.90347 2.73739 3.18146 2.89801
2.37887 2.77519 2.98518 4.58477 3.61503
0.01350 4.70791 5.43026 0.61958 0.77255 0.48576 0.95510
306 4.08953 5.29448 5.73196 5.26874 1.27959 5.26901 4.98724
2.79597 5.10486 0.80284 3.29693 5.27919 5.36642 4.96533 5.04513
4.68473 4.29660 3.05893 5.03715 3.57268 368 l - - -
2.68618 4.42225 2.77519 2.73123 3.46354 2.40513 3.72494
3.29354 2.67741 2.69355 4.24690 2.90347 2.73739 3.18146 2.89801
2.37887 2.77519 2.98518 4.58477 3.61503
0.01350 4.70791 5.43026 0.61958 0.77255 0.48576 0.95510
307 3.50534 5.73961 0.73019 2.80531 3.95322 3.82468 4.22506
4.50997 3.43720 3.99806 4.98127 3.35483 4.42959 3.58791 3.97126
3.43407 3.79939 4.18655 5.47836 2.01685 369 d - - -
2.68618 4.42225 2.77519 2.73123 3.46354 2.40513 3.72494
3.29354 2.67741 2.69355 4.24690 2.90347 2.73739 3.18146 2.89801
2.37887 2.77519 2.98518 4.58477 3.61503
0.01350 4.70791 5.43026 0.61958 0.77255 0.48576 0.95510
308 2.75560 4.82020 3.41250 3.08162 4.73503 3.45844 4.32012
4.18163 3.19765 3.79269 4.59515 2.54647 1.08932 3.50941 3.61190
2.19233 2.44188 3.67641 5.99979 4.70735 370 p - - -
2.68618 4.42225 2.77519 2.73123 3.46354 2.40513 3.72494
3.29354 2.67741 2.69355 4.24690 2.90347 2.73739 3.18146 2.89801
2.37887 2.77519 2.98518 4.58477 3.61503
0.01350 4.70791 5.43026 0.61958 0.77255 0.48576 0.95510
309 2.76849 3.23495 2.02738 2.58874 4.25408 3.59061 3.04199
3.67717 2.58182 2.67613 4.07357 2.62665 3.98155 2.93488 2.54867
2.79273 2.99987 2.78556 5.50042 4.15687 371 d - - -
2.68618 4.42225 2.77519 2.73123 3.46354 2.40513 3.72494
3.29354 2.67741 2.69355 4.24690 2.90347 2.73739 3.18146 2.89801
2.37887 2.77519 2.98518 4.58477 3.61503
0.01350 4.70791 5.43026 0.61958 0.77255 0.48576 0.95510
310 2.77867 4.96101 2.04508 2.62444 4.16026 3.60741 2.89968
2.80417 2.62383 2.65206 4.03383 2.34344 3.99986 2.97136 3.08516
2.81682 3.01036 3.29006 5.44521 3.03544 372 d - - -
2.68618 4.42225 2.77519 2.73123 3.46354 2.40513 3.72494
3.29354 2.67741 2.69355 4.24690 2.90347 2.73739 3.18146 2.89801
2.37887 2.77519 2.98518 4.58477 3.61503
0.01350 4.70791 5.43026 0.61958 0.77255 0.48576 0.95510
311 4.62705 5.77367 5.15902 5.08940 2.79635 4.87159 4.31504
4.56637 4.94449 3.79323 5.15160 4.87732 5.34883 5.02217 4.92947
4.65395 4.92861 4.47859 4.45019 0.24676 373 Y - - -
2.68618 4.42225 2.77519 2.73123 3.46354 2.40513 3.72494
3.29354 2.67741 2.69355 4.24690 2.90347 2.73739 3.18146 2.89801
2.37887 2.77519 2.98518 4.58477 3.61503
0.01350 4.70791 5.43026 0.61958 0.77255 0.48576 0.95510
312 2.81255 5.14998 3.07979 2.56835 4.47781 3.57883 2.99794
3.92553 2.56172 3.46935 4.25159 3.09810 1.89658 2.09991 3.02320
2.41090 2.55277 3.54490 5.65207 4.28533 374 p - - -
2.68618 4.42225 2.77519 2.73123 3.46354 2.40513 3.72494
3.29354 2.67741 2.69355 4.24690 2.90347 2.73739 3.18146 2.89801
2.37887 2.77519 2.98518 4.58477 3.61503
0.01350 4.70791 5.43026 0.61958 0.77255 0.48576 0.95510

```
    313   2.79463  4.89960  3.25166  2.69930  4.13159  3.63489  3.85021
2.32915  2.25084  3.15818  3.99140  3.20087  4.03021  2.58078  3.07754
1.73023  2.52344  3.22713  5.42641  4.11764    375 s - - -
          2.68618  4.42225  2.77519  2.73123  3.46354  2.40513  3.72494
3.29354  2.67741  2.69355  4.24690  2.90347  2.73739  3.18146  2.89801
2.37887  2.77519  2.98518  4.58477  3.61503
          0.01350  4.70791  5.43026  0.61958  0.77255  0.48576  0.95510
    314   2.80647  5.27124  2.98075  1.90146  4.59409  2.45534  3.76755
4.06793  2.52250  3.56752  4.32198  2.57851  3.96440  2.17684  3.01396
2.35898  3.04028  3.65112  5.71645  3.04785    376 e - - -
          2.68620  4.42227  2.77522  2.73126  3.46356  2.40515  3.72497
3.29356  2.67743  2.69357  4.24692  2.90349  2.73742  3.18149  2.89803
2.37862  2.77522  2.98521  4.58479  3.61505
          0.10075  2.39187  5.43026  0.28170  1.40447  0.48576  0.95510
    315   2.86913  4.40587  4.30019  3.75473  3.64401  1.97066  4.41699
1.85917  3.65853  2.63649  3.60997  3.97353  4.36600  3.90867  3.88384
3.24729  2.06019  1.75049  5.18855  3.98841    378 v - - -
          2.68618  4.42225  2.77519  2.73123  3.46354  2.40513  3.72494
3.29354  2.67741  2.69355  4.24690  2.90347  2.73739  3.18146  2.89801
2.37887  2.77519  2.98518  4.58477  3.61503
          0.01350  4.70791  5.43026  0.61958  0.77255  0.48576  0.95510
    316   2.10122  5.29821  1.92727  2.13324  4.62027  3.55696  3.78073
4.09605  2.00534  3.59349  4.34906  3.02916  3.97462  2.88898  3.03610
2.78966  3.06055  3.67746  5.74125  3.03156    379 d - - -
          2.68594  4.42240  2.77534  2.73138  3.46369  2.40527  3.72509
3.29239  2.67755  2.69370  4.24704  2.90361  2.73754  3.18107  2.89815
2.37874  2.77492  2.98533  4.58492  3.61518
          0.29394  1.38510  5.43026  0.68083  0.70562  0.48576  0.95510
    317   3.94235  5.14491  5.84444  5.31252  3.51548  5.37912  5.75160
1.34745  5.16591  0.72090  3.26441  5.52852  5.43163  5.12218  5.16543
4.80381  4.16273  2.47976  5.76289  4.75770    382 l - - -
          2.68618  4.42225  2.77519  2.73123  3.46354  2.40513  3.72494
3.29354  2.67741  2.69355  4.24690  2.90347  2.73739  3.18146  2.89801
2.37887  2.77519  2.98518  4.58477  3.61503
          0.01350  4.70791  5.43026  0.61958  0.77255  0.48576  0.95510
    318   2.99461  5.55640  2.01769  1.41415  4.88431  3.57320  3.89536
4.38751  2.20790  3.85292  4.61614  2.41181  4.05973  2.41968  3.23957
2.92396  3.24184  3.94056  5.98267  4.53384    383 e - - -
          2.68618  4.42225  2.77519  2.73123  3.46354  2.40513  3.72494
3.29354  2.67741  2.69355  4.24690  2.90347  2.73739  3.18146  2.89801
2.37887  2.77519  2.98518  4.58477  3.61503
          0.01350  4.70791  5.43026  0.61958  0.77255  0.48576  0.95510
    319   2.88062  4.81883  3.47249  2.89994  2.35677  3.75515  3.92753
3.34839  1.76541  2.13439  3.86872  3.36300  4.13438  2.23807  3.08100
2.99438  3.10518  3.10968  5.28237  3.98013    384 k - - -
          2.68618  4.42225  2.77519  2.73123  3.46354  2.40513  3.72494
3.29354  2.67741  2.69355  4.24690  2.90347  2.73739  3.18146  2.89801
2.37887  2.77519  2.98518  4.58477  3.61503
          0.01350  4.70791  5.43026  0.61958  0.77255  0.48576  0.95510
    320   3.14271  4.96644  4.48265  4.36539  5.02534  3.75813  5.27484
4.29643  4.33295  4.11500  5.15258  4.35762  4.54225  4.68540  4.49117
3.35803  0.30730  3.86803  6.34812  5.25906    385 T - - -
          2.68618  4.42225  2.77519  2.73123  3.46354  2.40513  3.72494
3.29354  2.67741  2.69355  4.24690  2.90347  2.73739  3.18146  2.89801
2.37887  2.77519  2.98518  4.58477  3.61503
```

```
          0.01350  4.70791  5.43026  0.61958  0.77255  0.48576  0.95510
    321   3.80835  5.78771  4.45236  3.53218  5.42981  4.29053  4.04567
4.68585  1.71241  4.00379  4.91487  3.82328  4.61433  3.17595  0.60889
3.78443  3.89992  4.38988  5.92787  4.88781    386 R - - -
          2.68618  4.42225  2.77519  2.73123  3.46354  2.40513  3.72494
3.29354  2.67741  2.69355  4.24690  2.90347  2.73739  3.18146  2.89801
2.37887  2.77519  2.98518  4.58477  3.61503
          0.01350  4.70791  5.43026  0.61958  0.77255  0.48576  0.95510
    322   2.83315  4.27752  4.39100  3.80189  3.37616  3.96320  4.27837
2.24607  3.66444  2.10234  2.63373  3.97783  4.32580  3.87485  2.84539
3.25722  2.07437  1.64074  4.91593  3.72949    387 v - - -
          2.68618  4.42225  2.77519  2.73123  3.46354  2.40513  3.72494
3.29354  2.67741  2.69355  4.24690  2.90347  2.73739  3.18146  2.89801
2.37887  2.77519  2.98518  4.58477  3.61503
          0.01350  4.70791  5.43026  0.61958  0.77255  0.48576  0.95510
    323   3.31018  4.61263  5.16964  4.62576  3.80638  4.66158  5.13376
1.03500  4.50318  2.00348  3.65481  4.76456  4.96265  4.70521  4.63080
4.01459  2.10678  1.69276  5.61849  4.43617    388 i - - -
          2.68618  4.42225  2.77519  2.73123  3.46354  2.40513  3.72494
3.29354  2.67741  2.69355  4.24690  2.90347  2.73739  3.18146  2.89801
2.37887  2.77519  2.98518  4.58477  3.61503
          0.01350  4.70791  5.43026  0.61958  0.77255  0.48576  0.95510
    324   2.78574  2.93753  4.34964  3.75910  2.02176  3.90181  4.19679
2.67852  3.61805  1.87603  3.35046  3.92097  4.26996  3.82207  2.82171
2.53897  3.01799  2.48737  4.81919  2.75784    389 l - - -
          2.68618  4.42225  2.77519  2.73123  3.46354  2.40513  3.72494
3.29354  2.67741  2.69355  4.24690  2.90347  2.73739  3.18146  2.89801
2.37887  2.77519  2.98518  4.58477  3.61503
          0.01350  4.70791  5.43026  0.61958  0.77255  0.48576  0.95510
    325   2.79849  4.62066  3.56137  3.03209  3.82076  3.70651  4.03988
2.48603  2.98990  2.36707  3.77927  2.66979  4.13509  3.32114  3.36441
1.36067  3.05721  2.89774  5.24782  3.98681    390 s - - -
          2.68618  4.42225  2.77519  2.73123  3.46354  2.40513  3.72494
3.29354  2.67741  2.69355  4.24690  2.90347  2.73739  3.18146  2.89801
2.37887  2.77519  2.98518  4.58477  3.61503
          0.01350  4.70791  5.43026  0.61958  0.77255  0.48576  0.95510
    326   3.01214  5.50270  1.50648  2.50034  4.89830  2.62065  3.96358
4.39671  2.82365  3.88493  4.66194  2.44206  2.24283  3.09223  3.35657
2.31014  3.28147  3.94849  6.03181  4.58745    391 d - - -
          2.68618  4.42225  2.77519  2.73123  3.46354  2.40513  3.72494
3.29354  2.67741  2.69355  4.24690  2.90347  2.73739  3.18146  2.89801
2.37887  2.77519  2.98518  4.58477  3.61503
          0.01350  4.70791  5.43026  0.61958  0.77255  0.48576  0.95510
    327   2.82902  4.27419  2.95361  3.79572  2.65534  3.95857  4.27410
1.66686  3.66530  2.41088  2.29624  3.97260  4.32173  3.87152  3.81986
3.25217  2.55867  2.10499  4.91186  3.72436    392 i - - -
          2.68618  4.42225  2.77519  2.73123  3.46354  2.40513  3.72494
3.29354  2.67741  2.69355  4.24690  2.90347  2.73739  3.18146  2.89801
2.37887  2.77519  2.98518  4.58477  3.61503
          0.01350  4.70791  5.43026  0.61958  0.77255  0.48576  0.95510
    328   2.10544  4.32985  4.65871  4.07705  3.45269  4.11478  4.46711
1.75368  3.92477  2.44586  3.44689  4.19269  4.47401  4.11521  4.03632
3.42725  3.17349  1.50946  5.03408  2.25011    393 v - - -
```

```
          2.68618  4.42225  2.77519  2.73123  3.46354  2.40513  3.72494
3.29354  2.67741  2.69355  4.24690  2.90347  2.73739  3.18146  2.89801
2.37887  2.77519  2.98518  4.58477  3.61503
          0.01350  4.70791  5.43026  0.61958  0.77255  0.48576  0.95510
    329   2.93734  5.22725  3.17147  2.70115  4.65171  1.38386  3.90453
4.08794  2.55532  3.61123  4.40827  3.20743  2.87589  2.39496  2.33669
2.93869  3.18891  3.69758  5.76368  4.43219    394 g - - -
          2.68618  4.42225  2.77519  2.73123  3.46354  2.40513  3.72494
3.29354  2.67741  2.69355  4.24690  2.90347  2.73739  3.18146  2.89801
2.37887  2.77519  2.98518  4.58477  3.61503
          0.01350  4.70791  5.43026  0.61958  0.77255  0.48576  0.95510
    330   2.83094  5.24163  2.46683  2.50680  4.54553  3.56744  3.80007
2.98157  2.57328  3.53187  4.30401  2.48842  1.93764  2.17262  3.06213
2.80748  3.06724  3.61195  5.70365  4.31229    395 p - - -
          2.68618  4.42225  2.77519  2.73123  3.46354  2.40513  3.72494
3.29354  2.67741  2.69355  4.24690  2.90347  2.73739  3.18146  2.89801
2.37887  2.77519  2.98518  4.58477  3.61503
          0.10075  4.70791  2.44396  0.61958  0.77255  0.48576  0.95510
    331   2.74053  4.61650  3.40716  2.08411  3.77949  3.65976  3.89207
2.65380  2.81469  2.83945  3.71088  3.30609  4.04398  2.56531  3.21767
2.46492  2.97213  2.08846  3.36438  3.91075    396 e - - -
          2.68618  4.42225  2.77519  2.73123  3.46354  2.40513  3.72494
3.29354  2.67741  2.69355  4.24690  2.90347  2.73739  3.18146  2.89801
2.37887  2.77519  2.98518  4.58477  3.61503
          0.01472  4.62188  5.34423  0.61958  0.77255  0.39730  1.11515
    332   2.86465  5.34913  3.01422  1.45365  4.69893  3.59081  3.78295
4.17204  2.11808  3.64411  4.39742  2.50974  2.91405  2.88712  2.44783
2.82369  3.09376  3.74424  5.76613  4.37367    397 e - - -
          2.68618  4.42225  2.77519  2.73123  3.46354  2.40513  3.72494
3.29354  2.67741  2.69355  4.24690  2.90347  2.73739  3.18146  2.89801
2.37887  2.77519  2.98518  4.58477  3.61503
          0.01350  4.70791  5.43026  0.61958  0.77255  0.48576  0.95510
    333   2.77857  5.17567  2.54481  2.23705  4.47630  2.49263  3.76109
2.48788  2.18367  3.46242  4.23073  3.04720  3.95819  2.87546  3.00541
2.40918  2.53926  3.54252  5.63733  4.25385    398 k - - -
          2.68618  4.42225  2.77519  2.73123  3.46354  2.40513  3.72494
3.29354  2.67741  2.69355  4.24690  2.90347  2.73739  3.18146  2.89801
2.37887  2.77519  2.98518  4.58477  3.61503
          0.01350  4.70791  5.43026  0.61958  0.77255  0.48576  0.95510
    334   2.35934  4.57293  3.52955  2.96509  2.85980  3.71695  3.95813
3.09151  2.92804  2.33851  3.67200  2.34140  4.09906  3.24503  2.72985
2.95227  2.18370  2.85440  5.14100  3.04635    399 t - - -
          2.68618  4.42225  2.77519  2.73123  3.46354  2.40513  3.72494
3.29354  2.67741  2.69355  4.24690  2.90347  2.73739  3.18146  2.89801
2.37887  2.77519  2.98518  4.58477  3.61503
          0.01350  4.70791  5.43026  0.61958  0.77255  0.48576  0.95510
    335   2.77475  5.08916  1.85532  2.54638  4.36040  3.57517  3.77741
3.79915  2.55185  3.36289  3.03634  3.07914  3.00138  2.43649  3.02828
2.77857  2.59398  3.44039  5.56806  3.08427    400 d - - -
          2.68618  4.42225  2.77519  2.73123  3.46354  2.40513  3.72494
3.29354  2.67741  2.69355  4.24690  2.90347  2.73739  3.18146  2.89801
2.37887  2.77519  2.98518  4.58477  3.61503
          0.01350  4.70791  5.43026  0.61958  0.77255  0.48576  0.95510
```

```
    336   2.85152  5.34342  2.39187  2.47433  4.68174  2.08808  3.80415
4.16503  2.57799  3.65328  4.40674  2.17976  2.93081  2.39982  3.08019
2.80947  2.60424  3.73484  5.79489  4.37872    401 g - - -
          2.68618  4.42225  2.77519  2.73123  3.46354  2.40513  3.72494
3.29354  2.67741  2.69355  4.24690  2.90347  2.73739  3.18146  2.89801
2.37887  2.77519  2.98518  4.58477  3.61503
          0.10420  4.70791  2.40875  0.61958  0.77255  0.48576  0.95510
    337   2.73729  5.09270  2.54117  2.49448  4.37938  2.90442  3.73153
3.02377  2.15703  2.74222  4.15050  2.51327  3.92812  2.85161  2.97558
2.38279  2.53004  3.45171  5.56325  4.19099    402 k - - -
          2.68618  4.42225  2.77519  2.73123  3.46354  2.40513  3.72494
3.29354  2.67741  2.69355  4.24690  2.90347  2.73739  3.18146  2.89801
2.37887  2.77519  2.98518  4.58477  3.61503
          0.01477  4.61848  5.34083  0.61958  0.77255  0.65138  0.73674
    338   2.73991  4.53228  2.84433  2.95289  3.68728  3.68875  3.94143
3.03252  2.91817  2.34990  2.76174  3.38591  4.07434  3.23217  3.29857
2.42825  1.89345  2.39592  5.11358  3.86561    403 t - - -
          2.68618  4.42225  2.77519  2.73123  3.46354  2.40513  3.72494
3.29354  2.67741  2.69355  4.24690  2.90347  2.73739  3.18146  2.89801
2.37887  2.77519  2.98518  4.58477  3.61503
          0.09964  4.61848  2.46544  0.61958  0.77255  0.65138  0.73674
    339   2.75634  5.00187  3.12229  2.56388  4.27155  3.55986  2.79625
3.69466  1.71339  2.63200  4.07623  3.08112  3.95206  2.88391  2.90189
2.31034  2.51110  3.35531  5.48244  4.14710    404 k - - -
          2.68678  4.42285  2.77495  2.73150  3.46414  2.40519  3.72465
3.29159  2.67762  2.69337  4.24750  2.90321  2.73692  3.18206  2.89813
2.37843  2.77510  2.98578  4.58537  3.61563
          0.65264  0.74629  5.25725  1.53321  0.24314  0.34014  1.24366
    340   2.77146  4.92232  3.20224  2.64415  4.14693  3.61267  3.81824
2.78562  2.30828  2.61665  3.99721  2.32037  4.00176  2.50065  3.08073
2.12822  3.00248  3.24702  5.43276  3.10861    416 s - - -
          2.68618  4.42225  2.77519  2.73123  3.46354  2.40513  3.72494
3.29354  2.67741  2.69355  4.24690  2.90347  2.73739  3.18146  2.89801
2.37887  2.77519  2.98518  4.58477  3.61503
          0.01350  4.70791  5.43026  0.61958  0.77255  0.48576  0.95510
    341   2.86122  5.19650  3.01634  2.60434  4.63677  3.56129  3.90629
4.09726  2.68169  3.62990  4.41050  1.50001  4.03722  2.43208  3.14992
1.97530  2.50150  3.68141  5.79936  4.42200    417 n - - -
          2.68618  4.42225  2.77519  2.73123  3.46354  2.40513  3.72494
3.29354  2.67741  2.69355  4.24690  2.90347  2.73739  3.18146  2.89801
2.37887  2.77519  2.98518  4.58477  3.61503
          0.01350  4.70791  5.43026  0.61958  0.77255  0.48576  0.95510
    342   2.77576  4.51196  3.65207  3.10361  3.68136  3.73092  2.88991
1.55149  3.05085  2.75521  3.64758  3.50100  2.96264  3.36368  3.39956
2.47146  2.51318  2.80535  5.12300  3.88761    418 i - - -
          2.68618  4.42225  2.77519  2.73123  3.46354  2.40513  3.72494
3.29354  2.67741  2.69355  4.24690  2.90347  2.73739  3.18146  2.89801
2.37887  2.77519  2.98518  4.58477  3.61503
          0.01350  4.70791  5.43026  0.61958  0.77255  0.48576  0.95510
    343   2.94985  4.34069  4.68860  4.09620  1.91306  4.12609  4.45276
1.60291  2.80442  2.00794  3.35567  4.20446  4.47072  4.10541  4.02620
3.43470  3.18245  1.97311  4.99151  3.81055    419 i - - -
          2.68618  4.42225  2.77519  2.73123  3.46354  2.40513  3.72494
3.29354  2.67741  2.69355  4.24690  2.90347  2.73739  3.18146  2.89801
2.37887  2.77519  2.98518  4.58477  3.61503
```

0.01350 4.70791 5.43026 0.61958 0.77255 0.48576 0.95510
344 2.27702 5.44954 1.02304 2.62680 5.04629 3.56515 4.18725
4.52930 3.15683 4.07242 4.89401 3.17239 2.06587 3.35138 3.70519
3.08031 3.44272 4.06016 6.24523 4.80834 420 d - - -
2.68618 4.42225 2.77519 2.73123 3.46354 2.40513 3.72494
3.29354 2.67741 2.69355 4.24690 2.90347 2.73739 3.18146 2.89801
2.37887 2.77519 2.98518 4.58477 3.61503
0.01350 4.70791 5.43026 0.61958 0.77255 0.48576 0.95510
345 2.75604 4.65241 3.46891 2.91745 3.88930 2.75490 3.97187
2.60548 2.89339 2.94851 3.81511 2.72556 4.07579 3.21786 3.29796
2.08689 1.91160 2.51935 5.27876 4.01405 421 t - - -
2.68618 4.42225 2.77519 2.73123 3.46354 2.40513 3.72494
3.29354 2.67741 2.69355 4.24690 2.90347 2.73739 3.18146 2.89801
2.37887 2.77519 2.98518 4.58477 3.61503
0.01350 4.70791 5.43026 0.61958 0.77255 0.48576 0.95510
346 2.79374 5.17294 2.17041 2.51926 4.47917 3.56216 3.78414
3.93241 2.55432 2.43325 4.24368 2.17863 3.09741 2.90291 3.04008
2.37573 2.57360 3.54638 5.65099 4.26954 422 d - - -
2.68618 4.42225 2.77519 2.73123 3.46354 2.40513 3.72494
3.29354 2.67741 2.69355 4.24690 2.90347 2.73739 3.18146 2.89801
2.37887 2.77519 2.98518 4.58477 3.61503
0.01350 4.70791 5.43026 0.61958 0.77255 0.48576 0.95510
347 2.79334 5.17632 2.19650 2.52145 4.49738 2.79693 3.78922
3.95316 2.55918 2.73954 4.25857 2.54845 3.97239 2.90705 3.04571
2.13662 2.22035 3.56127 5.66462 4.28144 423 s - - -
2.68618 4.42225 2.77519 2.73123 3.46354 2.40513 3.72494
3.29354 2.67741 2.69355 4.24690 2.90347 2.73739 3.18146 2.89801
2.37887 2.77519 2.98518 4.58477 3.61503
0.18053 4.70791 1.85696 0.61958 0.77255 0.48576 0.95510
348 2.82648 4.52692 3.70028 3.14230 2.52702 3.78111 3.93620
3.03137 2.39803 2.71092 3.63267 3.51874 2.45158 3.36400 3.34010
3.04305 3.05871 2.81440 2.00010 3.44019 424 w - - -
2.68618 4.42225 2.77519 2.73123 3.46354 2.40513 3.72494
3.29354 2.67741 2.69355 4.24690 2.90347 2.73739 3.18146 2.89801
2.37887 2.77519 2.98518 4.58477 3.61503
0.12400 4.54331 2.24444 0.61958 0.77255 0.76305 0.62781
349 2.70158 4.70894 3.23325 2.67667 3.90816 3.56932 2.79548
2.23051 2.20525 2.94836 3.79989 3.16220 2.91126 2.98325 3.02068
2.79557 2.48565 3.01722 5.24433 3.95647 425 k - - -
2.68618 4.42225 2.77519 2.73123 3.46354 2.40513 3.72494
3.29354 2.67741 2.69355 4.24690 2.90347 2.73739 3.18146 2.89801
2.37887 2.77519 2.98518 4.58477 3.61503
0.01773 4.43704 5.15939 0.61958 0.77255 0.63425 0.75573
350 2.27989 4.60781 3.43299 2.91050 3.62129 3.66841 3.90719
3.14277 2.87631 2.29413 3.72537 1.92108 4.07274 3.20888 3.25879
2.92630 3.00902 2.90055 5.06870 2.32649 426 n - - -
2.68618 4.42225 2.77519 2.73123 3.46354 2.40513 3.72494
3.29354 2.67741 2.69355 4.24690 2.90347 2.73739 3.18146 2.89801
2.37887 2.77519 2.98518 4.58477 3.61503
0.13107 4.54331 2.18744 0.61958 0.77255 0.34474 1.23239
351 2.77602 5.15746 2.12814 2.48285 4.44752 3.53312 3.75633
3.90746 2.52990 3.44481 4.22141 3.02443 3.94593 2.12306 3.01538
2.37741 2.54653 3.52412 5.62308 2.55219 427 q - - -

```
         2.68618  4.42225  2.77519  2.73123  3.46354  2.40513  3.72494
3.29354  2.67741  2.69355  4.24690  2.90347  2.73739  3.18146  2.89801
2.37887  2.77519  2.98518  4.58477  3.61503
         0.09629  4.61273  2.50258  0.61958  0.77255  0.66070  0.72668
    352  2.74242  5.17508  2.94156  2.11543  4.49329  2.34843  2.91372
3.95845  2.46565  3.47119  4.23309  2.98004  3.90678  2.40243  2.95094
2.29887  2.46221  3.55343  5.63082  4.23767    428 e - - -
         2.68618  4.42225  2.77519  2.73123  3.46354  2.40513  3.72494
3.29354  2.67741  2.69355  4.24690  2.90347  2.73739  3.18146  2.89801
2.37887  2.77519  2.98518  4.58477  3.61503
         0.01610  4.53254  5.25489  0.61958  0.77255  0.77745  0.61541
    353  2.72610  5.14057  2.45308  2.43991  4.44594  3.49894  2.90796
3.02705  2.45874  3.42891  4.19383  2.15762  3.89981  2.42245  2.94443
2.70592  2.46548  3.51033  5.59679  4.20868    429 n - - -
         2.68650  4.42257  2.77467  2.73113  3.46298  2.40545  3.72527
3.29315  2.67733  2.69306  4.24722  2.90331  2.73772  3.18179  2.89833
2.37889  2.77552  2.98444  4.58509  3.61535
         0.37569  1.51726  2.36580  1.62769  0.21863  0.77745  0.61541
    354  2.68415  4.79184  3.16007  2.62296  4.08057  2.29998  3.78321
3.48226  2.17333  2.48566  3.93874  3.11664  3.93175  2.95440  3.02099
2.31324  2.36944  3.16735  5.37207  4.06198    437 k - - -
         2.68628  4.42235  2.77529  2.73133  3.46292  2.40523  3.72504
3.29293  2.67751  2.69330  4.24700  2.90357  2.73749  3.18156  2.89761
2.37897  2.77529  2.98528  4.58487  3.61513
         1.08779  0.82632  1.48996  0.10876  2.27250  0.48640  0.95409
    355  2.68403  4.76444  3.15302  2.60476  3.98127  3.53161  2.46537
2.70420  2.56998  3.01807  3.85581  3.10419  3.92686  2.47069  3.00321
2.30163  2.38452  3.08814  5.29312  3.98622    439 s - - -
         2.68618  4.42225  2.77519  2.73123  3.46354  2.40513  3.72494
3.29354  2.67741  2.69355  4.24690  2.90347  2.73739  3.18146  2.89801
2.37887  2.77519  2.98518  4.58477  3.61503
         0.01825  4.40846  5.13081  0.61958  0.77255  0.66391  0.72327
    356  2.82836  5.34968  1.92408  2.06644  4.67929  2.72016  3.75991
4.17033  2.55468  3.65043  4.40708  2.94633  2.88151  2.33313  3.06740
2.34911  3.07028  3.73450  5.78825  4.35855    440 d - - -
         2.68618  4.42225  2.77519  2.73123  3.46354  2.40513  3.72494
3.29354  2.67741  2.69355  4.24690  2.90347  2.73739  3.18146  2.89801
2.37887  2.77519  2.98518  4.58477  3.61503
         0.01635  4.51768  5.24003  0.61958  0.77255  0.79672  0.59930
    357  2.69048  4.55391  3.42044  2.87215  2.91031  3.59726  3.90669
3.13055  2.84681  2.83008  3.70433  2.19939  4.01546  3.16825  3.24289
2.08965  2.39878  2.35825  5.16687  3.90413    441 s - - -
         2.68618  4.42225  2.77519  2.73123  3.46354  2.40513  3.72494
3.29354  2.67741  2.69355  4.24690  2.90347  2.73739  3.18146  2.89801
2.37887  2.77519  2.98518  4.58477  3.61503
         0.01635  4.51768  5.24003  0.61958  0.77255  0.79672  0.59930
    358  2.71176  4.81585  2.52642  2.61296  4.04625  2.75066  3.78748
2.32649  2.62376  3.08023  3.91332  2.57735  3.95216  2.96253  3.07583
2.38098  2.94971  2.63166  5.35550  4.04107    442 i - - -
         2.68618  4.42225  2.77519  2.73123  3.46354  2.40513  3.72494
3.29354  2.67741  2.69355  4.24690  2.90347  2.73739  3.18146  2.89801
2.37887  2.77519  2.98518  4.58477  3.61503
         0.01635  4.51768  5.24003  0.61958  0.77255  0.79672  0.59930
```

```
    359   2.22949  5.17432  2.29004  2.49386  4.66706  1.76664  3.88148
4.14245  2.72170  3.66779  4.44776  2.39105  3.97032  3.01493  3.22814
2.30566  3.10505  3.70073  5.83609  4.43372    443 g - - -
          2.68618  4.42225  2.77519  2.73123  3.46354  2.40513  3.72494
3.29354  2.67741  2.69355  4.24690  2.90347  2.73739  3.18146  2.89801
2.37887  2.77519  2.98518  4.58477  3.61503
          0.01635  4.51768  5.24003  0.61958  0.77255  0.79672  0.59930
    360   3.03597  4.41983  4.69408  4.12100  3.43371  4.24222  4.60563
1.87994  3.98576  1.37146  3.33468  4.29088  2.80995  4.17569  4.12307
3.56047  3.27352  1.60273  5.15335  3.99024    444 l - - -
          2.68618  4.42225  2.77519  2.73123  3.46354  2.40513  3.72494
3.29354  2.67741  2.69355  4.24690  2.90347  2.73739  3.18146  2.89801
2.37887  2.77519  2.98518  4.58477  3.61503
          0.01635  4.51768  5.24003  0.61958  0.77255  0.54821  0.86271
    361   2.32493  5.15264  2.57083  2.47677  4.46169  2.78008  3.73688
3.91861  2.49611  2.74158  4.21536  1.97846  3.93087  2.85162  2.54118
2.73772  2.98852  3.52626  5.61973  4.23541    445 n - - -
          2.68618  4.42225  2.77519  2.73123  3.46354  2.40513  3.72494
3.29354  2.67741  2.69355  4.24690  2.90347  2.73739  3.18146  2.89801
2.37887  2.77519  2.98518  4.58477  3.61503
          0.01485  4.61273  5.33507  0.61958  0.77255  0.66070  0.72668
    362   2.74544  4.81594  3.24941  2.69764  4.06150  2.79463  3.83829
3.45077  1.99113  2.56609  3.93353  3.19010  3.99864  3.01966  3.08724
1.87514  2.98731  2.55661  5.37446  4.07188    446 s - - -
          2.68618  4.42225  2.77519  2.73123  3.46354  2.40513  3.72494
3.29354  2.67741  2.69355  4.24690  2.90347  2.73739  3.18146  2.89801
2.37887  2.77519  2.98518  4.58477  3.61503
          0.12007  4.61273  2.27090  0.61958  0.77255  0.66070  0.72668
    363   2.27898  4.22849  4.35624  3.77089  2.61171  3.91959  4.24210
1.64983  3.63737  1.92778  3.28208  3.94319  2.87869  3.83928  3.78697
3.21758  3.02249  2.33739  4.86281  3.67652    447 i - - -
          2.68618  4.42225  2.77519  2.73123  3.46354  2.40513  3.72494
3.29354  2.67741  2.69355  4.24690  2.90347  2.73739  3.18146  2.89801
2.37887  2.77519  2.98518  4.58477  3.61503
          0.01649  4.50914  5.23149  0.61958  0.77255  0.80751  0.59053
    364   2.72909  4.20791  4.20437  3.62434  2.15244  3.82948  4.16370
2.13903  3.50613  2.37990  3.32601  3.82830  4.20938  3.72829  3.68912
2.15134  2.44984  2.06849  4.84618  3.64973    448 v - - -
          2.68618  4.42225  2.77519  2.73123  3.46354  2.40513  3.72494
3.29354  2.67741  2.69355  4.24690  2.90347  2.73739  3.18146  2.89801
2.37887  2.77519  2.98518  4.58477  3.61503
          0.01649  4.50914  5.23149  0.61958  0.77255  0.80751  0.59053
    365   3.39687  5.03233  4.03466  3.74839  2.88714  4.06309  4.07337
3.75325  3.68202  3.26123  4.35361  3.97030  2.44261  3.99205  3.91609
3.54212  3.71231  3.54411  4.50938  0.71023    449 y - - -
          2.68618  4.42225  2.77519  2.73123  3.46354  2.40513  3.72494
3.29354  2.67741  2.69355  4.24690  2.90347  2.73739  3.18146  2.89801
2.37887  2.77519  2.98518  4.58477  3.61503
          0.01649  4.50914  5.23149  0.61958  0.77255  0.80751  0.59053
    366   2.70833  4.99061  2.50819  2.49532  3.14091  2.76222  3.72048
3.68659  2.50505  3.26235  4.05806  2.51722  3.02367  2.85514  2.97947
2.37327  2.94110  3.33800  5.47720  3.07064    450 s - - -
          2.68618  4.42225  2.77519  2.73123  3.46354  2.40513  3.72494
3.29354  2.67741  2.69355  4.24690  2.90347  2.73739  3.18146  2.89801
2.37887  2.77519  2.98518  4.58477  3.61503
```

```
        0.01649  4.50914  5.23149  0.61958  0.77255  0.80751  0.59053
    367   2.76909  5.07680  3.07042  2.55677  4.46370  3.52192  3.77050
3.90369  2.47223  3.44132  4.22833  2.06794  2.37883  2.89657  2.10021
2.77272  2.46352  3.51618  5.61204  4.26323    451 n - - -
        2.68618  4.42225  2.77519  2.73123  3.46354  2.40513  3.72494
3.29354  2.67741  2.69355  4.24690  2.90347  2.73739  3.18146  2.89801
2.37887  2.77519  2.98518  4.58477  3.61503
        0.01649  4.50914  5.23149  0.61958  0.77255  0.80751  0.59053
    368   2.74759  4.97534  2.21344  2.54933  4.21929  3.55045  3.75600
2.84166  1.71916  2.57590  4.03831  3.07181  3.94843  2.89971  2.97557
2.77182  2.97892  3.26713  5.47034  4.12915    452 k - - -
        2.68618  4.42225  2.77519  2.73123  3.46354  2.40513  3.72494
3.29354  2.67741  2.69355  4.24690  2.90347  2.73739  3.18146  2.89801
2.37887  2.77519  2.98518  4.58477  3.61503
        0.01649  4.50914  5.23149  0.61958  0.77255  0.54040  0.87350
    369   2.94190  5.25250  2.85598  1.14894  4.52341  3.57972  3.90073
2.71654  2.71782  3.52775  4.35765  2.41189  4.05827  3.04916  3.18985
2.92790  3.19168  3.56592  5.74922  4.36663    453 e - - -
        2.68618  4.42225  2.77519  2.73123  3.46354  2.40513  3.72494
3.29354  2.67741  2.69355  4.24690  2.90347  2.73739  3.18146  2.89801
2.37887  2.77519  2.98518  4.58477  3.61503
        0.01485  4.61273  5.33507  0.61958  0.77255  0.66070  0.72668
    370   3.24401  4.68160  4.63838  4.06234  2.35984  4.33645  4.48863
2.72163  3.80285  0.87121  3.28990  4.27881  4.63991  4.07723  2.67315
3.65270  3.46904  2.75058  4.99039  3.68697    454 l - - -
        2.68618  4.42225  2.77519  2.73123  3.46354  2.40513  3.72494
3.29354  2.67741  2.69355  4.24690  2.90347  2.73739  3.18146  2.89801
2.37887  2.77519  2.98518  4.58477  3.61503
        0.01485  4.61273  5.33507  0.61958  0.77255  0.66070  0.72668
    371   2.76869  5.01814  3.13009  2.57705  4.28964  3.57442  3.77076
2.86506  2.16910  3.29066  4.09456  3.09708  3.96917  1.76704  2.96025
2.35083  2.46453  3.36898  5.50839  4.16892    455 q - - -
        2.68618  4.42225  2.77519  2.73123  3.46354  2.40513  3.72494
3.29354  2.67741  2.69355  4.24690  2.90347  2.73739  3.18146  2.89801
2.37887  2.77519  2.98518  4.58477  3.61503
        0.01485  4.61273  5.33507  0.61958  0.77255  0.66070  0.72668
    372   2.76168  5.14324  3.02738  2.49395  4.44943  2.33947  3.73953
3.90128  2.19005  2.74893  4.20582  2.55068  3.93897  2.47173  2.95623
2.07842  2.99421  3.51534  5.60777  4.23156    456 s - - -
        2.68618  4.42225  2.77519  2.73123  3.46354  2.40513  3.72494
3.29354  2.67741  2.69355  4.24690  2.90347  2.73739  3.18146  2.89801
2.37887  2.77519  2.98518  4.58477  3.61503
        0.01485  4.61273  5.33507  0.61958  0.77255  0.66070  0.72668
    373   2.36865  4.30422  4.28879  3.70324  3.36855  3.95118  4.25601
1.84380  2.70341  1.57356  2.66132  3.92363  4.30921  3.80223  3.75284
3.24113  3.06843  2.41919  4.93363  3.74991    457 l - - -
        2.68618  4.42225  2.77519  2.73123  3.46354  2.40513  3.72494
3.29354  2.67741  2.69355  4.24690  2.90347  2.73739  3.18146  2.89801
2.37887  2.77519  2.98518  4.58477  3.61503
        0.01485  4.61273  5.33507  0.61958  0.77255  0.66070  0.72668
    374   2.76810  5.26528  2.55663  2.15355  4.60932  3.52417  3.71456
4.08948  2.12090  3.56567  4.30898  2.99253  3.92418  1.91384  2.42768
2.72992  2.49608  3.65595  5.69350  4.28987    458 q - - -
```

```
          2.68618  4.42225  2.77519  2.73123  3.46354  2.40513  3.72494
 3.29354  2.67741  2.69355  4.24690  2.90347  2.73739  3.18146  2.89801
 2.37887  2.77519  2.98518  4.58477  3.61503
          0.12007  4.61273  2.27090  0.61958  0.77255  0.66070  0.72668
    375   3.56529  4.83397  5.39952  4.83636  1.54299  4.85405  5.03125
 1.60040  4.68601  1.22888  3.11824  4.95110  5.01749  4.67161  4.69016
 4.21237  3.78202  2.50455  5.21124  3.99846    459 l - - -
          2.68618  4.42225  2.77519  2.73123  3.46354  2.40513  3.72494
 3.29354  2.67741  2.69355  4.24690  2.90347  2.73739  3.18146  2.89801
 2.37887  2.77519  2.98518  4.58477  3.61503
          0.01649  4.50914  5.23149  0.61958  0.77255  0.80751  0.59053
    376   2.07899  5.18113  3.06381  2.51037  4.51267  3.55182  2.37417
 3.95925  2.04907  3.46434  4.23866  2.46613  3.94461  2.82503  2.36792
 2.77103  3.01553  3.56810  5.60871  4.25157    460 k - - -
          2.68618  4.42225  2.77519  2.73123  3.46354  2.40513  3.72494
 3.29354  2.67741  2.69355  4.24690  2.90347  2.73739  3.18146  2.89801
 2.37887  2.77519  2.98518  4.58477  3.61503
          0.01649  4.50914  5.23149  0.61958  0.77255  0.80751  0.59053
    377   2.70465  4.34482  3.72890  3.15871  3.47141  2.87658  3.99077
 2.80486  3.09109  2.16350  2.74495  3.52085  4.09717  2.63900  3.40402
 2.97687  2.48175  1.92465  4.94324  3.72423    461 v - - -
          2.68618  4.42225  2.77519  2.73123  3.46354  2.40513  3.72494
 3.29354  2.67741  2.69355  4.24690  2.90347  2.73739  3.18146  2.89801
 2.37887  2.77519  2.98518  4.58477  3.61503
          0.01649  4.50914  5.23149  0.61958  0.77255  0.80751  0.59053
    378   3.53671  5.18910  3.86970  3.56052  2.72118  4.18034  3.89043
 3.80457  3.33329  3.23007  4.34923  3.83935  4.61433  2.46792  3.56932
 3.60748  3.78230  3.62714  4.35164  0.75425    462 y - - -
          2.68660  4.42267  2.77482  2.73165  3.46396  2.40554  3.72441
 3.29396  2.67714  2.69397  4.24731  2.90305  2.73709  3.18188  2.89842
 2.37852  2.77488  2.98515  4.58519  3.61291
          0.87064  0.74358  2.24519  1.21375  0.35251  0.80751  0.59053
    379   2.65810  3.14624  3.07883  2.55699  4.24647  2.68091  3.76380
 3.67365  2.18089  3.26170  4.06431  2.11404  3.89405  2.90963  3.00202
 2.22527  2.92389  3.31055  5.48357  4.14697    470 n - - -
          2.68618  4.42225  2.77519  2.73123  3.46354  2.40513  3.72494
 3.29354  2.67741  2.69355  4.24690  2.90347  2.73739  3.18146  2.89801
 2.37887  2.77519  2.98518  4.58477  3.61503
          0.01835  4.40316  5.12550  0.61958  0.77255  0.46336  0.99201
    380   2.70455  4.91761  3.18357  2.72384  4.58421  2.61466  3.97340
 4.04120  2.77888  3.60060  4.37686  2.07102  2.76982  3.11287  3.24289
 1.63954  2.43196  3.58086  5.78382  4.43657    471 s - - -
          2.68618  4.42225  2.77519  2.73123  3.46354  2.40513  3.72494
 3.29354  2.67741  2.69355  4.24690  2.90347  2.73739  3.18146  2.89801
 2.37887  2.77519  2.98518  4.58477  3.61503
          0.01485  4.61273  5.33507  0.61958  0.77255  0.66070  0.72668
    381   2.75704  5.05102  2.53020  2.55223  4.40882  3.52095  3.81020
 3.84749  2.60157  3.41400  4.20280  2.49363  3.95978  2.94262  3.07995
 1.62275  2.43809  2.77391  5.61657  4.25271    472 s - - -
          2.68618  4.42225  2.77519  2.73123  3.46354  2.40513  3.72494
 3.29354  2.67741  2.69355  4.24690  2.90347  2.73739  3.18146  2.89801
 2.37887  2.77519  2.98518  4.58477  3.61503
          0.01485  4.61273  5.33507  0.61958  0.77255  0.66070  0.72668
```

```
    382   2.73950  3.15203  3.65794  2.58048  2.71957  2.87857  3.98464
2.92904  3.04394  2.64538  3.54179  2.81793  4.10801  3.34159  3.38421
2.97773  2.97286  2.70764  2.18956  3.76464    473 w - - -
          2.68618  4.42225  2.77519  2.73123  3.46354  2.40513  3.72494
3.29354  2.67741  2.69355  4.24690  2.90347  2.73739  3.18146  2.89801
2.37887  2.77519  2.98518  4.58477  3.61503
          0.01485  4.61273  5.33507  0.61958  0.77255  0.66070  0.72668
    383   2.98630  4.82640  3.61625  3.07025  2.06231  3.86383  2.25962
3.39304  3.00003  3.00510  3.93155  2.69754  4.23938  3.33241  2.55868
3.10470  3.21450  3.15381  4.69134  2.02979    474 y - - -
          2.68618  4.42225  2.77519  2.73123  3.46354  2.40513  3.72494
3.29354  2.67741  2.69355  4.24690  2.90347  2.73739  3.18146  2.89801
2.37887  2.77519  2.98518  4.58477  3.61503
          0.01485  4.61273  5.33507  0.61958  0.77255  0.66070  0.72668
    384   1.80362  1.65608  3.70607  3.19683  4.10940  3.49016  4.19480
3.48067  2.32524  3.17628  4.03674  3.51849  4.06722  3.45975  3.45967
2.21314  3.02459  3.14431  5.48999  4.25364    475 c - - -
          2.68618  4.42225  2.77519  2.73123  3.46354  2.40513  3.72494
3.29354  2.67741  2.69355  4.24690  2.90347  2.73739  3.18146  2.89801
2.37887  2.77519  2.98518  4.58477  3.61503
          0.01485  4.61273  5.33507  0.61958  0.77255  0.66070  0.72668
    385   2.86669  4.65276  3.59816  3.05260  3.31925  2.27548  2.92760
3.19115  3.00596  2.85597  3.76368  3.46437  4.17045  3.32005  3.37533
2.39917  3.09998  2.95749  3.13728  1.91792    476 y - - -
          2.68618  4.42225  2.77519  2.73123  3.46354  2.40513  3.72494
3.29354  2.67741  2.69355  4.24690  2.90347  2.73739  3.18146  2.89801
2.37887  2.77519  2.98518  4.58477  3.61503
          0.01485  4.61273  5.33507  0.61958  0.77255  0.66070  0.72668
    386   2.77289  4.77902  3.29759  2.80154  4.06685  3.59599  3.92941
2.63214  2.76281  3.10770  3.96591  3.26935  1.59060  2.49777  3.16723
2.39742  3.03786  3.15616  5.40472  4.11560    477 p - - -
          2.68618  4.42225  2.77519  2.73123  3.46354  2.40513  3.72494
3.29354  2.67741  2.69355  4.24690  2.90347  2.73739  3.18146  2.89801
2.37887  2.77519  2.98518  4.58477  3.61503
          0.01485  4.61273  5.33507  0.61958  0.77255  0.66070  0.72668
    387   3.09199  5.45181  1.82920  2.53152  4.24068  3.60086  3.95843
4.19338  2.92400  3.72134  4.57254  1.89805  4.13215  3.16832  3.44538
3.04180  3.35403  3.82922  5.62185  1.61864    478 y - - -
          2.68618  4.42225  2.77519  2.73123  3.46354  2.40513  3.72494
3.29354  2.67741  2.69355  4.24690  2.90347  2.73739  3.18146  2.89801
2.37887  2.77519  2.98518  4.58477  3.61503
          0.01485  4.61273  5.33507  0.61958  0.77255  0.66070  0.72668
    388   2.68846  4.61308  3.99718  3.87526  5.03005  0.60681  4.94924
4.45680  4.02036  4.17525  5.00778  3.89517  4.17340  4.27308  4.25316
2.88054  2.09718  3.79145  6.33596  5.19973    479 G - - -
          2.68618  4.42225  2.77519  2.73123  3.46354  2.40513  3.72494
3.29354  2.67741  2.69355  4.24690  2.90347  2.73739  3.18146  2.89801
2.37887  2.77519  2.98518  4.58477  3.61503
          0.01485  4.61273  5.33507  0.61958  0.77255  0.66070  0.72668
    389   2.74364  4.17897  4.36841  3.77471  2.62010  2.86159  2.90368
2.20066  3.62688  1.85066  3.29292  3.91239  4.23696  3.81830  3.75196
3.16563  2.97566  2.11177  4.77426  2.72141    480 l - - -
          2.68618  4.42225  2.77519  2.73123  3.46354  2.40513  3.72494
3.29354  2.67741  2.69355  4.24690  2.90347  2.73739  3.18146  2.89801
2.37887  2.77519  2.98518  4.58477  3.61503
```

```
          0.01485  4.61273  5.33507  0.61958  0.77255  0.66070  0.72668
    390   2.75386  4.99003  3.13348  2.20278  4.24409  3.57202  3.76280
2.48333  2.22615  2.65732  4.05741  3.09257  3.96088  2.43733  2.55410
2.77687  2.48600  3.33055  5.47765  4.13939    481 e - - -
          2.68618  4.42225  2.77519  2.73123  3.46354  2.40513  3.72494
3.29354  2.67741  2.69355  4.24690  2.90347  2.73739  3.18146  2.89801
2.37887  2.77519  2.98518  4.58477  3.61503
          0.01485  4.61273  5.33507  0.61958  0.77255  0.66070  0.72668
    391   2.77793  4.58964  3.51170  3.00097  3.78884  2.72785  4.00114
3.14398  2.92574  1.44501  3.73387  3.42014  4.10409  2.55764  3.28018
2.95803  2.40790  2.90499  5.20996  3.96452    482 l - - -
          2.68607  4.42251  2.77545  2.73149  3.46380  2.40539  3.72406
3.29311  2.67767  2.69343  4.24716  2.90373  2.73726  3.18111  2.89827
2.37913  2.77545  2.98490  4.58254  3.61332
          0.22792  1.61450  5.33507  1.44102  0.27008  0.39012  1.13004
    392   2.82977  5.31289  2.43289  2.15043  4.63762  3.55463  3.78309
3.00318  2.54811  3.60961  4.36322  1.76238  3.97524  2.42055  3.04610
2.42311  3.06531  3.69335  5.75487  4.34451    490 n - - -
          2.68618  4.42225  2.77519  2.73123  3.46354  2.40513  3.72494
3.29354  2.67741  2.69355  4.24690  2.90347  2.73739  3.18146  2.89801
2.37887  2.77519  2.98518  4.58477  3.61503
          0.01350  4.70791  5.43026  0.61958  0.77255  0.48576  0.95510
    393   3.16507  4.60074  4.55600  4.03620  2.20074  4.19149  4.08127
3.05182  3.90645  2.72637  3.72560  4.11868  4.56048  4.06570  4.04876
2.49058  3.40315  2.26025  4.45170  1.11940    491 y - - -
          2.68618  4.42225  2.77519  2.73123  3.46354  2.40513  3.72494
3.29354  2.67741  2.69355  4.24690  2.90347  2.73739  3.18146  2.89801
2.37887  2.77519  2.98518  4.58477  3.61503
          0.01350  4.70791  5.43026  0.61958  0.77255  0.48576  0.95510
    394   2.40645  5.21308  3.04045  2.50340  4.54457  3.55609  3.76124
4.00953  2.22078  3.52013  4.27852  2.24238  3.95853  2.44294  2.98605
2.04626  2.24560  3.60092  5.67667  4.28654    492 s - - -
          2.68618  4.42225  2.77519  2.73123  3.46354  2.40513  3.72494
3.29354  2.67741  2.69355  4.24690  2.90347  2.73739  3.18146  2.89801
2.37887  2.77519  2.98518  4.58477  3.61503
          0.01350  4.70791  5.43026  0.61958  0.77255  0.48576  0.95510
    395   2.86290  5.34280  1.88112  2.47903  4.66051  3.56191  3.80944
4.14288  2.24544  3.63884  4.39849  1.91509  3.99576  2.92131  3.08533
2.35796  3.10081  3.72280  5.78505  3.00859    493 d - - -
          2.68618  4.42225  2.77519  2.73123  3.46354  2.40513  3.72494
3.29354  2.67741  2.69355  4.24690  2.90347  2.73739  3.18146  2.89801
2.37887  2.77519  2.98518  4.58477  3.61503
          0.01350  4.70791  5.43026  0.61958  0.77255  0.48576  0.95510
    396   2.80787  5.13314  3.07683  2.56925  4.45591  2.41339  3.81492
3.89958  2.25291  2.72517  4.23614  1.92546  3.99476  2.94158  3.04166
2.09134  3.04987  3.52392  5.64164  4.27650    494 n - - -
          2.68618  4.42225  2.77519  2.73123  3.46354  2.40513  3.72494
3.29354  2.67741  2.69355  4.24690  2.90347  2.73739  3.18146  2.89801
2.37887  2.77519  2.98518  4.58477  3.61503
          0.18053  4.70791  1.85696  0.61958  0.77255  0.48576  0.95510
    397   2.58155  4.46149  3.89001  3.44534  4.24843  3.37783  4.40824
3.49628  3.40270  3.30415  4.18282  3.64074  4.05082  3.70287  3.72290
1.18967  1.78329  2.26185  5.65836  4.44070    495 s - - -
```

```
          2.68618  4.42225  2.77519  2.73123  3.46354  2.40513  3.72494
3.29354  2.67741  2.69355  4.24690  2.90347  2.73739  3.18146  2.89801
2.37887  2.77519  2.98518  4.58477  3.61503
          0.12400  4.54331  2.24444  0.61958  0.77255  0.34474  1.23239
    398   2.75829  5.11226  3.05978  2.18557  4.40347  3.54872  3.73871
2.50967  2.48236  3.39082  4.17002  2.56562  3.94218  2.07653  2.53911
2.75168  2.54530  3.47374  5.57568  4.20828    496 q - - -
          2.68618  4.42225  2.77519  2.73123  3.46354  2.40513  3.72494
3.29354  2.67741  2.69355  4.24690  2.90347  2.73739  3.18146  2.89801
2.37887  2.77519  2.98518  4.58477  3.61503
          0.01477  4.61851  5.34086  0.61958  0.77255  0.65133  0.73679
    399   2.75676  4.22305  4.25464  3.67002  2.65131  3.86159  4.15542
1.90652  3.54326  2.40874  3.33783  3.85817  4.23259  3.75647  3.71065
2.49525  2.12521  2.46312  4.80348  2.37257    497 i - - -
          2.68620  4.42227  2.77522  2.73126  3.46356  2.40515  3.72497
3.29356  2.67743  2.69357  4.24692  2.90349  2.73742  3.18149  2.89757
2.37889  2.77522  2.98521  4.58479  3.61505
          0.11071  2.30247  5.34086  0.28170  1.40447  0.65133  0.73679
    400   2.39177  5.08643  2.61543  2.50963  4.37195  3.54341  3.74075
3.81390  2.19797  2.31016  4.14815  3.04319  3.93808  2.86374  2.53804
2.04332  2.97928  3.44542  5.55965  4.19317    499 s - - -
          2.68618  4.42225  2.77519  2.73123  3.46354  2.40513  3.72494
3.29354  2.67741  2.69355  4.24690  2.90347  2.73739  3.18146  2.89801
2.37887  2.77519  2.98518  4.58477  3.61503
          0.12097  4.61851  2.26264  0.61958  0.77255  0.65133  0.73679
    401   3.25111  4.96923  3.87162  3.62564  3.13430  1.74787  4.17984
3.84094  3.66305  3.40075  4.43139  3.88633  4.47663  3.95687  3.92688
3.40110  3.60529  3.57491  4.72943  0.88327    500 y - - -
          2.68618  4.42225  2.77519  2.73123  3.46354  2.40513  3.72494
3.29354  2.67741  2.69355  4.24690  2.90347  2.73739  3.18146  2.89801
2.37887  2.77519  2.98518  4.58477  3.61503
          0.01641  4.51395  5.23629  0.61958  0.77255  0.80147  0.59542
    402   2.67153  4.65682  3.35191  3.05729  4.42010  1.13320  4.25523
3.80177  3.17900  3.50795  4.35872  2.31240  4.03673  3.48872  3.56159
2.80866  3.08003  2.42962  5.76225  4.48124    501 g - - -
          2.68618  4.42225  2.77519  2.73123  3.46354  2.40513  3.72494
3.29354  2.67741  2.69355  4.24690  2.90347  2.73739  3.18146  2.89801
2.37887  2.77519  2.98518  4.58477  3.61503
          0.12370  4.51395  2.24999  0.61958  0.77255  0.80147  0.59542
    403   2.73511  5.05785  1.85139  2.44468  4.34298  3.47616  3.72366
2.74747  2.11598  3.34253  4.13866  2.98210  3.90299  2.85653  2.98590
2.31461  2.97400  3.40991  5.54719  4.17727    502 d - - -
          2.68618  4.42225  2.77519  2.73123  3.46354  2.40513  3.72494
3.29354  2.67741  2.69355  4.24690  2.90347  2.73739  3.18146  2.89801
2.37887  2.77519  2.98518  4.58477  3.61503
          0.12443  4.40850  2.25546  0.61958  0.77255  0.46653  0.98665
    404   2.78879  5.11197  2.94063  2.52857  4.52783  3.49120  3.82520
3.98267  2.59960  3.52319  4.30946  1.60402  3.96262  2.37208  3.06348
2.24063  2.40852  3.57894  5.69975  4.32722    503 n - - -
          2.68618  4.42225  2.77519  2.73123  3.46354  2.40513  3.72494
3.29354  2.67741  2.69355  4.24690  2.90347  2.73739  3.18146  2.89801
2.37887  2.77519  2.98518  4.58477  3.61503
          0.01617  4.52865  5.25099  0.61958  0.77255  0.78256  0.61107
```

```
    405   2.80555  5.20283  1.62548  2.47347  4.50283  3.52937  3.74728
3.96131  2.48165  2.69558  4.26297  3.01376  3.94968  2.42989  2.37698
2.78135  3.03828  3.57469  5.64628  4.26625    504 d - - -
          2.68618  4.42225  2.77519  2.73123  3.46354  2.40513  3.72494
3.29354  2.67741  2.69355  4.24690  2.90347  2.73739  3.18146  2.89801
2.37887  2.77519  2.98518  4.58477  3.61503
          0.01617  4.52865  5.25099  0.61958  0.77255  0.51198  0.91456
    406   2.77809  5.02768  3.07518  2.59027  4.36194  2.69910  3.83039
3.79309  2.61869  3.37270  4.17389  2.48091  1.90103  2.43067  3.08139
2.79897  3.03138  2.67310  5.58873  4.23917    505 p - - -
          2.68618  4.42225  2.77519  2.73123  3.46354  2.40513  3.72494
3.29354  2.67741  2.69355  4.24690  2.90347  2.73739  3.18146  2.89801
2.37887  2.77519  2.98518  4.58477  3.61503
          0.01457  4.63176  5.35411  0.61958  0.77255  0.40544  1.09866
    407   2.38735  5.26615  2.57259  2.47860  4.60878  2.87380  3.76265
4.08658  2.51532  3.57940  4.32800  2.21175  3.95618  2.42296  3.00905
2.05350  2.56263  3.65991  5.72246  4.31701    506 s - - -
          2.68618  4.42225  2.77519  2.73123  3.46354  2.40513  3.72494
3.29354  2.67741  2.69355  4.24690  2.90347  2.73739  3.18146  2.89801
2.37887  2.77519  2.98518  4.58477  3.61503
          0.01350  4.70791  5.43026  0.61958  0.77255  0.48576  0.95510
    408   2.32114  4.21360  4.44341  3.84865  2.71694  2.94121  4.24049
1.94744  3.69698  2.10248  3.33238  3.97570  4.28622  3.88523  3.81373
2.54889  3.01793  1.83626  4.83927  3.65444    507 v - - -
          2.68618  4.42225  2.77519  2.73123  3.46354  2.40513  3.72494
3.29354  2.67741  2.69355  4.24690  2.90347  2.73739  3.18146  2.89801
2.37887  2.77519  2.98518  4.58477  3.61503
          0.01350  4.70791  5.43026  0.61958  0.77255  0.48576  0.95510
    409   2.87676  5.36991  1.83144  2.47813  4.71016  3.55885  3.82439
4.19440  2.23869  3.68242  4.43929  2.45314  4.00326  2.93560  3.10895
1.81553  2.60565  3.76371  5.82432  4.40559    508 s - - -
          2.68618  4.42225  2.77519  2.73123  3.46354  2.40513  3.72494
3.29354  2.67741  2.69355  4.24690  2.90347  2.73739  3.18146  2.89801
2.37887  2.77519  2.98518  4.58477  3.61503
          0.11004  4.70791  2.35204  0.61958  0.77255  0.48576  0.95510
    410   2.75581  4.89787  3.14738  2.28130  4.17322  2.82180  3.83498
3.57883  2.65379  2.56856  4.02585  3.14629  2.09444  3.00034  3.10537
2.80778  2.16367  3.26024  5.45953  4.13711    509 p - - -
          2.68618  4.42225  2.77519  2.73123  3.46354  2.40513  3.72494
3.29354  2.67741  2.69355  4.24690  2.90347  2.73739  3.18146  2.89801
2.37887  2.77519  2.98518  4.58477  3.61503
          0.23223  4.61273  1.62299  0.61958  0.77255  0.66070  0.72668
    411   2.72121  5.12795  2.40756  2.39806  4.43140  3.45687  3.69152
3.89215  2.46585  2.70362  4.19248  2.40208  3.87859  2.33647  2.95431
2.03455  2.95796  3.49949  5.59179  4.20106    510 s - - -
          2.68618  4.42225  2.77519  2.73123  3.46354  2.40513  3.72494
3.29354  2.67741  2.69355  4.24690  2.90347  2.73739  3.18146  2.89801
2.37887  2.77519  2.98518  4.58477  3.61503
          0.01843  4.39893  5.12128  0.61958  0.77255  0.46088  0.99622
    412   2.29400  4.52779  3.59632  3.04495  3.74302  3.69179  4.02413
2.38964  3.00703  2.78470  3.68624  2.03673  4.10945  3.32062  3.37832
2.95500  2.07015  2.31806  5.18285  3.93844    511 n - - -
          2.68644  4.42110  2.77542  2.73188  3.46419  2.40525  3.72377
3.29419  2.67735  2.69381  4.24755  2.90067  2.73682  3.18157  2.89866
2.37925  2.77585  2.98533  4.58542  3.61310
```

```
          1.26081  0.60727  1.76178  1.41675  0.27773  0.39012  1.13004
    413   2.73128  4.36461  3.77206  3.21175  3.50569  3.74309  4.03952
1.72791  3.14145  2.15888  3.48301  2.81725  3.01547  3.43298  3.44857
3.01139  2.38489  2.57296  4.98458  3.76587    520 i - - -
          2.68618  4.42225  2.77519  2.73123  3.46354  2.40513  3.72494
3.29354  2.67741  2.69355  4.24690  2.90347  2.73739  3.18146  2.89801
2.37887  2.77519  2.98518  4.58477  3.61503
          0.01593  4.54331  5.26566  0.61958  0.77255  0.34474  1.23239
    414   2.37474  5.13760  2.22826  2.52076  4.42726  3.56476  3.76213
3.87601  2.22851  3.41915  4.19437  3.05804  3.95922  2.49380  3.00512
2.43012  3.00323  2.87587  5.60520  2.55924    521 d - - -
          2.68618  4.42225  2.77519  2.73123  3.46354  2.40513  3.72494
3.29354  2.67741  2.69355  4.24690  2.90347  2.73739  3.18146  2.89801
2.37887  2.77519  2.98518  4.58477  3.61503
          0.01350  4.70791  5.43026  0.61958  0.77255  0.48576  0.95510
    415   2.77716  4.31549  4.04000  3.46148  3.42328  3.84314  4.13053
2.05492  3.36516  1.69246  3.42485  3.74399  4.21585  2.83713  3.60774
2.54886  2.44579  2.56833  4.91911  2.93492    522 l - - -
          2.68618  4.42225  2.77519  2.73123  3.46354  2.40513  3.72494
3.29354  2.67741  2.69355  4.24690  2.90347  2.73739  3.18146  2.89801
2.37887  2.77519  2.98518  4.58477  3.61503
          0.01350  4.70791  5.43026  0.61958  0.77255  0.48576  0.95510
    416   2.82828  5.32730  1.89710  2.46914  4.66593  3.55076  3.78126
4.15001  2.17072  3.63360  4.38177  2.18702  3.03970  2.88624  3.04126
2.36689  2.60382  3.71702  5.77059  4.35589    523 d - - -
          2.68618  4.42225  2.77519  2.73123  3.46354  2.40513  3.72494
3.29354  2.67741  2.69355  4.24690  2.90347  2.73739  3.18146  2.89801
2.37887  2.77519  2.98518  4.58477  3.61503
          0.01350  4.70791  5.43026  0.61958  0.77255  0.48576  0.95510
    417   2.03296  4.82224  3.32322  2.82554  2.91296  2.74098  3.99268
3.65014  2.84744  3.28509  4.11433  1.61195  4.04652  3.17784  3.28736
2.38138  3.04973  3.30834  5.54753  4.24159    524 n - - -
          2.68618  4.42225  2.77519  2.73123  3.46354  2.40513  3.72494
3.29354  2.67741  2.69355  4.24690  2.90347  2.73739  3.18146  2.89801
2.37887  2.77519  2.98518  4.58477  3.61503
          0.01350  4.70791  5.43026  0.61958  0.77255  0.48576  0.95510
    418   2.80212  4.74181  3.91263  3.80282  5.06054  3.45751  4.94108
4.60698  3.97198  4.26710  5.10201  3.90654  0.58852  4.25203  4.22957
2.05816  3.33436  3.93150  6.36965  5.17210    525 P - - -
          2.68618  4.42225  2.77519  2.73123  3.46354  2.40513  3.72494
3.29354  2.67741  2.69355  4.24690  2.90347  2.73739  3.18146  2.89801
2.37887  2.77519  2.98518  4.58477  3.61503
          0.01350  4.70791  5.43026  0.61958  0.77255  0.48576  0.95510
    419   3.80308  5.03136  5.73481  5.16830  3.48511  5.20334  5.55665
1.06767  5.03116  1.27877  1.92611  5.34512  5.29916  4.99073  5.02633
4.58698  4.01978  2.48277  5.65573  4.65462    526 i - - -
          2.68620  4.42227  2.77522  2.73126  3.46356  2.40515  3.72497
3.29356  2.67743  2.69318  4.24692  2.90349  2.73742  3.18149  2.89803
2.37889  2.77522  2.98521  4.58479  3.61506
          0.10417  2.35870  5.43026  0.27611  1.42185  0.48576  0.95510
    420   2.77239  4.49993  3.63724  3.07444  2.34354  3.74590  4.00369
2.13957  3.02893  2.71411  3.60610  2.74029  4.12872  2.71441  3.38395
2.53136  2.24265  2.77455  5.08253  3.84577    528 i - - -
```

```
          2.68618  4.42225  2.77519  2.73123  3.46354  2.40513  3.72494
3.29354  2.67741  2.69355  4.24690  2.90347  2.73739  3.18146  2.89801
2.37887  2.77519  2.98518  4.58477  3.61503
          0.01350  4.70791  5.43026  0.61958  0.77255  0.48576  0.95510
    421   2.77196  5.18962  2.61059  2.49819  4.49890  2.83242  3.75025
3.95948  2.18631  2.88873  4.24158  3.03961  3.95091  1.92377  2.98521
2.34888  3.00351  2.90460  5.64526  4.25782    529 q - - -
          2.68618  4.42225  2.77519  2.73123  3.46354  2.40513  3.72494
3.29354  2.67741  2.69355  4.24690  2.90347  2.73739  3.18146  2.89801
2.37887  2.77519  2.98518  4.58477  3.61503
          0.01350  4.70791  5.43026  0.61958  0.77255  0.48576  0.95510
    422   2.95028  4.32232  4.77164  4.18090  3.42906  4.15145  4.50083
1.40760  4.01195  2.01401  3.40668  4.25773  4.49910  4.17981  4.08803
3.46599  2.47293  1.72912  5.02647  2.73279    530 i - - -
          2.68618  4.42225  2.77519  2.73123  3.46354  2.40513  3.72494
3.29354  2.67741  2.69355  4.24690  2.90347  2.73739  3.18146  2.89801
2.37887  2.77519  2.98518  4.58477  3.61503
          0.01350  4.70791  5.43026  0.61958  0.77255  0.48576  0.95510
    423   2.92056  2.50340  3.96547  3.72231  4.67621  3.59554  4.76458
4.18428  3.73579  3.93240  4.82418  0.60277  4.32654  4.08426  4.01699
3.09823  3.39603  3.71805  6.03572  4.76852    531 N - - -
          2.68618  4.42225  2.77519  2.73123  3.46354  2.40513  3.72494
3.29354  2.67741  2.69355  4.24690  2.90347  2.73739  3.18146  2.89801
2.37887  2.77519  2.98518  4.58477  3.61503
          0.01350  4.70791  5.43026  0.61958  0.77255  0.48576  0.95510
    424   1.83999  4.33180  4.80839  4.20779  2.56756  4.14530  4.47982
2.08253  4.02831  1.87272  2.13751  4.26645  4.48399  4.16673  4.07895
3.45852  3.19052  2.05915  4.97080  3.80981    532 a - - -
          2.68618  4.42225  2.77519  2.73123  3.46354  2.40513  3.72494
3.29354  2.67741  2.69355  4.24690  2.90347  2.73739  3.18146  2.89801
2.37887  2.77519  2.98518  4.58477  3.61503
          0.01350  4.70791  5.43026  0.61958  0.77255  0.48576  0.95510
    425   2.42198  5.33896  2.11047  2.48987  4.67802  3.56873  3.78912
4.15657  2.52820  3.63899  4.39199  2.56133  3.98909  2.46484  1.70591
2.80963  3.08486  3.72874  5.77295  4.36748    533 r - - -
          2.68623  4.42230  2.77525  2.73128  3.46359  2.40518  3.72500
3.29359  2.67746  2.69360  4.24695  2.90302  2.73745  3.18152  2.89806
2.37892  2.77525  2.98524  4.58482  3.61410
          0.32741  1.69263  2.35204  0.17539  1.82716  0.48576  0.95510
    426   2.72588  4.86875  3.22320  2.72884  4.32725  3.50651  2.91863
3.75672  2.73398  3.36092  4.17237  3.20552  2.80916  3.08625  3.17621
1.69874  1.81215  3.38498  5.59072  4.26747    535 s - - -
          2.68618  4.42225  2.77519  2.73123  3.46354  2.40513  3.72494
3.29354  2.67741  2.69355  4.24690  2.90347  2.73739  3.18146  2.89801
2.37887  2.77519  2.98518  4.58477  3.61503
          0.01485  4.61273  5.33507  0.61958  0.77255  0.66070  0.72668
    427   3.25722  4.71088  4.44363  3.92917  2.05635  4.23571  3.94690
3.19318  3.81255  2.23452  3.81537  4.04880  4.58269  1.80256  3.99742
3.53092  3.48368  3.02234  2.09710  2.08101    536 q - - -
          2.68618  4.42225  2.77519  2.73123  3.46354  2.40513  3.72494
3.29354  2.67741  2.69355  4.24690  2.90347  2.73739  3.18146  2.89801
2.37887  2.77519  2.98518  4.58477  3.61503
          0.10025  4.61273  2.45969  0.61958  0.77255  0.66070  0.72668
```

428 2.30385 4.93789 3.09009 2.54405 4.19267 3.53350 3.74525
3.61393 2.18958 3.20865 4.01709 2.56105 2.92959 2.89104 2.99331
2.74454 2.50471 3.28218 5.44257 2.53184 537 k - - -
2.68618 4.42225 2.77519 2.73123 3.46354 2.40513 3.72494
3.29354 2.67741 2.69355 4.24690 2.90347 2.73739 3.18146 2.89801
2.37887 2.77519 2.98518 4.58477 3.61503
0.01617 4.52865 5.25099 0.61958 0.77255 0.78256 0.61107
429 2.33650 4.95603 3.08959 2.54118 4.25289 3.51695 3.74916
3.67772 1.89396 3.26231 4.06212 2.58848 3.92398 2.88788 2.98996
2.33985 2.45786 2.65444 5.48383 4.13838 538 k - - -
2.68618 4.42225 2.77519 2.73123 3.46354 2.40513 3.72494
3.29354 2.67741 2.69355 4.24690 2.90347 2.73739 3.18146 2.89801
2.37887 2.77519 2.98518 4.58477 3.61503
0.22015 4.52865 1.67770 0.61958 0.77255 0.78256 0.61107
430 1.13968 4.38379 3.58622 3.26163 4.51092 2.09060 4.36077
3.93239 3.33022 3.60161 4.41531 3.45649 2.31913 3.61111 3.67294
2.58883 2.89714 3.38069 5.83476 4.61635 539 a - - -
2.68618 4.42225 2.77519 2.73123 3.46354 2.40513 3.72494
3.29354 2.67741 2.69355 4.24690 2.90347 2.73739 3.18146 2.89801
2.37887 2.77519 2.98518 4.58477 3.61503
0.15093 4.32829 2.06434 0.61958 0.77255 0.77176 0.62027
431 2.67935 4.55945 3.32278 2.76900 2.72766 3.58310 3.80090
2.42611 1.95923 2.76495 3.65157 2.60207 3.97009 3.05427 3.07141
2.82262 2.91075 2.82578 5.08223 3.79417 540 k - - -
2.68618 4.42225 2.77519 2.73123 3.46354 2.40513 3.72494
3.29354 2.67741 2.69355 4.24690 2.90347 2.73739 3.18146 2.89801
2.37887 2.77519 2.98518 4.58477 3.61503
0.02006 4.31488 5.03723 0.61958 0.77255 1.00710 0.45456
432 1.60434 4.35835 3.59155 3.10208 3.86436 2.49032 4.07856
2.30479 3.07494 2.93274 3.80937 3.40886 3.93308 3.37651 3.42331
2.14686 2.87128 2.88126 5.28066 4.04964 541 a - - -
2.68665 4.42272 2.77439 2.73170 3.46400 2.40529 3.72541
3.29330 2.67787 2.69290 4.24552 2.90393 2.73786 3.18141 2.89847
2.37794 2.77566 2.98404 4.58523 3.61550
0.71257 0.68692 5.03723 1.44153 0.26993 0.58879 0.80968
433 2.72533 4.90533 3.12120 2.57149 2.98783 3.55045 3.75177
3.55296 2.20612 3.15987 3.97963 2.20955 3.94261 2.17188 2.98361
2.76353 2.95603 2.60808 5.40157 4.07055 548 q - - -
2.68618 4.42225 2.77519 2.73123 3.46354 2.40513 3.72494
3.29354 2.67741 2.69355 4.24690 2.90347 2.73739 3.18146 2.89801
2.37887 2.77519 2.98518 4.58477 3.61503
0.01635 4.51768 5.24003 0.61958 0.77255 0.52614 0.89375
434 3.90608 5.13055 5.18106 4.84554 1.45986 4.76720 3.92760
3.51129 4.66372 2.94109 4.16632 4.52693 5.06892 4.58445 4.63529
4.12439 4.12769 2.42123 4.06321 0.87444 549 y - - -
2.68618 4.42225 2.77519 2.73123 3.46354 2.40513 3.72494
3.29354 2.67741 2.69355 4.24690 2.90347 2.73739 3.18146 2.89801
2.37887 2.77519 2.98518 4.58477 3.61503
0.01472 4.62188 5.34423 0.61958 0.77255 0.64581 0.74283
435 2.75478 4.91718 3.17517 2.30353 4.14893 3.58892 2.85706
3.55653 1.94398 2.30477 3.99116 3.12896 3.97790 2.94601 3.01980
2.79868 2.53746 2.63423 5.42181 4.09907 550 k - - -
2.68618 4.42225 2.77519 2.73123 3.46354 2.40513 3.72494
3.29354 2.67741 2.69355 4.24690 2.90347 2.73739 3.18146 2.89801
2.37887 2.77519 2.98518 4.58477 3.61503

```
         0.01472  4.62188  5.34423  0.61958  0.77255  0.64581  0.74283
    436  3.26059  5.83353  1.01971  2.44175  5.21529  3.54378  4.12483
4.76475  3.16072  4.23937  5.07898  1.76802  4.17939  3.28928  3.77531
2.39249  3.56695  4.29457  6.38514  4.85763    551 d - - -
         2.68618  4.42225  2.77519  2.73123  3.46354  2.40513  3.72494
3.29354  2.67741  2.69355  4.24690  2.90347  2.73739  3.18146  2.89801
2.37887  2.77519  2.98518  4.58477  3.61503
         0.31916  4.62188  1.33406  0.61958  0.77255  0.64581  0.74283
    437  2.79319  4.96642  1.75243  2.49869  4.24299  2.42515  3.84751
3.64773  2.73983  2.05391  4.14319  3.02589  3.95130  3.02713  3.21823
2.82026  3.06481  3.32798  5.54829  4.19137    552 d - - -
         2.68618  4.42225  2.77519  2.73123  3.46354  2.40513  3.72494
3.29354  2.67741  2.69355  4.24690  2.90347  2.73739  3.18146  2.89801
2.37887  2.77519  2.98518  4.58477  3.61503
         0.01990  4.32262  5.04497  0.61958  0.77255  0.74374  0.64500
    438  2.26015  5.05902  2.44633  2.43558  4.35827  3.47426  3.68963
3.80605  2.45848  3.35093  4.12796  2.51464  3.87926  2.35483  2.94104
2.68734  2.41188  2.79824  5.53788  4.16157    553 a - - -
         2.68618  4.42225  2.77519  2.73123  3.46354  2.40513  3.72494
3.29354  2.67741  2.69355  4.24690  2.90347  2.73739  3.18146  2.89801
2.37887  2.77519  2.98518  4.58477  3.61503
         0.14626  4.44108  2.08520  0.61958  0.77255  0.46832  0.98365
    439  2.74450  5.11056  2.40790  2.46455  4.44114  2.34319  3.74738
3.89473  2.52872  3.43492  4.21258  3.00261  3.91915  2.35063  3.01522
2.01337  2.98930  2.80862  5.61770  4.23598    554 s - - -
         2.68629  4.42236  2.77530  2.73134  3.46364  2.40493  3.72505
3.29365  2.67751  2.69366  4.24700  2.90309  2.73750  3.18157  2.89811
2.37897  2.77530  2.98529  4.58488  3.61313
         0.25863  1.50240  5.24003  0.67718  0.70938  0.79672  0.59930
    440  2.29146  5.03376  2.48926  1.94274  4.30710  3.50948  3.71997
3.74484  2.49926  3.30962  4.09858  3.01371  3.90986  2.84883  2.97781
2.37165  2.95105  2.77051  5.51530  3.00948    557 e - - -
         2.68618  4.42225  2.77519  2.73123  3.46354  2.40513  3.72494
3.29354  2.67741  2.69355  4.24690  2.90347  2.73739  3.18146  2.89801
2.37887  2.77519  2.98518  4.58477  3.61503
         0.01635  4.51768  5.24003  0.61958  0.77255  0.79672  0.59930
    441  2.70499  4.21625  4.04770  3.46656  2.56986  3.78606  4.07599
2.66338  3.36404  2.40689  2.67859  2.85576  4.15743  3.60288  3.58254
3.06512  2.12928  2.09454  4.81353  2.72736    558 v - - -
         2.68618  4.42225  2.77519  2.73123  3.46354  2.40513  3.72494
3.29354  2.67741  2.69355  4.24690  2.90347  2.73739  3.18146  2.89801
2.37887  2.77519  2.98518  4.58477  3.61503
         0.01635  4.51768  5.24003  0.61958  0.77255  0.54821  0.86271
    442  2.22823  4.35278  3.96549  3.41222  3.55879  2.73457  4.17248
2.00036  3.33843  2.61700  3.54003  3.70107  2.34007  3.60639  3.61382
3.04668  3.01011  1.92345  5.05092  3.84265    559 v - - -
         2.68618  4.42225  2.77519  2.73123  3.46354  2.40513  3.72494
3.29354  2.67741  2.69355  4.24690  2.90347  2.73739  3.18146  2.89801
2.37887  2.77519  2.98518  4.58477  3.61503
         0.01485  4.61273  5.33507  0.61958  0.77255  0.66070  0.72668
    443  2.80475  5.08091  3.07662  2.57350  4.30974  3.57429  3.79175
3.80702  2.54819  3.37044  4.17312  1.74213  3.98784  2.07066  2.99257
2.34024  3.04155  3.45387  3.33824  4.16681    560 n - - -
```

2.68618 4.42225 2.77519 2.73123 3.46354 2.40513 3.72494
3.29354 2.67741 2.69355 4.24690 2.90347 2.73739 3.18146 2.89801
2.37887 2.77519 2.98518 4.58477 3.61503
0.01485 4.61273 5.33507 0.61958 0.77255 0.66070 0.72668
444 2.06004 4.46960 3.85950 3.39225 2.63032 1.28697 4.31783
3.37599 3.36674 3.10490 3.98811 3.63762 4.09407 3.65008 3.68821
2.85774 2.35036 3.05481 5.44839 4.22068 561 g - - -
2.68573 4.42258 2.77515 2.73156 3.46387 2.40431 3.72527
3.29387 2.67773 2.69388 4.24722 2.90379 2.73730 3.18179 2.89792
2.37867 2.77471 2.98551 4.58510 3.61536
0.77093 1.27007 1.36022 1.36379 0.29530 0.66070 0.72668
445 2.80230 4.42239 3.79000 3.26397 3.11572 3.76612 3.92076
2.25938 3.11519 2.55958 3.53078 2.52636 4.16191 3.45183 3.38879
3.06168 3.04471 2.66479 1.83626 3.23495 567 w - - -
2.68618 4.42225 2.77519 2.73123 3.46354 2.40513 3.72494
3.29354 2.67741 2.69355 4.24690 2.90347 2.73739 3.18146 2.89801
2.37887 2.77519 2.98518 4.58477 3.61503
0.01990 4.32266 5.04501 0.61958 0.77255 1.00049 0.45839
446 2.76995 5.10318 2.15259 2.39380 4.41116 2.08559 3.75959
3.86436 2.60029 2.59971 4.23150 2.31013 3.90202 2.90309 3.09749
2.75509 3.02632 3.49111 5.62642 4.23786 568 g - - -
2.68618 4.42225 2.77519 2.73123 3.46354 2.40513 3.72494
3.29354 2.67741 2.69355 4.24690 2.90347 2.73739 3.18146 2.89801
2.37887 2.77519 2.98518 4.58477 3.61503
0.01990 4.32266 5.04501 0.61958 0.77255 1.00049 0.45839
447 2.11128 2.89429 3.50424 2.97129 3.74928 3.45462 2.11360
3.11472 2.90970 2.82316 3.70089 3.34326 3.95066 3.23992 3.26916
2.76036 2.26278 2.83728 5.16146 3.91618 569 a - - -
2.68618 4.42225 2.77519 2.73123 3.46354 2.40513 3.72494
3.29354 2.67741 2.69355 4.24690 2.90347 2.73739 3.18146 2.89801
2.37887 2.77519 2.98518 4.58477 3.61503
0.01990 4.32266 5.04501 0.61958 0.77255 1.00049 0.45839
448 1.95265 4.58386 3.25076 2.81671 4.35489 3.26710 4.00164
3.77968 2.85973 3.40340 4.21026 2.30960 3.86946 3.18406 3.28284
1.65101 2.22098 3.33022 5.63264 4.33937 570 s - - -
2.68618 4.42225 2.77519 2.73123 3.46354 2.40513 3.72494
3.29354 2.67741 2.69355 4.24690 2.90347 2.73739 3.18146 2.89801
2.37887 2.77519 2.98518 4.58477 3.61503
0.01990 4.32266 5.04501 0.61958 0.77255 1.00049 0.45839
449 3.49211 4.76813 5.26541 4.69766 2.15605 4.77920 4.98867
1.78807 4.55739 0.91513 2.98408 4.86006 4.92798 4.55133 4.58257
4.13569 3.70656 2.44218 5.17240 4.01779 571 l - - -
2.68628 4.42235 2.77529 2.73133 3.46364 2.40522 3.72504
3.29364 2.67750 2.69365 4.24522 2.90356 2.73712 3.18156 2.89769
2.37896 2.77529 2.98528 4.58487 3.61415
0.97074 0.72723 1.98083 0.11042 2.25818 0.60125 0.79436
450 2.96079 4.92639 3.51592 3.19487 4.72088 0.85034 4.27588
4.22270 2.91200 3.79413 4.66471 3.55580 4.17835 3.49070 2.05611
3.06920 3.34374 3.77943 5.84110 4.66357 573 g - - -
2.68618 4.42225 2.77519 2.73123 3.46354 2.40513 3.72494
3.29354 2.67741 2.69355 4.24690 2.90347 2.73739 3.18146 2.89801
2.37887 2.77519 2.98518 4.58477 3.61503
0.14472 4.39893 2.10006 0.61958 0.77255 0.93023 0.50164

```
    451   2.67548  4.74728  3.05733  2.58583  3.92796  2.60957  3.75198
3.41336  2.59024  3.04399  3.88882  3.07911  3.90869  2.40480  3.01623
2.29790  2.92572  3.11180  5.26365  2.32066    574 s - - -
          2.68618  4.42225  2.77519  2.73123  3.46354  2.40513  3.72494
3.29354  2.67741  2.69355  4.24690  2.90347  2.73739  3.18146  2.89801
2.37887  2.77519  2.98518  4.58477  3.61503
          0.02088  4.27509  4.99743  0.61958  0.77255  1.03949  0.43639
    452   2.54085  2.21106  3.36249  2.85777  4.00059  2.52114  3.92952
3.39041  2.21676  3.05676  3.90522  3.24773  3.89293  3.15456  3.16870
2.10313  2.87317  3.05205  5.34591  4.08481    575 s - - -
          2.68618  4.42225  2.77519  2.73123  3.46354  2.40513  3.72494
3.29354  2.67741  2.69355  4.24690  2.90347  2.73739  3.18146  2.89801
2.37887  2.77519  2.98518  4.58477  3.61503
          0.02088  4.27509  4.99743  0.61958  0.77255  1.03949  0.43639
    453   2.51035  4.28932  3.79959  3.33376  3.83594  3.36738  4.22234
2.93477  3.27141  2.86033  3.78646  3.55289  3.97791  3.56994  3.56805
2.13934  1.75922  1.66118  5.30497  4.09049    576 v - - -
          2.68618  4.42225  2.77519  2.73123  3.46354  2.40513  3.72494
3.29354  2.67741  2.69355  4.24690  2.90347  2.73739  3.18146  2.89801
2.37887  2.77519  2.98518  4.58477  3.61503
          0.02088  4.27509  4.99743  0.61958  0.77255  1.03949  0.43639
    454   2.36606  2.52775  3.97040  3.53990  4.08909  3.15998  4.37993
3.41562  3.45827  3.17896  4.04704  3.58996  3.87753  3.73222  3.71820
1.88513  1.31584  3.00738  5.51195  4.32424    577 t - - -
          2.68618  4.42225  2.77519  2.73123  3.46354  2.40513  3.72494
3.29354  2.67741  2.69355  4.24690  2.90347  2.73739  3.18146  2.89801
2.37887  2.77519  2.98518  4.58477  3.61503
          0.02088  4.27509  4.99743  0.61958  0.77255  0.78277  0.61090
    455   2.67730  4.63728  3.23125  2.87310  4.09613  1.39673  4.03586
2.53814  2.93722  3.17540  4.05073  2.43623  3.99372  3.27278  3.32552
2.80726  3.01522  3.12761  5.46664  4.17453    578 g - - -
          2.68618  4.42225  2.77519  2.73123  3.46354  2.40513  3.72494
3.29354  2.67741  2.69355  4.24690  2.90347  2.73739  3.18146  2.89801
2.37887  2.77519  2.98518  4.58477  3.61503
          0.13988  4.39893  2.13498  0.61958  0.77255  0.93023  0.50164
    456   3.14462  0.39050  4.72412  4.56998  4.49620  3.66359  5.13092
3.73245  4.40986  3.61704  4.75765  4.45458  4.39863  4.73908  4.45435
3.41431  3.64628  3.44289  5.79867  4.77508    579 C - - -
          2.68618  4.42225  2.77519  2.73123  3.46354  2.40513  3.72494
3.29354  2.67741  2.69355  4.24690  2.90347  2.73739  3.18146  2.89801
2.37887  2.77519  2.98518  4.58477  3.61503
          0.02078  4.27983  5.00218  0.61958  0.77255  1.03575  0.43844
    457   2.69138  4.93500  2.37793  2.44147  2.93433  3.43023  3.71715
3.65104  2.54883  3.23986  4.05331  2.35653  3.87498  2.87216  3.02722
1.94281  2.94043  3.30656  5.44155  4.06999    580 s - - -
          2.68618  4.42225  2.77519  2.73123  3.46354  2.40513  3.72494
3.29354  2.67741  2.69355  4.24690  2.90347  2.73739  3.18146  2.89801
2.37887  2.77519  2.98518  4.58477  3.61503
          0.02078  4.27983  5.00218  0.61958  0.77255  0.78723  0.60716
    458   1.78542  4.49642  3.45590  2.96885  3.98118  2.44703  4.02706
3.34487  2.95409  2.30951  3.90493  3.34126  2.19497  3.27687  3.33626
2.73956  2.92036  3.02367  5.36312  4.10876    581 a - - -
          2.68665  4.41883  2.77566  2.73133  3.46240  2.40509  3.72541
3.29343  2.67787  2.69402  4.24736  2.90312  2.73749  3.18136  2.89806
2.37855  2.77490  2.98565  4.58312  3.61465
```

```
          1.04701  0.61031  2.24589  1.29966  0.31831  0.93023  0.50164
    459   2.75607  4.43023  3.64176  3.10893  2.37026  3.71143  3.83380
2.93487  3.05674  2.61991  3.54967  2.60581  4.09862  3.34352  3.38176
2.97934  2.35984  2.71976  4.61695  2.02745    591 y - - -
          2.68618  4.42225  2.77519  2.73123  3.46354  2.40513  3.72494
3.29354  2.67741  2.69355  4.24690  2.90347  2.73739  3.18146  2.89801
2.37887  2.77519  2.98518  4.58477  3.61503
          0.02049  4.29372  5.01607  0.61958  0.77255  0.58178  0.81849
    460   2.27340  4.26580  4.03625  3.46779  3.41053  3.77904  4.13652
2.16890  3.37121  1.63779  3.39907  3.73112  4.17809  3.62460  3.60903
2.49835  2.38135  2.44540  4.92209  3.72205    592 l - - -
          2.68618  4.42225  2.77519  2.73123  3.46354  2.40513  3.72494
3.29354  2.67741  2.69355  4.24690  2.90347  2.73739  3.18146  2.89801
2.37887  2.77519  2.98518  4.58477  3.61503
          0.13341  4.51391  2.17209  0.61958  0.77255  0.80151  0.59538
    461   2.69141  5.08999  2.95447  2.42246  4.42170  2.69951  3.67169
3.87919  2.41078  3.39976  4.16636  2.47323  3.86754  2.34024  2.45968
2.30604  2.46821  3.48037  5.56380  4.18337    593 s - - -
          2.68618  4.42225  2.77519  2.73123  3.46354  2.40513  3.72494
3.29354  2.67741  2.69355  4.24690  2.90347  2.73739  3.18146  2.89801
2.37887  2.77519  2.98518  4.58477  3.61503
          0.01843  4.39893  5.12128  0.61958  0.77255  0.93023  0.50164
    462   2.63859  4.82079  3.13596  2.65214  4.40614  2.07131  3.85879
3.84567  2.62542  3.41909  4.21291  3.12125  2.80339  3.00568  2.35636
1.86616  2.95125  3.42572  5.61486  4.28751    594 s - - -
          2.68618  4.42225  2.77519  2.73123  3.46354  2.40513  3.72494
3.29354  2.67741  2.69355  4.24690  2.90347  2.73739  3.18146  2.89801
2.37887  2.77519  2.98518  4.58477  3.61503
          0.01843  4.39893  5.12128  0.61958  0.77255  0.93023  0.50164
    463   2.78502  5.10483  2.27117  2.43157  4.30607  3.47017  3.75805
3.82763  2.59292  3.38972  4.19660  2.42315  2.30786  2.90737  3.08354
2.77383  3.03030  3.46777  5.55352  2.36931    595 d - - -
          2.68618  4.42225  2.77519  2.73123  3.46354  2.40513  3.72494
3.29354  2.67741  2.69355  4.24690  2.90347  2.73739  3.18146  2.89801
2.37887  2.77519  2.98518  4.58477  3.61503
          0.01843  4.39893  5.12128  0.61958  0.77255  0.93023  0.50164
    464   2.84261  5.19842  2.97141  2.50772  4.52754  1.89941  2.67511
3.97249  2.00875  3.48003  4.27427  3.03166  3.96042  2.35912  2.75669
2.81981  3.06868  3.59341  5.62004  4.27455    596 g - - -
          2.68618  4.42225  2.77519  2.73123  3.46354  2.40513  3.72494
3.29354  2.67741  2.69355  4.24690  2.90347  2.73739  3.18146  2.89801
2.37887  2.77519  2.98518  4.58477  3.61503
          0.01843  4.39893  5.12128  0.61958  0.77255  0.93023  0.50164
    465   2.25865  4.99939  2.93168  2.11905  4.36776  3.44125  3.74213
3.81164  2.53326  3.37051  4.15837  2.46505  3.88865  2.87424  3.01108
2.22625  2.07936  3.42759  5.56774  4.20056    597 t - - -
          2.68618  4.42225  2.77519  2.73123  3.46354  2.40513  3.72494
3.29354  2.67741  2.69355  4.24690  2.90347  2.73739  3.18146  2.89801
2.37887  2.77519  2.98518  4.58477  3.61503
          0.01843  4.39893  5.12128  0.61958  0.77255  0.66312  0.72410
    466   2.81520  5.23842  2.89952  2.06274  4.58301  3.51442  3.75653
4.04636  2.48838  3.55046  4.32307  2.41208  3.94982  2.87360  2.39468
1.60934  3.05414  3.63892  5.69923  4.31290    598 s - - -
```

```
          2.68618  4.42225  2.77519  2.73123  3.46354  2.40513  3.72494
3.29354  2.67741  2.69355  4.24690  2.90347  2.73739  3.18146  2.89801
2.37887  2.77519  2.98518  4.58477  3.61503
          0.01641  4.51391  5.23626  0.61958  0.77255  0.80151  0.59538
    467   2.71087  2.59902  3.28360  2.73242  3.91851  3.58007  3.83367
3.30322  2.31794  2.44206  3.81850  2.21931  3.98507  3.04197  3.09597
2.31975  2.95284  3.02577  5.26648  3.98222    599 n - - -
          2.68618  4.42225  2.77519  2.73123  3.46354  2.40513  3.72494
3.29354  2.67741  2.69355  4.24690  2.90347  2.73739  3.18146  2.89801
2.37887  2.77519  2.98518  4.58477  3.61503
          0.01641  4.51391  5.23626  0.61958  0.77255  0.80151  0.59538
    468   2.86749  5.28036  2.16290  2.44633  4.69454  3.46842  3.86425
4.17381  2.70962  3.69085  4.47414  1.53955  3.98092  2.99665  3.22419
2.25648  2.39792  3.74388  5.85503  4.43736    600 n - - -
          2.68618  4.42225  2.77519  2.73123  3.46354  2.40513  3.72494
3.29354  2.67741  2.69355  4.24690  2.90347  2.73739  3.18146  2.89801
2.37887  2.77519  2.98518  4.58477  3.61503
          0.01641  4.51391  5.23626  0.61958  0.77255  0.54473  0.86750
    469   2.10381  5.12049  3.03888  2.21603  4.41793  2.77611  3.72685
3.86882  2.47889  2.73613  4.17690  3.02443  3.03031  2.45637  2.48100
2.73133  2.97352  3.48585  5.58406  4.20777    601 a - - -
          2.68618  4.42225  2.77519  2.73123  3.46354  2.40513  3.72494
3.29354  2.67741  2.69355  4.24690  2.90347  2.73739  3.18146  2.89801
2.37887  2.77519  2.98518  4.58477  3.61503
          0.01485  4.61273  5.33507  0.61958  0.77255  0.66070  0.72668
    470   1.97539  4.86555  3.19221  2.68417  4.23079  2.71082  3.87829
3.65281  2.70353  3.26681  4.08461  2.52199  3.98435  3.04294  3.15688
1.82351  3.00038  3.30851  5.51260  2.92467    602 s - - -
          2.68618  4.42225  2.77519  2.73123  3.46354  2.40513  3.72494
3.29354  2.67741  2.69355  4.24690  2.90347  2.73739  3.18146  2.89801
2.37887  2.77519  2.98518  4.58477  3.61503
          0.01485  4.61273  5.33507  0.61958  0.77255  0.39012  1.13004
    471   2.77227  4.43703  2.91210  3.17595  3.56293  3.77590  4.03787
2.40745  3.11894  1.80692  2.78458  3.55382  2.97073  3.41002  2.81849
3.02825  3.00401  2.70366  5.02541  2.87656    603 l - - -
          2.68618  4.42225  2.77519  2.73123  3.46354  2.40513  3.72494
3.29354  2.67741  2.69355  4.24690  2.90347  2.73739  3.18146  2.89801
2.37887  2.77519  2.98518  4.58477  3.61503
          0.01350  4.70791  5.43026  0.61958  0.77255  0.48576  0.95510
    472   2.37176  5.27204  2.45067  2.13550  4.61209  2.78789  3.80283
4.08396  2.57642  3.59170  4.35245  3.04286  2.24780  2.91522  3.07294
2.79831  2.17485  3.66801  5.74808  4.34543    604 e - - -
          2.68618  4.42225  2.77519  2.73123  3.46354  2.40513  3.72494
3.29354  2.67741  2.69355  4.24690  2.90347  2.73739  3.18146  2.89801
2.37887  2.77519  2.98518  4.58477  3.61503
          0.01350  4.70791  5.43026  0.61958  0.77255  0.48576  0.95510
    473   3.31975  5.87680  1.68617  2.50999  5.29449  1.65546  4.19806
4.84357  3.24138  4.31724  5.15819  1.35021  4.24183  3.36411  3.85824
3.21262  3.63234  4.36567  6.46156  4.93730    605 n - - -
          2.68618  4.42225  2.77519  2.73123  3.46354  2.40513  3.72494
3.29354  2.67741  2.69355  4.24690  2.90347  2.73739  3.18146  2.89801
2.37887  2.77519  2.98518  4.58477  3.61503
          0.01350  4.70791  5.43026  0.61958  0.77255  0.48576  0.95510
```

```
    474   2.33869  5.20585  3.05380  2.60115  4.55636  2.75295  2.32130
4.01438  2.60198  3.55122  4.33995  3.12845  4.03900  1.52343  3.04657
2.86860  3.12323  3.62754  5.72037  4.35355    606 q - - -
          2.68618  4.42225  2.77519  2.73123  3.46354  2.40513  3.72494
3.29354  2.67741  2.69355  4.24690  2.90347  2.73739  3.18146  2.89801
2.37887  2.77519  2.98518  4.58477  3.61503
          0.01350  4.70791  5.43026  0.61958  0.77255  0.48576  0.95510
    475   2.81195  4.97818  2.57404  2.65686  3.05079  3.63288  3.83880
2.79790  1.37191  3.20797  4.04746  3.17123  4.02855  3.00025  3.08104
2.85352  3.04370  3.27728  5.46421  4.12393    607 k - - -
          2.68618  4.42225  2.77519  2.73123  3.46354  2.40513  3.72494
3.29354  2.67741  2.69355  4.24690  2.90347  2.73739  3.18146  2.89801
2.37887  2.77519  2.98518  4.58477  3.61503
          0.01350  4.70791  5.43026  0.61958  0.77255  0.48576  0.95510
    476   2.49836  4.71588  5.25064  4.71944  3.73350  4.74087  5.23078
0.93868  4.58270  1.54906  3.55414  4.86516  5.03006  4.76230  4.69673
4.10899  3.66195  2.07453  5.65031  4.50175    608 i - - -
          2.68618  4.42225  2.77519  2.73123  3.46354  2.40513  3.72494
3.29354  2.67741  2.69355  4.24690  2.90347  2.73739  3.18146  2.89801
2.37887  2.77519  2.98518  4.58477  3.61503
          0.01350  4.70791  5.43026  0.61958  0.77255  0.48576  0.95510
    477   3.05506  5.59567  2.22525  2.47848  4.89902  3.58964  2.28575
4.41088  2.78146  3.88766  4.66819  1.37142  4.09710  2.40286  3.30207
2.98149  3.30663  3.97692  6.02048  4.56982    609 n - - -
          2.68618  4.42225  2.77519  2.73123  3.46354  2.40513  3.72494
3.29354  2.67741  2.69355  4.24690  2.90347  2.73739  3.18146  2.89801
2.37887  2.77519  2.98518  4.58477  3.61503
          0.01350  4.70791  5.43026  0.61958  0.77255  0.48576  0.95510
    478   1.60254  4.29097  4.30511  3.72411  3.41542  3.92962  4.26108
2.17590  3.60433  2.47193  3.41537  3.92706  4.30534  3.82718  3.78540
3.22354  2.48682  1.88387  4.94137  2.76670    610 a - - -
          2.68618  4.42225  2.77519  2.73123  3.46354  2.40513  3.72494
3.29354  2.67741  2.69355  4.24690  2.90347  2.73739  3.18146  2.89801
2.37887  2.77519  2.98518  4.58477  3.61503
          0.01350  4.70791  5.43026  0.61958  0.77255  0.48576  0.95510
    479   3.68218  4.89038  5.70571  5.19452  3.80156  5.21197  5.75452
0.94505  5.10033  1.35993  3.54860  5.36042  5.37158  5.20048  5.17927
4.62231  3.92420  1.67790  5.94612  4.85031    611 i - - -
          2.68618  4.42225  2.77519  2.73123  3.46354  2.40513  3.72494
3.29354  2.67741  2.69355  4.24690  2.90347  2.73739  3.18146  2.89801
2.37887  2.77519  2.98518  4.58477  3.61503
          0.01350  4.70791  5.43026  0.61958  0.77255  0.48576  0.95510
    480   3.49830  5.14070  4.26834  4.04287  2.82265  4.17617  4.13136
3.99146  3.99671  3.46877  4.58325  4.14529  4.74034  4.24678  4.19171
2.33675  3.86154  3.75502  4.46825  0.58572    612 Y - - -
          2.68618  4.42225  2.77519  2.73123  3.46354  2.40513  3.72494
3.29354  2.67741  2.69355  4.24690  2.90347  2.73739  3.18146  2.89801
2.37887  2.77519  2.98518  4.58477  3.61503
          0.21526  4.70791  1.68934  0.61958  0.77255  0.48576  0.95510
    481   2.24175  4.98558  2.97950  2.10760  4.48399  3.45008  3.88331
3.92724  2.70074  3.49737  4.29283  3.09344  1.94114  3.02808  3.17166
1.91748  3.03799  3.51948  5.69820  4.34042    613 s - - -
          2.68618  4.42225  2.77519  2.73123  3.46354  2.40513  3.72494
3.29354  2.67741  2.69355  4.24690  2.90347  2.73739  3.18146  2.89801
2.37887  2.77519  2.98518  4.58477  3.61503
```

```
          0.01649  4.50914  5.23149  0.61958  0.77255  0.80751  0.59053
    482   2.30415  4.31976  4.77785  4.19215  2.12270  4.17868  4.53352
1.94855  4.03263  1.84303  3.30523  4.28278  4.50945  4.18969  4.11101
3.49818  3.19442  1.57436  5.03253  3.86022    614 v - - -
          2.68618  4.42225  2.77519  2.73123  3.46354  2.40513  3.72494
3.29354  2.67741  2.69355  4.24690  2.90347  2.73739  3.18146  2.89801
2.37887  2.77519  2.98518  4.58477  3.61503
          0.01649  4.50914  5.23149  0.61958  0.77255  0.54040  0.87350
    483   2.75371  4.65932  3.39925  2.87174  2.82706  3.63257  3.93412
3.25414  2.83921  2.92589  3.80451  3.32283  2.92071  2.60219  3.23676
2.89217  1.48940  2.99303  5.24724  3.96556    615 t - - -
          2.68618  4.42225  2.77519  2.73123  3.46354  2.40513  3.72494
3.29354  2.67741  2.69355  4.24690  2.90347  2.73739  3.18146  2.89801
2.37887  2.77519  2.98518  4.58477  3.61503
          0.01485  4.61273  5.33507  0.61958  0.77255  0.66070  0.72668
    484   2.04782  5.04419  3.06549  2.24059  4.32281  3.54977  3.76596
3.75431  2.53999  2.66933  4.12173  3.06609  3.95319  2.16923  3.00737
2.06842  2.99193  3.40135  5.53842  4.18294    616 a - - -
          2.68618  4.42225  2.77519  2.73123  3.46354  2.40513  3.72494
3.29354  2.67741  2.69355  4.24690  2.90347  2.73739  3.18146  2.89801
2.37887  2.77519  2.98518  4.58477  3.61503
          0.01485  4.61273  5.33507  0.61958  0.77255  0.39012  1.13004
    485   2.75765  4.90101  3.29564  2.87057  4.60747  3.49143  4.10696
4.05176  2.93555  3.64000  4.43360  2.06968  2.17881  3.26592  3.38145
2.24632  1.55385  3.59985  5.84405  4.51958    617 t - - -
          2.68618  4.42225  2.77519  2.73123  3.46354  2.40513  3.72494
3.29354  2.67741  2.69355  4.24690  2.90347  2.73739  3.18146  2.89801
2.37887  2.77519  2.98518  4.58477  3.61503
          0.01350  4.70791  5.43026  0.61958  0.77255  0.48576  0.95510
    486   2.86638  5.28737  2.46865  2.50587  4.59987  2.28457  2.87685
4.06851  2.62419  3.59077  4.36476  1.68699  4.00841  2.95358  3.12131
2.83751  3.10846  2.90087  5.75881  4.35854    618 n - - -
          2.68618  4.42225  2.77519  2.73123  3.46354  2.40513  3.72494
3.29354  2.67741  2.69355  4.24690  2.90347  2.73739  3.18146  2.89801
2.37887  2.77519  2.98518  4.58477  3.61503
          0.01350  4.70791  5.43026  0.61958  0.77255  0.48576  0.95510
    487   2.76839  4.99916  2.24391  2.59117  4.24556  3.59141  3.79448
3.66743  2.28637  3.26131  2.99975  2.66477  3.00739  2.93826  3.05421
2.79424  2.99982  2.40549  5.49565  3.09258    619 d - - -
          2.68618  4.42225  2.77519  2.73123  3.46354  2.40513  3.72494
3.29354  2.67741  2.69355  4.24690  2.90347  2.73739  3.18146  2.89801
2.37887  2.77519  2.98518  4.58477  3.61503
          0.39456  4.70791  1.14886  0.61958  0.77255  0.48576  0.95510
    488   2.64651  4.66121  3.18344  2.68515  3.96473  3.47101  3.82212
3.34137  2.67011  2.38106  3.86177  2.43819  2.27671  3.02377  3.08652
2.74593  2.29466  3.04380  5.30414  4.01130    620 p - - -
          2.68618  4.42225  2.77519  2.73123  3.46354  2.40513  3.72494
3.29354  2.67741  2.69355  4.24690  2.90347  2.73739  3.18146  2.89801
2.37887  2.77519  2.98518  4.58477  3.61503
          0.01970  4.33305  5.05539  0.61958  0.77255  0.72093  0.66612
    489   2.73614  4.30779  3.88259  2.64232  3.44082  3.79099  4.08210
1.84213  3.23803  2.06155  3.41504  3.64294  4.16429  3.51395  3.51957
2.45329  2.97070  2.03028  4.94882  3.73756    621 i - - -
```

```
        2.68618  4.42225  2.77519  2.73123  3.46354  2.40513  3.72494
3.29354  2.67741  2.69355  4.24690  2.90347  2.73739  3.18146  2.89801
2.37887  2.77519  2.98518  4.58477  3.61503
        0.11816  4.45711  2.30407  0.61958  0.77255  0.86902  0.54363
    490  2.69115  4.49755  3.44612  2.88345  3.59602  3.61657  3.84650
3.02156  2.76631  2.71494  3.60700  3.31026  2.82701  3.13896  2.44146
2.86749  2.92608  2.25136  5.02183  2.26075    622 v - - -
        2.68618  4.42225  2.77519  2.73123  3.46354  2.40513  3.72494
3.29354  2.67741  2.69355  4.24690  2.90347  2.73739  3.18146  2.89801
2.37887  2.77519  2.98518  4.58477  3.61503
        0.01920  4.35815  5.08050  0.61958  0.77255  0.74519  0.64368
    491  2.74064  5.03996  2.02854  2.47857  4.31842  3.50063  3.73551
3.73803  2.17836  2.62277  4.11843  3.01159  3.91882  2.87027  2.98248
2.74366  2.06888  3.38603  5.53132  4.16996    623 d - - -
        2.68618  4.42225  2.77519  2.73123  3.46354  2.40513  3.72494
3.29354  2.67741  2.69355  4.24690  2.90347  2.73739  3.18146  2.89801
2.37887  2.77519  2.98518  4.58477  3.61503
        0.01738  4.45711  5.17946  0.61958  0.77255  0.86902  0.54363
    492  3.28945  4.68843  4.53085  4.04539  1.88039  4.25465  1.94946
2.19809  3.91429  2.75154  3.80256  4.07180  4.59405  4.03663  4.05047
3.56320  3.51482  3.00737  4.11390  1.54664    624 y - - -
        2.68636  4.42243  2.77537  2.73141  3.46371  2.40502  3.72512
3.29372  2.67758  2.69372  4.24707  2.90364  2.73718  3.18103  2.89818
2.37877  2.77456  2.98536  4.58495  3.61426
        0.62499  2.14107  1.05784  1.61947  0.22065  0.30419  1.33836
    493  2.31692  4.90810  2.50107  2.49967  4.14699  3.49746  3.70402
3.57210  1.94048  2.61967  3.97905  3.02202  3.89318  2.85076  2.95262
2.71042  2.91610  3.24302  5.39990  2.94407    632 k - - -
        2.68618  4.42225  2.77519  2.73123  3.46354  2.40513  3.72494
3.29354  2.67741  2.69355  4.24690  2.90347  2.73739  3.18146  2.89801
2.37887  2.77519  2.98518  4.58477  3.61503
        0.01843  4.39893  5.12128  0.61958  0.77255  0.65965  0.72781
    494  2.90526  5.32223  1.85430  2.42575  4.58702  1.88951  3.84633
4.09764  2.70558  3.62774  4.42554  2.34889  3.99344  2.98712  3.22307
2.86328  3.16004  3.70395  5.78679  2.82414    633 d - - -
        2.68618  4.42225  2.77519  2.73123  3.46354  2.40513  3.72494
3.29354  2.67741  2.69355  4.24690  2.90347  2.73739  3.18146  2.89801
2.37887  2.77519  2.98518  4.58477  3.61503
        0.01638  4.51552  5.23787  0.61958  0.77255  0.79947  0.59705
    495  2.72636  4.98768  3.04247  2.51968  4.25343  3.52102  3.74253
3.69028  2.52415  3.27010  4.07133  2.12248  3.92678  2.44810  2.98846
2.34263  2.20402  3.34460  5.48512  3.00029    634 n - - -
        2.68618  4.42225  2.77519  2.73123  3.46354  2.40513  3.72494
3.29354  2.67741  2.69355  4.24690  2.90347  2.73739  3.18146  2.89801
2.37887  2.77519  2.98518  4.58477  3.61503
        0.01638  4.51552  5.23787  0.61958  0.77255  0.79947  0.59705
    496  2.25637  4.48028  3.50035  2.94067  3.64694  2.87027  3.91709
3.02007  2.90264  2.72231  3.60553  3.36267  4.03500  3.21544  3.27712
2.08926  2.44609  2.78091  3.08875  2.92728    635 s - - -
        2.68618  4.42225  2.77519  2.73123  3.46354  2.40513  3.72494
3.29354  2.67741  2.69355  4.24690  2.90347  2.73739  3.18146  2.89801
2.37887  2.77519  2.98518  4.58477  3.61503
        0.01638  4.51552  5.23787  0.61958  0.77255  0.33012  1.26881
```

```
     497   2.37080  5.13854  2.54258  2.53255  4.43611  1.95900  3.78205
 2.92334  2.55076  3.43033  4.20944  2.52411  3.97141  2.90396  2.58446
 2.77958  3.02077  3.50647  5.62015  4.24683    636 g - - -
           2.68618  4.42225  2.77519  2.73123  3.46354  2.40513  3.72494
 3.29354  2.67741  2.69355  4.24690  2.90347  2.73739  3.18146  2.89801
 2.37887  2.77519  2.98518  4.58477  3.61503
           0.01350  4.70791  5.43026  0.61958  0.77255  0.48576  0.95510
     498   2.91133  5.25594  3.17644  2.63558  4.57196  3.66017  2.86333
 4.01754  1.36003  3.53391  4.32307  2.20264  4.05683  2.93723  2.84581
 2.89747  3.13400  2.77137  5.67809  4.33449    637 k - - -
           2.68618  4.42225  2.77519  2.73123  3.46354  2.40513  3.72494
 3.29354  2.67741  2.69355  4.24690  2.90347  2.73739  3.18146  2.89801
 2.37887  2.77519  2.98518  4.58477  3.61503
           0.01350  4.70791  5.43026  0.61958  0.77255  0.48576  0.95510
     499   2.78459  4.24473  4.32844  3.74089  3.34588  2.86690  4.21675
 2.67057  3.60399  1.69844  3.35989  3.91452  4.26903  3.81536  3.75924
 3.18898  2.08713  1.85104  3.24382  3.67393    638 l - - -
           2.68618  4.42225  2.77519  2.73123  3.46354  2.40513  3.72494
 3.29354  2.67741  2.69355  4.24690  2.90347  2.73739  3.18146  2.89801
 2.37887  2.77519  2.98518  4.58477  3.61503
           0.01350  4.70791  5.43026  0.61958  0.77255  0.48576  0.95510
     500   2.17374  4.62034  3.67152  3.21478  4.13251  1.23424  4.24944
 3.53206  3.21293  3.22664  4.09109  3.55248  4.12377  3.52377  3.57892
 2.90074  2.33313  3.19957  5.52951  2.73988    639 g - - -
           2.68588  4.42233  2.77527  2.73131  3.46362  2.40521  3.72502
 3.29362  2.67749  2.69363  4.24697  2.90354  2.73747  3.18154  2.89808
 2.37866  2.77483  2.98526  4.58485  3.61511
           0.31588  1.32249  5.43026  0.13135  2.09486  0.48576  0.95510
     501   3.21118  4.55961  4.96974  4.41296  2.32399  4.35273  4.37288
 2.74079  4.23144  1.40298  3.42076  4.40627  4.67004  4.31611  4.26186
 3.67380  3.43842  1.87686  4.69572  1.71218    641 l - - -
           2.68618  4.42225  2.77519  2.73123  3.46354  2.40513  3.72494
 3.29354  2.67741  2.69355  4.24690  2.90347  2.73739  3.18146  2.89801
 2.37887  2.77519  2.98518  4.58477  3.61503
           0.01350  4.70791  5.43026  0.61958  0.77255  0.48576  0.95510
     502   3.69106  4.92928  5.66050  5.09708  3.53155  5.09138  5.47777
 1.38020  4.96593  1.02003  2.48607  5.24144  5.23347  4.96101  4.97204
 4.46749  3.91434  1.91844  5.64171  4.61059    642 l - - -
           2.68618  4.42225  2.77519  2.73123  3.46354  2.40513  3.72494
 3.29354  2.67741  2.69355  4.24690  2.90347  2.73739  3.18146  2.89801
 2.37887  2.77519  2.98518  4.58477  3.61503
           0.10420  4.70791  2.40875  0.61958  0.77255  0.48576  0.95510
     503   1.28262  4.50508  4.06203  3.77253  4.92405  2.23393  4.79132
 4.38198  3.82635  4.04568  4.82722  3.79021  4.06739  4.06866  4.11506
 1.08895  3.07791  3.69014  6.24276  5.07036    643 s - - -
           2.68618  4.42225  2.77519  2.73123  3.46354  2.40513  3.72494
 3.29354  2.67741  2.69355  4.24690  2.90347  2.73739  3.18146  2.89801
 2.37887  2.77519  2.98518  4.58477  3.61503
           0.12098  4.61848  2.26261  0.61958  0.77255  0.65138  0.73674
     504   2.07179  4.43975  3.81774  3.33425  4.14022  3.37711  4.30110
 3.44248  3.29581  3.20581  4.07251  3.57103  4.01784  3.59125  3.63381
 1.51229  1.75214  2.26970  5.54425  4.32295    644 s - - -
           2.68618  4.42225  2.77519  2.73123  3.46354  2.40513  3.72494
 3.29354  2.67741  2.69355  4.24690  2.90347  2.73739  3.18146  2.89801
 2.37887  2.77519  2.98518  4.58477  3.61503
```

```
          0.01641  4.51391  5.23626  0.61958  0.77255  0.80151  0.59538
    505   2.72584  4.56906  3.41803  2.86427  3.64467  3.64690  2.47026
3.10509  2.82048  2.02733  3.67009  3.31070  2.87735  3.15236  3.20860
2.39245  2.95882  2.86122  5.07006  2.93161    645 l - - -
          2.68618  4.42225  2.77519  2.73123  3.46354  2.40513  3.72494
3.29354  2.67741  2.69355  4.24690  2.90347  2.73739  3.18146  2.89801
2.37887  2.77519  2.98518  4.58477  3.61503
          0.01641  4.51391  5.23626  0.61958  0.77255  0.80151  0.59538
    506   2.79239  4.31269  4.20755  3.65255  3.52648  3.86545  4.31051
1.80294  3.55332  2.51607  3.49238  3.88408  4.28673  3.80495  3.77946
2.43631  1.97145  1.69896  5.07389  3.87271    646 v - - -
          2.68618  4.42225  2.77519  2.73123  3.46354  2.40513  3.72494
3.29354  2.67741  2.69355  4.24690  2.90347  2.73739  3.18146  2.89801
2.37887  2.77519  2.98518  4.58477  3.61503
          0.01641  4.51391  5.23626  0.61958  0.77255  0.54473  0.86750
    507   3.05456  4.77711  4.14110  3.81177  4.07502  3.83733  4.70705
2.13973  3.70578  2.92687  4.09548  4.04274  0.78800  4.08065  3.95369
3.31584  3.44399  2.91145  5.67886  4.42875    647 p - - -
          2.68618  4.42225  2.77519  2.73123  3.46354  2.40513  3.72494
3.29354  2.67741  2.69355  4.24690  2.90347  2.73739  3.18146  2.89801
2.37887  2.77519  2.98518  4.58477  3.61503
          0.01485  4.61273  5.33507  0.61958  0.77255  0.66070  0.72668
    508   2.74661  4.35855  3.82346  3.25062  3.45684  3.77349  4.03813
2.34840  3.17400  2.16556  2.79592  2.72907  4.14816  2.71228  3.47004
3.03466  2.97817  2.61759  4.93897  2.12419    648 y - - -
          2.68618  4.42225  2.77519  2.73123  3.46354  2.40513  3.72494
3.29354  2.67741  2.69355  4.24690  2.90347  2.73739  3.18146  2.89801
2.37887  2.77519  2.98518  4.58477  3.61503
          0.01485  4.61273  5.33507  0.61958  0.77255  0.66070  0.72668
    509   3.44442  6.15369  1.34264  0.91116  5.42772  3.57246  4.19529
5.00679  3.31669  4.45488  5.33995  3.02067  4.24276  3.37257  3.98946
3.28066  3.75168  4.53632  6.58510  5.00778    649 e - - -
          2.68618  4.42225  2.77519  2.73123  3.46354  2.40513  3.72494
3.29354  2.67741  2.69355  4.24690  2.90347  2.73739  3.18146  2.89801
2.37887  2.77519  2.98518  4.58477  3.61503
          0.01485  4.61273  5.33507  0.61958  0.77255  0.66070  0.72668
    510   2.24354  4.48840  4.32428  3.83888  3.66222  3.85924  4.53177
2.72645  3.70882  0.98095  3.58175  4.02044  4.37256  3.98352  3.91955
3.23224  2.37933  2.58291  5.30835  4.13270    650 l - - -
          2.68618  4.42225  2.77519  2.73123  3.46354  2.40513  3.72494
3.29354  2.67741  2.69355  4.24690  2.90347  2.73739  3.18146  2.89801
2.37887  2.77519  2.98518  4.58477  3.61503
          0.01485  4.61273  5.33507  0.61958  0.77255  0.66070  0.72668
    511   2.72603  4.45970  3.64071  3.08592  2.75279  3.68363  4.00946
2.98999  3.04219  2.71491  3.60793  2.73621  4.09684  3.34326  3.39254
2.08653  2.08407  2.06965  5.08404  3.84803    651 v - - -
          2.68618  4.42225  2.77519  2.73123  3.46354  2.40513  3.72494
3.29354  2.67741  2.69355  4.24690  2.90347  2.73739  3.18146  2.89801
2.37887  2.77519  2.98518  4.58477  3.61503
          0.01485  4.61273  5.33507  0.61958  0.77255  0.66070  0.72668
    512   2.79874  4.56614  3.84067  3.37493  3.87115  3.66491  4.29643
3.10770  3.27821  1.81837  3.82091  3.68212  1.40698  3.62449  3.58209
3.02690  2.32630  2.89202  5.38314  4.15736    652 p - - -
```

```
          2.68618  4.42225  2.77519  2.73123  3.46354  2.40513  3.72494
3.29354  2.67741  2.69355  4.24690  2.90347  2.73739  3.18146  2.89801
2.37887  2.77519  2.98518  4.58477  3.61503
          0.01485  4.61273  5.33507  0.61958  0.77255  0.66070  0.72668
    513   2.78815  5.28949  2.92644  1.71581  4.62820  3.52080  3.73413
4.11087  2.16590  3.59003  4.33650  2.52679  2.95070  2.37975  2.96651
2.32180  3.02170  3.67767  5.72215  4.31177    653 e - - -
          2.68618  4.42225  2.77519  2.73123  3.46354  2.40513  3.72494
3.29354  2.67741  2.69355  4.24690  2.90347  2.73739  3.18146  2.89801
2.37887  2.77519  2.98518  4.58477  3.61503
          0.01485  4.61273  5.33507  0.61958  0.77255  0.66070  0.72668
    514   3.02178  5.36775  2.91244  2.61304  4.79284  3.60357  3.94242
4.25577  2.09564  3.76110  4.56933  1.16713  2.71890  3.08095  3.04253
2.99392  3.28436  3.84847  5.89233  4.52904    654 n - - -
          2.68618  4.42225  2.77519  2.73123  3.46354  2.40513  3.72494
3.29354  2.67741  2.69355  4.24690  2.90347  2.73739  3.18146  2.89801
2.37887  2.77519  2.98518  4.58477  3.61503
          0.01485  4.61273  5.33507  0.61958  0.77255  0.66070  0.72668
    515   3.35903  4.63196  5.29022  4.79062  3.99534  4.79029  5.42467
1.01410  4.69424  2.57157  3.79271  4.93455  5.09881  4.94230  4.85509
4.18143  2.60520  1.16827  5.90372  4.69055    655 i - - -
          2.68618  4.42225  2.77519  2.73123  3.46354  2.40513  3.72494
3.29354  2.67741  2.69355  4.24690  2.90347  2.73739  3.18146  2.89801
2.37887  2.77519  2.98518  4.58477  3.61503
          0.01485  4.61273  5.33507  0.61958  0.77255  0.66070  0.72668
    516   2.98389  4.40452  4.54068  3.95708  2.58929  4.13663  4.43292
1.25343  2.66791  1.86756  3.33552  4.14305  4.47294  4.01321  3.94248
3.44040  3.21520  2.36484  5.01904  3.82071    656 i - - -
          2.68618  4.42225  2.77519  2.73123  3.46354  2.40513  3.72494
3.29354  2.67741  2.69355  4.24690  2.90347  2.73739  3.18146  2.89801
2.37887  2.77519  2.98518  4.58477  3.61503
          0.01485  4.61273  5.33507  0.61958  0.77255  0.66070  0.72668
    517   3.81989  5.46157  4.63177  4.64160  5.65875  0.15555  5.64864
5.50050  4.91378  5.02620  6.04887  4.79961  4.86342  5.18091  5.01101
4.02051  4.35117  4.88796  6.50019  5.80465    657 G - - -
          2.68618  4.42225  2.77519  2.73123  3.46354  2.40513  3.72494
3.29354  2.67741  2.69355  4.24690  2.90347  2.73739  3.18146  2.89801
2.37887  2.77519  2.98518  4.58477  3.61503
          0.01485  4.61273  5.33507  0.61958  0.77255  0.66070  0.72668
    518   2.74196  5.05361  2.05323  2.20883  3.06543  3.54244  3.74516
3.76050  2.52115  3.32565  3.03154  3.04655  3.93779  2.43909  2.99786
2.74638  2.48873  3.40319  5.53312  4.17079    658 d - - -
          2.68618  4.42225  2.77519  2.73123  3.46354  2.40513  3.72494
3.29354  2.67741  2.69355  4.24690  2.90347  2.73739  3.18146  2.89801
2.37887  2.77519  2.98518  4.58477  3.61503
          0.01485  4.61273  5.33507  0.61958  0.77255  0.66070  0.72668
    519   2.74299  4.75917  2.63964  2.71310  3.95405  3.61784  3.83851
2.73536  2.33845  2.52071  3.84417  3.20522  2.13311  3.03660  3.13004
2.83608  2.97498  2.59006  5.30084  4.00657    659 p - - -
          2.68618  4.42225  2.77519  2.73123  3.46354  2.40513  3.72494
3.29354  2.67741  2.69355  4.24690  2.90347  2.73739  3.18146  2.89801
2.37887  2.77519  2.98518  4.58477  3.61503
          0.11519  4.61273  2.31386  0.61958  0.77255  0.66070  0.72668
```

```
     520   2.21870  5.59458  1.00487  2.41144  5.02498  3.47177  4.04268
4.53522  3.04421  4.04780  4.88006  2.29335  4.09243  3.20571  3.62886
3.03487  3.42358  4.08213  6.21049  4.72216    660 d - - -
           2.68618  4.42225  2.77519  2.73123  3.46354  2.40513  3.72494
3.29354  2.67741  2.69355  4.24690  2.90347  2.73739  3.18146  2.89801
2.37887  2.77519  2.98518  4.58477  3.61503
           0.01641  4.51395  5.23629  0.61958  0.77255  0.80147  0.59542
     521   2.34034  4.91672  3.09111  1.90433  4.21744  3.50811  3.76156
3.63657  2.55841  3.23376  4.03953  3.06886  2.99358  2.90695  3.02385
2.33105  2.48428  2.62419  5.46675  4.12611    661 e - - -
           2.68618  4.42225  2.77519  2.73123  3.46354  2.40513  3.72494
3.29354  2.67741  2.69355  4.24690  2.90347  2.73739  3.18146  2.89801
2.37887  2.77519  2.98518  4.58477  3.61503
           0.01641  4.51395  5.23629  0.61958  0.77255  0.80147  0.59542
     522   2.97200  5.52578  1.42156  2.38269  4.84717  3.49680  3.84988
4.34818  2.12383  3.81744  4.59670  2.29170  4.00663  2.32143  3.19784
2.89468  3.22330  3.90896  5.95062  4.50095    662 d - - -
           2.68618  4.42225  2.77519  2.73123  3.46354  2.40513  3.72494
3.29354  2.67741  2.69355  4.24690  2.90347  2.73739  3.18146  2.89801
2.37887  2.77519  2.98518  4.58477  3.61503
           0.01641  4.51395  5.23629  0.61958  0.77255  0.80147  0.59542
     523   2.50987  4.46701  3.81862  3.52094  4.72869  1.33398  4.58959
4.16724  3.58514  3.83486  4.63589  3.63966  3.99679  3.85383  3.90079
2.01952  1.42202  3.55456  6.05261  4.85278    663 g - - -
           2.68636  4.42243  2.77537  2.73141  3.46372  2.40530  3.72512
3.29372  2.67694  2.69341  4.24708  2.90321  2.73689  3.18110  2.89818
2.37905  2.77501  2.98536  4.58495  3.61521
           0.46936  0.99621  5.23629  0.67042  0.71640  0.54476  0.86746
     524   2.83806  4.57553  3.64581  3.09693  2.58803  3.78485  3.93530
3.08906  3.05462  2.35285  3.68303  1.72110  4.16673  3.35488  3.41137
2.49936  3.07115  2.86432  4.85203  2.60502    667 n - - -
           2.68618  4.42225  2.77519  2.73123  3.46354  2.40513  3.72494
3.29354  2.67741  2.69355  4.24690  2.90347  2.73739  3.18146  2.89801
2.37887  2.77519  2.98518  4.58477  3.61503
           0.01485  4.61273  5.33507  0.61958  0.77255  0.66070  0.72668
     525   2.88731  4.33432  4.37487  3.79631  3.45155  4.03082  4.35711
1.55973  3.67128  2.06532  3.41494  4.01291  4.38710  2.77969  3.85781
3.32685  2.54622  1.70091  5.02901  3.83873    668 i - - -
           2.68618  4.42225  2.77519  2.73123  3.46354  2.40513  3.72494
3.29354  2.67741  2.69355  4.24690  2.90347  2.73739  3.18146  2.89801
2.37887  2.77519  2.98518  4.58477  3.61503
           0.01485  4.61273  5.33507  0.61958  0.77255  0.39012  1.13004
     526   2.77196  4.78303  3.30916  2.74898  3.97530  3.64962  3.86708
2.75634  2.31276  2.57467  3.86881  2.64333  4.03637  2.62170  3.15553
2.10739  3.00325  2.25067  5.32041  4.02841    669 s - - -
           2.68618  4.42225  2.77519  2.73123  3.46354  2.40513  3.72494
3.29354  2.67741  2.69355  4.24690  2.90347  2.73739  3.18146  2.89801
2.37887  2.77519  2.98518  4.58477  3.61503
           0.01350  4.70791  5.43026  0.61958  0.77255  0.48576  0.95510
     527   2.04557  4.35855  4.03725  3.46856  3.49182  3.85958  4.17663
1.96090  3.38090  1.75343  3.47975  2.78553  4.24425  3.64332  3.63737
3.13995  2.48798  2.53556  4.99493  3.79017    670 l - - -
           2.68618  4.42225  2.77519  2.73123  3.46354  2.40513  3.72494
3.29354  2.67741  2.69355  4.24690  2.90347  2.73739  3.18146  2.89801
2.37887  2.77519  2.98518  4.58477  3.61503
```

```
         0.01350  4.70791  5.43026  0.61958  0.77255  0.48576  0.95510
    528   2.82776  5.11442  3.17405  2.61013  4.39609  3.62354  3.79616
3.82043  1.47620  3.38083  3.05533  2.56859  4.01236  2.42932  2.93038
2.83480  3.05398  2.71616  5.57826  4.23707    671 k - - -
         2.68618  4.42225  2.77519  2.73123  3.46354  2.40513  3.72494
3.29354  2.67741  2.69355  4.24690  2.90347  2.73739  3.18146  2.89801
2.37887  2.77519  2.98518  4.58477  3.61503
         0.01350  4.70791  5.43026  0.61958  0.77255  0.48576  0.95510
    529   2.77370  4.69497  4.12790  4.03296  5.17069  0.52046  5.09569
4.61229  4.19293  4.32850  5.15679  4.01442  4.26460  4.43247  4.40577
2.96875  2.20059  3.91395  6.46858  5.35139    672 G - - -
         2.68618  4.42225  2.77519  2.73123  3.46354  2.40513  3.72494
3.29354  2.67741  2.69355  4.24690  2.90347  2.73739  3.18146  2.89801
2.37887  2.77519  2.98518  4.58477  3.61503
         0.01350  4.70791  5.43026  0.61958  0.77255  0.48576  0.95510
    530   3.43901  4.70961  5.39449  4.85426  1.54713  4.80560  5.18293
1.36098  4.71832  2.23437  3.53161  4.93891  5.06268  4.83735  4.77893
4.16989  3.67967  1.47034  5.51059  4.27970    673 i - - -
         2.68618  4.42225  2.77519  2.73123  3.46354  2.40513  3.72494
3.29354  2.67741  2.69355  4.24690  2.90347  2.73739  3.18146  2.89801
2.37887  2.77519  2.98518  4.58477  3.61503
         0.10856  4.70791  2.36608  0.61958  0.77255  0.48576  0.95510
    531   2.72262  4.66751  3.78938  3.65488  4.93202  3.38221  4.80089
4.46232  3.80993  4.12337  4.96071  3.79079  0.67717  4.09962  4.08411
1.99029  3.24440  3.81722  6.24474  5.03410    674 P - - -
         2.68618  4.42225  2.77519  2.73123  3.46354  2.40513  3.72494
3.29354  2.67741  2.69355  4.24690  2.90347  2.73739  3.18146  2.89801
2.37887  2.77519  2.98518  4.58477  3.61503
         0.01483  4.61419  5.33653  0.61958  0.77255  0.65835  0.72920
    532   1.74474  4.31950  4.50964  3.92818  1.86133  4.02009  4.36417
2.53713  3.78826  2.25186  2.50283  4.07714  4.38486  3.97573  3.92090
3.32851  3.12767  2.05402  4.94613  3.75553    675 a - - -
         2.68618  4.42225  2.77519  2.73123  3.46354  2.40513  3.72494
3.29354  2.67741  2.69355  4.24690  2.90347  2.73739  3.18146  2.89801
2.37887  2.77519  2.98518  4.58477  3.61503
         0.12153  4.61419  2.25831  0.61958  0.77255  0.65835  0.72920
    533   3.70054  5.79882  2.98312  0.37938  5.25819  3.83617  4.54223
4.86347  3.52561  4.38836  5.38639  3.52432  4.48461  3.77691  3.96851
3.64215  4.02579  4.49630  6.29258  5.08982    676 E - - -
         2.68618  4.42225  2.77519  2.73123  3.46354  2.40513  3.72494
3.29354  2.67741  2.69355  4.24690  2.90347  2.73739  3.18146  2.89801
2.37887  2.77519  2.98518  4.58477  3.61503
         0.01649  4.50914  5.23149  0.61958  0.77255  0.80751  0.59053
    534   3.35788  5.53754  3.53700  2.99009  5.01893  3.90402  3.88084
4.38644  0.81176  3.77942  4.65002  3.41444  4.29798  2.20788  2.37294
3.32475  3.52540  4.04835  5.78579  4.60765    677 k - - -
         2.68618  4.42225  2.77519  2.73123  3.46354  2.40513  3.72494
3.29354  2.67741  2.69355  4.24690  2.90347  2.73739  3.18146  2.89801
2.37887  2.77519  2.98518  4.58477  3.61503
         0.01649  4.50914  5.23149  0.61958  0.77255  0.80751  0.59053
    535   2.19541  4.47841  3.54232  3.00761  3.75703  1.95662  3.99362
3.12617  2.97703  2.83236  3.71135  3.39788  4.02676  3.28702  3.34740
2.29414  2.94437  2.30244  5.17737  2.90492    678 g - - -
```

```
          2.68618  4.42225  2.77519  2.73123  3.46354  2.40513  3.72494
3.29354  2.67741  2.69355  4.24690  2.90347  2.73739  3.18146  2.89801
2.37887  2.77519  2.98518  4.58477  3.61503
          0.01649  4.50914  5.23149  0.61958  0.77255  0.80751  0.59053
    536   2.71199  4.41017  3.66462  3.12900  3.50475  2.82478  3.99432
2.36308  3.07662  2.66358  3.56685  3.49406  4.09089  3.37547  3.40426
2.01405  2.96744  2.71336  4.97773  2.01393    679 y - - -
          2.68618  4.42225  2.77519  2.73123  3.46354  2.40513  3.72494
3.29354  2.67741  2.69355  4.24690  2.90347  2.73739  3.18146  2.89801
2.37887  2.77519  2.98518  4.58477  3.61503
          0.23112  4.50914  1.63296  0.61958  0.77255  0.32700  1.27685
    537   2.32750  4.53461  3.44172  2.87916  2.86762  3.64169  3.89079
3.06489  2.36830  2.76108  2.75978  3.32220  4.02932  3.16473  3.23273
2.11466  2.40390  2.82265  5.10915  3.85490    680 s - - -
          2.68618  4.42225  2.77519  2.73123  3.46354  2.40513  3.72494
3.29354  2.67741  2.69355  4.24690  2.90347  2.73739  3.18146  2.89801
2.37887  2.77519  2.98518  4.58477  3.61503
          0.01601  4.53860  5.26094  0.61958  0.77255  0.76940  0.62230
    538   2.74500  5.20706  2.93483  2.15210  4.53784  3.49608  3.70668
4.01046  2.10664  3.50837  4.26226  2.10806  2.97578  2.81319  2.94010
2.34359  2.46565  3.59226  5.65532  4.25527    681 k - - -
          2.68618  4.42225  2.77519  2.73123  3.46354  2.40513  3.72494
3.29354  2.67741  2.69355  4.24690  2.90347  2.73739  3.18146  2.89801
2.37887  2.77519  2.98518  4.58477  3.61503
          0.01601  4.53860  5.26094  0.61958  0.77255  0.76940  0.62230
    539   2.85451  4.84309  3.25062  2.86091  3.65551  3.61113  3.93640
3.59554  2.91544  3.21489  4.09930  2.50557  4.10293  3.24961  3.31997
1.62986  3.14116  3.29268  5.12746  1.75772    682 s - - -
          2.68623  4.42230  2.77524  2.73128  3.46359  2.40518  3.72499
3.29359  2.67746  2.69360  4.24694  2.90312  2.73744  3.18151  2.89805
2.37861  2.77524  2.98523  4.58482  3.61508
          0.72361  1.60338  1.15904  0.18612  1.77299  0.54551  0.86642
    540   2.76363  4.87179  2.82169  2.70218  4.69606  1.68939  4.14134
4.24548  3.08097  3.83125  4.67174  1.27165  3.97453  3.34923  3.51526
2.82726  3.17308  3.73251  5.93008  4.59335    684 n - - -
          2.68618  4.42225  2.77519  2.73123  3.46354  2.40513  3.72494
3.29354  2.67741  2.69355  4.24690  2.90347  2.73739  3.18146  2.89801
2.37887  2.77519  2.98518  4.58477  3.61503
          0.02061  4.28789  5.01024  0.61958  0.77255  0.78081  0.61256
    541   2.31166  5.11508  2.48946  2.39834  4.42581  3.45992  3.65962
3.88959  2.40942  3.40528  4.16700  2.41547  3.85999  2.37958  2.48169
2.66580  2.92060  3.48827  5.56660  3.02596    685 a - - -
          2.68618  4.42225  2.77519  2.73123  3.46354  2.40513  3.72494
3.29354  2.67741  2.69355  4.24690  2.90347  2.73739  3.18146  2.89801
2.37887  2.77519  2.98518  4.58477  3.61503
          0.01830  4.40609  5.12843  0.61958  0.77255  0.92309  0.50632
    542   2.77410  5.16667  3.03068  2.48579  4.51500  2.29955  3.68476
3.96044  2.02634  3.45755  4.23210  3.01092  3.91997  2.27184  2.29506
2.32420  2.99883  3.56481  5.59407  4.24041    686 k - - -
          2.68618  4.42225  2.77519  2.73123  3.46354  2.40513  3.72494
3.29354  2.67741  2.69355  4.24690  2.90347  2.73739  3.18146  2.89801
2.37887  2.77519  2.98518  4.58477  3.61503
          0.01830  4.40609  5.12843  0.61958  0.77255  0.66637  0.72066
```

```
    543   2.70892  4.38082  3.85031  3.32281  3.64140  1.79971  4.16354
1.97922  3.26042  2.67085  3.60742  3.63090  2.85205  3.54875  3.56192
2.98484  2.99904  2.15455  5.13107  3.91402    687 g - - -
          2.68618  4.42225  2.77519  2.73123  3.46354  2.40513  3.72494
3.29354  2.67741  2.69355  4.24690  2.90347  2.73739  3.18146  2.89801
2.37887  2.77519  2.98518  4.58477  3.61503
          0.01638  4.51552  5.23787  0.61958  0.77255  0.33012  1.26881
    544   2.42020  5.08020  3.10296  2.25750  4.35512  3.57824  3.78139
3.79080  2.19974  3.35713  4.14713  3.08670  3.97382  2.91076  3.02459
2.13395  1.97749  3.43395  5.56427  3.04922    688 t - - -
          2.68618  4.42225  2.77519  2.73123  3.46354  2.40513  3.72494
3.29354  2.67741  2.69355  4.24690  2.90347  2.73739  3.18146  2.89801
2.37887  2.77519  2.98518  4.58477  3.61503
          0.01350  4.70791  5.43026  0.61958  0.77255  0.48576  0.95510
    545   2.78357  4.27008  4.20607  3.62086  3.37452  2.84664  4.18045
2.31582  2.71918  1.87795  3.38250  3.84497  4.25010  3.73277  3.69418
3.16496  3.01575  1.68951  4.88376  2.88875    689 v - - -
          2.68618  4.42225  2.77519  2.73123  3.46354  2.40513  3.72494
3.29354  2.67741  2.69355  4.24690  2.90347  2.73739  3.18146  2.89801
2.37887  2.77519  2.98518  4.58477  3.61503
          0.01350  4.70791  5.43026  0.61958  0.77255  0.48576  0.95510
    546   2.81455  4.33993  4.10527  3.52073  2.68527  3.88853  4.17201
2.32641  2.62865  2.50893  3.44485  3.79493  4.25764  3.66192  2.75698
3.16732  3.04615  1.39510  4.95479  3.75246    690 v - - -
          2.68618  4.42225  2.77519  2.73123  3.46354  2.40513  3.72494
3.29354  2.67741  2.69355  4.24690  2.90347  2.73739  3.18146  2.89801
2.37887  2.77519  2.98518  4.58477  3.61503
          0.01350  4.70791  5.43026  0.61958  0.77255  0.48576  0.95510
    547   2.77933  5.00181  3.16890  2.60833  4.25229  3.60225  3.79962
3.67115  2.00553  2.66607  4.07282  3.12826  2.97189  2.94408  2.29067
2.43914  2.49461  2.70740  5.49738  4.16039    691 k - - -
          2.68618  4.42225  2.77519  2.73123  3.46354  2.40513  3.72494
3.29354  2.67741  2.69355  4.24690  2.90347  2.73739  3.18146  2.89801
2.37887  2.77519  2.98518  4.58477  3.61503
          0.01350  4.70791  5.43026  0.61958  0.77255  0.48576  0.95510
    548   4.02385  6.06573  3.23490  0.26426  5.57841  4.08320  4.84596
5.24987  3.87442  4.74179  5.76500  3.80524  4.74659  4.09772  4.31868
3.95941  4.36027  4.86907  6.55423  5.40950    692 E - - -
          2.68618  4.42225  2.77519  2.73123  3.46354  2.40513  3.72494
3.29354  2.67741  2.69355  4.24690  2.90347  2.73739  3.18146  2.89801
2.37887  2.77519  2.98518  4.58477  3.61503
          0.01350  4.70791  5.43026  0.61958  0.77255  0.48576  0.95510
    549   3.81846  5.18732  4.81359  4.42066  2.51854  4.61844  3.96425
3.76529  4.22151  3.21915  4.35349  4.36382  2.78703  4.36502  4.34176
3.97758  4.05307  3.61350  0.98406  1.53581    693 w - - -
          2.68618  4.42225  2.77519  2.73123  3.46354  2.40513  3.72494
3.29354  2.67741  2.69355  4.24690  2.90347  2.73739  3.18146  2.89801
2.37887  2.77519  2.98518  4.58477  3.61503
          0.01350  4.70791  5.43026  0.61958  0.77255  0.48576  0.95510
    550   3.78823  4.99919  5.72050  5.22423  3.70422  5.26893  5.76038
0.72333  5.08525  1.45553  3.46988  5.41514  5.40957  5.17882  5.15476
4.70143  4.02953  2.16211  5.89323  4.79308    694 i - - -
          2.68618  4.42225  2.77519  2.73123  3.46354  2.40513  3.72494
3.29354  2.67741  2.69355  4.24690  2.90347  2.73739  3.18146  2.89801
2.37887  2.77519  2.98518  4.58477  3.61503
```

```
         0.01350  4.70791  5.43026  0.61958  0.77255  0.48576  0.95510
    551   2.96927  5.02401  3.24001  3.11703  4.92692  3.54459  4.48996
4.40898  3.39413  4.06867  4.93102  0.67992  4.25093  3.71296  3.77426
3.06686  2.33808  3.90086  6.20711  4.87517    695 N - - -
         2.68618  4.42225  2.77519  2.73123  3.46354  2.40513  3.72494
3.29354  2.67741  2.69355  4.24690  2.90347  2.73739  3.18146  2.89801
2.37887  2.77519  2.98518  4.58477  3.61503
         0.01350  4.70791  5.43026  0.61958  0.77255  0.48576  0.95510
    552   3.98868  5.60669  4.81325  4.83535  5.82610  0.12867  5.81900
5.70431  5.11516  5.20759  6.24044  4.98127  5.00675  5.37566  5.18924
4.19341  4.52384  5.07668  6.63103  5.97905    696 G - - -
         2.68618  4.42225  2.77519  2.73123  3.46354  2.40513  3.72494
3.29354  2.67741  2.69355  4.24690  2.90347  2.73739  3.18146  2.89801
2.37887  2.77519  2.98518  4.58477  3.61503
         0.01350  4.70791  5.43026  0.61958  0.77255  0.48576  0.95510
    553   1.08597  4.71958  3.62474  3.26605  4.81672  2.35627  4.45055
4.27273  3.36044  3.88884  4.67576  2.57920  4.10180  3.65039  3.76123
1.73955  3.13570  3.70050  6.08867  4.82322    697 a - - -
         2.68618  4.42225  2.77519  2.73123  3.46354  2.40513  3.72494
3.29354  2.67741  2.69355  4.24690  2.90347  2.73739  3.18146  2.89801
2.37887  2.77519  2.98518  4.58477  3.61503
         0.01350  4.70791  5.43026  0.61958  0.77255  0.48576  0.95510
    554   2.32176  5.18919  3.01016  2.61792  4.66341  3.55320  3.93239
4.12641  2.72008  3.66014  4.44100  1.47638  4.04447  2.41131  3.19238
1.98227  3.14421  3.70174  5.82972  4.45109    698 n - - -
         2.68618  4.42225  2.77519  2.73123  3.46354  2.40513  3.72494
3.29354  2.67741  2.69355  4.24690  2.90347  2.73739  3.18146  2.89801
2.37887  2.77519  2.98518  4.58477  3.61503
         0.01350  4.70791  5.43026  0.61958  0.77255  0.48576  0.95510
    555   1.39749  4.51220  3.99351  3.49992  4.07564  3.55608  4.40223
3.31262  3.45003  3.13071  4.02486  3.73046  2.12347  3.73625  3.76463
2.92906  2.30623  1.93475  5.51820  4.30510    699 a - - -
         2.68618  4.42225  2.77519  2.73123  3.46354  2.40513  3.72494
3.29354  2.67741  2.69355  4.24690  2.90347  2.73739  3.18146  2.89801
2.37887  2.77519  2.98518  4.58477  3.61503
         0.01350  4.70791  5.43026  0.61958  0.77255  0.48576  0.95510
    556   3.53685  4.80235  5.43757  4.90032  3.71217  4.93384  5.39959
2.17665  4.76674  1.77916  2.51927  5.05513  5.16250  4.90723  4.86017
4.30970  3.77892  0.76213  5.72367  4.60027    700 v - - -
         2.68618  4.42225  2.77519  2.73123  3.46354  2.40513  3.72494
3.29354  2.67741  2.69355  4.24690  2.90347  2.73739  3.18146  2.89801
2.37887  2.77519  2.98518  4.58477  3.61503
         0.01350  4.70791  5.43026  0.61958  0.77255  0.48576  0.95510
    557   2.80489  5.28089  3.02760  2.16874  4.61792  3.56475  3.75260
4.09195  1.60277  3.57748  4.32666  2.51246  3.04513  2.46467  2.94484
2.77071  3.03487  3.66806  5.71218  3.10167    701 k - - -
         2.68618  4.42225  2.77519  2.73123  3.46354  2.40513  3.72494
3.29354  2.67741  2.69355  4.24690  2.90347  2.73739  3.18146  2.89801
2.37887  2.77519  2.98518  4.58477  3.61503
         0.01350  4.70791  5.43026  0.61958  0.77255  0.48576  0.95510
    558   4.06456  5.26859  5.91347  5.33125  2.42464  5.41652  5.58041
2.64575  5.19240  0.53701  2.27456  5.54193  5.39760  5.00879  5.11952
4.80867  4.25567  2.97052  5.54016  4.51742    702 L - - -
```

```
          2.68618  4.42225  2.77519  2.73123  3.46354  2.40513  3.72494
3.29354  2.67741  2.69355  4.24690  2.90347  2.73739  3.18146  2.89801
2.37887  2.77519  2.98518  4.58477  3.61503
          0.01350  4.70791  5.43026  0.61958  0.77255  0.48576  0.95510
    559   2.78082  5.18975  3.04758  2.17286  4.49931  2.79376  3.75859
3.95800  1.94383  3.48075  4.24558  3.04685  3.95834  2.87056  2.98915
1.93089  2.58217  3.56243  5.64830  3.17008    703 s - - -
          2.68618  4.42225  2.77519  2.73123  3.46354  2.40513  3.72494
3.29354  2.67741  2.69355  4.24690  2.90347  2.73739  3.18146  2.89801
2.37887  2.77519  2.98518  4.58477  3.61503
          0.01350  4.70791  5.43026  0.61958  0.77255  0.48576  0.95510
    560   2.78722  5.00180  3.14643  2.61862  4.26656  3.59396  2.87058
2.85144  2.62119  3.28917  4.09920  2.25673  2.19469  2.97513  3.08169
2.13986  3.02642  3.35904  5.52085  4.17990    704 s - - -
          2.68618  4.42225  2.77519  2.73123  3.46354  2.40513  3.72494
3.29354  2.67741  2.69355  4.24690  2.90347  2.73739  3.18146  2.89801
2.37887  2.77519  2.98518  4.58477  3.61503
          0.01350  4.70791  5.43026  0.61958  0.77255  0.48576  0.95510
    561   2.77707  4.91449  3.20237  2.65549  4.13603  2.17120  3.83003
3.54832  2.64675  3.16985  2.95329  2.60401  4.00901  2.48575  3.09911
2.46365  3.01003  3.24143  5.42967  2.58290    705 g - - -
          2.68620  4.42228  2.77480  2.73126  3.46356  2.40515  3.72497
3.29356  2.67743  2.69357  4.24692  2.90349  2.73742  3.18149  2.89803
2.37889  2.77522  2.98521  4.58479  3.61506
          0.10420  2.35842  5.43026  0.27606  1.42199  0.48576  0.95510
    562   3.04855  5.37508  2.93566  2.66922  4.84289  3.62032  4.03246
4.33832  2.77950  3.84946  4.65749  2.46923  4.15386  1.01825  3.19978
2.33708  3.33394  3.91170  5.97762  4.59657    707 q - - -
          2.68618  4.42225  2.77519  2.73123  3.46354  2.40513  3.72494
3.29354  2.67741  2.69355  4.24690  2.90347  2.73739  3.18146  2.89801
2.37887  2.77519  2.98518  4.58477  3.61503
          0.01350  4.70791  5.43026  0.61958  0.77255  0.48576  0.95510
    563   2.76458  4.58831  3.56937  3.01908  3.81474  3.68296  4.02310
2.50185  2.98524  2.87434  3.75688  2.68004  4.10994  3.30269  3.36719
1.90176  1.85684  2.44470  5.23237  3.98227    708 t - - -
          2.68618  4.42225  2.77519  2.73123  3.46354  2.40513  3.72494
3.29354  2.67741  2.69355  4.24690  2.90347  2.73739  3.18146  2.89801
2.37887  2.77519  2.98518  4.58477  3.61503
          0.01350  4.70791  5.43026  0.61958  0.77255  0.48576  0.95510
    564   2.36713  4.31370  4.78454  4.18386  3.33229  4.11941  4.45276
1.81088  4.00107  1.33744  3.30331  4.24036  4.46271  4.14463  4.05305
3.43220  3.16982  2.07325  3.15708  3.78235    709 l - - -
          2.68618  4.42225  2.77519  2.73123  3.46354  2.40513  3.72494
3.29354  2.67741  2.69355  4.24690  2.90347  2.73739  3.18146  2.89801
2.37887  2.77519  2.98518  4.58477  3.61503
          0.01350  4.70791  5.43026  0.61958  0.77255  0.48576  0.95510
    565   2.77499  4.63386  3.46178  2.90079  2.95093  2.18705  3.93420
2.21085  2.40651  2.85698  3.73104  3.35502  4.08347  2.66878  3.25800
2.93039  2.47165  2.92069  5.19688  3.93628    710 g - - -
          2.68618  4.42225  2.77519  2.73123  3.46354  2.40513  3.72494
3.29354  2.67741  2.69355  4.24690  2.90347  2.73739  3.18146  2.89801
2.37887  2.77519  2.98518  4.58477  3.61503
          0.01350  4.70791  5.43026  0.61958  0.77255  0.48576  0.95510
```

566 3.12323 4.47909 4.95830 4.36992 2.42414 4.29947 4.51324
1.59799 4.18548 2.10002 1.59592 4.40467 4.61372 4.28442 4.21558
3.61811 3.35058 2.56466 4.90262 2.62792 711 m - - -
2.68618 4.42225 2.77519 2.73123 3.46354 2.40513 3.72494
3.29354 2.67741 2.69355 4.24690 2.90347 2.73739 3.18146 2.89801
2.37887 2.77519 2.98518 4.58477 3.61503
0.01350 4.70791 5.43026 0.61958 0.77255 0.48576 0.95510
567 2.81793 5.32821 2.51168 1.91472 4.66560 3.54782 3.76876
4.15206 2.52554 3.63069 4.37485 2.50466 2.46444 2.48475 3.02492
2.35436 2.56738 3.71550 5.76365 4.34716 712 e - - -
2.68618 4.42225 2.77519 2.73123 3.46354 2.40513 3.72494
3.29354 2.67741 2.69355 4.24690 2.90347 2.73739 3.18146 2.89801
2.37887 2.77519 2.98518 4.58477 3.61503
0.01350 4.70791 5.43026 0.61958 0.77255 0.48576 0.95510
568 2.47129 4.46928 5.04299 4.46039 2.14538 4.39283 4.76485
1.30451 4.29384 1.87969 3.41921 4.52465 4.70839 4.43296 4.34748
3.72211 3.37202 1.88485 5.21824 4.05356 713 i - - -
2.68618 4.42225 2.77519 2.73123 3.46354 2.40513 3.72494
3.29354 2.67741 2.69355 4.24690 2.90347 2.73739 3.18146 2.89801
2.37887 2.77519 2.98518 4.58477 3.61503
0.01350 4.70791 5.43026 0.61958 0.77255 0.48576 0.95510
569 2.88319 4.62225 3.73033 3.21266 3.68676 3.79741 2.81778
3.11944 3.06420 1.91999 3.74658 3.59206 4.22568 3.44940 3.37583
3.09643 1.31949 2.90723 5.16508 3.87540 714 t - - -
2.68618 4.42225 2.77519 2.73123 3.46354 2.40513 3.72494
3.29354 2.67741 2.69355 4.24690 2.90347 2.73739 3.18146 2.89801
2.37887 2.77519 2.98518 4.58477 3.61503
0.01350 4.70791 5.43026 0.61958 0.77255 0.48576 0.95510
570 2.14292 4.75798 3.53996 3.19356 4.45806 3.53790 4.35524
3.65792 3.26238 3.49471 4.37815 0.98657 4.16785 3.58591 3.64334
2.94125 3.18666 2.36719 5.82828 4.55009 715 n - - -
2.68618 4.42225 2.77519 2.73123 3.46354 2.40513 3.72494
3.29354 2.67741 2.69355 4.24690 2.90347 2.73739 3.18146 2.89801
2.37887 2.77519 2.98518 4.58477 3.61503
0.01350 4.70791 5.43026 0.61958 0.77255 0.48576 0.95510
571 2.80415 4.47441 3.76461 3.19709 3.59711 3.80423 4.07160
2.40024 3.13041 1.95682 3.56257 3.57911 4.18297 2.06646 3.45928
2.16785 3.03783 2.28248 5.07357 3.84922 716 l - - -
2.68618 4.42225 2.77519 2.73123 3.46354 2.40513 3.72494
3.29354 2.67741 2.69355 4.24690 2.90347 2.73739 3.18146 2.89801
2.37887 2.77519 2.98518 4.58477 3.61503
0.01350 4.70791 5.43026 0.61958 0.77255 0.48576 0.95510
572 2.90694 5.28656 2.33852 2.55231 4.65866 2.68915 3.89999
4.11909 2.21820 3.65020 4.43434 3.09493 4.04672 3.02922 3.18945
2.88575 1.35475 3.71442 5.82430 4.43132 717 t - - -
2.68618 4.42225 2.77519 2.73123 3.46354 2.40513 3.72494
3.29354 2.67741 2.69355 4.24690 2.90347 2.73739 3.18146 2.89801
2.37887 2.77519 2.98518 4.58477 3.61503
0.01350 4.70791 5.43026 0.61958 0.77255 0.48576 0.95510
573 2.36069 4.83023 3.29760 2.75071 4.11236 2.86131 3.89580
3.51066 2.37061 2.55916 3.98588 3.23655 4.02996 3.07877 3.16880
2.39119 1.68259 3.20448 5.42867 4.12620 718 t - - -
2.68618 4.42225 2.77519 2.73123 3.46354 2.40513 3.72494
3.29354 2.67741 2.69355 4.24690 2.90347 2.73739 3.18146 2.89801
2.37887 2.77519 2.98518 4.58477 3.61503

0.01350 4.70791 5.43026 0.61958 0.77255 0.48576 0.95510
574 2.34583 4.91122 3.20650 2.65482 3.04472 2.46303 2.89333
3.55129 2.64551 3.17217 3.99689 3.16505 4.00540 2.27053 3.09938
2.82316 2.20797 3.24238 5.43286 4.11090 719 t - - -
2.68618 4.42225 2.77519 2.73123 3.46354 2.40513 3.72494
3.29354 2.67741 2.69355 4.24690 2.90347 2.73739 3.18146 2.89801
2.37887 2.77519 2.98518 4.58477 3.61503
0.01350 4.70791 5.43026 0.61958 0.77255 0.48576 0.95510
575 2.83982 5.29284 3.04095 2.18532 4.62814 3.58703 3.77398
4.09324 1.96866 3.58400 4.34267 2.52466 3.98974 1.69742 2.93197
2.80745 3.06865 2.80439 5.72126 4.33543 720 q - - -
2.68618 4.42225 2.77519 2.73123 3.46354 2.40513 3.72494
3.29354 2.67741 2.69355 4.24690 2.90347 2.73739 3.18146 2.89801
2.37887 2.77519 2.98518 4.58477 3.61503
0.01350 4.70791 5.43026 0.61958 0.77255 0.48576 0.95510
576 4.62705 5.77367 5.15902 5.08940 2.79635 4.87159 4.31504
4.56637 4.94449 3.79323 5.15160 4.87732 5.34883 5.02217 4.92947
4.65395 4.92861 4.47859 4.45019 0.24676 721 Y - - -
2.68618 4.42225 2.77519 2.73123 3.46354 2.40513 3.72494
3.29354 2.67741 2.69355 4.24690 2.90347 2.73739 3.18146 2.89801
2.37887 2.77519 2.98518 4.58477 3.61503
0.01350 4.70791 5.43026 0.61958 0.77255 0.48576 0.95510
577 2.82839 5.26671 3.10832 2.25056 4.60599 3.59821 3.75862
4.06521 1.70585 3.55534 3.18103 3.07488 3.98670 2.09849 2.47329
2.80059 2.51239 3.65542 5.68985 4.31551 722 k - - -
2.68618 4.42225 2.77519 2.73123 3.46354 2.40513 3.72494
3.29354 2.67741 2.69355 4.24690 2.90347 2.73739 3.18146 2.89801
2.37887 2.77519 2.98518 4.58477 3.61503
0.01350 4.70791 5.43026 0.61958 0.77255 0.48576 0.95510
578 3.62673 4.82968 5.68948 5.24576 4.16117 5.22456 6.02826
0.68258 5.18369 2.55662 3.89050 5.40784 5.45896 5.44325 5.35445
4.67801 3.89482 1.31256 6.32102 5.10169 723 I - - -
2.68618 4.42225 2.77519 2.73123 3.46354 2.40513 3.72494
3.29354 2.67741 2.69355 4.24690 2.90347 2.73739 3.18146 2.89801
2.37887 2.77519 2.98518 4.58477 3.61503
0.01350 4.70791 5.43026 0.61958 0.77255 0.48576 0.95510
579 4.23731 5.86418 4.82648 4.24561 5.45647 4.45840 4.80535
5.13616 3.05989 4.49477 5.52683 4.51437 4.97129 4.03592 0.23170
4.32043 4.47918 4.83283 6.22544 5.27786 724 R - - -
2.68618 4.42225 2.77519 2.73123 3.46354 2.40513 3.72494
3.29354 2.67741 2.69355 4.24690 2.90347 2.73739 3.18146 2.89801
2.37887 2.77519 2.98518 4.58477 3.61503
0.01350 4.70791 5.43026 0.61958 0.77255 0.48576 0.95510
580 2.36070 2.10811 4.80534 4.20819 2.59096 4.11626 4.46701
1.60496 4.02446 1.96092 3.35604 4.25007 4.46746 4.17288 4.07293
3.43269 3.15942 2.28697 4.96862 3.79166 725 i - - -
2.68618 4.42225 2.77519 2.73123 3.46354 2.40513 3.72494
3.29354 2.67741 2.69355 4.24690 2.90347 2.73739 3.18146 2.89801
2.37887 2.77519 2.98518 4.58477 3.61503
0.01350 4.70791 5.43026 0.61958 0.77255 0.48576 0.95510
581 4.23731 5.86418 4.82648 4.24561 5.45647 4.45840 4.80535
5.13616 3.05989 4.49477 5.52683 4.51437 4.97129 4.03592 0.23170
4.32043 4.47918 4.83283 6.22544 5.27786 726 R - - -

```
          2.68618  4.42225  2.77519  2.73123  3.46354  2.40513  3.72494
3.29354  2.67741  2.69355  4.24690  2.90347  2.73739  3.18146  2.89801
2.37887  2.77519  2.98518  4.58477  3.61503
          0.01350  4.70791  5.43026  0.61958  0.77255  0.48576  0.95510
    582   3.29321  4.66462  4.86185  4.34215  3.11234  4.42419  4.44168
2.07686  4.17255  2.46175  3.63281  4.41754  4.76244  4.34554  4.28663
3.75750  3.53439  2.00229  4.80106  0.99726    727 y - - -
          2.68618  4.42225  2.77519  2.73123  3.46354  2.40513  3.72494
3.29354  2.67741  2.69355  4.24690  2.90347  2.73739  3.18146  2.89801
2.37887  2.77519  2.98518  4.58477  3.61503
          0.01350  4.70791  5.43026  0.61958  0.77255  0.48576  0.95510
    583   0.28347  4.85192  4.55918  4.47483  5.13285  3.64247  5.35360
4.41924  4.52049  4.24599  5.22420  4.33753  4.45844  4.77872  4.64959
3.21876  3.55442  3.89557  6.44482  5.38924    728 A - - -
          2.68618  4.42225  2.77519  2.73123  3.46354  2.40513  3.72494
3.29354  2.67741  2.69355  4.24690  2.90347  2.73739  3.18146  2.89801
2.37887  2.77519  2.98518  4.58477  3.61503
          0.01350  4.70791  5.43026  0.61958  0.77255  0.48576  0.95510
    584   2.72879  4.76525  3.52729  3.18351  4.73316  3.44299  4.38316
4.17701  3.27079  3.80409  4.60827  2.51926  4.11403  3.58371  3.66821
1.32433  1.33399  3.65959  6.01285  4.73507    729 s - - -
          2.68618  4.42225  2.77519  2.73123  3.46354  2.40513  3.72494
3.29354  2.67741  2.69355  4.24690  2.90347  2.73739  3.18146  2.89801
2.37887  2.77519  2.98518  4.58477  3.61503
          0.01350  4.70791  5.43026  0.61958  0.77255  0.48576  0.95510
    585   2.81092  5.27630  2.98122  2.13862  4.60752  2.87496  3.77233
4.08150  2.23784  3.57885  4.33285  2.18921  3.03032  2.87930  3.01409
2.77811  1.95005  3.66205  5.72582  4.32341    730 t - - -
          2.68618  4.42225  2.77519  2.73123  3.46354  2.40513  3.72494
3.29354  2.67741  2.69355  4.24690  2.90347  2.73739  3.18146  2.89801
2.37887  2.77519  2.98518  4.58477  3.61503
          0.01350  4.70791  5.43026  0.61958  0.77255  0.48576  0.95510
    586   2.94034  5.45404  1.98428  2.47311  4.78977  2.36258  3.87106
4.28103  2.68167  3.76497  4.52846  1.83534  4.03674  2.39124  3.19815
2.88496  2.51833  3.84618  5.90670  4.47417    731 n - - -
          2.68618  4.42225  2.77519  2.73123  3.46354  2.40513  3.72494
3.29354  2.67741  2.69355  4.24690  2.90347  2.73739  3.18146  2.89801
2.37887  2.77519  2.98518  4.58477  3.61503
          0.01350  4.70791  5.43026  0.61958  0.77255  0.48576  0.95510
    587   3.12927  5.64602  1.39990  2.50170  5.05764  2.52638  4.05683
4.57256  2.98251  4.05386  4.85004  1.91788  4.14802  3.19871  3.54416
1.95343  3.41503  4.11088  6.20058  4.72689    732 d - - -
          2.68618  4.42225  2.77519  2.73123  3.46354  2.40513  3.72494
3.29354  2.67741  2.69355  4.24690  2.90347  2.73739  3.18146  2.89801
2.37887  2.77519  2.98518  4.58477  3.61503
          0.01350  4.70791  5.43026  0.61958  0.77255  0.48576  0.95510
    588   2.80937  5.09481  2.53521  2.59103  4.43910  2.74965  3.85767
3.87458  2.65778  3.44682  4.24051  2.21503  4.00680  2.99372  3.13724
2.82067  1.71520  2.68901  5.65541  4.29272    733 t - - -
          2.68618  4.42225  2.77519  2.73123  3.46354  2.40513  3.72494
3.29354  2.67741  2.69355  4.24690  2.90347  2.73739  3.18146  2.89801
2.37887  2.77519  2.98518  4.58477  3.61503
          0.01350  4.70791  5.43026  0.61958  0.77255  0.48576  0.95510
```

```
    589   2.42694  5.26232  3.00657  1.91283  4.59822  3.54606  3.74513
 4.07626  2.16144  3.56553  4.31114  2.26759  3.07858  2.84696  2.97919
 2.35611  2.56540  3.64924  5.70534  4.30063    734 e - - -
          2.68618  4.42225  2.77519  2.73123  3.46354  2.40513  3.72494
 3.29354  2.67741  2.69355  4.24690  2.90347  2.73739  3.18146  2.89801
 2.37887  2.77519  2.98518  4.58477  3.61503
          0.01350  4.70791  5.43026  0.61958  0.77255  0.48576  0.95510
    590   2.04905  4.39331  3.84803  3.27648  3.52052  2.83650  4.07882
 2.43208  3.20940  2.21561  3.50554  3.62382  4.17890  2.78207  3.51078
 3.06169  2.47677  2.01722  4.99601  3.78032    735 v - - -
          2.68618  4.42225  2.77519  2.73123  3.46354  2.40513  3.72494
 3.29354  2.67741  2.69355  4.24690  2.90347  2.73739  3.18146  2.89801
 2.37887  2.77519  2.98518  4.58477  3.61503
          0.01350  4.70791  5.43026  0.61958  0.77255  0.48576  0.95510
    591   2.78975  4.22981  4.39139  3.79971  2.26328  3.91264  4.21172
 2.27518  3.65585  2.06892  3.34132  3.94632  4.27972  3.85078  3.78850
 2.22389  2.43711  2.47403  4.81783  2.35277    736 l - - -
          2.68618  4.42225  2.77519  2.73123  3.46354  2.40513  3.72494
 3.29354  2.67741  2.69355  4.24690  2.90347  2.73739  3.18146  2.89801
 2.37887  2.77519  2.98518  4.58477  3.61503
          0.01350  4.70791  5.43026  0.61958  0.77255  0.48576  0.95510
    592   2.81349  4.23566  4.50001  3.90612  1.74414  2.93535  4.27763
 1.94411  3.74877  2.36731  2.58250  4.02136  4.31788  3.93094  3.85566
 3.25318  2.46609  2.43314  4.85812  3.67252    737 f - - -
          2.68618  4.42225  2.77519  2.73123  3.46354  2.40513  3.72494
 3.29354  2.67741  2.69355  4.24690  2.90347  2.73739  3.18146  2.89801
 2.37887  2.77519  2.98518  4.58477  3.61503
          0.01350  4.70791  5.43026  0.61958  0.77255  0.48576  0.95510
    593   2.77674  4.51389  3.63031  3.06213  3.65338  3.74705  3.99633
 2.49014  3.00159  2.72609  3.61738  2.11549  4.12762  3.31834  2.65673
 2.99156  2.48749  2.38398  2.64612  3.85478    738 n - - -
          2.68618  4.42225  2.77519  2.73123  3.46354  2.40513  3.72494
 3.29354  2.67741  2.69355  4.24690  2.90347  2.73739  3.18146  2.89801
 2.37887  2.77519  2.98518  4.58477  3.61503
          0.01350  4.70791  5.43026  0.61958  0.77255  0.48576  0.95510
    594   2.90932  4.30015  4.70030  4.09999  2.64566  4.07319  4.39756
 2.57672  3.92583  1.50771  2.12076  4.17682  4.42020  4.07565  3.99292
 2.60386  3.14067  1.84641  4.91282  3.74402    739 l - - -
          2.68618  4.42225  2.77519  2.73123  3.46354  2.40513  3.72494
 3.29354  2.67741  2.69355  4.24690  2.90347  2.73739  3.18146  2.89801
 2.37887  2.77519  2.98518  4.58477  3.61503
          0.01350  4.70791  5.43026  0.61958  0.77255  0.48576  0.95510
    595   2.77233  3.13909  2.06826  2.80557  3.89768  3.66707  3.03475
 2.30661  2.78864  2.95071  3.81129  2.66060  4.05508  3.12014  3.21078
 2.89127  3.00489  2.55848  5.26996  3.98998    740 d - - -
          2.68618  4.42225  2.77519  2.73123  3.46354  2.40513  3.72494
 3.29354  2.67741  2.69355  4.24690  2.90347  2.73739  3.18146  2.89801
 2.37887  2.77519  2.98518  4.58477  3.61503
          0.01350  4.70791  5.43026  0.61958  0.77255  0.48576  0.95510
    596   2.77408  4.46898  3.69314  3.12675  3.60053  3.76294  4.02258
 2.14339  3.07456  2.33731  3.57461  2.31154  2.99869  3.37347  3.41595
 3.01270  2.18100  2.73457  5.05460  2.96806    741 i - - -
          2.68618  4.42225  2.77519  2.73123  3.46354  2.40513  3.72494
 3.29354  2.67741  2.69355  4.24690  2.90347  2.73739  3.18146  2.89801
 2.37887  2.77519  2.98518  4.58477  3.61503
```

0.01350 4.70791 5.43026 0.61958 0.77255 0.48576 0.95510
597 2.77012 5.26079 2.59194 2.46765 4.59810 2.85155 3.73261
3.15580 2.16627 3.56223 4.30398 2.53810 3.93832 2.14309 2.48081
2.41506 3.00126 3.64736 5.69757 4.29214 742 q - - -
2.68618 4.42225 2.77519 2.73123 3.46354 2.40513 3.72494
3.29354 2.67741 2.69355 4.24690 2.90347 2.73739 3.18146 2.89801
2.37887 2.77519 2.98518 4.58477 3.61503
0.01350 4.70791 5.43026 0.61958 0.77255 0.48576 0.95510
598 2.82529 5.18084 3.00884 1.94698 4.48668 2.12040 3.81671
3.93414 2.59659 2.73464 4.26481 2.17817 3.99623 2.94217 3.07860
2.81549 2.51593 3.55577 5.67099 4.29374 743 e - - -
2.68640 4.42247 2.77497 2.73146 3.46376 2.40458 3.72517
3.29376 2.67763 2.69377 4.24712 2.90318 2.73727 3.18101 2.89823
2.37885 2.77497 2.98541 4.58499 3.61526
0.53581 1.12423 2.40904 0.81631 0.58350 0.48576 0.95510
599 2.25104 4.73899 3.32553 2.83889 4.10187 3.55236 3.97619
3.48690 2.84627 2.05181 4.00306 1.88425 4.03449 3.18351 3.26538
2.02062 3.02503 3.17532 5.44585 4.15666 748 n - - -
2.68618 4.42225 2.77519 2.73123 3.46354 2.40513 3.72494
3.29354 2.67741 2.69355 4.24690 2.90347 2.73739 3.18146 2.89801
2.37887 2.77519 2.98518 4.58477 3.61503
0.01477 4.61851 5.34086 0.61958 0.77255 0.65133 0.73679
600 2.75843 5.19312 2.54316 2.45969 4.50806 3.52332 3.73086
2.93383 2.48736 3.48711 4.24713 2.21665 3.07420 2.10723 2.97638
2.32913 2.99182 3.56853 5.64758 4.25353 749 q - - -
2.68618 4.42225 2.77519 2.73123 3.46354 2.40513 3.72494
3.29354 2.67741 2.69355 4.24690 2.90347 2.73739 3.18146 2.89801
2.37887 2.77519 2.98518 4.58477 3.61503
0.01477 4.61851 5.34086 0.61958 0.77255 0.65133 0.73679
601 2.81227 4.28912 4.25767 3.67655 2.20838 3.91543 4.22429
2.22236 3.55899 2.04014 3.34209 3.89377 2.06984 3.78071 3.74604
3.20584 2.41928 2.45968 4.89870 3.70144 750 l - - -
2.68618 4.42225 2.77519 2.73123 3.46354 2.40513 3.72494
3.29354 2.67741 2.69355 4.24690 2.90347 2.73739 3.18146 2.89801
2.37887 2.77519 2.98518 4.58477 3.61503
0.01477 4.61851 5.34086 0.61958 0.77255 0.39462 1.12067
602 2.79321 4.34802 4.02246 3.44475 3.46792 3.84750 4.14227
1.80566 3.33092 1.86402 3.46044 3.73871 4.22563 3.60898 2.85966
2.56990 2.16073 2.57456 4.96300 3.75937 751 i - - -
2.68618 4.42225 2.77519 2.73123 3.46354 2.40513 3.72494
3.29354 2.67741 2.69355 4.24690 2.90347 2.73739 3.18146 2.89801
2.37887 2.77519 2.98518 4.58477 3.61503
0.01350 4.70791 5.43026 0.61958 0.77255 0.48576 0.95510
603 2.92148 4.74833 3.57514 3.06131 2.63298 3.80954 3.95845
3.30179 3.04117 2.95978 3.87195 1.34675 4.21218 3.35502 3.42269
3.07011 2.49346 3.06200 4.92695 2.70650 752 n - - -
2.68618 4.42225 2.77519 2.73123 3.46354 2.40513 3.72494
3.29354 2.67741 2.69355 4.24690 2.90347 2.73739 3.18146 2.89801
2.37887 2.77519 2.98518 4.58477 3.61503
0.18511 4.70791 1.83283 0.61958 0.77255 0.48576 0.95510
604 2.72558 4.96916 3.06323 2.22017 4.22330 3.53340 3.74437
2.35393 2.53291 3.23580 4.04083 2.57127 3.93134 2.42786 2.99884
2.35805 2.43437 3.31007 5.46519 4.11984 753 e - - -

```
         2.68618  4.42225  2.77519  2.73123  3.46354  2.40513  3.72494
3.29354  2.67741  2.69355  4.24690  2.90347  2.73739  3.18146  2.89801
2.37887  2.77519  2.98518  4.58477  3.61503
         0.01600  4.53881  5.26115  0.61958  0.77255  0.55420  0.85457
    605  2.77881  5.24395  2.97004  2.46064  4.58364  2.84462  3.73648
4.05701  2.12128  3.54984  4.30265  2.52734  2.42817  2.12154  2.95383
2.38970  3.01297  3.63530  5.69167  4.29287    754 k - - -
         2.68618  4.42225  2.77519  2.73123  3.46354  2.40513  3.72494
3.29354  2.67741  2.69355  4.24690  2.90347  2.73739  3.18146  2.89801
2.37887  2.77519  2.98518  4.58477  3.61503
         0.01477  4.61851  5.34086  0.61958  0.77255  0.39462  1.12067
    606  2.85741  4.29037  4.46452  3.87148  3.33234  3.98976  3.07121
1.96456  3.71995  1.37957  2.66698  4.02519  4.34638  3.91584  3.85088
3.28695  2.45814  2.47925  4.90109  3.72305    755 l - - -
         2.68618  4.42225  2.77519  2.73123  3.46354  2.40513  3.72494
3.29354  2.67741  2.69355  4.24690  2.90347  2.73739  3.18146  2.89801
2.37887  2.77519  2.98518  4.58477  3.61503
         0.10856  4.70791  2.36608  0.61958  0.77255  0.48576  0.95510
    607  2.71718  4.90111  3.19251  2.75439  4.56167  3.45192  4.00230
4.01085  2.81655  3.58505  4.37200  1.75774  2.72801  3.15074  3.27221
1.89267  2.03513  3.56267  5.77962  4.44143    756 n - - -
         2.68618  4.42225  2.77519  2.73123  3.46354  2.40513  3.72494
3.29354  2.67741  2.69355  4.24690  2.90347  2.73739  3.18146  2.89801
2.37887  2.77519  2.98518  4.58477  3.61503
         0.01483  4.61419  5.33653  0.61958  0.77255  0.65835  0.72920
    608  2.98171  4.41909  4.46023  3.89926  1.37626  4.08770  4.26076
2.15290  3.76445  1.96332  3.39834  2.76432  4.43811  3.96060  3.91929
3.39007  3.21453  2.52573  4.77716  3.41641    757 f - - -
         2.68618  4.42225  2.77519  2.73123  3.46354  2.40513  3.72494
3.29354  2.67741  2.69355  4.24690  2.90347  2.73739  3.18146  2.89801
2.37887  2.77519  2.98518  4.58477  3.61503
         0.01483  4.61419  5.33653  0.61958  0.77255  0.65835  0.72920
    609  1.98892  4.54115  3.49861  2.93815  3.71646  2.90851  3.93767
2.51359  2.90500  2.78379  3.66303  3.37087  3.03823  3.22047  3.28959
2.46604  2.44577  2.84225  5.13385  2.94722    758 a - - -
         2.68618  4.42225  2.77519  2.73123  3.46354  2.40513  3.72494
3.29354  2.67741  2.69355  4.24690  2.90347  2.73739  3.18146  2.89801
2.37887  2.77519  2.98518  4.58477  3.61503
         0.01483  4.61419  5.33653  0.61958  0.77255  0.65835  0.72920
    610  1.65654  4.68458  3.49923  3.18249  4.75217  3.35932  4.39077
4.20443  3.30145  3.83045  4.62730  2.37874  4.05569  3.59593  3.69679
1.06434  3.10134  3.64648  6.02993  4.75863    759 s - - -
         2.68661  4.42302  2.77437  2.73124  3.46346  2.40590  3.72476
3.29431  2.67746  2.69432  4.24416  2.90279  2.73740  3.18223  2.89877
2.37861  2.77278  2.98595  4.58554  3.61482
         1.01188  1.00934  1.30194  1.93763  0.15554  0.65835  0.72920
    611  2.58463  4.63944  3.18754  2.67014  4.04249  3.40188  3.82025
3.43932  2.66879  3.08457  3.91721  2.45914  2.70531  3.00724  3.10208
2.21731  2.27731  2.47239  5.35816  4.06020    768 s - - -
         2.68618  4.42225  2.77519  2.73123  3.46354  2.40513  3.72494
3.29354  2.67741  2.69355  4.24690  2.90347  2.73739  3.18146  2.89801
2.37887  2.77519  2.98518  4.58477  3.61503
         0.02030  4.30332  5.02566  0.61958  0.77255  0.59681  0.79977
```

```
    612   2.70320  4.67966  2.59175  2.73377  3.90394  3.57508  3.84793
3.24911  2.72776  2.45397  3.80605  3.21106  3.98537  3.06020  3.15505
2.39028  1.86357  2.48849  5.26673  3.98082    769 t - - -
          2.68618  4.42225  2.77519  2.73123  3.46354  2.40513  3.72494
3.29354  2.67741  2.69355  4.24690  2.90347  2.73739  3.18146  2.89801
2.37887  2.77519  2.98518  4.58477  3.61503
          0.11737  4.50914  2.30527  0.61958  0.77255  0.53693  0.87837
    613   2.30707  5.06659  2.54561  2.18937  4.36505  3.50101  3.71240
3.81177  2.48047  3.35932  4.13698  3.00319  2.89263  2.83331  2.96201
2.35221  2.45738  2.74544  5.54895  4.17556    770 e - - -
          2.68618  4.42225  2.77519  2.73123  3.46354  2.40513  3.72494
3.29354  2.67741  2.69355  4.24690  2.90347  2.73739  3.18146  2.89801
2.37887  2.77519  2.98518  4.58477  3.61503
          0.01622  4.52538  5.24773  0.61958  0.77255  0.33512  1.25616
    614   2.37144  5.06315  2.60481  2.55434  3.13303  3.57654  3.77540
3.76494  2.55167  3.33518  3.02731  2.62816  3.06971  2.90575  2.59588
2.12771  2.99756  3.41164  5.54659  4.18857    771 s - - -
          2.68618  4.42225  2.77519  2.73123  3.46354  2.40513  3.72494
3.29354  2.67741  2.69355  4.24690  2.90347  2.73739  3.18146  2.89801
2.37887  2.77519  2.98518  4.58477  3.61503
          0.01350  4.70791  5.43026  0.61958  0.77255  0.48576  0.95510
    615   2.80688  5.26505  3.03118  2.49992  4.59678  3.56727  2.42518
4.06629  2.19695  3.56079  4.31494  1.96978  3.96751  2.45541  2.95469
2.42635  2.52834  3.64991  5.70297  4.31008    772 n - - -
          2.68618  4.42225  2.77519  2.73123  3.46354  2.40513  3.72494
3.29354  2.67741  2.69355  4.24690  2.90347  2.73739  3.18146  2.89801
2.37887  2.77519  2.98518  4.58477  3.61503
          0.01350  4.70791  5.43026  0.61958  0.77255  0.48576  0.95510
    616   2.34661  4.56953  3.54812  2.98548  2.81751  3.72656  3.97324
2.57987  2.51027  2.78424  3.67108  1.80926  4.10998  3.26357  3.32523
2.96489  3.01011  2.44897  5.14647  3.89725    773 n - - -
          2.68618  4.42225  2.77519  2.73123  3.46354  2.40513  3.72494
3.29354  2.67741  2.69355  4.24690  2.90347  2.73739  3.18146  2.89801
2.37887  2.77519  2.98518  4.58477  3.61503
          0.01350  4.70791  5.43026  0.61958  0.77255  0.48576  0.95510
    617   2.77885  4.33748  3.99589  3.42135  3.45810  2.83596  4.12864
2.77997  3.33575  1.92908  2.78095  2.78386  4.21126  3.59612  3.59469
3.10637  2.48635  1.75799  4.95007  3.74487    774 v - - -
          2.68618  4.42225  2.77519  2.73123  3.46354  2.40513  3.72494
3.29354  2.67741  2.69355  4.24690  2.90347  2.73739  3.18146  2.89801
2.37887  2.77519  2.98518  4.58477  3.61503
          0.01350  4.70791  5.43026  0.61958  0.77255  0.48576  0.95510
    618   2.94182  4.66808  3.72929  3.20020  2.16167  1.76996  3.98512
3.19319  2.46864  2.86395  3.78532  3.58396  4.25798  3.44876  3.46671
3.13498  3.17688  2.96969  4.83811  2.17023    775 g - - -
          2.68618  4.42225  2.77519  2.73123  3.46354  2.40513  3.72494
3.29354  2.67741  2.69355  4.24690  2.90347  2.73739  3.18146  2.89801
2.37887  2.77519  2.98518  4.58477  3.61503
          0.01350  4.70791  5.43026  0.61958  0.77255  0.48576  0.95510
    619   3.27722  4.60864  5.08514  4.56687  3.89106  4.58460  5.13616
1.31659  4.44864  2.57670  3.74683  4.71048  2.87039  4.68443  4.60221
3.95138  3.54107  0.98911  5.67915  4.47639    776 v - - -
          2.68618  4.42225  2.77519  2.73123  3.46354  2.40513  3.72494
3.29354  2.67741  2.69355  4.24690  2.90347  2.73739  3.18146  2.89801
2.37887  2.77519  2.98518  4.58477  3.61503
```

0.01350 4.70791 5.43026 0.61958 0.77255 0.48576 0.95510
620 3.04706 5.36200 3.28089 2.75470 4.75636 3.74367 3.87771
4.17065 1.78068 3.65703 4.46196 3.25233 4.15114 1.39639 2.73663
3.02949 2.11862 3.79404 5.77172 4.47008 777 q - - -
2.68618 4.42225 2.77519 2.73123 3.46354 2.40513 3.72494
3.29354 2.67741 2.69355 4.24690 2.90347 2.73739 3.18146 2.89801
2.37887 2.77519 2.98518 4.58477 3.61503
0.01350 4.70791 5.43026 0.61958 0.77255 0.48576 0.95510
621 3.98868 5.60669 4.81325 4.83535 5.82610 0.12867 5.81900
5.70431 5.11516 5.20759 6.24044 4.98127 5.00675 5.37566 5.18924
4.19341 4.52384 5.07668 6.63103 5.97905 778 G - - -
2.68618 4.42225 2.77519 2.73123 3.46354 2.40513 3.72494
3.29354 2.67741 2.69355 4.24690 2.90347 2.73739 3.18146 2.89801
2.37887 2.77519 2.98518 4.58477 3.61503
0.01350 4.70791 5.43026 0.61958 0.77255 0.48576 0.95510
622 2.39343 5.23567 3.02708 1.78061 4.55407 3.56618 3.76607
3.08470 1.91572 3.52737 4.28889 2.22417 3.96872 2.87576 2.98292
2.77805 3.03507 3.61139 5.68422 4.29481 779 e - - -
2.68618 4.42225 2.77519 2.73123 3.46354 2.40513 3.72494
3.29354 2.67741 2.69355 4.24690 2.90347 2.73739 3.18146 2.89801
2.37887 2.77519 2.98518 4.58477 3.61503
0.01350 4.70791 5.43026 0.61958 0.77255 0.48576 0.95510
623 2.82675 4.72875 3.42286 2.89607 3.85969 3.71152 2.86221
2.56799 2.85083 2.95393 3.83868 1.47540 4.11351 3.20427 3.24549
2.96207 3.06543 2.45129 5.26216 3.96273 780 n - - -
2.68618 4.42225 2.77519 2.73123 3.46354 2.40513 3.72494
3.29354 2.67741 2.69355 4.24690 2.90347 2.73739 3.18146 2.89801
2.37887 2.77519 2.98518 4.58477 3.61503
0.01350 4.70791 5.43026 0.61958 0.77255 0.48576 0.95510
624 3.07487 5.07984 3.36138 3.33869 5.23285 0.53940 4.78532
4.86179 3.83460 4.45705 5.30831 2.31810 4.35885 4.05107 4.21160
3.19054 3.56788 4.21199 6.45628 5.20299 781 G - - -
2.68618 4.42225 2.77519 2.73123 3.46354 2.40513 3.72494
3.29354 2.67741 2.69355 4.24690 2.90347 2.73739 3.18146 2.89801
2.37887 2.77519 2.98518 4.58477 3.61503
0.01350 4.70791 5.43026 0.61958 0.77255 0.48576 0.95510
625 2.80737 5.24945 3.02244 2.15883 4.57553 3.56565 3.76758
4.04124 1.77397 3.54607 4.30492 2.25797 3.97014 2.87598 2.98026
2.06920 3.03983 2.85713 5.69736 4.30580 782 k - - -
2.68623 4.42230 2.77525 2.73128 3.46359 2.40518 3.72500
3.29359 2.67746 2.69360 4.24695 2.90302 2.73745 3.18088 2.89806
2.37892 2.77525 2.98524 4.58482 3.61508
0.20877 1.69263 5.43026 0.17539 1.82716 0.48576 0.95510
626 4.62705 5.77367 5.15902 5.08940 2.79635 4.87159 4.31504
4.56637 4.94449 3.79323 5.15160 4.87732 5.34883 5.02217 4.92947
4.65395 4.92861 4.47859 4.45019 0.24676 784 Y - - -
2.68618 4.42225 2.77519 2.73123 3.46354 2.40513 3.72494
3.29354 2.67741 2.69355 4.24690 2.90347 2.73739 3.18146 2.89801
2.37887 2.77519 2.98518 4.58477 3.61503
0.01350 4.70791 5.43026 0.61958 0.77255 0.48576 0.95510
627 2.79424 4.39840 2.85627 3.31745 3.53157 3.82871 4.11233
2.40548 3.25126 2.59560 2.73110 3.65995 4.20612 3.52765 3.54860
2.57557 2.52122 1.54867 5.01631 3.80129 785 v - - -

```
         2.68618  4.42225  2.77519  2.73123  3.46354  2.40513  3.72494
3.29354  2.67741  2.69355  4.24690  2.90347  2.73739  3.18146  2.89801
2.37887  2.77519  2.98518  4.58477  3.61503
         0.01350  4.70791  5.43026  0.61958  0.77255  0.48576  0.95510
    628  3.72545  4.94720  5.69747  5.16121  3.64311  5.18753  5.64038
1.80036  5.04705  0.82282  3.39588  5.33208  5.32525  5.08291  5.08933
4.58393  3.95761  1.52832  5.79491  4.74508    786 l - - -
         2.68618  4.42225  2.77519  2.73123  3.46354  2.40513  3.72494
3.29354  2.67741  2.69355  4.24690  2.90347  2.73739  3.18146  2.89801
2.37887  2.77519  2.98518  4.58477  3.61503
         0.01350  4.70791  5.43026  0.61958  0.77255  0.48576  0.95510
    629  2.77450  4.41306  3.79856  3.22706  2.75484  3.78987  4.05398
2.88995  2.26926  2.26085  2.82263  2.37109  4.16612  3.44857  3.47651
3.04613  3.00623  2.27284  4.99891  2.83375    787 l - - -
         2.68618  4.42225  2.77519  2.73123  3.46354  2.40513  3.72494
3.29354  2.67741  2.69355  4.24690  2.90347  2.73739  3.18146  2.89801
2.37887  2.77519  2.98518  4.58477  3.61503
         0.01350  4.70791  5.43026  0.61958  0.77255  0.48576  0.95510
    630  2.78056  5.14458  2.23177  2.52759  4.45214  3.56138  3.77976
3.05100  2.54790  3.44469  4.22002  2.60106  2.98487  2.89942  3.03098
2.12979  2.15782  3.51955  5.62992  4.25323    788 s - - -
         2.68618  4.42225  2.77519  2.73123  3.46354  2.40513  3.72494
3.29354  2.67741  2.69355  4.24690  2.90347  2.73739  3.18146  2.89801
2.37887  2.77519  2.98518  4.58477  3.61503
         0.01350  4.70791  5.43026  0.61958  0.77255  0.48576  0.95510
    631  3.11164  4.47270  4.89260  4.32325  3.57097  2.84378  4.73959
1.28442  4.17399  1.51583  3.48760  4.44108  4.68016  4.35749  4.28043
3.67183  3.35385  1.85936  5.26237  4.08774    789 i - - -
         2.68618  4.42225  2.77519  2.73123  3.46354  2.40513  3.72494
3.29354  2.67741  2.69355  4.24690  2.90347  2.73739  3.18146  2.89801
2.37887  2.77519  2.98518  4.58477  3.61503
         0.01350  4.70791  5.43026  0.61958  0.77255  0.48576  0.95510
    632  2.38672  5.16116  3.06034  2.17586  4.46274  2.81153  3.75405
3.91779  2.22964  3.44894  4.21665  3.04749  3.00682  2.86783  2.99345
2.36563  2.29776  3.52887  5.62427  3.17670    790 e - - -
         2.68618  4.42225  2.77519  2.73123  3.46354  2.40513  3.72494
3.29354  2.67741  2.69355  4.24690  2.90347  2.73739  3.18146  2.89801
2.37887  2.77519  2.98518  4.58477  3.61503
         0.29905  4.70791  1.38849  0.61958  0.77255  0.48576  0.95510
    633  2.80375  5.08467  1.52857  2.50701  4.63259  3.39868  3.93050
4.08642  2.81702  3.65452  4.45972  3.03152  3.96439  3.08431  3.32092
2.19906  1.84753  3.65329  5.84555  4.45386    791 d - - -
         2.68618  4.42225  2.77519  2.73123  3.46354  2.40513  3.72494
3.29354  2.67741  2.69355  4.24690  2.90347  2.73739  3.18146  2.89801
2.37887  2.77519  2.98518  4.58477  3.61503
         0.01792  4.42678  5.14913  0.61958  0.77255  0.29277  1.37118
    634  1.89137  4.95339  2.44464  2.78030  4.58335  3.50207  4.03558
4.02516  2.86066  3.60657  4.39573  3.25280  4.03979  3.18422  3.32529
1.70681  1.81103  3.58786  5.80882  4.46768    792 s - - -
         2.68618  4.42225  2.77519  2.73123  3.46354  2.40513  3.72494
3.29354  2.67741  2.69355  4.24690  2.90347  2.73739  3.18146  2.89801
2.37887  2.77519  2.98518  4.58477  3.61503
         0.01350  4.70791  5.43026  0.61958  0.77255  0.48576  0.95510
```

635 2.77630 4.68954 2.30503 2.39525 3.86195 3.68175 3.91359
2.29583 2.82117 2.91448 3.78235 3.30952 4.06823 3.14889 3.23672
2.90884 2.53961 2.23701 5.24728 2.93868 793 v - - -
2.68618 4.42225 2.77519 2.73123 3.46354 2.40513 3.72494
3.29354 2.67741 2.69355 4.24690 2.90347 2.73739 3.18146 2.89801
2.37887 2.77519 2.98518 4.58477 3.61503
0.01350 4.70791 5.43026 0.61958 0.77255 0.48576 0.95510
636 2.90759 5.37103 2.88604 1.72807 4.69128 3.57681 2.83252
4.16868 2.62410 3.66986 4.43946 1.85093 4.02604 2.96289 3.11527
2.86568 2.12343 3.75476 5.82025 4.41072 794 e - - -
2.68663 4.42270 2.77522 2.73086 3.46150 2.40558 3.72540
3.29324 2.67745 2.69400 4.24735 2.90245 2.73785 3.18191 2.89846
2.37904 2.77395 2.98511 4.58522 3.61548
0.31766 1.31762 5.43026 1.61971 0.22059 0.48576 0.95510
637 3.21737 4.56010 5.00781 4.43779 3.56667 4.47416 4.86126
1.26999 4.29172 1.43313 3.44591 4.56729 2.89273 4.45667 4.39216
3.80601 3.45549 1.86421 5.33932 4.18536 803 i - - -
2.68618 4.42225 2.77519 2.73123 3.46354 2.40513 3.72494
3.29354 2.67741 2.69355 4.24690 2.90347 2.73739 3.18146 2.89801
2.37887 2.77519 2.98518 4.58477 3.61503
0.01350 4.70791 5.43026 0.61958 0.77255 0.48576 0.95510
638 2.76604 4.84240 3.30735 2.80060 4.22523 3.57508 3.96528
3.62972 2.80211 2.61469 4.09531 2.56909 1.65066 3.14639 3.23732
2.31297 2.43242 3.29665 5.53096 4.22637 804 p - - -
2.68618 4.42225 2.77519 2.73123 3.46354 2.40513 3.72494
3.29354 2.67741 2.69355 4.24690 2.90347 2.73739 3.18146 2.89801
2.37887 2.77519 2.98518 4.58477 3.61503
0.01350 4.70791 5.43026 0.61958 0.77255 0.48576 0.95510
639 1.82150 4.96180 3.15724 2.24085 4.24265 3.59681 3.88312
3.66630 2.70570 3.28067 4.10330 3.18007 4.02989 3.04724 3.16171
1.67645 3.05591 3.33957 5.52570 2.98243 805 s - - -
2.68618 4.42225 2.77519 2.73123 3.46354 2.40513 3.72494
3.29354 2.67741 2.69355 4.24690 2.90347 2.73739 3.18146 2.89801
2.37887 2.77519 2.98518 4.58477 3.61503
0.01350 4.70791 5.43026 0.61958 0.77255 0.48576 0.95510
640 2.89801 4.96222 3.37501 3.06965 4.78656 0.91796 4.27917
4.20042 2.27409 3.80531 4.63418 3.47097 4.18515 3.46473 3.35432
2.99220 2.43006 3.74207 5.99311 4.72050 806 g - - -
2.68618 4.42225 2.77519 2.73123 3.46354 2.40513 3.72494
3.29354 2.67741 2.69355 4.24690 2.90347 2.73739 3.18146 2.89801
2.37887 2.77519 2.98518 4.58477 3.61503
0.01350 4.70791 5.43026 0.61958 0.77255 0.48576 0.95510
641 2.79245 5.22874 3.02811 2.50255 4.56592 2.85836 3.76729
4.03320 2.21777 3.53984 4.29711 2.51390 3.96293 2.48058 2.99293
1.86715 2.19860 3.62066 5.69307 4.30054 807 s - - -
2.68618 4.42225 2.77519 2.73123 3.46354 2.40513 3.72494
3.29354 2.67741 2.69355 4.24690 2.90347 2.73739 3.18146 2.89801
2.37887 2.77519 2.98518 4.58477 3.61503
0.01350 4.70791 5.43026 0.61958 0.77255 0.48576 0.95510
642 2.77696 4.42687 2.91964 3.20647 2.43837 3.78611 2.92961
2.10432 3.14807 2.63115 3.53343 3.57492 4.16297 2.78606 3.46814
3.04077 3.00873 2.28362 4.99831 2.81899 808 i - - -
2.68618 4.42225 2.77519 2.73123 3.46354 2.40513 3.72494
3.29354 2.67741 2.69355 4.24690 2.90347 2.73739 3.18146 2.89801
2.37887 2.77519 2.98518 4.58477 3.61503

```
          0.01350  4.70791  5.43026  0.61958  0.77255  0.48576  0.95510
    643   2.77589  4.91029  3.20416  2.28373  4.12684  3.61697  3.01123
2.85337  2.64510  3.16047  3.98728  2.62256  4.00819  2.99399  3.09755
2.82878  1.74530  3.23248  5.42230  3.16010    809 t - - -
          2.68618  4.42225  2.77519  2.73123  3.46354  2.40513  3.72494
3.29354  2.67741  2.69355  4.24690  2.90347  2.73739  3.18146  2.89801
2.37887  2.77519  2.98518  4.58477  3.61503
          0.01350  4.70791  5.43026  0.61958  0.77255  0.48576  0.95510
    644   3.18722  4.50755  5.10201  4.52607  3.57044  4.47135  4.86265
1.63623  4.36750  1.62754  2.55548  4.60123  4.77825  4.51328  4.42932
3.80568  2.59686  1.41358  5.31234  4.15257    810 v - - -
          2.68618  4.42225  2.77519  2.73123  3.46354  2.40513  3.72494
3.29354  2.67741  2.69355  4.24690  2.90347  2.73739  3.18146  2.89801
2.37887  2.77519  2.98518  4.58477  3.61503
          0.01350  4.70791  5.43026  0.61958  0.77255  0.48576  0.95510
    645   2.78304  4.93617  3.19340  2.27595  2.55947  3.61717  2.54402
3.57137  2.63428  2.32973  4.01113  2.60038  4.00894  2.48073  3.08691
2.82948  3.01425  3.26181  5.43061  4.09775    811 e - - -
          2.68618  4.42225  2.77519  2.73123  3.46354  2.40513  3.72494
3.29354  2.67741  2.69355  4.24690  2.90347  2.73739  3.18146  2.89801
2.37887  2.77519  2.98518  4.58477  3.61503
          0.01350  4.70791  5.43026  0.61958  0.77255  0.48576  0.95510
    646   3.00422  4.38417  4.75107  4.16320  2.67634  4.20050  4.54022
1.23190  2.87386  1.96638  3.39457  4.27829  4.53871  4.18049  4.10252
3.51291  3.23796  1.92694  5.07565  3.89861    812 i - - -
          2.68618  4.42225  2.77519  2.73123  3.46354  2.40513  3.72494
3.29354  2.67741  2.69355  4.24690  2.90347  2.73739  3.18146  2.89801
2.37887  2.77519  2.98518  4.58477  3.61503
          0.01350  4.70791  5.43026  0.61958  0.77255  0.48576  0.95510
    647   2.82469  5.15892  3.13470  2.22046  4.45485  3.60934  3.78562
3.89114  2.49441  2.78184  4.21918  3.10695  4.00114  2.14618  2.42887
2.82020  1.79021  3.52381  5.61443  4.26042    813 t - - -
          2.68618  4.42225  2.77519  2.73123  3.46354  2.40513  3.72494
3.29354  2.67741  2.69355  4.24690  2.90347  2.73739  3.18146  2.89801
2.37887  2.77519  2.98518  4.58477  3.61503
          0.01350  4.70791  5.43026  0.61958  0.77255  0.48576  0.95510
    648   2.82885  4.95798  3.20051  2.72100  4.23634  3.61590  2.82781
3.66258  2.71887  3.28903  4.12084  1.39214  4.05736  3.08499  3.15197
2.88355  2.53106  2.61936  5.52703  4.19724    814 n - - -
          2.68618  4.42225  2.77519  2.73123  3.46354  2.40513  3.72494
3.29354  2.67741  2.69355  4.24690  2.90347  2.73739  3.18146  2.89801
2.37887  2.77519  2.98518  4.58477  3.61503
          0.01350  4.70791  5.43026  0.61958  0.77255  0.48576  0.95510
    649   2.79755  5.05565  3.11478  2.59750  4.34392  2.85121  3.82264
2.84048  2.60278  3.35220  4.15360  1.74603  3.99991  2.19750  3.06548
2.81514  2.57058  3.42146  5.57169  4.22259    815 n - - -
          2.68618  4.42225  2.77519  2.73123  3.46354  2.40513  3.72494
3.29354  2.67741  2.69355  4.24690  2.90347  2.73739  3.18146  2.89801
2.37887  2.77519  2.98518  4.58477  3.61503
          0.01350  4.70791  5.43026  0.61958  0.77255  0.48576  0.95510
    650   2.82244  5.16596  2.45078  2.55149  4.50249  1.87251  3.83798
3.95345  2.62930  3.50167  2.97218  2.57906  4.00030  2.96534  3.11588
2.03812  3.07151  3.56807  5.69166  4.31385    816 g - - -
```

```
          2.68618  4.42225  2.77519  2.73123  3.46354  2.40513  3.72494
3.29354  2.67741  2.69355  4.24690  2.90347  2.73739  3.18146  2.89801
2.37887  2.77519  2.98518  4.58477  3.61503
          0.01350  4.70791  5.43026  0.61958  0.77255  0.48576  0.95510
    651   2.93496  5.47098  2.31453  2.07028  4.80106  3.56534  3.85565
4.29627  2.20205  3.77031  4.52824  2.10360  4.02937  2.96881  3.17117
1.65097  3.17822  3.85602  5.90420  4.46855    817 s - - -
          2.68618  4.42225  2.77519  2.73123  3.46354  2.40513  3.72494
3.29354  2.67741  2.69355  4.24690  2.90347  2.73739  3.18146  2.89801
2.37887  2.77519  2.98518  4.58477  3.61503
          0.01350  4.70791  5.43026  0.61958  0.77255  0.48576  0.95510
    652   2.80080  5.29915  2.49261  2.46776  4.63726  3.54824  3.75613
4.11985  2.15839  3.60176  4.34596  2.50018  3.95600  2.17839  2.99562
1.91586  2.52827  3.68694  5.73620  4.32585    818 s - - -
          2.68618  4.42225  2.77519  2.73123  3.46354  2.40513  3.72494
3.29354  2.67741  2.69355  4.24690  2.90347  2.73739  3.18146  2.89801
2.37887  2.77519  2.98518  4.58477  3.61503
          0.01350  4.70791  5.43026  0.61958  0.77255  0.48576  0.95510
    653   2.43250  5.36652  1.90812  2.46941  4.70547  2.72678  3.80817
4.19225  2.23186  3.67504  4.42694  2.11061  2.92451  2.91633  3.08840
2.81575  3.10128  3.75765  5.81273  4.39225    819 d - - -
          2.68618  4.42225  2.77519  2.73123  3.46354  2.40513  3.72494
3.29354  2.67741  2.69355  4.24690  2.90347  2.73739  3.18146  2.89801
2.37887  2.77519  2.98518  4.58477  3.61503
          0.01350  4.70791  5.43026  0.61958  0.77255  0.48576  0.95510
    654   3.64714  4.88791  5.62528  5.06708  3.57073  5.05449  5.46038
1.19331  4.93767  1.23469  2.39382  5.20378  5.21601  4.95972  4.95662
4.42997  3.87432  1.87099  5.65561  4.60512    820 i - - -
          2.68618  4.42225  2.77519  2.73123  3.46354  2.40513  3.72494
3.29354  2.67741  2.69355  4.24690  2.90347  2.73739  3.18146  2.89801
2.37887  2.77519  2.98518  4.58477  3.61503
          0.01350  4.70791  5.43026  0.61958  0.77255  0.48576  0.95510
    655   3.23361  4.57493  4.96968  4.43354  1.25980  4.35812  4.27955
2.19034  4.25041  2.41887  3.54305  4.38804  4.68553  4.33106  4.27738
3.68147  3.46265  2.10662  4.57710  1.96710    821 f - - -
          2.68618  4.42225  2.77519  2.73123  3.46354  2.40513  3.72494
3.29354  2.67741  2.69355  4.24690  2.90347  2.73739  3.18146  2.89801
2.37887  2.77519  2.98518  4.58477  3.61503
          0.01350  4.70791  5.43026  0.61958  0.77255  0.48576  0.95510
    656   3.99460  5.19534  5.87063  5.33498  3.47790  5.41443  5.75827
1.62743  5.18073  0.58404  3.22591  5.56316  5.44531  5.11323  5.16811
4.84243  4.21035  2.57645  5.74101  4.75006    822 L - - -
          2.68618  4.42225  2.77519  2.73123  3.46354  2.40513  3.72494
3.29354  2.67741  2.69355  4.24690  2.90347  2.73739  3.18146  2.89801
2.37887  2.77519  2.98518  4.58477  3.61503
          0.01350  4.70791  5.43026  0.61958  0.77255  0.48576  0.95510
    657   4.14564  6.17171  0.22568  3.25148  5.72363  4.09097  4.97558
5.55962  4.24873  5.02011  6.08237  3.82748  4.79485  4.26004  4.82119
4.05906  4.51846  5.12596  6.68028  5.54848    823 D - - -
          2.68618  4.42225  2.77519  2.73123  3.46354  2.40513  3.72494
3.29354  2.67741  2.69355  4.24690  2.90347  2.73739  3.18146  2.89801
2.37887  2.77519  2.98518  4.58477  3.61503
          0.01350  4.70791  5.43026  0.61958  0.77255  0.48576  0.95510
```

```
    658   4.23731  5.86418  4.82648  4.24561  5.45647  4.45840  4.80535
5.13616  3.05989  4.49477  5.52683  4.51437  4.97129  4.03592  0.23170
4.32043  4.47918  4.83283  6.22544  5.27786    824 R - - -
         2.68618  4.42225  2.77519  2.73123  3.46354  2.40513  3.72494
3.29354  2.67741  2.69355  4.24690  2.90347  2.73739  3.18146  2.89801
2.37887  2.77519  2.98518  4.58477  3.61503
         0.01350  4.70791  5.43026  0.61958  0.77255  0.48576  0.95510
    659   3.70510  4.94333  5.61859  5.07953  2.46184  5.08942  5.36460
0.77700  4.94665  1.65416  3.35944  5.20585  5.24665  4.96332  4.97024
4.47021  3.93235  2.33733  5.53294  4.33222    825 i - - -
         2.68618  4.42225  2.77519  2.73123  3.46354  2.40513  3.72494
3.29354  2.67741  2.69355  4.24690  2.90347  2.73739  3.18146  2.89801
2.37887  2.77519  2.98518  4.58477  3.61503
         0.01350  4.70791  5.43026  0.61958  0.77255  0.48576  0.95510
    660   4.02385  6.06573  3.23490  0.26426  5.57841  4.08320  4.84596
5.24987  3.87442  4.74179  5.76500  3.80524  4.74659  4.09772  4.31868
3.95941  4.36027  4.86907  6.55423  5.40950    826 E - - -
         2.68618  4.42225  2.77519  2.73123  3.46354  2.40513  3.72494
3.29354  2.67741  2.69355  4.24690  2.90347  2.73739  3.18146  2.89801
2.37887  2.77519  2.98518  4.58477  3.61503
         0.01350  4.70791  5.43026  0.61958  0.77255  0.48576  0.95510
    661   4.52873  5.69215  5.41913  5.33881  0.26038  4.88194  4.53878
4.05250  5.25383  3.24788  4.67964  5.07809  5.36224  5.20709  5.16724
4.70684  4.84949  4.09132  4.65325  2.96389    827 F - - -
         2.68618  4.42225  2.77519  2.73123  3.46354  2.40513  3.72494
3.29354  2.67741  2.69355  4.24690  2.90347  2.73739  3.18146  2.89801
2.37887  2.77519  2.98518  4.58477  3.61503
         0.01350  4.70791  5.43026  0.61958  0.77255  0.48576  0.95510
    662   3.26127  4.56862  5.13951  4.59208  3.65682  4.57757  4.96209
1.06937  4.44376  2.45148  3.62009  4.68599  4.88711  4.62890  4.53953
3.92478  3.50507  1.32430  5.40927  2.70585    828 i - - -
         2.68618  4.42225  2.77519  2.73123  3.46354  2.40513  3.72494
3.29354  2.67741  2.69355  4.24690  2.90347  2.73739  3.18146  2.89801
2.37887  2.77519  2.98518  4.58477  3.61503
         0.01350  4.70791  5.43026  0.61958  0.77255  0.48576  0.95510
    663   2.96097  5.07771  3.28021  2.95879  4.77913  3.60690  4.17617
4.21607  2.17490  3.78490  4.61267  3.39958  1.00627  3.34622  3.20105
2.36368  3.30521  3.78109  5.94775  4.65551    829 p - - -
         2.68618  4.42225  2.77519  2.73123  3.46354  2.40513  3.72494
3.29354  2.67741  2.69355  4.24690  2.90347  2.73739  3.18146  2.89801
2.37887  2.77519  2.98518  4.58477  3.61503
         0.01350  4.70791  5.43026  0.61958  0.77255  0.48576  0.95510
    664   2.77442  4.41751  3.79000  3.21823  2.77952  3.78761  4.05160
2.41342  2.23872  2.02418  3.52212  3.58306  4.16394  2.68288  3.46705
3.04335  2.52015  2.68249  5.00607  2.86559    830 l - - -
         2.68618  4.42225  2.77519  2.73123  3.46354  2.40513  3.72494
3.29354  2.67741  2.69355  4.24690  2.90347  2.73739  3.18146  2.89801
2.37887  2.77519  2.98518  4.58477  3.61503
         0.01350  4.70791  5.43026  0.61958  0.77255  0.48576  0.95510
    665   2.36040  4.89287  2.29830  2.66198  4.12022  3.61255  3.83106
2.89473  2.65394  3.15092  3.05287  3.17054  2.56731  2.99885  3.10791
2.41048  2.48649  3.21858  5.41872  4.10051    831 d - - -
         2.68618  4.42225  2.77519  2.73123  3.46354  2.40513  3.72494
3.29354  2.67741  2.69355  4.24690  2.90347  2.73739  3.18146  2.89801
2.37887  2.77519  2.98518  4.58477  3.61503
```

```
          0.01350  4.70791  5.43026  0.61958  0.77255  0.48576  0.95510
    666   2.77347  4.93029  3.19530  2.64273  4.16194  3.60985  3.82172
2.78709  2.63024  2.63585  4.00983  2.60173  2.54686  2.26385  3.08480
2.12349  3.00620  3.25919  5.44496  4.12030    832 s - - -
          2.68618  4.42225  2.77519  2.73123  3.46354  2.40513  3.72494
3.29354  2.67741  2.69355  4.24690  2.90347  2.73739  3.18146  2.89801
2.37887  2.77519  2.98518  4.58477  3.61503
          0.01350  4.70791  5.43026  0.61958  0.77255  0.48576  0.95510
    667   2.76708  4.85584  2.62603  2.68418  3.15018  3.62473  3.83797
3.46491  2.67296  2.55377  3.93616  3.18828  2.58268  2.52116  3.12135
2.40727  2.56745  2.65471  5.38061  4.07085    833 s - - -
          2.68618  4.42225  2.77519  2.73123  3.46354  2.40513  3.72494
3.29354  2.67741  2.69355  4.24690  2.90347  2.73739  3.18146  2.89801
2.37887  2.77519  2.98518  4.58477  3.61503
          0.01350  4.70791  5.43026  0.61958  0.77255  0.48576  0.95510
    668   2.41190  4.96365  3.17541  2.61778  4.20226  3.60201  3.80616
2.84830  2.25281  2.66196  4.03657  3.13573  3.06692  2.20829  3.06228
2.12020  3.00212  3.29567  5.46804  4.13558    834 s - - -
          2.68618  4.42225  2.77519  2.73123  3.46354  2.40513  3.72494
3.29354  2.67741  2.69355  4.24690  2.90347  2.73739  3.18146  2.89801
2.37887  2.77519  2.98518  4.58477  3.61503
          0.01350  4.70791  5.43026  0.61958  0.77255  0.48576  0.95510
    669   2.14493  5.04452  2.57515  2.56475  3.25664  2.84025  3.78127
2.93193  2.22865  3.31384  4.10953  2.55370  3.97248  2.91545  3.03525
2.78064  2.57629  3.38947  5.53226  4.17884    835 a - - -
          2.68618  4.42225  2.77519  2.73123  3.46354  2.40513  3.72494
3.29354  2.67741  2.69355  4.24690  2.90347  2.73739  3.18146  2.89801
2.37887  2.77519  2.98518  4.58477  3.61503
          0.01350  4.70791  5.43026  0.61958  0.77255  0.48576  0.95510
    670   2.76841  3.16973  3.95194  3.37961  2.70963  3.80535  4.11212
2.83238  3.30099  2.56683  3.47621  3.68781  2.21959  3.56502  3.57238
2.48674  2.09824  2.62378  4.95918  2.84953    836 t - - -
          2.68618  4.42225  2.77519  2.73123  3.46354  2.40513  3.72494
3.29354  2.67741  2.69355  4.24690  2.90347  2.73739  3.18146  2.89801
2.37887  2.77519  2.98518  4.58477  3.61503
          0.01350  4.70791  5.43026  0.61958  0.77255  0.48576  0.95510
    671   2.77065  5.17138  2.63205  2.50528  4.47439  3.55846  2.94412
3.93104  2.19101  2.78440  4.22503  3.04530  2.97793  2.86604  2.99059
2.34570  2.04464  3.53958  5.63121  4.24792    837 t - - -
          2.68618  4.42225  2.77519  2.73123  3.46354  2.40513  3.72494
3.29354  2.67741  2.69355  4.24690  2.90347  2.73739  3.18146  2.89801
2.37887  2.77519  2.98518  4.58477  3.61503
          0.01350  4.70791  5.43026  0.61958  0.77255  0.48576  0.95510
    672   2.77361  5.05939  3.12275  2.56636  4.32562  2.85682  3.78088
2.88148  2.55125  2.70904  4.12386  2.31881  3.97558  2.17755  2.52974
2.42874  3.00461  3.40732  5.54295  4.18930    838 q - - -
          2.68618  4.42225  2.77519  2.73123  3.46354  2.40513  3.72494
3.29354  2.67741  2.69355  4.24690  2.90347  2.73739  3.18146  2.89801
2.37887  2.77519  2.98518  4.58477  3.61503
          0.10420  4.70791  2.40875  0.61958  0.77255  0.48576  0.95510
    673   2.78359  5.15788  2.09274  2.49266  4.47346  3.52983  3.77663
3.92662  2.55762  2.68124  4.24426  2.21942  3.95437  2.89871  3.04557
2.06354  2.54270  3.53951  5.64937  4.26588    839 s - - -
```

```
          2.68618  4.42225  2.77519  2.73123  3.46354  2.40513  3.72494
3.29354  2.67741  2.69355  4.24690  2.90347  2.73739  3.18146  2.89801
2.37887  2.77519  2.98518  4.58477  3.61503
          0.01477  4.61848  5.34083  0.61958  0.77255  0.65138  0.73674
    674   2.74009  4.60217  3.42801  2.87631  3.77158  3.65598  3.91340
2.52507  2.84805  2.84099  3.71488  2.35875  4.05180  3.17354  3.24579
2.38732  1.91095  2.89903  5.17460  2.92285    840 t - - -
          2.68618  4.42225  2.77519  2.73123  3.46354  2.40513  3.72494
3.29354  2.67741  2.69355  4.24690  2.90347  2.73739  3.18146  2.89801
2.37887  2.77519  2.98518  4.58477  3.61503
          0.01477  4.61848  5.34083  0.61958  0.77255  0.65138  0.73674
    675   2.74326  4.58389  3.44942  2.88862  2.93134  3.67409  3.90999
2.53846  2.85461  2.41748  3.67698  2.30819  2.41444  2.63918  3.24811
2.90680  2.97513  2.86528  5.14262  3.88092    841 n - - -
          2.68618  4.42225  2.77519  2.73123  3.46354  2.40513  3.72494
3.29354  2.67741  2.69355  4.24690  2.90347  2.73739  3.18146  2.89801
2.37887  2.77519  2.98518  4.58477  3.61503
          0.01477  4.61848  5.34083  0.61958  0.77255  0.65138  0.73674
    676   2.74084  4.94142  3.13438  2.60091  4.23114  2.73162  2.98775
3.65168  2.60383  3.25126  4.06145  2.58424  2.12835  2.95395  3.06490
2.77707  2.48214  2.72183  5.48895  4.15293    842 p - - -
          2.68636  4.42244  2.77500  2.73142  3.46372  2.40505  3.72513
3.29311  2.67759  2.69373  4.24708  2.90236  2.73723  3.18165  2.89819
2.37905  2.77538  2.98537  4.58495  3.61436
          0.58783  1.73824  1.31439  1.24314  0.34035  0.65138  0.73674
    677   2.64279  3.18841  3.16705  2.18660  3.86509  2.64414  3.76021
2.62316  2.62517  2.91036  3.76142  2.53590  3.91078  2.96385  3.05551
2.74041  2.88369  2.96622  5.21289  3.92156    847 e - - -
          2.68618  4.42225  2.77519  2.73123  3.46354  2.40513  3.72494
3.29354  2.67741  2.69355  4.24690  2.90347  2.73739  3.18146  2.89801
2.37887  2.77519  2.98518  4.58477  3.61503
          0.02011  4.31217  5.03451  0.61958  0.77255  1.00938  0.45325
    678   2.69236  5.01583  2.40315  2.01899  4.30841  2.67911  3.69251
3.74266  2.49172  3.31068  4.10393  2.94503  3.86463  2.82536  2.97706
2.68909  2.42844  2.57452  5.51646  4.14456    848 e - - -
          2.68618  4.42225  2.77519  2.73123  3.46354  2.40513  3.72494
3.29354  2.67741  2.69355  4.24690  2.90347  2.73739  3.18146  2.89801
2.37887  2.77519  2.98518  4.58477  3.61503
          0.02011  4.31217  5.03451  0.61958  0.77255  1.00938  0.45325
    679   2.63896  3.14235  3.31286  2.76046  3.69564  3.54428  2.75624
3.08547  2.71835  2.25787  3.63995  2.57849  3.94440  3.05614  3.11474
2.34352  2.87955  2.83054  5.09054  3.81930    849 l - - -
          2.68618  4.42225  2.77519  2.73123  3.46354  2.40513  3.72494
3.29354  2.67741  2.69355  4.24690  2.90347  2.73739  3.18146  2.89801
2.37887  2.77519  2.98518  4.58477  3.61503
          0.02011  4.31217  5.03451  0.61958  0.77255  1.00938  0.45325
    680   2.87682  5.27140  2.86075  1.97737  4.62766  3.51055  3.71971
4.07474  2.34698  3.55706  4.35352  1.97974  3.95427  2.84353  1.80697
2.83686  3.10456  3.68128  5.67477  4.32505    850 r - - -
          2.68618  4.42225  2.77519  2.73123  3.46354  2.40513  3.72494
3.29354  2.67741  2.69355  4.24690  2.90347  2.73739  3.18146  2.89801
2.37887  2.77519  2.98518  4.58477  3.61503
          0.02011  4.31217  5.03451  0.61958  0.77255  0.57526  0.82681
```

681   2.79139 5.15172 2.89722 1.85166 4.45573 3.50809 3.77059
3.90707 2.55539 2.72520 4.24097 2.15062 3.94605 2.89922 3.03546
1.93547 3.03373 3.52805 5.63901 4.25752   851 e - - -
2.68618 4.42225 2.77519 2.73123 3.46354 2.40513 3.72494
3.29354 2.67741 2.69355 4.24690 2.90347 2.73739 3.18146 2.89801
2.37887 2.77519 2.98518 4.58477 3.61503
0.01624 4.52401 5.24636 0.61958 0.77255 0.53181 0.88562
682   2.76044 4.53827 3.86182 3.38765 3.95552 3.62913 4.32120
2.32174 3.31608 2.93155 3.89793 3.68201 1.39652 3.64355 3.63175
2.99141 1.89682 2.82061 5.43809 4.21169   852 p - - -
2.68618 4.42225 2.77519 2.73123 3.46354 2.40513 3.72494
3.29354 2.67741 2.69355 4.24690 2.90347 2.73739 3.18146 2.89801
2.37887 2.77519 2.98518 4.58477 3.61503
0.01472 4.62188 5.34423 0.61958 0.77255 0.64581 0.74283
683   2.74022 4.87989 3.17094 2.29645 4.10259 3.58225 2.98316
2.34306 2.61126 3.13175 3.95699 2.57665 3.03550 2.95806 3.06635
2.34752 2.97205 2.70137 5.39525 4.07405   853 e - - -
2.68618 4.42225 2.77519 2.73123 3.46354 2.40513 3.72494
3.29354 2.67741 2.69355 4.24690 2.90347 2.73739 3.18146 2.89801
2.37887 2.77519 2.98518 4.58477 3.61503
0.01472 4.62188 5.34423 0.61958 0.77255 0.64581 0.74283
684   2.74790 5.04958 2.62307 2.52678 4.32098 2.76852 2.94747
2.41960 2.52319 3.32365 4.11466 3.05741 3.94504 2.88183 2.20727
2.39573 2.97984 3.40105 5.53165 4.17303   854 r - - -
2.68618 4.42225 2.77519 2.73123 3.46354 2.40513 3.72494
3.29354 2.67741 2.69355 4.24690 2.90347 2.73739 3.18146 2.89801
2.37887 2.77519 2.98518 4.58477 3.61503
0.01472 4.62188 5.34423 0.61958 0.77255 0.39730 1.11515
685   2.76905 4.81893 3.27470 2.71605 2.97411 2.81370 2.99817
3.41547 2.31309 2.60404 3.90331 3.21325 4.02448 3.04175 3.13935
2.15480 2.23156 3.13058 5.35054 4.04934   855 s - - -
2.68618 4.42225 2.77519 2.73123 3.46354 2.40513 3.72494
3.29354 2.67741 2.69355 4.24690 2.90347 2.73739 3.18146 2.89801
2.37887 2.77519 2.98518 4.58477 3.61503
0.01350 4.70791 5.43026 0.61958 0.77255 0.48576 0.95510
686   3.23253 5.79629 1.75070 2.05341 5.15070 1.26855 4.10529
4.67247 3.08187 4.15201 4.96908 3.06701 4.19089 3.25713 3.66961
2.36319 3.51914 4.21941 6.30549 4.80677   856 g - - -
2.68618 4.42225 2.77519 2.73123 3.46354 2.40513 3.72494
3.29354 2.67741 2.69355 4.24690 2.90347 2.73739 3.18146 2.89801
2.37887 2.77519 2.98518 4.58477 3.61503
0.01350 4.70791 5.43026 0.61958 0.77255 0.48576 0.95510
687   2.79091 2.82254 2.51528 2.20342 4.56116 2.40446 3.76077
4.03015 1.96058 3.53670 4.29301 3.03286 2.94797 2.86852 3.00246
2.76349 3.02459 3.61866 5.69115 4.29411   857 k - - -
2.68618 4.42225 2.77519 2.73123 3.46354 2.40513 3.72494
3.29354 2.67741 2.69355 4.24690 2.90347 2.73739 3.18146 2.89801
2.37887 2.77519 2.98518 4.58477 3.61503
0.09517 4.70791 2.50404 0.61958 0.77255 0.48576 0.95510
688   2.35620 5.11408 2.53622 2.49044 4.41397 2.77233 3.73342
3.03998 2.49422 3.40409 4.17625 3.02774 3.05883 2.41704 2.97661
2.32322 2.56634 3.48211 5.58628 4.20896   858 s - - -
2.68618 4.42225 2.77519 2.73123 3.46354 2.40513 3.72494
3.29354 2.67741 2.69355 4.24690 2.90347 2.73739 3.18146 2.89801
2.37887 2.77519 2.98518 4.58477 3.61503

0.01464 4.62738 5.34973 0.61958 0.77255 0.63669 0.75298
689 2.73960 4.39323 2.85165 3.16499 2.83931 3.74812 4.01282
2.44723 3.10751 2.59927 3.50017 3.53607 3.06352 3.39465 3.42958
2.10719 2.97168 2.25269 3.11976 3.75844 859 s - - -
2.68618 4.42225 2.77519 2.73123 3.46354 2.40513 3.72494
3.29354 2.67741 2.69355 4.24690 2.90347 2.73739 3.18146 2.89801
2.37887 2.77519 2.98518 4.58477 3.61503
0.01464 4.62738 5.34973 0.61958 0.77255 0.63669 0.75298
690 2.38084 3.41012 2.59070 2.56360 4.23012 3.55839 3.76852
3.65356 2.56020 3.24501 4.04937 2.61734 2.94607 2.91098 3.02829
2.35386 2.46410 3.31768 5.47684 3.03833 860 s - - -
2.68618 4.42225 2.77519 2.73123 3.46354 2.40513 3.72494
3.29354 2.67741 2.69355 4.24690 2.90347 2.73739 3.18146 2.89801
2.37887 2.77519 2.98518 4.58477 3.61503
0.01464 4.62738 5.34973 0.61958 0.77255 0.63669 0.75298
691 2.74041 3.28094 3.42202 2.39746 3.76829 3.66436 3.89976
2.24011 2.83108 2.82857 3.70136 2.63085 4.04857 2.63829 3.23270
2.89363 2.44858 2.42898 5.16846 3.90624 861 i - - -
2.68618 4.42225 2.77519 2.73123 3.46354 2.40513 3.72494
3.29354 2.67741 2.69355 4.24690 2.90347 2.73739 3.18146 2.89801
2.37887 2.77519 2.98518 4.58477 3.61503
0.01464 4.62738 5.34973 0.61958 0.77255 0.63669 0.75298
692 3.59265 4.81259 5.59770 5.07788 3.73157 5.09260 5.61134
1.23435 4.97691 1.23003 3.48892 5.23562 5.26882 5.08183 5.05503
4.49188 3.83371 1.45952 5.83777 4.73651 862 l - - -
2.68618 4.42225 2.77519 2.73123 3.46354 2.40513 3.72494
3.29354 2.67741 2.69355 4.24690 2.90347 2.73739 3.18146 2.89801
2.37887 2.77519 2.98518 4.58477 3.61503
0.01464 4.62738 5.34973 0.61958 0.77255 0.63669 0.75298
693 2.93569 2.88221 4.17721 3.68975 3.29758 3.82570 4.19884
3.21799 3.49007 2.91153 3.86449 2.68177 4.33469 3.82550 3.71138
3.19779 3.24824 2.98523 1.08170 3.38595 863 w - - -
2.68618 4.42225 2.77519 2.73123 3.46354 2.40513 3.72494
3.29354 2.67741 2.69355 4.24690 2.90347 2.73739 3.18146 2.89801
2.37887 2.77519 2.98518 4.58477 3.61503
0.01464 4.62738 5.34973 0.61958 0.77255 0.40178 1.10603
694 2.40818 5.18268 3.05599 2.22395 4.49171 3.55834 3.75114
3.95037 1.94620 3.47273 4.23614 3.04375 3.95240 2.48705 2.98083
2.08239 2.59744 2.80013 5.63990 4.25501 864 k - - -
2.68618 4.42225 2.77519 2.73123 3.46354 2.40513 3.72494
3.29354 2.67741 2.69355 4.24690 2.90347 2.73739 3.18146 2.89801
2.37887 2.77519 2.98518 4.58477 3.61503
0.18053 4.70791 1.85696 0.61958 0.77255 0.48576 0.95510
695 2.21406 4.89806 2.43569 2.63300 4.27704 3.49897 3.87048
3.68144 2.70257 2.62434 4.12433 3.13324 3.97085 3.03180 3.16428
1.50722 3.01037 3.33263 5.55081 4.21641 865 s - - -
2.68618 4.42225 2.77519 2.73123 3.46354 2.40513 3.72494
3.29354 2.67741 2.69355 4.24690 2.90347 2.73739 3.18146 2.89801
2.37887 2.77519 2.98518 4.58477 3.61503
0.01593 4.54331 5.26566 0.61958 0.77255 0.76305 0.62781
696 2.71675 4.85086 3.14312 2.20589 3.08302 3.55638 3.77262
3.48021 2.59370 2.62616 3.93157 2.17661 3.95009 2.93934 3.04811
2.35354 2.45409 3.17472 5.36900 4.04797 866 n - - -

2.68618 4.42225 2.77519 2.73123 3.46354 2.40513 3.72494
3.29354 2.67741 2.69355 4.24690 2.90347 2.73739 3.18146 2.89801
2.37887 2.77519 2.98518 4.58477 3.61503
0.01593 4.54331 5.26566 0.61958 0.77255 0.76305 0.62781
697 2.74493 5.21410 2.43077 2.42176 4.54776 3.49216 3.70968
4.02339 2.11392 3.51953 4.27153 2.47420 2.87536 2.81574 2.95707
2.34692 2.18158 3.60151 5.66566 4.26139 867 k - - -
2.68618 4.42225 2.77519 2.73123 3.46354 2.40513 3.72494
3.29354 2.67741 2.69355 4.24690 2.90347 2.73739 3.18146 2.89801
2.37887 2.77519 2.98518 4.58477 3.61503
0.01593 4.54331 5.26566 0.61958 0.77255 0.76305 0.62781
698 2.71648 5.04267 2.59181 2.48376 4.31683 2.77830 2.84314
3.75898 2.48877 3.31827 3.04116 2.48469 3.91079 2.84258 2.96674
2.36620 2.94828 3.39598 5.51846 3.03301 868 s - - -
2.68618 4.42225 2.77519 2.73123 3.46354 2.40513 3.72494
3.29354 2.67741 2.69355 4.24690 2.90347 2.73739 3.18146 2.89801
2.37887 2.77519 2.98518 4.58477 3.61503
0.22698 4.54331 1.64805 0.61958 0.77255 0.76305 0.62781
699 2.65390 3.21798 3.16330 2.61684 3.88850 3.51301 3.74673
3.29992 2.59338 2.94728 3.79245 2.51333 3.91182 2.37820 3.02489
2.26341 2.89110 3.01460 5.22614 2.85629 869 s - - -
2.68618 4.42225 2.77519 2.73123 3.46354 2.40513 3.72494
3.29354 2.67741 2.69355 4.24690 2.90347 2.73739 3.18146 2.89801
2.37887 2.77519 2.98518 4.58477 3.61503
0.15494 4.33597 2.03681 0.61958 0.77255 0.76214 0.62861
700 2.68053 4.67959 2.59463 2.63792 3.80838 3.53737 3.75669
2.51568 2.23673 2.90561 3.76699 3.12674 3.93371 2.97129 3.04423
2.77501 2.91283 2.97114 5.17524 2.30211 870 k - - -
2.68594 4.42242 2.77536 2.73097 3.46281 2.40498 3.72511
3.29371 2.67717 2.69372 4.24706 2.90363 2.73756 3.18163 2.89817
2.37903 2.77491 2.98535 4.58494 3.61520
0.16680 1.91617 5.04201 1.50909 0.24989 1.00305 0.45690
701 2.61904 2.94958 3.90066 3.33961 3.34330 3.63669 4.02948
2.68854 3.24660 2.45399 3.37338 3.60452 4.06412 3.50749 3.49773
1.96229 2.88199 2.02095 4.84177 2.64598 877 s - - -
2.68618 4.42225 2.77519 2.73123 3.46354 2.40513 3.72494
3.29354 2.67741 2.69355 4.24690 2.90347 2.73739 3.18146 2.89801
2.37887 2.77519 2.98518 4.58477 3.61503
0.01996 4.31966 5.04201 0.61958 0.77255 1.00305 0.45690
702 2.86185 5.36048 2.04531 2.30697 4.66963 2.53394 2.54555
4.17011 2.63161 3.66777 4.45529 1.85731 3.91562 2.90450 3.15985
2.79785 3.11828 3.74800 5.81876 4.37545 878 n - - -
2.68618 4.42225 2.77519 2.73123 3.46354 2.40513 3.72494
3.29354 2.67741 2.69355 4.24690 2.90347 2.73739 3.18146 2.89801
2.37887 2.77519 2.98518 4.58477 3.61503
0.01996 4.31966 5.04201 0.61958 0.77255 1.00305 0.45690
703 2.65586 4.53489 3.29952 2.85711 3.82658 3.49208 3.93657
1.92085 2.85917 2.89116 3.78502 2.09135 3.98004 3.19788 3.23823
2.17167 2.94611 2.85199 5.23589 3.95943 879 i - - -
2.68618 4.42225 2.77519 2.73123 3.46354 2.40513 3.72494
3.29354 2.67741 2.69355 4.24690 2.90347 2.73739 3.18146 2.89801
2.37887 2.77519 2.98518 4.58477 3.61503
0.01996 4.31966 5.04201 0.61958 0.77255 0.55999 0.84681

```
    704   2.75947  4.29402  4.02159  3.45516  3.40717  3.82510  2.84226
 1.67093  3.34487  2.45878  3.40609  3.73591  3.03635  3.61582  3.58611
 3.10994  2.99734  1.79863  4.92264  3.71173    880 i - - -
          2.68618  4.42225  2.77519  2.73123  3.46354  2.40513  3.72494
 3.29354  2.67741  2.69355  4.24690  2.90347  2.73739  3.18146  2.89801
 2.37887  2.77519  2.98518  4.58477  3.61503
          0.01607  4.53491  5.25725  0.61958  0.77255  0.77432  0.61807
    705   2.32540  4.76481  3.22650  2.66969  3.98460  2.76751  3.80319
 3.37537  2.63666  2.46447  3.86446  2.53555  3.96980  2.99084  2.25189
 2.79506  2.95003  2.53641  5.30966  4.01040    881 r - - -
          2.68618  4.42225  2.77519  2.73123  3.46354  2.40513  3.72494
 3.29354  2.67741  2.69355  4.24690  2.90347  2.73739  3.18146  2.89801
 2.37887  2.77519  2.98518  4.58477  3.61503
          0.01607  4.53491  5.25725  0.61958  0.77255  0.55043  0.85967
    706   3.01655  5.41300  2.69998  2.00368  4.87740  1.30758  4.01461
 4.36403  2.91016  3.88246  4.68645  2.34024  4.09069  3.16284  3.43304
 2.35029  3.31231  3.92352  6.05013  4.62099    882 g - - -
          2.68618  4.42225  2.77519  2.73123  3.46354  2.40513  3.72494
 3.29354  2.67741  2.69355  4.24690  2.90347  2.73739  3.18146  2.89801
 2.37887  2.77519  2.98518  4.58477  3.61503
          0.01477  4.61848  5.34083  0.61958  0.77255  0.65138  0.73674
    707   2.75070  5.01567  3.08669  2.56116  4.31929  2.01016  3.78627
 3.75081  2.56783  2.75042  4.12461  2.58319  3.95818  2.43074  3.03466
 2.38487  2.51277  3.39565  5.54315  4.19183    883 g - - -
          2.68618  4.42225  2.77519  2.73123  3.46354  2.40513  3.72494
 3.29354  2.67741  2.69355  4.24690  2.90347  2.73739  3.18146  2.89801
 2.37887  2.77519  2.98518  4.58477  3.61503
          0.01477  4.61848  5.34083  0.61958  0.77255  0.65138  0.73674
    708   2.99835  5.54097  2.24315  1.99057  4.86090  3.53860  3.89277
 4.36011  2.73716  3.83805  4.61564  2.99829  4.04517  1.35276  3.26397
 2.28925  3.25074  3.92378  5.97885  4.52915    884 q - - -
          2.68618  4.42225  2.77519  2.73123  3.46354  2.40513  3.72494
 3.29354  2.67741  2.69355  4.24690  2.90347  2.73739  3.18146  2.89801
 2.37887  2.77519  2.98518  4.58477  3.61503
          0.11449  4.61848  2.31961  0.61958  0.77255  0.65138  0.73674
    709   2.27238  3.12625  3.77907  3.20727  3.44176  3.72488  4.00345
 2.79446  3.13385  2.18459  3.43151  3.55185  4.10471  2.70893  3.43201
 2.98762  2.46412  2.58355  4.91667  2.26927    885 l - - -
          2.68618  4.42225  2.77519  2.73123  3.46354  2.40513  3.72494
 3.29354  2.67741  2.69355  4.24690  2.90347  2.73739  3.18146  2.89801
 2.37887  2.77519  2.98518  4.58477  3.61503
          0.01630  4.52030  5.24264  0.61958  0.77255  0.53660  0.87884
    710   2.75564  5.12418  3.03489  2.20655  4.42008  3.53972  3.73911
 3.86849  2.14555  2.77518  4.18391  2.53437  3.93764  2.85773  2.96659
 2.33800  1.97470  3.48907  5.59041  4.21648    886 t - - -
          2.68726  4.42141  2.77489  2.73229  3.46057  2.40509  3.72413
 3.29282  2.67890  2.69301  4.24643  2.90293  2.73846  3.18094  2.89950
 2.37861  2.77536  2.98504  4.58626  3.61037
          0.65407  1.20221  1.71734  2.74753  0.06623  0.65133  0.73679
    711   2.91772  4.37396  4.39409  3.84193  3.38781  4.03759  4.41850
 1.66532  3.70135  1.37415  3.32601  4.05513  4.40858  3.94279  3.88445
 2.41442  3.16762  2.25303  5.06065  3.87036    923 l - - -
          2.68618  4.42225  2.77519  2.73123  3.46354  2.40513  3.72494
 3.29354  2.67741  2.69355  4.24690  2.90347  2.73739  3.18146  2.89801
 2.37887  2.77519  2.98518  4.58477  3.61503
```

```
          0.01792  4.42644  5.14879  0.61958  0.77255  0.90220  0.52033
    712   2.24447  2.33783  4.26783  3.68692  2.48878  3.76751  4.14712
2.11269  3.55034  2.34450  3.27879  3.83471  4.16591  3.75394  3.69641
2.48367  2.91898  2.35653  4.78702  3.59645    924 i - - -
          2.68566  4.42256  2.77550  2.73111  3.46295  2.40512  3.72525
3.29234  2.67731  2.69386  4.24720  2.90326  2.73770  3.18177  2.89831
2.37887  2.77550  2.98549  4.58234  3.61534
          0.14858  2.02295  5.14879  2.04628  0.13836  0.66729  0.71970
    713   2.26494  2.50361  3.29298  2.78343  4.02705  3.52823  2.29762
3.44161  2.75798  3.09536  3.94255  2.07007  3.99220  3.11167  3.17083
2.80588  2.98016  3.13367  5.36837  4.07195    936 n - - -
          2.68618  4.42225  2.77519  2.73123  3.46354  2.40513  3.72494
3.29354  2.67741  2.69355  4.24690  2.90347  2.73739  3.18146  2.89801
2.37887  2.77519  2.98518  4.58477  3.61503
          0.01624  4.52404  5.24639  0.61958  0.77255  0.78856  0.60605
    714   2.71519  4.95041  3.09148  2.61050  4.42178  3.47023  3.84474
3.86145  2.62026  3.42964  4.22223  3.10948  2.24129  2.10055  3.07132
1.91256  2.45002  3.46398  5.62842  4.28260    937 s - - -
          2.68618  4.42225  2.77519  2.73123  3.46354  2.40513  3.72494
3.29354  2.67741  2.69355  4.24690  2.90347  2.73739  3.18146  2.89801
2.37887  2.77519  2.98518  4.58477  3.61503
          0.01624  4.52404  5.24639  0.61958  0.77255  0.78856  0.60605
    715   2.01394  4.44841  3.76906  3.27410  4.09135  2.00455  4.24881
3.45467  3.24225  3.16779  4.02743  3.53811  4.01308  3.53668  3.58990
2.09015  2.19574  1.96161  5.49092  4.26516    938 v - - -
          2.68618  4.42225  2.77519  2.73123  3.46354  2.40513  3.72494
3.29354  2.67741  2.69355  4.24690  2.90347  2.73739  3.18146  2.89801
2.37887  2.77519  2.98518  4.58477  3.61503
          0.20879  4.52404  1.72829  0.61958  0.77255  0.78856  0.60605
    716   2.65702  4.52635  2.57187  2.76397  3.66523  3.57238  3.80946
3.04431  2.74408  2.25110  3.61930  3.22266  2.92645  3.06759  3.14716
2.80739  2.89027  2.36432  5.07448  2.80368    939 l - - -
          2.68618  4.42225  2.77519  2.73123  3.46354  2.40513  3.72494
3.29354  2.67741  2.69355  4.24690  2.90347  2.73739  3.18146  2.89801
2.37887  2.77519  2.98518  4.58477  3.61503
          0.01966  4.33491  5.05726  0.61958  0.77255  0.59064  0.80738
    717   2.97444  5.49825  2.08954  1.68474  4.82772  3.48868  3.87931
4.32565  2.75471  3.81484  4.60137  2.95809  1.84238  3.01170  3.29491
2.32765  3.23514  3.89229  5.96616  4.51249    940 e - - -
          2.68618  4.42225  2.77519  2.73123  3.46354  2.40513  3.72494
3.29354  2.67741  2.69355  4.24690  2.90347  2.73739  3.18146  2.89801
2.37887  2.77519  2.98518  4.58477  3.61503
          0.01624  4.52404  5.24639  0.61958  0.77255  0.78856  0.60605
    718   2.81200  5.30394  2.36745  2.08543  4.62330  3.49212  3.74906
4.10648  2.16326  3.59777  4.35814  1.81185  3.93359  2.86161  3.02955
2.76614  3.05060  3.68330  5.74180  2.90867    941 n - - -
          2.68618  4.42225  2.77519  2.73123  3.46354  2.40513  3.72494
3.29354  2.67741  2.69355  4.24690  2.90347  2.73739  3.18146  2.89801
2.37887  2.77519  2.98518  4.58477  3.61503
          0.10571  4.52404  2.41389  0.61958  0.77255  0.54004  0.87401
    719   2.71873  4.70670  3.27146  2.71472  2.41870  3.59964  3.81689
3.28428  2.68978  2.94276  3.79696  2.64699  2.93530  3.03096  2.51965
2.37633  2.95094  3.01274  5.22391  3.00613    942 s - - -
```

```
         2.68618  4.42225  2.77519  2.73123  3.46354  2.40513  3.72494
3.29354  2.67741  2.69355  4.24690  2.90347  2.73739  3.18146  2.89801
2.37887  2.77519  2.98518  4.58477  3.61503
         0.01600  4.53881  5.26115  0.61958  0.77255  0.76912  0.62254
    720  2.76601  5.22160  2.93188  2.12581  4.55601  3.50509  3.71946
4.02611  1.87337  3.52353  4.28132  2.43393  3.91879  2.82790  2.93508
2.04032  2.46231  3.60987  5.66914  4.27291    943 k - - -
         2.68684  4.42291  2.77457  2.73069  3.46335  2.40578  3.72332
3.29345  2.67772  2.69344  4.24755  2.90412  2.73805  3.18155  2.89765
2.37899  2.77499  2.98375  4.58543  3.61471
         1.18689  0.66415  1.71415  1.54362  0.24030  0.76912  0.62254
    721  2.82778  4.33425  4.07870  3.55079  2.38912  3.89161  4.13800
1.46470  3.45487  2.34766  3.39137  2.58247  4.27013  3.70786  3.68392
3.19559  3.07070  2.34509  4.80059  3.46012    951 i - - -
         2.68618  4.42225  2.77519  2.73123  3.46354  2.40513  3.72494
3.29354  2.67741  2.69355  4.24690  2.90347  2.73739  3.18146  2.89801
2.37887  2.77519  2.98518  4.58477  3.61503
         0.01943  4.34652  5.06887  0.61958  0.77255  0.97960  0.47074
    722  2.25972  5.14889  2.31634  2.36992  4.47720  3.43210  3.67580
3.94495  2.05276  3.45622  4.22095  2.35225  3.86024  2.78983  2.93619
2.68013  2.43165  3.53544  5.61503  4.21562    952 k - - -
         2.68618  4.42225  2.77519  2.73123  3.46354  2.40513  3.72494
3.29354  2.67741  2.69355  4.24690  2.90347  2.73739  3.18146  2.89801
2.37887  2.77519  2.98518  4.58477  3.61503
         0.01943  4.34652  5.06887  0.61958  0.77255  0.97960  0.47074
    723  2.64042  4.81881  3.07519  2.61363  4.35675  1.99309  3.83065
3.78714  2.06201  3.36952  4.17121  3.09073  3.89315  2.98149  3.03171
2.15412  2.32359  3.38552  5.57560  4.24886    953 g - - -
         2.68618  4.42225  2.77519  2.73123  3.46354  2.40513  3.72494
3.29354  2.67741  2.69355  4.24690  2.90347  2.73739  3.18146  2.89801
2.37887  2.77519  2.98518  4.58477  3.61503
         0.01943  4.34652  5.06887  0.61958  0.77255  0.75278  0.63687
    724  2.90529  5.44292  2.08560  1.93852  4.76165  3.45875  3.80463
4.25897  2.64484  3.73906  4.51483  2.91890  3.95953  1.65519  3.17071
2.24939  3.15635  3.82436  5.88113  4.43428    954 q - - -
         2.68618  4.42225  2.77519  2.73123  3.46354  2.40513  3.72494
3.29354  2.67741  2.69355  4.24690  2.90347  2.73739  3.18146  2.89801
2.37887  2.77519  2.98518  4.58477  3.61503
         0.01754  4.44798  5.17032  0.61958  0.77255  0.87912  0.53639
    725  2.99220  4.36401  4.69227  4.12303  3.47728  4.22259  4.61046
1.82699  3.99111  2.25261  1.98736  4.28231  4.55604  4.19146  4.13041
3.54493  2.39946  1.44641  5.17060  3.99375    955 v - - -
         2.68618  4.42225  2.77519  2.73123  3.46354  2.40513  3.72494
3.29354  2.67741  2.69355  4.24690  2.90347  2.73739  3.18146  2.89801
2.37887  2.77519  2.98518  4.58477  3.61503
         0.01754  4.44798  5.17032  0.61958  0.77255  0.87912  0.53639
    726  2.78667  4.94921  3.07207  2.59737  3.82692  3.56406  2.75714
3.62110  2.61283  3.20650  4.04852  2.02146  3.98070  2.96905  3.05589
2.28911  3.02454  3.30555  5.21100  2.33045    956 n - - -
         2.68618  4.42225  2.77519  2.73123  3.46354  2.40513  3.72494
3.29354  2.67741  2.69355  4.24690  2.90347  2.73739  3.18146  2.89801
2.37887  2.77519  2.98518  4.58477  3.61503
         0.12088  4.44798  2.28123  0.61958  0.77255  0.87912  0.53639
```

```
    727   2.77377  4.25767  4.18560  3.61083  3.30175  3.87607  4.20916
2.45859  3.49219  1.58383  2.50758  3.84795  4.24274  3.72746  3.69441
2.38238  3.01332  1.90848  4.90128  3.71668    957 l - - -
          2.68618  4.42225  2.77519  2.73123  3.46354  2.40513  3.72494
3.29354  2.67741  2.69355  4.24690  2.90347  2.73739  3.18146  2.89801
2.37887  2.77519  2.98518  4.58477  3.61503
          0.01943  4.34652  5.06887  0.61958  0.77255  0.97960  0.47074
    728   2.77751  4.79728  2.22166  2.63489  4.03190  2.48022  3.90172
3.40379  2.82750  1.59682  3.97089  3.13398  3.98963  3.11989  3.27134
2.85908  3.05023  3.12734  5.40488  4.08897    958 l - - -
          2.68618  4.42225  2.77519  2.73123  3.46354  2.40513  3.72494
3.29354  2.67741  2.69355  4.24690  2.90347  2.73739  3.18146  2.89801
2.37887  2.77519  2.98518  4.58477  3.61503
          0.01943  4.34652  5.06887  0.61958  0.77255  0.97960  0.47074
    729   2.77398  4.42221  3.70750  3.17353  1.96202  3.74187  3.86746
2.92003  3.11708  2.60948  3.54161  2.57649  4.12770  3.39683  3.42946
3.01281  2.32980  2.70854  4.62339  2.38309    959 f - - -
          2.68618  4.42225  2.77519  2.73123  3.46354  2.40513  3.72494
3.29354  2.67741  2.69355  4.24690  2.90347  2.73739  3.18146  2.89801
2.37887  2.77519  2.98518  4.58477  3.61503
          0.01943  4.34652  5.06887  0.61958  0.77255  0.97960  0.47074
    730   2.68097  4.71066  3.20697  2.66518  3.96995  2.62206  3.78052
2.52733  1.87660  2.99533  3.84718  3.14437  3.93723  2.97109  2.97715
2.76981  2.35573  3.03459  5.28811  3.99899    960 k - - -
          2.68618  4.42225  2.77519  2.73123  3.46354  2.40513  3.72494
3.29354  2.67741  2.69355  4.24690  2.90347  2.73739  3.18146  2.89801
2.37887  2.77519  2.98518  4.58477  3.61503
          0.01943  4.34652  5.06887  0.61958  0.77255  0.97960  0.47074
    731   2.68519  4.85676  3.02925  2.64690  4.48196  2.41686  3.89175
3.92286  2.03409  3.49311  4.29884  3.11549  3.92371  3.04737  3.04924
1.45104  3.00956  3.49473  5.67619  4.35281    961 s - - -
          2.68672  4.42279  2.77484  2.73177  3.46319  2.40567  3.72549
3.29257  2.67679  2.69368  4.24744  2.90299  2.73794  3.18133  2.89855
2.37859  2.77486  2.98522  4.58257  3.61453
          0.99460  0.70233  2.00469  1.56873  0.23359  0.75571  0.63427
    732   3.11635  4.46999  4.70008  4.17866  2.03353  4.22138  4.09160
1.34732  4.01779  2.18961  3.37054  4.19387  4.54004  4.12193  4.09113
3.54459  3.34523  2.49615  4.38993  2.15705    976 i - - -
          2.68618  4.42225  2.77519  2.73123  3.46354  2.40513  3.72494
3.29354  2.67741  2.69355  4.24690  2.90347  2.73739  3.18146  2.89801
2.37887  2.77519  2.98518  4.58477  3.61503
          0.01989  4.32308  5.04542  0.61958  0.77255  1.00013  0.45860
    733   2.92984  5.50855  1.64183  1.87115  4.81980  3.40019  3.79607
4.32950  2.09114  3.79789  4.58543  2.16868  3.93534  2.92821  3.21612
2.84123  3.18799  3.88609  5.93575  4.46622    977 d - - -
          2.68618  4.42225  2.77519  2.73123  3.46354  2.40513  3.72494
3.29354  2.67741  2.69355  4.24690  2.90347  2.73739  3.18146  2.89801
2.37887  2.77519  2.98518  4.58477  3.61503
          0.01989  4.32308  5.04542  0.61958  0.77255  1.00013  0.45860
    734   2.82033  5.17407  2.79157  2.02161  4.50430  3.46595  3.74499
3.94277  2.47658  3.47637  4.28215  2.97018  3.92979  1.60770  2.90720
2.79608  2.36133  3.56562  5.64941  4.28111    978 q - - -
          2.68565  4.42248  2.77542  2.73105  3.46292  2.40535  3.72517
3.29377  2.67763  2.69342  4.24712  2.90369  2.73721  3.18169  2.89823
2.37909  2.77542  2.98396  4.58500  3.61526
```

```
          0.69360  0.70565  5.04542  0.79198  0.60321  1.00013  0.45860
    735   3.11189  4.48958  4.68608  4.12159  3.05598  4.27375  4.43865
1.91278  3.95851  1.11620  3.13910  4.27837  4.56215  4.11436  4.07388
3.59321  3.33830  2.40360  4.84623  2.41370    982 l - - -
          2.68618  4.42225  2.77519  2.73123  3.46354  2.40513  3.72494
3.29354  2.67741  2.69355  4.24690  2.90347  2.73739  3.18146  2.89801
2.37887  2.77519  2.98518  4.58477  3.61503
          0.01989  4.32308  5.04542  0.61958  0.77255  1.00013  0.45860
    736   2.86101  5.31281  2.09370  2.36280  4.67114  2.45233  3.77360
4.14929  1.47771  3.64720  4.43733  2.91811  3.92929  2.90448  3.04508
2.80890  3.11637  3.73052  5.79024  4.37919    983 k - - -
          2.68618  4.42225  2.77519  2.73123  3.46354  2.40513  3.72494
3.29354  2.67741  2.69355  4.24690  2.90347  2.73739  3.18146  2.89801
2.37887  2.77519  2.98518  4.58477  3.61503
          0.01989  4.32308  5.04542  0.61958  0.77255  0.73302  0.65480
    737   3.02754  4.50060  2.66007  3.71769  3.75711  4.18248  4.58859
1.49865  3.69958  2.52754  3.63601  4.05819  4.54919  3.98046  3.99857
3.50292  3.27920  1.23380  5.43828  4.20652    984 v - - -
          2.68618  4.42225  2.77519  2.73123  3.46354  2.40513  3.72494
3.29354  2.67741  2.69355  4.24690  2.90347  2.73739  3.18146  2.89801
2.37887  2.77519  2.98518  4.58477  3.61503
          0.12544  4.44658  2.24271  0.61958  0.77255  0.88065  0.53532
    738   2.73204  5.06501  2.89359  2.45849  4.45606  3.44119  3.73183
3.90637  2.02169  3.43951  4.22621  2.41590  2.21604  2.86018  2.91553
2.18271  2.98740  3.50968  5.60907  4.24351    985 k - - -
          2.68618  4.42225  2.77519  2.73123  3.46354  2.40513  3.72494
3.29354  2.67741  2.69355  4.24690  2.90347  2.73739  3.18146  2.89801
2.37887  2.77519  2.98518  4.58477  3.61503
          0.01954  4.34069  5.06304  0.61958  0.77255  0.98479  0.46763
    739   2.19327  4.55710  3.33032  2.81672  3.87892  2.58387  3.88676
3.19120  2.20626  2.92031  3.79110  3.24886  3.95564  3.12059  3.14221
2.77860  2.91794  1.87901  5.25146  3.98728    986 v - - -
          2.68618  4.42225  2.77519  2.73123  3.46354  2.40513  3.72494
3.29354  2.67741  2.69355  4.24690  2.90347  2.73739  3.18146  2.89801
2.37887  2.77519  2.98518  4.58477  3.61503
          0.01954  4.34069  5.06304  0.61958  0.77255  0.98479  0.46763
    740   2.18272  4.30748  3.79401  3.22751  3.23262  3.72264  2.78058
2.77287  3.10710  1.77745  3.40609  3.55026  4.09972  3.41098  3.38381
2.99436  2.94115  2.57286  2.94292  3.42854    987 l - - -
          2.68618  4.42225  2.77519  2.73123  3.46354  2.40513  3.72494
3.29354  2.67741  2.69355  4.24690  2.90347  2.73739  3.18146  2.89801
2.37887  2.77519  2.98518  4.58477  3.61503
          0.01954  4.34069  5.06304  0.61958  0.77255  0.98479  0.46763
    741   2.88778  5.30439  1.94520  2.35680  4.74221  3.37889  3.87285
4.24349  2.78727  3.76080  4.56609  2.16704  3.94918  3.02219  3.32501
1.46034  3.18265  3.80208  5.92187  4.47898    988 s - - -
          2.68618  4.42225  2.77519  2.73123  3.46354  2.40513  3.72494
3.29354  2.67741  2.69355  4.24690  2.90347  2.73739  3.18146  2.89801
2.37887  2.77519  2.98518  4.58477  3.61503
          0.01954  4.34069  5.06304  0.61958  0.77255  0.98479  0.46763
    742   3.01053  5.59746  1.93115  1.81293  4.90461  1.83095  3.86386
4.42282  2.81332  3.89883  4.70793  2.09155  3.97246  3.00961  3.39064
2.90912  3.28316  3.97941  6.04908  4.55593    989 e - - -
```

```
          2.68618  4.42225  2.77519  2.73123  3.46354  2.40513  3.72494
3.29354  2.67741  2.69355  4.24690  2.90347  2.73739  3.18146  2.89801
2.37887  2.77519  2.98518  4.58477  3.61503
          0.01954  4.34069  5.06304  0.61958  0.77255  0.98479  0.46763
    743   2.69153  5.08830  2.43321  2.03173  4.38773  3.44621  3.66202
3.84043  2.07323  2.61484  4.14587  2.93198  3.85667  2.77959  2.90419
2.67247  2.41378  3.45303  5.54764  4.16363    990 e - - -
          2.68618  4.42225  2.77519  2.73123  3.46354  2.40513  3.72494
3.29354  2.67741  2.69355  4.24690  2.90347  2.73739  3.18146  2.89801
2.37887  2.77519  2.98518  4.58477  3.61503
          0.01954  4.34069  5.06304  0.61958  0.77255  0.98479  0.46763
    744   2.66176  4.35130  3.58309  3.01716  2.67140  3.65248  3.90918
2.26910  2.38722  2.54403  3.45350  2.59609  4.03138  3.26033  3.28966
2.90446  2.89420  2.20700  4.93793  3.70242    991 v - - -
          2.68618  4.42225  2.77519  2.73123  3.46354  2.40513  3.72494
3.29354  2.67741  2.69355  4.24690  2.90347  2.73739  3.18146  2.89801
2.37887  2.77519  2.98518  4.58477  3.61503
          0.01954  4.34069  5.06304  0.61958  0.77255  0.98479  0.46763
    745   3.92422  5.19320  4.89214  4.71671  0.47529  4.45496  4.17998
3.41983  4.61999  2.68933  4.09098  4.58516  4.91874  4.66242  4.62220
4.15289  4.23348  3.44128  4.33097  2.65623    992 F - - -
          2.68618  4.42225  2.77519  2.73123  3.46354  2.40513  3.72494
3.29354  2.67741  2.69355  4.24690  2.90347  2.73739  3.18146  2.89801
2.37887  2.77519  2.98518  4.58477  3.61503
          0.01954  4.34069  5.06304  0.61958  0.77255  0.98479  0.46763
    746   1.94381  4.67424  3.04520  2.79714  4.59056  1.74573  4.11476
4.04176  3.02121  3.65350  4.46183  1.68599  3.91112  3.30581  3.45197
2.68736  3.00274  3.53000  5.85140  4.54017    993 n - - -
          2.68793  4.42560  2.77677  2.73159  3.46172  2.40694  3.72716
3.29242  2.67591  2.69075  4.24638  2.90230  2.73778  3.18347  2.89987
2.37928  2.77207  2.98423  4.58269  3.61431
          0.73203  0.65572        *  3.31748  0.03692  0.00000        *
//
HMMER3/f [3.3 | Nov 2019]
NAME  CryM6
DESC  Cry toxin (IDOPS Model 6)
LENG  483
ALPH  amino
RF    no
MM    no
CONS  yes
CS    no
MAP   yes
DATE  Wed Feb  3 11:08:42 2021
NSEQ  14
EFFN  0.037598
CKSUM 1103034061
GA 80.0 80.0
STATS LOCAL MSV      -16.0605  0.73905
STATS LOCAL VITERBI  -19.2694  0.73905
STATS LOCAL FORWARD   -3.8865  0.73905
HMM          A        C        D        E        F        G        H
I        K        L        M        N        P        Q        R        S
T        V        W        Y
            m->m     m->i     m->d     i->m     i->i     d->m     d->d
```

```
  COMPO   2.44323  4.25988  2.88767  2.69270  3.34655  2.97835  3.74653
2.79342  2.63746  2.51254  3.69028  3.03263  3.57937  3.09964  2.90374
2.57695  2.80436  2.58277  4.81523  3.47559
          2.68618  4.42225  2.77519  2.73123  3.46354  2.40513  3.72494
3.29354  2.67741  2.69355  4.24690  2.90347  2.73739  3.18146  2.89801
2.37887  2.77519  2.98518  4.58477  3.61503
          0.06018  3.44667  3.62853  0.61958  0.77255  0.00000        *
      1   2.45910  4.35166  2.77675  2.52838  3.50901  3.07425  3.70814
2.89630  2.49121  2.67479  3.77558  2.94969  3.69469  2.96611  2.78391
2.57413  2.82765  2.67895  4.98908  3.58673      1 a - - -
          2.68618  4.42225  2.77519  2.73123  3.46354  2.40513  3.72494
3.29354  2.67741  2.69355  4.24690  2.90347  2.73739  3.18146  2.89801
2.37887  2.77519  2.98518  4.58477  3.61503
          0.04903  3.43552  4.15786  0.61958  0.77255  0.49236  0.94465
      2   2.41174  4.32304  2.53497  2.46851  3.64891  2.97262  3.71760
3.02312  2.54961  2.84809  3.88211  2.89667  3.62171  3.01393  2.90412
2.49377  2.77385  2.73996  5.06890  3.70582      2 a - - -
          2.68618  4.42225  2.77519  2.73123  3.46354  2.40513  3.72494
3.29354  2.67741  2.69355  4.24690  2.90347  2.73739  3.18146  2.89801
2.37887  2.77519  2.98518  4.58477  3.61503
          0.04903  3.43552  4.15786  0.61958  0.77255  0.49236  0.94465
      3   2.56556  4.24222  3.30323  3.06355  3.07297  3.20019  3.89579
2.39046  2.87310  1.96465  3.41282  3.34538  3.80175  3.35326  3.09099
2.80245  2.94483  2.30267  4.71271  3.31369      3 l - - -
          2.68618  4.42225  2.77519  2.73123  3.46354  2.40513  3.72494
3.29354  2.67741  2.69355  4.24690  2.90347  2.73739  3.18146  2.89801
2.37887  2.77519  2.98518  4.58477  3.61503
          0.04903  3.43552  4.15786  0.61958  0.77255  0.48975  0.94877
      4   2.52970  4.37176  2.55128  2.24942  3.64144  2.97961  3.68318
3.04571  2.53884  2.79098  3.87136  2.89836  3.60356  2.94267  2.83083
2.64165  2.88778  2.79581  4.98326  3.71007      4 e - - -
          2.68618  4.42225  2.77519  2.73123  3.46354  2.40513  3.72494
3.29354  2.67741  2.69355  4.24690  2.90347  2.73739  3.18146  2.89801
2.37887  2.77519  2.98518  4.58477  3.61503
          0.04890  3.43824  4.16059  0.61958  0.77255  0.49076  0.94717
      5   2.38997  4.30914  2.84397  2.61732  3.55606  3.06129  3.73095
2.89968  2.45851  2.69734  3.69792  2.94905  3.68807  3.00328  2.77974
2.56441  2.77785  2.64800  5.03114  3.64694      5 a - - -
          2.68618  4.42225  2.77519  2.73123  3.46354  2.40513  3.72494
3.29354  2.67741  2.69355  4.24690  2.90347  2.73739  3.18146  2.89801
2.37887  2.77519  2.98518  4.58477  3.61503
          0.04890  3.43824  4.16059  0.61958  0.77255  0.48813  0.95132
      6   2.39837  4.22380  3.35146  3.06573  3.24455  3.23186  3.97002
2.30644  2.87086  2.13458  3.37792  3.33293  3.84449  3.34192  3.11508
2.70647  2.85561  2.21156  4.93811  3.54060      6 l - - -
          2.68618  4.42225  2.77519  2.73123  3.46354  2.40513  3.72494
3.29354  2.67741  2.69355  4.24690  2.90347  2.73739  3.18146  2.89801
2.37887  2.77519  2.98518  4.58477  3.61503
          0.04876  3.44097  4.16332  0.61958  0.77255  0.48915  0.94971
      7   2.45358  4.33290  2.85796  2.64693  3.53117  3.08521  3.73598
2.85605  2.41860  2.60174  3.71887  3.01755  3.70354  2.96020  2.76504
2.60041  2.75386  2.65836  5.02083  3.65285      7 k - - -
          2.68618  4.42225  2.77519  2.73123  3.46354  2.40513  3.72494
3.29354  2.67741  2.69355  4.24690  2.90347  2.73739  3.18146  2.89801
2.37887  2.77519  2.98518  4.58477  3.61503
```

```
          0.04876  3.44097  4.16332  0.61958  0.77255  0.48915  0.94971
      8   2.41461  4.32225  2.75348  2.57402  3.61008  3.01501  3.70834
3.03573  2.41078  2.79436  3.84249  2.88741  3.65368  2.94939  2.75895
2.54686  2.72536  2.75981  5.04485  3.67065      8 k - - -
          2.68618  4.42225  2.77519  2.73123  3.46354  2.40513  3.72494
3.29354  2.67741  2.69355  4.24690  2.90347  2.73739  3.18146  2.89801
2.37887  2.77519  2.98518  4.58477  3.61503
          0.04876  3.44097  4.16332  0.61958  0.77255  0.48915  0.94971
      9   2.32873  4.26065  2.96184  2.76234  3.38905  3.06563  3.80894
2.74030  2.65988  2.41344  3.62512  3.09465  3.70522  3.00498  2.91915
2.59166  2.81490  2.56520  4.95366  3.56730      9 a - - -
          2.68618  4.42225  2.77519  2.73123  3.46354  2.40513  3.72494
3.29354  2.67741  2.69355  4.24690  2.90347  2.73739  3.18146  2.89801
2.37887  2.77519  2.98518  4.58477  3.61503
          0.04876  3.44097  4.16332  0.61958  0.77255  0.48915  0.94971
     10   2.41338  4.16918  3.32050  3.07838  3.26086  3.13411  3.98704
2.38855  2.93114  2.19167  3.46039  3.31942  3.77968  3.38020  3.16580
2.65201  2.79310  2.11020  4.93069  3.54878     10 v - - -
          2.68618  4.42225  2.77519  2.73123  3.46354  2.40513  3.72494
3.29354  2.67741  2.69355  4.24690  2.90347  2.73739  3.18146  2.89801
2.37887  2.77519  2.98518  4.58477  3.61503
          0.04876  3.44097  4.16332  0.61958  0.77255  0.48915  0.94971
     11   2.36897  4.28847  2.68589  2.56264  3.44973  2.97642  3.70129
3.05855  2.60543  2.85450  3.88235  2.73874  3.63027  3.04295  2.89847
2.50022  2.78220  2.76307  4.91915  3.42139     11 a - - -
          2.68618  4.42225  2.77519  2.73123  3.46354  2.40513  3.72494
3.29354  2.67741  2.69355  4.24690  2.90347  2.73739  3.18146  2.89801
2.37887  2.77519  2.98518  4.58477  3.61503
          0.04876  3.44097  4.16332  0.61958  0.77255  0.48915  0.94971
     12   2.25946  4.18753  2.91702  2.74362  3.52568  2.98538  3.82563
2.80704  2.68971  2.62966  3.66983  3.05524  3.65394  3.11084  2.96168
2.43409  2.69457  2.55240  5.05431  3.66784     12 a - - -
          2.68618  4.42225  2.77519  2.73123  3.46354  2.40513  3.72494
3.29354  2.67741  2.69355  4.24690  2.90347  2.73739  3.18146  2.89801
2.37887  2.77519  2.98518  4.58477  3.61503
          0.04876  3.44097  4.16332  0.61958  0.77255  0.48915  0.94971
     13   2.59706  4.23676  3.31653  3.07898  2.99391  3.19121  3.88598
2.42931  2.90856  1.89944  3.40526  3.36462  3.78606  3.37188  3.12130
2.83788  2.97040  2.31871  4.65429  3.26789     13 l - - -
          2.68618  4.42225  2.77519  2.73123  3.46354  2.40513  3.72494
3.29354  2.67741  2.69355  4.24690  2.90347  2.73739  3.18146  2.89801
2.37887  2.77519  2.98518  4.58477  3.61503
          0.04876  3.44097  4.16332  0.61958  0.77255  0.48915  0.94971
     14   2.65781  4.27871  3.40323  3.13572  2.43969  3.30673  3.80155
2.44338  3.04868  2.03206  3.34384  3.41088  3.86597  3.41853  3.27803
2.89623  3.00942  2.35274  4.40998  2.87780     14 l - - -
          2.68618  4.42225  2.77519  2.73123  3.46354  2.40513  3.72494
3.29354  2.67741  2.69355  4.24690  2.90347  2.73739  3.18146  2.89801
2.37887  2.77519  2.98518  4.58477  3.61503
          0.04876  3.44097  4.16332  0.61958  0.77255  0.48915  0.94971
     15   2.22860  4.15932  2.91032  2.73944  3.60435  2.93612  3.84121
2.83303  2.67020  2.70304  3.79320  3.00211  3.62211  3.15663  2.96021
2.43815  2.61592  2.56014  5.10311  3.74157     15 a - - -
```

```
         2.68618  4.42225  2.77519  2.73123  3.46354  2.40513  3.72494
3.29354  2.67741  2.69355  4.24690  2.90347  2.73739  3.18146  2.89801
2.37887  2.77519  2.98518  4.58477  3.61503
         0.04876  3.44097  4.16332  0.61958  0.77255  0.48915  0.94971
     16  2.34981  4.26631  2.67750  2.51225  3.61748  2.94091  3.71940
3.10765  2.57485  2.91333  3.91330  2.84940  3.60503  3.02717  2.83712
2.38472  2.75410  2.79012  5.04106  3.65242      16 a - - -
         2.68618  4.42225  2.77519  2.73123  3.46354  2.40513  3.72494
3.29354  2.67741  2.69355  4.24690  2.90347  2.73739  3.18146  2.89801
2.37887  2.77519  2.98518  4.58477  3.61503
         0.06438  3.44097  3.49598  0.61958  0.77255  0.48915  0.94971
     17  2.29496  4.10502  2.79512  2.69651  3.51063  2.78386  3.78053
2.97945  2.72512  2.77253  3.85806  3.00926  3.34762  3.15379  2.97673
2.36944  2.72982  2.68450  4.90149  3.60681      17 a - - -
         2.68618  4.42225  2.77519  2.73123  3.46354  2.40513  3.72494
3.29354  2.67741  2.69355  4.24690  2.90347  2.73739  3.18146  2.89801
2.37887  2.77519  2.98518  4.58477  3.61503
         0.04951  3.42610  4.14844  0.61958  0.77255  0.48370  0.95840
     18  2.21267  4.11105  2.97209  2.79388  3.57891  2.85716  3.86542
2.80631  2.74898  2.69984  3.78657  3.06537  3.60446  3.19467  3.00947
2.36321  2.60150  2.54538  5.08794  3.72183      18 a - - -
         2.68618  4.42225  2.77519  2.73123  3.46354  2.40513  3.72494
3.29354  2.67741  2.69355  4.24690  2.90347  2.73739  3.18146  2.89801
2.37887  2.77519  2.98518  4.58477  3.61503
         0.04876  3.44097  4.16332  0.61958  0.77255  0.48915  0.94971
     19  2.53719  4.42548  2.80645  2.57769  3.47942  3.12778  3.64428
3.05416  2.31790  2.67684  3.75889  2.97665  3.71117  2.85968  2.56264
2.65446  2.87339  2.82345  4.92306  3.48215      19 k - - -
         2.68618  4.42225  2.77519  2.73123  3.46354  2.40513  3.72494
3.29354  2.67741  2.69355  4.24690  2.90347  2.73739  3.18146  2.89801
2.37887  2.77519  2.98518  4.58477  3.61503
         0.04876  3.44097  4.16332  0.61958  0.77255  0.48915  0.94971
     20  2.40601  4.32985  2.48305  2.42644  3.65430  2.94468  3.69896
3.13309  2.60220  2.94122  3.94192  2.71360  3.59942  3.00061  2.92198
2.52007  2.76484  2.82377  5.05219  3.67983      20 a - - -
         2.68618  4.42225  2.77519  2.73123  3.46354  2.40513  3.72494
3.29354  2.67741  2.69355  4.24690  2.90347  2.73739  3.18146  2.89801
2.37887  2.77519  2.98518  4.58477  3.61503
         0.04876  3.44097  4.16332  0.61958  0.77255  0.48915  0.94971
     21  2.33325  4.21118  3.00069  2.72652  3.54152  2.99577  3.83802
2.69626  2.66403  2.55177  3.57952  3.09432  3.69322  3.14382  2.93152
2.54162  2.69539  2.45147  5.07348  3.72065      21 a - - -
         2.68618  4.42225  2.77519  2.73123  3.46354  2.40513  3.72494
3.29354  2.67741  2.69355  4.24690  2.90347  2.73739  3.18146  2.89801
2.37887  2.77519  2.98518  4.58477  3.61503
         0.04876  3.44097  4.16332  0.61958  0.77255  0.48915  0.94971
     22  2.59257  4.20386  3.26665  3.05259  3.08845  3.11229  3.87668
2.47478  2.88614  1.88462  3.44925  3.34258  3.71824  3.36036  3.08650
2.83077  2.97148  2.35670  4.65419  3.32450      22 l - - -
         2.68618  4.42225  2.77519  2.73123  3.46354  2.40513  3.72494
3.29354  2.67741  2.69355  4.24690  2.90347  2.73739  3.18146  2.89801
2.37887  2.77519  2.98518  4.58477  3.61503
         0.04876  3.44097  4.16332  0.61958  0.77255  0.48915  0.94971
```

```
     23   2.36263  4.31817  2.79813  2.61174  3.57353  3.03199  3.72749
 2.96946  2.43021  2.66879  3.77088  2.98600  3.66416  2.94047  2.74821
 2.57655  2.81663  2.72613  5.02689  3.67883     23 a - - -
          2.68618  4.42225  2.77519  2.73123  3.46354  2.40513  3.72494
 3.29354  2.67741  2.69355  4.24690  2.90347  2.73739  3.18146  2.89801
 2.37887  2.77519  2.98518  4.58477  3.61503
          0.04876  3.44097  4.16332  0.61958  0.77255  0.48915  0.94971
     24   2.42236  4.27687  3.01104  2.75834  3.28014  3.11002  3.62615
 2.78372  2.57604  2.50144  3.65535  3.08749  3.67928  3.09301  2.85230
 2.55729  2.78199  2.59205  4.83205  3.38082     24 a - - -
          2.68618  4.42225  2.77519  2.73123  3.46354  2.40513  3.72494
 3.29354  2.67741  2.69355  4.24690  2.90347  2.73739  3.18146  2.89801
 2.37887  2.77519  2.98518  4.58477  3.61503
          0.04876  3.44097  4.16332  0.61958  0.77255  0.48915  0.94971
     25   2.40804  4.34403  2.45892  2.41610  3.70344  2.92456  3.69588
 3.14408  2.56206  2.95585  3.94291  2.77250  3.60333  2.98491  2.91160
 2.51863  2.76409  2.83162  5.09078  3.72753     25 a - - -
          2.68618  4.42225  2.77519  2.73123  3.46354  2.40513  3.72494
 3.29354  2.67741  2.69355  4.24690  2.90347  2.73739  3.18146  2.89801
 2.37887  2.77519  2.98518  4.58477  3.61503
          0.04876  3.44097  4.16332  0.61958  0.77255  0.48915  0.94971
     26   2.37836  4.15732  3.24912  3.01972  3.28655  3.08651  3.94711
 2.48021  2.88635  2.27833  3.52839  3.26472  3.74294  3.33845  3.12495
 2.57634  2.77566  2.17096  4.92505  3.52862     26 v - - -
          2.68618  4.42225  2.77519  2.73123  3.46354  2.40513  3.72494
 3.29354  2.67741  2.69355  4.24690  2.90347  2.73739  3.18146  2.89801
 2.37887  2.77519  2.98518  4.58477  3.61503
          0.04876  3.44097  4.16332  0.61958  0.77255  0.48915  0.94971
     27   2.27995  4.14254  2.93810  2.76717  3.56476  2.92401  3.84658
 2.83242  2.69206  2.67511  3.78070  3.05822  3.61213  3.17600  2.97464
 2.37963  2.56309  2.56351  5.06341  3.70750     27 a - - -
          2.68618  4.42225  2.77519  2.73123  3.46354  2.40513  3.72494
 3.29354  2.67741  2.69355  4.24690  2.90347  2.73739  3.18146  2.89801
 2.37887  2.77519  2.98518  4.58477  3.61503
          0.04876  3.44097  4.16332  0.61958  0.77255  0.48915  0.94971
     28   2.45577  4.28620  2.24355  2.45823  3.59064  2.88816  3.71189
 3.07840  2.71079  2.85751  3.92745  2.88627  3.54153  3.05296  3.03221
 2.53636  2.84519  2.80008  4.93313  3.64977     28 d - - -
          2.68618  4.42225  2.77519  2.73123  3.46354  2.40513  3.72494
 3.29354  2.67741  2.69355  4.24690  2.90347  2.73739  3.18146  2.89801
 2.37887  2.77519  2.98518  4.58477  3.61503
          0.04876  3.44097  4.16332  0.61958  0.77255  0.48915  0.94971
     29   2.62991  4.37183  3.04266  2.79506  2.83230  3.22189  3.53983
 2.86341  2.64828  2.41844  3.60023  3.14471  3.77924  3.12487  2.86730
 2.77919  2.95868  2.70074  4.40736  2.59784     29 l - - -
          2.68618  4.42225  2.77519  2.73123  3.46354  2.40513  3.72494
 3.29354  2.67741  2.69355  4.24690  2.90347  2.73739  3.18146  2.89801
 2.37887  2.77519  2.98518  4.58477  3.61503
          0.04876  3.44097  4.16332  0.61958  0.77255  0.48915  0.94971
     30   2.53998  4.42413  2.74885  2.53474  3.43636  3.13643  3.62955
 3.05454  2.38358  2.68719  3.74207  2.96508  3.70521  2.77837  2.69466
 2.65425  2.86390  2.81887  4.70509  3.48678     30 k - - -
          2.68618  4.42225  2.77519  2.73123  3.46354  2.40513  3.72494
 3.29354  2.67741  2.69355  4.24690  2.90347  2.73739  3.18146  2.89801
 2.37887  2.77519  2.98518  4.58477  3.61503
```

```
          0.04876  3.44097  4.16332  0.61958  0.77255  0.48915  0.94971
     31   2.53656  4.23778  3.43888  3.14985  3.13992  3.29215  3.99560
2.15844  2.95519  2.04904  3.35111  3.41298  3.88571  3.41195  3.18896
2.79828  2.91561  2.13038  4.85055  3.44960      31 l - - -
          2.68618  4.42225  2.77519  2.73123  3.46354  2.40513  3.72494
3.29354  2.67741  2.69355  4.24690  2.90347  2.73739  3.18146  2.89801
2.37887  2.77519  2.98518  4.58477  3.61503
          0.04876  3.44097  4.16332  0.61958  0.77255  0.48915  0.94971
     32   2.46965  4.37013  2.31313  2.34395  3.68027  2.93698  3.68101
3.15097  2.61914  2.94346  3.95836  2.76498  3.58055  2.98430  2.95486
2.56959  2.84353  2.85536  5.02864  3.70598      32 d - - -
          2.68618  4.42225  2.77519  2.73123  3.46354  2.40513  3.72494
3.29354  2.67741  2.69355  4.24690  2.90347  2.73739  3.18146  2.89801
2.37887  2.77519  2.98518  4.58477  3.61503
          0.04876  3.44097  4.16332  0.61958  0.77255  0.48915  0.94971
     33   2.52076  4.37784  2.82547  2.60252  3.40911  3.10002  3.70131
2.93863  2.49709  2.53713  3.63048  3.01348  3.70424  2.73361  2.76016
2.65515  2.87795  2.74071  4.90410  3.52237      33 k - - -
          2.68618  4.42225  2.77519  2.73123  3.46354  2.40513  3.72494
3.29354  2.67741  2.69355  4.24690  2.90347  2.73739  3.18146  2.89801
2.37887  2.77519  2.98518  4.58477  3.61503
          0.04876  3.44097  4.16332  0.61958  0.77255  0.48915  0.94971
     34   2.34667  4.17684  3.29526  3.04963  3.28845  3.13732  3.97836
2.39706  2.90179  2.20246  3.47310  3.29810  3.78290  3.35555  3.14286
2.63692  2.81586  2.17728  4.96086  3.57229      34 v - - -
          2.68618  4.42225  2.77519  2.73123  3.46354  2.40513  3.72494
3.29354  2.67741  2.69355  4.24690  2.90347  2.73739  3.18146  2.89801
2.37887  2.77519  2.98518  4.58477  3.61503
          0.04876  3.44097  4.16332  0.61958  0.77255  0.48915  0.94971
     35   2.20233  4.11886  3.11208  2.91873  3.43959  2.97928  3.92813
2.62106  2.84489  2.46471  3.64728  3.16873  3.66502  3.28606  3.09325
2.46411  2.73096  2.33296  5.02637  3.65342      35 a - - -
          2.68618  4.42225  2.77519  2.73123  3.46354  2.40513  3.72494
3.29354  2.67741  2.69355  4.24690  2.90347  2.73739  3.18146  2.89801
2.37887  2.77519  2.98518  4.58477  3.61503
          0.04876  3.44097  4.16332  0.61958  0.77255  0.48618  0.95444
     36   2.43722  4.26080  3.09002  2.82788  3.13809  3.13996  3.75440
2.68273  2.67780  2.45065  3.59180  2.96235  3.75224  3.15539  2.94139
2.61720  2.81594  2.48865  4.75656  3.27400      36 a - - -
          2.68618  4.42225  2.77519  2.73123  3.46354  2.40513  3.72494
3.29354  2.67741  2.69355  4.24690  2.90347  2.73739  3.18146  2.89801
2.37887  2.77519  2.98518  4.58477  3.61503
          0.04861  3.44405  4.16640  0.61958  0.77255  0.48732  0.95261
     37   2.57464  4.35879  3.11759  2.80842  3.18862  3.23867  3.70754
2.74014  2.44145  2.26677  3.54595  3.15949  3.79104  3.03437  2.76215
2.74365  2.90392  2.57955  4.60277  3.32040      37 l - - -
          2.68618  4.42225  2.77519  2.73123  3.46354  2.40513  3.72494
3.29354  2.67741  2.69355  4.24690  2.90347  2.73739  3.18146  2.89801
2.37887  2.77519  2.98518  4.58477  3.61503
          0.04861  3.44405  4.16640  0.61958  0.77255  0.48732  0.95261
     38   2.46258  4.19228  3.40121  3.13361  3.20051  3.20259  4.00244
2.30277  2.96240  2.11965  3.40596  3.37509  3.82816  3.41053  3.19512
2.71419  2.86366  2.06129  4.89332  3.50218      38 v - - -
```

```
          2.68618  4.42225  2.77519  2.73123  3.46354  2.40513  3.72494
 3.29354  2.67741  2.69355  4.24690  2.90347  2.73739  3.18146  2.89801
 2.37887  2.77519  2.98518  4.58477  3.61503
          0.04861  3.44405  4.16640  0.61958  0.77255  0.48732  0.95261
     39   2.46649  4.41171  2.50817  2.36093  3.67309  3.04433  3.68438
 3.03783  2.54303  2.86126  3.79534  2.84555  3.66356  2.95971  2.87223
 2.55618  2.82375  2.76161  5.09415  3.71308     39 e - - -
          2.68618  4.42225  2.77519  2.73123  3.46354  2.40513  3.72494
 3.29354  2.67741  2.69355  4.24690  2.90347  2.73739  3.18146  2.89801
 2.37887  2.77519  2.98518  4.58477  3.61503
          0.04861  3.44405  4.16640  0.61958  0.77255  0.48732  0.95261
     40   2.12358  3.45570  3.44807  3.14926  3.46401  2.91462  4.00104
 2.56571  2.96574  2.48193  3.61669  3.27804  3.61138  3.38569  3.18779
 2.39780  2.64063  2.31973  5.01213  3.71110     40 a - - -
          2.68618  4.42225  2.77519  2.73123  3.46354  2.40513  3.72494
 3.29354  2.67741  2.69355  4.24690  2.90347  2.73739  3.18146  2.89801
 2.37887  2.77519  2.98518  4.58477  3.61503
          0.04861  3.44405  4.16640  0.61958  0.77255  0.48732  0.95261
     41   2.58759  4.28407  3.43372  3.13656  3.06803  3.34191  3.95291
 2.25658  2.90724  1.93824  3.33628  3.41323  3.91608  3.38537  3.13872
 2.84251  2.95307  2.19480  4.78147  3.34976     41 l - - -
          2.68618  4.42225  2.77519  2.73123  3.46354  2.40513  3.72494
 3.29354  2.67741  2.69355  4.24690  2.90347  2.73739  3.18146  2.89801
 2.37887  2.77519  2.98518  4.58477  3.61503
          0.04861  3.44405  4.16640  0.61958  0.77255  0.48479  0.95666
     42   2.24324  4.11680  2.86471  2.71482  3.54574  2.85023  3.80084
 3.05272  2.71986  2.85527  3.89495  2.95899  3.48913  3.15381  2.98187
 2.19463  2.68821  2.72485  4.99092  3.61938     42 s - - -
          2.68618  4.42225  2.77519  2.73123  3.46354  2.40513  3.72494
 3.29354  2.67741  2.69355  4.24690  2.90347  2.73739  3.18146  2.89801
 2.37887  2.77519  2.98518  4.58477  3.61503
          0.04848  3.44667  4.16901  0.61958  0.77255  0.48576  0.95510
     43   2.49474  4.37860  2.27211  2.27325  3.76822  2.85029  3.67951
 3.18522  2.61465  2.96306  3.97119  2.82575  3.56294  2.97286  2.95373
 2.58940  2.86037  2.88882  5.05385  3.79971     43 d - - -
          2.68618  4.42225  2.77519  2.73123  3.46354  2.40513  3.72494
 3.29354  2.67741  2.69355  4.24690  2.90347  2.73739  3.18146  2.89801
 2.37887  2.77519  2.98518  4.58477  3.61503
          0.04848  3.44667  4.16901  0.61958  0.77255  0.48576  0.95510
     44   2.54243  4.42293  2.33165  2.18495  3.75195  2.96439  3.67052
 3.14068  2.57587  2.91336  3.94506  2.83141  3.58925  2.95924  2.90776
 2.63183  2.89326  2.86736  5.04975  3.79027     44 e - - -
          2.68618  4.42225  2.77519  2.73123  3.46354  2.40513  3.72494
 3.29354  2.67741  2.69355  4.24690  2.90347  2.73739  3.18146  2.89801
 2.37887  2.77519  2.98518  4.58477  3.61503
          0.04848  3.44667  4.16901  0.61958  0.77255  0.48576  0.95510
     45   2.53035  4.28736  3.30956  3.00486  3.11047  3.29258  3.88190
 2.41904  2.77169  2.09409  3.40710  3.30515  3.87047  3.24347  3.04549
 2.75421  2.89343  2.25536  4.81455  3.37604     45 l - - -
          2.68618  4.42225  2.77519  2.73123  3.46354  2.40513  3.72494
 3.29354  2.67741  2.69355  4.24690  2.90347  2.73739  3.18146  2.89801
 2.37887  2.77519  2.98518  4.58477  3.61503
          0.04848  3.44667  4.16901  0.61958  0.77255  0.48576  0.95510
```

```
     46   2.57033  4.22754  3.26696  2.89842  2.77041  3.32767  3.68909
2.61227  2.76989  2.31529  3.48266  3.20274  3.85819  3.19526  3.04322
2.76308  2.89595  2.44994  4.47909  2.80873      46 l - - -
          2.68618  4.42225  2.77519  2.73123  3.46354  2.40513  3.72494
3.29354  2.67741  2.69355  4.24690  2.90347  2.73739  3.18146  2.89801
2.37887  2.77519  2.98518  4.58477  3.61503
          0.04848  3.44667  4.16901  0.61958  0.77255  0.48576  0.95510
     47   2.17125  3.93614  3.09054  2.85642  3.60043  2.74285  3.86360
2.89945  2.75905  2.68993  3.76807  3.09597  3.29153  3.19834  3.01175
2.38855  2.64188  2.57583  5.04657  3.75348      47 a - - -
          2.68618  4.42225  2.77519  2.73123  3.46354  2.40513  3.72494
3.29354  2.67741  2.69355  4.24690  2.90347  2.73739  3.18146  2.89801
2.37887  2.77519  2.98518  4.58477  3.61503
          0.04848  3.44667  4.16901  0.61958  0.77255  0.48576  0.95510
     48   2.48267  4.43306  2.61373  2.44610  3.62646  3.08332  3.65350
3.09789  2.39143  2.86329  3.85457  2.85289  3.68703  2.90097  2.75496
2.57191  2.82903  2.79566  5.04911  3.60335      48 k - - -
          2.68618  4.42225  2.77519  2.73123  3.46354  2.40513  3.72494
3.29354  2.67741  2.69355  4.24690  2.90347  2.73739  3.18146  2.89801
2.37887  2.77519  2.98518  4.58477  3.61503
          0.04848  3.44667  4.16901  0.61958  0.77255  0.48576  0.95510
     49   2.56053  4.35866  2.54942  2.12029  3.67724  2.95383  3.68782
3.05650  2.55796  2.81395  3.89849  2.90674  3.57609  3.00045  2.84598
2.67298  2.91488  2.80784  4.96026  3.74990      49 e - - -
          2.68618  4.42225  2.77519  2.73123  3.46354  2.40513  3.72494
3.29354  2.67741  2.69355  4.24690  2.90347  2.73739  3.18146  2.89801
2.37887  2.77519  2.98518  4.58477  3.61503
          0.04848  3.44667  4.16901  0.61958  0.77255  0.48576  0.95510
     50   2.54283  4.29922  2.80624  2.62408  3.57318  2.97610  3.69246
3.01064  2.09585  2.71905  3.83360  3.01321  3.59068  3.02082  2.70765
2.68222  2.90286  2.77518  4.87989  3.66145      50 k - - -
          2.68618  4.42225  2.77519  2.73123  3.46354  2.40513  3.72494
3.29354  2.67741  2.69355  4.24690  2.90347  2.73739  3.18146  2.89801
2.37887  2.77519  2.98518  4.58477  3.61503
          0.04848  3.44667  4.16901  0.61958  0.77255  0.48576  0.95510
     51   2.51484  4.36385  3.07495  2.74489  3.41781  3.22302  3.71255
2.75902  2.37954  2.47444  3.48056  3.10517  3.77909  2.98562  2.62175
2.67427  2.84679  2.59840  4.93151  3.55692      51 k - - -
          2.68618  4.42225  2.77519  2.73123  3.46354  2.40513  3.72494
3.29354  2.67741  2.69355  4.24690  2.90347  2.73739  3.18146  2.89801
2.37887  2.77519  2.98518  4.58477  3.61503
          0.04848  3.44667  4.16901  0.61958  0.77255  0.48576  0.95510
     52   2.40988  4.29629  3.00717  2.69571  3.45993  3.15591  3.79501
2.64199  2.58400  2.46380  3.59054  3.07876  3.75926  3.10278  2.86715
2.63014  2.81896  2.43699  5.00626  3.62038      52 a - - -
          2.68618  4.42225  2.77519  2.73123  3.46354  2.40513  3.72494
3.29354  2.67741  2.69355  4.24690  2.90347  2.73739  3.18146  2.89801
2.37887  2.77519  2.98518  4.58477  3.61503
          0.04848  3.44667  4.16901  0.61958  0.77255  0.48576  0.95510
     53   2.61297  4.25221  3.34582  3.09729  2.98847  3.22616  3.89734
2.41047  2.91821  1.86368  3.38690  3.38379  3.81339  3.38139  3.13300
2.85763  2.98138  2.30504  4.66178  3.26884      53 l - - -
          2.68618  4.42225  2.77519  2.73123  3.46354  2.40513  3.72494
3.29354  2.67741  2.69355  4.24690  2.90347  2.73739  3.18146  2.89801
2.37887  2.77519  2.98518  4.58477  3.61503
```

```
          0.04848  3.44667  4.16901  0.61958  0.77255  0.48576  0.95510
     54   2.47841  4.30338  3.08147  2.80437  3.23941  3.16053  3.74957
2.75885  2.57198  2.34857  3.59115  3.13527  3.75600  3.07729  2.71038
2.63144  2.82326  2.57242  4.83215  3.40951     54 l - - -
          2.68618  4.42225  2.77519  2.73123  3.46354  2.40513  3.72494
3.29354  2.67741  2.69355  4.24690  2.90347  2.73739  3.18146  2.89801
2.37887  2.77519  2.98518  4.58477  3.61503
          0.04848  3.44667  4.16901  0.61958  0.77255  0.48576  0.95510
     55   2.48355  4.44681  2.46847  2.35577  3.77860  3.02296  3.65193
3.20389  2.39569  2.96295  3.93131  2.78976  3.59605  2.91209  2.76415
2.57351  2.83487  2.90093  5.12950  3.78503     55 e - - -
          2.68618  4.42225  2.77519  2.73123  3.46354  2.40513  3.72494
3.29354  2.67741  2.69355  4.24690  2.90347  2.73739  3.18146  2.89801
2.37887  2.77519  2.98518  4.58477  3.61503
          0.04848  3.44667  4.16901  0.61958  0.77255  0.48576  0.95510
     56   2.33553  4.14177  3.07844  2.78149  3.44364  3.08632  3.81975
2.66663  2.63227  2.44202  3.65355  3.09267  3.71400  3.14982  2.90983
2.58192  2.78151  2.44552  4.97511  3.60214     56 a - - -
          2.68618  4.42225  2.77519  2.73123  3.46354  2.40513  3.72494
3.29354  2.67741  2.69355  4.24690  2.90347  2.73739  3.18146  2.89801
2.37887  2.77519  2.98518  4.58477  3.61503
          0.04848  3.44667  4.16901  0.61958  0.77255  0.48576  0.95510
     57   2.47511  4.20060  3.43014  3.15008  3.19674  3.23045  4.01316
2.27068  2.97083  2.10011  3.38886  3.39266  3.84876  3.41844  3.20524
2.73149  2.87162  2.04738  4.90050  3.50850     57 v - - -
          2.68618  4.42225  2.77519  2.73123  3.46354  2.40513  3.72494
3.29354  2.67741  2.69355  4.24690  2.90347  2.73739  3.18146  2.89801
2.37887  2.77519  2.98518  4.58477  3.61503
          0.04848  3.44667  4.16901  0.61958  0.77255  0.48576  0.95510
     58   2.46845  4.37187  2.73671  2.53866  3.63656  3.03031  3.65440
3.16161  2.28218  2.88524  3.89235  2.81067  3.64712  2.94072  2.59205
2.58398  2.83003  2.86949  5.00410  3.65449     58 k - - -
          2.68618  4.42225  2.77519  2.73123  3.46354  2.40513  3.72494
3.29354  2.67741  2.69355  4.24690  2.90347  2.73739  3.18146  2.89801
2.37887  2.77519  2.98518  4.58477  3.61503
          0.04848  3.44667  4.16901  0.61958  0.77255  0.48576  0.95510
     59   2.54750  4.42952  2.88764  2.61164  3.10868  3.23114  3.57992
2.94419  2.49010  2.57025  3.65388  3.00559  3.77892  2.92459  2.79282
2.68871  2.87820  2.73601  4.69453  3.09649     59 k - - -
          2.68618  4.42225  2.77519  2.73123  3.46354  2.40513  3.72494
3.29354  2.67741  2.69355  4.24690  2.90347  2.73739  3.18146  2.89801
2.37887  2.77519  2.98518  4.58477  3.61503
          0.04848  3.44667  4.16901  0.61958  0.77255  0.48576  0.95510
     60   2.01129  4.08183  3.03857  2.88807  3.49899  2.88360  3.91566
2.70751  2.87250  2.51233  3.70596  3.15505  3.57096  3.29808  3.10187
2.52494  2.76285  2.48103  4.96008  3.72186     60 a - - -
          2.68618  4.42225  2.77519  2.73123  3.46354  2.40513  3.72494
3.29354  2.67741  2.69355  4.24690  2.90347  2.73739  3.18146  2.89801
2.37887  2.77519  2.98518  4.58477  3.61503
          0.04848  3.44667  4.16901  0.61958  0.77255  0.48576  0.95510
     61   2.51031  4.33354  2.76903  2.58509  3.59705  3.00294  3.68013
3.05089  2.16143  2.79133  3.86406  2.92726  3.62055  2.99041  2.69304
2.63947  2.87263  2.79368  4.93775  3.65389     61 k - - -
```

```
         2.68618  4.42225  2.77519  2.73123  3.46354  2.40513  3.72494
3.29354  2.67741  2.69355  4.24690  2.90347  2.73739  3.18146  2.89801
2.37887  2.77519  2.98518  4.58477  3.61503
         0.04848  3.44667  4.16901  0.61958  0.77255  0.48576  0.95510
     62  2.52306  4.40188  2.81252  2.58492  3.54086  3.08632  3.64887
3.11845  2.34047  2.70880  3.78897  2.97698  3.67988  2.77592  2.47376
2.64054  2.86772  2.87664  4.94455  3.59992     62 k - - -
         2.68618  4.42225  2.77519  2.73123  3.46354  2.40513  3.72494
3.29354  2.67741  2.69355  4.24690  2.90347  2.73739  3.18146  2.89801
2.37887  2.77519  2.98518  4.58477  3.61503
         0.04848  3.44667  4.16901  0.61958  0.77255  0.48576  0.95510
     63  2.58922  4.25673  3.24927  3.01562  3.08889  3.18027  3.86462
2.49710  2.83186  1.91599  3.43659  3.31697  3.77653  3.26493  3.04984
2.81436  2.96050  2.38142  4.69544  3.31817     63 l - - -
         2.68618  4.42225  2.77519  2.73123  3.46354  2.40513  3.72494
3.29354  2.67741  2.69355  4.24690  2.90347  2.73739  3.18146  2.89801
2.37887  2.77519  2.98518  4.58477  3.61503
         0.04848  3.44667  4.16901  0.61958  0.77255  0.48576  0.95510
     64  2.27073  4.13552  2.90498  2.74332  3.46582  2.89046  3.79775
2.97354  2.71829  2.75737  3.85484  2.98642  3.58268  3.16347  2.97706
2.23163  2.70906  2.67078  4.94412  3.54282     64 s - - -
         2.68618  4.42225  2.77519  2.73123  3.46354  2.40513  3.72494
3.29354  2.67741  2.69355  4.24690  2.90347  2.73739  3.18146  2.89801
2.37887  2.77519  2.98518  4.58477  3.61503
         0.04848  3.44667  4.16901  0.61958  0.77255  0.48576  0.95510
     65  2.52275  4.45482  2.67903  2.47290  3.59048  3.08866  3.63712
3.11650  2.36234  2.77218  3.81410  2.91512  3.70050  2.80512  2.63959
2.62574  2.85573  2.85687  5.00688  3.58415     65 k - - -
         2.68618  4.42225  2.77519  2.73123  3.46354  2.40513  3.72494
3.29354  2.67741  2.69355  4.24690  2.90347  2.73739  3.18146  2.89801
2.37887  2.77519  2.98518  4.58477  3.61503
         0.04848  3.44667  4.16901  0.61958  0.77255  0.48576  0.95510
     66  2.26816  4.20419  2.93628  2.70047  3.53730  3.00664  3.82876
2.74529  2.68316  2.60286  3.73421  3.06291  3.66849  3.14648  2.95384
2.44010  2.74964  2.51952  5.06017  3.68260     66 a - - -
         2.68618  4.42225  2.77519  2.73123  3.46354  2.40513  3.72494
3.29354  2.67741  2.69355  4.24690  2.90347  2.73739  3.18146  2.89801
2.37887  2.77519  2.98518  4.58477  3.61503
         0.04848  3.44667  4.16901  0.61958  0.77255  0.48576  0.95510
     67  2.50891  4.27502  2.94570  2.71095  3.45945  2.99842  3.68563
3.04179  2.44421  2.64123  3.76828  3.06333  3.60812  2.97844  2.28027
2.65704  2.87357  2.81264  4.82039  3.55211     67 r - - -
         2.68618  4.42225  2.77519  2.73123  3.46354  2.40513  3.72494
3.29354  2.67741  2.69355  4.24690  2.90347  2.73739  3.18146  2.89801
2.37887  2.77519  2.98518  4.58477  3.61503
         0.04848  3.44667  4.16901  0.61958  0.77255  0.48576  0.95510
     68  2.38952  4.20316  2.74807  2.61836  3.46324  2.87214  3.71856
3.12161  2.65048  2.87046  3.93540  2.44245  3.53997  3.09647  2.91039
2.53297  2.80322  2.81914  4.83809  3.50411     68 a - - -
         2.68618  4.42225  2.77519  2.73123  3.46354  2.40513  3.72494
3.29354  2.67741  2.69355  4.24690  2.90347  2.73739  3.18146  2.89801
2.37887  2.77519  2.98518  4.58477  3.61503
         0.04848  3.44667  4.16901  0.61958  0.77255  0.48576  0.95510
```

69 2.56914 4.25380 3.34430 3.09080 3.08551 3.23737 3.92342
2.39576 2.89560 1.91959 3.39283 3.36922 3.83452 3.37120 3.11755
2.81134 2.94674 2.24628 4.74700 3.34400 69 l - - -
2.68618 4.42225 2.77519 2.73123 3.46354 2.40513 3.72494
3.29354 2.67741 2.69355 4.24690 2.90347 2.73739 3.18146 2.89801
2.37887 2.77519 2.98518 4.58477 3.61503
0.04848 3.44667 4.16901 0.61958 0.77255 0.48576 0.95510
70 2.61456 4.25587 3.35325 3.10140 2.97479 3.23633 3.89803
2.40484 2.92245 1.86480 3.38099 3.38742 3.82161 3.38350 3.13865
2.85982 2.98198 2.30051 4.65937 3.26017 70 l - - -
2.68618 4.42225 2.77519 2.73123 3.46354 2.40513 3.72494
3.29354 2.67741 2.69355 4.24690 2.90347 2.73739 3.18146 2.89801
2.37887 2.77519 2.98518 4.58477 3.61503
0.04848 3.44667 4.16901 0.61958 0.77255 0.48576 0.95510
71 2.50045 4.32608 3.08421 2.80723 3.21335 3.21417 3.76055
2.64032 2.62295 2.34596 3.54037 3.14878 3.79698 3.00755 2.88644
2.64606 2.85641 2.48824 4.81358 3.32095 71 l - - -
2.68618 4.42225 2.77519 2.73123 3.46354 2.40513 3.72494
3.29354 2.67741 2.69355 4.24690 2.90347 2.73739 3.18146 2.89801
2.37887 2.77519 2.98518 4.58477 3.61503
0.04848 3.44667 4.16901 0.61958 0.77255 0.48576 0.95510
72 2.35550 4.27643 2.41340 2.44626 3.71739 2.75057 3.71733
3.21370 2.66282 3.00847 3.98031 2.80187 3.55558 3.02034 2.99387
2.44505 2.76069 2.87179 5.07875 3.74200 72 a - - -
2.68618 4.42225 2.77519 2.73123 3.46354 2.40513 3.72494
3.29354 2.67741 2.69355 4.24690 2.90347 2.73739 3.18146 2.89801
2.37887 2.77519 2.98518 4.58477 3.61503
0.04848 3.44667 4.16901 0.61958 0.77255 0.48576 0.95510
73 2.25906 4.10411 2.83212 2.67271 3.68683 2.60079 3.78199
3.16039 2.67834 2.89594 3.90936 2.98583 3.14070 3.11396 2.88277
2.39295 2.69417 2.81435 5.01241 3.77428 73 a - - -
2.68618 4.42225 2.77519 2.73123 3.46354 2.40513 3.72494
3.29354 2.67741 2.69355 4.24690 2.90347 2.73739 3.18146 2.89801
2.37887 2.77519 2.98518 4.58477 3.61503
0.04848 3.44667 4.16901 0.61958 0.77255 0.48576 0.95510
74 2.42014 4.38537 2.44326 2.36385 3.72621 2.97124 3.68606
3.18148 2.57297 2.98404 3.95730 2.71544 3.61941 2.96505 2.90868
2.50297 2.77981 2.86423 5.12429 3.73726 74 e - - -
2.68618 4.42225 2.77519 2.73123 3.46354 2.40513 3.72494
3.29354 2.67741 2.69355 4.24690 2.90347 2.73739 3.18146 2.89801
2.37887 2.77519 2.98518 4.58477 3.61503
0.05271 3.44667 3.93754 0.61958 0.77255 0.48576 0.95510
75 2.64896 4.23243 3.40805 3.15978 2.36705 3.22946 3.82303
2.44740 3.08662 2.03972 3.35797 3.42549 3.80470 3.44896 3.29840
2.89611 3.01055 2.35450 4.41713 2.95760 75 l - - -
2.68618 4.42225 2.77519 2.73123 3.46354 2.40513 3.72494
3.29354 2.67741 2.69355 4.24690 2.90347 2.73739 3.18146 2.89801
2.37887 2.77519 2.98518 4.58477 3.61503
0.04868 3.44263 4.16498 0.61958 0.77255 0.48816 0.95128
76 2.49049 4.44419 2.51399 2.32484 3.72439 3.02678 3.65166
3.18141 2.44411 2.93701 3.92517 2.77714 3.64726 2.89716 2.74819
2.58304 2.84414 2.88697 5.09595 3.73377 76 e - - -
2.68618 4.42225 2.77519 2.73123 3.46354 2.40513 3.72494
3.29354 2.67741 2.69355 4.24690 2.90347 2.73739 3.18146 2.89801
2.37887 2.77519 2.98518 4.58477 3.61503

```
          0.04868  3.44263  4.16498  0.61958  0.77255  0.48816  0.95128
     77   2.34410  4.26424  2.71106  2.55027  3.64062  2.93414  3.73274
3.04888  2.55272  2.86750  3.89092  2.83986  3.62152  3.03122  2.86243
2.45433  2.70056  2.73906  5.07576  3.68651     77 a - - -
          2.68618  4.42225  2.77519  2.73123  3.46354  2.40513  3.72494
3.29354  2.67741  2.69355  4.24690  2.90347  2.73739  3.18146  2.89801
2.37887  2.77519  2.98518  4.58477  3.61503
          0.04868  3.44263  4.16498  0.61958  0.77255  0.48427  0.95750
     78   2.46574  4.25000  3.34372  2.98130  3.11136  3.22905  3.82261
2.46694  2.74326  2.23825  3.45015  3.27229  3.83229  3.22028  3.00031
2.67028  2.79556  2.34547  4.63761  3.32230     78 l - - -
          2.68618  4.42225  2.77520  2.73123  3.46354  2.40513  3.72495
3.29354  2.67741  2.69355  4.24690  2.90344  2.73740  3.18145  2.89800
2.37886  2.77519  2.98519  4.58477  3.61503
          0.08899  2.87809  3.54383  0.67481  0.71183  0.48576  0.95510
     79   2.43614  4.32394  2.62925  2.41845  3.62532  2.94697  3.71192
3.01321  2.55667  2.79591  3.85690  2.91714  3.62370  2.96720  2.84708
2.54359  2.79435  2.74458  5.03585  3.69465     83 e - - -
          2.68618  4.42225  2.77519  2.73123  3.46354  2.40513  3.72494
3.29354  2.67741  2.69355  4.24690  2.90347  2.73739  3.18146  2.89801
2.37887  2.77519  2.98518  4.58477  3.61503
          0.04915  3.43314  4.15549  0.61958  0.77255  0.48080  0.96308
     80   2.43308  4.35579  2.75233  2.56091  3.45009  3.06453  3.60487
3.06922  2.46771  2.80351  3.82608  2.82567  3.67907  2.94564  2.74726
2.55555  2.79769  2.79736  4.91535  3.47184     84 a - - -
          2.68618  4.42225  2.77519  2.73123  3.46354  2.40513  3.72494
3.29354  2.67741  2.69355  4.24690  2.90347  2.73739  3.18146  2.89801
2.37887  2.77519  2.98518  4.58477  3.61503
          0.04848  3.44667  4.16901  0.61958  0.77255  0.48576  0.95510
     81   2.24676  4.19180  2.67178  2.54181  3.73014  2.69903  3.76664
3.12980  2.65864  2.96083  3.93931  2.88502  3.55896  3.06812  2.95538
2.38930  2.70442  2.78506  5.12136  3.78528     85 a - - -
          2.68618  4.42225  2.77519  2.73123  3.46354  2.40513  3.72494
3.29354  2.67741  2.69355  4.24690  2.90347  2.73739  3.18146  2.89801
2.37887  2.77519  2.98518  4.58477  3.61503
          0.04848  3.44667  4.16901  0.61958  0.77255  0.48576  0.95510
     82   2.73105  4.24311  3.42506  3.09489  2.60188  3.19509  3.59135
2.79739  2.82846  2.24506  3.47777  3.36273  3.72698  3.28511  3.01673
2.93448  3.04184  2.68286  2.82800  2.66464     86 l - - -
          2.68618  4.42225  2.77519  2.73123  3.46354  2.40513  3.72494
3.29354  2.67741  2.69355  4.24690  2.90347  2.73739  3.18146  2.89801
2.37887  2.77519  2.98518  4.58477  3.61503
          0.04848  3.44667  4.16901  0.61958  0.77255  0.48576  0.95510
     83   2.39473  4.26651  3.02736  2.77930  3.36713  3.12463  3.79226
2.70910  2.62535  2.47637  3.61835  3.11195  3.74139  3.12854  2.88856
2.58410  2.73382  2.46628  4.93297  3.40633     87 a - - -
          2.68618  4.42225  2.77519  2.73123  3.46354  2.40513  3.72494
3.29354  2.67741  2.69355  4.24690  2.90347  2.73739  3.18146  2.89801
2.37887  2.77519  2.98518  4.58477  3.61503
          0.04848  3.44667  4.16901  0.61958  0.77255  0.48576  0.95510
     84   2.27083  4.17378  3.03460  2.81038  3.53661  2.93511  3.85418
2.71940  2.67364  2.53473  3.63941  3.10606  3.67224  3.17062  2.95548
2.50857  2.67371  2.47877  5.06722  3.71090     88 a - - -
```

```
         2.68618  4.42225  2.77519  2.73123  3.46354  2.40513  3.72494
3.29354  2.67741  2.69355  4.24690  2.90347  2.73739  3.18146  2.89801
2.37887  2.77519  2.98518  4.58477  3.61503
         0.04848  3.44667  4.16901  0.61958  0.77255  0.48576  0.95510
     85  2.22337  4.09663  2.84789  2.70397  3.59948  2.72120  3.80339
3.09966  2.72608  2.91616  3.92914  2.96135  3.52672  3.15211  2.98845
2.19798  2.67280  2.75070  5.01293  3.66694     89 s - - -
         2.68618  4.42225  2.77519  2.73123  3.46354  2.40513  3.72494
3.29354  2.67741  2.69355  4.24690  2.90347  2.73739  3.18146  2.89801
2.37887  2.77519  2.98518  4.58477  3.61503
         0.04848  3.44667  4.16901  0.61958  0.77255  0.48576  0.95510
     86  2.36781  4.28935  2.73578  2.57038  3.64382  2.98465  3.71550
3.08744  2.50114  2.85787  3.86683  2.84145  3.63293  2.97818  2.75192
2.50149  2.66063  2.78445  5.06901  3.69352     90 a - - -
         2.68618  4.42225  2.77519  2.73123  3.46354  2.40513  3.72494
3.29354  2.67741  2.69355  4.24690  2.90347  2.73739  3.18146  2.89801
2.37887  2.77519  2.98518  4.58477  3.61503
         0.04848  3.44667  4.16901  0.61958  0.77255  0.48576  0.95510
     87  2.33477  4.28264  2.53253  2.44646  3.74687  2.81862  3.72130
3.19546  2.61390  3.00568  3.96159  2.78791  3.58356  3.00740  2.93571
2.42283  2.72047  2.85032  5.13735  3.77068     91 a - - -
         2.68618  4.42225  2.77519  2.73123  3.46354  2.40513  3.72494
3.29354  2.67741  2.69355  4.24690  2.90347  2.73739  3.18146  2.89801
2.37887  2.77519  2.98518  4.58477  3.61503
         0.04848  3.44667  4.16901  0.61958  0.77255  0.48576  0.95510
     88  2.43188  4.20708  3.41557  3.12517  3.23808  3.24276  4.01366
2.24087  2.94346  2.11510  3.39271  3.37728  3.85724  3.39526  3.18557
2.72144  2.86021  2.10328  4.94681  3.55956     92 v - - -
         2.68618  4.42225  2.77519  2.73123  3.46354  2.40513  3.72494
3.29354  2.67741  2.69355  4.24690  2.90347  2.73739  3.18146  2.89801
2.37887  2.77519  2.98518  4.58477  3.61503
         0.04848  3.44667  4.16901  0.61958  0.77255  0.48576  0.95510
     89  2.33698  4.19922  3.02530  2.78782  3.46917  3.04738  3.85354
2.68015  2.71807  2.52178  3.66047  3.11358  3.70049  3.18073  2.98533
2.50055  2.64754  2.41230  5.03243  3.58906     93 a - - -
         2.68618  4.42225  2.77519  2.73123  3.46354  2.40513  3.72494
3.29354  2.67741  2.69355  4.24690  2.90347  2.73739  3.18146  2.89801
2.37887  2.77519  2.98518  4.58477  3.61503
         0.04848  3.44667  4.16901  0.61958  0.77255  0.48576  0.95510
     90  2.54808  4.26966  3.40174  3.08219  3.19726  3.33180  3.96000
2.18089  2.83390  2.09347  3.32656  3.37410  3.90307  3.33731  3.10073
2.79999  2.88502  2.18280  4.89478  3.50024     94 l - - -
         2.68618  4.42225  2.77519  2.73123  3.46354  2.40513  3.72494
3.29354  2.67741  2.69355  4.24690  2.90347  2.73739  3.18146  2.89801
2.37887  2.77519  2.98518  4.58477  3.61503
         0.04848  3.44667  4.16901  0.61958  0.77255  0.48576  0.95510
     91  2.42577  4.29663  2.94320  2.67996  3.37457  3.08875  3.72707
2.80703  2.57015  2.61246  3.69353  3.02166  3.72125  2.99535  2.84569
2.55230  2.79506  2.55851  4.89965  3.41759     95 a - - -
         2.68618  4.42225  2.77519  2.73123  3.46354  2.40513  3.72494
3.29354  2.67741  2.69355  4.24690  2.90347  2.73739  3.18146  2.89801
2.37887  2.77519  2.98518  4.58477  3.61503
         0.04848  3.44667  4.16901  0.61958  0.77255  0.48576  0.95510
```

```
     92   2.43203  4.39786  2.65746  2.40304  3.58352  3.02876  3.60182
3.10802  2.48174  2.85047  3.85518  2.87992  3.67250  2.95499  2.78067
2.53930  2.82045  2.82503  5.01749  3.55746     96 e - - -
          2.68618  4.42225  2.77519  2.73123  3.46354  2.40513  3.72494
3.29354  2.67741  2.69355  4.24690  2.90347  2.73739  3.18146  2.89801
2.37887  2.77519  2.98518  4.58477  3.61503
          0.04848  3.44667  4.16901  0.61958  0.77255  0.48576  0.95510
     93   2.35113  4.19499  2.45863  2.50818  3.70658  2.52630  3.73199
3.21589  2.69883  2.99202  3.98509  2.84575  3.49633  3.06374  3.00070
2.48397  2.76491  2.87299  4.98906  3.75252     97 a - - -
          2.68618  4.42225  2.77519  2.73123  3.46354  2.40513  3.72494
3.29354  2.67741  2.69355  4.24690  2.90347  2.73739  3.18146  2.89801
2.37887  2.77519  2.98518  4.58477  3.61503
          0.04848  3.44667  4.16901  0.61958  0.77255  0.48576  0.95510
     94   2.46113  4.43804  2.43055  2.37759  3.60385  2.97955  3.48091
3.30212  2.49563  2.99560  3.94891  2.77925  3.63423  2.92465  2.85117
2.52640  2.82124  2.97661  5.01302  3.58668     98 e - - -
          2.68618  4.42225  2.77519  2.73123  3.46354  2.40513  3.72494
3.29354  2.67741  2.69355  4.24690  2.90347  2.73739  3.18146  2.89801
2.37887  2.77519  2.98518  4.58477  3.61503
          0.04848  3.44667  4.16901  0.61958  0.77255  0.48576  0.95510
     95   2.40926  4.30366  2.81755  2.56013  3.44781  3.08001  3.73872
2.83720  2.56979  2.65023  3.71091  3.00067  3.48036  3.03658  2.89290
2.57184  2.78681  2.61614  5.01287  3.64772     99 a - - -
          2.68618  4.42225  2.77519  2.73123  3.46354  2.40513  3.72494
3.29354  2.67741  2.69355  4.24690  2.90347  2.73739  3.18146  2.89801
2.37887  2.77519  2.98518  4.58477  3.61503
          0.04848  3.44667  4.16901  0.61958  0.77255  0.48576  0.95510
     96   2.52852  4.26249  3.39134  3.08540  3.07517  3.29776  3.92975
2.34968  2.88639  2.01619  3.37040  3.36219  3.80331  3.34630  3.13124
2.76917  2.90040  2.21630  4.80371  3.36713    100 l - - -
          2.68618  4.42225  2.77519  2.73123  3.46354  2.40513  3.72494
3.29354  2.67741  2.69355  4.24690  2.90347  2.73739  3.18146  2.89801
2.37887  2.77519  2.98518  4.58477  3.61503
          0.04848  3.44667  4.16901  0.61958  0.77255  0.48576  0.95510
     97   2.70094  4.33844  3.42438  3.12327  2.43026  3.41400  3.75143
2.46276  3.02014  2.02885  3.32932  3.40960  3.93849  3.38865  3.26478
2.92919  3.03004  2.37824  4.34402  2.72465    101 l - - -
          2.68618  4.42225  2.77519  2.73123  3.46354  2.40513  3.72494
3.29354  2.67741  2.69355  4.24690  2.90347  2.73739  3.18146  2.89801
2.37887  2.77519  2.98518  4.58477  3.61503
          0.04848  3.44667  4.16901  0.61958  0.77255  0.48576  0.95510
     98   2.49107  4.34699  2.74248  2.56096  3.61200  3.00816  3.67349
3.08567  2.20516  2.83710  3.88468  2.86526  3.62911  2.97622  2.69312
2.61468  2.85576  2.81409  4.96581  3.65178    102 k - - -
          2.68618  4.42225  2.77519  2.73123  3.46354  2.40513  3.72494
3.29354  2.67741  2.69355  4.24690  2.90347  2.73739  3.18146  2.89801
2.37887  2.77519  2.98518  4.58477  3.61503
          0.04848  3.44667  4.16901  0.61958  0.77255  0.48576  0.95510
     99   2.29170  4.15058  2.68045  2.56564  3.69619  2.51544  3.76902
3.14532  2.69451  2.94498  3.94765  2.94293  3.51434  3.09777  2.97578
2.38948  2.72083  2.80351  5.03021  3.76235    103 a - - -
          2.68618  4.42225  2.77519  2.73123  3.46354  2.40513  3.72494
3.29354  2.67741  2.69355  4.24690  2.90347  2.73739  3.18146  2.89801
2.37887  2.77519  2.98518  4.58477  3.61503
```

0.04848 3.44667 4.16901 0.61958 0.77255 0.48576 0.95510
100 2.47952 4.42550 2.59718 2.46022 3.50024 3.06716 3.53942
3.22555 2.45410 2.89647 3.86786 2.81691 3.61841 2.92066 2.68724
2.57718 2.82438 2.92558 4.92681 3.44265 104 k - - -
2.68618 4.42225 2.77519 2.73123 3.46354 2.40513 3.72494
3.29354 2.67741 2.69355 4.24690 2.90347 2.73739 3.18146 2.89801
2.37887 2.77519 2.98518 4.58477 3.61503
0.04848 3.44667 4.16901 0.61958 0.77255 0.48576 0.95510
101 2.50964 4.38999 2.94449 2.69756 3.02403 3.20950 3.48562
2.92030 2.57675 2.51778 3.64217 3.06812 3.77678 3.05636 2.85037
2.69308 2.86038 2.72350 4.59121 2.90181 105 a - - -
2.68618 4.42225 2.77519 2.73123 3.46354 2.40513 3.72494
3.29354 2.67741 2.69355 4.24690 2.90347 2.73739 3.18146 2.89801
2.37887 2.77519 2.98518 4.58477 3.61503
0.04848 3.44667 4.16901 0.61958 0.77255 0.48576 0.95510
102 2.55673 4.26834 3.43703 3.14008 3.09732 3.32230 3.96785
2.29012 2.91755 1.95776 3.34615 3.40756 3.90737 3.39042 3.15120
2.81072 2.93043 2.15472 4.81553 3.38599 106 l - - -
2.68618 4.42225 2.77519 2.73123 3.46354 2.40513 3.72494
3.29354 2.67741 2.69355 4.24690 2.90347 2.73739 3.18146 2.89801
2.37887 2.77519 2.98518 4.58477 3.61503
0.04848 3.44667 4.16901 0.61958 0.77255 0.48576 0.95510
103 2.44466 4.32021 2.96001 2.70371 3.34386 3.12038 3.66358
2.89886 2.50929 2.53801 3.68639 3.03058 3.72489 2.96953 2.73554
2.57606 2.79109 2.66645 4.86852 3.42986 107 a - - -
2.68618 4.42225 2.77519 2.73123 3.46354 2.40513 3.72494
3.29354 2.67741 2.69355 4.24690 2.90347 2.73739 3.18146 2.89801
2.37887 2.77519 2.98518 4.58477 3.61503
0.04848 3.44667 4.16901 0.61958 0.77255 0.48576 0.95510
104 2.62977 4.31244 3.48591 3.15639 2.98830 3.40339 3.94058
2.30953 2.90888 1.87324 3.18242 3.44092 3.95099 3.38303 3.14273
2.88760 2.97914 2.21971 4.73731 3.29836 108 l - - -
2.68618 4.42225 2.77519 2.73123 3.46354 2.40513 3.72494
3.29354 2.67741 2.69355 4.24690 2.90347 2.73739 3.18146 2.89801
2.37887 2.77519 2.98518 4.58477 3.61503
0.05881 3.44667 3.67828 0.61958 0.77255 0.48576 0.95510
105 2.30673 4.18012 2.93007 2.75322 3.42181 2.95772 3.79958
2.86813 2.69075 2.58246 3.75034 3.05690 3.57681 3.11168 2.94848
2.38860 2.74636 2.61389 4.94131 3.53904 109 a - - -
2.68618 4.42225 2.77519 2.73123 3.46354 2.40513 3.72494
3.29354 2.67741 2.69355 4.24690 2.90347 2.73739 3.18146 2.89801
2.37887 2.77519 2.98518 4.58477 3.61503
0.04897 3.43682 4.15917 0.61958 0.77255 0.48214 0.96093
106 2.22238 4.11632 2.83372 2.67019 3.69770 2.66935 3.79120
3.13820 2.67148 2.89780 3.90153 2.97859 3.27748 3.10884 2.89352
2.35581 2.67517 2.78984 5.07102 3.78274 110 a - - -
2.68618 4.42225 2.77520 2.73123 3.46354 2.40513 3.72494
3.29354 2.67741 2.69355 4.24690 2.90347 2.73740 3.18146 2.89801
2.37886 2.77519 2.98518 4.58477 3.61503
0.05271 3.32757 4.16901 0.64482 0.74393 0.48576 0.95510
107 2.17282 4.12673 2.98949 2.80516 3.58154 2.93133 3.88270
2.78929 2.76254 2.57673 3.72815 3.08484 3.45317 3.20409 3.02437
2.43395 2.66065 2.53237 5.10310 3.76872 115 a - - -

```
          2.68618  4.42225  2.77519  2.73123  3.46354  2.40513  3.72494
3.29354  2.67741  2.69355  4.24690  2.90347  2.73739  3.18146  2.89801
2.37887  2.77519  2.98518  4.58477  3.61503
          0.06268  3.44667  3.54383  0.61958  0.77255  0.48576  0.95510
    108   2.39805  4.23606  2.92945  2.71652  3.30108  3.00100  3.71398
2.94758  2.56532  2.67283  3.76773  2.97701  3.63968  3.07797  2.66569
2.52530  2.79122  2.69235  4.82624  3.42047    116 a - - -
          2.68618  4.42225  2.77519  2.73123  3.46354  2.40513  3.72494
3.29354  2.67741  2.69355  4.24690  2.90347  2.73739  3.18146  2.89801
2.37887  2.77519  2.98518  4.58477  3.61503
          0.05251  3.43314  3.97003  0.61958  0.77255  0.49375  0.94247
    109   2.49197  4.26615  2.38588  2.37271  3.59758  2.88062  3.69660
3.02862  2.65075  2.80920  3.89535  2.90589  3.52158  3.04110  2.94334
2.61872  2.86909  2.76736  4.88912  3.67351    117 e - - -
          2.68618  4.42225  2.77518  2.73123  3.46354  2.40513  3.72495
3.29351  2.67741  2.69355  4.24690  2.90347  2.73740  3.18146  2.89801
2.37887  2.77519  2.98518  4.58477  3.61503
          0.07463  3.10814  3.60335  0.62444  0.76693  0.48267  0.96007
    110   2.24440  4.12608  2.79323  2.68059  3.60479  2.79832  3.80363
2.97619  2.71542  2.78737  3.85135  2.99588  3.34736  3.13956  2.98357
2.40814  2.67786  2.67411  5.02467  3.71289    120 a - - -
          2.68618  4.42225  2.77519  2.73123  3.46354  2.40513  3.72494
3.29354  2.67741  2.69355  4.24690  2.90347  2.73739  3.18146  2.89801
2.37887  2.77519  2.98518  4.58477  3.61503
          0.04920  3.43210  4.15444  0.61958  0.77255  0.48043  0.96369
    111   2.36727  4.23484  2.75994  2.49313  3.70995  2.83768  3.77402
2.95248  2.61467  2.78933  3.84474  2.97062  3.60203  3.06955  2.89436
2.51785  2.60906  2.67369  5.10015  3.81971    121 a - - -
          2.68618  4.42225  2.77519  2.73123  3.46354  2.40513  3.72494
3.29354  2.67741  2.69355  4.24690  2.90347  2.73739  3.18146  2.89801
2.37887  2.77519  2.98518  4.58477  3.61503
          0.04848  3.44667  4.16901  0.61958  0.77255  0.48576  0.95510
    112   2.37987  4.12600  3.19946  2.88687  3.16002  3.12540  3.78561
2.64164  2.71223  2.46434  3.60730  3.06251  3.73973  3.15042  2.97811
2.55779  2.74698  2.44930  4.79827  3.33980    122 a - - -
          2.68618  4.42225  2.77519  2.73123  3.46354  2.40513  3.72494
3.29354  2.67741  2.69355  4.24690  2.90347  2.73739  3.18146  2.89801
2.37887  2.77519  2.98518  4.58477  3.61503
          0.04848  3.44667  4.16901  0.61958  0.77255  0.48576  0.95510
    113   2.64238  4.37778  3.25991  2.93583  2.61640  3.39223  3.59508
2.64497  2.79641  2.24681  3.45483  3.25722  3.90703  3.21307  3.06567
2.80115  2.95817  2.52906  4.34193  2.65350    123 l - - -
          2.68618  4.42225  2.77519  2.73123  3.46354  2.40513  3.72494
3.29354  2.67741  2.69355  4.24690  2.90347  2.73739  3.18146  2.89801
2.37887  2.77519  2.98518  4.58477  3.61503
          0.04848  3.44667  4.16901  0.61958  0.77255  0.48576  0.95510
    114   2.32295  4.15252  2.87057  2.70363  3.56814  2.88280  3.79557
3.00747  2.69171  2.68071  3.79000  3.02690  2.91628  3.06962  2.95114
2.48704  2.70556  2.73440  4.97563  3.70625    124 a - - -
          2.68618  4.42225  2.77519  2.73123  3.46354  2.40513  3.72494
3.29354  2.67741  2.69355  4.24690  2.90347  2.73739  3.18146  2.89801
2.37887  2.77519  2.98518  4.58477  3.61503
          0.04848  3.44667  4.16901  0.61958  0.77255  0.48576  0.95510
```

```
    115   2.25432  4.10831  2.99027  2.81805  3.56601  2.90580  3.88172
2.80252  2.77390  2.65338  3.76910  3.08605  3.60302  3.21880  3.02803
2.36147  2.48600  2.53407  5.07319  3.72905    125 a - - -
          2.68618  4.42225  2.77519  2.73123  3.46354  2.40513  3.72494
3.29354  2.67741  2.69355  4.24690  2.90347  2.73739  3.18146  2.89801
2.37887  2.77519  2.98518  4.58477  3.61503
          0.04848  3.44667  4.16901  0.61958  0.77255  0.48576  0.95510
    116   2.67100  4.32662  3.14266  2.90501  2.76585  3.21412  3.64677
2.73706  2.78299  2.31410  3.54759  3.23369  3.77502  3.22834  3.01820
2.84765  3.00524  2.60041  4.34618  2.40052    126 l - - -
          2.68618  4.42225  2.77519  2.73123  3.46354  2.40513  3.72494
3.29354  2.67741  2.69355  4.24690  2.90347  2.73739  3.18146  2.89801
2.37887  2.77519  2.98518  4.58477  3.61503
          0.04848  3.44667  4.16901  0.61958  0.77255  0.48576  0.95510
    117   2.51056  4.26504  3.40343  3.10722  3.15131  3.31293  3.96827
2.23287  2.89611  2.00995  3.36253  3.38760  3.89638  3.36971  3.13476
2.80348  2.92109  2.19394  4.85687  3.44862    127 l - - -
          2.68618  4.42225  2.77519  2.73123  3.46354  2.40513  3.72494
3.29354  2.67741  2.69355  4.24690  2.90347  2.73739  3.18146  2.89801
2.37887  2.77519  2.98518  4.58477  3.61503
          0.04848  3.44667  4.16901  0.61958  0.77255  0.48576  0.95510
    118   2.69125  4.36743  3.28030  2.98833  2.59636  3.36748  3.65725
2.61892  2.86000  2.19058  3.44143  3.30153  3.89261  3.27194  3.11147
2.88139  3.01134  2.50550  4.30377  2.49418    128 l - - -
          2.68618  4.42225  2.77519  2.73123  3.46354  2.40513  3.72494
3.29354  2.67741  2.69355  4.24690  2.90347  2.73739  3.18146  2.89801
2.37887  2.77519  2.98518  4.58477  3.61503
          0.04848  3.44667  4.16901  0.61958  0.77255  0.48576  0.95510
    119   2.53595  4.37723  2.74994  2.57556  3.47118  3.04081  3.67841
3.06211  2.44534  2.64442  3.77898  2.97620  3.65335  2.58768  2.73610
2.65765  2.89418  2.84254  4.89491  3.55551    129 k - - -
          2.68618  4.42225  2.77519  2.73123  3.46354  2.40513  3.72494
3.29354  2.67741  2.69355  4.24690  2.90347  2.73739  3.18146  2.89801
2.37887  2.77519  2.98518  4.58477  3.61503
          0.04848  3.44667  4.16901  0.61958  0.77255  0.48576  0.95510
    120   2.54283  4.29922  2.80624  2.62408  3.57318  2.97610  3.69246
3.01064  2.09585  2.71905  3.83360  3.01321  3.59068  3.02082  2.70765
2.68222  2.90286  2.77518  4.87989  3.66145    130 k - - -
          2.68618  4.42225  2.77519  2.73123  3.46354  2.40513  3.72494
3.29354  2.67741  2.69355  4.24690  2.90347  2.73739  3.18146  2.89801
2.37887  2.77519  2.98518  4.58477  3.61503
          0.04848  3.44667  4.16901  0.61958  0.77255  0.48576  0.95510
    121   2.54574  4.24296  3.48066  3.17557  3.14736  3.32481  4.01787
2.13044  2.97545  2.02901  3.33251  3.43757  3.91068  3.42787  3.21181
2.81499  2.92073  2.09215  4.87254  3.47492    131 l - - -
          2.68618  4.42225  2.77519  2.73123  3.46354  2.40513  3.72494
3.29354  2.67741  2.69355  4.24690  2.90347  2.73739  3.18146  2.89801
2.37887  2.77519  2.98518  4.58477  3.61503
          0.04848  3.44667  4.16901  0.61958  0.77255  0.48576  0.95510
    122   2.52888  4.40359  2.22191  2.25763  3.73601  2.93996  3.67688
3.15134  2.62161  2.92232  3.95703  2.82610  3.57199  2.97423  2.96402
2.61937  2.88704  2.87340  5.03377  3.77497    132 d - - -
          2.68618  4.42225  2.77519  2.73123  3.46354  2.40513  3.72494
3.29354  2.67741  2.69355  4.24690  2.90347  2.73739  3.18146  2.89801
2.37887  2.77519  2.98518  4.58477  3.61503
```

0.04848 3.44667 4.16901 0.61958 0.77255 0.48576 0.95510
123 2.51291 4.34504 2.64426 2.17427 3.64399 2.97543 3.71157
2.98178 2.56319 2.73410 3.84795 2.94145 3.59945 3.02404 2.84193
2.67005 2.90607 2.74838 4.97193 3.74427 133 e - - -
2.68618 4.42225 2.77519 2.73123 3.46354 2.40513 3.72494
3.29354 2.67741 2.69355 4.24690 2.90347 2.73739 3.18146 2.89801
2.37887 2.77519 2.98518 4.58477 3.61503
0.04848 3.44667 4.16901 0.61958 0.77255 0.48576 0.95510
124 2.25081 4.08814 2.90084 2.75674 3.52193 2.79550 3.81768
3.01129 2.76205 2.81056 3.88021 3.03634 3.53035 3.19108 3.01139
2.14311 2.69959 2.69712 4.94646 3.61412 134 s - - -
2.68618 4.42225 2.77519 2.73123 3.46354 2.40513 3.72494
3.29354 2.67741 2.69355 4.24690 2.90347 2.73739 3.18146 2.89801
2.37887 2.77519 2.98518 4.58477 3.61503
0.04848 3.44667 4.16901 0.61958 0.77255 0.48576 0.95510
125 2.47739 4.35150 2.83277 2.60279 3.60162 3.08850 3.72740
2.88256 2.32685 2.64584 3.75920 3.00595 3.70040 3.01070 2.73490
2.62008 2.78915 2.63168 5.04989 3.70969 135 k - - -
2.68618 4.42225 2.77519 2.73123 3.46354 2.40513 3.72494
3.29354 2.67741 2.69355 4.24690 2.90347 2.73739 3.18146 2.89801
2.37887 2.77519 2.98518 4.58477 3.61503
0.04848 3.44667 4.16901 0.61958 0.77255 0.48576 0.95510
126 2.60779 4.22059 3.29496 3.07074 3.08236 3.14663 3.88887
2.45605 2.89514 1.84735 3.43157 3.36130 3.74617 3.36991 3.09781
2.84937 2.98220 2.34315 4.66431 3.32605 136 l - - -
2.68618 4.42225 2.77519 2.73123 3.46354 2.40513 3.72494
3.29354 2.67741 2.69355 4.24690 2.90347 2.73739 3.18146 2.89801
2.37887 2.77519 2.98518 4.58477 3.61503
0.04848 3.44667 4.16901 0.61958 0.77255 0.48576 0.95510
127 2.54283 4.29922 2.80624 2.62408 3.57318 2.97610 3.69246
3.01064 2.09585 2.71905 3.83360 3.01321 3.59068 3.02082 2.70765
2.68222 2.90286 2.77518 4.87989 3.66145 137 k - - -
2.68618 4.42225 2.77519 2.73123 3.46354 2.40513 3.72494
3.29354 2.67741 2.69355 4.24690 2.90347 2.73739 3.18146 2.89801
2.37887 2.77519 2.98518 4.58477 3.61503
0.04848 3.44667 4.16901 0.61958 0.77255 0.48576 0.95510
128 2.25448 4.14711 2.85570 2.66651 3.62740 2.88130 3.80841
2.98930 2.69791 2.73886 3.81844 3.01233 3.03486 3.13259 2.96583
2.43136 2.72060 2.69983 5.04248 3.75757 138 a - - -
2.68618 4.42225 2.77519 2.73123 3.46354 2.40513 3.72494
3.29354 2.67741 2.69355 4.24690 2.90347 2.73739 3.18146 2.89801
2.37887 2.77519 2.98518 4.58477 3.61503
0.04848 3.44667 4.16901 0.61958 0.77255 0.48576 0.95510
129 2.58001 4.35205 2.99912 2.76416 2.89134 3.19021 3.61771
2.86377 2.65927 2.53455 3.67097 3.00131 3.76436 3.12017 2.92150
2.72927 2.92138 2.66578 4.45643 2.63117 139 l - - -
2.68618 4.42225 2.77519 2.73123 3.46354 2.40513 3.72494
3.29354 2.67741 2.69355 4.24690 2.90347 2.73739 3.18146 2.89801
2.37887 2.77519 2.98518 4.58477 3.61503
0.04848 3.44667 4.16901 0.61958 0.77255 0.48576 0.95510
130 2.32477 4.10199 3.05870 2.88846 3.50259 2.91659 3.91041
2.69332 2.83000 2.49906 3.68789 3.15692 3.59821 3.27653 3.06312
2.52783 2.35199 2.47008 4.98625 3.72681 140 a - - -

```
          2.68618  4.42225  2.77519  2.73123  3.46354  2.40513  3.72494
 3.29354  2.67741  2.69355  4.24690  2.90347  2.73739  3.18146  2.89801
 2.37887  2.77519  2.98518  4.58477  3.61503
          0.04848  3.44667  4.16901  0.61958  0.77255  0.48576  0.95510
    131   2.51989  4.21799  2.97482  2.74923  3.45306  2.93924  3.69587
 3.03192  2.48593  2.64008  3.78141  3.09297  3.55424  3.06326  2.18711
 2.67954  2.88963  2.80440  4.75964  3.55703    141 r - - -
          2.68618  4.42225  2.77519  2.73123  3.46354  2.40513  3.72494
 3.29354  2.67741  2.69355  4.24690  2.90347  2.73739  3.18146  2.89801
 2.37887  2.77519  2.98518  4.58477  3.61503
          0.04848  3.44667  4.16901  0.61958  0.77255  0.48576  0.95510
    132   2.67100  4.32662  3.14266  2.90501  2.76585  3.21412  3.64677
 2.73706  2.78299  2.31410  3.54759  3.23369  3.77502  3.22834  3.01820
 2.84765  3.00524  2.60041  4.34618  2.40052    142 l - - -
          2.68618  4.42225  2.77519  2.73123  3.46354  2.40513  3.72494
 3.29354  2.67741  2.69355  4.24690  2.90347  2.73739  3.18146  2.89801
 2.37887  2.77519  2.98518  4.58477  3.61503
          0.04848  3.44667  4.16901  0.61958  0.77255  0.48576  0.95510
    133   2.48680  4.36860  2.90688  2.65804  3.48434  3.14142  3.70859
 2.87937  2.40725  2.53793  3.68390  3.03221  3.73420  2.91509  2.67598
 2.62957  2.79558  2.68726  4.98016  3.59868    143 k - - -
          2.68618  4.42225  2.77519  2.73123  3.46354  2.40513  3.72494
 3.29354  2.67741  2.69355  4.24690  2.90347  2.73739  3.18146  2.89801
 2.37887  2.77519  2.98518  4.58477  3.61503
          0.04848  3.44667  4.16901  0.61958  0.77255  0.48576  0.95510
    134   2.51861  4.23979  3.42109  3.13526  3.13792  3.27537  3.98040
 2.31253  2.93173  2.01118  3.37034  3.39310  3.87709  3.39600  3.16559
 2.77091  2.90341  2.11356  4.84748  3.43062    144 l - - -
          2.68618  4.42225  2.77519  2.73123  3.46354  2.40513  3.72494
 3.29354  2.67741  2.69355  4.24690  2.90347  2.73739  3.18146  2.89801
 2.37887  2.77519  2.98518  4.58477  3.61503
          0.04848  3.44667  4.16901  0.61958  0.77255  0.48576  0.95510
    135   2.40034  4.28769  2.79563  2.61361  3.57935  2.99760  3.69723
 3.08894  2.45913  2.81521  3.84414  2.97581  3.63173  3.00032  2.56460
 2.45267  2.78367  2.79920  4.98550  3.63008    145 a - - -
          2.68618  4.42225  2.77519  2.73123  3.46354  2.40513  3.72494
 3.29354  2.67741  2.69355  4.24690  2.90347  2.73739  3.18146  2.89801
 2.37887  2.77519  2.98518  4.58477  3.61503
          0.04848  3.44667  4.16901  0.61958  0.77255  0.48576  0.95510
    136   2.33836  3.98629  2.87374  2.76616  3.60078  2.00361  3.79029
 3.15638  2.82691  2.87178  3.96579  3.06707  3.35543  3.23202  3.02483
 2.52219  2.78238  2.83289  4.74403  3.71835    146 g - - -
          2.68618  4.42225  2.77519  2.73123  3.46354  2.40513  3.72494
 3.29354  2.67741  2.69355  4.24690  2.90347  2.73739  3.18146  2.89801
 2.37887  2.77519  2.98518  4.58477  3.61503
          0.04848  3.44667  4.16901  0.61958  0.77255  0.48576  0.95510
    137   2.67872  4.29762  3.43457  3.15470  2.39435  3.35049  3.79522
 2.43818  3.06546  2.01534  3.32823  3.42864  3.89788  3.42766  3.29776
 2.91846  3.02331  2.35313  4.38559  2.83585    147 l - - -
          2.68618  4.42225  2.77519  2.73123  3.46354  2.40513  3.72494
 3.29354  2.67741  2.69355  4.24690  2.90347  2.73739  3.18146  2.89801
 2.37887  2.77519  2.98518  4.58477  3.61503
          0.04848  3.44667  4.16901  0.61958  0.77255  0.48576  0.95510
```

```
    138   2.53386  4.23668  3.48281  3.17661  3.15648  3.31590  4.02093
2.15190  2.97659  2.03593  3.33709  3.43450  3.90577  3.42798  3.21347
2.80214  2.91182  2.07723  4.88228  3.48502    148 l - - -
          2.68618  4.42225  2.77519  2.73123  3.46354  2.40513  3.72494
3.29354  2.67741  2.69355  4.24690  2.90347  2.73739  3.18146  2.89801
2.37887  2.77519  2.98518  4.58477  3.61503
          0.04848  3.44667  4.16901  0.61958  0.77255  0.48576  0.95510
    139   2.29458  4.24894  2.71113  2.48616  3.74058  2.79727  3.75388
3.04610  2.56904  2.87474  3.88975  2.93328  3.59819  3.04115  2.87895
2.46220  2.75210  2.74105  5.12943  3.81599    149 a - - -
          2.68618  4.42225  2.77519  2.73123  3.46354  2.40513  3.72494
3.29354  2.67741  2.69355  4.24690  2.90347  2.73739  3.18146  2.89801
2.37887  2.77519  2.98518  4.58477  3.61503
          0.04848  3.44667  4.16901  0.61958  0.77255  0.48576  0.95510
    140   2.40249  4.35856  2.60778  2.45182  3.59780  2.94278  3.55983
3.27092  2.49854  2.96394  3.92530  2.84206  3.62917  2.89866  2.80077
2.44010  2.78197  2.93774  5.01656  3.59661    150 a - - -
          2.68618  4.42225  2.77519  2.73123  3.46354  2.40513  3.72494
3.29354  2.67741  2.69355  4.24690  2.90347  2.73739  3.18146  2.89801
2.37887  2.77519  2.98518  4.58477  3.61503
          0.04848  3.44667  4.16901  0.61958  0.77255  0.48576  0.95510
    141   2.23804  4.08352  2.88489  2.74160  3.55853  2.74653  3.81551
3.04471  2.75437  2.85033  3.89999  3.02287  3.52184  3.18083  3.00673
2.15583  2.68812  2.71698  4.96973  3.64482    151 s - - -
          2.68618  4.42225  2.77519  2.73123  3.46354  2.40513  3.72494
3.29354  2.67741  2.69355  4.24690  2.90347  2.73739  3.18146  2.89801
2.37887  2.77519  2.98518  4.58477  3.61503
          0.04848  3.44667  4.16901  0.61958  0.77255  0.48576  0.95510
    142   2.54180  4.49127  2.60115  2.36490  3.71564  3.08369  3.63597
3.17313  2.32053  2.86846  3.88592  2.84330  3.68290  2.78684  2.66183
2.63096  2.87747  2.90302  5.08766  3.73430    152 k - - -
          2.68618  4.42225  2.77519  2.73123  3.46354  2.40513  3.72494
3.29354  2.67741  2.69355  4.24690  2.90347  2.73739  3.18146  2.89801
2.37887  2.77519  2.98518  4.58477  3.61503
          0.04848  3.44667  4.16901  0.61958  0.77255  0.48576  0.95510
    143   2.45572  4.40234  2.31221  2.36651  3.64868  2.89669  3.58402
3.27655  2.59387  2.98375  3.96659  2.78785  3.59540  2.91796  2.93995
2.54482  2.82709  2.95753  5.02682  3.65032    153 d - - -
          2.68618  4.42225  2.77519  2.73123  3.46354  2.40513  3.72494
3.29354  2.67741  2.69355  4.24690  2.90347  2.73739  3.18146  2.89801
2.37887  2.77519  2.98518  4.58477  3.61503
          0.04848  3.44667  4.16901  0.61958  0.77255  0.48576  0.95510
    144   2.55765  4.25281  3.35909  3.09885  3.09506  3.24766  3.93463
2.38196  2.90124  1.93868  3.38721  3.37310  3.84571  3.37504  3.12615
2.80145  2.93689  2.21668  4.76842  3.35999    154 l - - -
          2.68618  4.42225  2.77519  2.73123  3.46354  2.40513  3.72494
3.29354  2.67741  2.69355  4.24690  2.90347  2.73739  3.18146  2.89801
2.37887  2.77519  2.98518  4.58477  3.61503
          0.04848  3.44667  4.16901  0.61958  0.77255  0.48576  0.95510
    145   2.56125  4.35645  2.55503  2.11873  3.67459  2.95302  3.68852
3.05402  2.55841  2.81030  3.89687  2.90916  3.57527  3.00213  2.84524
2.67444  2.91577  2.80625  4.95707  3.74859    155 e - - -
          2.68618  4.42225  2.77519  2.73123  3.46354  2.40513  3.72494
3.29354  2.67741  2.69355  4.24690  2.90347  2.73739  3.18146  2.89801
2.37887  2.77519  2.98518  4.58477  3.61503
```

```
          0.04848  3.44667  4.16901  0.61958  0.77255  0.48576  0.95510
    146   2.56166  4.27851  3.49079  3.16497  3.05904  3.38086  3.98419
2.20561  2.94998  1.96189  3.31122  3.43794  3.94645  3.40552  3.19224
2.84039  2.93845  2.13619  4.81888  3.38530    156 l - - -
          2.68618  4.42225  2.77519  2.73123  3.46354  2.40513  3.72494
3.29354  2.67741  2.69355  4.24690  2.90347  2.73739  3.18146  2.89801
2.37887  2.77519  2.98518  4.58477  3.61503
          0.04848  3.44667  4.16901  0.61958  0.77255  0.48576  0.95510
    147   2.54821  4.32189  3.12547  2.87580  2.99410  3.30028  3.76114
2.53315  2.77044  2.29072  3.48190  3.21706  3.85339  3.19778  3.05252
2.74362  2.88726  2.33229  4.65679  3.03633    157 l - - -
          2.68618  4.42225  2.77519  2.73123  3.46354  2.40513  3.72494
3.29354  2.67741  2.69355  4.24690  2.90347  2.73739  3.18146  2.89801
2.37887  2.77519  2.98518  4.58477  3.61503
          0.04848  3.44667  4.16901  0.61958  0.77255  0.48576  0.95510
    148   2.51787  4.24380  3.44953  3.14301  3.16607  3.30478  3.99705
2.25297  2.92843  2.02579  3.35649  3.40336  3.89858  3.39390  3.16767
2.77505  2.87692  2.10968  4.88918  3.47793    158 l - - -
          2.68618  4.42225  2.77519  2.73123  3.46354  2.40513  3.72494
3.29354  2.67741  2.69355  4.24690  2.90347  2.73739  3.18146  2.89801
2.37887  2.77519  2.98518  4.58477  3.61503
          0.04848  3.44667  4.16901  0.61958  0.77255  0.48576  0.95510
    149   2.34361  4.16335  3.18124  2.93115  3.34531  3.06815  3.88449
2.50970  2.79027  2.44141  3.60754  3.19387  3.72161  3.25182  3.04376
2.44611  2.73892  2.35266  4.94439  3.52847    159 a - - -
          2.68618  4.42225  2.77519  2.73123  3.46354  2.40513  3.72494
3.29354  2.67741  2.69355  4.24690  2.90347  2.73739  3.18146  2.89801
2.37887  2.77519  2.98518  4.58477  3.61503
          0.04848  3.44667  4.16901  0.61958  0.77255  0.48576  0.95510
    150   2.51675  4.28507  2.93046  2.69291  3.42738  2.99870  3.56345
3.10357  2.42459  2.68747  3.79301  3.04956  3.60410  3.00818  2.26819
2.65795  2.87618  2.86237  4.78280  3.49391    160 r - - -
          2.68618  4.42225  2.77519  2.73123  3.46354  2.40513  3.72494
3.29354  2.67741  2.69355  4.24690  2.90347  2.73739  3.18146  2.89801
2.37887  2.77519  2.98518  4.58477  3.61503
          0.04848  3.44667  4.16901  0.61958  0.77255  0.48576  0.95510
    151   2.66015  4.39485  3.05441  2.80362  2.73802  3.25481  3.45865
2.89792  2.66848  2.43678  3.61160  3.15168  3.80122  3.13143  2.92231
2.80248  2.98052  2.73504  4.32251  2.47570    161 l - - -
          2.68618  4.42225  2.77519  2.73123  3.46354  2.40513  3.72494
3.29354  2.67741  2.69355  4.24690  2.90347  2.73739  3.18146  2.89801
2.37887  2.77519  2.98518  4.58477  3.61503
          0.04848  3.44667  4.16901  0.61958  0.77255  0.48576  0.95510
    152   2.37333  4.28840  2.56501  2.44579  3.68364  2.68278  3.62235
3.25700  2.57777  2.98028  3.95013  2.84294  3.57049  2.96291  2.88357
2.49079  2.76848  2.91613  5.04109  3.70437    162 a - - -
          2.68618  4.42225  2.77519  2.73123  3.46354  2.40513  3.72494
3.29354  2.67741  2.69355  4.24690  2.90347  2.73739  3.18146  2.89801
2.37887  2.77519  2.98518  4.58477  3.61503
          0.06062  3.44667  3.61324  0.61958  0.77255  0.48576  0.95510
    153   2.38188  4.30970  2.72658  2.54509  3.60219  2.96051  3.70789
3.03912  2.44780  2.79871  3.85142  2.90465  3.63497  2.94451  2.78111
2.54682  2.80083  2.76246  5.02520  3.66331    163 a - - -
```

```
          2.68618  4.42225  2.77519  2.73123  3.46354  2.40513  3.72494
 3.29354  2.67741  2.69355  4.24690  2.90347  2.73739  3.18146  2.89801
 2.37887  2.77519  2.98518  4.58477  3.61503
          0.04905  3.43510  4.15745  0.61958  0.77255  0.48151  0.96194
    154   2.45644  4.41016  2.63548  2.35792  3.73736  3.04945  3.69397
 3.02439  2.42085  2.82902  3.86588  2.87546  3.66999  2.95890  2.76158
 2.60016  2.82346  2.75987  5.12988  3.79564    164 e - - -
          2.68618  4.42225  2.77519  2.73123  3.46354  2.40513  3.72494
 3.29354  2.67741  2.69355  4.24690  2.90347  2.73739  3.18146  2.89801
 2.37887  2.77519  2.98518  4.58477  3.61503
          0.04848  3.44667  4.16901  0.61958  0.77255  0.48576  0.95510
    155   2.39205  4.23339  3.12898  2.85369  3.28817  3.12787  3.70601
 2.60630  2.68139  2.43424  3.58604  3.13148  3.74609  3.16528  2.94156
 2.59355  2.73681  2.43216  4.87199  3.44135    165 a - - -
          2.68618  4.42225  2.77519  2.73123  3.46354  2.40513  3.72494
 3.29354  2.67741  2.69355  4.24690  2.90347  2.73739  3.18146  2.89801
 2.37887  2.77519  2.98518  4.58477  3.61503
          0.04848  3.44667  4.16901  0.61958  0.77255  0.48576  0.95510
    156   2.54453  4.50507  2.50654  2.38372  3.51190  3.08561  3.35673
 3.28712  2.42660  2.90379  3.88885  2.84863  3.67560  2.84221  2.70199
 2.62077  2.87204  2.99871  4.92722  3.49814    166 e - - -
          2.68618  4.42225  2.77519  2.73123  3.46354  2.40513  3.72494
 3.29354  2.67741  2.69355  4.24690  2.90347  2.73739  3.18146  2.89801
 2.37887  2.77519  2.98518  4.58477  3.61503
          0.04848  3.44667  4.16901  0.61958  0.77255  0.48576  0.95510
    157   2.37107  4.25132  2.94756  2.74432  3.53310  3.08600  3.81894
 2.66196  2.58291  2.53884  3.67303  3.08426  3.71668  3.12000  2.91086
 2.57158  2.70082  2.45577  5.06625  3.70061    167 a - - -
          2.68618  4.42225  2.77519  2.73123  3.46354  2.40513  3.72494
 3.29354  2.67741  2.69355  4.24690  2.90347  2.73739  3.18146  2.89801
 2.37887  2.77519  2.98518  4.58477  3.61503
          0.04848  3.44667  4.16901  0.61958  0.77255  0.48576  0.95510
    158   2.45191  4.22541  3.33722  3.01933  3.22771  3.22228  3.89718
 2.40459  2.79033  2.24275  3.42858  3.25022  3.82494  3.27619  3.01472
 2.67831  2.83257  2.20228  4.87683  3.45338    168 v - - -
          2.68618  4.42225  2.77519  2.73123  3.46354  2.40513  3.72494
 3.29354  2.67741  2.69355  4.24690  2.90347  2.73739  3.18146  2.89801
 2.37887  2.77519  2.98518  4.58477  3.61503
          0.05456  3.44667  3.85157  0.61958  0.77255  0.48576  0.95510
    159   2.54858  4.29349  3.33734  3.00853  3.19489  3.30929  3.88883
 2.41623  2.70698  2.10395  3.15977  3.31558  3.87356  3.25500  2.98890
 2.77695  2.90170  2.27627  4.86151  3.46460    169 l - - -
          2.68618  4.42225  2.77519  2.73123  3.46354  2.40513  3.72494
 3.29354  2.67741  2.69355  4.24690  2.90347  2.73739  3.18146  2.89801
 2.37887  2.77519  2.98518  4.58477  3.61503
          0.04876  3.44087  4.16322  0.61958  0.77255  0.48362  0.95854
    160   2.41266  4.33277  2.56270  2.47578  3.56968  2.95016  3.67922
 3.20865  2.56685  2.94377  3.94827  2.61842  3.60296  2.92964  2.86998
 2.52486  2.80264  2.89180  4.97930  3.58041    170 a - - -
          2.68618  4.42225  2.77519  2.73123  3.46354  2.40513  3.72494
 3.29354  2.67741  2.69355  4.24690  2.90347  2.73739  3.18146  2.89801
 2.37887  2.77519  2.98518  4.58477  3.61503
          0.04848  3.44667  4.16901  0.61958  0.77255  0.48576  0.95510
```

161 2.44875 4.17787 3.37861 3.12443 3.21923 3.16920 4.00169
2.34091 2.96824 2.14508 3.42746 3.36322 3.80321 3.41200 3.19855
2.69780 2.85537 2.04045 4.89584 3.51708 171 v - - -
2.68618 4.42225 2.77519 2.73123 3.46354 2.40513 3.72494
3.29354 2.67741 2.69355 4.24690 2.90347 2.73739 3.18146 2.89801
2.37887 2.77519 2.98518 4.58477 3.61503
0.04848 3.44667 4.16901 0.61958 0.77255 0.48576 0.95510
162 2.33320 4.16279 2.83691 2.67002 3.51930 2.87416 3.63031
3.11060 2.66133 2.76467 3.83745 3.00243 2.87835 3.10485 2.92238
2.46375 2.75046 2.81337 4.90641 3.60886 172 a - - -
2.68618 4.42225 2.77519 2.73123 3.46354 2.40513 3.72494
3.29354 2.67741 2.69355 4.24690 2.90347 2.73739 3.18146 2.89801
2.37887 2.77519 2.98518 4.58477 3.61503
0.04848 3.44667 4.16901 0.61958 0.77255 0.48576 0.95510
163 2.44814 4.34270 2.69925 2.56002 3.35681 2.97803 3.66156
3.01116 2.57679 2.76365 3.78546 2.93594 3.67620 3.00894 2.85544
2.59035 2.81741 2.74556 4.83019 3.18651 173 a - - -
2.68618 4.42225 2.77519 2.73123 3.46354 2.40513 3.72494
3.29354 2.67741 2.69355 4.24690 2.90347 2.73739 3.18146 2.89801
2.37887 2.77519 2.98518 4.58477 3.61503
0.04848 3.44667 4.16901 0.61958 0.77255 0.48576 0.95510
164 2.37595 4.35090 2.45957 2.38166 3.77353 2.92601 3.70334
3.16372 2.58981 2.97348 3.94402 2.77388 3.55481 2.97668 2.92501
2.50316 2.75710 2.84335 5.15630 3.80039 174 a - - -
2.68618 4.42225 2.77519 2.73123 3.46354 2.40513 3.72494
3.29354 2.67741 2.69355 4.24690 2.90347 2.73739 3.18146 2.89801
2.37887 2.77519 2.98518 4.58477 3.61503
0.05458 3.44667 3.85073 0.61958 0.77255 0.48576 0.95510
165 2.49489 4.29569 2.89695 2.60807 3.35347 3.01482 3.69950
2.83896 2.55548 2.48947 3.68503 3.06192 3.68537 3.04957 2.82237
2.65234 2.84270 2.61694 4.63607 3.44675 175 l - - -
2.68618 4.42225 2.77519 2.73123 3.46354 2.40513 3.72494
3.29354 2.67741 2.69355 4.24690 2.90347 2.73739 3.18146 2.89801
2.37887 2.77519 2.98518 4.58477 3.61503
0.04876 3.44085 4.16320 0.61958 0.77255 0.48922 0.94961
166 2.29321 4.23401 2.76450 2.58934 3.61498 2.97263 3.77796
2.90992 2.64008 2.73800 3.81093 2.95685 3.53013 3.08042 2.92998
2.47561 2.74438 2.61297 5.08439 3.71571 176 a - - -
2.68618 4.42225 2.77519 2.73123 3.46354 2.40513 3.72494
3.29354 2.67741 2.69354 4.24690 2.90347 2.73740 3.18146 2.89801
2.37887 2.77519 2.98518 4.58474 3.61503
0.05629 3.23971 4.16320 0.62251 0.76915 0.48361 0.95855
167 2.52315 4.30461 3.21558 2.90761 3.07477 3.26678 3.78767
2.54491 2.71264 2.20364 3.47394 3.22863 3.73460 3.19272 2.94945
2.70258 2.87490 2.41979 4.73226 3.21364 179 l - - -
2.68618 4.42225 2.77519 2.73123 3.46354 2.40513 3.72494
3.29354 2.67741 2.69355 4.24690 2.90347 2.73739 3.18146 2.89801
2.37887 2.77519 2.98518 4.58477 3.61503
0.06268 3.44667 3.54383 0.61958 0.77255 0.48576 0.95510
168 2.44695 4.24513 2.96665 2.80878 3.25216 3.10393 3.79167
2.57238 2.71849 2.40829 3.63814 3.08957 3.72714 3.18103 2.98128
2.63376 2.83128 2.43136 4.81605 3.38080 180 l - - -
2.68618 4.42225 2.77519 2.73123 3.46354 2.40513 3.72494
3.29354 2.67741 2.69355 4.24690 2.90347 2.73739 3.18146 2.89801
2.37887 2.77519 2.98518 4.58477 3.61503

0.04915 3.43314 4.15549 0.61958 0.77255 0.49375 0.94247
169 2.34165 4.21828 2.83718 2.68755 3.42964 2.98092 3.77226
2.88802 2.66910 2.66820 3.76656 3.01911 3.59702 3.08944 2.94425
2.43092 2.73031 2.60998 4.94971 3.48614 181 a - - -
2.68618 4.42225 2.77519 2.73123 3.46354 2.40513 3.72494
3.29354 2.67741 2.69355 4.24690 2.90347 2.73739 3.18146 2.89801
2.37887 2.77519 2.98518 4.58477 3.61503
0.04915 3.43314 4.15549 0.61958 0.77255 0.49375 0.94247
170 2.36913 4.25448 2.74532 2.62983 3.44361 2.99841 3.74905
2.89406 2.66020 2.66951 3.76134 2.99405 3.49390 3.08820 2.94974
2.52005 2.75699 2.64194 4.93866 3.44172 182 a - - -
2.68618 4.42222 2.77520 2.73122 3.46354 2.40513 3.72495
3.29352 2.67741 2.69355 4.24690 2.90346 2.73739 3.18146 2.89801
2.37886 2.77520 2.98518 4.58478 3.61504
0.06897 3.06015 3.92401 0.78193 0.61161 0.48080 0.96308
171 2.27626 4.14073 2.91772 2.73646 3.52644 2.90389 3.80595
2.95150 2.70101 2.69696 3.79792 3.00447 3.17533 3.14610 2.96502
2.41070 2.70657 2.63942 4.99601 3.63923 196 a - - -
2.68618 4.42225 2.77519 2.73123 3.46354 2.40513 3.72494
3.29354 2.67741 2.69355 4.24690 2.90347 2.73739 3.18146 2.89801
2.37887 2.77519 2.98518 4.58477 3.61503
0.04868 3.44263 4.16498 0.61958 0.77255 0.48816 0.95128
172 2.45584 4.29253 2.99975 2.75373 3.31203 3.17510 3.72094
2.62242 2.63394 2.44198 3.55820 3.11733 3.76691 3.11092 2.91121
2.61892 2.80057 2.46195 4.87968 3.44790 197 l - - -
2.68618 4.42225 2.77519 2.73123 3.46354 2.40513 3.72494
3.29354 2.67741 2.69355 4.24690 2.90347 2.73739 3.18146 2.89801
2.37887 2.77519 2.98518 4.58477 3.61503
0.05152 3.44263 4.00439 0.61958 0.77255 0.48816 0.95128
173 2.31567 4.21097 2.76129 2.63351 3.51627 2.92429 3.76396
2.95838 2.65357 2.80587 3.83424 2.92911 3.61726 3.08439 2.91800
2.45011 2.64075 2.66340 5.02486 3.63146 198 a - - -
2.68618 4.42225 2.77519 2.73123 3.46354 2.40513 3.72494
3.29354 2.67741 2.69355 4.24690 2.90347 2.73739 3.18146 2.89801
2.37887 2.77519 2.98518 4.58477 3.61503
0.04881 3.43992 4.16227 0.61958 0.77255 0.48716 0.95288
174 2.25780 4.16418 2.81839 2.68391 3.59565 2.90712 3.80846
2.94645 2.67048 2.79012 3.84674 2.99979 3.59624 3.13168 2.96739
2.30745 2.66821 2.64858 5.06930 3.69374 199 a - - -
2.68618 4.42225 2.77519 2.73123 3.46354 2.40513 3.72494
3.29354 2.67741 2.69355 4.24690 2.90347 2.73739 3.18146 2.89801
2.37887 2.77519 2.98518 4.58477 3.61503
0.04868 3.44263 4.16498 0.61958 0.77255 0.48816 0.95128
175 2.26179 3.46553 3.04178 2.85862 3.49238 2.93531 3.84925
2.73017 2.79294 2.64759 3.69318 3.10427 3.59143 3.20983 3.05306
2.46576 2.67694 2.45913 4.93600 3.64678 200 a - - -
2.68618 4.42225 2.77519 2.73123 3.46354 2.40513 3.72494
3.29354 2.67741 2.69355 4.24690 2.90347 2.73739 3.18146 2.89801
2.37887 2.77519 2.98518 4.58477 3.61503
0.05206 3.44263 3.97647 0.61958 0.77255 0.48427 0.95750
176 2.27894 4.19153 2.81400 2.60247 3.63151 2.78168 3.77529
2.95437 2.63555 2.84235 3.86035 2.95055 3.59988 3.08547 2.91499
2.41711 2.71467 2.68494 5.07192 3.70921 201 a - - -

2.68618 4.42225 2.77519 2.73123 3.46354 2.40513 3.72494
3.29354 2.67741 2.69355 4.24690 2.90347 2.73739 3.18146 2.89801
2.37887 2.77519 2.98518 4.58477 3.61503
0.04864 3.44345 4.16580 0.61958 0.77255 0.48457 0.95701
177 2.31328 4.24144 2.76824 2.56666 3.65031 2.89650 3.74359
3.00283 2.57899 2.86391 3.85544 2.93110 3.62411 3.03705 2.83885
2.42100 2.72617 2.70112 5.08691 3.71113 202 a - - -
2.68618 4.42225 2.77519 2.73123 3.46354 2.40513 3.72494
3.29354 2.67741 2.69355 4.24690 2.90347 2.73739 3.18146 2.89801
2.37887 2.77519 2.98518 4.58477 3.61503
0.04848 3.44667 4.16901 0.61958 0.77255 0.48576 0.95510
178 2.41225 4.25318 3.06634 2.79588 3.26743 3.12467 3.77937
2.68085 2.66926 2.43642 3.58937 3.12222 3.50585 3.09700 2.94287
2.56088 2.78937 2.53338 4.86989 3.41172 203 a - - -
2.68618 4.42225 2.77519 2.73123 3.46354 2.40513 3.72494
3.29354 2.67741 2.69355 4.24690 2.90347 2.73739 3.18146 2.89801
2.37887 2.77519 2.98518 4.58477 3.61503
0.04848 3.44667 4.16901 0.61958 0.77255 0.48576 0.95510
179 2.36667 4.31029 2.59581 2.49902 3.56908 2.96289 3.64812
3.16931 2.58539 2.93707 3.91971 2.73171 3.61932 3.00494 2.89663
2.47068 2.73716 2.82107 5.01519 3.58454 204 a - - -
2.68618 4.42225 2.77519 2.73123 3.46354 2.40513 3.72494
3.29354 2.67741 2.69355 4.24690 2.90347 2.73739 3.18146 2.89801
2.37887 2.77519 2.98518 4.58477 3.61503
0.04848 3.44667 4.16901 0.61958 0.77255 0.48576 0.95510
180 2.35555 3.71870 3.22020 2.83838 3.47038 3.00816 3.78408
2.81379 2.59120 2.56525 3.65283 3.15995 3.62927 3.12126 2.58749
2.54724 2.74345 2.57073 4.89835 3.62590 205 a - - -
2.68618 4.42225 2.77519 2.73123 3.46354 2.40513 3.72494
3.29354 2.67741 2.69355 4.24690 2.90347 2.73739 3.18146 2.89801
2.37887 2.77519 2.98518 4.58477 3.61503
0.04848 3.44667 4.16901 0.61958 0.77255 0.48576 0.95510
181 2.27440 2.77977 3.50507 3.22957 3.37093 2.86610 3.98765
2.54605 3.05371 2.41406 3.59295 3.35582 3.54323 3.45824 3.22969
2.52853 2.71816 2.32436 4.81160 3.63071 206 a - - -
2.68618 4.42225 2.77519 2.73123 3.46354 2.40513 3.72494
3.29354 2.67741 2.69355 4.24690 2.90347 2.73739 3.18146 2.89801
2.37887 2.77519 2.98518 4.58477 3.61503
0.04848 3.44667 4.16901 0.61958 0.77255 0.48576 0.95510
182 2.37986 4.35001 2.68970 2.49339 3.70754 3.03619 3.71546
3.01122 2.44292 2.80149 3.82988 2.92732 3.61544 2.95810 2.79499
2.53516 2.79537 2.71784 5.13192 3.78155 207 a - - -
2.68618 4.42225 2.77519 2.73123 3.46354 2.40513 3.72494
3.29354 2.67741 2.69355 4.24690 2.90347 2.73739 3.18146 2.89801
2.37887 2.77519 2.98518 4.58477 3.61503
0.04848 3.44667 4.16901 0.61958 0.77255 0.48576 0.95510
183 2.32317 4.24782 2.77581 2.56559 3.68289 2.96225 3.76536
3.01924 2.59006 2.76168 3.81358 2.96094 3.37689 2.99787 2.87593
2.45961 2.68393 2.73338 5.12842 3.79063 208 a - - -
2.68618 4.42225 2.77519 2.73123 3.46354 2.40513 3.72494
3.29354 2.67741 2.69355 4.24690 2.90347 2.73739 3.18146 2.89801
2.37887 2.77519 2.98518 4.58477 3.61503
0.04848 3.44667 4.16901 0.61958 0.77255 0.48576 0.95510

184 2.43742 4.40451 2.67655 2.47309 3.58654 3.04958 3.55231
3.22677 2.39882 2.89169 3.87973 2.87465 3.66707 2.86583 2.68240
2.50508 2.81372 2.92375 5.00691 3.59384 209 k - - -
2.68618 4.42225 2.77519 2.73123 3.46354 2.40513 3.72494
3.29354 2.67741 2.69355 4.24690 2.90347 2.73739 3.18146 2.89801
2.37887 2.77519 2.98518 4.58477 3.61503
0.04848 3.44667 4.16901 0.61958 0.77255 0.48576 0.95510
185 2.40154 4.26822 3.05330 2.79742 3.34670 3.13211 3.78675
2.64565 2.61093 2.43787 3.62705 3.10000 3.74740 3.07744 2.88735
2.57652 2.80707 2.49355 4.91213 3.48799 210 a - - -
2.68618 4.42225 2.77519 2.73123 3.46354 2.40513 3.72494
3.29354 2.67741 2.69355 4.24690 2.90347 2.73739 3.18146 2.89801
2.37887 2.77519 2.98518 4.58477 3.61503
0.05510 3.44667 3.82783 0.61958 0.77255 0.48576 0.95510
186 2.43311 4.19090 2.98864 2.67781 3.37850 3.14813 3.74150
2.74551 2.59435 2.54938 3.58744 3.08343 3.67445 3.06836 2.87812
2.58759 2.78907 2.51048 4.90349 3.40086 211 a - - -
2.68618 4.42225 2.77519 2.73123 3.46354 2.40513 3.72494
3.29354 2.67741 2.69355 4.24690 2.90347 2.73739 3.18146 2.89801
2.37887 2.77519 2.98518 4.58477 3.61503
0.05590 3.44036 3.80264 0.61958 0.77255 0.48951 0.94914
187 2.43000 4.22081 3.20078 2.94798 3.16758 3.14370 3.87174
2.52812 2.80318 2.23567 3.48596 3.23124 3.67098 3.22201 3.05375
2.63833 2.82554 2.33635 4.84452 3.41872 212 l - - -
2.68618 4.42225 2.77519 2.73123 3.46354 2.40513 3.72494
3.29354 2.67741 2.69355 4.24690 2.90347 2.73739 3.18146 2.89801
2.37887 2.77519 2.98518 4.58477 3.61503
0.04913 3.43359 4.15594 0.61958 0.77255 0.49348 0.94288
188 2.27188 4.04641 2.98465 2.80879 3.42994 2.98342 3.81784
2.72431 2.72233 2.64650 3.72805 3.01009 3.64583 3.17065 2.98945
2.48229 2.71356 2.47785 4.95050 3.54764 213 a - - -
2.68618 4.42225 2.77519 2.73123 3.46354 2.40513 3.72494
3.29354 2.67741 2.69355 4.24690 2.90347 2.73739 3.18146 2.89801
2.37887 2.77519 2.98518 4.58477 3.61503
0.05185 3.43359 4.00292 0.61958 0.77255 0.49051 0.94755
189 2.42007 4.24518 3.18221 2.85619 3.13318 3.16446 3.76871
2.67564 2.67693 2.38301 3.50700 3.17842 3.71224 3.15967 2.93583
2.57496 2.80608 2.47139 4.62693 3.30973 214 l - - -
2.68618 4.42225 2.77519 2.73123 3.46354 2.40513 3.72494
3.29354 2.67741 2.69355 4.24690 2.90347 2.73739 3.18146 2.89801
2.37887 2.77519 2.98518 4.58477 3.61503
0.05284 3.43411 3.95195 0.61958 0.77255 0.49071 0.94725
190 2.38870 4.30909 2.65685 2.54799 3.46224 2.99503 3.69384
3.04633 2.57864 2.81617 3.85497 2.86024 3.64066 2.99452 2.84869
2.51327 2.79084 2.76223 4.93355 3.45740 215 a - - -
2.68619 4.42217 2.77519 2.73123 3.46356 2.40513 3.72492
3.29355 2.67743 2.69353 4.24689 2.90346 2.73739 3.18146 2.89802
2.37885 2.77519 2.98520 4.58479 3.61498
0.08122 2.77529 4.15549 1.12677 0.39168 0.48080 0.96308
191 2.37680 4.30732 2.75305 2.56371 3.64310 2.96045 3.69566
3.10338 2.45748 2.86134 3.78952 2.91731 3.64747 2.92942 2.73302
2.47597 2.73701 2.79786 5.06386 3.68513 240 a - - -
2.68618 4.42225 2.77519 2.73123 3.46354 2.40513 3.72494
3.29354 2.67741 2.69355 4.24690 2.90347 2.73739 3.18146 2.89801
2.37887 2.77519 2.98518 4.58477 3.61503

```
          0.05429  3.44667  3.86376  0.61958  0.77255  0.48576  0.95510
    192   2.28055  3.97861  3.02321  2.76653  3.51083  2.95998  3.69841
2.89394  2.58709  2.71170  3.72615  3.06298  3.54592  3.08886  2.88723
2.43802  2.66381  2.62743  4.97968  3.61397    241 a - - -
          2.68618  4.42225  2.77519  2.73123  3.46354  2.40513  3.72494
3.29354  2.67741  2.69355  4.24690  2.90347  2.73739  3.18146  2.89801
2.37887  2.77519  2.98518  4.58477  3.61503
          0.04875  3.44113  4.16348  0.61958  0.77255  0.48643  0.95404
    193   2.42486  4.25852  2.54351  2.45285  3.55961  3.01931  3.57048
3.16428  2.53616  2.88490  3.85954  2.83820  3.63751  2.92170  2.85851
2.53500  2.78725  2.86009  4.98053  3.57850    242 a - - -
          2.68618  4.42225  2.77519  2.73123  3.46354  2.40513  3.72494
3.29354  2.67741  2.69355  4.24690  2.90347  2.73739  3.18146  2.89801
2.37887  2.77519  2.98518  4.58477  3.61503
          0.04862  3.44385  4.16620  0.61958  0.77255  0.48744  0.95243
    194   2.27992  4.07020  2.95566  2.70614  3.58574  2.81201  3.78336
2.95402  2.60140  2.75811  3.78663  3.03294  3.60991  3.04016  2.88789
2.42568  2.69444  2.62259  5.03845  3.68808    243 a - - -
          2.68618  4.42225  2.77519  2.73123  3.46354  2.40513  3.72494
3.29354  2.67741  2.69355  4.24690  2.90347  2.73739  3.18146  2.89801
2.37887  2.77519  2.98518  4.58477  3.61503
          0.04862  3.44385  4.16620  0.61958  0.77255  0.48744  0.95243
    195   2.27426  4.03822  3.18746  2.89108  3.48144  2.97516  3.88391
2.62273  2.75385  2.55896  3.66366  3.16306  3.67072  3.22002  3.00910
2.42586  2.65740  2.37358  5.03085  3.65875    244 a - - -
          2.68618  4.42225  2.77519  2.73123  3.46354  2.40513  3.72494
3.29354  2.67741  2.69355  4.24690  2.90347  2.73739  3.18146  2.89801
2.37887  2.77519  2.98518  4.58477  3.61503
          0.04862  3.44385  4.16620  0.61958  0.77255  0.48472  0.95678
    196   2.26348  3.91881  3.13799  2.86868  3.43501  3.01674  3.82351
2.75504  2.69777  2.54594  3.66657  3.11105  3.66437  3.09686  2.95970
2.45177  2.69639  2.49834  4.95848  3.57530    245 a - - -
          2.68618  4.42225  2.77519  2.73123  3.46354  2.40513  3.72494
3.29354  2.67741  2.69355  4.24690  2.90347  2.73739  3.18146  2.89801
2.37887  2.77519  2.98518  4.58477  3.61503
          0.04848  3.44667  4.16901  0.61958  0.77255  0.48576  0.95510
    197   2.27046  4.22471  2.74459  2.58284  3.57694  2.82013  3.61585
3.21202  2.58630  2.93867  3.91608  2.89567  3.58884  3.01411  2.87020
2.36933  2.71497  2.86289  5.01376  3.60378    246 a - - -
          2.68618  4.42225  2.77519  2.73123  3.46354  2.40513  3.72494
3.29354  2.67741  2.69355  4.24690  2.90347  2.73739  3.18146  2.89801
2.37887  2.77519  2.98518  4.58477  3.61503
          0.04848  3.44667  4.16901  0.61958  0.77255  0.48576  0.95510
    198   2.50217  4.39777  2.21356  2.37727  3.59161  2.94554  3.58286
3.21464  2.63395  2.90240  3.94230  2.83274  3.58194  2.93906  2.97726
2.59258  2.86854  2.92900  4.95822  3.61611    247 d - - -
          2.68618  4.42225  2.77519  2.73123  3.46354  2.40513  3.72494
3.29354  2.67741  2.69355  4.24690  2.90347  2.73739  3.18146  2.89801
2.37887  2.77519  2.98518  4.58477  3.61503
          0.04848  3.44667  4.16901  0.61958  0.77255  0.48576  0.95510
    199   2.22435  4.13069  2.89235  2.71554  3.64784  2.88323  3.82134
3.00356  2.68669  2.78325  3.83136  3.01103  3.34283  3.13281  2.93046
2.32234  2.62484  2.69168  5.10622  3.76466    248 a - - -
```

2.68618 4.42225 2.77519 2.73123 3.46354 2.40513 3.72494
3.29354 2.67741 2.69355 4.24690 2.90347 2.73739 3.18146 2.89801
2.37887 2.77519 2.98518 4.58477 3.61503
0.04848 3.44667 4.16901 0.61958 0.77255 0.48576 0.95510
200 2.59410 4.34516 2.81696 2.63333 3.07523 3.02498 2.74066
3.19456 2.53094 2.73265 3.83912 3.01548 3.62399 3.03396 2.77714
2.71065 2.94150 2.95450 4.51985 3.07947 249 k - - -
2.68618 4.42225 2.77519 2.73123 3.46354 2.40513 3.72494
3.29354 2.67741 2.69355 4.24690 2.90347 2.73739 3.18146 2.89801
2.37887 2.77519 2.98518 4.58477 3.61503
0.04848 3.44667 4.16901 0.61958 0.77255 0.48576 0.95510
201 2.44096 4.31267 3.02426 2.72046 3.08767 3.19100 3.69047
2.71389 2.65858 2.42119 3.56434 3.11113 3.77286 3.06602 2.94291
2.63371 2.83158 2.54791 4.78676 3.33094 250 l - - -
2.68618 4.42225 2.77519 2.73123 3.46354 2.40513 3.72494
3.29354 2.67741 2.69355 4.24690 2.90347 2.73739 3.18146 2.89801
2.37887 2.77519 2.98518 4.58477 3.61503
0.04848 3.44667 4.16901 0.61958 0.77255 0.48576 0.95510
202 2.66159 4.24297 3.42943 3.17494 2.33086 3.25331 3.82289
2.44309 3.10117 2.02860 3.34838 3.43896 3.82310 3.45826 3.31394
2.91032 3.01981 2.35382 4.40558 2.93698 251 l - - -
2.68618 4.42225 2.77519 2.73123 3.46354 2.40513 3.72494
3.29354 2.67741 2.69355 4.24690 2.90347 2.73739 3.18146 2.89801
2.37887 2.77519 2.98518 4.58477 3.61503
0.04848 3.44667 4.16901 0.61958 0.77255 0.48576 0.95510
203 2.26445 4.14157 2.79348 2.67694 3.54161 2.86287 3.78666
3.05300 2.71559 2.87377 3.90611 2.94621 3.55853 3.13813 2.98712
2.20467 2.70330 2.72743 4.98512 3.60487 252 s - - -
2.68618 4.42225 2.77519 2.73123 3.46354 2.40513 3.72494
3.29354 2.67741 2.69355 4.24690 2.90347 2.73739 3.18146 2.89801
2.37887 2.77519 2.98518 4.58477 3.61503
0.04848 3.44667 4.16901 0.61958 0.77255 0.48576 0.95510
204 2.66133 4.36348 3.34092 3.02010 2.64023 3.41998 3.71684
2.51770 2.87563 2.10540 3.39062 3.32940 3.93834 3.28827 3.13623
2.84541 2.98400 2.40604 4.40087 2.73133 253 l - - -
2.68618 4.42225 2.77519 2.73123 3.46354 2.40513 3.72494
3.29354 2.67741 2.69355 4.24690 2.90347 2.73739 3.18146 2.89801
2.37887 2.77519 2.98518 4.58477 3.61503
0.04848 3.44667 4.16901 0.61958 0.77255 0.48576 0.95510
205 2.41037 4.33774 2.70401 2.53876 3.40300 3.02486 3.48556
3.18127 2.52929 2.86843 3.86027 2.90392 3.61106 2.98766 2.82413
2.44251 2.76930 2.87391 4.87517 3.37197 254 a - - -
2.68618 4.42225 2.77519 2.73123 3.46354 2.40513 3.72494
3.29354 2.67741 2.69355 4.24690 2.90347 2.73739 3.18146 2.89801
2.37887 2.77519 2.98518 4.58477 3.61503
0.04848 3.44667 4.16901 0.61958 0.77255 0.48576 0.95510
206 2.58500 4.23643 3.42849 3.15230 3.12939 3.27801 3.98747
2.05607 2.96980 2.04211 3.35119 3.42981 3.86215 3.42376 3.19374
2.85382 2.95445 2.17250 4.80271 3.44079 255 l - - -
2.68618 4.42225 2.77519 2.73123 3.46354 2.40513 3.72494
3.29354 2.67741 2.69355 4.24690 2.90347 2.73739 3.18146 2.89801
2.37887 2.77519 2.98518 4.58477 3.61503
0.04848 3.44667 4.16901 0.61958 0.77255 0.48576 0.95510

```
    207   2.48168  4.30337  2.16603  2.44260  3.62630  2.89064  3.71393
3.08584  2.71763  2.85453  3.93146  2.88179  3.53943  3.04955  3.04516
2.59753  2.82412  2.81380  4.94617  3.69234    256 d - - -
          2.68618  4.42225  2.77519  2.73123  3.46354  2.40513  3.72494
3.29354  2.67741  2.69355  4.24690  2.90347  2.73739  3.18146  2.89801
2.37887  2.77519  2.98518  4.58477  3.61503
          0.04848  3.44667  4.16901  0.61958  0.77255  0.48576  0.95510
    208   2.37272  4.16175  3.28484  3.03044  3.36870  3.11384  3.98256
2.41182  2.87871  2.26795  3.50248  3.27769  3.76493  3.33526  3.12406
2.60271  2.64962  2.19888  5.02559  3.66005    257 v - - -
          2.68618  4.42225  2.77519  2.73123  3.46354  2.40513  3.72494
3.29354  2.67741  2.69355  4.24690  2.90347  2.73739  3.18146  2.89801
2.37887  2.77519  2.98518  4.58477  3.61503
          0.04848  3.44667  4.16901  0.61958  0.77255  0.48576  0.95510
    209   2.33836  3.98629  2.87374  2.76616  3.60078  2.00361  3.79029
3.15638  2.82691  2.87178  3.96579  3.06707  3.35543  3.23202  3.02483
2.52219  2.78238  2.83289  4.74403  3.71835    258 g - - -
          2.68618  4.42225  2.77519  2.73123  3.46354  2.40513  3.72494
3.29354  2.67741  2.69355  4.24690  2.90347  2.73739  3.18146  2.89801
2.37887  2.77519  2.98518  4.58477  3.61503
          0.04848  3.44667  4.16901  0.61958  0.77255  0.48576  0.95510
    210   2.27051  4.12079  2.81432  2.60073  3.67242  2.97544  3.79601
2.87746  2.64739  2.76757  3.79417  3.00093  3.63319  3.08497  2.94026
2.42613  2.68036  2.59340  5.12881  3.78749    259 a - - -
          2.68618  4.42225  2.77519  2.73123  3.46354  2.40513  3.72494
3.29354  2.67741  2.69355  4.24690  2.90347  2.73739  3.18146  2.89801
2.37887  2.77519  2.98518  4.58477  3.61503
          0.04848  3.44667  4.16901  0.61958  0.77255  0.48576  0.95510
    211   2.53250  4.26769  3.40930  3.10407  3.15407  3.31099  3.96170
2.32682  2.87028  1.99488  3.32743  3.37743  3.89828  3.35453  3.10913
2.77919  2.86517  2.19196  4.86894  3.44880    260 l - - -
          2.68618  4.42225  2.77519  2.73123  3.46354  2.40513  3.72494
3.29354  2.67741  2.69355  4.24690  2.90347  2.73739  3.18146  2.89801
2.37887  2.77519  2.98518  4.58477  3.61503
          0.04848  3.44667  4.16901  0.61958  0.77255  0.48576  0.95510
    212   2.48935  4.46548  2.30713  2.31908  3.65635  2.99392  3.57580
3.27501  2.55944  2.98120  3.96280  2.75021  3.62432  2.89755  2.91209
2.56658  2.84743  2.96598  5.05132  3.64943    261 d - - -
          2.68618  4.42225  2.77519  2.73123  3.46354  2.40513  3.72494
3.29354  2.67741  2.69355  4.24690  2.90347  2.73739  3.18146  2.89801
2.37887  2.77519  2.98518  4.58477  3.61503
          0.04848  3.44667  4.16901  0.61958  0.77255  0.48576  0.95510
    213   2.43899  4.27134  3.10563  2.81169  3.14663  3.16645  3.69317
2.72805  2.64534  2.44240  3.56112  3.11229  3.70395  3.12185  2.91493
2.56740  2.78874  2.53374  4.75649  3.25553    262 a - - -
          2.68618  4.42225  2.77519  2.73123  3.46354  2.40513  3.72494
3.29354  2.67741  2.69355  4.24690  2.90347  2.73739  3.18146  2.89801
2.37887  2.77519  2.98518  4.58477  3.61503
          0.04848  3.44667  4.16901  0.61958  0.77255  0.48576  0.95510
    214   2.40361  4.35323  2.58445  2.42960  3.66594  2.97723  3.68853
3.17157  2.53298  2.94940  3.93617  2.70126  3.62711  2.94920  2.83939
2.49825  2.76859  2.85568  5.08032  3.68141    263 a - - -
          2.68618  4.42225  2.77519  2.73123  3.46354  2.40513  3.72494
3.29354  2.67741  2.69355  4.24690  2.90347  2.73739  3.18146  2.89801
2.37887  2.77519  2.98518  4.58477  3.61503
```

```
         0.04848  3.44667  4.16901  0.61958  0.77255  0.48576  0.95510
    215  2.39928  4.31652  2.81909  2.52703  3.58887  3.06965  3.75386
2.84592  2.55447  2.65172  3.75555  2.96643  3.69586  3.04326  2.81758
2.57803  2.78621  2.56660  5.06995  3.70137    264 a - - -
         2.68618  4.42225  2.77519  2.73123  3.46354  2.40513  3.72494
3.29354  2.67741  2.69355  4.24690  2.90347  2.73739  3.18146  2.89801
2.37887  2.77519  2.98518  4.58477  3.61503
         0.04848  3.44667  4.16901  0.61958  0.77255  0.48576  0.95510
    216  2.43026  4.32667  2.61230  2.51455  3.45408  2.97912  3.67563
3.12161  2.59124  2.88711  3.90711  2.62402  3.62189  3.01749  2.89225
2.54965  2.81389  2.82506  4.89360  3.39993    265 a - - -
         2.68618  4.42225  2.77519  2.73123  3.46354  2.40513  3.72494
3.29354  2.67741  2.69355  4.24690  2.90347  2.73739  3.18146  2.89801
2.37887  2.77519  2.98518  4.58477  3.61503
         0.04848  3.44667  4.16901  0.61958  0.77255  0.48576  0.95510
    217  2.41206  4.23720  3.08879  2.84734  3.32001  3.10029  3.82800
2.66216  2.70312  2.26587  3.56644  3.15980  3.47458  3.14769  2.95718
2.61657  2.81734  2.49809  4.89776  3.51368    266 l - - -
         2.68618  4.42225  2.77519  2.73123  3.46354  2.40513  3.72494
3.29354  2.67741  2.69355  4.24690  2.90347  2.73739  3.18146  2.89801
2.37887  2.77519  2.98518  4.58477  3.61503
         0.04848  3.44667  4.16901  0.61958  0.77255  0.48576  0.95510
    218  2.33836  3.98629  2.87374  2.76616  3.60078  2.00361  3.79029
3.15638  2.82691  2.87178  3.96579  3.06707  3.35543  3.23202  3.02483
2.52219  2.78238  2.83289  4.74403  3.71835    267 g - - -
         2.68618  4.42225  2.77519  2.73123  3.46354  2.40513  3.72494
3.29354  2.67741  2.69355  4.24690  2.90347  2.73739  3.18146  2.89801
2.37887  2.77519  2.98518  4.58477  3.61503
         0.04848  3.44667  4.16901  0.61958  0.77255  0.48576  0.95510
    219  2.56291  4.24877  3.46423  3.16750  3.13444  3.32407  4.00789
2.10049  2.97156  2.02676  3.33326  3.43632  3.90666  3.42561  3.20543
2.83209  2.93458  2.12286  4.85026  3.45605    268 l - - -
         2.68618  4.42225  2.77519  2.73123  3.46354  2.40513  3.72494
3.29354  2.67741  2.69355  4.24690  2.90347  2.73739  3.18146  2.89801
2.37887  2.77519  2.98518  4.58477  3.61503
         0.04848  3.44667  4.16901  0.61958  0.77255  0.48576  0.95510
    220  2.61841  4.35407  3.14828  2.64531  3.01535  3.26726  3.61388
2.85812  2.52401  2.41929  3.53544  3.15397  3.77392  3.01076  2.77760
2.77787  2.90889  2.68554  3.76611  3.09076    269 l - - -
         2.68618  4.42225  2.77519  2.73123  3.46354  2.40513  3.72494
3.29354  2.67741  2.69355  4.24690  2.90347  2.73739  3.18146  2.89801
2.37887  2.77519  2.98518  4.58477  3.61503
         0.04848  3.44667  4.16901  0.61958  0.77255  0.48576  0.95510
    221  2.51905  4.24418  3.50511  3.17982  2.99053  3.34446  3.99406
2.23303  3.01635  2.01860  3.31566  3.43732  3.92378  3.43077  3.26569
2.79980  2.90778  2.08978  4.80744  3.36885    270 l - - -
         2.68618  4.42225  2.77519  2.73123  3.46354  2.40513  3.72494
3.29354  2.67741  2.69355  4.24690  2.90347  2.73739  3.18146  2.89801
2.37887  2.77519  2.98518  4.58477  3.61503
         0.04848  3.44667  4.16901  0.61958  0.77255  0.48576  0.95510
    222  2.37136  4.19165  3.18234  2.91043  3.33555  2.95523  3.85715
2.52192  2.74414  2.37741  3.54626  3.19443  3.73524  3.21896  2.99882
2.58996  2.78117  2.38714  4.91751  3.51779    271 a - - -
```

2.68618 4.42225 2.77519 2.73123 3.46354 2.40513 3.72494
3.29354 2.67741 2.69355 4.24690 2.90347 2.73739 3.18146 2.89801
2.37887 2.77519 2.98518 4.58477 3.61503
0.04848 3.44667 4.16901 0.61958 0.77255 0.48576 0.95510
223 2.66481 4.29422 3.48015 3.18558 2.54644 3.37144 3.85936
2.36744 3.06116 1.92180 3.29908 3.45628 3.92021 3.44384 3.29312
2.91837 3.01354 2.28398 4.48603 2.99090 272 l - - -
2.68618 4.42225 2.77519 2.73123 3.46354 2.40513 3.72494
3.29354 2.67741 2.69355 4.24690 2.90347 2.73739 3.18146 2.89801
2.37887 2.77519 2.98518 4.58477 3.61503
0.04848 3.44667 4.16901 0.61958 0.77255 0.48576 0.95510
224 2.52936 4.35426 2.81020 2.59804 3.60668 3.03420 3.66660
3.06831 2.16609 2.75156 3.82868 2.98923 3.63659 2.96476 2.54385
2.65643 2.87921 2.82071 4.94236 3.67172 273 k - - -
2.68618 4.42225 2.77519 2.73123 3.46354 2.40513 3.72494
3.29354 2.67741 2.69355 4.24690 2.90347 2.73739 3.18146 2.89801
2.37887 2.77519 2.98518 4.58477 3.61503
0.04848 3.44667 4.16901 0.61958 0.77255 0.48576 0.95510
225 2.57977 4.26303 3.47006 3.16761 3.11479 3.34690 3.99979
2.10787 2.96106 2.00060 3.32410 3.44100 3.92153 3.42044 3.19518
2.84913 2.94620 2.13474 4.83505 3.43165 274 l - - -
2.68618 4.42225 2.77519 2.73123 3.46354 2.40513 3.72494
3.29354 2.67741 2.69355 4.24690 2.90347 2.73739 3.18146 2.89801
2.37887 2.77519 2.98518 4.58477 3.61503
0.04848 3.44667 4.16901 0.61958 0.77255 0.48576 0.95510
226 2.25721 4.15646 2.98892 2.78469 3.53426 2.96261 3.83633
2.82631 2.67159 2.65687 3.75010 3.07224 3.63982 3.16102 2.92626
2.37268 2.69105 2.50456 5.05388 3.67220 275 a - - -
2.68618 4.42225 2.77519 2.73123 3.46354 2.40513 3.72494
3.29354 2.67741 2.69355 4.24690 2.90347 2.73739 3.18146 2.89801
2.37887 2.77519 2.98518 4.58477 3.61503
0.04848 3.44667 4.16901 0.61958 0.77255 0.48576 0.95510
227 2.37203 4.30589 2.68675 2.52082 3.69981 2.98785 3.72187
3.06705 2.49683 2.87950 3.87950 2.86587 3.63782 2.97074 2.82308
2.47601 2.67092 2.76426 5.12304 3.75050 276 a - - -
2.68618 4.42225 2.77519 2.73123 3.46354 2.40513 3.72494
3.29354 2.67741 2.69355 4.24690 2.90347 2.73739 3.18146 2.89801
2.37887 2.77519 2.98518 4.58477 3.61503
0.04848 3.44667 4.16901 0.61958 0.77255 0.48576 0.95510
228 2.30657 4.17915 2.91997 2.72652 3.54623 2.95009 3.80901
2.91759 2.67232 2.60515 3.74629 3.04009 3.25408 3.09435 2.94055
2.45248 2.67228 2.65329 5.03697 3.68734 277 a - - -
2.68618 4.42225 2.77519 2.73123 3.46354 2.40513 3.72494
3.29354 2.67741 2.69355 4.24690 2.90347 2.73739 3.18146 2.89801
2.37887 2.77519 2.98518 4.58477 3.61503
0.04848 3.44667 4.16901 0.61958 0.77255 0.48576 0.95510
229 2.42812 4.38284 2.40217 2.36719 3.73079 2.96897 3.69362
3.14710 2.59649 2.95874 3.94623 2.74578 3.61586 2.97357 2.93807
2.53033 2.75715 2.83983 5.12415 3.75351 278 e - - -
2.68618 4.42225 2.77519 2.73123 3.46354 2.40513 3.72494
3.29354 2.67741 2.69355 4.24690 2.90347 2.73739 3.18146 2.89801
2.37887 2.77519 2.98518 4.58477 3.61503
0.04848 3.44667 4.16901 0.61958 0.77255 0.48576 0.95510

```
    230   2.33836  3.98629  2.87374  2.76616  3.60078  2.00361  3.79029
3.15638  2.82691  2.87178  3.96579  3.06707  3.35543  3.23202  3.02483
2.52219  2.78238  2.83289  4.74403  3.71835    279 g - - -
          2.68618  4.42225  2.77519  2.73123  3.46354  2.40513  3.72494
3.29354  2.67741  2.69355  4.24690  2.90347  2.73739  3.18146  2.89801
2.37887  2.77519  2.98518  4.58477  3.61503
          0.04848  3.44667  4.16901  0.61958  0.77255  0.48576  0.95510
    231   2.62785  4.38671  3.21436  2.88713  2.76245  3.36691  3.59525
2.70593  2.70099  2.29302  3.40869  3.22050  3.88470  3.15957  2.96600
2.78068  2.94210  2.56320  4.41332  2.68881    280 l - - -
          2.68618  4.42225  2.77519  2.73123  3.46354  2.40513  3.72494
3.29354  2.67741  2.69355  4.24690  2.90347  2.73739  3.18146  2.89801
2.37887  2.77519  2.98518  4.58477  3.61503
          0.04848  3.44667  4.16901  0.61958  0.77255  0.48576  0.95510
    232   2.10323  4.12042  3.09729  2.91642  3.45528  2.96749  3.93410
2.58864  2.86023  2.43745  3.63981  3.17887  3.64772  3.29733  3.10233
2.53359  2.76021  2.40857  5.00842  3.69195    281 a - - -
          2.68618  4.42225  2.77519  2.73123  3.46354  2.40513  3.72494
3.29354  2.67741  2.69355  4.24690  2.90347  2.73739  3.18146  2.89801
2.37887  2.77519  2.98518  4.58477  3.61503
          0.04848  3.44667  4.16901  0.61958  0.77255  0.48576  0.95510
    233   2.35722  4.23773  2.94162  2.71501  3.57831  3.02292  3.77674
2.85667  2.52259  2.66568  3.74672  3.04332  3.66781  3.07380  2.75180
2.48597  2.64051  2.62164  5.06021  3.70135    282 a - - -
          2.68618  4.42225  2.77519  2.73123  3.46354  2.40513  3.72494
3.29354  2.67741  2.69355  4.24690  2.90347  2.73739  3.18146  2.89801
2.37887  2.77519  2.98518  4.58477  3.61503
          0.04848  3.44667  4.16901  0.61958  0.77255  0.48576  0.95510
    234   2.61996  4.30140  3.54029  3.19942  2.91334  3.43831  3.96410
2.21586  2.99597  1.90688  3.27244  3.47275  3.98272  3.42919  3.23983
2.88926  2.97115  2.14190  4.72164  3.25694    283 l - - -
          2.68618  4.42225  2.77519  2.73123  3.46354  2.40513  3.72494
3.29354  2.67741  2.69355  4.24690  2.90347  2.73739  3.18146  2.89801
2.37887  2.77519  2.98518  4.58477  3.61503
          0.04848  3.44667  4.16901  0.61958  0.77255  0.48576  0.95510
    235   2.23267  4.12042  2.75113  2.63940  3.66506  2.62972  3.78740
3.15458  2.70993  2.96823  3.95308  2.92400  3.52262  3.11858  2.98802
2.27700  2.67610  2.79237  5.05348  3.72207    284 a - - -
          2.68618  4.42225  2.77519  2.73123  3.46354  2.40513  3.72494
3.29354  2.67741  2.69355  4.24690  2.90347  2.73739  3.18146  2.89801
2.37887  2.77519  2.98518  4.58477  3.61503
          0.04848  3.44667  4.16901  0.61958  0.77255  0.48576  0.95510
    236   2.38952  4.20316  2.74807  2.61836  3.46324  2.87214  3.71856
3.12161  2.65048  2.87046  3.93540  2.44245  3.53997  3.09647  2.91039
2.53297  2.80322  2.81914  4.83809  3.50411    285 a - - -
          2.68618  4.42225  2.77519  2.73123  3.46354  2.40513  3.72494
3.29354  2.67741  2.69355  4.24690  2.90347  2.73739  3.18146  2.89801
2.37887  2.77519  2.98518  4.58477  3.61503
          0.04848  3.44667  4.16901  0.61958  0.77255  0.48576  0.95510
    237   2.59839  4.26445  3.34882  3.09329  3.06133  3.24980  3.91197
2.37277  2.88958  1.88775  3.38464  3.37829  3.83804  3.36974  3.10836
2.84209  2.96860  2.28598  4.71617  3.31542    286 l - - -
          2.68618  4.42225  2.77519  2.73123  3.46354  2.40513  3.72494
3.29354  2.67741  2.69355  4.24690  2.90347  2.73739  3.18146  2.89801
2.37887  2.77519  2.98518  4.58477  3.61503
```

```
          0.04848  3.44667  4.16901  0.61958  0.77255  0.48576  0.95510
    238   2.57503  4.32219  2.63991  2.09280  3.63257  2.93857  3.70027
3.01680  2.57038  2.74746  3.86829  2.94752  3.56048  3.03028  2.83956
2.69983  2.93190  2.78454  4.90613  3.72994    287 e - - -
          2.68618  4.42225  2.77519  2.73123  3.46354  2.40513  3.72494
3.29354  2.67741  2.69355  4.24690  2.90347  2.73739  3.18146  2.89801
2.37887  2.77519  2.98518  4.58477  3.61503
          0.04848  3.44667  4.16901  0.61958  0.77255  0.48576  0.95510
    239   2.53373  4.15390  3.59768  3.22848  3.07493  3.36343  4.01180
2.19343  2.99012  1.98100  3.30778  3.47038  3.93535  3.43400  3.23065
2.80808  2.90352  2.08510  4.83705  3.41526    288 l - - -
          2.68618  4.42225  2.77519  2.73123  3.46354  2.40513  3.72494
3.29354  2.67741  2.69355  4.24690  2.90347  2.73739  3.18146  2.89801
2.37887  2.77519  2.98518  4.58477  3.61503
          0.04848  3.44667  4.16901  0.61958  0.77255  0.48576  0.95510
    240   2.50466  4.27033  3.29589  2.97933  3.23622  3.26044  3.87737
2.36350  2.71294  2.20093  3.44465  3.28092  3.84204  3.23993  2.93298
2.72561  2.86819  2.27468  4.88090  3.47880    289 l - - -
          2.68618  4.42225  2.77519  2.73123  3.46354  2.40513  3.72494
3.29354  2.67741  2.69355  4.24690  2.90347  2.73739  3.18146  2.89801
2.37887  2.77519  2.98518  4.58477  3.61503
          0.04848  3.44667  4.16901  0.61958  0.77255  0.48576  0.95510
    241   2.57503  4.32219  2.63991  2.09280  3.63257  2.93857  3.70027
3.01680  2.57038  2.74746  3.86829  2.94752  3.56048  3.03028  2.83956
2.69983  2.93190  2.78454  4.90613  3.72994    290 e - - -
          2.68618  4.42225  2.77519  2.73123  3.46354  2.40513  3.72494
3.29354  2.67741  2.69355  4.24690  2.90347  2.73739  3.18146  2.89801
2.37887  2.77519  2.98518  4.58477  3.61503
          0.04848  3.44667  4.16901  0.61958  0.77255  0.48576  0.95510
    242   2.49544  4.40193  2.52880  2.33876  3.65383  3.04799  3.70393
2.96216  2.56910  2.76075  3.83299  2.89912  3.66448  2.98704  2.89088
2.61040  2.85041  2.66540  5.07212  3.72598    291 e - - -
          2.68618  4.42225  2.77519  2.73123  3.46354  2.40513  3.72494
3.29354  2.67741  2.69355  4.24690  2.90347  2.73739  3.18146  2.89801
2.37887  2.77519  2.98518  4.58477  3.61503
          0.04848  3.44667  4.16901  0.61958  0.77255  0.48576  0.95510
    243   2.37431  4.17869  2.83534  2.65185  3.63134  2.58348  3.72080
3.15271  2.51850  2.85673  3.89049  2.99647  3.52664  3.05388  2.57985
2.52448  2.77651  2.84214  4.92164  3.70105    292 a - - -
          2.68618  4.42225  2.77519  2.73123  3.46354  2.40513  3.72494
3.29354  2.67741  2.69355  4.24690  2.90347  2.73739  3.18146  2.89801
2.37887  2.77519  2.98518  4.58477  3.61503
          0.04848  3.44667  4.16901  0.61958  0.77255  0.48576  0.95510
    244   2.35861  4.09453  2.94236  2.77901  3.50071  2.83446  3.80548
2.95698  2.75771  2.57900  3.77877  3.08755  2.65780  3.19435  2.98946
2.53906  2.78204  2.70197  4.84345  3.65632    293 a - - -
          2.68618  4.42225  2.77519  2.73123  3.46354  2.40513  3.72494
3.29354  2.67741  2.69355  4.24690  2.90347  2.73739  3.18146  2.89801
2.37887  2.77519  2.98518  4.58477  3.61503
          0.04848  3.44667  4.16901  0.61958  0.77255  0.48576  0.95510
    245   2.58257  4.27266  3.36453  3.09969  3.06633  3.27688  3.92452
2.36135  2.89096  1.91107  3.37430  3.37993  3.86530  3.37066  3.11591
2.82643  2.95482  2.25267  4.74633  3.32592    294 l - - -
```

```
         2.68618  4.42225  2.77519  2.73123  3.46354  2.40513  3.72494
3.29354  2.67741  2.69355  4.24690  2.90347  2.73739  3.18146  2.89801
2.37887  2.77519  2.98518  4.58477  3.61503
         0.04848  3.44667  4.16901  0.61958  0.77255  0.48576  0.95510
    246  2.25721  4.13720  2.98877  2.81009  3.54376  2.96307  3.87241
2.72732  2.75750  2.63283  3.73389  3.09075  3.64528  3.19859  3.02577
2.38828  2.61282  2.45385  5.08224  3.70315    295 a - - -
         2.68618  4.42225  2.77519  2.73123  3.46354  2.40513  3.72494
3.29354  2.67741  2.69355  4.24690  2.90347  2.73739  3.18146  2.89801
2.37887  2.77519  2.98518  4.58477  3.61503
         0.04848  3.44667  4.16901  0.61958  0.77255  0.48576  0.95510
    247  2.29868  4.18108  2.73290  2.48610  3.71051  2.56074  3.76403
3.07288  2.65351  2.86917  3.90783  2.95900  3.53084  3.08568  2.92566
2.49849  2.76321  2.76573  5.02410  3.80081    296 a - - -
         2.68618  4.42225  2.77519  2.73123  3.46354  2.40513  3.72494
3.29354  2.67741  2.69355  4.24690  2.90347  2.73739  3.18146  2.89801
2.37887  2.77519  2.98518  4.58477  3.61503
         0.04848  3.44667  4.16901  0.61958  0.77255  0.48576  0.95510
    248  2.49529  4.42527  2.63018  2.35199  3.70583  3.04585  3.67209
3.03879  2.42063  2.84624  3.78218  2.87312  3.67892  2.93739  2.75382
2.60443  2.84104  2.76951  5.09660  3.74746    297 e - - -
         2.68618  4.42225  2.77519  2.73123  3.46354  2.40513  3.72494
3.29354  2.67741  2.69355  4.24690  2.90347  2.73739  3.18146  2.89801
2.37887  2.77519  2.98518  4.58477  3.61503
         0.04848  3.44667  4.16901  0.61958  0.77255  0.48576  0.95510
    249  2.28668  4.27214  2.75480  2.48714  3.65988  2.98849  3.78158
2.88331  2.62120  2.72305  3.80930  2.97658  3.64322  3.07545  2.90636
2.50465  2.78982  2.62639  5.10969  3.78119    298 a - - -
         2.68618  4.42225  2.77519  2.73123  3.46354  2.40513  3.72494
3.29354  2.67741  2.69355  4.24690  2.90347  2.73739  3.18146  2.89801
2.37887  2.77519  2.98518  4.58477  3.61503
         0.04848  3.44667  4.16901  0.61958  0.77255  0.48576  0.95510
    250  2.58022  4.29563  3.34556  3.04653  3.12648  3.31771  3.90215
2.31145  2.77821  2.01866  3.39197  3.34770  3.88698  3.30756  3.04660
2.81782  2.93677  2.25428  4.80048  3.37976    299 l - - -
         2.68618  4.42225  2.77519  2.73123  3.46354  2.40513  3.72494
3.29354  2.67741  2.69355  4.24690  2.90347  2.73739  3.18146  2.89801
2.37887  2.77519  2.98518  4.58477  3.61503
         0.04848  3.44667  4.16901  0.61958  0.77255  0.48576  0.95510
    251  2.36368  4.34356  2.76298  2.52032  3.66423  3.04428  3.71229
3.01509  2.45185  2.75130  3.80516  2.95591  3.66982  2.95525  2.67739
2.54985  2.80498  2.75180  5.09146  3.74806    300 a - - -
         2.68618  4.42225  2.77519  2.73123  3.46354  2.40513  3.72494
3.29354  2.67741  2.69355  4.24690  2.90347  2.73739  3.18146  2.89801
2.37887  2.77519  2.98518  4.58477  3.61503
         0.04848  3.44667  4.16901  0.61958  0.77255  0.48576  0.95510
    252  2.47933  4.39275  2.80403  2.58386  3.55046  3.08307  3.66437
3.09388  2.37079  2.69671  3.77992  2.97382  3.68415  2.78983  2.53665
2.62136  2.85275  2.85155  4.97411  3.61921    301 k - - -
         2.68618  4.42225  2.77519  2.73123  3.46354  2.40513  3.72494
3.29354  2.67741  2.69355  4.24690  2.90347  2.73739  3.18146  2.89801
2.37887  2.77519  2.98518  4.58477  3.61503
         0.04848  3.44667  4.16901  0.61958  0.77255  0.48576  0.95510
```

```
     253   2.47491  4.22187  3.33899  3.03093  3.24328  3.22702  3.96681
 2.32962  2.88360  2.15615  3.42961  3.33271  3.83847  3.34696  3.12833
 2.71657  2.86263  2.10114  4.92250  3.52974    302 v - - -
           2.68618  4.42225  2.77519  2.73123  3.46354  2.40513  3.72494
 3.29354  2.67741  2.69355  4.24690  2.90347  2.73739  3.18146  2.89801
 2.37887  2.77519  2.98518  4.58477  3.61503
           0.04848  3.44667  4.16901  0.61958  0.77255  0.48576  0.95510
     254   2.50772  4.41868  2.76486  2.55438  3.53568  3.10727  3.66059
 3.01904  2.34143  2.72763  3.79260  2.92681  3.70494  2.78934  2.67640
 2.62442  2.85491  2.80892  4.98100  3.58916    303 k - - -
           2.68618  4.42225  2.77519  2.73123  3.46354  2.40513  3.72494
 3.29354  2.67741  2.69355  4.24690  2.90347  2.73739  3.18146  2.89801
 2.37887  2.77519  2.98518  4.58477  3.61503
           0.04848  3.44667  4.16901  0.61958  0.77255  0.48576  0.95510
     255   2.52303  4.39617  2.84797  2.60183  3.47191  3.09081  3.52491
 3.16723  2.32454  2.74854  3.80487  2.98523  3.67852  2.89191  2.39268
 2.63941  2.86428  2.91171  4.87559  3.50435    304 k - - -
           2.68618  4.42225  2.77519  2.73123  3.46354  2.40513  3.72494
 3.29354  2.67741  2.69355  4.24690  2.90347  2.73739  3.18146  2.89801
 2.37887  2.77519  2.98518  4.58477  3.61503
           0.04848  3.44667  4.16901  0.61958  0.77255  0.48576  0.95510
     256   2.43708  4.35532  2.85247  2.61212  3.56886  3.13171  3.71870
 2.83726  2.40852  2.64409  3.67565  3.01144  3.72563  2.97712  2.73770
 2.60795  2.81555  2.61286  5.04300  3.67622    305 k - - -
           2.68618  4.42225  2.77519  2.73123  3.46354  2.40513  3.72494
 3.29354  2.67741  2.69355  4.24690  2.90347  2.73739  3.18146  2.89801
 2.37887  2.77519  2.98518  4.58477  3.61503
           0.04848  3.44667  4.16901  0.61958  0.77255  0.48576  0.95510
     257   2.44818  4.39234  2.55265  2.26644  3.71698  3.00419  3.69701
 3.04506  2.53813  2.85511  3.89557  2.85044  3.63410  2.97897  2.84523
 2.60065  2.85466  2.77637  5.08759  3.77147    306 e - - -
           2.68618  4.42225  2.77519  2.73123  3.46354  2.40513  3.72494
 3.29354  2.67741  2.69355  4.24690  2.90347  2.73739  3.18146  2.89801
 2.37887  2.77519  2.98518  4.58477  3.61503
           0.04848  3.44667  4.16901  0.61958  0.77255  0.48576  0.95510
     258   2.48333  4.42013  2.70976  2.49618  3.65905  3.07598  3.65641
 3.13807  2.34699  2.81849  3.84407  2.88864  3.68298  2.81076  2.62195
 2.59265  2.79487  2.86483  5.06298  3.69634    307 k - - -
           2.68618  4.42225  2.77519  2.73123  3.46354  2.40513  3.72494
 3.29354  2.67741  2.69355  4.24690  2.90347  2.73739  3.18146  2.89801
 2.37887  2.77519  2.98518  4.58477  3.61503
           0.04848  3.44667  4.16901  0.61958  0.77255  0.48576  0.95510
     259   2.50168  4.40844  2.67169  2.50586  3.65744  3.05036  3.65218
 3.13532  2.23898  2.85743  3.87851  2.88852  3.65857  2.90441  2.63850
 2.61048  2.85248  2.85888  5.02400  3.68398    308 k - - -
           2.68618  4.42225  2.77519  2.73123  3.46354  2.40513  3.72494
 3.29354  2.67741  2.69355  4.24690  2.90347  2.73739  3.18146  2.89801
 2.37887  2.77519  2.98518  4.58477  3.61503
           0.04848  3.44667  4.16901  0.61958  0.77255  0.48576  0.95510
     260   2.72925  4.29213  3.47937  3.11500  2.51544  3.31047  3.58946
 2.73944  2.85093  2.18933  3.42500  3.37721  3.81990  3.29036  3.05701
 2.93110  3.03303  2.63504  3.06274  2.60845    309 l - - -
           2.68618  4.42225  2.77519  2.73123  3.46354  2.40513  3.72494
 3.29354  2.67741  2.69355  4.24690  2.90347  2.73739  3.18146  2.89801
 2.37887  2.77519  2.98518  4.58477  3.61503
```

```
          0.04848  3.44667  4.16901  0.61958  0.77255  0.48576  0.95510
    261   2.46160  4.35846  2.76699  2.56073  3.65432  3.00649  3.66681
3.12140  2.25799  2.85449  3.87151  2.92398  3.65056  2.94888  2.59017
2.56218  2.82470  2.83642  5.02218  3.68715    310 k - - -
          2.68618  4.42225  2.77519  2.73123  3.46354  2.40513  3.72494
3.29354  2.67741  2.69355  4.24690  2.90347  2.73739  3.18146  2.89801
2.37887  2.77519  2.98518  4.58477  3.61503
          0.04848  3.44667  4.16901  0.61958  0.77255  0.48576  0.95510
    262   2.41049  4.38010  2.66679  2.49370  3.66667  3.05933  3.68839
3.03120  2.44627  2.81014  3.82604  2.89803  3.67618  2.89538  2.74815
2.56116  2.78335  2.77850  5.09416  3.72215    311 a - - -
          2.68618  4.42225  2.77519  2.73123  3.46354  2.40513  3.72494
3.29354  2.67741  2.69355  4.24690  2.90347  2.73739  3.18146  2.89801
2.37887  2.77519  2.98518  4.58477  3.61503
          0.04848  3.44667  4.16901  0.61958  0.77255  0.48576  0.95510
    263   2.45993  4.41958  2.66123  2.43440  3.69694  3.06436  3.66808
3.09967  2.36468  2.85849  3.86888  2.83942  3.67843  2.89349  2.70992
2.58572  2.83214  2.80112  5.09839  3.72994    312 k - - -
          2.68618  4.42225  2.77519  2.73123  3.46354  2.40513  3.72494
3.29354  2.67741  2.69355  4.24690  2.90347  2.73739  3.18146  2.89801
2.37887  2.77519  2.98518  4.58477  3.61503
          0.04848  3.44667  4.16901  0.61958  0.77255  0.48576  0.95510
    264   2.58105  4.35329  3.25893  2.89974  3.10682  3.35860  3.77740
2.51897  2.60398  2.19530  3.34649  3.24401  3.88259  3.15052  2.88262
2.78090  2.88537  2.39272  4.76355  3.26084    313 l - - -
          2.68618  4.42225  2.77519  2.73123  3.46354  2.40513  3.72494
3.29354  2.67741  2.69355  4.24690  2.90347  2.73739  3.18146  2.89801
2.37887  2.77519  2.98518  4.58477  3.61503
          0.04848  3.44667  4.16901  0.61958  0.77255  0.48576  0.95510
    265   2.39572  4.31604  2.78439  2.49529  3.62713  3.02576  3.75099
2.84268  2.55764  2.69644  3.78392  2.95195  3.68416  3.03639  2.84443
2.56834  2.76250  2.61357  5.09105  3.72945    314 a - - -
          2.68618  4.42225  2.77519  2.73123  3.46354  2.40513  3.72494
3.29354  2.67741  2.69355  4.24690  2.90347  2.73739  3.18146  2.89801
2.37887  2.77519  2.98518  4.58477  3.61503
          0.04848  3.44667  4.16901  0.61958  0.77255  0.48576  0.95510
    266   2.45059  4.39466  2.76541  2.54017  3.60660  3.08911  3.67819
3.05053  2.37381  2.71597  3.79348  2.95291  3.69564  2.86514  2.65156
2.57545  2.83106  2.79595  5.04327  3.67205    315 k - - -
          2.68618  4.42225  2.77519  2.73123  3.46354  2.40513  3.72494
3.29354  2.67741  2.69355  4.24690  2.90347  2.73739  3.18146  2.89801
2.37887  2.77519  2.98518  4.58477  3.61503
          0.04848  3.44667  4.16901  0.61958  0.77255  0.48576  0.95510
    267   2.53814  4.44075  2.77334  2.48331  3.59835  3.14982  3.67293
2.93269  2.33803  2.65202  3.75589  2.96583  3.72961  2.91280  2.65639
2.65843  2.86965  2.72750  5.03608  3.67745    316 k - - -
          2.68618  4.42225  2.77519  2.73123  3.46354  2.40513  3.72494
3.29354  2.67741  2.69355  4.24690  2.90347  2.73739  3.18146  2.89801
2.37887  2.77519  2.98518  4.58477  3.61503
          0.04848  3.44667  4.16901  0.61958  0.77255  0.48576  0.95510
    268   2.42386  4.13819  3.03996  2.72031  3.50738  3.06152  3.71821
2.94450  2.45266  2.61961  3.70680  3.07623  3.66819  2.98605  2.48514
2.58252  2.77046  2.70321  4.93992  3.61894    317 a - - -
```

```
          2.68618  4.42225  2.77519  2.73123  3.46354  2.40513  3.72494
3.29354  2.67741  2.69355  4.24690  2.90347  2.73739  3.18146  2.89801
2.37887  2.77519  2.98518  4.58477  3.61503
          0.04848  3.44667  4.16901  0.61958  0.77255  0.48576  0.95510
    269   2.33332  4.26839  2.97219  2.66598  3.46371  3.08986  3.75082
2.82291  2.56528  2.59533  3.68917  3.02688  3.70414  3.06528  2.83570
2.55605  2.74204  2.58424  4.80105  3.57891    318 a - - -
          2.68618  4.42225  2.77519  2.73123  3.46354  2.40513  3.72494
3.29354  2.67741  2.69355  4.24690  2.90347  2.73739  3.18146  2.89801
2.37887  2.77519  2.98518  4.58477  3.61503
          0.04848  3.44667  4.16901  0.61958  0.77255  0.48576  0.95510
    270   2.48441  4.38806  2.61973  2.28491  3.68084  3.01900  3.70143
3.02358  2.52039  2.82309  3.87762  2.87263  3.64950  2.98559  2.81647
2.57378  2.82498  2.76056  5.07832  3.73999    319 e - - -
          2.68618  4.42225  2.77519  2.73123  3.46354  2.40513  3.72494
3.29354  2.67741  2.69355  4.24690  2.90347  2.73739  3.18146  2.89801
2.37887  2.77519  2.98518  4.58477  3.61503
          0.04848  3.44667  4.16901  0.61958  0.77255  0.48576  0.95510
    271   2.31875  4.14294  3.20121  2.97313  3.43880  3.04971  3.96444
2.50511  2.85122  2.35655  3.56712  3.22314  3.71809  3.30560  3.09904
2.54873  2.57919  2.27999  5.06381  3.70944    320 v - - -
          2.68618  4.42225  2.77519  2.73123  3.46354  2.40513  3.72494
3.29354  2.67741  2.69355  4.24690  2.90347  2.73739  3.18146  2.89801
2.37887  2.77519  2.98518  4.58477  3.61503
          0.04848  3.44667  4.16901  0.61958  0.77255  0.48576  0.95510
    272   2.34982  4.33050  2.62641  2.48485  3.68696  2.99628  3.72064
3.06482  2.55532  2.84509  3.86369  2.88140  3.64347  2.91133  2.86089
2.49610  2.73566  2.77325  5.12500  3.74704    321 a - - -
          2.68618  4.42225  2.77519  2.73123  3.46354  2.40513  3.72494
3.29354  2.67741  2.69355  4.24690  2.90347  2.73739  3.18146  2.89801
2.37887  2.77519  2.98518  4.58477  3.61503
          0.04848  3.44667  4.16901  0.61958  0.77255  0.48576  0.95510
    273   2.42203  4.36088  2.81597  2.57305  3.61624  3.08705  3.69918
2.97658  2.40594  2.71074  3.76758  2.97543  3.69658  2.91217  2.65438
2.56365  2.78678  2.74691  5.06295  3.70066    322 k - - -
          2.68618  4.42225  2.77519  2.73123  3.46354  2.40513  3.72494
3.29354  2.67741  2.69355  4.24690  2.90347  2.73739  3.18146  2.89801
2.37887  2.77519  2.98518  4.58477  3.61503
          0.04848  3.44667  4.16901  0.61958  0.77255  0.48576  0.95510
    274   2.44145  4.23476  3.32478  3.02891  3.26853  3.23138  3.94209
2.35069  2.81822  2.14978  3.43368  3.30710  3.83785  3.30168  3.04113
2.69809  2.80600  2.23485  4.94418  3.55120    323 l - - -
          2.68618  4.42225  2.77519  2.73123  3.46354  2.40513  3.72494
3.29354  2.67741  2.69355  4.24690  2.90347  2.73739  3.18146  2.89801
2.37887  2.77519  2.98518  4.58477  3.61503
          0.04848  3.44667  4.16901  0.61958  0.77255  0.48576  0.95510
    275   2.61635  4.35709  3.27267  2.96216  2.92295  3.36980  3.76522
2.48686  2.76655  2.14216  3.41384  3.28554  3.90749  3.23596  3.02263
2.82061  2.91904  2.38229  4.58589  2.89663    324 l - - -
          2.68618  4.42225  2.77519  2.73123  3.46354  2.40513  3.72494
3.29354  2.67741  2.69355  4.24690  2.90347  2.73739  3.18146  2.89801
2.37887  2.77519  2.98518  4.58477  3.61503
          0.04848  3.44667  4.16901  0.61958  0.77255  0.48576  0.95510
```

```
    276   2.36522  4.18505  2.93106  2.69225  3.53611  3.06072  3.74727
2.89692  2.52558  2.64909  3.66907  3.01510  3.68305  2.91547  2.81390
2.53372  2.69762  2.64329  5.02010  3.64963    325 a - - -
          2.68618  4.42225  2.77519  2.73123  3.46354  2.40513  3.72494
3.29354  2.67741  2.69355  4.24690  2.90347  2.73739  3.18146  2.89801
2.37887  2.77519  2.98518  4.58477  3.61503
          0.04848  3.44667  4.16901  0.61958  0.77255  0.48576  0.95510
    277   2.32062  4.27852  2.71885  2.52833  3.68593  2.98066  3.74160
3.04057  2.56990  2.79618  3.82522  2.93984  3.43125  2.98631  2.84504
2.47943  2.75103  2.73348  5.12009  3.77321    326 a - - -
          2.68618  4.42225  2.77519  2.73123  3.46354  2.40513  3.72494
3.29354  2.67741  2.69355  4.24690  2.90347  2.73739  3.18146  2.89801
2.37887  2.77519  2.98518  4.58477  3.61503
          0.04848  3.44667  4.16901  0.61958  0.77255  0.48576  0.95510
    278   2.17750  4.11955  3.10670  2.89923  3.50758  2.95605  3.92675
2.61354  2.81807  2.51296  3.66661  3.15385  3.66628  3.26234  3.07373
2.46623  2.68965  2.37198  5.08461  3.72055    327 a - - -
          2.68618  4.42225  2.77519  2.73123  3.46354  2.40513  3.72494
3.29354  2.67741  2.69355  4.24690  2.90347  2.73739  3.18146  2.89801
2.37887  2.77519  2.98518  4.58477  3.61503
          0.04848  3.44667  4.16901  0.61958  0.77255  0.48576  0.95510
    279   2.52676  4.38678  2.91439  2.64637  3.47143  3.17493  3.71258
2.77248  2.35088  2.52018  3.67488  3.04771  3.75443  2.97495  2.71685
2.67352  2.86738  2.61489  4.96693  3.58948    328 k - - -
          2.68618  4.42225  2.77519  2.73123  3.46354  2.40513  3.72494
3.29354  2.67741  2.69355  4.24690  2.90347  2.73739  3.18146  2.89801
2.37887  2.77519  2.98518  4.58477  3.61503
          0.04848  3.44667  4.16901  0.61958  0.77255  0.48576  0.95510
    280   2.45042  4.40586  2.59583  2.44621  3.61150  3.02328  3.66134
3.17704  2.47611  2.87608  3.89519  2.80293  3.65092  2.75992  2.77263
2.57001  2.83211  2.88807  5.02670  3.63264    329 e - - -
          2.68618  4.42225  2.77519  2.73123  3.46354  2.40513  3.72494
3.29354  2.67741  2.69355  4.24690  2.90347  2.73739  3.18146  2.89801
2.37887  2.77519  2.98518  4.58477  3.61503
          0.05154  3.44667  3.99609  0.61958  0.77255  0.48576  0.95510
    281   2.27376  4.22781  2.89084  2.70228  3.52749  2.99284  3.79270
2.90023  2.62407  2.62435  3.73870  3.03008  3.65485  3.00907  2.86580
2.46970  2.73420  2.63235  5.04368  3.66152    330 a - - -
          2.68618  4.42225  2.77519  2.73123  3.46354  2.40513  3.72494
3.29354  2.67741  2.69355  4.24690  2.90347  2.73739  3.18146  2.89801
2.37887  2.77519  2.98518  4.58477  3.61503
          0.04862  3.44374  4.16609  0.61958  0.77255  0.48750  0.95233
    282   2.55229  4.25137  3.46557  3.16349  3.12968  3.32715  4.00192
2.14850  2.95772  2.02005  3.33417  3.42900  3.91119  3.41627  3.19341
2.81790  2.92570  2.11890  4.85292  3.44543    331 l - - -
          2.68618  4.42225  2.77519  2.73123  3.46354  2.40513  3.72494
3.29354  2.67741  2.69355  4.24690  2.90347  2.73739  3.18146  2.89801
2.37887  2.77519  2.98518  4.58477  3.61503
          0.04862  3.44374  4.16609  0.61958  0.77255  0.48468  0.95684
    283   2.39955  4.33265  2.42964  2.43089  3.63515  2.92865  3.69211
3.20280  2.62519  2.98748  3.97436  2.65378  3.58836  3.00099  2.95499
2.48845  2.79306  2.87736  5.03346  3.64517    332 a - - -
          2.68618  4.42225  2.77519  2.73123  3.46354  2.40513  3.72494
3.29354  2.67741  2.69355  4.24690  2.90347  2.73739  3.18146  2.89801
2.37887  2.77519  2.98518  4.58477  3.61503
```

```
          0.04848  3.44667  4.16901  0.61958  0.77255  0.48576  0.95510
    284   2.27080  4.24954  2.76330  2.58981  3.58094  2.92124  3.66349
3.15763  2.55482  2.88598  3.88440  2.89452  3.61255  2.97931  2.81397
2.40893  2.73170  2.83125  5.02638  3.61915    333 a - - -
          2.68618  4.42225  2.77519  2.73123  3.46354  2.40513  3.72494
3.29354  2.67741  2.69355  4.24690  2.90347  2.73739  3.18146  2.89801
2.37887  2.77519  2.98518  4.58477  3.61503
          0.04848  3.44667  4.16901  0.61958  0.77255  0.48576  0.95510
    285   2.60779  4.22059  3.29496  3.07074  3.08236  3.14663  3.88887
2.45605  2.89514  1.84735  3.43157  3.36130  3.74617  3.36991  3.09781
2.84937  2.98220  2.34315  4.66431  3.32605    334 l - - -
          2.68618  4.42225  2.77519  2.73123  3.46354  2.40513  3.72494
3.29354  2.67741  2.69355  4.24690  2.90347  2.73739  3.18146  2.89801
2.37887  2.77519  2.98518  4.58477  3.61503
          0.04848  3.44667  4.16901  0.61958  0.77255  0.48576  0.95510
    286   2.67512  4.34851  3.38451  3.07253  2.53370  3.42205  3.74693
2.47773  2.96133  2.06282  3.34431  3.37212  3.94322  3.34357  3.21812
2.89500  2.97514  2.38267  4.38901  2.74552    335 l - - -
          2.68618  4.42225  2.77519  2.73123  3.46354  2.40513  3.72494
3.29354  2.67741  2.69355  4.24690  2.90347  2.73739  3.18146  2.89801
2.37887  2.77519  2.98518  4.58477  3.61503
          0.04848  3.44667  4.16901  0.61958  0.77255  0.48576  0.95510
    287   2.33355  4.25970  2.85528  2.62395  3.62888  3.02594  3.78891
2.84592  2.57370  2.67375  3.75629  2.98911  3.67345  3.04481  2.87104
2.52786  2.68671  2.55913  5.11740  3.76245    336 a - - -
          2.68618  4.42225  2.77519  2.73123  3.46354  2.40513  3.72494
3.29354  2.67741  2.69355  4.24690  2.90347  2.73739  3.18146  2.89801
2.37887  2.77519  2.98518  4.58477  3.61503
          0.04848  3.44667  4.16901  0.61958  0.77255  0.48576  0.95510
    288   2.40370  4.33894  2.40935  2.42254  3.64122  2.92994  3.69101
3.20375  2.62755  2.98880  3.97555  2.66448  3.58881  2.99830  2.96081
2.49086  2.79584  2.87924  5.03825  3.65095    337 a - - -
          2.68618  4.42225  2.77519  2.73123  3.46354  2.40513  3.72494
3.29354  2.67741  2.69355  4.24690  2.90347  2.73739  3.18146  2.89801
2.37887  2.77519  2.98518  4.58477  3.61503
          0.04848  3.44667  4.16901  0.61958  0.77255  0.48576  0.95510
    289   2.32854  4.25723  2.84994  2.61342  3.54989  3.00101  3.76682
2.87601  2.61267  2.69674  3.75800  3.00646  3.62221  3.07259  2.89813
2.48067  2.73359  2.60096  5.04283  3.56566    338 a - - -
          2.68618  4.42225  2.77519  2.73123  3.46354  2.40513  3.72494
3.29354  2.67741  2.69355  4.24690  2.90346  2.73739  3.18146  2.89801
2.37887  2.77520  2.98518  4.58477  3.61503
          0.06597  3.02882  4.16901  0.57951  0.82138  0.48576  0.95510
    290   2.61890  4.39666  3.13157  2.77562  3.02546  3.27620  3.60651
2.92028  2.45184  2.41165  3.55006  3.13803  3.78925  2.77999  2.71607
2.76455  2.91632  2.75630  4.07665  3.09756    340 l - - -
          2.68618  4.42225  2.77519  2.73123  3.46354  2.40513  3.72494
3.29354  2.67741  2.69355  4.24690  2.90347  2.73739  3.18146  2.89801
2.37887  2.77519  2.98518  4.58477  3.61503
          0.04848  3.44667  4.16901  0.61958  0.77255  0.48576  0.95510
    291   2.47513  4.38944  2.58296  2.49924  3.38774  3.05834  3.65515
2.99636  2.58472  2.80196  3.81451  2.84653  3.68635  2.98940  2.91089
2.59046  2.82629  2.75321  4.87016  3.28224    341 a - - -
```

2.68618 4.42225 2.77519 2.73123 3.46354 2.40513 3.72494
3.29354 2.67741 2.69355 4.24690 2.90347 2.73739 3.18146 2.89801
2.37887 2.77519 2.98518 4.58477 3.61503
0.04848 3.44667 4.16901 0.61958 0.77255 0.48576 0.95510
292 2.35103 4.28573 2.67259 2.50208 3.68841 2.88471 3.72222
3.10832 2.55886 2.88281 3.89197 2.88324 3.61991 2.95733 2.85698
2.44722 2.71038 2.79176 5.10844 3.73296 342 a - - -
2.68618 4.42225 2.77519 2.73123 3.46354 2.40513 3.72494
3.29354 2.67741 2.69355 4.24690 2.90347 2.73739 3.18146 2.89801
2.37887 2.77519 2.98518 4.58477 3.61503
0.04848 3.44667 4.16901 0.61958 0.77255 0.48576 0.95510
293 2.40991 4.37646 2.62332 2.47702 3.62592 3.01350 3.62045
3.21295 2.43013 2.92268 3.90351 2.85432 3.64704 2.88098 2.76691
2.46823 2.79490 2.89924 5.04506 3.63614 343 a - - -
2.68618 4.42225 2.77519 2.73123 3.46354 2.40513 3.72494
3.29354 2.67741 2.69355 4.24690 2.90347 2.73739 3.18146 2.89801
2.37887 2.77519 2.98518 4.58477 3.61503
0.04848 3.44667 4.16901 0.61958 0.77255 0.48576 0.95510
294 2.59403 4.28881 3.45479 3.14885 3.06827 3.36154 3.96209
2.24191 2.91498 1.92598 3.32504 3.42553 3.93057 3.39189 3.14770
2.85248 2.95682 2.17936 4.78947 3.35763 344 l - - -
2.68618 4.42225 2.77519 2.73123 3.46354 2.40513 3.72494
3.29354 2.67741 2.69355 4.24690 2.90347 2.73739 3.18146 2.89801
2.37887 2.77519 2.98518 4.58477 3.61503
0.04848 3.44667 4.16901 0.61958 0.77255 0.48576 0.95510
295 2.50518 4.42923 2.77625 2.54354 3.53704 3.10649 3.56634
3.18632 2.29531 2.80331 3.83792 2.89615 3.69544 2.86519 2.52199
2.61250 2.84493 2.90891 4.94827 3.54824 345 k - - -
2.68618 4.42225 2.77519 2.73123 3.46354 2.40513 3.72494
3.29354 2.67741 2.69355 4.24690 2.90347 2.73739 3.18146 2.89801
2.37887 2.77519 2.98518 4.58477 3.61503
0.04848 3.44667 4.16901 0.61958 0.77255 0.48576 0.95510
296 2.33939 4.19732 3.04654 2.79594 3.16986 3.04972 3.78060
2.79114 2.71987 2.53437 3.65552 3.07598 3.48002 3.15346 2.97290
2.49782 2.74934 2.55409 4.85064 3.41791 346 a - - -
2.68618 4.42225 2.77519 2.73123 3.46354 2.40513 3.72494
3.29354 2.67741 2.69355 4.24690 2.90347 2.73739 3.18146 2.89801
2.37887 2.77519 2.98518 4.58477 3.61503
0.04848 3.44667 4.16901 0.61958 0.77255 0.48576 0.95510
297 2.45326 4.45386 2.41765 2.34524 3.63003 3.03746 3.59437
3.16058 2.54631 2.90445 3.89588 2.80956 3.65544 2.93716 2.89035
2.57649 2.83830 2.87247 5.05184 3.59731 347 e - - -
2.68618 4.42225 2.77519 2.73123 3.46354 2.40513 3.72495
3.29353 2.67741 2.69355 4.24690 2.90347 2.73740 3.18147 2.89801
2.37886 2.77519 2.98517 4.58477 3.61499
0.06579 3.03226 4.16901 0.67008 0.71676 0.48576 0.95510
298 2.40992 4.28291 2.94587 2.71933 3.41646 3.07935 3.71169
2.83832 2.58766 2.51255 3.65372 3.06095 3.70708 2.97289 2.85396
2.56992 2.68035 2.61382 4.96019 3.55924 351 a - - -
2.68618 4.42225 2.77519 2.73123 3.46354 2.40513 3.72494
3.29354 2.67741 2.69355 4.24690 2.90347 2.73739 3.18146 2.89801
2.37887 2.77519 2.98518 4.58477 3.61503
0.04848 3.44667 4.16901 0.61958 0.77255 0.48576 0.95510

```
     299   2.35539  4.29179  2.66316  2.49308  3.66906  2.93640  3.65152
 3.13612  2.55293  2.92196  3.90158  2.83525  3.61822  3.00189  2.85139
 2.46685  2.65002  2.81459  5.08773  3.70355    352 a - - -
           2.68618  4.42225  2.77519  2.73123  3.46354  2.40513  3.72494
 3.29354  2.67741  2.69355  4.24690  2.90347  2.73739  3.18146  2.89801
 2.37887  2.77519  2.98518  4.58477  3.61503
           0.04848  3.44667  4.16901  0.61958  0.77255  0.48576  0.95510
     300   2.61589  4.31964  3.45763  3.10437  2.95338  3.42705  3.90331
 2.28739  2.84911  1.97498  3.28232  3.40450  3.95949  3.33830  3.12666
 2.86559  2.95415  2.21619  4.72841  3.26781    353 l - - -
           2.68618  4.42225  2.77519  2.73123  3.46354  2.40513  3.72494
 3.29354  2.67741  2.69355  4.24690  2.90347  2.73739  3.18146  2.89801
 2.37887  2.77519  2.98518  4.58477  3.61503
           0.04848  3.44667  4.16901  0.61958  0.77255  0.48576  0.95510
     301   2.30978  4.27105  2.76731  2.58730  3.65927  3.00173  3.76012
 2.95284  2.55522  2.75861  3.80438  2.96942  3.65217  3.00930  2.84083
 2.47454  2.71921  2.65838  5.12139  3.75873    354 a - - -
           2.68618  4.42225  2.77519  2.73123  3.46354  2.40513  3.72494
 3.29354  2.67741  2.69355  4.24690  2.90347  2.73739  3.18146  2.89801
 2.37887  2.77519  2.98518  4.58477  3.61503
           0.05154  3.44667  3.99609  0.61958  0.77255  0.48576  0.95510
     302   2.40341  4.37703  2.64271  2.48044  3.58809  3.04306  3.62656
 3.12191  2.47299  2.86016  3.79922  2.83081  3.66421  2.91404  2.78893
 2.54695  2.79781  2.82932  5.02470  3.61452    355 a - - -
           2.68618  4.42225  2.77519  2.73123  3.46354  2.40513  3.72494
 3.29354  2.67741  2.69355  4.24690  2.90347  2.73739  3.18146  2.89801
 2.37887  2.77519  2.98518  4.58477  3.61503
           0.04862  3.44374  4.16609  0.61958  0.77255  0.48468  0.95684
     303   2.57590  4.25938  3.46742  3.16708  3.11966  3.34052  4.00157
 2.10394  2.96379  2.00804  3.32675  3.43956  3.91725  3.42176  3.19775
 2.84520  2.94364  2.13303  4.83830  3.43751    356 l - - -
           2.68618  4.42225  2.77519  2.73123  3.46354  2.40513  3.72494
 3.29354  2.67741  2.69355  4.24690  2.90347  2.73739  3.18146  2.89801
 2.37887  2.77519  2.98518  4.58477  3.61503
           0.04848  3.44667  4.16901  0.61958  0.77255  0.48576  0.95510
     304   2.48517  4.27850  3.30250  2.96367  3.15120  3.27276  3.79685
 2.49971  2.73667  2.16716  3.31922  3.26863  3.84402  3.19670  2.99407
 2.70927  2.81261  2.34892  4.82983  3.40689    357 l - - -
           2.68618  4.42225  2.77519  2.73123  3.46354  2.40513  3.72494
 3.29354  2.67741  2.69355  4.24690  2.90347  2.73739  3.18146  2.89801
 2.37887  2.77519  2.98518  4.58477  3.61503
           0.06268  3.44667  3.54383  0.61958  0.77255  0.48576  0.95510
     305   2.34844  4.27756  2.72597  2.54864  3.52465  2.99703  3.74550
 2.90352  2.61770  2.74296  3.81509  2.94279  3.64816  3.06644  2.90492
 2.50581  2.78853  2.64087  5.00175  3.55944    358 a - - -
           2.68618  4.42225  2.77519  2.73123  3.46354  2.40513  3.72494
 3.29354  2.67741  2.69355  4.24690  2.90347  2.73739  3.18146  2.89801
 2.37887  2.77519  2.98518  4.58477  3.61503
           0.04915  3.43314  4.15549  0.61958  0.77255  0.49375  0.94247
     306   2.14789  4.10383  3.04825  2.89289  3.40277  2.91749  3.89432
 2.65457  2.85017  2.40386  3.65191  3.16773  3.59313  3.29093  3.07574
 2.56464  2.79092  2.45049  4.89576  3.62334    359 a - - -
           2.68618  4.42225  2.77519  2.73123  3.46354  2.40513  3.72494
 3.29354  2.67741  2.69355  4.24690  2.90347  2.73739  3.18146  2.89801
 2.37887  2.77519  2.98518  4.58477  3.61503
```

```
          0.04915  3.43314  4.15549  0.61958  0.77255  0.49375  0.94247
    307   2.45631  4.35940  2.55630  2.44014  3.59082  2.98955  3.67135
3.11532  2.51899  2.84859  3.88167  2.85106  3.62206  2.91154  2.76413
2.56893  2.82718  2.83335  4.99055  3.62681    360 e - - -
          2.68618  4.42225  2.77519  2.73123  3.46354  2.40513  3.72494
3.29354  2.67741  2.69355  4.24690  2.90347  2.73739  3.18146  2.89801
2.37887  2.77519  2.98518  4.58477  3.61503
          0.04915  3.43314  4.15549  0.61958  0.77255  0.49375  0.94247
    308   2.42037  4.32067  2.70033  2.55052  3.48648  2.97654  3.62715
3.07262  2.47118  2.82855  3.85627  2.88108  3.64261  3.00105  2.80285
2.54743  2.76835  2.78696  4.93326  3.47676    361 a - - -
          2.68618  4.42225  2.77519  2.73123  3.46354  2.40513  3.72494
3.29354  2.67741  2.69355  4.24690  2.90347  2.73739  3.18146  2.89801
2.37887  2.77519  2.98518  4.58477  3.61503
          0.04915  3.43314  4.15549  0.61958  0.77255  0.49375  0.94247
    309   2.56175  4.29523  3.35152  3.02163  3.03526  3.29170  3.81943
2.44725  2.73507  2.08457  3.39285  3.31293  3.85530  3.24822  2.94147
2.77872  2.89027  2.38241  4.56132  3.23923    362 l - - -
          2.68618  4.42225  2.77519  2.73123  3.46354  2.40513  3.72494
3.29354  2.67741  2.69355  4.24690  2.90347  2.73739  3.18146  2.89801
2.37887  2.77519  2.98518  4.58477  3.61503
          0.04915  3.43314  4.15549  0.61958  0.77255  0.48080  0.96308
    310   2.46839  4.22260  3.39301  3.11467  3.18611  3.24204  3.98586
2.33010  2.92491  2.06098  3.39524  3.36960  3.85570  3.38615  3.16200
2.73288  2.87709  2.11643  4.88990  3.48178    363 l - - -
          2.68618  4.42225  2.77519  2.73123  3.46354  2.40513  3.72494
3.29354  2.67741  2.69355  4.24690  2.90347  2.73739  3.18146  2.89801
2.37887  2.77519  2.98518  4.58477  3.61503
          0.04848  3.44667  4.16901  0.61958  0.77255  0.48576  0.95510
    311   2.43194  4.37589  2.71361  2.50228  3.55117  3.05247  3.62249
3.12926  2.47833  2.76369  3.80362  2.92512  3.47080  2.85782  2.76849
2.54802  2.81024  2.85823  4.99947  3.56523    364 a - - -
          2.68618  4.42225  2.77519  2.73123  3.46354  2.40513  3.72494
3.29354  2.67741  2.69355  4.24690  2.90347  2.73739  3.18146  2.89801
2.37887  2.77519  2.98518  4.58477  3.61503
          0.04848  3.44667  4.16901  0.61958  0.77255  0.48576  0.95510
    312   2.30418  4.21189  2.80653  2.64597  3.60959  2.95561  3.76745
2.95986  2.59890  2.82733  3.84331  2.94911  3.62316  3.07284  2.87246
2.37655  2.65531  2.68091  5.07187  3.68319    365 a - - -
          2.68618  4.42225  2.77519  2.73123  3.46354  2.40513  3.72494
3.29354  2.67741  2.69355  4.24690  2.90347  2.73739  3.18146  2.89801
2.37887  2.77519  2.98518  4.58477  3.61503
          0.04848  3.44667  4.16901  0.61958  0.77255  0.48576  0.95510
    313   2.58465  4.28487  3.39718  3.08962  3.12908  3.33923  3.93874
2.20194  2.83752  2.02683  3.36288  3.38454  3.90512  3.34555  3.09910
2.83609  2.94104  2.20187  4.81922  3.40892    366 l - - -
          2.68618  4.42225  2.77519  2.73123  3.46354  2.40513  3.72494
3.29354  2.67741  2.69355  4.24690  2.90347  2.73739  3.18146  2.89801
2.37887  2.77519  2.98518  4.58477  3.61503
          0.04848  3.44667  4.16901  0.61958  0.77255  0.48576  0.95510
    314   2.34539  4.19338  2.95163  2.73035  3.42035  2.97099  3.76908
2.94322  2.63603  2.61248  3.73085  3.05214  3.19090  3.10533  2.81761
2.48865  2.74871  2.68448  4.93767  3.59206    367 a - - -
```

```
          2.68618  4.42223  2.77519  2.73123  3.46353  2.40513  3.72494
3.29354  2.67741  2.69354  4.24690  2.90346  2.73739  3.18147  2.89801
2.37887  2.77519  2.98519  4.58477  3.61502
          0.05171  3.35435  4.16901  0.74153  0.64700  0.48576  0.95510
    315   2.56632  4.47249  2.64870  2.43661  3.51534  3.14974  3.63749
3.00710  2.36546  2.75097  3.78346  2.92240  3.71918  2.92957  2.74343
2.67072  2.88269  2.76713  4.94986  3.38870    384 k - - -
          2.68618  4.42225  2.77519  2.73123  3.46354  2.40513  3.72494
3.29354  2.67741  2.69355  4.24690  2.90347  2.73739  3.18146  2.89801
2.37887  2.77519  2.98518  4.58477  3.61503
          0.04848  3.44667  4.16901  0.61958  0.77255  0.48576  0.95510
    316   2.43200  4.22945  3.31389  3.03461  3.23421  3.21462  3.94433
2.39530  2.84946  2.14092  3.42744  3.30700  3.82949  3.27920  3.09556
2.69013  2.85000  2.19774  4.91732  3.51315    385 l - - -
          2.68618  4.42225  2.77519  2.73123  3.46354  2.40513  3.72494
3.29354  2.67741  2.69355  4.24690  2.90347  2.73739  3.18146  2.89801
2.37887  2.77519  2.98518  4.58477  3.61503
          0.04848  3.44667  4.16901  0.61958  0.77255  0.48576  0.95510
    317   2.58900  4.38378  2.93685  2.70682  3.04673  3.21053  3.63224
2.86857  2.54834  2.51361  3.63941  3.08513  3.76608  3.05821  2.78455
2.73233  2.91220  2.67721  4.58857  2.81068    386 l - - -
          2.68618  4.42225  2.77519  2.73123  3.46354  2.40513  3.72494
3.29354  2.67741  2.69355  4.24690  2.90347  2.73739  3.18146  2.89801
2.37887  2.77519  2.98518  4.58477  3.61503
          0.04848  3.44667  4.16901  0.61958  0.77255  0.48576  0.95510
    318   2.43008  4.34313  2.80758  2.59444  3.31739  3.06137  3.45051
3.14506  2.49034  2.80688  3.78729  2.85195  3.68016  2.98969  2.76742
2.52411  2.78123  2.85194  4.80655  3.32248    387 a - - -
          2.68618  4.42225  2.77519  2.73123  3.46354  2.40513  3.72494
3.29354  2.67741  2.69355  4.24690  2.90347  2.73739  3.18146  2.89801
2.37887  2.77519  2.98518  4.58477  3.61503
          0.04848  3.44667  4.16901  0.61958  0.77255  0.48576  0.95510
    319   2.47713  4.33878  3.03096  2.68966  3.19784  3.24069  3.64825
2.84556  2.51885  2.51772  3.57385  3.08961  3.72435  2.98589  2.80096
2.64704  2.81875  2.65016  4.29589  3.27753    388 a - - -
          2.68618  4.42225  2.77519  2.73123  3.46354  2.40513  3.72494
3.29354  2.67741  2.69355  4.24690  2.90347  2.73739  3.18146  2.89801
2.37887  2.77519  2.98518  4.58477  3.61503
          0.04848  3.44667  4.16901  0.61958  0.77255  0.48576  0.95510
    320   2.62085  4.34369  3.47126  3.01461  2.73746  3.46682  3.72806
2.53056  2.79218  2.06611  3.28179  3.34798  3.94301  3.23162  3.05417
2.85106  2.91703  2.41764  4.18677  2.96438    389 l - - -
          2.68618  4.42225  2.77519  2.73123  3.46354  2.40513  3.72494
3.29354  2.67741  2.69355  4.24690  2.90347  2.73739  3.18146  2.89801
2.37887  2.77519  2.98518  4.58477  3.61503
          0.04848  3.44667  4.16901  0.61958  0.77255  0.48576  0.95510
    321   2.53811  4.27779  3.40798  3.06126  3.07193  3.33476  3.88373
2.36681  2.81694  2.09326  3.29416  3.31665  3.89821  3.29750  3.06627
2.75828  2.89438  2.23353  4.77421  3.31748    390 l - - -
          2.68618  4.42225  2.77519  2.73123  3.46354  2.40513  3.72494
3.29354  2.67741  2.69355  4.24690  2.90347  2.73739  3.18146  2.89801
2.37887  2.77519  2.98518  4.58477  3.61503
          0.04848  3.44667  4.16901  0.61958  0.77255  0.48576  0.95510
```

322 2.38246 4.28073 2.77684 2.58808 3.50405 3.02631 3.73128
2.95059 2.60164 2.72276 3.78391 2.97820 3.55711 3.04734 2.89602
2.44260 2.74748 2.67954 4.98705 3.49172 391 a - - -
2.68618 4.42225 2.77519 2.73123 3.46354 2.40513 3.72494
3.29354 2.67741 2.69355 4.24690 2.90347 2.73739 3.18146 2.89801
2.37887 2.77519 2.98518 4.58477 3.61503
0.04848 3.44667 4.16901 0.61958 0.77255 0.48576 0.95510
323 2.39013 4.33386 2.59578 2.47586 3.62545 2.97462 3.63619
3.11711 2.56090 2.87066 3.85745 2.87262 3.59476 2.95731 2.87885
2.50998 2.74226 2.75813 5.06032 3.66504 392 a - - -
2.68618 4.42225 2.77519 2.73123 3.46354 2.40513 3.72494
3.29354 2.67741 2.69355 4.24690 2.90347 2.73739 3.18146 2.89801
2.37887 2.77519 2.98518 4.58477 3.61503
0.04848 3.44667 4.16901 0.61958 0.77255 0.48576 0.95510
324 2.53292 4.32021 3.19916 2.85362 3.23857 3.27611 3.80379
2.47181 2.63695 2.26456 3.47978 3.21643 3.83691 3.15508 2.82740
2.73100 2.87648 2.37746 4.85061 3.40201 393 l - - -
2.68618 4.42225 2.77519 2.73123 3.46354 2.40513 3.72494
3.29354 2.67741 2.69355 4.24690 2.90347 2.73739 3.18146 2.89801
2.37887 2.77519 2.98518 4.58477 3.61503
0.04848 3.44667 4.16901 0.61958 0.77255 0.48576 0.95510
325 2.35245 4.22241 2.91126 2.67306 3.43922 3.00289 3.77612
2.87370 2.64947 2.61920 3.71430 3.03583 3.24345 3.06653 2.92942
2.49730 2.75087 2.64903 4.98448 3.61634 394 a - - -
2.68618 4.42225 2.77519 2.73123 3.46354 2.40513 3.72494
3.29354 2.67741 2.69355 4.24690 2.90347 2.73739 3.18146 2.89801
2.37887 2.77519 2.98518 4.58477 3.61503
0.04848 3.44667 4.16901 0.61958 0.77255 0.48576 0.95510
326 2.28058 4.06606 2.84778 2.71280 3.61755 2.31492 3.79052
3.13272 2.73963 2.88203 3.93092 3.01605 3.46047 3.16546 2.92524
2.41333 2.72272 2.79825 4.90819 3.70949 395 a - - -
2.68618 4.42225 2.77519 2.73123 3.46354 2.40513 3.72494
3.29354 2.67741 2.69355 4.24690 2.90347 2.73739 3.18146 2.89801
2.37887 2.77519 2.98518 4.58477 3.61503
0.04848 3.44667 4.16901 0.61958 0.77255 0.48576 0.95510
327 2.52722 4.31566 3.19485 2.79543 3.34093 3.28675 3.85126
2.46338 2.66240 2.24460 3.33301 3.22383 3.84814 3.16947 2.92677
2.73468 2.85429 2.30051 4.96278 3.58796 396 l - - -
2.68618 4.42225 2.77519 2.73123 3.46354 2.40513 3.72494
3.29354 2.67741 2.69355 4.24690 2.90347 2.73739 3.18146 2.89801
2.37887 2.77519 2.98518 4.58477 3.61503
0.04848 3.44667 4.16901 0.61958 0.77255 0.48576 0.95510
328 2.46289 4.38979 2.69734 2.52222 3.32968 3.04864 3.51880
3.21954 2.49256 2.87752 3.88638 2.72080 3.67096 2.94503 2.78028
2.55456 2.82975 2.92102 4.80923 3.27725 397 a - - -
2.68618 4.42225 2.77519 2.73123 3.46354 2.40513 3.72494
3.29354 2.67741 2.69355 4.24690 2.90347 2.73739 3.18146 2.89801
2.37887 2.77519 2.98518 4.58477 3.61503
0.04848 3.44667 4.16901 0.61958 0.77255 0.48576 0.95510
329 2.50293 4.35326 2.98528 2.71611 2.96662 3.14337 3.53321
2.91363 2.60542 2.57267 3.65639 3.05254 3.76940 3.06381 2.88625
2.66191 2.85605 2.67429 4.56179 2.90056 398 a - - -
2.68618 4.42225 2.77519 2.73123 3.46354 2.40513 3.72494
3.29354 2.67741 2.69355 4.24690 2.90347 2.73739 3.18146 2.89801
2.37887 2.77519 2.98518 4.58477 3.61503

```
         0.04848  3.44667  4.16901  0.61958  0.77255  0.48576  0.95510
    330  2.36365  4.29903  2.61205  2.49892  3.68015  3.00205  3.75836
2.93002  2.63675  2.78642  3.82162  2.93100  3.60625  3.03953  2.95750
2.49958  2.72783  2.61147  5.13898  3.77397    399 a - - -
         2.68618  4.42225  2.77519  2.73123  3.46354  2.40513  3.72494
3.29354  2.67741  2.69355  4.24690  2.90347  2.73739  3.18146  2.89801
2.37887  2.77519  2.98518  4.58477  3.61503
         0.04848  3.44667  4.16901  0.61958  0.77255  0.48576  0.95510
    331  2.58869  4.29046  3.39545  3.08752  3.11715  3.34226  3.93142
2.23250  2.83060  2.00634  3.36242  3.38266  3.90703  3.34107  3.09027
2.83804  2.94437  2.21206  4.80791  3.39153    400 l - - -
         2.68618  4.42225  2.77519  2.73123  3.46354  2.40513  3.72494
3.29354  2.67741  2.69355  4.24690  2.90347  2.73739  3.18146  2.89801
2.37887  2.77519  2.98518  4.58477  3.61503
         0.04848  3.44667  4.16901  0.61958  0.77255  0.48576  0.95510
    332  2.63169  4.34875  3.33945  2.97523  2.75049  3.42295  3.78791
2.44887  2.85927  2.08697  3.31710  3.32859  3.93865  3.27656  3.12986
2.85109  2.92841  2.33765  4.56382  2.98756    401 l - - -
         2.68618  4.42225  2.77519  2.73123  3.46354  2.40513  3.72494
3.29354  2.67741  2.69355  4.24690  2.90347  2.73739  3.18146  2.89801
2.37887  2.77519  2.98518  4.58477  3.61503
         0.04848  3.44667  4.16901  0.61958  0.77255  0.48576  0.95510
    333  2.37199  4.26976  2.97014  2.70881  3.49719  3.08840  3.78189
2.79079  2.58389  2.55180  3.58573  3.03530  3.71230  3.00093  2.85361
2.56561  2.72776  2.53919  5.02505  3.64723    402 a - - -
         2.68618  4.42225  2.77519  2.73123  3.46354  2.40513  3.72494
3.29354  2.67741  2.69355  4.24690  2.90347  2.73739  3.18146  2.89801
2.37887  2.77519  2.98518  4.58477  3.61503
         0.04848  3.44667  4.16901  0.61958  0.77255  0.48576  0.95510
    334  2.52422  4.45884  2.61211  2.31900  3.71717  3.06944  3.66031
3.10601  2.38340  2.83594  3.86974  2.88401  3.67658  2.89379  2.68810
2.59763  2.86852  2.84293  5.09886  3.75727    403 e - - -
         2.68618  4.42225  2.77519  2.73123  3.46354  2.40513  3.72494
3.29354  2.67741  2.69355  4.24690  2.90347  2.73739  3.18146  2.89801
2.37887  2.77519  2.98518  4.58477  3.61503
         0.04848  3.44667  4.16901  0.61958  0.77255  0.48576  0.95510
    335  2.65420  4.32005  3.51539  3.18505  2.78439  3.43866  3.91210
2.30809  2.99307  1.86868  3.22751  3.46433  3.97478  3.41884  3.23334
2.91408  2.99823  2.22513  4.61883  3.13517    404 l - - -
         2.68618  4.42225  2.77519  2.73123  3.46354  2.40513  3.72494
3.29354  2.67741  2.69355  4.24690  2.90347  2.73739  3.18146  2.89801
2.37887  2.77519  2.98518  4.58477  3.61503
         0.04848  3.44667  4.16901  0.61958  0.77255  0.48576  0.95510
    336  2.47906  4.44423  2.57943  2.38098  3.73763  3.05242  3.65887
3.15997  2.39806  2.90552  3.89748  2.84197  3.66965  2.84878  2.73353
2.54664  2.81406  2.86975  5.12845  3.75679    405 e - - -
         2.68618  4.42225  2.77519  2.73123  3.46354  2.40513  3.72494
3.29354  2.67741  2.69355  4.24690  2.90347  2.73739  3.18146  2.89801
2.37887  2.77519  2.98518  4.58477  3.61503
         0.04848  3.44667  4.16901  0.61958  0.77255  0.48576  0.95510
    337  2.34808  4.32424  2.55547  2.42211  3.75706  2.87842  3.70846
3.16545  2.56490  2.96390  3.93359  2.79869  3.60920  2.95324  2.88167
2.48958  2.74280  2.84013  5.14787  3.78659    406 a - - -
```

```
         2.68618  4.42225  2.77519  2.73123  3.46354  2.40513  3.72494
3.29354  2.67741  2.69355  4.24690  2.90347  2.73739  3.18146  2.89801
2.37887  2.77519  2.98518  4.58477  3.61503
         0.04848  3.44667  4.16901  0.61958  0.77255  0.48576  0.95510
    338  2.34292  4.27421  2.91966  2.69580  3.52030  3.02234  3.73333
2.97177  2.49890  2.62646  3.74133  3.04046  3.65150  2.99786  2.54358
2.57678  2.80854  2.73263  4.96313  3.63585    407 a - - -
         2.68618  4.42225  2.77519  2.73123  3.46354  2.40513  3.72494
3.29354  2.67741  2.69355  4.24690  2.90347  2.73739  3.18146  2.89801
2.37887  2.77519  2.98518  4.58477  3.61503
         0.04848  3.44667  4.16901  0.61958  0.77255  0.48576  0.95510
    339  2.58989  4.28563  3.47364  3.16006  3.07877  3.37123  3.97502
2.21589  2.92567  1.93720  3.31808  3.43454  3.93879  3.39929  3.16019
2.85214  2.95256  2.15524  4.80706  3.37778    408 l - - -
         2.68618  4.42225  2.77519  2.73123  3.46354  2.40513  3.72494
3.29354  2.67741  2.69355  4.24690  2.90347  2.73739  3.18146  2.89801
2.37887  2.77519  2.98518  4.58477  3.61503
         0.04848  3.44667  4.16901  0.61958  0.77255  0.48576  0.95510
    340  2.44651  4.39932  2.66186  2.50701  3.44100  3.08700  3.66094
3.05606  2.49483  2.78018  3.80368  2.87102  3.69171  2.86473  2.81920
2.58513  2.82244  2.79004  4.94905  3.47599    409 a - - -
         2.68618  4.42225  2.77519  2.73123  3.46354  2.40513  3.72494
3.29354  2.67741  2.69355  4.24690  2.90347  2.73739  3.18146  2.89801
2.37887  2.77519  2.98518  4.58477  3.61503
         0.04848  3.44667  4.16901  0.61958  0.77255  0.48576  0.95510
    341  2.49339  4.43059  2.73463  2.51990  3.56003  3.08746  3.56950
3.21947  2.33644  2.84126  3.85171  2.88146  3.68587  2.80647  2.55451
2.59718  2.81809  2.93591  4.97290  3.57116    410 k - - -
         2.68618  4.42225  2.77519  2.73123  3.46354  2.40513  3.72494
3.29354  2.67741  2.69355  4.24690  2.90347  2.73739  3.18146  2.89801
2.37887  2.77519  2.98518  4.58477  3.61503
         0.04848  3.44667  4.16901  0.61958  0.77255  0.48576  0.95510
    342  2.01129  4.08183  3.03857  2.88807  3.49899  2.88360  3.91566
2.70751  2.87250  2.51233  3.70596  3.15505  3.57096  3.29808  3.10187
2.52494  2.76285  2.48103  4.96008  3.72186    411 a - - -
         2.68618  4.42225  2.77519  2.73123  3.46354  2.40513  3.72494
3.29354  2.67741  2.69355  4.24690  2.90347  2.73739  3.18146  2.89801
2.37887  2.77519  2.98518  4.58477  3.61503
         0.05154  3.44667  3.99609  0.61958  0.77255  0.48576  0.95510
    343  2.62020  4.37726  3.28600  2.90499  2.69952  3.38899  3.56938
2.71718  2.67430  2.26775  3.45396  3.23262  3.88647  3.13850  2.90440
2.77398  2.92392  2.58981  4.31654  2.80432    412 l - - -
         2.68618  4.42225  2.77519  2.73123  3.46354  2.40513  3.72494
3.29354  2.67741  2.69355  4.24690  2.90347  2.73739  3.18146  2.89801
2.37887  2.77519  2.98518  4.58477  3.61503
         0.04862  3.44374  4.16609  0.61958  0.77255  0.48468  0.95684
    344  2.40136  4.33880  2.76239  2.56481  3.48096  3.07649  3.70600
2.95138  2.55221  2.71784  3.76703  2.94789  3.69486  2.96864  2.84645
2.53894  2.75891  2.69495  4.97255  3.44252    413 a - - -
         2.68618  4.42225  2.77519  2.73123  3.46354  2.40513  3.72494
3.29354  2.67741  2.69355  4.24690  2.90347  2.73739  3.18146  2.89801
2.37887  2.77519  2.98518  4.58477  3.61503
         0.04848  3.44667  4.16901  0.61958  0.77255  0.48576  0.95510
```

345 2.56348 4.31671 3.09451 2.85915 3.16071 3.18051 3.78762
2.65828 2.67526 2.10054 3.50553 3.19639 3.77224 3.01141 2.91508
2.74916 2.92482 2.52373 4.75115 3.34592 414 l - - -
2.68618 4.42225 2.77519 2.73123 3.46354 2.40513 3.72494
3.29354 2.67741 2.69355 4.24690 2.90347 2.73739 3.18146 2.89801
2.37887 2.77519 2.98518 4.58477 3.61503
0.04848 3.44667 4.16901 0.61958 0.77255 0.48576 0.95510
346 2.66582 4.37846 3.25569 2.96094 2.79631 3.37047 3.70422
2.55945 2.77312 2.14476 3.43724 3.28386 3.90149 3.23751 3.02206
2.85869 2.99023 2.46466 4.44536 2.68165 415 l - - -
2.68618 4.42225 2.77519 2.73123 3.46354 2.40513 3.72494
3.29354 2.67741 2.69355 4.24690 2.90347 2.73739 3.18146 2.89801
2.37887 2.77519 2.98518 4.58477 3.61503
0.04848 3.44667 4.16901 0.61958 0.77255 0.48576 0.95510
347 2.47135 4.41697 2.48167 2.38597 3.71128 3.05263 3.69772
3.00332 2.53304 2.82510 3.78981 2.87482 3.66940 2.96347 2.88939
2.58004 2.78295 2.73720 5.13340 3.77178 416 e - - -
2.68618 4.42225 2.77519 2.73123 3.46354 2.40513 3.72494
3.29354 2.67741 2.69355 4.24690 2.90347 2.73739 3.18146 2.89801
2.37887 2.77519 2.98518 4.58477 3.61503
0.04848 3.44667 4.16901 0.61958 0.77255 0.48576 0.95510
348 2.36507 4.27304 2.94364 2.67253 3.52493 3.08540 3.79049
2.77397 2.59439 2.54365 3.61466 3.06082 3.71251 3.03784 2.86721
2.53634 2.74982 2.53515 5.05250 3.68061 417 a - - -
2.68618 4.42225 2.77519 2.73123 3.46354 2.40513 3.72494
3.29354 2.67741 2.69355 4.24690 2.90347 2.73739 3.18146 2.89801
2.37887 2.77519 2.98518 4.58477 3.61503
0.04848 3.44667 4.16901 0.61958 0.77255 0.48576 0.95510
349 2.46118 4.34994 2.85326 2.61984 3.54602 3.06107 3.67001
3.09490 2.37301 2.74377 3.80308 2.98937 3.67155 2.90796 2.47426
2.55142 2.82444 2.83239 4.96420 3.60127 418 k - - -
2.68618 4.42225 2.77519 2.73123 3.46354 2.40513 3.72494
3.29354 2.67741 2.69355 4.24690 2.90347 2.73739 3.18146 2.89801
2.37887 2.77519 2.98518 4.58477 3.61503
0.04848 3.44667 4.16901 0.61958 0.77255 0.48576 0.95510
350 2.36159 4.21695 2.75450 2.61687 3.49390 2.89908 3.73249
3.08908 2.64011 2.86049 3.91653 2.54397 3.57107 3.08903 2.90883
2.50529 2.74340 2.78573 4.90869 3.53902 419 a - - -
2.68618 4.42225 2.77519 2.73123 3.46354 2.40513 3.72494
3.29354 2.67741 2.69355 4.24690 2.90347 2.73739 3.18146 2.89801
2.37887 2.77519 2.98518 4.58477 3.61503
0.04848 3.44667 4.16901 0.61958 0.77255 0.48576 0.95510
351 2.46579 4.24330 3.36388 3.08039 3.19916 3.26009 3.96715
2.33307 2.87923 2.04983 3.39932 3.35019 3.86555 3.35325 3.12046
2.73806 2.86146 2.19134 4.90172 3.49001 420 l - - -
2.68618 4.42225 2.77519 2.73123 3.46354 2.40513 3.72494
3.29354 2.67741 2.69355 4.24690 2.90347 2.73739 3.18146 2.89801
2.37887 2.77519 2.98518 4.58477 3.61503
0.04848 3.44667 4.16901 0.61958 0.77255 0.48576 0.95510
352 2.56482 4.26437 3.49584 3.17708 3.11826 3.36047 4.00506
2.16904 2.95478 1.98288 3.31846 3.44338 3.93502 3.41704 3.19217
2.83307 2.93308 2.10252 4.85326 3.43792 421 l - - -
2.68618 4.42225 2.77519 2.73123 3.46354 2.40513 3.72494
3.29354 2.67741 2.69355 4.24690 2.90347 2.73739 3.18146 2.89801
2.37887 2.77519 2.98518 4.58477 3.61503

0.04848 3.44667 4.16901 0.61958 0.77255 0.48576 0.95510
353 2.51539 4.45314 2.70993 2.50045 3.51941 3.10431 3.43026
3.21740 2.32597 2.81771 3.83420 2.91452 3.64978 2.83999 2.62806
2.61414 2.81608 2.94360 4.94492 3.53225 422 k - - -
2.68618 4.42225 2.77519 2.73123 3.46354 2.40513 3.72494
3.29354 2.67741 2.69355 4.24690 2.90347 2.73739 3.18146 2.89801
2.37887 2.77519 2.98518 4.58477 3.61503
0.04848 3.44667 4.16901 0.61958 0.77255 0.48576 0.95510
354 2.38952 4.20316 2.74807 2.61836 3.46324 2.87214 3.71856
3.12161 2.65048 2.87046 3.93540 2.44245 3.53997 3.09647 2.91039
2.53297 2.80322 2.81914 4.83809 3.50411 423 a - - -
2.68618 4.42225 2.77519 2.73123 3.46354 2.40513 3.72494
3.29354 2.67741 2.69355 4.24690 2.90347 2.73739 3.18146 2.89801
2.37887 2.77519 2.98518 4.58477 3.61503
0.04848 3.44667 4.16901 0.61958 0.77255 0.48576 0.95510
355 2.31391 4.00183 2.86219 2.74722 3.62984 2.09506 3.79332
3.16450 2.80577 2.88453 3.96034 3.04858 3.25960 3.21223 3.01702
2.49308 2.75869 2.83228 4.79820 3.74388 424 g - - -
2.68618 4.42225 2.77519 2.73123 3.46354 2.40513 3.72494
3.29354 2.67741 2.69355 4.24690 2.90347 2.73739 3.18146 2.89801
2.37887 2.77519 2.98518 4.58477 3.61503
0.04848 3.44667 4.16901 0.61958 0.77255 0.48576 0.95510
356 2.48546 4.45211 2.44557 2.36785 3.60629 2.99493 3.46151
3.30763 2.48376 2.98044 3.93587 2.78031 3.63740 2.90988 2.76651
2.56823 2.83487 2.98931 4.99716 3.58940 425 e - - -
2.68618 4.42225 2.77519 2.73123 3.46354 2.40513 3.72494
3.29354 2.67741 2.69355 4.24690 2.90347 2.73739 3.18146 2.89801
2.37887 2.77519 2.98518 4.58477 3.61503
0.04848 3.44667 4.16901 0.61958 0.77255 0.48576 0.95510
357 2.66128 4.26791 3.45278 3.18121 2.40691 3.30959 3.83414
2.41237 3.08976 1.98731 3.32568 3.44593 3.87061 3.45373 3.31254
2.91083 3.01587 2.32594 4.43025 2.94204 426 l - - -
2.68618 4.42225 2.77519 2.73123 3.46354 2.40513 3.72494
3.29354 2.67741 2.69355 4.24690 2.90347 2.73739 3.18146 2.89801
2.37887 2.77519 2.98518 4.58477 3.61503
0.04848 3.44667 4.16901 0.61958 0.77255 0.48576 0.95510
358 2.33279 4.22955 2.99113 2.75626 3.50678 3.04860 3.79523
2.76108 2.58675 2.58069 3.71115 3.02205 3.69016 3.10804 2.82587
2.53672 2.68765 2.54495 5.02302 3.63970 427 a - - -
2.68618 4.42225 2.77519 2.73123 3.46354 2.40513 3.72494
3.29354 2.67741 2.69355 4.24690 2.90347 2.73739 3.18146 2.89801
2.37887 2.77519 2.98518 4.58477 3.61503
0.04848 3.44667 4.16901 0.61958 0.77255 0.48576 0.95510
359 2.37089 4.31119 2.64998 2.51605 3.63088 2.96755 3.70327
3.15033 2.52503 2.92200 3.91652 2.74060 3.62409 2.95421 2.83851
2.47844 2.72082 2.83251 5.06217 3.65617 428 a - - -
2.68618 4.42225 2.77519 2.73123 3.46354 2.40513 3.72494
3.29354 2.67741 2.69355 4.24690 2.90347 2.73739 3.18146 2.89801
2.37887 2.77519 2.98518 4.58477 3.61503
0.04848 3.44667 4.16901 0.61958 0.77255 0.48576 0.95510
360 2.36455 4.23573 2.47044 2.45172 3.73609 2.59253 3.72211
3.21207 2.66138 2.99496 3.97806 2.83284 3.52288 3.03590 2.97152
2.49026 2.77021 2.87317 5.03485 3.77577 429 a - - -

```
          2.68618  4.42225  2.77519  2.73123  3.46354  2.40513  3.72494
 3.29354  2.67741  2.69355  4.24690  2.90347  2.73739  3.18146  2.89801
 2.37887  2.77519  2.98518  4.58477  3.61503
          0.04848  3.44667  4.16901  0.61958  0.77255  0.48576  0.95510
    361   2.46357  4.23564  3.28320  3.02999  3.22093  3.20374  3.94097
 2.42096  2.85131  2.06310  3.44288  3.30693  3.82216  3.32833  3.09013
 2.71215  2.81551  2.25484  4.89306  3.49115    430 l - - -
          2.68618  4.42225  2.77519  2.73123  3.46354  2.40513  3.72494
 3.29354  2.67741  2.69355  4.24690  2.90347  2.73739  3.18146  2.89801
 2.37887  2.77519  2.98518  4.58477  3.61503
          0.04848  3.44667  4.16901  0.61958  0.77255  0.48576  0.95510
    362   2.30120  4.20214  2.98126  2.76858  3.53291  3.04044  3.82457
 2.71100  2.64158  2.60973  3.61853  3.08452  3.68686  3.13432  2.93767
 2.46167  2.69963  2.48751  5.05839  3.68308    431 a - - -
          2.68618  4.42225  2.77519  2.73123  3.46354  2.40513  3.72494
 3.29354  2.67741  2.69355  4.24690  2.90347  2.73739  3.18146  2.89801
 2.37887  2.77519  2.98518  4.58477  3.61503
          0.04848  3.44667  4.16901  0.61958  0.77255  0.48576  0.95510
    363   2.32814  4.15108  2.72783  2.59260  3.53099  2.92864  3.71566
 3.06119  2.61194  2.87757  3.84770  2.83798  3.61915  3.03409  2.91613
 2.43901  2.72280  2.74406  4.97800  3.50869    432 a - - -
          2.68618  4.42225  2.77519  2.73123  3.46354  2.40513  3.72494
 3.29354  2.67741  2.69355  4.24690  2.90347  2.73739  3.18146  2.89801
 2.37887  2.77519  2.98518  4.58477  3.61503
          0.04848  3.44667  4.16901  0.61958  0.77255  0.48576  0.95510
    364   2.73105  4.24311  3.42506  3.09489  2.60188  3.19509  3.59135
 2.79739  2.82846  2.24506  3.47777  3.36273  3.72698  3.28511  3.01673
 2.93448  3.04184  2.68286  2.82800  2.66464    433 l - - -
          2.68618  4.42225  2.77519  2.73123  3.46354  2.40513  3.72494
 3.29354  2.67741  2.69355  4.24690  2.90347  2.73739  3.18146  2.89801
 2.37887  2.77519  2.98518  4.58477  3.61503
          0.04848  3.44667  4.16901  0.61958  0.77255  0.48576  0.95510
    365   2.39837  4.31417  2.81837  2.58520  3.48881  3.04728  3.60795
 3.04015  2.49741  2.79166  3.76211  2.88165  3.67204  2.96829  2.77700
 2.51305  2.71519  2.77659  4.95654  3.53095    434 a - - -
          2.68618  4.42225  2.77519  2.73123  3.46354  2.40513  3.72494
 3.29354  2.67741  2.69355  4.24690  2.90347  2.73739  3.18146  2.89801
 2.37887  2.77519  2.98518  4.58477  3.61503
          0.04848  3.44667  4.16901  0.61958  0.77255  0.48576  0.95510
    366   2.35674  4.17925  3.34375  3.07357  3.31764  3.16989  4.00281
 2.32871  2.91283  2.19807  3.45182  3.32152  3.80695  3.36455  3.15832
 2.64953  2.79473  2.14600  5.00332  3.62794    435 v - - -
          2.68618  4.42225  2.77519  2.73123  3.46354  2.40513  3.72494
 3.29354  2.67741  2.69355  4.24690  2.90347  2.73739  3.18146  2.89801
 2.37887  2.77519  2.98518  4.58477  3.61503
          0.04848  3.44667  4.16901  0.61958  0.77255  0.48576  0.95510
    367   2.36373  4.24950  2.81430  2.57778  3.66561  2.99240  3.79140
 2.86962  2.58862  2.72035  3.79673  2.99728  3.64929  3.08068  2.88566
 2.48269  2.60525  2.60740  5.12692  3.79114    436 a - - -
          2.68618  4.42225  2.77519  2.73123  3.46354  2.40513  3.72494
 3.29354  2.67741  2.69355  4.24690  2.90347  2.73739  3.18146  2.89801
 2.37887  2.77519  2.98518  4.58477  3.61503
          0.04848  3.44667  4.16901  0.61958  0.77255  0.48576  0.95510
```

```
    368   2.18474  4.10784  2.85819  2.69773  3.69476  2.68192  3.82013
3.06429  2.69987  2.87567  3.88918  2.99106  3.49563  3.13428  2.94163
2.33118  2.62894  2.72426  5.11708  3.79119    437 a - - -
          2.68618  4.42225  2.77519  2.73123  3.46354  2.40513  3.72494
3.29354  2.67741  2.69355  4.24690  2.90347  2.73739  3.18146  2.89801
2.37887  2.77519  2.98518  4.58477  3.61503
          0.04848  3.44667  4.16901  0.61958  0.77255  0.48576  0.95510
    369   2.42543  4.36423  2.35154  2.38639  3.65751  2.86728  3.59991
3.28699  2.60562  3.01746  3.98532  2.71977  3.58000  2.97034  2.94553
2.52173  2.80719  2.95239  5.02651  3.65569    438 d - - -
          2.68618  4.42225  2.77519  2.73123  3.46354  2.40513  3.72494
3.29354  2.67741  2.69355  4.24690  2.90347  2.73739  3.18146  2.89801
2.37887  2.77519  2.98518  4.58477  3.61503
          0.04848  3.44667  4.16901  0.61958  0.77255  0.48576  0.95510
    370   2.21679  4.12657  3.19594  2.98501  3.42906  3.02656  3.97776
2.52076  2.89339  2.35297  3.57145  3.22801  3.70263  3.33146  3.13856
2.54331  2.73538  2.23578  5.05023  3.70306    439 a - - -
          2.68618  4.42225  2.77519  2.73123  3.46354  2.40513  3.72494
3.29354  2.67741  2.69355  4.24690  2.90347  2.73739  3.18146  2.89801
2.37887  2.77519  2.98518  4.58477  3.61503
          0.04848  3.44667  4.16901  0.61958  0.77255  0.48576  0.95510
    371   2.51622  4.49644  2.53434  2.34652  3.69094  3.07709  3.53078
3.25523  2.36980  2.93348  3.90181  2.84045  3.67593  2.85771  2.68500
2.56559  2.85115  2.95848  5.07356  3.68474    440 e - - -
          2.68618  4.42225  2.77519  2.73123  3.46354  2.40513  3.72494
3.29354  2.67741  2.69355  4.24690  2.90347  2.73739  3.18146  2.89801
2.37887  2.77519  2.98518  4.58477  3.61503
          0.04848  3.44667  4.16901  0.61958  0.77255  0.48576  0.95510
    372   2.47869  4.26281  3.25787  2.96498  3.15778  3.21728  3.79108
2.55136  2.75467  2.18581  3.40633  3.25590  3.81578  3.20774  3.00254
2.68680  2.85482  2.29463  4.80749  3.36923    441 l - - -
          2.68618  4.42225  2.77519  2.73123  3.46354  2.40513  3.72494
3.29354  2.67741  2.69355  4.24690  2.90347  2.73739  3.18146  2.89801
2.37887  2.77519  2.98518  4.58477  3.61503
          0.04848  3.44667  4.16901  0.61958  0.77255  0.48576  0.95510
    373   2.49913  4.41371  2.76056  2.51694  3.59064  3.11217  3.68650
2.97579  2.41510  2.67165  3.75487  2.95877  3.71114  2.81581  2.69263
2.62071  2.80290  2.76816  5.04638  3.67958    442 k - - -
          2.68618  4.42225  2.77519  2.73123  3.46354  2.40513  3.72494
3.29354  2.67741  2.69355  4.24690  2.90347  2.73739  3.18146  2.89801
2.37887  2.77519  2.98518  4.58477  3.61503
          0.04848  3.44667  4.16901  0.61958  0.77255  0.48576  0.95510
    374   2.49764  4.38491  2.85667  2.59591  3.49427  3.14702  3.71620
2.83185  2.46312  2.55880  3.67781  3.01614  3.73934  2.86092  2.75521
2.63748  2.82609  2.64707  5.00038  3.61773    443 k - - -
          2.68618  4.42225  2.77519  2.73123  3.46354  2.40513  3.72494
3.29354  2.67741  2.69355  4.24690  2.90347  2.73739  3.18146  2.89801
2.37887  2.77519  2.98518  4.58477  3.61503
          0.04848  3.44667  4.16901  0.61958  0.77255  0.48576  0.95510
    375   2.54197  4.30423  3.24707  2.92981  3.14222  3.32480  3.85458
2.37592  2.76249  2.16603  3.39279  3.27678  3.87854  3.20274  3.03025
2.76151  2.88585  2.27419  4.82801  3.39467    444 l - - -
          2.68618  4.42225  2.77519  2.73123  3.46354  2.40513  3.72494
3.29354  2.67741  2.69355  4.24690  2.90347  2.73739  3.18146  2.89801
2.37887  2.77519  2.98518  4.58477  3.61503
```

0.04848 3.44667 4.16901 0.61958 0.77255 0.48576 0.95510
376 2.43117 4.40044 2.47043 2.34350 3.74091 2.95007 3.67126
3.22874 2.53260 3.00177 3.96373 2.71589 3.62098 2.92349 2.86032
2.51023 2.80578 2.90536 5.12307 3.74243 445 e - - -
2.68618 4.42225 2.77519 2.73123 3.46354 2.40513 3.72494
3.29354 2.67741 2.69355 4.24690 2.90346 2.73740 3.18146 2.89801
2.37887 2.77519 2.98518 4.58477 3.61503
0.06221 3.10455 4.16901 0.58761 0.81115 0.48576 0.95510
377 2.34797 4.28912 2.66772 2.49101 3.67791 2.76377 3.64096
3.22958 2.50275 2.95775 3.93051 2.83698 3.59552 2.95990 2.81737
2.48431 2.75807 2.89253 5.06136 3.70017 447 a - - -
2.68618 4.42225 2.77519 2.73123 3.46354 2.40513 3.72494
3.29354 2.67741 2.69355 4.24690 2.90347 2.73739 3.18146 2.89801
2.37887 2.77519 2.98518 4.58477 3.61503
0.04848 3.44667 4.16901 0.61958 0.77255 0.48576 0.95510
378 2.35231 4.29954 2.80367 2.58814 3.58231 3.04458 3.67986
2.95409 2.52082 2.74393 3.77868 2.95881 3.67158 3.01176 2.74428
2.53343 2.73242 2.69148 5.04208 3.66208 448 a - - -
2.68618 4.42225 2.77519 2.73123 3.46354 2.40513 3.72494
3.29354 2.67741 2.69355 4.24690 2.90347 2.73739 3.18146 2.89801
2.37887 2.77519 2.98518 4.58477 3.61503
0.04848 3.44667 4.16901 0.61958 0.77255 0.48576 0.95510
379 2.31778 4.22741 2.84322 2.64173 3.55081 2.95924 3.67178
3.10552 2.55571 2.79801 3.83817 2.95412 3.47726 3.04112 2.78212
2.39320 2.69730 2.79253 5.00302 3.60689 449 a - - -
2.68618 4.42225 2.77519 2.73123 3.46354 2.40513 3.72494
3.29354 2.67741 2.69355 4.24690 2.90347 2.73739 3.18146 2.89801
2.37887 2.77519 2.98518 4.58477 3.61503
0.04848 3.44667 4.16901 0.61958 0.77255 0.48576 0.95510
380 2.40529 4.09324 3.50234 3.18412 3.17932 3.21007 4.02264
2.29995 2.99410 2.12783 3.39492 3.39943 3.83519 3.42542 3.23315
2.68072 2.82572 2.06334 4.91608 3.52055 450 v - - -
2.68618 4.42225 2.77519 2.73123 3.46354 2.40513 3.72494
3.29354 2.67741 2.69355 4.24690 2.90347 2.73739 3.18146 2.89801
2.37887 2.77519 2.98518 4.58477 3.61503
0.04848 3.44667 4.16901 0.61958 0.77255 0.48576 0.95510
381 2.65378 4.27828 3.39105 3.09458 2.95360 3.25809 3.82989
2.48193 2.83328 1.85355 3.36953 3.38475 3.81856 3.32802 3.04304
2.88831 3.00062 2.38897 4.40353 3.16572 451 l - - -
2.68618 4.42225 2.77519 2.73123 3.46354 2.40513 3.72494
3.29354 2.67741 2.69355 4.24690 2.90347 2.73739 3.18146 2.89801
2.37887 2.77519 2.98518 4.58477 3.61503
0.04848 3.44667 4.16901 0.61958 0.77255 0.48576 0.95510
382 2.46023 4.27523 3.08332 2.80011 3.31446 3.15195 3.80666
2.66391 2.65671 2.37229 3.55877 3.15436 3.75873 3.11698 2.86472
2.64575 2.83392 2.35140 4.89645 3.46046 452 v - - -
2.68618 4.42225 2.77519 2.73123 3.46354 2.40513 3.72494
3.29354 2.67741 2.69355 4.24690 2.90347 2.73739 3.18146 2.89801
2.37887 2.77519 2.98518 4.58477 3.61503
0.04848 3.44667 4.16901 0.61958 0.77255 0.48576 0.95510
383 2.54174 4.26679 3.33009 3.06277 3.09586 3.26102 3.90800
2.39006 2.85692 1.99524 3.41475 3.34172 3.85793 3.33950 3.08992
2.75567 2.91920 2.26391 4.77268 3.33256 453 l - - -

```
         2.68618  4.42225  2.77519  2.73123  3.46354  2.40513  3.72494
3.29354  2.67741  2.69355  4.24690  2.90347  2.73739  3.18146  2.89801
2.37887  2.77519  2.98518  4.58477  3.61503
         0.04848  3.44667  4.16901  0.61958  0.77255  0.48576  0.95510
    384  2.22382  4.12928  2.88128  2.71241  3.59136  2.87723  3.80864
3.01789  2.69957  2.79588  3.85614  2.97990  3.37053  3.13821  2.96861
2.28688  2.67966  2.69901  5.05087  3.68321    454 a - - -
         2.68618  4.42225  2.77519  2.73123  3.46354  2.40513  3.72494
3.29354  2.67741  2.69355  4.24690  2.90347  2.73739  3.18146  2.89801
2.37887  2.77519  2.98518  4.58477  3.61503
         0.04848  3.44667  4.16901  0.61958  0.77255  0.48576  0.95510
    385  2.42278  4.38299  2.53391  2.41276  3.58860  2.95846  3.53839
3.29346  2.51856  3.00117  3.95750  2.69991  3.62268  2.95016  2.83311
2.49905  2.79929  2.95762  5.00173  3.57491    455 e - - -
         2.68618  4.42225  2.77519  2.73123  3.46354  2.40513  3.72494
3.29354  2.67741  2.69355  4.24690  2.90347  2.73739  3.18146  2.89801
2.37887  2.77519  2.98518  4.58477  3.61503
         0.04848  3.44667  4.16901  0.61958  0.77255  0.48576  0.95510
    386  2.73105  4.24311  3.42506  3.09489  2.60188  3.19509  3.59135
2.79739  2.82846  2.24506  3.47777  3.36273  3.72698  3.28511  3.01673
2.93448  3.04184  2.68286  2.82800  2.66464    456 l - - -
         2.68618  4.42225  2.77519  2.73123  3.46354  2.40513  3.72494
3.29354  2.67741  2.69355  4.24690  2.90347  2.73739  3.18146  2.89801
2.37887  2.77519  2.98518  4.58477  3.61503
         0.04848  3.44667  4.16901  0.61958  0.77255  0.48576  0.95510
    387  2.41730  4.37830  2.40860  2.35860  3.72517  2.96320  3.69180
3.17080  2.59756  2.97873  3.95620  2.78692  3.61359  2.97328  2.93954
2.45343  2.79906  2.85516  5.12316  3.74101    457 e - - -
         2.68618  4.42225  2.77519  2.73123  3.46354  2.40513  3.72494
3.29354  2.67741  2.69355  4.24690  2.90347  2.73739  3.18146  2.89801
2.37887  2.77519  2.98518  4.58477  3.61503
         0.04848  3.44667  4.16901  0.61958  0.77255  0.48576  0.95510
    388  2.12854  4.13369  2.92906  2.74582  3.58509  2.91463  3.86841
2.80749  2.76325  2.66531  3.77953  3.06328  3.60640  3.19974  3.02534
2.39904  2.71730  2.54224  5.08030  3.74424    458 a - - -
         2.68618  4.42225  2.77519  2.73123  3.46354  2.40513  3.72494
3.29354  2.67741  2.69355  4.24690  2.90347  2.73739  3.18146  2.89801
2.37887  2.77519  2.98518  4.58477  3.61503
         0.04848  3.44667  4.16901  0.61958  0.77255  0.48576  0.95510
    389  2.38618  4.31413  2.69298  2.51382  3.61529  2.92835  3.69597
3.16474  2.51472  2.88541  3.89566  2.88859  3.62560  2.83630  2.79941
2.43648  2.77772  2.85491  5.03827  3.64796    459 a - - -
         2.68618  4.42225  2.77519  2.73123  3.46354  2.40513  3.72494
3.29354  2.67741  2.69355  4.24690  2.90347  2.73739  3.18146  2.89801
2.37887  2.77519  2.98518  4.58477  3.61503
         0.04848  3.44667  4.16901  0.61958  0.77255  0.48576  0.95510
    390  2.34776  4.16411  3.32442  3.07532  3.30409  3.13407  4.00081
2.38075  2.93353  2.21087  3.46879  3.31533  3.78204  3.37799  3.17370
2.63612  2.81200  2.11399  4.97763  3.60269    460 v - - -
         2.68618  4.42225  2.77519  2.73123  3.46354  2.40513  3.72494
3.29354  2.67741  2.69355  4.24690  2.90347  2.73739  3.18146  2.89801
2.37887  2.77519  2.98518  4.58477  3.61503
         0.04848  3.44667  4.16901  0.61958  0.77255  0.48576  0.95510
```

```
    391   2.24726  4.15053  2.91649  2.74494  3.54747  2.92114  3.82607
2.89746  2.68250  2.70681  3.80831  3.03975  3.60990  3.15900  2.96847
2.29418  2.68506  2.61333  5.04470  3.66209    461 a - - -
          2.68618  4.42225  2.77519  2.73123  3.46354  2.40513  3.72494
3.29354  2.67741  2.69355  4.24690  2.90347  2.73739  3.18146  2.89801
2.37887  2.77519  2.98518  4.58477  3.61503
          0.04848  3.44667  4.16901  0.61958  0.77255  0.48576  0.95510
    392   2.54138  4.33434  2.78198  2.61539  3.41279  3.00795  3.69249
3.03730  2.52843  2.59184  3.75763  3.00667  3.62602  2.50915  2.77977
2.67310  2.90673  2.83135  4.82965  3.51417    462 q - - -
          2.68618  4.42225  2.77519  2.73123  3.46354  2.40513  3.72494
3.29354  2.67741  2.69355  4.24690  2.90347  2.73739  3.18146  2.89801
2.37887  2.77519  2.98518  4.58477  3.61503
          0.04848  3.44667  4.16901  0.61958  0.77255  0.48576  0.95510
    393   2.38539  4.30415  2.80817  2.58325  3.57706  3.02837  3.70730
3.04654  2.49567  2.79374  3.76107  2.90652  3.66247  2.92417  2.75316
2.47760  2.72743  2.76082  5.03070  3.63419    463 a - - -
          2.68618  4.42225  2.77519  2.73123  3.46354  2.40513  3.72494
3.29354  2.67741  2.69355  4.24690  2.90347  2.73739  3.18146  2.89801
2.37887  2.77519  2.98518  4.58477  3.61503
          0.04848  3.44667  4.16901  0.61958  0.77255  0.48576  0.95510
    394   2.57825  4.26771  3.54412  3.20656  3.00688  3.39846  4.00564
2.16008  3.01401  1.96974  3.28698  3.47083  3.96004  3.44255  3.25900
2.85287  2.94036  2.08272  4.80362  3.36803    464 l - - -
          2.68618  4.42225  2.77519  2.73123  3.46354  2.40513  3.72494
3.29354  2.67741  2.69355  4.24690  2.90347  2.73739  3.18146  2.89801
2.37887  2.77519  2.98518  4.58477  3.61503
          0.04848  3.44667  4.16901  0.61958  0.77255  0.48576  0.95510
    395   2.47969  4.42217  2.66858  2.44271  3.59443  3.08658  3.56795
3.13479  2.41008  2.82641  3.84248  2.86694  3.68563  2.92285  2.67911
2.58638  2.80649  2.83107  5.01299  3.61378    465 k - - -
          2.68618  4.42225  2.77519  2.73123  3.46354  2.40513  3.72494
3.29354  2.67741  2.69355  4.24690  2.90347  2.73739  3.18146  2.89801
2.37887  2.77519  2.98518  4.58477  3.61503
          0.04848  3.44667  4.16901  0.61958  0.77255  0.48576  0.95510
    396   2.50223  4.23925  3.45696  3.15049  3.16869  3.30385  4.00451
2.23368  2.94147  2.03383  3.35441  3.40888  3.89853  3.40222  3.18092
2.77554  2.89684  2.09772  4.89308  3.48483    466 l - - -
          2.68618  4.42225  2.77519  2.73122  3.46350  2.40513  3.72495
3.29354  2.67741  2.69355  4.24688  2.90346  2.73740  3.18147  2.89801
2.37887  2.77520  2.98519  4.58477  3.61503
          0.06268  3.09475  4.16901  0.66187  0.72543  0.48576  0.95510
    397   2.46984  4.30064  2.70870  2.50584  3.56698  3.09739  3.65695
3.06292  2.42506  2.78550  3.79111  2.91141  3.68854  2.81320  2.74704
2.58693  2.81291  2.79354  4.99583  3.57159    470 k - - -
          2.68618  4.42225  2.77519  2.73123  3.46354  2.40513  3.72494
3.29354  2.67741  2.69355  4.24690  2.90347  2.73739  3.18146  2.89801
2.37887  2.77519  2.98518  4.58477  3.61503
          0.04848  3.44667  4.16901  0.61958  0.77255  0.48576  0.95510
    398   2.42673  4.35917  2.68311  2.46178  3.64145  2.98440  3.57629
3.19532  2.44118  2.85752  3.86823  2.87816  3.41402  2.92466  2.75317
2.54041  2.79371  2.89007  5.03595  3.66847    471 a - - -
          2.68618  4.42225  2.77519  2.73123  3.46354  2.40513  3.72494
3.29354  2.67741  2.69355  4.24690  2.90347  2.73739  3.18146  2.89801
2.37887  2.77519  2.98518  4.58477  3.61503
```

0.04848 3.44667 4.16901 0.61958 0.77255 0.48576 0.95510
399 2.44995 4.41814 2.38834 2.37568 3.65175 2.95284 3.59985
3.28745 2.54486 2.99019 3.95642 2.75262 3.61676 2.90165 2.85283
2.53843 2.81727 2.96344 5.04469 3.64516 472 e - - -
2.68618 4.42225 2.77519 2.73123 3.46354 2.40513 3.72494
3.29354 2.67741 2.69355 4.24690 2.90347 2.73739 3.18146 2.89801
2.37887 2.77519 2.98518 4.58477 3.61503
0.04848 3.44667 4.16901 0.61958 0.77255 0.48576 0.95510
400 2.46176 4.17293 2.95013 2.64357 3.43145 3.12630 3.47319
3.09019 2.33843 2.71841 3.73189 3.01225 3.70002 2.87864 2.61488
2.59369 2.80089 2.82501 4.87085 3.47490 473 k - - -
2.68618 4.42225 2.77519 2.73123 3.46354 2.40513 3.72494
3.29354 2.67741 2.69355 4.24690 2.90347 2.73739 3.18146 2.89801
2.37887 2.77519 2.98518 4.58477 3.61503
0.04848 3.44667 4.16901 0.61958 0.77255 0.48576 0.95510
401 2.35340 4.24988 2.75267 2.52706 3.70718 2.72543 3.71895
3.12326 2.50132 2.87271 3.88517 2.94852 3.58124 3.01454 2.69545
2.51500 2.77113 2.81568 5.04533 3.77068 474 a - - -
2.68618 4.42225 2.77519 2.73123 3.46354 2.40513 3.72494
3.29354 2.67741 2.69355 4.24690 2.90347 2.73739 3.18146 2.89801
2.37887 2.77519 2.98518 4.58477 3.61503
0.04848 3.44667 4.16901 0.61958 0.77255 0.48576 0.95510
402 2.65886 4.36127 3.19400 2.92584 2.76602 3.30456 3.66690
2.63754 2.77465 2.25803 3.49466 3.25039 3.85037 3.22634 3.02153
2.84003 2.98876 2.53569 4.38820 2.52933 475 l - - -
2.68618 4.42225 2.77519 2.73123 3.46354 2.40513 3.72494
3.29354 2.67741 2.69355 4.24690 2.90347 2.73739 3.18146 2.89801
2.37887 2.77519 2.98518 4.58477 3.61503
0.04848 3.44667 4.16901 0.61958 0.77255 0.48576 0.95510
403 2.45265 4.26542 3.15184 2.87102 3.28728 3.18005 3.82035
2.55621 2.65387 2.34846 3.54768 3.15488 3.78342 3.11395 2.93140
2.64753 2.82663 2.34942 4.88879 3.46729 476 l - - -
2.68618 4.42225 2.77519 2.73123 3.46354 2.40513 3.72494
3.29354 2.67741 2.69355 4.24690 2.90347 2.73739 3.18146 2.89801
2.37887 2.77519 2.98518 4.58477 3.61503
0.04848 3.44667 4.16901 0.61958 0.77255 0.48576 0.95510
404 2.56564 4.23974 3.28395 3.04870 3.12257 3.18436 3.90421
2.44150 2.86875 1.92376 3.43071 3.33760 3.78791 3.34810 3.08731
2.80095 2.89955 2.32015 4.74448 3.37281 477 l - - -
2.68618 4.42225 2.77519 2.73123 3.46354 2.40513 3.72494
3.29354 2.67741 2.69355 4.24690 2.90347 2.73739 3.18146 2.89801
2.37887 2.77519 2.98518 4.58477 3.61503
0.04848 3.44667 4.16901 0.61958 0.77255 0.48576 0.95510
405 2.44332 4.31032 2.97454 2.71605 3.40382 3.08016 3.64070
2.97545 2.45442 2.57959 3.71496 3.06013 3.68678 3.02323 2.48488
2.61295 2.83125 2.72164 4.87184 3.49448 478 a - - -
2.68618 4.42225 2.77519 2.73123 3.46354 2.40513 3.72494
3.29354 2.67741 2.69355 4.24690 2.90347 2.73739 3.18146 2.89801
2.37887 2.77519 2.98518 4.58477 3.61503
0.04848 3.44667 4.16901 0.61958 0.77255 0.48576 0.95510
406 2.42933 4.06178 3.53537 3.21062 3.15357 3.21306 4.02534
2.29398 3.01359 2.11443 3.38594 3.41782 3.83638 3.44167 3.24840
2.69118 2.83182 2.03968 4.88990 3.49375 479 v - - -

2.68618 4.42225 2.77519 2.73123 3.46354 2.40513 3.72494
3.29354 2.67741 2.69355 4.24690 2.90347 2.73739 3.18146 2.89801
2.37887 2.77519 2.98518 4.58477 3.61503
0.04848 3.44667 4.16901 0.61958 0.77255 0.48576 0.95510
407 2.44219 4.27692 3.05128 2.79374 3.27285 3.13846 3.66220
2.68616 2.64024 2.43798 3.59391 3.12340 3.74693 3.12816 2.90395
2.61729 2.66450 2.54744 4.84671 3.36844 480 l - - -
2.68618 4.42225 2.77519 2.73123 3.46354 2.40513 3.72494
3.29354 2.67741 2.69355 4.24690 2.90347 2.73739 3.18146 2.89801
2.37887 2.77519 2.98518 4.58477 3.61503
0.04848 3.44667 4.16901 0.61958 0.77255 0.48576 0.95510
408 2.13705 4.14461 3.12173 2.88289 3.35399 2.94646 3.82678
2.74737 2.74078 2.47817 3.63877 3.16458 3.63437 3.21041 2.97933
2.56685 2.77813 2.53062 4.58414 3.52358 481 a - - -
2.68618 4.42225 2.77519 2.73123 3.46354 2.40513 3.72494
3.29354 2.67741 2.69355 4.24690 2.90347 2.73739 3.18146 2.89801
2.37887 2.77519 2.98518 4.58477 3.61503
0.04848 3.44667 4.16901 0.61958 0.77255 0.48576 0.95510
409 2.55478 4.46841 2.75054 2.50879 3.59600 3.13854 3.63287
3.09497 2.24267 2.75183 3.79973 2.94294 3.71437 2.85211 2.55523
2.65991 2.87990 2.85105 5.00037 3.58938 482 k - - -
2.68618 4.42225 2.77519 2.73123 3.46354 2.40513 3.72494
3.29354 2.67741 2.69355 4.24690 2.90347 2.73739 3.18146 2.89801
2.37887 2.77519 2.98518 4.58477 3.61503
0.04848 3.44667 4.16901 0.61958 0.77255 0.48576 0.95510
410 2.46236 4.28986 2.76318 2.57979 3.66528 2.82512 3.68724
3.11420 2.23159 2.84529 3.88542 2.96488 3.58575 2.99217 2.67582
2.59429 2.83527 2.83146 4.96641 3.72126 483 k - - -
2.68618 4.42225 2.77519 2.73123 3.46354 2.40513 3.72494
3.29354 2.67741 2.69355 4.24690 2.90347 2.73739 3.18146 2.89801
2.37887 2.77519 2.98518 4.58477 3.61503
0.04848 3.44667 4.16901 0.61958 0.77255 0.48576 0.95510
411 2.49962 4.41397 2.70314 2.39041 3.61957 3.09889 3.70116
2.92382 2.46835 2.71368 3.78703 2.93990 3.70607 2.94837 2.77506
2.60051 2.85174 2.68697 5.07360 3.70296 484 e - - -
2.68618 4.42225 2.77519 2.73123 3.46354 2.40513 3.72494
3.29354 2.67741 2.69355 4.24690 2.90347 2.73739 3.18146 2.89801
2.37887 2.77519 2.98518 4.58477 3.61503
0.06268 3.44667 3.54383 0.61958 0.77255 0.48576 0.95510
412 2.35112 4.01776 2.70766 2.69195 3.56640 2.25910 3.75321
3.10531 2.77641 2.85489 3.94209 3.02033 3.37143 3.17929 2.99335
2.52583 2.78343 2.79348 4.74347 3.66819 485 g - - -
2.68618 4.42225 2.77519 2.73123 3.46354 2.40513 3.72494
3.29354 2.67741 2.69355 4.24690 2.90347 2.73739 3.18146 2.89801
2.37887 2.77519 2.98518 4.58477 3.61503
0.04915 3.43314 4.15549 0.61958 0.77255 0.49375 0.94247
413 2.44313 4.23121 3.23842 2.95776 3.22315 3.15322 3.87014
2.46252 2.77004 2.26941 3.43729 3.24404 3.75061 3.24955 3.02112
2.65671 2.80961 2.31310 4.86403 3.39665 486 l - - -
2.68618 4.42225 2.77519 2.73123 3.46354 2.40513 3.72494
3.29354 2.67741 2.69355 4.24690 2.90347 2.73739 3.18146 2.89801
2.37887 2.77519 2.98518 4.58477 3.61503
0.04915 3.43314 4.15549 0.61958 0.77255 0.49375 0.94247

414 2.35463 3.96069 2.87393 2.77067 3.52767 2.11625 3.76504
3.08925 2.81505 2.80425 3.92806 3.07199 3.32651 3.22956 2.99883
2.54447 2.79458 2.78752 4.66813 3.65179 487 g - - -
2.68618 4.42225 2.77519 2.73123 3.46354 2.40513 3.72494
3.29354 2.67741 2.69355 4.24690 2.90347 2.73739 3.18146 2.89801
2.37887 2.77519 2.98518 4.58477 3.61503
0.04915 3.43314 4.15549 0.61958 0.77255 0.49375 0.94247
415 2.39295 4.30297 2.65902 2.49285 3.59715 2.96756 3.69883
3.06290 2.51623 2.85673 3.88776 2.81528 3.61398 3.00767 2.78030
2.54038 2.79932 2.77338 5.00166 3.64003 488 a - - -
2.68618 4.42225 2.77519 2.73123 3.46354 2.40513 3.72494
3.29354 2.67741 2.69355 4.24690 2.90347 2.73739 3.18146 2.89801
2.37887 2.77519 2.98518 4.58477 3.61503
0.04915 3.43314 4.15549 0.61958 0.77255 0.48080 0.96308
416 2.32886 4.03015 2.85982 2.73451 3.60742 2.14300 3.77926
3.15724 2.76362 2.87066 3.94521 3.04317 3.40154 3.18832 2.89431
2.50378 2.76661 2.83219 4.80343 3.71236 489 g - - -
2.68618 4.42225 2.77519 2.73123 3.46354 2.40513 3.72494
3.29354 2.67741 2.69355 4.24690 2.90347 2.73739 3.18146 2.89801
2.37887 2.77519 2.98518 4.58477 3.61503
0.04848 3.44667 4.16901 0.61958 0.77255 0.48576 0.95510
417 2.43132 4.18070 3.06453 2.78324 3.16099 3.13581 3.70366
2.80833 2.63288 2.58115 3.66331 3.01641 3.73538 3.10598 2.90293
2.58328 2.75415 2.57612 4.71191 3.04583 490 a - - -
2.68618 4.42225 2.77519 2.73123 3.46354 2.40513 3.72494
3.29354 2.67741 2.69355 4.24690 2.90347 2.73739 3.18146 2.89801
2.37887 2.77519 2.98518 4.58477 3.61503
0.04848 3.44667 4.16901 0.61958 0.77255 0.48576 0.95510
418 2.43487 4.18811 3.37777 3.10021 3.22936 3.19314 3.98156
2.32278 2.92553 2.18360 3.44282 3.34362 3.82149 3.37758 3.16600
2.65644 2.83639 2.09722 4.91252 3.50550 491 v - - -
2.68618 4.42225 2.77519 2.73123 3.46354 2.40513 3.72494
3.29354 2.67741 2.69355 4.24690 2.90347 2.73739 3.18146 2.89801
2.37887 2.77519 2.98518 4.58477 3.61503
0.05217 3.44667 3.96452 0.61958 0.77255 0.48576 0.95510
419 2.31953 4.17832 2.99431 2.79356 3.48988 2.99348 3.84199
2.73478 2.70074 2.58002 3.70950 3.05921 3.66245 3.13360 2.96049
2.48554 2.57054 2.52142 5.03016 3.64653 492 a - - -
2.68618 4.42225 2.77519 2.73123 3.46354 2.40513 3.72494
3.29354 2.67741 2.69355 4.24690 2.90347 2.73739 3.18146 2.89801
2.37887 2.77519 2.98518 4.58477 3.61503
0.04865 3.44315 4.16550 0.61958 0.77255 0.48786 0.95177
420 2.61922 4.29876 3.53263 3.19178 2.99887 3.43257 3.98019
2.15227 2.96542 1.93331 3.24682 3.47008 3.97759 3.41887 3.20587
2.89169 2.96959 2.14554 4.77355 3.33278 493 l - - -
2.68618 4.42225 2.77519 2.73123 3.46354 2.40513 3.72494
3.29354 2.67741 2.69355 4.24690 2.90347 2.73739 3.18146 2.89801
2.37887 2.77519 2.98518 4.58477 3.61503
0.04865 3.44315 4.16550 0.61958 0.77255 0.48446 0.95719
421 2.46153 4.34245 2.94073 2.64930 3.37858 3.11446 3.63494
2.93778 2.47363 2.60291 3.61739 3.03295 3.72954 2.97659 2.71053
2.57077 2.79387 2.69994 4.87769 3.41190 494 a - - -
2.68618 4.42225 2.77519 2.73123 3.46354 2.40513 3.72494
3.29354 2.67741 2.69355 4.24690 2.90347 2.73739 3.18146 2.89801
2.37887 2.77519 2.98518 4.58477 3.61503

0.04848 3.44667 4.16901 0.61958 0.77255 0.48576 0.95510
422 2.49838 4.45892 2.32637 2.33401 3.62797 3.00049 3.51680
3.27856 2.54090 2.96863 3.94898 2.77913 3.62313 2.92547 2.85291
2.57717 2.85090 2.97186 5.01360 3.62102 495 d - - -
2.68618 4.42225 2.77519 2.73123 3.46354 2.40513 3.72494
3.29354 2.67741 2.69355 4.24690 2.90347 2.73739 3.18146 2.89801
2.37887 2.77519 2.98518 4.58477 3.61503
0.04848 3.44667 4.16901 0.61958 0.77255 0.48576 0.95510
423 2.20074 3.55168 3.24852 2.94876 3.51669 2.84670 3.89445
2.68612 2.82143 2.64246 3.68038 3.17978 3.60216 3.24749 3.07388
2.39334 2.63107 2.42838 5.00107 3.68866 496 a - - -
2.68618 4.42225 2.77519 2.73123 3.46354 2.40513 3.72494
3.29354 2.67741 2.69355 4.24690 2.90347 2.73739 3.18146 2.89801
2.37887 2.77519 2.98518 4.58477 3.61503
0.04848 3.44667 4.16901 0.61958 0.77255 0.48576 0.95510
424 2.36966 4.30232 2.78107 2.49869 3.63372 3.01194 3.76259
2.86223 2.57788 2.68818 3.78705 2.95931 3.67547 3.05009 2.86420
2.55985 2.77047 2.59147 5.09938 3.74353 497 a - - -
2.68618 4.42225 2.77519 2.73123 3.46354 2.40513 3.72494
3.29354 2.67741 2.69355 4.24690 2.90347 2.73739 3.18146 2.89801
2.37887 2.77519 2.98518 4.58477 3.61503
0.04848 3.44667 4.16901 0.61958 0.77255 0.48576 0.95510
425 2.46966 4.37967 2.72515 2.47292 3.46887 3.02105 3.56981
3.16908 2.44592 2.86321 3.82861 2.78783 3.66555 2.93215 2.73893
2.58178 2.81113 2.87378 4.69608 3.47547 498 k - - -
2.68618 4.42225 2.77519 2.73123 3.46354 2.40513 3.72494
3.29354 2.67741 2.69355 4.24690 2.90347 2.73739 3.18146 2.89801
2.37887 2.77519 2.98518 4.58477 3.61503
0.05230 3.44667 3.95789 0.61958 0.77255 0.48576 0.95510
426 2.45278 4.37726 2.70484 2.52531 3.39580 3.03115 3.44534
3.24824 2.45875 2.88828 3.89349 2.77077 3.65596 2.93562 2.71298
2.56096 2.79746 2.94279 4.85011 3.38890 499 a - - -
2.68618 4.42225 2.77519 2.73123 3.46354 2.40513 3.72494
3.29354 2.67741 2.69355 4.24690 2.90347 2.73739 3.18146 2.89801
2.37887 2.77519 2.98518 4.58477 3.61503
0.04866 3.44303 4.16537 0.61958 0.77255 0.48793 0.95165
427 2.41023 4.28854 2.94628 2.69652 3.50553 3.10364 3.78262
2.69835 2.54089 2.54238 3.66640 3.06627 3.72354 3.03785 2.84367
2.57616 2.74435 2.53866 5.03356 3.66007 500 a - - -
2.68618 4.42225 2.77520 2.73123 3.46354 2.40513 3.72494
3.29353 2.67741 2.69354 4.24690 2.90347 2.73740 3.18146 2.89800
2.37887 2.77520 2.98518 4.58477 3.61501
0.05904 3.17480 4.16537 0.62357 0.76793 0.48441 0.95727
428 2.48651 4.41459 2.43288 2.22181 3.74349 2.99006 3.68631
3.08007 2.56294 2.87416 3.91234 2.85287 3.61635 2.96811 2.88708
2.61671 2.87375 2.81214 5.08318 3.79473 503 e - - -
2.68618 4.42225 2.77519 2.73123 3.46354 2.40513 3.72494
3.29354 2.67741 2.69355 4.24690 2.90347 2.73739 3.18146 2.89801
2.37887 2.77519 2.98518 4.58477 3.61503
0.04848 3.44667 4.16901 0.61958 0.77255 0.48576 0.95510
429 2.34915 4.23902 2.87332 2.66699 3.62413 3.00476 3.80880
2.82937 2.57592 2.63385 3.74545 3.03161 3.65967 3.07435 2.88660
2.53209 2.58360 2.58498 5.10917 3.78100 504 a - - -

```
          2.68618  4.42225  2.77519  2.73123  3.46354  2.40513  3.72494
3.29354  2.67741  2.69355  4.24690  2.90347  2.73739  3.18146  2.89801
2.37887  2.77519  2.98518  4.58477  3.61503
          0.04848  3.44667  4.16901  0.61958  0.77255  0.48576  0.95510
    430   2.57712  4.33522  3.28225  2.96249  3.01141  3.31717  3.67322
2.52123  2.70243  2.09324  3.38445  3.27341  3.87189  3.21119  2.94664
2.77843  2.92137  2.43465  4.66157  3.17915    505 l - - -
          2.68618  4.42225  2.77519  2.73123  3.46354  2.40513  3.72494
3.29354  2.67741  2.69355  4.24690  2.90347  2.73739  3.18146  2.89801
2.37887  2.77519  2.98518  4.58477  3.61503
          0.04848  3.44667  4.16901  0.61958  0.77255  0.48576  0.95510
    431   2.36563  4.24207  2.85878  2.68890  3.56806  3.02026  3.79368
2.81425  2.60459  2.63334  3.73188  3.03310  3.66759  3.06040  2.87736
2.52904  2.59184  2.59092  5.06570  3.70818    506 a - - -
          2.68618  4.42225  2.77519  2.73123  3.46354  2.40513  3.72494
3.29354  2.67741  2.69355  4.24690  2.90347  2.73739  3.18146  2.89801
2.37887  2.77519  2.98518  4.58477  3.61503
          0.04848  3.44667  4.16901  0.61958  0.77255  0.48576  0.95510
    432   2.62265  4.29581  3.28352  3.01022  2.41670  3.26632  3.54807
2.64608  2.91983  2.18897  3.44236  3.30249  3.82570  3.30547  3.16440
2.82088  2.96862  2.52692  4.33820  2.80439    507 l - - -
          2.68618  4.42225  2.77519  2.73123  3.46354  2.40513  3.72494
3.29354  2.67741  2.69355  4.24690  2.90347  2.73739  3.18146  2.89801
2.37887  2.77519  2.98518  4.58477  3.61503
          0.04848  3.44667  4.16901  0.61958  0.77255  0.48576  0.95510
    433   2.31587  4.23324  2.78786  2.61428  3.52340  2.94387  3.61387
3.13054  2.58218  2.87854  3.88229  2.87154  3.61242  3.04871  2.86036
2.39948  2.63042  2.80614  4.98509  3.55947    508 a - - -
          2.68618  4.42225  2.77519  2.73123  3.46354  2.40513  3.72494
3.29354  2.67741  2.69355  4.24690  2.90347  2.73739  3.18146  2.89801
2.37887  2.77519  2.98518  4.58477  3.61503
          0.06268  3.44667  3.54383  0.61958  0.77255  0.48576  0.95510
    434   2.19653  4.11904  2.90048  2.74855  3.52703  2.87917  3.82811
2.90111  2.73636  2.72893  3.82139  2.99970  3.49651  3.17802  2.99382
2.36323  2.70130  2.61367  5.00827  3.64179    509 a - - -
          2.68618  4.42225  2.77519  2.73123  3.46354  2.40513  3.72494
3.29354  2.67741  2.69355  4.24690  2.90347  2.73739  3.18146  2.89801
2.37887  2.77519  2.98518  4.58477  3.61503
          0.04915  3.43314  4.15549  0.61958  0.77255  0.49375  0.94247
    435   2.25350  3.38313  3.35043  3.05607  3.29691  2.91581  3.74391
2.70235  2.85524  2.48883  3.62043  3.23530  3.58607  3.29987  3.07244
2.44952  2.68631  2.45899  4.79850  3.46673    510 a - - -
          2.68618  4.42225  2.77519  2.73123  3.46354  2.40513  3.72494
3.29354  2.67741  2.69355  4.24690  2.90347  2.73739  3.18146  2.89801
2.37887  2.77519  2.98518  4.58477  3.61503
          0.04915  3.43314  4.15549  0.61958  0.77255  0.49375  0.94247
    436   2.44087  4.32420  2.53314  2.43729  3.59445  2.97485  3.70275
3.01514  2.59002  2.82105  3.86772  2.87333  3.61431  3.01274  2.86391
2.56485  2.81926  2.71439  5.00007  3.65123    511 e - - -
          2.68618  4.42225  2.77519  2.73123  3.46354  2.40513  3.72494
3.29354  2.67741  2.69355  4.24690  2.90347  2.73739  3.18146  2.89801
2.37887  2.77519  2.98518  4.58477  3.61503
          0.05476  3.43314  3.86316  0.61958  0.77255  0.49375  0.94247
```

```
    437   2.40179  4.08051  2.96453  2.71704  3.32671  3.04827  3.76144
 2.74825  2.66166  2.56339  3.67862  3.09273  3.67559  3.13005  2.92560
 2.54032  2.78809  2.51987  4.84085  3.34374    512 a - - -
          2.68618  4.42225  2.77519  2.73123  3.46354  2.40513  3.72494
 3.29354  2.67741  2.69355  4.24690  2.90347  2.73739  3.18146  2.89801
 2.37887  2.77519  2.98518  4.58477  3.61503
          0.04942  3.42780  4.15015  0.61958  0.77255  0.49685  0.93763
    438   2.40518  4.26495  2.62428  2.53421  3.53461  2.92230  3.71994
 2.93245  2.58915  2.80049  3.85604  2.89768  3.60257  3.05048  2.90311
 2.54493  2.79720  2.69013  4.95381  3.59707    513 a - - -
          2.68618  4.42225  2.77519  2.73123  3.46354  2.40513  3.72494
 3.29354  2.67741  2.69355  4.24690  2.90347  2.73739  3.18146  2.89801
 2.37887  2.77519  2.98518  4.58477  3.61503
          0.04942  3.42780  4.15015  0.61958  0.77255  0.49685  0.93763
    439   2.40598  4.23086  3.02260  2.79749  3.27882  3.10017  3.81925
 2.60581  2.71904  2.45081  3.55320  3.13564  3.73255  3.18312  2.98474
 2.56062  2.77881  2.42159  4.89600  3.46379    514 a - - -
          2.68618  4.42223  2.77519  2.73122  3.46355  2.40513  3.72495
 3.29355  2.67742  2.69357  4.24692  2.90343  2.73740  3.18148  2.89800
 2.37887  2.77518  2.98518  4.58479  3.61495
          0.07250  2.91566  4.15015  1.07610  0.41692  0.49685  0.93763
    440   2.41380  4.23956  3.01765  2.80969  3.22617  3.08345  3.78297
 2.66894  2.70239  2.48033  3.64861  3.09282  3.72214  3.17716  2.95937
 2.56062  2.76437  2.48905  4.80966  3.26835    582 a - - -
          2.68618  4.42225  2.77519  2.73123  3.46354  2.40513  3.72494
 3.29354  2.67741  2.69355  4.24690  2.90347  2.73739  3.18146  2.89801
 2.37887  2.77519  2.98518  4.58477  3.61503
          0.04942  3.42780  4.15015  0.61958  0.77255  0.49685  0.93763
    441   2.32473  4.19606  2.84833  2.68995  3.41861  2.93432  3.75750
 2.94428  2.65163  2.70033  3.80533  2.88570  3.54107  3.06610  2.91080
 2.50029  2.76003  2.64921  4.90240  3.50287    583 a - - -
          2.68618  4.42225  2.77519  2.73123  3.46354  2.40513  3.72494
 3.29354  2.67741  2.69355  4.24690  2.90347  2.73739  3.18146  2.89801
 2.37887  2.77519  2.98518  4.58477  3.61503
          0.04942  3.42780  4.15015  0.61958  0.77255  0.49685  0.93763
    442   2.42244  4.25403  2.89177  2.68192  3.31318  3.06106  3.77378
 2.69851  2.67191  2.53940  3.69504  3.02647  3.69523  3.13288  2.94327
 2.59378  2.78490  2.50128  4.88756  3.46782    584 a - - -
          2.68618  4.42225  2.77519  2.73123  3.46354  2.40513  3.72494
 3.29354  2.67741  2.69355  4.24690  2.90347  2.73739  3.18146  2.89801
 2.37887  2.77519  2.98518  4.58477  3.61503
          0.04942  3.42780  4.15015  0.61958  0.77255  0.48391  0.95808
    443   2.36092  4.21627  2.96873  2.77127  3.36021  3.05262  3.80974
 2.72070  2.70128  2.51283  3.66594  3.09059  3.64590  3.12453  2.97663
 2.50231  2.69523  2.50892  4.95632  3.54165    585 a - - -
          2.68618  4.42225  2.77519  2.73123  3.46354  2.40513  3.72494
 3.29354  2.67741  2.69355  4.24690  2.90347  2.73739  3.18146  2.89801
 2.37887  2.77519  2.98518  4.58477  3.61503
          0.04874  3.44140  4.16375  0.61958  0.77255  0.48381  0.95823
    444   2.29466  4.20958  2.88111  2.65441  3.51470  2.98728  3.79029
 2.86928  2.65228  2.74153  3.79017  2.93705  3.64902  3.10397  2.93447
 2.44961  2.67103  2.57526  5.05398  3.65811    586 a - - -
          2.68618  4.42225  2.77519  2.73123  3.46354  2.40513  3.72494
 3.29354  2.67741  2.69355  4.24690  2.90347  2.73739  3.18146  2.89801
 2.37887  2.77519  2.98518  4.58477  3.61503
```

0.04848 3.44667 4.16901 0.61958 0.77255 0.48576 0.95510
445 2.25274 4.20535 2.68677 2.53984 3.73808 2.77948 3.76467
3.11856 2.63722 2.96018 3.93262 2.86523 3.57582 3.05762 2.93481
2.38268 2.67925 2.77512 5.14722 3.79028 587 a - - -
2.68618 4.42225 2.77519 2.73123 3.46354 2.40513 3.72494
3.29354 2.67741 2.69355 4.24690 2.90347 2.73739 3.18146 2.89801
2.37887 2.77519 2.98518 4.58477 3.61503
0.04848 3.44667 4.16901 0.61958 0.77255 0.48576 0.95510
446 2.70686 4.37046 3.35156 3.04096 2.51696 3.41982 3.67623
2.55800 2.91849 2.12197 3.38874 3.34561 3.93134 3.30955 3.17230
2.90932 3.02255 2.45816 4.29192 2.54758 588 l - - -
2.68618 4.42225 2.77519 2.73123 3.46354 2.40513 3.72494
3.29354 2.67741 2.69355 4.24690 2.90347 2.73739 3.18146 2.89801
2.37887 2.77519 2.98518 4.58477 3.61503
0.04848 3.44667 4.16901 0.61958 0.77255 0.48576 0.95510
447 2.45956 4.22334 3.27364 3.01748 3.27821 3.22807 3.94750
2.30062 2.85730 2.21006 3.45611 3.30226 3.83500 3.31313 3.11435
2.69535 2.79688 2.17225 4.94501 3.54683 589 v - - -
2.68618 4.42225 2.77519 2.73123 3.46354 2.40513 3.72494
3.29354 2.67741 2.69355 4.24690 2.90347 2.73739 3.18146 2.89801
2.37887 2.77519 2.98518 4.58477 3.61503
0.05510 3.26647 4.16901 0.60367 0.79142 0.48576 0.95510
448 2.35281 4.18869 3.07019 2.84916 3.41527 3.04918 3.86446
2.63130 2.74400 2.47962 3.63950 3.14139 3.70421 3.20854 3.00377
2.51150 2.57826 2.44016 4.99212 3.54038 591 a - - -
2.68618 4.42225 2.77519 2.73123 3.46354 2.40513 3.72494
3.29354 2.67741 2.69355 4.24690 2.90347 2.73739 3.18146 2.89801
2.37887 2.77519 2.98518 4.58477 3.61503
0.04848 3.44667 4.16901 0.61958 0.77255 0.48576 0.95510
449 2.54021 4.41524 2.73279 2.54111 3.59594 3.06804 3.65922
3.08936 2.22003 2.74007 3.82276 2.95064 3.66945 2.81673 2.65264
2.65316 2.88590 2.84790 4.98111 3.65564 592 k - - -
2.68618 4.42225 2.77519 2.73123 3.46354 2.40513 3.72494
3.29354 2.67741 2.69355 4.24690 2.90347 2.73739 3.18146 2.89801
2.37887 2.77519 2.98518 4.58477 3.61503
0.04848 3.44667 4.16901 0.61958 0.77255 0.48576 0.95510
450 2.40086 4.30516 2.74551 2.48107 3.68351 3.01019 3.74409
2.95697 2.54334 2.77953 3.82924 2.92880 3.65330 3.02473 2.80291
2.52260 2.66462 2.68655 5.11650 3.77289 593 a - - -
2.68618 4.42225 2.77519 2.73123 3.46354 2.40513 3.72494
3.29354 2.67741 2.69355 4.24690 2.90347 2.73739 3.18146 2.89801
2.37887 2.77519 2.98518 4.58477 3.61503
0.04848 3.44667 4.16901 0.61958 0.77255 0.48576 0.95510
451 2.51751 4.29707 3.24050 2.94152 3.21063 3.26377 3.85536
2.45527 2.69464 2.16523 3.44449 3.25552 3.84436 3.15578 2.96695
2.72753 2.87896 2.31598 4.86135 3.44202 594 l - - -
2.68618 4.42225 2.77519 2.73123 3.46354 2.40513 3.72494
3.29354 2.67741 2.69355 4.24690 2.90347 2.73739 3.18146 2.89801
2.37887 2.77519 2.98518 4.58477 3.61503
0.04848 3.44667 4.16901 0.61958 0.77255 0.48576 0.95510
452 2.43687 4.35916 2.70320 2.44322 3.62626 3.03058 3.63443
2.98521 2.50952 2.77811 3.80226 2.93167 3.62647 2.97791 2.80880
2.56244 2.75857 2.72588 5.06442 3.69328 595 a - - -

2.68618 4.42225 2.77519 2.73123 3.46354 2.40513 3.72494
3.29354 2.67741 2.69355 4.24690 2.90347 2.73739 3.18146 2.89801
2.37887 2.77519 2.98518 4.58477 3.61503
0.04848 3.44667 4.16901 0.61958 0.77255 0.48576 0.95510
453 2.65749 4.32702 3.53621 3.18694 2.69070 3.48258 3.88685
2.27098 3.03021 1.93899 3.25963 3.46725 4.00247 3.42170 3.28354
2.91685 2.99314 2.20241 4.56242 3.02464 596 l - - -
2.68618 4.42225 2.77519 2.73123 3.46354 2.40513 3.72494
3.29354 2.67741 2.69355 4.24690 2.90347 2.73739 3.18146 2.89801
2.37887 2.77519 2.98518 4.58477 3.61503
0.04848 3.44667 4.16901 0.61958 0.77255 0.48576 0.95510
454 2.56021 4.32203 3.23682 2.92448 2.74883 3.31764 3.67232
2.59538 2.80207 2.24825 3.45251 3.24312 3.86568 3.21962 3.07526
2.72902 2.85710 2.46413 4.51677 2.93029 597 l - - -
2.68618 4.42225 2.77519 2.73123 3.46354 2.40513 3.72494
3.29354 2.67741 2.69355 4.24690 2.90347 2.73739 3.18146 2.89801
2.37887 2.77519 2.98518 4.58477 3.61503
0.04848 3.44667 4.16901 0.61958 0.77255 0.48576 0.95510
455 2.39964 4.15374 3.08488 2.85747 3.03151 2.99221 3.81077
2.75692 2.81756 2.42955 3.60620 3.16044 3.02806 3.22497 3.07234
2.59219 2.79897 2.55462 4.76606 3.42580 598 a - - -
2.68618 4.42225 2.77519 2.73123 3.46354 2.40513 3.72494
3.29354 2.67741 2.69355 4.24690 2.90347 2.73739 3.18146 2.89801
2.37887 2.77519 2.98518 4.58477 3.61503
0.04848 3.44667 4.16901 0.61958 0.77255 0.48576 0.95510
456 2.51917 4.48533 2.36905 2.28465 3.71260 3.03695 3.65180
3.15038 2.51377 2.93119 3.92188 2.78299 3.65134 2.92310 2.87898
2.60062 2.86245 2.86712 5.09799 3.67101 599 e - - -
2.68618 4.42225 2.77519 2.73123 3.46354 2.40513 3.72494
3.29354 2.67741 2.69355 4.24690 2.90347 2.73739 3.18146 2.89801
2.37887 2.77519 2.98518 4.58477 3.61503
0.04848 3.44667 4.16901 0.61958 0.77255 0.48576 0.95510
457 2.22717 4.09663 2.97473 2.80485 3.61920 2.82215 3.88251
2.82425 2.77149 2.69115 3.78999 3.07498 3.58550 3.21220 3.02671
2.40907 2.46604 2.54555 5.09839 3.78334 600 a - - -
2.68618 4.42225 2.77519 2.73123 3.46354 2.40513 3.72494
3.29354 2.67741 2.69355 4.24690 2.90347 2.73739 3.18146 2.89801
2.37887 2.77519 2.98518 4.58477 3.61503
0.04848 3.44667 4.16901 0.61958 0.77255 0.48576 0.95510
458 2.47962 4.45061 2.37147 2.28202 3.77268 2.99345 3.66132
3.21911 2.54052 2.97660 3.95428 2.75502 3.55957 2.90021 2.88538
2.56379 2.83850 2.91404 5.13554 3.78082 601 e - - -
2.68618 4.42225 2.77519 2.73123 3.46354 2.40513 3.72494
3.29354 2.67741 2.69355 4.24690 2.90347 2.73739 3.18146 2.89801
2.37887 2.77519 2.98518 4.58477 3.61503
0.04848 3.44667 4.16901 0.61958 0.77255 0.48576 0.95510
459 2.47998 4.41737 2.71284 2.51707 3.57292 3.07439 3.60005
3.16977 2.35950 2.80301 3.83681 2.92101 3.68386 2.79955 2.65943
2.55439 2.81076 2.89643 5.00403 3.60453 602 k - - -
2.68618 4.42225 2.77519 2.73123 3.46354 2.40513 3.72494
3.29354 2.67741 2.69355 4.24690 2.90347 2.73739 3.18146 2.89801
2.37887 2.77519 2.98518 4.58477 3.61503
0.04848 3.44667 4.16901 0.61958 0.77255 0.48576 0.95510

460 2.53016 4.24279 3.52921 3.19163 3.16222 3.34351 4.02670
2.20619 2.96071 2.01636 3.25289 3.44564 3.92515 3.41939 3.20048
2.79950 2.90451 2.05424 4.90360 3.50495 603 l - - -
2.68618 4.42225 2.77519 2.73123 3.46354 2.40513 3.72494
3.29354 2.67741 2.69355 4.24690 2.90347 2.73739 3.18146 2.89801
2.37887 2.77519 2.98518 4.58477 3.61503
0.04848 3.44667 4.16901 0.61958 0.77255 0.48576 0.95510
461 2.55224 4.40364 3.01952 2.66006 3.17942 3.26308 3.54768
3.00681 2.39415 2.62337 3.64129 3.03052 3.77270 2.93709 2.59147
2.68363 2.82724 2.77624 4.37880 3.18373 604 k - - -
2.68618 4.42225 2.77519 2.73123 3.46354 2.40513 3.72494
3.29354 2.67741 2.69355 4.24690 2.90347 2.73739 3.18146 2.89801
2.37887 2.77519 2.98518 4.58477 3.61503
0.04848 3.44667 4.16901 0.61958 0.77255 0.48576 0.95510
462 2.55220 4.25223 3.49140 3.17820 3.13627 3.34304 4.01447
2.14926 2.96723 2.00880 3.32519 3.44124 3.92391 3.42377 3.20470
2.82136 2.92442 2.09110 4.86841 3.46174 605 l - - -
2.68618 4.42225 2.77519 2.73123 3.46354 2.40513 3.72494
3.29354 2.67741 2.69355 4.24690 2.90347 2.73739 3.18146 2.89801
2.37887 2.77519 2.98518 4.58477 3.61503
0.04848 3.44667 4.16901 0.61958 0.77255 0.48576 0.95510
463 2.54267 4.48161 2.52415 2.26669 3.62697 3.05670 3.45273
3.20397 2.45022 2.88384 3.89449 2.85033 3.66002 2.91228 2.75930
2.62679 2.85102 2.92831 5.01112 3.63784 606 e - - -
2.68618 4.42225 2.77519 2.73123 3.46354 2.40513 3.72494
3.29354 2.67741 2.69355 4.24690 2.90347 2.73739 3.18146 2.89801
2.37887 2.77519 2.98518 4.58477 3.61503
0.04848 3.44667 4.16901 0.61958 0.77255 0.48576 0.95510
464 2.56009 4.24836 3.46806 3.16935 3.13635 3.32584 4.01002
2.10542 2.97230 2.02632 3.33237 3.43699 3.90871 3.42612 3.20679
2.82939 2.93221 2.11708 4.85476 3.45927 607 l - - -
2.68618 4.42225 2.77519 2.73123 3.46354 2.40513 3.72494
3.29354 2.67741 2.69355 4.24690 2.90347 2.73739 3.18146 2.89801
2.37887 2.77519 2.98518 4.58477 3.61503
0.04848 3.44667 4.16901 0.61958 0.77255 0.48576 0.95510
465 2.26739 4.17890 2.77624 2.62114 3.65960 2.71336 3.76798
3.13100 2.63301 2.90458 3.90648 2.92178 3.57061 3.00294 2.91205
2.38263 2.65172 2.79168 5.08422 3.72115 608 a - - -
2.68618 4.42225 2.77519 2.73123 3.46354 2.40513 3.72494
3.29354 2.67741 2.69355 4.24690 2.90347 2.73739 3.18146 2.89801
2.37887 2.77519 2.98518 4.58477 3.61503
0.04848 3.44667 4.16901 0.61958 0.77255 0.48576 0.95510
466 2.46471 4.38087 2.63167 2.34178 3.66366 3.01715 3.69763
3.06284 2.51343 2.82077 3.86955 2.90190 3.65008 2.94300 2.80836
2.49500 2.83560 2.79323 5.07475 3.72016 609 e - - -
2.68618 4.42225 2.77519 2.73123 3.46354 2.40513 3.72494
3.29354 2.67741 2.69355 4.24690 2.90347 2.73739 3.18146 2.89801
2.37887 2.77519 2.98518 4.58477 3.61503
0.04848 3.44667 4.16901 0.61958 0.77255 0.48576 0.95510
467 2.36676 4.24790 2.80663 2.55196 3.65655 2.98814 3.79553
2.86175 2.62462 2.71042 3.79469 2.99758 3.64637 3.08856 2.90415
2.48193 2.60918 2.60259 5.11958 3.78612 610 a - - -
2.68618 4.42225 2.77519 2.73123 3.46354 2.40513 3.72494
3.29354 2.67741 2.69355 4.24690 2.90347 2.73739 3.18146 2.89801
2.37887 2.77519 2.98518 4.58477 3.61503

```
         0.04848  3.44667  4.16901  0.61958  0.77255  0.48576  0.95510
    468  2.49536  4.42458  2.43596  2.24512  3.77135  2.94602  3.66340
3.18496  2.52010  2.96069  3.94908  2.79763  3.61310  2.93739  2.81898
2.58692  2.85175  2.88804  5.09841  3.78786    611 e - - -
         2.68618  4.42225  2.77519  2.73123  3.46354  2.40513  3.72494
3.29354  2.67741  2.69355  4.24690  2.90347  2.73739  3.18146  2.89801
2.37887  2.77519  2.98518  4.58477  3.61503
         0.04848  3.44667  4.16901  0.61958  0.77255  0.48576  0.95510
    469  2.23847  4.08319  2.81980  2.68677  3.63548  2.44041  3.79678
3.14495  2.73590  2.93812  3.95152  2.93483  3.49122  3.15175  2.99415
2.34971  2.68684  2.78988  4.98667  3.70655    612 a - - -
         2.68618  4.42225  2.77519  2.73123  3.46354  2.40513  3.72494
3.29354  2.67741  2.69355  4.24690  2.90347  2.73739  3.18146  2.89801
2.37887  2.77519  2.98518  4.58477  3.61503
         0.04848  3.44667  4.16901  0.61958  0.77255  0.48576  0.95510
    470  2.36434  4.23423  2.99919  2.70660  3.54765  3.07852  3.83870
2.65827  2.65511  2.53847  3.61918  3.09674  3.71540  3.13706  2.92665
2.53386  2.67978  2.43211  5.09020  3.73197    613 a - - -
         2.68618  4.42225  2.77519  2.73123  3.46354  2.40513  3.72494
3.29354  2.67741  2.69355  4.24690  2.90347  2.73739  3.18146  2.89801
2.37887  2.77519  2.98518  4.58477  3.61503
         0.04848  3.44667  4.16901  0.61958  0.77255  0.48576  0.95510
    471  2.66159  4.24297  3.42943  3.17494  2.33086  3.25331  3.82289
2.44309  3.10117  2.02860  3.34838  3.43896  3.82310  3.45826  3.31394
2.91032  3.01981  2.35382  4.40558  2.93698    614 l - - -
         2.68618  4.42225  2.77519  2.73123  3.46354  2.40513  3.72494
3.29354  2.67741  2.69355  4.24690  2.90347  2.73739  3.18146  2.89801
2.37887  2.77519  2.98518  4.58477  3.61503
         0.04848  3.44667  4.16901  0.61958  0.77255  0.48576  0.95510
    472  2.60908  4.39089  3.14501  2.84016  2.95098  3.32912  3.63313
2.67468  2.60708  2.26503  3.50453  3.18827  3.86294  3.12685  2.88807
2.77918  2.93008  2.52681  4.56767  2.92160    615 l - - -
         2.68618  4.42225  2.77519  2.73123  3.46354  2.40513  3.72494
3.29354  2.67741  2.69355  4.24690  2.90347  2.73739  3.18146  2.89801
2.37887  2.77519  2.98518  4.58477  3.61503
         0.04848  3.44667  4.16901  0.61958  0.77255  0.48576  0.95510
    473  2.51797  4.22755  3.47702  3.17365  3.16783  3.29792  4.02164
2.18189  2.97625  2.04935  3.34726  3.42665  3.89446  3.42659  3.21310
2.78389  2.90044  2.06361  4.89089  3.49452    616 l - - -
         2.68618  4.42225  2.77519  2.73123  3.46354  2.40513  3.72494
3.29354  2.67741  2.69355  4.24690  2.90347  2.73739  3.18146  2.89801
2.37887  2.77519  2.98518  4.58477  3.61503
         0.04848  3.44667  4.16901  0.61958  0.77255  0.48576  0.95510
    474  2.50786  4.46590  2.37897  2.24880  3.75526  3.01158  3.66642
3.15632  2.54141  2.92468  3.93178  2.81491  3.63697  2.90635  2.88288
2.56838  2.86087  2.87292  5.12717  3.77873    617 e - - -
         2.68618  4.42225  2.77519  2.73123  3.46354  2.40513  3.72494
3.29354  2.67741  2.69355  4.24690  2.90347  2.73739  3.18146  2.89801
2.37887  2.77519  2.98518  4.58477  3.61503
         0.04848  3.44667  4.16901  0.61958  0.77255  0.48576  0.95510
    475  2.28491  4.17893  2.80992  2.65534  3.47355  2.89146  3.67659
3.13681  2.65178  2.88883  3.90771  2.91008  3.57786  3.09907  2.92192
2.24733  2.71483  2.80284  4.93355  3.51099    618 s - - -
```

2.68618 4.42225 2.77519 2.73123 3.46354 2.40513 3.72494
3.29354 2.67741 2.69355 4.24690 2.90347 2.73739 3.18146 2.89801
2.37887 2.77519 2.98518 4.58477 3.61503
0.04848 3.44667 4.16901 0.61958 0.77255 0.48576 0.95510
476 2.53878 4.23937 3.48272 3.17658 3.15276 3.32020 4.01998
2.14290 2.97629 2.03266 3.33481 3.43612 3.90829 3.42808 3.21303
2.80756 2.91547 2.08284 4.87867 3.48118 619 l - - -
2.68618 4.42225 2.77519 2.73123 3.46354 2.40513 3.72494
3.29354 2.67741 2.69355 4.24690 2.90347 2.73739 3.18146 2.89801
2.37887 2.77519 2.98518 4.58477 3.61503
0.04848 3.44667 4.16901 0.61958 0.77255 0.48576 0.95510
477 2.48057 4.40304 2.52172 2.25379 3.72633 3.01087 3.70104
3.02372 2.55055 2.82821 3.88051 2.87917 3.63758 2.97985 2.86383
2.61167 2.83259 2.76396 5.09852 3.79291 620 e - - -
2.68618 4.42225 2.77519 2.73123 3.46354 2.40513 3.72494
3.29354 2.67741 2.69355 4.24690 2.90347 2.73739 3.18146 2.89801
2.37887 2.77519 2.98518 4.58477 3.61503
0.04848 3.44667 4.16901 0.61958 0.77255 0.48576 0.95510
478 2.59573 4.27278 3.38076 3.11256 2.98380 3.28581 3.91600
2.37429 2.92384 1.89640 3.36103 3.39259 3.86859 3.38472 3.15013
2.84166 2.96451 2.25245 4.69989 3.26976 621 l - - -
2.68618 4.42225 2.77519 2.73123 3.46354 2.40513 3.72494
3.29354 2.67741 2.69355 4.24690 2.90347 2.73739 3.18146 2.89801
2.37887 2.77519 2.98518 4.58477 3.61503
0.04848 3.44667 4.16901 0.61958 0.77255 0.48576 0.95510
479 2.53924 4.25563 3.45612 3.12511 3.02844 3.34289 3.94154
2.22210 2.93530 2.08521 3.36529 3.36609 3.91438 3.37578 3.18615
2.79402 2.90348 2.13963 4.78159 3.32850 622 l - - -
2.68618 4.42225 2.77519 2.73123 3.46354 2.40513 3.72494
3.29354 2.67741 2.69355 4.24690 2.90347 2.73739 3.18146 2.89801
2.37887 2.77519 2.98518 4.58477 3.61503
0.04848 3.44667 4.16901 0.61958 0.77255 0.48576 0.95510
480 2.40596 3.96871 3.15974 2.71866 3.44858 3.11849 3.80322
2.66994 2.61568 2.43975 3.59940 3.15482 3.71937 3.12165 2.83171
2.59789 2.77662 2.44087 4.95933 3.62536 623 a - - -
2.68618 4.42225 2.77519 2.73123 3.46354 2.40513 3.72494
3.29354 2.67741 2.69355 4.24690 2.90347 2.73739 3.18146 2.89801
2.37887 2.77519 2.98518 4.58477 3.61503
0.04848 3.44667 4.16901 0.61958 0.77255 0.48576 0.95510
481 2.48999 4.25725 3.34563 3.03605 3.24597 3.30222 3.93806
2.34661 2.82141 2.13009 3.19240 3.33225 3.87626 3.29309 3.07865
2.74407 2.84643 2.19685 4.92832 3.54112 624 l - - -
2.68618 4.42225 2.77519 2.73123 3.46354 2.40513 3.72494
3.29354 2.67741 2.69355 4.24690 2.90347 2.73739 3.18146 2.89801
2.37887 2.77519 2.98518 4.58477 3.61503
0.04848 3.44667 4.16901 0.61958 0.77255 0.48576 0.95510
482 2.41669 4.34003 2.74874 2.47984 3.64569 3.04545 3.70215
2.98961 2.47442 2.82153 3.83183 2.89340 3.62127 2.98103 2.75517
2.51294 2.78784 2.71947 5.07657 3.70011 625 a - - -
2.68618 4.42225 2.77519 2.73123 3.46354 2.40513 3.72494
3.29354 2.67741 2.69355 4.24690 2.90347 2.73739 3.18146 2.89801
2.37887 2.77519 2.98518 4.58477 3.61503
0.04848 3.44667 4.16901 0.61958 0.77255 0.48576 0.95510

```
    483   2.52095  4.46021  2.56334  2.34604  3.65596  3.06219  3.65269
3.06843  2.43505  2.83472  3.85861  2.85300  3.68460  2.92597  2.77929
2.61947  2.85691  2.80003  5.05827  3.61659    626 e - - -
          2.68620  4.42226  2.77518  2.73124  3.46356  2.40514  3.72496
3.29353  2.67742  2.69356  4.24687  2.90338  2.73741  3.18145  2.89802
2.37885  2.77519  2.98520  4.58479  3.61499
          0.05286  2.96641        *  1.18043  0.36694  0.00000        *
//
HMMER3/f [3.3 | Nov 2019]
NAME  CytM1
DESC  Cyt toxin (IDOPS Model 1)
LENG  523
ALPH  amino
RF    no
MM    no
CONS  yes
CS    no
MAP   yes
DATE  Wed Feb  3 11:08:20 2021
NSEQ  10
EFFN  0.844727
CKSUM 487187701
GA 430.0 430.0
STATS LOCAL MSV      -11.8719  0.69782
STATS LOCAL VITERBI  -12.9936  0.69782
STATS LOCAL FORWARD   -6.0091  0.69782
HMM          A        C        D        E        F        G        H
I        K        L        M        N        P        Q        R        S
T        V        W        Y
            m->m     m->i     m->d     i->m     i->i     d->m     d->d
  COMPO   2.54657  4.57690  3.01008  2.77158  3.22221  3.03735  3.87224
2.69741  2.74988  2.43759  3.66582  2.75212  3.62543  2.95752  3.08454
2.64671  2.76592  2.56162  4.24937  3.40467
          2.68639  4.42246  2.77508  2.73071  3.46332  2.40534  3.72515
3.29320  2.67762  2.69346  4.24663  2.90306  2.73760  3.18151  2.89821
2.37878  2.77519  2.98539  4.58498  3.61524
          0.16281  2.70287  2.48605  2.37027  0.09812  0.00000        *
      1   2.77085  2.57469  4.26007  3.69924  3.21493  3.86193  4.27908
2.27483  3.53851  1.85126  1.94499  3.90671  4.24995  3.78703  3.72398
3.19084  3.03199  2.17544  4.91895  3.72908     32 l - - -
          2.68618  4.42225  2.77519  2.73123  3.46354  2.40513  3.72494
3.29354  2.67741  2.69355  4.24690  2.90347  2.73739  3.18146  2.89801
2.37887  2.77519  2.98518  4.58477  3.61503
          0.02657  4.03696  4.75931  0.61958  0.77255  0.55503  0.85344
      2   2.37292  4.79454  2.93850  2.41818  3.50920  3.39889  3.62109
3.46480  2.23120  3.06505  3.88053  2.50457  2.99851  2.61740  2.85944
2.62639  2.82972  3.13897  5.30305  3.96877     33 k - - -
          2.68618  4.42225  2.77519  2.73123  3.46354  2.40513  3.72494
3.29354  2.67741  2.69355  4.24690  2.90347  2.73739  3.18146  2.89801
2.37887  2.77519  2.98518  4.58477  3.61503
          0.02657  4.03696  4.75931  0.61958  0.77255  0.55503  0.85344
      3   2.63094  4.67748  3.07517  2.57131  3.91424  3.43755  3.69671
3.28745  2.45668  2.43183  3.79538  3.05192  3.32229  2.00811  2.83959
2.70923  2.67335  3.00980  5.23307  3.93882     34 q - - -
```

```
          2.68618  4.42225  2.77519  2.73123  3.46354  2.40513  3.72494
3.29354  2.67741  2.69355  4.24690  2.90347  2.73739  3.18146  2.89801
2.37887  2.77519  2.98518  4.58477  3.61503
          0.02657  4.03696  4.75931  0.61958  0.77255  0.55503  0.85344
      4   2.62328  4.68457  2.90844  2.02587  3.94054  3.06235  3.70869
3.32273  2.54959  2.22330  3.83352  2.99747  3.84393  2.90221  2.97390
2.40069  2.87818  3.02810  5.26293  3.95418      35 e - - -
          2.68618  4.42225  2.77519  2.73123  3.46354  2.40513  3.72494
3.29354  2.67741  2.69355  4.24690  2.90347  2.73739  3.18146  2.89801
2.37887  2.77519  2.98518  4.58477  3.61503
          0.02657  4.03696  4.75931  0.61958  0.77255  0.55503  0.85344
      5   2.97623  5.55116  1.57448  1.33210  4.83807  3.24489  3.79077
4.35885  2.80930  3.85839  4.71303  2.70831  3.86772  2.95732  3.40590
2.85704  3.26508  3.93052  6.00689  4.50221      36 e - - -
          2.68618  4.42225  2.77519  2.73123  3.46354  2.40513  3.72494
3.29354  2.67741  2.69355  4.24690  2.90347  2.73739  3.18146  2.89801
2.37887  2.77519  2.98518  4.58477  3.61503
          0.02657  4.03696  4.75931  0.61958  0.77255  0.55503  0.85344
      6   3.26325  4.58503  4.86546  4.30748  2.48281  4.51117  4.73241
1.78340  4.18124  1.03938  2.91145  4.52468  4.70746  4.26843  4.28758
3.85391  3.48486  2.22551  5.02713  3.83457      37 l - - -
          2.68618  4.42225  2.77519  2.73123  3.46354  2.40513  3.72494
3.29354  2.67741  2.69355  4.24690  2.90347  2.73739  3.18146  2.89801
2.37887  2.77519  2.98518  4.58477  3.61503
          0.02657  4.03696  4.75931  0.61958  0.77255  0.55503  0.85344
      7   2.61508  4.79800  2.70738  2.41289  4.03604  3.06937  3.63486
2.52221  2.24642  3.05690  3.87984  2.62172  3.81380  2.63608  2.89015
2.64819  2.84990  3.13038  5.30459  3.97277      38 k - - -
          2.68618  4.42225  2.77519  2.73123  3.46354  2.40513  3.72494
3.29354  2.67741  2.69355  4.24690  2.90347  2.73739  3.18146  2.89801
2.37887  2.77519  2.98518  4.58477  3.61503
          0.02657  4.03696  4.75931  0.61958  0.77255  0.55503  0.85344
      8   2.63050  4.59736  3.17083  2.62690  3.72753  3.48931  3.25084
2.89455  2.50154  2.78524  3.67851  3.09865  3.88365  2.06620  2.88023
2.73597  2.86245  2.44177  5.09737  3.79507      39 q - - -
          2.68618  4.42225  2.77519  2.73123  3.46354  2.40513  3.72494
3.29354  2.67741  2.69355  4.24690  2.90347  2.73739  3.18146  2.89801
2.37887  2.77519  2.98518  4.58477  3.61503
          0.02657  4.03696  4.75931  0.61958  0.77255  0.55503  0.85344
      9   2.86782  4.86478  2.81216  2.71576  4.25498  3.30073  4.06570
4.00394  2.98495  3.63287  4.58936  0.89670  3.96196  3.34115  3.34313
2.93025  3.25659  3.60934  5.54217  4.19337      40 n - - -
          2.68618  4.42225  2.77519  2.73123  3.46354  2.40513  3.72494
3.29354  2.67741  2.69355  4.24690  2.90347  2.73739  3.18146  2.89801
2.37887  2.77519  2.98518  4.58477  3.61503
          0.02657  4.03696  4.75931  0.61958  0.77255  0.55503  0.85344
     10   2.68964  4.51704  3.43920  3.08080  3.72717  3.41661  4.08137
3.07468  2.97337  2.20236  3.81944  3.42555  1.38827  3.39125  3.27238
2.87026  3.04563  2.86813  5.23360  3.93399      41 p - - -
          2.68618  4.42225  2.77519  2.73123  3.46354  2.40513  3.72494
3.29354  2.67741  2.69355  4.24690  2.90347  2.73739  3.18146  2.89801
2.37887  2.77519  2.98518  4.58477  3.61503
          0.02657  4.03696  4.75931  0.61958  0.77255  0.55503  0.85344
```

```
     11   3.00180  4.66398  3.80114  3.26678  3.02847  3.74148  3.79661
3.25420  2.71980  2.79564  3.82895  3.56325  4.16861  3.32801  2.32002
3.17221  3.24447  3.06137  1.40646  3.03973     42 w - - -
          2.68618  4.42225  2.77519  2.73123  3.46354  2.40513  3.72494
3.29354  2.67741  2.69355  4.24690  2.90347  2.73739  3.18146  2.89801
2.37887  2.77519  2.98518  4.58477  3.61503
          0.02657  4.03696  4.75931  0.61958  0.77255  0.55503  0.85344
     12   2.93029  5.18029  3.08961  2.25263  4.58367  3.55791  3.61775
3.96317  1.32831  3.42707  4.26287  3.05389  3.95887  2.74718  2.11868
2.90604  3.12331  3.61488  5.50887  4.26386     43 k - - -
          2.68618  4.42225  2.77519  2.73123  3.46354  2.40513  3.72494
3.29354  2.67741  2.69355  4.24690  2.90347  2.73739  3.18146  2.89801
2.37887  2.77519  2.98518  4.58477  3.61503
          0.02657  4.03696  4.75931  0.61958  0.77255  0.55503  0.85344
     13   2.64266  4.84892  2.92391  2.45049  4.18501  3.38939  3.64742
3.59960  2.15580  3.17942  3.99694  2.95581  3.82682  2.31349  2.76015
2.25938  2.28123  3.25478  5.38892  4.06645     44 k - - -
          2.68618  4.42225  2.77519  2.73123  3.46354  2.40513  3.72494
3.29354  2.67741  2.69355  4.24690  2.90347  2.73739  3.18146  2.89801
2.37887  2.77519  2.98518  4.58477  3.61503
          0.02657  4.03696  4.75931  0.61958  0.77255  0.44476  1.02437
     14   2.60087  4.57724  3.18116  2.62186  3.78619  2.99213  3.70658
3.16590  2.53289  2.55860  3.17177  3.09494  2.61757  2.91588  2.48969
2.70780  2.83839  2.89521  5.13375  3.85386     45 r - - -
          2.68618  4.42225  2.77519  2.73123  3.46354  2.40513  3.72494
3.29354  2.67741  2.69355  4.24690  2.90347  2.73739  3.18146  2.89801
2.37887  2.77519  2.98518  4.58477  3.61503
          0.02489  4.10147  4.82382  0.61958  0.77255  0.48576  0.95510
     15   2.96689  5.16398  2.96343  2.59213  4.50208  3.53253  3.71170
3.94520  1.95179  3.43760  4.31249  3.07280  3.99227  1.35860  2.46990
2.95349  3.18930  3.61258  5.54264  4.26613     46 q - - -
          2.68618  4.42225  2.77519  2.73123  3.46354  2.40513  3.72494
3.29354  2.67741  2.69355  4.24690  2.90347  2.73739  3.18146  2.89801
2.37887  2.77519  2.98518  4.58477  3.61503
          0.02489  4.10147  4.82382  0.61958  0.77255  0.48576  0.95510
     16   2.30997  4.25576  3.44326  3.17582  4.34005  3.02211  4.26179
3.71469  3.20984  3.44641  4.30137  3.35121  3.76748  3.53250  3.51428
1.04474  2.28039  3.20053  5.69385  4.45865     47 s - - -
          2.68618  4.42225  2.77519  2.73123  3.46354  2.40513  3.72494
3.29354  2.67741  2.69355  4.24690  2.90347  2.73739  3.18146  2.89801
2.37887  2.77519  2.98518  4.58477  3.61503
          0.02489  4.10147  4.82382  0.61958  0.77255  0.48576  0.95510
     17   2.65367  4.94640  2.94564  2.42238  4.27320  3.41620  3.62163
3.70289  2.06253  3.25105  4.04695  2.48758  3.82734  2.75150  2.46228
2.29169  2.42891  3.33867  5.43375  4.09331     48 k - - -
          2.68618  4.42225  2.77519  2.73123  3.46354  2.40513  3.72494
3.29354  2.67741  2.69355  4.24690  2.90347  2.73739  3.18146  2.89801
2.37887  2.77519  2.98518  4.58477  3.61503
          0.02489  4.10147  4.82382  0.61958  0.77255  0.48576  0.95510
     18   1.07239  4.23502  3.83100  3.47667  3.98592  3.21234  4.39304
2.85379  3.41343  2.88040  3.92369  3.58644  3.91670  3.72619  3.67665
2.65790  2.86995  2.19057  5.52595  4.30734     49 a - - -
          2.68618  4.42225  2.77519  2.73123  3.46354  2.40513  3.72494
3.29354  2.67741  2.69355  4.24690  2.90347  2.73739  3.18146  2.89801
2.37887  2.77519  2.98518  4.58477  3.61503
```

```
          0.02489  4.10147  4.82382  0.61958  0.77255  0.48576  0.95510
     19   3.15417  5.17396  3.59863  2.95266  4.62619  3.72929  3.71137
3.99670  1.83625  3.45380  4.35618  3.32358  4.12346  2.86081  1.01503
3.17061  3.33170  3.69427  5.48703  4.33737     50 r - - -
          2.68618  4.42225  2.77519  2.73123  3.46354  2.40513  3.72494
3.29354  2.67741  2.69355  4.24690  2.90347  2.73739  3.18146  2.89801
2.37887  2.77519  2.98518  4.58477  3.61503
          0.02489  4.10147  4.82382  0.61958  0.77255  0.48576  0.95510
     20   2.99291  4.47905  4.43777  4.11384  3.72844  3.95560  4.87955
2.06808  3.98532  2.37923  3.66423  4.26538  4.50765  4.33128  4.17498
3.47823  3.35157  0.77812  5.54966  4.29308     51 v - - -
          2.68618  4.42225  2.77519  2.73123  3.46354  2.40513  3.72494
3.29354  2.67741  2.69355  4.24690  2.90347  2.73739  3.18146  2.89801
2.37887  2.77519  2.98518  4.58477  3.61503
          0.02489  4.10147  4.82382  0.61958  0.77255  0.48576  0.95510
     21   3.16820  4.46542  4.92809  4.44044  3.61888  4.54308  5.14535
1.07791  4.32770  2.09867  3.41517  4.64620  4.84613  4.58577  4.51467
3.94190  3.43543  1.31237  5.61780  4.41619     52 i - - -
          2.68618  4.42225  2.77519  2.73123  3.46354  2.40513  3.72494
3.29354  2.67741  2.69355  4.24690  2.90347  2.73739  3.18146  2.89801
2.37887  2.77519  2.98518  4.58477  3.61503
          0.02489  4.10147  4.82382  0.61958  0.77255  0.48576  0.95510
     22   2.73260  4.20305  4.12892  3.56398  3.31582  3.86149  4.20274
1.63913  3.45250  2.22615  3.27633  3.81650  4.22585  3.70662  3.67262
3.16115  2.43704  1.78884  4.91803  3.19658     53 i - - -
          2.68618  4.42225  2.77519  2.73123  3.46354  2.40513  3.72494
3.29354  2.67741  2.69355  4.24690  2.90347  2.73739  3.18146  2.89801
2.37887  2.77519  2.98518  4.58477  3.61503
          0.02489  4.10147  4.82382  0.61958  0.77255  0.48576  0.95510
     23   3.42193  4.81975  4.55640  4.20329  3.23883  4.27043  4.78781
2.46132  3.96896  0.63698  3.20089  4.45661  4.67022  4.28182  4.10509
3.87730  3.70786  2.55699  5.19486  3.96904     54 L - - -
          2.68618  4.42225  2.77519  2.73123  3.46354  2.40513  3.72494
3.29354  2.67741  2.69355  4.24690  2.90347  2.73739  3.18146  2.89801
2.37887  2.77519  2.98518  4.58477  3.61503
          0.02489  4.10147  4.82382  0.61958  0.77255  0.48576  0.95510
     24   2.81290  5.07615  3.11391  2.54898  4.44192  3.52715  3.62250
3.84492  1.99778  3.34543  4.16025  3.03518  3.91760  2.28603  1.81921
2.25367  3.02147  3.49059  5.46949  4.18851     55 r - - -
          2.68618  4.42225  2.77519  2.73123  3.46354  2.40513  3.72494
3.29354  2.67741  2.69355  4.24690  2.90347  2.73739  3.18146  2.89801
2.37887  2.77519  2.98518  4.58477  3.61503
          0.02489  4.10147  4.82382  0.61958  0.77255  0.48576  0.95510
     25   2.84332  4.32797  4.29493  3.78336  3.61615  3.96379  4.55742
1.87547  3.67551  2.33329  3.48497  4.02646  4.39934  3.97289  3.92494
3.32132  2.31557  1.18704  5.33926  4.12580     56 v - - -
          2.68618  4.42225  2.77519  2.73123  3.46354  2.40513  3.72494
3.29354  2.67741  2.69355  4.24690  2.90347  2.73739  3.18146  2.89801
2.37887  2.77519  2.98518  4.58477  3.61503
          0.09585  4.10147  2.59224  0.61958  0.77255  0.48576  0.95510
     26   2.75964  5.23570  2.30080  2.00175  4.53885  3.30591  3.66006
4.01746  2.30435  3.52457  4.31853  1.87237  3.29069  2.79431  3.01233
2.69894  3.01155  3.60972  5.68724  4.25911     57 n - - -
```

2.68618 4.42225 2.77519 2.73123 3.46354 2.40513 3.72494
3.29354 2.67741 2.69355 4.24690 2.90347 2.73739 3.18146 2.89801
2.37887 2.77519 2.98518 4.58477 3.61503
0.02670 4.03232 4.75466 0.61958 0.77255 0.44217 1.02901
27 2.96350 5.46006 1.51827 2.18508 4.78238 3.26795 3.83512
4.34439 2.85135 3.85683 4.71023 1.54746 3.89501 3.00773 3.43410
2.86753 3.26642 3.90946 5.98287 4.48610 58 d - - -
2.68618 4.42225 2.77519 2.73123 3.46354 2.40513 3.72494
3.29354 2.67741 2.69355 4.24690 2.90347 2.73739 3.18146 2.89801
2.37887 2.77519 2.98518 4.58477 3.61503
0.02489 4.10147 4.82382 0.61958 0.77255 0.48576 0.95510
28 2.01937 4.35837 3.36560 3.14253 4.32280 3.10860 4.27096
3.64969 3.21038 3.38454 4.30403 3.37507 1.15943 3.54820 3.51326
2.60176 2.90182 3.20542 5.67515 4.45582 59 p - - -
2.68618 4.42225 2.77519 2.73123 3.46354 2.40513 3.72494
3.29354 2.67741 2.69355 4.24690 2.90347 2.73739 3.18146 2.89801
2.37887 2.77519 2.98518 4.58477 3.61503
0.02489 4.10147 4.82382 0.61958 0.77255 0.48576 0.95510
29 2.23445 4.42624 3.29576 2.74759 3.63562 2.98547 3.78185
2.72168 2.71433 2.44403 3.56844 2.50244 3.89505 3.04202 3.10722
2.73388 2.82061 2.46791 5.04324 3.78365 60 a - - -
2.68618 4.42225 2.77519 2.73123 3.46354 2.40513 3.72494
3.29354 2.67741 2.69355 4.24690 2.90347 2.73739 3.18146 2.89801
2.37887 2.77519 2.98518 4.58477 3.61503
0.02489 4.10147 4.82382 0.61958 0.77255 0.48576 0.95510
30 2.63472 4.99351 2.39794 1.99964 4.26940 3.38126 3.61169
3.72110 2.39344 3.26980 4.05584 2.87325 3.79945 2.37033 2.87606
2.62244 2.86973 2.96591 5.45943 3.33773 61 e - - -
2.68618 4.42225 2.77519 2.73123 3.46354 2.40513 3.72494
3.29354 2.67741 2.69355 4.24690 2.90347 2.73739 3.18146 2.89801
2.37887 2.77519 2.98518 4.58477 3.61503
0.02489 4.10147 4.82382 0.61958 0.77255 0.48576 0.95510
31 2.59537 4.46322 3.27500 2.79887 3.74609 3.45412 3.86387
2.26719 2.76354 2.76495 3.68150 2.22132 3.92277 3.11754 3.13936
2.76006 2.20496 2.67803 5.16584 3.89538 62 t - - -
2.68618 4.42225 2.77519 2.73123 3.46354 2.40513 3.72494
3.29354 2.67741 2.69355 4.24690 2.90347 2.73739 3.18146 2.89801
2.37887 2.77519 2.98518 4.58477 3.61503
0.02489 4.10147 4.82382 0.61958 0.77255 0.48576 0.95510
32 2.57422 4.57779 3.10824 2.67252 3.96860 3.35644 3.82450
2.93027 2.66211 3.00554 3.88560 1.91861 3.86542 3.03120 3.05934
2.68379 2.08172 2.95477 5.31984 4.01801 63 n - - -
2.68618 4.42225 2.77519 2.73123 3.46354 2.40513 3.72494
3.29354 2.67741 2.69355 4.24690 2.90347 2.73739 3.18146 2.89801
2.37887 2.77519 2.98518 4.58477 3.61503
0.02489 4.10147 4.82382 0.61958 0.77255 0.48576 0.95510
33 2.86217 5.13824 2.58914 2.39400 4.43873 3.36023 3.77615
4.00519 2.53164 3.53689 4.39429 1.44366 3.91240 2.24368 2.92549
2.83814 3.14324 3.62575 5.63655 4.24346 64 n - - -
2.68618 4.42225 2.77519 2.73123 3.46354 2.40513 3.72494
3.29354 2.67741 2.69355 4.24690 2.90347 2.73739 3.18146 2.89801
2.37887 2.77519 2.98518 4.58477 3.61503
0.02489 4.10147 4.82382 0.61958 0.77255 0.48576 0.95510

```
     34   2.52211  4.31221  3.51771  3.03175  3.65387  3.39350  3.97827
2.89631  2.95005  1.93315  3.61659  3.36323  3.92089  3.29275  3.27482
2.22852  1.99197  2.64805  5.11582  3.87508      65 l - - -
          2.68618  4.42225  2.77519  2.73123  3.46354  2.40513  3.72494
3.29354  2.67741  2.69355  4.24690  2.90347  2.73739  3.18146  2.89801
2.37887  2.77519  2.98518  4.58477  3.61503
          0.02489  4.10147  4.82382  0.61958  0.77255  0.48576  0.95510
     35   3.42193  4.81975  4.55640  4.20329  3.23883  4.27043  4.78781
2.46132  3.96896  0.63698  3.20089  4.45661  4.67022  4.28182  4.10509
3.87730  3.70786  2.55699  5.19486  3.96904      66 L - - -
          2.68618  4.42225  2.77519  2.73123  3.46354  2.40513  3.72494
3.29354  2.67741  2.69355  4.24690  2.90347  2.73739  3.18146  2.89801
2.37887  2.77519  2.98518  4.58477  3.61503
          0.02489  4.10147  4.82382  0.61958  0.77255  0.48576  0.95510
     36   2.91700  5.44922  1.39248  1.86251  4.76543  3.27751  3.78151
4.27140  2.75630  3.77556  4.60412  2.74871  3.87247  2.93850  3.33311
2.22063  3.19974  3.84412  5.94114  4.45510      67 d - - -
          2.68618  4.42225  2.77519  2.73123  3.46354  2.40513  3.72494
3.29354  2.67741  2.69355  4.24690  2.90347  2.73739  3.18146  2.89801
2.37887  2.77519  2.98518  4.58477  3.61503
          0.02489  4.10147  4.82382  0.61958  0.77255  0.48576  0.95510
     37   3.16999  4.48699  4.87608  4.39934  3.57550  4.49778  5.10359
0.97907  4.26912  2.04962  3.38441  4.60794  4.81895  4.53612  4.45568
3.90336  3.44299  1.51225  5.58057  4.37547      68 i - - -
          2.68618  4.42225  2.77519  2.73123  3.46354  2.40513  3.72494
3.29354  2.67741  2.69355  4.24690  2.90347  2.73739  3.18146  2.89801
2.37887  2.77519  2.98518  4.58477  3.61503
          0.02489  4.10147  4.82382  0.61958  0.77255  0.48576  0.95510
     38   2.11587  4.43810  3.15651  2.80907  4.18079  3.17545  3.98224
3.52699  2.86515  3.23478  4.08267  2.38558  3.80614  3.20161  3.24629
2.57785  1.54085  3.12404  5.50839  4.23623      69 t - - -
          2.68618  4.42225  2.77519  2.73123  3.46354  2.40513  3.72494
3.29354  2.67741  2.69355  4.24690  2.90347  2.73739  3.18146  2.89801
2.37887  2.77519  2.98518  4.58477  3.61503
          0.02489  4.10147  4.82382  0.61958  0.77255  0.48576  0.95510
     39   2.85922  5.39044  2.09829  1.61001  4.68890  3.31340  3.72035
4.18655  2.59795  3.67563  4.48080  2.77277  3.85690  1.92005  3.12422
2.77543  3.11920  3.76476  5.82596  4.37034      70 e - - -
          2.68618  4.42225  2.77519  2.73123  3.46354  2.40513  3.72494
3.29354  2.67741  2.69355  4.24690  2.90347  2.73739  3.18146  2.89801
2.37887  2.77519  2.98518  4.58477  3.61503
          0.02489  4.10147  4.82382  0.61958  0.77255  0.48576  0.95510
     40   3.16798  4.50212  4.82780  4.36538  3.54723  4.45444  5.06666
0.94500  4.21879  2.02675  3.37211  4.57326  4.79460  4.49859  4.40425
3.86854  3.44703  1.61831  5.55038  4.33474      71 i - - -
          2.68618  4.42225  2.77519  2.73123  3.46354  2.40513  3.72494
3.29354  2.67741  2.69355  4.24690  2.90347  2.73739  3.18146  2.89801
2.37887  2.77519  2.98518  4.58477  3.61503
          0.02489  4.10147  4.82382  0.61958  0.77255  0.48576  0.95510
     41   2.89757  5.43242  2.06533  1.29163  4.73010  3.30814  3.74283
4.22467  2.31521  3.71774  4.53418  2.76741  3.86994  2.88946  3.16805
2.80646  3.16239  3.80492  5.87015  4.40869      72 e - - -
          2.68618  4.42225  2.77519  2.73123  3.46354  2.40513  3.72494
3.29354  2.67741  2.69355  4.24690  2.90347  2.73739  3.18146  2.89801
2.37887  2.77519  2.98518  4.58477  3.61503
```

0.02489 4.10147 4.82382 0.61958 0.77255 0.48576 0.95510
42 2.96069 5.45237 1.55787 2.18783 4.77584 3.26853 3.83496
4.33761 2.84905 3.85142 4.70463 1.51188 3.89483 3.00766 3.42973
2.86622 3.26368 3.90346 5.97715 4.48227 73 n - - -
2.68618 4.42225 2.77519 2.73123 3.46354 2.40513 3.72494
3.29354 2.67741 2.69355 4.24690 2.90347 2.73739 3.18146 2.89801
2.37887 2.77519 2.98518 4.58477 3.61503
0.02489 4.10147 4.82382 0.61958 0.77255 0.48576 0.95510
43 2.69882 4.28958 3.82253 3.27089 3.37539 3.73191 4.07654
2.00602 3.17149 1.79902 3.30816 3.60409 2.67268 3.47133 3.45718
3.02303 2.72729 2.28066 4.96173 3.75525 74 l - - -
2.68618 4.42225 2.77519 2.73123 3.46354 2.40513 3.72494
3.29354 2.67741 2.69355 4.24690 2.90347 2.73739 3.18146 2.89801
2.37887 2.77519 2.98518 4.58477 3.61503
0.02489 4.10147 4.82382 0.61958 0.77255 0.48576 0.95510
44 2.31259 4.53292 3.15753 2.68811 3.90931 3.37430 3.81924
2.70493 2.68140 2.93209 3.80897 1.90891 3.86495 3.03207 3.08584
2.68083 2.51658 2.87126 5.27172 3.98723 75 n - - -
2.68618 4.42225 2.77519 2.73123 3.46354 2.40513 3.72494
3.29354 2.67741 2.69355 4.24690 2.90347 2.73739 3.18146 2.89801
2.37887 2.77519 2.98518 4.58477 3.61503
0.02489 4.10147 4.82382 0.61958 0.77255 0.48576 0.95510
45 3.26332 4.91951 3.71188 3.31987 2.34383 3.96823 1.58770
3.54916 3.14964 2.98210 4.07779 3.58555 4.36103 3.50756 3.41614
3.32194 3.49566 3.35894 3.98029 1.55032 76 y - - -
2.68618 4.42225 2.77519 2.73123 3.46354 2.40513 3.72494
3.29354 2.67741 2.69355 4.24690 2.90347 2.73739 3.18146 2.89801
2.37887 2.77519 2.98518 4.58477 3.61503
0.02489 4.10147 4.82382 0.61958 0.77255 0.48576 0.95510
46 3.30216 4.62242 4.90762 4.36213 3.22094 4.58890 4.95573
1.82474 4.21905 0.94275 3.01405 4.61664 4.79769 4.36979 4.35943
3.95000 3.53670 1.90007 5.29765 4.20094 77 l - - -
2.68618 4.42225 2.77519 2.73123 3.46354 2.40513 3.72494
3.29354 2.67741 2.69355 4.24690 2.90347 2.73739 3.18146 2.89801
2.37887 2.77519 2.98518 4.58477 3.61503
0.02489 4.10147 4.82382 0.61958 0.77255 0.48576 0.95510
47 2.63248 4.76144 2.97187 2.28065 3.99421 3.43368 3.66492
3.39765 2.45916 2.28280 3.85223 2.98763 3.19749 2.42089 2.88889
2.45831 2.86747 3.09321 5.27892 3.96282 78 e - - -
2.68618 4.42225 2.77519 2.73123 3.46354 2.40513 3.72494
3.29354 2.67741 2.69355 4.24690 2.90347 2.73739 3.18146 2.89801
2.37887 2.77519 2.98518 4.58477 3.61503
0.02489 4.10147 4.82382 0.61958 0.77255 0.48576 0.95510
48 3.08480 5.06424 3.07612 2.84103 4.26228 3.53160 3.99906
3.93485 2.64723 3.41356 4.44127 3.30042 4.09235 0.87547 2.92413
3.12578 3.38456 3.65206 5.49605 4.20789 79 q - - -
2.68618 4.42225 2.77519 2.73123 3.46354 2.40513 3.72494
3.29354 2.67741 2.69355 4.24690 2.90347 2.73739 3.18146 2.89801
2.37887 2.77519 2.98518 4.58477 3.61503
0.02489 4.10147 4.82382 0.61958 0.77255 0.48576 0.95510
49 0.72813 4.32237 3.71027 3.51950 4.29523 3.13943 4.51231
3.45615 3.54208 3.32659 4.33814 3.60635 3.90095 3.86312 3.77237
2.67322 2.96682 3.07018 5.69905 4.52711 80 a - - -

```
         2.68618  4.42225  2.77519  2.73123  3.46354  2.40513  3.72494
3.29354  2.67741  2.69355  4.24690  2.90347  2.73739  3.18146  2.89801
2.37887  2.77519  2.98518  4.58477  3.61503
         0.02489  4.10147  4.82382  0.61958  0.77255  0.48576  0.95510
     50  3.21326  4.57656  4.64906  4.27850  3.53877  4.30474  4.96974
0.83207  4.11119  2.08442  3.44515  4.49270  4.71954  4.43969  4.28244
3.82758  3.51063  1.88006  5.47271  4.22685     81 i - - -
         2.68618  4.42225  2.77519  2.73123  3.46354  2.40513  3.72494
3.29354  2.67741  2.69355  4.24690  2.90347  2.73739  3.18146  2.89801
2.37887  2.77519  2.98518  4.58477  3.61503
         0.02489  4.10147  4.82382  0.61958  0.77255  0.48576  0.95510
     51  2.67075  4.93998  2.35045  2.06423  4.18772  3.39026  3.65557
3.61277  2.46805  3.19556  2.44113  2.89888  3.82899  2.59601  2.94013
2.67475  2.90930  3.27601  5.42838  4.06924     82 e - - -
         2.68618  4.42225  2.77519  2.73123  3.46354  2.40513  3.72494
3.29354  2.67741  2.69355  4.24690  2.90347  2.73739  3.18146  2.89801
2.37887  2.77519  2.98518  4.58477  3.61503
         0.02489  4.10147  4.82382  0.61958  0.77255  0.48576  0.95510
     52  3.42193  4.81975  4.55640  4.20329  3.23883  4.27043  4.78781
2.46132  3.96896  0.63698  3.20089  4.45661  4.67022  4.28182  4.10509
3.87730  3.70786  2.55699  5.19486  3.96904     83 L - - -
         2.68618  4.42225  2.77519  2.73123  3.46354  2.40513  3.72494
3.29354  2.67741  2.69355  4.24690  2.90347  2.73739  3.18146  2.89801
2.37887  2.77519  2.98518  4.58477  3.61503
         0.02489  4.10147  4.82382  0.61958  0.77255  0.48576  0.95510
     53  1.17764  4.19767  3.55617  3.27666  4.34048  2.99062  4.31949
3.69848  3.31858  3.44220  4.28683  3.39258  3.74961  3.60592  3.61301
1.62756  2.76186  3.17043  5.70395  4.49368     84 a - - -
         2.68618  4.42225  2.77519  2.73123  3.46354  2.40513  3.72494
3.29354  2.67741  2.69355  4.24690  2.90347  2.73739  3.18146  2.89801
2.37887  2.77519  2.98518  4.58477  3.61503
         0.02489  4.10147  4.82382  0.61958  0.77255  0.48576  0.95510
     54  2.27674  4.63888  2.80673  2.61241  4.41783  3.16675  3.96297
3.86953  2.85103  3.50765  4.34512  1.33775  3.82196  3.16384  3.26358
2.37962  2.94824  3.40618  5.69551  4.36033     85 n - - -
         2.68618  4.42225  2.77519  2.73123  3.46354  2.40513  3.72494
3.29354  2.67741  2.69355  4.24690  2.90347  2.73739  3.18146  2.89801
2.37887  2.77519  2.98518  4.58477  3.61503
         0.02489  4.10147  4.82382  0.61958  0.77255  0.48576  0.95510
     55  1.23891  4.45649  3.07575  2.83221  4.28121  3.13936  4.06259
3.66160  2.97133  3.37669  4.23673  2.18876  3.81598  3.29548  3.34154
2.59392  2.88532  3.22384  5.61203  4.32331     86 a - - -
         2.68618  4.42225  2.77519  2.73123  3.46354  2.40513  3.72494
3.29354  2.67741  2.69355  4.24690  2.90347  2.73739  3.18146  2.89801
2.37887  2.77519  2.98518  4.58477  3.61503
         0.02489  4.10147  4.82382  0.61958  0.77255  0.48576  0.95510
     56  3.56471  4.90103  4.55393  4.31729  0.70012  4.18950  4.00019
3.06652  4.22012  2.38609  3.76599  4.28501  4.64698  4.32610  4.28291
3.81902  3.86801  3.07315  4.19139  2.53382     87 f - - -
         2.68618  4.42225  2.77519  2.73123  3.46354  2.40513  3.72494
3.29354  2.67741  2.69355  4.24690  2.90347  2.73739  3.18146  2.89801
2.37887  2.77519  2.98518  4.58477  3.61503
         0.02489  4.10147  4.82382  0.61958  0.77255  0.48576  0.95510
```

```
     57   3.08480  5.06424  3.07612  2.84103  4.26228  3.53160  3.99906
3.93485  2.64723  3.41356  4.44127  3.30042  4.09235  0.87547  2.92413
3.12578  3.38456  3.65206  5.49605  4.20789     88 q - - -
          2.68618  4.42225  2.77519  2.73123  3.46354  2.40513  3.72494
3.29354  2.67741  2.69355  4.24690  2.90347  2.73739  3.18146  2.89801
2.37887  2.77519  2.98518  4.58477  3.61503
          0.02489  4.10147  4.82382  0.61958  0.77255  0.48576  0.95510
     58   2.20063  5.11786  1.93909  2.27650  4.45873  3.32671  3.67860
3.92492  2.36650  3.45546  4.24666  2.49924  3.81957  2.81309  3.02419
2.37522  2.97138  3.52349  5.63375  4.22557     89 d - - -
          2.68618  4.42225  2.77519  2.73123  3.46354  2.40513  3.72494
3.29354  2.67741  2.69355  4.24690  2.90347  2.73739  3.18146  2.89801
2.37887  2.77519  2.98518  4.58477  3.61503
          0.02489  4.10147  4.82382  0.61958  0.77255  0.48576  0.95510
     59   0.72813  4.32237  3.71027  3.51950  4.29523  3.13943  4.51231
3.45615  3.54208  3.32659  4.33814  3.60635  3.90095  3.86312  3.77237
2.67322  2.96682  3.07018  5.69905  4.52711     90 a - - -
          2.68618  4.42225  2.77519  2.73123  3.46354  2.40513  3.72494
3.29354  2.67741  2.69355  4.24690  2.90347  2.73739  3.18146  2.89801
2.37887  2.77519  2.98518  4.58477  3.61503
          0.02489  4.10147  4.82382  0.61958  0.77255  0.48576  0.95510
     60   3.42193  4.81975  4.55640  4.20329  3.23883  4.27043  4.78781
2.46132  3.96896  0.63698  3.20089  4.45661  4.67022  4.28182  4.10509
3.87730  3.70786  2.55699  5.19486  3.96904     91 L - - -
          2.68618  4.42225  2.77519  2.73123  3.46354  2.40513  3.72494
3.29354  2.67741  2.69355  4.24690  2.90347  2.73739  3.18146  2.89801
2.37887  2.77519  2.98518  4.58477  3.61503
          0.02489  4.10147  4.82382  0.61958  0.77255  0.48576  0.95510
     61   3.11551  4.45215  4.79999  4.32265  3.63844  4.42061  5.05286
1.60577  4.19976  2.15928  3.45732  4.53151  4.77137  4.48617  4.40393
3.82192  3.39488  0.91476  5.59619  4.37038     92 v - - -
          2.68618  4.42225  2.77519  2.73123  3.46354  2.40513  3.72494
3.29354  2.67741  2.69355  4.24690  2.90347  2.73739  3.18146  2.89801
2.37887  2.77519  2.98518  4.58477  3.61503
          0.02489  4.10147  4.82382  0.61958  0.77255  0.48576  0.95510
     62   2.45156  4.39996  3.24432  3.05639  4.32945  3.10448  4.22642
3.81590  3.15453  3.49744  4.38369  3.32177  1.10339  3.50328  3.46261
2.29638  2.92145  3.32530  5.66447  4.40074     93 p - - -
          2.68618  4.42225  2.77519  2.73123  3.46354  2.40513  3.72494
3.29354  2.67741  2.69355  4.24690  2.90347  2.73739  3.18146  2.89801
2.37887  2.77519  2.98518  4.58477  3.61503
          0.02489  4.10147  4.82382  0.61958  0.77255  0.48576  0.95510
     63   2.55301  4.39644  3.65012  3.42066  4.18963  3.22621  4.41809
3.34560  3.36819  3.20076  4.24138  3.58759  3.95032  3.75275  3.61152
2.75749  0.83539  3.01507  5.60667  4.40820     94 t - - -
          2.68618  4.42225  2.77519  2.73123  3.46354  2.40513  3.72494
3.29354  2.67741  2.69355  4.24690  2.90347  2.73739  3.18146  2.89801
2.37887  2.77519  2.98518  4.58477  3.61503
          0.02489  4.10147  4.82382  0.61958  0.77255  0.48576  0.95510
     64   2.55314  4.66568  2.92178  2.33885  4.20387  3.27138  3.80258
3.61652  2.64127  3.23727  4.06198  3.01927  3.27953  2.97905  3.07446
1.57970  2.47837  3.23409  5.47695  4.16030     95 s - - -
          2.68618  4.42225  2.77519  2.73123  3.46354  2.40513  3.72494
3.29354  2.67741  2.69355  4.24690  2.90347  2.73739  3.18146  2.89801
2.37887  2.77519  2.98518  4.58477  3.61503
```

```
          0.02489  4.10147  4.82382  0.61958  0.77255  0.48576  0.95510
     65   2.45755  4.35322  3.40525  2.94353  3.87884  3.26835  3.97449
3.17235  2.88780  2.49817  3.82359  3.27873  3.85009  3.23704  3.23958
1.73604  1.94863  2.85835  5.28243  4.02585     96 s - - -
          2.68618  4.42225  2.77519  2.73123  3.46354  2.40513  3.72494
3.29354  2.67741  2.69355  4.24690  2.90347  2.73739  3.18146  2.89801
2.37887  2.77519  2.98518  4.58477  3.61503
          0.02489  4.10147  4.82382  0.61958  0.77255  0.48576  0.95510
     66   2.96640  5.57558  1.74634  1.34373  4.86151  3.27413  3.78693
4.38912  2.77973  3.86638  4.69768  2.30775  3.88034  2.94349  3.37364
2.84857  3.24731  3.94772  6.01786  4.50479     97 e - - -
          2.68618  4.42225  2.77519  2.73123  3.46354  2.40513  3.72494
3.29354  2.67741  2.69355  4.24690  2.90347  2.73739  3.18146  2.89801
2.37887  2.77519  2.98518  4.58477  3.61503
          0.02489  4.10147  4.82382  0.61958  0.77255  0.48576  0.95510
     67   3.56471  4.90103  4.55393  4.31729  0.70012  4.18950  4.00019
3.06652  4.22012  2.38609  3.76599  4.28501  4.64698  4.32610  4.28291
3.81902  3.86801  3.07315  4.19139  2.53382     98 f - - -
          2.68618  4.42225  2.77519  2.73123  3.46354  2.40513  3.72494
3.29354  2.67741  2.69355  4.24690  2.90347  2.73739  3.18146  2.89801
2.37887  2.77519  2.98518  4.58477  3.61503
          0.02489  4.10147  4.82382  0.61958  0.77255  0.48576  0.95510
     68   2.11914  4.32019  3.37182  3.24000  4.51581  0.92192  4.40693
3.91743  3.42742  3.64991  4.52345  3.41340  3.81517  3.70794  3.71084
2.56750  2.90197  3.35849  5.82284  4.64059     99 g - - -
          2.68618  4.42225  2.77519  2.73123  3.46354  2.40513  3.72494
3.29354  2.67741  2.69355  4.24690  2.90347  2.73739  3.18146  2.89801
2.37887  2.77519  2.98518  4.58477  3.61503
          0.19808  4.10147  1.81309  0.61958  0.77255  0.48576  0.95510
     69   2.72789  5.09675  2.68621  1.97452  4.41883  3.36315  3.59405
3.86158  2.26519  3.37057  4.17398  2.32126  3.81313  2.42513  2.36094
2.69429  2.95848  3.48403  5.51693  4.16209    100 e - - -
          2.68618  4.42225  2.77519  2.73123  3.46354  2.40513  3.72494
3.29354  2.67741  2.69355  4.24690  2.90347  2.73739  3.18146  2.89801
2.37887  2.77519  2.98518  4.58477  3.61503
          0.02953  3.93292  4.65526  0.61958  0.77255  0.39543  1.11899
     70   3.00754  5.62041  1.23909  1.94386  4.90226  3.26471  3.81533
4.43736  2.84730  3.91932  4.76809  2.10878  3.89283  2.98068  3.45873
2.88113  3.29776  3.99657  6.07413  4.54686    101 d - - -
          2.68618  4.42225  2.77519  2.73123  3.46354  2.40513  3.72494
3.29354  2.67741  2.69355  4.24690  2.90347  2.73739  3.18146  2.89801
2.37887  2.77519  2.98518  4.58477  3.61503
          0.02489  4.10147  4.82382  0.61958  0.77255  0.48576  0.95510
     71   1.79086  4.23027  3.71266  3.26829  3.92046  3.22483  4.20782
2.91828  3.20890  2.89211  3.84068  3.46821  3.87679  3.51772  3.51563
2.62689  1.59390  2.26677  5.38891  4.17490    102 t - - -
          2.68618  4.42225  2.77519  2.73123  3.46354  2.40513  3.72494
3.29354  2.67741  2.69355  4.24690  2.90347  2.73739  3.18146  2.89801
2.37887  2.77519  2.98518  4.58477  3.61503
          0.02489  4.10147  4.82382  0.61958  0.77255  0.48576  0.95510
     72   3.37982  4.72229  4.85146  4.33914  3.14015  4.58280  4.90747
1.97180  4.13187  0.79905  2.97616  4.60400  4.80356  4.32743  4.27006
3.97304  3.61885  2.24396  5.22995  4.08186    103 l - - -
```

```
          2.68618  4.42225  2.77519  2.73123  3.46354  2.40513  3.72494
3.29354  2.67741  2.69355  4.24690  2.90347  2.73739  3.18146  2.89801
2.37887  2.77519  2.98518  4.58477  3.61503
          0.02489  4.10147  4.82382  0.61958  0.77255  0.48576  0.95510
     73   3.10635  5.14510  3.49955  2.90205  4.52214  3.69404  3.71157
3.96014  2.00107  3.40945  4.32010  3.28873  4.10029  2.57223  1.05453
3.12411  3.29654  3.65941  5.46278  4.27337    104 r - - -
          2.68618  4.42225  2.77519  2.73123  3.46354  2.40513  3.72494
3.29354  2.67741  2.69355  4.24690  2.90347  2.73739  3.18146  2.89801
2.37887  2.77519  2.98518  4.58477  3.61503
          0.02489  4.10147  4.82382  0.61958  0.77255  0.48576  0.95510
     74   3.56471  4.90103  4.55393  4.31729  0.70012  4.18950  4.00019
3.06652  4.22012  2.38609  3.76599  4.28501  4.64698  4.32610  4.28291
3.81902  3.86801  3.07315  4.19139  2.53382    105 f - - -
          2.68618  4.42225  2.77519  2.73123  3.46354  2.40513  3.72494
3.29354  2.67741  2.69355  4.24690  2.90347  2.73739  3.18146  2.89801
2.37887  2.77519  2.98518  4.58477  3.61503
          0.02489  4.10147  4.82382  0.61958  0.77255  0.48576  0.95510
     75   2.79680  5.11511  1.39820  2.29587  4.62596  3.23861  3.85721
4.13423  2.83734  3.69940  4.54023  2.84202  3.86292  3.03287  3.37677
1.83271  3.13030  3.69260  5.85850  4.42025    106 d - - -
          2.68618  4.42225  2.77519  2.73123  3.46354  2.40513  3.72494
3.29354  2.67741  2.69355  4.24690  2.90347  2.73739  3.18146  2.89801
2.37887  2.77519  2.98518  4.58477  3.61503
          0.02489  4.10147  4.82382  0.61958  0.77255  0.48576  0.95510
     76   3.08993  4.42557  4.75654  4.20386  3.35489  4.38159  4.78908
1.65577  4.08418  1.86532  2.07020  4.41938  4.65595  4.27369  4.24164
3.72369  3.33048  1.41315  5.26581  4.12611    107 v - - -
          2.68618  4.42225  2.77519  2.73123  3.46354  2.40513  3.72494
3.29354  2.67741  2.69355  4.24690  2.90347  2.73739  3.18146  2.89801
2.37887  2.77519  2.98518  4.58477  3.61503
          0.02489  4.10147  4.82382  0.61958  0.77255  0.48576  0.95510
     77   2.48924  4.94352  2.77374  1.99336  4.30327  3.34494  3.65802
3.74912  2.45805  3.30554  4.09716  2.59714  3.07531  2.79272  2.93793
2.14225  2.69949  3.36574  5.50191  4.12987    108 e - - -
          2.68618  4.42225  2.77519  2.73123  3.46354  2.40513  3.72494
3.29354  2.67741  2.69355  4.24690  2.90347  2.73739  3.18146  2.89801
2.37887  2.77519  2.98518  4.58477  3.61503
          0.02489  4.10147  4.82382  0.61958  0.77255  0.48576  0.95510
     78   3.04252  5.24946  3.41911  2.74823  4.70810  3.68443  3.62282
4.05802  1.48270  3.47684  4.32005  3.17719  4.04036  2.13211  1.64956
3.01942  3.20711  3.71709  5.51442  4.32387    109 k - - -
          2.68618  4.42225  2.77519  2.73123  3.46354  2.40513  3.72494
3.29354  2.67741  2.69355  4.24690  2.90347  2.73739  3.18146  2.89801
2.37887  2.77519  2.98518  4.58477  3.61503
          0.02489  4.10147  4.82382  0.61958  0.77255  0.48576  0.95510
     79   2.97529  4.72177  3.69547  3.62698  4.75326  0.44951  4.72741
4.41436  3.83906  4.03771  5.01959  3.86061  4.11981  4.14550  4.04435
3.15472  3.47432  3.89839  5.78195  4.86495    110 G - - -
          2.68618  4.42225  2.77519  2.73123  3.46354  2.40513  3.72494
3.29354  2.67741  2.69355  4.24690  2.90347  2.73739  3.18146  2.89801
2.37887  2.77519  2.98518  4.58477  3.61503
          0.02489  4.10147  4.82382  0.61958  0.77255  0.48576  0.95510
```

80 3.36016 4.67209 4.95768 4.40151 2.50679 4.60831 4.80319
1.96642 4.27175 0.88584 2.90609 4.62219 4.78118 4.33619 4.36579
3.95680 3.57858 2.32759 5.05525 3.86322 111 l - - -
2.68618 4.42225 2.77519 2.73123 3.46354 2.40513 3.72494
3.29354 2.67741 2.69355 4.24690 2.90347 2.73739 3.18146 2.89801
2.37887 2.77519 2.98518 4.58477 3.61503
0.02489 4.10147 4.82382 0.61958 0.77255 0.48576 0.95510
81 2.73840 5.18210 2.56824 1.77387 4.50174 2.82098 3.66436
3.97309 2.47505 3.48555 4.27306 2.49804 3.82598 2.31717 2.96950
2.41847 2.98753 3.56838 5.64910 4.23765 112 e - - -
2.68618 4.42225 2.77519 2.73123 3.46354 2.40513 3.72494
3.29354 2.67741 2.69355 4.24690 2.90347 2.73739 3.18146 2.89801
2.37887 2.77519 2.98518 4.58477 3.61503
0.02489 4.10147 4.82382 0.61958 0.77255 0.48576 0.95510
82 3.17012 4.48038 4.89404 4.41300 3.58842 4.51387 5.11775
1.00022 4.28890 2.06284 3.39241 4.62119 4.82832 4.55205 4.47565
3.91683 3.44099 1.45929 5.59258 4.38981 113 i - - -
2.68618 4.42225 2.77519 2.73123 3.46354 2.40513 3.72494
3.29354 2.67741 2.69355 4.24690 2.90347 2.73739 3.18146 2.89801
2.37887 2.77519 2.98518 4.58477 3.61503
0.02489 4.10147 4.82382 0.61958 0.77255 0.48576 0.95510
83 0.72813 4.32237 3.71027 3.51950 4.29523 3.13943 4.51231
3.45615 3.54208 3.32659 4.33814 3.60635 3.90095 3.86312 3.77237
2.67322 2.96682 3.07018 5.69905 4.52711 114 a - - -
2.68618 4.42225 2.77519 2.73123 3.46354 2.40513 3.72494
3.29354 2.67741 2.69355 4.24690 2.90347 2.73739 3.18146 2.89801
2.37887 2.77519 2.98518 4.58477 3.61503
0.02489 4.10147 4.82382 0.61958 0.77255 0.48576 0.95510
84 2.73682 4.97078 2.78500 2.44790 4.37809 3.37584 3.72102
3.79780 2.10077 3.36835 4.19519 1.68660 3.87261 2.87348 2.81223
2.74509 2.56458 3.42939 5.54783 4.21202 115 n - - -
2.68618 4.42225 2.77519 2.73123 3.46354 2.40513 3.72494
3.29354 2.67741 2.69355 4.24690 2.90347 2.73739 3.18146 2.89801
2.37887 2.77519 2.98518 4.58477 3.61503
0.02489 4.10147 4.82382 0.61958 0.77255 0.48576 0.95510
85 2.66586 4.78241 2.92062 2.52697 4.04479 3.38535 2.17974
3.55330 2.50839 3.16192 4.00539 2.63793 3.86702 2.91712 2.90427
2.71698 2.09873 3.22118 5.33802 3.98806 116 t - - -
2.68618 4.42225 2.77519 2.73123 3.46354 2.40513 3.72494
3.29354 2.67741 2.69355 4.24690 2.90347 2.73739 3.18146 2.89801
2.37887 2.77519 2.98518 4.58477 3.61503
0.02489 4.10147 4.82382 0.61958 0.77255 0.48576 0.95510
86 3.22935 4.51911 4.96319 4.42650 3.38049 4.59586 5.03163
1.30382 4.30732 1.39728 3.17120 4.64957 4.82378 4.48014 4.45367
3.96219 3.47037 1.62577 5.41024 4.28241 117 i - - -
2.68618 4.42225 2.77519 2.73123 3.46354 2.40513 3.72494
3.29354 2.67741 2.69355 4.24690 2.90347 2.73739 3.18146 2.89801
2.37887 2.77519 2.98518 4.58477 3.61503
0.02489 4.10147 4.82382 0.61958 0.77255 0.48576 0.95510
87 2.58443 4.22874 3.59093 3.02346 2.90466 3.59317 3.84858
2.33188 2.95597 2.42324 3.33314 3.38716 3.97098 2.76571 3.27042
2.68531 2.54803 2.48947 4.78836 2.72657 118 i - - -
2.68618 4.42225 2.77519 2.73123 3.46354 2.40513 3.72494
3.29354 2.67741 2.69355 4.24690 2.90347 2.73739 3.18146 2.89801
2.37887 2.77519 2.98518 4.58477 3.61503

0.02489 4.10147 4.82382 0.61958 0.77255 0.48576 0.95510
88 3.08477 4.79735 3.71766 3.59413 4.55974 3.47220 4.64759
4.17071 3.67237 3.76988 4.83344 3.87496 0.49405 4.05396 3.88672
3.26074 3.54544 3.78874 5.67713 4.68541 119 P - - -
2.68618 4.42225 2.77519 2.73123 3.46354 2.40513 3.72494
3.29354 2.67741 2.69355 4.24690 2.90347 2.73739 3.18146 2.89801
2.37887 2.77519 2.98518 4.58477 3.61503
0.02489 4.10147 4.82382 0.61958 0.77255 0.48576 0.95510
89 2.76771 4.95347 3.18786 2.57989 4.26341 3.53415 3.62839
3.65348 1.61940 3.20104 3.23284 3.05951 3.43374 2.76829 2.05758
2.78733 2.97716 3.32986 5.37750 4.10173 120 k - - -
2.68618 4.42225 2.77519 2.73123 3.46354 2.40513 3.72494
3.29354 2.67741 2.69355 4.24690 2.90347 2.73739 3.18146 2.89801
2.37887 2.77519 2.98518 4.58477 3.61503
0.02489 4.10147 4.82382 0.61958 0.77255 0.48576 0.95510
90 1.17849 4.22614 3.50321 3.27128 4.41547 1.72053 4.35474
3.78420 3.37164 3.51868 4.36188 3.39977 3.76330 3.64382 3.66636
2.47765 2.79590 3.23546 5.75531 4.56477 121 a - - -
2.68618 4.42225 2.77519 2.73123 3.46354 2.40513 3.72494
3.29354 2.67741 2.69355 4.24690 2.90347 2.73739 3.18146 2.89801
2.37887 2.77519 2.98518 4.58477 3.61503
0.02489 4.10147 4.82382 0.61958 0.77255 0.48576 0.95510
91 1.95933 4.31276 4.27443 3.75908 3.60381 3.93654 4.52960
1.80404 3.66841 2.33830 3.47683 4.00100 4.37465 3.95055 3.92036
3.29011 3.11329 1.34629 5.31199 4.10140 122 v - - -
2.68618 4.42225 2.77519 2.73123 3.46354 2.40513 3.72494
3.29354 2.67741 2.69355 4.24690 2.90347 2.73739 3.18146 2.89801
2.37887 2.77519 2.98518 4.58477 3.61503
0.02489 4.10147 4.82382 0.61958 0.77255 0.48576 0.95510
92 2.61674 4.73271 2.35273 2.48053 4.03244 3.38302 3.72069
3.37281 2.57429 3.05846 3.90103 2.62566 3.84149 2.89725 3.03107
2.67496 2.40621 2.23886 5.34038 4.01512 123 v - - -
2.68618 4.42225 2.77519 2.73123 3.46354 2.40513 3.72494
3.29354 2.67741 2.69355 4.24690 2.90347 2.73739 3.18146 2.89801
2.37887 2.77519 2.98518 4.58477 3.61503
0.02489 4.10147 4.82382 0.61958 0.77255 0.48576 0.95510
93 3.15149 4.45081 4.91144 4.42235 3.64698 4.52754 5.13501
1.36005 4.31392 2.14477 3.44522 4.62809 4.83666 4.57850 4.50688
3.92393 3.41941 1.02935 5.62830 4.41873 124 v - - -
2.68618 4.42225 2.77519 2.73123 3.46354 2.40513 3.72494
3.29354 2.67741 2.69355 4.24690 2.90347 2.73739 3.18146 2.89801
2.37887 2.77519 2.98518 4.58477 3.61503
0.02489 4.10147 4.82382 0.61958 0.77255 0.48576 0.95510
94 2.50253 5.16049 2.13850 2.25995 4.49856 3.32185 3.68392
3.97249 2.53032 3.49450 4.28537 2.03662 3.82310 2.61528 3.04438
2.24510 2.99140 3.56494 5.66683 4.24918 125 n - - -
2.68618 4.42225 2.77519 2.73123 3.46354 2.40513 3.72494
3.29354 2.67741 2.69355 4.24690 2.90347 2.73739 3.18146 2.89801
2.37887 2.77519 2.98518 4.58477 3.61503
0.02489 4.10147 4.82382 0.61958 0.77255 0.48576 0.95510
95 2.95139 4.69050 2.53038 3.04967 2.26862 3.77609 3.63463
3.22121 3.08004 2.78834 3.79936 3.41234 4.17014 3.33868 3.43547
3.07094 3.18731 3.01529 4.22103 1.50719 126 y - - -

2.68618 4.42225 2.77519 2.73123 3.46354 2.40513 3.72494
3.29354 2.67741 2.69355 4.24690 2.90347 2.73739 3.18146 2.89801
2.37887 2.77519 2.98518 4.58477 3.61503
0.02489 4.10147 4.82382 0.61958 0.77255 0.48576 0.95510
96 2.49018 4.30775 3.63703 3.07750 3.49664 3.66277 3.96713
2.09189 2.46637 2.47736 3.43618 3.45567 4.05170 3.31158 3.30197
2.93413 2.65951 1.85434 4.99400 3.76672 127 v - - -
2.68618 4.42225 2.77519 2.73123 3.46354 2.40513 3.72494
3.29354 2.67741 2.69355 4.24690 2.90347 2.73739 3.18146 2.89801
2.37887 2.77519 2.98518 4.58477 3.61503
0.02489 4.10147 4.82382 0.61958 0.77255 0.48576 0.95510
97 2.63403 4.84077 2.14870 2.40833 4.09104 3.37855 3.67456
3.54244 2.51091 3.14660 3.96792 2.93187 3.82582 2.83599 2.98188
2.22237 2.56508 3.20832 5.36829 2.98234 128 d - - -
2.68618 4.42225 2.77519 2.73123 3.46354 2.40513 3.72494
3.29354 2.67741 2.69355 4.24690 2.90347 2.73739 3.18146 2.89801
2.37887 2.77519 2.98518 4.58477 3.61503
0.02489 4.10147 4.82382 0.61958 0.77255 0.48576 0.95510
98 2.78875 5.01718 3.08438 2.55683 4.39212 3.49628 3.64843
3.81002 2.18094 3.32809 4.15028 3.03929 3.91162 2.03442 1.87100
2.29974 3.01394 3.45595 5.46332 4.17553 129 r - - -
2.68618 4.42225 2.77519 2.73123 3.46354 2.40513 3.72494
3.29354 2.67741 2.69355 4.24690 2.90347 2.73739 3.18146 2.89801
2.37887 2.77519 2.98518 4.58477 3.61503
0.02489 4.10147 4.82382 0.61958 0.77255 0.48576 0.95510
99 2.57851 4.42867 3.32187 2.84325 3.73036 3.43990 3.88560
2.37902 2.79618 2.75526 3.67257 2.57742 3.92151 3.15047 3.16158
2.75399 1.84197 2.66991 5.15593 3.89164 130 t - - -
2.68618 4.42225 2.77519 2.73123 3.46354 2.40513 3.72494
3.29354 2.67741 2.69355 4.24690 2.90347 2.73739 3.18146 2.89801
2.37887 2.77519 2.98518 4.58477 3.61503
0.02489 4.10147 4.82382 0.61958 0.77255 0.48576 0.95510
100 3.25154 4.57836 4.86187 4.31760 3.26156 4.53847 4.92334
1.88254 4.18087 1.02532 3.06069 4.56652 4.76808 4.34881 4.33202
3.89637 3.48966 1.68854 5.30529 4.19348 131 l - - -
2.68618 4.42225 2.77519 2.73123 3.46354 2.40513 3.72494
3.29354 2.67741 2.69355 4.24690 2.90347 2.73739 3.18146 2.89801
2.37887 2.77519 2.98518 4.58477 3.61503
0.02489 4.10147 4.82382 0.61958 0.77255 0.48576 0.95510
101 2.31459 4.25774 3.46030 3.14172 4.27480 3.04430 4.21148
3.63631 3.15404 3.36502 4.21579 3.33817 3.76864 3.47578 3.47220
1.38702 1.66814 3.15116 5.62951 4.39734 132 s - - -
2.68618 4.42225 2.77519 2.73123 3.46354 2.40513 3.72494
3.29354 2.67741 2.69355 4.24690 2.90347 2.73739 3.18146 2.89801
2.37887 2.77519 2.98518 4.58477 3.61503
0.02489 4.10147 4.82382 0.61958 0.77255 0.48576 0.95510
102 3.08480 5.06424 3.07612 2.84103 4.26228 3.53160 3.99906
3.93485 2.64723 3.41356 4.44127 3.30042 4.09235 0.87547 2.92413
3.12578 3.38456 3.65206 5.49605 4.20789 133 q - - -
2.68618 4.42225 2.77519 2.73123 3.46354 2.40513 3.72494
3.29354 2.67741 2.69355 4.24690 2.90347 2.73739 3.18146 2.89801
2.37887 2.77519 2.98518 4.58477 3.61503
0.02489 4.10147 4.82382 0.61958 0.77255 0.48576 0.95510

```
    103   2.42841  4.44923  3.13453  2.78121  4.23646  3.15288  3.96907
3.65266  2.84651  3.30436  4.13158  2.59443  3.78773  3.17753  3.23877
1.70002  1.81656  3.21165  5.54153  4.26064    134 s - - -
          2.68618  4.42225  2.77519  2.73123  3.46354  2.40513  3.72494
3.29354  2.67741  2.69355  4.24690  2.90347  2.73739  3.18146  2.89801
2.37887  2.77519  2.98518  4.58477  3.61503
          0.02489  4.10147  4.82382  0.61958  0.77255  0.48576  0.95510
    104   2.93125  4.92464  2.86249  2.77433  4.32616  3.35217  4.13174
4.09283  3.05859  3.71503  4.67362  0.80845  4.01796  3.41004  3.41667
2.99289  3.32381  3.69136  5.60586  4.26204    135 n - - -
          2.68618  4.42225  2.77519  2.73123  3.46354  2.40513  3.72494
3.29354  2.67741  2.69355  4.24690  2.90347  2.73739  3.18146  2.89801
2.37887  2.77519  2.98518  4.58477  3.61503
          0.02489  4.10147  4.82382  0.61958  0.77255  0.48576  0.95510
    105   2.93125  4.92464  2.86249  2.77433  4.32616  3.35217  4.13174
4.09283  3.05859  3.71503  4.67362  0.80845  4.01796  3.41004  3.41667
2.99289  3.32381  3.69136  5.60586  4.26204    136 n - - -
          2.68618  4.42225  2.77519  2.73123  3.46354  2.40513  3.72494
3.29354  2.67741  2.69355  4.24690  2.90347  2.73739  3.18146  2.89801
2.37887  2.77519  2.98518  4.58477  3.61503
          0.02489  4.10147  4.82382  0.61958  0.77255  0.48576  0.95510
    106   2.66129  4.80096  2.90927  2.55520  4.29481  3.33340  3.77982
3.72098  2.49503  3.31265  4.15542  3.02799  3.86394  1.53332  2.86401
2.36346  2.68637  3.34667  5.51289  4.19483    137 q - - -
          2.68618  4.42225  2.77519  2.73123  3.46354  2.40513  3.72494
3.29354  2.67741  2.69355  4.24690  2.90347  2.73739  3.18146  2.89801
2.37887  2.77519  2.98518  4.58477  3.61503
          0.02489  4.10147  4.82382  0.61958  0.77255  0.48576  0.95510
    107   3.11551  4.45215  4.79999  4.32265  3.63844  4.42061  5.05286
1.60577  4.19976  2.15928  3.45732  4.53151  4.77137  4.48617  4.40393
3.82192  3.39488  0.91476  5.59619  4.37038    138 v - - -
          2.68618  4.42225  2.77519  2.73123  3.46354  2.40513  3.72494
3.29354  2.67741  2.69355  4.24690  2.90347  2.73739  3.18146  2.89801
2.37887  2.77519  2.98518  4.58477  3.61503
          0.02489  4.10147  4.82382  0.61958  0.77255  0.48576  0.95510
    108   2.64401  4.90493  2.72410  2.39548  4.36903  2.56111  3.73053
3.82224  2.54614  3.38326  4.18724  2.28278  3.81941  2.49745  3.00906
1.95581  2.93427  3.42010  5.57558  4.21048    139 s - - -
          2.68618  4.42225  2.77519  2.73123  3.46354  2.40513  3.72494
3.29354  2.67741  2.69355  4.24690  2.90347  2.73739  3.18146  2.89801
2.37887  2.77519  2.98518  4.58477  3.61503
          0.02489  4.10147  4.82382  0.61958  0.77255  0.48576  0.95510
    109   2.42039  4.21952  3.76358  3.22002  3.47774  3.57068  4.04319
2.12258  3.14900  2.48165  3.43342  3.52589  4.02527  3.43591  3.44615
2.28227  2.52348  1.86618  4.98505  3.76943    140 v - - -
          2.68618  4.42225  2.77519  2.73123  3.46354  2.40513  3.72494
3.29354  2.67741  2.69355  4.24690  2.90347  2.73739  3.18146  2.89801
2.37887  2.77519  2.98518  4.58477  3.61503
          0.02489  4.10147  4.82382  0.61958  0.77255  0.48576  0.95510
    110   3.16507  4.68081  4.30383  3.92065  3.32960  4.00980  4.60293
2.52909  3.67450  1.89478  0.99811  4.16802  4.47683  4.04418  3.85168
3.53531  3.47762  2.56065  5.19741  3.97669    141 m - - -
          2.68618  4.42225  2.77519  2.73123  3.46354  2.40513  3.72494
3.29354  2.67741  2.69355  4.24690  2.90347  2.73739  3.18146  2.89801
2.37887  2.77519  2.98518  4.58477  3.61503
```

```
         0.02489  4.10147  4.82382  0.61958  0.77255  0.48576  0.95510
    111  3.16970  4.47369  4.91056  4.42608  3.60184  4.52832  5.13098
1.02814  4.30749  2.07790  3.40184  4.63340  4.83698  4.56774  4.49433
3.92916  3.43873  1.39948  5.60420  4.40262    142 i - - -
         2.68618  4.42225  2.77519  2.73123  3.46354  2.40513  3.72494
3.29354  2.67741  2.69355  4.24690  2.90347  2.73739  3.18146  2.89801
2.37887  2.77519  2.98518  4.58477  3.61503
         0.02489  4.10147  4.82382  0.61958  0.77255  0.48576  0.95510
    112  2.96016  5.45084  1.56543  2.18839  4.77455  3.26864  3.83496
4.33629  2.84866  3.85039  4.70357  1.50535  3.89481  3.00769  3.42893
2.86599  3.26318  3.90230  5.97604  4.48153    143 n - - -
         2.68618  4.42225  2.77519  2.73123  3.46354  2.40513  3.72494
3.29354  2.67741  2.69355  4.24690  2.90347  2.73739  3.18146  2.89801
2.37887  2.77519  2.98518  4.58477  3.61503
         0.02489  4.10147  4.82382  0.61958  0.77255  0.48576  0.95510
    113  2.84640  4.93382  3.34470  2.70208  4.22646  3.60536  3.65270
3.58926  1.62122  3.14301  2.74272  3.14941  3.97268  2.80567  1.91004
2.88621  3.04442  3.29855  5.34148  4.10399    144 k - - -
         2.68618  4.42225  2.77519  2.73123  3.46354  2.40513  3.72494
3.29354  2.67741  2.69355  4.24690  2.90347  2.73739  3.18146  2.89801
2.37887  2.77519  2.98518  4.58477  3.61503
         0.02489  4.10147  4.82382  0.61958  0.77255  0.48576  0.95510
    114  2.99291  4.47905  4.43777  4.11384  3.72844  3.95560  4.87955
2.06808  3.98532  2.37923  3.66423  4.26538  4.50765  4.33128  4.17498
3.47823  3.35157  0.77812  5.54966  4.29308    145 v - - -
         2.68618  4.42225  2.77519  2.73123  3.46354  2.40513  3.72494
3.29354  2.67741  2.69355  4.24690  2.90347  2.73739  3.18146  2.89801
2.37887  2.77519  2.98518  4.58477  3.61503
         0.02489  4.10147  4.82382  0.61958  0.77255  0.48576  0.95510
    115  2.91888  4.32308  4.42938  3.87192  3.40045  4.13131  4.52239
1.70268  3.76302  1.63699  3.26960  4.11781  4.46061  4.00208  3.97001
3.45090  2.45498  1.63618  5.15777  3.98017    146 v - - -
         2.68618  4.42225  2.77519  2.73123  3.46354  2.40513  3.72494
3.29354  2.67741  2.69355  4.24690  2.90347  2.73739  3.18146  2.89801
2.37887  2.77519  2.98518  4.58477  3.61503
         0.02489  4.10147  4.82382  0.61958  0.77255  0.48576  0.95510
    116  2.64262  4.95547  2.62967  1.93205  4.23038  3.38047  3.63273
3.66661  2.43195  2.66550  4.03303  2.61665  3.80945  2.76892  2.91244
2.47123  2.59708  3.30922  5.44430  4.07600    147 e - - -
         2.68618  4.42225  2.77519  2.73123  3.46354  2.40513  3.72494
3.29354  2.67741  2.69355  4.24690  2.90347  2.73739  3.18146  2.89801
2.37887  2.77519  2.98518  4.58477  3.61503
         0.02489  4.10147  4.82382  0.61958  0.77255  0.48576  0.95510
    117  2.71224  4.61369  3.20846  1.79035  3.81376  3.57843  3.87298
2.55634  2.72466  2.70068  3.69694  3.21600  3.99605  3.08669  3.12733
2.86016  2.96011  2.04303  5.27135  3.98212    148 e - - -
         2.68618  4.42225  2.77519  2.73123  3.46354  2.40513  3.72494
3.29354  2.67741  2.69355  4.24690  2.90347  2.73739  3.18146  2.89801
2.37887  2.77519  2.98518  4.58477  3.61503
         0.02489  4.10147  4.82382  0.61958  0.77255  0.48576  0.95510
    118  3.42193  4.81975  4.55640  4.20329  3.23883  4.27043  4.78781
2.46132  3.96896  0.63698  3.20089  4.45661  4.67022  4.28182  4.10509
3.87730  3.70786  2.55699  5.19486  3.96904    149 L - - -
```

```
          2.68618  4.42225  2.77519  2.73123  3.46354  2.40513  3.72494
 3.29354  2.67741  2.69355  4.24690  2.90347  2.73739  3.18146  2.89801
 2.37887  2.77519  2.98518  4.58477  3.61503
          0.02489  4.10147  4.82382  0.61958  0.77255  0.48576  0.95510
     119  3.12049  5.22355  3.49521  2.85671  4.68758  3.71192  3.67356
 4.04202  1.03586  3.47927  4.35744  3.25593  4.09189  2.81071  1.90155
 3.11503  3.29012  3.72326  5.51021  4.34669    150 k - - -
          2.68618  4.42225  2.77519  2.73123  3.46354  2.40513  3.72494
 3.29354  2.67741  2.69355  4.24690  2.90347  2.73739  3.18146  2.89801
 2.37887  2.77519  2.98518  4.58477  3.61503
          0.02489  4.10147  4.82382  0.61958  0.77255  0.48576  0.95510
     120  2.49800  4.33432  3.45825  2.97680  3.73695  3.34606  3.96277
 3.00197  2.90721  2.16927  3.69218  3.31734  3.88996  3.25259  3.24729
 2.06103  1.90740  2.73018  5.17336  3.92540    151 t - - -
          2.68618  4.42225  2.77519  2.73123  3.46354  2.40513  3.72494
 3.29354  2.67741  2.69355  4.24690  2.90347  2.73739  3.18146  2.89801
 2.37887  2.77519  2.98518  4.58477  3.61503
          0.02489  4.10147  4.82382  0.61958  0.77255  0.48576  0.95510
     121  3.21872  4.54295  4.85047  4.30782  3.30386  4.51792  4.91866
 1.85457  4.17658  1.13155  3.10650  4.54952  4.75875  4.35526  4.33282
 3.87509  3.45903  1.52909  5.32367  4.19957    152 l - - -
          2.68618  4.42225  2.77519  2.73123  3.46354  2.40513  3.72494
 3.29354  2.67741  2.69355  4.24690  2.90347  2.73739  3.18146  2.89801
 2.37887  2.77519  2.98518  4.58477  3.61503
          0.02489  4.10147  4.82382  0.61958  0.77255  0.48576  0.95510
     122  3.33224  4.65995  4.91432  4.34525  2.26728  4.55084  4.73683
 2.29634  4.21205  0.94947  2.30761  4.56362  4.72812  4.26869  4.29988
 3.88866  3.54615  2.46323  5.00223  3.83153    153 l - - -
          2.68618  4.42225  2.77519  2.73123  3.46354  2.40513  3.72494
 3.29354  2.67741  2.69355  4.24690  2.90347  2.73739  3.18146  2.89801
 2.37887  2.77519  2.98518  4.58477  3.61503
          0.02489  4.10147  4.82382  0.61958  0.77255  0.48576  0.95510
     123  2.97529  4.72177  3.69547  3.62698  4.75326  0.44951  4.72741
 4.41436  3.83906  4.03771  5.01959  3.86061  4.11981  4.14550  4.04435
 3.15472  3.47432  3.89839  5.78195  4.86495    154 G - - -
          2.68618  4.42225  2.77519  2.73123  3.46354  2.40513  3.72494
 3.29354  2.67741  2.69355  4.24690  2.90347  2.73739  3.18146  2.89801
 2.37887  2.77519  2.98518  4.58477  3.61503
          0.02489  4.10147  4.82382  0.61958  0.77255  0.48576  0.95510
     124  3.16584  4.45930  4.93711  4.44782  3.63168  4.55027  5.15313
 1.13610  4.33863  2.11569  3.42624  4.65236  4.85078  4.59635  4.52606
 3.94792  3.43217  1.23134  5.62686  4.42407    155 i - - -
          2.68618  4.42225  2.77519  2.73123  3.46354  2.40513  3.72494
 3.29354  2.67741  2.69355  4.24690  2.90347  2.73739  3.18146  2.89801
 2.37887  2.77519  2.98518  4.58477  3.61503
          0.02489  4.10147  4.82382  0.61958  0.77255  0.48576  0.95510
     125  1.70324  4.47090  3.07281  2.74708  4.30505  3.13632  3.97382
 3.73186  2.86217  3.37174  4.19074  2.21949  3.78047  3.17493  3.26957
 1.80635  2.83180  3.26691  5.59718  4.30691    156 a - - -
          2.68618  4.42225  2.77519  2.73123  3.46354  2.40513  3.72494
 3.29354  2.67741  2.69355  4.24690  2.90347  2.73739  3.18146  2.89801
 2.37887  2.77519  2.98518  4.58477  3.61503
          0.02489  4.10147  4.82382  0.61958  0.77255  0.48576  0.95510
```
[truncated: 642,036 more chars]
